# Entropy Scaling of Viscosity IV—Application to 124 Industrially Important Fluids

**DOI:** 10.1021/acs.jced.4c00451

**Published:** 2025-01-10

**Authors:** Viktor Martinek, Ian Bell, Roland Herzog, Markus Richter, Xiaoxian Yang

**Affiliations:** † Interdisciplinary Center for Scientific Computing, Heidelberg University, 69120 Heidelberg, Germany; ‡ Applied Chemicals and Materials Division, National Institute of Standards and Technology, Boulder, Colorado 80305, United States; § Faculty of Mechanical Engineering, Applied Thermodynamics, Chemnitz University of Technology, 09107 Chemnitz, Germany

## Abstract

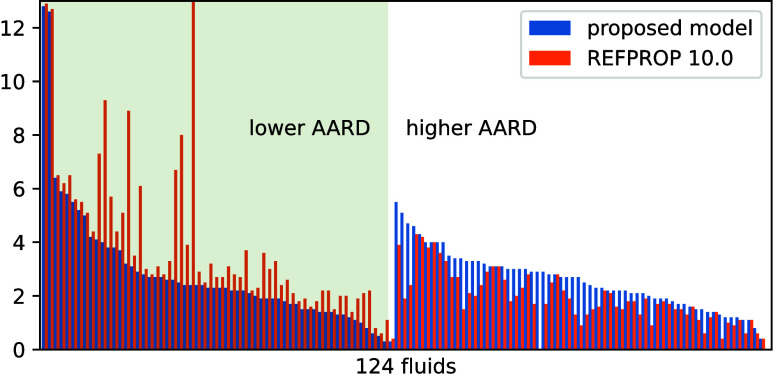

In our previous work [YangX.J. Chem. Eng. Data2021, 66, 1385–1398], a residual entropy scaling (RES) approach was developed to link viscosity to residual entropy using a 4-term power function for 39 refrigerants. In further research [YangX.Int. J. Thermophys.2022, 43, 183], this RES approach was extended to 124 pure fluids containing fluids from light gases (hydrogen and helium) to dense fluids (e.g., heavy hydrocarbons) and fluids with strong association force (e.g., water). In these previous research studies, the model was developed by manual optimization of the power function. The average absolute relative deviation (AARD) of experimental data from the RES model is approximately 3.36%, which is higher than the 2.74% obtained with the various models in REFPROP 10.0. In the present work, the power function was optimized by iteratively fitting the global (fluid-independent power terms) and local parameters (fluid-specific and group-specific parameters) and screening the experimental data. The resulting equation has only three terms instead of four. Most notably, the AARD of the new RES model is reduced down to 2.76%; this is very close to the various multiparameter models in REFPROP 10.0, while the average relative deviation (ARD) amounts to 0.03%, which is smaller than REFPROP 10.0’s 0.7%. A Python package is provided for the use of the developped model.

## 1 Introduction

With a residual entropy scaling (RES) approach, the residual part of the transport properties (mainly viscosity, thermal conductivity, and self-diffusion) of pure fluids can be expressed using a single variable: residual entropy. The residual entropy is a thermodynamic property and can be easily calculated with many well-developed and accurate equations of state (EOS). With this feature, the RES approach has been attracting attention in recent years and has been developed in many ways and used successfully by different authors. For example for viscosity, RES approaches have been applied to the Lennard-Jones (L-J) fluid and other spherically symmetric simple potentials,^1,2^ as well as hundreds of real fluids including hydrocarbons,^3−5^ refrigerants,^6−9^ and other fluids of industrial interest.^10−13^ Specifically, Lötgering-Lin et al.^11^ and Dehlouz et al.^14^ developed a generalized RES approach for viscosity of more than 100 pure fluids. For the thermal conductivity, RES approaches are also successful for many real fluids.^9,15−19^

In our previous work,^6^ an RES approach was developed to link residual viscosity to residual entropy using a 4-term power function for 39 refrigerants. Later, this approach was extended to 124 pure fluids^12,13^ containing a variety of fluids from light gases (hydrogen and helium) to dense fluids (e.g., heavy hydrocarbons) and fluids with strong association (e.g., water). This simple 4-term power function consists of different types of parameters: global parameters (i.e., the structure and power terms) for all fluids, group-specific parameters for a group of similar fluids, and fluid-specific parameters for each fluid. These parameters are explained in detail in Section 2.2. In the above-mentioned papers,^6,12,13^ the global parameters (structure and power terms) were determined manually by choosing the ones that best fit the approximately 50,000 well-evaluated experimental data points. With such a trial-and-error method, the average absolute relative deviation (AARD) (corresponding to scattering) of these experimental data from the RES model is approximately 3.36%, which is higher than the 2.74% obtained with the various state-of-the-art models in REFPROP 10.0.^20^

The primary aim of this work is to optimize these global parameters of the power function by means of a self-developed constrained numerical optimization routine. We aim at delivering an RES approach that has the same level of accuracy as the state-of-the-art models implemented in REFPROP 10.0, while using fewer parameters and an approach that is reproducible.

The RES approach calculates only the residual part of the viscosity; the remaining dilute gas viscosity η_ρ→0_(*T*) still needs to be determined by another method. If the highest accuracy is the aim, η_ρ→0_(*T*) should be calculated with the pure-fluid correlations implemented in REFPROP 10.0. These fluid-specific correlations are available when low-density viscosity data are available in the literature. REFPROP 10.0 cannot calculate η_ρ→0_(*T*) for some mixtures, especially those involving alcohols. Therefore, in the present work, other methods are also adopted for pure-fluid η_ρ→0_(*T*) calculations: (1) η_ρ→0_(*T*) is modeled as a polynomial of temperature with parameters fitted against REFPROP 10.0 results, (2) calculation with known L-J parameters as in our previous work,^6^ and (3) calculation with L-J parameters which are estimated according to critical point information. The first method aims at achieving high accuracy, while the latter two aim at using fewer parameters for the viscosity calculation, allowing for a broader predictive application.

This work is structured as follows. In Section 2.1, we provide a brief overview of the RES approach and methods to calculate η_ρ→0_(*T*). Thereafter, in Section 2.2, we discuss the design of the power function, the different types of parameters, how and when they are used, and how they were determined. In Section 3, the results are presented and compared to previous work and to REFPROP version 10.0. Finally, conclusions are drawn in Section 4. Part of this endeavor was supported by subproject 3 of the KETEC (Research Platform Refrigeration and Energy Technology) project.^21^ One important goal within KETEC subproject 3 is to model thermophysical properties of lubricant + refrigerant mixtures, and this work focuses on the viscosity of more general types of fluids.

## 2 Computational Methods

### 2.1 Fundamentals

In this section, we provide an overview of the RES approach and various methods to calculate the dilute gas viscosity η_ρ→0_(*T*). The fluid viscosity η at temperature *T* can be expressed as the sum of the dilute gas part η_ρ→0_(*T*) and the residual part η_res_(*s*^r^)
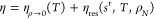
1Here *s*^r^ denotes the molar residual entropy.

**Table 1:** Brief Descriptions for Each of the Fluid Groups

Gr.	description
1	light gaseous fluids with quantum effects in the low temperature range, mainly hydrogen, its spin isomers, and helium
2	gaseous fluids, e.g., the noble gases
3	a majority of light hydrocarbons and halogenated hydrocarbons (most of refrigerants belong to this group)
4	fluids with benzene rings and similar fluids
5	medium hydrocarbons and similar fluids
6	heavy hydrocarbons and dense fluids
7	fluids with light intermolecular association among molecules like methanol
8	fluids with strong intermolecular association among molecules like water

**Table 2:** Group-Specific Parameters *n*_*g*,1_, *n*_*g*,2_, and *n*_*g*,3_ for the Eight Groups of Fluids

group	*n*_*g*,1_	*n*_*g*,2_	*n*_*g*,3_
1	1.297005	–3.104217	2.257168
2	0.363576	–0.074938	0.005159
3	0.392983	–0.167528	0.037984
4	0.305935	–0.128391	0.028658
5	0.286312	–0.111090	0.022642
6	0.220249	–0.070232	0.011963
7	0.305932	–0.171762	0.050058
8	0.297539	–0.209949	0.068151

**Table 3:** Parameters for All Pure Fluids Considered in This Work^a^

REFPROP fluid name	best available	group	ξ	*z*^*a*^	*n*_1_	*n*_2_	*n*_3_
13BUTADIENE	REFPROP 10.0	2	1.2847	1	0.258856	–0.078886	0.017208
1BUTENE	both	5	0.8356	1	0.430921	–0.203191	0.047206
1BUTYNE	predictive	4	0.9723	0	0.305935	–0.128391	0.028658
1PENTENE	predictive	4	0.9938	0	0.305935	–0.128391	0.028658
22DIMETHYLBUTANE	proposed model	3	0.9674	1	0.644029	–0.388594	0.104904
23DIMETHYLBUTANE	proposed model	3	1.0369	1	0.328817	–0.118197	0.023611
3METHYLPENTANE	REFPROP 10.0	5	0.8682	1	0.516076	–0.282909	0.0711
ACETONE	REFPROP 10.0	4	0.9358	1	0.442736	–0.224432	0.054166
ACETYLENE	proposed model	3	0.9977	0	0.392983	–0.167528	0.037984
AMMONIA	REFPROP 10.0	7	0.9102	1	0.238911	–0.07776	0.017714
ARGON	REFPROP 10.0	2	0.957	1	0.441631	–0.198307	0.064599
BENZENE	REFPROP 10.0	4	0.8377	1	0.252163	–0.043119	0.000423
BUTANE	REFPROP 10.0	3	1.0263	1	0.369686	–0.148651	0.032041
C11	proposed model	5	1.0597	1	0.298732	–0.119667	0.024296
C12	REFPROP 10.0	5	1.1021	1	0.26995	–0.10285	0.020241
C16	REFPROP 10.0	5	1.2385	1	0.236788	–0.085333	0.015984
C1CC6	REFPROP 10.0	3	0.9783	1	0.418776	–0.188387	0.044392
C22	proposed model	6	1.159	1	0.191955	–0.062655	0.01086
C2BUTENE	proposed model	5	0.8195	1	0.512632	–0.272262	0.067693
C3CC6	predictive	3	1.1154	0	0.392983	–0.167528	0.037984
C4F10	predictive	3	1.0628	0	0.392983	–0.167528	0.037984
C5F12	few data	5	0.852	1	0.409441	–0.185661	0.04174
C6F14	both	5	0.8902	1	0.443823	–0.211872	0.048157
CF3I	few data	3	0.9358	1	0.697509	–0.480812	0.145142
CHLORINE	REFPROP 10.0	3	0.8866	0	0.392983	–0.167528	0.037984
CHLOROBENZENE	proposed model	4	0.9249	1	0.323888	–0.130118	0.028328
CO	proposed model	2	1	0	0.363576	–0.074938	0.005159
CO2	REFPROP 10.0	3	1.0174	1	0.2335	0.007837	–0.023471
COS	predictive	3	0.9325	0	0.392983	–0.167528	0.037984
CYCLOBUTENE	predictive	3	0.9789	0	0.392983	–0.167528	0.037984
CYCLOHEX	proposed model	3	0.8973	1	0.741397	–0.472354	0.132227
CYCLOPEN	REFPROP 10.0	3	0.9495	1	0.41325	–0.175764	0.040135
CYCLOPRO	few data	3	0.9517	0	0.392983	–0.167528	0.037984
D2	proposed model	1	1.3518	1	0.396609	–0.510562	0.40281
D2O	REFPROP 10.0	8	1.0445	1	0.280658	–0.182178	0.056524
D4	both	6	0.8426	1	0.3353	–0.131842	0.026054
D5	both	6	0.8501	1	0.33123	–0.124703	0.023571
D6	predictive	5	1.0959	0	0.286312	–0.11109	0.022642
DEA	proposed model	7	1.301	1	0.365242	–0.196452	0.04811
DECANE	REFPROP 10.0	5	1.026	1	0.306021	–0.125138	0.02603
DEE	REFPROP 10.0	3	1.148	1	0.319833	–0.124679	0.025516
DMC	REFPROP 10.0	3	1.1865	1	0.322188	–0.139703	0.031902
DME	REFPROP 10.0	3	1.0772	1	0.357695	–0.15162	0.03393
EBENZENE	REFPROP 10.0	4	0.9678	1	0.371309	–0.168943	0.038494
EGLYCOL	both	7	1.2176	1	0.422328	–0.22843	0.056447
ETHANE	REFPROP 10.0	3	0.9456	1	0.404667	–0.168529	0.0384
ETHANOL	proposed model	7	1.1362	1	0.292038	–0.160178	0.043583
ETHYLENE	both	3	0.9348	1	0.391366	–0.159554	0.036436
ETHYLENEOXIDE	proposed model	3	1.046	1	0.599879	–0.367331	0.099849
FLUORINE	both	2	1.0632	1	0.453053	–0.206686	0.053423
H2S	proposed model	7	0.7585	1	0.225851	–0.00749	–0.007898
HCL	proposed model	7	0.7276	1	0.028574	0.241739	–0.097719
HELIUM	both	1	1.2661	0	1.297005	–3.104217	2.257168
HEPTANE	REFPROP 10.0	5	0.9283	1	0.321695	–0.130973	0.027822
HEXANE	proposed model	5	0.8913	1	0.355662	–0.151115	0.032927
HYDROGEN	REFPROP 10.0	1	1.0136	1	2.272439	–6.277765	4.310689
IBUTENE	both	3	1.0146	1	0.406355	–0.188449	0.045383
IHEXANE	proposed model	5	0.87	1	0.368669	–0.158143	0.034655
IOCTANE	proposed model	5	0.8323	1	0.416715	–0.191216	0.043679
IPENTANE	proposed model	3	1.0344	1	0.391104	–0.168977	0.038118
ISOBUTAN	proposed model	3	0.9504	1	0.443856	–0.203979	0.048521
KRYPTON	REFPROP 10.0	2	0.9497	1	0.574447	–0.432866	0.18256
MD2M	predictive	6	1.0013	0	0.220249	–0.070232	0.011963
MD3M	predictive	6	1.0947	0	0.220249	–0.070232	0.011963
MD4M	predictive	6	1.1733	0	0.220249	–0.070232	0.011963
MDM	proposed model	6	0.9043	1	0.272782	–0.094278	0.01691
MEA	proposed model	7	1.179	1	0.394192	–0.200601	0.048884
METHANE	REFPROP 10.0	2	1.0301	1	0.436026	–0.208491	0.062506
METHANOL	proposed model	7	1.0736	1	0.3169	–0.194229	0.056838
MILPRF23699	predictive	6	1.3482	0	0.220249	–0.070232	0.011963
MLINOLEA	REFPROP 10.0	6	1.2558	1	0.151958	–0.041349	0.006241
MLINOLEN	REFPROP 10.0	6	1.2728	1	0.153776	–0.044009	0.006933
MM	few data	6	0.8248	1	0.575147	–0.299109	0.069042
MOLEATE	REFPROP 10.0	6	1.1938	1	0.183461	–0.055158	0.008903
MPALMITA	both	6	1.0704	1	0.254341	–0.090117	0.016221
MSTEARAT	few data	6	1.11	1	0.078226	–0.007601	–0.000082
MXYLENE	REFPROP 10.0	4	0.996	1	0.317989	–0.135573	0.030302
N2O	proposed model	2	1.2009	1	0.102561	0.326024	–0.210582
NEON	REFPROP 10.0	2	0.8561	1	0.375722	–0.010707	–0.018149
NEOPENTN	both	3	0.8922	1	0.415494	–0.153463	0.030807
NF3	proposed model	3	0.8253	0	0.392983	–0.167528	0.037984
NITROGEN	REFPROP 10.0	2	1.0092	1	0.340291	–0.091078	0.021042
NONANE	proposed model	5	0.9937	1	0.35713	–0.159116	0.034753
NOVEC649	predictive	4	1.048	0	0.305935	–0.128391	0.028658
OCTANE	REFPROP 10.0	5	0.9592	1	0.327911	–0.138181	0.029726
ORTHOHYD	predictive	1	1	0	1.297005	–3.104217	2.257168
OXYGEN	both	2	0.5879	0	0.363576	–0.074938	0.005159
OXYLENE	REFPROP 10.0	4	0.9475	1	0.381394	–0.178626	0.041899
PARAHYD	predictive	1	1.0284	0	1.297005	–3.104217	2.257168
PENTANE	proposed model	3	1.0806	1	0.404638	–0.184815	0.042214
POE5	predictive	6	1.2675	0	0.220249	–0.070232	0.011963
POE7	predictive	6	1.4492	0	0.220249	–0.070232	0.011963
POE9	predictive	6	1.706	0	0.220249	–0.070232	0.011963
PROPADIENE	predictive	3	0.9724	0	0.392983	–0.167528	0.037984
PROPANE	REFPROP 10.0	3	0.9744	1	0.406055	–0.178357	0.041571
PROPYLEN	proposed model	4	0.8228	1	0.511568	–0.269974	0.067762
PROPYLENEOXIDE	proposed model	3	1.074	1	0.490791	–0.265874	0.067684
PROPYNE	few data	3	1.0069	0	0.392983	–0.167528	0.037984
PXYLENE	REFPROP 10.0	4	0.9814	1	0.35906	–0.16616	0.038731
R11	proposed model	3	0.9674	1	0.424119	–0.188906	0.04421
R1123	predictive	3	1.1188	0	0.392983	–0.167528	0.037984
R113	proposed model	3	0.9528	1	0.470361	–0.225442	0.054716
R114	both	3	0.9733	1	0.615057	–0.378672	0.10544
R115	proposed model	3	0.9497	1	0.416234	–0.185289	0.044498
R116	both	3	1.1981	0	0.392983	–0.167528	0.037984
R12	proposed model	3	0.9752	1	0.336945	–0.094121	0.011411
R1216	predictive	3	1.1017	0	0.392983	–0.167528	0.037984
R1224YDZ	REFPROP 10.0	3	1.1264	1	0.146773	0.050263	–0.031134
R123	proposed model	3	1.0434	1	0.34979	–0.135478	0.028526
R1233ZDE	REFPROP 10.0	3	1.1227	1	0.3948	–0.184015	0.042551
R1234YF	REFPROP 10.0	3	1.0676	1	0.267769	–0.055341	0.001703
R1234ZEE	proposed model	3	1.0887	1	0.307227	–0.107687	0.020815
R1234ZEZ	predictive	3	1.1051	0	0.392983	–0.167528	0.037984
R124	proposed model	3	1.0288	1	0.41212	–0.18945	0.044478
R1243ZF	predictive	3	1.0406	0	0.392983	–0.167528	0.037984
R125	REFPROP 10.0	3	1.0298	1	0.326005	–0.109154	0.019807
R13	REFPROP 10.0	3	0.9866	1	0.385255	–0.158415	0.035273
R1336MZZZ	REFPROP 10.0	3	1.1513	1	0.095382	0.091688	–0.043208
R134A	REFPROP 10.0	3	1.0588	1	0.310059	–0.106155	0.020416
R14	both	3	1.0194	1	0.450154	–0.30721	0.112828
R141B	proposed model	3	0.9981	1	0.368577	–0.146787	0.032034
R142B	REFPROP 10.0	3	1.0054	1	0.198686	0.025088	–0.024214
R143A	REFPROP 10.0	3	1.0306	1	0.151459	0.08917	–0.050429
R150	proposed model	3	1.0268	1	0.535701	–0.305261	0.080679
R152A	REFPROP 10.0	3	1.0587	1	0.265764	–0.066609	0.008754
R161	proposed model	3	1.0527	0	0.392983	–0.167528	0.037984
R21	both	3	1.0294	1	0.335415	–0.112703	0.019639
R218	REFPROP 10.0	3	0.9965	1	0.361546	–0.142715	0.031279
R22	both	3	1.0343	1	0.392154	–0.174282	0.040375
R227EA	proposed model	3	1.0571	1	0.333557	–0.123002	0.02453
R23	REFPROP 10.0	3	1.0851	1	0.23995	–0.053348	0.006059
R236EA	proposed model	3	1.0467	1	0.336455	–0.126899	0.026339
R236FA	REFPROP 10.0	3	1.0897	1	0.323674	–0.119077	0.023505
R245CA	proposed model	3	1.0442	1	0.3904	–0.168551	0.037777
R245FA	REFPROP 10.0	3	1.0804	1	0.354663	–0.153191	0.035329
*R*32	REFPROP 10.0	3	1.1102	1	0.015284	0.220607	–0.095156
R365MFC	predictive	3	1.0755	0	0.392983	–0.167528	0.037984
R40	REFPROP 10.0	3	0.9811	1	0.142319	0.110023	–0.057116
R41	few data	3	0.9871	0	0.392983	–0.167528	0.037984
RC318	both	3	0.9792	1	0.490322	–0.247741	0.061422
RE143A	predictive	3	1.0141	0	0.392983	–0.167528	0.037984
RE245CB2	predictive	3	1.09	0	0.392983	–0.167528	0.037984
RE245FA2	proposed model	3	1.1249	1	0.386539	–0.176311	0.040336
RE347MCC	both	3	1.119	1	0.314588	–0.111438	0.020902
SF6	REFPROP 10.0	2	1.0969	1	0.182282	0.122179	–0.070003
SO2	proposed model	2	1.3355	1	0.000376	0.167659	–0.062185
T2BUTENE	predictive	3	1.0062	0	0.392983	–0.167528	0.037984
TOLUENE	REFPROP 10.0	4	0.9191	1	0.377943	–0.174981	0.041297
VINYLCHLORIDE	predictive	3	1.0429	0	0.392983	–0.167528	0.037984
WATER	REFPROP 10.0	8	1.0038	1	0.294605	–0.21161	0.069198
XENON	proposed model	2	0.9514	1	0.421622	–0.101908	0.008931

a The column “best available” indicates whether the proposed model is the best available based solely on our data set. The entries are as follows: If ARD and AARD are both lower compared to using REFPROP 10.0’s default model, “proposed model” is assigned. If the AARD of the proposed model is lower, while the ARD is higher, “both” is indicated. For fluids, where REFPROP 10.0’s default models perform better on both indicators, “REFPROP 10.0” is written. In cases, where our data set contains less than 20 data points, “few data” is specified. “predictive” is used in cases, where the coefficients are determined without any data. The column *z^a^* indicates whether fluid-specific parameters are available (*z*^*a*^ = 1) or not (*z*^*a*^ = 0). If fluid-specific parameters are available, then ξ = 1 is used in the calculation instead of the shown value for ξ. The equation with appropriate global exponents is .

**Figure 1:**
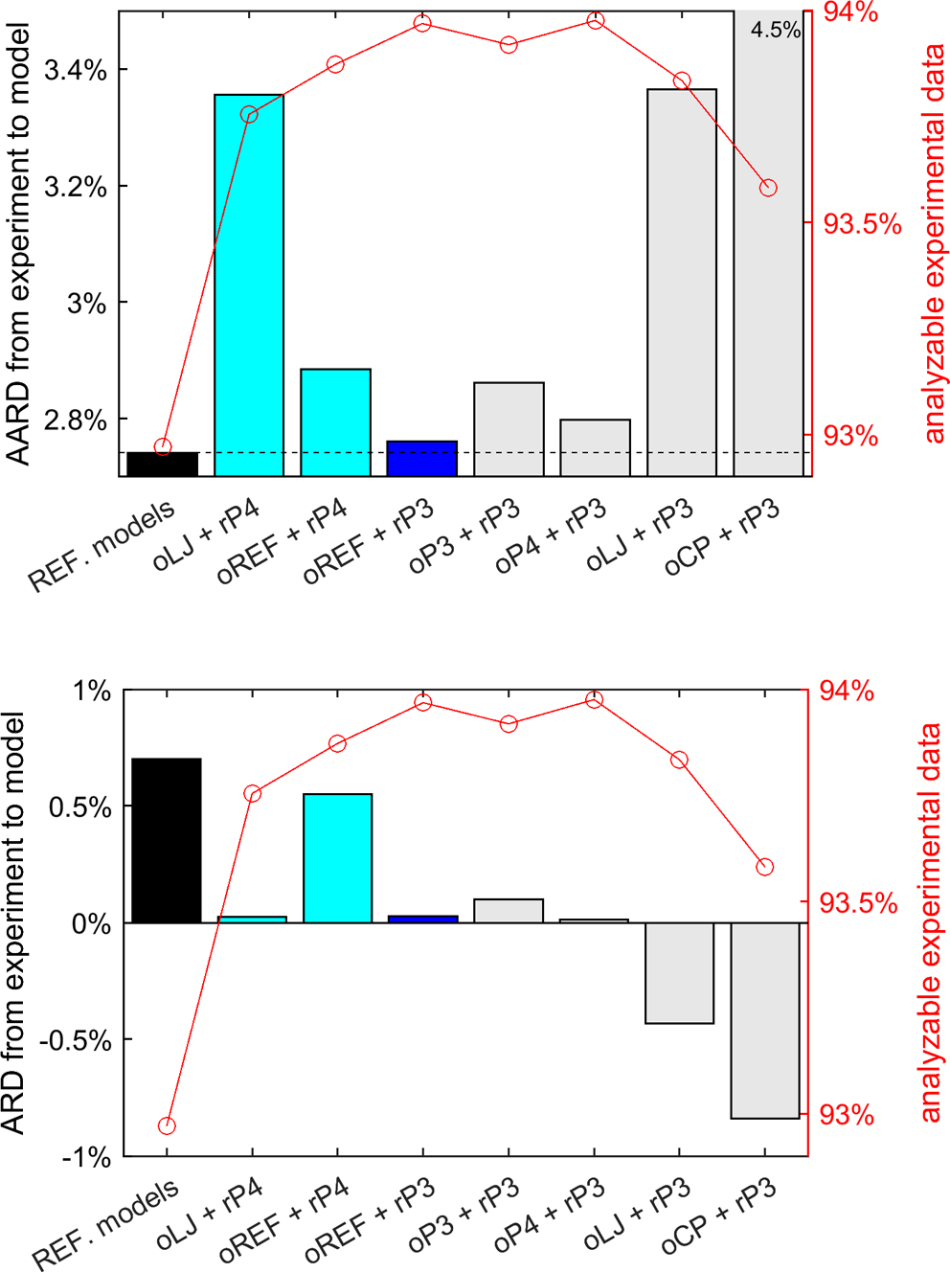
AARD (upper plot) and ARD (lower plot) of the filtered (or analyzable) experimental data from the model predictions. REF. models refers to the default viscosity models in REFPROP 10.0. For the remaining models, the labels comprise two parts connected by a “+” symbol. The former part denotes the method for dilute gas viscosity η_ρ→0_(*T*) calculation (see Section 2.1): oLJ uses L-J parameters, oREF uses REFPROP 10.0, oP3 uses a third-order polynomial, oP4 uses a fourth-order polynomial, and oCP uses the critical point information. The latter part refers to the residual viscosity calculation method: rP4 is a 4-term power function used in the previous work^6^ and rP3 is the 3-term power function developed in the present work.

**Figure 2:**
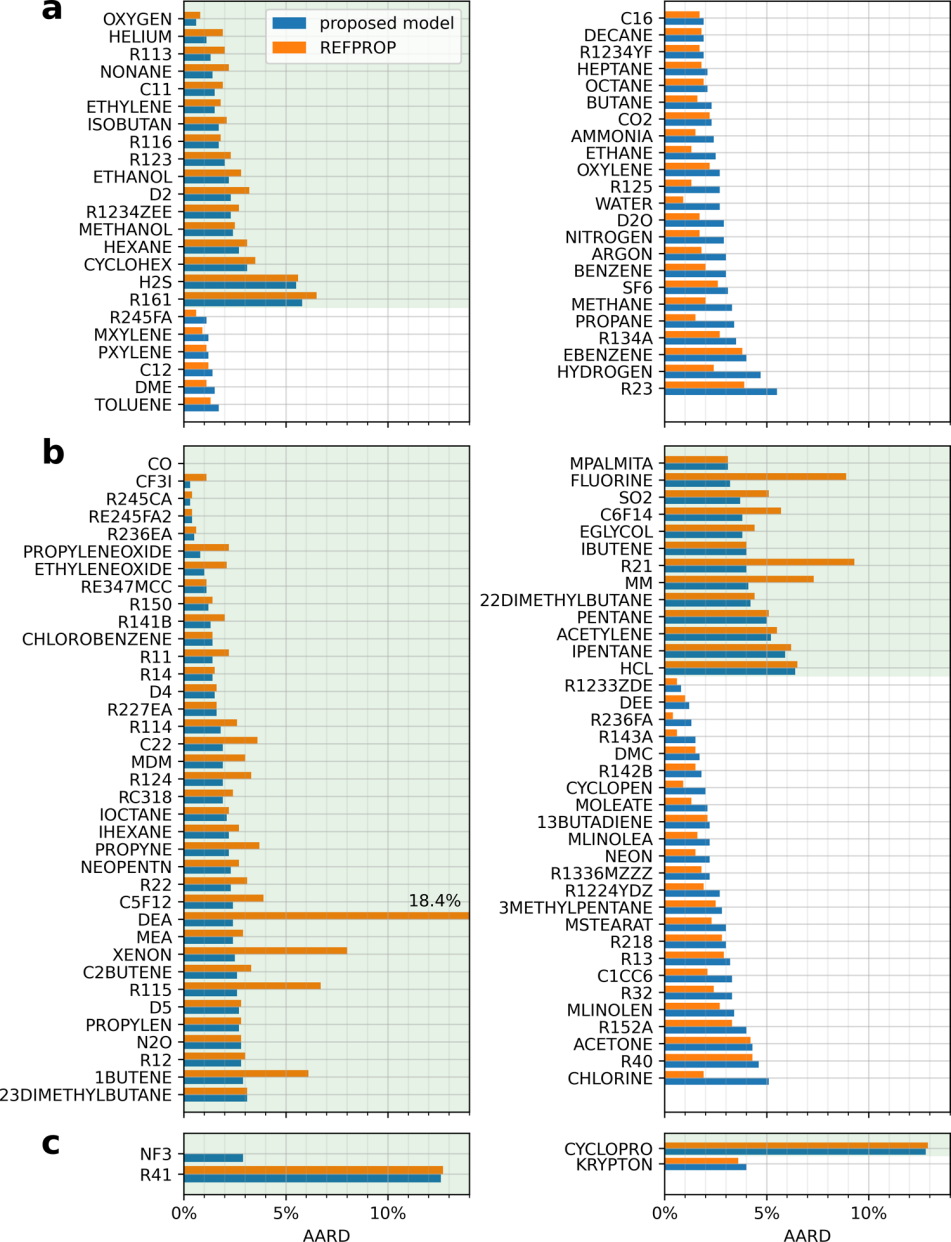
AARD of the REFPROP 10.0 models and the oREF + rP3 model predictions to the filtered experimental data for each pure fluid. The fluids are further classified according to the model types used by REFPROP 10.0: (a) reference correlations,^32−67^ (b) ECS model with fitted parameters or friction theory models,^47,58,61^ and (c) ECS model without fitted parameters and other models.^68−70^ Fluid names in REFPROP 10.0 are adopted (see the Appendix for their respective IUPAC chemical names and CAS registry numbers). The entries are sorted primarily by whether the proposed RES model has a lower AARD than that of REFPROP 10.0 and secondarily according to increasing AARD of the proposed model. The green background indicates that the proposed model is better.

**Figure 3:**
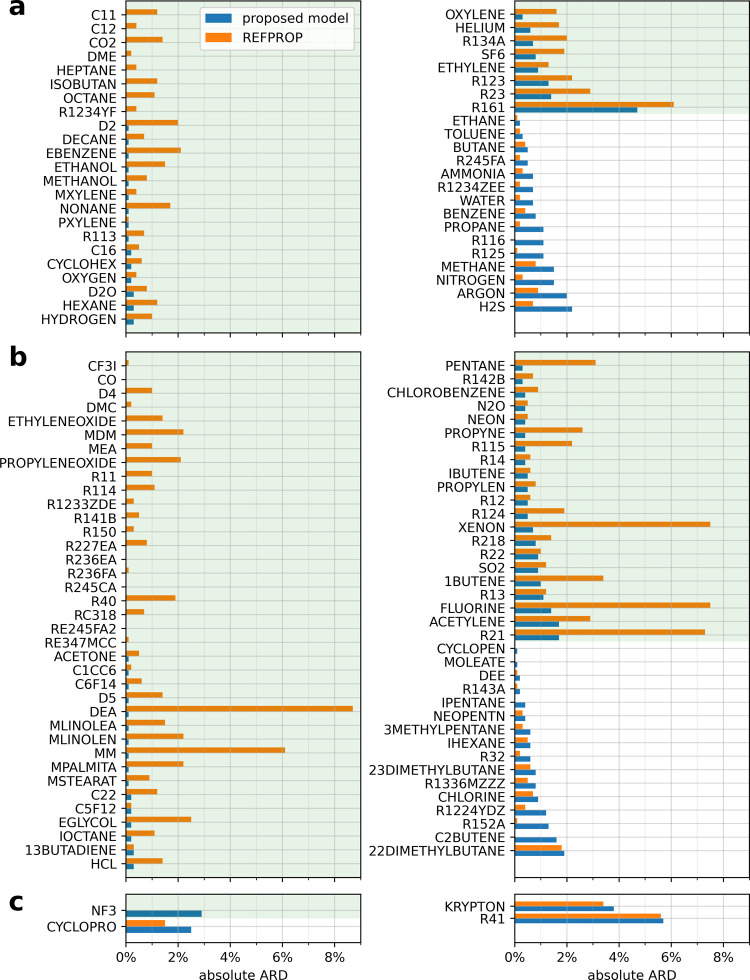
ARD of the REFPROP 10.0 models and the oREF + rP3 model predictions to the filtered experimental data for each pure fluid. The fluids are further classified according to the model types used by REFPROP 10.0: (a) reference correlations,^32−67^ (b) ECS model with fitted parameters or friction theory models,^47,58,61^ and (c) ECS model without fitted parameters and other models.^68−70^ Fluid names in REFPROP 10.0 are adopted (see the Appendix for their respective IUPAC chemical names and CAS registry numbers). The entries are sorted primarily by whether the proposed RES model has a lower absolute ARD than that of REFPROP 10.0 and secondarily according to increasing ARD of the proposed model. The green background indicates that the proposed model is better.

**Figure 4:**
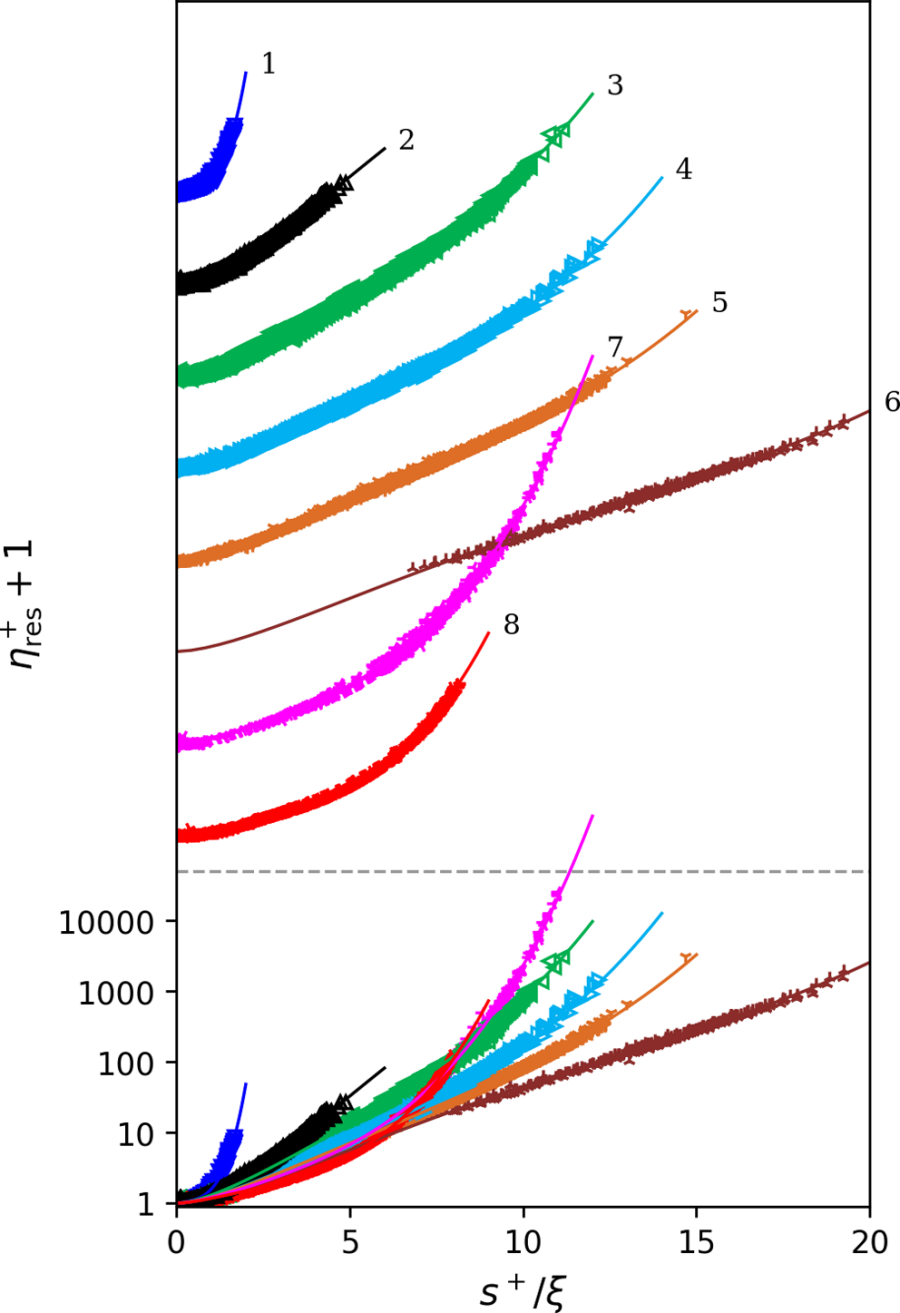
Plus-scaled dimensionless residual viscosity η_res_^+^ as a function of  for each group of pure fluids, where *s*^+^ is the plus-scaled residual entropy and ξ is the fluid-specific scaling factor. The group-specific parameters *n*_*i*,*g*_ are used to calculate the curves. All groups are shown at the bottom. Each group is also illustrated and annotated stacked by a power of 20 at the top to highlight the qualitative differences.

**Figure 5:**
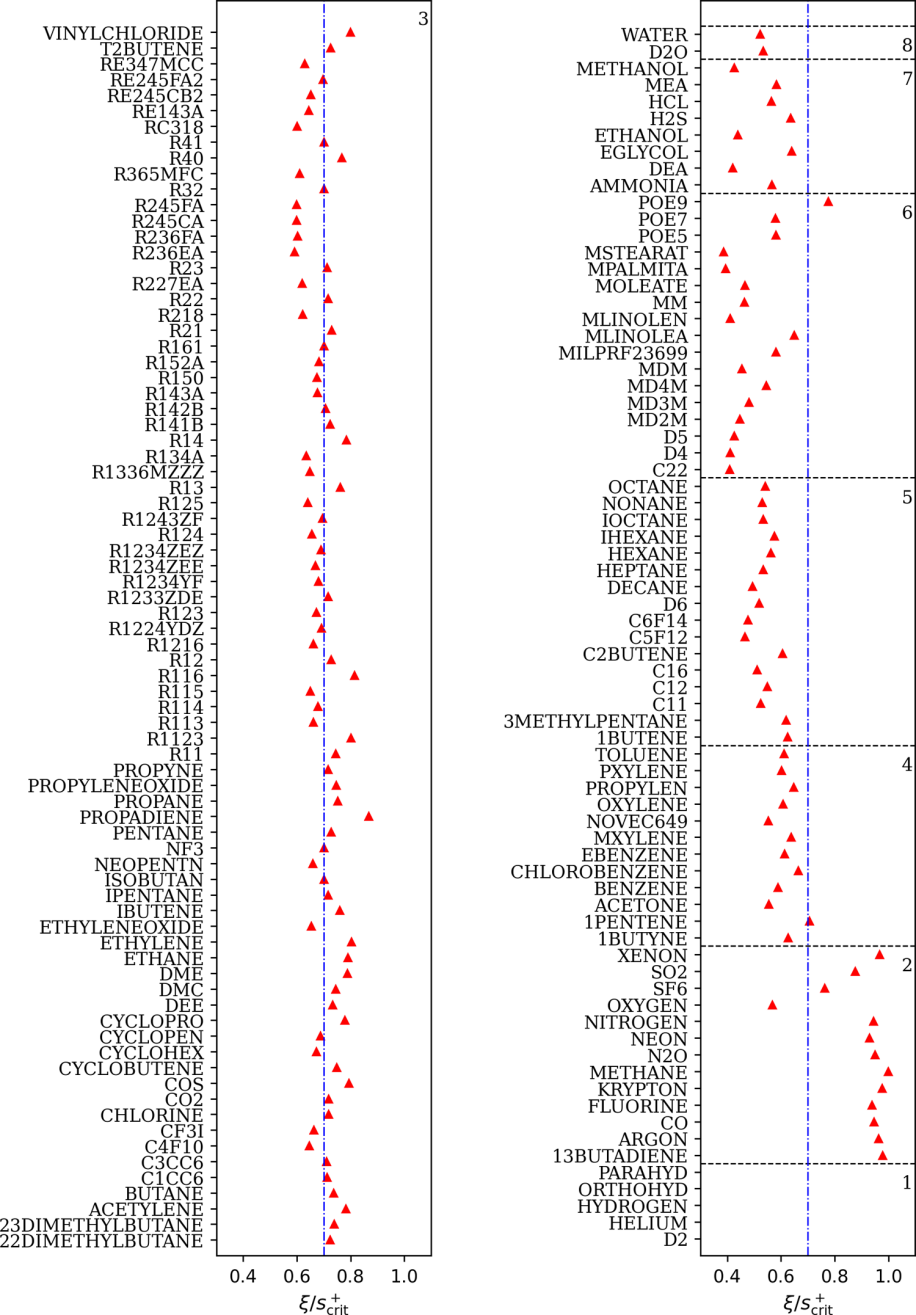
Ratio  of all considered pure fluids, where ξ is the fluid-specific scaling factor and *s*_crit_^+^ is the plus-scaled dimensionless residual entropy at the critical point, which is obtained from REFPROP 10.0. The group numbers are annotated in the top right of each box. At , a vertical dashed dotted line is drawn. Values for group 1 exceed the plot limits, i.e., PARAHYD: 1.67, ORTHOHYD: 1.61, HYDROGEN: 1.64, HELIUM: 4.09, D2:1.86.

**Figure 6:**
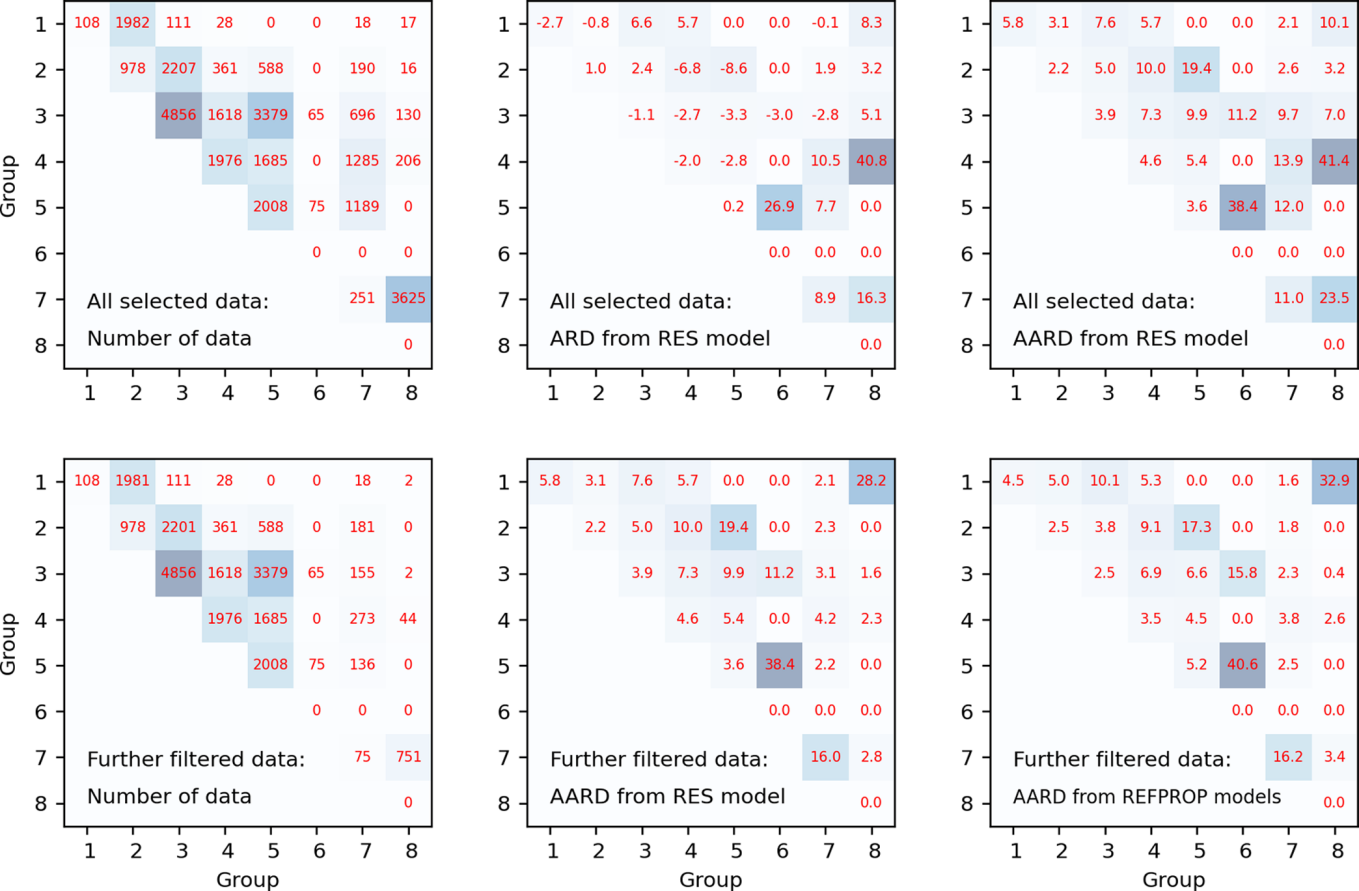
ARD and AARD of the experimental data of 351 binary mixtures from predictions of models. “All selected data”: for calculations with the RES model, similar filters as used in pure fluids were applied for mixture data. “Further filtered data”: one more filter is applied to the “All selected data” so that calculations are also available with REFPROP 10.0 models. For combinations with no available data, 0.0 is given.

For pure fluids, four methods are used for the η_ρ→0_(*T*) calculation in the present work. The first method (denoted as oREF) is simply using REFPROP 10.0, which implements a variety of different methods depending on the particular fluid. The second method (denoted as oP3 and oP4) is to use a polynomial as a function of temperature
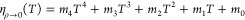
2where *T* is the temperature in K. The parameters *m*_0_, *m*_1_, *m*_2_, *m*_3_, *m*_4_ are fitted against calculations of REFPROP 10.0 from the triple point temperature to generally 1000 K or the highest calculable temperature and are implemented in the software package oilmixprop 1.0.^22^ The parameters are also included in the Supporting Information (SI) of the present work. Note that the fourth-order polynomial for η_ρ→0_(*T*) (denoted as oP4) yields better results than the third-order polynomial (denoted as oP3), while a fifth-order polynomial does not achieve notably better results. The third method (denoted as oLJ) is the same as the one used in our previous work^6^ and is described below. Assuming the molecular interactions can be approximated by those of L-J particles, η_ρ→0_(*T*) can be calculated using the Chapman–Enskog^23^ solution of the Boltzmann transport equation
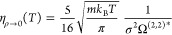
3where *m* is the molecular mass in units of kg, *k*_B_ = 1.380649 × 10^–23^ J/K is the Boltzmann constant, σ is the collision diameter of the L-J particle in unit of m, and Ω^(2,2)^* is the reduced collision integral obtained by integrating the possible approach trajectories of the particles. An empirical correlation of Ω^(2,2)^* for the Lennard–Jones fluid as a function of *T* was used
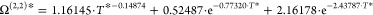
4where T* = *k*_B_*T*/ϵ is the dimensionless temperature, and ϵ/*k*_B_ is the reduced L-J pair-potential energy. This equation is also used in REFPROP 10.0^20^ and is a simplification of the original one proposed by Neufeld et al.^24^ In the third method, σ and ϵ were obtained from REFPROP 10.0 and are listed in the SI of our previous work.^6^ The fourth method (denoted as oCP) is almost the same as the third one, except that the L–J parameters σ and ϵ are estimated according to the predictive correlations of Chung et al.^25^
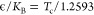
5
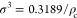
6where *T*_c_ and ρ_c_ are critical temperature in units of K and critical density in units of m^–3^, respectively. These fluid constants were obtained from REFPROP 10.0 and can be found in the SI of our previous work.^13^ Please note that in the third and fourth methods of calculating the dilute gas viscosity, the molecular interactions are approximated by those of L-J particles. However, this does not mean that all molecules are assumed to be spherical in this work. It only means that only the dilute gas portion is treated in that manner.

Now, we discuss the residual part of viscosity η_res_(*s*^r^), which, as introduced by Bell et al.,^3,10^ can be calculated by
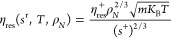
7
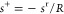
8Here, ρ*_N_* is the number density in unit of 1/m^3^, *s*^r^ is the molar residual entropy in unit of J/(mol K), defined as the difference between the real fluid entropy and the ideal gas entropy at the same temperature and density, i.e., *s*^r^(*T*, ρ) = *s*(*T*, ρ) – *s*^0^(*T*, ρ), and *R* ≈ 8.3145 J/(mol K) is the molar gas constant.^26^ The plus-scaled dimensionless residual viscosity η_res_^+^ (see eq 7) is linked to the plus-scaled dimensionless residual entropy *s*^+^ (see eq 8) using power functions to be discussed in the following section; see eq 13.

The Python interface of package CoolProp 6.4.1^27^ was used to call REFPROP 10.0^20^ and calculate the number density ρ_N_ and molar residual entropy *s*^r^ for both pure fluids and mixtures in the present work. The reference EOS used for each pure fluid are listed in Table S2 in the SI in our previous work.^13^

For mixtures, a predictive mixing rule was utilized to allow the RES model to predict the viscosity of mixtures without any additional adjustable parameters. The dilute gas viscosity for mixtures η_ρ→0,mix_ is obtained with the approximation of Wilke^28^
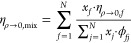
9with
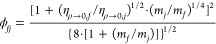
10where *x*_*f*_ and *m*_*f*_ are the mole fraction and the mass of one molecule of component *f*. In eqs 7 and 8, the effective mass of one particle *m* is substituted by the mole fraction weighted average *m*_mix_
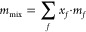
11Then, the mole fraction weighted average coefficients *n*_*i*,mix_ are utilized to substitute the fluid-specific and group-specific parameters *n*_*i*_, i.e.
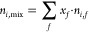
12where *n*_*i,f*_ (*i* = 1, 2, 3) are fitted parameters of pure fluid *f* (see the following section). The utilized mixing rules allow predictions of not only binaries but also multicomponent mixtures.

### 2.2 Equation Design, Predictions, and Parameter Identification

As mentioned in our previous work,^6,12,13^ the plus-scaled dimensionless residual viscosity η_res_^+^ is linked to the plus-scaled dimensionless residual entropy *s*^+^ with the following equation
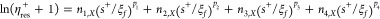
13

In eq 13, *p*_*i*_ (*i* = 1, 2, 3, 4) are global parameters that are the same for all fluids. *n*_*i*,*X*_ (*i* = 1, 2, 3, 4) are either fluid-specific (*X* = *f*) parameters, which are different for each fluid *f*, or group-specific parameters (*X* = *g*), which are different for each group of fluids *g*. The classification of the studied 124 pure fluids into eight groups is exactly the same as in our previous work.^13^ In Table 1, a short description for each group is provided, while the group numbers for each fluid are indicated in Table 3.

For a pure fluid, when there are sufficient good-quality data, fluid-specific parameters *n*_*i*,*f*_ can be obtained; otherwise, group-specific parameters *n*_*i*,*g*_ must be used. ξ_*f*_ is the fluid-specific scaling factor, which is equal to 1 when *n*_*i*,*f*_ is in use, or is a fitted value when *n*_*i*,*g*_ is in use.

In the previous work, *p*_*i*_ values (*i* = 1, 2, 3, 4) were taken equal to 1.0, 1.5, 2.0, 2.5, respectively, which were determined by the trial-and-error method. In the present work, *p*_*i*_ values were optimized with a comprehensive optimization procedure as summarized in Algorithms 1 and 2.
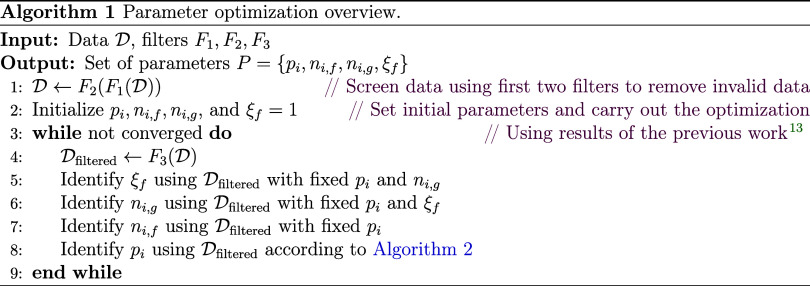

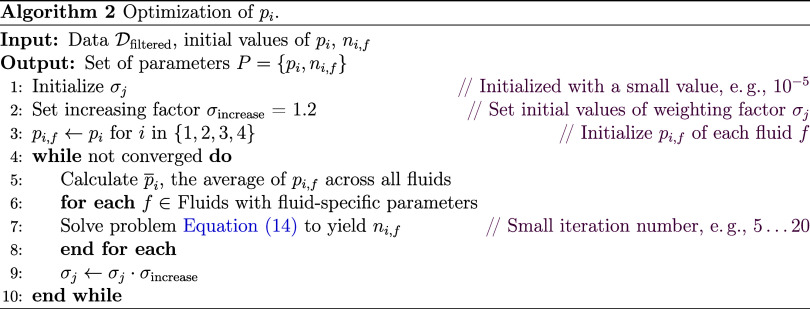


The same data filters *F*_1_, *F*_2_, *F*_3_ as in the previous work^13^ were adopted. The first filter *F*_1_ removes data that exceed the limitation of the used EOS in calculating density and residual entropy. These data amount to approximately 3.5% of all data. The second filter *F*_2_ removes data that suggest conflicting phase information. For instance, a high viscosity value suggests the fluid is in the liquid phase while the given temperature and pressure indicates that the fluid is in the gas phase. About 1.3% of the data were screened out with this filter. To handle outliers, the third filter *F*_3_ removes data whose relative deviation from the model prediction is larger than a set threshold. We used 30% for the threshold, which is the same as in the previous work. Filter *F*_3_ is involved in the iteration of Algorithm 1, and, in the end, only approximately 1.1% of the data are filtered out with this filter. Due to the very few data filtered out, there is limited risk that filter *F*_3_ is too selective, making the model appear to have a small deviation while actually steering the model in the wrong direction. For each fluid, the number of all data and of those filtered by each filter are summarized in a table in the SI of the present work.

As shown in Algorithm 1, the third filter *F*_3_ is carried out in every optimization loop. In each loop, the method to determine *n*_*i*,*f*_, *n*_*i*,*g*_, and ξ_*f*_ is the same as in the previous work,^13^ while the method to determine *p*_*i*_ is inspired by the literature^29−31^ and summarized in Algorithm 2.

In Algorithm 2, for *p*_*i*_ optimization, only fluids with fluid-specific parameters *n*_*i*,*f*_, i.e., with data of sufficiently good quality, are involved. This reduces the complexity of Algorithm 2 while there are still more than 90% of all screened data in use. For each studied fluid in Algorithm 2, an objective function is defined

Minimize
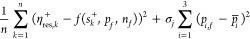
14w.r.t. *p*_*f*_, *n*_*f*_, where *p*_*i*,*f*_ are the fluid-specific *p*_*i*_ parameters for fluid *f*, *p̅*_*i*_ is the mean of *p*_*i,f*_ across all fluids, σ_*j*_ is a penalty weighting factor in the iteration step *j*, and η_res,*k*_^+^ and *s*_*k*_^+^ are the *k*-th data points to be fitted. This objective function couples the individual optimization problems (the first term in the objective function) by applying a penalty (the second term in the objective function) to the parameters intended to be global for each individual problem. The strategy to push fluid-specific *p*_*i,f*_ toward their mean *p̅*_*i*_ is to start with a very small penalty weighting factor, i.e., σ_*j*_ = 10^–5^, and then increase it step by step σ_*j*_ = σ_*j*_ · σ_increase_ with an increasing factor σ_increase_ = 1.2.

## 3 Results and Discussion

### 3.1 New RES Model

With the algorithms described in the previous section, the final optimized global parameters *p*_*i*_ (*i* = 1, 2, 3) are 1.8, 2.4, and 2.8, respectively. The fluid-specific parameters *n*_*i,f*_ are listed in Table 3, the group-specific parameters are listed in Table 2 and their fluid-specific scaling factors are listed in Table 3.

To note, the 4-term power function is reduced to a 3-term one. The 4-term power function is denoted as rP4, while the 3-term one is denoted as rP3. There are two key arguments for this simplification. First, to avoid negative slope of the ln(η_res_^+^ + 1) vs. *s*^+^ curve at the condition *s*^+^ = 0, the lower bound of *n*_1,*X*_ in the lowest power term is zero in the fitting. Although a negative slope is physical,^3^ a positive slope helps to avoid overfitting for those fluids without data in the gas phase. As a result, *n*_1,*X*_ = 0 holds in many fluids and groups. Second, compared to the 4-term equation, a 3-term one allows more fluids, particularly those without gas phase data, to have fluid-specific parameters. This is because, when there are no data in the gas phase, the 4-term equation overfits the data and delivers unreasonable parameters, while this problem is avoided by using the 3-term one.

To evaluate the new RES model, two statistical values AARD and the average relative deviation (ARD) are defined
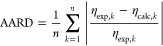
15
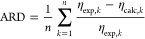
16where η_exp,*k*_ is the experimental viscosity of data point *k* and η_calc,*k*_ is the prediction by a model. The AARD is a measure of the scattering, while the ARD denotes systematic offsets. The AARD (upper plot) and ARD (lower plot) plots of the filtered (or analyzable) experimental data from different models are presented in Figure 1. REFPROP 10.0, as the state-of-the-art software package to accurately calculate thermophysical properties, was adopted as a baseline. For the fluids studied here, the default viscosity models in REFPROP 10.0 are reference correlations^32−67^ for 43 pure fluids, extended corresponding states models^58,68−70^ for 77 pure fluids, and friction theory models^47,61^ for three pure fluids. For fluid NF3, REFPROP 10.0 does not provide a viscosity model. The various RES-based models with different η_ρ→0_(*T*) calculation methods (see Section 2.1) and residual viscosity calculation with different power functions (the 4-term one from the previous work and the 3-term one in the present work) are also shown in Figure 1.

As can be seen in Figure 1, REFPROP 10.0 yields the lowest AARD (2.74%), i.e., the lowest scattering of experimental data from model, but with almost the largest ARD (0.7%), i.e., the largest systematic offset from experimental data to the model. The best RES approach is the one with η_ρ→0_(*T*) calculated using REFPROP 10.0 (oREF in Figure 1) and with residual viscosity calculated using the 3-term power function (rP3) from the present work. Compared to REFPROP 10.0, it yields slightly higher AARD (only 0.02% higher) but almost zero absolute ARD and with 1% more data analyzable (or 1% fewer data filtered out). Of course, the comparison to REFPROP 10.0 models is not entirely fair. A fair comparison should be that only data used to develop the REFPROP 10.0 models were adopted to assess our models. Such a comparison is however not straightforward to achieve given the documentary standards in the open literature, so it was not attempted. The parameters listed in Tables 2 and 3 are all based on this RES approach (oREF + rP3). A Python package containing the proposed model including the corresponding parameters is provided in the SI.

If η_ρ→0_(*T*) is calculated with polynomials (oP3 or oP4), the AARD slightly increases (less than 0.1%). This method is favored as the η_ρ→0_(*T*) is a relatively simple correlation with few parameters, and it helps to develop an RES approach more independent of REFPROP 10.0 in the future. If η_ρ→0_(*T*) is calculated with reliable L-J parameters (oLJ), the AARD reaches up to 3.4%; the benefits here are that fewer parameters are needed and fluids not implemented in REFPROP 10.0 can also be studied. If η_ρ→0_(*T*) is calculated with L-J parameters estimated using critical point information (oCP), the AARD reaches up to 4.5%. In this case, if critical point, mole mass, and acentric factor of a pure or quasi-pure fluid are known, only three additional *n*_*i,f*_ (*i* = 1, 2, 3) parameters are needed to enable reliable viscosity prediction (with an estimated standard uncertainty of 4.5%). Such applications have been seen in our previous work in fluid optimization^71^ and oil property modeling.^72^

Compared to the previous work^13^ in which a 4-term power function is used for residual viscosity calculation (rP4), the 3-term equation, in the present work, rP3 yields improved performance. The key reasons are (1) the function form with power terms is optimized and (2) overfitting is avoided so that more fluids have individual fluid-specific parameters.

### 3.2 Details for Pure-Fluid Correlation

The previous section provides the overall statistics of the performance of the best RES approach (oREF + rP3), while this section presents the details of its correlation of each pure fluid.

The AARD and ARD of the filtered experimental data from model predictions for each pure fluid are illustrated in Figures 2 and 3, respectively. Although overall REFPROP 10.0 has a lower AARD than the proposed RES model (see Figure 1), the proposed RES model shows a smaller AARD than REFPROP 10.0 for more than half of the 124 fluids. The proposed approach yields both, a lower AARD and ARD, for 48 of the pure fluids. The new RES model could be considered as the best available one according to the considered data. Direct comparison with the primary data sets selected by the authors of the reference correlation is required to make a more authoritative comparison. Such a comparison is not feasible to do because many of the data sets are not included in the NIST Thermo Data Engine database. This is also indicated in Table 3. The standard uncertainty of the RES model is estimated to be 2.8%, as approximately 68.4% (corresponding to one standard deviation of a normal distribution) of the data agree with the model within this threshold. The ARD of the proposed RES model for most of the pure fluids is significantly lower than that of REFPROP 10.0. One hundred and one fluids exhibit an absolute ARD of less than 1%, while 117 are below 2%. Most fluids with more than 1000 points have an absolute ARD of below 1%, while all are 1.5% or lower. Fluids with an absolute ARD larger than 2% are generally those with data only in the gas phase (e.g., R161), with very few data (e.g., CYCLOPRO, R41), or with less accurate dilute gas viscosity calculations (e.g., KRYPTON). The AARD and ARD of some fluids seem to be zero because either no model is available, e.g., for NF3 in REFPROP 10.0, or only dilute gas viscosity data are available, e.g., for CO.

Figure 4 shows the values of η_res_^+^ + 1 as a function of  for each group of pure fluids. As expected and similar to the previous work,^13^ each group’s fluids collapse into one curve. The fluid-specific scaling factors ξ_*f*_ of each fluid are listed in Table 3 and the ratio  are shown in Figure 5, where *s*_crit_^+^ is the plus-scaled dimensionless residual entropy at the critical point obtained from REFPROP 10.0. The ratios in group 3 are approximately 0.7, and for groups with heavier components, the ratios generally decrease, which is in good agreement to literature.^4,13,73^ With the group-specific parameters and the almost-constant  ratio in each group, one may obtain rough estimates of viscosity for fluids without data if the EOS yields a reasonable value for the residual entropy at the critical point. This can be done by classifying a fluid into one of the groups and use its constant  ratio to estimate its ξ_*f*_, based on the similarity of molecular structure, or on existing models. For example, the latter method has been applied to some fluids in the present work. These are indicated with “predictive” in the “best available” column in Table 3. For these 27 pure fluids, which are available in REFPROP 10.0 but for which no viscosity were available in the NIST ThermoDataEngine, the group number was determined by the following steps: (1) carrying out calculations using REFPROP 10.0 in both liquid and gas phases and using these data as “experimental data”; (2) fitting ξ_*f*_ using each group’s group-specific parameters with these “experimental data”; (3) assigning the group number of this fluid where its ξ_*f*_ is the most close to 1. These results are listed in Table 3. Finally, we would like to mention, compared to our previous work,^13^ group 1 is still a noticeable outlier in terms of fluid-specific scaling factors ξ_*f*_ as shown in Figure 5.

Group 1 comprises fluids with quantum effects at low temperatures, i.e., hydrogen, deuterium, and helium. Not only is the data availability low but also it is crucially low in areas where we expect the RES approach to worsen for those fluids. A simple empirical means of handling at least some of the quantum correction to the RES approach for the cryogens has been proposed,^74^ but further study is required. Furthermore, the results for water look better than they actually are, as the available data in the NIST ThermoDataEngine is not comprehensive. More experimental data of water and maybe alcohols need to be collected to further evaluate the RES model for these fluids.

### 3.3 Prediction for Mixtures

For fluid mixtures, the developed RES approach is a pure prediction method, i.e., no additional adjustable parameter is needed. In total, 33,109 data were collected from 351 binary mixtures as used in our previous work.^13^ An overview of statistical measures of the prediction performance of the developed RES approach is shown in Figure 6. Please note: (1) REFPROP 10.0 does not calculate dilute gas viscosity of many mixtures, especially those involving alcohols. Therefore, for the dilute gas viscosity of a mixture, the fourth-order polynomial (eq 2) was adopted for pure component calculation with the mixing rule of the approximation of Wilke^28^ as described in Section 2.2. (2) For all the collected mixture data, similar filters as used in pure fluids were applied, and the remaining data were named “all selected data” in Figure 6. REFPROP 10.0 cannot calculate about 20.5% of “all selected data”; therefore, one more filter was applied so that calculations were also available with REFPROP 10.0 models, and the remaining data are named “Further filtered data” in Figure 6. In summary, the proposed model agrees with 68.2% of “all selected data” within 7.8%.

With the RES model, for binary mixtures of the same group, the ARD is generally less than 2.7%. Mixtures within group 5 show particularly low ARD, namely 0.2%. The ARD for mixtures of different groups varies widely, ranging from 0.8% for groups 1 and 2 (with 1982 data points) up to 16.3% for groups 7 and 8 (with 3625 data points). As shown in Figure 6, the AARD of the all selected data has a relatively wide range depending on the group combination. The scatter for different groups are, however, mostly in the same order of magnitude as REFPROP 10.0’s, while some are lower. In total, the proposed model has a lower AARD than REFPROP 10.0 for 185 out of the 351 considered mixtures.

Modeling the viscosity of mixtures has been a challenging issue, especially for those composed of molecules of very different sizes. For instance, the AARD are very high for both the new RES model and the models in REFPROP 10.0 in comparison to experimental data of groups 1 and 8 (light gas and liquid with strong association force) and those involving group 6 (heavy molecules). One of the key reasons is that the viscosity changes thousands of times from a pure component of small molecular size (for example, a refrigerant *R*32 in group 3) to a pure component in group 6 of large molecular size (for example, dodecane as a lubricant oil in group 6). This, on the one hand, makes accurate measurements very hard and, on the other hand, makes the model generally yield very large deviations (e.g., on the order of 30%) if the given composition has a 1% uncertainty. Modeling mixtures using proper mixing rules is an important and interesting topic of future work.

## 4 Conclusions

The RES approach developed in our previous work was successfully improved. The approach demonstrates an accuracy on par with that of the fluids in REFPROP 10.0 not modeled by fluid-specific correlations. The key improvement lies in the optimization of the relations between the residual viscosity and the residual entropy. In the previous work, they are related by a 4-term power function determined manually, while in the present work they are related by a 3-term power function determined with a comprehensive fitting procedure. As a result, the average absolute relative deviation, corresponding to scattering of the experimental data from the model, is reduced down to 2.76%; this is very close to 2.74% obtained with the various multiparameter models in REFPROP 10.0. Meanwhile, the average relative deviation, corresponding to systematic offset of the experimental data to the model, is 0.03%, which is significantly better than REFPROP 10.0’s 0.7%. Based solely on the data available to us, for 48 of the 124 pure fluids, the proposed approach yields both a lower AARD and ARD. For mixture predictions, the RES approach is a pure prediction method, which agree with more than 68.4% of the data within 7.8%. Therefore, we could roughly estimate our RES having a standard uncertainty of 2.8% for pure fluids and 7.8% for mixtures.

The present work also investigates various methods to calculate the dilute gas viscosity. The method using REFPROP 10.0 calculation yields the best agreement with the experimental data. A fourth-order polynomial for dilute gas viscosity was developed in the present work which yields slightly worse results compared to REFPROP 10.0 but has a simple functional form which can be used for all studied fluids. Methods based on L-J parameters either obtained from reliable sources or estimations with critical point information were evaluated, as well. Both yield higher AARD. Nonetheless, with the last method, no additional parameters are needed for dilute gas viscosity calculation, while it yields AARD only twice (4.49% compared to 2.76%) of the best RES method here. To use the model developed in the present work, a Python package is provided in the SI. Fluid-specific parameters are suggested to be used, and only if they are not available should group-specific parameters with the fluid-specific scaling factor be used.

## Appendix: Fluid Name Glossary

In the following, the REFPROP 10.0 names of all considered fluids are listed with their corresponding IUPAC chemical names and their CAS Registry numbers:

13BUTADIENE: buta-1,3-diene; 106-99-0 | 1BUTENE: 1-butene; 106-98-9 | 1BUTYNE: but-1-yne; 107-00-6 | 1PENTENE: pent-1-ene; 109-67-1 | 22DIMETHYLBUTANE: 2,2-dimethylbutane; 75-83-2 | 23DIMETHYLBUTANE: 2,3-dimethylbutane; 79-29-8 | 3METHYLPENTANE: 3-methylpentane; 96-14-0 | ACETONE: propanone; 67-64-1 | ACETYLENE: ethyne; 74-86-2 | AMMONIA: ammonia; 7664-41-7 | ARGON: argon; 7440-37-1 | BENZENE: benzene; 71-43-2 | BUTANE: *n*-butane; 106-97-8 | C11: undecane; 1120-21-4 | C12: dodecane; 112-40-3 | C16: hexadecane; 544-76-3 | C1CC6: methylcyclohexane; 108-87-2 | C22: docosane; 629-97-0 | C2BUTENE: cis-2-butene; 590-18-1 | C3CC6: *n*-propylcyclohexane; 1678-92-8 | C4F10: decafluorobutane; 355-25-9 | C5F12: dodecafluoropentane; 678-26-2 | C6F14: tetradecafluorohexane; 355-42-0 | CF3I: trifluoroiodomethane; 2314-97-8 | CHLORINE: chlorine; 7782-50-5 | CHLOROBENZENE: chlorobenzene; 108-90-7 | CO: carbon monoxide; 630-08-0 | CO2: carbon dioxide; 124-38-9 | COS: carbon oxide sulfide; 463-58-1 | CYCLOBUTENE: 1-cyclobutene; 822-35-5 | CYCLOHEX: cyclohexane; 110-82-7 | CYCLOPEN: cyclopentane; 287-92-3 | CYCLOPRO: cyclopropane; 75-19-4 | D2: deuterium; 7782-39-0 | D2O: deuterium oxide; 7789-20-0 | D4: octamethylcyclotetrasiloxane; 556-67-2 | D5: decamethylcyclopentasiloxane; 541-02-6 | D6: dodecamethylcyclohexasiloxane; 540-97-6 | DEA: 2,2′-iminodiethanol; 111-42-2 | DECANE: decane; 124-18-5 | DEE: diethyl ether; 60-29-7 | DMC: dimethyl ester carbonic acid; 616-38-6 | DME: methoxymethane; 115-10-6 | EBENZENE: phenylethane; 100-41-4 | EGLYCOL: 1,2-ethandiol; 107-21-1 | ETHANE: ethane; 74-84-0 | ETHANOL: ethyl alcohol; 64-17-5 | ETHYLENE: ethene; 74-85-1 | ETHYLENEOXIDE: ethylene oxide; 75-21-8 | FLUORINE: fluorine; 7782-41-4 | H2S: hydrogen sulfide; 7783-06-4 | HCL: hydrogen chloride; 7647-01-0 | HELIUM: helium-4; 7440-59-7 | HEPTANE: heptane; 142-82-5 | HEXANE: hexane; 110-54-3 | HYDROGEN: hydrogen (normal); 1333-74-0 | IBUTENE: 2-methyl-1-propene; 115-11-7 | IHEXANE: 2-methylpentane; 107-83-5 | IOCTANE: 2,2,4-trimethylpentane; 540-84-1 | IPENTANE: 2-methylbutane; 78-78-4 | ISOBUTAN: 2-methylpropane; 75-28-5 | KRYPTON: krypton; 7439-90-9 | MD2M: decamethyltetrasiloxane; 141-62-8 | MD3M: dodecamethylpentasiloxane; 141-63-9 | MD4M: tetradecamethylhexasiloxane; 107-52-8 | MDM: octamethyltrisiloxane; 107-51-7 | MEA: ethanolamine; 141-43-5 | METHANE: methane; 74-82-8 | METHANOL: methanol; 67-56-1 | MILPRF23699: mIL-PRF-23699; 1-1-1 | MLINOLEA: methyl (*Z*,*Z*)-9,12-octadecadienoate; 112-63-0 | MLINOLEN: methyl (*Z*,*Z*,*Z*)-9,12,15-octadecatrienoate; 301-00-8 | MM: hexamethyldisiloxane; 107-46-0 | MOLEATE: methyl cis-9-octadecenoate; 112-62-9 | MPALMITA: methyl hexadecanoate; 112-39-0 | MSTEARAT: methyl octadecanoate; 112-61-8 | MXYLENE: 1,3-dimethylbenzene; 108-38-3 | N2O: dinitrogen monoxide; 10024-97-2 | NEON: neon; 7440-01-9 | NEOPENTN: 2,2-dimethylpropane; 463-82-1 | NF3: nitrogen trifluoride; 7783-54-2 | NITROGEN: nitrogen; 7727-37-9 | NONANE: nonane; 111-84-2 | NOVEC649:1,1,1,2,2,4,5,5,5-nonafluoro-4-(trifluoromethyl)-3-pentanone; 756-13-8| OCTANE: octane; 111-65-9 | ORTHOHYD: orthohydrogen; 1333-74-0o | OXYGEN: oxygen; 7782-44-7 | OXYLENE: 1,2-dimethylbenzene; 95-47-6 | PARAHYD: parahydrogen; 1333-74-0p | PENTANE: pentane; 109-66-0 | POE5: pentaerythritol tetrapentanoate; 15834-04-5 | POE7: pentaerythritol tetraheptanoate; 25811-35-2 | POE9: pentaerythritol tetranonanoate; 14450-05-6 | PROPADIENE: 1,2-propadiene; 463-49-0 | PROPANE: propane; 74-98-6 | PROPYLEN: propene; 115-07-1 | PROPYLENEOXIDE: 1,2-epoxypropane; 75-56-9 | PROPYNE: propyne; 74-99-7 | PXYLENE: 1,4-dimethylbenzene; 106-42-3 | R11: trichlorofluoromethane; 75-69-4 | R1123: trifluoroethylene; 359-11-5 | R113: 1,1,2-trichloro-1,2,2-trifluoroethane; 76-13-1 | R114: 1,2-dichloro-1,1,2,2-tetrafluoroethane; 76-14-2 | R115: chloropentafluoroethane; 76-15-3 | R116: hexafluoroethane; 76-16-4 | R12: dichlorodifluoromethane; 75-71-8 | R1216: hexafluoropropene; 116-15-4 | R1224YDZ: (*Z*)-1-chloro-2,3,3,3-tetrafluoropropene; 108 | R123:2,2-dichloro-1,1,1-trifluoroethane; 306-83-2 | R1233ZDE: trans-1-chloro-3,3,3-trifluoro-1-propene; 110 | R1234YF: 2,3,3,3-tetrafluoroprop-1-ene; 754-12-1 | R1234ZEE: trans-1,3,3,3-tetrafluoropropene; 29118-24-9 | R1234ZEZ: cis-1,3,3,3-tetrafluoropropene; 29118-25-0 | R124:1-chloro-1,2,2,2-tetrafluoroethane; 2837-89-0 | R1243ZF: 3,3,3-trifluoropropene; 677-21-4 | R125: pentafluoroethane; 354-33-6 | R13: chlorotrifluoromethane; 75-72-9 | R1336MZZZ: (*Z*)-1,1,1,4,4,4-hexafluoro-2-butene; 692-49-9 | R134A: 1,1,1,2-tetrafluoroethane; 811-97-2 | R14: tetrafluoromethane; 75-73-0 | R141B: 1,1-dichloro-1-fluoroethane; 1717-00-6 | R142B: 1-chloro-1,1-difluoroethane; 75-68-3 | R143A: 1,1,1-trifluoroethane; 420-46-2 | R150:1,2-dichloroethane; 107-06-2 | R152A: 1,1-difluoroethane; 75-37-6 | R161: fluoroethane; 353-36-6 | R21: dichlorofluoromethane; 75-43-4 | R218: octafluoropropane; 76-19-7 | R22: chlorodifluoromethane; 75-45-6 | R227EA: 1,1,1,2,3,3,3-heptafluoropropane; 431-89-0 | R23: trifluoromethane; 75-46-7 | R236EA: 1,1,1,2,3,3-hexafluoropropane; 431-63-0 | R236FA: 1,1,1,3,3,3-hexafluoropropane; 690-39-1 | R245CA: 1,1,2,2,3-pentafluoropropane; 679-86-7 | R245FA: 1,1,1,3,3-pentafluoropropane; 460-73-1 | *R*32: difluoromethane; 75-10-5 | R365MFC: 1,1,1,3,3-pentafluorobutane; 406-58-6 | R40: methyl chloride; 74-87-3 | R41: fluoromethane; 593-53-3 | RC318: octafluorocyclobutane; 115-25-3 | RE143A: methyl trifluoromethyl ether; 421-14-7 | RE245CB2: methyl-pentafluoroethyl-ether; 22410-44-2 | RE245FA2:2,2,2-trifluoroethyl-difluoromethyl-ether; 1885-48-9 | RE347MCC: 1,1,1,2,2,3,3-heptafluoro-3-methoxypropane; 375-03-1 | SF6: sulfur hexafluoride; 2551-62-4 | SO2: sulfur dioxide; 7446-09-5 | T2BUTENE: trans-2-butene; 624-64-6 | TOLUENE: methylbenzene; 108-88-3 | VINYLCHLORIDE: chloroethylene; 75-01-4 | WATER: water; 7732-18-5 | XENON: xenon; 7440-63-3.

## Supporting Information Available

main.py (PY): implementation of the core of the RES model; Dilute_gas_viscosity.txt (TXT): parameters for the calculation of the dilute gas viscosity; RES_Parameter.txt (TXT): parameters for the models; Samples_* (TXT): all files beginning with “Samples_” are sample calculations that may be used to verify the model; Data_evaluation_REF_15.txt (TXT): various statistics concerned with the data used for the pure fluid calculations; Data_evaluation_mix.txt (TXT): various statistics concerned with the data used for the mixtures calculations; Table_multi.txt (TXT): information about the experimental data of mixtures; Table_pure_REF_15.txt (TXT): various statistics of the model qualities for the pure fluids; Fluid_Constants.txt (TXT): fluid constants used; pure-fluid data and literature.docx (DOCX): detailed information on pure fluid data and the literature; figure_pure_devs (folder/*PNG): relative deviation for all used data points for the RES model and REFPROP models for all substances; figure_pure_groups (folder/*PNG): η_res_^+^ + 1 as a function of  for one group each; mix_dev_exp_res_ecs (folder/*PNG): relative deviations for all mixtures for the RES model and the REFPROP models individually, as well as their data sources; mix_s_eta_all_data (folder/*PNG): η_res_^+^ + 1 as a function of  for each mixture individually for all data, as well as the data sources; mix_s_eta_select_data (folder/*PNG): η_res_^+^ + 1 as a function of  for each mixtures individually for the filtered data, as well as the data sources; pure_dev_exp_res_ecs (folder/*PNG): relative deviations for all pure fluids for the RES model and the REFPROP models individually, as well as the data sources; pure_s_eta_all_data (folder/*PNG): η_res_^+^ + 1 as a function of  for each pure fluid individually for all data, as well as the data sources; pure_s_eta_fitted_data (folder/*PNG): η_res_^+^ + 1 as a function of  for each pure fluid individually for the filtered data, as well as the data sources (ZIP)

## References

[ref1] Bell I. H.; Messerly R.; Thol M.; Costigliola L.; Dyre J. C. Modified entropy scaling of the transport properties of the Lennard-Jones fluid. J. Phys. Chem. B 2019, 123, 6345–6363. 10.1021/acs.jpcb.9b05808. 31241958 PMC7147083

[ref2] Saric D.; Bell I. H.; Guevara-Carrion G.; Vrabec J. Influence of repulsion on entropy scaling and density scaling of monatomic fluids. J. Chem. Phys. 2024, 160, 104503 10.1063/5.0196592. 38456532

[ref3] Bell I. H. Entropy scaling of viscosity–I: a case study of propane. J. Chem. Eng. Data 2020, 65, 3203–3215. 10.1021/acs.jced.0c00209. PMC7754705 33364635

[ref4] Bell I. H. Entropy scaling of viscosity–II: predictive scheme for normal alkanes. J. Chem. Eng. Data 2020, 65, 5606–5616. 10.1021/acs.jced.0c00749. PMC8191377 34121765

[ref5] Binti Mohd Taib M.; Trusler J. P. M. Residual entropy model for predicting the viscosities of dense fluid mixtures. J. Chem. Phys. 2020, 152, 164104 10.1063/5.0002242. 32357798

[ref6] Yang X.; Xiao X.; May E. F.; Bell I. H. Entropy scaling of viscosity–III: application to refrigerants and their mixtures. J. Chem. Eng. Data 2021, 66, 1385–1398. 10.1021/acs.jced.0c01009.

[ref7] Liu H.; Yang F.; Yang Z.; Duan Y. Modeling the viscosity of hydrofluorocarbons, hydrofluoroolefins and their binary mixtures using residual entropy scaling and cubic-plus-association equation of state. J. Mol. Liq. 2020, 308, 113027 10.1016/j.molliq.2020.113027.

[ref8] Bell I.; Laesecke A. In Viscosity of Refrigerants and Other Working Fluids from Residual Entropy Scaling, 16th International Refrigeration and Air Conditioning Conference at Purdue, 2016; p 2287. https://tsapps.nist.gov/publication/get_pdf.cfm?pub_id=920655.

[ref9] Wang X.; Wright E.; Gao N.; Li Y. Evaluation on excess entropy scaling method predicting thermal transport properties of liquid HFC/HFO refrigerants. J. Therm. Sci. 2022, 31, 1465–1475. 10.1007/s11630-020-1383-2.

[ref10] Bell I. H. Probing the link between residual entropy and viscosity of molecular fluids and model potentials. Proc. Natl. Acad. Sci. U.S.A. 2019, 116, 4070–4079. 10.1073/pnas.1815943116. 30770449 PMC6410835

[ref11] Lötgering-Lin O.; Fischer M.; Hopp M.; Gross J. Pure substance and mixture viscosities based on entropy scaling and an analytic equation of state. Ind. Eng. Chem. Res. 2018, 57, 4095–4114. 10.1021/acs.iecr.7b04871.

[ref12] Yang X.; Xiao X.; Thol M.; Richter M.; Bell I. In A Residual Entropy Scaling Approach for Viscosity of Refrigerants, Other Fluids and Their Mixtures, 26th International Congress of Refrigeration, 2023.

[ref13] Yang X.; Xiao X.; Thol M.; Richter M.; Bell I. H. Linking viscosity to equations of state using residual entropy scaling theory. Int. J. Thermophys. 2022, 43, 183 10.1007/s10765-022-03096-9.

[ref14] Dehlouz A.; Privat R.; Galliero G.; Bonnissel M.; Jaubert J.-N. Revisiting the entropy-scaling concept for shear-viscosity estimation from cubic and SAFT equations of state: application to pure fluids in gas, liquid and supercritical states. Ind. Eng. Chem. Res. 2021, 60, 12719–12739. 10.1021/acs.iecr.1c01386.

[ref15] Liu H.; Yang F.; Yang X.; Yang Z.; Duan Y. Modeling the thermal conductivity of hydrofluorocarbons, hydrofluoroolefins and their binary mixtures using residual entropy scaling and cubic-plus-association equation of state. J. Mol. Liq. 2021, 330, 115612 10.1016/j.molliq.2021.115612.

[ref16] Fouad W. A. Thermal conductivity of pure fluids and multicomponent mixtures using residual entropy scaling with PC-SAFT–application to refrigerant blends. J. Chem. Eng. Data 2020, 65, 5688–5697. 10.1021/acs.jced.0c00682.

[ref17] Hopp M.; Gross J. Thermal conductivity of real substances from excess entropy scaling using PCP-SAFT. Ind. Eng. Chem. Res. 2017, 56, 4527–4538. 10.1021/acs.iecr.6b04289.

[ref18] Hopp M.; Mele J.; Hellmann R.; Gross J. Thermal conductivity via entropy scaling: an approach that captures the effect of intramolecular degrees of freedom. Ind. Eng. Chem. Res. 2019, 58, 18432–18438. 10.1021/acs.iecr.9b03998.

[ref19] Yang X.; Kim D.; May E. F.; Bell I. H. Entropy scaling of thermal conductivity: application to refrigerants and their mixtures. Ind. Eng. Chem. Res. 2021, 60, 13052–13070. 10.1021/acs.iecr.1c02154.

[ref20] Huber M.; Harvey A.; Lemmon E.; Hardin G.; Bell I.; McLinden M. NIST Standard Reference Database 23: Reference Fluid Thermodynamic and Transport Properties (REFPROP), Version 10, 2018. https://www.nist.gov/srd/refprop.

[ref21] Urbaneck T.; Matthes M.; Richter M.; Hempel O.; Safarik M.; Franzke U. Research Platform Refrigeration and Energy Technology (KETEC) 2022, 2022. www.ketec.online.

[ref22] Yang X.; Richter M. OilMixProp 1.0: Package for Thermophysical Properties of Oils, Common Fluids, and Their Mixtures. IOP Conf. Ser. Mater. Sci. Eng. 2024, 1322, 012009 10.1088/1757-899X/1322/1/012009.

[ref23] Hirschfelder J. O.; Curtiss C. F.; Bird R. B. Molecular Theory of Gases and Liquids; John Wiley & Sons, Ltd, 1966.

[ref24] Neufeld P. D.; Janzen A. R.; Aziz R. A. Empirical equations to calculate 16 of the transport collision integrals Ω^(*l*,*s*)^*^^ for the Lennard-Jones (12–6) potential. J. Chem. Phys. 1972, 57, 1100–1102. 10.1063/1.1678363.

[ref25] Chung T. H.; Lee L. L.; Starling K. E. Applications of kinetic gas theories and multiparameter correlation for prediction of dilute gas viscosity and thermal conductivity. IInd. Eng. Chem. Fundam. 1984, 23, 8–13. 10.1021/i100013a002.

[ref26] Tiesinga E.; Mohr P. J.; Newell D. B.; Taylor B. N. CODATA recommended values of the fundamental physical constants: 2018. J. Phys. Chem. Ref. Data 2021, 50, 033105 10.1063/5.0064853. 36726646 PMC9888147

[ref27] Bell I. H.; Wronski J.; Quoilin S.; Lemort V. Pure and pseudo-pure fluid thermophysical property evaluation and the open-source thermophysical property library CoolProp. Ind. Eng. Chem. Res. 2014, 53, 2498–2508. 10.1021/ie4033999. 24623957 PMC3944605

[ref28] Wilke C. R. A viscosity equation for gas mixtures. J. Chem. Phys. 1950, 18, 517–519. 10.1063/1.1747673.

[ref29] Glowinski R.; Marroco A. Sur l’approximation, par éléments finis d’ordre un, et la résolution, par pénalisation-dualité, d’une classe de problèmes de Dirichlet non linéaires. Rev. Fr. Autom. Inf. Rech. Oper. 1975, 9, 41–76. 10.1051/m2an/197509r200411.

[ref30] Gabay D.; Mercier B. A dual algorithm for the solution of nonlinear variational problems via finite element approximations. Comput. Math. Appl. 1976, 2, 17–40. 10.1016/0898-1221(76)90003-1.

[ref31] Boyd S.; Parikh N.; Chu E.; Peleato B.; Eckstein J. Distributed optimization and statistical learning via the alternating direction method of multipliers. Found. Trends Mach. Learn. 2010, 3, 1–122. 10.1561/2200000016.

[ref32] Lemmon E. W.; Jacobsen R. T. Viscosity and thermal conductivity equations for nitrogen, oxygen, argon, and air. Int. J. Thermophys. 2004, 25, 21–69. 10.1023/B:IJOT.0000022327.04529.f3.

[ref33] Avgeri S.; Assael M. J.; Huber M. L.; Perkins R. A. Reference correlation of the viscosity of toluene from the triple point to 675 K and up to 500 MPa. J. Phys. Chem. Ref. Data 2015, 44, 033101 10.1063/1.4926955.

[ref34] Herrmann S.; Vogel E. New formulation for the viscosity of n-butane. J. Phys. Chem. Ref. Data 2018, 47, 013104 10.1063/1.5020802.

[ref35] Assael M. J.; Papalas T. B.; Huber M. L. Reference correlations for the viscosity and thermal conductivity of n-undecane. J. Phys. Chem. Ref. Data 2017, 46, 033103 10.1063/1.4996885. 29230074 PMC5721360

[ref36] Huber M. L.; Laesecke A.; Perkins R. Transport properties of n-dodecane. Energy Fuels 2004, 18, 968–975. 10.1021/ef034109e.

[ref37] Meng X. Y.; Sun Y. K.; Cao F. L.; Wu J. T.; Vesovic V. Reference correlation of the viscosity of n-hexadecane from the triple point to 673 K and up to 425 MPa. J. Phys. Chem. Ref. Data 2018, 47, 033102 10.1063/1.5039595.

[ref38] Laesecke A.; Muzny C. D. Reference correlation for the viscosity of carbon dioxide. J. Phys. Chem. Ref. Data 2017, 46, 013107 10.1063/1.4977429. 28736460 PMC5514612

[ref39] Tariq U.; Jusoh A. R. B.; Riesco N.; Vesovic V. Reference correlation of the viscosity of cyclohexane from the triple point to 700 K and up to 110 MPa. J. Phys. Chem. Ref. Data 2014, 43, 033101 10.1063/1.4891103.

[ref40] Muzny C. D.; Huber M. L.; Kazakov A. F. Correlation for the viscosity of normal hydrogen obtained from symbolic regression. J. Chem. Eng. Data 2013, 58, 969–979. 10.1021/je301273j.

[ref41] Huber M. L.; Laesecke A.; Xiang H. W. Viscosity correlations for minor constituent fluids in natural gas: n-octane, n-nonane and n-decane. Fluid Phase Equilib. 2005, 228–229, 401–408. 10.1016/j.fluid.2005.03.008.

[ref42] Meng X.; Zhang J.; Wu J.; Liu Z. Experimental measurement and modeling of the viscosity of dimethyl ether. J. Chem. Eng. Data 2012, 57, 988–993. 10.1021/je201297j.

[ref43] Meng X. Y.; Cao F. L.; Wu J. T.; Vesovic V. Reference correlation of the viscosity of ethylbenzene from the triple point to 673 K and up to 110 MPa. J. Phys. Chem. Ref. Data 2017, 46, 013101 10.1063/1.4973501.

[ref44] Vogel E.; Span R.; Herrmann S. Reference correlation for the viscosity of ethane. J. Phys. Chem. Ref. Data 2015, 44, 043101 10.1063/1.4930838.

[ref45] Kiselev S. B.; Ely J. F.; Abdulagatov I. M.; Huber M. L. Generalized SAFT-DFT/DMT model for the thermodynamic, interfacial, and transport properties of associating fluids: application for n-alkanols. Ind. Eng. Chem. Res. 2005, 44, 6916–6927. 10.1021/ie050010e.

[ref46] Holland P. M.; Eaton B. E.; Hanley H. J. M. A correlation of the viscosity and thermal conductivity data of gaseous and liquid ethylene. J. Phys. Chem. Ref. Data 1983, 12, 917–932. 10.1063/1.555701.

[ref47] Schmidt K. A. G.; Quiñones-Cisneros S. E.; Carroll J. J.; Kvamme B. Hydrogen sulfide viscosity modeling. Energy Fuels 2008, 22, 3424–3434. 10.1021/ef700701h.

[ref48] Michailidou E. K.; Assael M. J.; Huber M. L.; Abdulagatov I. M.; Perkins R. A. Reference correlation of the viscosity of n-heptane from the triple point to 600 K and up to 248 MPa. J. Phys. Chem. Ref. Data 2014, 43, 023103 10.1063/1.4875930.

[ref49] Michailidou E. K.; Assael M. J.; Huber M. L.; Perkins R. A. Reference correlation of the viscosity of n-hexane from the triple point to 600 K and up to 100 MPa. J. Phys. Chem. Ref. Data 2013, 42, 033104 10.1063/1.4818980.

[ref50] Vogel E.; Küchenmeister C.; Bich E. Viscosity correlation for isobutane over wide ranges of the fluid region. Int. J. Thermophys. 2000, 21, 343–356. 10.1023/A:1006623310780.

[ref51] Xiang H. W.; Laesecke A.; Huber M. L. A new reference correlation for the viscosity of methanol. J. Phys. Chem. Ref. Data 2006, 35, 1597–1620. 10.1063/1.2360605.

[ref52] Cao F. L.; Meng X. Y.; Wu J. T.; Vesovic V. Reference correlation of the viscosity of meta-xylene from 273 to 673 K and up to 200 MPa. J. Phys. Chem. Ref. Data 2016, 45, 013103 10.1063/1.4941241.

[ref53] Cao F. L.; Meng X. Y.; Wu J. T.; Vesovic V. Reference correlation of the viscosity of ortho-xylene from 273 to 673 K and up to 110 MPa. J. Phys. Chem. Ref. Data 2016, 45, 023102 10.1063/1.4945663.

[ref54] Balogun B.; Riesco N.; Vesovic V. Reference correlation of the viscosity of para-xylene from the triple point to 673 K and up to 110 MPa. J. Phys. Chem. Ref. Data 2015, 44, 013103 10.1063/1.4908048.

[ref55] Tanaka Y.; Sotani T. Thermal conductivity and viscosity of 2,2-dichloro-1,1,1-trifluoroethane (HCFC-123). Int. J. Thermophys. 1996, 17, 293–328. 10.1007/BF01443394.

[ref56] Huber M. L.; Assael M. J. Correlations for the viscosity of 2,3,3,3-tetrafluoroprop-1-ene (R1234yf) and trans-1,3,3,3-tetrafluoropropene (R1234ze(E)). Int. J. Refrig. 2016, 71, 39–45. 10.1016/j.ijrefrig.2016.08.007. 27840461 PMC5103321

[ref57] Huber M. L.; Laesecke A. Correlation for the viscosity of pentafluoroethane (R125) from the triple point to 500 K at pressures up to 60 MPa. Ind. Eng. Chem. Res. 2006, 45, 4447–4453. 10.1021/ie051367l.

[ref58] Huber M. L.; Laesecke A.; Perkins R. A. Model for the viscosity and thermal conductivity of refrigerants, including a new correlation for the viscosity of R134a. Ind. Eng. Chem. Res. 2003, 42, 3163–3178. 10.1021/ie0300880.

[ref59] Tsolakidou C. M.; Assael M. J.; Huber M. L.; Perkins R. A. Correlations for the viscosity and thermal conductivity of ethyl fluoride (R161). J. Phys. Chem. Ref. Data 2017, 46, 023103 10.1063/1.4983027. 28785120 PMC5544035

[ref60] Perkins R. A.; Huber M. L.; Assael M. J. Measurements of the thermal conductivity of 1,1,1,3,3-pentafluoropropane (R245fa) and correlations for the viscosity and thermal conductivity surfaces. J. Chem. Eng. Data 2016, 61, 3286–3294. 10.1021/acs.jced.6b00350.

[ref61] Quiñones-Cisneros S. E.; Huber M. L.; Deiters U. K. Correlation for the viscosity of sulfur hexafluoride (SF6) from the triple point to 1000 K and pressures to 50 MPa. J. Phys. Chem. Ref. Data 2012, 41, 023102-023102-11 10.1063/1.3702441.

[ref62] Huber M. L.; Perkins R. A.; Laesecke A.; Friend D. G.; Sengers J. V.; Assael M. J.; Metaxa I. N.; Vogel E.; Mareš R.; Miyagawa K. New international formulation for the viscosity of H_2_O. J. Phys. Chem. Ref. Data 2009, 38, 101–125. 10.1063/1.3088050.

[ref63] Avgeri S.; Assael M. J.; Huber M. L.; Perkins R. A. Reference correlation of the viscosity of benzene from the triple point to 675 K and up to 300 MPa. J. Phys. Chem. Ref. Data 2014, 43, 033103 10.1063/1.4892935.

[ref64] Kestin J.; Sengers J. V.; Kamgar-Parsi B.; Sengers J. M. H. L. Thermophysical properties of fluid D_2_O. J. Phys. Chem. Ref. Data 1984, 13, 601–609. 10.1063/1.555714.

[ref65] Wen C.; Meng X.; Huber M. L.; Wu J. Measurement and correlation of the viscosity of 1,1,1,2,2,4,5,5,5-nonafluoro-4-(trifluoromethyl)-3-pentanone. J. Chem. Eng. Data 2017, 62, 3603–3609. 10.1021/acs.jced.7b00572. 29311751 PMC5755718

[ref66] Vogel E.; Herrmann S. New formulation for the viscosity of propane. J. Phys. Chem. Ref. Data 2016, 45, 043103 10.1063/1.4966928.

[ref67] Bruno T. J.; Fortin T. J.; Huber M. L.; Laesecke A.; Lemmon E. W.; Mansfield E.; McLinden M. O.; Outcalt S. L.; Perkins R. A.; Urness K. N.; Widegren J. A. Thermophysical Properties of Polyol Ester Lubricants, 2019.

[ref68] Chichester J. C.; Huber M. L. Documentation and Assessment of the Transport Property Model for Mixtures Implemented in NIST REFPROP (Version 8.0), 2008.

[ref69] Huber M. L. Models for Viscosity, Thermal Conductivity, and Surface Tension of Selected Pure Fluids as Implemented in REFPROP v10.0, Techreport Report, 2018.

[ref70] Klein S.; McLinden M.; Laesecke A. An improved extended corresponding states method for estimation of viscosity of pure refrigerants and mixtures. Int. J. Refrig. 1997, 20, 208–217. 10.1016/S0140-7007(96)00073-4.

[ref71] Yang X.; Yang F.; Yang F. Thermo-economic performance limit analysis of combined heat and power systems for optimal working fluid selections. Energy 2023, 272, 127041 10.1016/j.energy.2023.127041.

[ref72] Yang X.; Hanzelmann C.; Feja S.; Trusler J. P. M.; Richter M. Thermophysical property modeling of lubricant oils and their mixtures with refrigerants using a minimal set of experimental data. Ind. Eng. Chem. Res. 2023, 62, 18736–18749. 10.1021/acs.iecr.3c02474.

[ref73] Bell I. H.; Delage-Santacreu S.; Hoang H.; Galliero G. Dynamic crossover in fluids: from hard spheres to molecules. J. Phys. Chem. Lett. 2021, 12, 6411–6417. 10.1021/acs.jpclett.1c01594. 34232673

[ref74] Bell I. H.; Leachman J. W.; Rigosi A. F.; Hill H. M. Quantum entropic effects in the liquid viscosities of hydrogen, deuterium, and neon. Phys. Fluids 2023, 35, 081703 10.1063/5.0164037.

