# Supplementary figures and images for: Entropy Scaling of Viscosity IV—Application to 124 Industrially Important Fluids (part 2 of 2)
Source: J Chem Eng Data. 2025 Jan 10;70(2):727–42. doi: 10.1021/acs.jced.4c00451 (PMC11831593; doi:10.1021/acs.jced.4c00451)

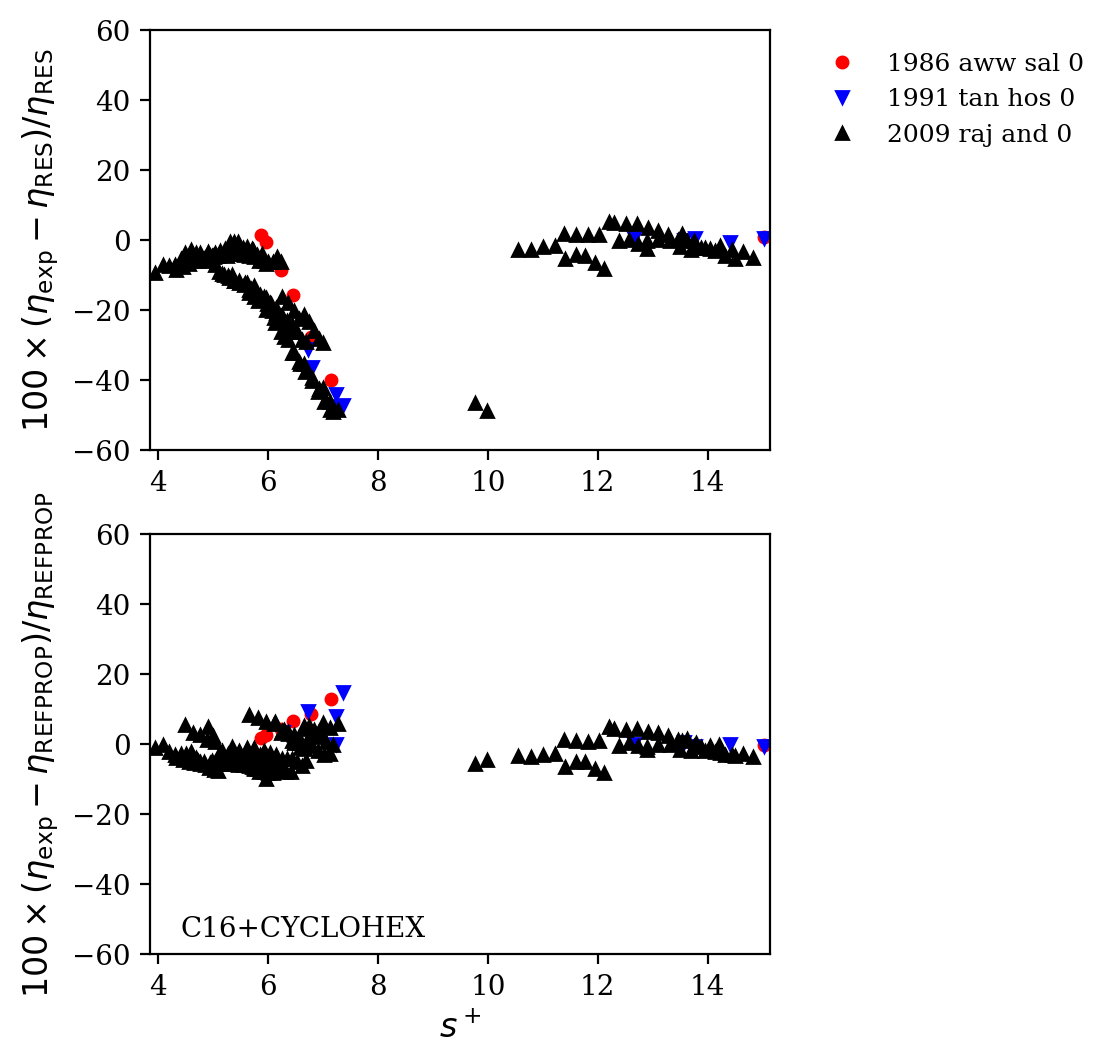

Supplement: Supplementary file 1 — je4c00451_si_001.zip [file je4c00451_si_001.zip › supporting_information/mix_dev_exp_res_ecs/C16+CYCLOHEX.png]

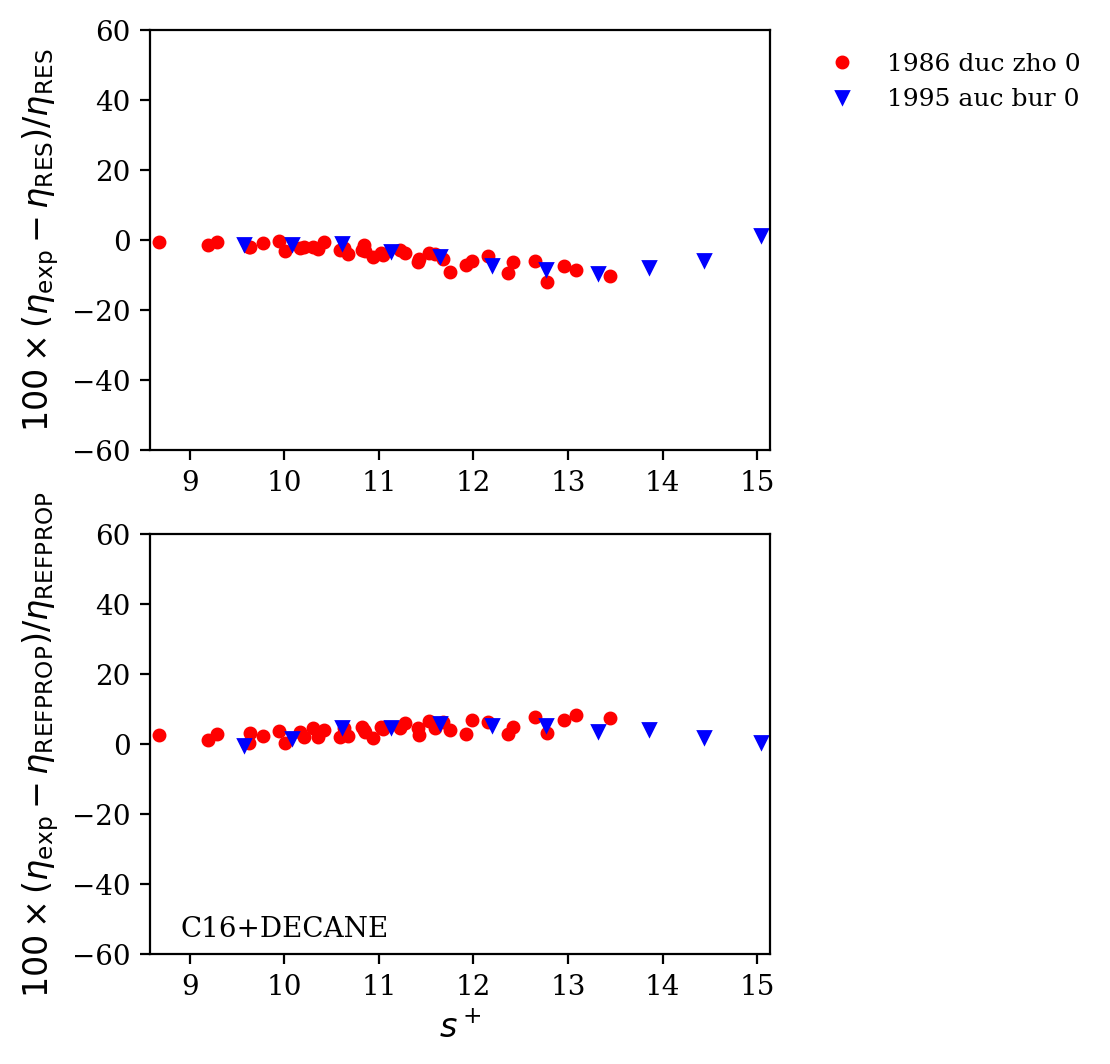

Supplement: Supplementary file 1 — je4c00451_si_001.zip [file je4c00451_si_001.zip › supporting_information/mix_dev_exp_res_ecs/C16+DECANE.png]

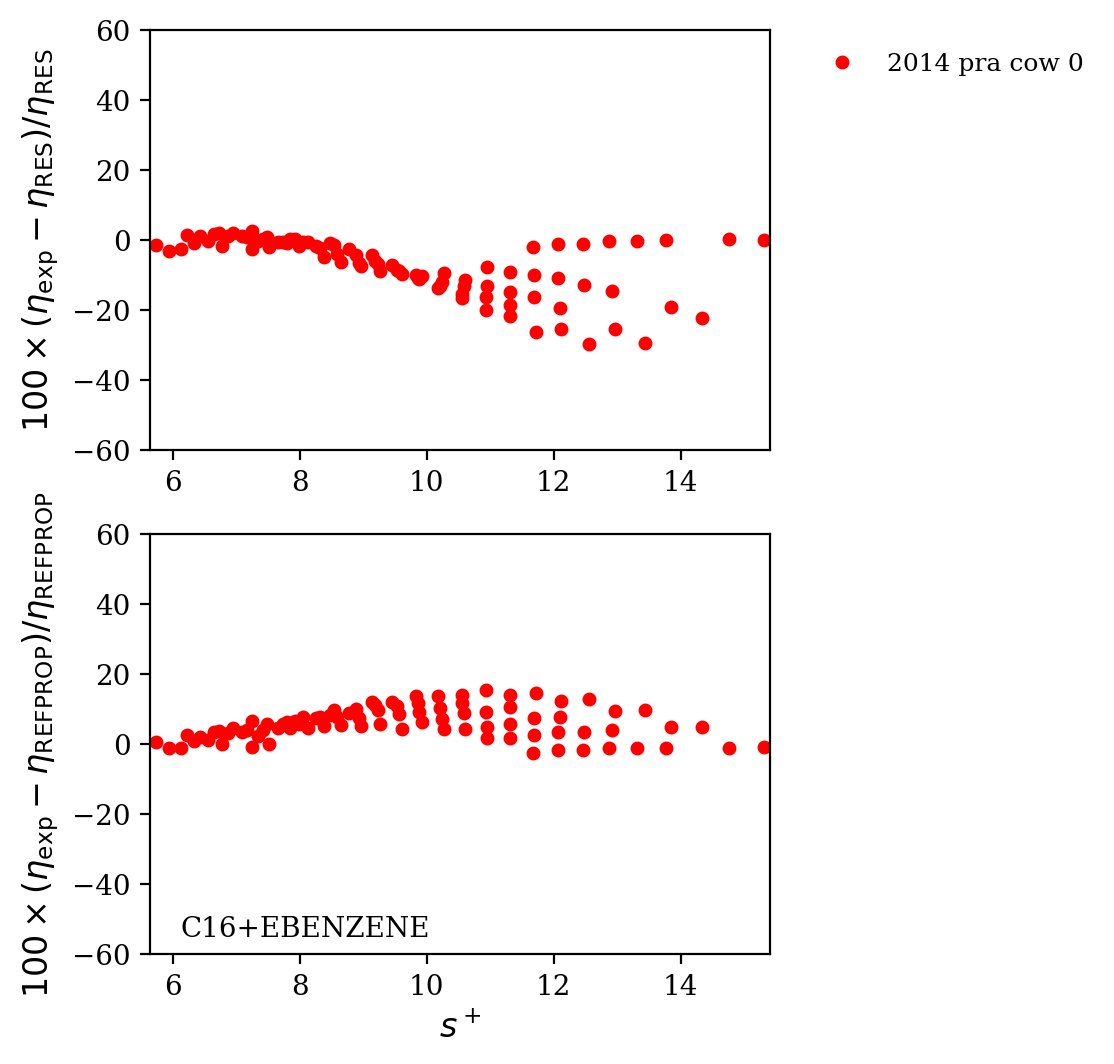

Supplement: Supplementary file 1 — je4c00451_si_001.zip [file je4c00451_si_001.zip › supporting_information/mix_dev_exp_res_ecs/C16+EBENZENE.png]

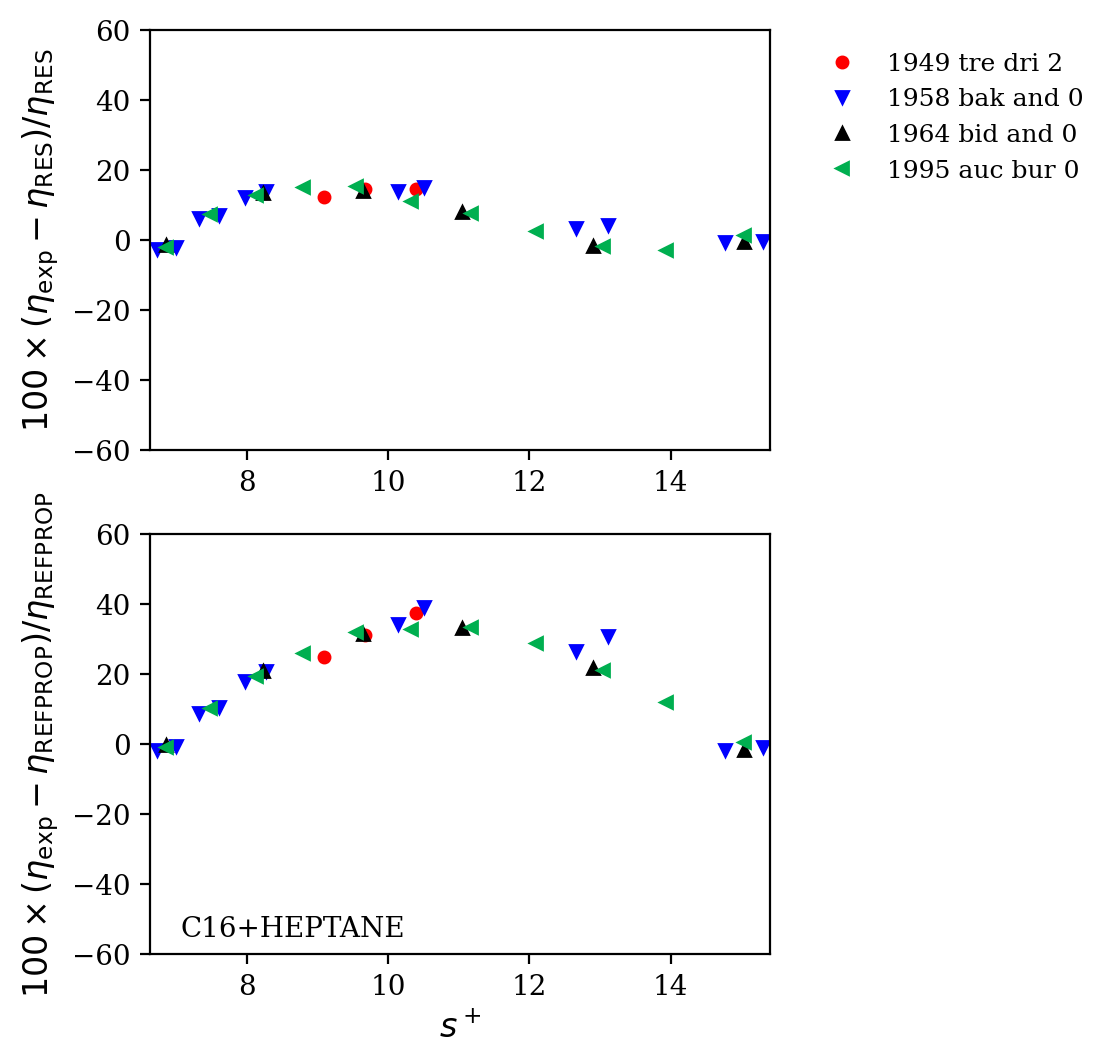

Supplement: Supplementary file 1 — je4c00451_si_001.zip [file je4c00451_si_001.zip › supporting_information/mix_dev_exp_res_ecs/C16+HEPTANE.png]

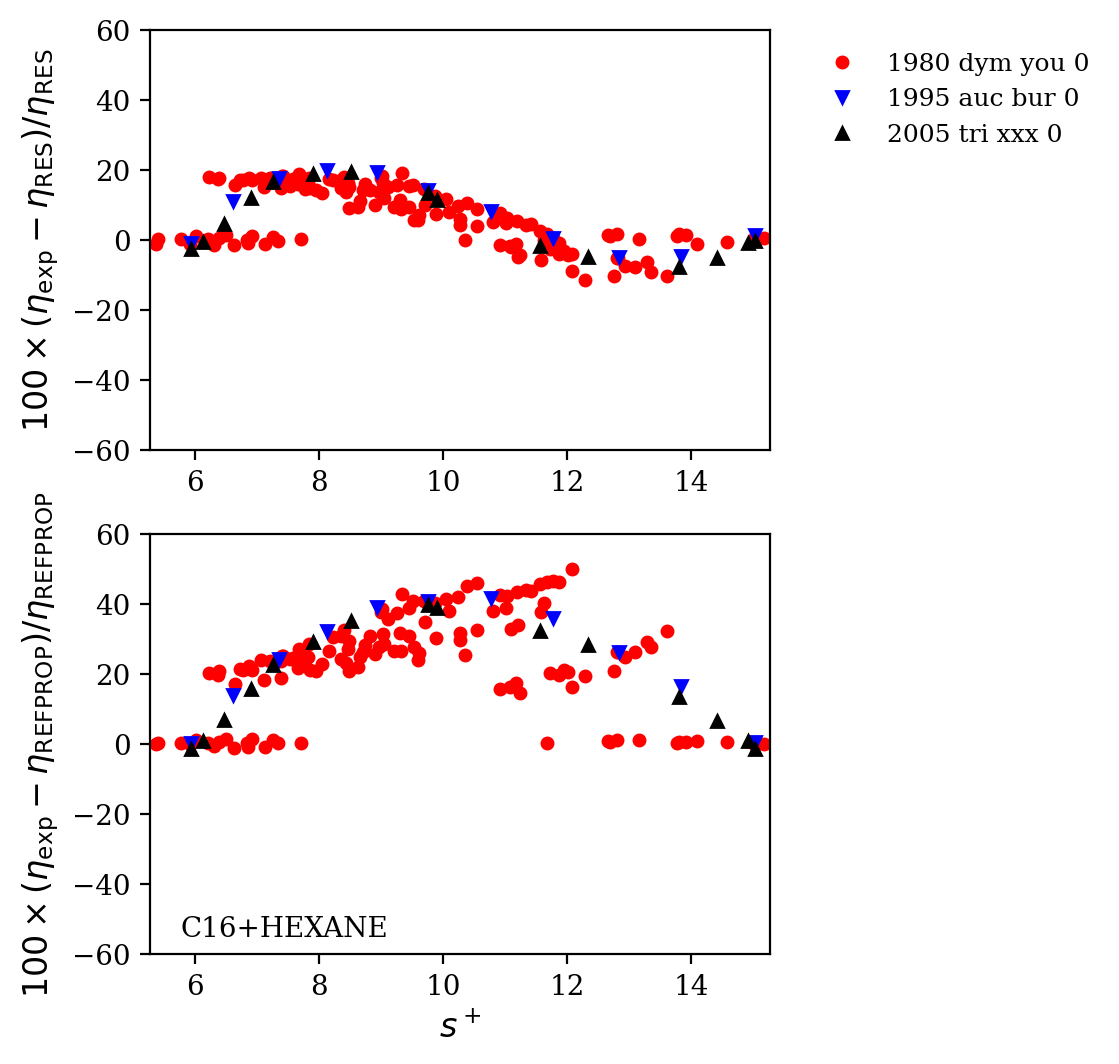

Supplement: Supplementary file 1 — je4c00451_si_001.zip [file je4c00451_si_001.zip › supporting_information/mix_dev_exp_res_ecs/C16+HEXANE.png]

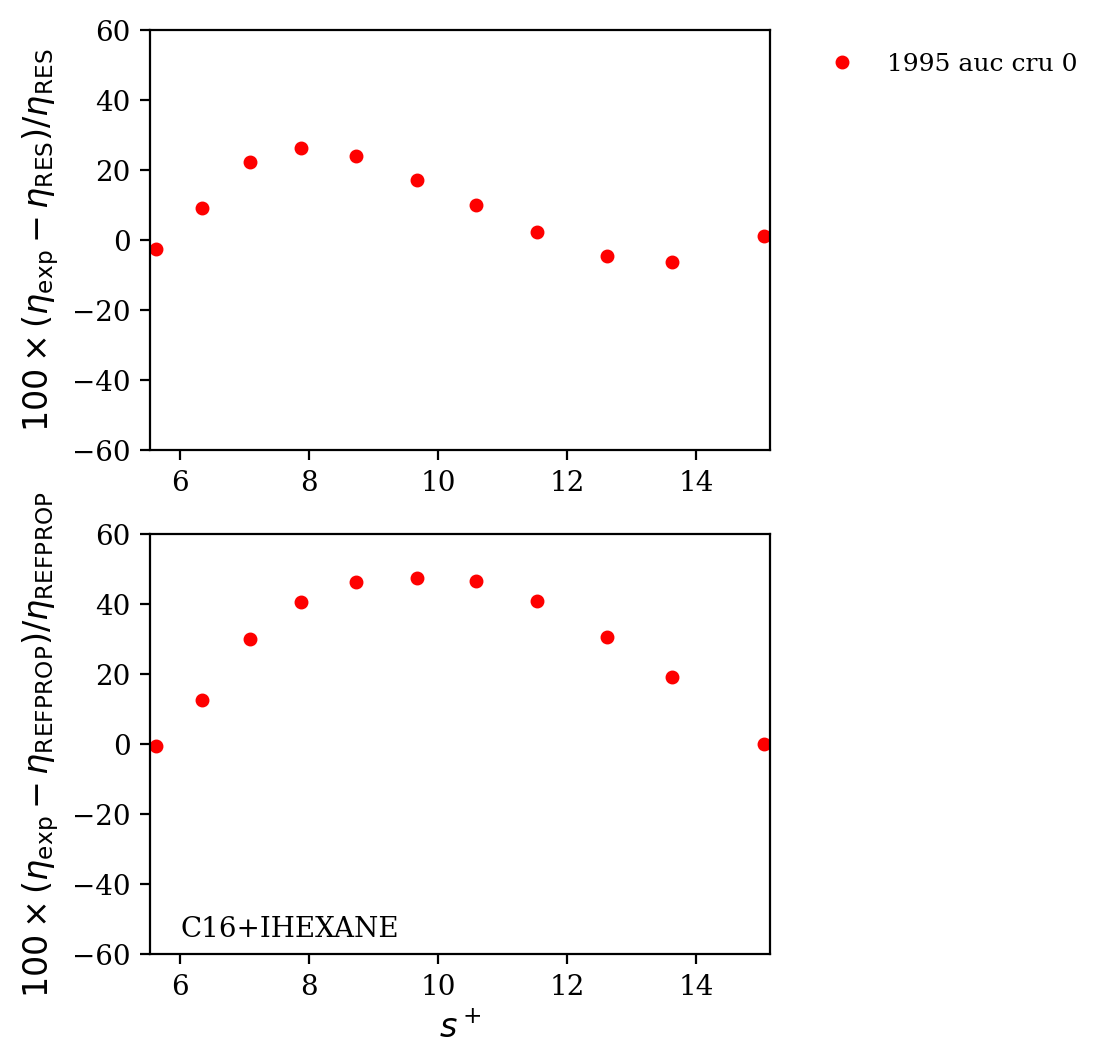

Supplement: Supplementary file 1 — je4c00451_si_001.zip [file je4c00451_si_001.zip › supporting_information/mix_dev_exp_res_ecs/C16+IHEXANE.png]

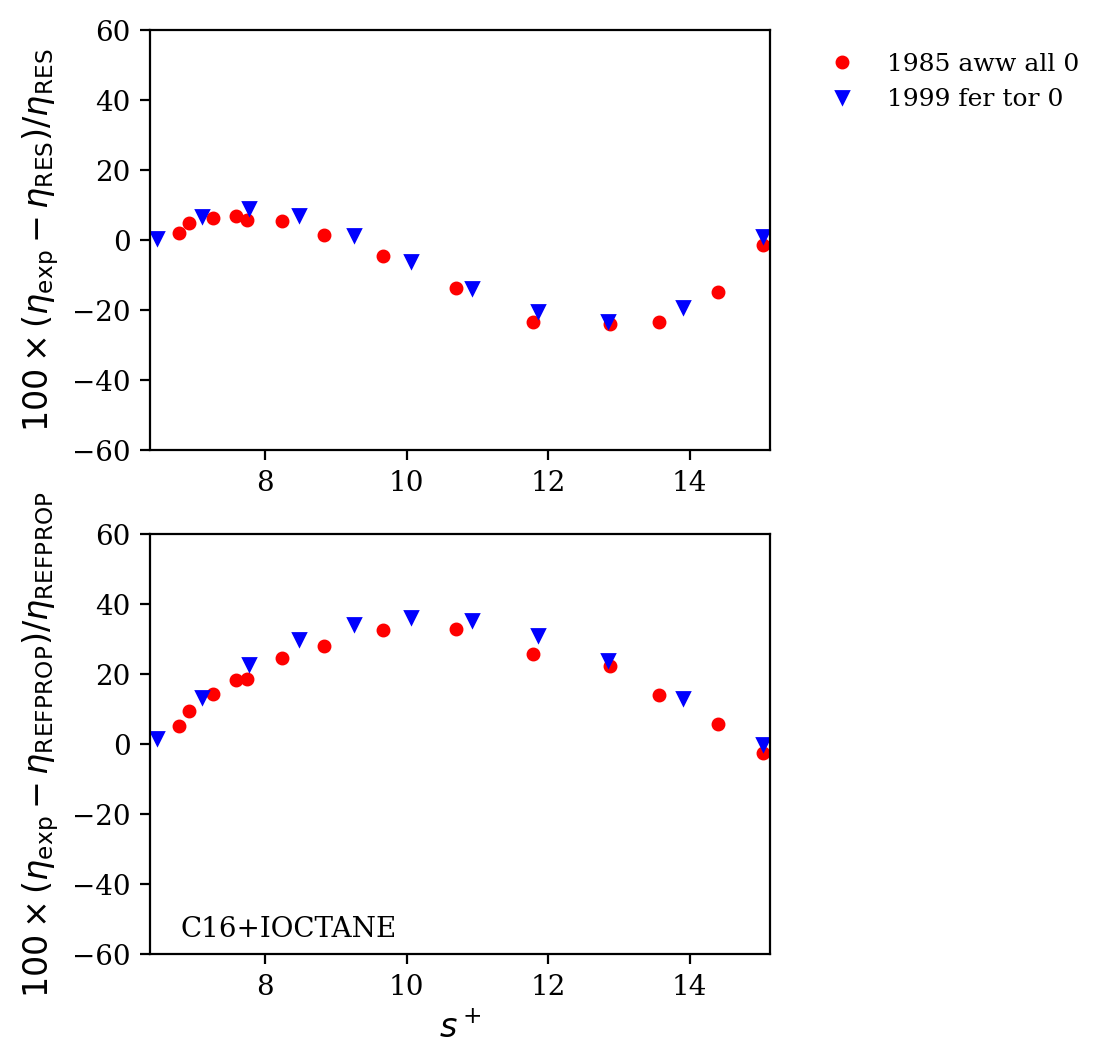

Supplement: Supplementary file 1 — je4c00451_si_001.zip [file je4c00451_si_001.zip › supporting_information/mix_dev_exp_res_ecs/C16+IOCTANE.png]

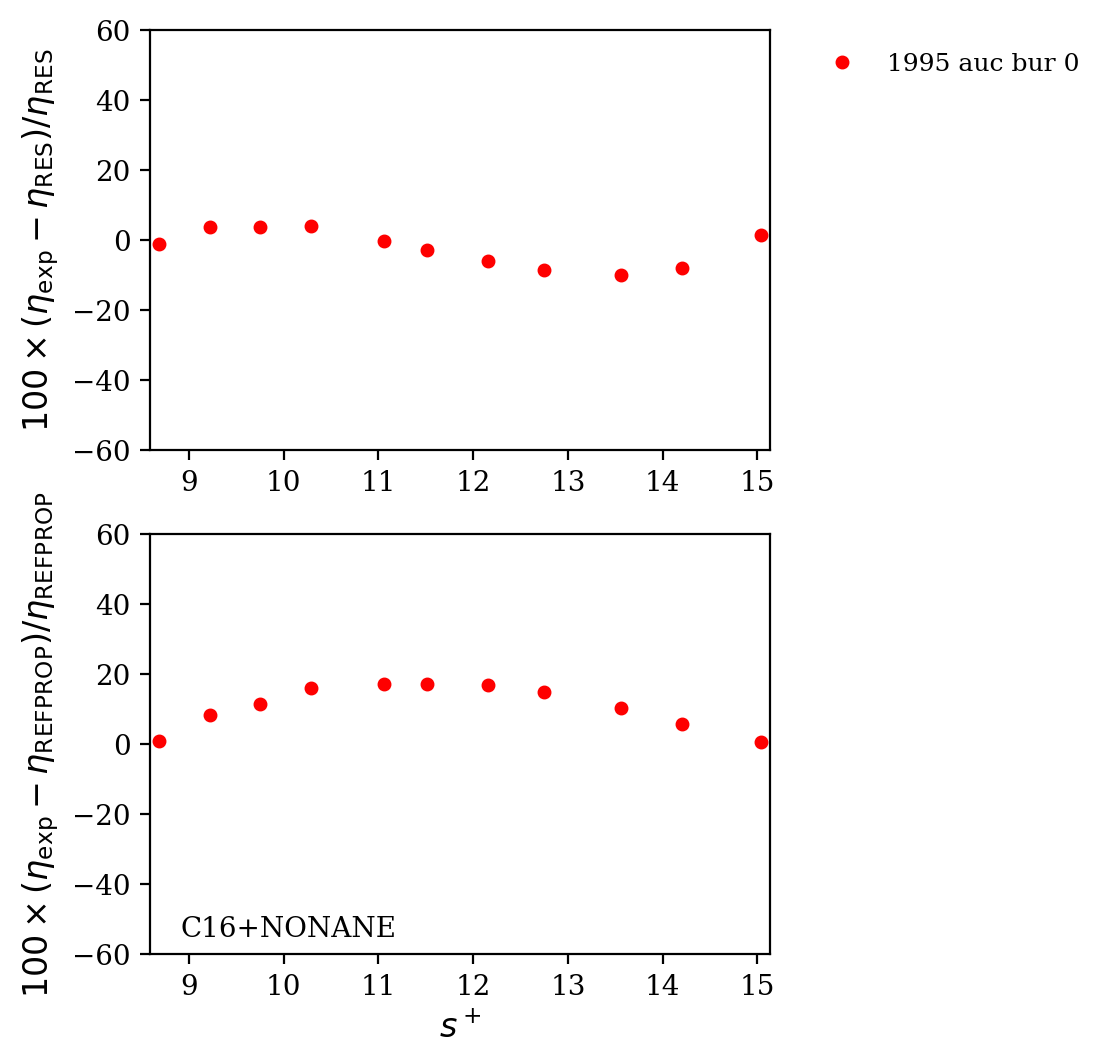

Supplement: Supplementary file 1 — je4c00451_si_001.zip [file je4c00451_si_001.zip › supporting_information/mix_dev_exp_res_ecs/C16+NONANE.png]

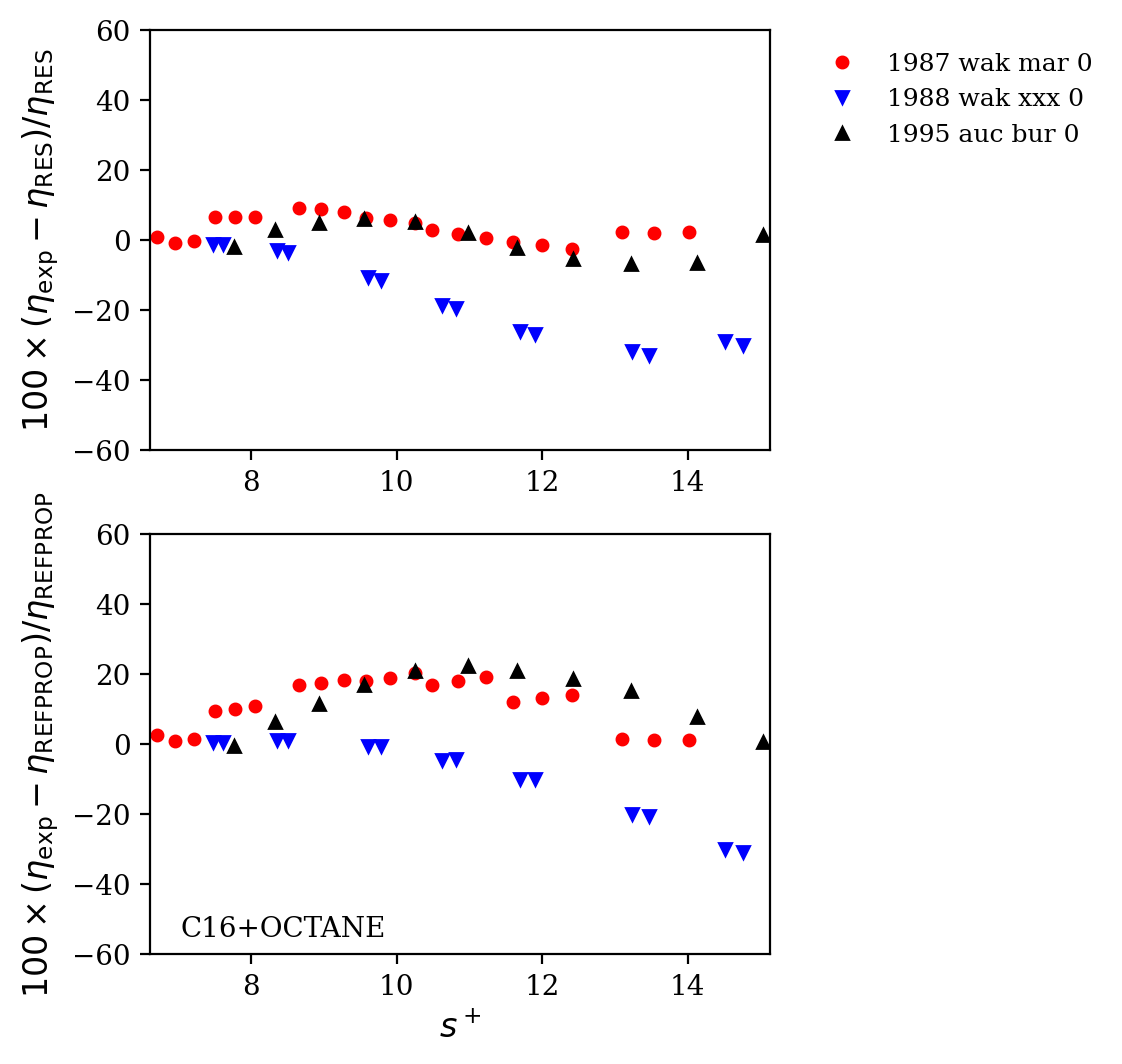

Supplement: Supplementary file 1 — je4c00451_si_001.zip [file je4c00451_si_001.zip › supporting_information/mix_dev_exp_res_ecs/C16+OCTANE.png]

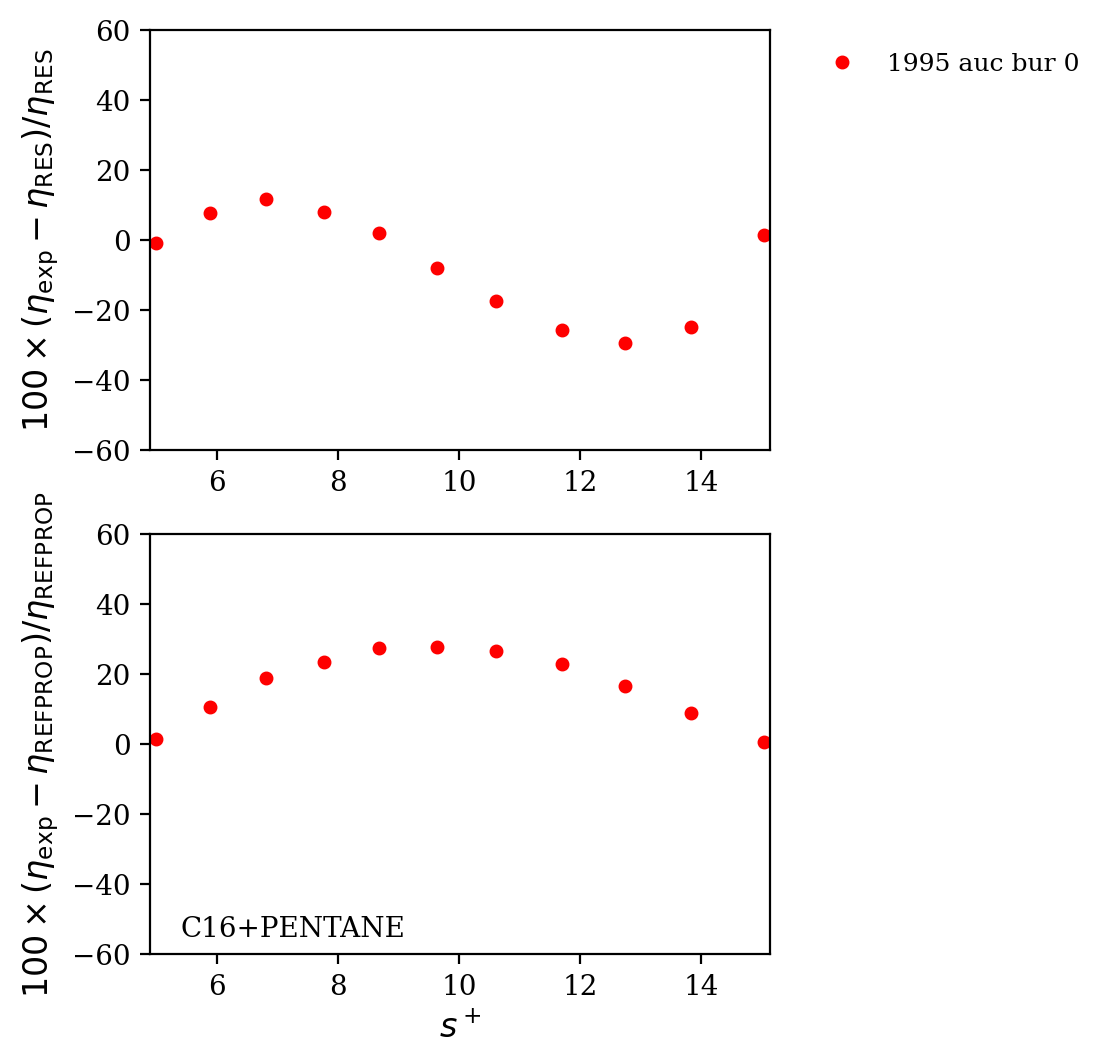

Supplement: Supplementary file 1 — je4c00451_si_001.zip [file je4c00451_si_001.zip › supporting_information/mix_dev_exp_res_ecs/C16+PENTANE.png]

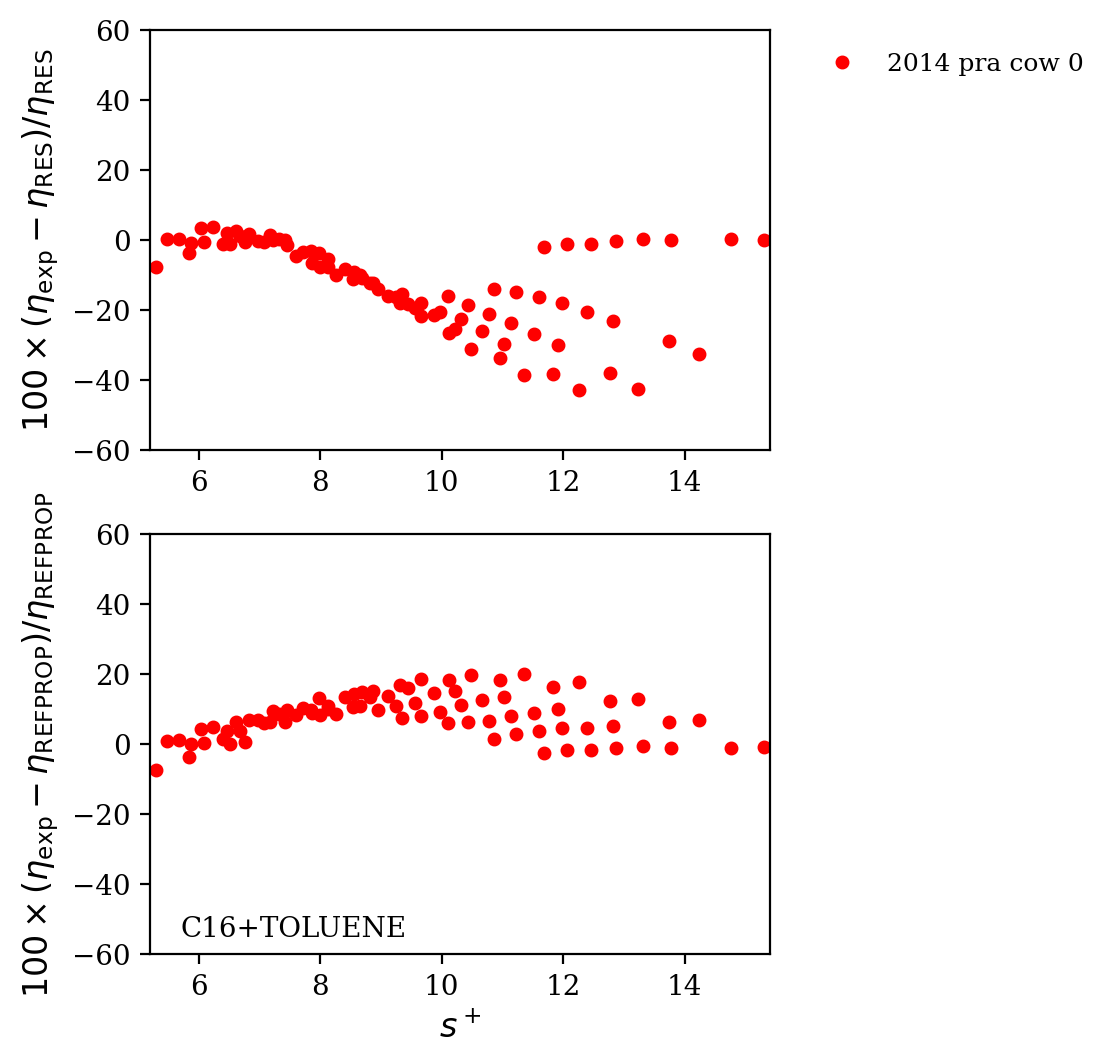

Supplement: Supplementary file 1 — je4c00451_si_001.zip [file je4c00451_si_001.zip › supporting_information/mix_dev_exp_res_ecs/C16+TOLUENE.png]

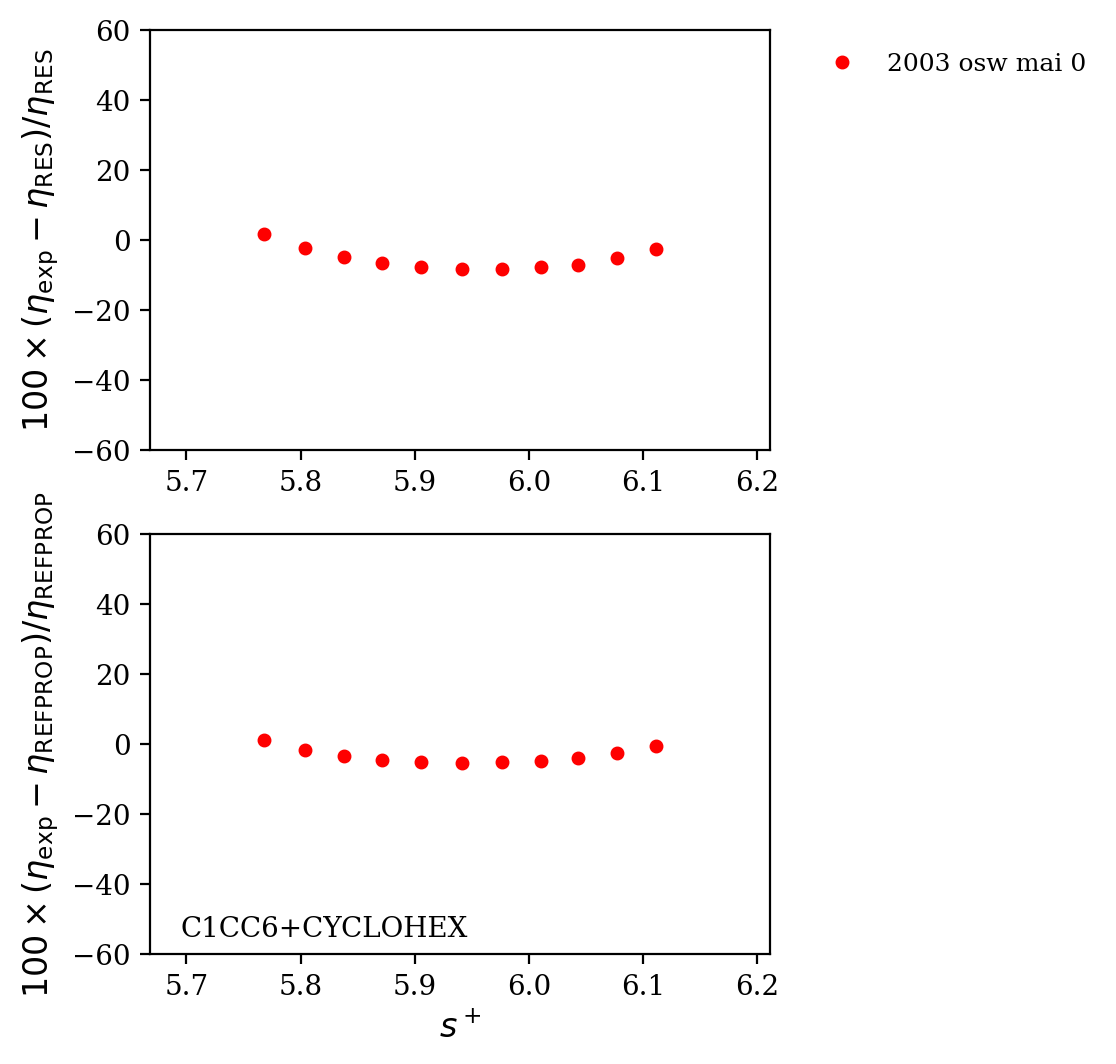

Supplement: Supplementary file 1 — je4c00451_si_001.zip [file je4c00451_si_001.zip › supporting_information/mix_dev_exp_res_ecs/C1CC6+CYCLOHEX.png]

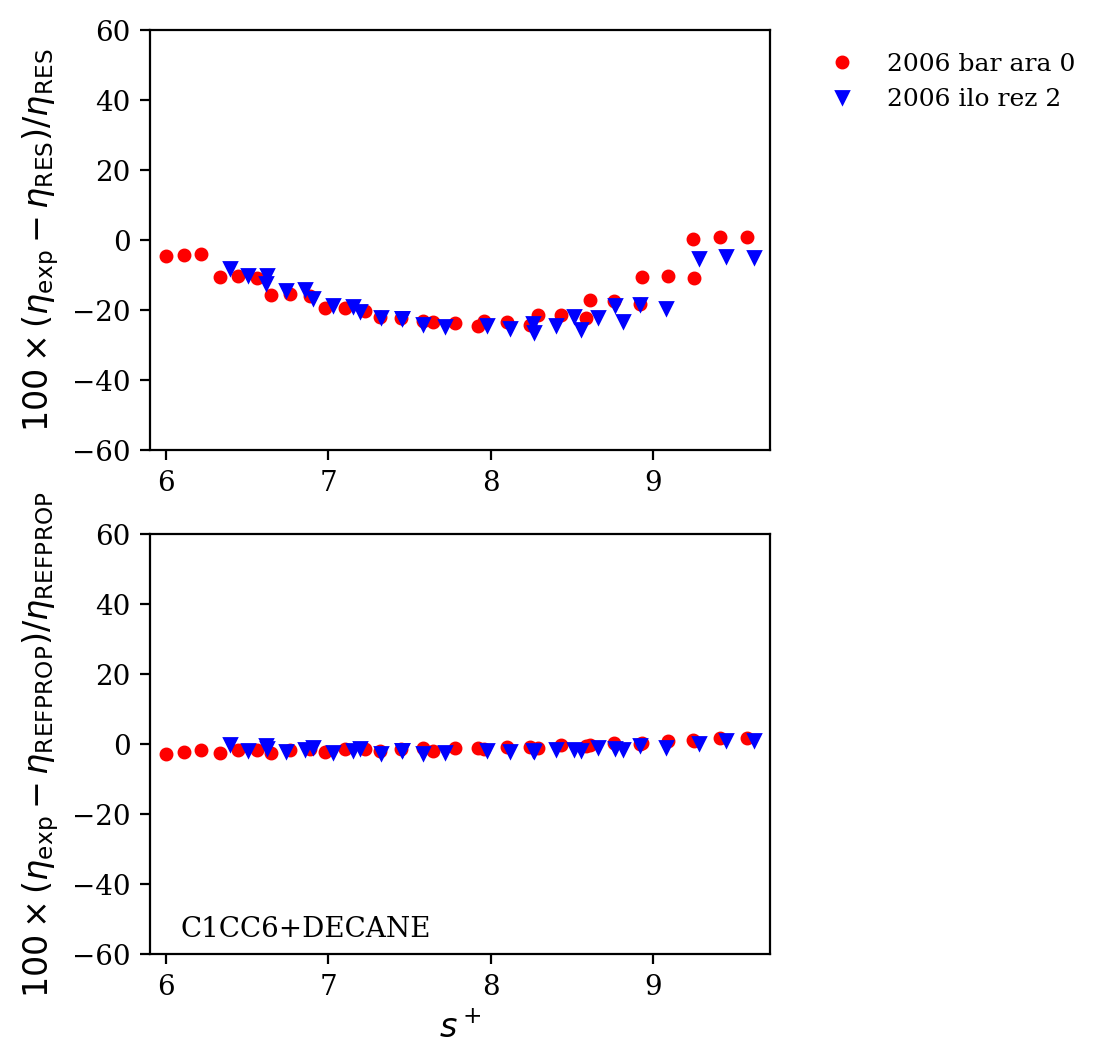

Supplement: Supplementary file 1 — je4c00451_si_001.zip [file je4c00451_si_001.zip › supporting_information/mix_dev_exp_res_ecs/C1CC6+DECANE.png]

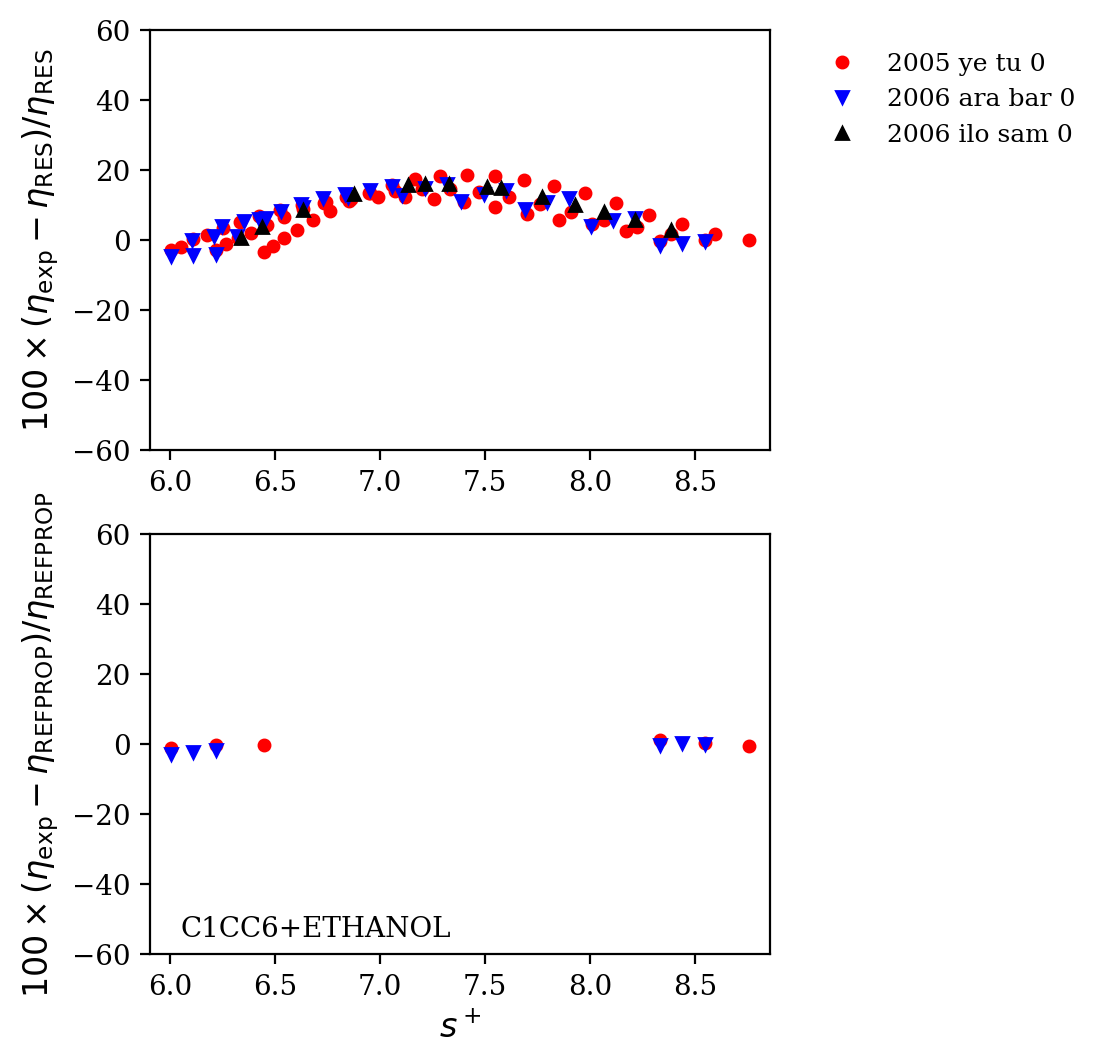

Supplement: Supplementary file 1 — je4c00451_si_001.zip [file je4c00451_si_001.zip › supporting_information/mix_dev_exp_res_ecs/C1CC6+ETHANOL.png]

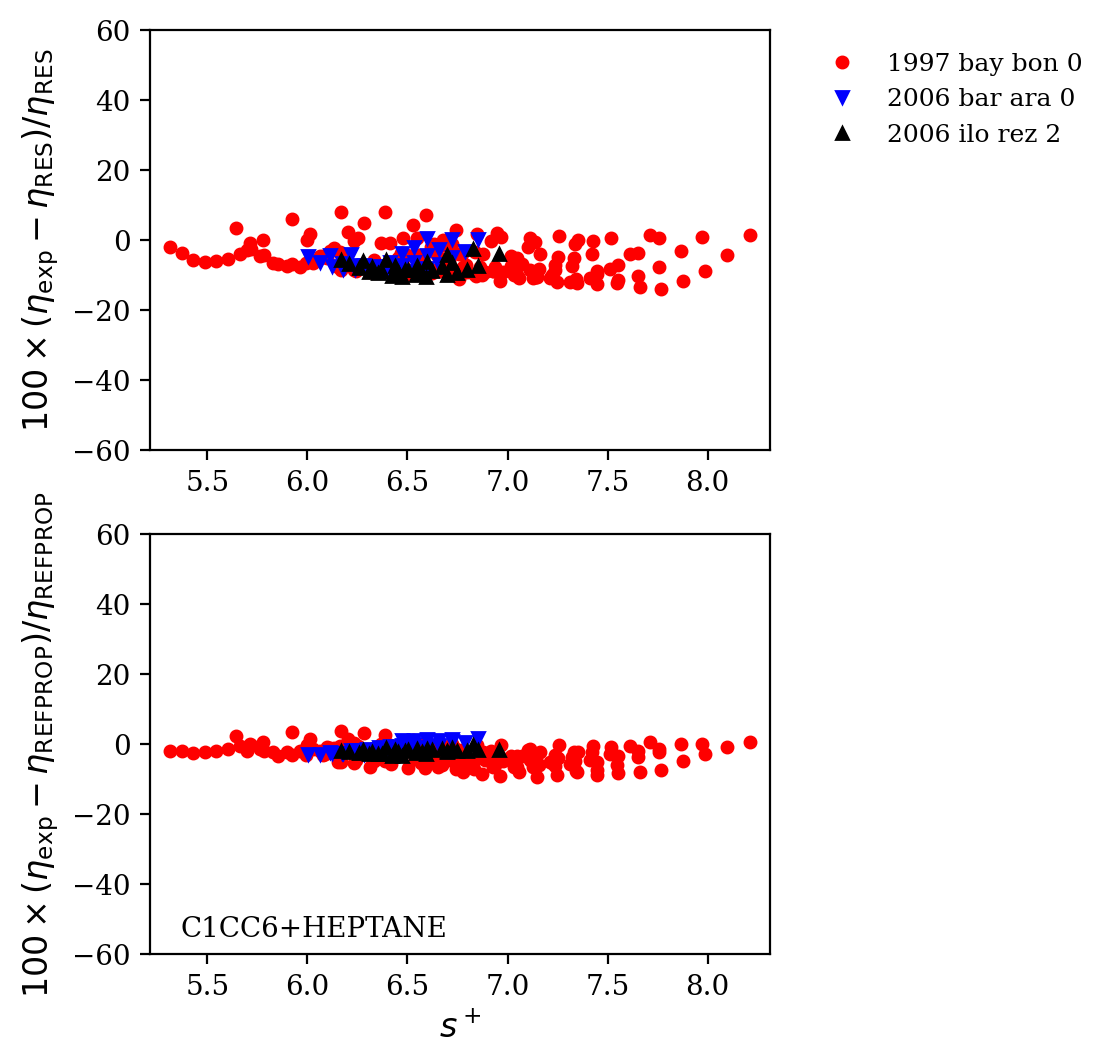

Supplement: Supplementary file 1 — je4c00451_si_001.zip [file je4c00451_si_001.zip › supporting_information/mix_dev_exp_res_ecs/C1CC6+HEPTANE.png]

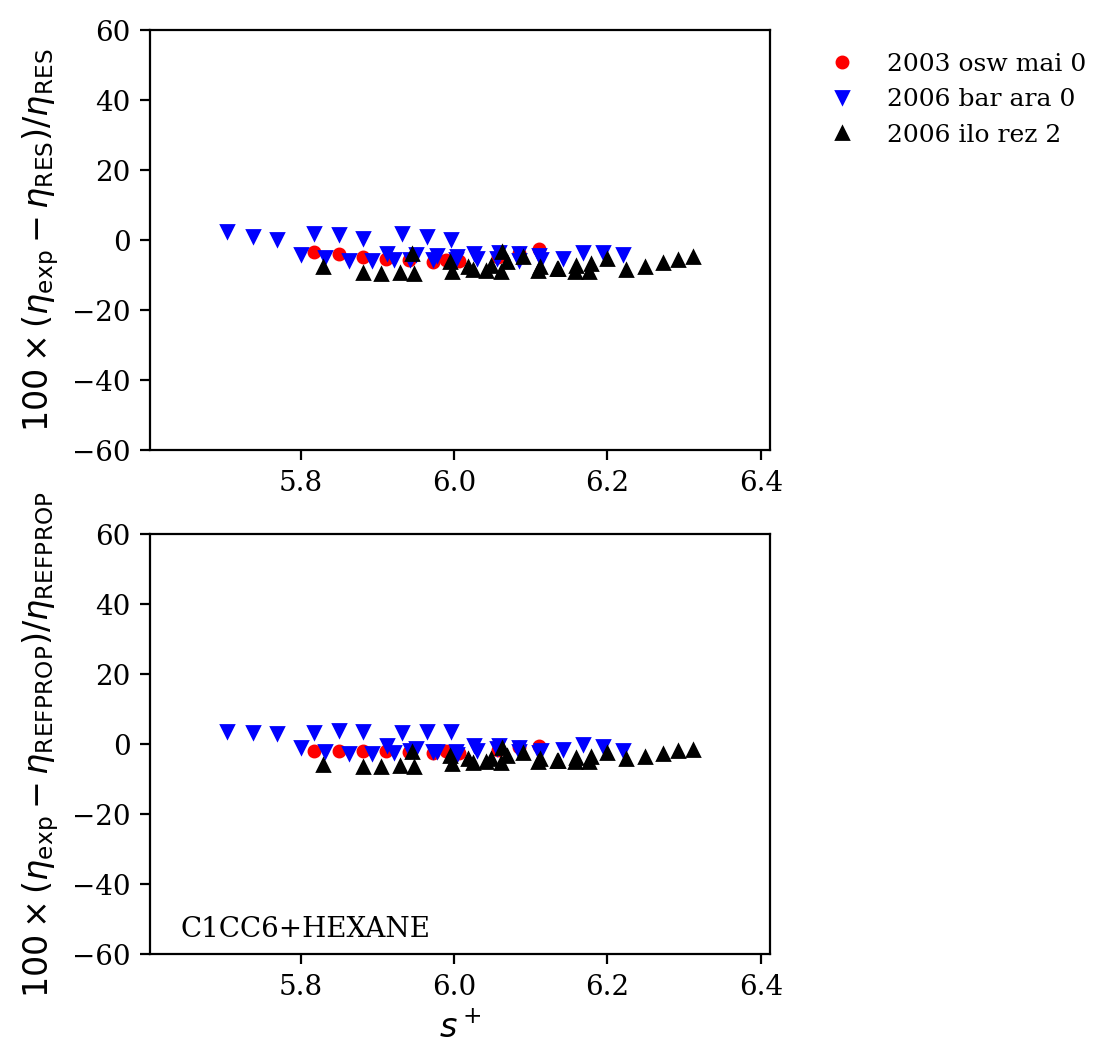

Supplement: Supplementary file 1 — je4c00451_si_001.zip [file je4c00451_si_001.zip › supporting_information/mix_dev_exp_res_ecs/C1CC6+HEXANE.png]

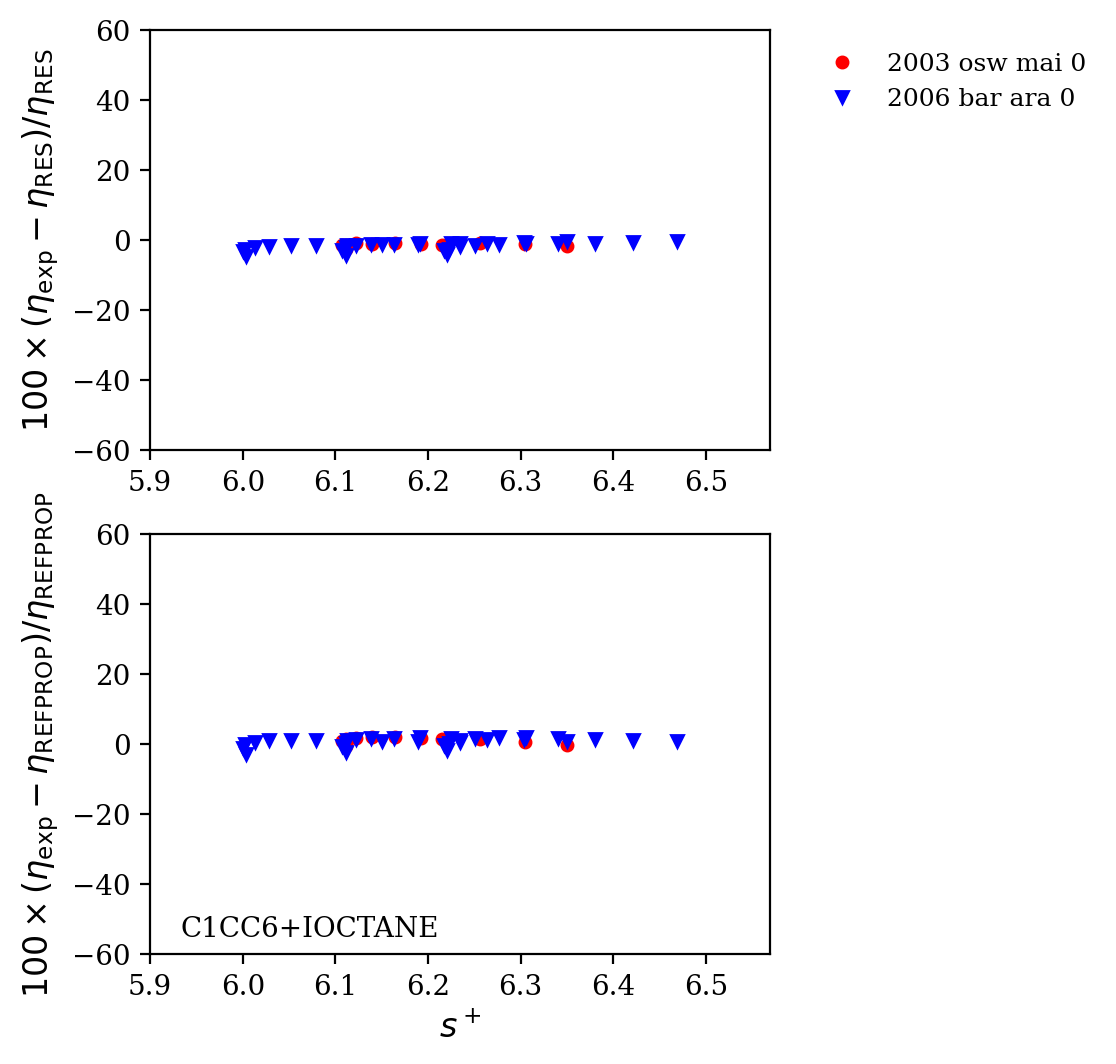

Supplement: Supplementary file 1 — je4c00451_si_001.zip [file je4c00451_si_001.zip › supporting_information/mix_dev_exp_res_ecs/C1CC6+IOCTANE.png]

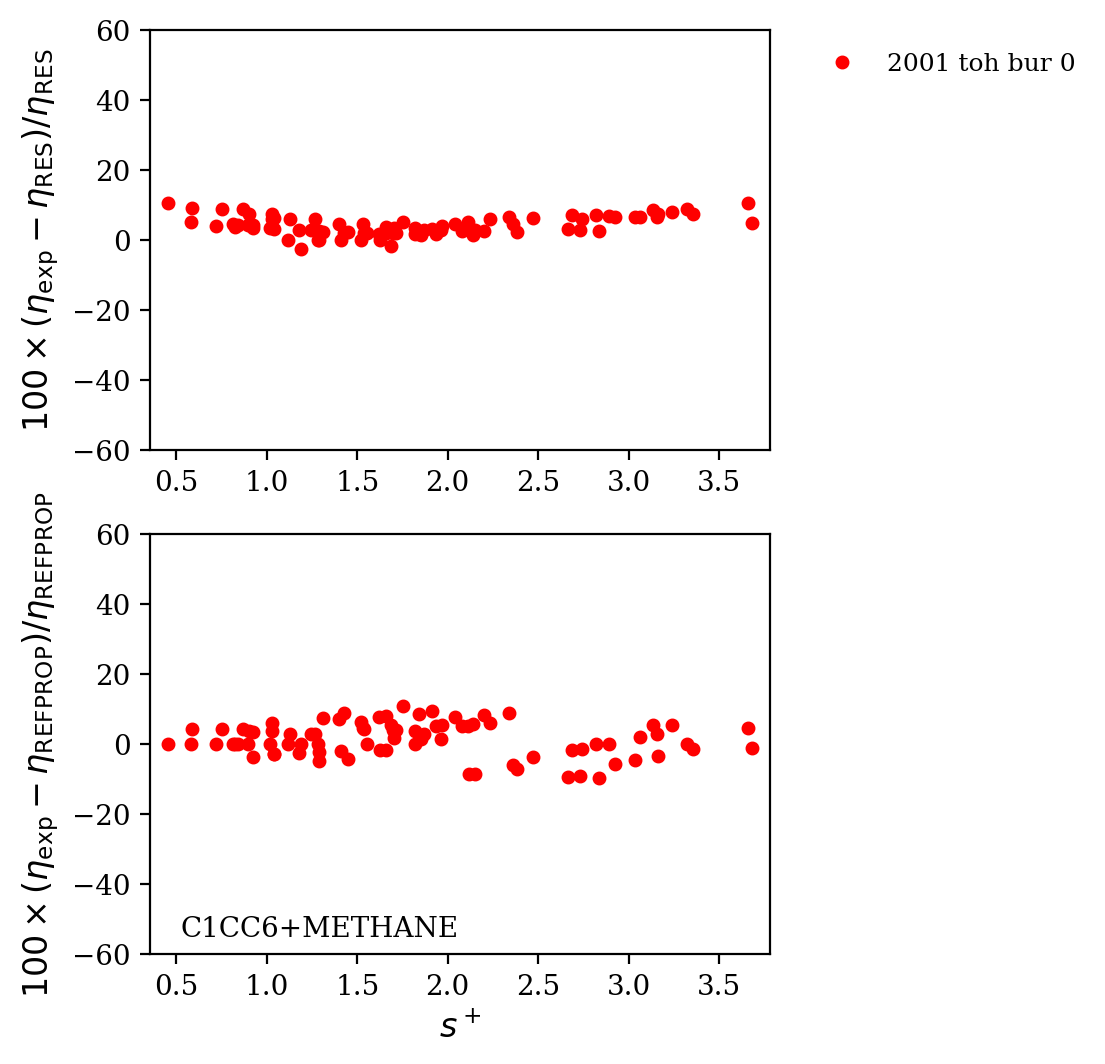

Supplement: Supplementary file 1 — je4c00451_si_001.zip [file je4c00451_si_001.zip › supporting_information/mix_dev_exp_res_ecs/C1CC6+METHANE.png]

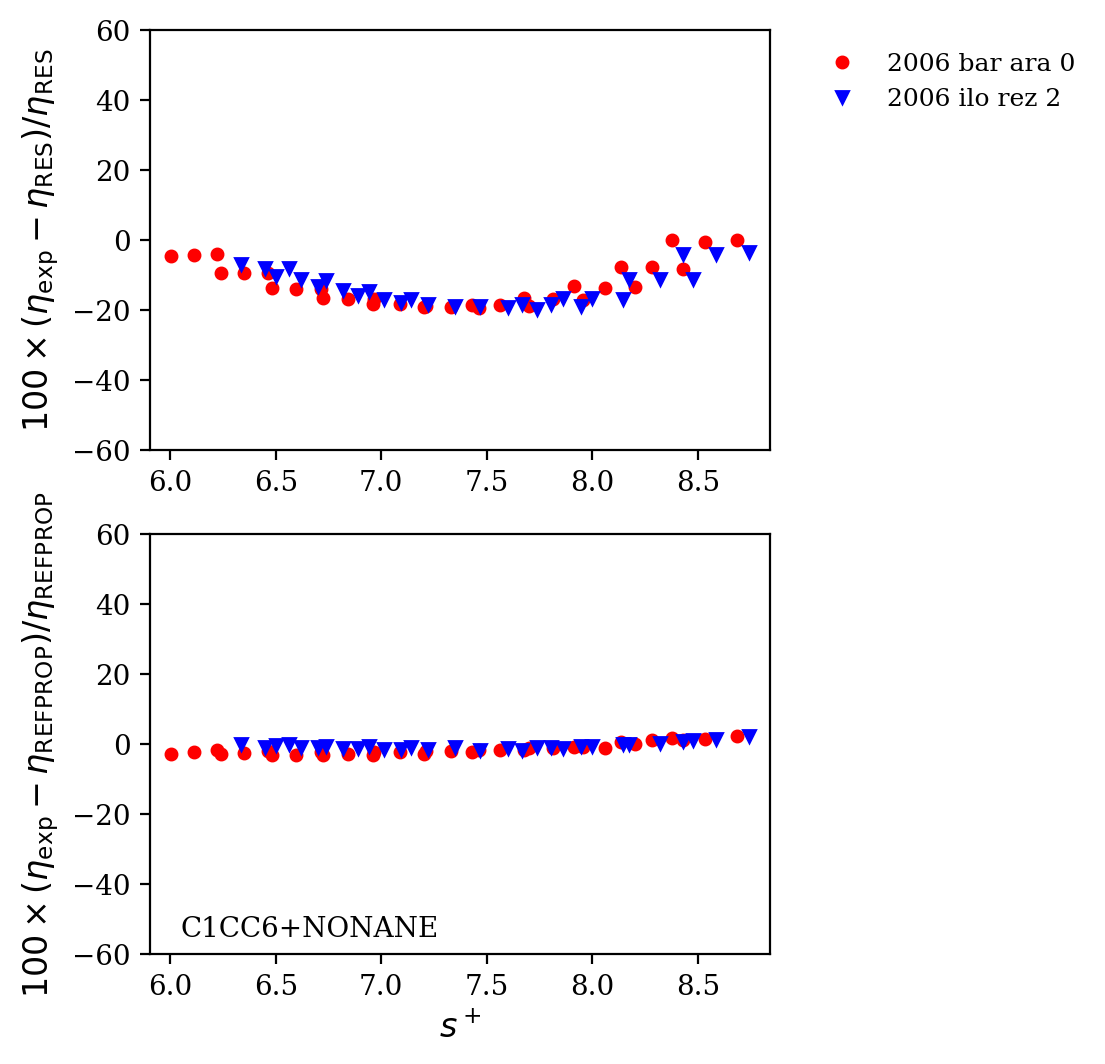

Supplement: Supplementary file 1 — je4c00451_si_001.zip [file je4c00451_si_001.zip › supporting_information/mix_dev_exp_res_ecs/C1CC6+NONANE.png]

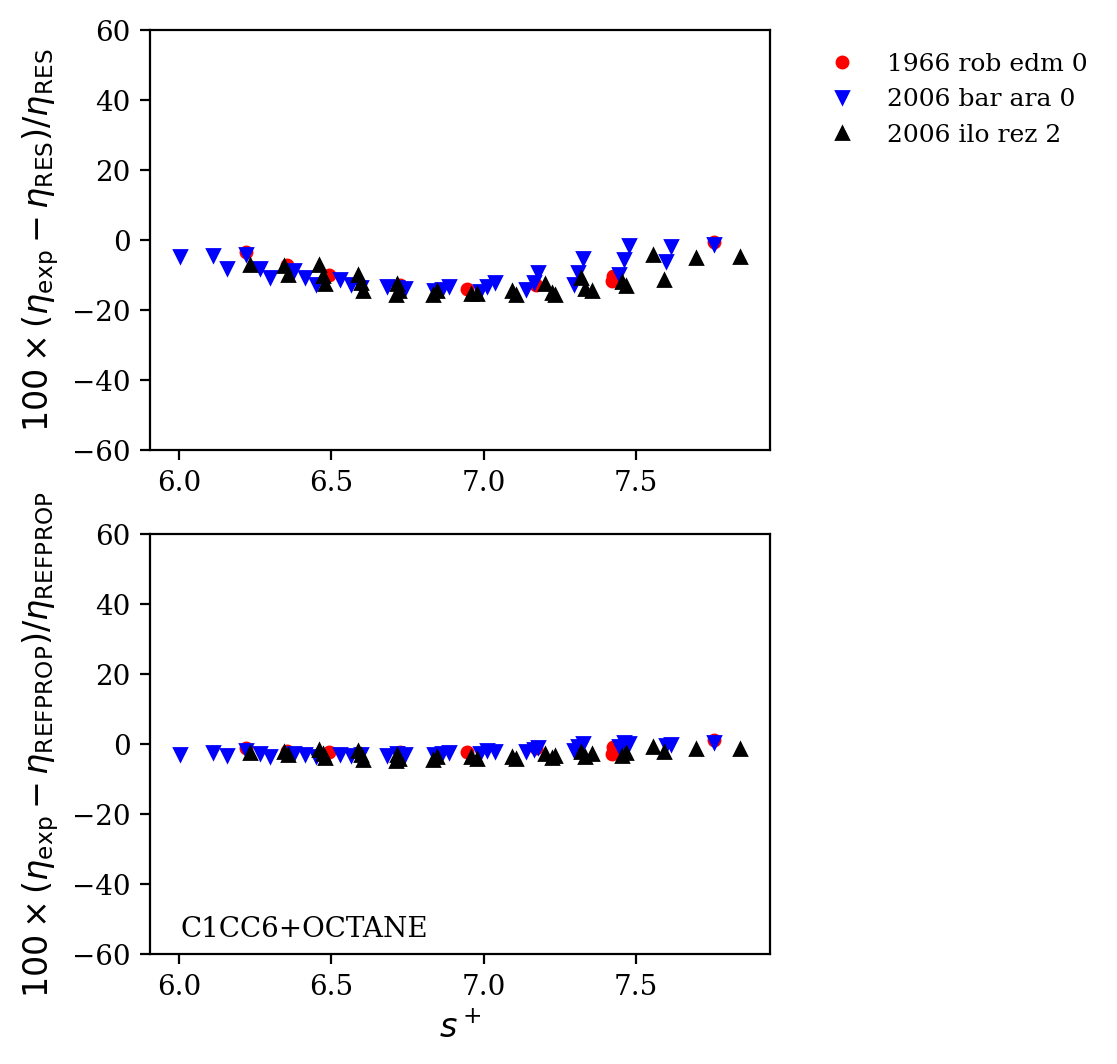

Supplement: Supplementary file 1 — je4c00451_si_001.zip [file je4c00451_si_001.zip › supporting_information/mix_dev_exp_res_ecs/C1CC6+OCTANE.png]

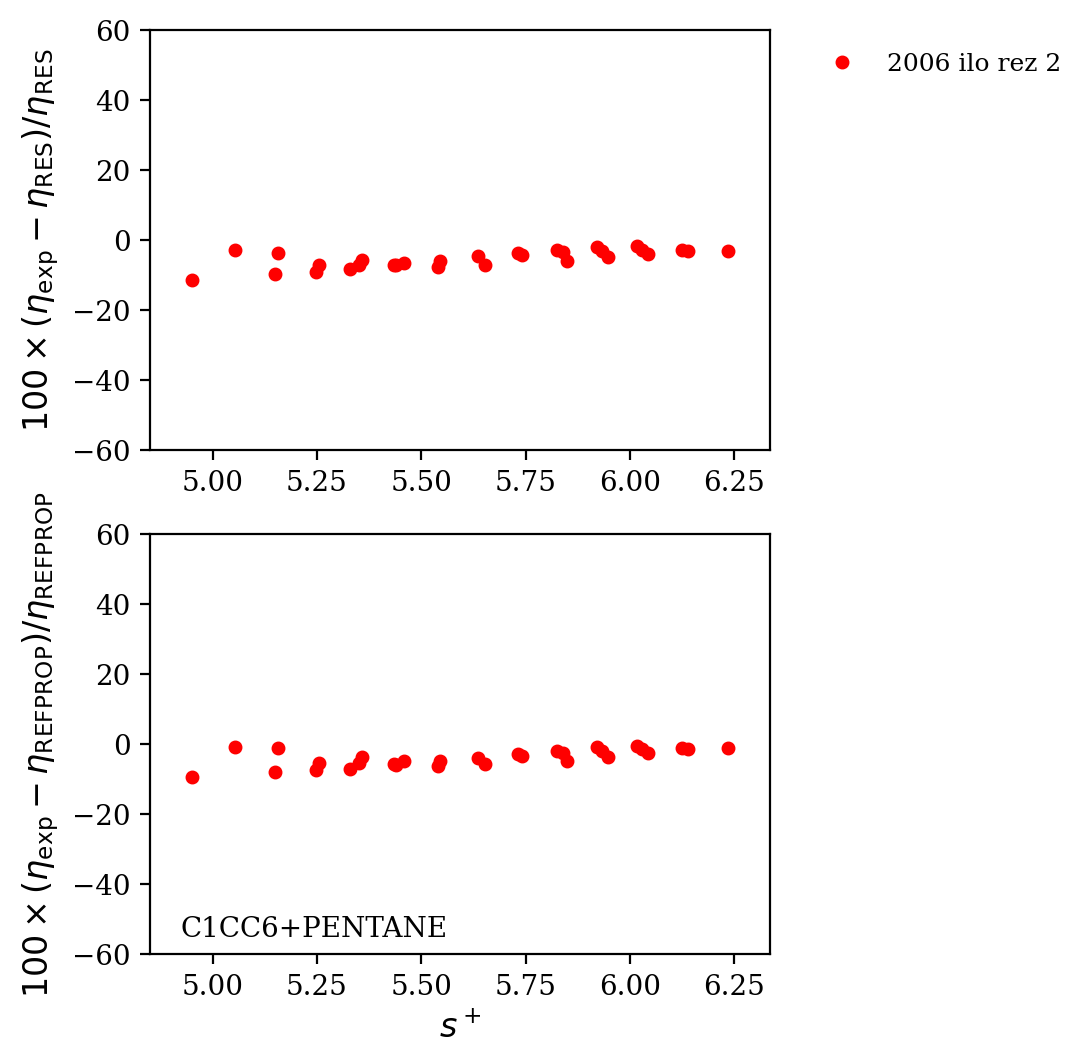

Supplement: Supplementary file 1 — je4c00451_si_001.zip [file je4c00451_si_001.zip › supporting_information/mix_dev_exp_res_ecs/C1CC6+PENTANE.png]

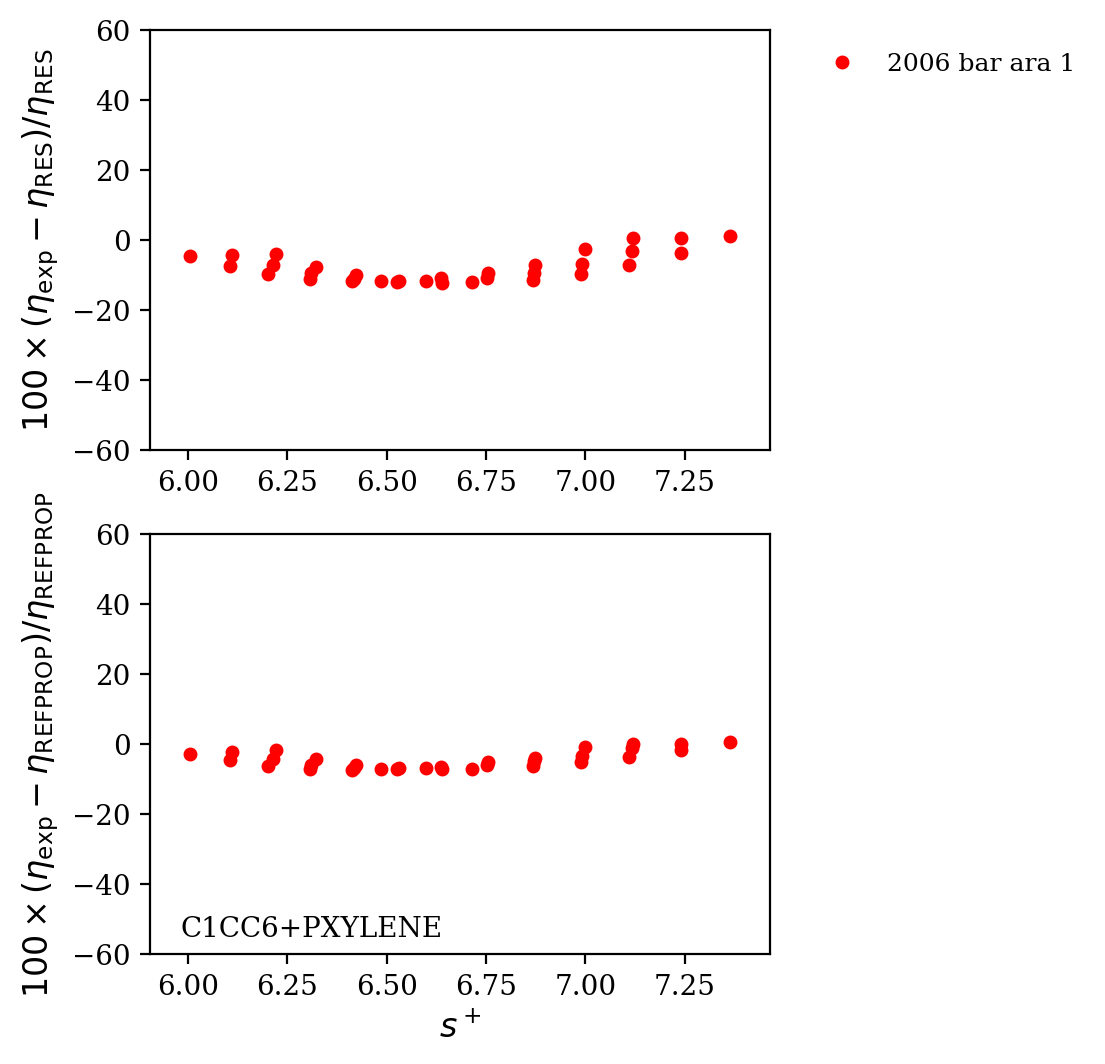

Supplement: Supplementary file 1 — je4c00451_si_001.zip [file je4c00451_si_001.zip › supporting_information/mix_dev_exp_res_ecs/C1CC6+PXYLENE.png]

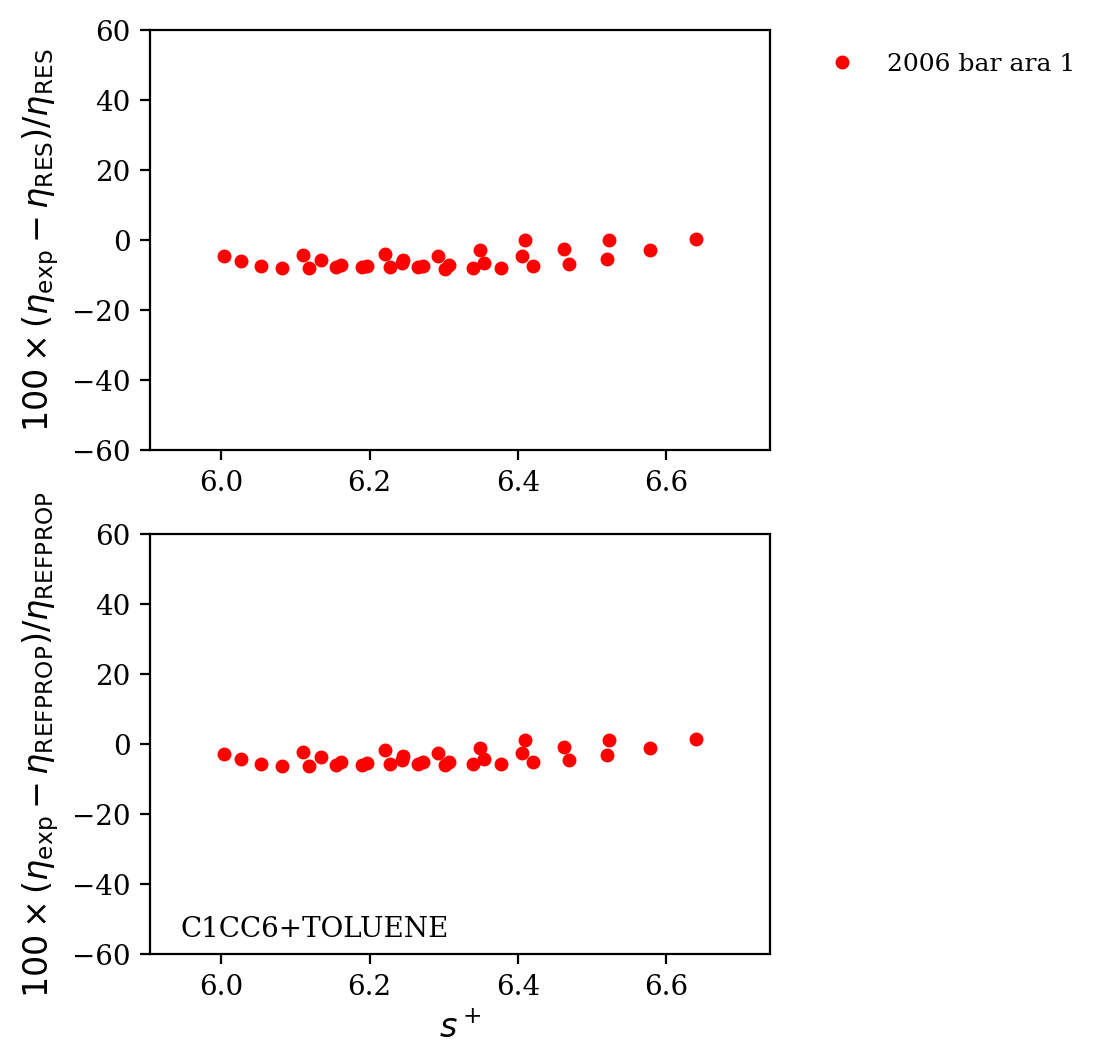

Supplement: Supplementary file 1 — je4c00451_si_001.zip [file je4c00451_si_001.zip › supporting_information/mix_dev_exp_res_ecs/C1CC6+TOLUENE.png]

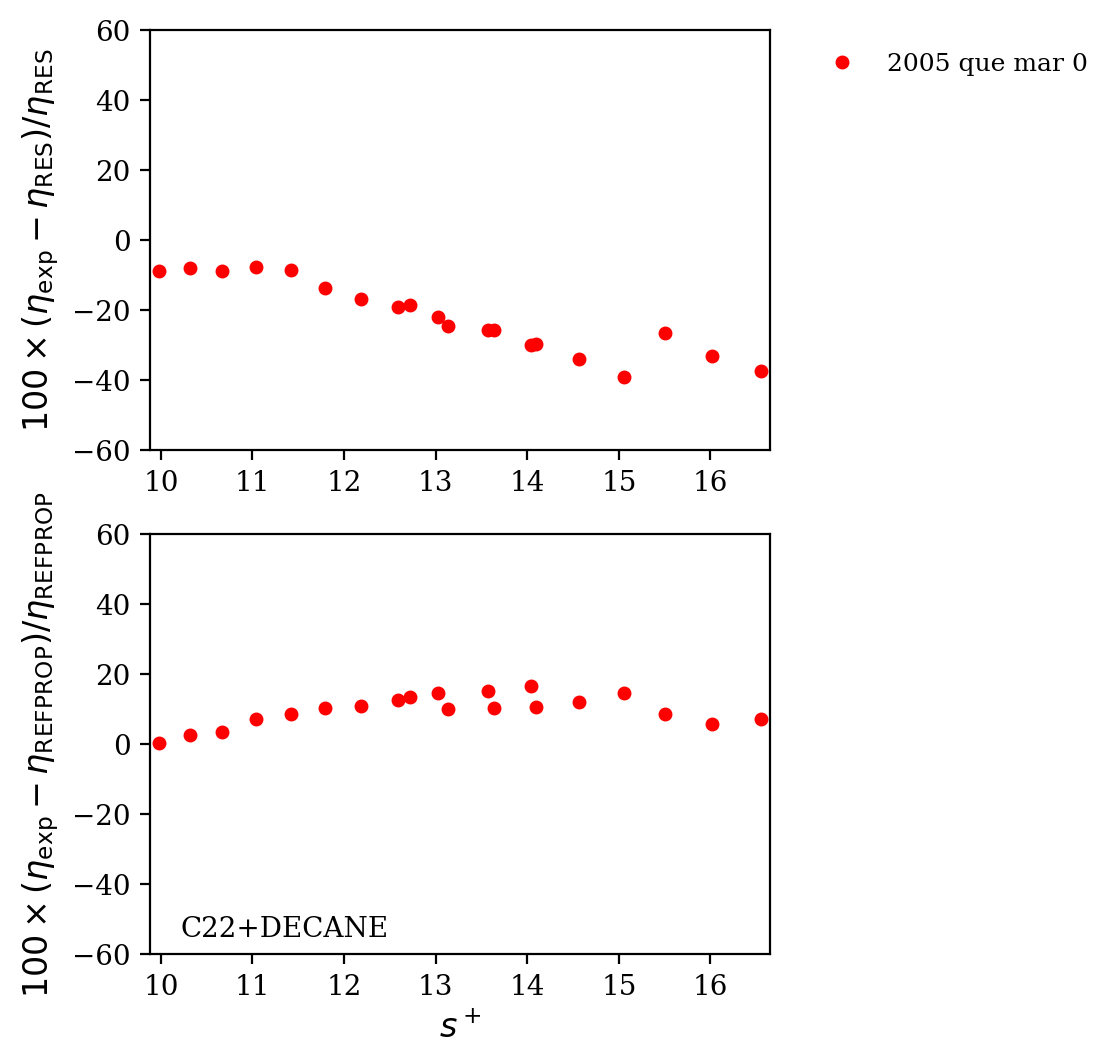

Supplement: Supplementary file 1 — je4c00451_si_001.zip [file je4c00451_si_001.zip › supporting_information/mix_dev_exp_res_ecs/C22+DECANE.png]

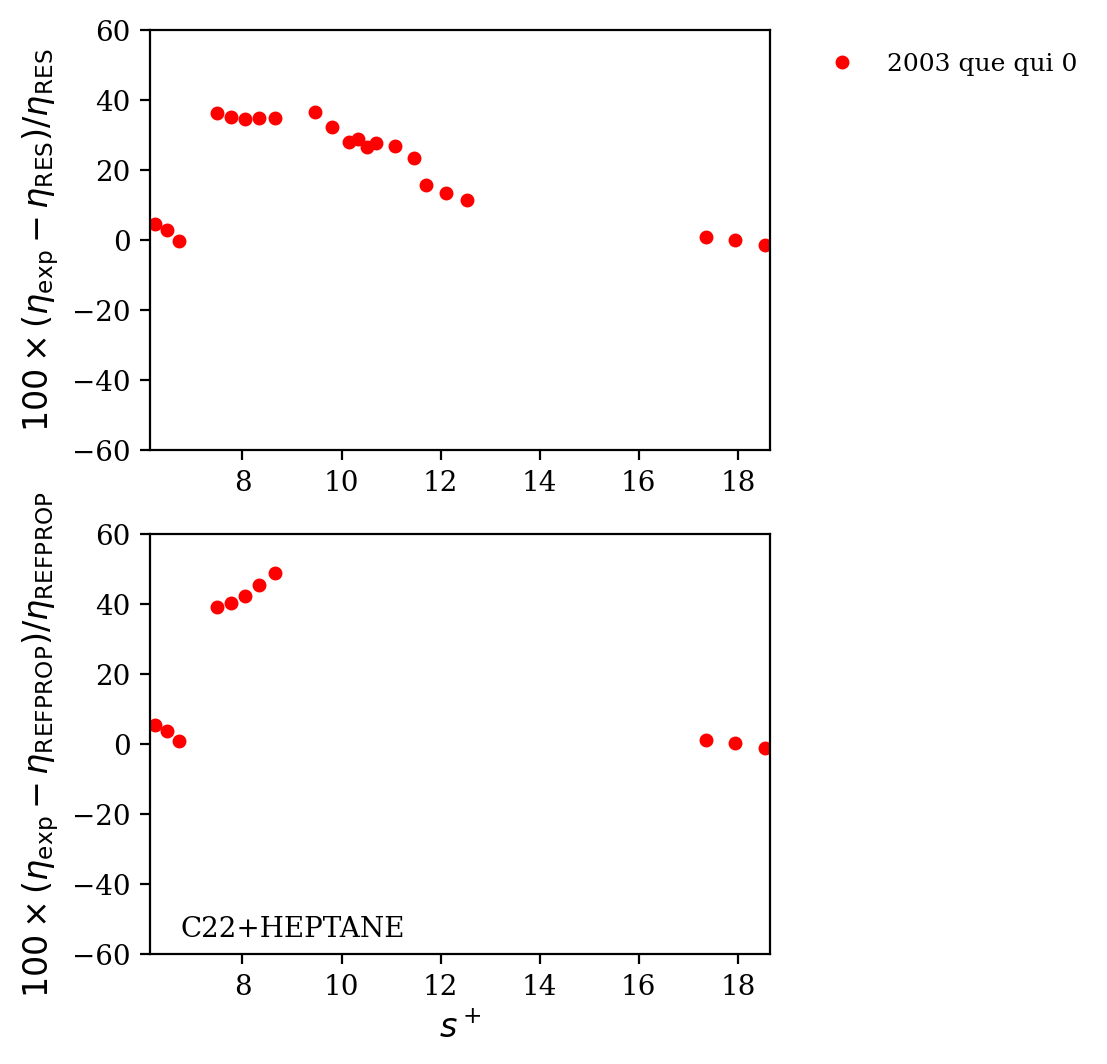

Supplement: Supplementary file 1 — je4c00451_si_001.zip [file je4c00451_si_001.zip › supporting_information/mix_dev_exp_res_ecs/C22+HEPTANE.png]

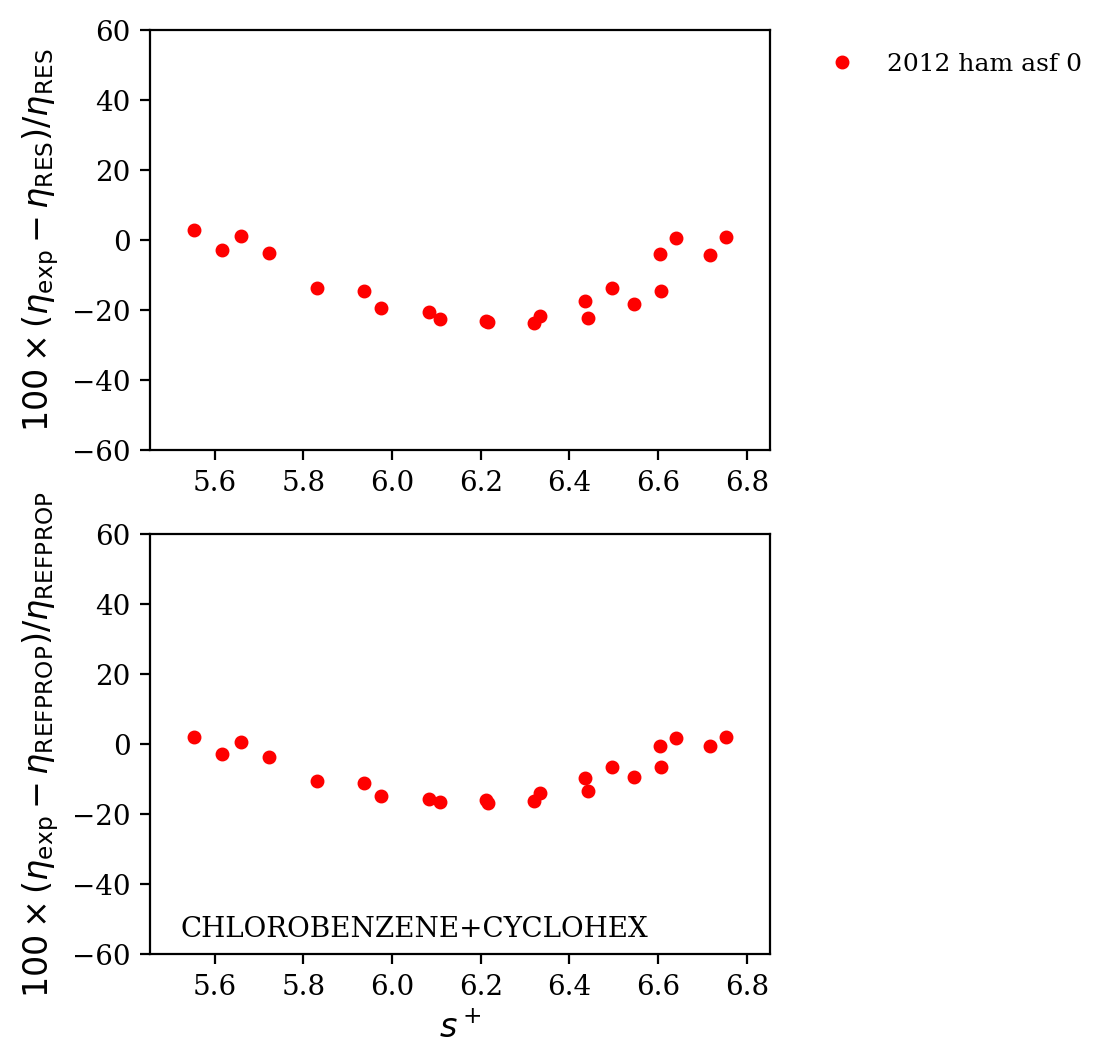

Supplement: Supplementary file 1 — je4c00451_si_001.zip [file je4c00451_si_001.zip › supporting_information/mix_dev_exp_res_ecs/CHLOROBENZENE+CYCLOHEX.png]

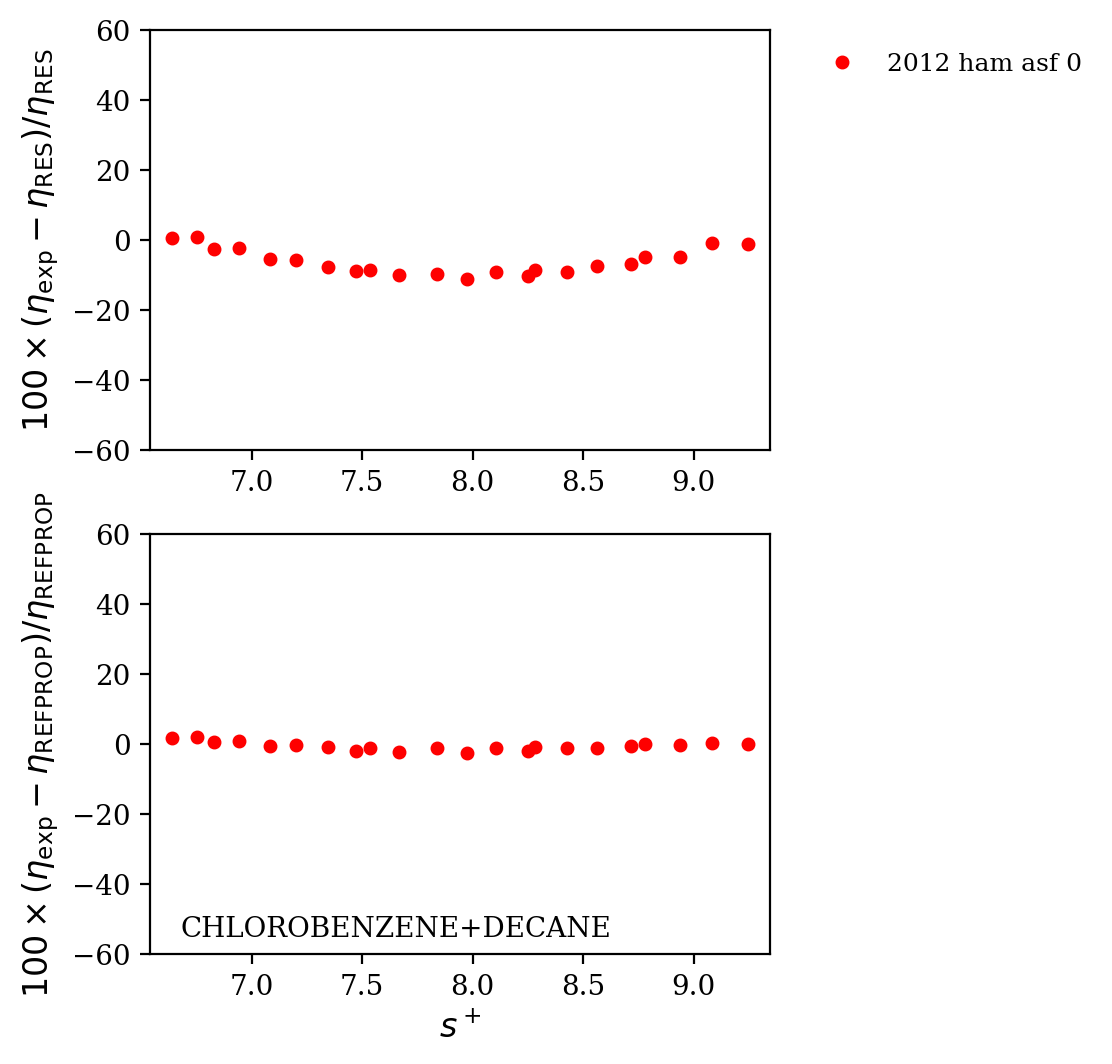

Supplement: Supplementary file 1 — je4c00451_si_001.zip [file je4c00451_si_001.zip › supporting_information/mix_dev_exp_res_ecs/CHLOROBENZENE+DECANE.png]

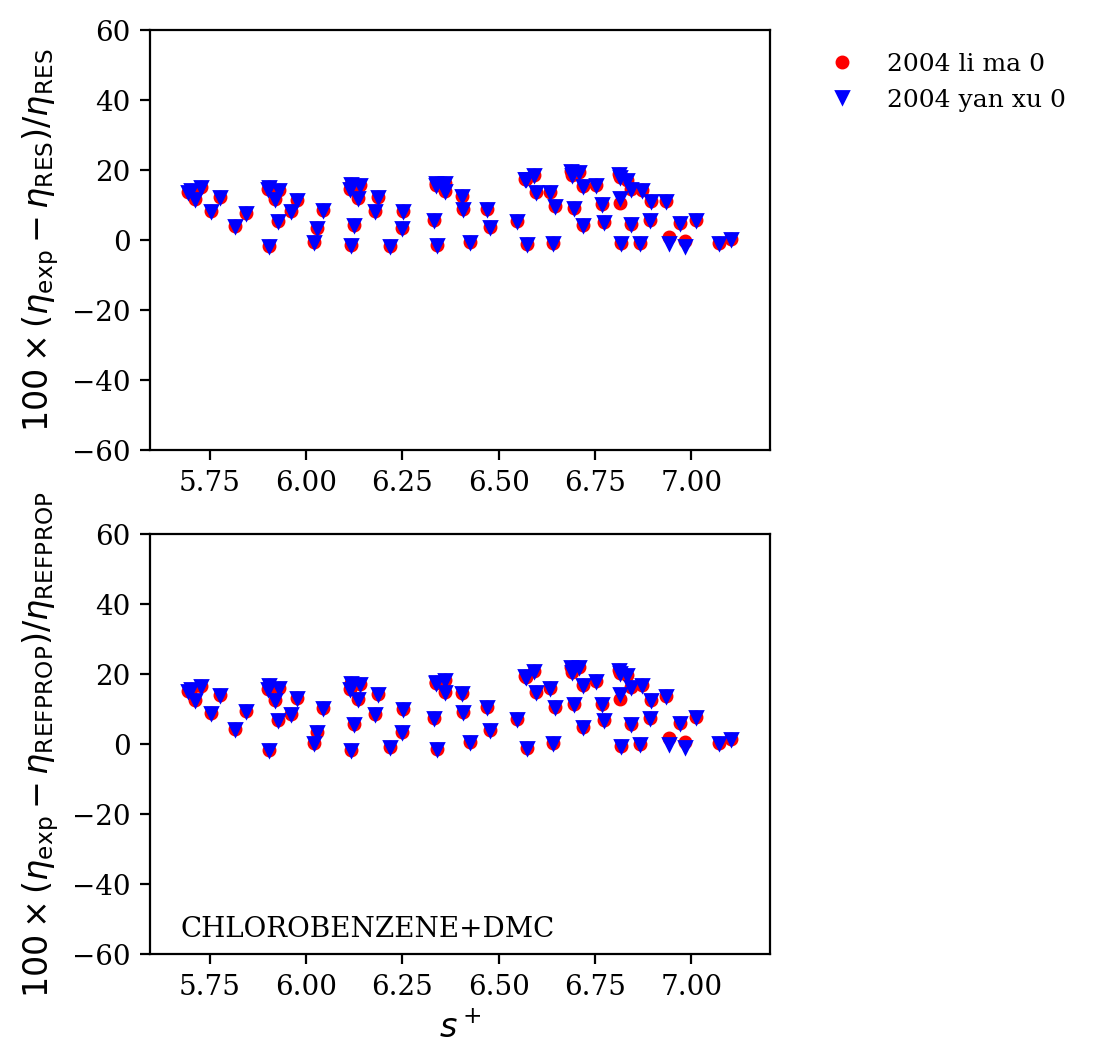

Supplement: Supplementary file 1 — je4c00451_si_001.zip [file je4c00451_si_001.zip › supporting_information/mix_dev_exp_res_ecs/CHLOROBENZENE+DMC.png]

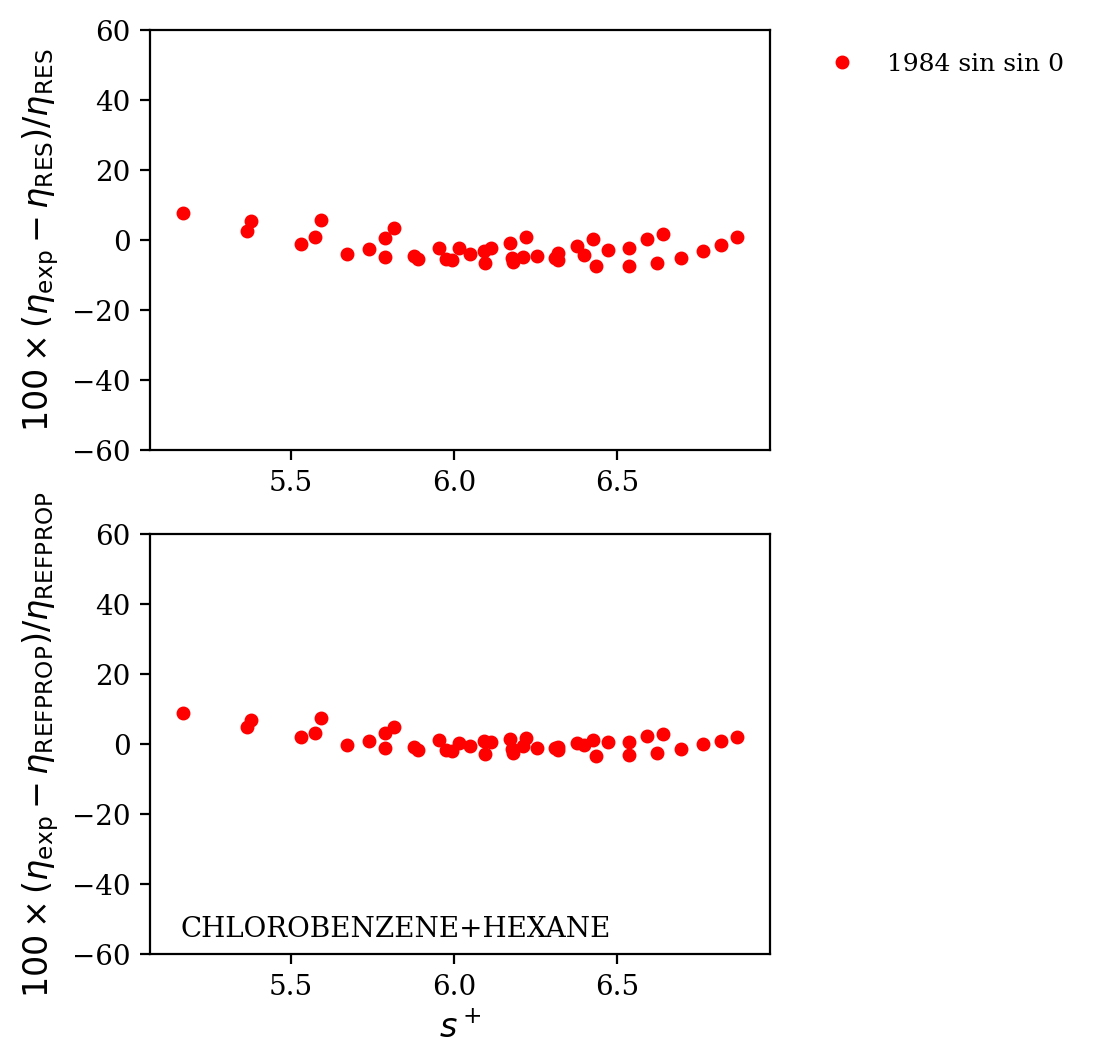

Supplement: Supplementary file 1 — je4c00451_si_001.zip [file je4c00451_si_001.zip › supporting_information/mix_dev_exp_res_ecs/CHLOROBENZENE+HEXANE.png]

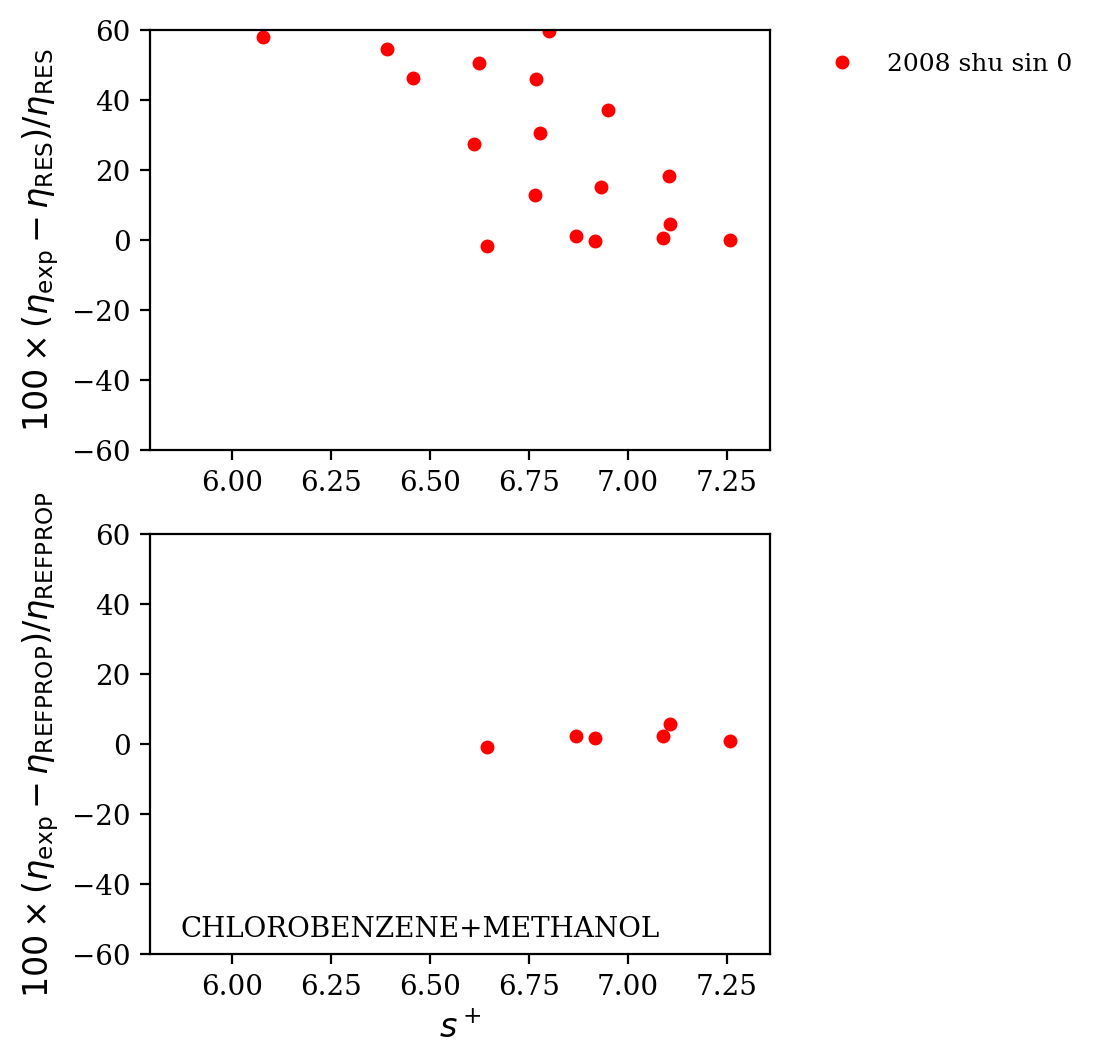

Supplement: Supplementary file 1 — je4c00451_si_001.zip [file je4c00451_si_001.zip › supporting_information/mix_dev_exp_res_ecs/CHLOROBENZENE+METHANOL.png]

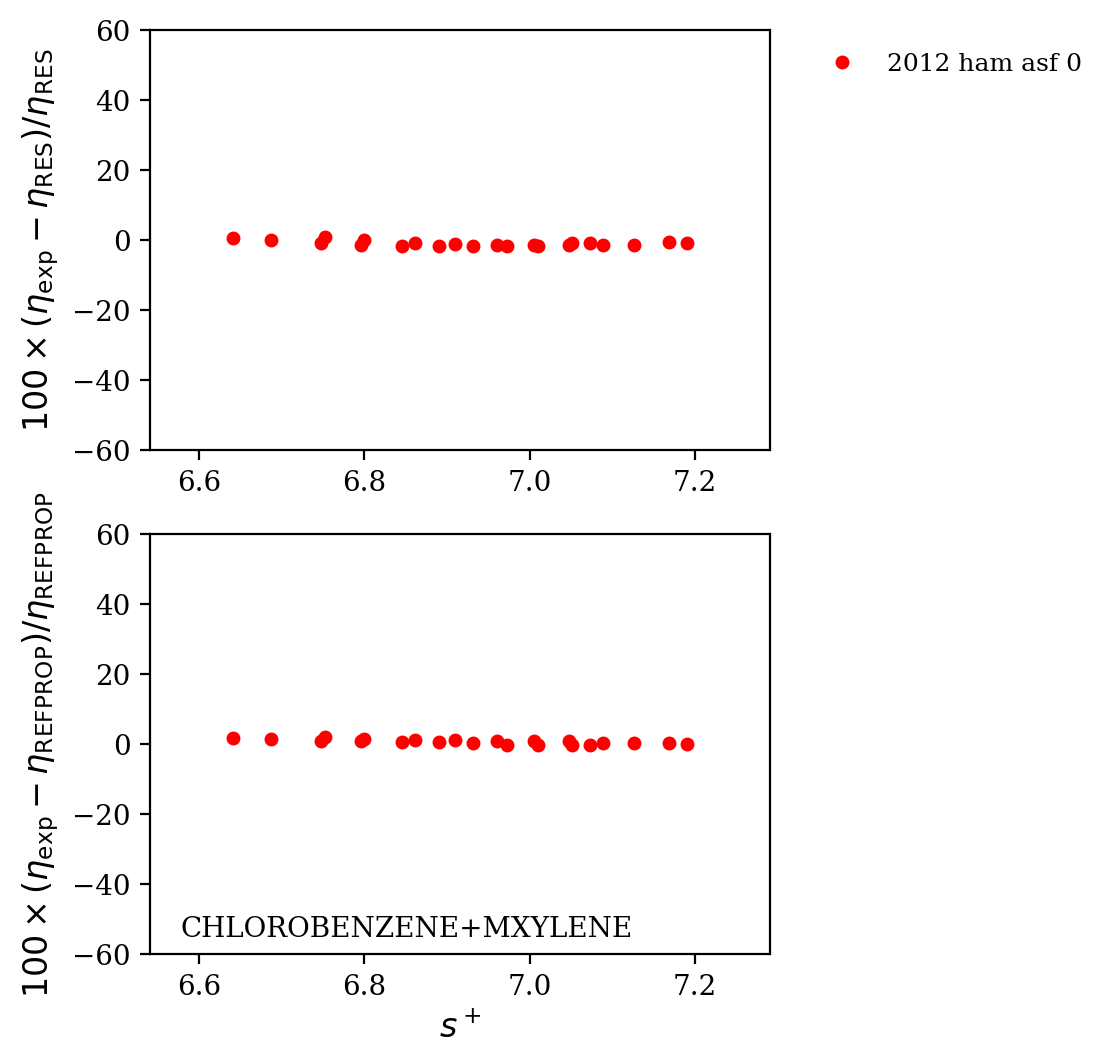

Supplement: Supplementary file 1 — je4c00451_si_001.zip [file je4c00451_si_001.zip › supporting_information/mix_dev_exp_res_ecs/CHLOROBENZENE+MXYLENE.png]

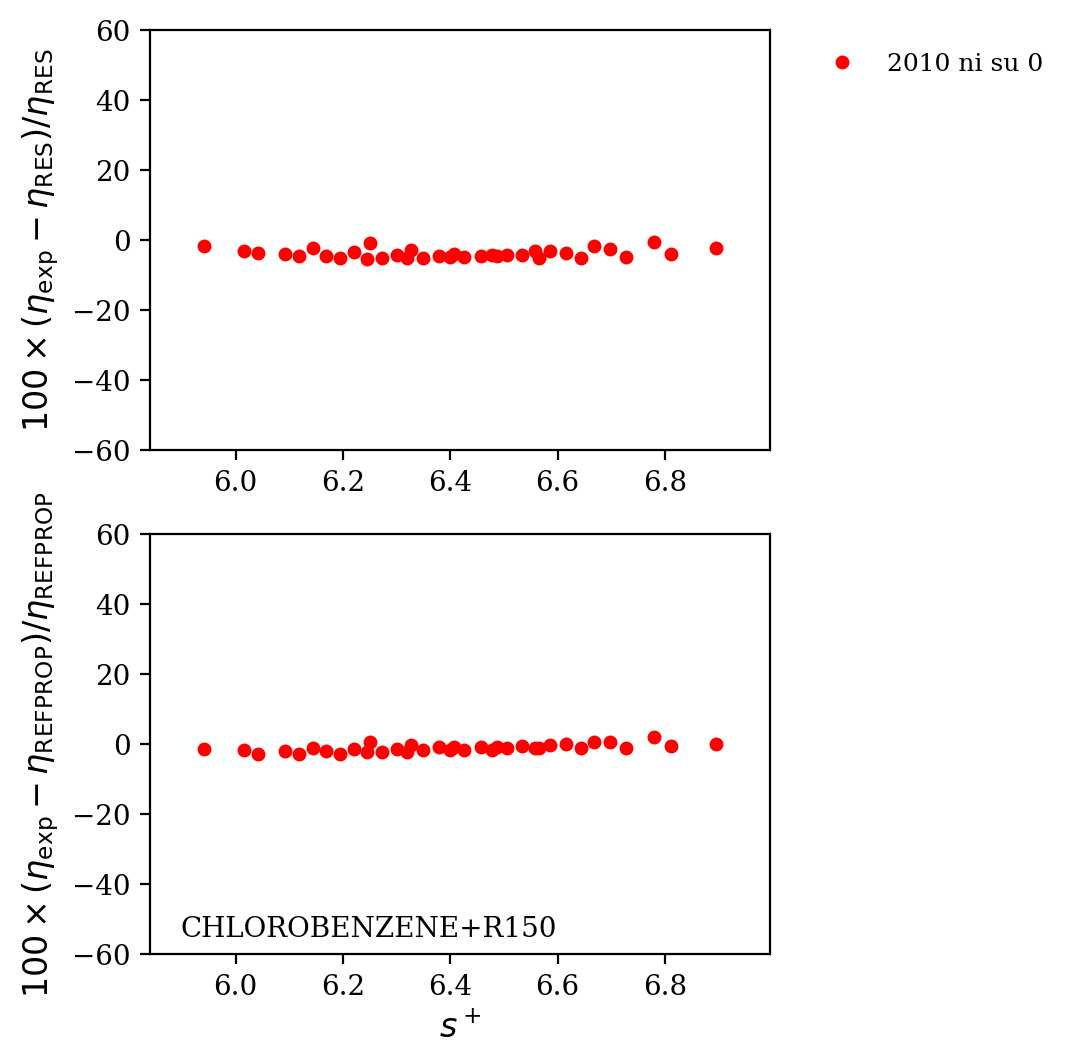

Supplement: Supplementary file 1 — je4c00451_si_001.zip [file je4c00451_si_001.zip › supporting_information/mix_dev_exp_res_ecs/CHLOROBENZENE+R150.png]

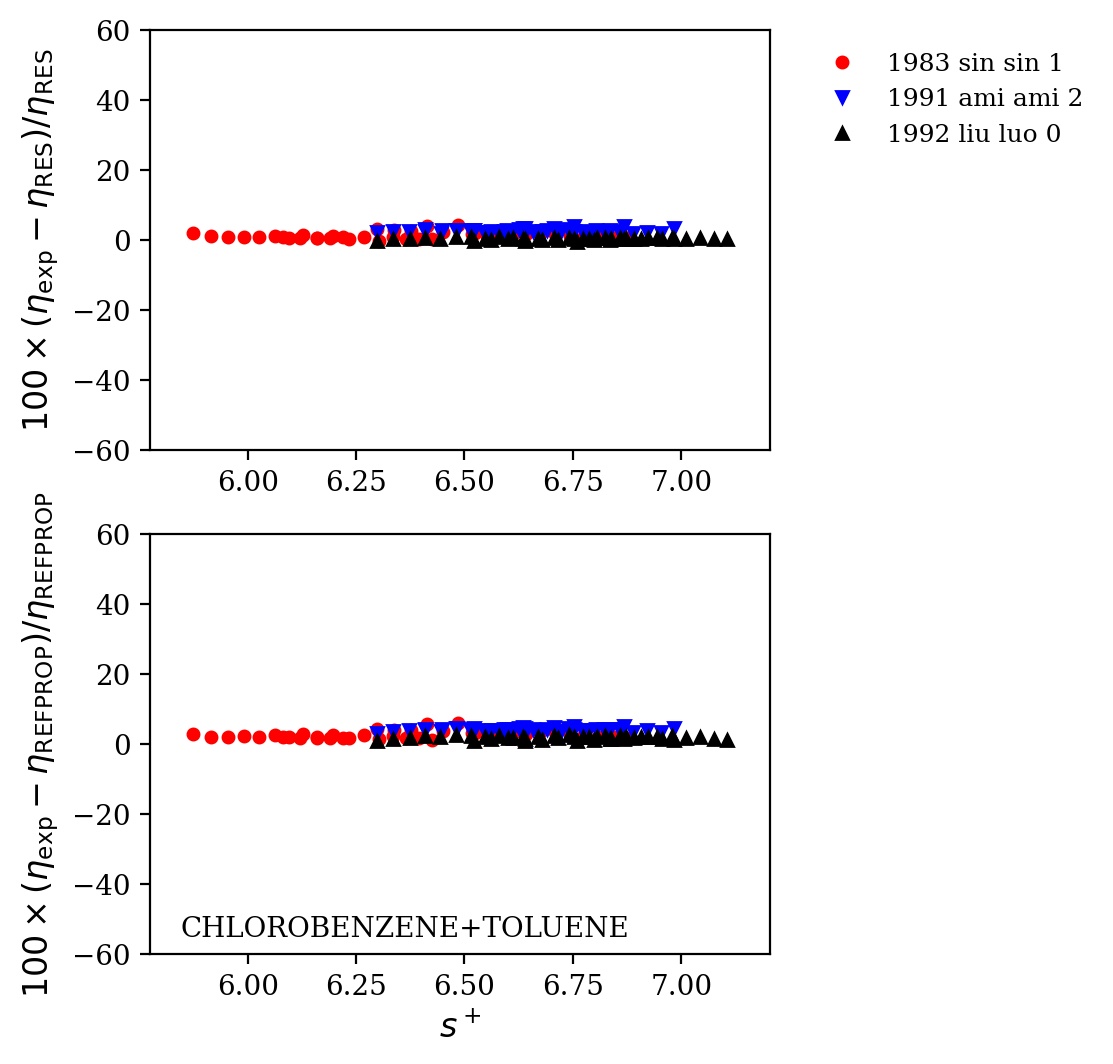

Supplement: Supplementary file 1 — je4c00451_si_001.zip [file je4c00451_si_001.zip › supporting_information/mix_dev_exp_res_ecs/CHLOROBENZENE+TOLUENE.png]

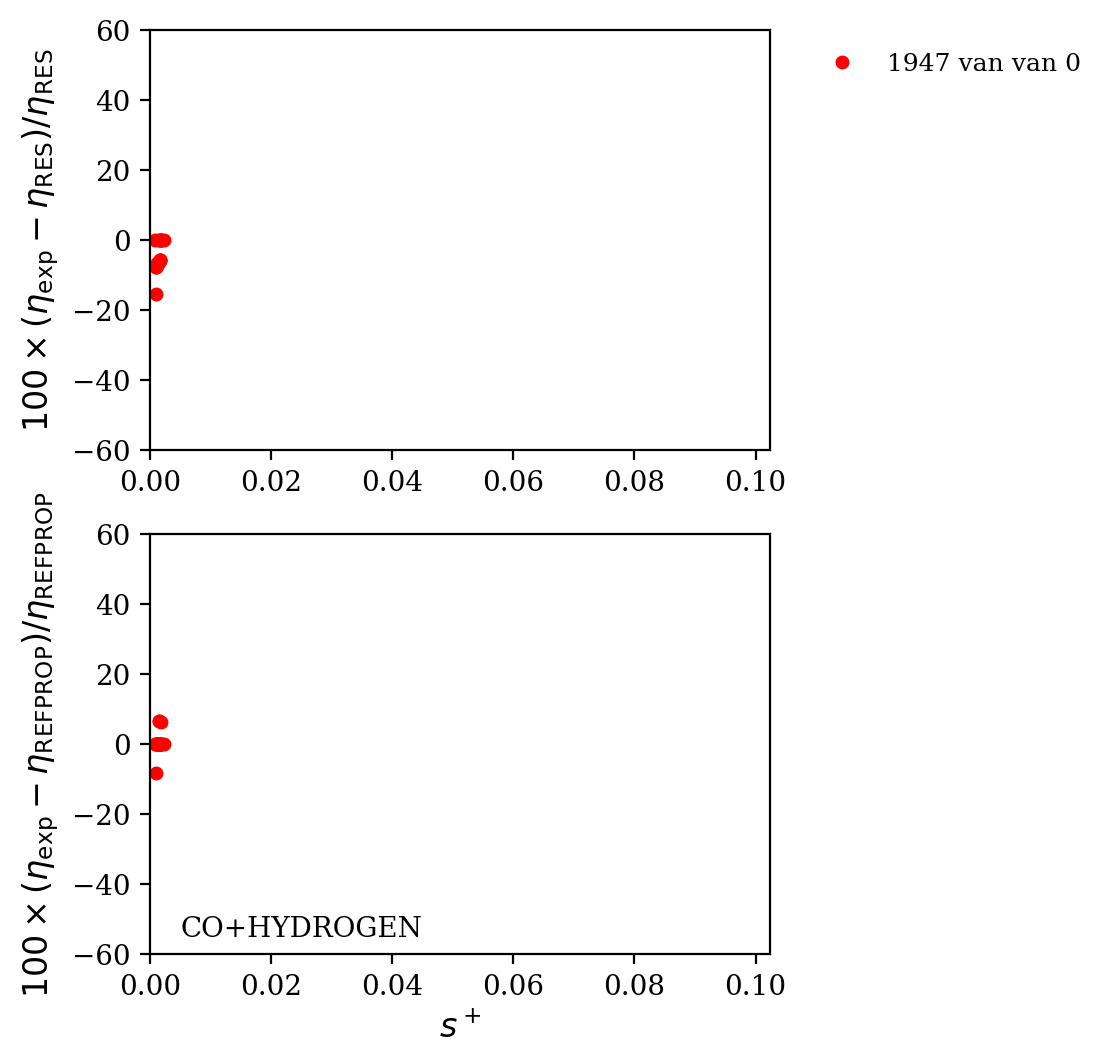

Supplement: Supplementary file 1 — je4c00451_si_001.zip [file je4c00451_si_001.zip › supporting_information/mix_dev_exp_res_ecs/CO+HYDROGEN.png]

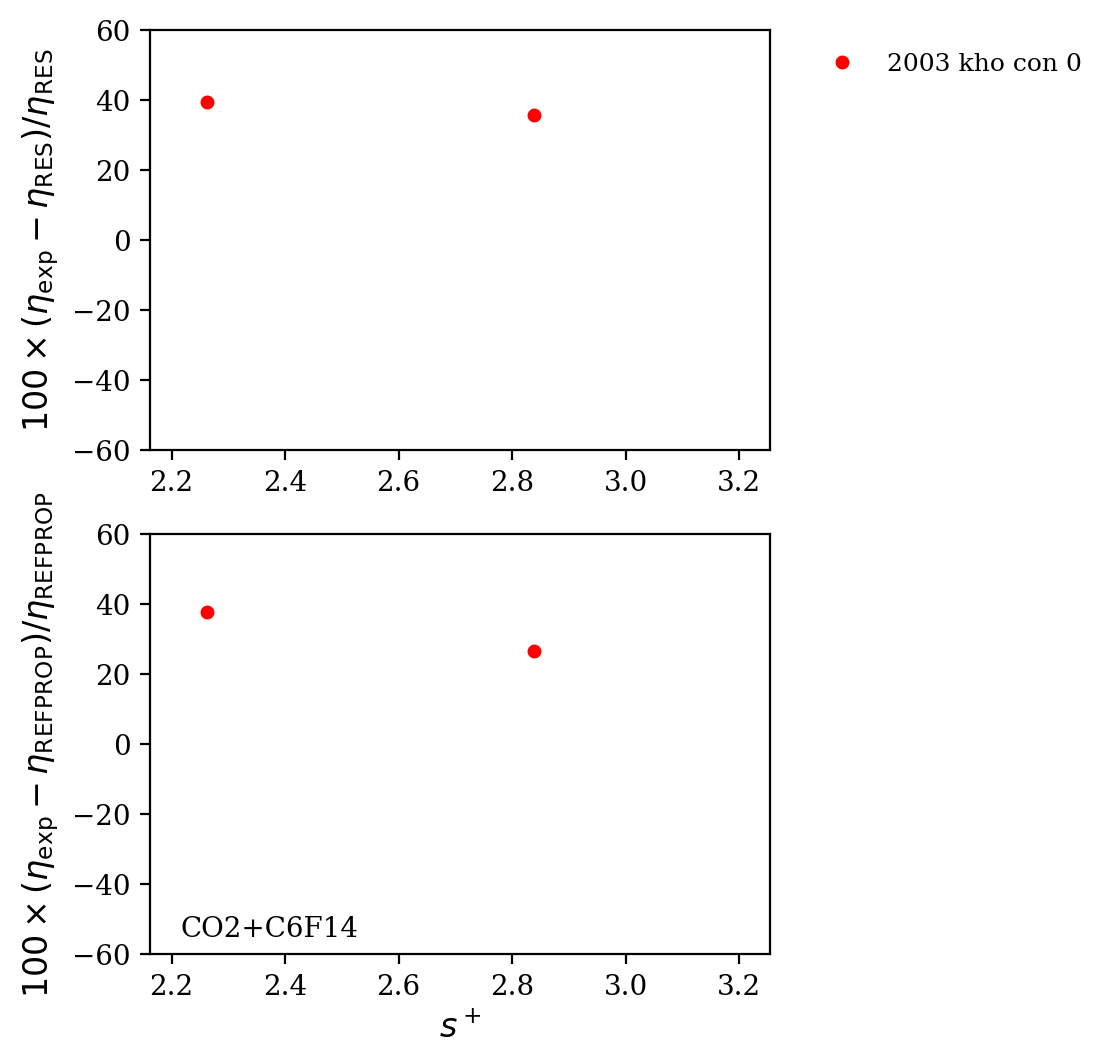

Supplement: Supplementary file 1 — je4c00451_si_001.zip [file je4c00451_si_001.zip › supporting_information/mix_dev_exp_res_ecs/CO2+C6F14.png]

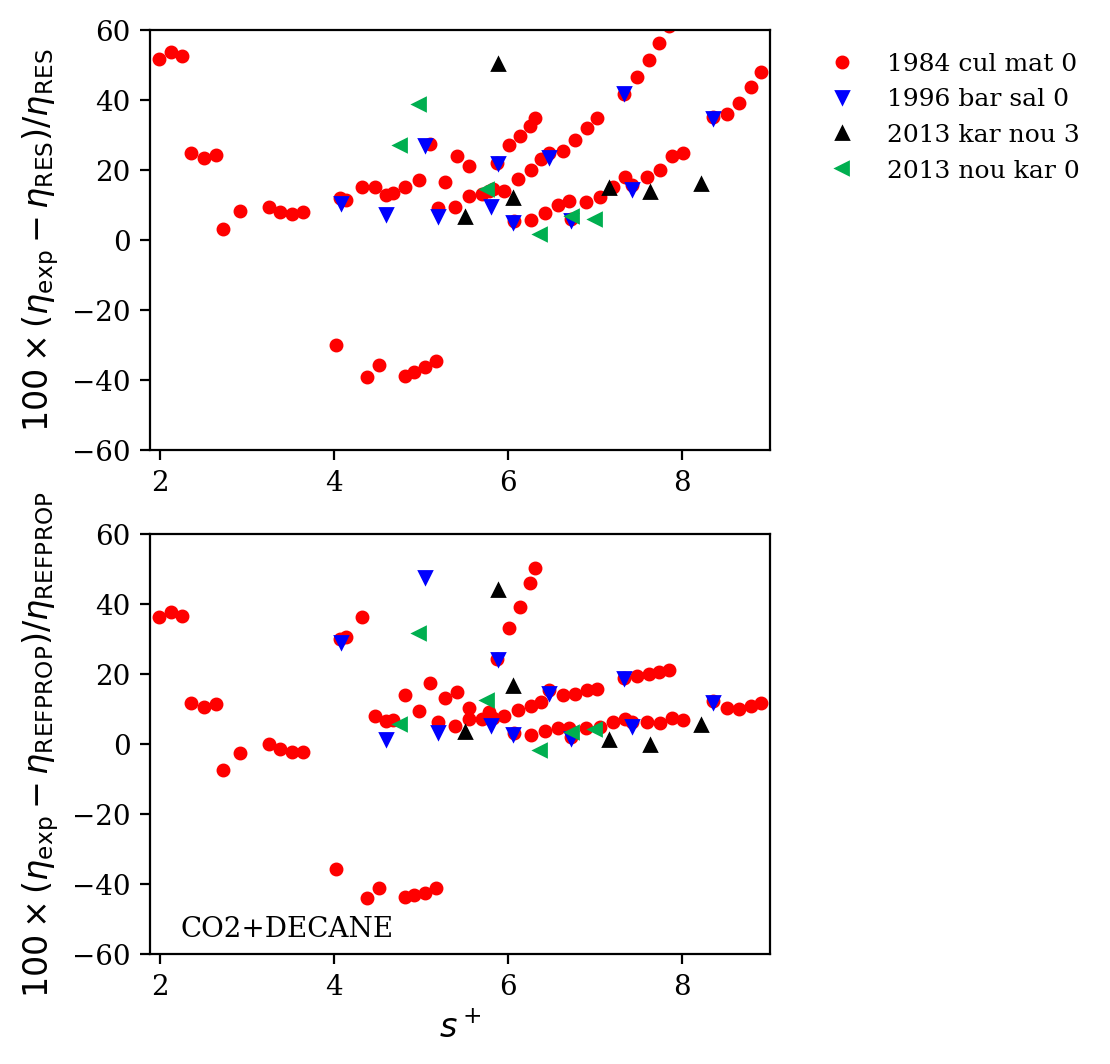

Supplement: Supplementary file 1 — je4c00451_si_001.zip [file je4c00451_si_001.zip › supporting_information/mix_dev_exp_res_ecs/CO2+DECANE.png]

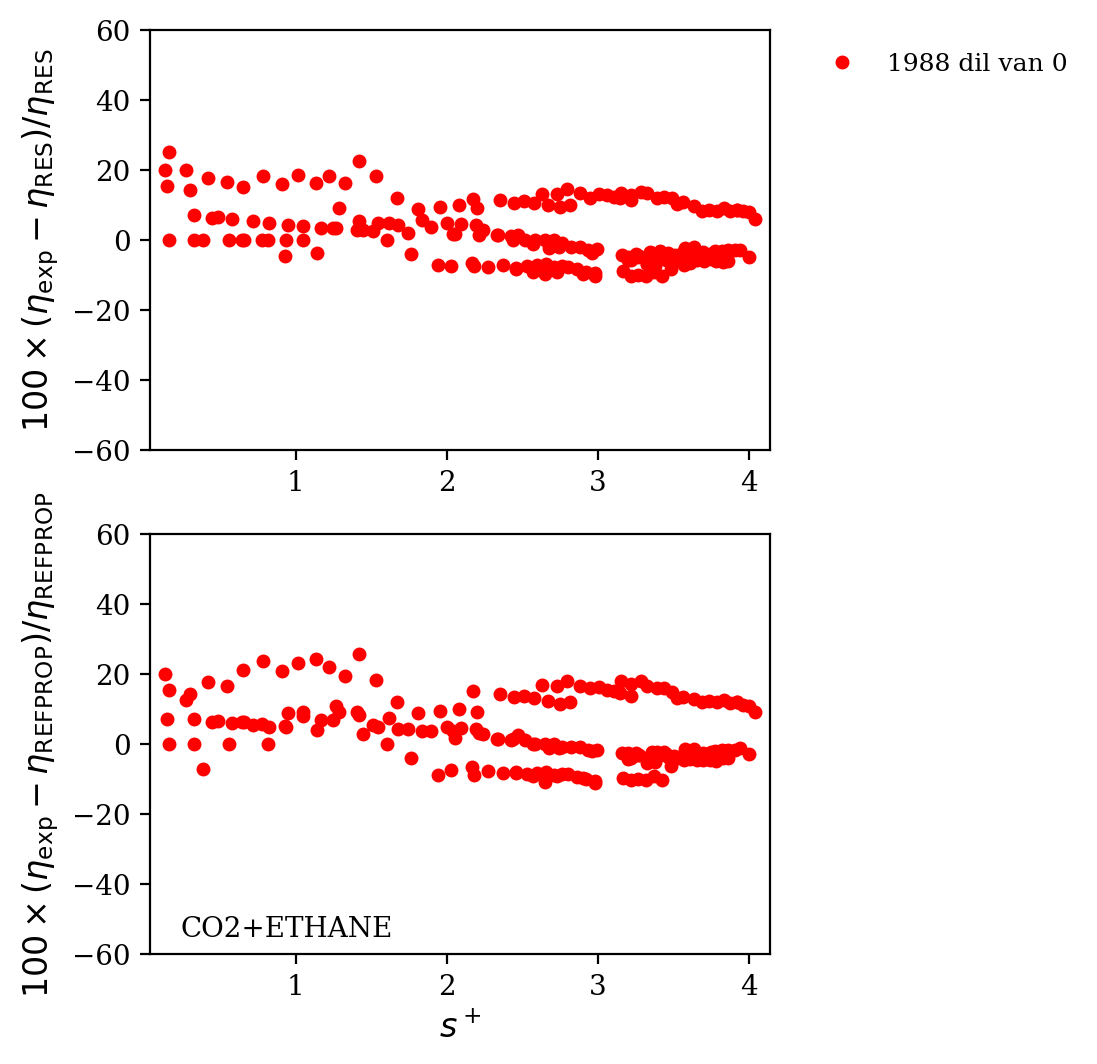

Supplement: Supplementary file 1 — je4c00451_si_001.zip [file je4c00451_si_001.zip › supporting_information/mix_dev_exp_res_ecs/CO2+ETHANE.png]

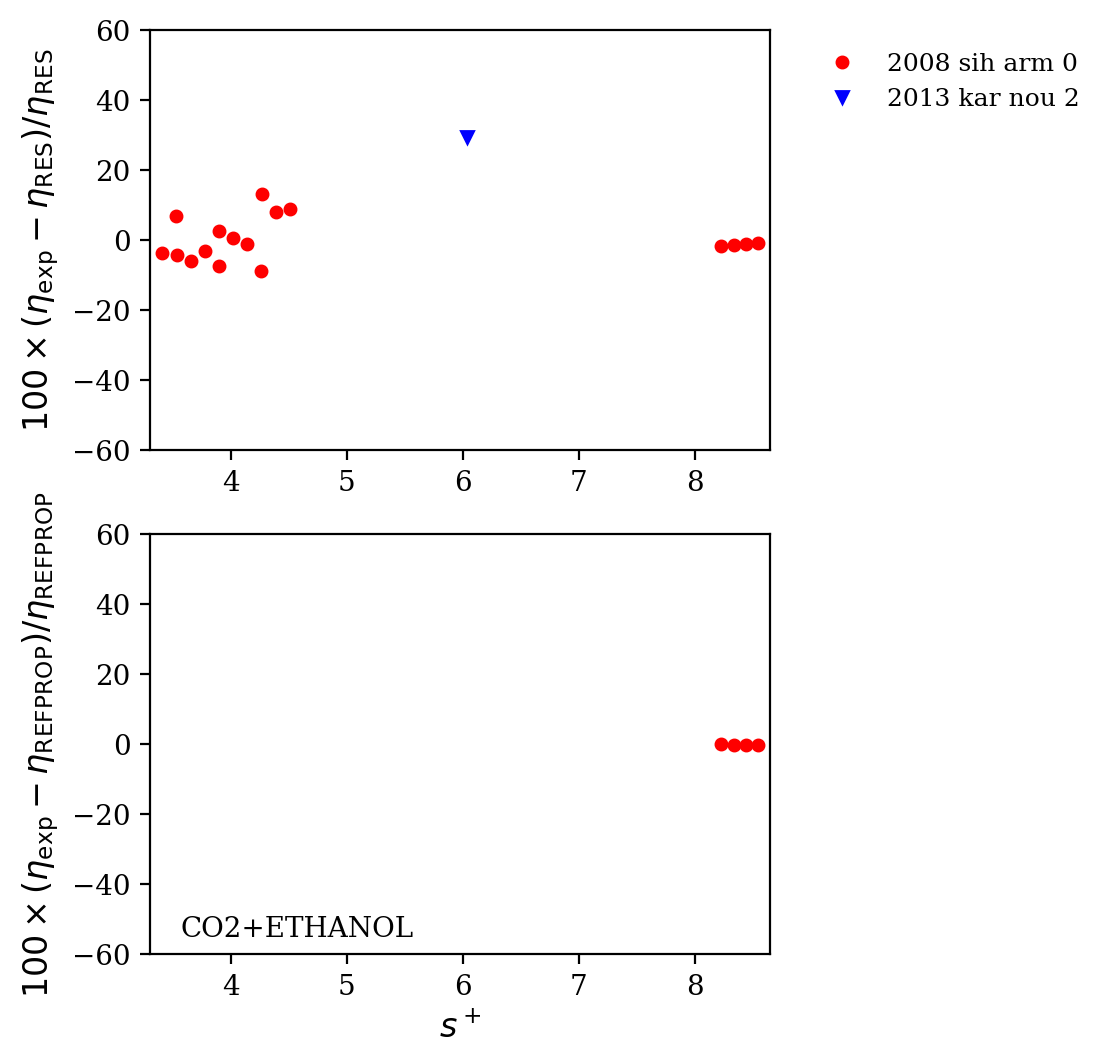

Supplement: Supplementary file 1 — je4c00451_si_001.zip [file je4c00451_si_001.zip › supporting_information/mix_dev_exp_res_ecs/CO2+ETHANOL.png]

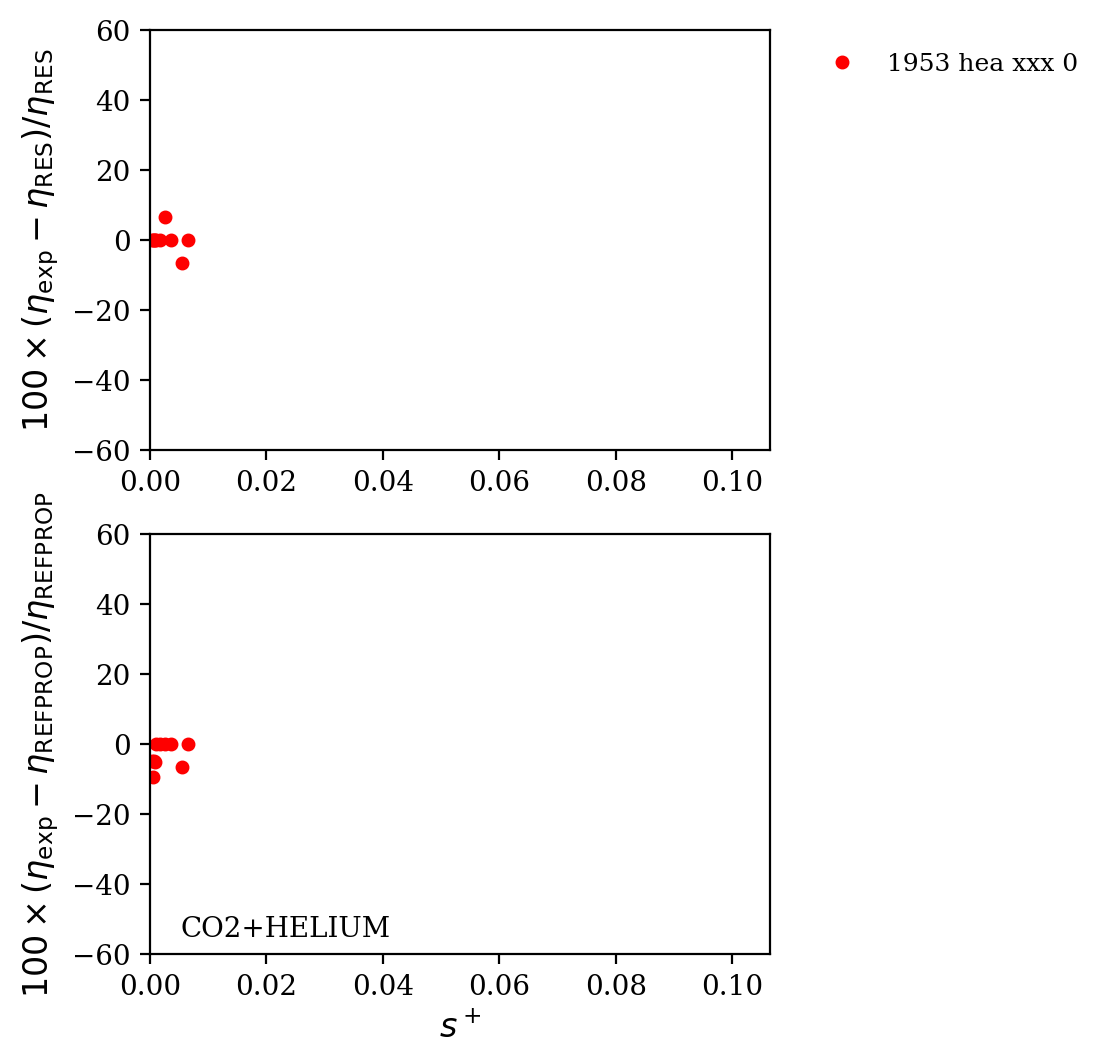

Supplement: Supplementary file 1 — je4c00451_si_001.zip [file je4c00451_si_001.zip › supporting_information/mix_dev_exp_res_ecs/CO2+HELIUM.png]

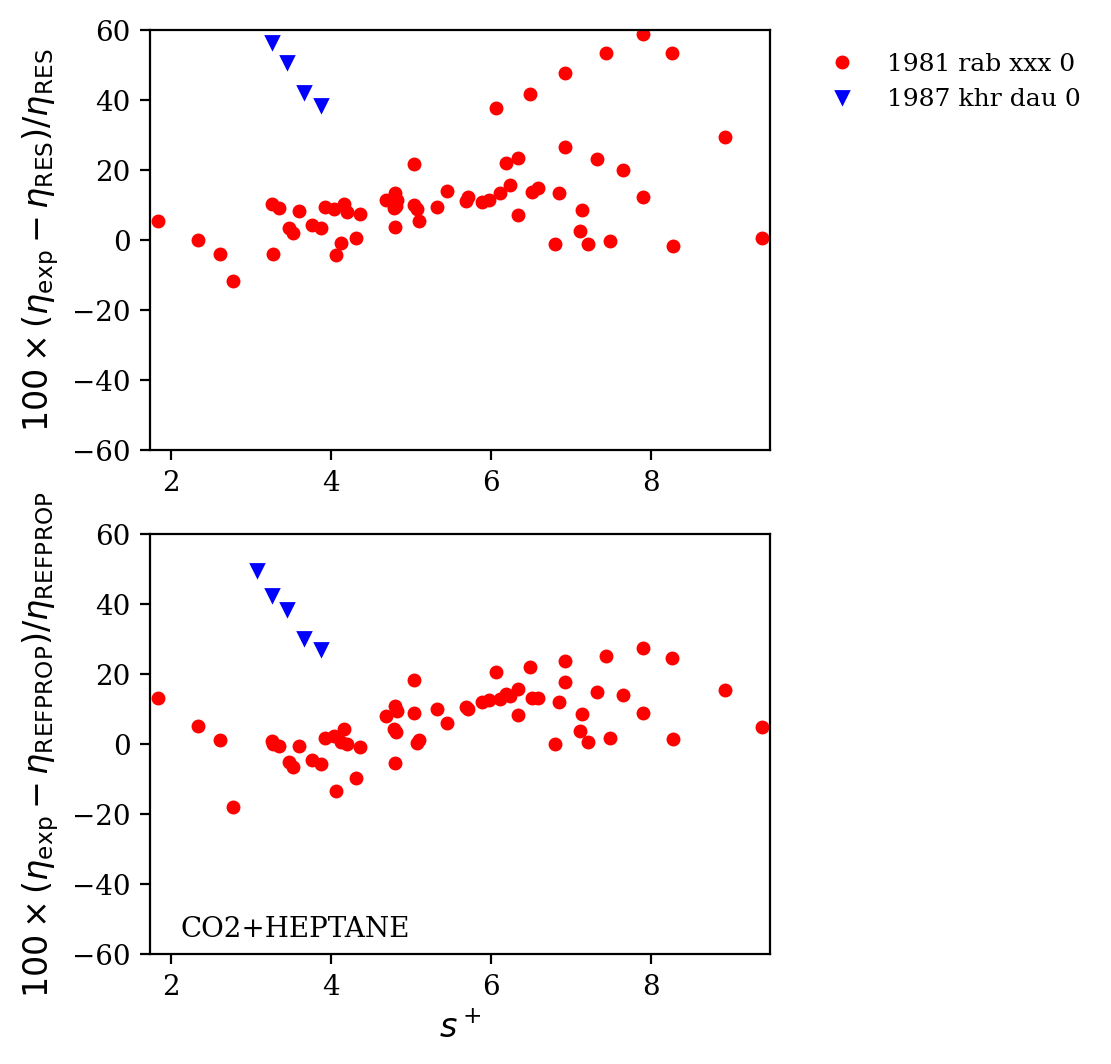

Supplement: Supplementary file 1 — je4c00451_si_001.zip [file je4c00451_si_001.zip › supporting_information/mix_dev_exp_res_ecs/CO2+HEPTANE.png]

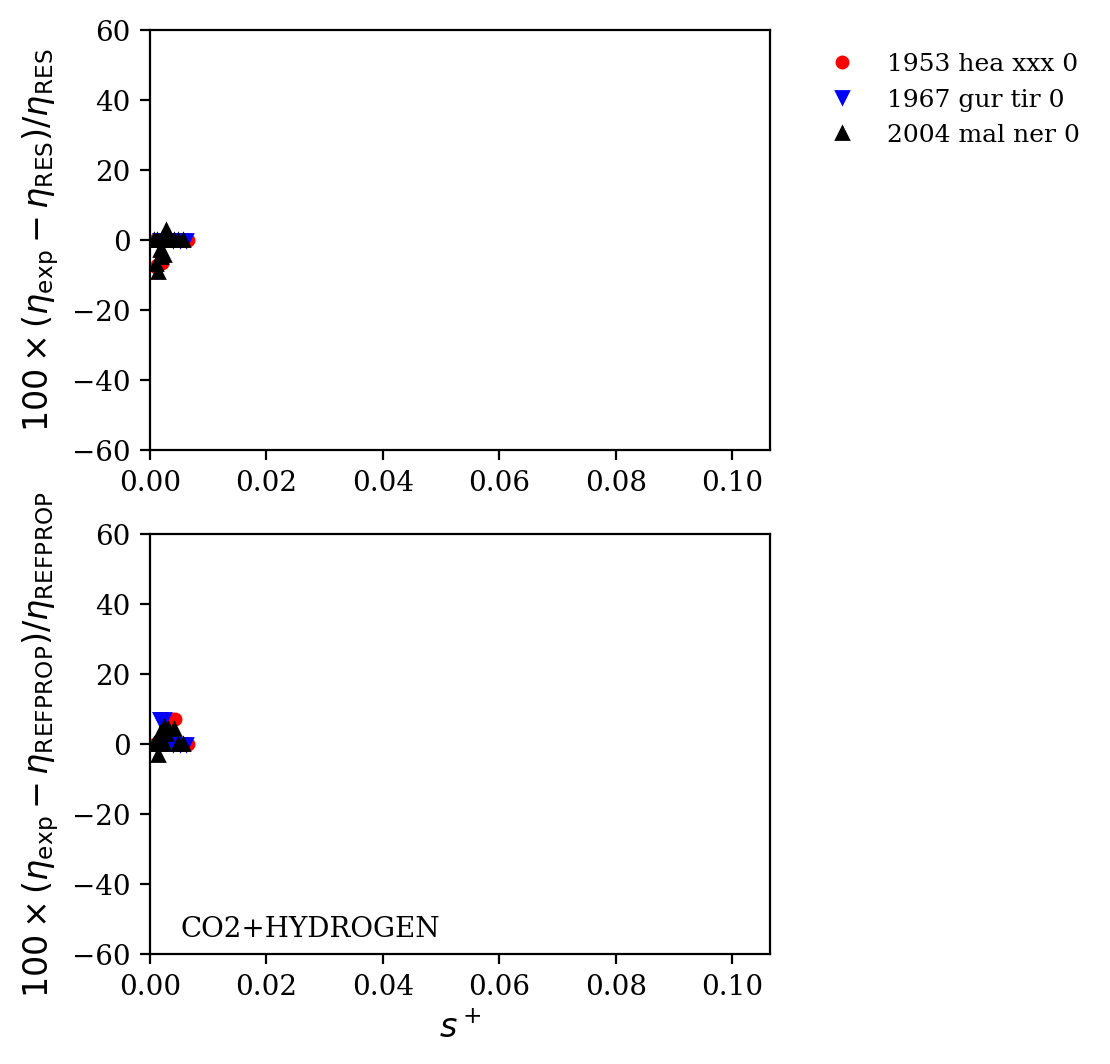

Supplement: Supplementary file 1 — je4c00451_si_001.zip [file je4c00451_si_001.zip › supporting_information/mix_dev_exp_res_ecs/CO2+HYDROGEN.png]

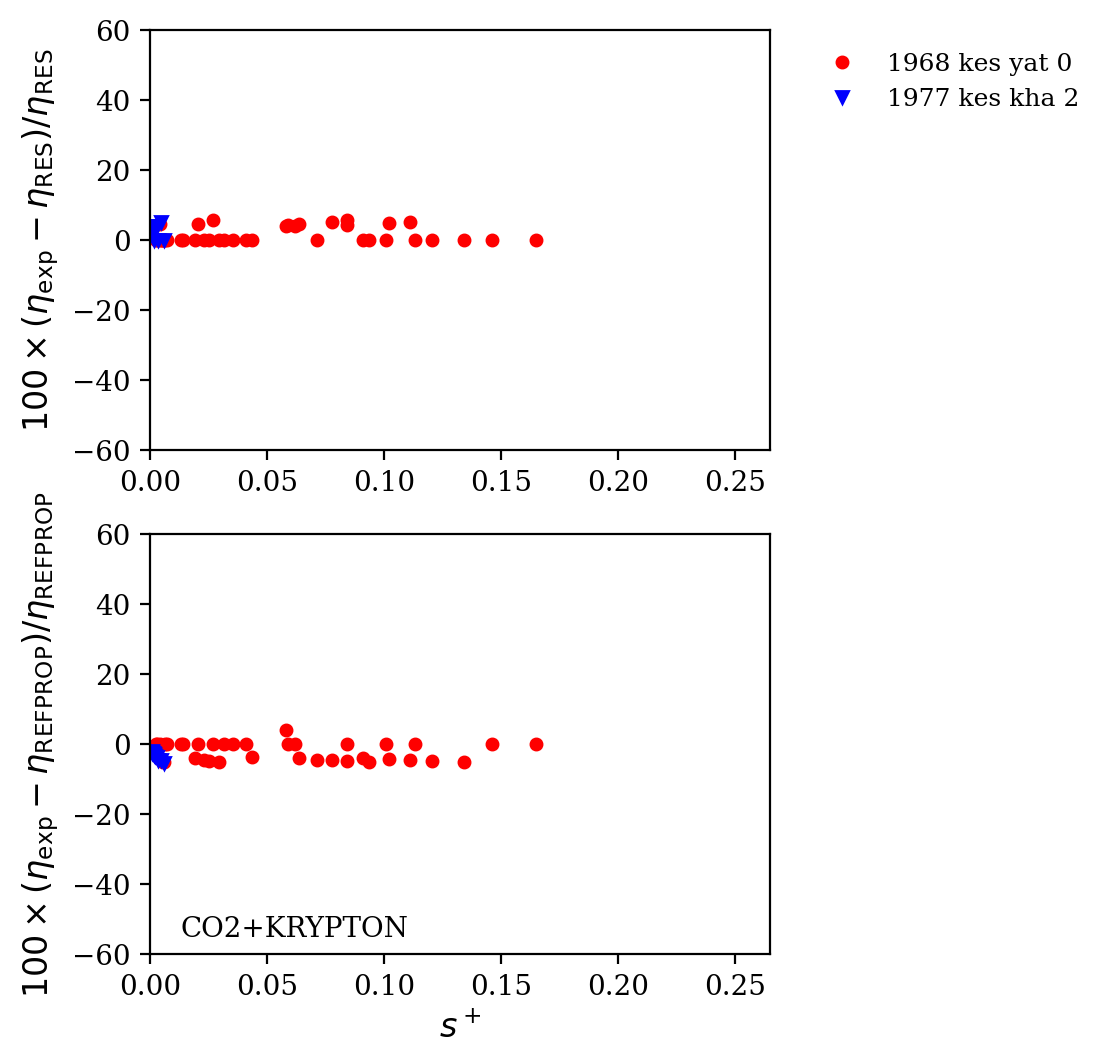

Supplement: Supplementary file 1 — je4c00451_si_001.zip [file je4c00451_si_001.zip › supporting_information/mix_dev_exp_res_ecs/CO2+KRYPTON.png]

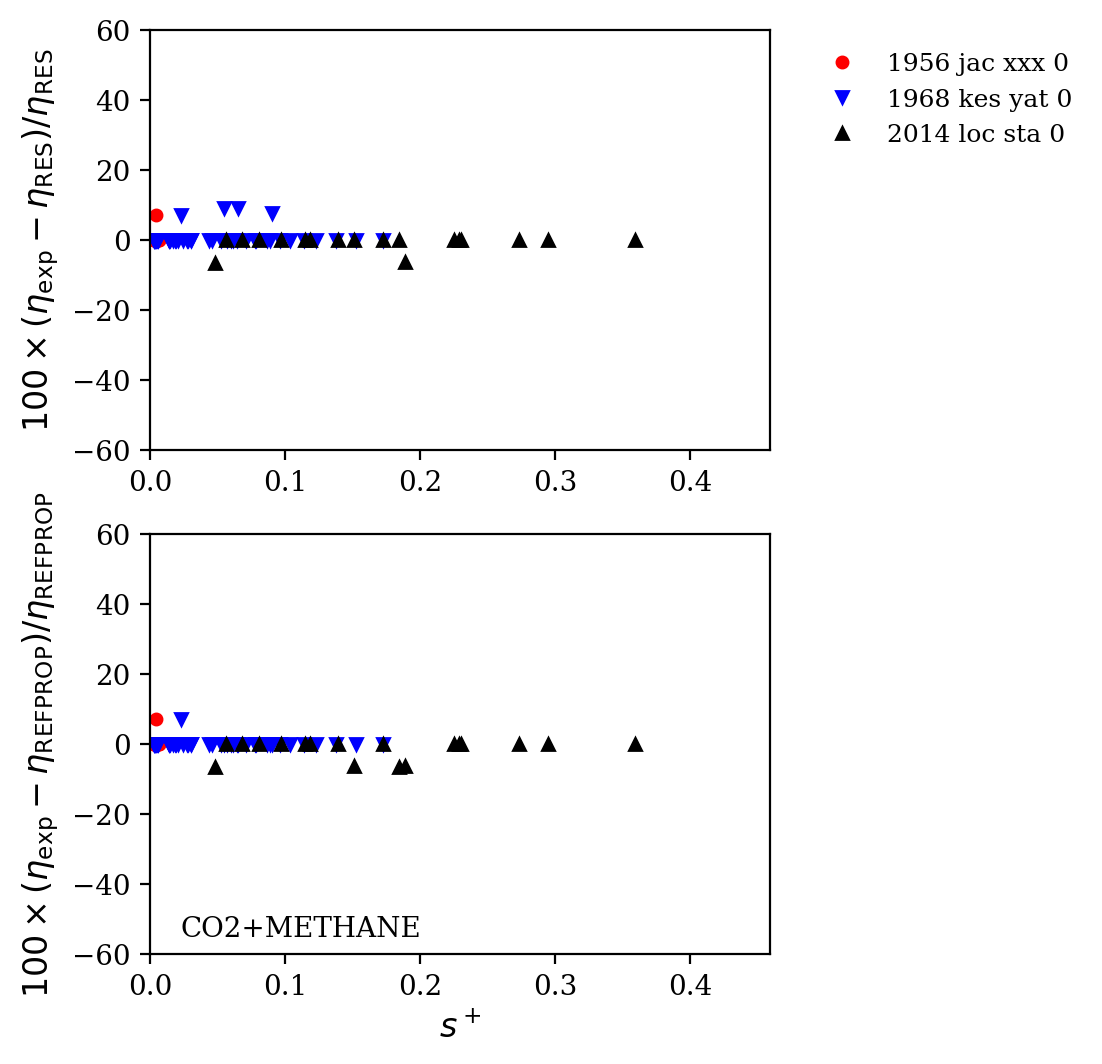

Supplement: Supplementary file 1 — je4c00451_si_001.zip [file je4c00451_si_001.zip › supporting_information/mix_dev_exp_res_ecs/CO2+METHANE.png]

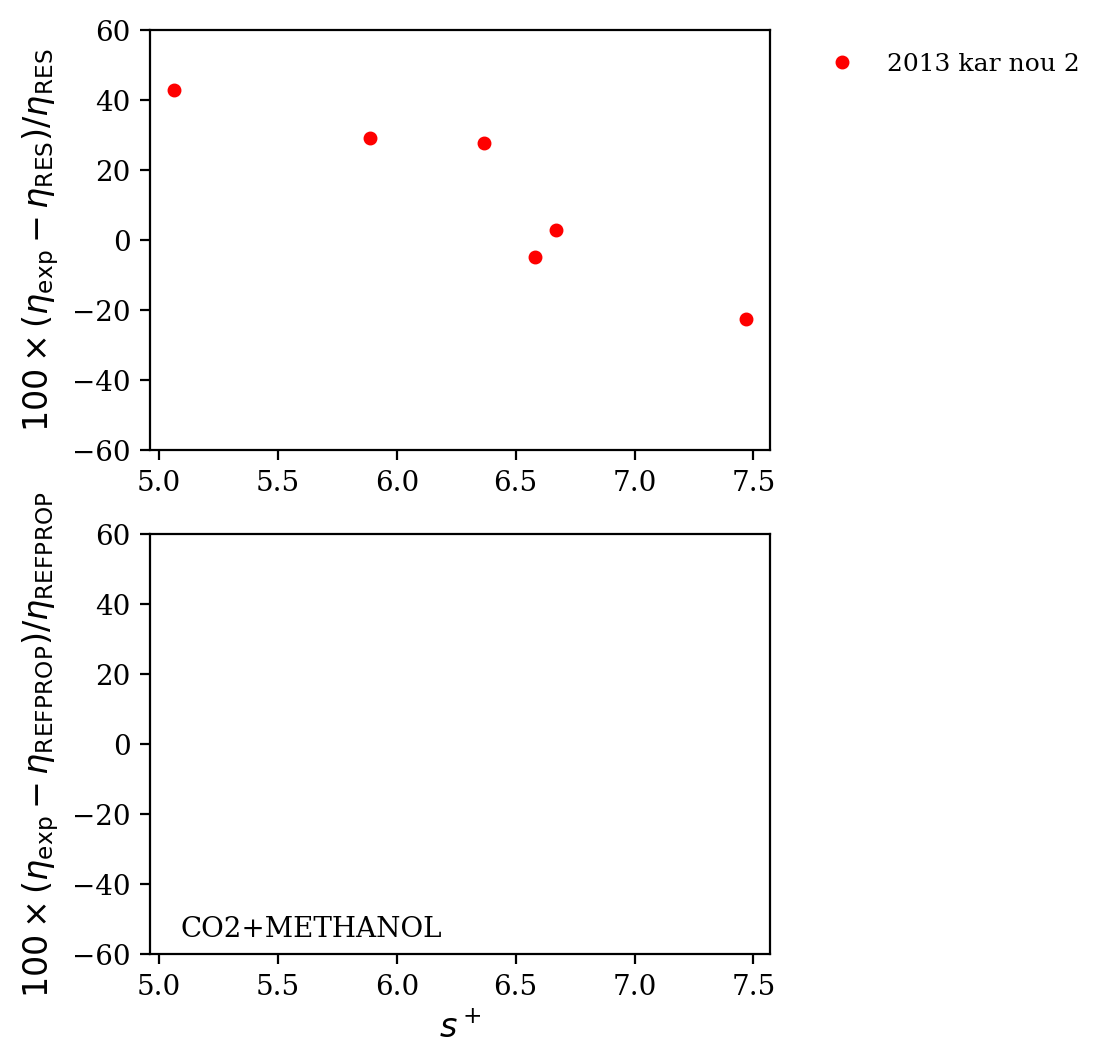

Supplement: Supplementary file 1 — je4c00451_si_001.zip [file je4c00451_si_001.zip › supporting_information/mix_dev_exp_res_ecs/CO2+METHANOL.png]

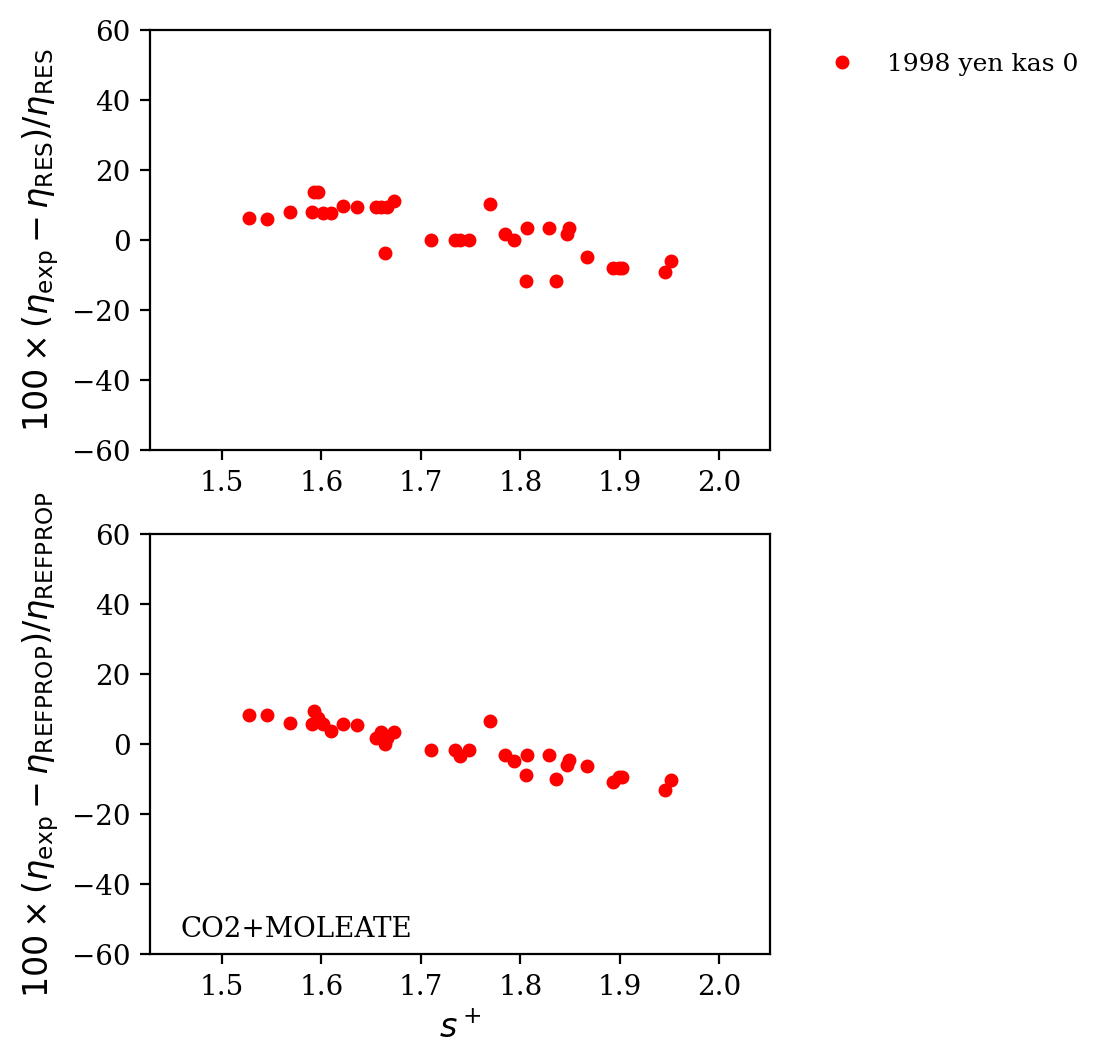

Supplement: Supplementary file 1 — je4c00451_si_001.zip [file je4c00451_si_001.zip › supporting_information/mix_dev_exp_res_ecs/CO2+MOLEATE.png]

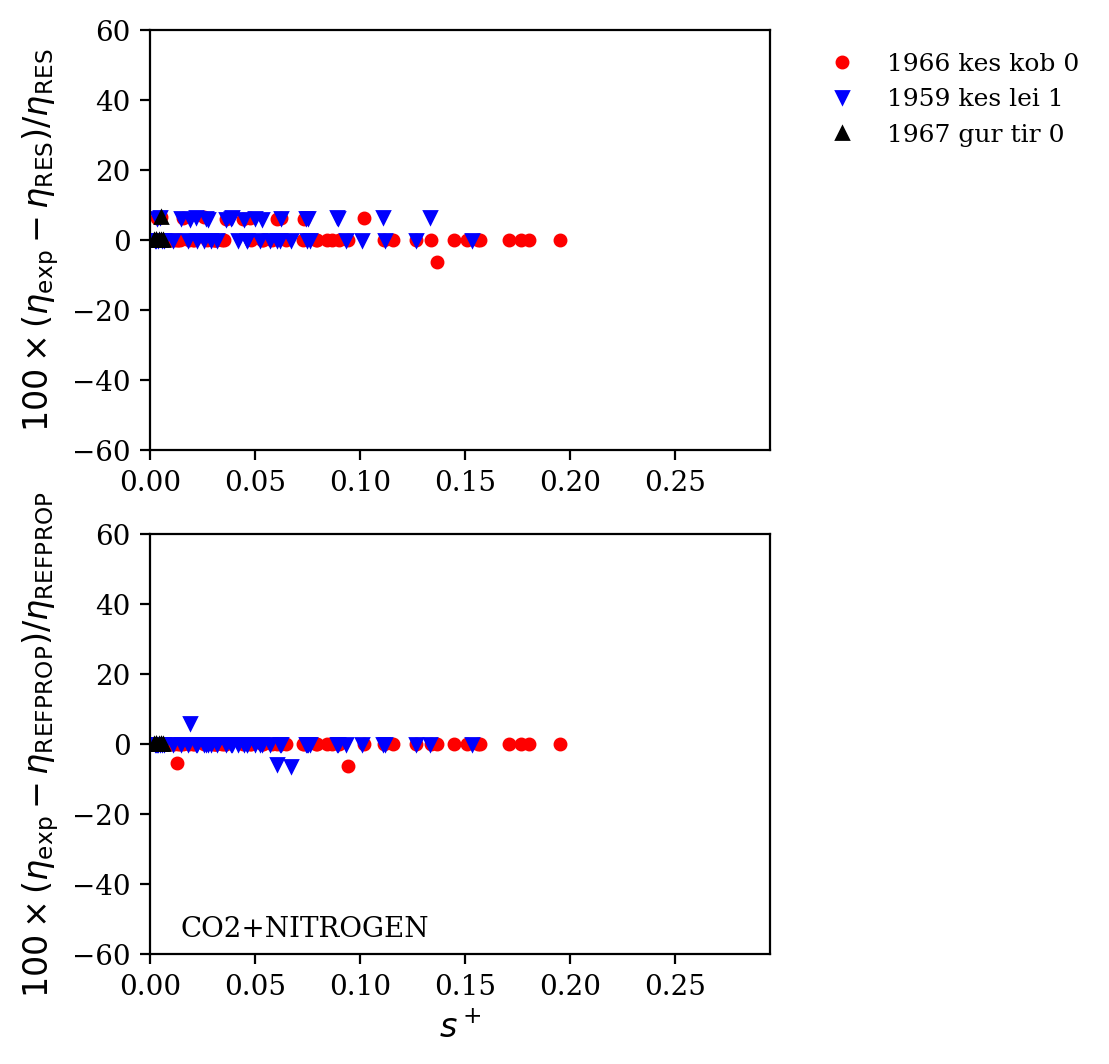

Supplement: Supplementary file 1 — je4c00451_si_001.zip [file je4c00451_si_001.zip › supporting_information/mix_dev_exp_res_ecs/CO2+NITROGEN.png]

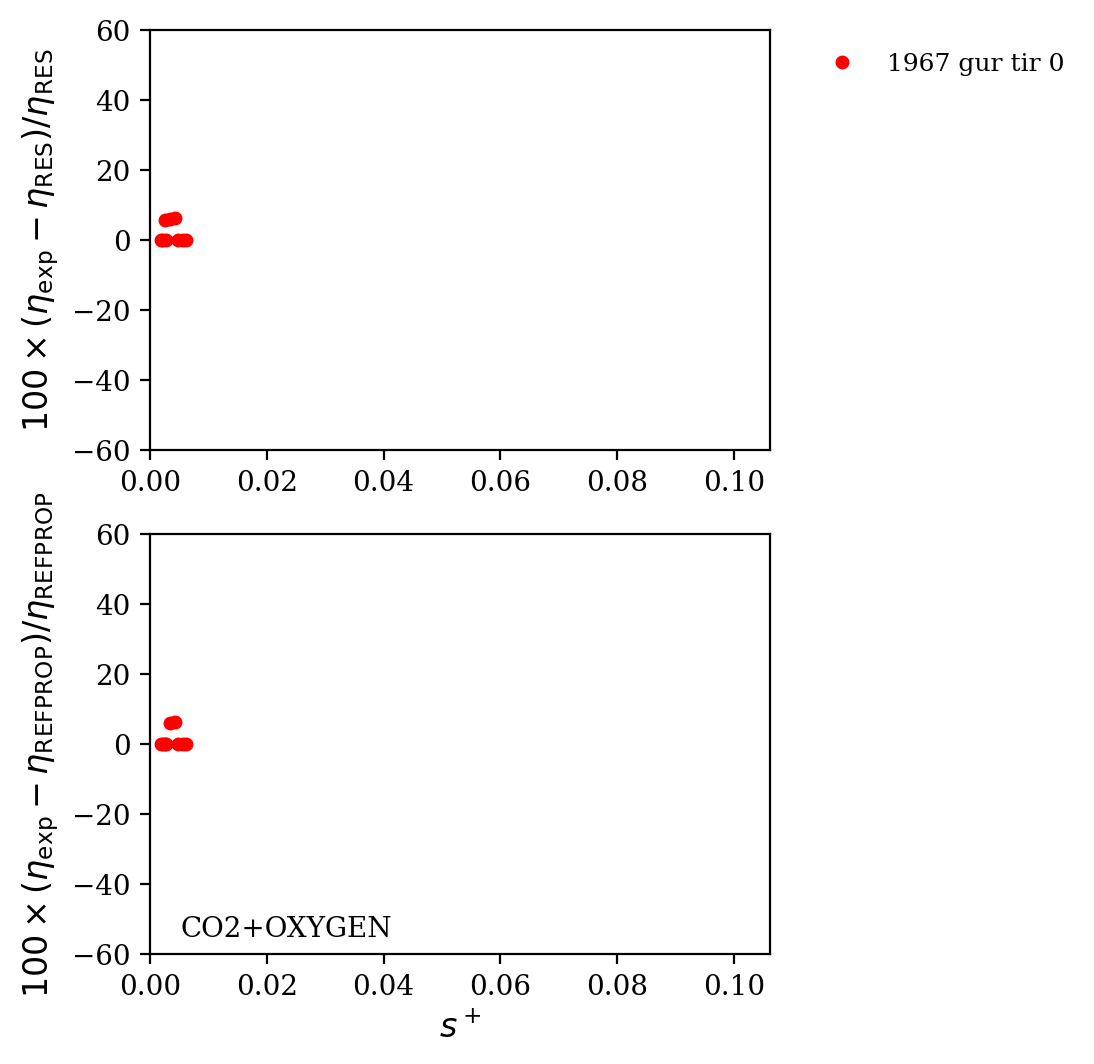

Supplement: Supplementary file 1 — je4c00451_si_001.zip [file je4c00451_si_001.zip › supporting_information/mix_dev_exp_res_ecs/CO2+OXYGEN.png]

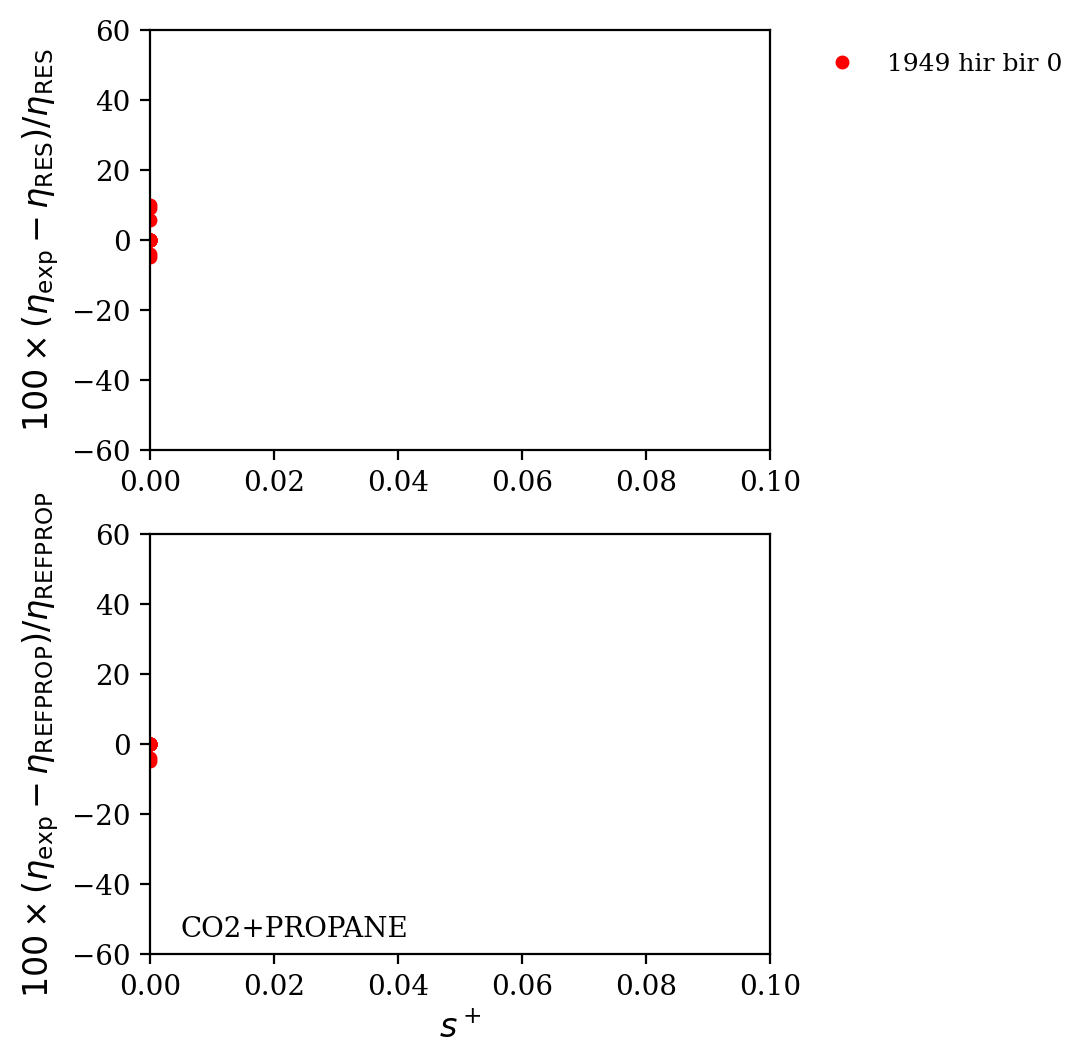

Supplement: Supplementary file 1 — je4c00451_si_001.zip [file je4c00451_si_001.zip › supporting_information/mix_dev_exp_res_ecs/CO2+PROPANE.png]

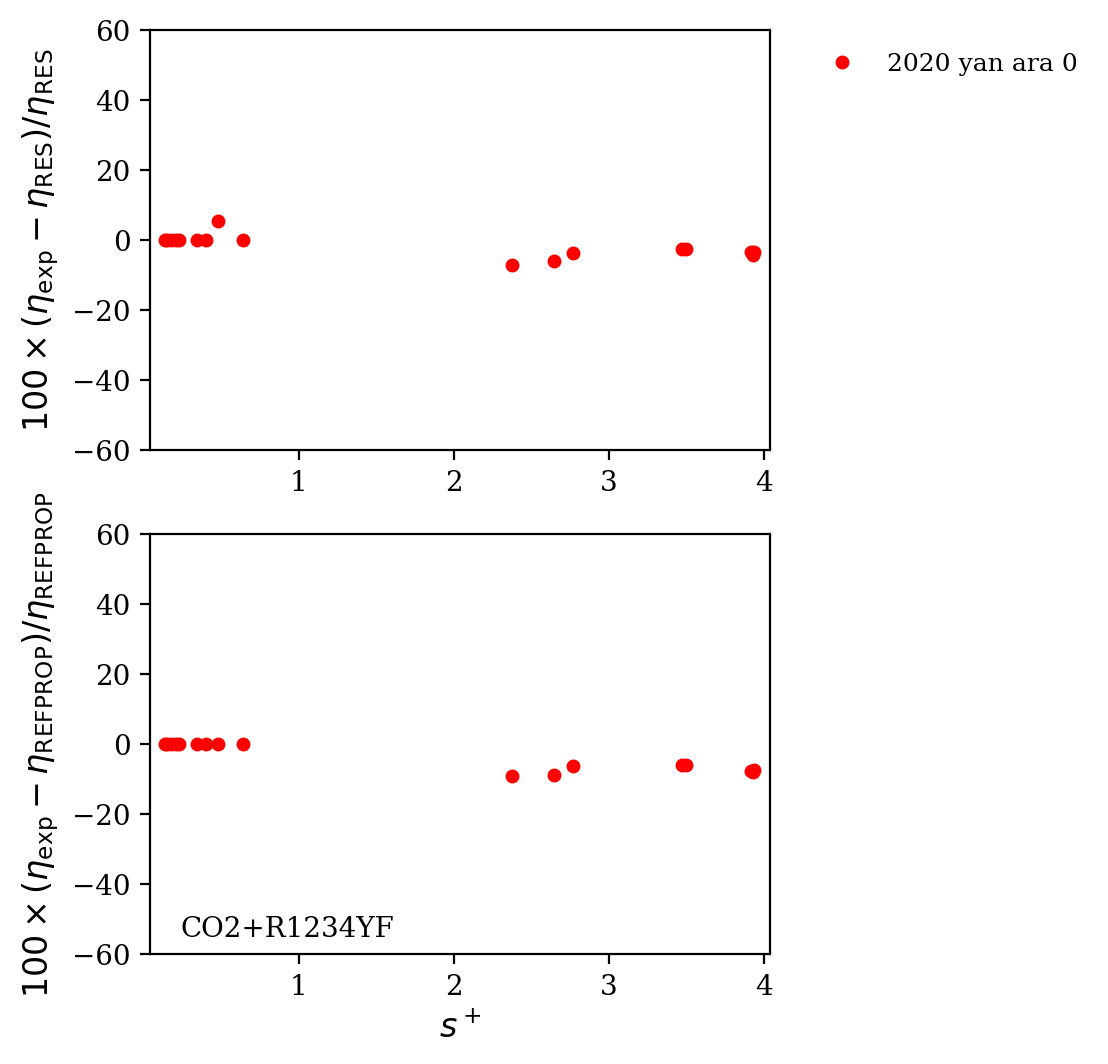

Supplement: Supplementary file 1 — je4c00451_si_001.zip [file je4c00451_si_001.zip › supporting_information/mix_dev_exp_res_ecs/CO2+R1234YF.png]

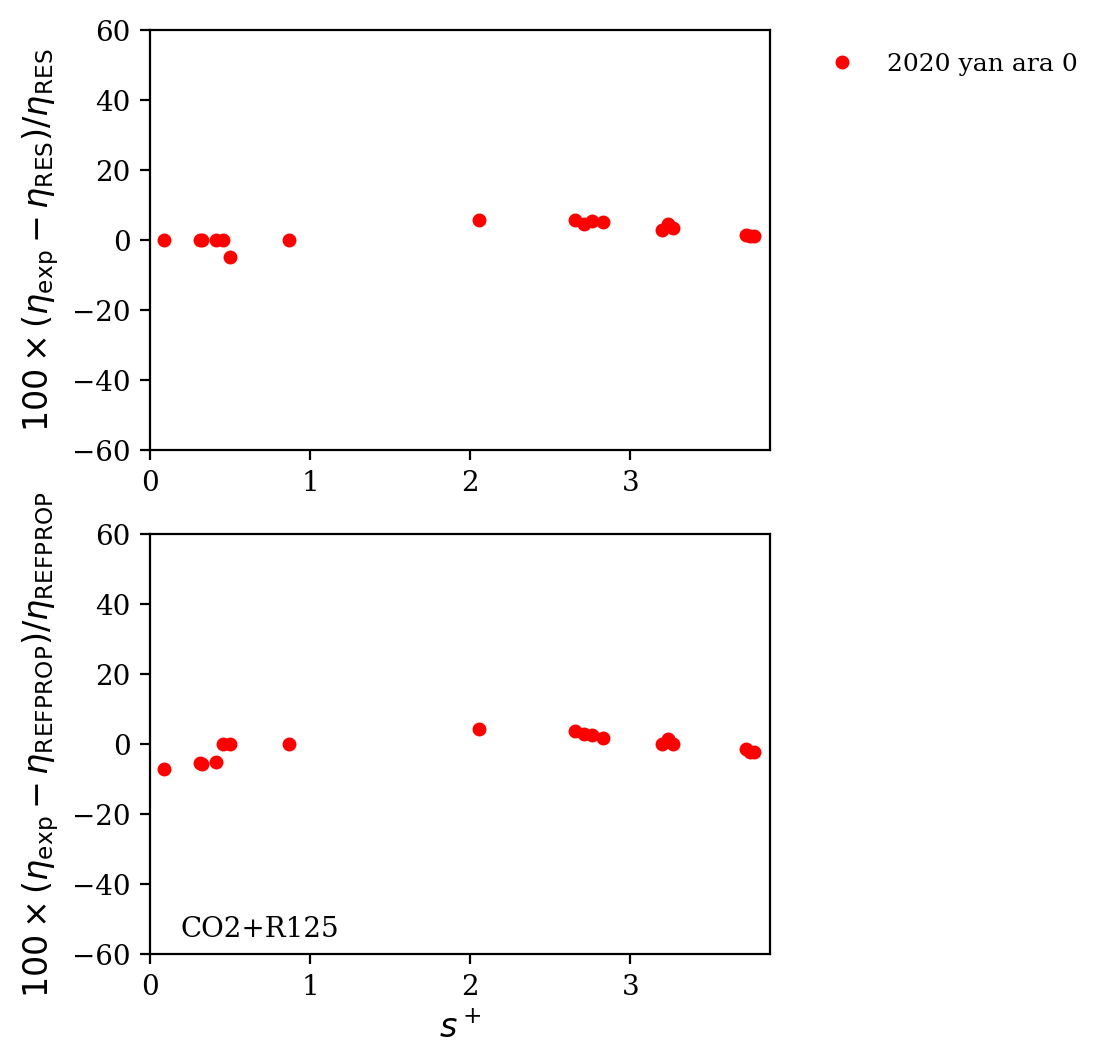

Supplement: Supplementary file 1 — je4c00451_si_001.zip [file je4c00451_si_001.zip › supporting_information/mix_dev_exp_res_ecs/CO2+R125.png]

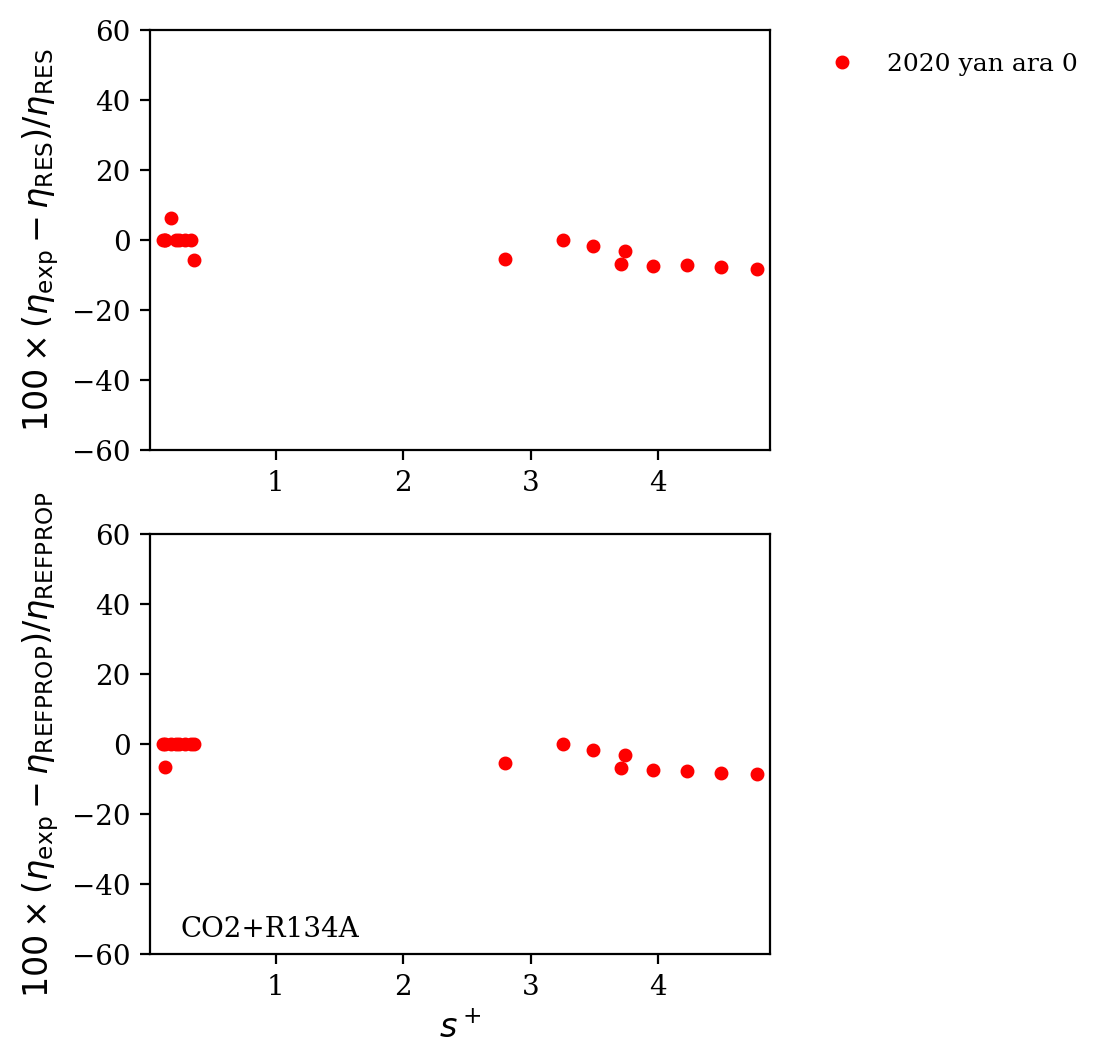

Supplement: Supplementary file 1 — je4c00451_si_001.zip [file je4c00451_si_001.zip › supporting_information/mix_dev_exp_res_ecs/CO2+R134A.png]

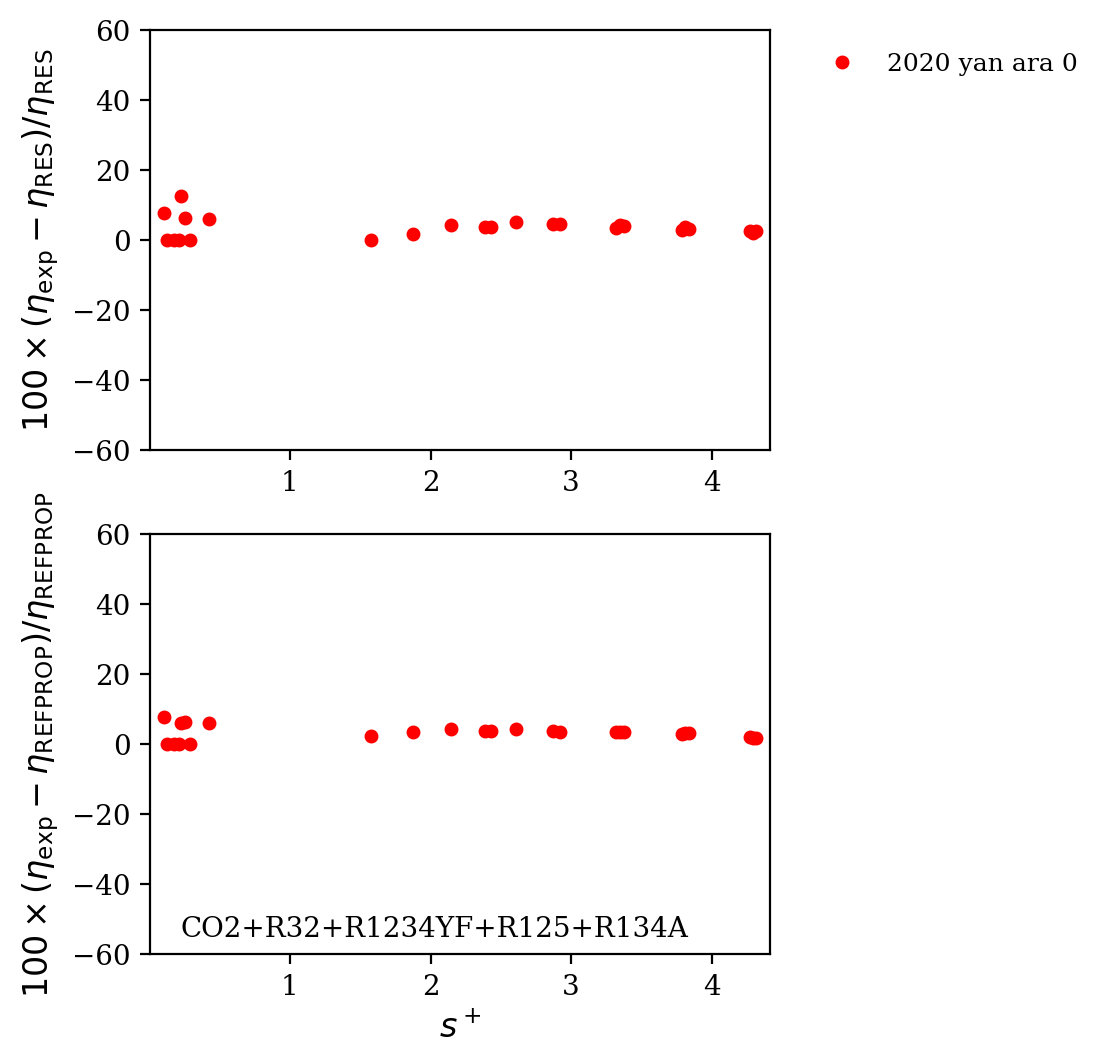

Supplement: Supplementary file 1 — je4c00451_si_001.zip [file je4c00451_si_001.zip › supporting_information/mix_dev_exp_res_ecs/CO2+R32+R1234YF+R125+R134A.png]

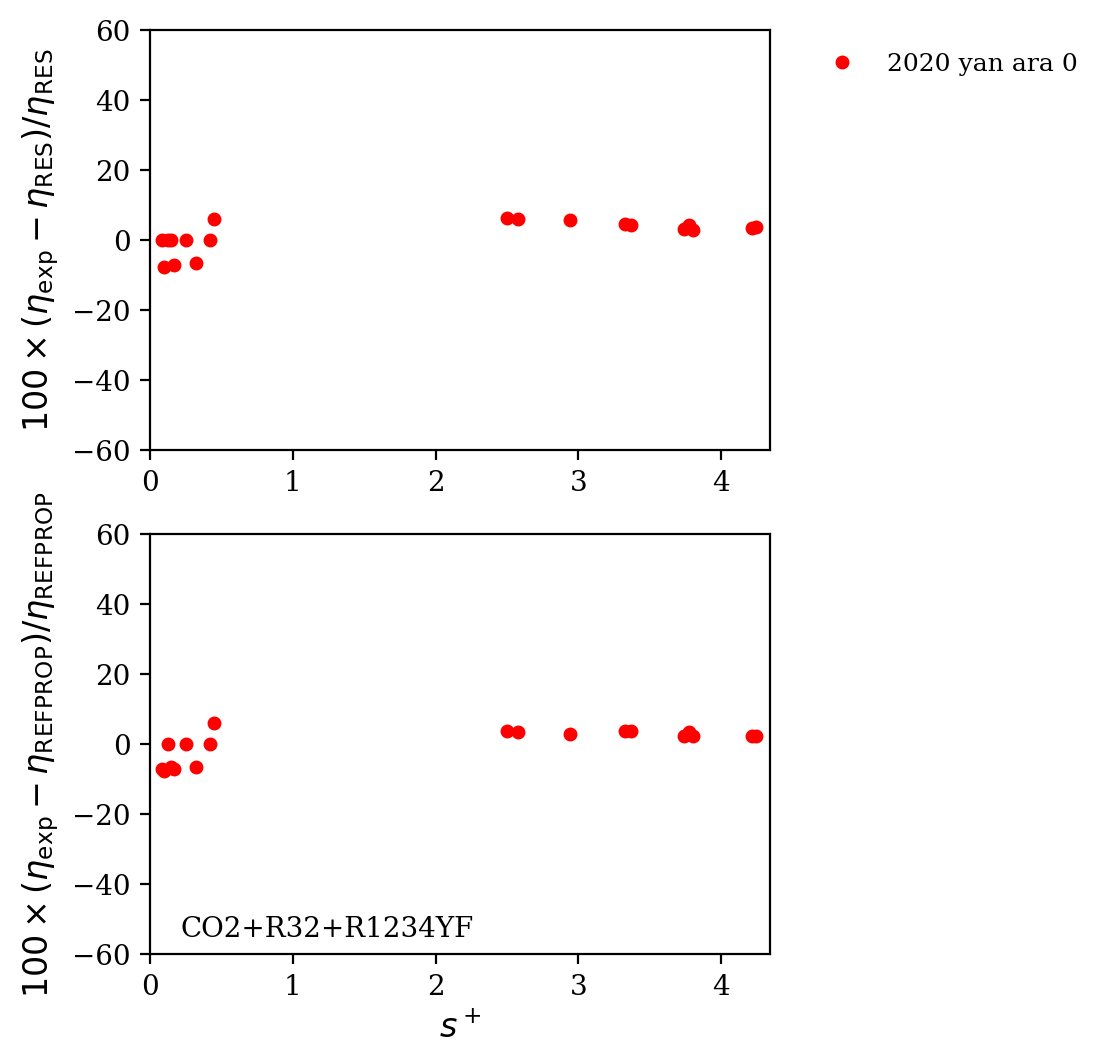

Supplement: Supplementary file 1 — je4c00451_si_001.zip [file je4c00451_si_001.zip › supporting_information/mix_dev_exp_res_ecs/CO2+R32+R1234YF.png]

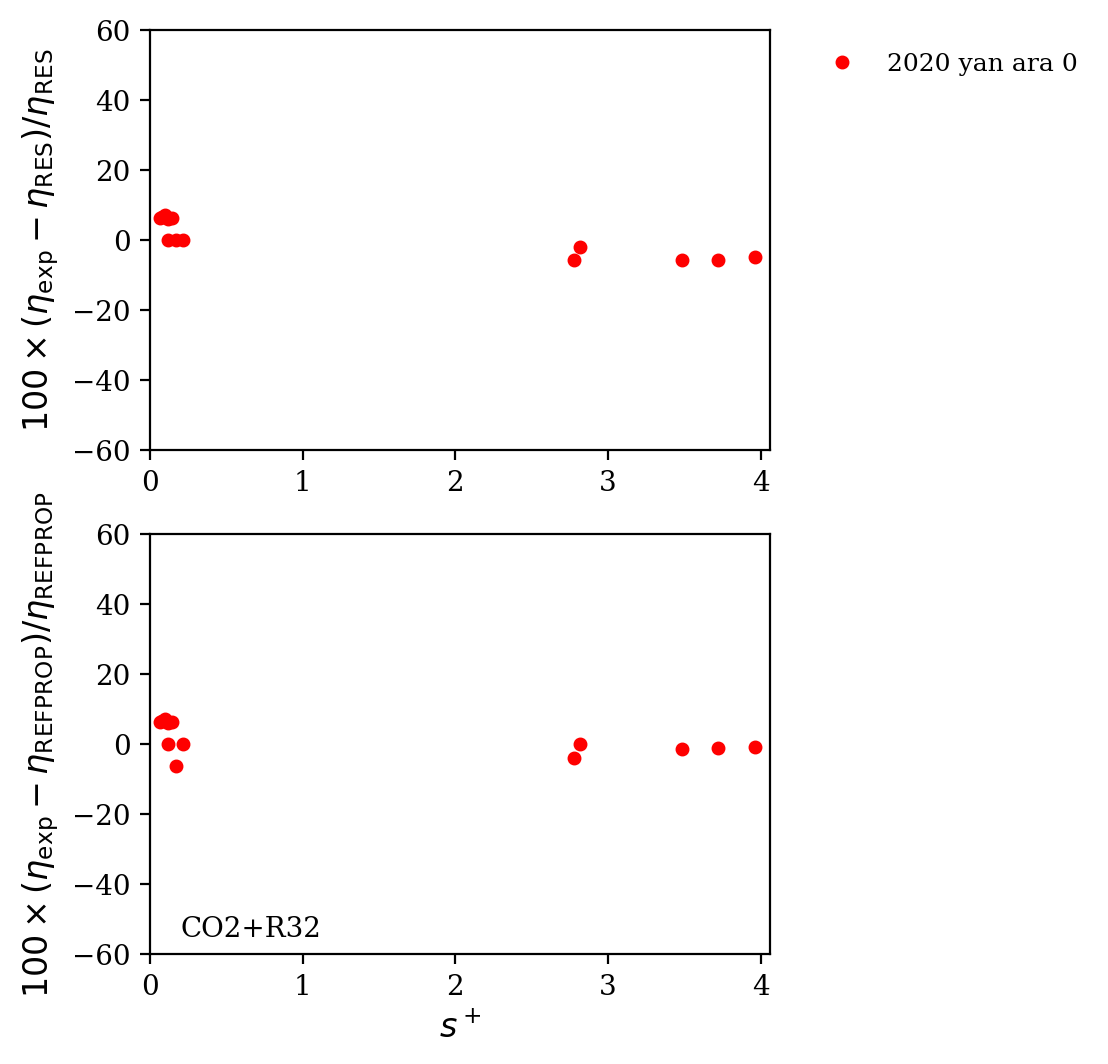

Supplement: Supplementary file 1 — je4c00451_si_001.zip [file je4c00451_si_001.zip › supporting_information/mix_dev_exp_res_ecs/CO2+R32.png]

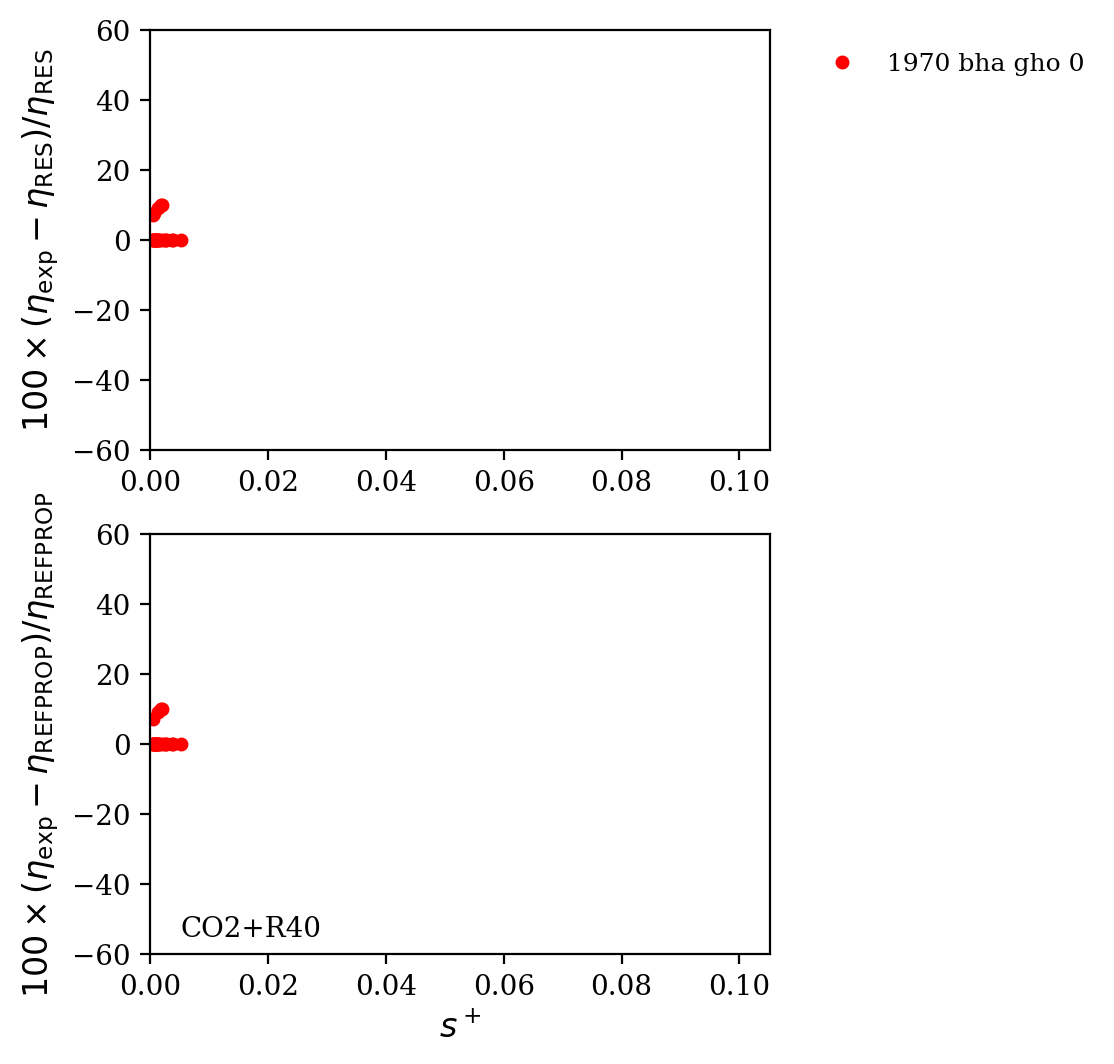

Supplement: Supplementary file 1 — je4c00451_si_001.zip [file je4c00451_si_001.zip › supporting_information/mix_dev_exp_res_ecs/CO2+R40.png]

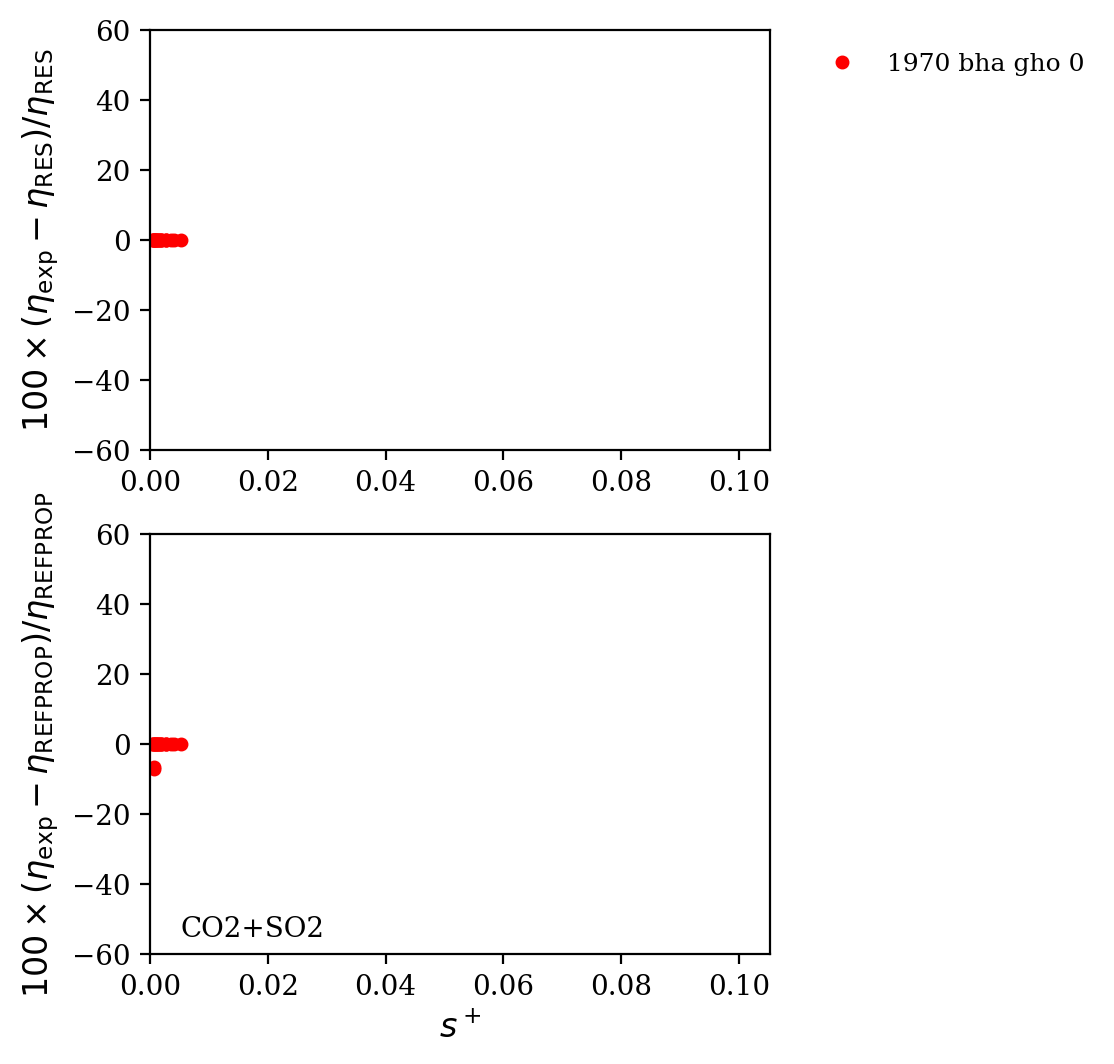

Supplement: Supplementary file 1 — je4c00451_si_001.zip [file je4c00451_si_001.zip › supporting_information/mix_dev_exp_res_ecs/CO2+SO2.png]

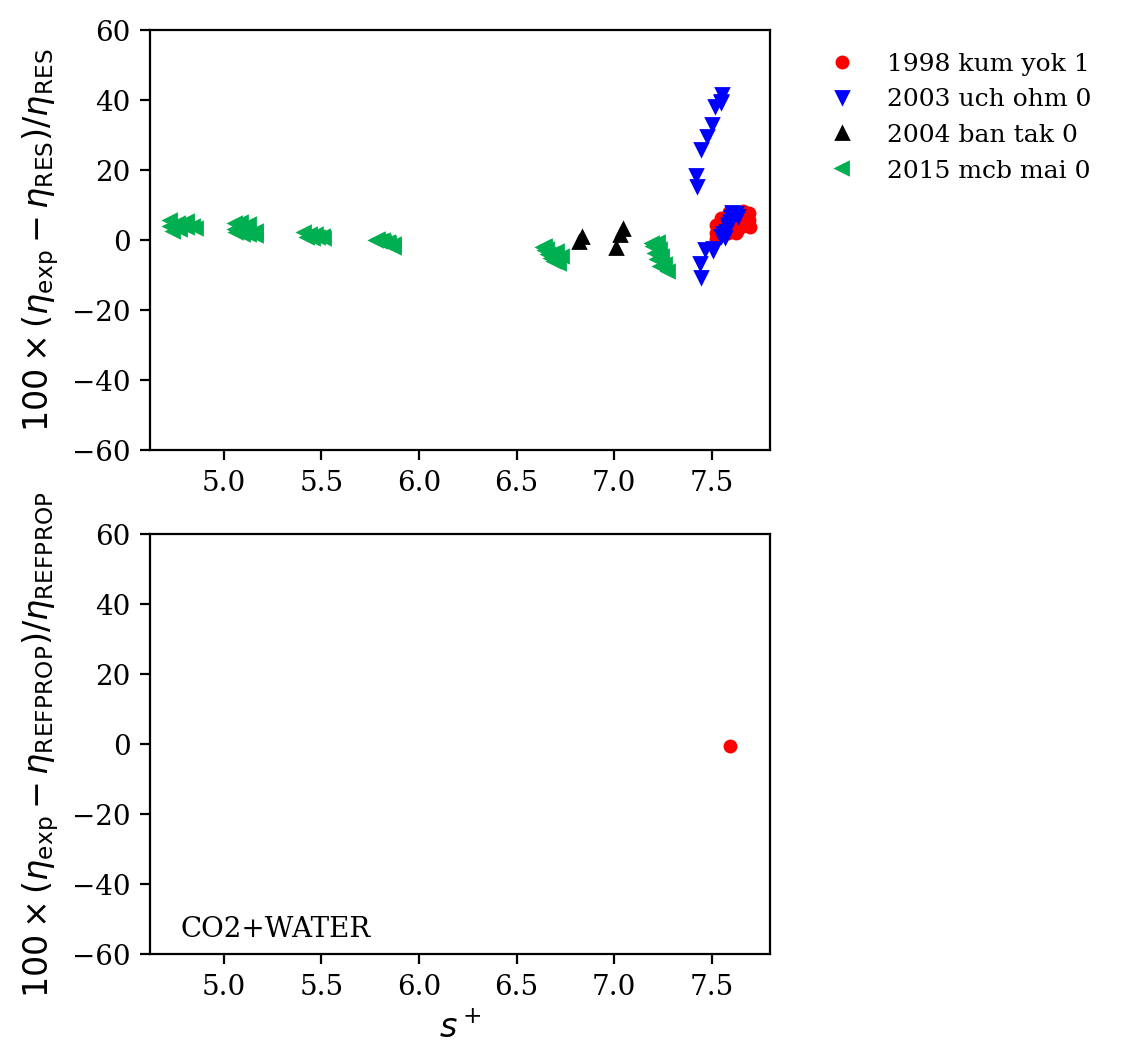

Supplement: Supplementary file 1 — je4c00451_si_001.zip [file je4c00451_si_001.zip › supporting_information/mix_dev_exp_res_ecs/CO2+WATER.png]

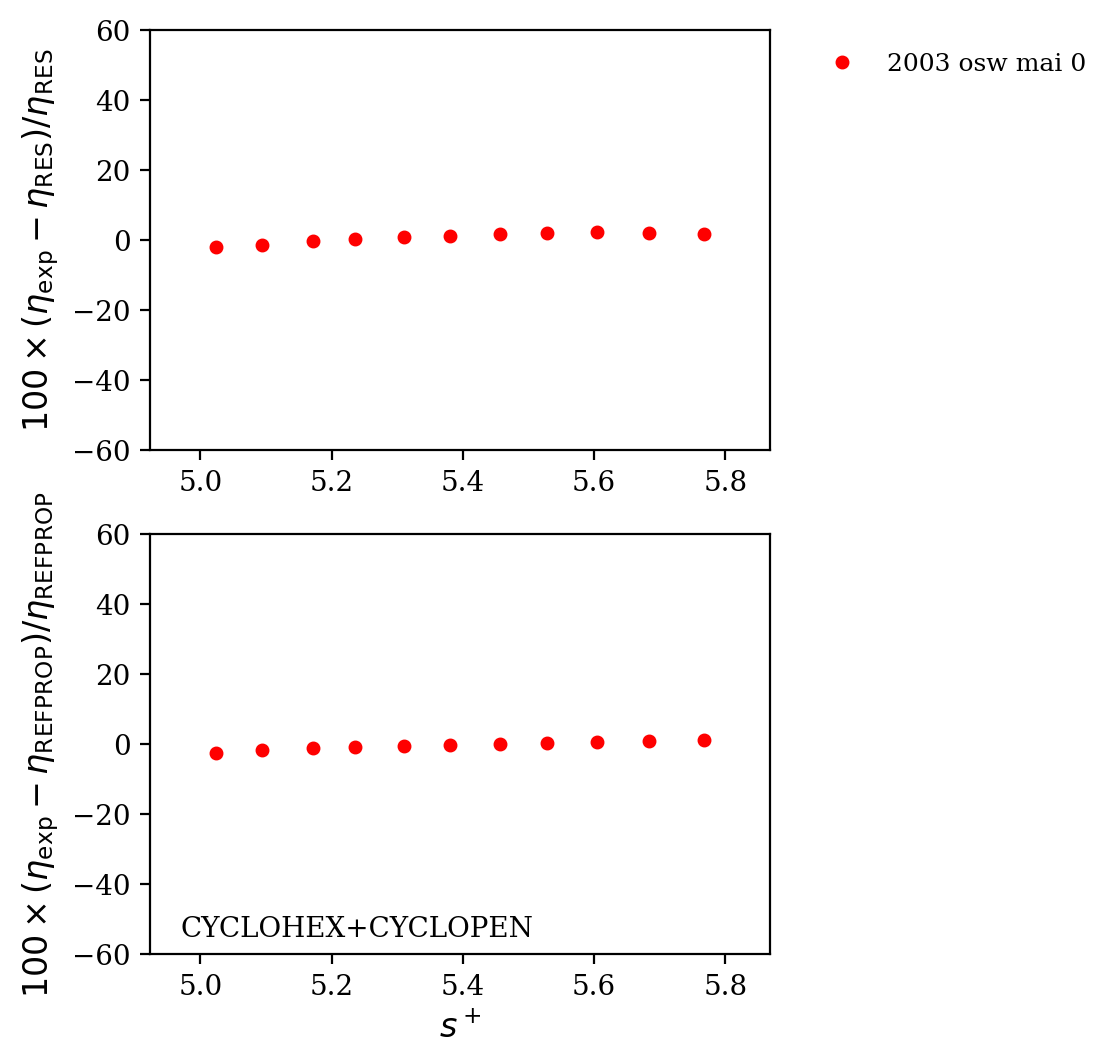

Supplement: Supplementary file 1 — je4c00451_si_001.zip [file je4c00451_si_001.zip › supporting_information/mix_dev_exp_res_ecs/CYCLOHEX+CYCLOPEN.png]

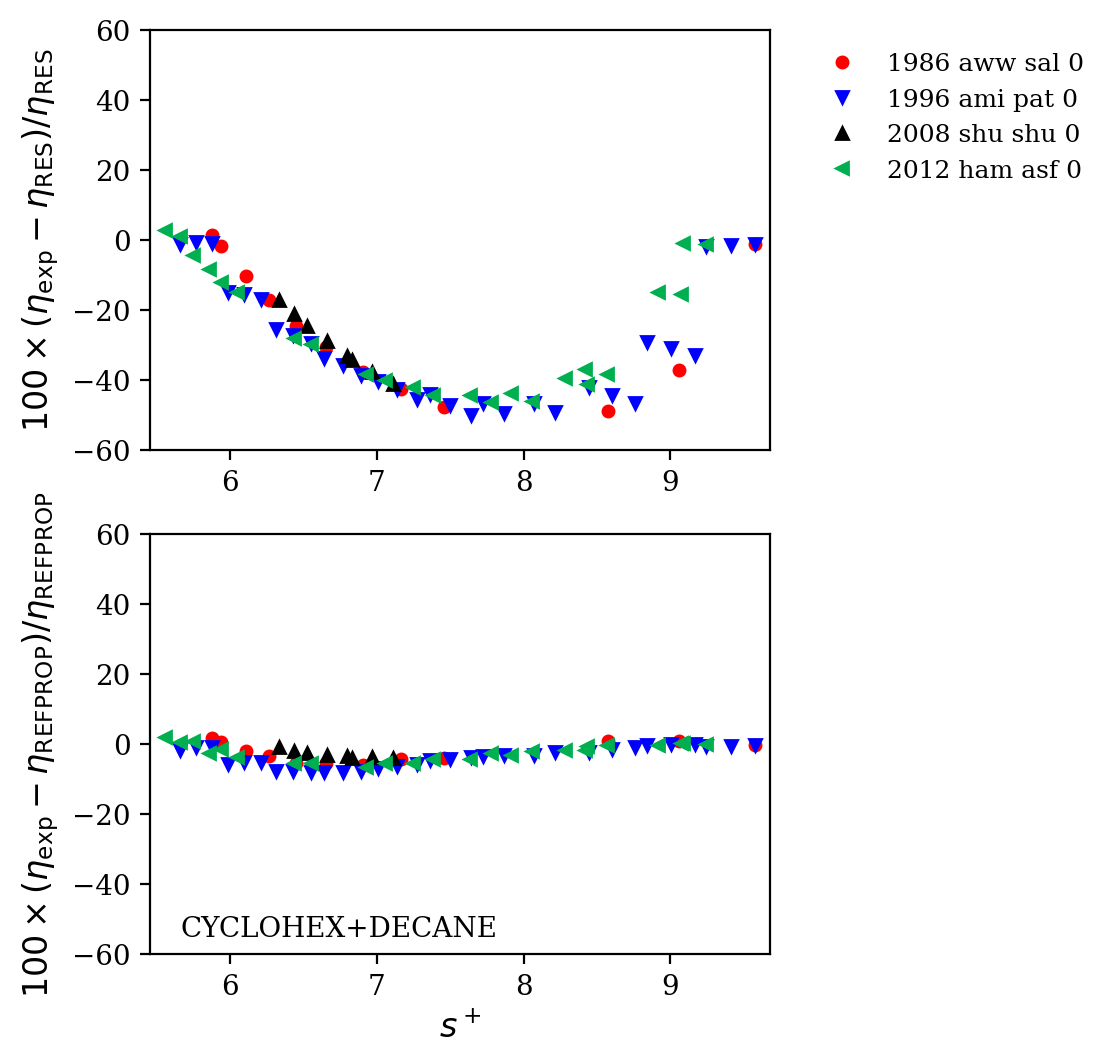

Supplement: Supplementary file 1 — je4c00451_si_001.zip [file je4c00451_si_001.zip › supporting_information/mix_dev_exp_res_ecs/CYCLOHEX+DECANE.png]

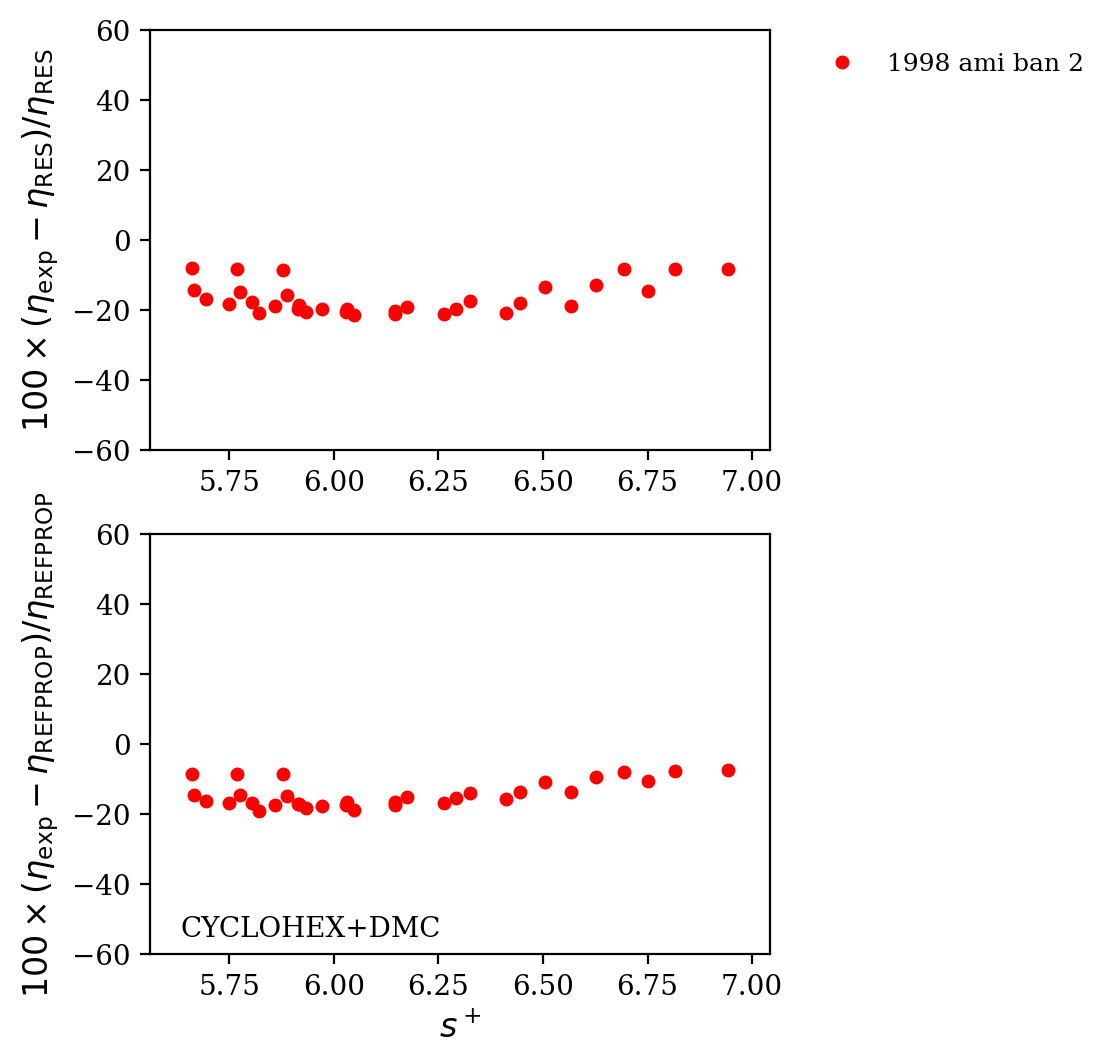

Supplement: Supplementary file 1 — je4c00451_si_001.zip [file je4c00451_si_001.zip › supporting_information/mix_dev_exp_res_ecs/CYCLOHEX+DMC.png]

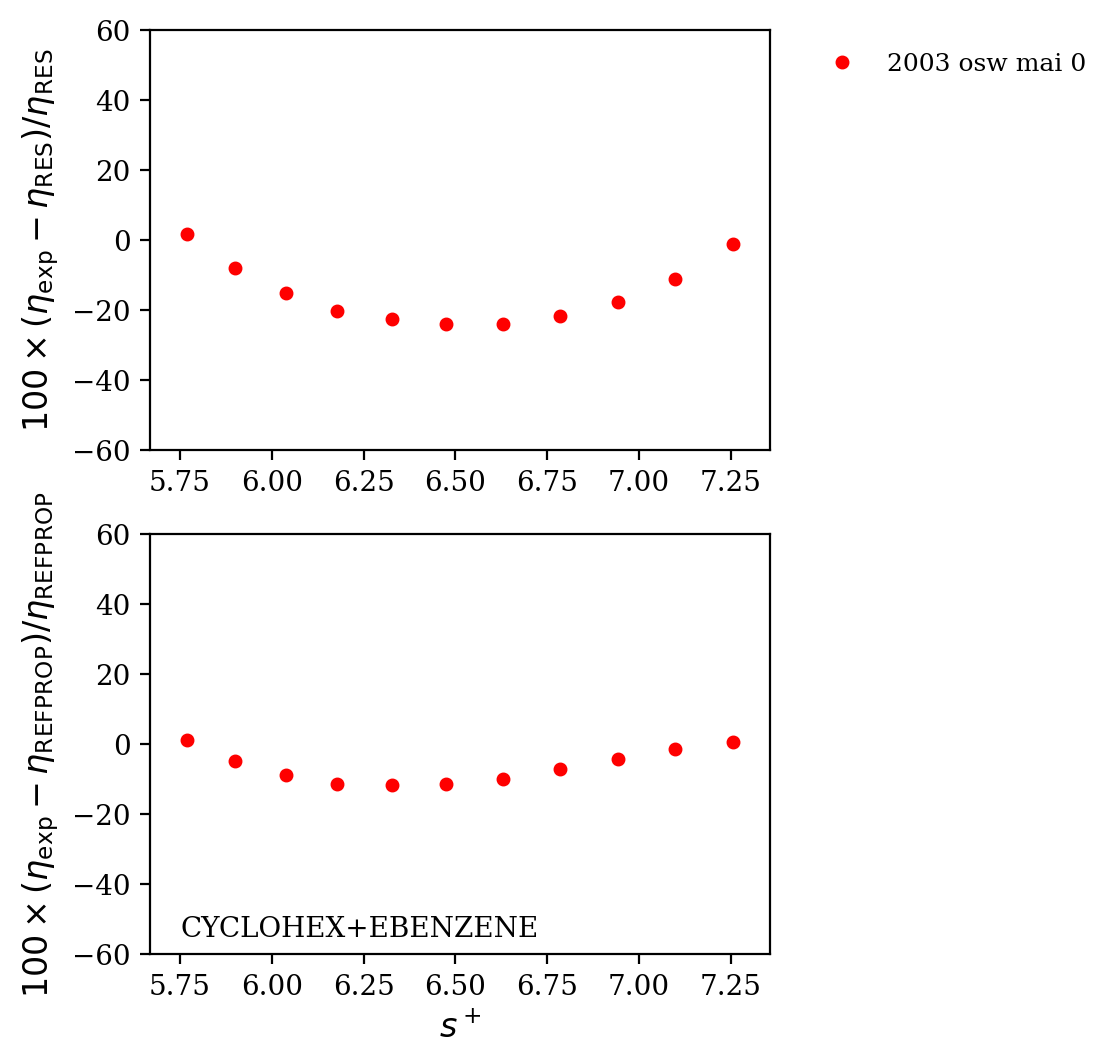

Supplement: Supplementary file 1 — je4c00451_si_001.zip [file je4c00451_si_001.zip › supporting_information/mix_dev_exp_res_ecs/CYCLOHEX+EBENZENE.png]

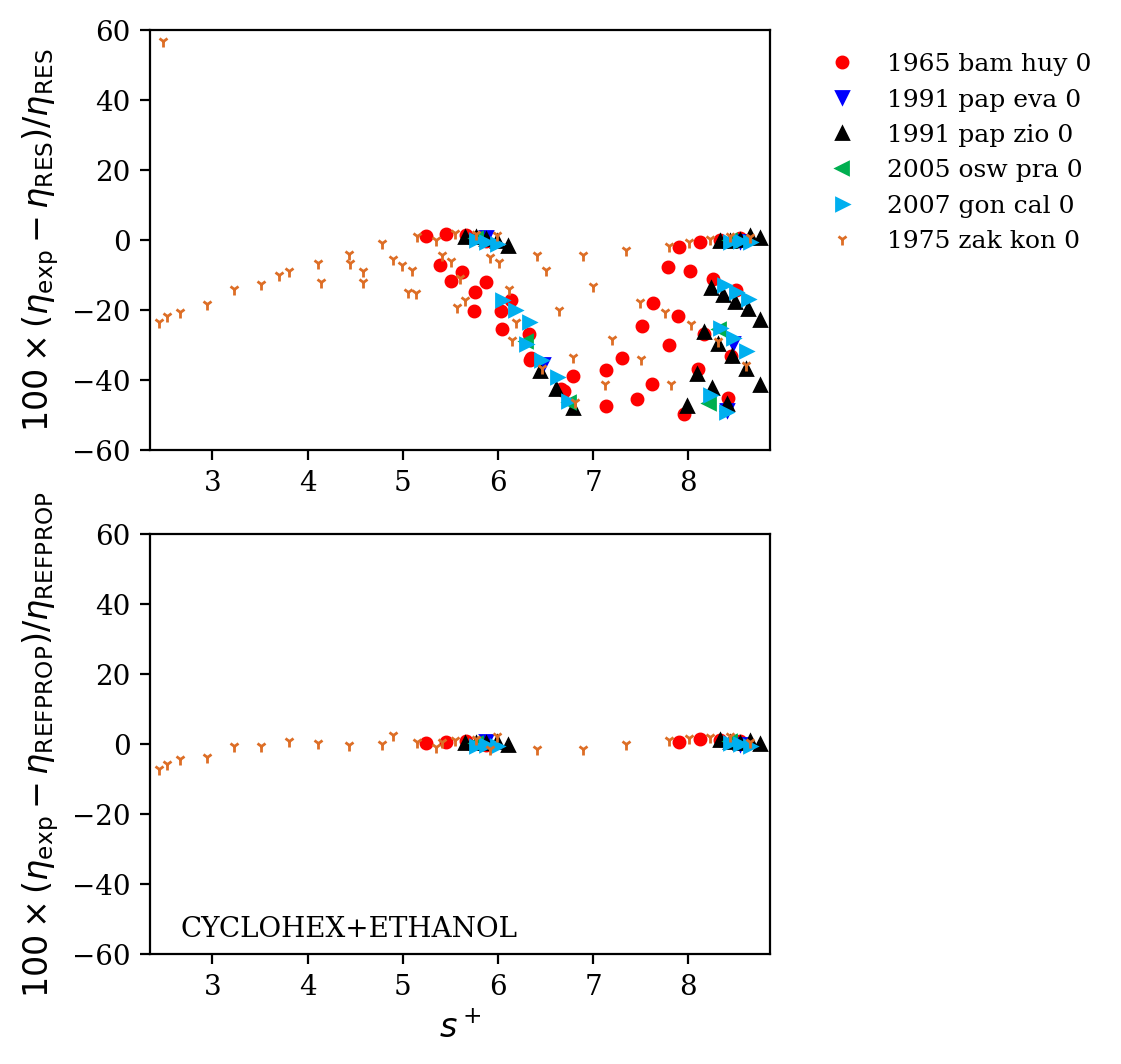

Supplement: Supplementary file 1 — je4c00451_si_001.zip [file je4c00451_si_001.zip › supporting_information/mix_dev_exp_res_ecs/CYCLOHEX+ETHANOL.png]

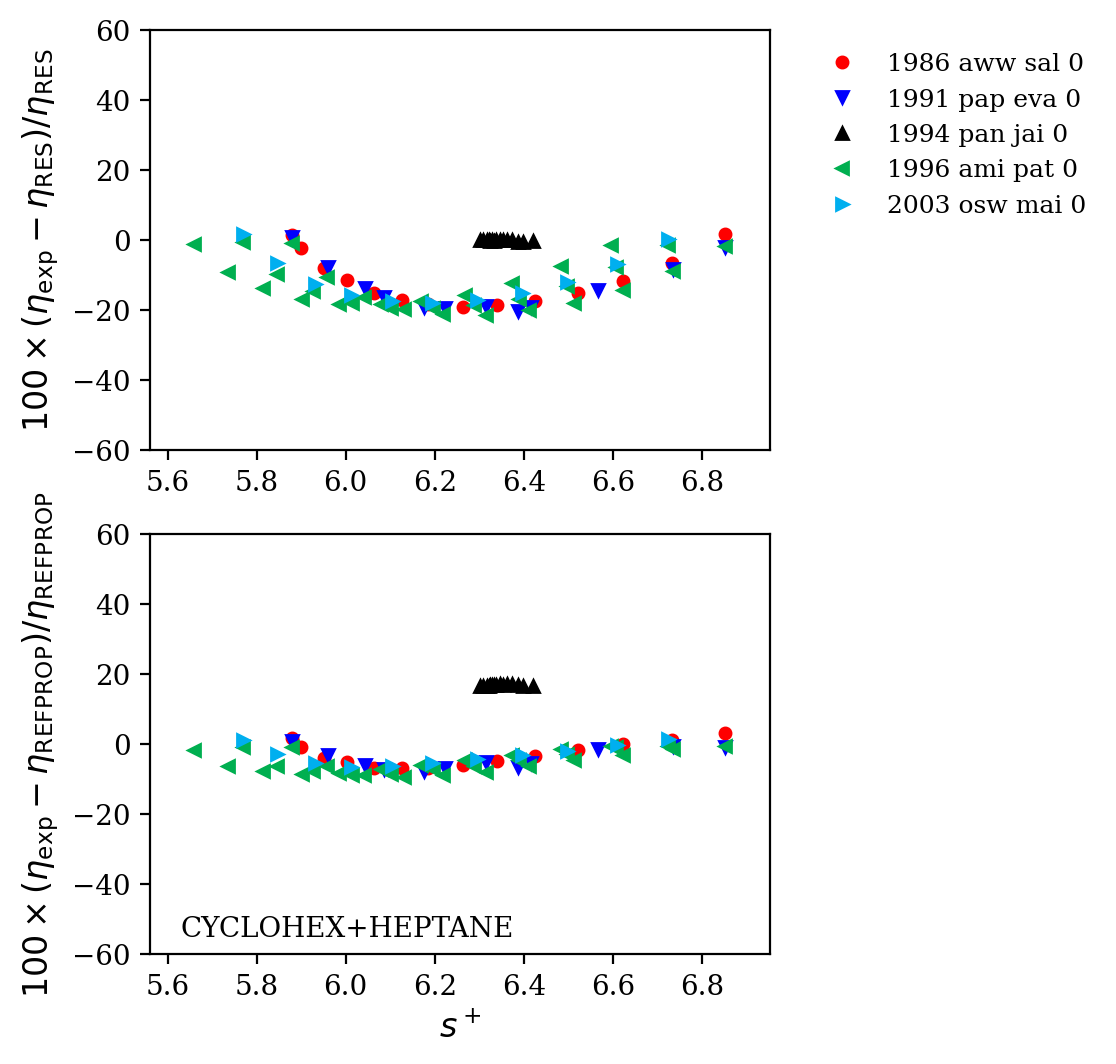

Supplement: Supplementary file 1 — je4c00451_si_001.zip [file je4c00451_si_001.zip › supporting_information/mix_dev_exp_res_ecs/CYCLOHEX+HEPTANE.png]

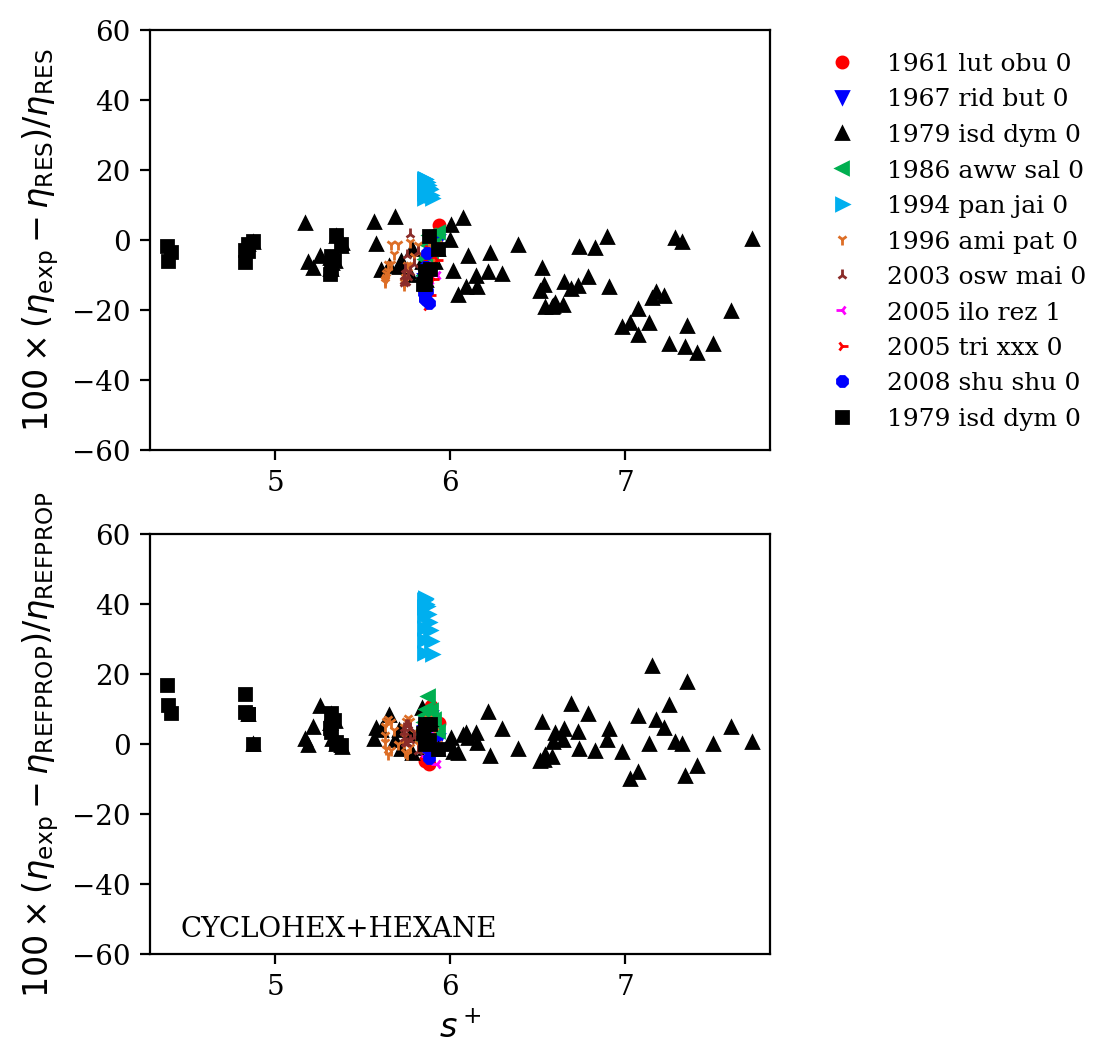

Supplement: Supplementary file 1 — je4c00451_si_001.zip [file je4c00451_si_001.zip › supporting_information/mix_dev_exp_res_ecs/CYCLOHEX+HEXANE.png]

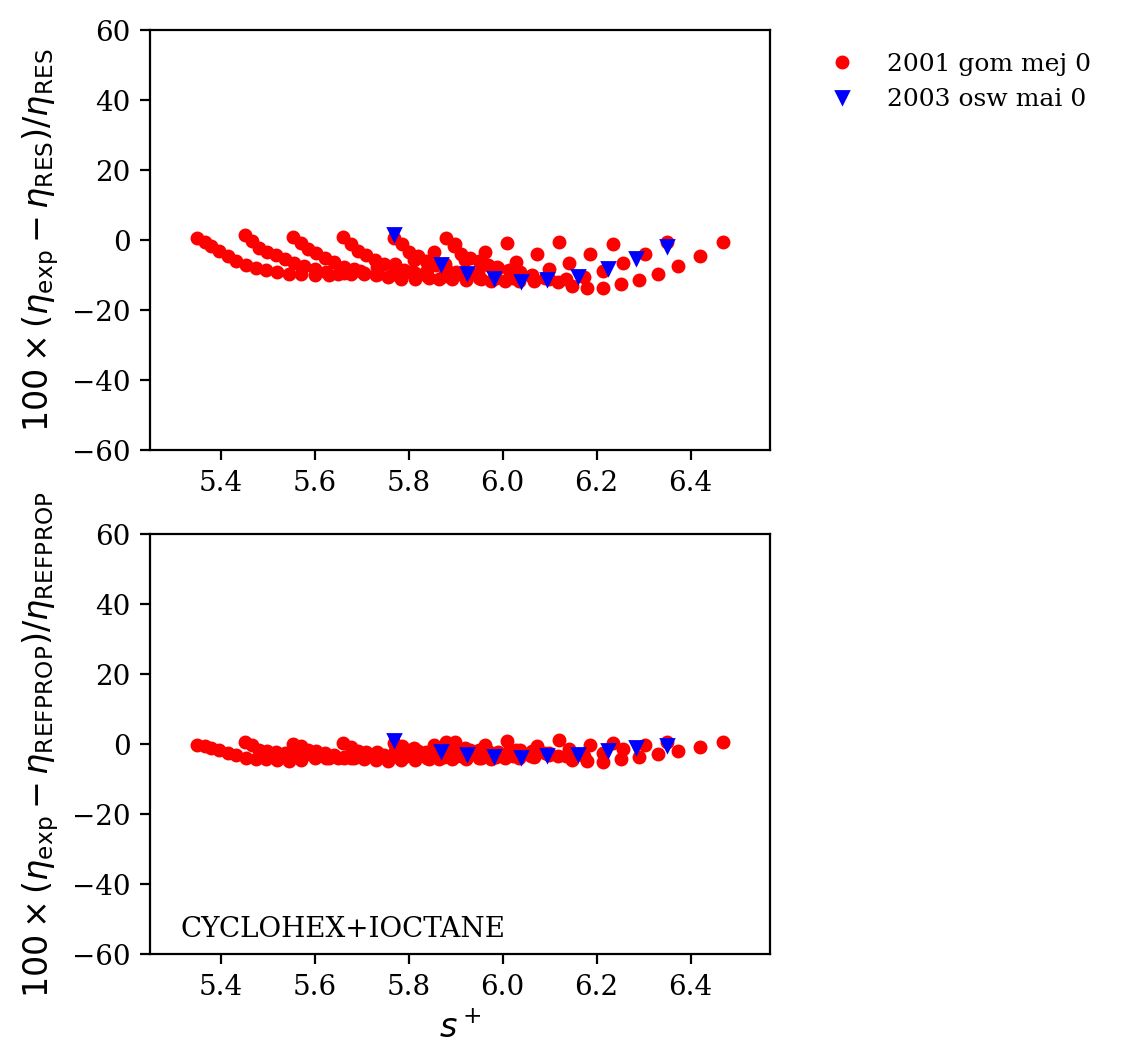

Supplement: Supplementary file 1 — je4c00451_si_001.zip [file je4c00451_si_001.zip › supporting_information/mix_dev_exp_res_ecs/CYCLOHEX+IOCTANE.png]

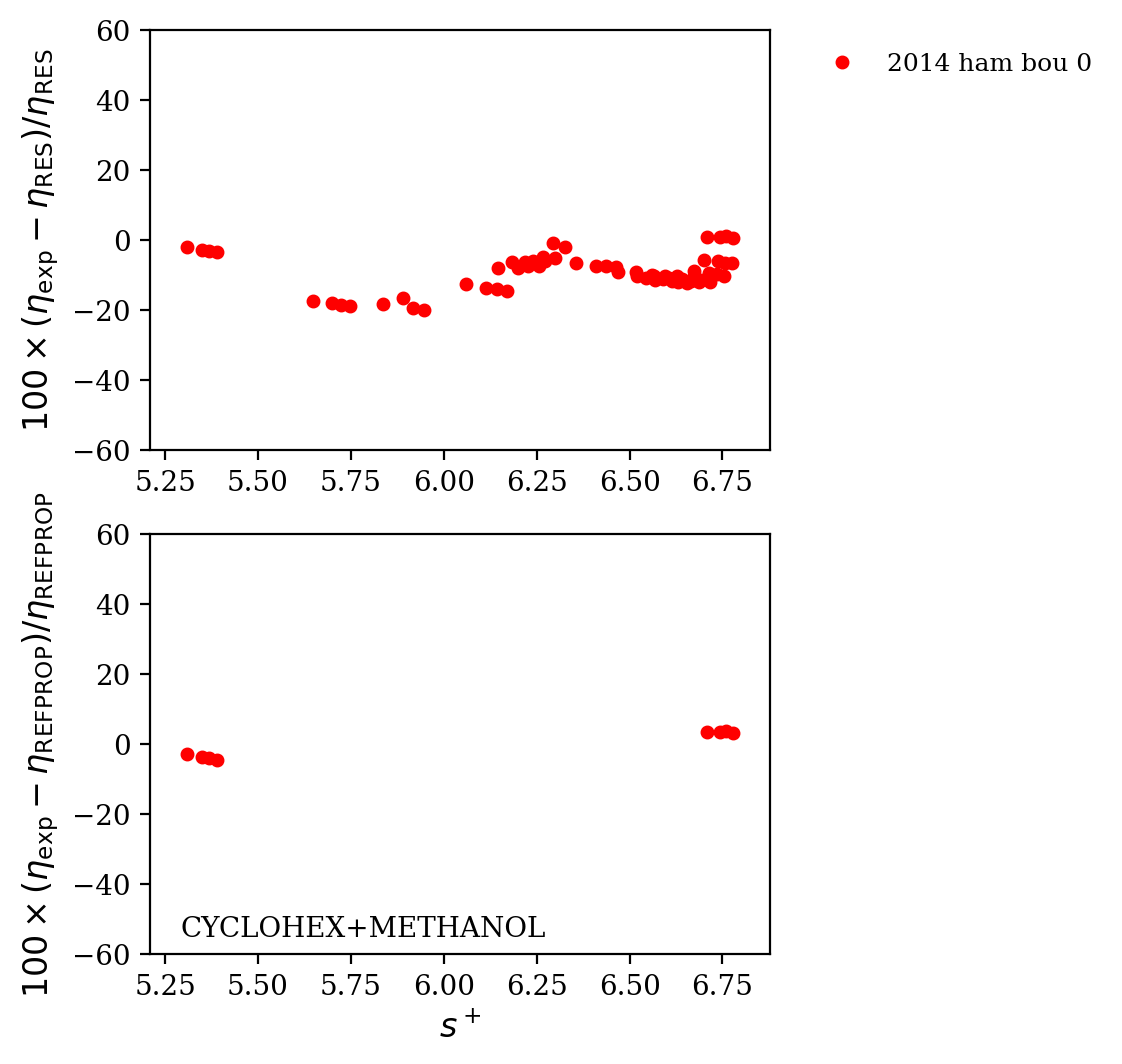

Supplement: Supplementary file 1 — je4c00451_si_001.zip [file je4c00451_si_001.zip › supporting_information/mix_dev_exp_res_ecs/CYCLOHEX+METHANOL.png]

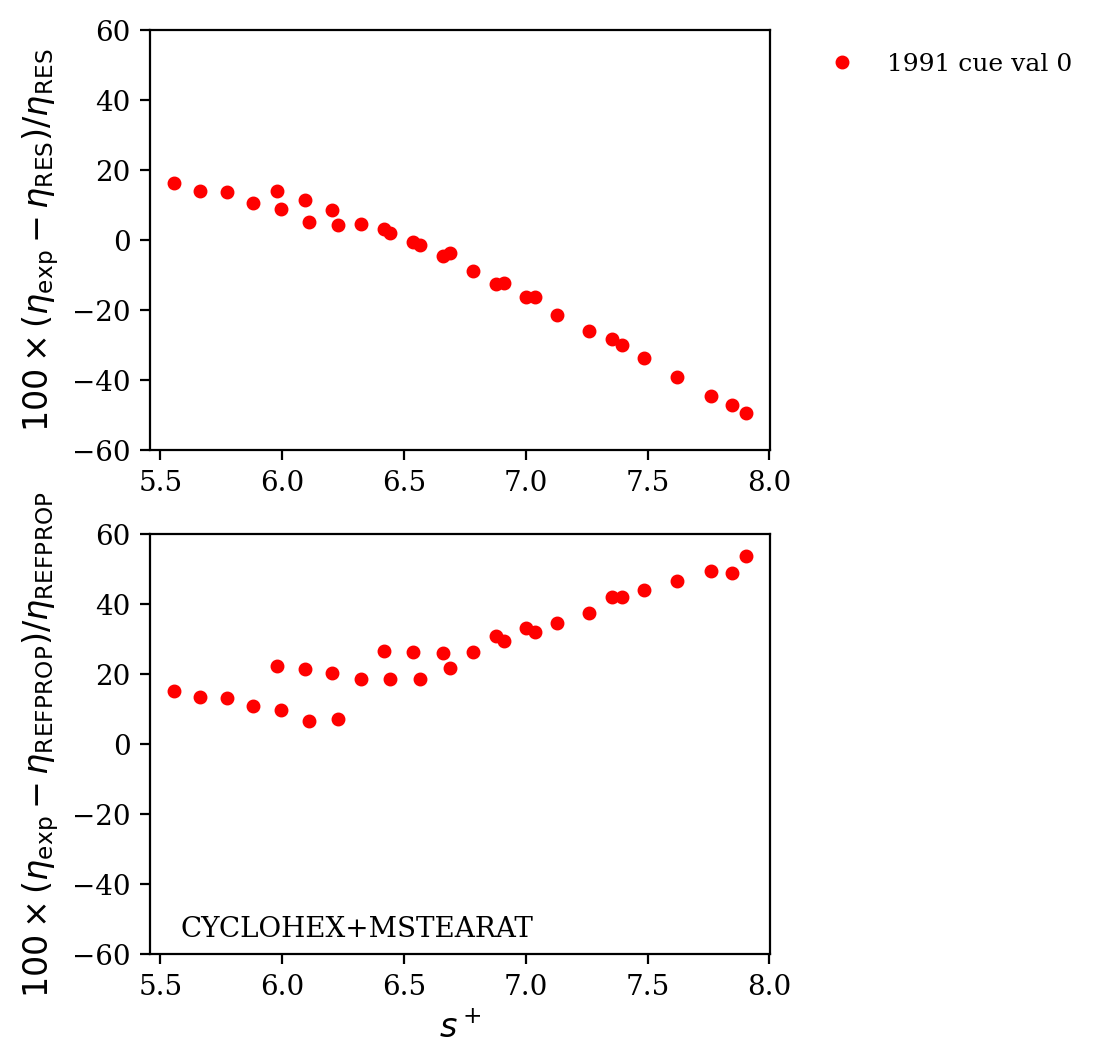

Supplement: Supplementary file 1 — je4c00451_si_001.zip [file je4c00451_si_001.zip › supporting_information/mix_dev_exp_res_ecs/CYCLOHEX+MSTEARAT.png]

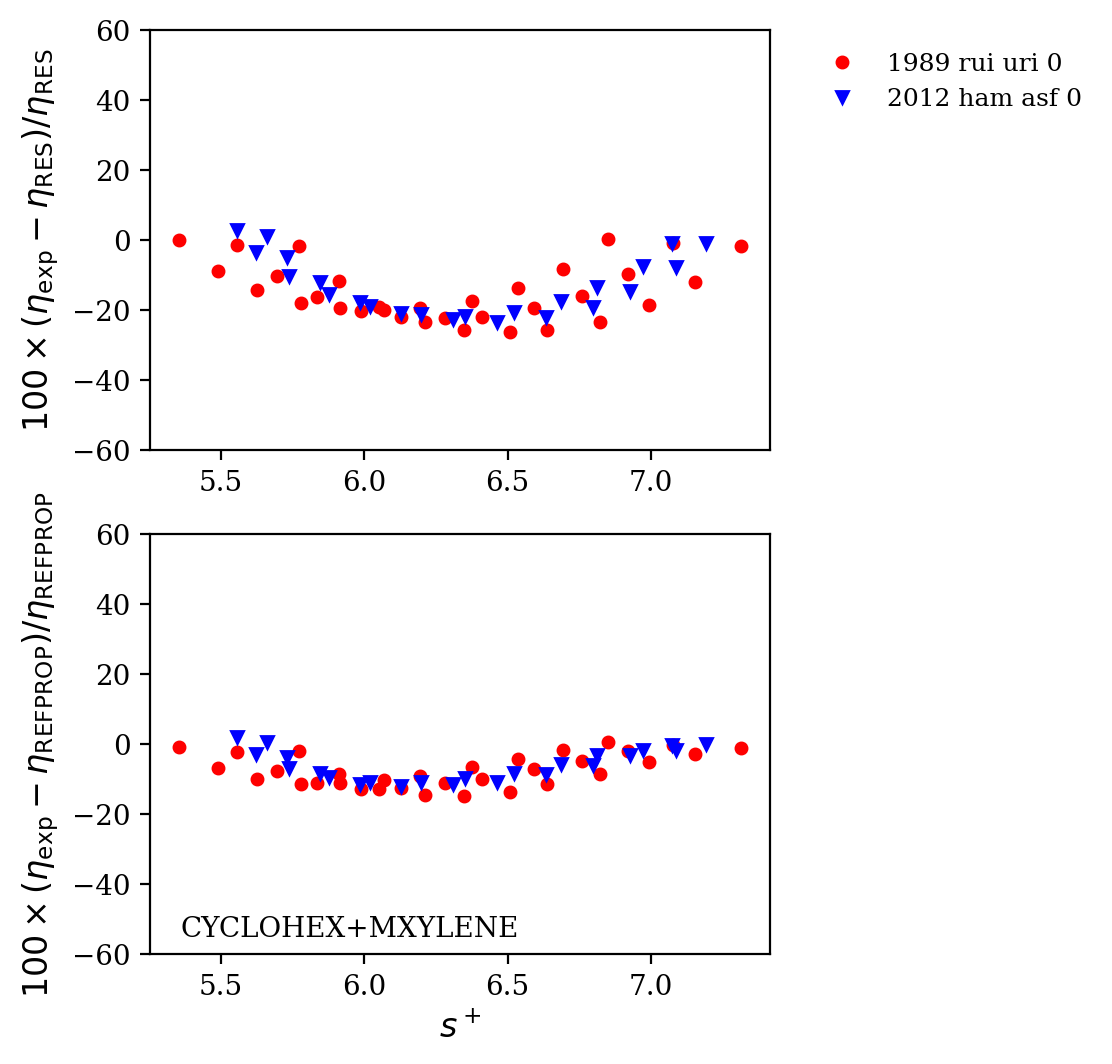

Supplement: Supplementary file 1 — je4c00451_si_001.zip [file je4c00451_si_001.zip › supporting_information/mix_dev_exp_res_ecs/CYCLOHEX+MXYLENE.png]

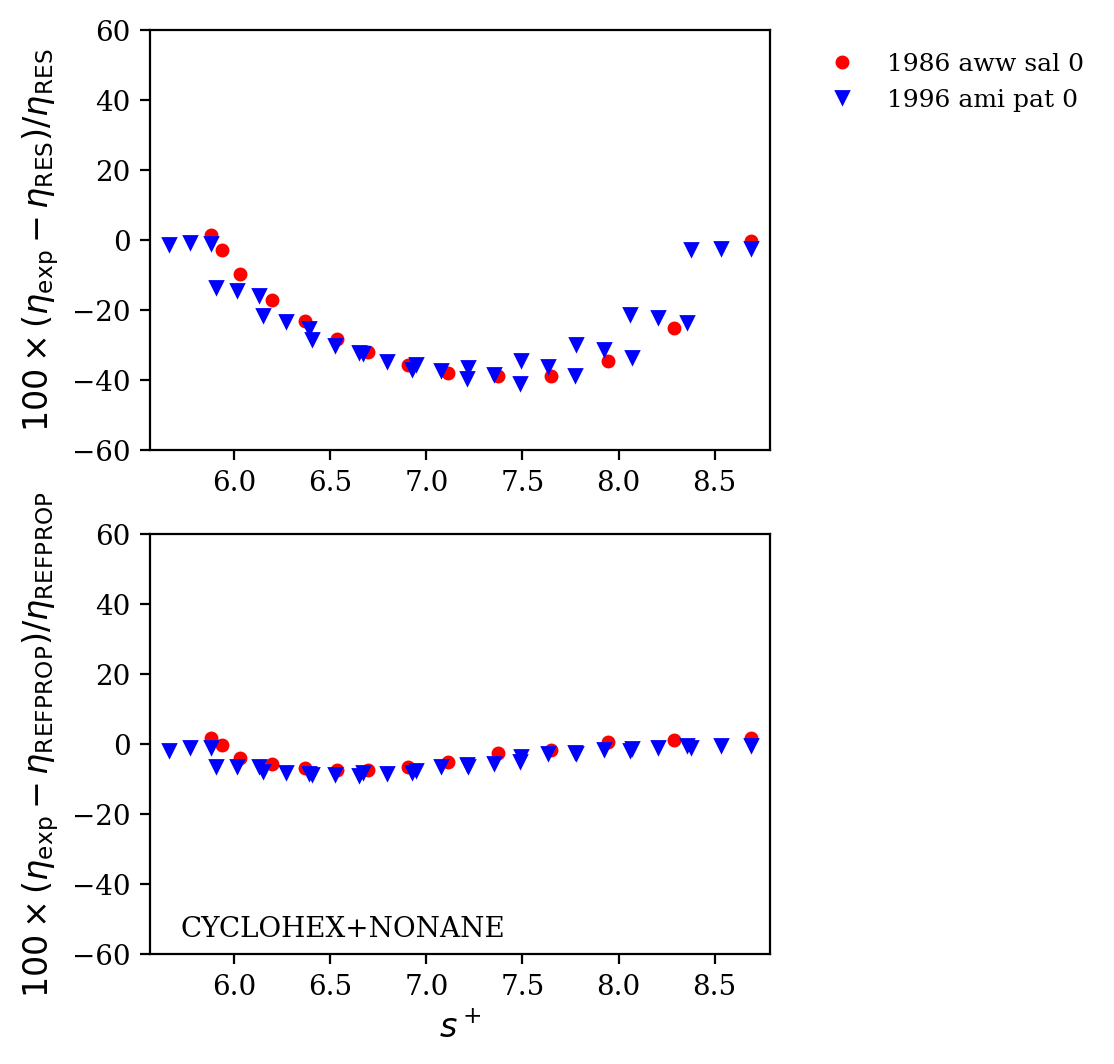

Supplement: Supplementary file 1 — je4c00451_si_001.zip [file je4c00451_si_001.zip › supporting_information/mix_dev_exp_res_ecs/CYCLOHEX+NONANE.png]

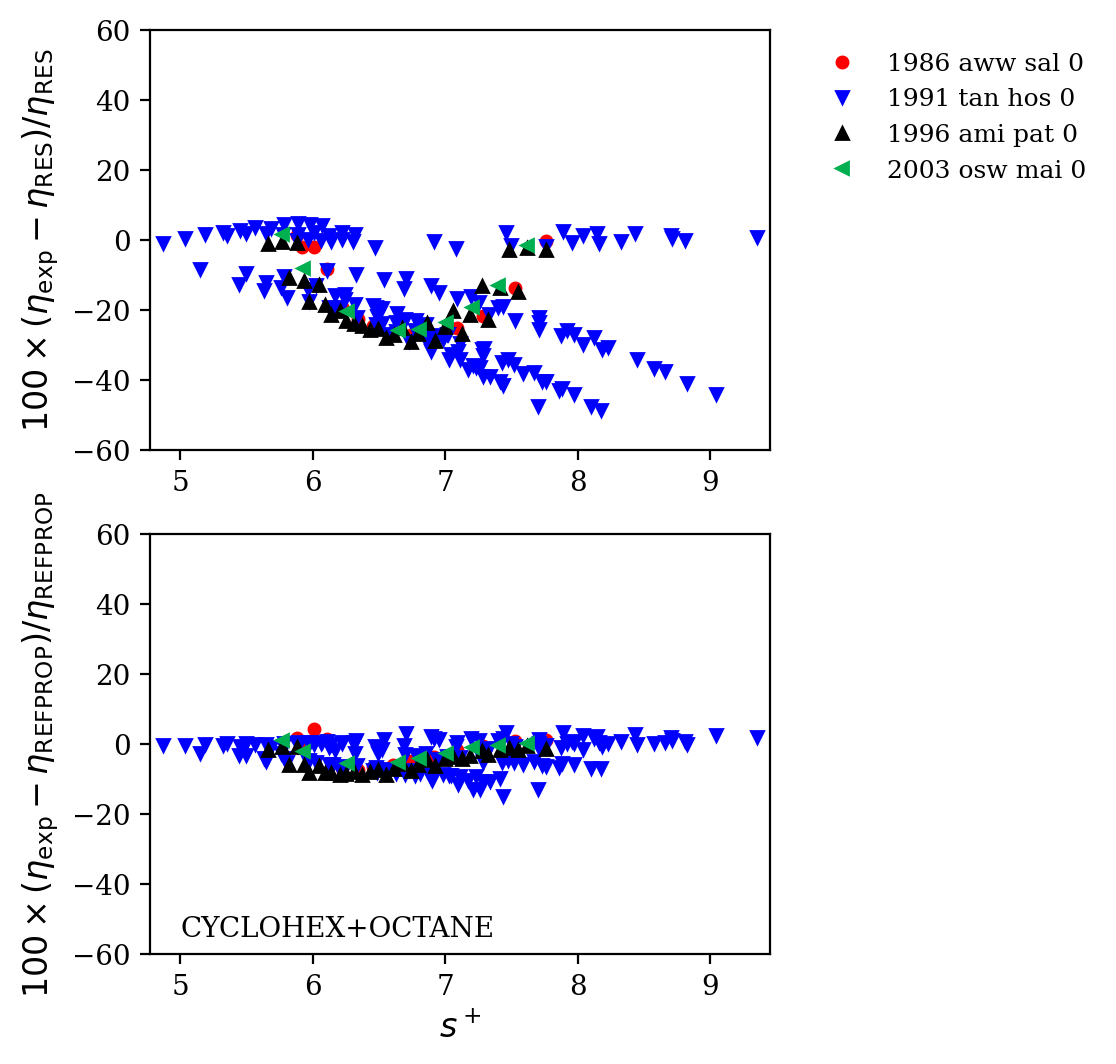

Supplement: Supplementary file 1 — je4c00451_si_001.zip [file je4c00451_si_001.zip › supporting_information/mix_dev_exp_res_ecs/CYCLOHEX+OCTANE.png]

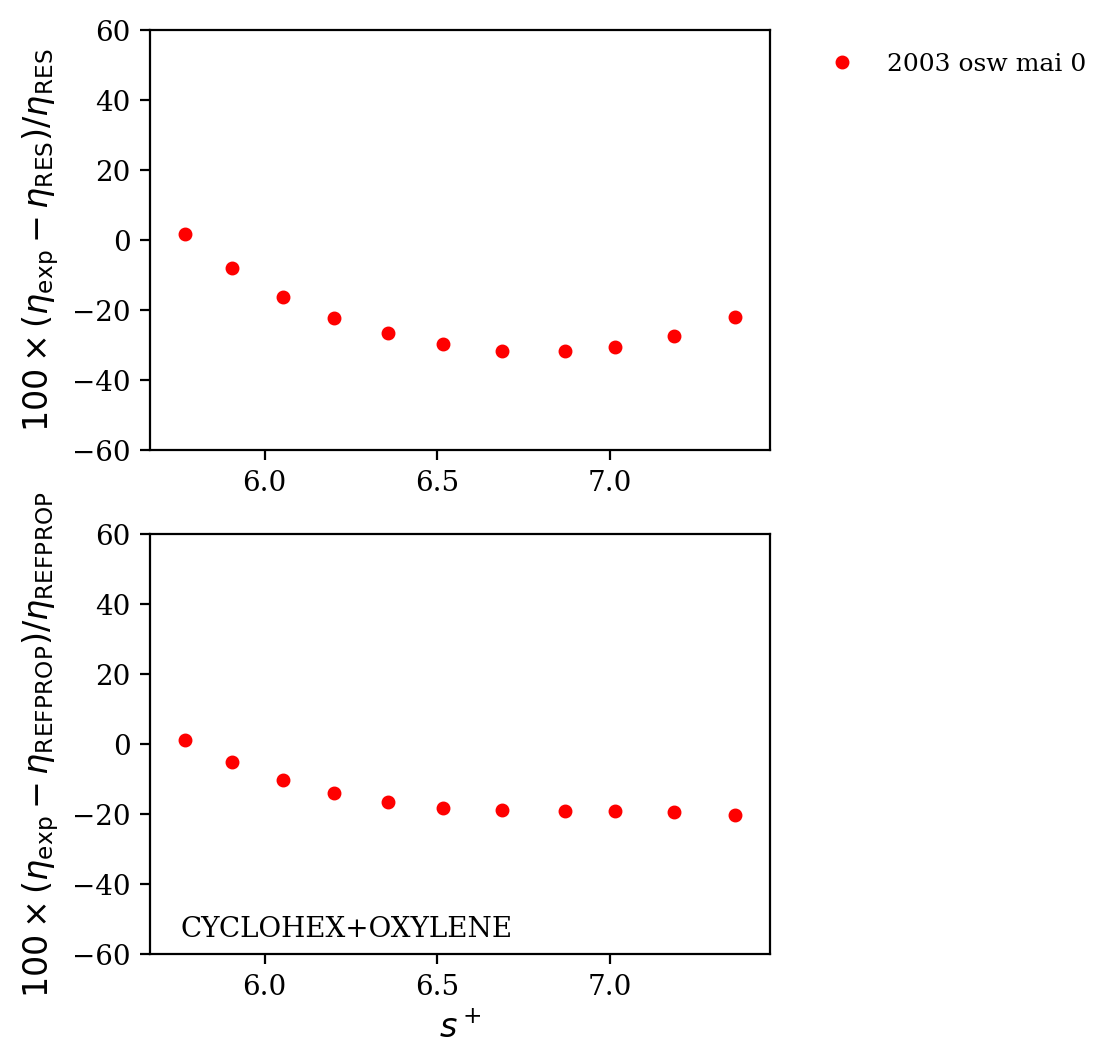

Supplement: Supplementary file 1 — je4c00451_si_001.zip [file je4c00451_si_001.zip › supporting_information/mix_dev_exp_res_ecs/CYCLOHEX+OXYLENE.png]

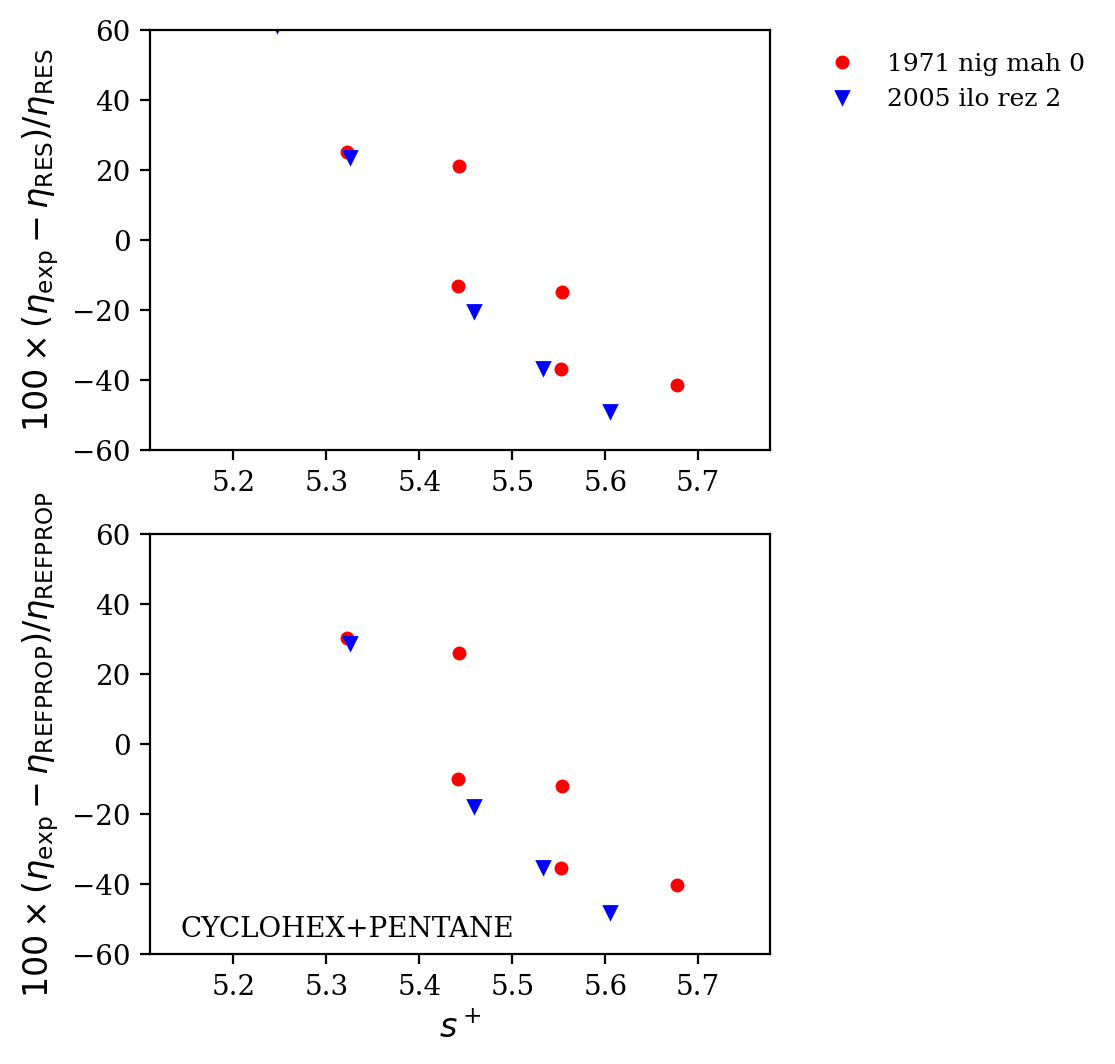

Supplement: Supplementary file 1 — je4c00451_si_001.zip [file je4c00451_si_001.zip › supporting_information/mix_dev_exp_res_ecs/CYCLOHEX+PENTANE.png]

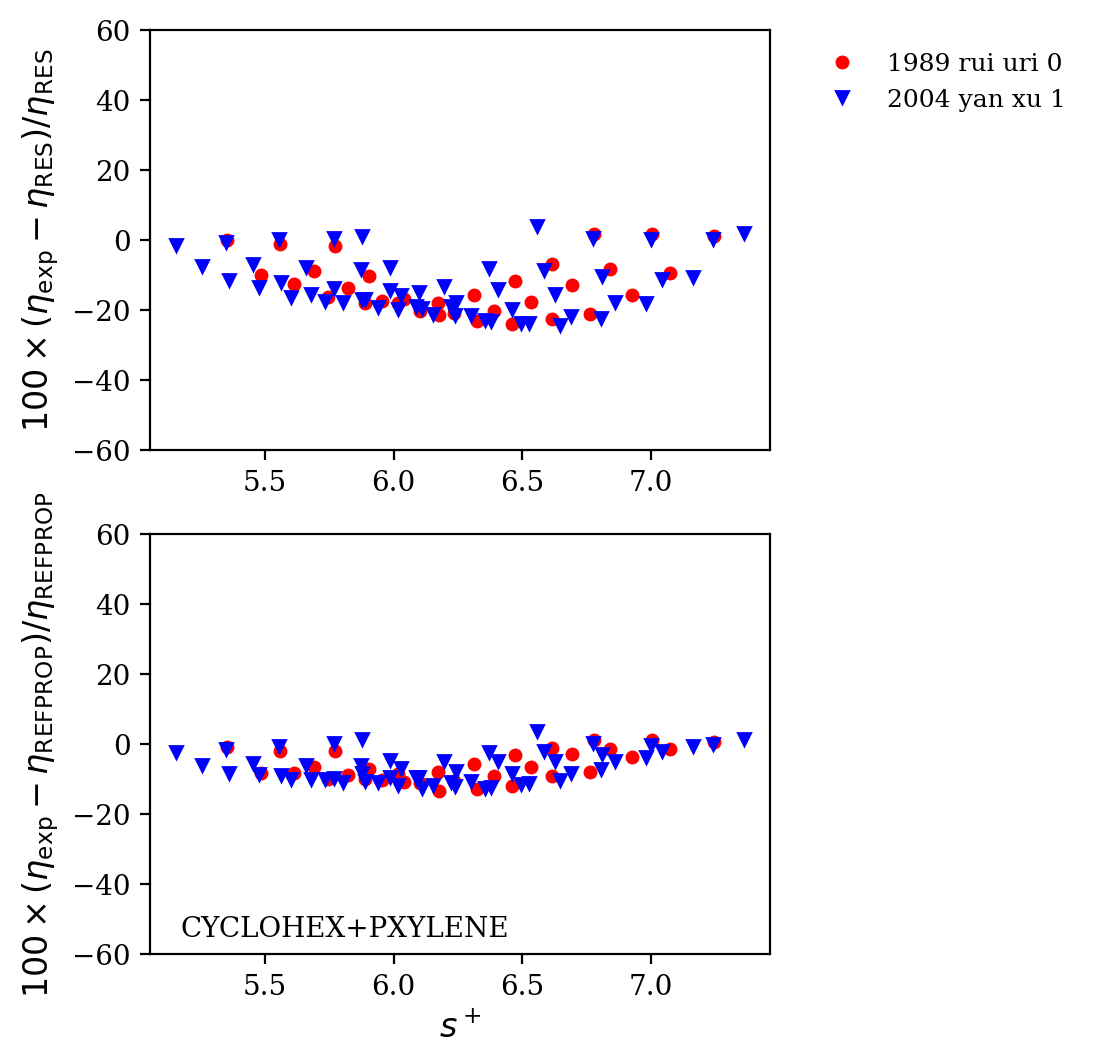

Supplement: Supplementary file 1 — je4c00451_si_001.zip [file je4c00451_si_001.zip › supporting_information/mix_dev_exp_res_ecs/CYCLOHEX+PXYLENE.png]

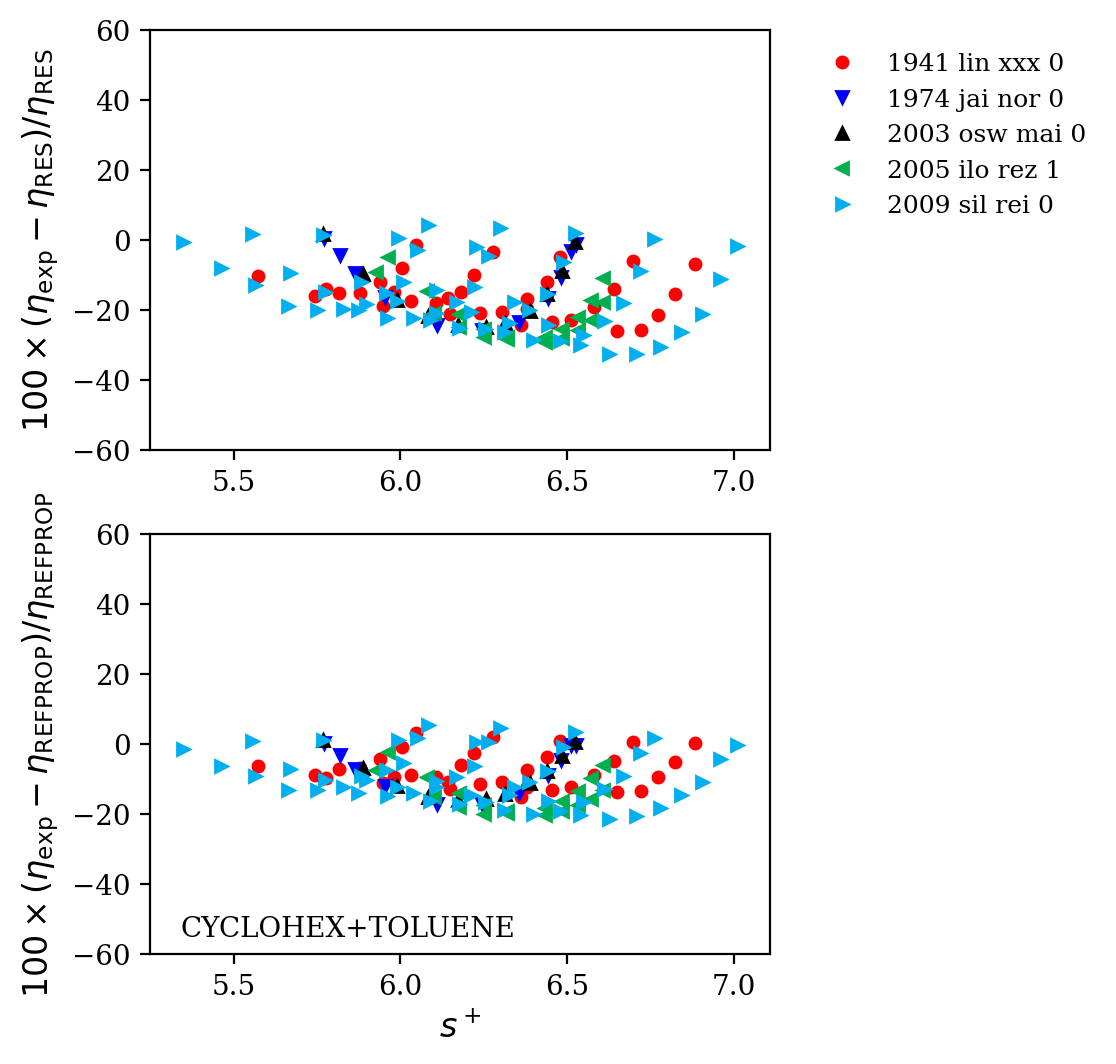

Supplement: Supplementary file 1 — je4c00451_si_001.zip [file je4c00451_si_001.zip › supporting_information/mix_dev_exp_res_ecs/CYCLOHEX+TOLUENE.png]

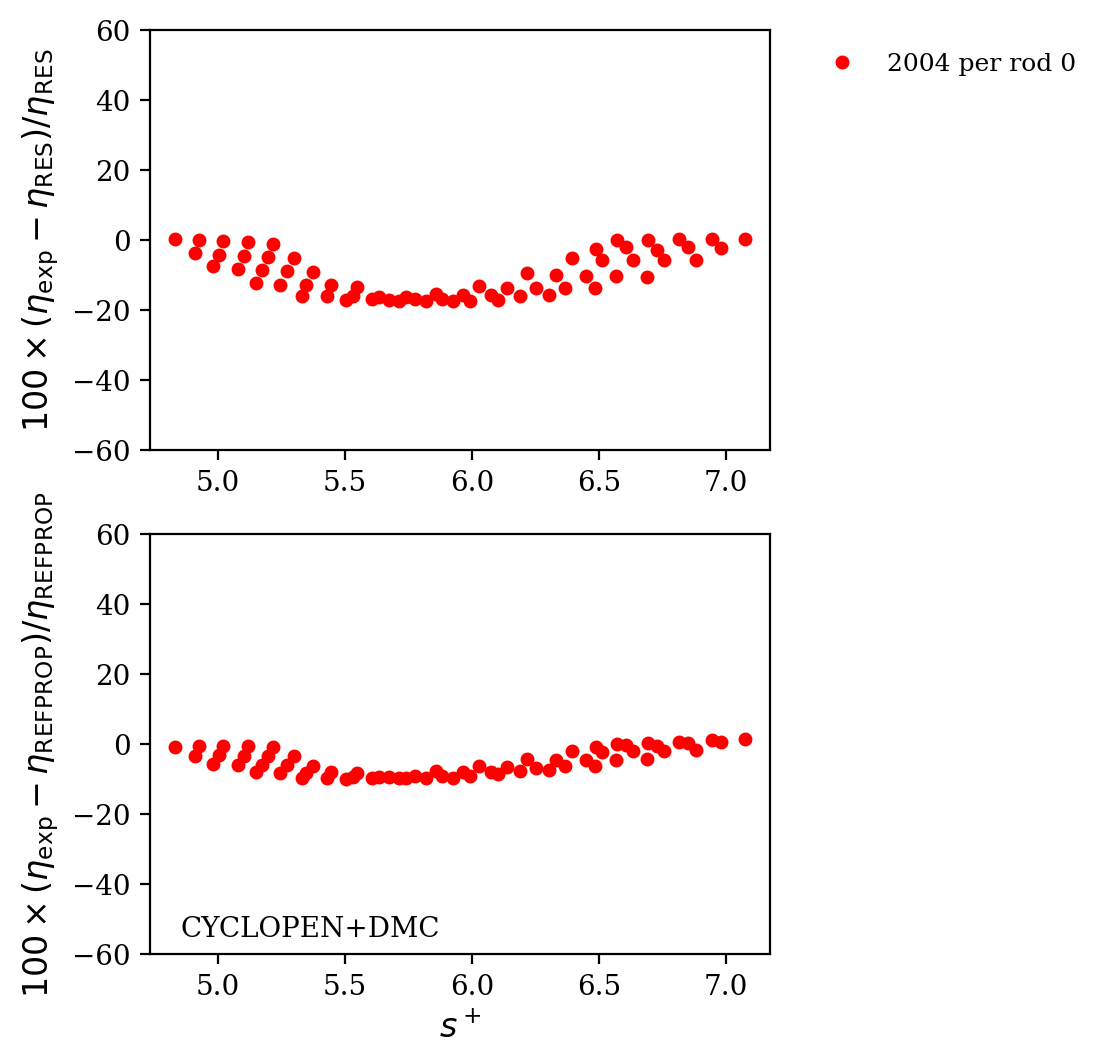

Supplement: Supplementary file 1 — je4c00451_si_001.zip [file je4c00451_si_001.zip › supporting_information/mix_dev_exp_res_ecs/CYCLOPEN+DMC.png]

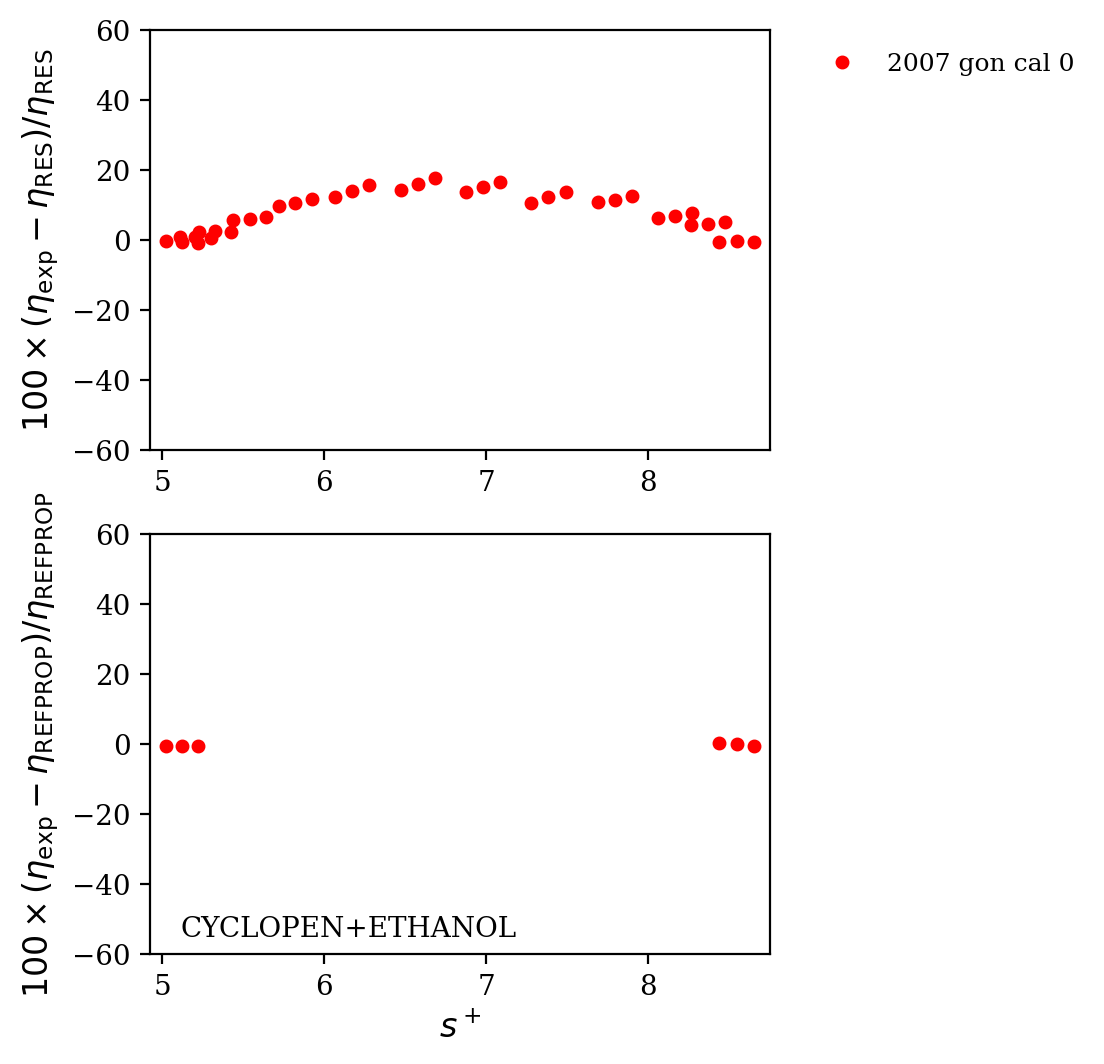

Supplement: Supplementary file 1 — je4c00451_si_001.zip [file je4c00451_si_001.zip › supporting_information/mix_dev_exp_res_ecs/CYCLOPEN+ETHANOL.png]

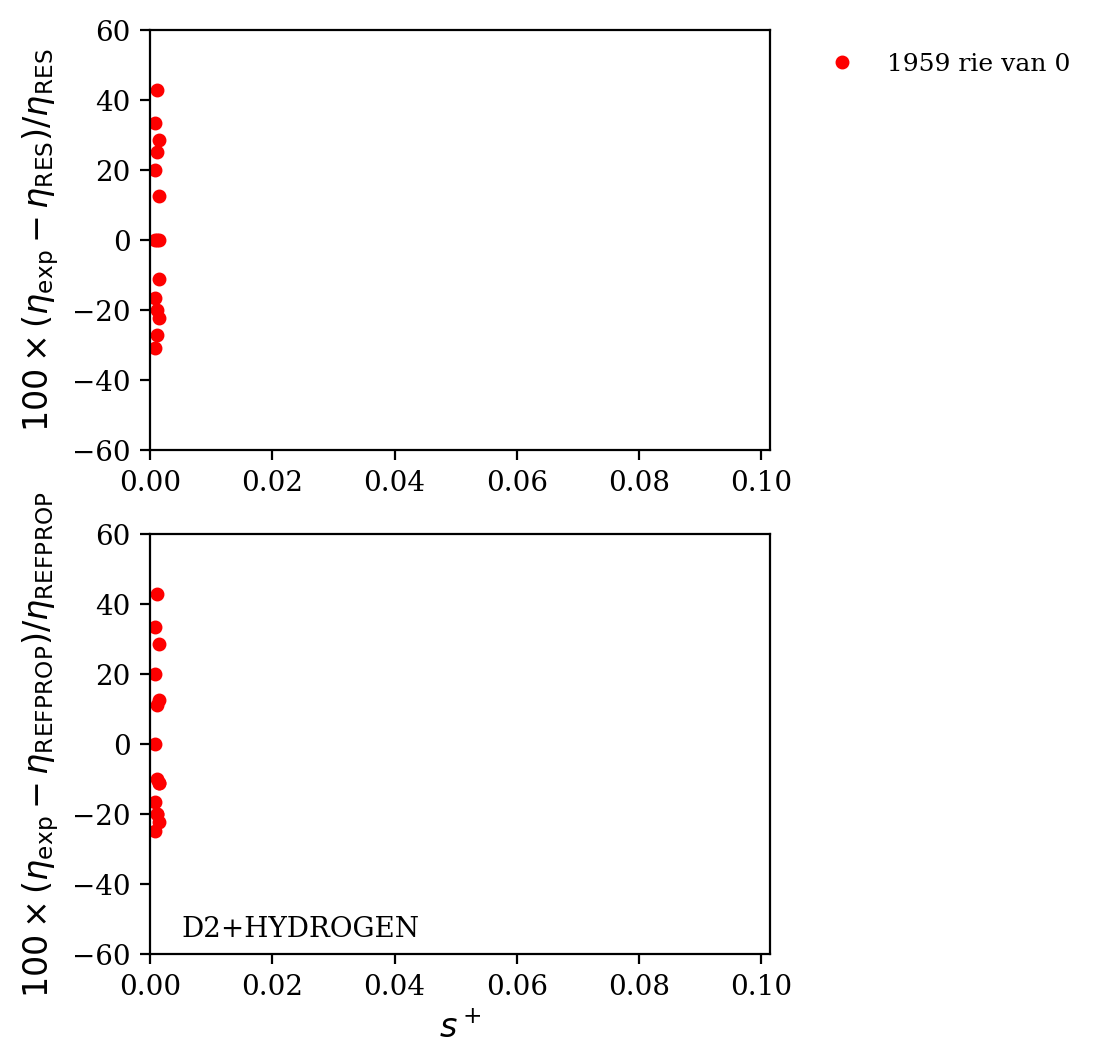

Supplement: Supplementary file 1 — je4c00451_si_001.zip [file je4c00451_si_001.zip › supporting_information/mix_dev_exp_res_ecs/D2+HYDROGEN.png]

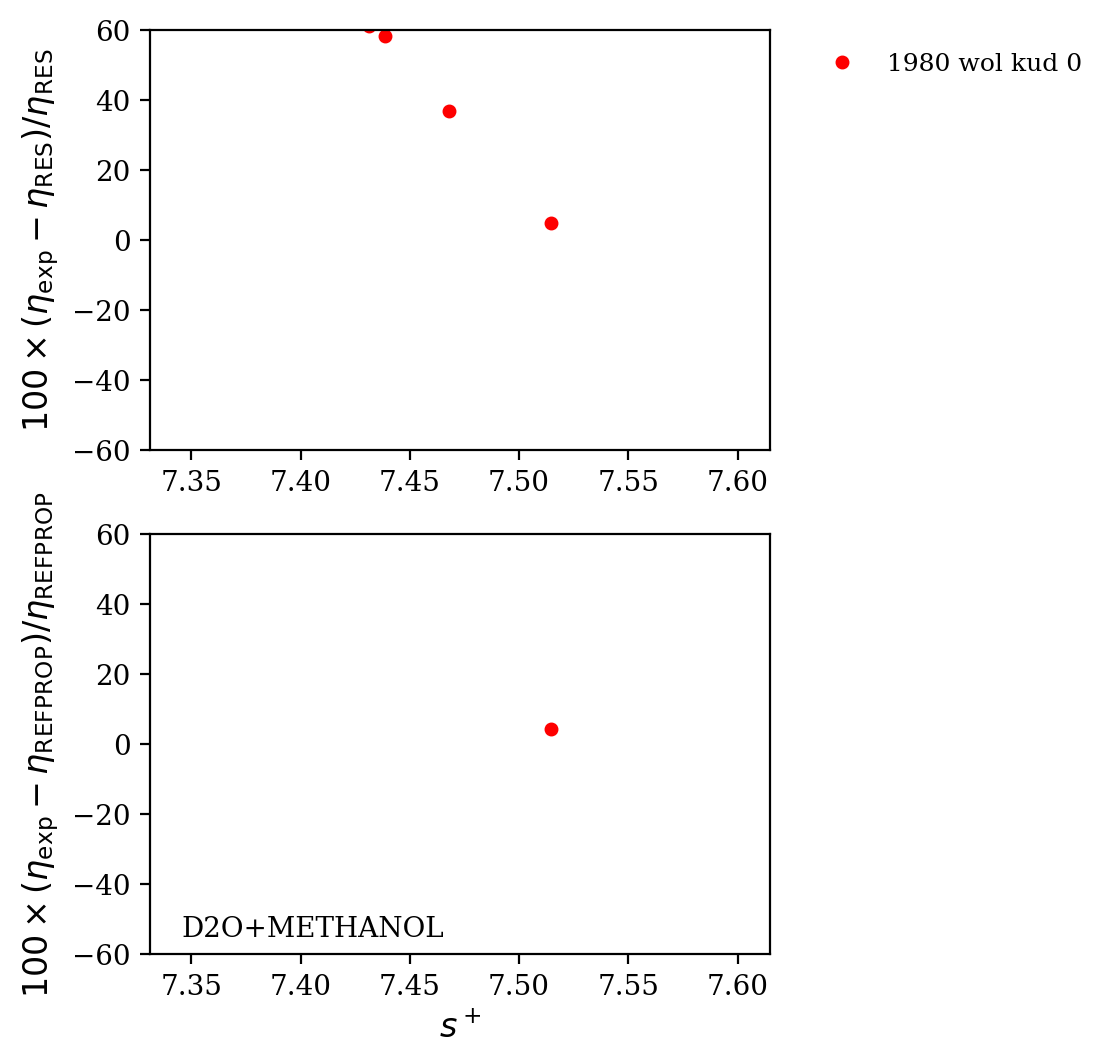

Supplement: Supplementary file 1 — je4c00451_si_001.zip [file je4c00451_si_001.zip › supporting_information/mix_dev_exp_res_ecs/D2O+METHANOL.png]

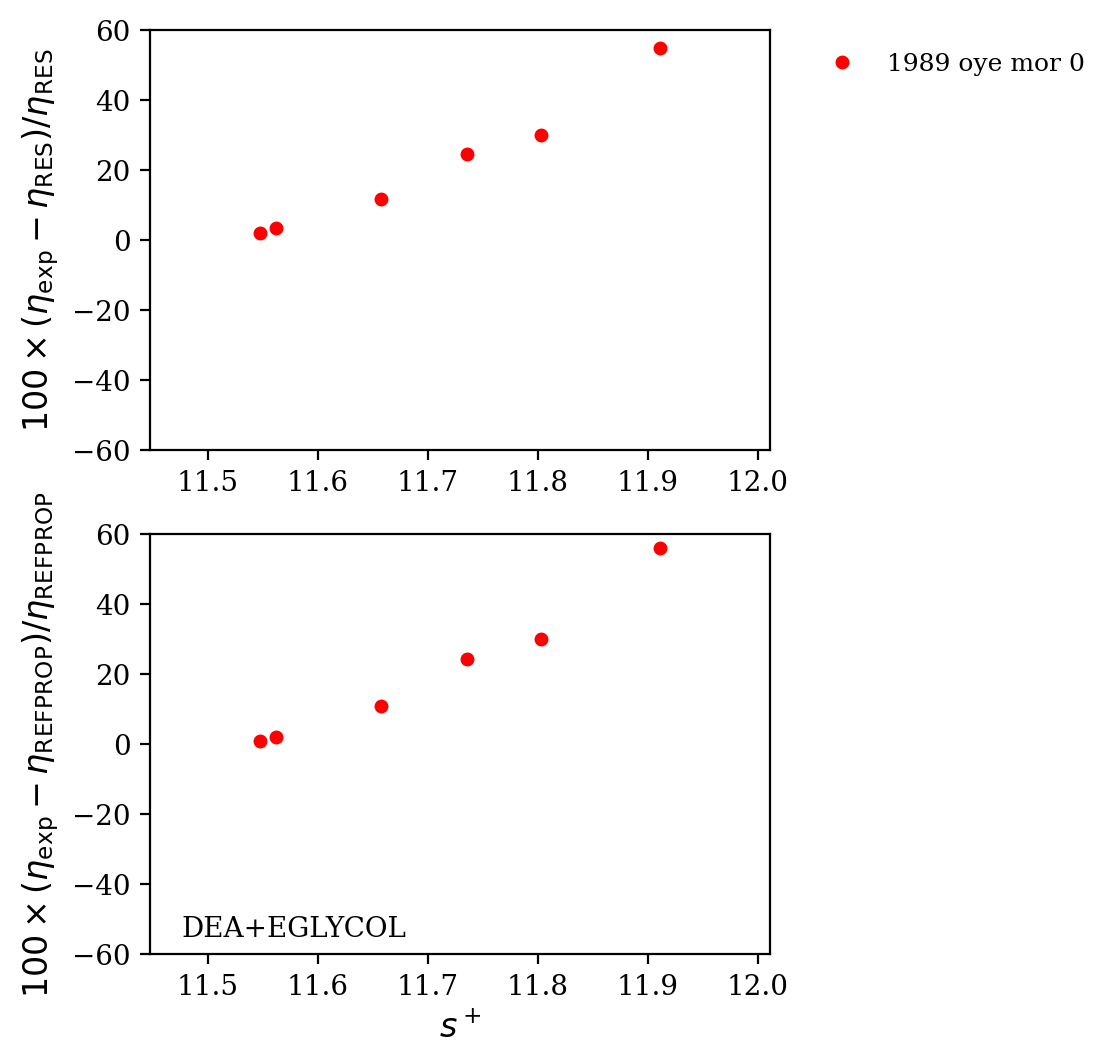

Supplement: Supplementary file 1 — je4c00451_si_001.zip [file je4c00451_si_001.zip › supporting_information/mix_dev_exp_res_ecs/DEA+EGLYCOL.png]

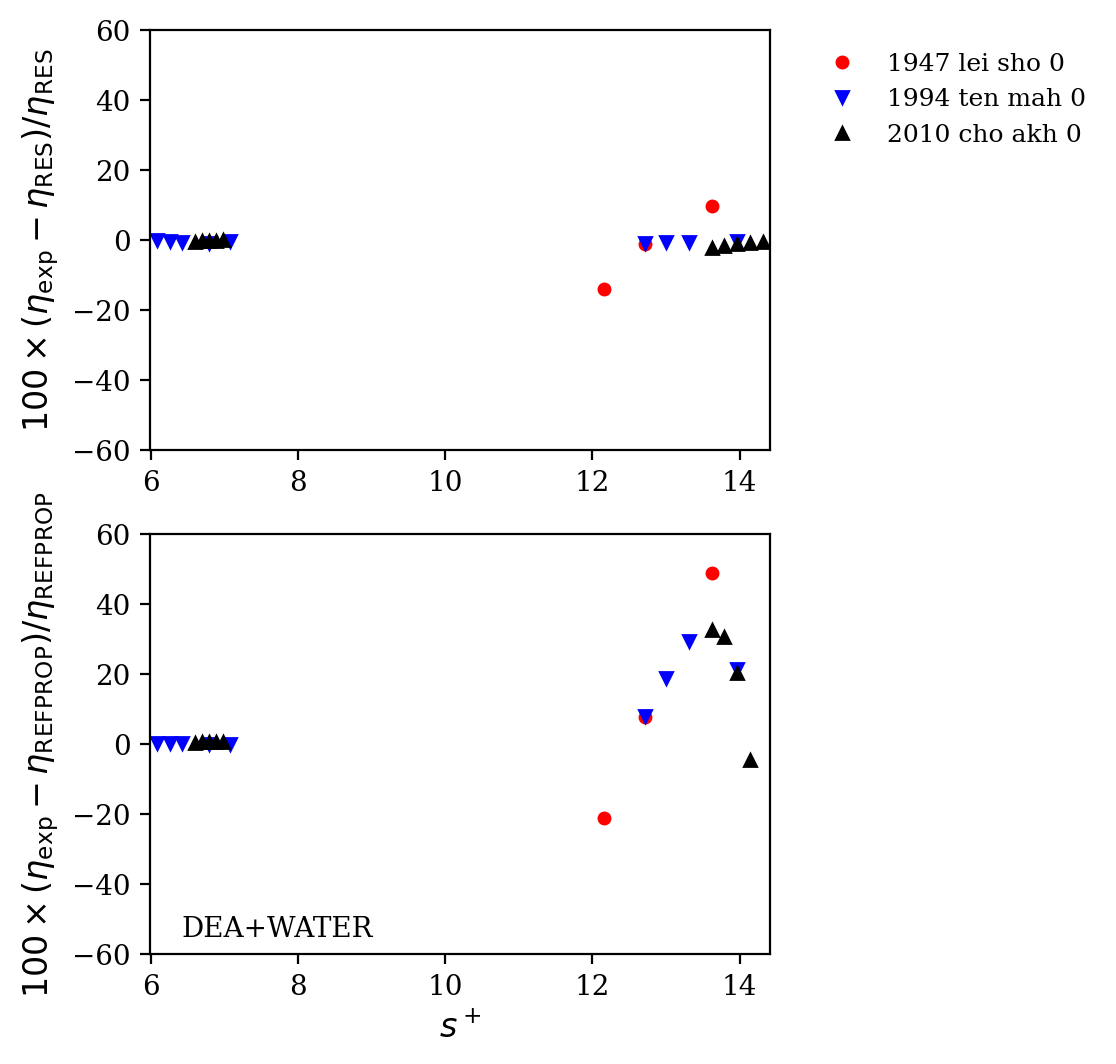

Supplement: Supplementary file 1 — je4c00451_si_001.zip [file je4c00451_si_001.zip › supporting_information/mix_dev_exp_res_ecs/DEA+WATER.png]

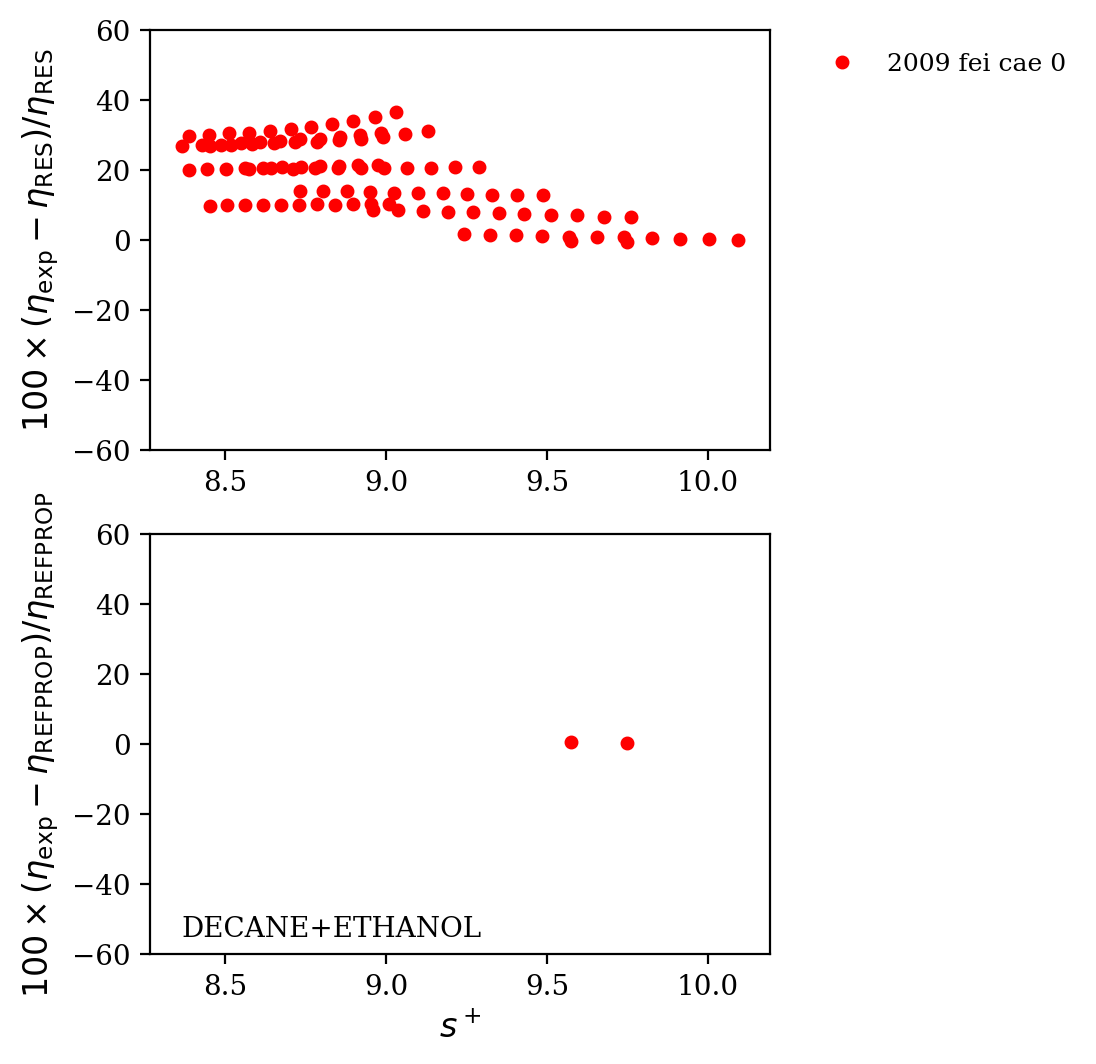

Supplement: Supplementary file 1 — je4c00451_si_001.zip [file je4c00451_si_001.zip › supporting_information/mix_dev_exp_res_ecs/DECANE+ETHANOL.png]

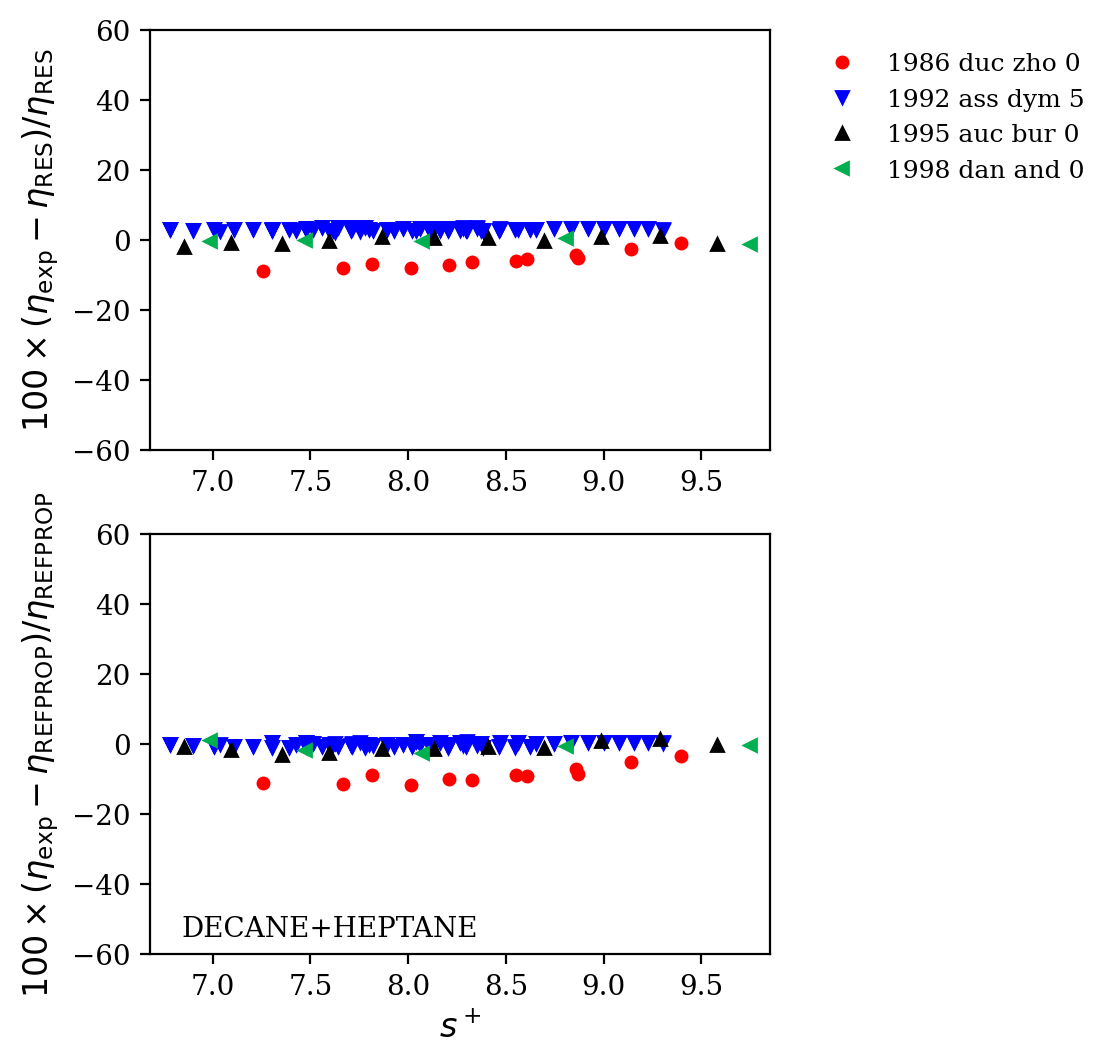

Supplement: Supplementary file 1 — je4c00451_si_001.zip [file je4c00451_si_001.zip › supporting_information/mix_dev_exp_res_ecs/DECANE+HEPTANE.png]

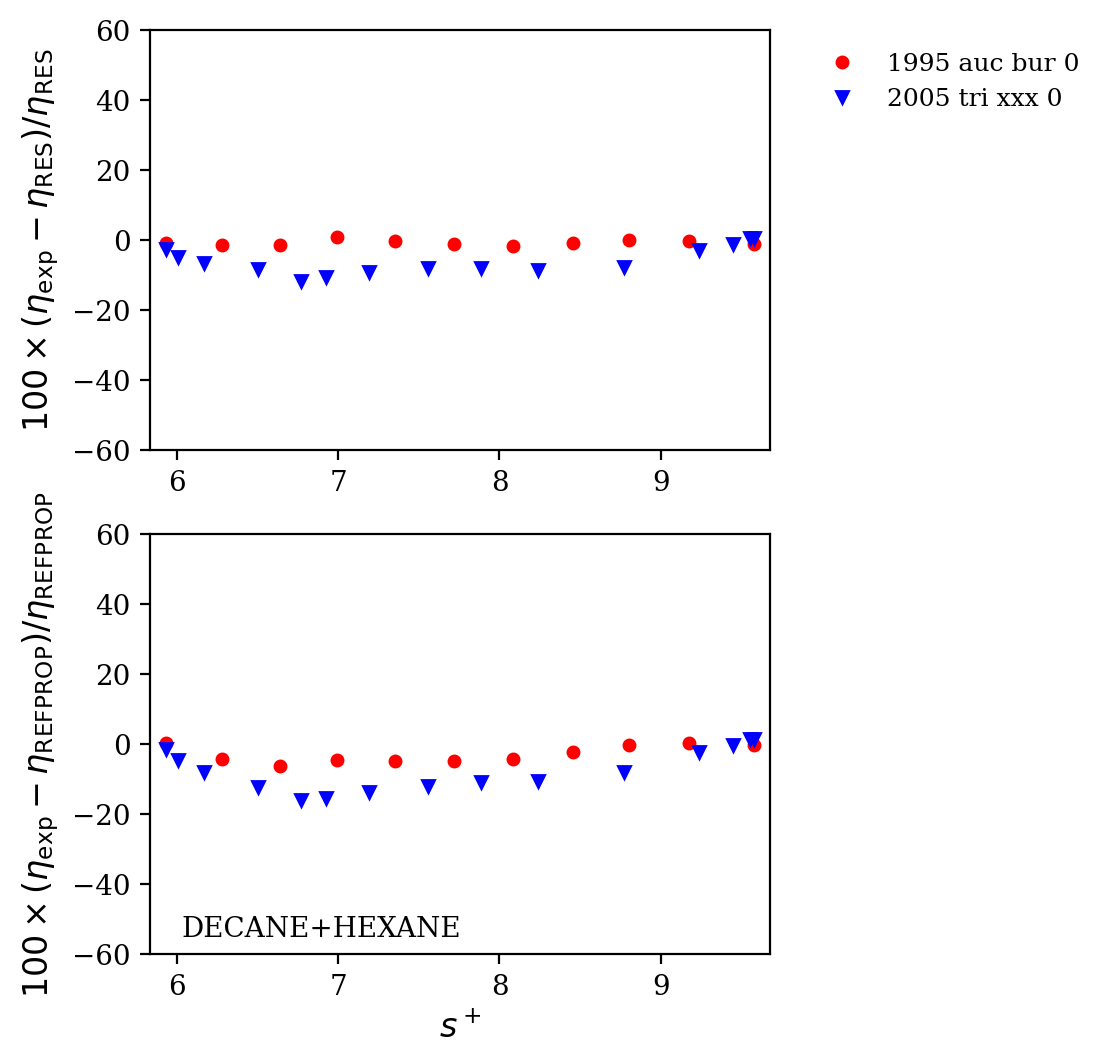

Supplement: Supplementary file 1 — je4c00451_si_001.zip [file je4c00451_si_001.zip › supporting_information/mix_dev_exp_res_ecs/DECANE+HEXANE.png]

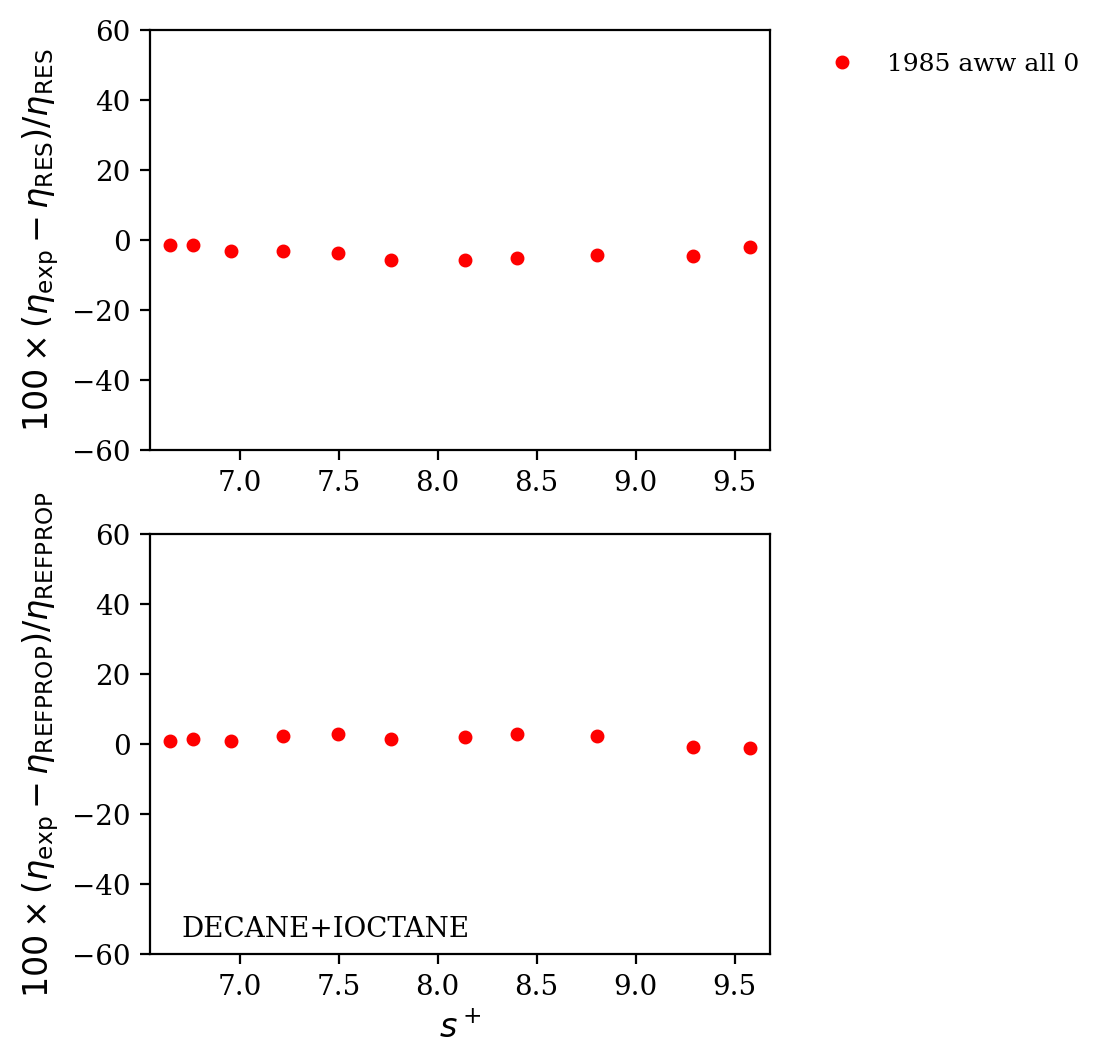

Supplement: Supplementary file 1 — je4c00451_si_001.zip [file je4c00451_si_001.zip › supporting_information/mix_dev_exp_res_ecs/DECANE+IOCTANE.png]

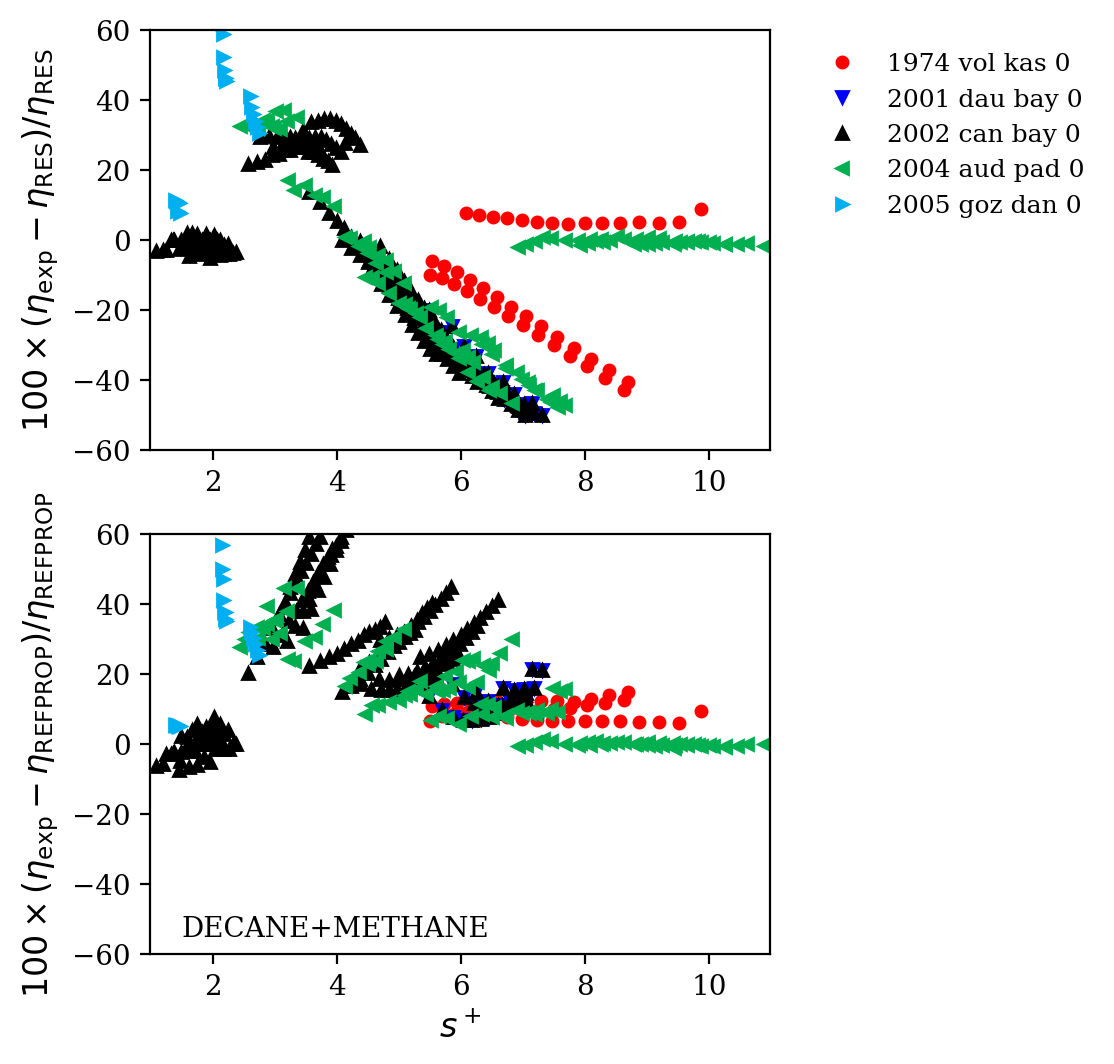

Supplement: Supplementary file 1 — je4c00451_si_001.zip [file je4c00451_si_001.zip › supporting_information/mix_dev_exp_res_ecs/DECANE+METHANE.png]

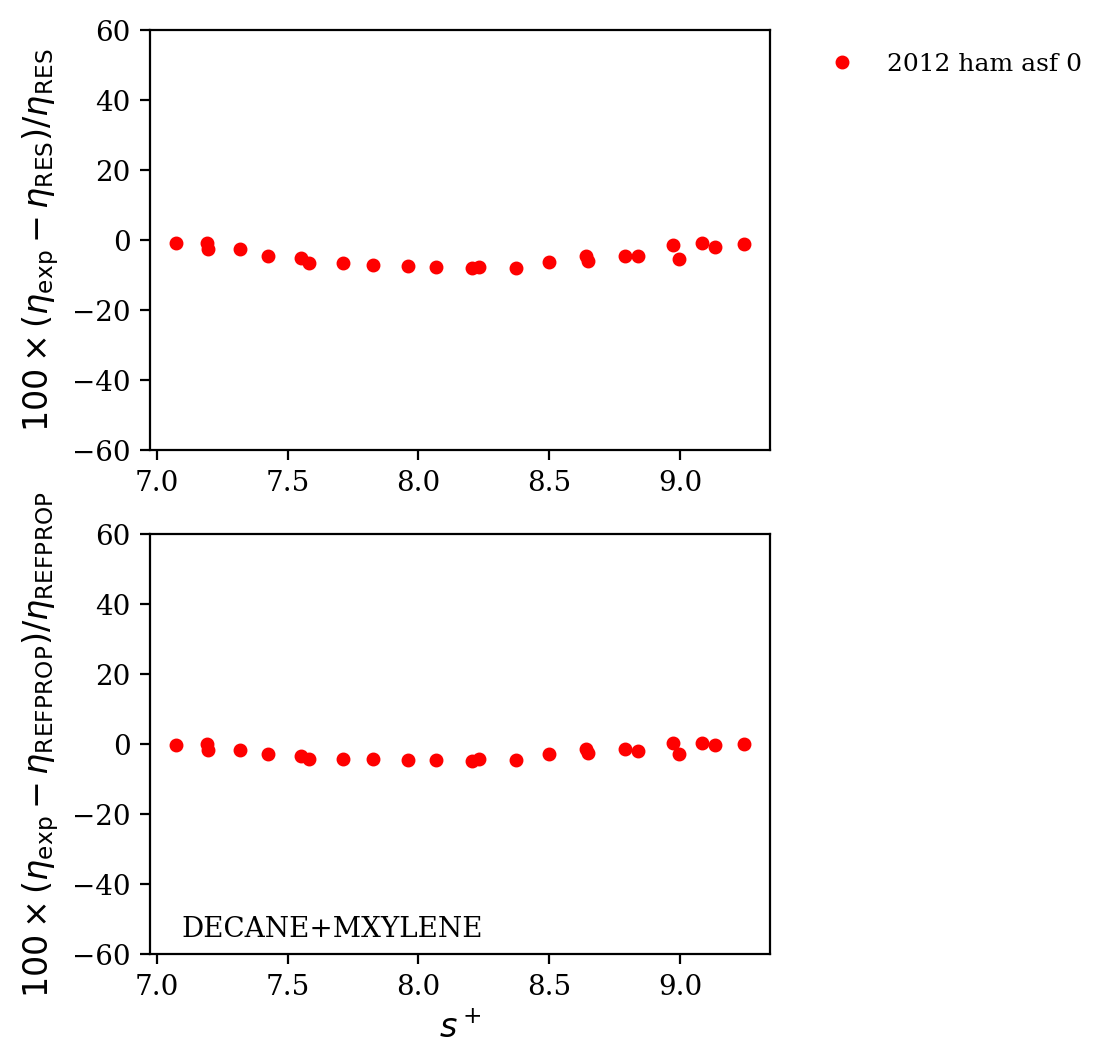

Supplement: Supplementary file 1 — je4c00451_si_001.zip [file je4c00451_si_001.zip › supporting_information/mix_dev_exp_res_ecs/DECANE+MXYLENE.png]

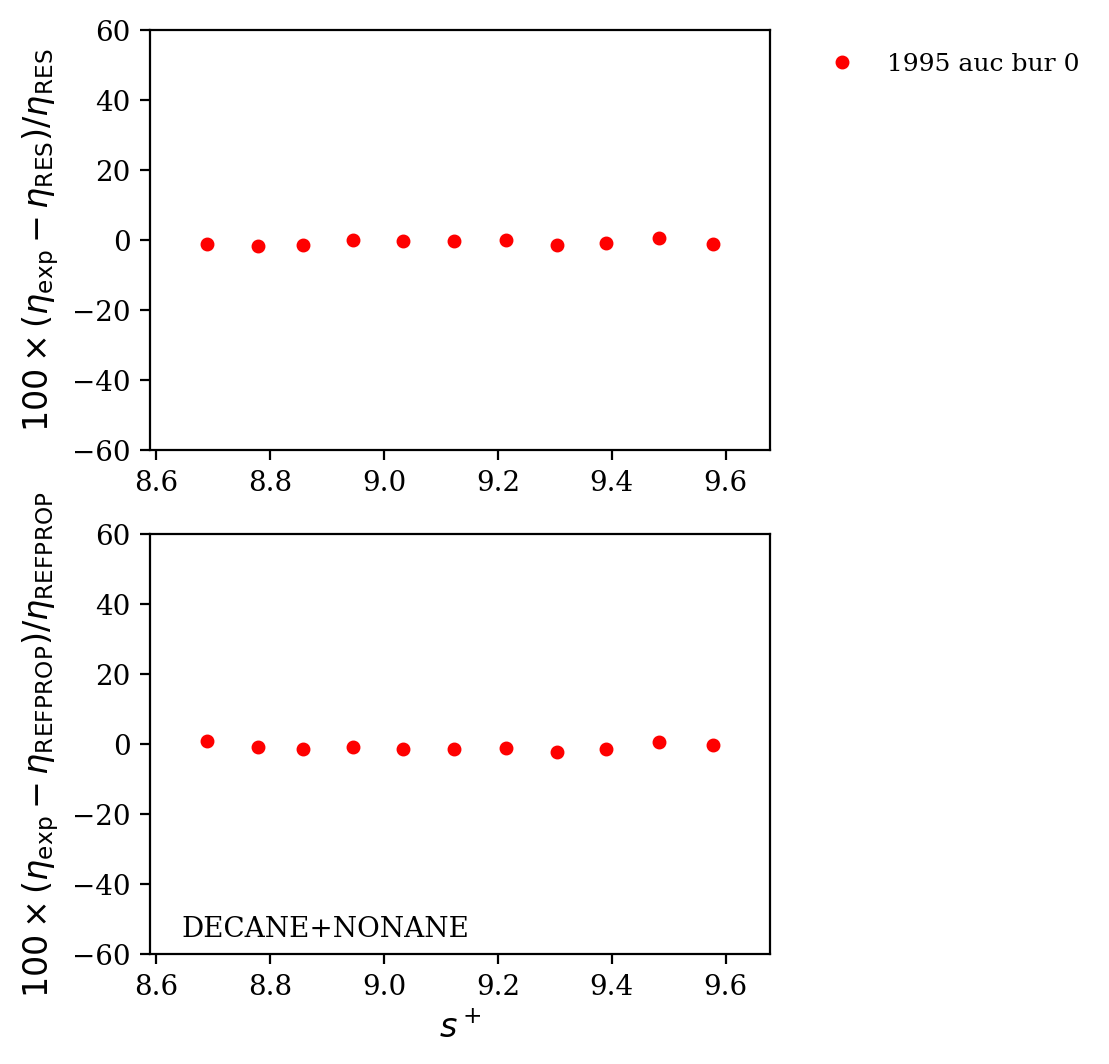

Supplement: Supplementary file 1 — je4c00451_si_001.zip [file je4c00451_si_001.zip › supporting_information/mix_dev_exp_res_ecs/DECANE+NONANE.png]

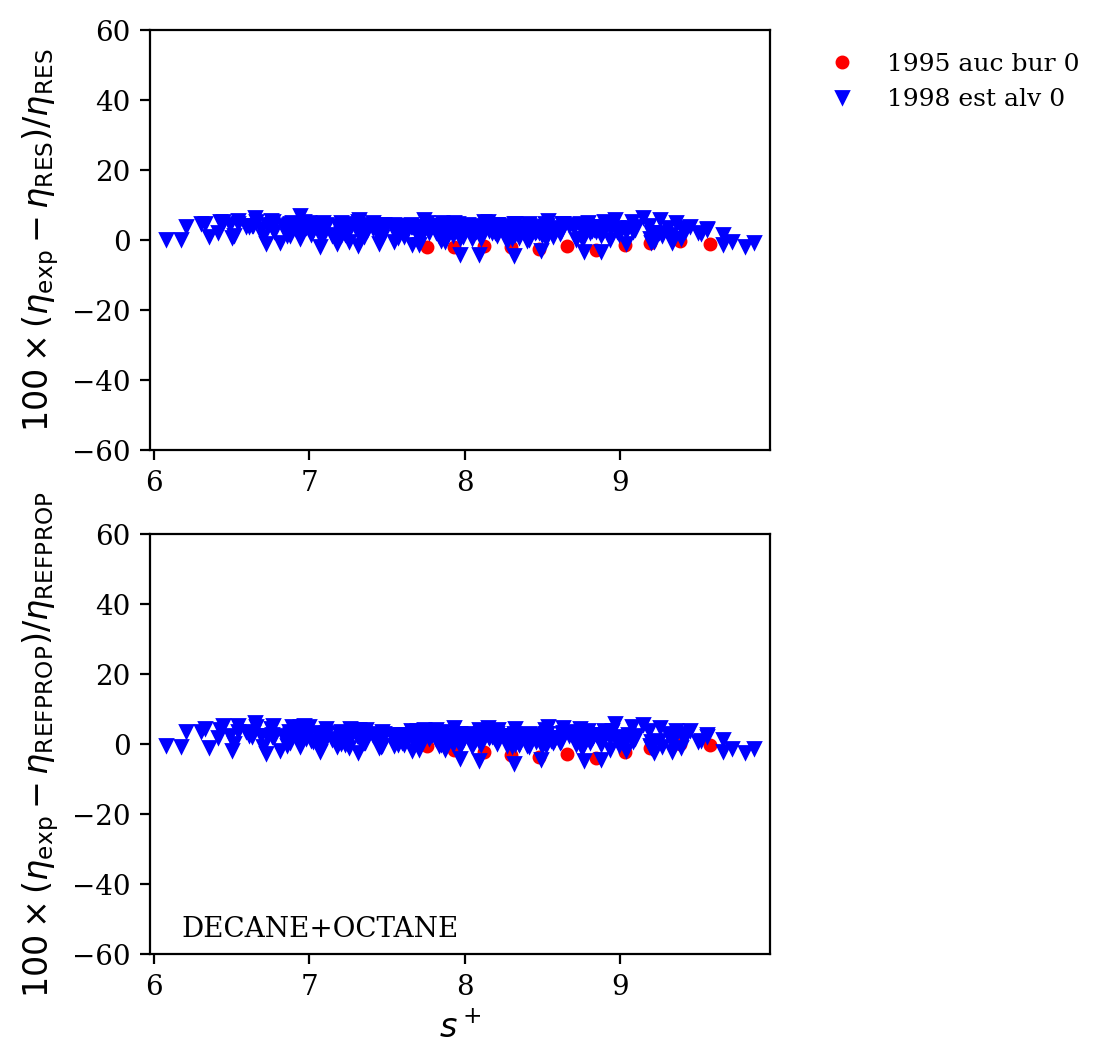

Supplement: Supplementary file 1 — je4c00451_si_001.zip [file je4c00451_si_001.zip › supporting_information/mix_dev_exp_res_ecs/DECANE+OCTANE.png]

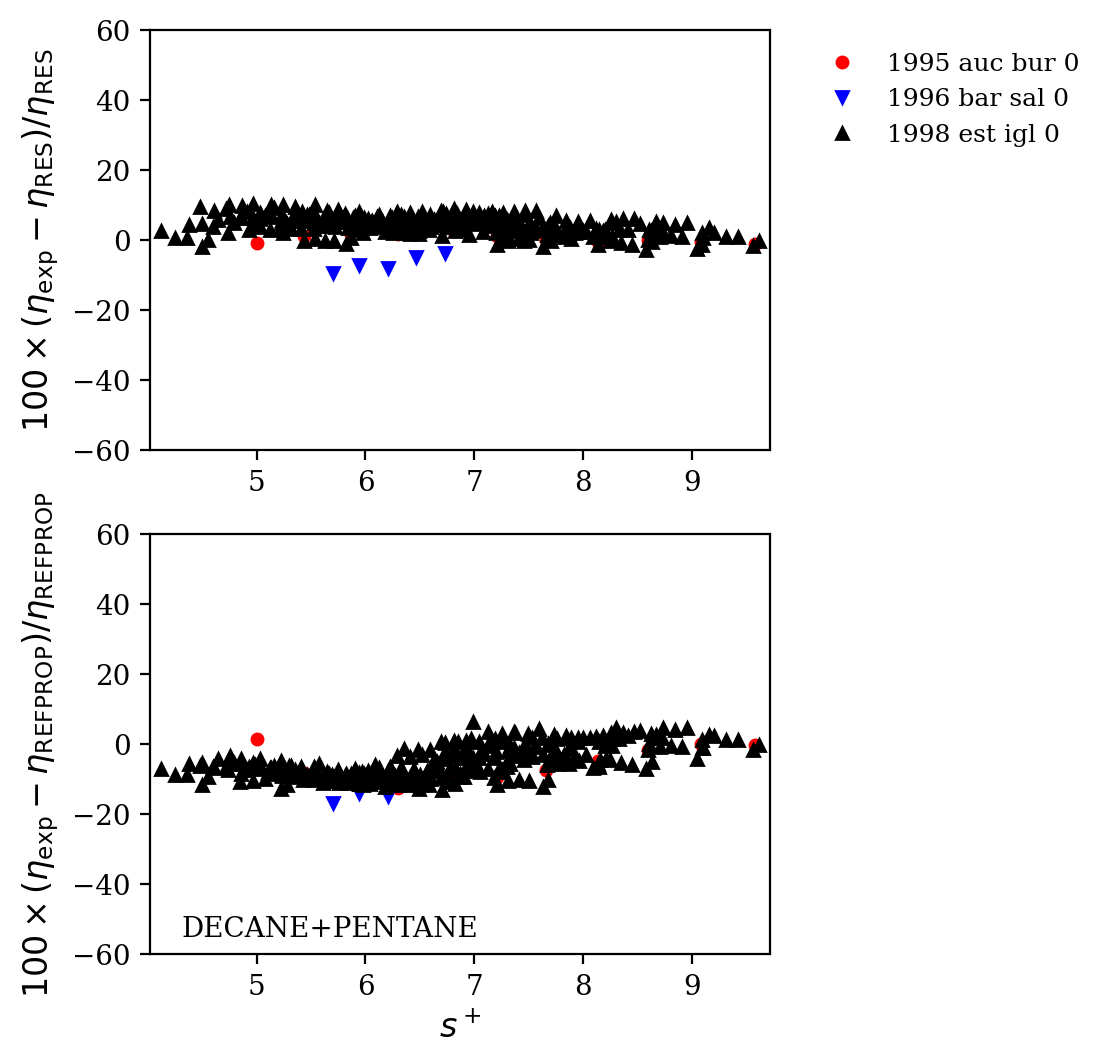

Supplement: Supplementary file 1 — je4c00451_si_001.zip [file je4c00451_si_001.zip › supporting_information/mix_dev_exp_res_ecs/DECANE+PENTANE.png]

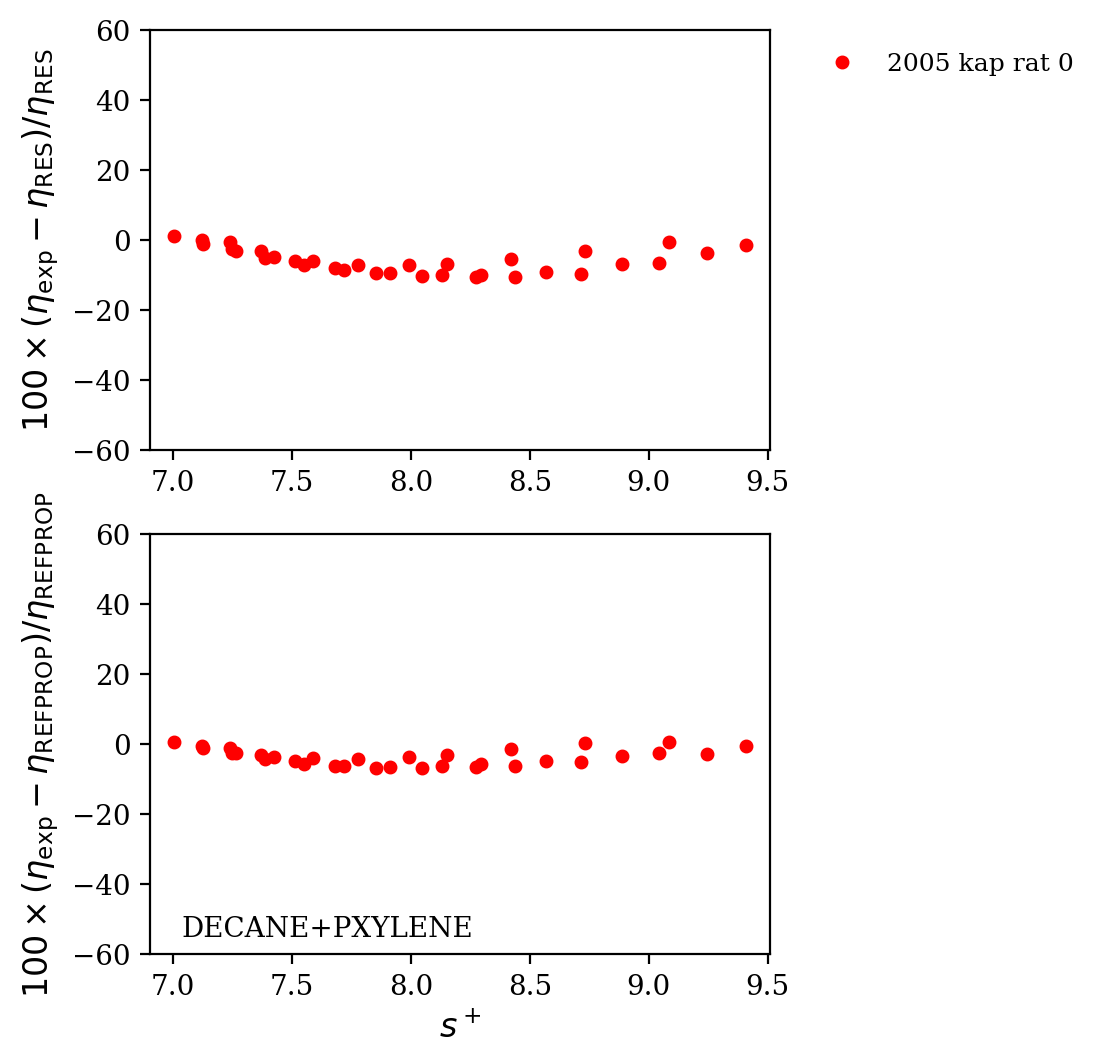

Supplement: Supplementary file 1 — je4c00451_si_001.zip [file je4c00451_si_001.zip › supporting_information/mix_dev_exp_res_ecs/DECANE+PXYLENE.png]
