# Supplementary figures and images for: Entropy Scaling of Viscosity IV—Application to 124 Industrially Important Fluids (part 1 of 2)
Source: J Chem Eng Data. 2025 Jan 10;70(2):727–42. doi: 10.1021/acs.jced.4c00451 (PMC11831593; doi:10.1021/acs.jced.4c00451)

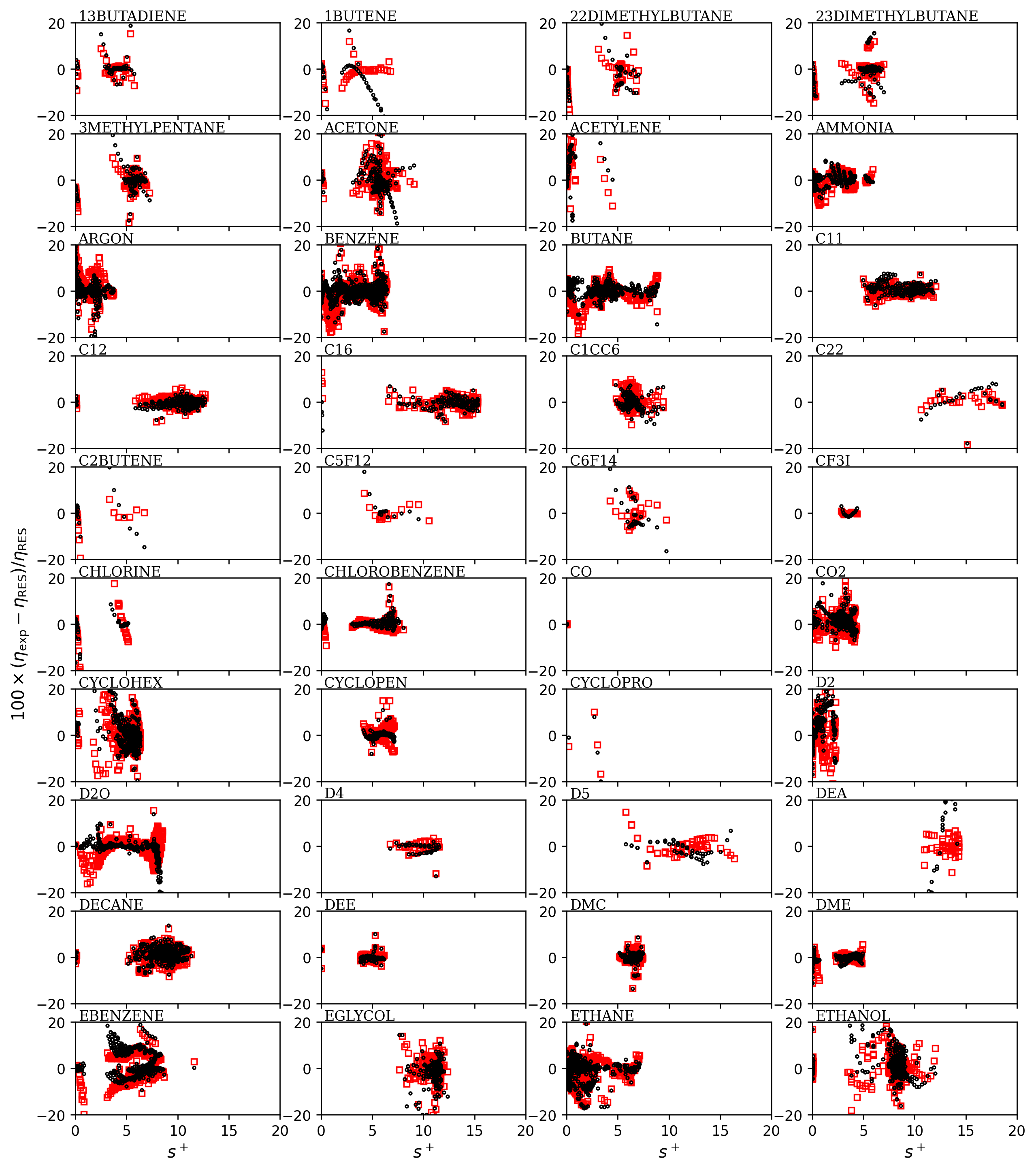

Supplement: Supplementary file 1 — je4c00451_si_001.zip [file je4c00451_si_001.zip › supporting_information/figure_pure_devs/figure_pure_dev_20.png]

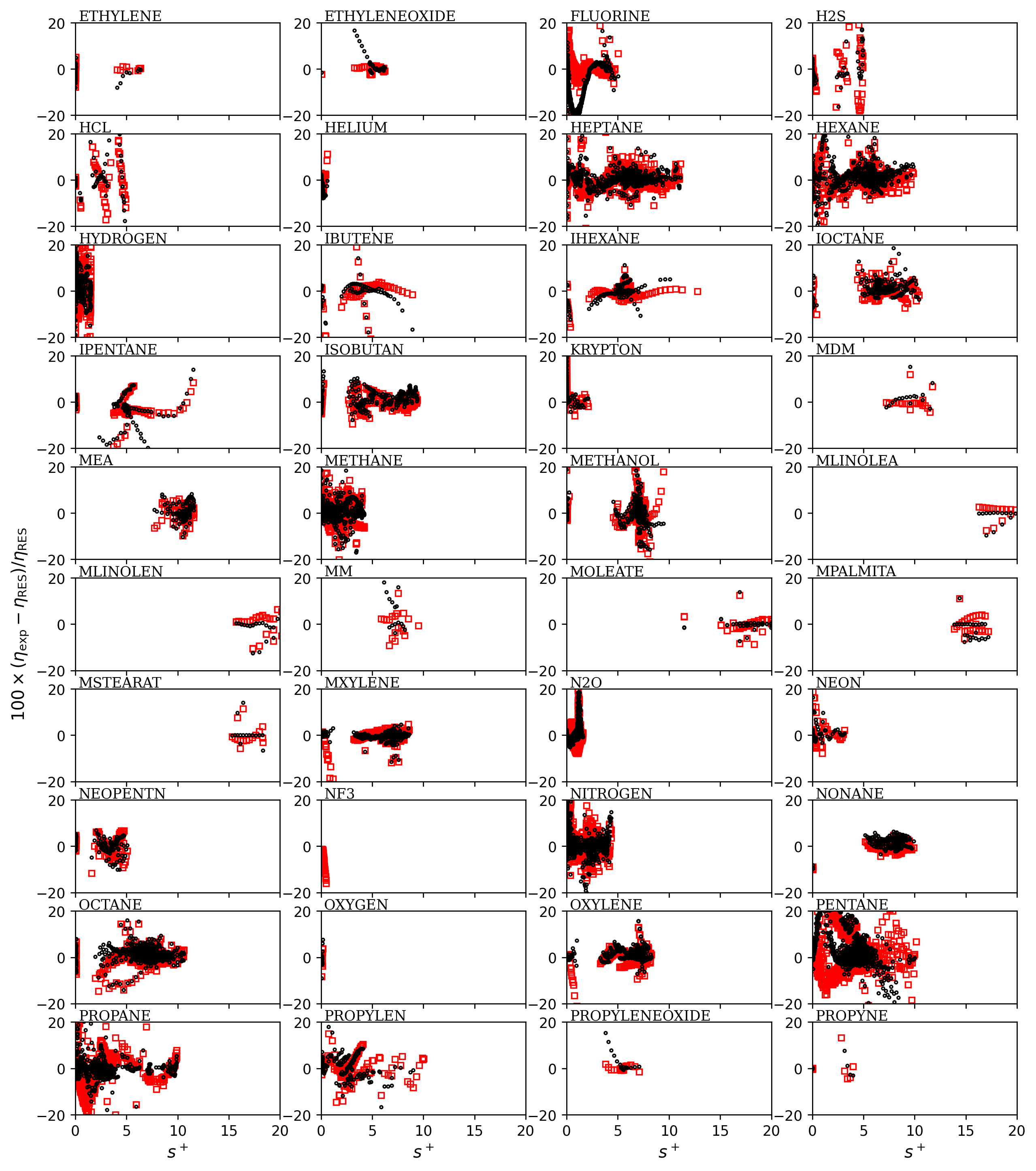

Supplement: Supplementary file 1 — je4c00451_si_001.zip [file je4c00451_si_001.zip › supporting_information/figure_pure_devs/figure_pure_dev_21.png]

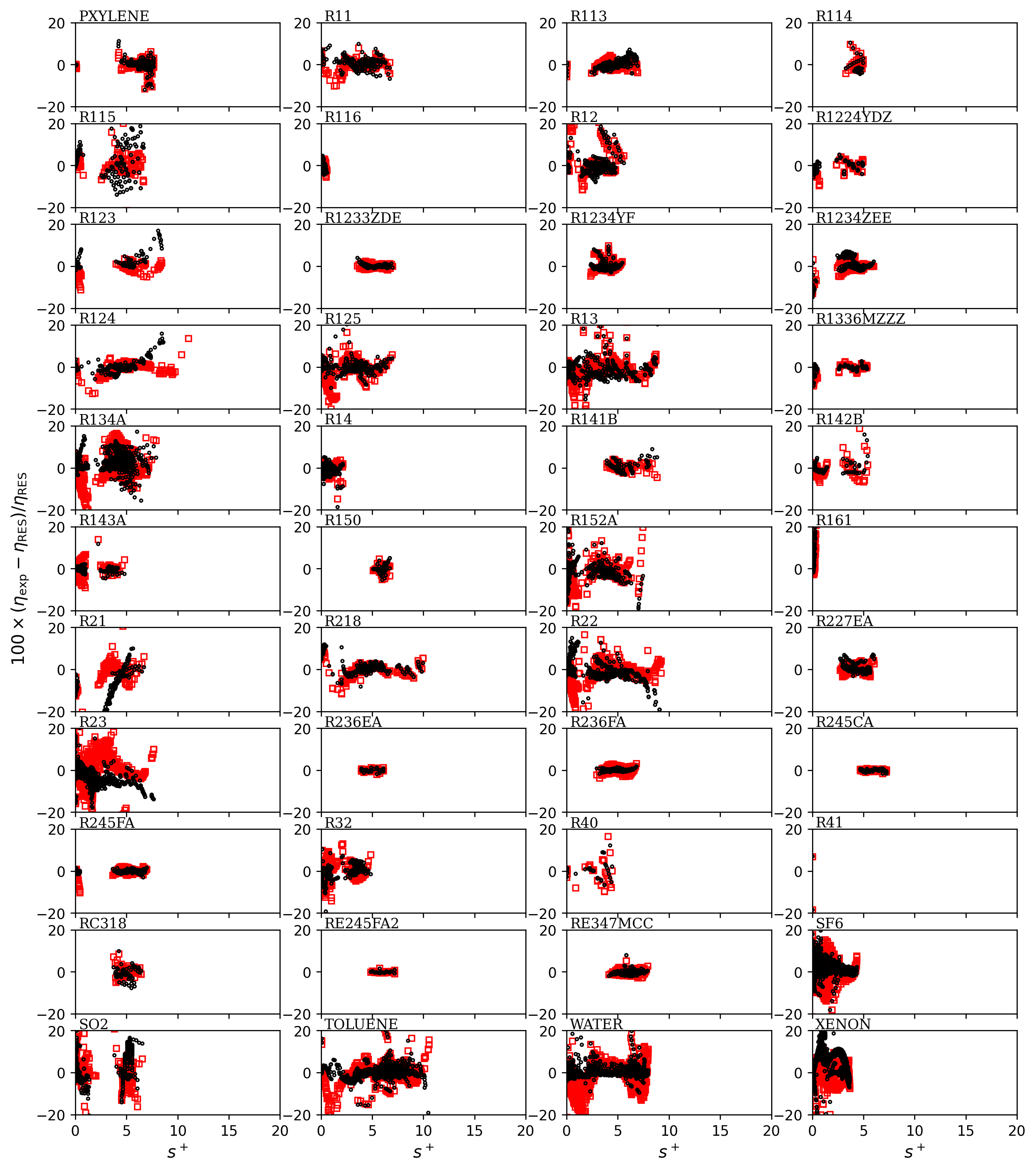

Supplement: Supplementary file 1 — je4c00451_si_001.zip [file je4c00451_si_001.zip › supporting_information/figure_pure_devs/figure_pure_dev_22.png]

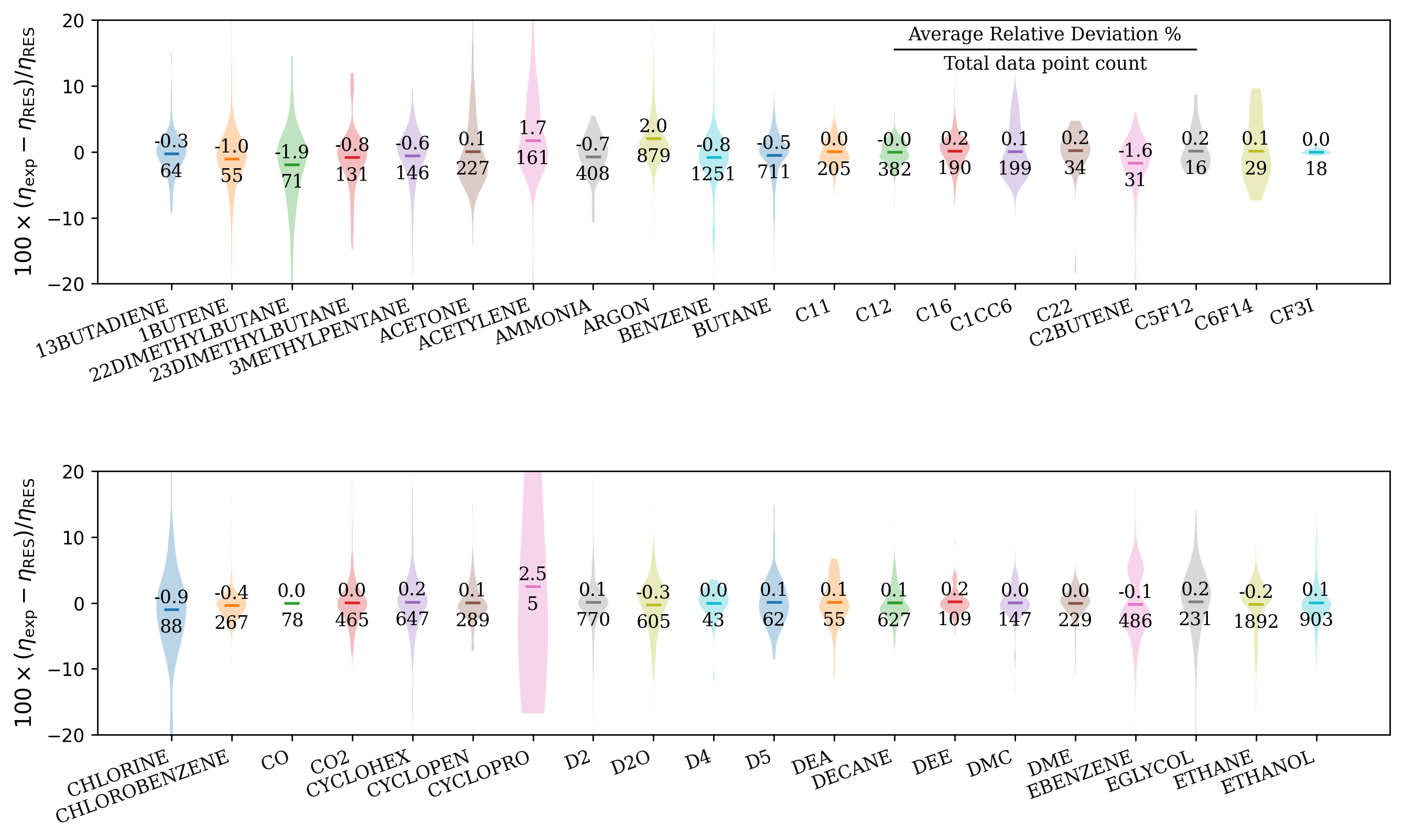

Supplement: Supplementary file 1 — je4c00451_si_001.zip [file je4c00451_si_001.zip › supporting_information/figure_pure_devs/figure_pure_dev_30.png]

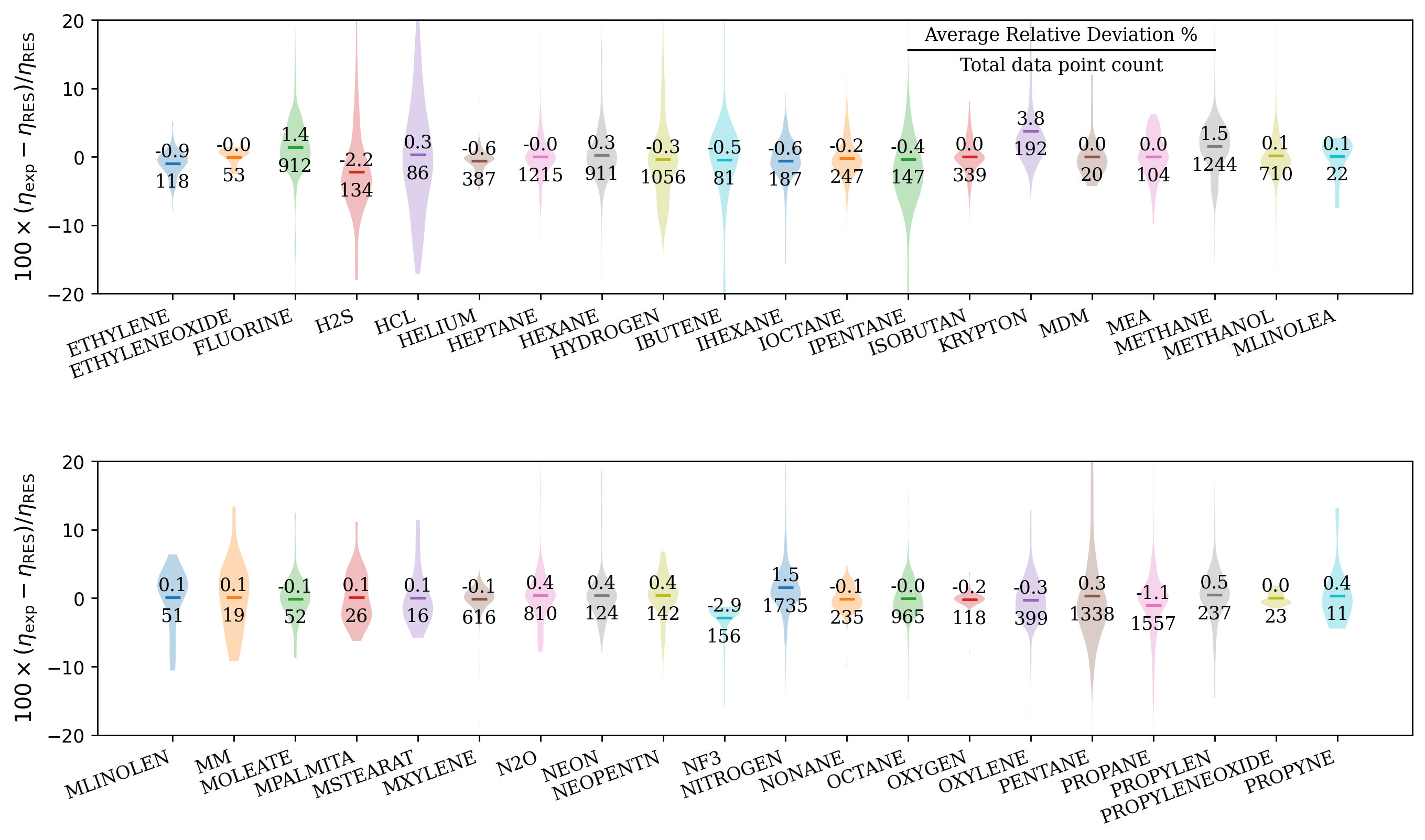

Supplement: Supplementary file 1 — je4c00451_si_001.zip [file je4c00451_si_001.zip › supporting_information/figure_pure_devs/figure_pure_dev_31.png]

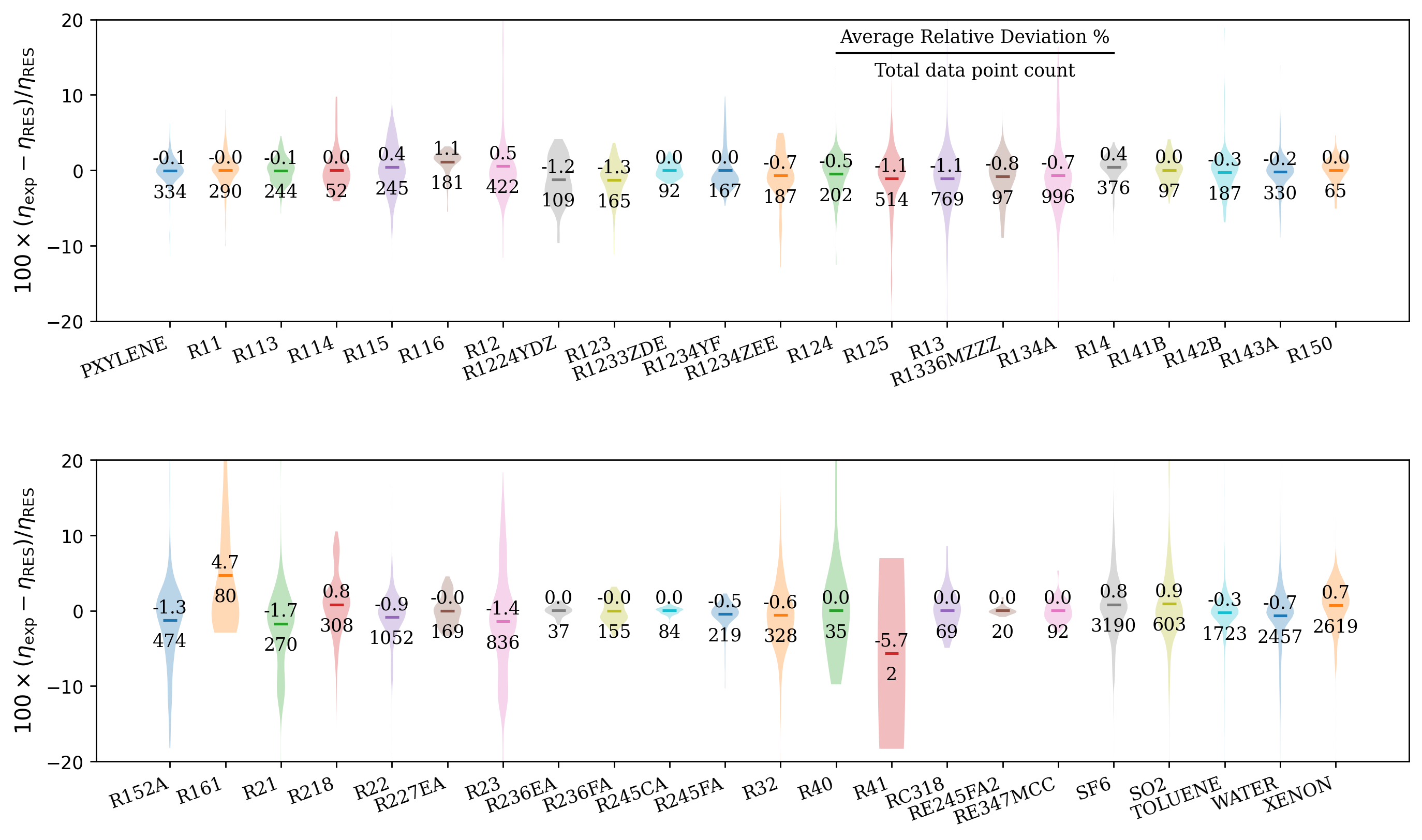

Supplement: Supplementary file 1 — je4c00451_si_001.zip [file je4c00451_si_001.zip › supporting_information/figure_pure_devs/figure_pure_dev_32.png]

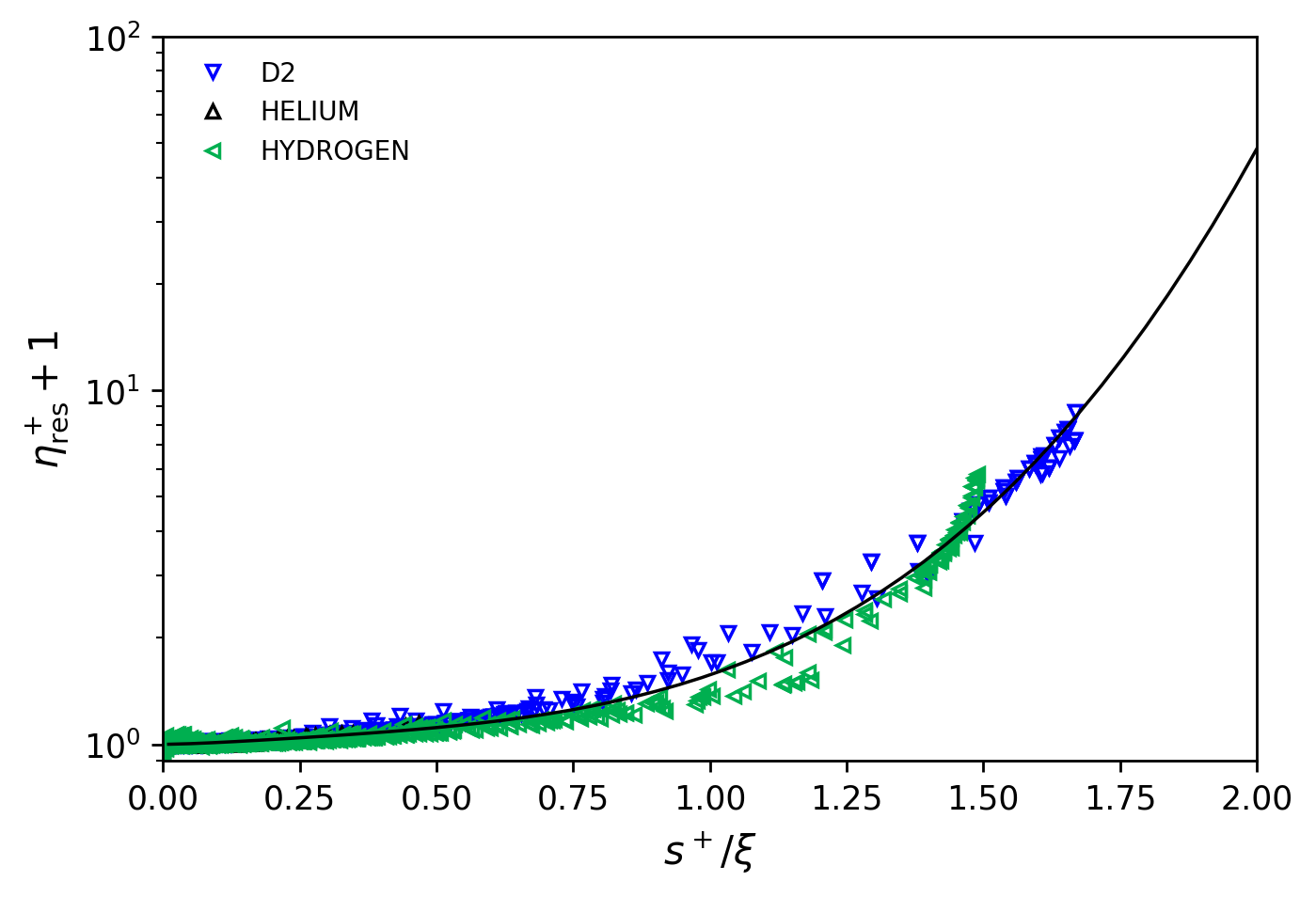

Supplement: Supplementary file 1 — je4c00451_si_001.zip [file je4c00451_si_001.zip › supporting_information/figure_pure_groups/Group1.png]

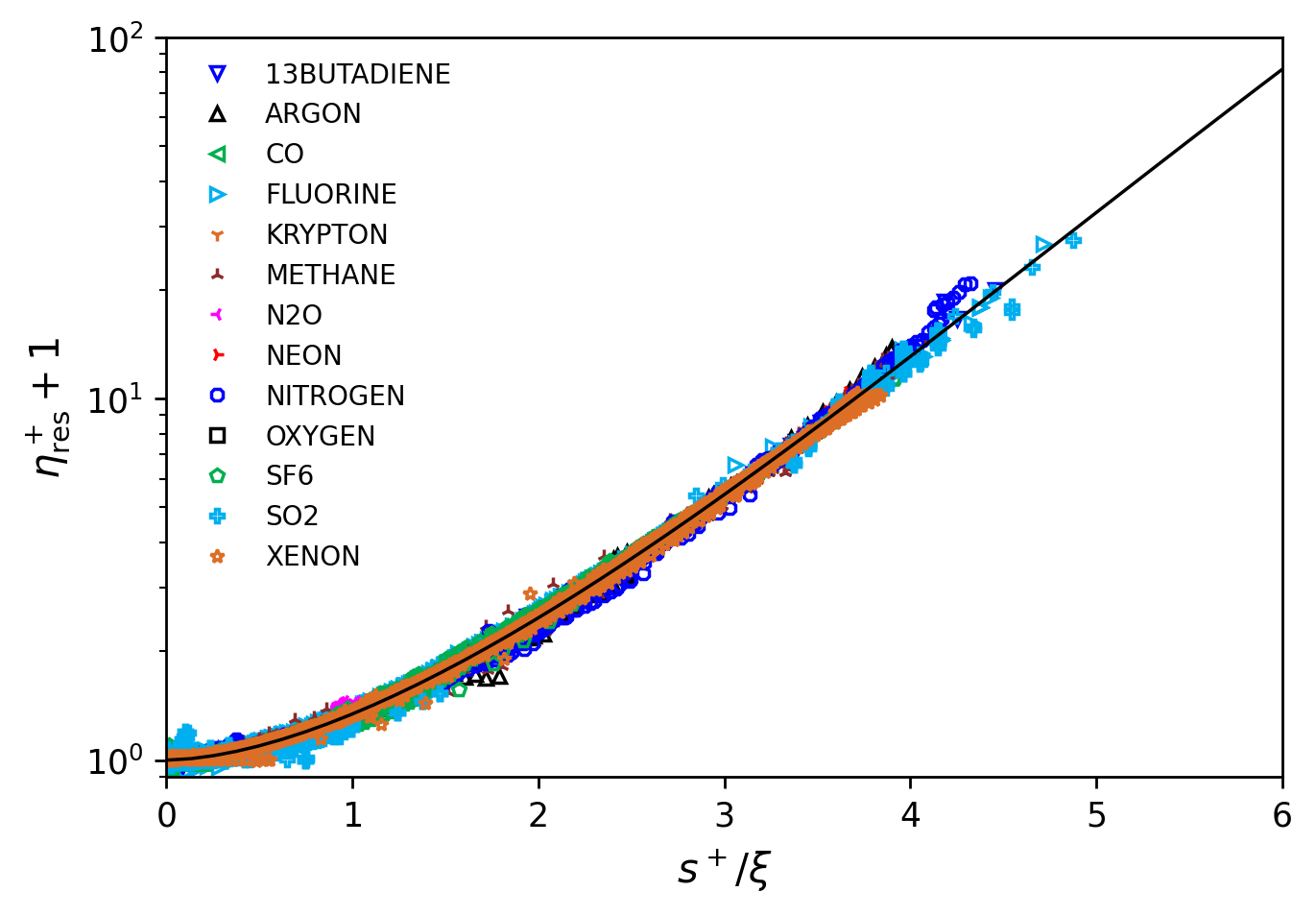

Supplement: Supplementary file 1 — je4c00451_si_001.zip [file je4c00451_si_001.zip › supporting_information/figure_pure_groups/Group2.png]

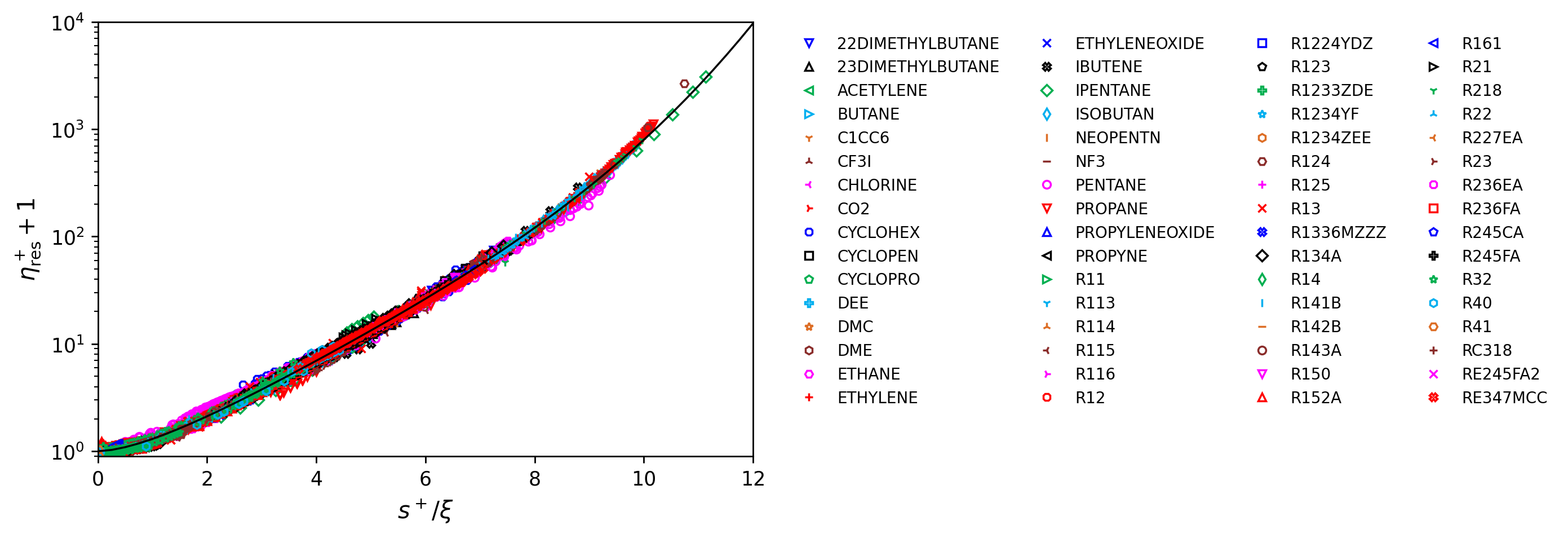

Supplement: Supplementary file 1 — je4c00451_si_001.zip [file je4c00451_si_001.zip › supporting_information/figure_pure_groups/Group3.png]

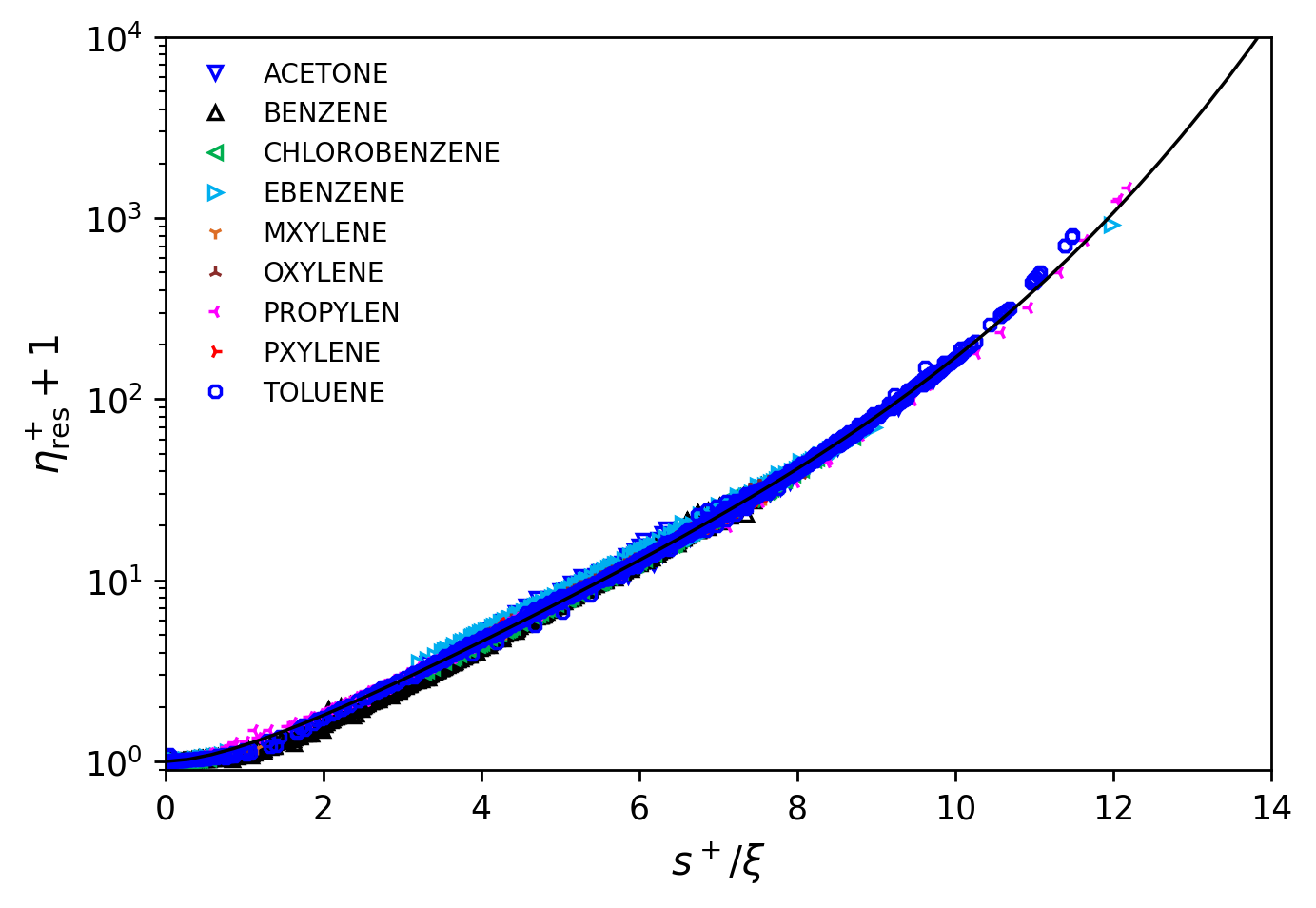

Supplement: Supplementary file 1 — je4c00451_si_001.zip [file je4c00451_si_001.zip › supporting_information/figure_pure_groups/Group4.png]

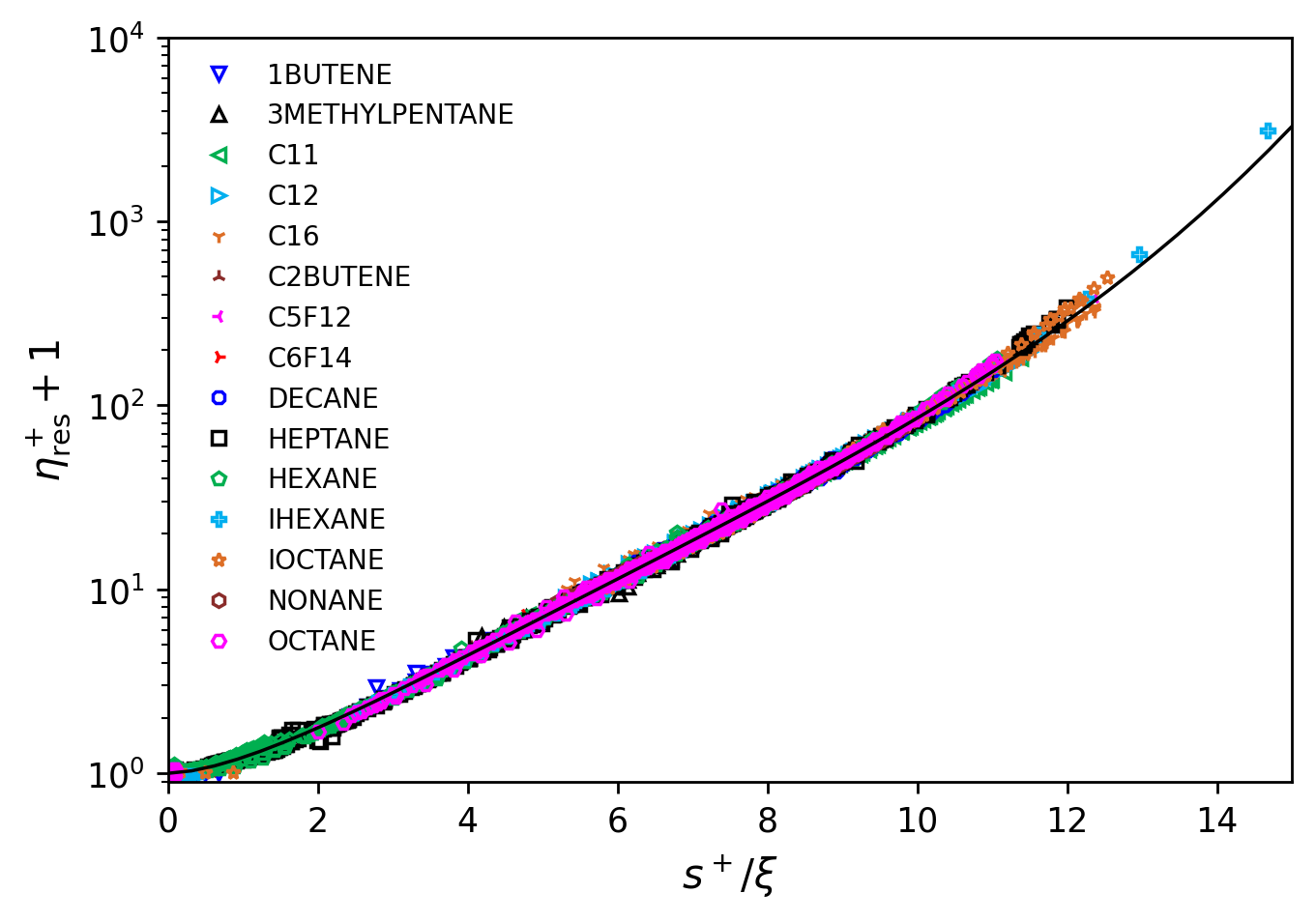

Supplement: Supplementary file 1 — je4c00451_si_001.zip [file je4c00451_si_001.zip › supporting_information/figure_pure_groups/Group5.png]

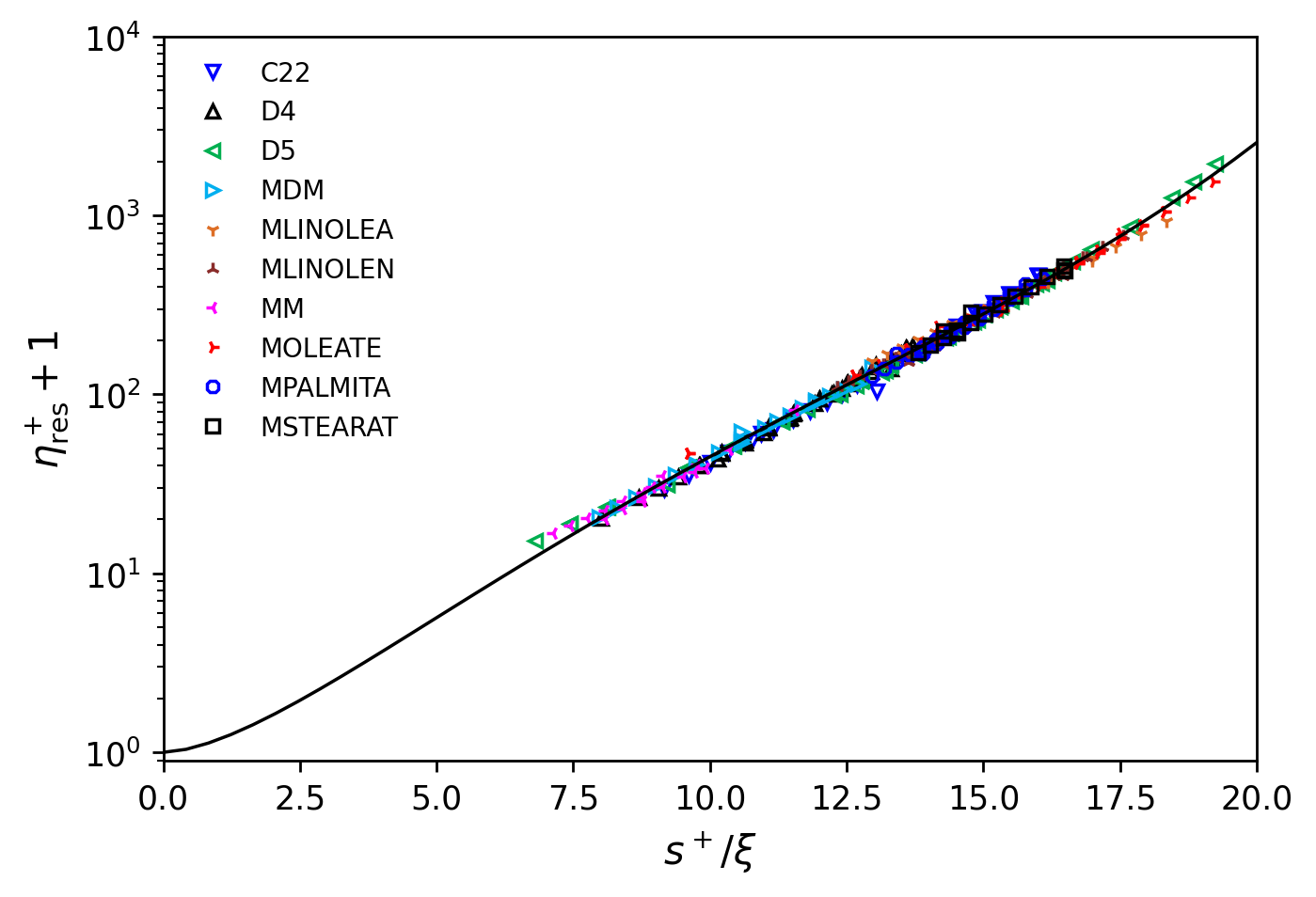

Supplement: Supplementary file 1 — je4c00451_si_001.zip [file je4c00451_si_001.zip › supporting_information/figure_pure_groups/Group6.png]

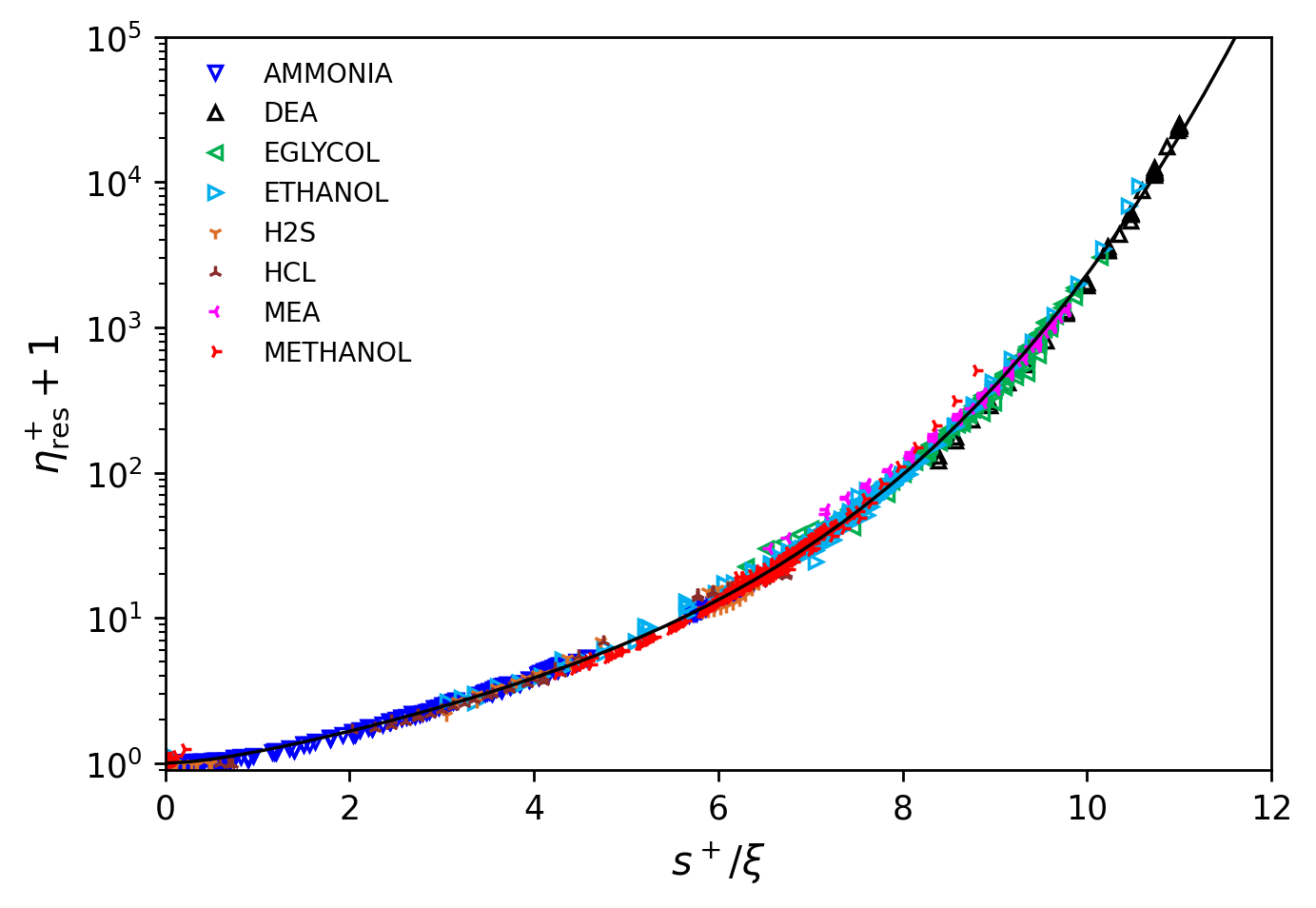

Supplement: Supplementary file 1 — je4c00451_si_001.zip [file je4c00451_si_001.zip › supporting_information/figure_pure_groups/Group7.png]

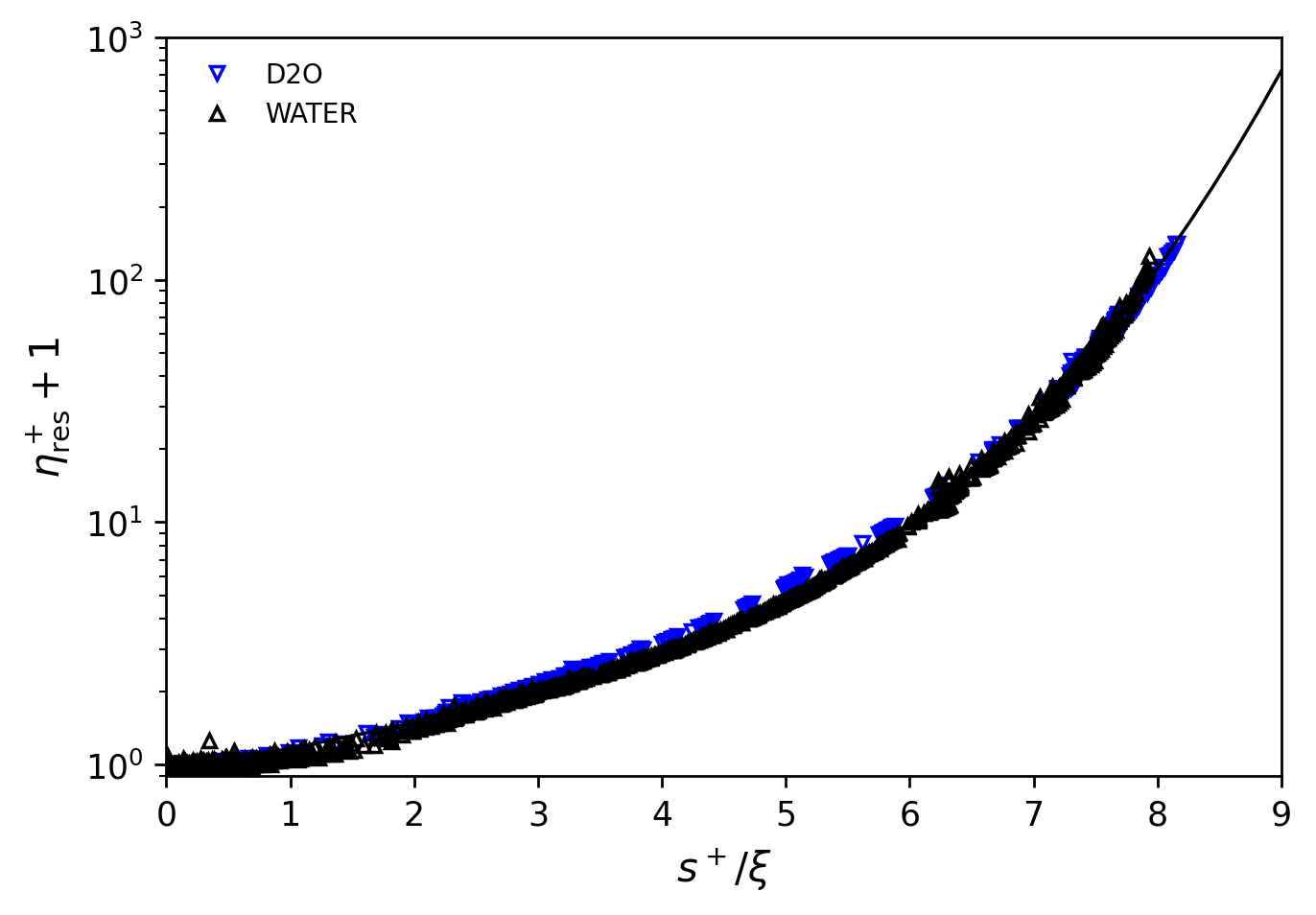

Supplement: Supplementary file 1 — je4c00451_si_001.zip [file je4c00451_si_001.zip › supporting_information/figure_pure_groups/Group8.png]

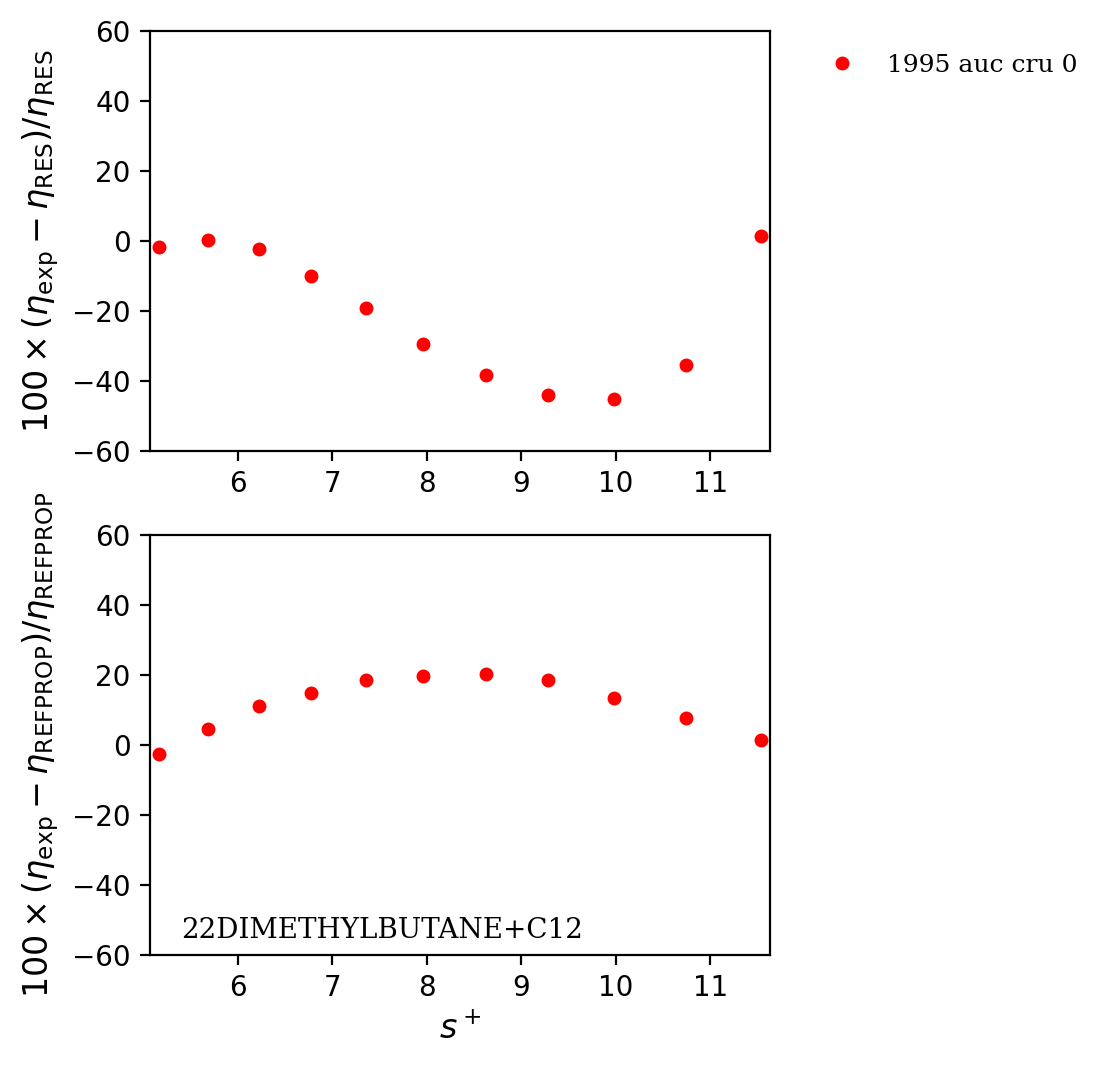

Supplement: Supplementary file 1 — je4c00451_si_001.zip [file je4c00451_si_001.zip › supporting_information/mix_dev_exp_res_ecs/22DIMETHYLBUTANE+C12.png]

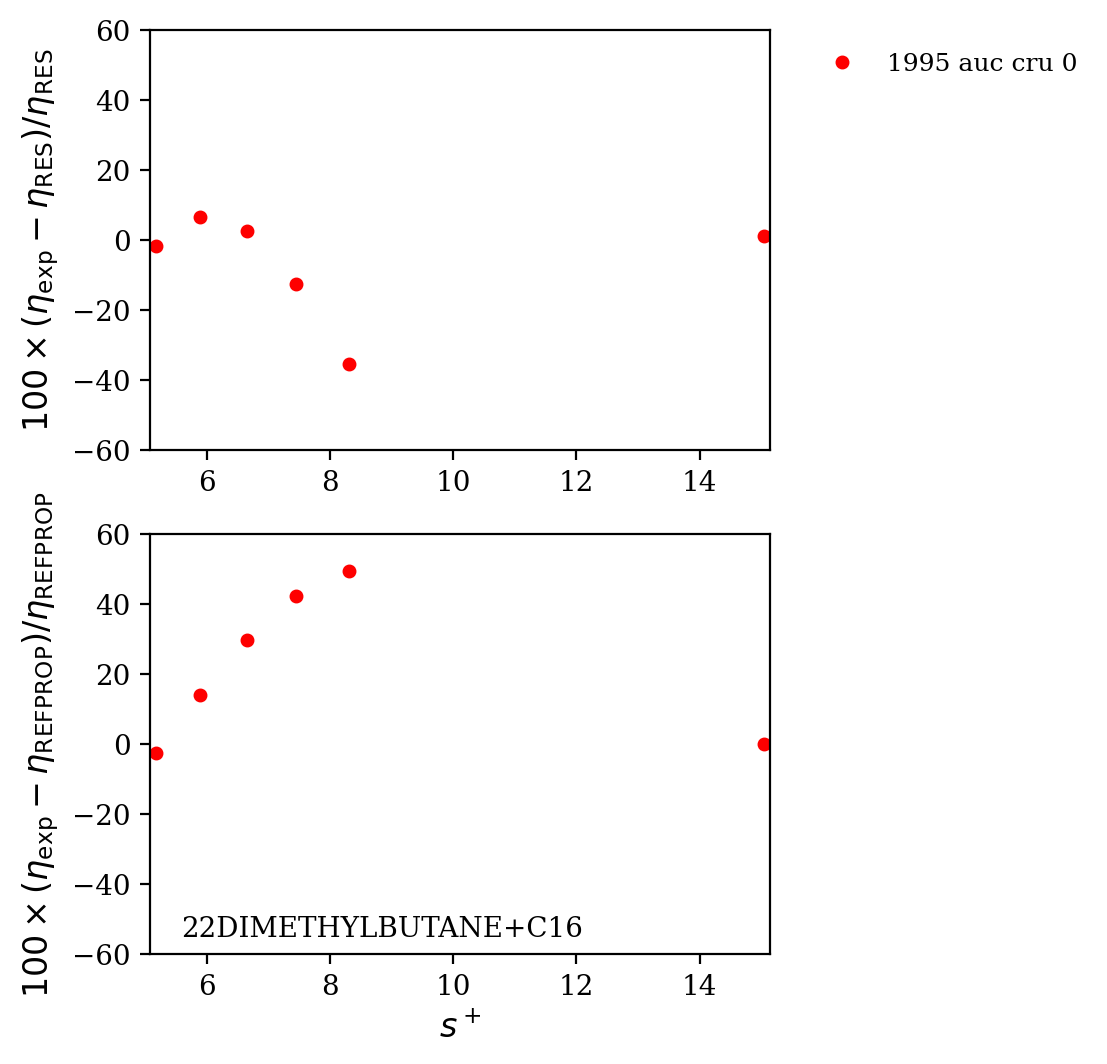

Supplement: Supplementary file 1 — je4c00451_si_001.zip [file je4c00451_si_001.zip › supporting_information/mix_dev_exp_res_ecs/22DIMETHYLBUTANE+C16.png]

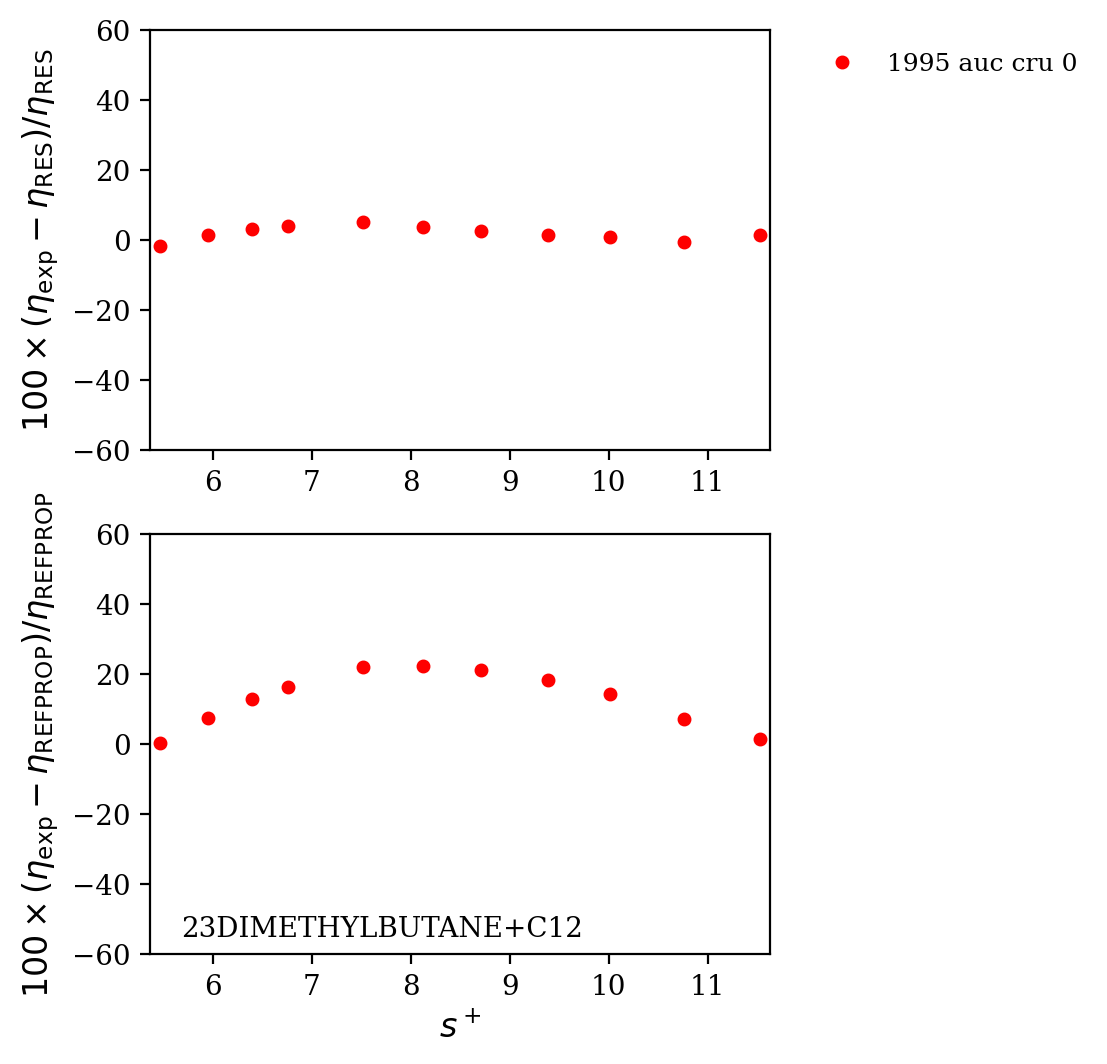

Supplement: Supplementary file 1 — je4c00451_si_001.zip [file je4c00451_si_001.zip › supporting_information/mix_dev_exp_res_ecs/23DIMETHYLBUTANE+C12.png]

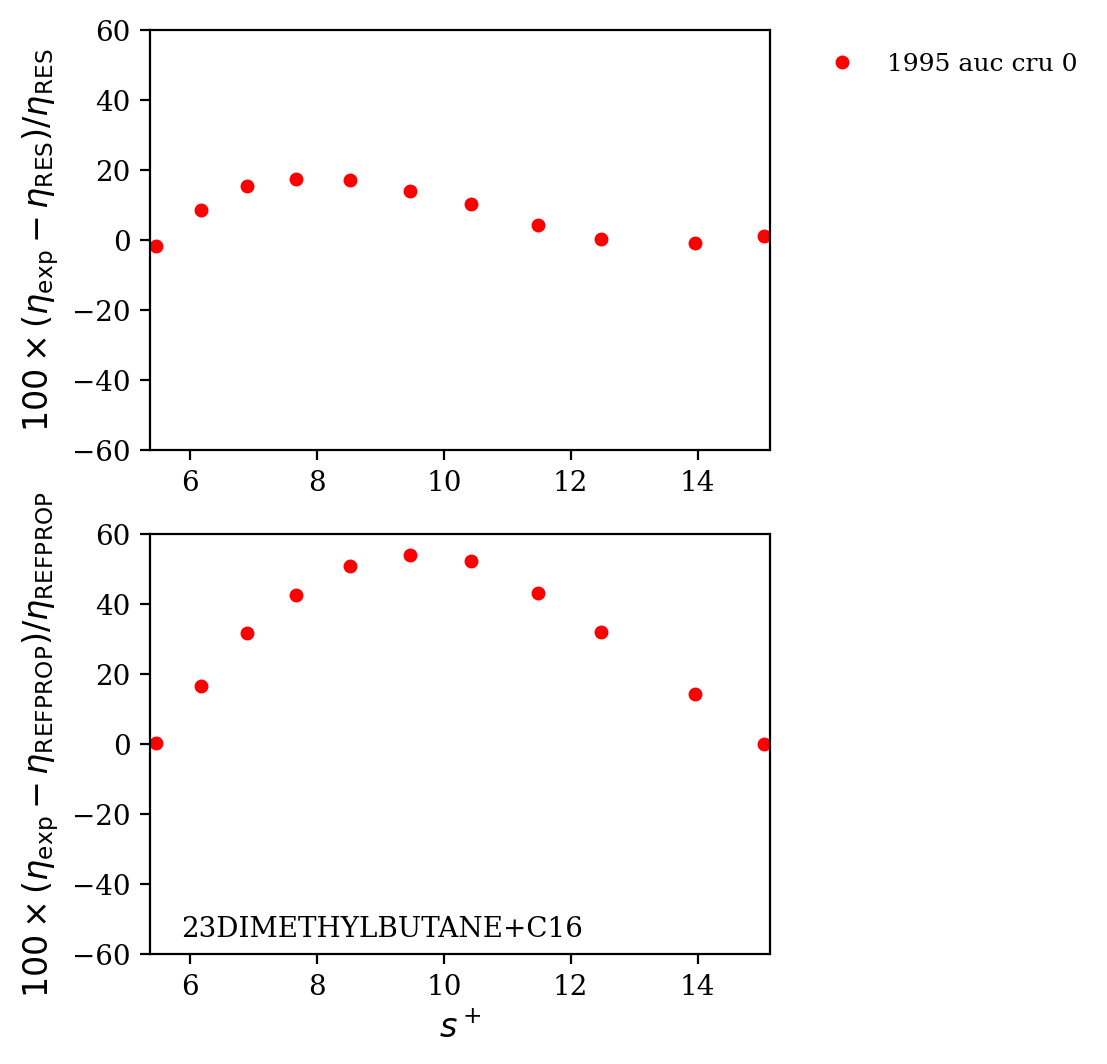

Supplement: Supplementary file 1 — je4c00451_si_001.zip [file je4c00451_si_001.zip › supporting_information/mix_dev_exp_res_ecs/23DIMETHYLBUTANE+C16.png]

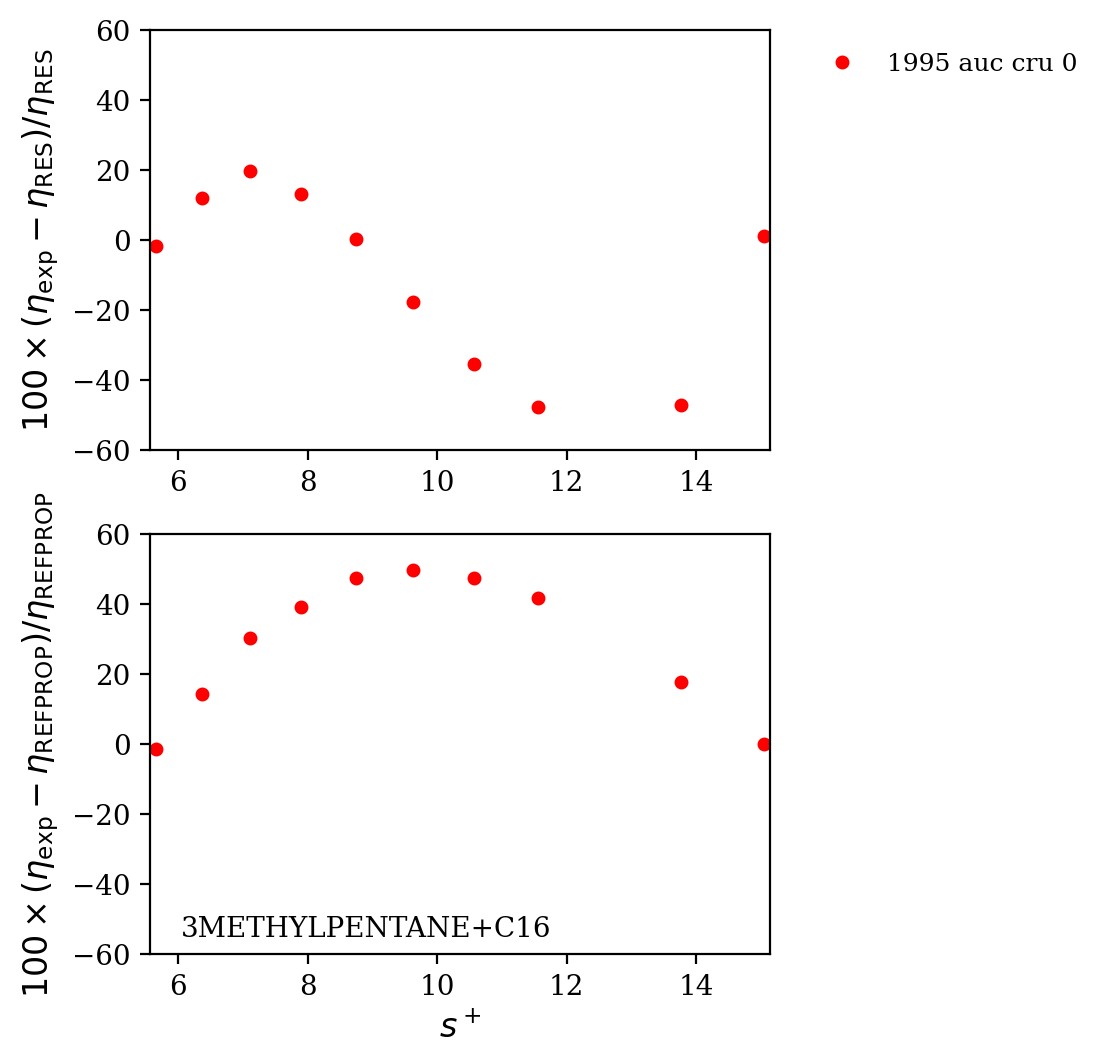

Supplement: Supplementary file 1 — je4c00451_si_001.zip [file je4c00451_si_001.zip › supporting_information/mix_dev_exp_res_ecs/3METHYLPENTANE+C16.png]

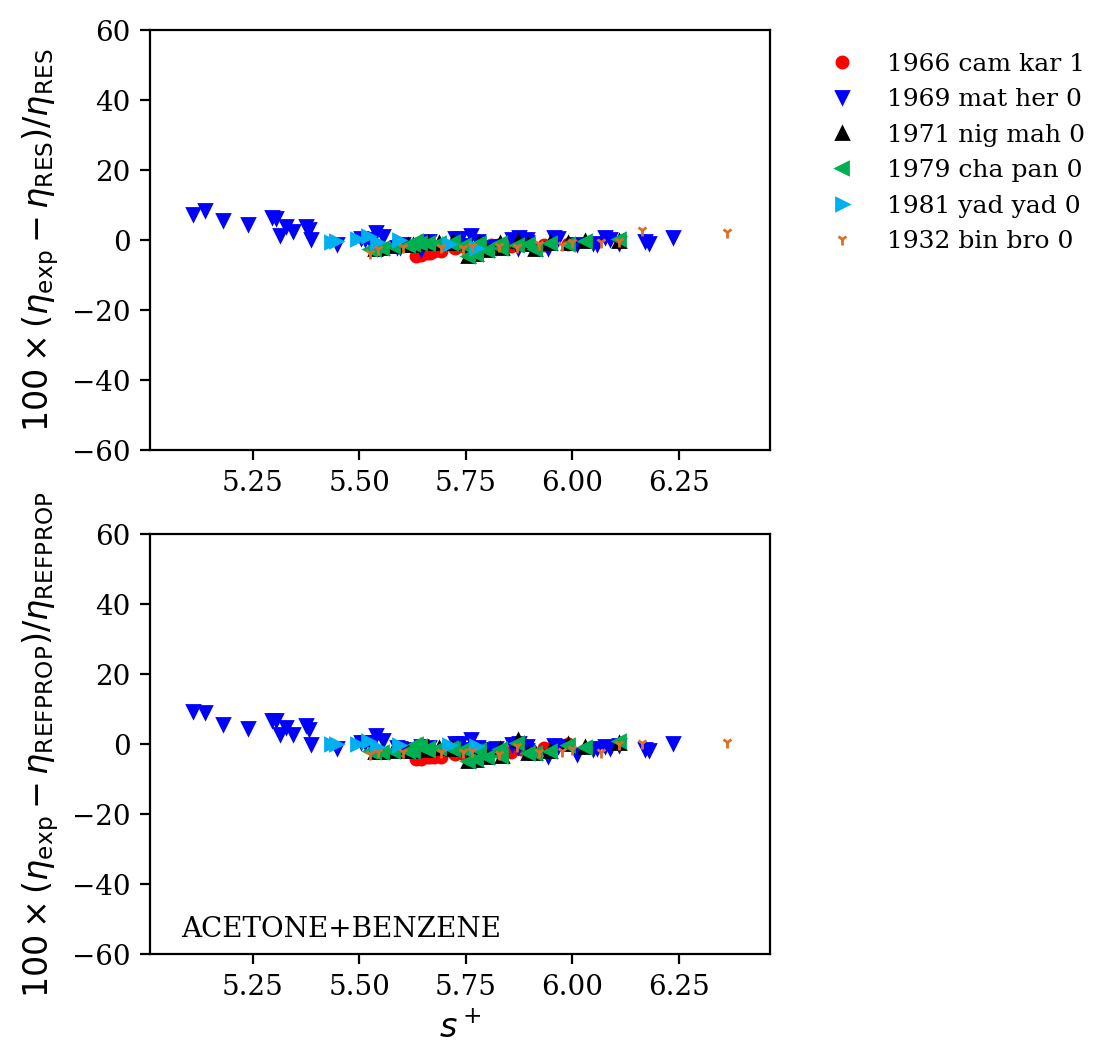

Supplement: Supplementary file 1 — je4c00451_si_001.zip [file je4c00451_si_001.zip › supporting_information/mix_dev_exp_res_ecs/ACETONE+BENZENE.png]

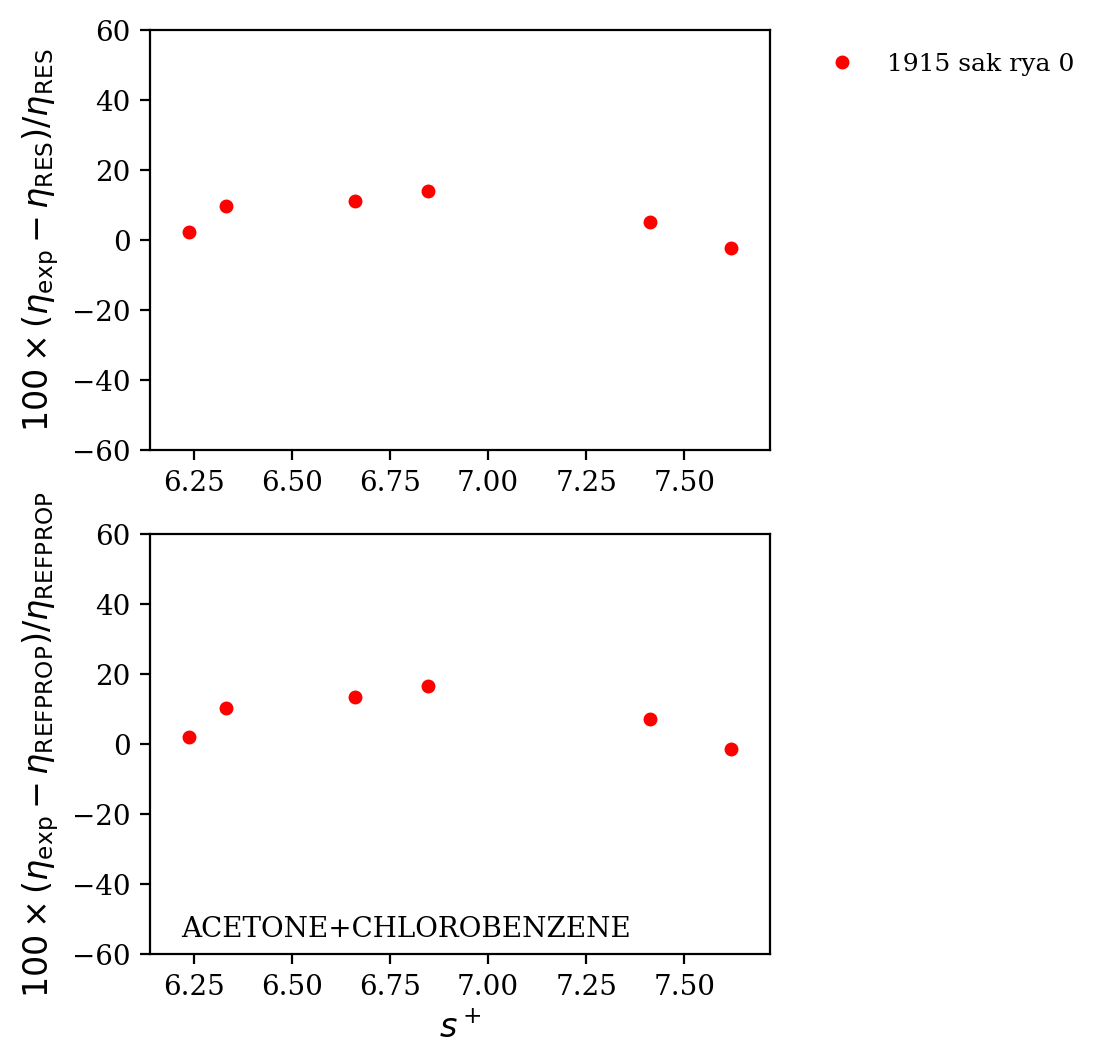

Supplement: Supplementary file 1 — je4c00451_si_001.zip [file je4c00451_si_001.zip › supporting_information/mix_dev_exp_res_ecs/ACETONE+CHLOROBENZENE.png]

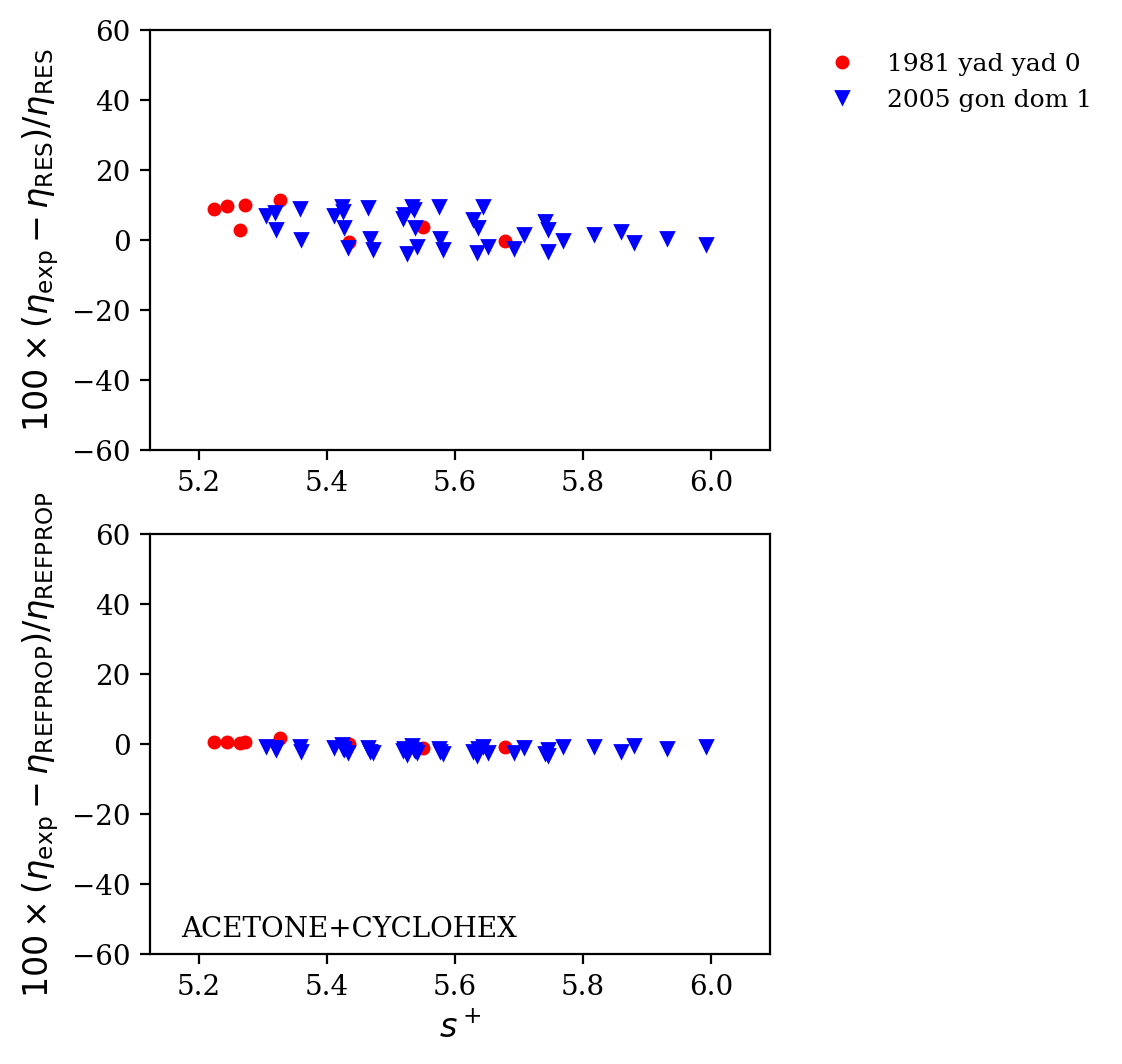

Supplement: Supplementary file 1 — je4c00451_si_001.zip [file je4c00451_si_001.zip › supporting_information/mix_dev_exp_res_ecs/ACETONE+CYCLOHEX.png]

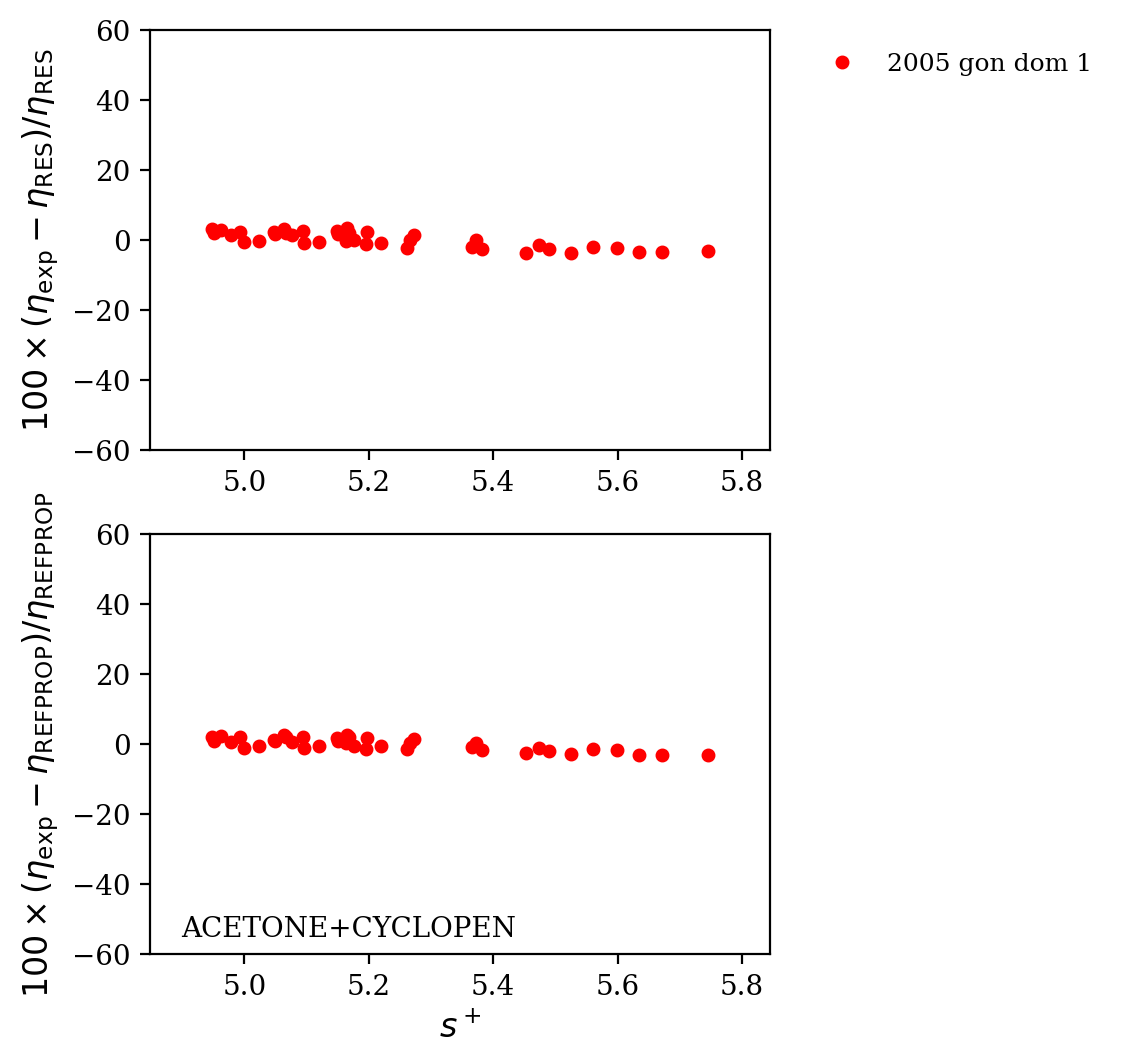

Supplement: Supplementary file 1 — je4c00451_si_001.zip [file je4c00451_si_001.zip › supporting_information/mix_dev_exp_res_ecs/ACETONE+CYCLOPEN.png]

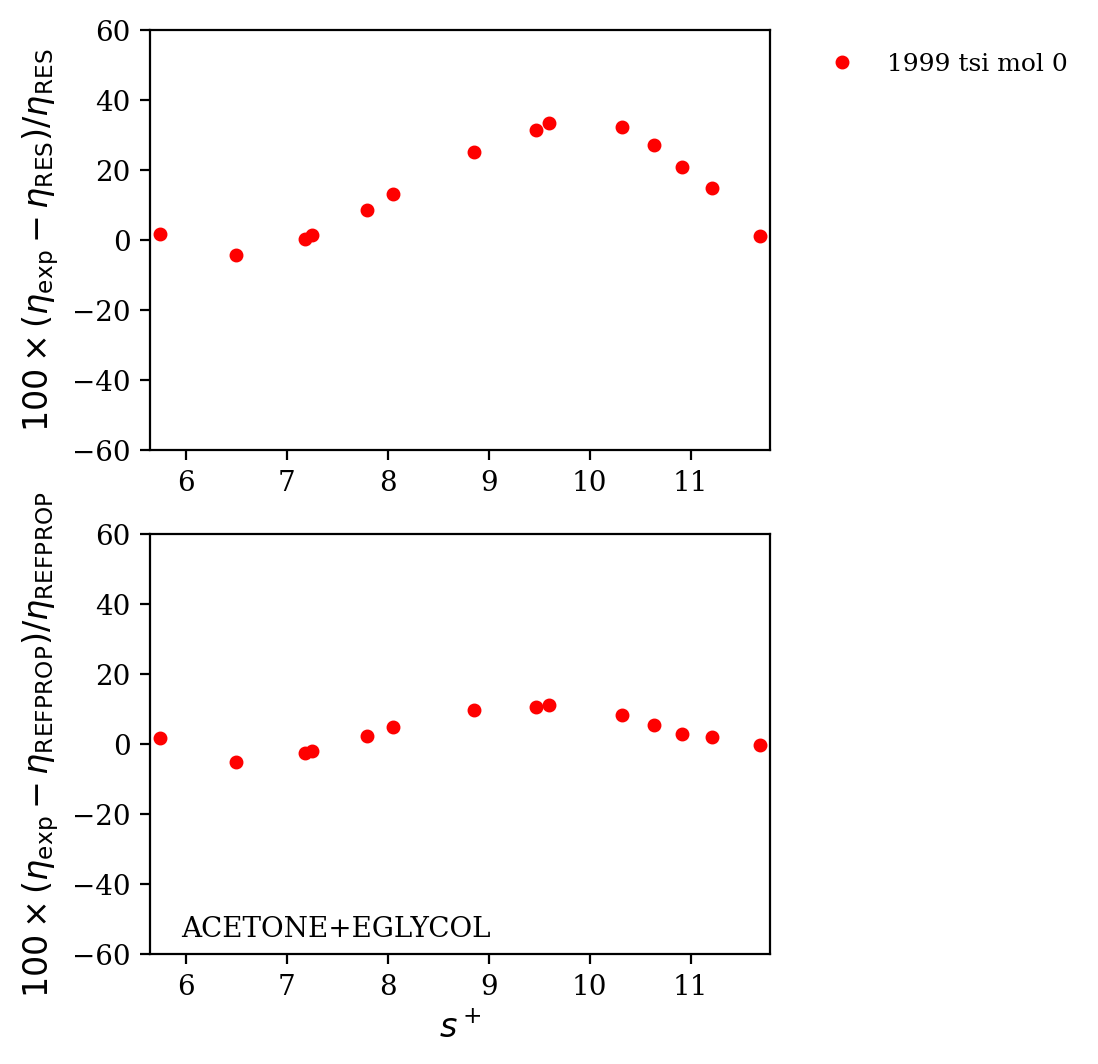

Supplement: Supplementary file 1 — je4c00451_si_001.zip [file je4c00451_si_001.zip › supporting_information/mix_dev_exp_res_ecs/ACETONE+EGLYCOL.png]

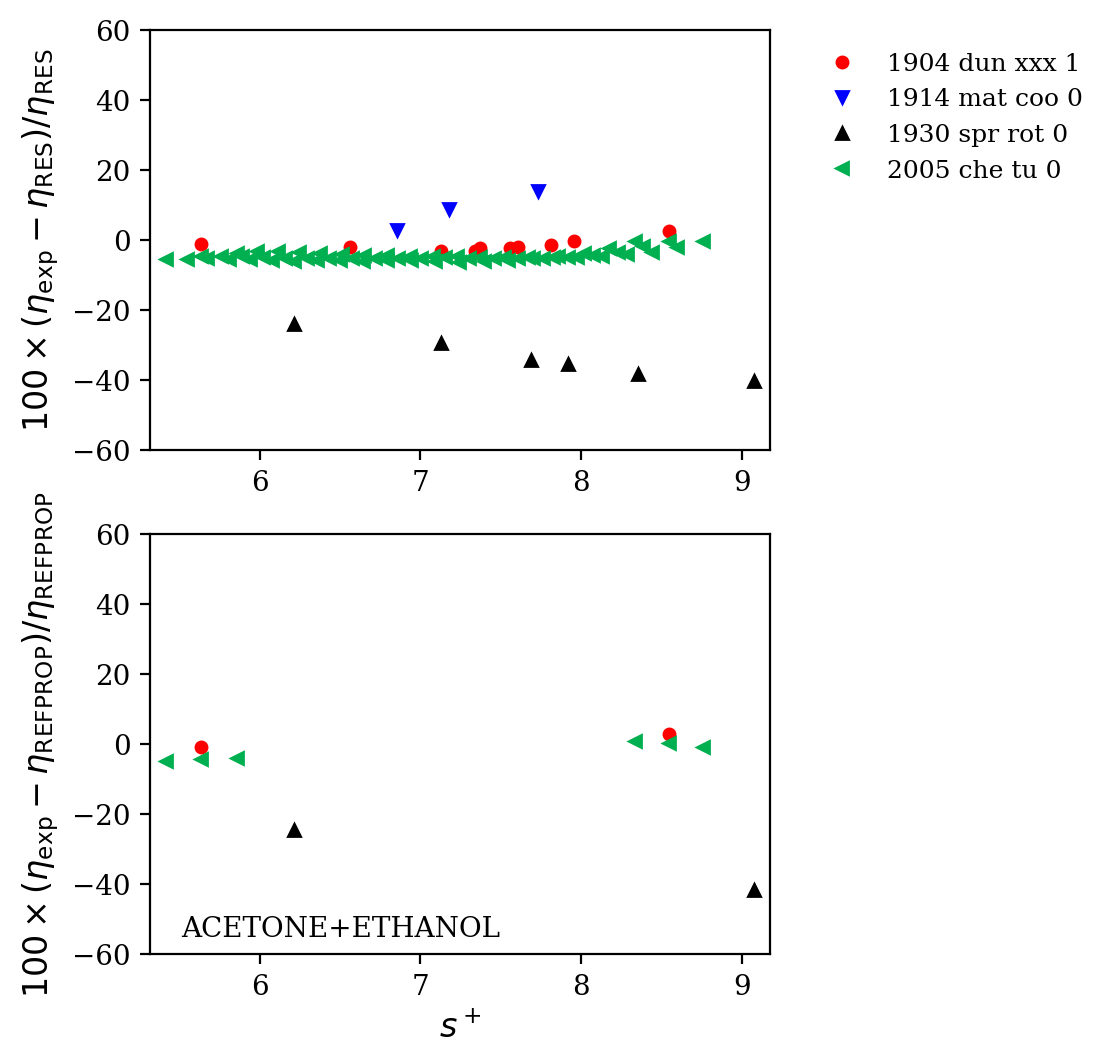

Supplement: Supplementary file 1 — je4c00451_si_001.zip [file je4c00451_si_001.zip › supporting_information/mix_dev_exp_res_ecs/ACETONE+ETHANOL.png]

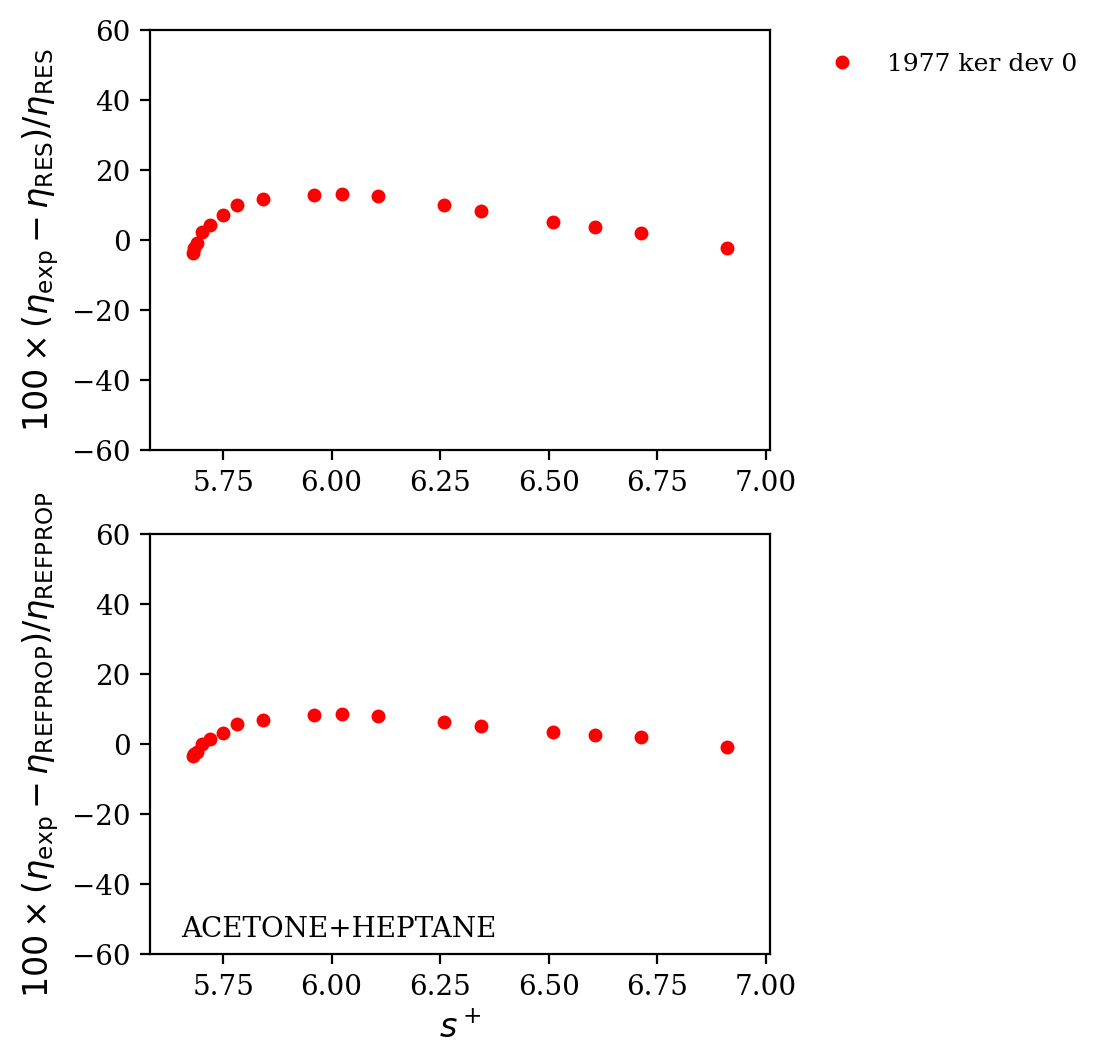

Supplement: Supplementary file 1 — je4c00451_si_001.zip [file je4c00451_si_001.zip › supporting_information/mix_dev_exp_res_ecs/ACETONE+HEPTANE.png]

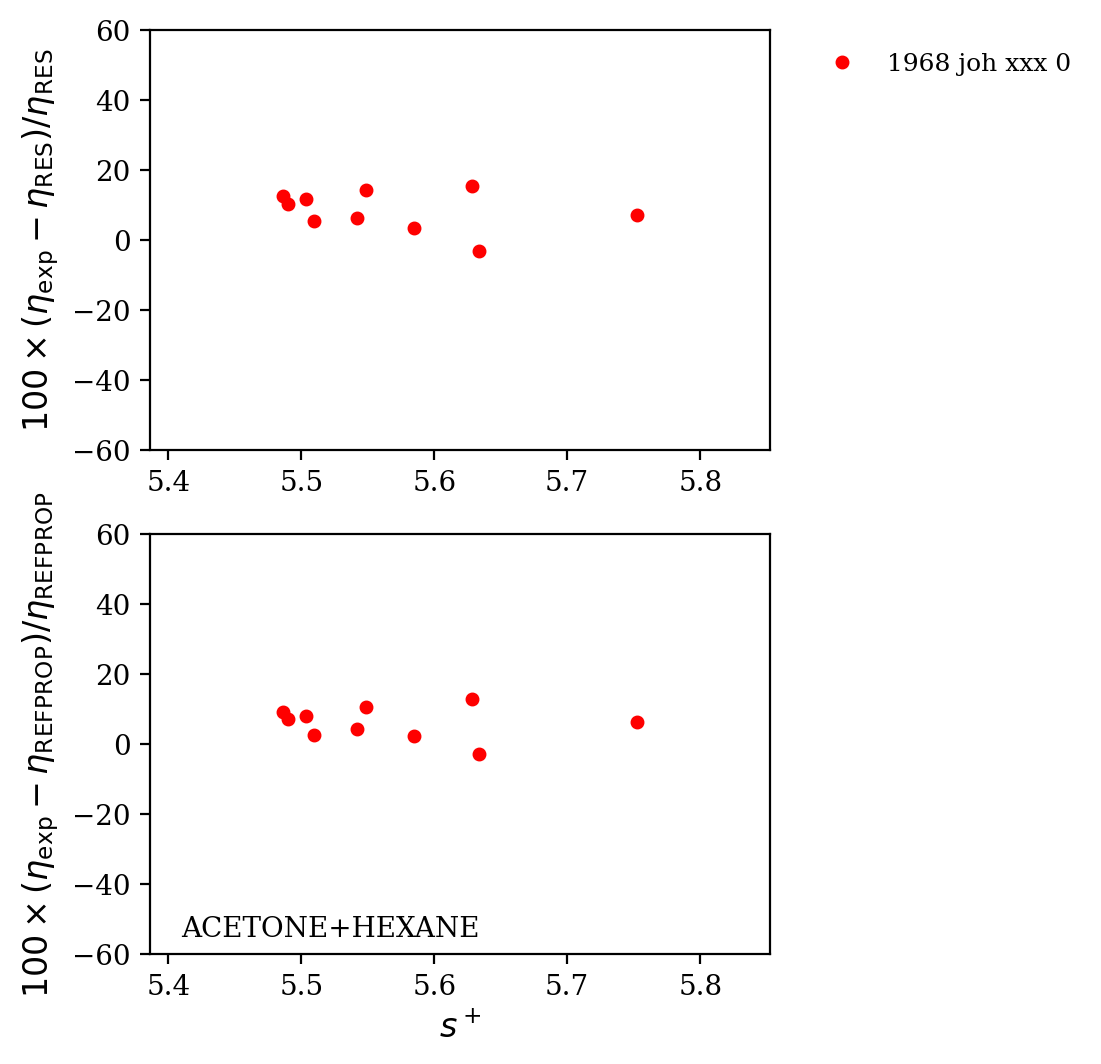

Supplement: Supplementary file 1 — je4c00451_si_001.zip [file je4c00451_si_001.zip › supporting_information/mix_dev_exp_res_ecs/ACETONE+HEXANE.png]

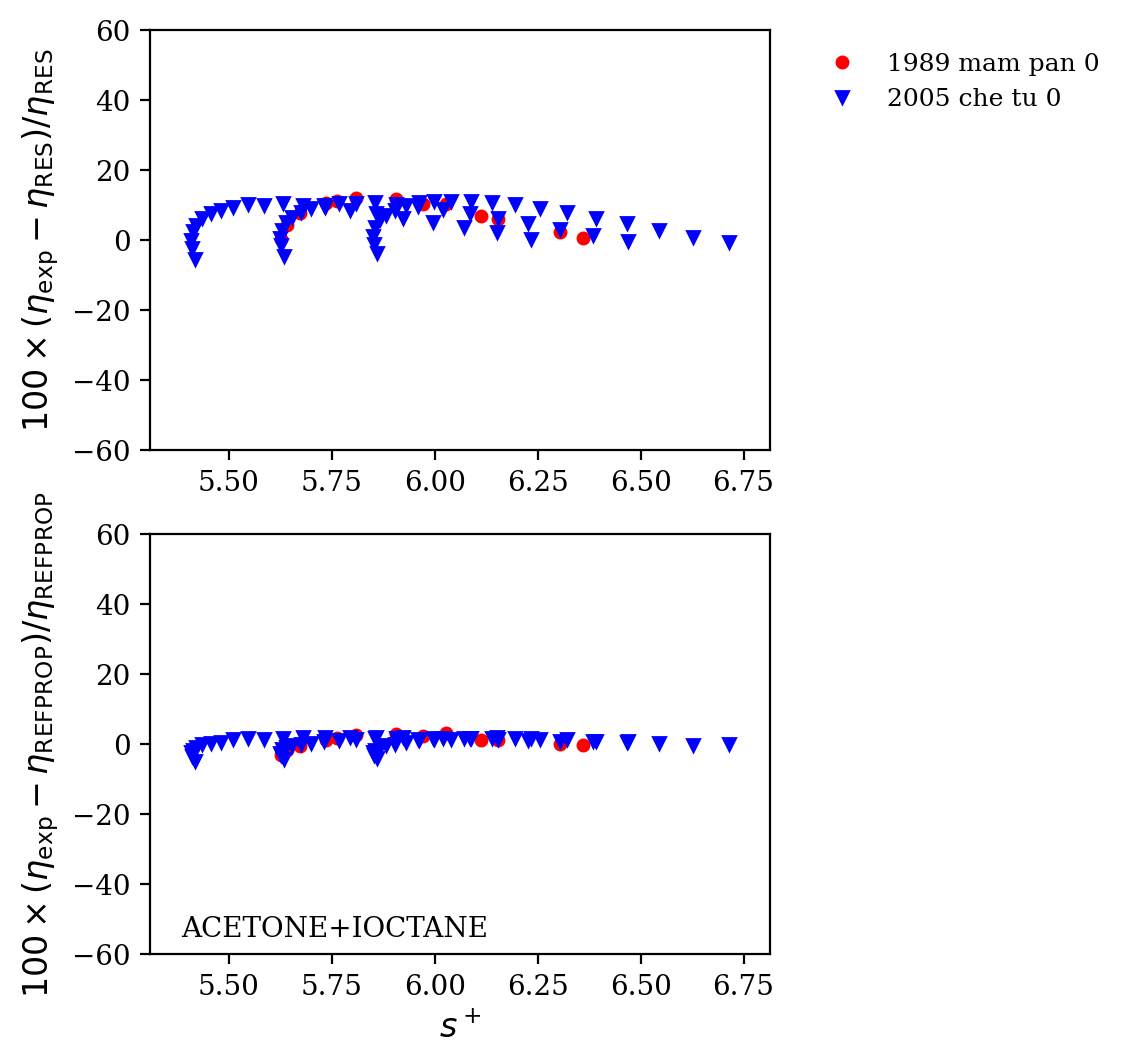

Supplement: Supplementary file 1 — je4c00451_si_001.zip [file je4c00451_si_001.zip › supporting_information/mix_dev_exp_res_ecs/ACETONE+IOCTANE.png]

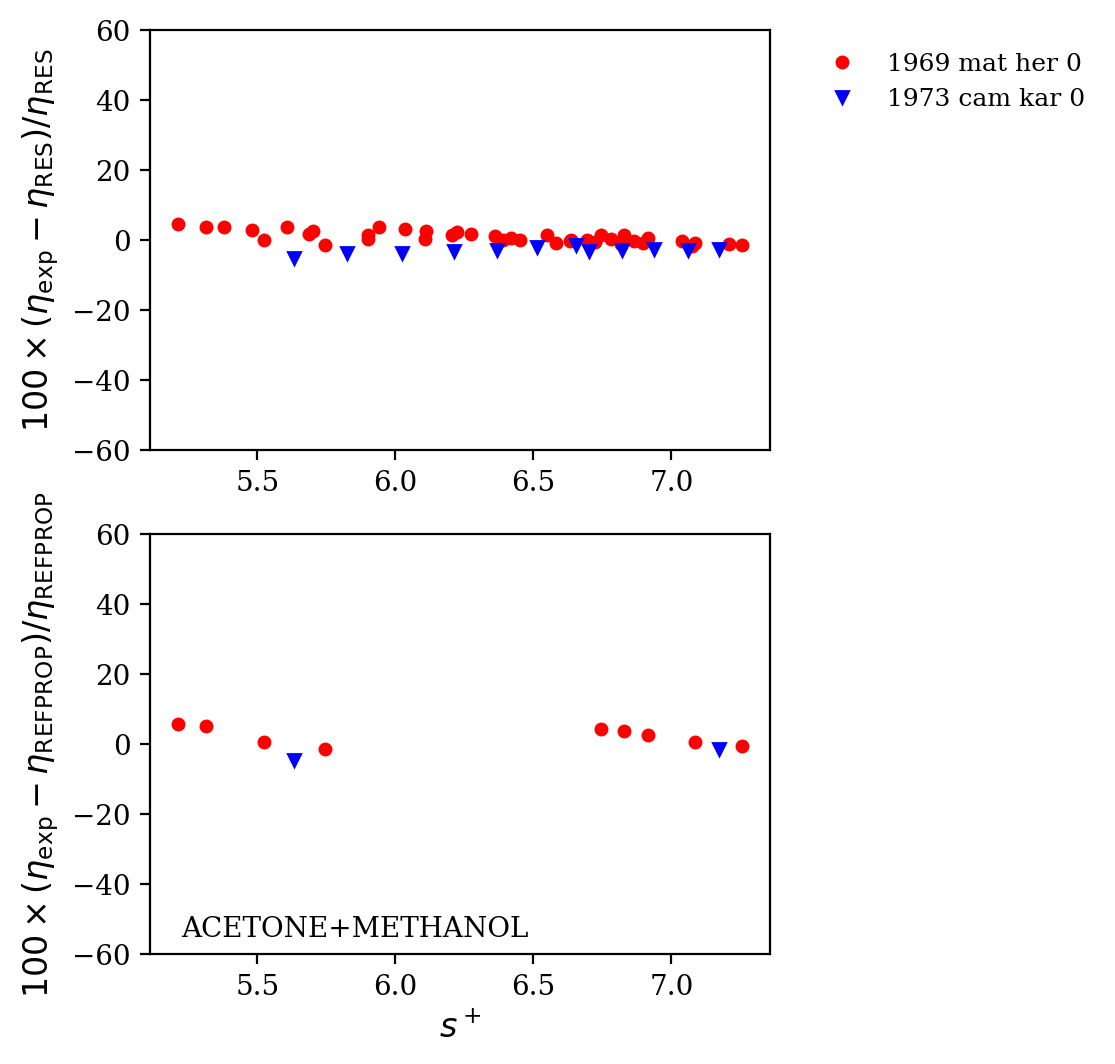

Supplement: Supplementary file 1 — je4c00451_si_001.zip [file je4c00451_si_001.zip › supporting_information/mix_dev_exp_res_ecs/ACETONE+METHANOL.png]

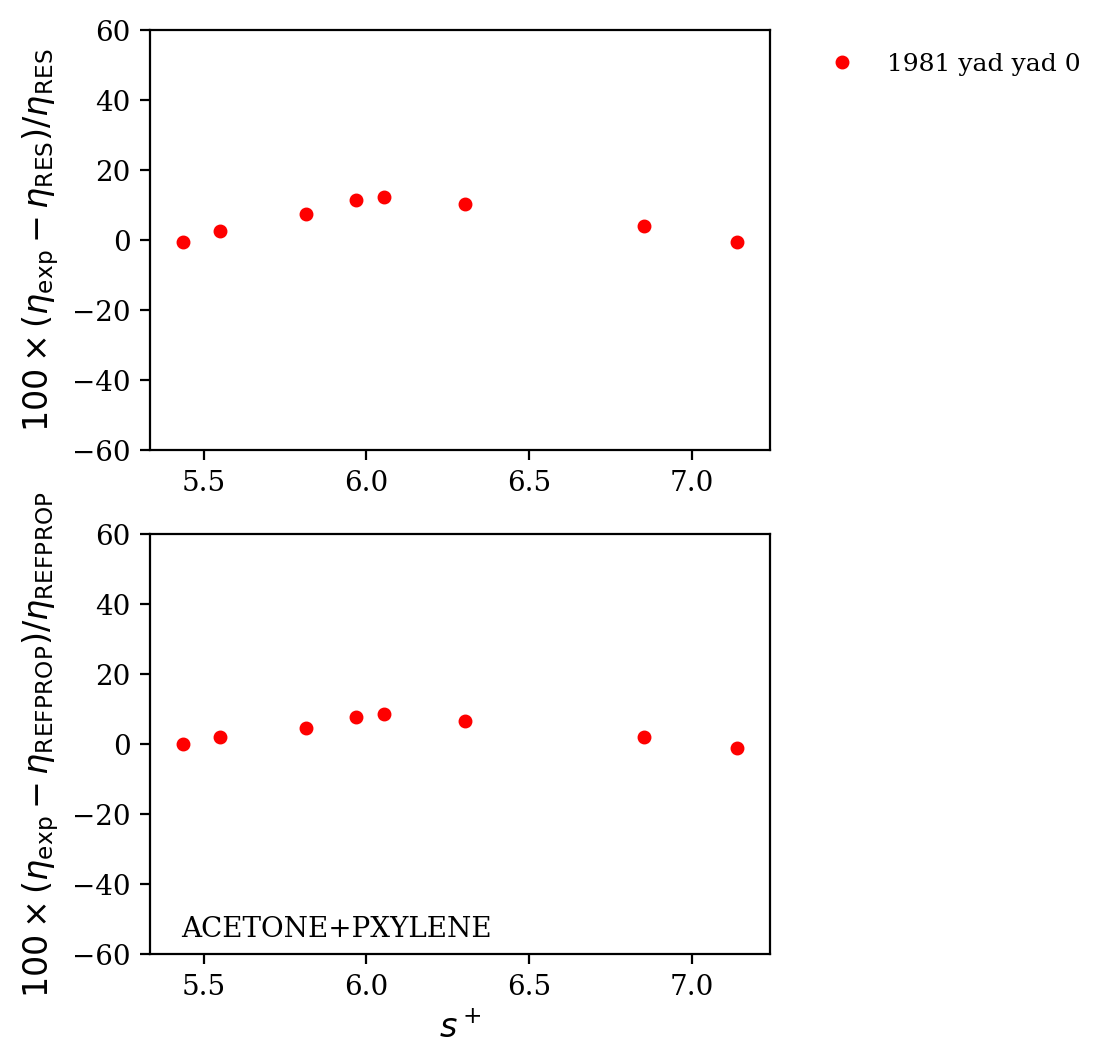

Supplement: Supplementary file 1 — je4c00451_si_001.zip [file je4c00451_si_001.zip › supporting_information/mix_dev_exp_res_ecs/ACETONE+PXYLENE.png]

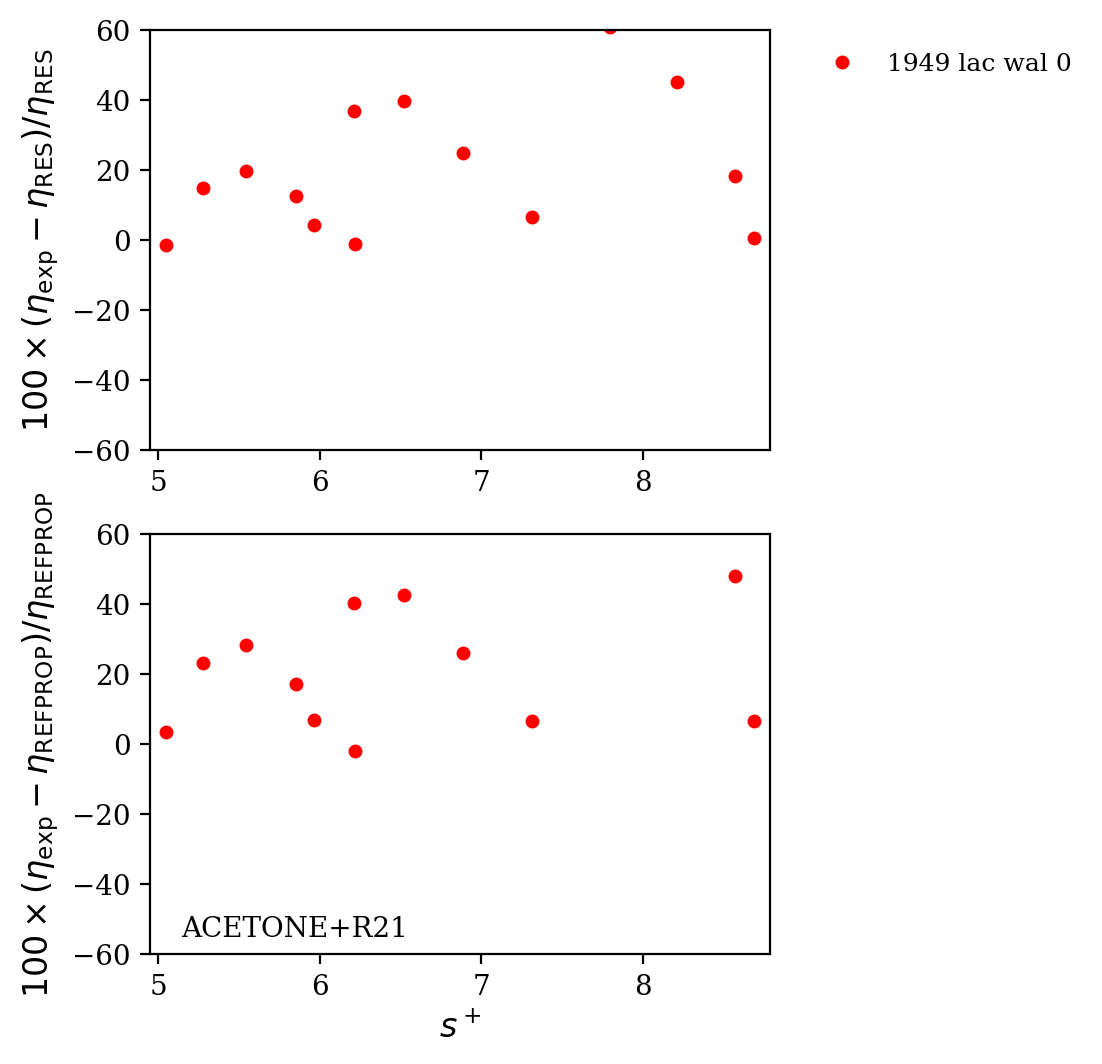

Supplement: Supplementary file 1 — je4c00451_si_001.zip [file je4c00451_si_001.zip › supporting_information/mix_dev_exp_res_ecs/ACETONE+R21.png]

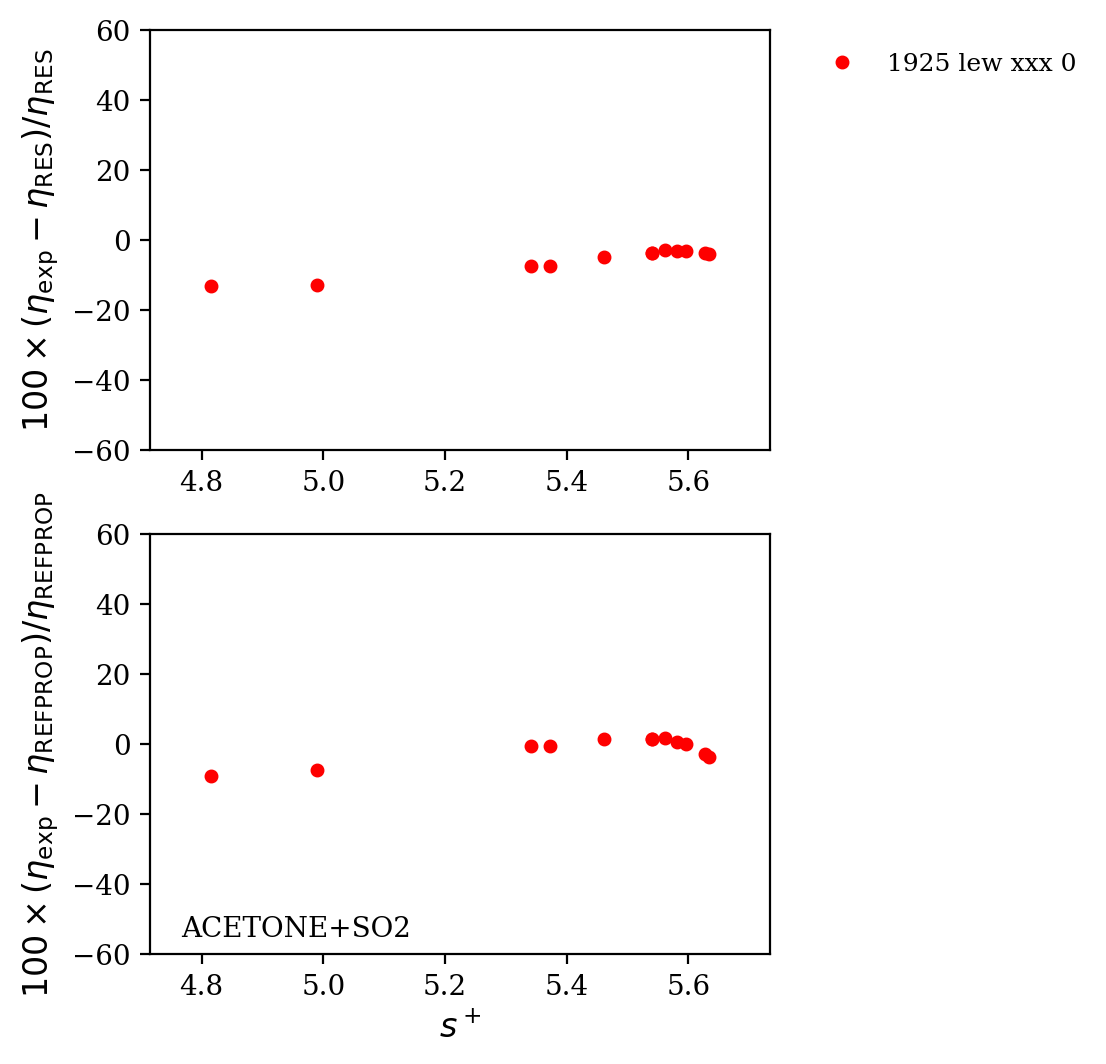

Supplement: Supplementary file 1 — je4c00451_si_001.zip [file je4c00451_si_001.zip › supporting_information/mix_dev_exp_res_ecs/ACETONE+SO2.png]

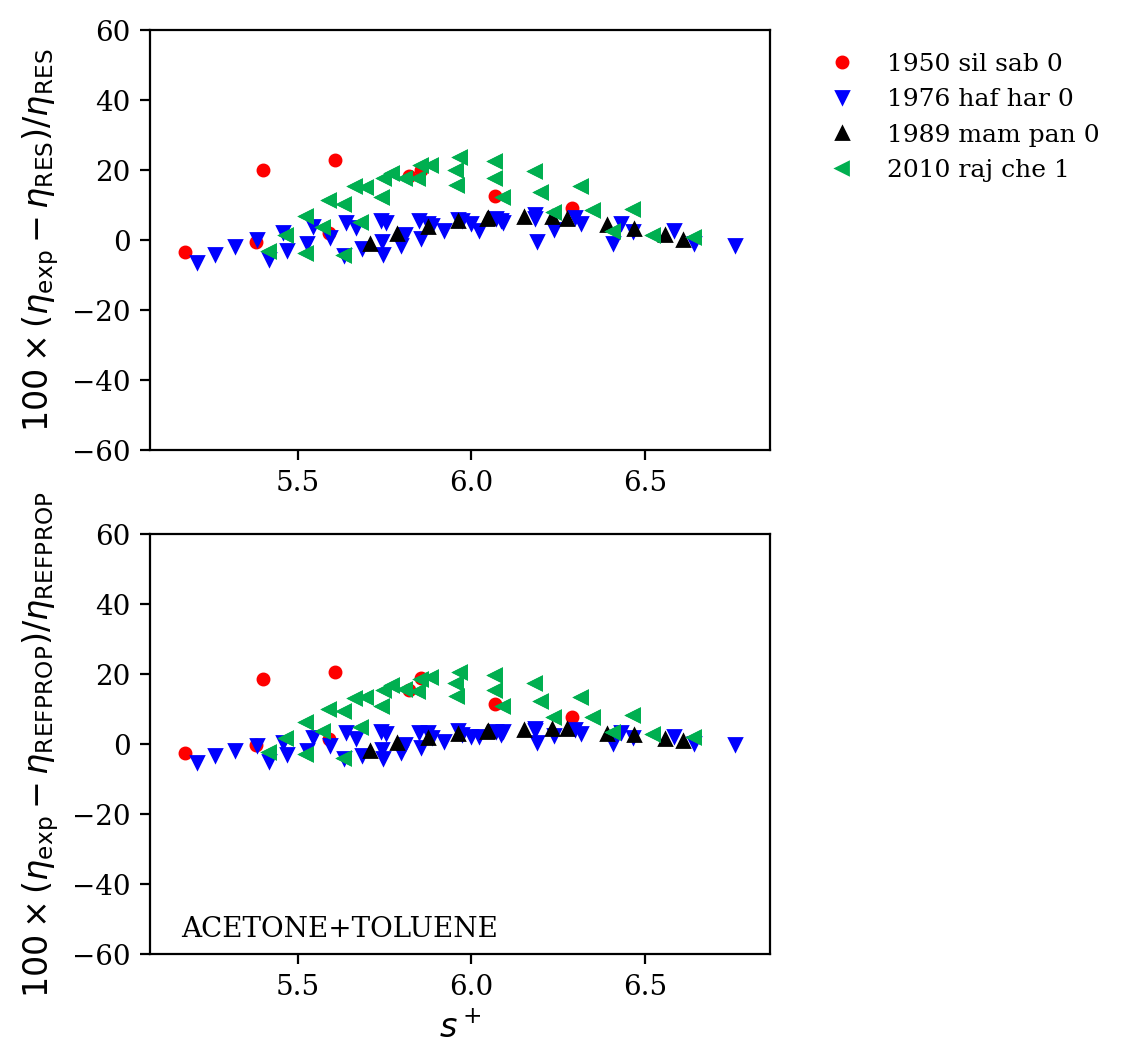

Supplement: Supplementary file 1 — je4c00451_si_001.zip [file je4c00451_si_001.zip › supporting_information/mix_dev_exp_res_ecs/ACETONE+TOLUENE.png]

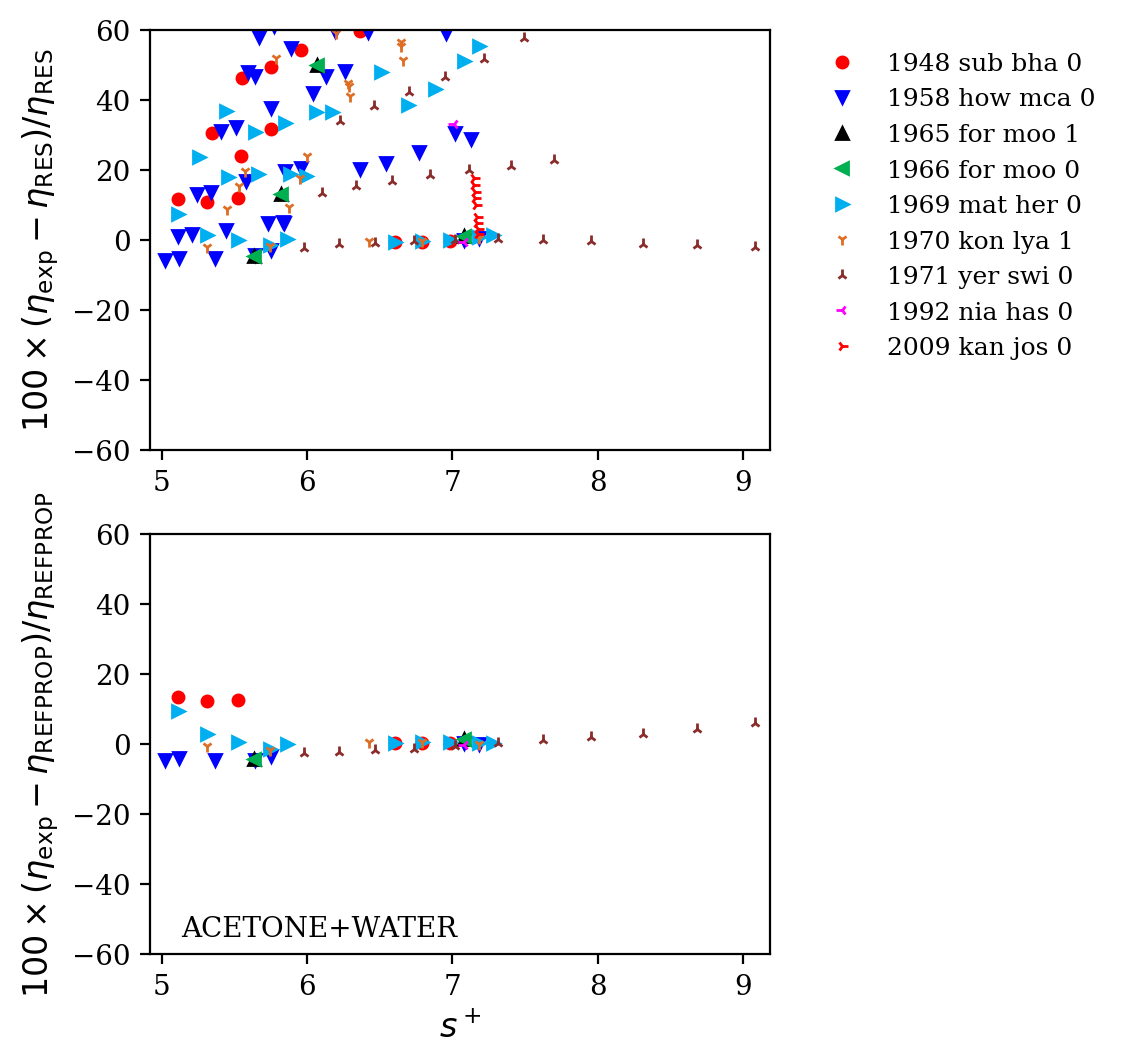

Supplement: Supplementary file 1 — je4c00451_si_001.zip [file je4c00451_si_001.zip › supporting_information/mix_dev_exp_res_ecs/ACETONE+WATER.png]

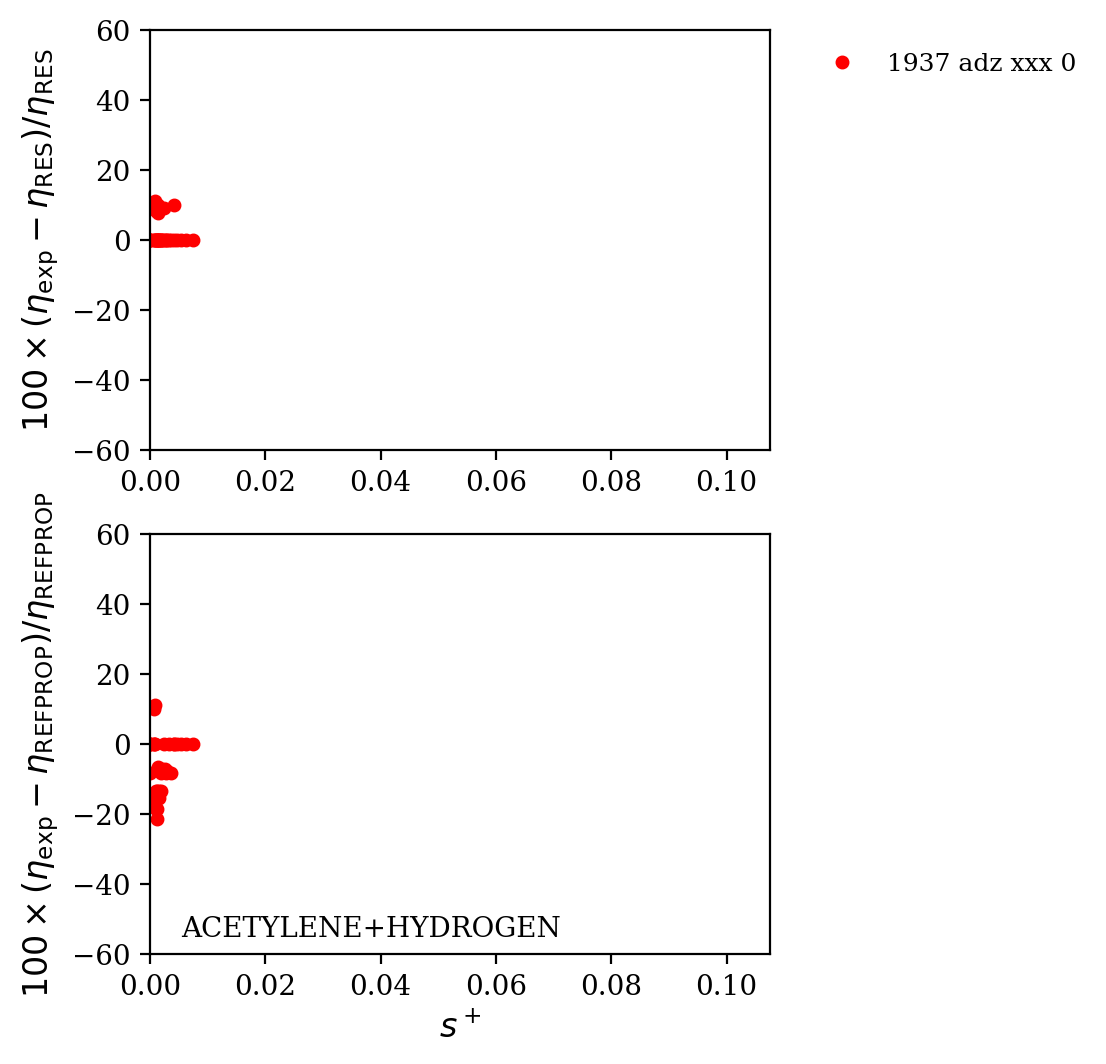

Supplement: Supplementary file 1 — je4c00451_si_001.zip [file je4c00451_si_001.zip › supporting_information/mix_dev_exp_res_ecs/ACETYLENE+HYDROGEN.png]

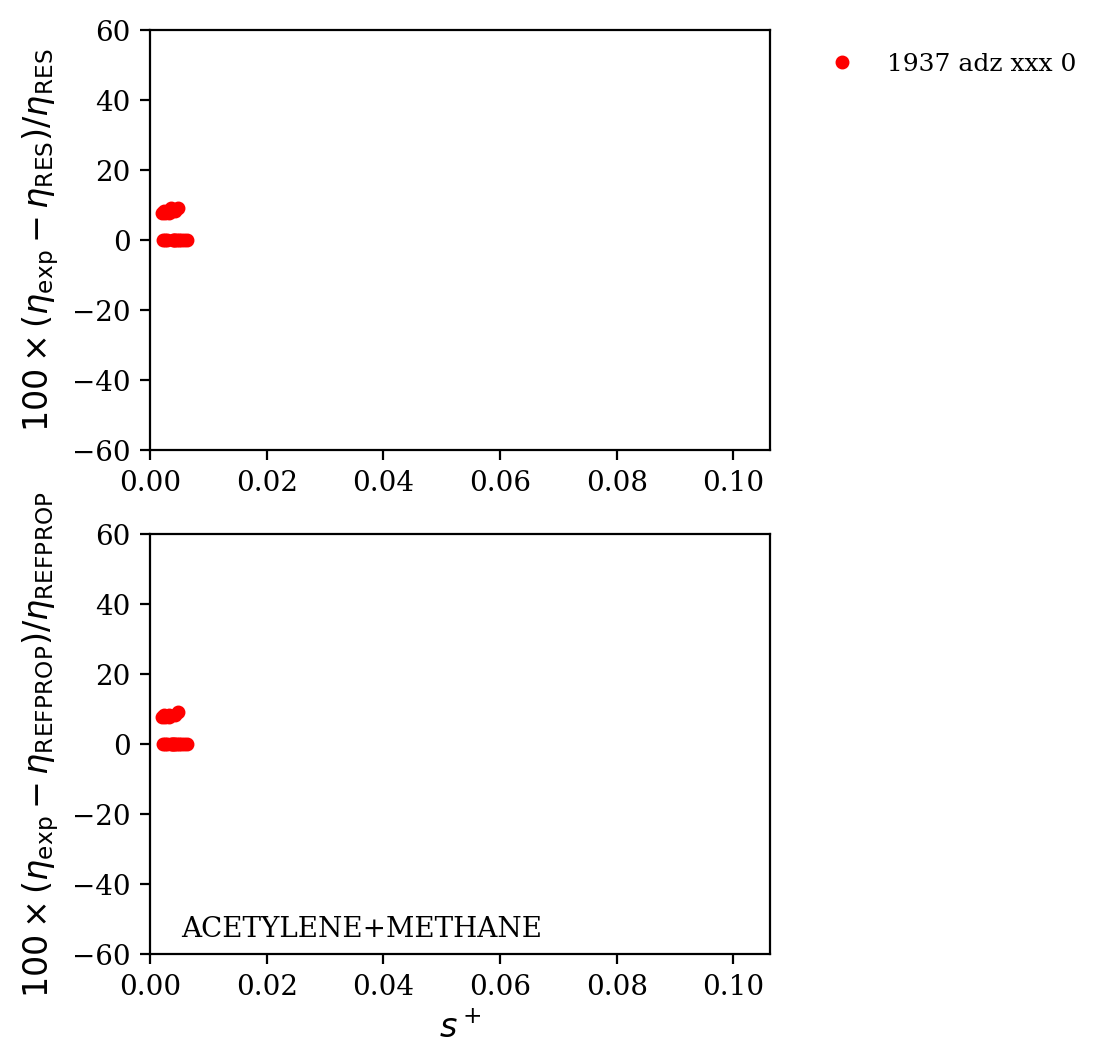

Supplement: Supplementary file 1 — je4c00451_si_001.zip [file je4c00451_si_001.zip › supporting_information/mix_dev_exp_res_ecs/ACETYLENE+METHANE.png]

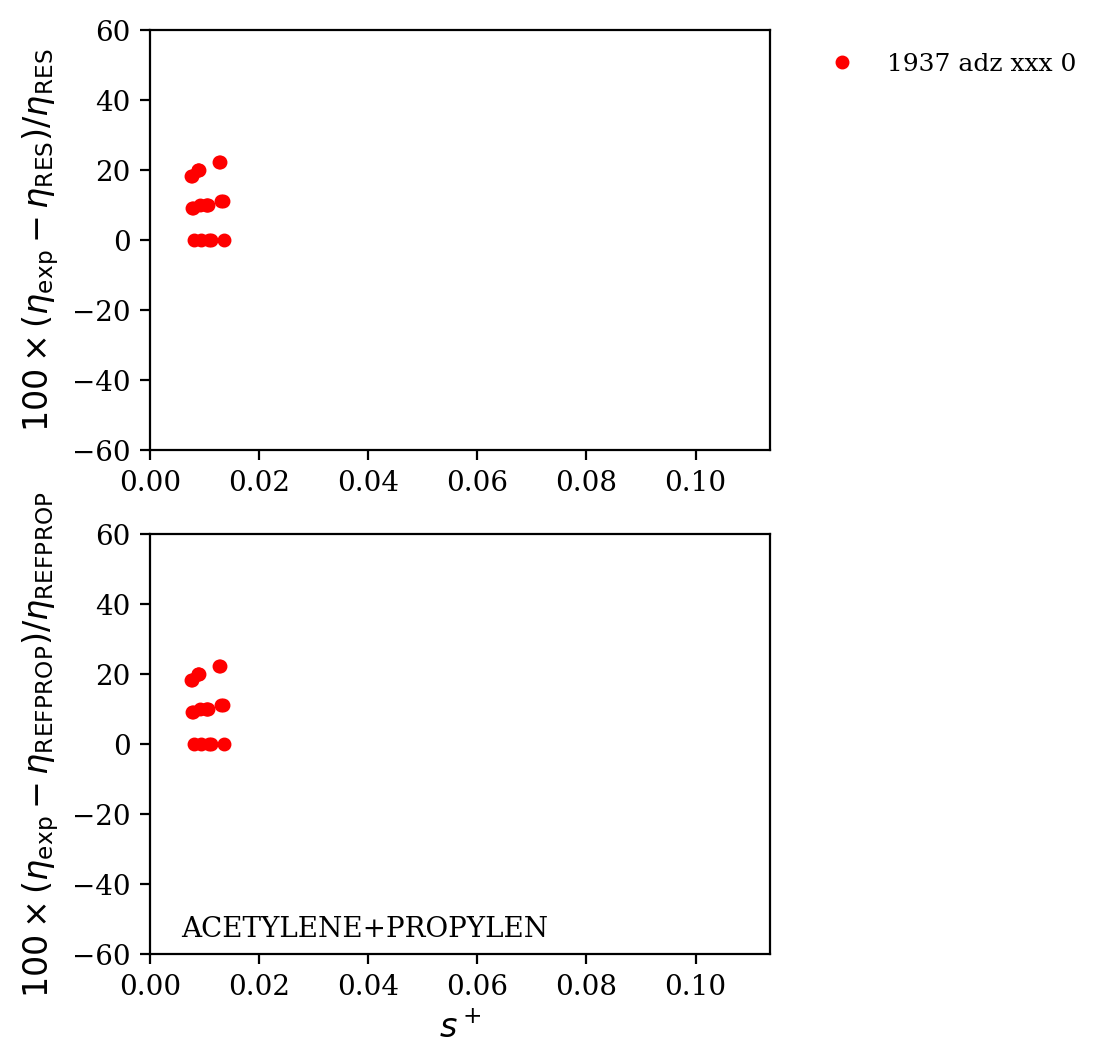

Supplement: Supplementary file 1 — je4c00451_si_001.zip [file je4c00451_si_001.zip › supporting_information/mix_dev_exp_res_ecs/ACETYLENE+PROPYLEN.png]

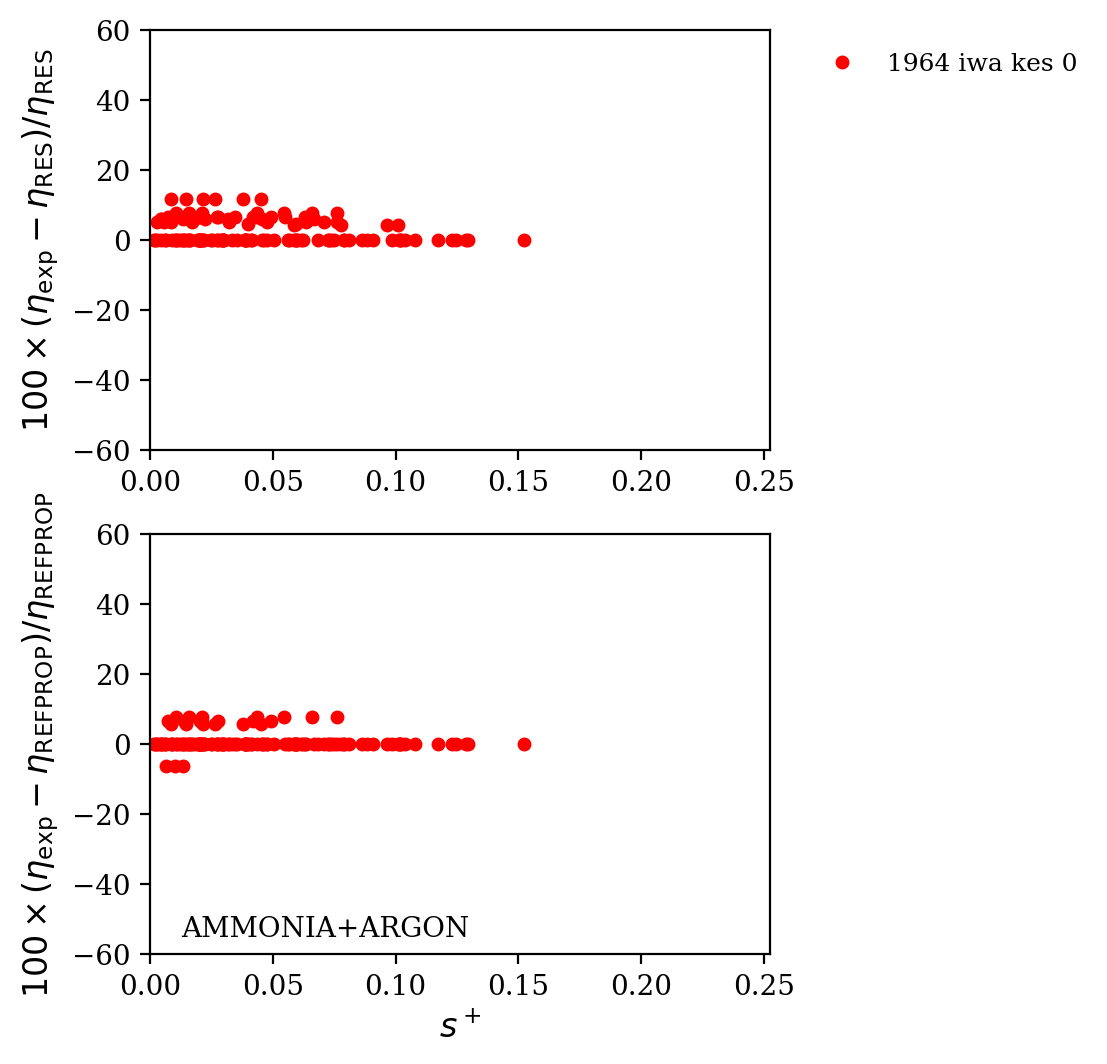

Supplement: Supplementary file 1 — je4c00451_si_001.zip [file je4c00451_si_001.zip › supporting_information/mix_dev_exp_res_ecs/AMMONIA+ARGON.png]

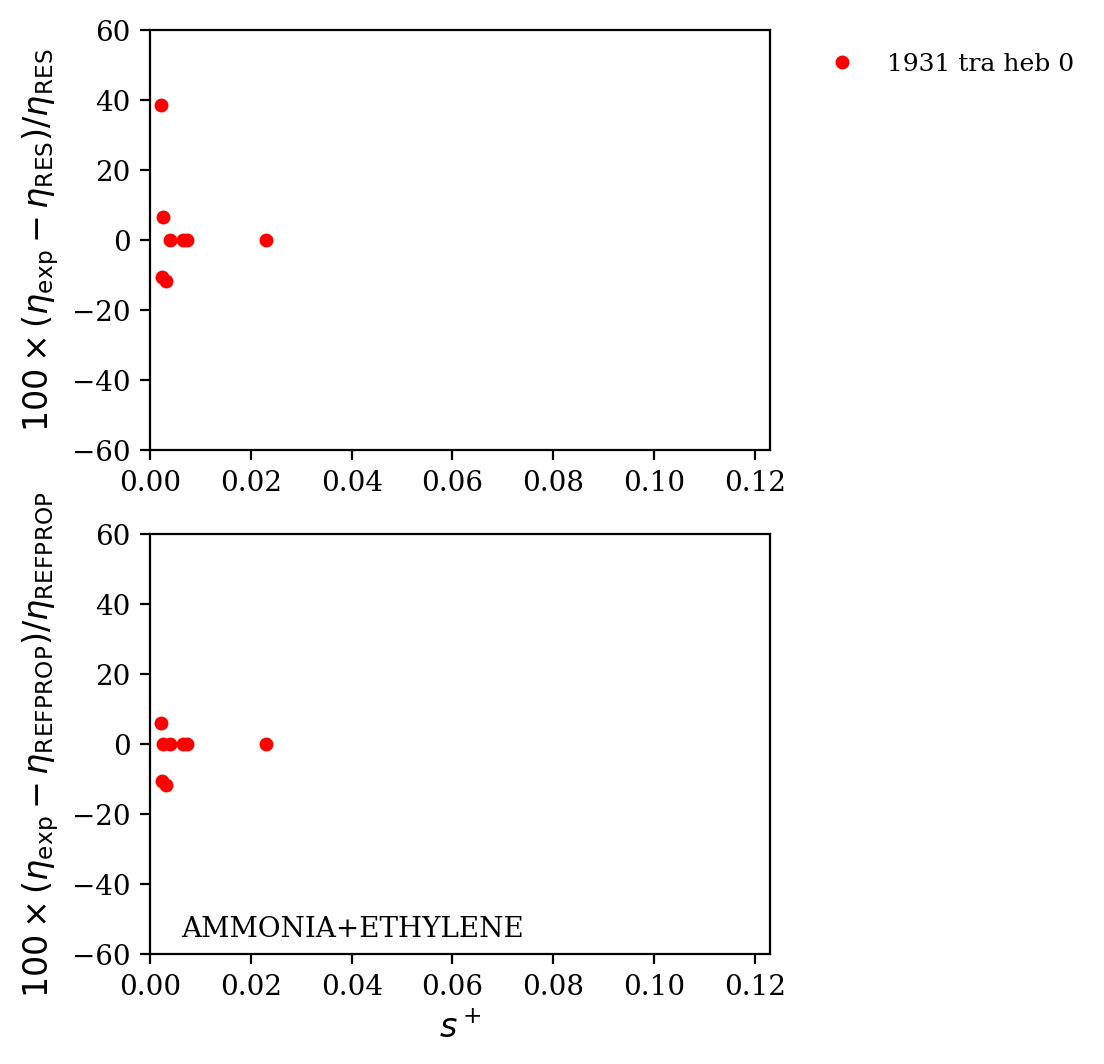

Supplement: Supplementary file 1 — je4c00451_si_001.zip [file je4c00451_si_001.zip › supporting_information/mix_dev_exp_res_ecs/AMMONIA+ETHYLENE.png]

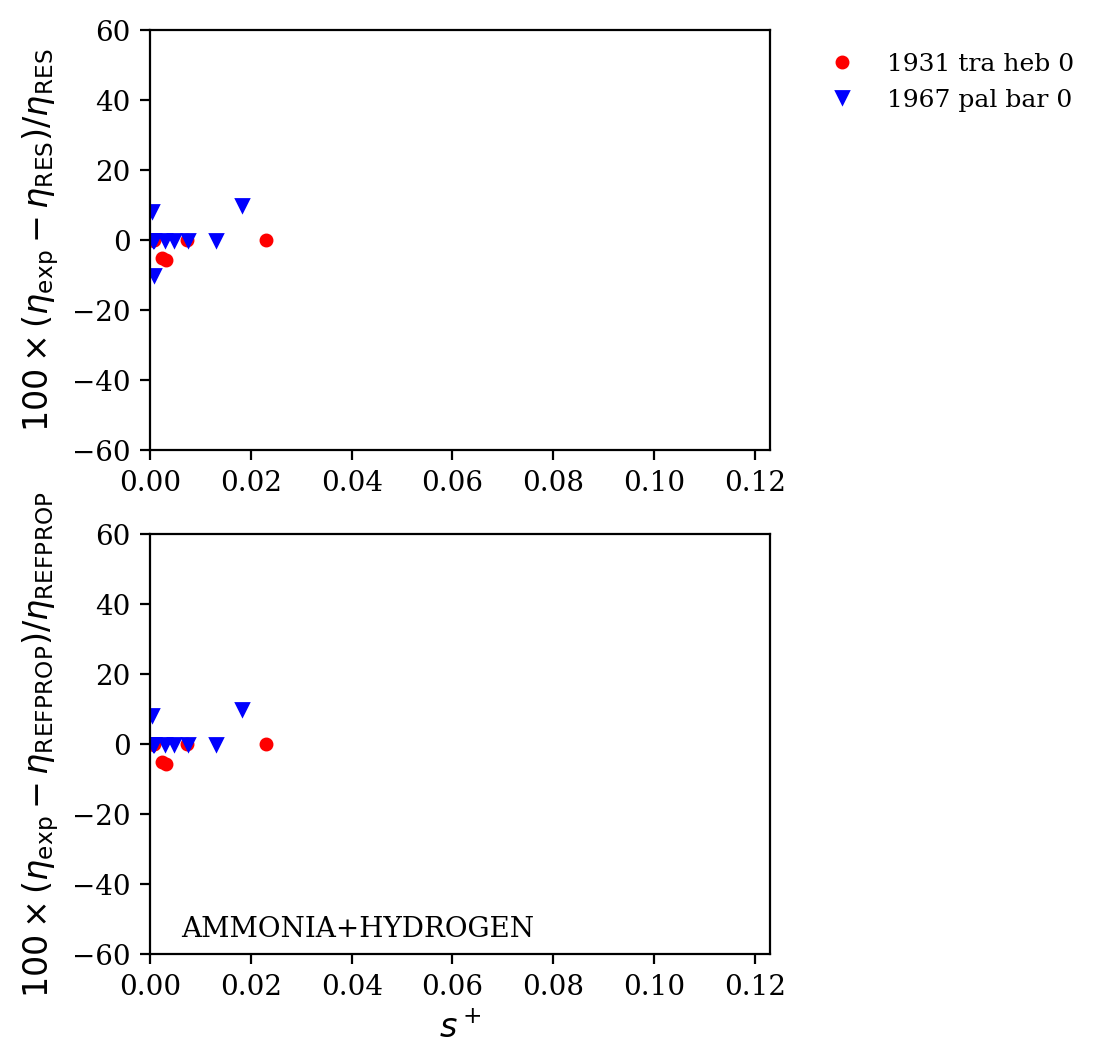

Supplement: Supplementary file 1 — je4c00451_si_001.zip [file je4c00451_si_001.zip › supporting_information/mix_dev_exp_res_ecs/AMMONIA+HYDROGEN.png]

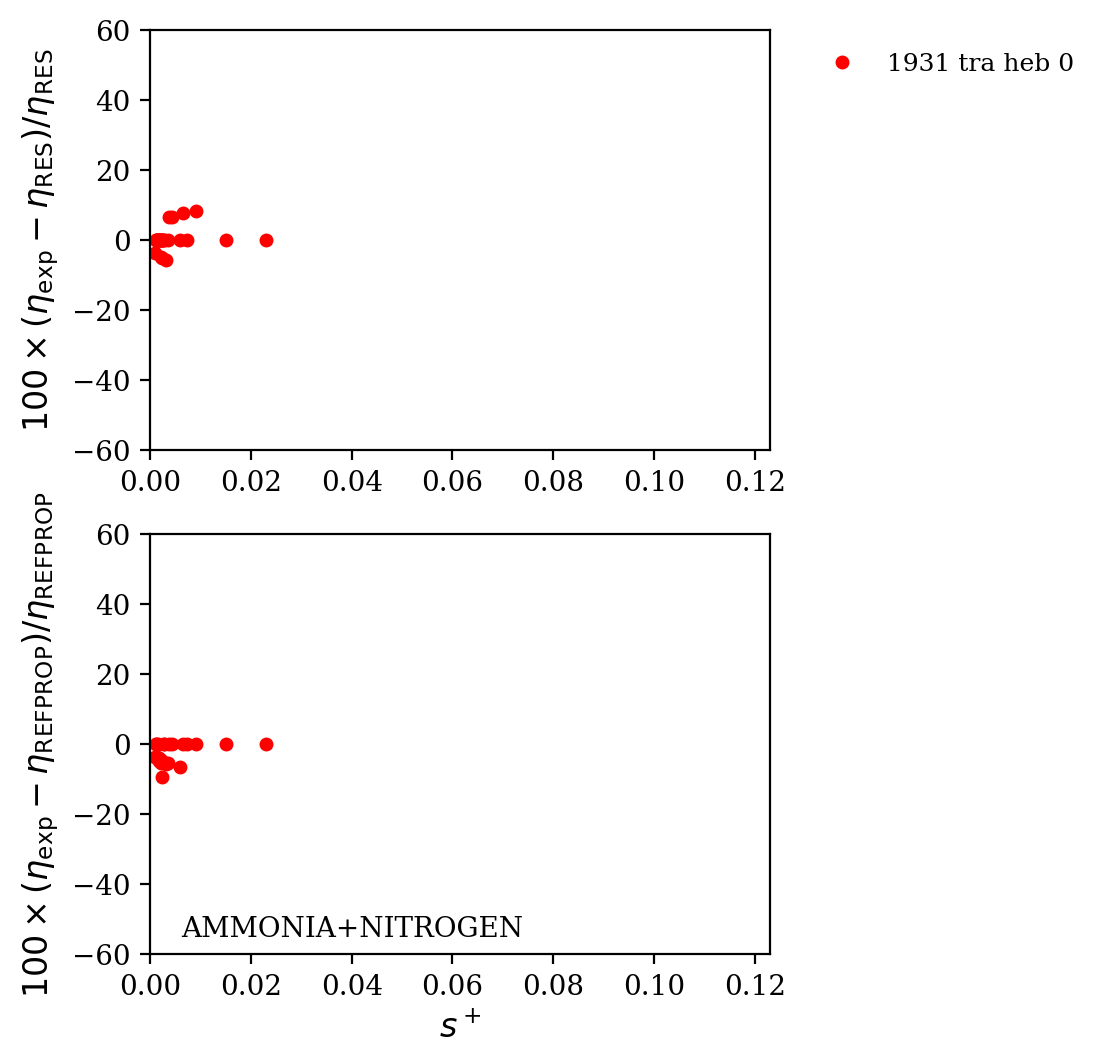

Supplement: Supplementary file 1 — je4c00451_si_001.zip [file je4c00451_si_001.zip › supporting_information/mix_dev_exp_res_ecs/AMMONIA+NITROGEN.png]

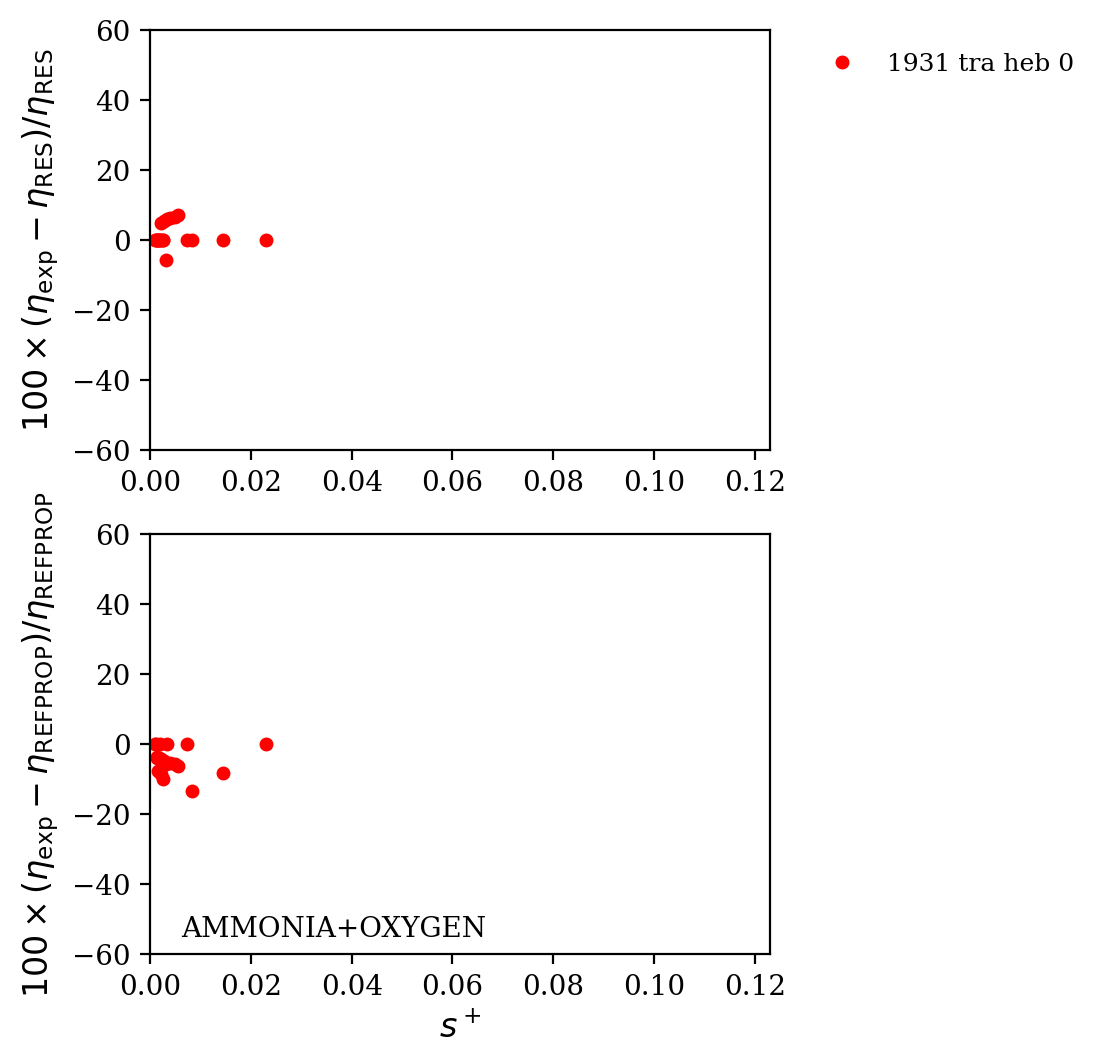

Supplement: Supplementary file 1 — je4c00451_si_001.zip [file je4c00451_si_001.zip › supporting_information/mix_dev_exp_res_ecs/AMMONIA+OXYGEN.png]

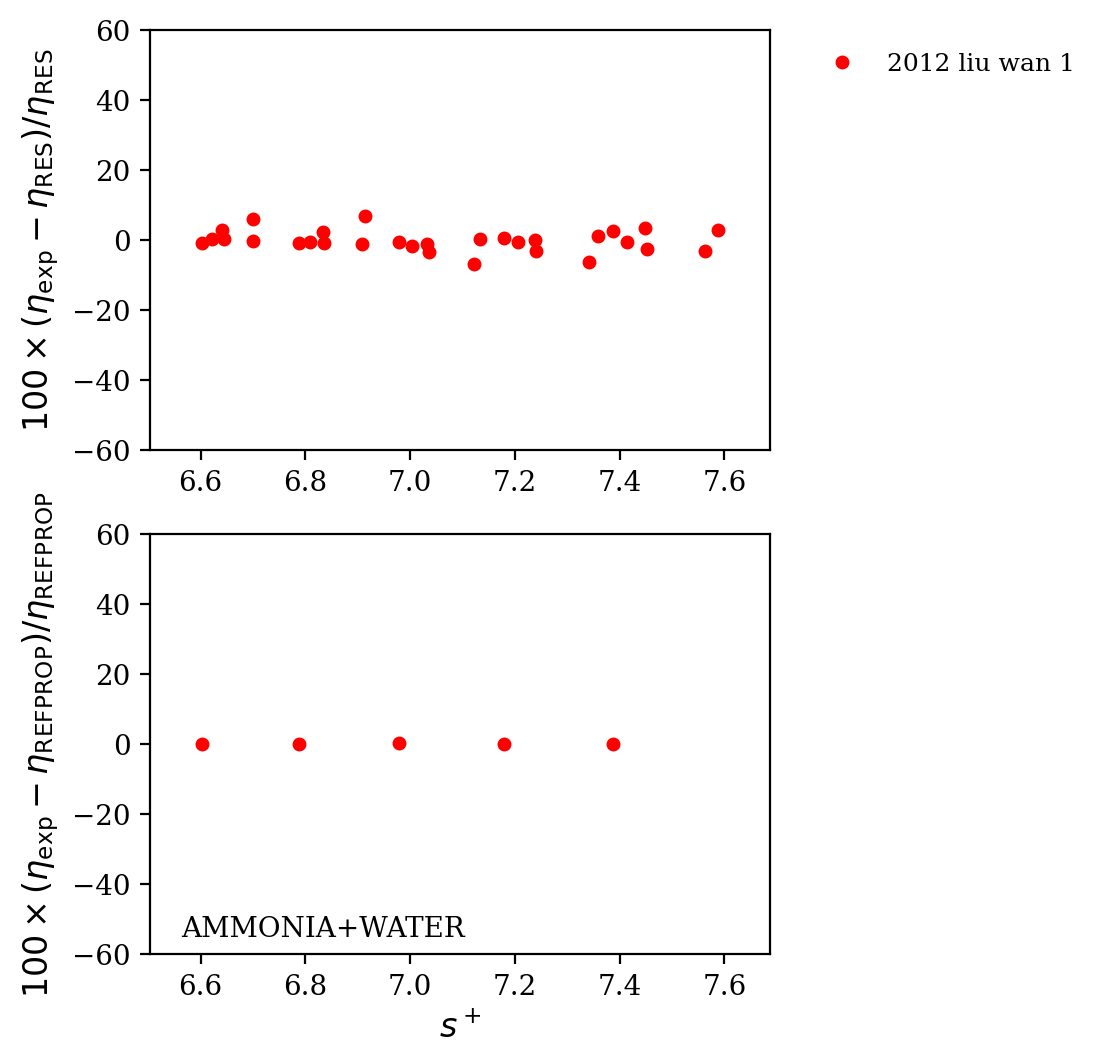

Supplement: Supplementary file 1 — je4c00451_si_001.zip [file je4c00451_si_001.zip › supporting_information/mix_dev_exp_res_ecs/AMMONIA+WATER.png]

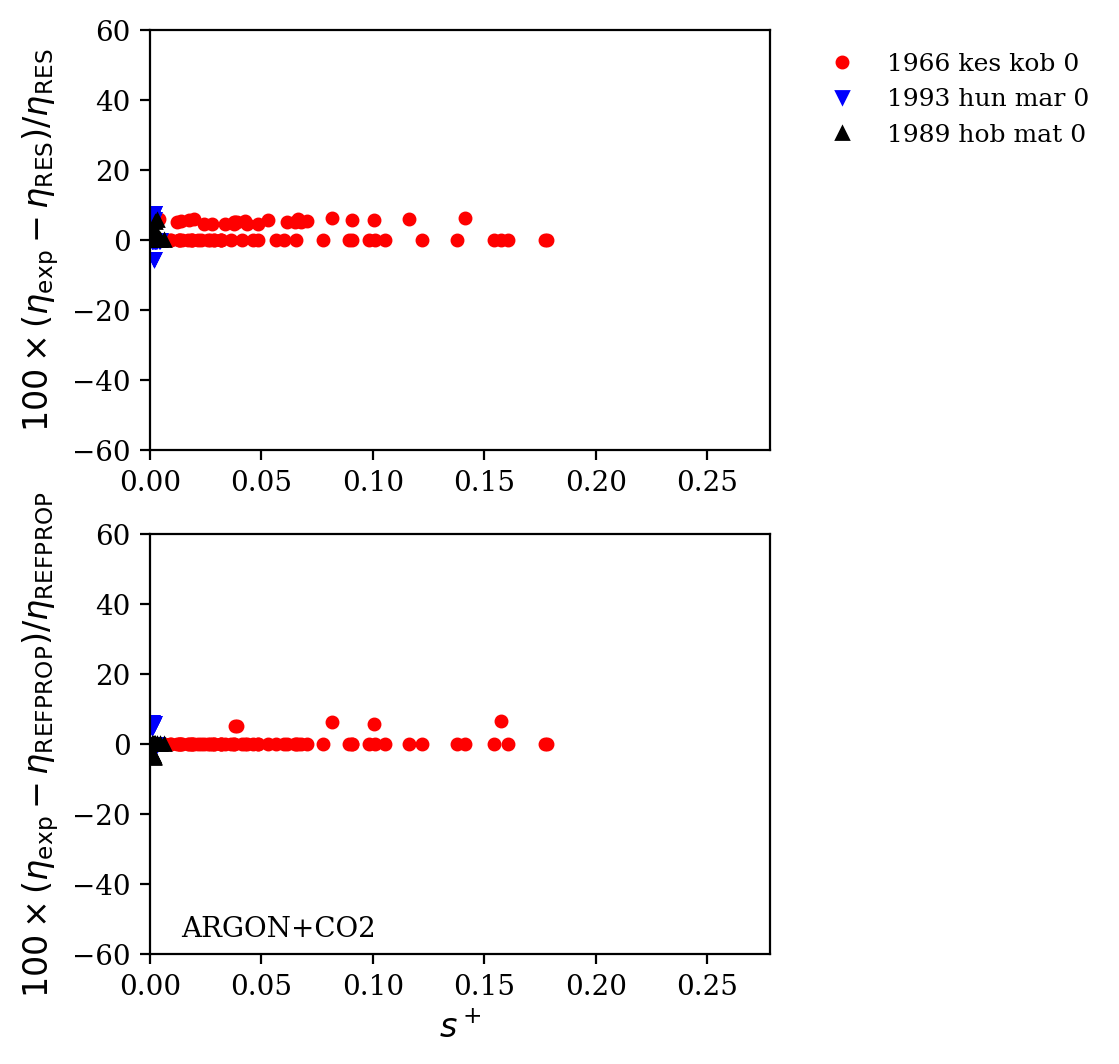

Supplement: Supplementary file 1 — je4c00451_si_001.zip [file je4c00451_si_001.zip › supporting_information/mix_dev_exp_res_ecs/ARGON+CO2.png]

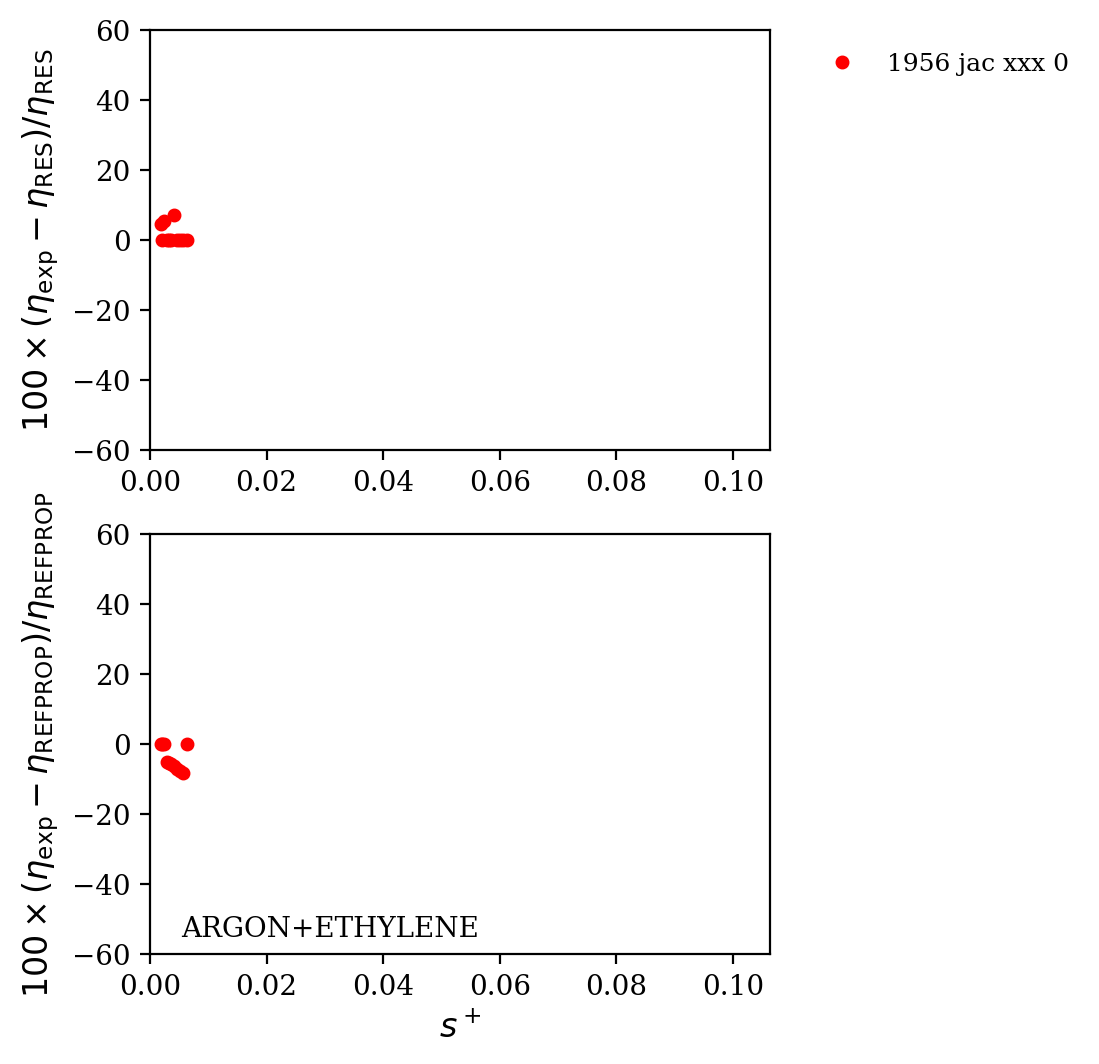

Supplement: Supplementary file 1 — je4c00451_si_001.zip [file je4c00451_si_001.zip › supporting_information/mix_dev_exp_res_ecs/ARGON+ETHYLENE.png]

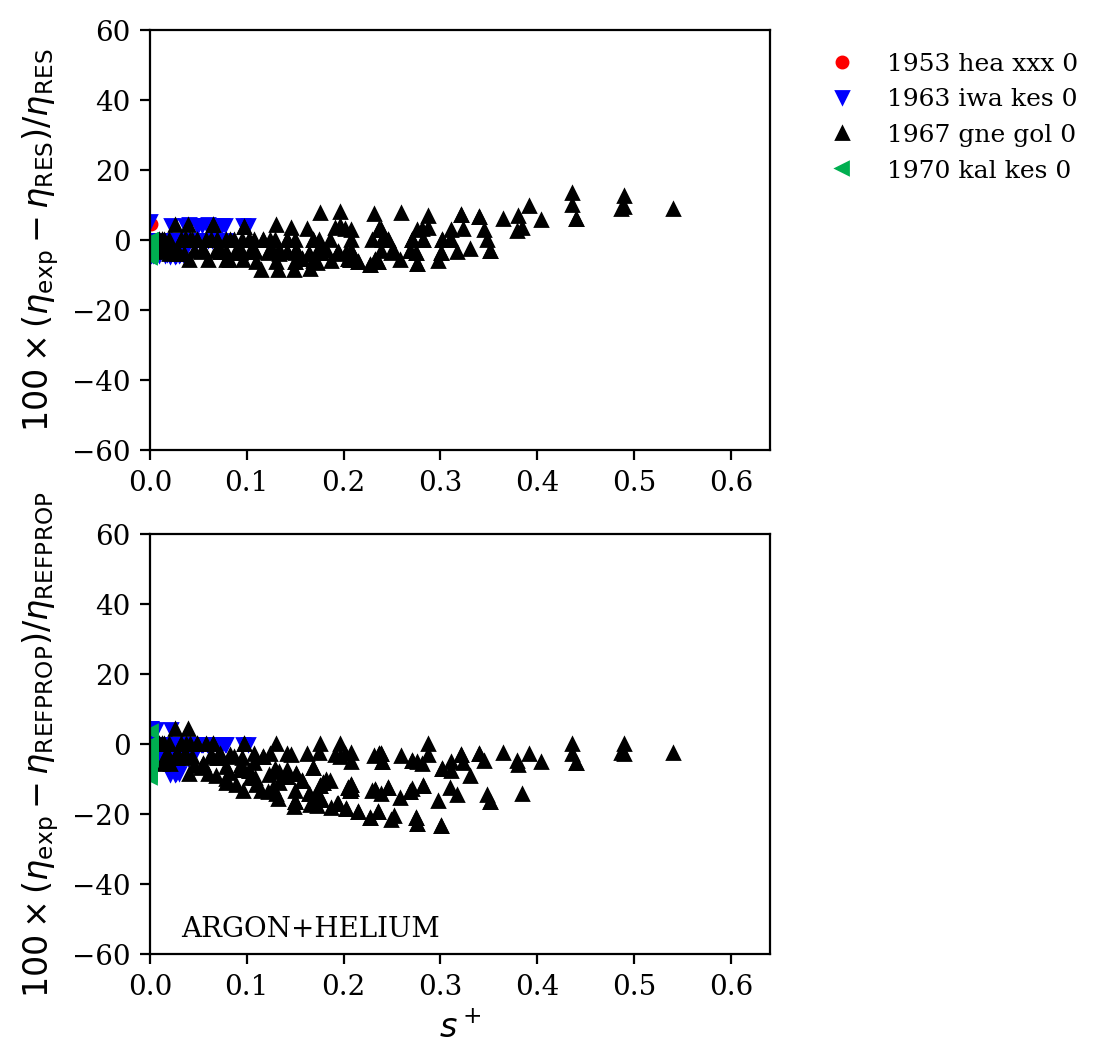

Supplement: Supplementary file 1 — je4c00451_si_001.zip [file je4c00451_si_001.zip › supporting_information/mix_dev_exp_res_ecs/ARGON+HELIUM.png]

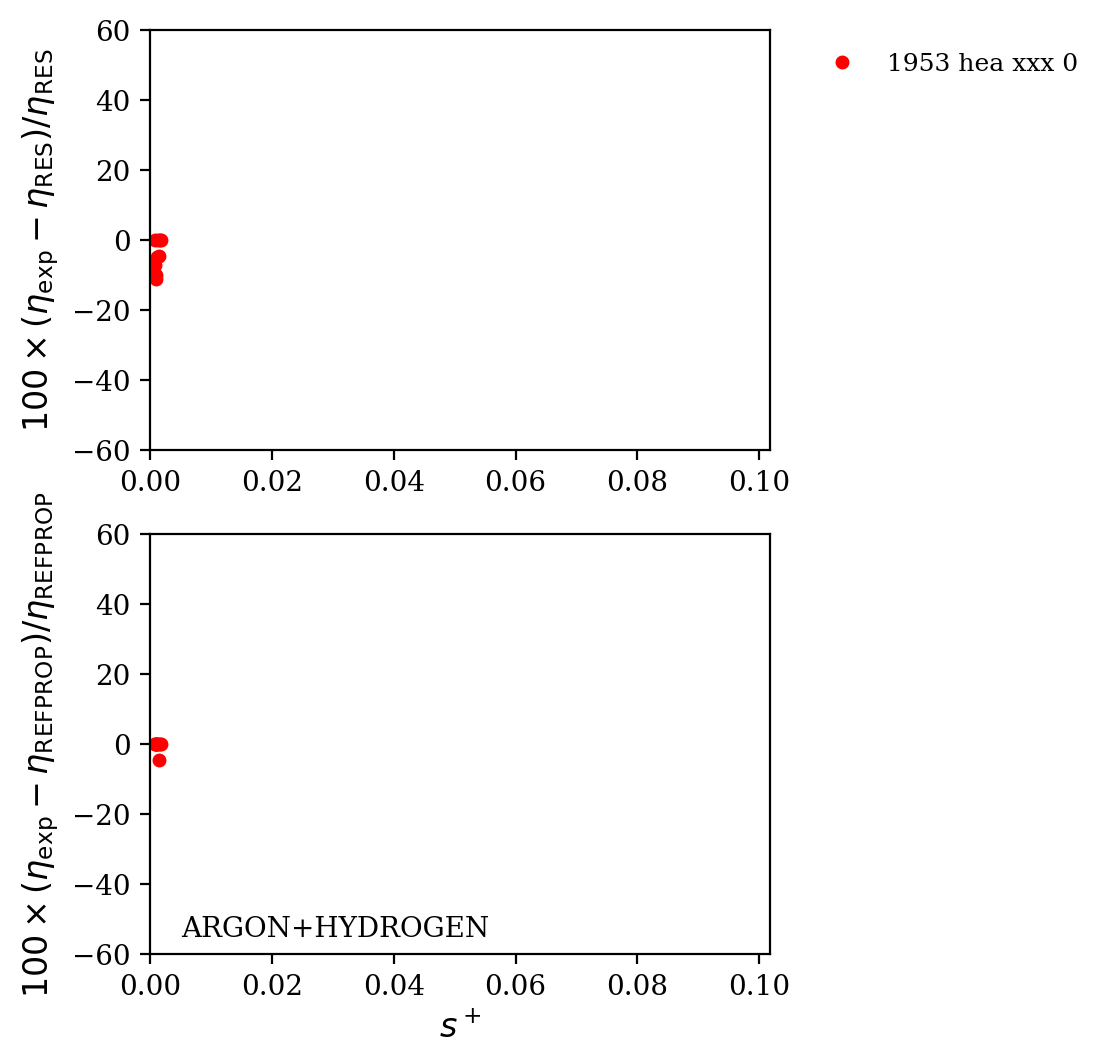

Supplement: Supplementary file 1 — je4c00451_si_001.zip [file je4c00451_si_001.zip › supporting_information/mix_dev_exp_res_ecs/ARGON+HYDROGEN.png]

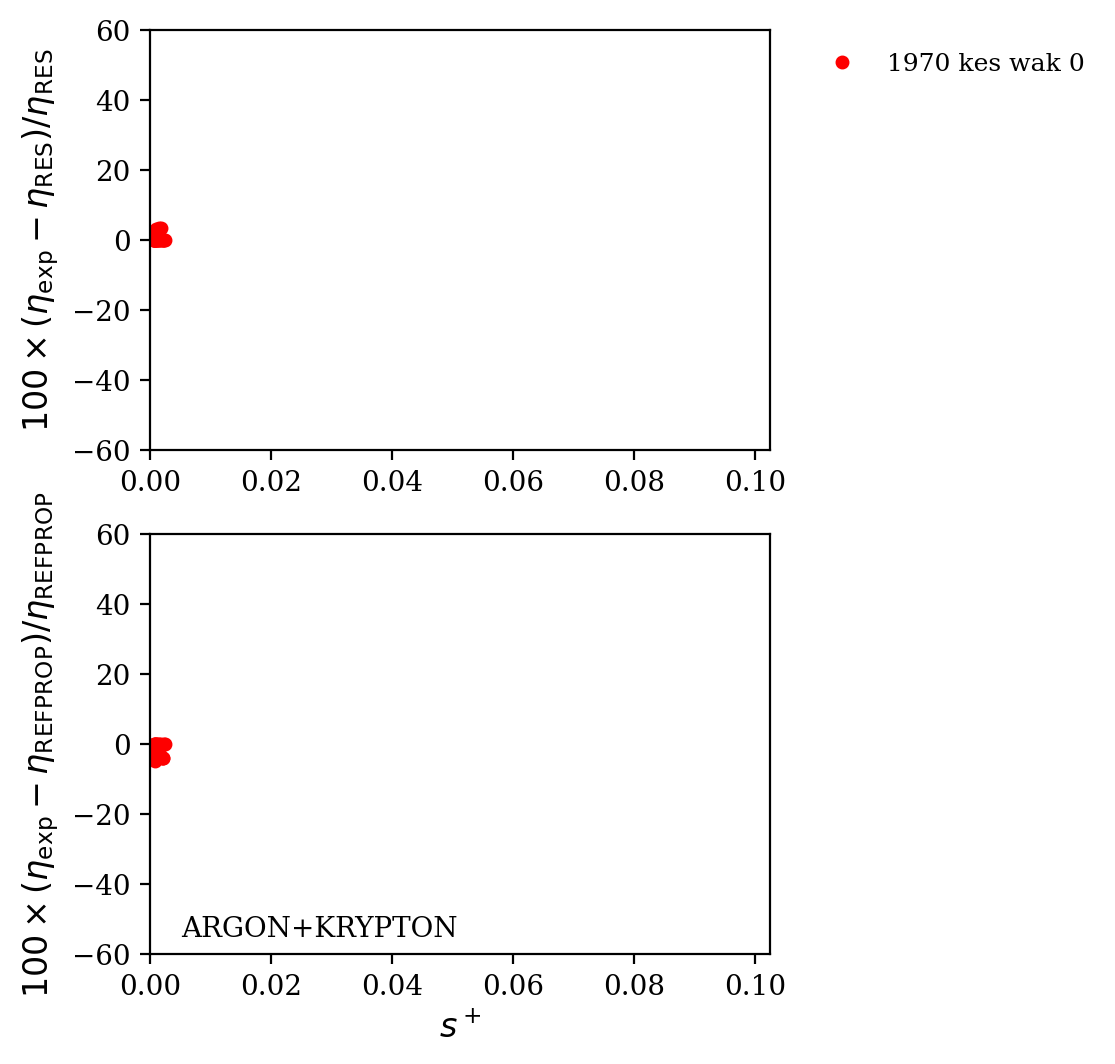

Supplement: Supplementary file 1 — je4c00451_si_001.zip [file je4c00451_si_001.zip › supporting_information/mix_dev_exp_res_ecs/ARGON+KRYPTON.png]

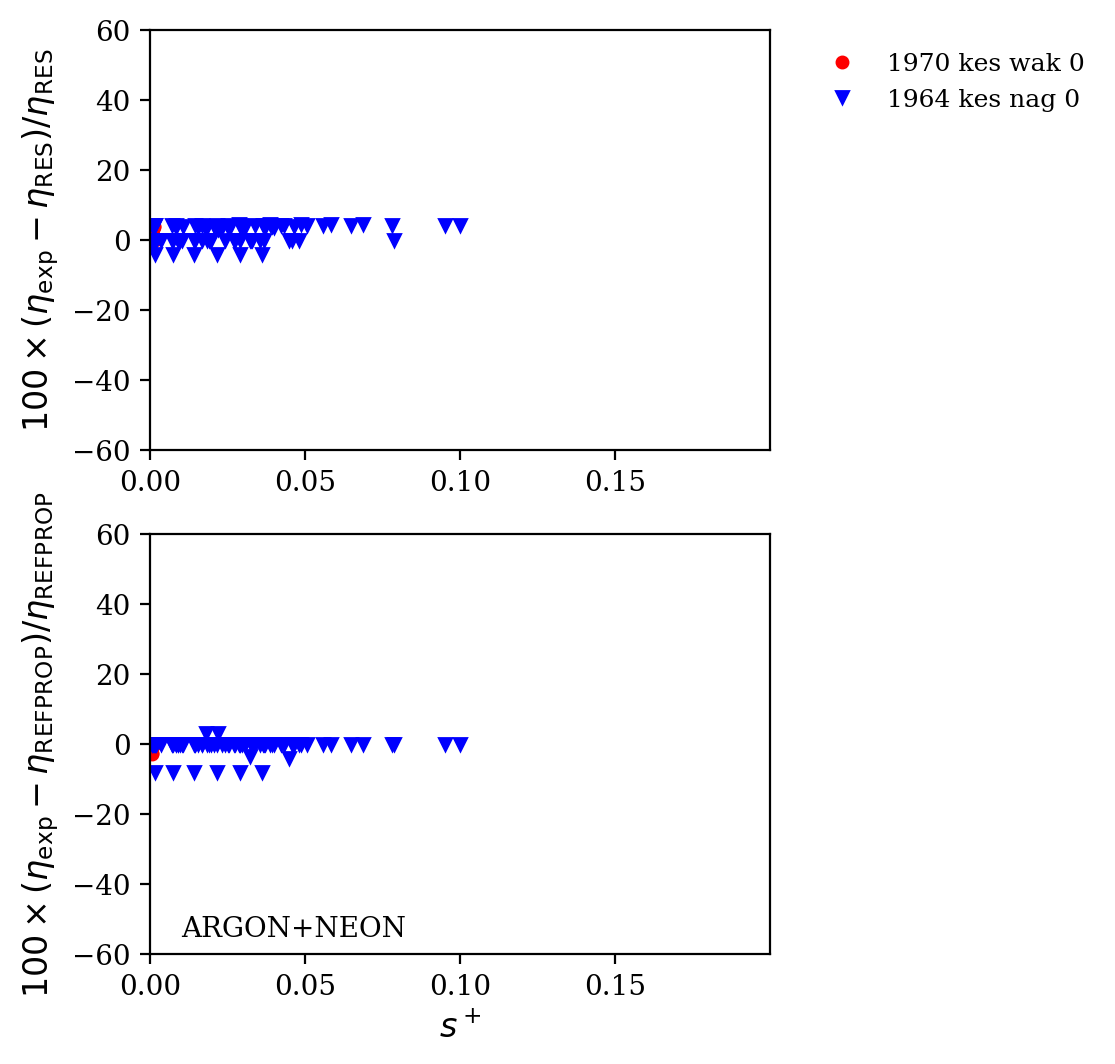

Supplement: Supplementary file 1 — je4c00451_si_001.zip [file je4c00451_si_001.zip › supporting_information/mix_dev_exp_res_ecs/ARGON+NEON.png]

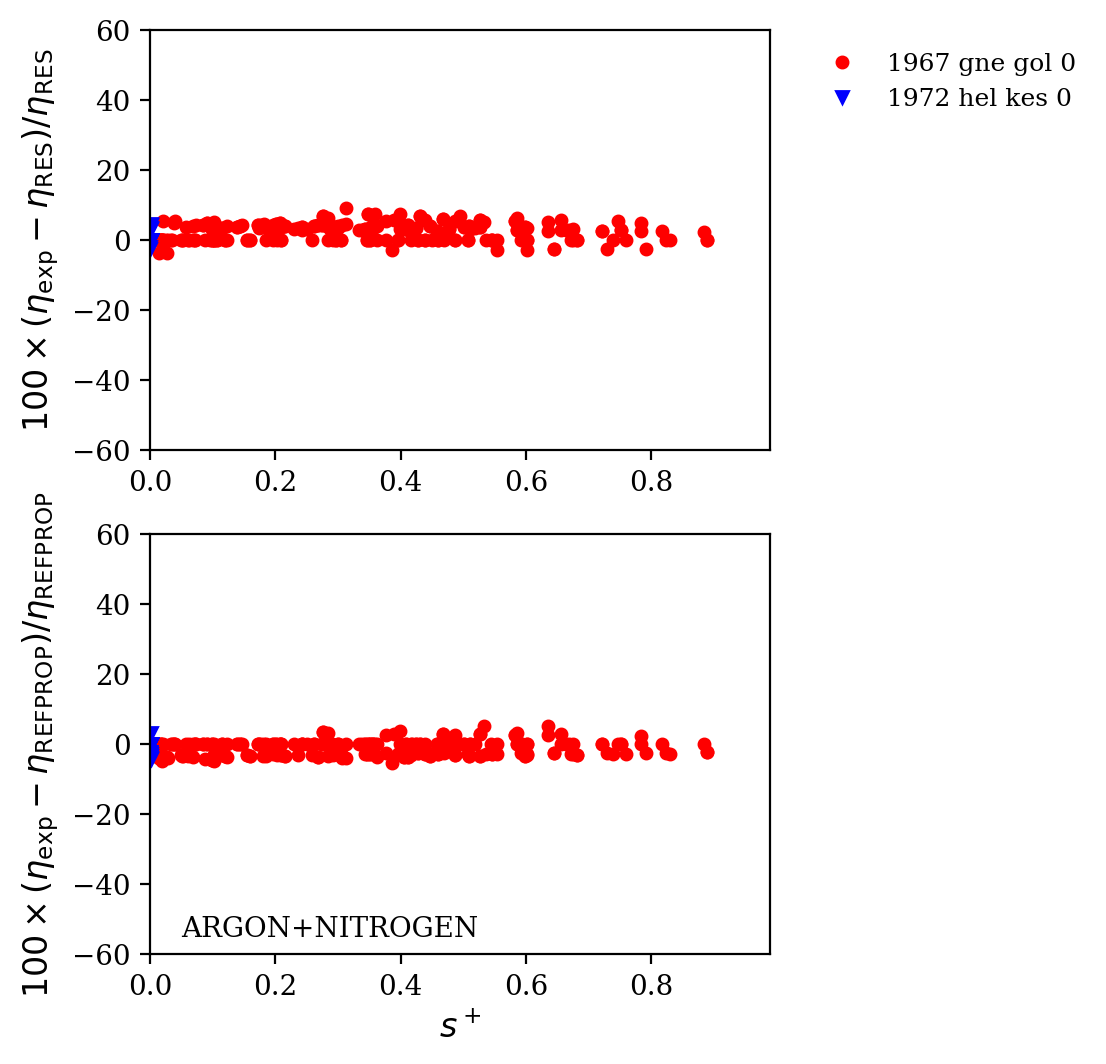

Supplement: Supplementary file 1 — je4c00451_si_001.zip [file je4c00451_si_001.zip › supporting_information/mix_dev_exp_res_ecs/ARGON+NITROGEN.png]

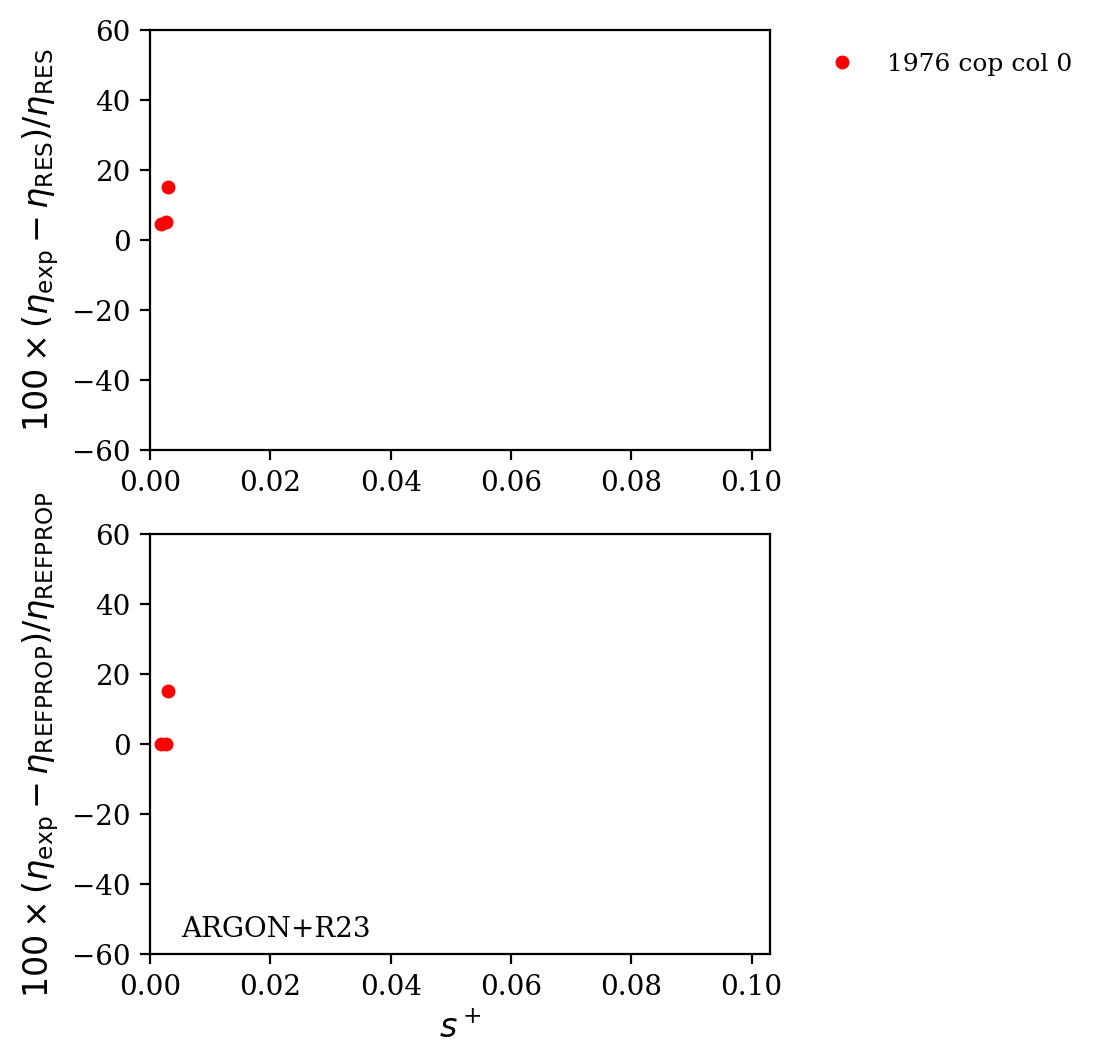

Supplement: Supplementary file 1 — je4c00451_si_001.zip [file je4c00451_si_001.zip › supporting_information/mix_dev_exp_res_ecs/ARGON+R23.png]

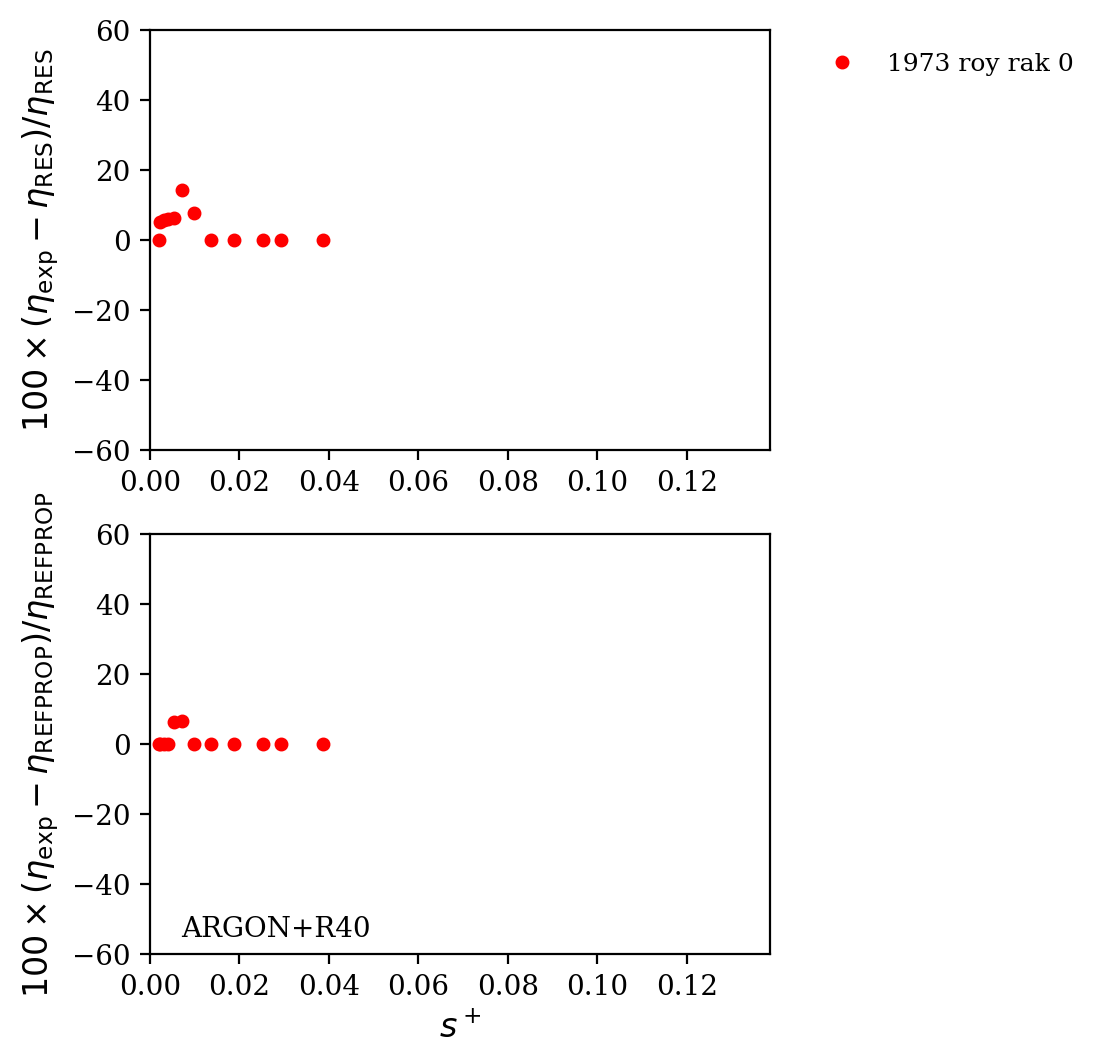

Supplement: Supplementary file 1 — je4c00451_si_001.zip [file je4c00451_si_001.zip › supporting_information/mix_dev_exp_res_ecs/ARGON+R40.png]

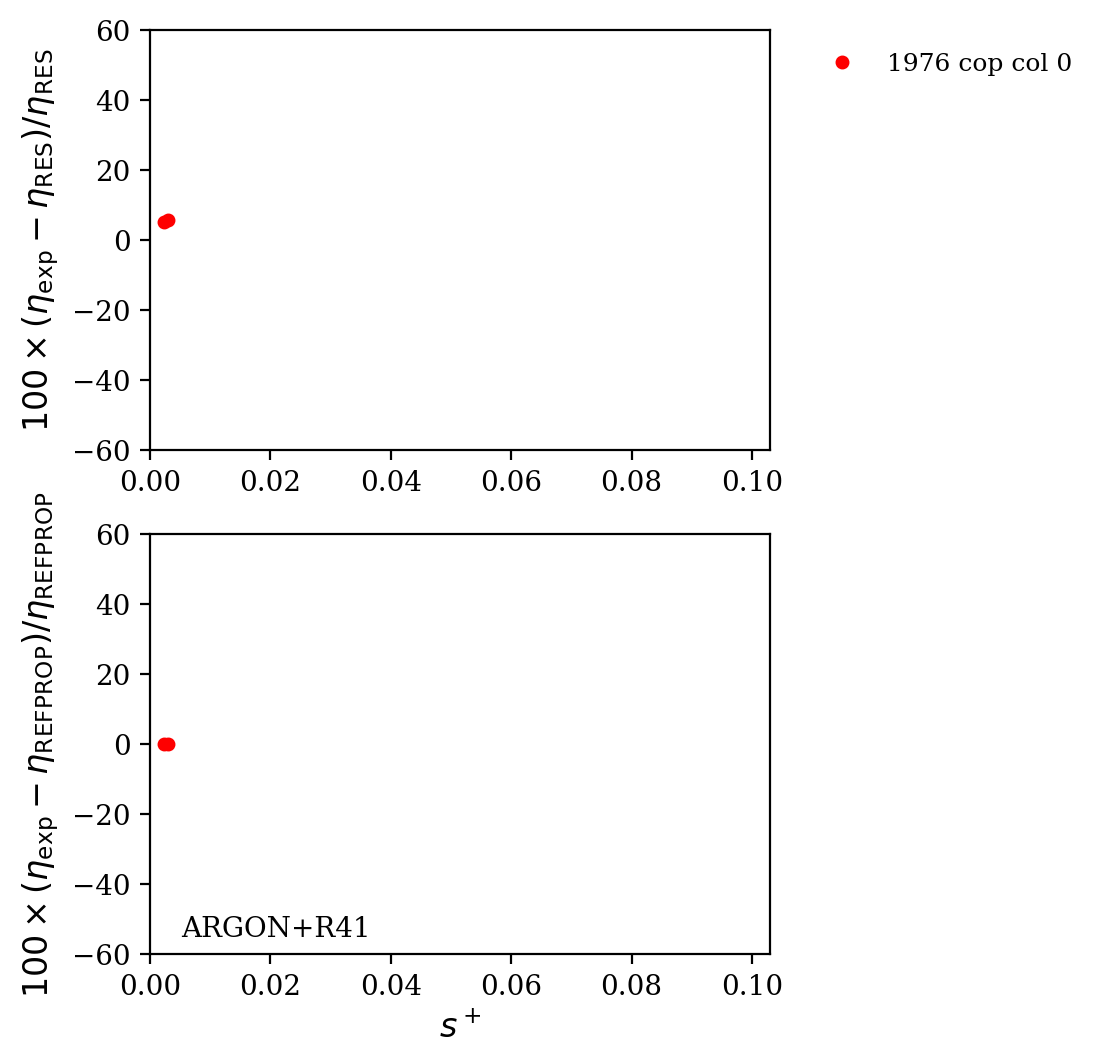

Supplement: Supplementary file 1 — je4c00451_si_001.zip [file je4c00451_si_001.zip › supporting_information/mix_dev_exp_res_ecs/ARGON+R41.png]

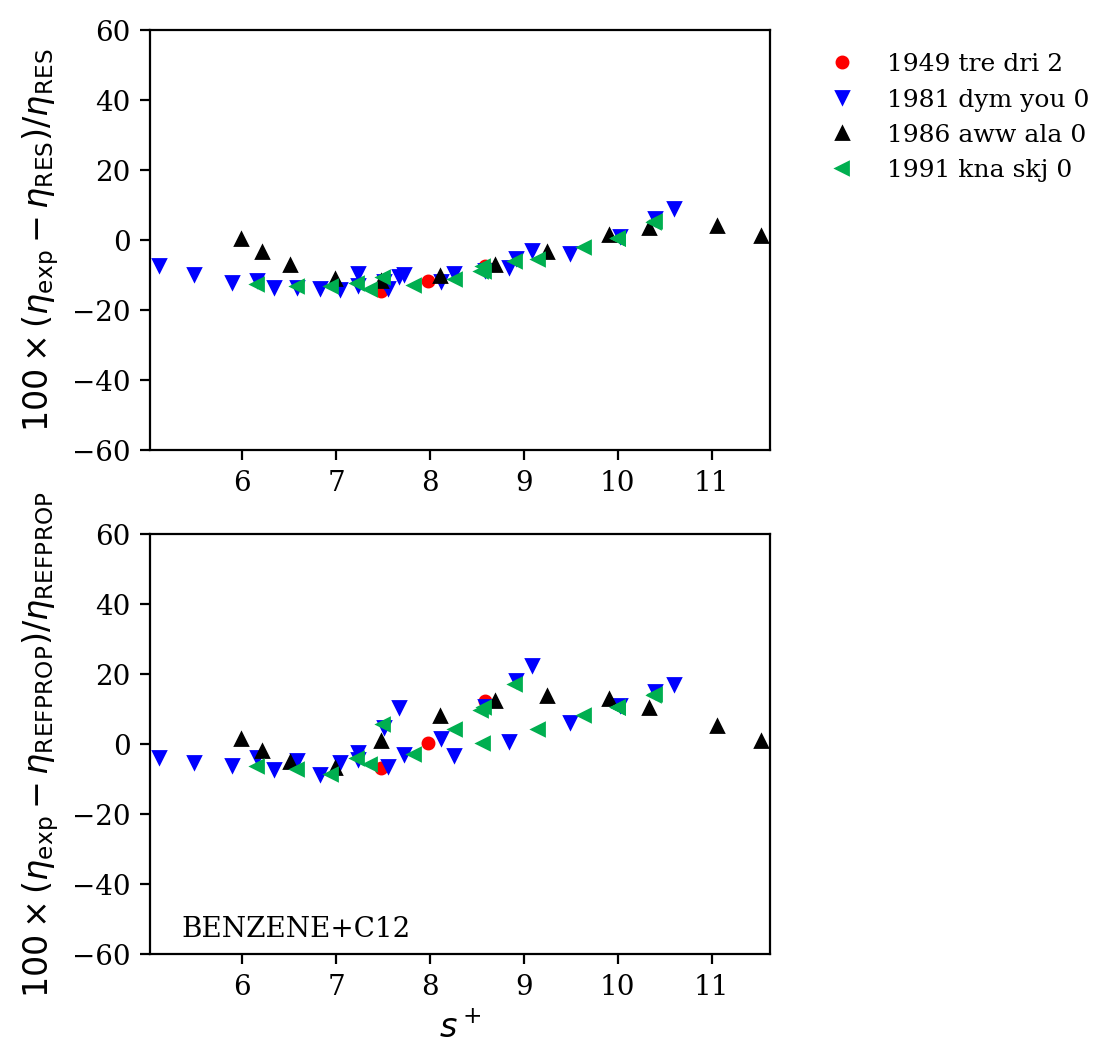

Supplement: Supplementary file 1 — je4c00451_si_001.zip [file je4c00451_si_001.zip › supporting_information/mix_dev_exp_res_ecs/BENZENE+C12.png]

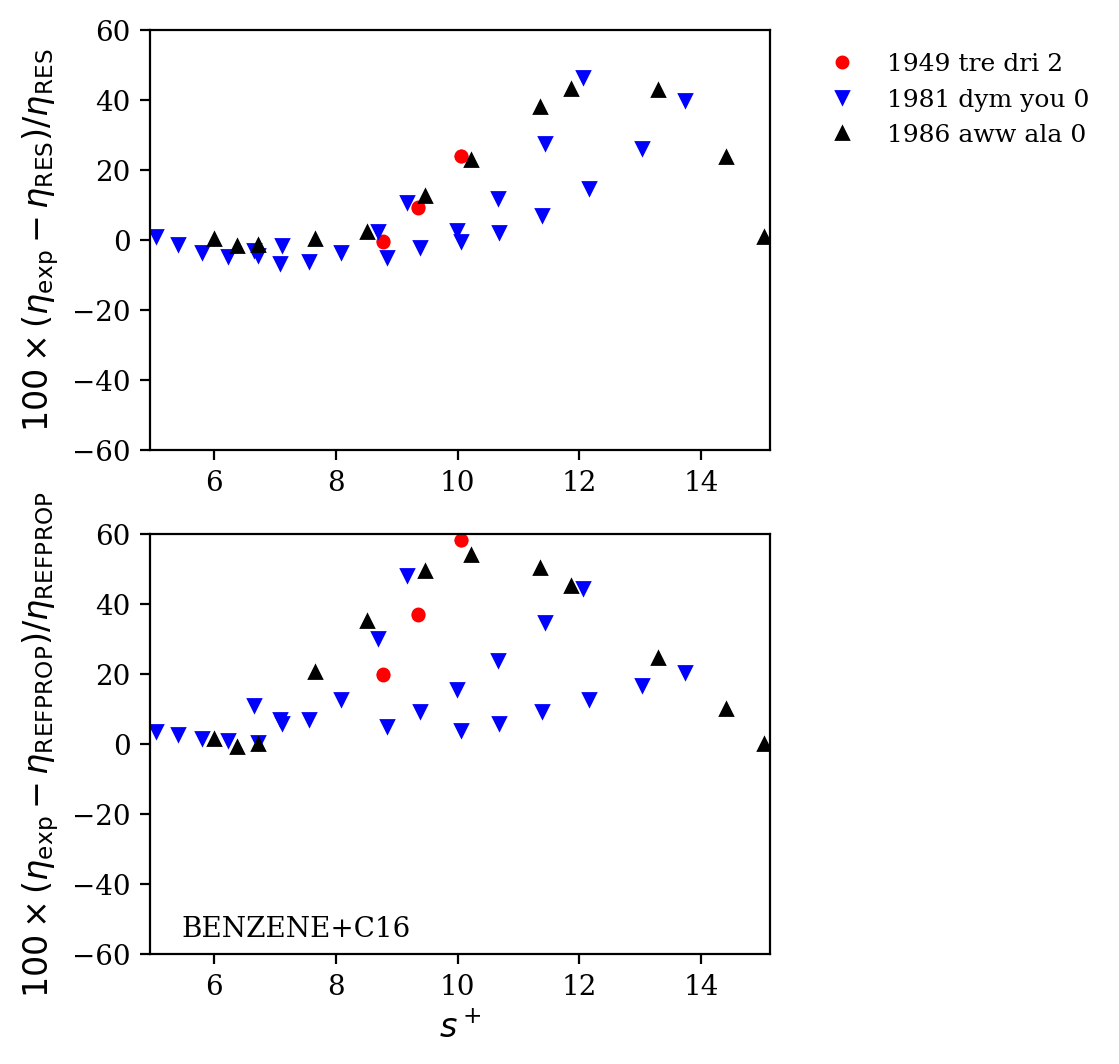

Supplement: Supplementary file 1 — je4c00451_si_001.zip [file je4c00451_si_001.zip › supporting_information/mix_dev_exp_res_ecs/BENZENE+C16.png]

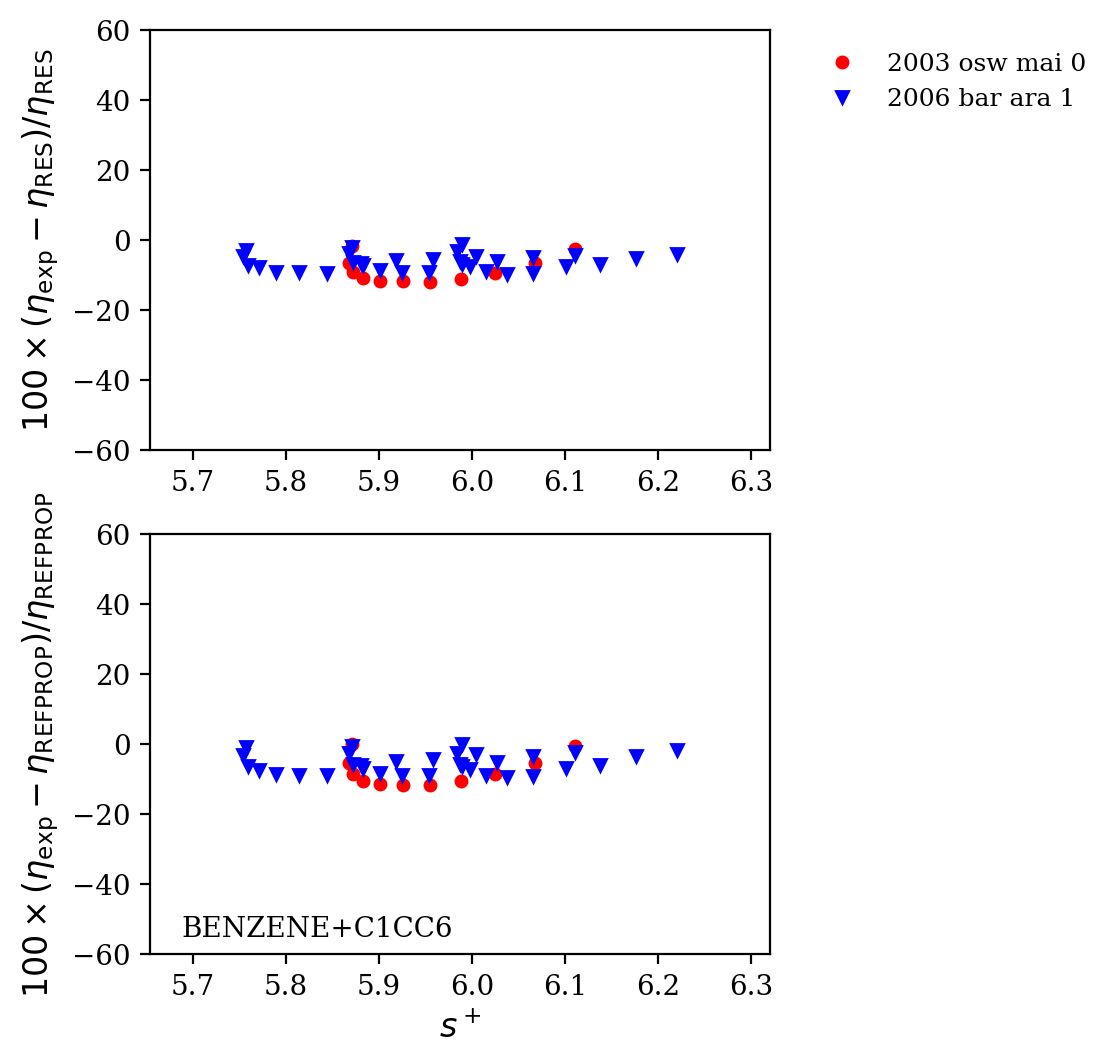

Supplement: Supplementary file 1 — je4c00451_si_001.zip [file je4c00451_si_001.zip › supporting_information/mix_dev_exp_res_ecs/BENZENE+C1CC6.png]

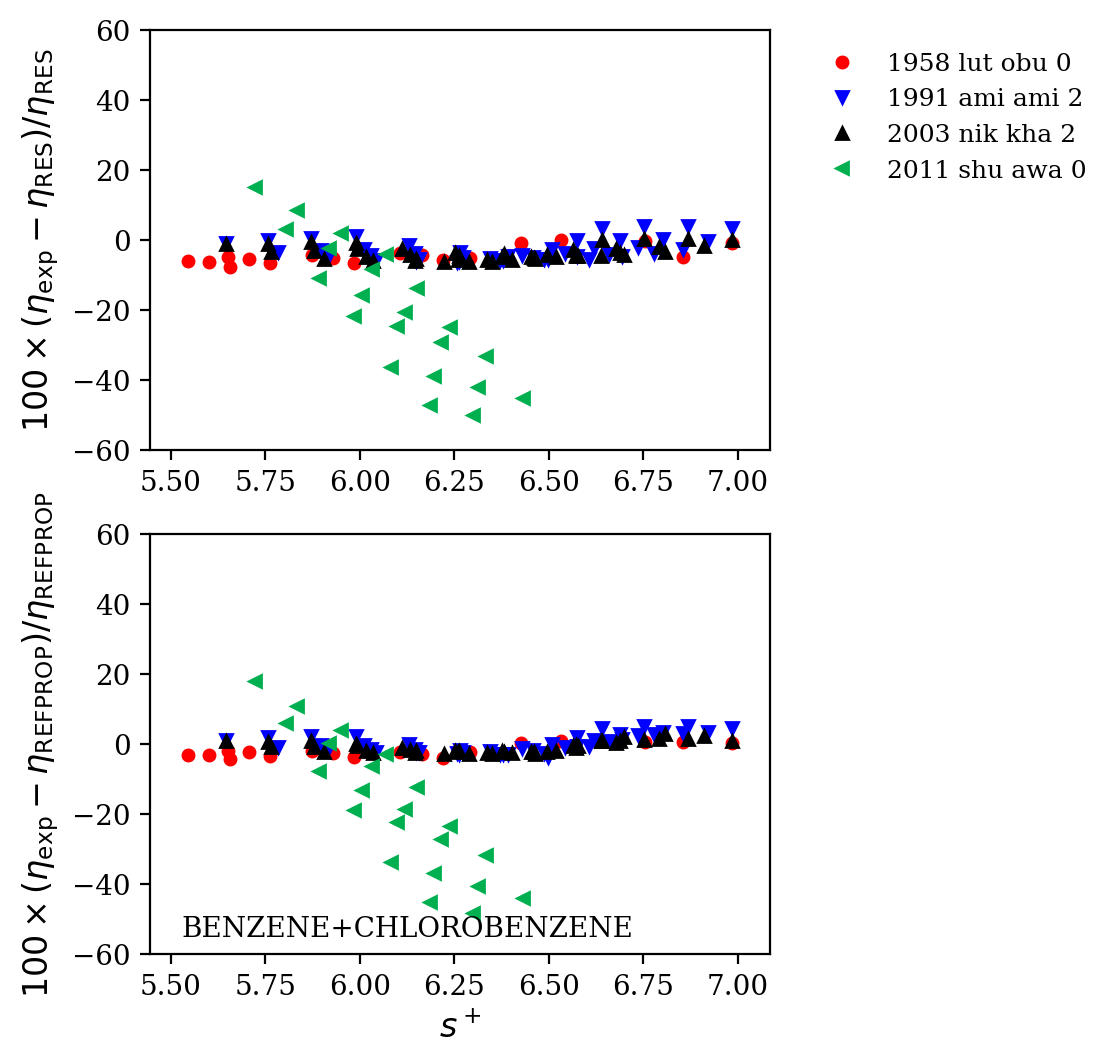

Supplement: Supplementary file 1 — je4c00451_si_001.zip [file je4c00451_si_001.zip › supporting_information/mix_dev_exp_res_ecs/BENZENE+CHLOROBENZENE.png]

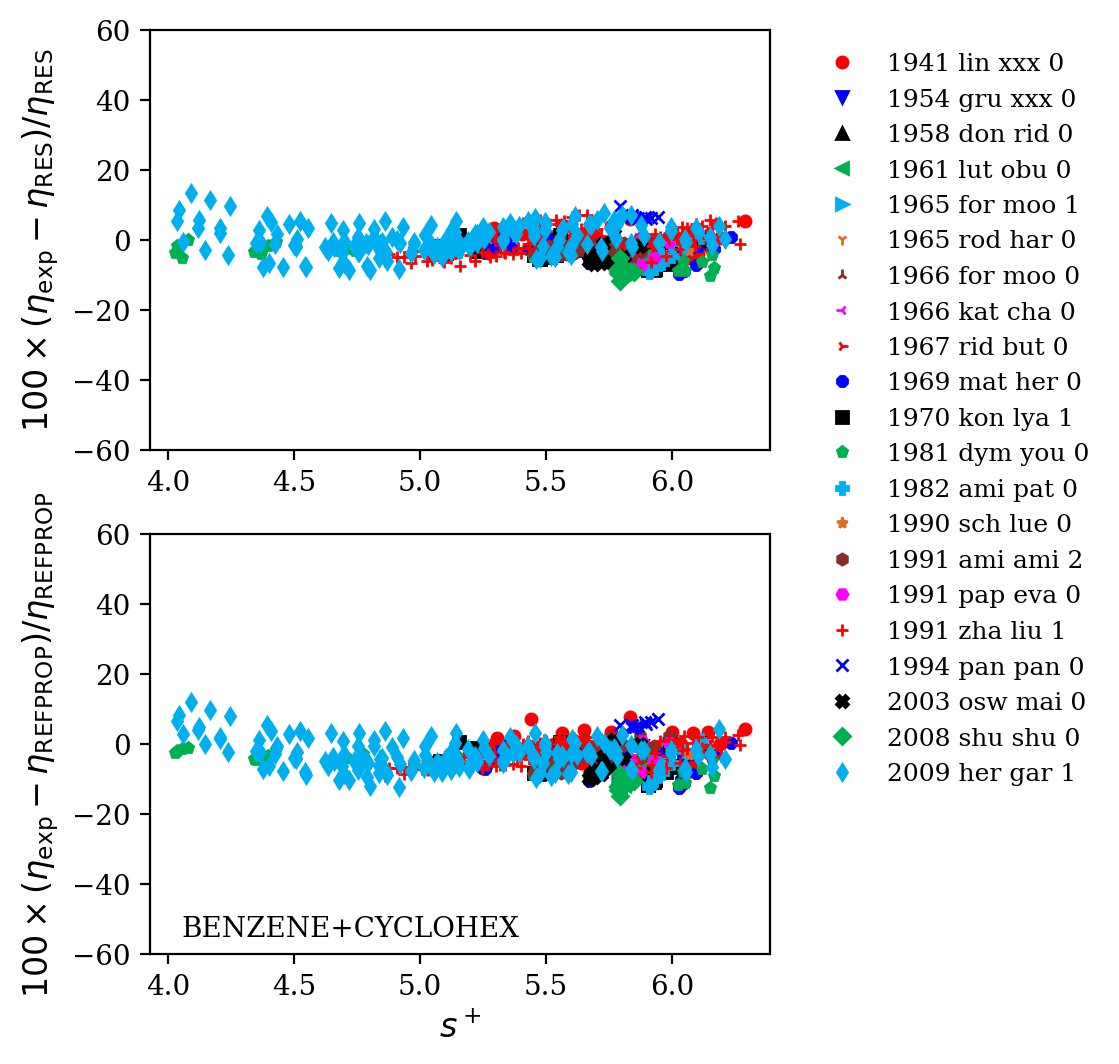

Supplement: Supplementary file 1 — je4c00451_si_001.zip [file je4c00451_si_001.zip › supporting_information/mix_dev_exp_res_ecs/BENZENE+CYCLOHEX.png]

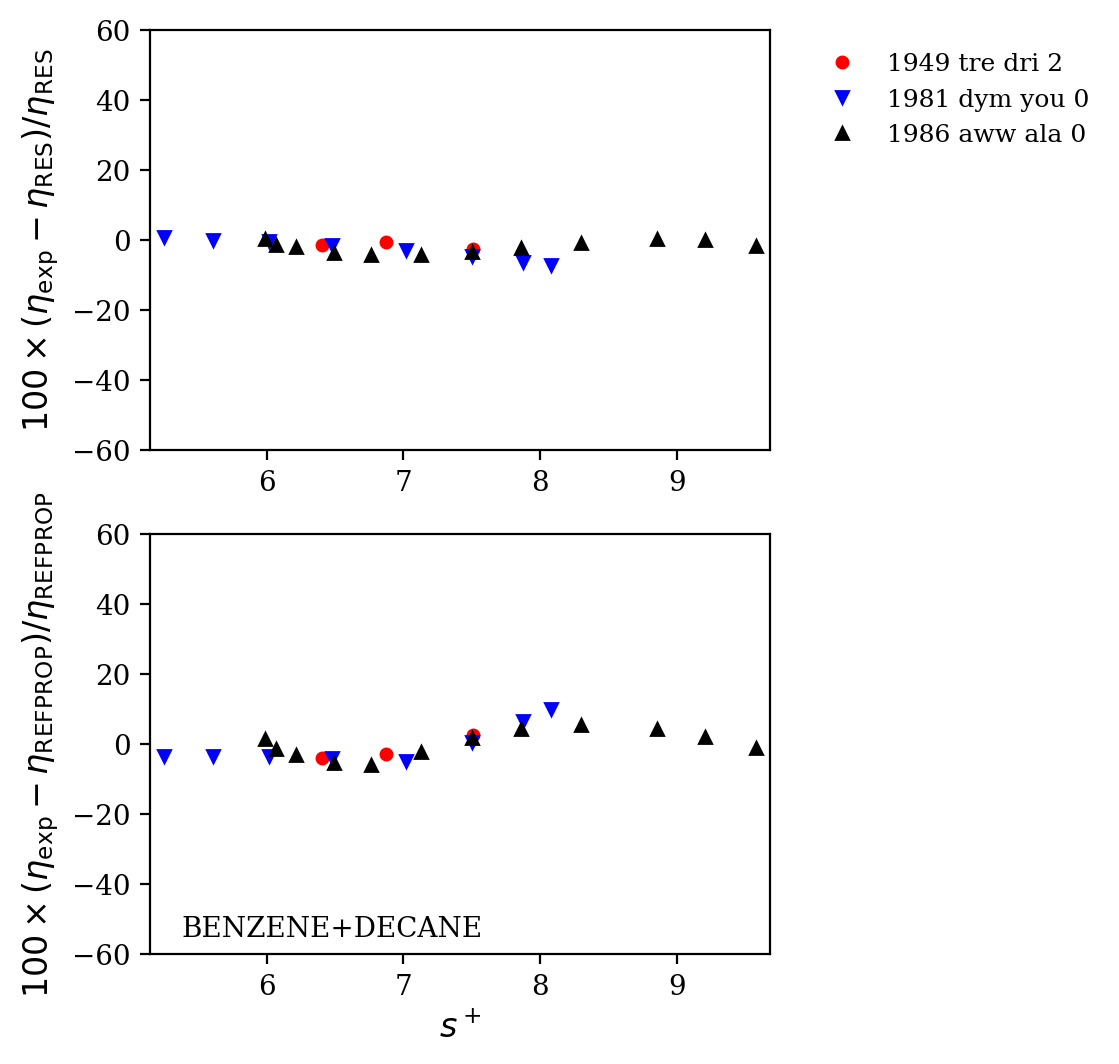

Supplement: Supplementary file 1 — je4c00451_si_001.zip [file je4c00451_si_001.zip › supporting_information/mix_dev_exp_res_ecs/BENZENE+DECANE.png]

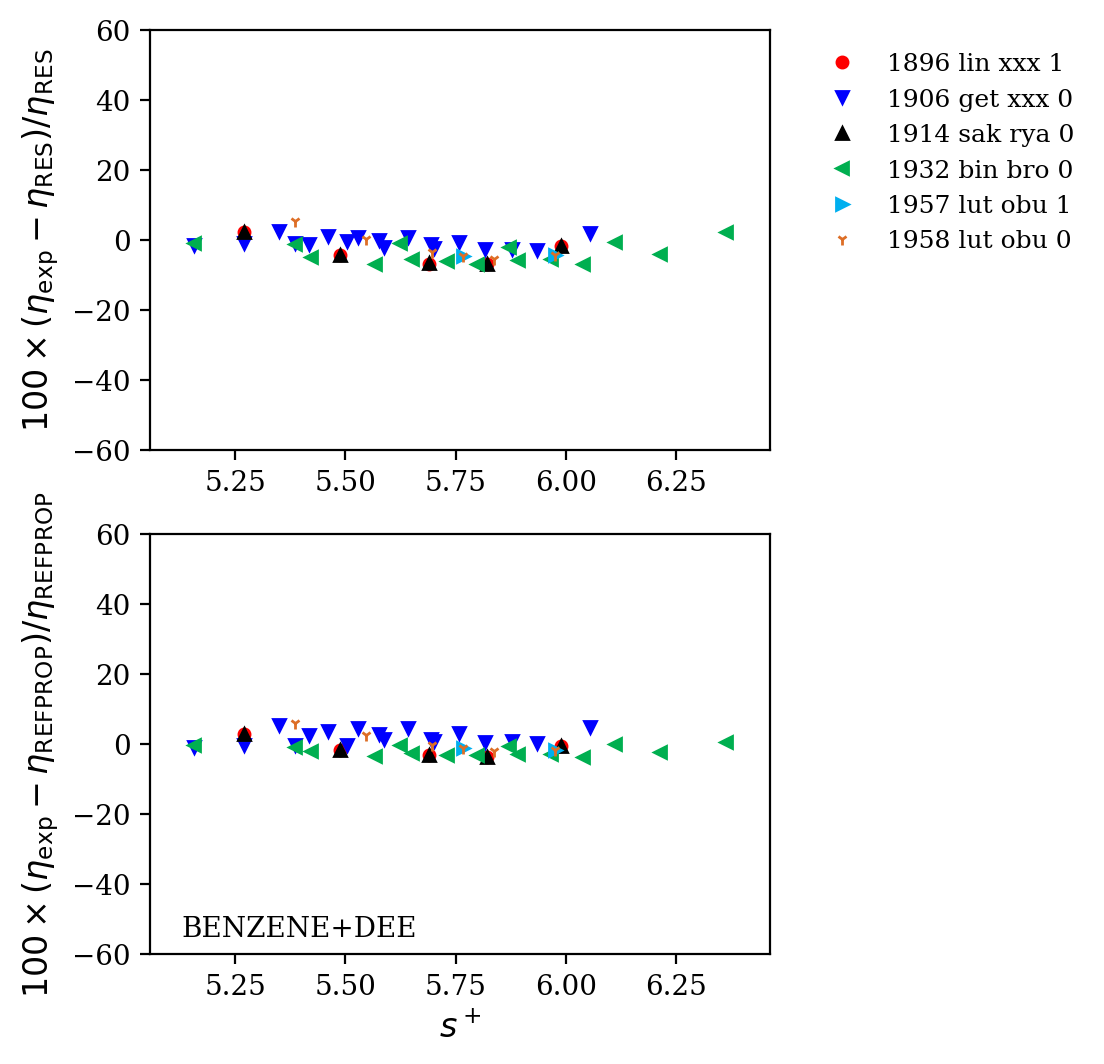

Supplement: Supplementary file 1 — je4c00451_si_001.zip [file je4c00451_si_001.zip › supporting_information/mix_dev_exp_res_ecs/BENZENE+DEE.png]

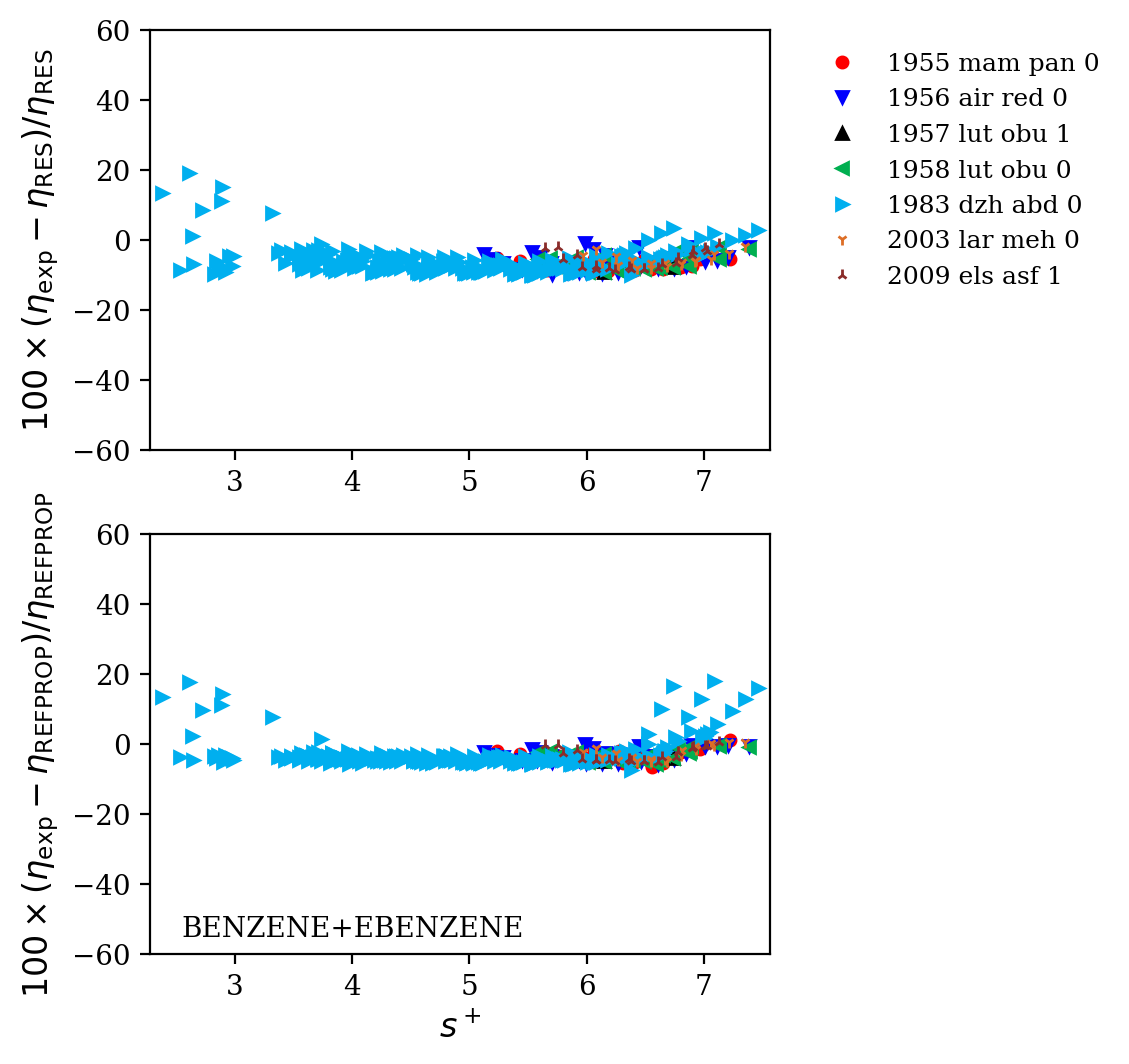

Supplement: Supplementary file 1 — je4c00451_si_001.zip [file je4c00451_si_001.zip › supporting_information/mix_dev_exp_res_ecs/BENZENE+EBENZENE.png]

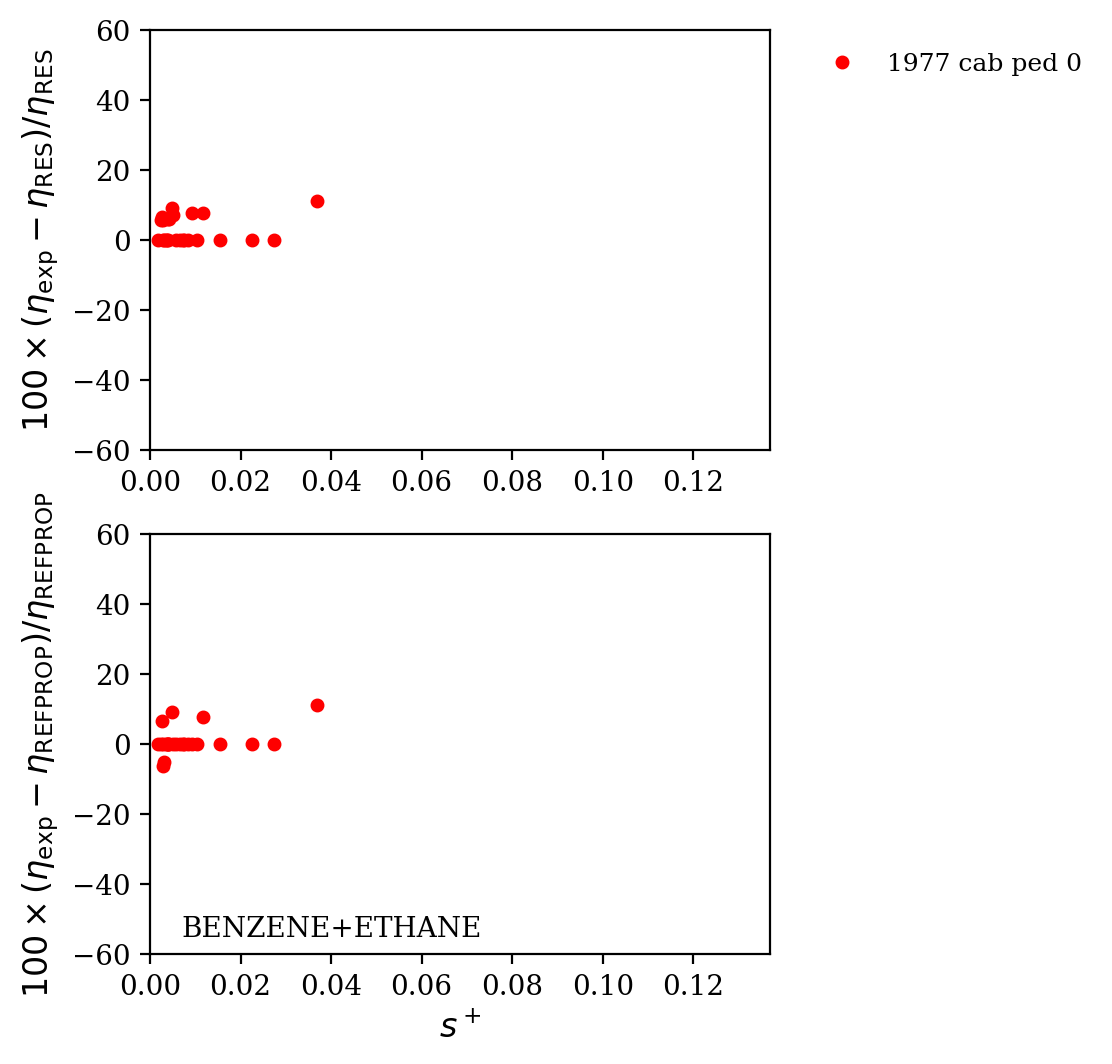

Supplement: Supplementary file 1 — je4c00451_si_001.zip [file je4c00451_si_001.zip › supporting_information/mix_dev_exp_res_ecs/BENZENE+ETHANE.png]

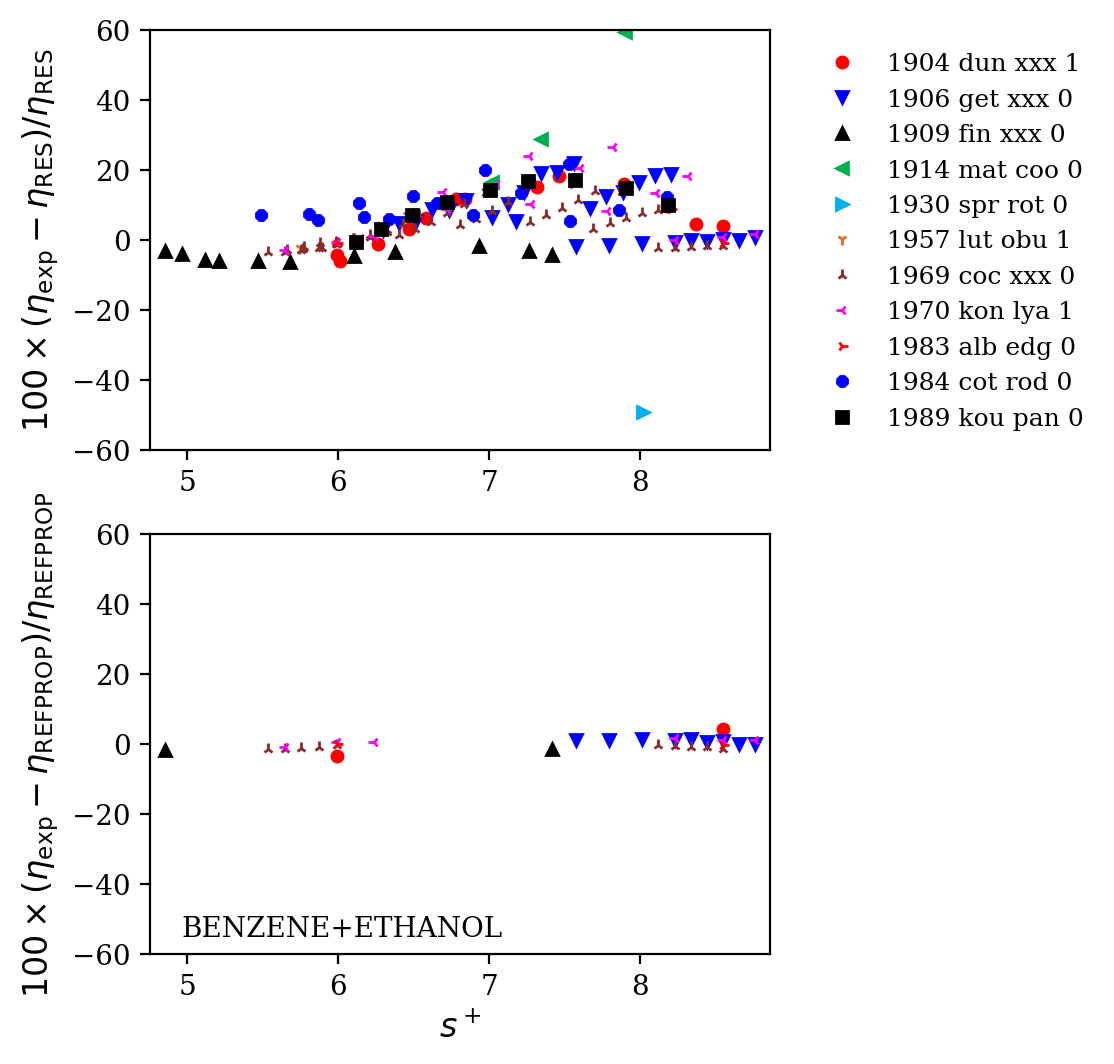

Supplement: Supplementary file 1 — je4c00451_si_001.zip [file je4c00451_si_001.zip › supporting_information/mix_dev_exp_res_ecs/BENZENE+ETHANOL.png]

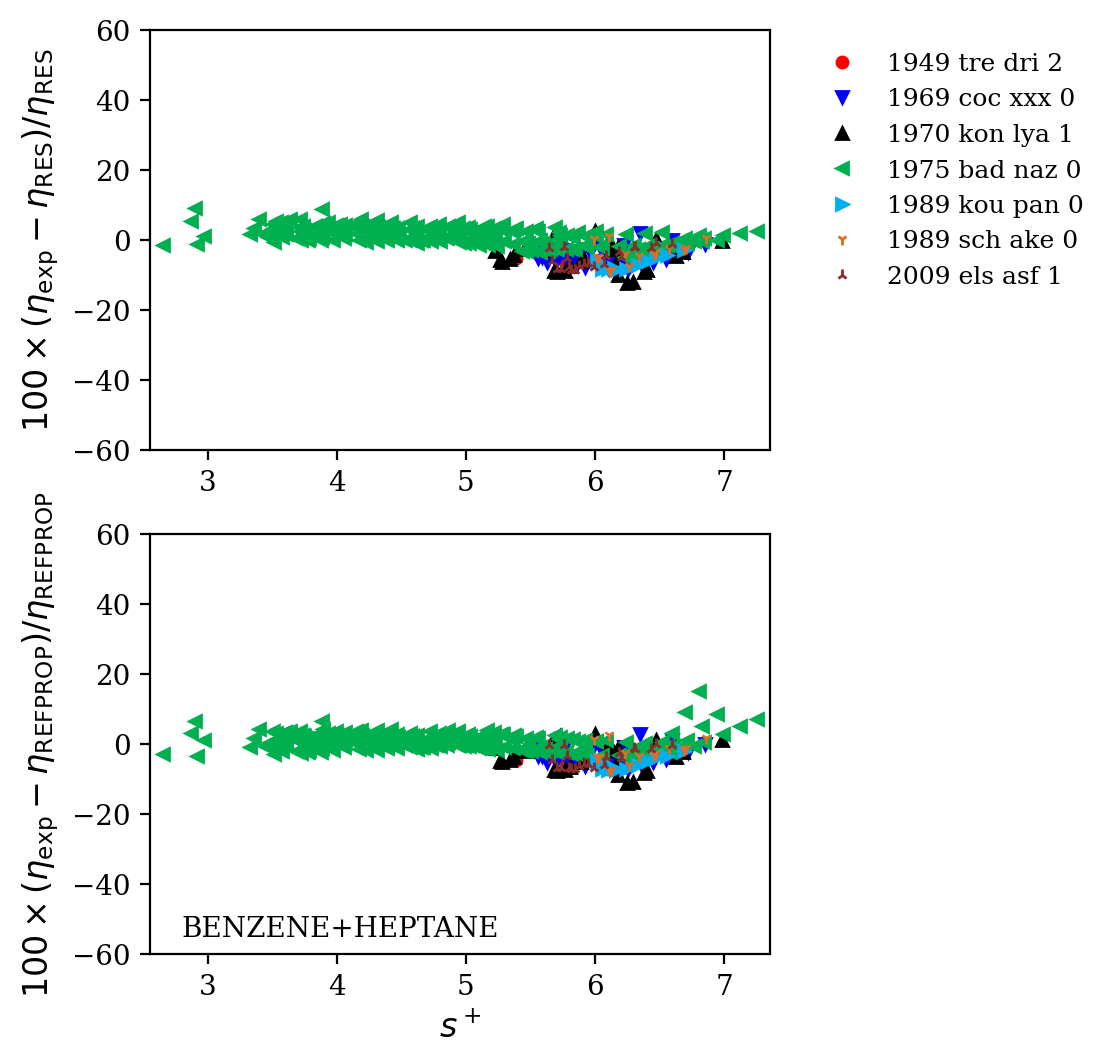

Supplement: Supplementary file 1 — je4c00451_si_001.zip [file je4c00451_si_001.zip › supporting_information/mix_dev_exp_res_ecs/BENZENE+HEPTANE.png]

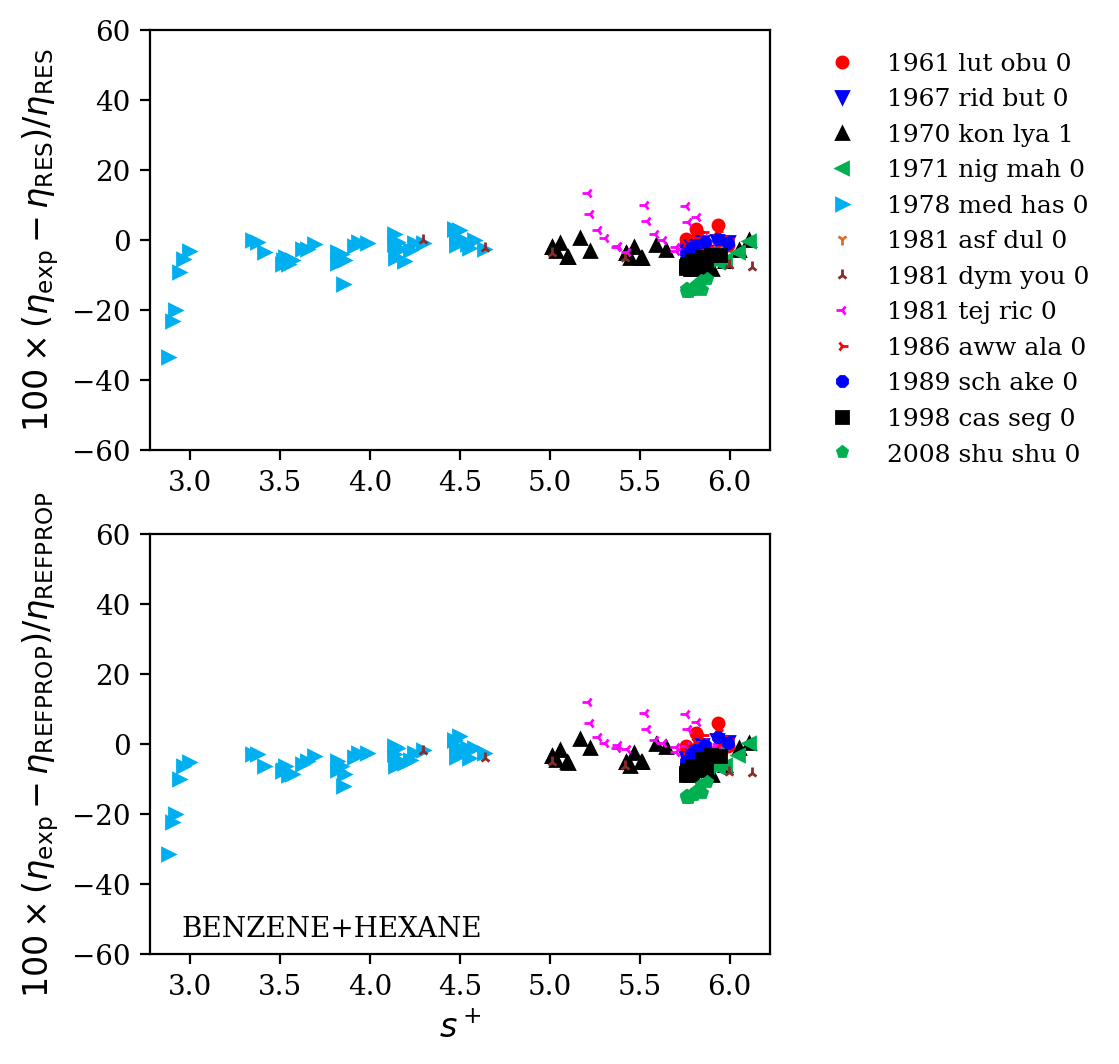

Supplement: Supplementary file 1 — je4c00451_si_001.zip [file je4c00451_si_001.zip › supporting_information/mix_dev_exp_res_ecs/BENZENE+HEXANE.png]

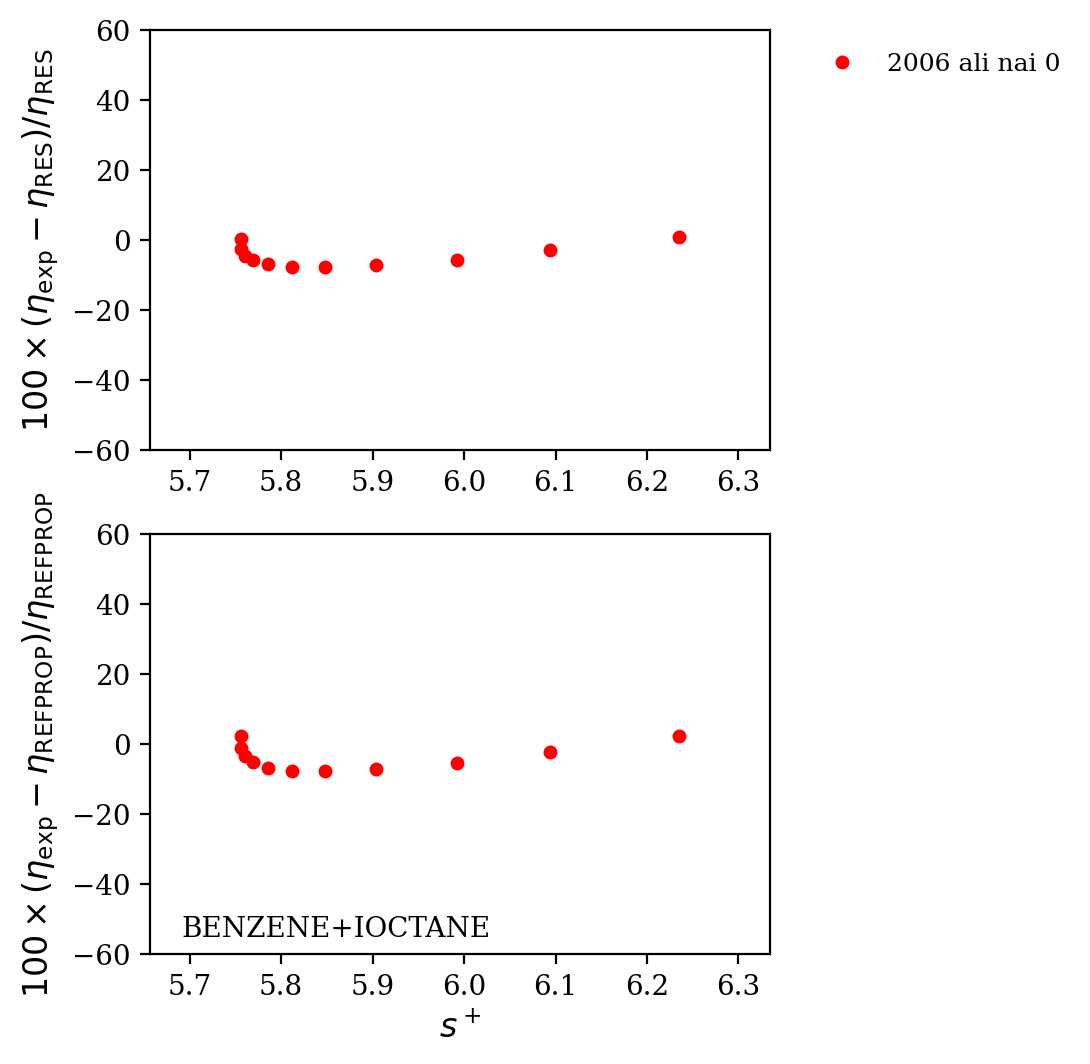

Supplement: Supplementary file 1 — je4c00451_si_001.zip [file je4c00451_si_001.zip › supporting_information/mix_dev_exp_res_ecs/BENZENE+IOCTANE.png]

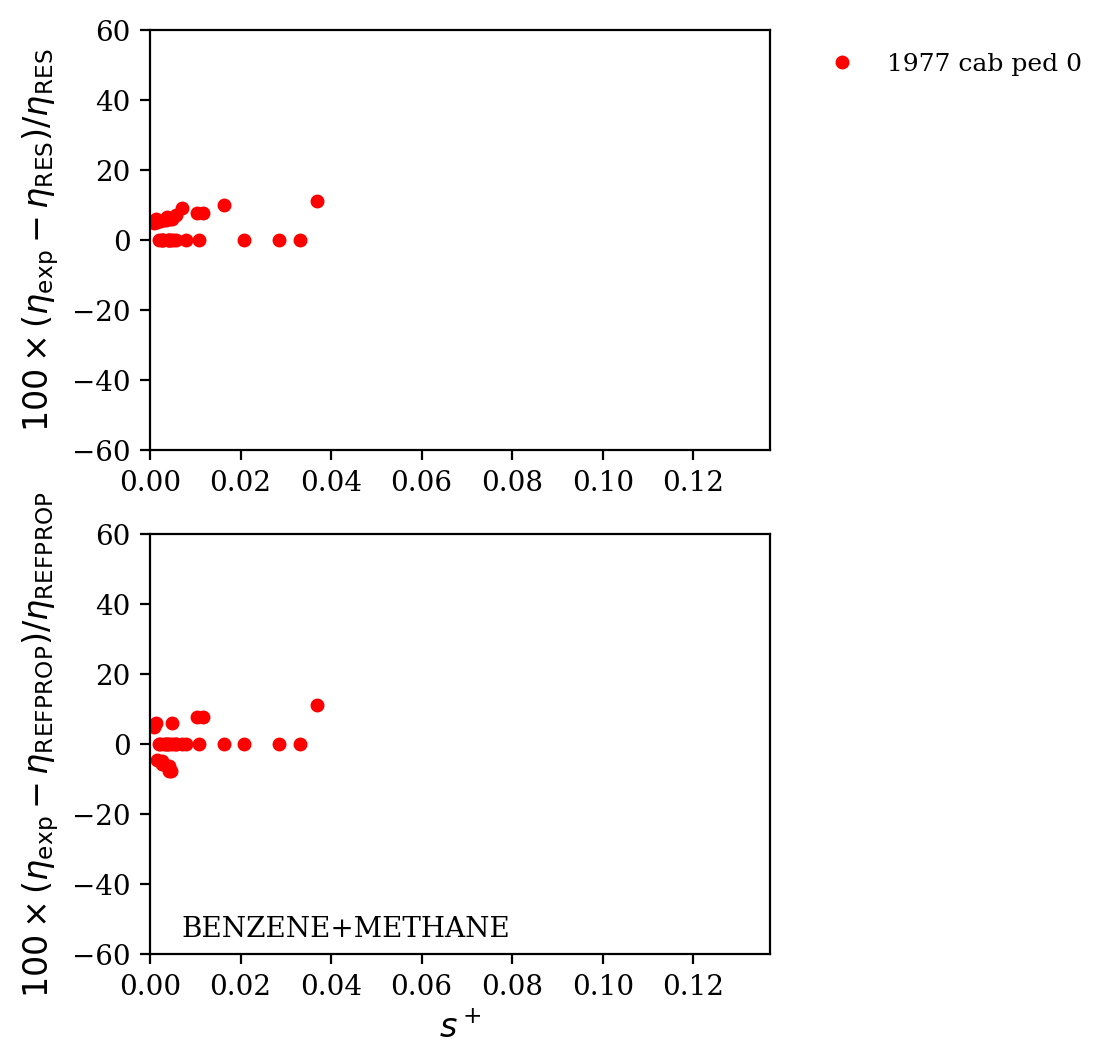

Supplement: Supplementary file 1 — je4c00451_si_001.zip [file je4c00451_si_001.zip › supporting_information/mix_dev_exp_res_ecs/BENZENE+METHANE.png]

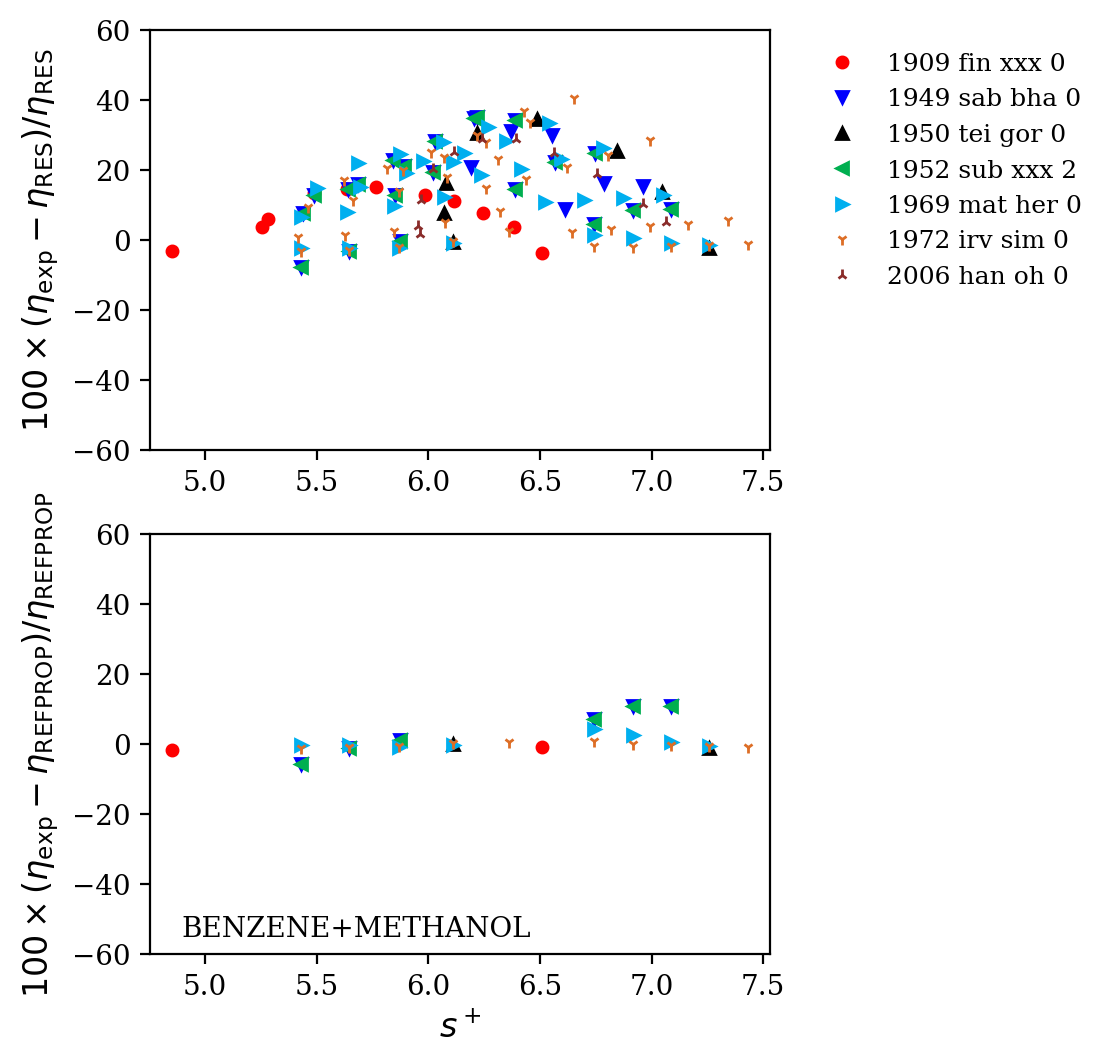

Supplement: Supplementary file 1 — je4c00451_si_001.zip [file je4c00451_si_001.zip › supporting_information/mix_dev_exp_res_ecs/BENZENE+METHANOL.png]

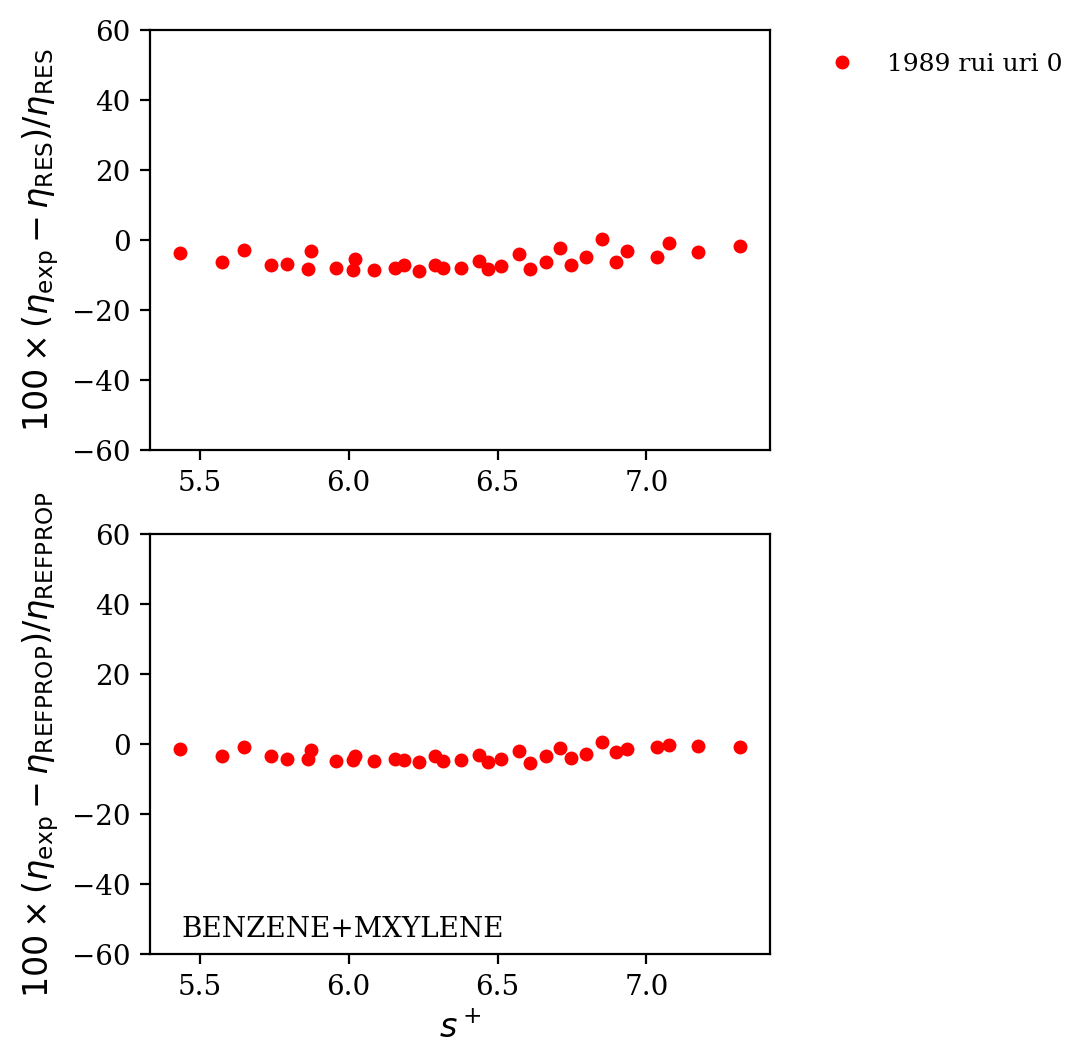

Supplement: Supplementary file 1 — je4c00451_si_001.zip [file je4c00451_si_001.zip › supporting_information/mix_dev_exp_res_ecs/BENZENE+MXYLENE.png]

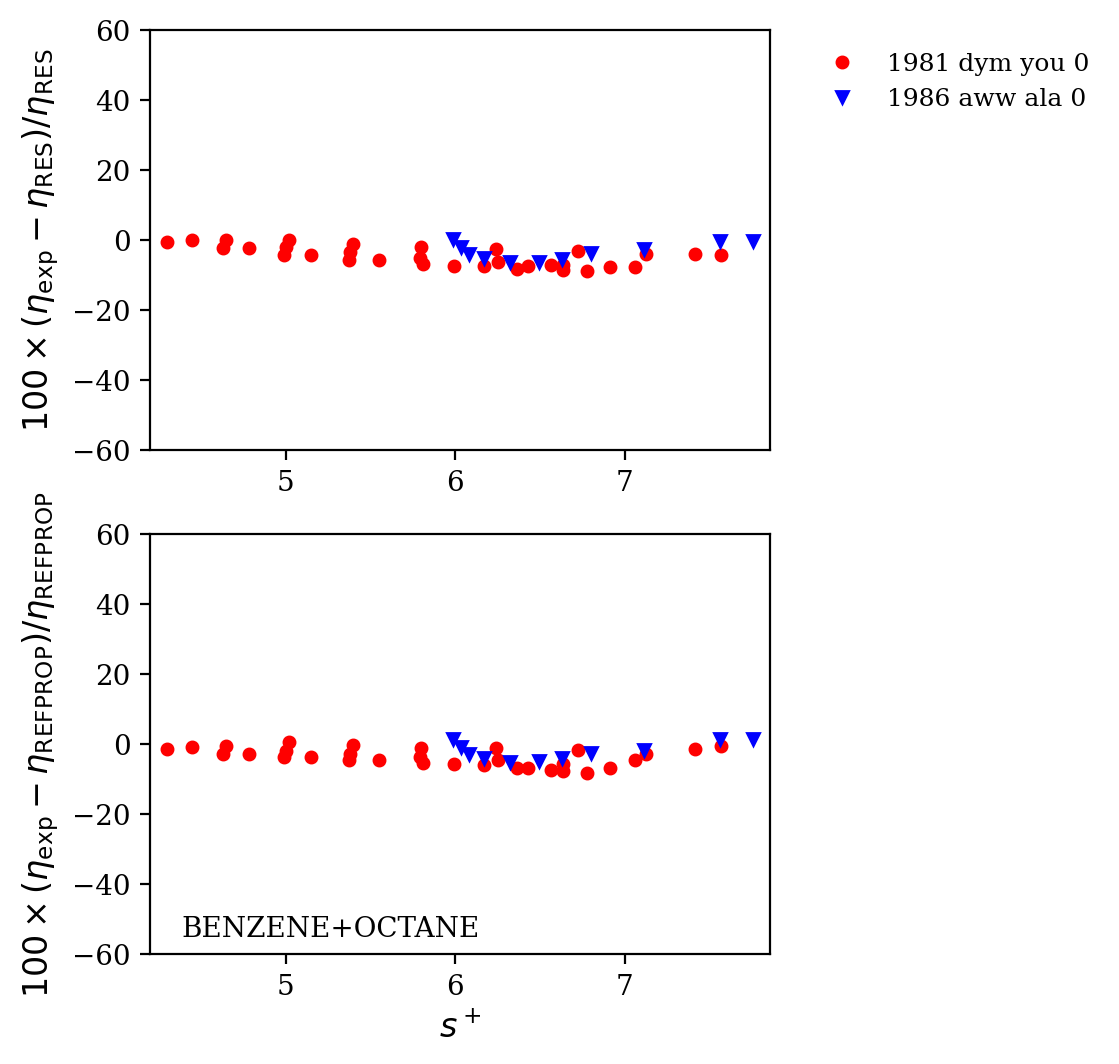

Supplement: Supplementary file 1 — je4c00451_si_001.zip [file je4c00451_si_001.zip › supporting_information/mix_dev_exp_res_ecs/BENZENE+OCTANE.png]

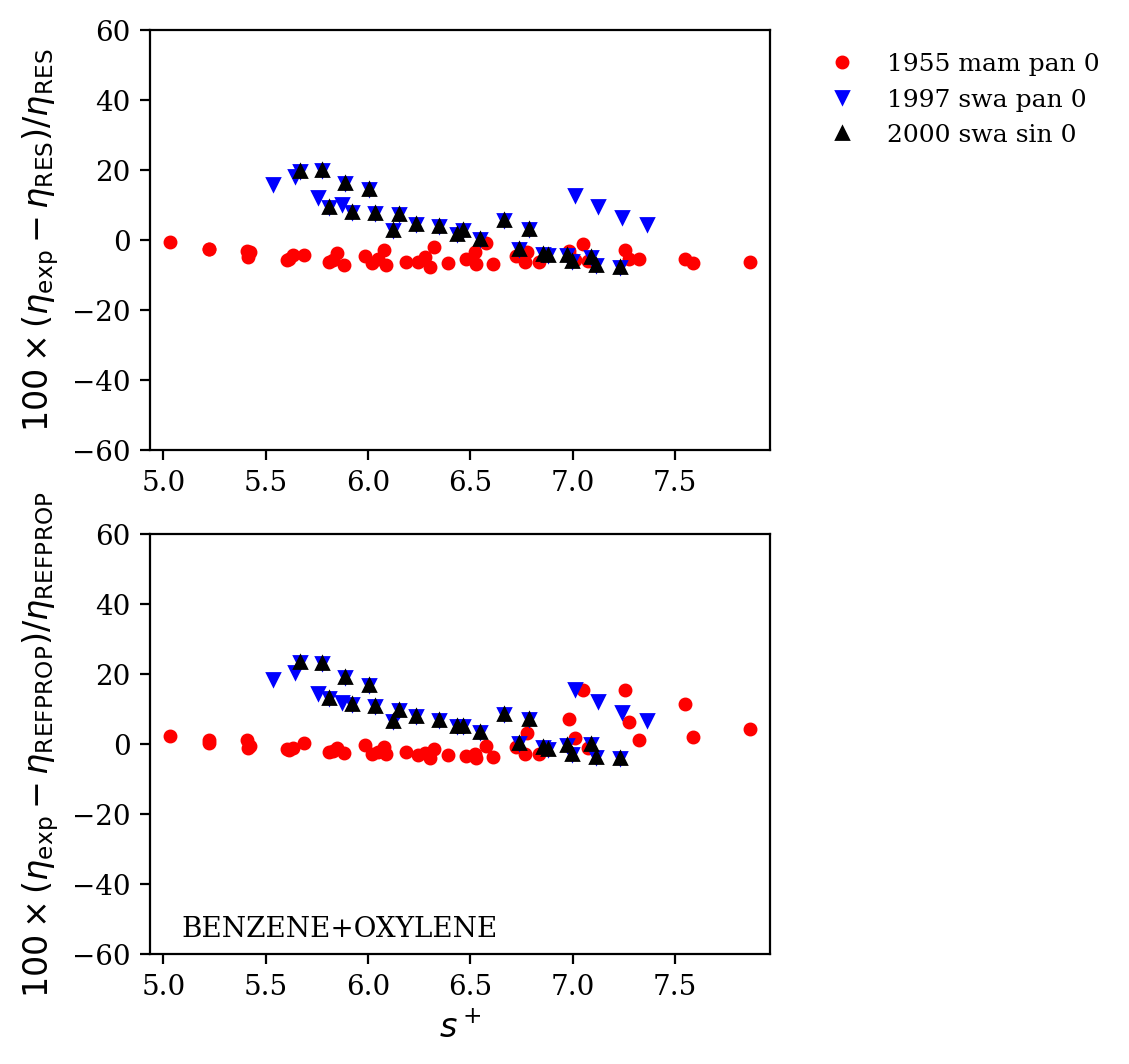

Supplement: Supplementary file 1 — je4c00451_si_001.zip [file je4c00451_si_001.zip › supporting_information/mix_dev_exp_res_ecs/BENZENE+OXYLENE.png]

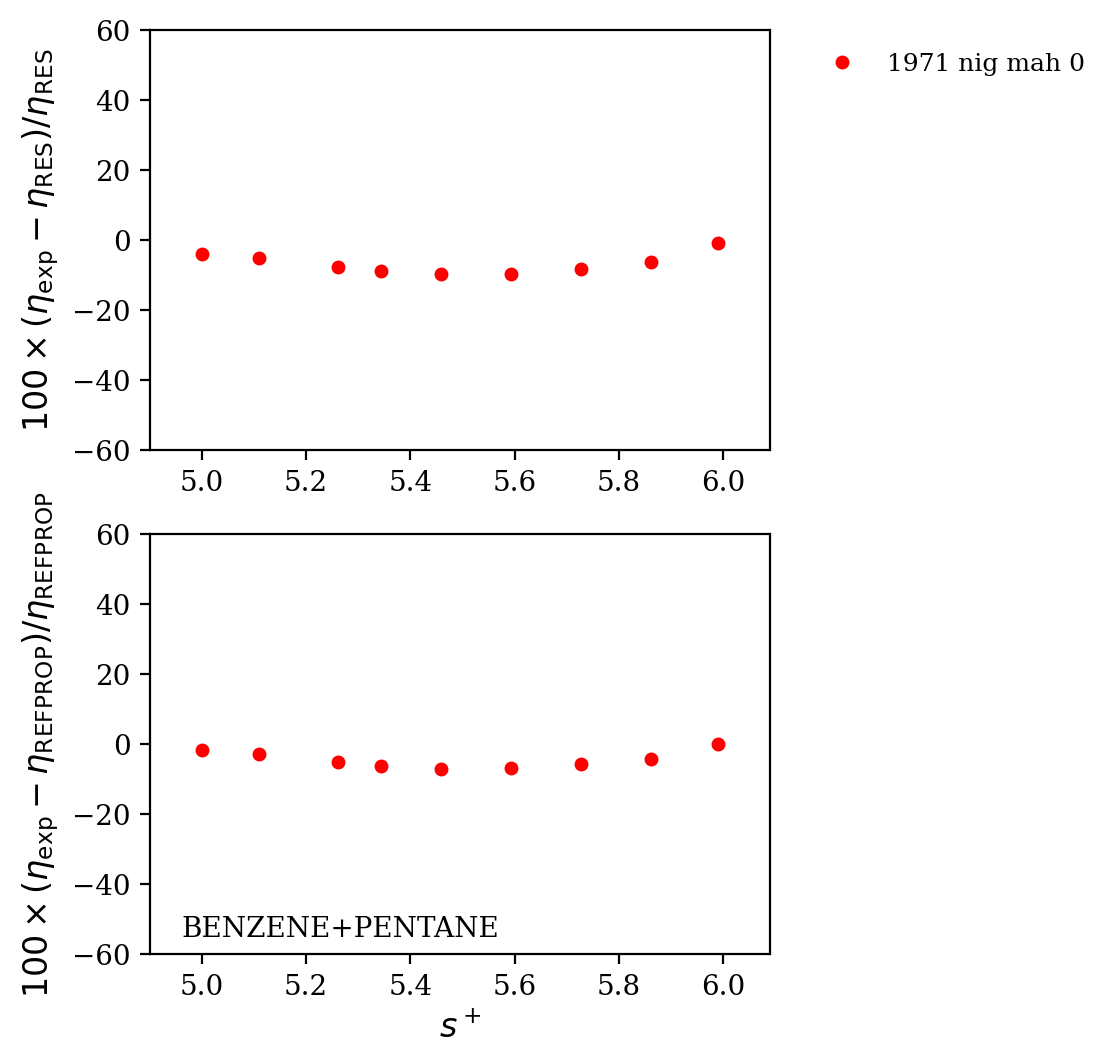

Supplement: Supplementary file 1 — je4c00451_si_001.zip [file je4c00451_si_001.zip › supporting_information/mix_dev_exp_res_ecs/BENZENE+PENTANE.png]

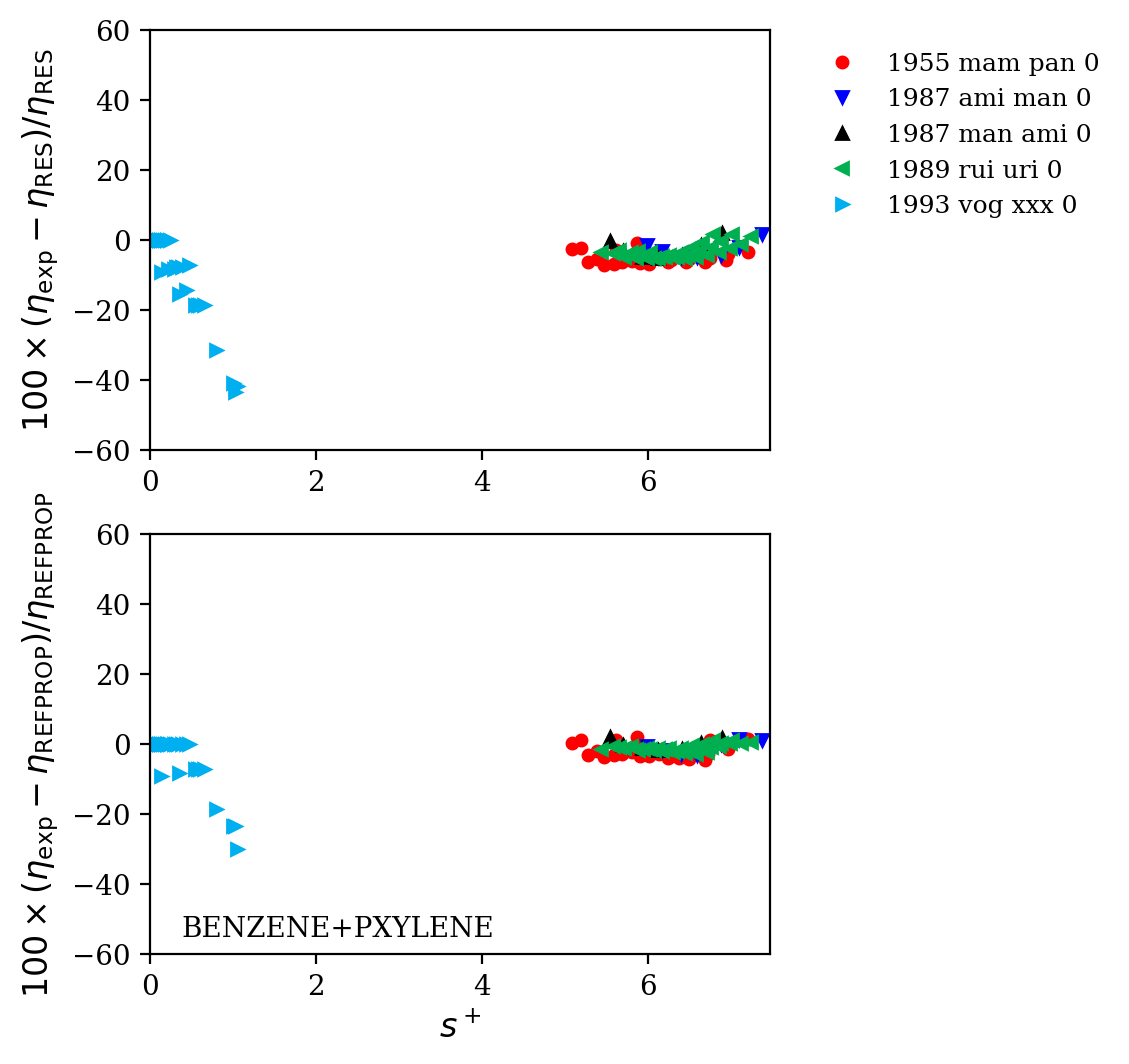

Supplement: Supplementary file 1 — je4c00451_si_001.zip [file je4c00451_si_001.zip › supporting_information/mix_dev_exp_res_ecs/BENZENE+PXYLENE.png]

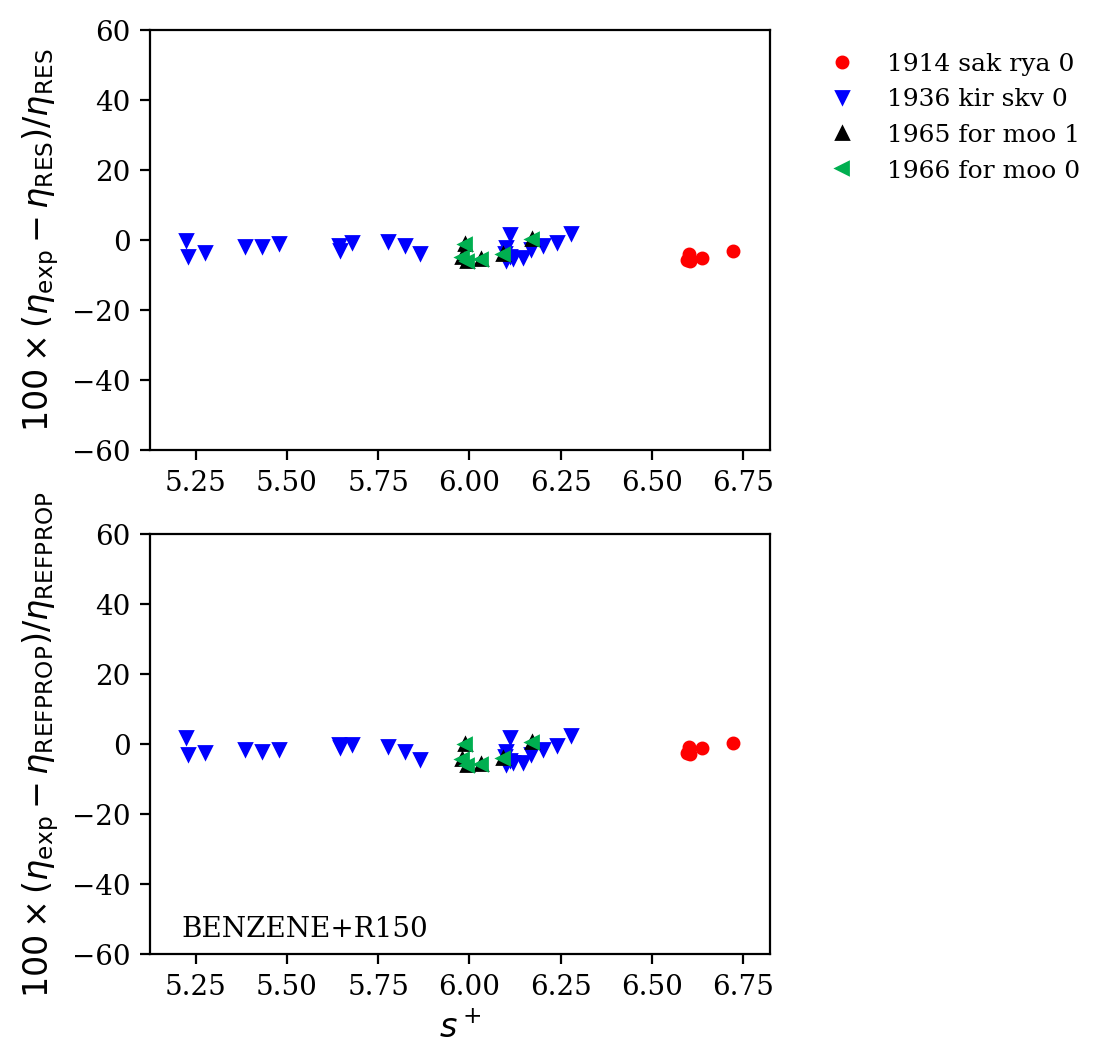

Supplement: Supplementary file 1 — je4c00451_si_001.zip [file je4c00451_si_001.zip › supporting_information/mix_dev_exp_res_ecs/BENZENE+R150.png]

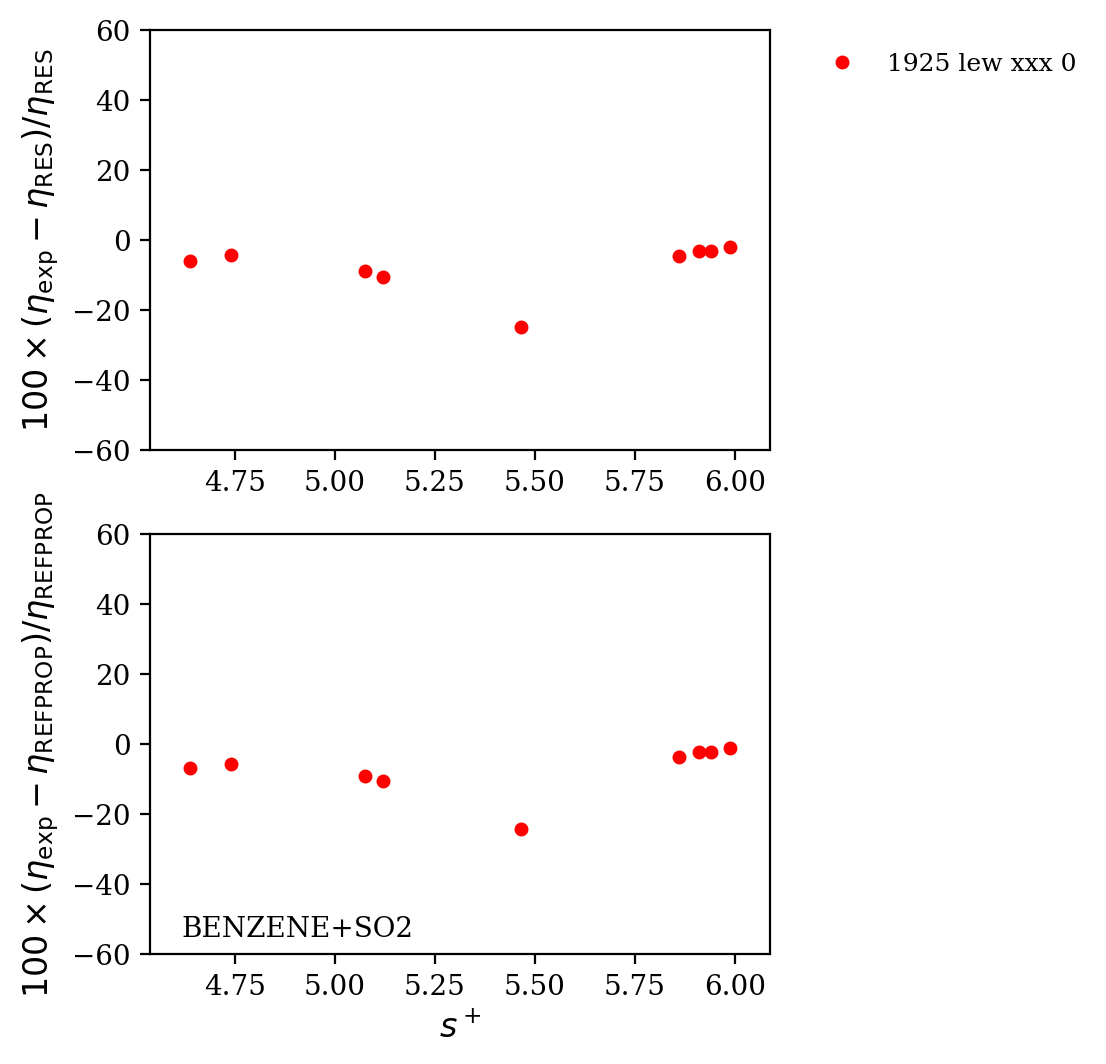

Supplement: Supplementary file 1 — je4c00451_si_001.zip [file je4c00451_si_001.zip › supporting_information/mix_dev_exp_res_ecs/BENZENE+SO2.png]

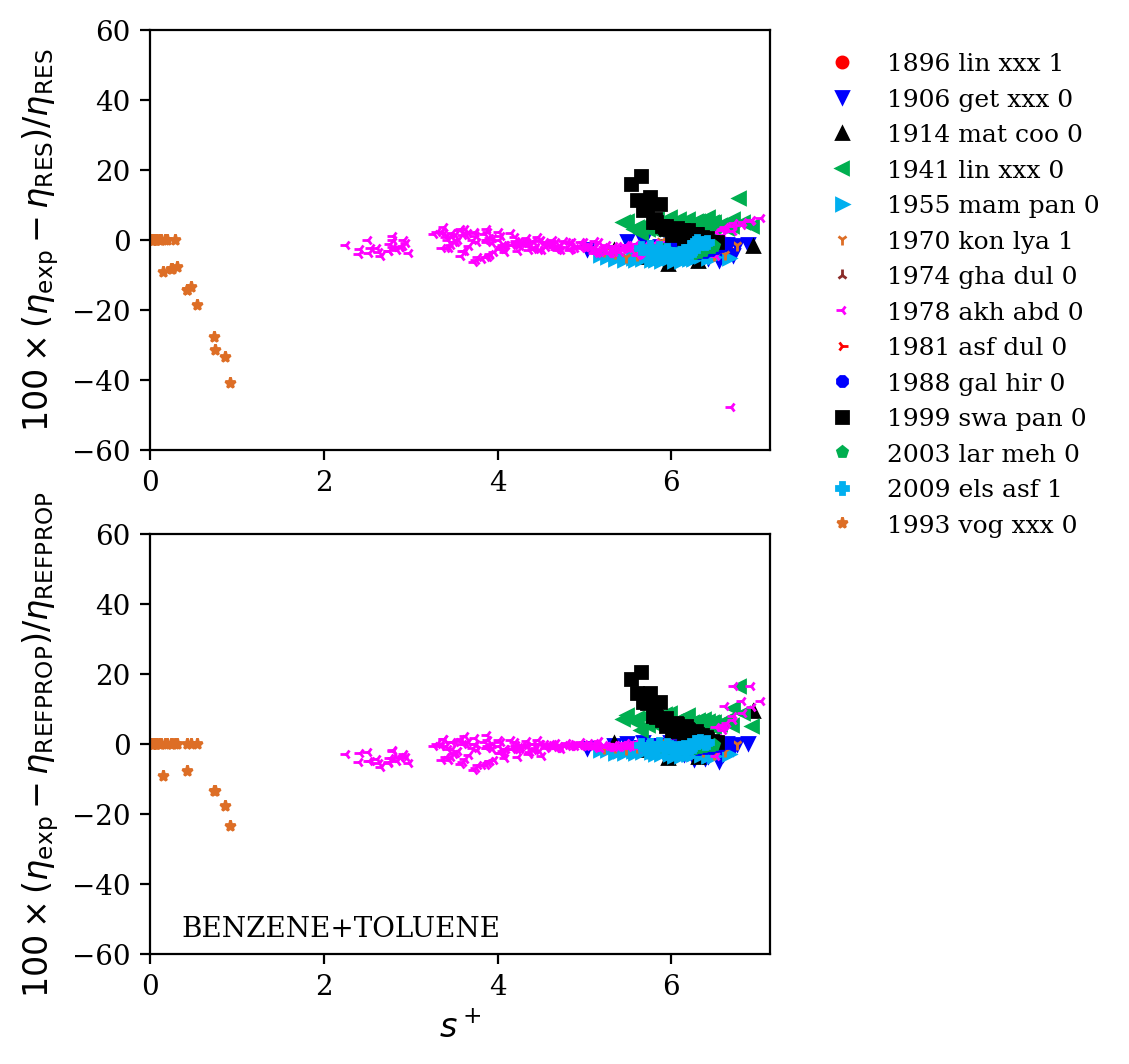

Supplement: Supplementary file 1 — je4c00451_si_001.zip [file je4c00451_si_001.zip › supporting_information/mix_dev_exp_res_ecs/BENZENE+TOLUENE.png]

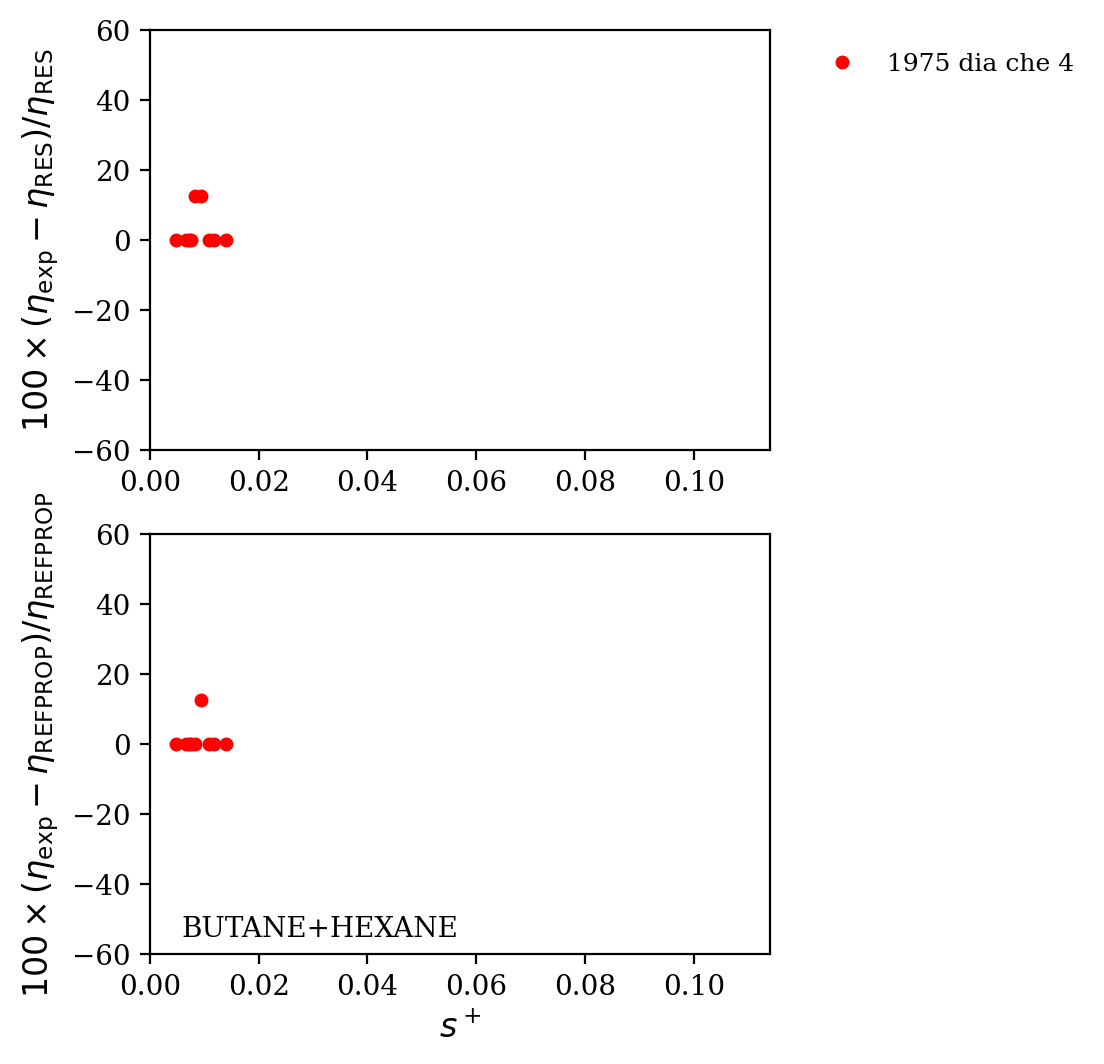

Supplement: Supplementary file 1 — je4c00451_si_001.zip [file je4c00451_si_001.zip › supporting_information/mix_dev_exp_res_ecs/BUTANE+HEXANE.png]

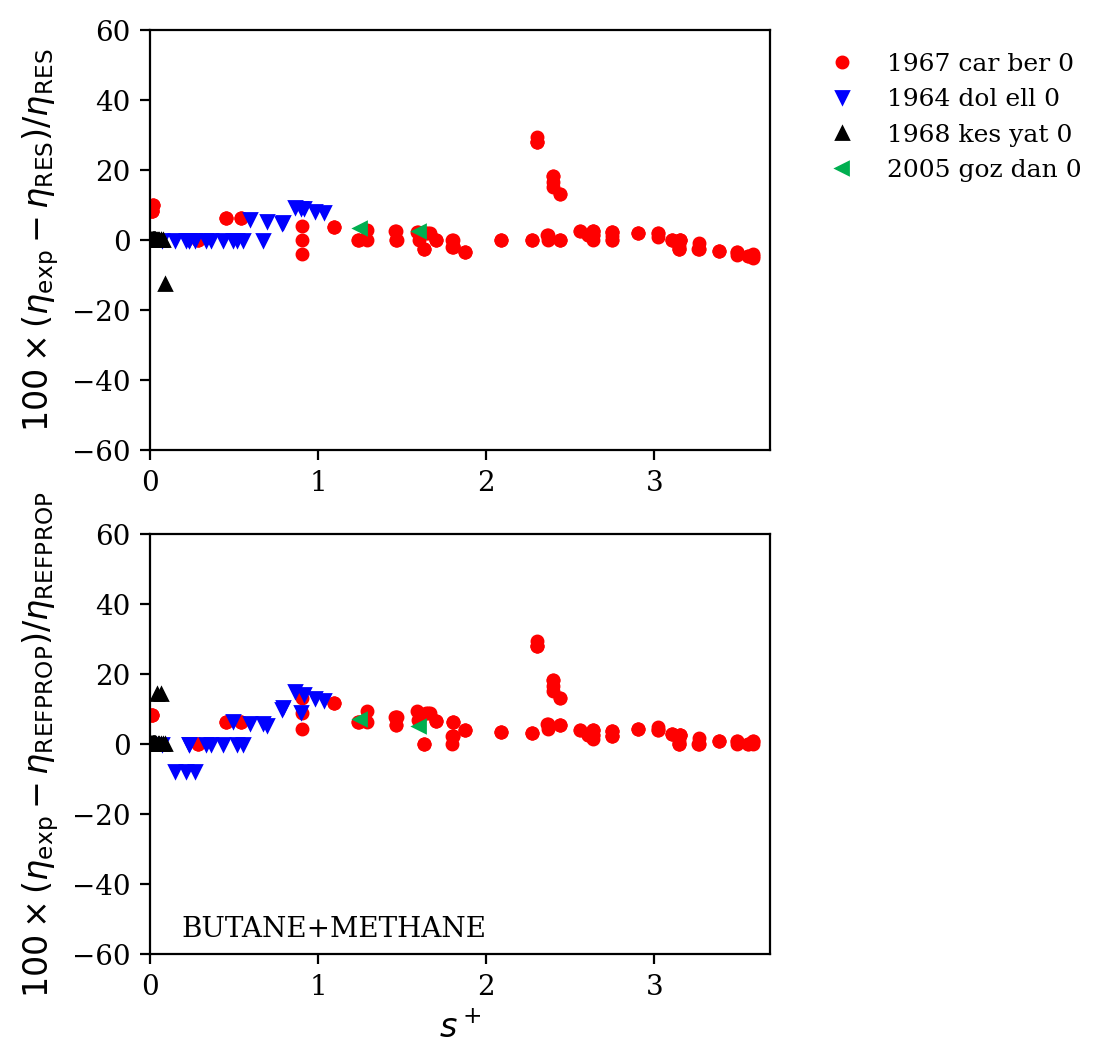

Supplement: Supplementary file 1 — je4c00451_si_001.zip [file je4c00451_si_001.zip › supporting_information/mix_dev_exp_res_ecs/BUTANE+METHANE.png]

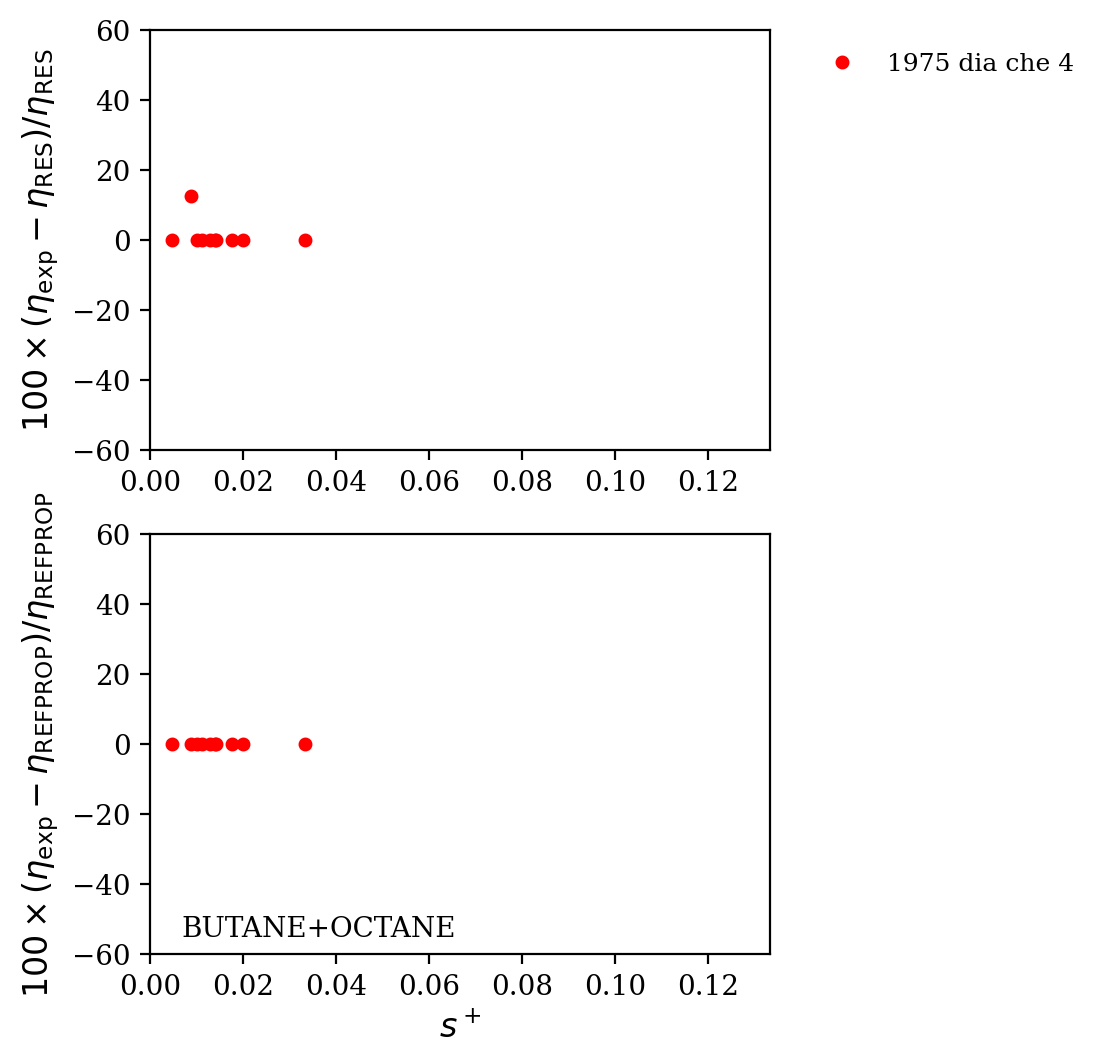

Supplement: Supplementary file 1 — je4c00451_si_001.zip [file je4c00451_si_001.zip › supporting_information/mix_dev_exp_res_ecs/BUTANE+OCTANE.png]

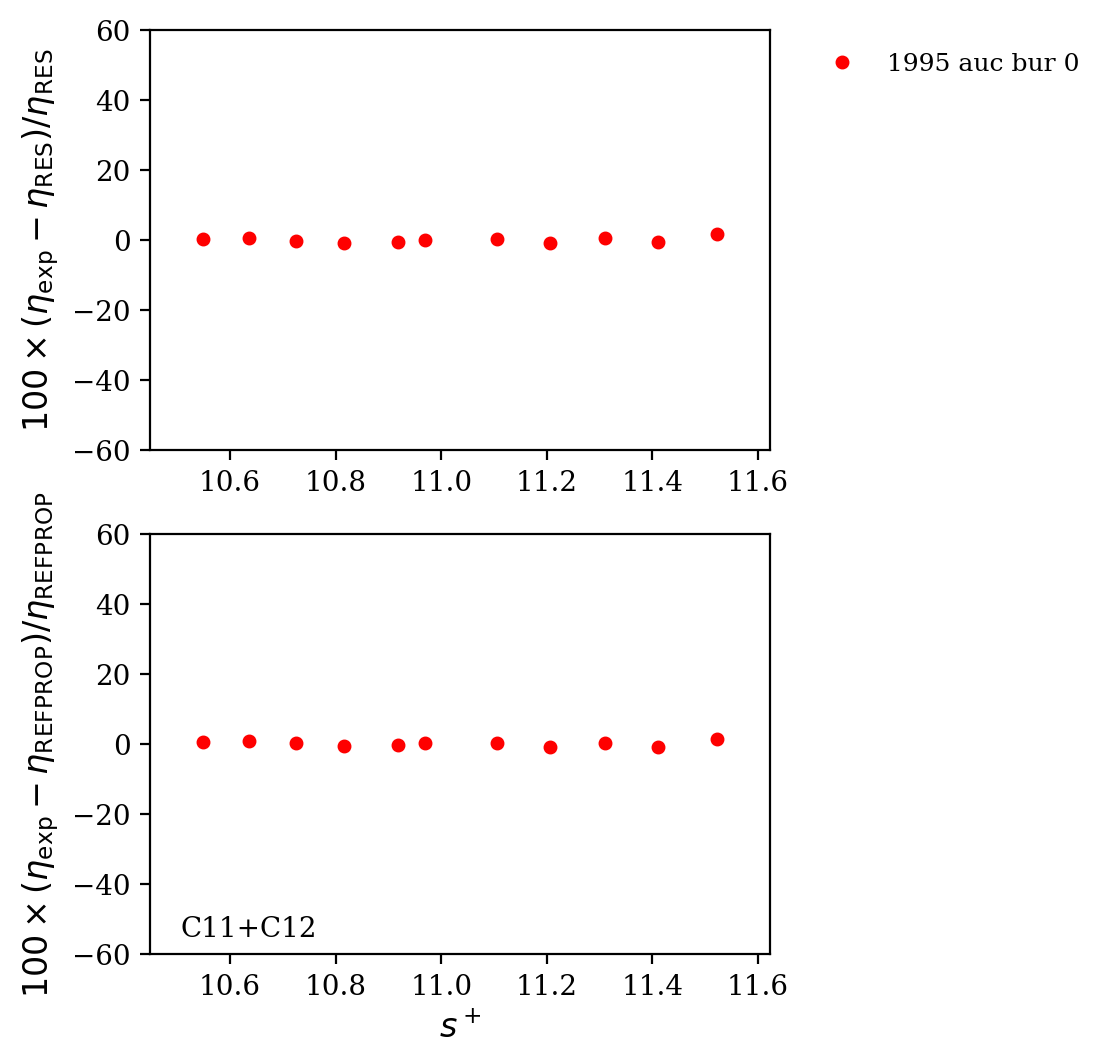

Supplement: Supplementary file 1 — je4c00451_si_001.zip [file je4c00451_si_001.zip › supporting_information/mix_dev_exp_res_ecs/C11+C12.png]

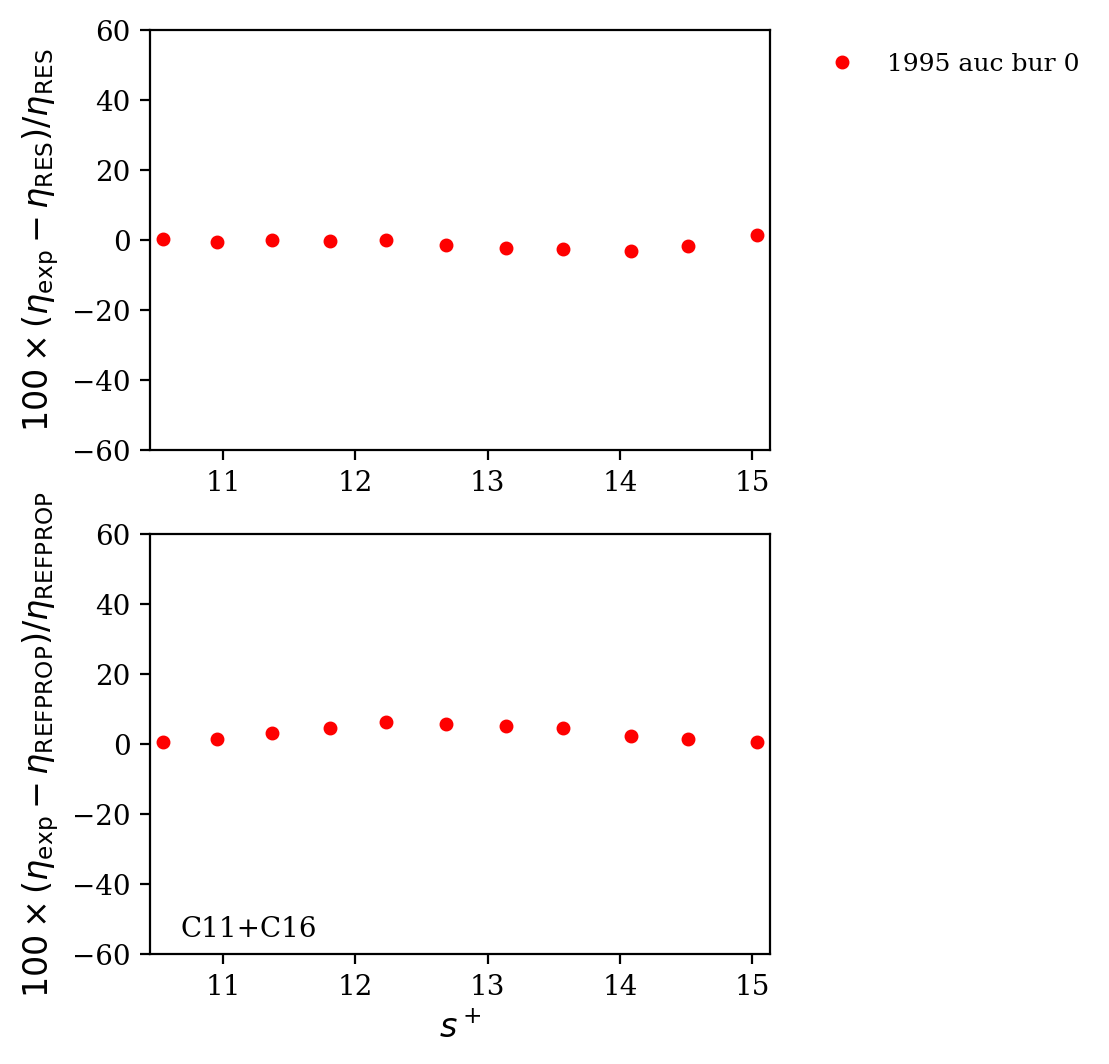

Supplement: Supplementary file 1 — je4c00451_si_001.zip [file je4c00451_si_001.zip › supporting_information/mix_dev_exp_res_ecs/C11+C16.png]

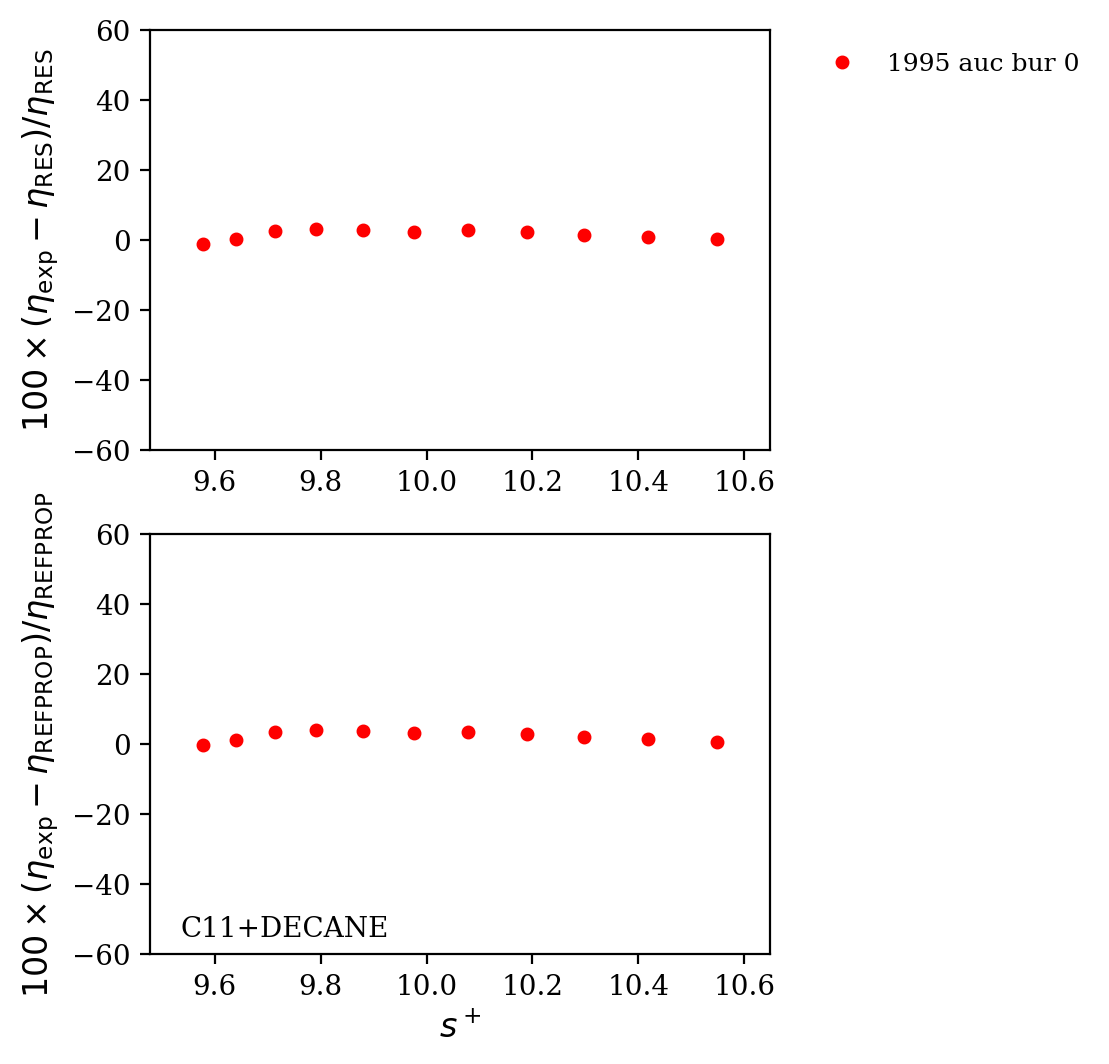

Supplement: Supplementary file 1 — je4c00451_si_001.zip [file je4c00451_si_001.zip › supporting_information/mix_dev_exp_res_ecs/C11+DECANE.png]

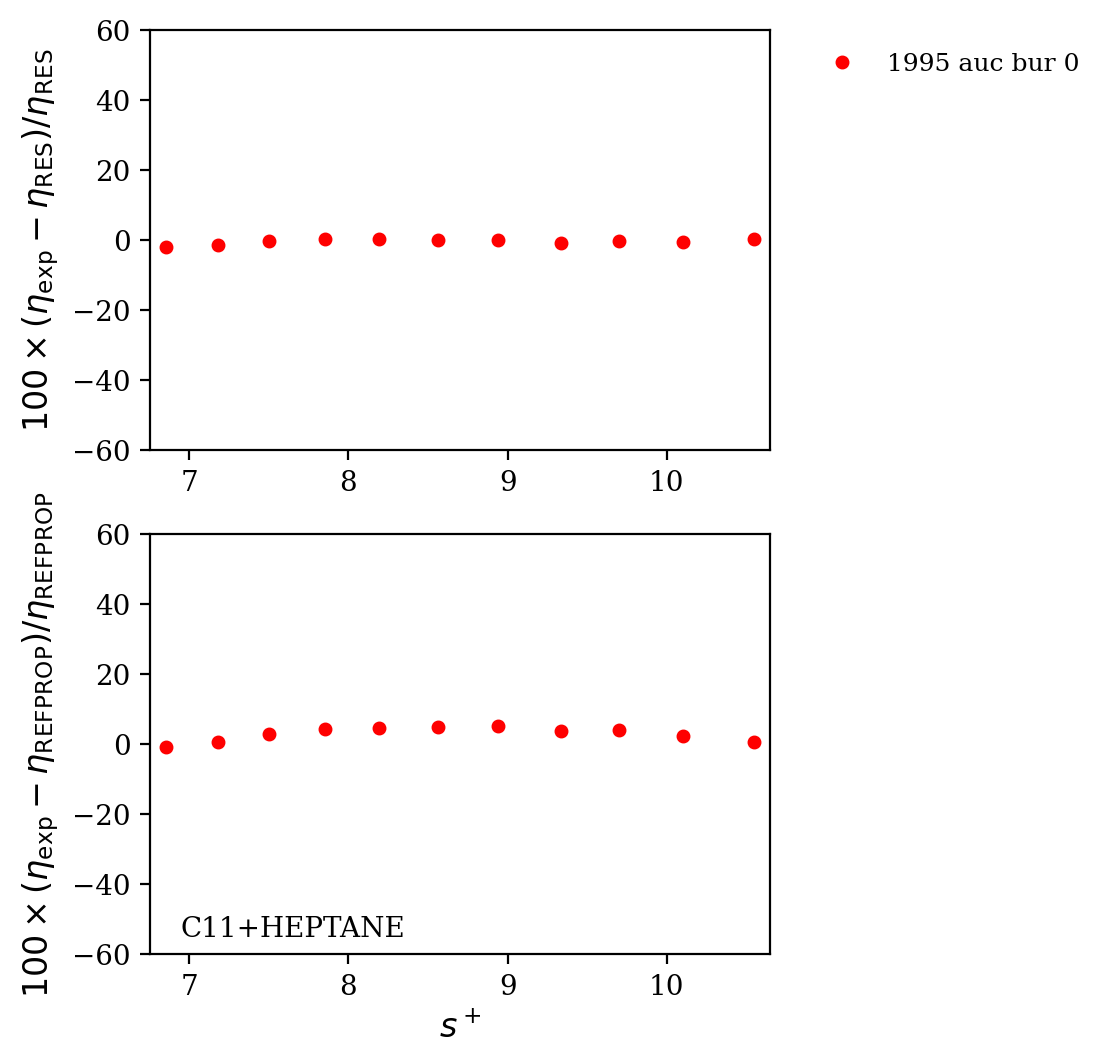

Supplement: Supplementary file 1 — je4c00451_si_001.zip [file je4c00451_si_001.zip › supporting_information/mix_dev_exp_res_ecs/C11+HEPTANE.png]

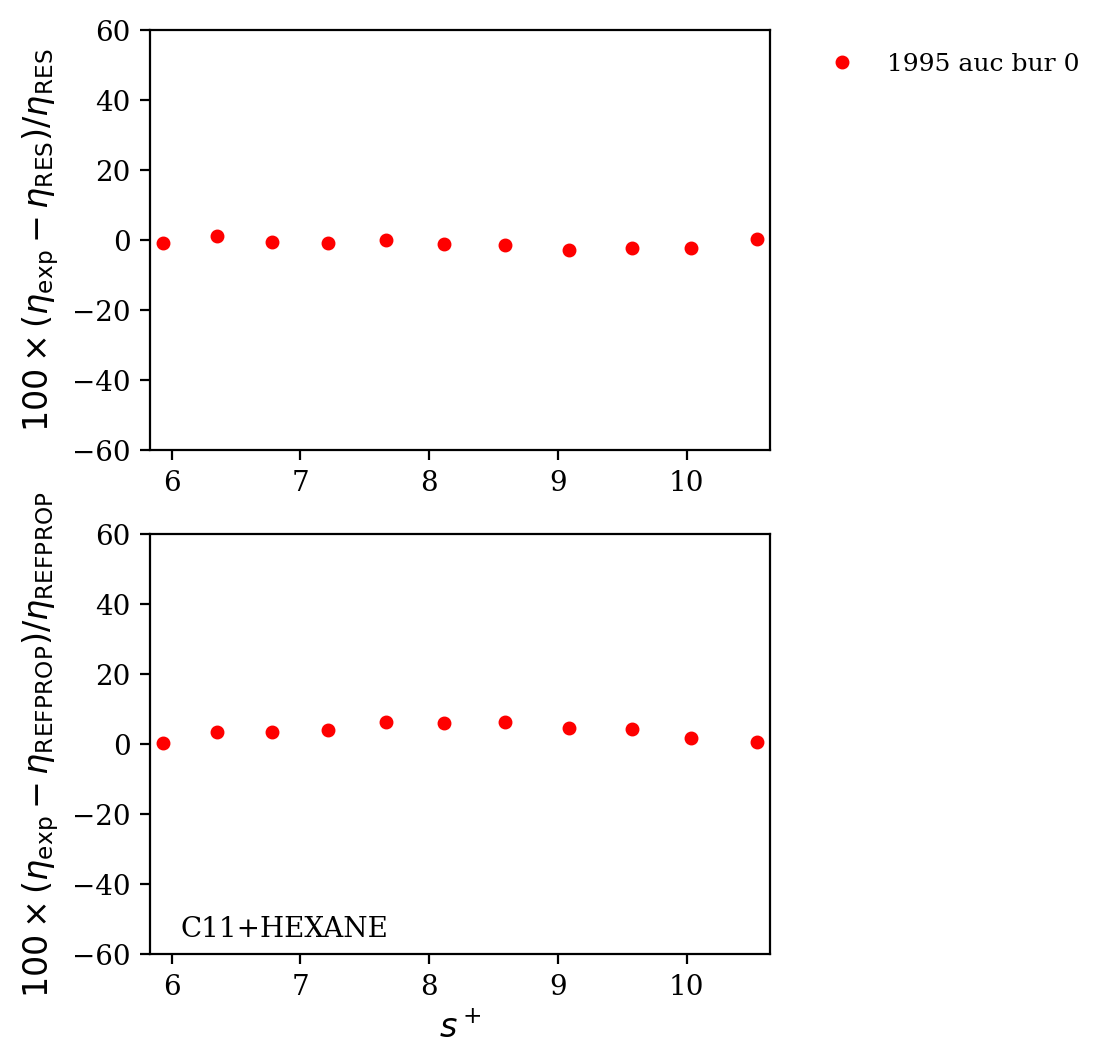

Supplement: Supplementary file 1 — je4c00451_si_001.zip [file je4c00451_si_001.zip › supporting_information/mix_dev_exp_res_ecs/C11+HEXANE.png]

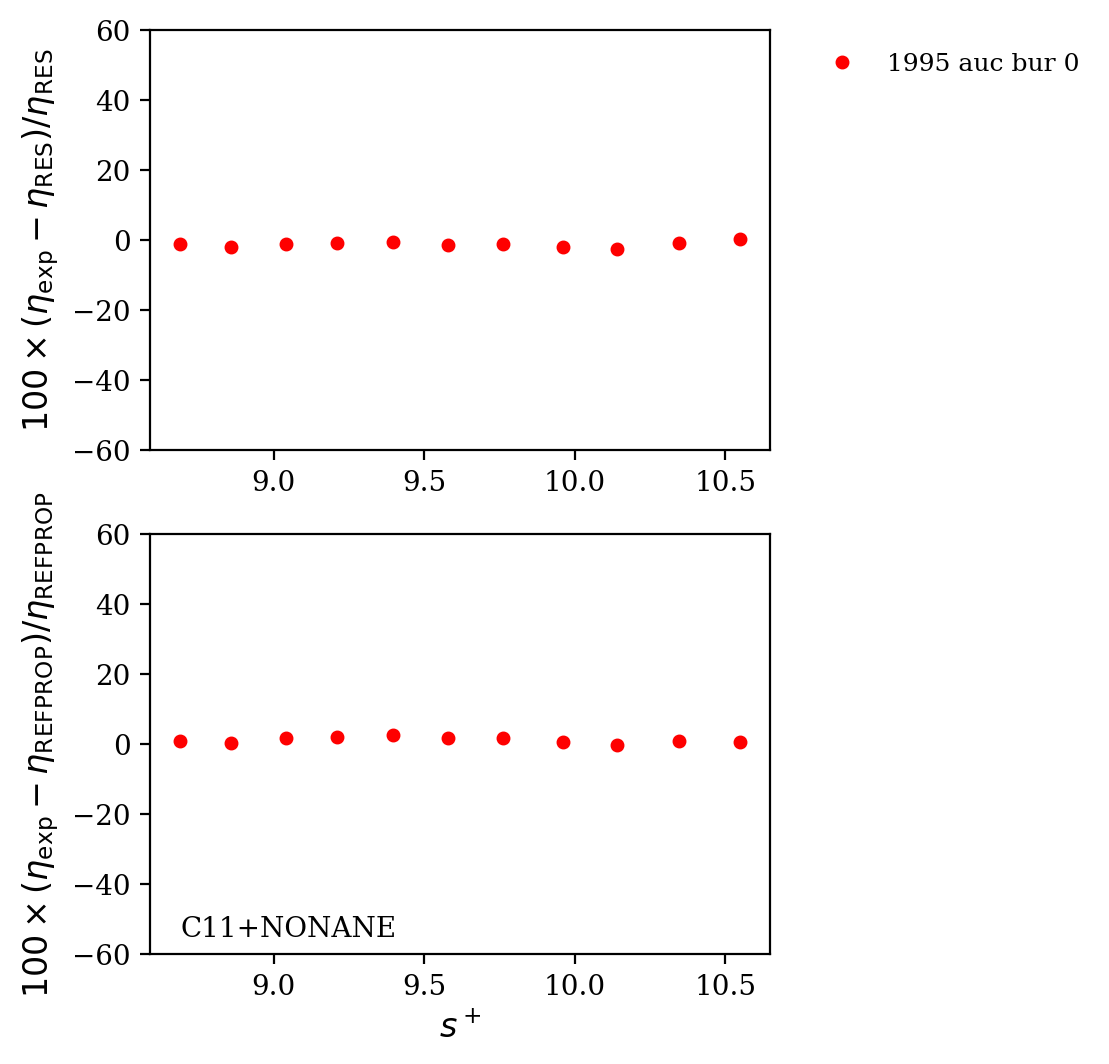

Supplement: Supplementary file 1 — je4c00451_si_001.zip [file je4c00451_si_001.zip › supporting_information/mix_dev_exp_res_ecs/C11+NONANE.png]

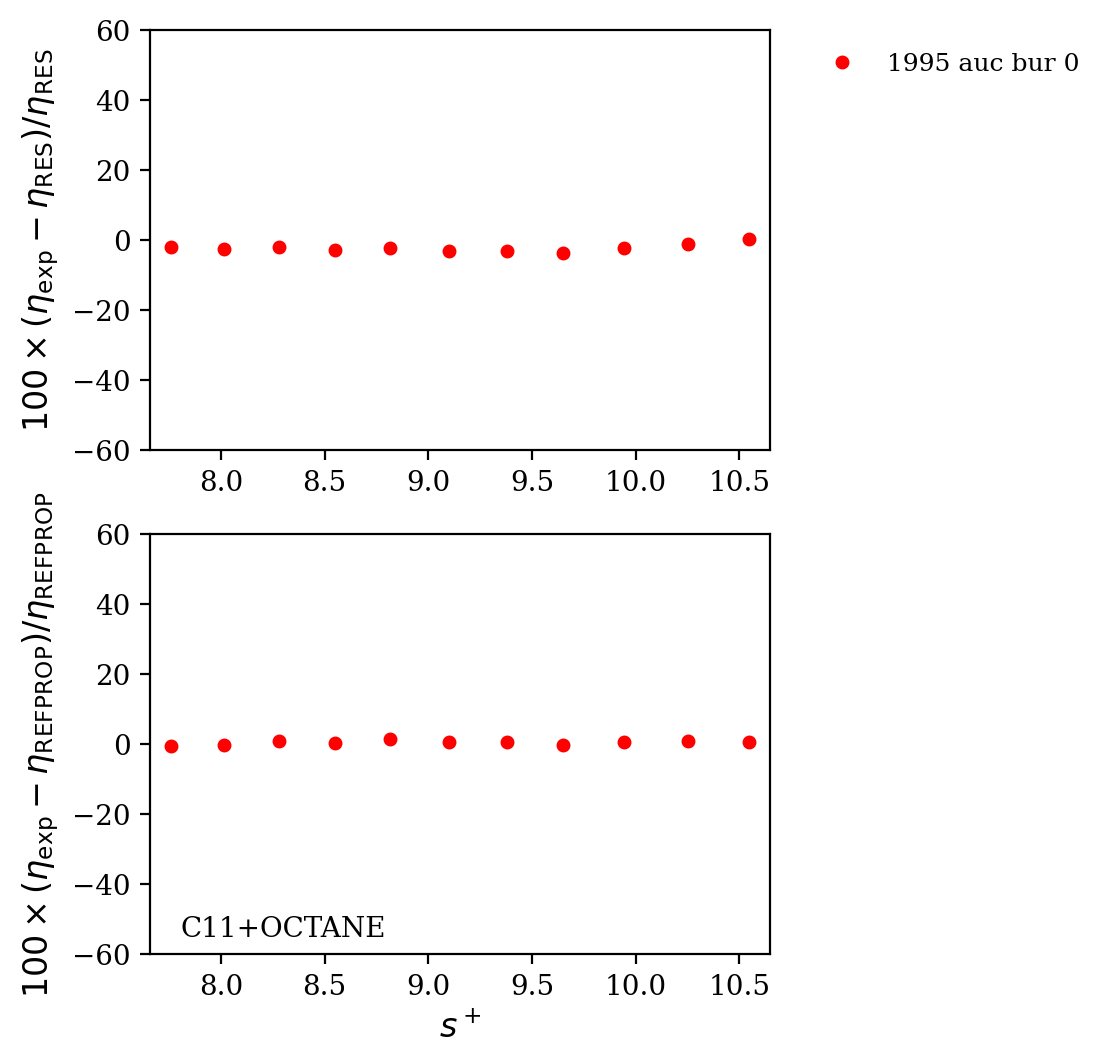

Supplement: Supplementary file 1 — je4c00451_si_001.zip [file je4c00451_si_001.zip › supporting_information/mix_dev_exp_res_ecs/C11+OCTANE.png]

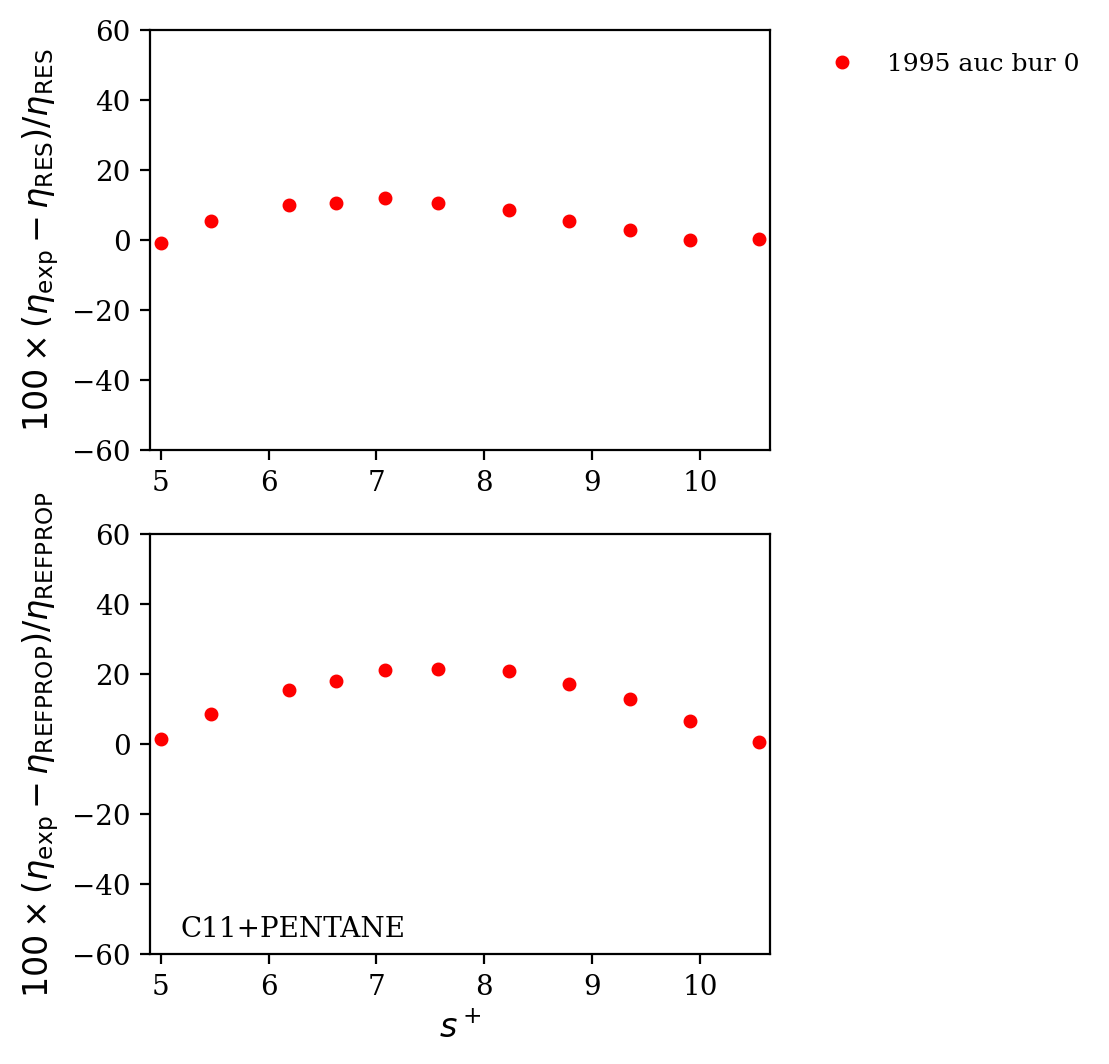

Supplement: Supplementary file 1 — je4c00451_si_001.zip [file je4c00451_si_001.zip › supporting_information/mix_dev_exp_res_ecs/C11+PENTANE.png]

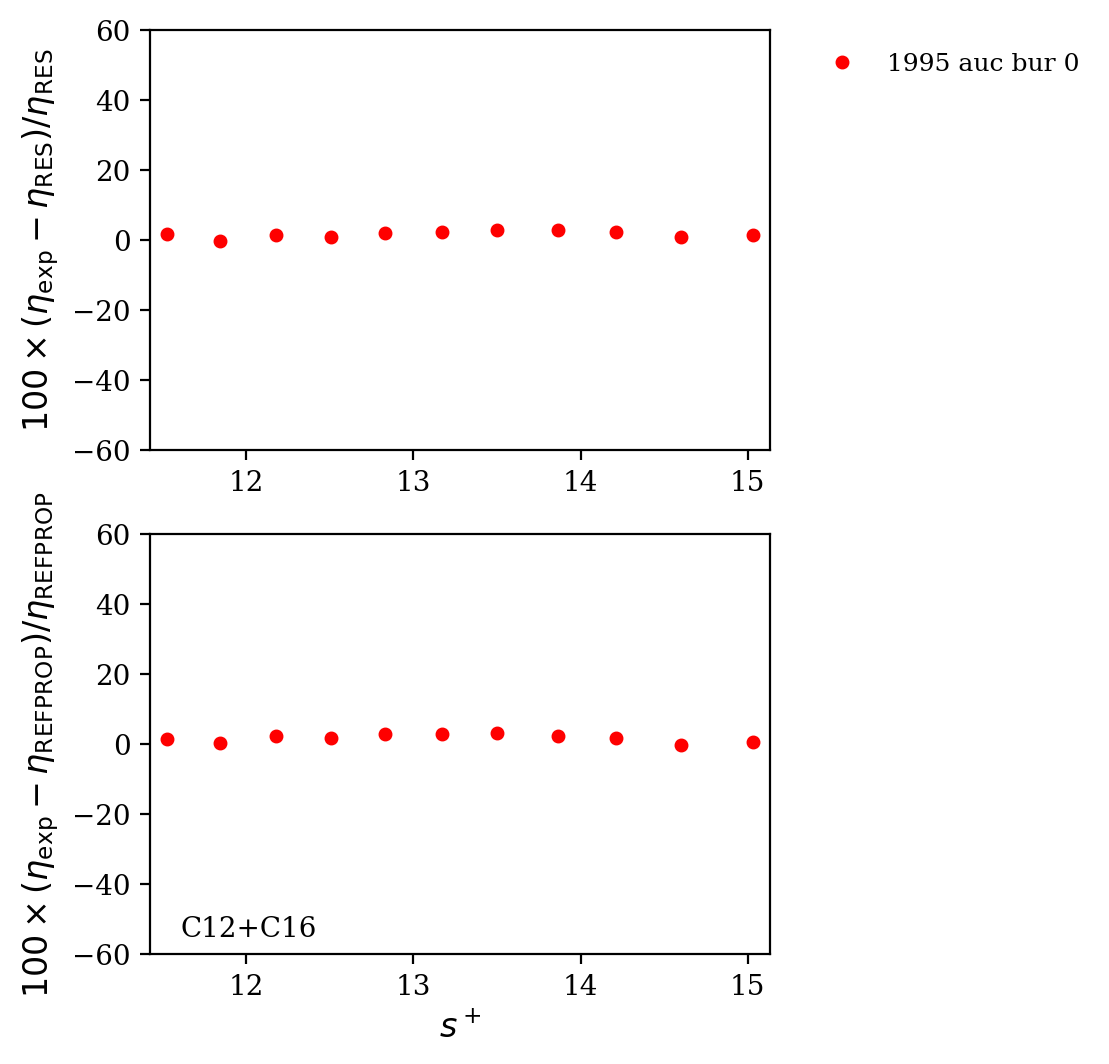

Supplement: Supplementary file 1 — je4c00451_si_001.zip [file je4c00451_si_001.zip › supporting_information/mix_dev_exp_res_ecs/C12+C16.png]

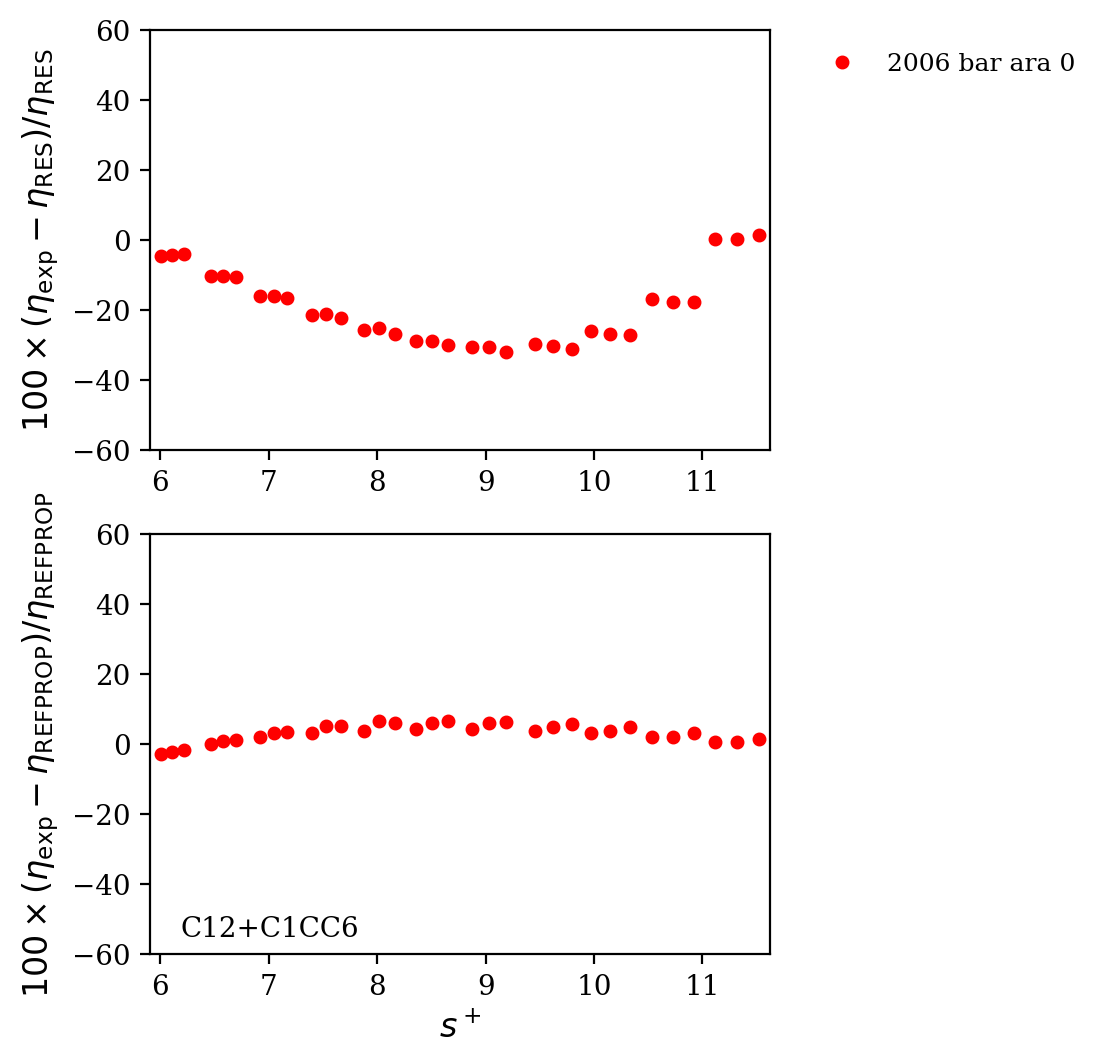

Supplement: Supplementary file 1 — je4c00451_si_001.zip [file je4c00451_si_001.zip › supporting_information/mix_dev_exp_res_ecs/C12+C1CC6.png]

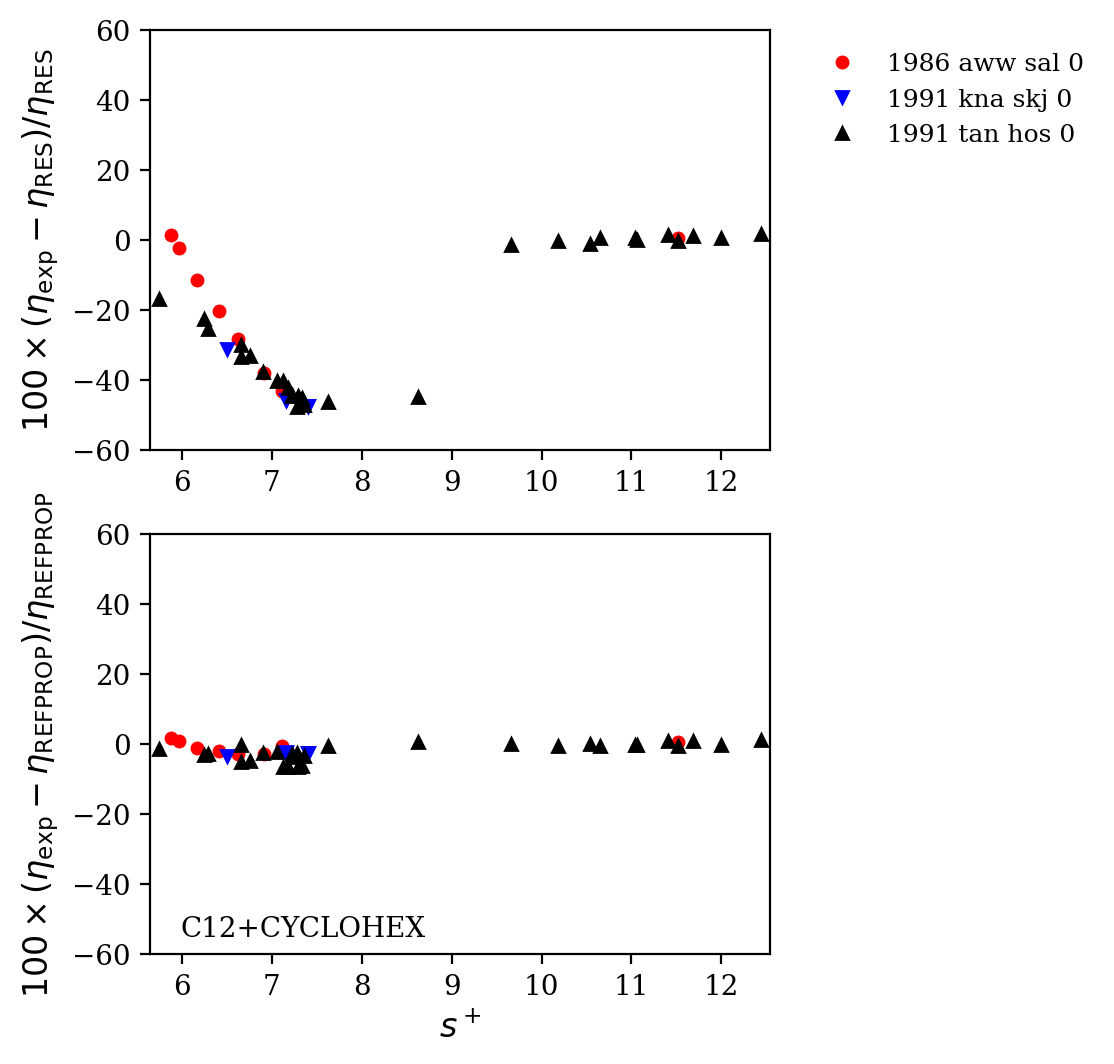

Supplement: Supplementary file 1 — je4c00451_si_001.zip [file je4c00451_si_001.zip › supporting_information/mix_dev_exp_res_ecs/C12+CYCLOHEX.png]

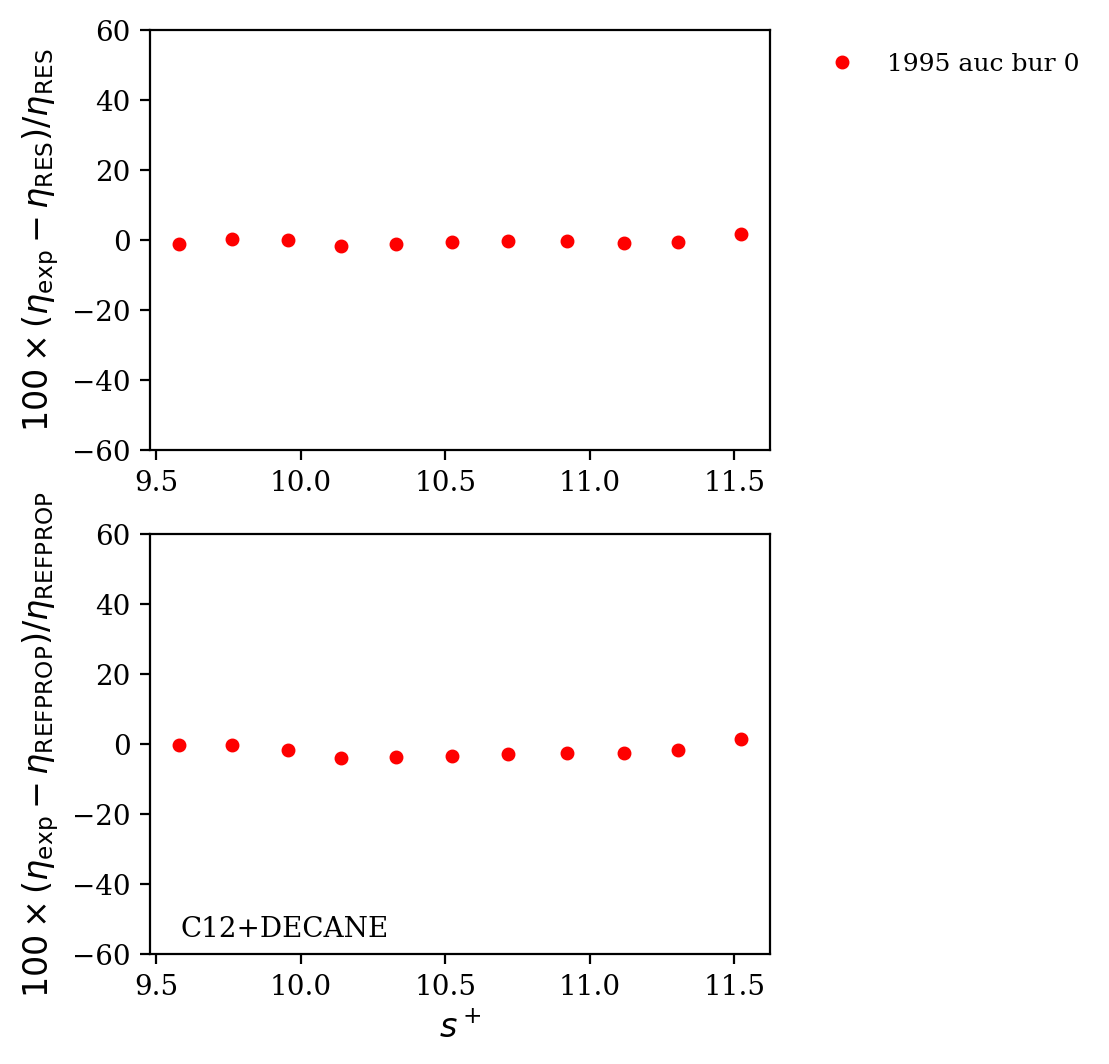

Supplement: Supplementary file 1 — je4c00451_si_001.zip [file je4c00451_si_001.zip › supporting_information/mix_dev_exp_res_ecs/C12+DECANE.png]

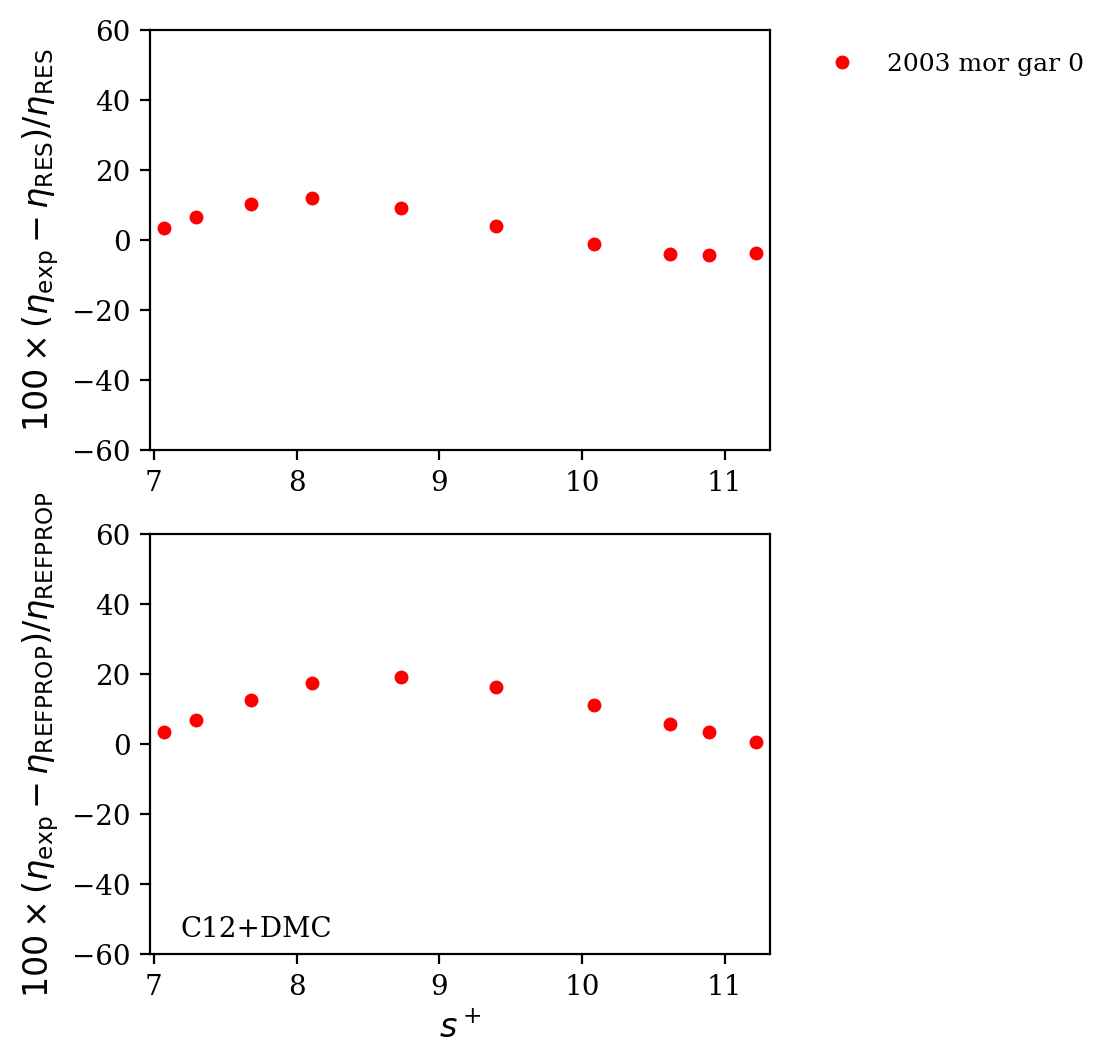

Supplement: Supplementary file 1 — je4c00451_si_001.zip [file je4c00451_si_001.zip › supporting_information/mix_dev_exp_res_ecs/C12+DMC.png]

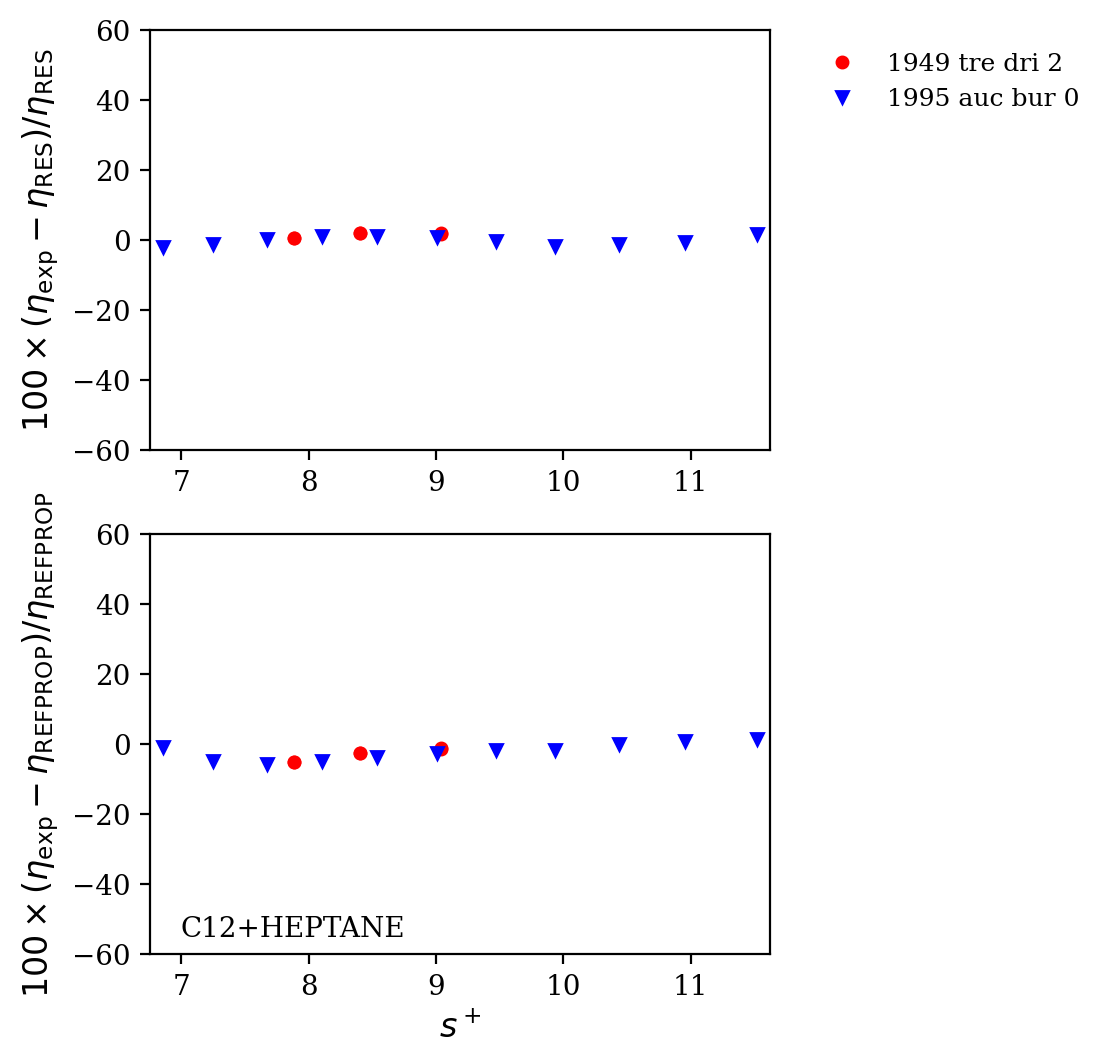

Supplement: Supplementary file 1 — je4c00451_si_001.zip [file je4c00451_si_001.zip › supporting_information/mix_dev_exp_res_ecs/C12+HEPTANE.png]

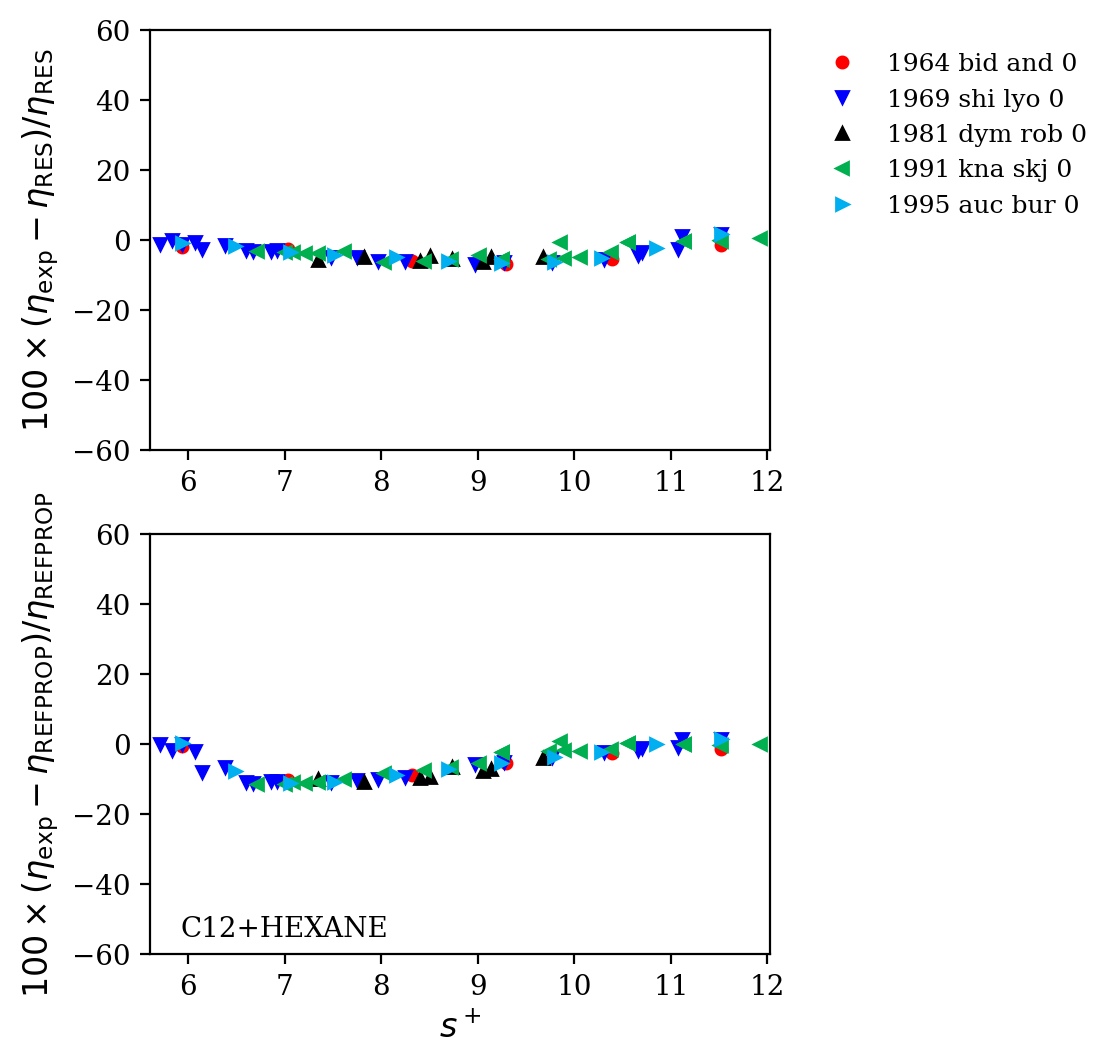

Supplement: Supplementary file 1 — je4c00451_si_001.zip [file je4c00451_si_001.zip › supporting_information/mix_dev_exp_res_ecs/C12+HEXANE.png]

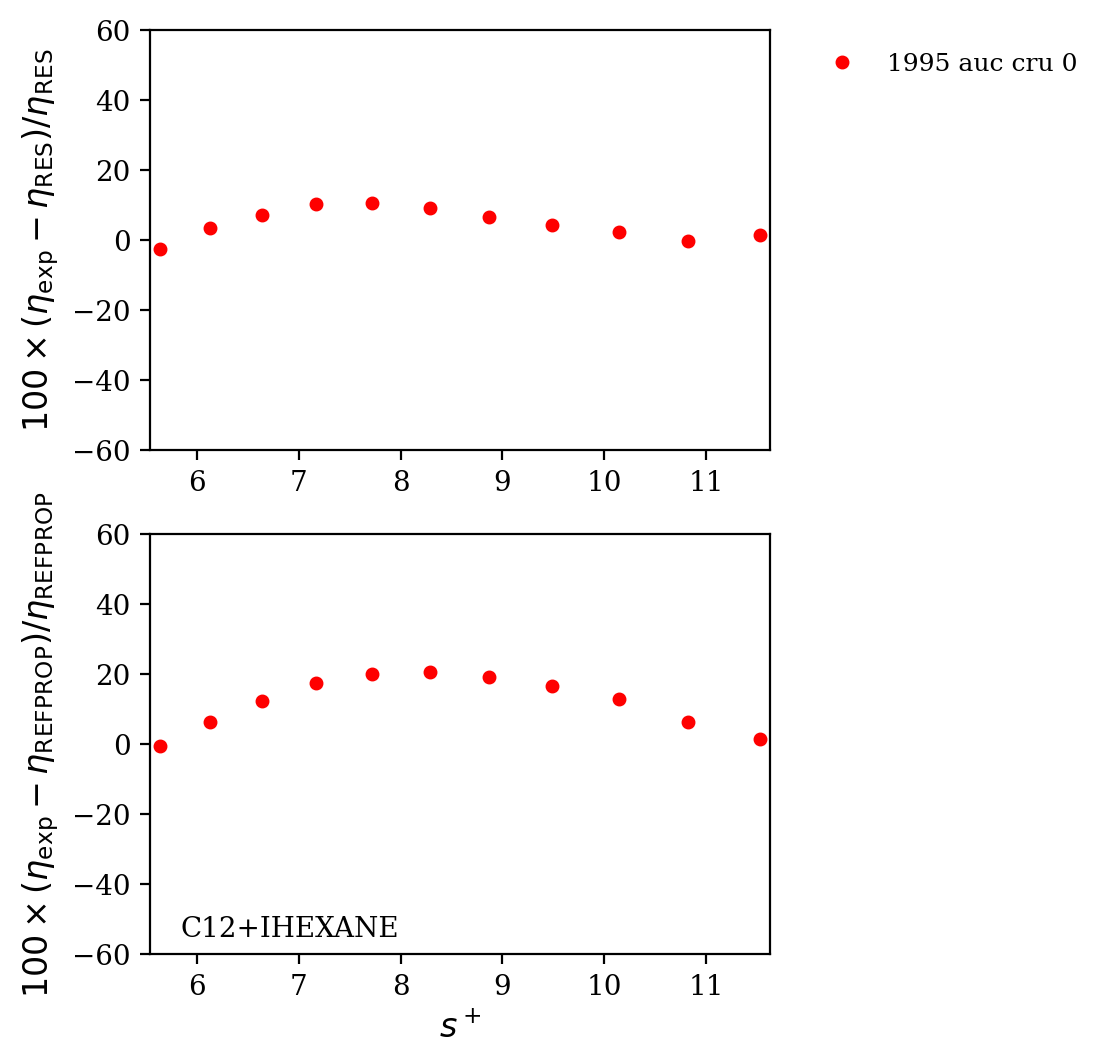

Supplement: Supplementary file 1 — je4c00451_si_001.zip [file je4c00451_si_001.zip › supporting_information/mix_dev_exp_res_ecs/C12+IHEXANE.png]

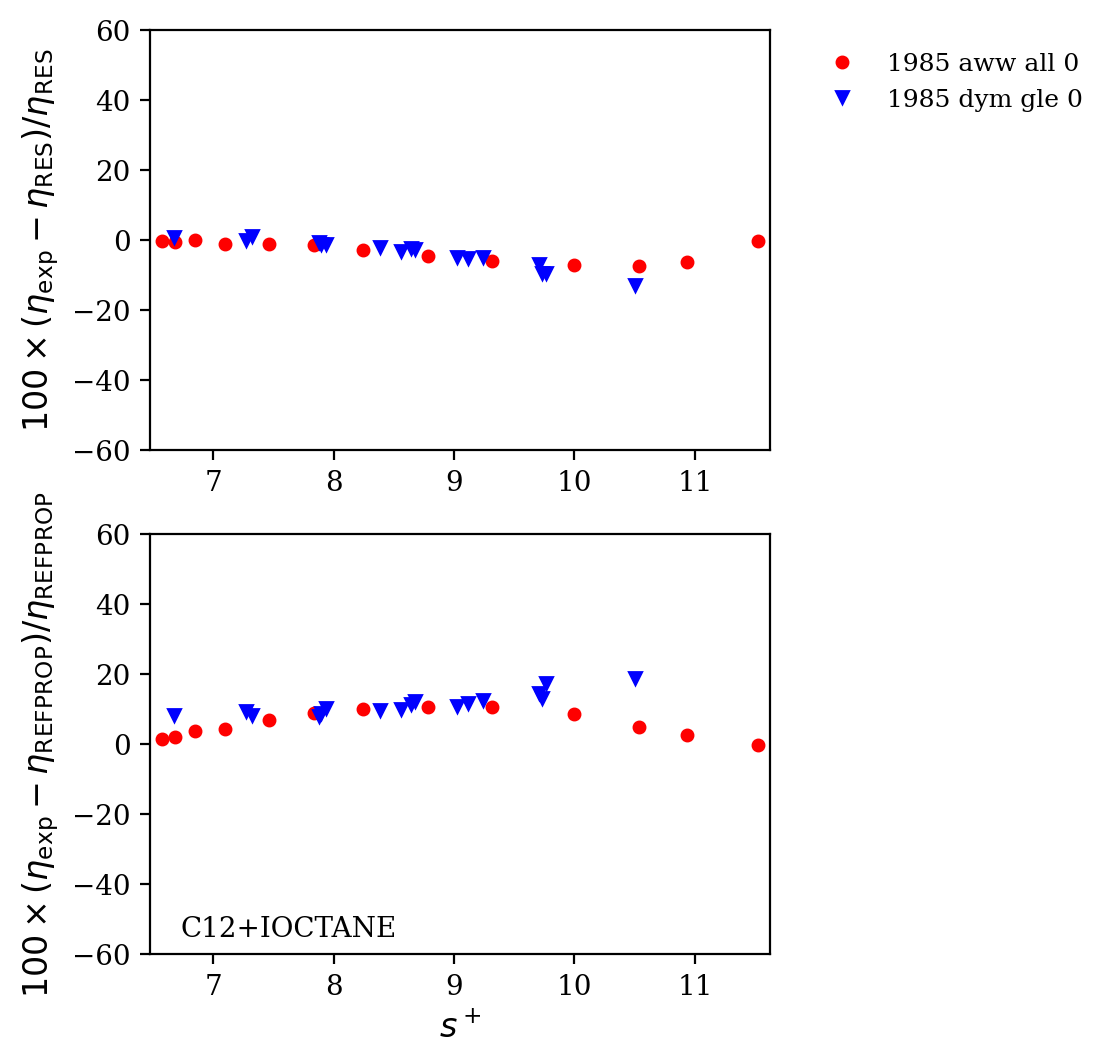

Supplement: Supplementary file 1 — je4c00451_si_001.zip [file je4c00451_si_001.zip › supporting_information/mix_dev_exp_res_ecs/C12+IOCTANE.png]

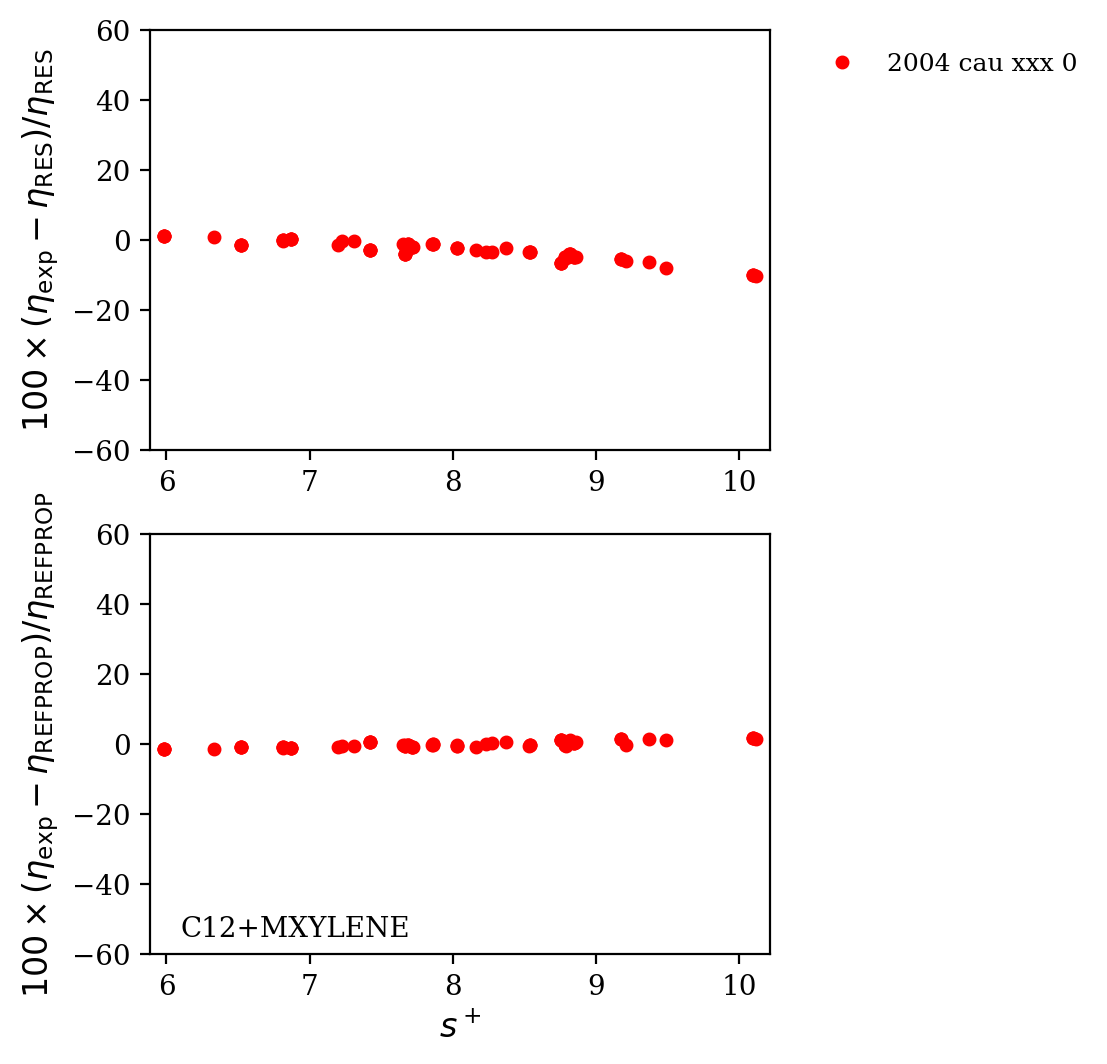

Supplement: Supplementary file 1 — je4c00451_si_001.zip [file je4c00451_si_001.zip › supporting_information/mix_dev_exp_res_ecs/C12+MXYLENE.png]

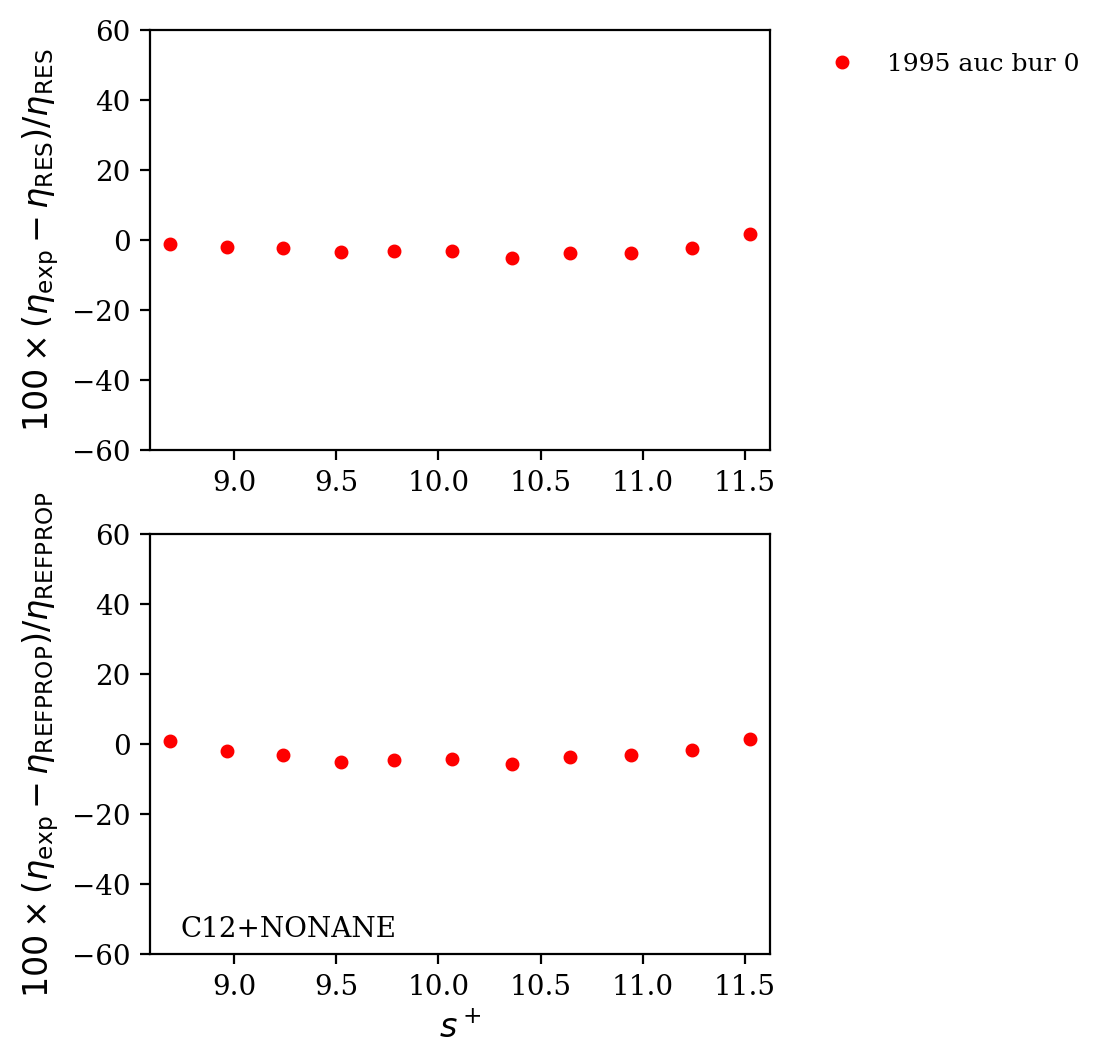

Supplement: Supplementary file 1 — je4c00451_si_001.zip [file je4c00451_si_001.zip › supporting_information/mix_dev_exp_res_ecs/C12+NONANE.png]

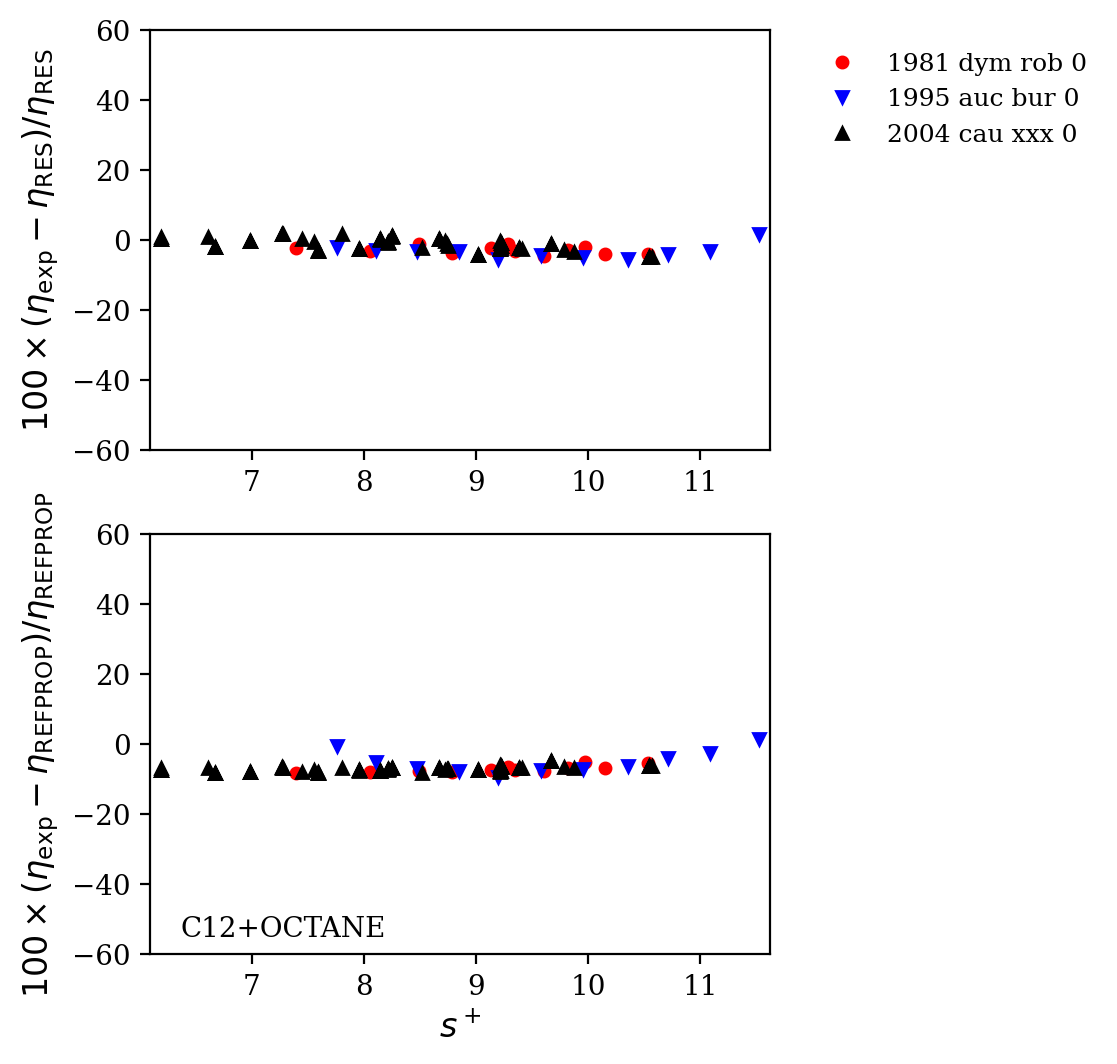

Supplement: Supplementary file 1 — je4c00451_si_001.zip [file je4c00451_si_001.zip › supporting_information/mix_dev_exp_res_ecs/C12+OCTANE.png]

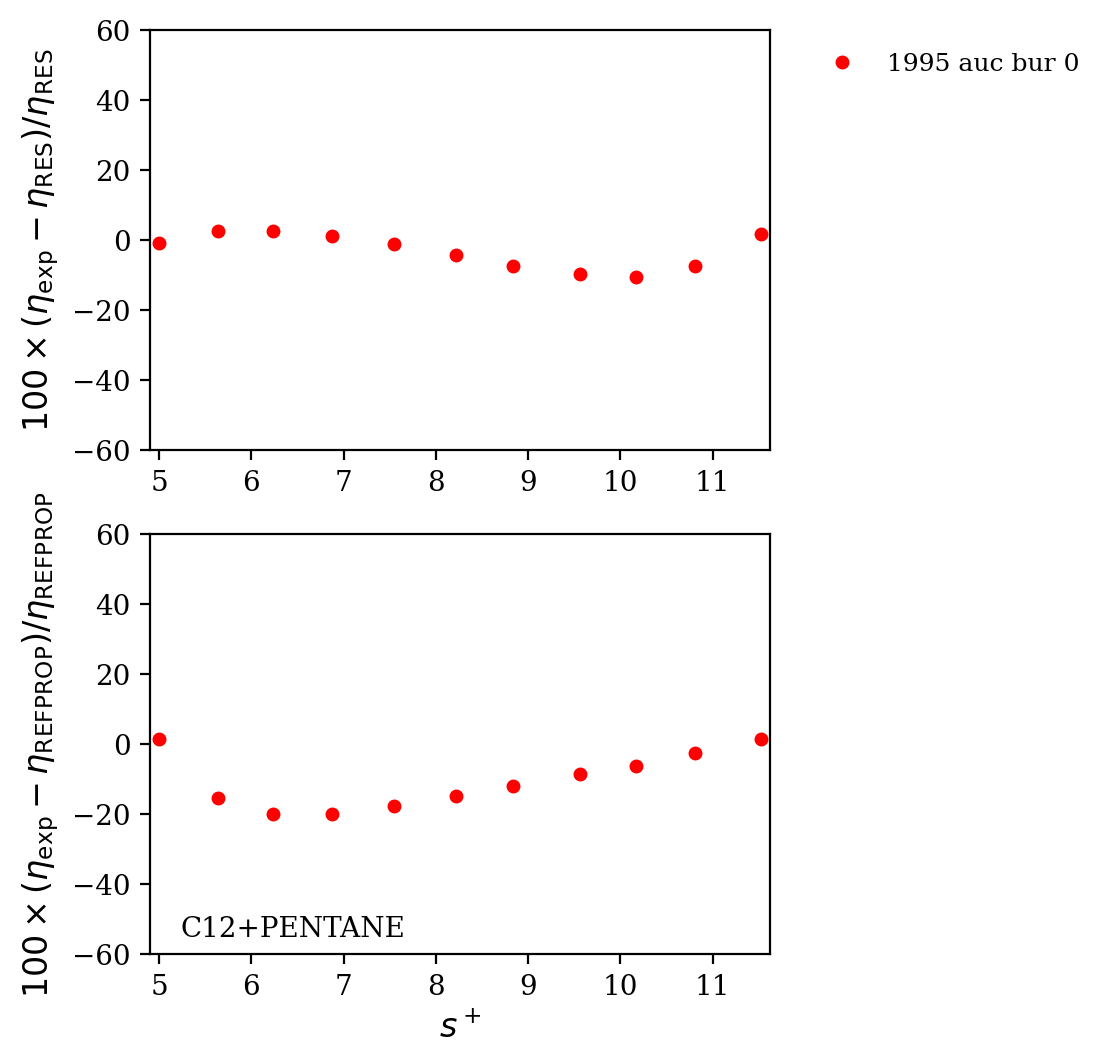

Supplement: Supplementary file 1 — je4c00451_si_001.zip [file je4c00451_si_001.zip › supporting_information/mix_dev_exp_res_ecs/C12+PENTANE.png]
