# Supplementary material for: Entropy Scaling of Viscosity IV—Application to 124 Industrially Important Fluids
Source: J Chem Eng Data. 2025 Jan 10;70(2):727–42. doi: 10.1021/acs.jced.4c00451 (PMC11831593; doi:10.1021/acs.jced.4c00451)
Supplement: Supplementary file 1 — je4c00451_si_001.zip [file je4c00451_si_001.zip › supporting_information/Pure fluid data and literature.docx]

| Material | Author | *N*_all_ | *N*_used_ | *T* / K | *p* / kPa |
| --- | --- | --- | --- | --- | --- |
| 13BUTADIENE | Edwards Bonilla, 1944 ^1^ | 15 | 15 | 258.2 - 333.2 | 65.0 - 726.0 |
| 13BUTADIENE | Neduzhii Khmara, 1968 ^2^ | 15 | 15 | 220.0 - 360.0 | 9.0 - 1333.0 |
| 13BUTADIENE | Senftleben Gladisch, 1949 ^3^ | 1 | 1 | 313.2 - 313.2 | 430.0 - 430.0 |
| 13BUTADIENE | Dunlop, 1994 ^4^ | 1 | 1 | 298.2 - 298.2 | 277.0 - 277.0 |
| 13BUTADIENE | Senftleben Gladisch, 1949 ^3^ | 1 | 1 | 313.2 - 313.2 | 430.0 - 430.0 |
| 13BUTADIENE | Liessmann et al, 1995 ^5^ | 11 | 11 | 233.2 - 383.2 | 20.0 - 2106.0 |
| 13BUTADIENE | Vargaftik, 1972 ^6^ | 9 | 9 | 253.2 - 333.2 | 52.0 - 726.0 |
| 13BUTADIENE | Scholz Kley, 1981^7^ | 4 | 4 | 308.2 - 351.8 | 374.0 - 1117.0 |
| 13BUTADIENE | Golubev, 1959^8^ | 6 | 6 | 283.1 - 333.1 | 170.0 - 725.0 |

| Material | Author | *N*_all_ | *N*_used_ | *T* / K | *p* / kPa |
| --- | --- | --- | --- | --- | --- |
| 1BUTENE | Liessmann, 1995 ^5^ | 9 | 9 | 188.2 - 388.2 | 1.0 - 2371.0 |
| 1BUTENE | Vargaftik, 1972 ^6^ | 9 | 9 | 273.2 - 573.2 | 100.0 - 100.0 |
| 1BUTENE | Scholz Kley, 1981^7^ | 23 | 23 | 183.2 - 403.2 | 1.0 - 3075.0 |
| 1BUTENE | Senftleben Gladisch, 1949 ^3^ | 1 | 1 | 313.2 - 313.2 | 454.0 - 454.0 |
| 1BUTENE | Scholz Kley, 1981^7^ | 6 | 6 | 293.2 - 393.2 | 252.0 - 2590.0 |
| 1BUTENE | Titani, 1930 ^9^ | 7 | 7 | 273.2 - 393.1 | 101.0 - 101.0 |

| Material | Author | | *N*_all_ | *N*_used_ | | *T* / K | | *p* / kPa |
| --- | --- | --- | --- | --- | --- | --- | --- | --- |
| 22DIMETHYLBUTANE | Smutny Bondi, 1961^10^ | 1 | 1 | | 293.2 - 293.2 | | 37.0 - 37.0 | |
| 22DIMETHYLBUTANE | Brazier Freeman, 1969 ^11^ | 9 | 9 | | 303.2 - 303.2 | | 100.0 - 400000.0 | |
| 22DIMETHYLBUTANE | Lambert et al, 1955 ^12^ | 4 | 4 | | 323.2 - 351.2 | | 50.0 - 50.0 | |
| 22DIMETHYLBUTANE | Chavane Van, 1922 ^13^ | 5 | 5 | | 273.2 - 303.2 | | 16.0 - 54.0 | |
| 22DIMETHYLBUTANE | Aucejo et al, 1955 ^14^ | 1 | 1 | | 298.2 - 298.2 | | 44.0 - 44.0 | |
| 22DIMETHYLBUTANE | Kuss Pollmann, 1969 ^15^ | 6 | 6 | | 313.2 - 313.2 | | 98.0 - 147100.0 | |
| 22DIMETHYLBUTANE | Diaz Pena et al, 1967 ^16^ | 13 | 13 | | 303.4 - 423.4 | | 54.0 - 1115.0 | |
| 22DIMETHYLBUTANE | McCall et al, 1959 ^17^ | 1 | 1 | | 298.2 - 298.2 | | 44.0 - 44.0 | |
| 22DIMETHYLBUTANE | Touazi et al, 2018 ^18^ | 7 | 7 | | 288.2 - 318.2 | | 8568.0 - 12364.0 | |
| 22DIMETHYLBUTANE | Liessmann et al, 1995 ^5^ | 10 | 4 | | 253.2 - 313.2 | | 6.0 - 76.0 | |
| 22DIMETHYLBUTANE | Scholz Kley, 1981 ^7^ | 4 | 4 | | 323.2 - 351.2 | | 105.0 - 235.0 | |
| 22DIMETHYLBUTANE | Chavane Van, 1922 ^13^ | 5 | 5 | | 273.2 - 303.1 | | 101.0 - 101.0 | |
| 22DIMETHYLBUTANE | Brazier Freemann, 1969 ^11^ | 9 | 9 | | 303.1 - 303.1 | | 100.0 - 400000.0 | |
| 22DIMETHYLBUTANE | Aucejo et al, 1995 ^14^ | 1 | 1 | | 298.0 - 298.0 | | 101.0 - 101.0 | |
| 22DIMETHYLBUTANE | Lambert et al, 1955 ^12^ | 5 | 5 | | 323.1 - 351.1 | | 101.0 - 101.0 | |

| Material | Author | *N*_all_ | *N*_used_ | | *T* / K | *p* / kPa |
| --- | --- | --- | --- | --- | --- | --- |
| 3METHYLPENTANE | Neumann, 1995 ^19^ | 9 | 9 | 283.0 - 323.0 | | 9091.0 - 13725.0 |
| 3METHYLPENTANE | Gorbachev et al, 1989 ^20^ | 1 | 1 | 293.2 - 293.2 | | 21.0 - 21.0 |
| 3METHYLPENTANE | Chavane Van, 1922 ^13^ | 4 | 4 | 273.2 - 303.2 | | 9.0 - 32.0 |
| 3METHYLPENTANE | Eicher Zwolinski, 1972 ^21^ | 7 | 7 | 265.6 - 332.7 | | 6.0 - 92.0 |
| 3METHYLPENTANE | Aucejo et al, 1995 ^14^ | 1 | 1 | 298.2 - 298.2 | | 26.0 - 26.0 |
| 3METHYLPENTANE | Ling Willard, 1968 ^22^ | 18 | 0 | 10000.0 - 0.0 | | 1000000000.0 - 0.0 |
| 3METHYLPENTANE | Kuss Pollmann, 1969 ^15^ | 6 | 6 | 313.2 - 313.2 | | 98.0 - 147100.0 |
| 3METHYLPENTANE | Gramajo et al, 2009 ^23^ | 1 | 1 | 298.2 - 298.2 | | 12489.0 - 12489.0 |
| 3METHYLPENTANE | Diaz Pena et al, 1967 ^16^ | 13 | 13 | 303.2 - 423.2 | | 32.0 - 846.0 |
| 3METHYLPENTANE | McCall et al, 1959 ^17^ | 1 | 1 | 298.2 - 298.2 | | 26.0 - 26.0 |
| 3METHYLPENTANE | Tu et al, 2001 ^24^ | 1 | 1 | 298.2 - 298.2 | | 12160.0 - 12160.0 |
| 3METHYLPENTANE | Bouzas er al, 2000 ^25^ | 1 | 1 | 298.2 - 298.2 | | 101.0 - 101.0 |
| 3METHYLPENTANE | Touazi et al, 2018 ^18^ | 7 | 7 | 288.2 - 318.2 | | 9693.0 - 13031.0 |
| 3METHYLPENTANE | Wen et al, 2017 ^26^ | 72 | 72 | 273.1 - 343.2 | | 1000.0 - 40000.0 |
| 3METHYLPENTANE | Mohammod Oudah, 2015 ^27^ | 3 | 3 | 293.2 - 313.2 | | 101.0 - 101.0 |
| 3METHYLPENTANE | Liessmann et al,1995 ^5^ | 11 | 11 | 253.2 - 403.2 | | 3.0 - 568.0 |
| 3METHYLPENTANE | Chavane Van, 1922 ^13^ | 4 | 4 | 273.2 - 303.1 | | 101.0 - 101.0 |
| 3METHYLPENTANE | Aucejo et al, 1995 ^14^ | 1 | 1 | 298.0 - 298.0 | | 101.0 - 101.0 |
| 3METHYLPENTANE | Bouzas er al, 2000 ^25^ | 1 | 1 | 298.2 - 298.2 | | 101.0 - 101.0 |

| Material | Author | | *N*_all_ | | | *N*_used_ | | *T* / K | | *p* / kPa |
| --- | --- | --- | --- | --- | --- | --- | --- | --- | --- | --- |
| ACETONE | | Thorpe Rodger, 1894 ^28^ | | 13 | 13 | | 281.0 - 327.0 | | 12.0 - 86.0 | |
| ACETONE | | Batschinski,1913 ^29^ | | 6 | 6 | | 273.1 - 323.1 | | 8.0 - 74.0 | |
| ACETONE | | Bagdasaryan, 1964 ^30^ | | 12 | 9 | | 233.0 - 323.0 | | 1.0 - 74.0 | |
| ACETONE | | Dunstan, 1904 ^31^ | | 1 | 1 | | 298.1 - 298.1 | | 101.0 - 101.0 | |
| ACETONE | | Dunstan Hilditch, 1912 ^32^ | | 1 | 1 | | 298.1 - 298.1 | | 101.0 - 101.0 | |
| ACETONE | | Faust, 1912 ^33^ | | 15 | 15 | | 260.2 - 315.1 | | 101.0 - 101.0 | |
| ACETONE | | Weissenberger Piatti, 1924 ^34^ | | 1 | 1 | | 291.1 - 291.1 | | 101.0 - 101.0 | |
| ACETONE | | Weissenberger Schuster, 1924 ^35^ | | 1 | 1 | | 293.1 - 293.1 | | 101.0 - 101.0 | |
| ACETONE | | Lewis, 1925^36^ | | 1 | 1 | | 298.1 - 298.1 | | 101.0 - 101.0 | |
| ACETONE | | Timmermans Martin, 1928 ^37^ | | 2 | 2 | | 288.1 - 303.1 | | 101.0 - 101.0 | |
| ACETONE | | Bingham Brown, 1932 ^38^ | | 3 | 3 | | 283.1 - 303.1 | | 101.0 - 101.0 | |
| ACETONE | | Hughes Hartley,1933 ^39^ | | 1 | 1 | | 298.1 - 298.1 | | 101.0 - 101.0 | |
| ACETONE | | Reynolds Kraus, 1948 ^40^ | | 1 | 1 | | 298.1 - 298.1 | | 101.0 - 101.0 | |
| ACETONE | | Karr et al, 1951 ^41^ | | 1 | 1 | | 298.1 - 298.1 | | 101.0 - 101.0 | |
| ACETONE | | Jullander et al, 1955 ^42^ | | 1 | 1 | | 293.1 - 293.1 | | 101.0 - 101.0 | |
| ACETONE | | Ling Van Winkle, 1958 ^43^ | | 2 | 2 | | 303.1 - 328.1 | | 101.0 - 101.0 | |
| ACETONE | | Blank, 1968 ^44^ | | 1 | 1 | | 298.1 - 298.1 | | 101.0 - 101.0 | |
| ACETONE | | Konobeev Lyapin, 1970 ^45^ | | 2 | 2 | | 293.1 - 313.1 | | 101.0 - 101.0 | |
| ACETONE | | Campbell Kartzmark, 1973 ^46^ | | 1 | 1 | | 298.1 - 298.1 | | 101.0 - 101.0 | |
| ACETONE | | Terent eva et al, 1973 ^47^ | | 6 | 6 | | 223.2 - 273.2 | | 101.0 - 101.0 | |
| ACETONE | | Atoyan Mamedov, 1976 ^48^ | | 51 | 51 | | 273.1 - 473.1 | | 101.0 - 162120.0 | |
| ACETONE | | Hafez Hartland, 1976 ^49^ | | 3 | 3 | | 298.1 - 318.1 | | 101.0 - 101.0 | |
| ACETONE | | Kerchove De Vijlder, 1977 ^50^ | | 1 | 1 | | 296.0 - 296.0 | | 101.0 - 101.0 | |
| ACETONE | | Yadava Yadava, 1981 ^51^ | | 1 | 1 | | 307.3 - 307.3 | | 101.0 - 101.0 | |
| ACETONE | | Noda et al, 1982 ^52^ | | 1 | 1 | | 298.1 - 298.1 | | 101.0 - 101.0 | |
| ACETONE | | Prakash et al, 1990 ^53^ | | 3 | 3 | | 303.2 - 313.2 | | 101.0 - 101.0 | |
| ACETONE | | Schumpe Luehring, 1990 ^54^ | | 1 | 1 | | 293.2 - 293.2 | | 101.0 - 101.0 | |
| ACETONE | | Das Jha,1991 ^55^ | | 1 | 0 | | 10000.0 - 0.0 | | 1000000000.0 - 0.0 | |
| ACETONE | | Zhang Liu, 1991 ^56^ | | 21 | 20 | | 298.2 - 338.9 | | 100.0 - 29500.0 | |
| ACETONE | | Liu Luo, 1992 ^57^ | | 4 | 4 | | 293.2 - 313.2 | | 101.0 - 101.0 | |
| ACETONE | | Lorenzi et al, 1995 ^58^ | | 1 | 1 | | 298.2 - 298.2 | | 101.0 - 101.0 | |
| ACETONE | | Tsierkezos Molinou, 1999 ^59^ | | 1 | 1 | | 293.2 - 293.2 | | 101.0 - 101.0 | |
| ACETONE | | Kinart et al, 2002 ^60^ | | 3 | 3 | | 293.2 - 303.2 | | 101.0 - 101.0 | |
| ACETONE | | Peng Tu, 2002 ^61^ | | 1 | 1 | | 298.2 - 298.2 | | 101.0 - 101.0 | |
| ACETONE | | Govindarajan et al, 2003 ^62^ | | 5 | 5 | | 293.2 - 313.2 | | 101.0 - 101.0 | |
| ACETONE | | Agarwal Singh, 2004 ^63^ | | 1 | 1 | | 298.2 - 298.2 | | 101.0 - 101.0 | |
| ACETONE | | Jain Singh, 2004 ^64^ | | 1 | 1 | | 303.2 - 303.2 | | 101.0 - 101.0 | |
| ACETONE | | Chen et al, 2005 ^65^ | | 3 | 3 | | 288.2 - 308.2 | | 101.0 - 101.0 | |
| ACETONE | | Gonzalez et al, 2005 ^66^ | | 3 | 3 | | 293.2 - 303.2 | | 101.0 - 101.0 | |
| ACETONE | | Wang et al, 2005 ^67^ | | 1 | 1 | | 298.2 - 298.2 | | 101.0 - 101.0 | |
| ACETONE | | Gonzalez et al, 2006 ^68^ | | 3 | 3 | | 293.2 - 303.2 | | 101.0 - 101.0 | |
| ACETONE | | Ren et al, 2007 ^69^ | | 1 | 1 | | 298.2 - 298.2 | | 101.0 - 101.0 | |
| ACETONE | | Sih et al, 2008 ^70^ | | 4 | 4 | | 298.2 - 313.2 | | 100.0 - 100.0 | |
| ACETONE | | Tian et al, 2008 ^71^ | | 1 | 1 | | 298.2 - 298.2 | | 101.0 - 101.0 | |
| ACETONE | | Rajapogal Chenthilnath, 2010 ^72^ | | 3 | 3 | | 298.2 - 308.2 | | 101.0 - 101.0 | |
| ACETONE | | Rajapogal Chenthilnath, 2010 ^73^ | | 3 | 3 | | 298.2 - 308.2 | | 101.0 - 101.0 | |
| ACETONE | | Yang et al, 2010 ^74^ | | 8 | 8 | | 278.2 - 313.2 | | 101.0 - 101.0 | |
| ACETONE | | Titanti, 1933 ^75^ | | 7 | 7 | | 392.1 - 579.6 | | 101.0 - 101.0 | |
| ACETONE | | Craven Lambert, 1951 ^76^ | | 6 | 6 | | 308.1 - 359.1 | | 42.0 - 237.0 | |
| ACETONE | | Ling Van Winkle, 1958 ^43^ | | 3 | 3 | | 383.1 - 423.1 | | 101.0 - 101.0 | |
| ACETONE | | Atoyan Mamedov, 1980 ^77^ | | 11 | 11 | | 297.1 - 455.1 | | 14566.0 - 479874.0 | |

| Material | Author | | *N*_all_ | | | *N*_used_ | | *T* / K | *p* / kPa |
| --- | --- | --- | --- | --- | --- | --- | --- | --- | --- |
| ^1^ACETYLENE | | Kiyama Makita, 1952 ^78^ | | 53 | 48 | | 293.2 - 523.2 | | 101.0 - 9512.0 |
| ACETYLENE | | Titani, 1930 ^9^ | | 7 | 7 | | 293.2 - 393.2 | | 101.0 - 4257.0 |
| ACETYLENE | | Wobser Mueller, 1941 ^79^ | | 5 | 5 | | 293.2 - 371.2 | | 101.0 - 101.0 |
| ACETYLENE | | Vogel, 1914 ^80^ | | 1 | 1 | | 273.1 - 273.1 | | 79.0 - 79.0 |
| ACETYLENE | | Bailey, 1970 ^81^ | | 20 | 20 | | 293.2 - 609.8 | | 101.0 - 101.0 |
| ACETYLENE | | Pal Barua, 1968 ^82^ | | 5 | 5 | | 303.2 - 473.1 | | 13.0 - 13.0 |
| ACETYLENE | | Senftleben Gladisch, 1949 ^3^ | | 1 | 1 | | 313.2 - 313.2 | | 101.0 - 101.0 |
| ACETYLENE | | Makita, 1955 ^83^ | | 37 | 36 | | 293.2 - 523.2 | | 101.0 - 10132.0 |
| ACETYLENE | | Liessmann, 1995 ^5^ | | 5 | 5 | | 198.2 - 258.2 | | 168.0 - 1709.0 |
| ACETYLENE | | Vargrafik, 1972 ^6^ | | 6 | 6 | | 273.2 - 423.2 | | 101.0 - 101.0 |
| ACETYLENE | | Kiyama Makita, 1952 ^78^ | | 44 | 39 | | 293.2 - 523.2 | | 101.0 - 9512.0 |
| ACETYLENE | | Pal Barua, 1968 ^82^ | | 5 | 4 | | 328.2 - 473.1 | | 101.0 - 101.0 |
| ACETYLENE | | Scholz Kley, 1981 ^7^ | | 32 | 32 | | 273.2 - 523.2 | | 101.0 - 5334.0 |
| ACETYLENE | | Senftleben Gladisch, 1949 ^3^ | | 1 | 1 | | 313.2 - 313.2 | | 101.0 - 101.0 |

| ^1^Material | Author | | *N*_all_ | | *N*_used_ | | *T* / K | *p* / kPa |
| --- | --- | --- | --- | --- | --- | --- | --- | --- |
| AMMONIA | | Makhija Stairs, 1970 ^84^ | 20 | 20 | | 208.2 - 238.2 | | 14.0 - 84.0 |
| AMMONIA | | Laesecke et al, 1999 ^85^ | 51 | 51 | | 285.1 - 335.1 | | 616.0 - 2646.0 |
| AMMONIA | | Trautz Heberling, 1931 ^86^ | 21 | 21 | | 291.3 - 573.7 | | 101.0 - 101.0 |
| AMMONIA | | Van Maass, 1935 ^87^ | 9 | 9 | | 201.9 - 297.4 | | 0.0 - 0.0 |
| AMMONIA | | Burch Raw, 1967 ^88^ | 11 | 11 | | 273.0 - 673.0 | | 101.0 - 101.0 |
| AMMONIA | | Carmichael, 1963 ^89^ | 261 | 261 | | 310.9 - 477.6 | | 101.0 - 40782.0 |
| AMMONIA | | Sun Storvick, 1979 ^90^ | 35 | 35 | | 448.1 - 598.1 | | 652.0 - 12167.0 |

| Material | Author | | *N*_all_ | | | *N*_used_ | | *T* / K | *p* / kPa |
| --- | --- | --- | --- | --- | --- | --- | --- | --- | --- |
| ARGON | | Hellemans, 1970 ^91^ | | 44 | 42 | | 104.5 - 147.1 | | 466.0 - 9808.0 |
| ARGON | | VanderGulik Trappeniers, 1986 ^92^ | | 25 | 25 | | 174.5 - 174.5 | | 16100.0 - 470700.0 |
| ARGON | | Andreev et al, 1966 ^93^ | | 40 | 0 | | 10000.0 - 0.0 | | 1000000000.0 - 0.0 |
| ARGON | | Timrot et al, 1969 ^94^ | | 7 | 7 | | 300.0 - 600.0 | | 101.0 - 101.0 |
| ARGON | | Kalelkar Kestin, 1970 ^95^ | | 9 | 2 | | 729.9 - 850.9 | | 101.0 - 101.0 |
| ARGON | | Kestin et al, 1970 ^96^ | | 8 | 8 | | 298.1 - 973.1 | | 101.0 - 101.0 |
| ARGON | | Kestin et al, 1972 ^97^ | | 7 | 7 | | 298.1 - 973.1 | | 101.0 - 101.0 |
| ARGON | | Borisov, 1973 ^98^ | | 1 | 1 | | 300.0 - 300.0 | | 101.0 - 101.0 |
| ARGON | | Kurin Golubev, 1974 ^99^ | | 104 | 104 | | 273.1 - 423.1 | | 0.0 - 379517.0 |
| ARGON | | Timrot et al, 1975 ^100^ | | 39 | 39 | | 292.4 - 574.6 | | 98.0 - 14430.0 |
| ARGON | | lyusternik lavushev, 1976 ^101^ | | 49 | 49 | | 403.0 - 1949.5 | | 20.0 - 100.0 |
| ARGON | | Kestin Wakeham, 1979 ^102^ | | 5 | 5 | | 300.6 - 473.1 | | 101.0 - 101.0 |
| ARGON | | Hobley et al, 1989 ^103^ | | 5 | 5 | | 301.0 - 521.0 | | 101.0 - 101.0 |
| ARGON | | Evers et al, 2002 ^104^ | | 81 | 81 | | 233.2 - 523.2 | | 94.0 - 28139.0 |
| ARGON | | Hurly et al, 2003 ^105^ | | 124 | 124 | | 293.2 - 373.2 | | 120.0 - 3374.0 |
| ARGON | | Wang et al, 2010 ^106^ | | 17 | 17 | | 300.1 - 300.9 | | 103.0 - 4503.0 |
| ARGON | | Wilhelm Vogel, 2000 ^107^ | | 160 | 156 | | 298.2 - 423.2 | | 0.0 - 20.0 |
| ARGON | | Vogel, 2010 ^108^ | | 49 | 49 | | 291.1 - 682.0 | | 67.0 - 156.0 |
| ARGON | | Evers, 2002 ^104^ | | 81 | 81 | | 233.2 - 523.2 | | 94.0 - 28219.0 |
| ARGON | | Carey, 1974 ^109^ | | 31 | 31 | | 294.1 - 602.3 | | 51.0 - 48646.0 |
| ARGON | | Goodwin, 2006 ^110^ | | 43 | 43 | | 323.2 - 423.1 | | 6978.0 - 68620.0 |

| Material | Author | | *N*_all_ | | | *N*_used_ | *T* / K | | *p* / kPa |
| --- | --- | --- | --- | --- | --- | --- | --- | --- | --- |
| BENZENE | | Thorpe Rodger, 1894 ^28^ | | 12 | 12 | | | 280.8 - 348.5 | 6.0 - 88.0 |
| BENZENE | | Heydweiller, 1896 ^111^ | | 17 | 17 | | | 287.9 - 458.9 | 8.0 - 1128.0 |
| BENZENE | | Batschinski, 1913 ^29^ | | 18 | 18 | | | 283.1 - 458.9 | 7.0 - 1128.0 |
| BENZENE | | Lewis, 1925 ^36^ | | 1 | 1 | | | 298.1 - 298.1 | 13.0 - 13.0 |
| BENZENE | | Khalilov, 1939 ^112^ | | 18 | 18 | | | 293.1 - 483.2 | 11.0 - 1681.0 |
| BENZENE | | Hammond Stokes, 1955 ^113^ | | 1 | 1 | | | 298.1 - 298.1 | 13.0 - 13.0 |
| BENZENE | | Kuss, 1955 ^114^ | | 7 | 7 | | | 293.1 - 343.1 | 11.0 - 74.0 |
| BENZENE | | Heiks Orban, 1956 ^115^ | | 12 | 9 | | | 363.1 - 548.2 | 136.0 - 4136.0 |
| BENZENE | | Teller Walsh, 1959 ^116^ | | 2 | 2 | | | 283.0 - 299.1 | 7.0 - 14.0 |
| BENZENE | | Bagdasaryan, 1964 ^30^ | | 9 | 9 | | | 293.0 - 453.0 | 11.0 - 1018.0 |
| BENZENE | | Bulanov, 1974 ^117^ | | 12 | 12 | | | 293.1 - 402.1 | 11.0 - 367.0 |
| BENZENE | | Gartenmeister, 1890 ^118^ | | 1 | 1 | | | 293.1 - 293.1 | 101.0 - 101.0 |
| BENZENE | | Thorpe Rodger, 1894 ^28^ | | 12 | 12 | | | 280.8 - 348.5 | 101.0 - 101.0 |
| BENZENE | | Dunstan, 1904 ^31^ | | 2 | 2 | | | 298.1 - 298.1 | 101.0 - 101.0 |
| BENZENE | | Dunstan et al, 1907 ^119^ | | 1 | 1 | | | 298.1 - 298.1 | 101.0 - 101.0 |
| BENZENE | | Dunstan Stubbs, 1908 ^120^ | | 1 | 1 | | | 298.1 - 298.1 | 101.0 - 101.0 |
| BENZENE | | Finney, 1909 ^121^ | | 1 | 1 | | | 298.1 - 298.1 | 101.0 - 101.0 |
| BENZENE | | Faust, 1912 ^33^ | | 3 | 3 | | | 273.2 - 323.1 | 101.0 - 101.0 |
| BENZENE | | Dunstan et al, 1913 ^122^ | | 1 | 1 | | | 298.1 - 298.1 | 101.0 - 101.0 |
| BENZENE | | Muchin, 1913 ^123^ | | 3 | 3 | | | 291.5 - 293.2 | 101.0 - 101.0 |
| BENZENE | | Bingham Sarver, 1920 ^124^ | | 6 | 6 | | | 278.1 - 348.1 | 101.0 - 101.0 |
| BENZENE | | Meyer Mylius, 1920 ^125^ | | 9 | 9 | | | 273.3 - 345.8 | 101.0 - 101.0 |
| BENZENE | | Schaaf, 1923 ^126^ | | 1 | 1 | | | 290.1 - 290.1 | 101.0 - 101.0 |
| BENZENE | | Miller, 1924 ^127^ | | 2 | 2 | | | 282.0 - 293.1 | 101.0 - 101.0 |
| BENZENE | | Weissenberger Piatti, 1924 ^34^ | | 1 | 1 | | | 291.1 - 291.1 | 101.0 - 101.0 |
| BENZENE | | Weissenberger Schuster, 1924 ^35^ | | 1 | 1 | | | 293.1 - 293.1 | 101.0 - 101.0 |
| BENZENE | | kolossowsky, 1925 ^128^ | | 8 | 8 | | | 285.3 - 313.2 | 101.0 - 101.0 |
| BENZENE | | Martin, 1925 ^129^ | | 7 | 7 | | | 288.1 - 303.1 | 101.0 - 101.0 |
| BENZENE | | Dessart, 1926 ^130^ | | 2 | 2 | | | 288.1 - 303.1 | 101.0 - 101.0 |
| BENZENE | | Timmermans Martin, 1926 ^131^ | | 3 | 3 | | | 288.1 - 333.1 | 101.0 - 101.0 |
| BENZENE | | Madge, 1930 ^132^ | | 8 | 8 | | | 273.2 - 343.1 | 101.0 - 101.0 |
| BENZENE | | Tausz Staab, 1930 ^133^ | | 1 | 1 | | | 293.1 - 293.1 | 101.0 - 101.0 |
| BENZENE | | Bingham Brown, 1932 ^38^ | | 3 | 3 | | | 283.1 - 303.1 | 101.0 - 101.0 |
| BENZENE | | Kireev Skvortsova, 1936 ^134^ | | 1 | 1 | | | 293.1 - 293.1 | 101.0 - 101.0 |
| BENZENE | | Shallberov Ostroumov, 1936 ^135^ | | 1 | 1 | | | 298.1 - 298.1 | 101.0 - 101.0 |
| BENZENE | | Lemonde, 1938 ^136^ | | 3 | 3 | | | 278.2 - 288.1 | 101.0 - 101.0 |
| BENZENE | | Salceanu, 1939 ^137^ | | 1 | 1 | | | 293.1 - 293.1 | 101.0 - 101.0 |
| BENZENE | | Udovenko Toropov, 1940 ^138^ | | 1 | 1 | | | 298.1 - 298.1 | 101.0 - 101.0 |
| BENZENE | | Linke, 1941 ^139^ | | 13 | 13 | | | 286.0 - 333.2 | 101.0 - 101.0 |
| BENZENE | | Teitel´baum, 1950 ^140^ | | 1 | 1 | | | 293.1 - 293.1 | 101.0 - 101.0 |
| BENZENE | | Subnis, 1952 ^141^ | | 6 | 6 | | | 293.1 - 323.1 | 101.0 - 101.0 |
| BENZENE | | Dixon Schiessler, 1954 ^142^ | | 3 | 3 | | | 293.2 - 333.2 | 101.0 - 101.0 |
| BENZENE | | Ling Van Winkle, 1958 ^43^ | | 3 | 3 | | | 303.1 - 348.1 | 101.0 - 101.0 |
| BENZENE | | Teller Walsh, 1959 ^116^ | | 3 | 3 | | | 271.5 - 299.1 | 101.0 - 101.0 |
| BENZENE | | Katti, 1966 ^143^ | | 1 | 1 | | | 298.1 - 298.1 | 101.0 - 101.0 |
| BENZENE | | Ridgway Butler, 1967 ^144^ | | 1 | 1 | | | 298.1 - 298.1 | 101.0 - 101.0 |
| BENZENE | | Blank, 1968 ^44^ | | 1 | 1 | | | 298.1 - 298.1 | 101.0 - 101.0 |
| BENZENE | | Konobeev Lyapin, 1970 ^45^ | | 5 | 5 | | | 288.2 - 333.1 | 101.0 - 101.0 |
| BENZENE | | Collings Mclaughlin, 1971 ^145^ | | 19 | 19 | | | 303.1 - 323.1 | 98.0 - 171570.0 |
| BENZENE | | Mamedov, 1971 ^146^ | | 136 | 136 | | | 293.1 - 548.1 | 100.0 - 40000.0 |
| BENZENE | | Alms, 1973 ^147^ | | 1 | 1 | | | 296.8 - 296.8 | 101.0 - 101.0 |
| BENZENE | | Parkhurst Jonas, 1975 ^148^ | | 35 | 31 | | | 303.0 - 433.0 | 100.0 - 350000.0 |
| BENZENE | | Dhillon Chugh, 1976 ^149^ | | 2 | 2 | | | 298.1 - 308.1 | 101.0 - 101.0 |
| BENZENE | | Asfour Dullien, 1981 ^150^ | | 1 | 1 | | | 298.1 - 298.1 | 101.0 - 101.0 |
| BENZENE | | Dymond, 1981 ^151^ | | 28 | 23 | | | 298.4 - 373.1 | 100.0 - 251100.0 |
| BENZENE | | Yadava Yadava, 1981 ^51^ | | 1 | 1 | | | 307.3 - 307.3 | 101.0 - 101.0 |
| BENZENE | | kashiwagi Makita, 1982^152^ | | 48 | 48 | | | 298.1 - 348.1 | 100.0 - 110000.0 |
| BENZENE | | Abdullaev Akhundov, 1983 ^153^ | | 115 | 112 | | | 423.1 - 560.1 | 582.0 - 39437.0 |
| BENZENE | | Albright, 1983 ^154^ | | 1 | 1 | | | 298.2 - 298.2 | 101.0 - 101.0 |
| BENZENE | | Oswal Rathnam, 1984 ^155^ | | 1 | 1 | | | 303.1 - 303.1 | 101.0 - 101.0 |
| BENZENE | | Awwad, 1985 ^156^ | | 1 | 1 | | | 298.1 - 298.1 | 101.0 - 101.0 |
| BENZENE | | Oswal Rao, 1985 ^157^ | | 1 | 1 | | | 303.1 - 303.1 | 101.0 - 101.0 |
| BENZENE | | Awwad, 1986 ^156^ | | 1 | 1 | | | 298.1 - 298.1 | 101.0 - 101.0 |
| BENZENE | | Fischer Weiss, 1986 ^158^ | | 7 | 7 | | | 285.0 - 345.0 | 101.0 - 101.0 |
| BENZENE | | Manjeshwar Amiabhavi, 1987 ^159^ | | 1 | 1 | | | 318.0 - 318.0 | 101.0 - 101.0 |
| BENZENE | | Ramanjaneyulu, 1987 ^160^ | | 1 | 1 | | | 303.1 - 303.1 | 101.0 - 101.0 |
| BENZENE | | Chandrasekhar, 1988 ^161^ | | 1 | 1 | | | 303.1 - 303.1 | 101.0 - 101.0 |
| BENZENE | | Rao Reddy, 1988 ^162^ | | 1 | 1 | | | 298.1 - 298.1 | 101.0 - 101.0 |
| BENZENE | | Rathnam , 1988 ^163^ | | 2 | 2 | | | 303.1 - 315.1 | 101.0 - 101.0 |
| BENZENE | | knapstad, 1989 ^164^ | | 6 | 6 | | | 288.6 - 332.9 | 101.0 - 101.0 |
| BENZENE | | Kouris Panayioton, 1989 ^165^ | | 1 | 1 | | | 298.1 - 298.1 | 101.0 - 101.0 |
| BENZENE | | Ruiz, 1989 ^166^ | | 3 | 3 | | | 303.1 - 323.1 | 101.0 - 101.0 |
| BENZENE | | Schrodt Akel, 1989 ^167^ | | 2 | 2 | | | 298.0 - 298.0 | 101.0 - 101.0 |
| BENZENE | | Joshi, 1990 ^168^ | | 4 | 4 | | | 298.2 - 313.2 | 101.0 - 101.0 |
| BENZENE | | Schumpe Luehring, 1990 ^54^ | | 1 | 1 | | | 293.2 - 293.2 | 101.0 - 101.0 |
| BENZENE | | Sekar et al, 1990 ^169^ | | 1 | 1 | | | 303.2 - 303.2 | 101.0 - 101.0 |
| BENZENE | | Sivaramprasad et al, 1990 ^170^ | | 1 | 1 | | | 298.2 - 298.2 | 101.0 - 101.0 |
| BENZENE | | Aminabhavi et al, 1991 ^171^ | | 5 | 5 | | | 298.2 - 313.2 | 101.0 - 101.0 |
| BENZENE | | Assael et al, 1991 ^172^ | | 28 | 28 | | | 303.2 - 323.2 | 100.0 - 69410.0 |
| BENZENE | | Das Jha, 1991 ^55^ | | 1 | 0 | | | 10000.0 - 0.0 | 1000000000.0 - 0.0 |
| BENZENE | | Papaioannou et al, 1991 ^173^ | | 1 | 1 | | | 298.2 - 298.2 | 101.0 - 101.0 |
| BENZENE | | Ramakanth et al, 1991 ^174^ | | 1 | 1 | | | 293.2 - 293.2 | 101.0 - 101.0 |
| BENZENE | | Zhang Liu, 1991 ^56^ | | 21 | 21 | | | 298.2 - 343.2 | 100.0 - 29500.0 |
| BENZENE | | Aralaguppi et al, 1992 ^175^ | | 3 | 3 | | | 298.2 - 308.2 | 101.0 - 101.0 |
| BENZENE | | Klofutar et al, 1992 ^176^ | | 6 | 6 | | | 293.0 - 333.0 | 101.0 - 101.0 |
| BENZENE | | Oswal Patel, 1992 ^177^ | | 2 | 2 | | | 303.2 - 303.2 | 101.0 - 101.0 |
| BENZENE | | Tasioula demetropoulos, 1992 ^178^ | | 3 | 3 | | | 298.2 - 308.2 | 101.0 - 101.0 |
| BENZENE | | Shastri et al, 1993^179^ | | 1 | 1 | | | 293.2 - 293.2 | 101.0 - 101.0 |
| BENZENE | | Lafuente et al, 1994 ^180^ | | 1 | 1 | | | 298.2 - 298.2 | 101.0 - 101.0 |
| BENZENE | | Exarchos et al, 1995 ^181^ | | 4 | 4 | | | 293.2 - 308.2 | 101.0 - 101.0 |
| BENZENE | | Krishnan et al, 1995 ^182^ | | 1 | 1 | | | 303.2 - 303.2 | 101.0 - 101.0 |
| BENZENE | | Rived et al, 1995 ^183^ | | 1 | 1 | | | 303.2 - 303.2 | 101.0 - 101.0 |
| BENZENE | | Swain et al, 1997 ^184^ | | 4 | 4 | | | 303.2 - 318.2 | 101.0 - 101.0 |
| BENZENE | | Viera Nieto, 1997 ^185^ | | 30 | 30 | | | 298.2 - 348.2 | 100.0 - 177600.0 |
| BENZENE | | Casad et al, 1998 ^186^ | | 1 | 1 | | | 298.2 - 298.2 | 101.0 - 101.0 |
| BENZENE | | Swain et al, 1998 ^187^ | | 4 | 4 | | | 303.2 - 318.2 | 101.0 - 101.0 |
| BENZENE | | Aminabhavi Banerjee, 1999 ^188^ | | 3 | 3 | | | 298.2 - 308.2 | 101.0 - 101.0 |
| BENZENE | | Aralaguppi et al, 1999 ^189^ | | 3 | 3 | | | 298.2 - 308.2 | 101.0 - 101.0 |
| BENZENE | | Guzman et al, 1999 ^190^ | | 3 | 3 | | | 283.2 - 313.2 | 101.0 - 101.0 |
| BENZENE | | Sastry et al, 1999 ^191^ | | 2 | 2 | | | 308.2 - 318.2 | 101.0 - 101.0 |
| BENZENE | | Swain et al, 1999 ^192^ | | 5 | 5 | | | 298.2 - 318.2 | 101.0 - 101.0 |
| BENZENE | | Mariano et al, 2000 ^193^ | | 1 | 1 | | | 298.2 - 298.2 | 101.0 - 101.0 |
| BENZENE | | Swain et al, 2000 ^194^ | | 13 | 13 | | | 298.2 - 318.2 | 101.0 - 101.0 |
| BENZENE | | Tsierkezos et al, 2000 ^195^ | | 1 | 1 | | | 293.2 - 293.2 | 101.0 - 101.0 |
| BENZENE | | Casad et al, 2001 ^196^ | | 1 | 1 | | | 298.2 - 298.2 | 101.0 - 101.0 |
| BENZENE | | Chowdhury et al, 2001 ^197^ | | 3 | 3 | | | 303.0 - 323.0 | 101.0 - 101.0 |
| BENZENE | | Kapadi et al, 2001 ^198^ | | 1 | 1 | | | 293.2 - 293.2 | 101.0 - 101.0 |
| BENZENE | | Pandey et al, 2001 ^199^ | | 1 | 1 | | | 298.2 - 298.2 | 101.0 - 101.0 |
| BENZENE | | Swain et al, 2001 ^200^ | | 5 | 5 | | | 298.2 - 318.2 | 101.0 - 101.0 |
| BENZENE | | Nayak et al, 2002 ^201^ | | 3 | 3 | | | 298.2 - 308.2 | 101.0 - 101.0 |
| BENZENE | | George Sastry, 2003 ^202^ | | 2 | 2 | | | 298.2 - 308.2 | 101.0 - 101.0 |
| BENZENE | | George Sastry, 2003 ^202^ | | 2 | 2 | | | 298.2 - 308.2 | 101.0 - 101.0 |
| BENZENE | | Lark et al, 2003 ^203^ | | 2 | 2 | | | 298.2 - 303.2 | 101.0 - 101.0 |
| BENZENE | | Nikam Kharat, 2003 ^204^ | | 4 | 4 | | | 298.2 - 313.2 | 101.0 - 101.0 |
| BENZENE | | Oswal et al, 2003 ^205^ | | 1 | 1 | | | 303.2 - 303.2 | 101.0 - 101.0 |
| BENZENE | | Agarwal Singh, 2004 ^63^ | | 1 | 1 | | | 298.2 - 298.2 | 101.0 - 101.0 |
| BENZENE | | Ali et al, 2004 ^206^ | | 1 | 1 | | | 303.2 - 303.2 | 101.0 - 101.0 |
| BENZENE | | Jain Singh, 2004 ^64^ | | 1 | 1 | | | 303.2 - 303.2 | 101.0 - 101.0 |
| BENZENE | | Ma et al, 2004 ^207^ | | 22 | 22 | | | 303.2 - 445.2 | 100.0 - 6100.0 |
| BENZENE | | Oswal et al, 2004 ^208^ | | 2 | 2 | | | 303.2 - 313.2 | 101.0 - 101.0 |
| BENZENE | | Yang et al, 2004 ^209^ | | 3 | 3 | | | 298.2 - 323.2 | 101.0 - 101.0 |
| BENZENE | | Oswal et al, 2005 ^210^ | | 1 | 1 | | | 303.2 - 303.2 | 101.0 - 101.0 |
| BENZENE | | Ali et al, 2006 ^211^ | | 1 | 1 | | | 308.2 - 308.2 | 101.0 - 101.0 |
| BENZENE | | Ali Tariq, 2006 ^212^ | | 1 | 1 | | | 303.2 - 303.2 | 101.0 - 101.0 |
| BENZENE | | Baragi, 2006 ^213^ | | 2 | 2 | | | 293.2 - 303.2 | 101.0 - 101.0 |
| BENZENE | | Baragi et al, 2006 ^213^ | | 4 | 4 | | | 288.2 - 303.2 | 101.0 - 101.0 |
| BENZENE | | Baragi et al, 2006 ^213^ | | 3 | 3 | | | 298.2 - 308.2 | 101.0 - 101.0 |
| BENZENE | | Han et al, 2006 ^214^ | | 1 | 1 | | | 298.2 - 298.2 | 101.0 - 101.0 |
| BENZENE | | Suresh Reddy et al, 2006 ^215^ | | 1 | 1 | | | 303.2 - 303.2 | 101.0 - 101.0 |
| BENZENE | | Varshney Singh, 2006 ^216^ | | 1 | 1 | | | 303.2 - 303.2 | 101.0 - 101.0 |
| BENZENE | | Hernandez Galvan, 2007 ^217^ | | 40 | 40 | | | 313.2 - 393.2 | 690.0 - 60000.0 |
| BENZENE | | Kharat Nikam, 2007 ^218^ | | 4 | 4 | | | 298.2 - 313.2 | 101.0 - 101.0 |
| BENZENE | | Nain, 2007 ^219^ | | 4 | 4 | | | 288.2 - 318.2 | 101.0 - 101.0 |
| BENZENE | | Nain, 2007 ^219^ | | 4 | 4 | | | 288.2 - 318.2 | 101.0 - 101.0 |
| BENZENE | | Reddy et al, 2007 ^220^ | | 1 | 1 | | | 303.2 - 303.2 | 101.0 - 101.0 |
| BENZENE | | Ren et al, 2007 ^69^ | | 1 | 1 | | | 298.2 - 298.2 | 101.0 - 101.0 |
| BENZENE | | Song et al, 2007 ^221^ | | 6 | 6 | | | 298.2 - 343.2 | 101.0 - 101.0 |
| BENZENE | | Yang et al, 2007 ^222^ | | 5 | 5 | | | 303.2 - 343.2 | 101.0 - 101.0 |
| BENZENE | | Awwad Abu-Daabes, 2008 ^223^ | | 4 | 4 | | | 298.2 - 323.2 | 101.0 - 101.0 |
| BENZENE | | Shukla, 2008 ^224^ | | 1 | 1 | | | 298.2 - 298.2 | 101.0 - 101.0 |
| BENZENE | | Song, 2008 ^225^ | | 7 | 7 | | | 303.2 - 333.2 | 101.0 - 101.0 |
| BENZENE | | Wankhede, 2008 ^226^ | | 3 | 3 | | | 288.2 - 308.2 | 101.0 - 101.0 |
| BENZENE | | Ali et al, 2009 ^227^ | | 4 | 4 | | | 298.2 - 313.2 | 101.0 - 101.0 |
| BENZENE | | Baylaucq, 2009 ^228^ | | 2 | 2 | | | 308.2 - 313.2 | 101.0 - 101.0 |
| BENZENE | | Nain et al, 2009 ^229^ | | 1 | 1 | | | 298.2 - 298.2 | 101.0 - 101.0 |
| BENZENE | | Oswal , 2009 ^230^ | | 1 | 1 | | | 303.2 - 303.2 | 101.0 - 101.0 |
| BENZENE | | Sastry, 2009 ^231^ | | 2 | 2 | | | 298.2 - 308.2 | 101.0 - 101.0 |
| BENZENE | | Yan et al, 2009 ^232^ | | 3 | 3 | | | 298.2 - 318.2 | 101.0 - 101.0 |
| BENZENE | | Ikeuchi, 2010 ^233^ | | 6 | 6 | | | 288.1 - 313.2 | 101.0 - 101.0 |
| BENZENE | | Rathnam, 2010 ^234^ | | 3 | 3 | | | 303.2 - 313.2 | 101.0 - 101.0 |
| BENZENE | | Rathnam, 2010 ^234^ | | 3 | 3 | | | 303.2 - 313.2 | 101.0 - 101.0 |
| BENZENE | | Yadava Yadava, 2010 ^51^ | | 1 | 1 | | | 308.2 - 308.2 | 101.0 - 101.0 |
| BENZENE | | Mariano, 2011 ^235^ | | 3 | 3 | | | 283.1 - 313.2 | 101.0 - 101.0 |
| BENZENE | | Pradhan Roy, 2011 ^236^ | | 1 | 1 | | | 298.2 - 298.2 | 101.0 - 101.0 |
| BENZENE | | Shukla, 2011 ^237^ | | 4 | 4 | | | 298.2 - 313.2 | 101.0 - 101.0 |
| BENZENE | | Yadava, 2011 ^238^ | | 1 | 1 | | | 308.2 - 308.2 | 101.0 - 101.0 |
| BENZENE | | Sarkar, 2012 ^239^ | | 1 | 1 | | | 298.2 - 298.2 | 101.0 - 101.0 |
| BENZENE | | Yadava, 2012 ^240^ | | 2 | 2 | | | 303.2 - 308.2 | 101.0 - 101.0 |
| BENZENE | | Bhatia, 2013 ^241^ | | 3 | 3 | | | 298.2 - 308.2 | 101.0 - 101.0 |
| BENZENE | | Bhalodia Sharma, 2013 ^242^ | | 3 | 3 | | | 303.2 - 313.2 | 101.0 - 101.0 |
| BENZENE | | Lowry Nasini, 1929 ^243^ | | 1 | 1 | | | 373.1 - 373.1 | 101.0 - 101.0 |
| BENZENE | | Titani, 1933 ^75^ | | 7 | 7 | | | 404.3 - 586.0 | 101.0 - 101.0 |
| BENZENE | | Adzumi, 1937 ^244^ | | 1 | 1 | | | 293.1 - 293.1 | 0.0 - 0.0 |
| BENZENE | | Ling Van Winkle, 1958 ^43^ | | 3 | 3 | | | 383.1 - 423.1 | 101.0 - 101.0 |
| BENZENE | | Maczek Edwards, 1979 ^245^ | | 2 | 1 | | | 373.0 - 373.0 | 101.0 - 101.0 |
| BENZENE | | Abdullaev Akhundov, 1983 ^153^ | | 51 | 43 | | | 353.1 - 560.1 | 101.0 - 4745.0 |
| BENZENE | | Vogel et al, 1986 ^246^ | | 66 | 66 | | | 305.6 - 636.9 | 12.0 - 289.0 |
| BENZENE | | Abdullaev Akhundov, 1983 ^153^ | | 252 | 251 | | | 562.6 - 673.1 | 103.0 - 39880.0 |

| Material | Author | | *N*_all_ | | | *N*_used_ | | *T* / K | *p* / kPa |
| --- | --- | --- | --- | --- | --- | --- | --- | --- | --- |
| BUTANE | | Kuenen Visser, 1913 ^247^ | | 4 | 4 | | 249.6 - 307.6 | | 39.0 - 324.0 |
| BUTANE | | Sage et al, 1939 ^248^ | | 4 | 4 | | 310.9 - 360.9 | | 356.0 - 1200.0 |
| BUTANE | | Goldhahn, 1955 ^249^ | | 8 | 8 | | 203.2 - 273.2 | | 3.0 - 104.0 |
| BUTANE | | Jullander, 1955 ^42^ | | 4 | 4 | | 183.2 - 273.2 | | 1.0 - 104.0 |
| BUTANE | | Ratkovics et al, 1974 ^250^ | | 1 | 1 | | 293.1 - 293.1 | | 208.0 - 208.0 |
| BUTANE | | Goldhahn, 1955 ^249^ | | 12 | 5 | | 273.2 - 373.1 | | 104.0 - 1536.0 |
| BUTANE | | Kuenen Visser, 1913 ^247^ | | 2 | 2 | | 289.1 - 373.1 | | 101.0 - 101.0 |
| BUTANE | | Titani, 1930 ^9^ | | 6 | 6 | | 293.1 - 393.1 | | 101.0 - 101.0 |
| BUTANE | | Wobser Muller, 1941 ^251^ | | 5 | 5 | | 293.1 - 371.1 | | 101.0 - 101.0 |
| BUTANE | | Lambert, 1955 ^12^ | | 11 | 11 | | 308.1 - 363.5 | | 101.0 - 101.0 |
| BUTANE | | Carmichael, 1963 ^89^ | | 74 | 74 | | 277.6 - 477.6 | | 113.0 - 4418.0 |
| BUTANE | | Dolan et al, 1963 ^252^ | | 9 | 9 | | 444.3 - 444.3 | | 689.0 - 6895.0 |
| BUTANE | | Golubev Agaev, 1964 ^253^ | | 36 | 36 | | 310.9 - 511.0 | | 100.0 - 3400.0 |
| BUTANE | | Kestin et al, 1971 ^254^ | | 2 | 2 | | 297.1 - 303.1 | | 101.0 - 101.0 |
| BUTANE | | Dia Pena Cheda, 1975 ^255^ | | 10 | 10 | | 313.1 - 403.1 | | 27.0 - 27.0 |
| BUTANE | | Kestin, 1977 ^256^ | | 5 | 5 | | 299.4 - 478.3 | | 101.0 - 101.0 |
| BUTANE | | Abe et al, 1978 ^257^ | | 5 | 5 | | 328.1 - 468.1 | | 101.0 - 101.0 |
| BUTANE | | Abe et al, 1979 ^258^ | | 7 | 7 | | 298.1 - 468.1 | | 101.0 - 101.0 |
| BUTANE | | Lipkin et al, 1942 ^259^ | | 11 | 7 | | 199.9 - 266.5 | | 101.0 - 101.0 |
| BUTANE | | Carmichael Sage, 1963 ^260^ | | 47 | 47 | | 277.6 - 410.9 | | 229.0 - 35365.0 |
| BUTANE | | Dolan et al, 1963 ^252^ | | 64 | 64 | | 310.9 - 410.9 | | 101.0 - 55158.0 |
| BUTANE | | Golubev Agaev, 1963 ^261^ | | 1 | 0 | | 10000.0 - 0.0 | | 1000000000.0 - 0.0 |
| BUTANE | | Diller Van Poolen, 1985 ^262^ | | 73 | 73 | | 140.0 - 300.0 | | 1460.0 - 33917.0 |
| BUTANE | | Kiran Sen, 1992 ^263^ | | 100 | 100 | | 323.0 - 443.0 | | 13310.0 - 69170.0 |
| BUTANE | | Kumagai et al, 2006 ^264^ | | 16 | 13 | | 273.2 - 333.2 | | 400.0 - 30000.0 |
| BUTANE | | Carmichael Sage, 1963 ^260^ | | 34 | 34 | | 277.6 - 433.1 | | 101.0 - 34474.0 |
| BUTANE | | Golubev Agaev, 1964 ^253^ | | 164 | 164 | | 310.9 - 511.0 | | 680.0 - 68050.0 |
| BUTANE | | Diller Van Poolen, 1985 ^262^ | | 16 | 15 | | 130.0 - 260.0 | | 101.0 - 101.0 |

| Material | | Author | *N*_all_ | | *N*_used_ | | *T* / K | *p* / kPa | |
| --- | --- | --- | --- | --- | --- | --- | --- | --- | --- |
| C11 | Golubev, 1959 ^8^ | | | 18 | 11 | 323.1 - 473.2 | | | 0.0 - 109.0 |
| C11 | Bingham Fornwalt, 1930 ^265^ | | | 8 | 8 | 273.2 - 373.1 | | | 101.0 - 101.0 |
| C11 | Shepard et al, 1931 ^266^ | | | 1 | 1 | 298.1 - 298.1 | | | 101.0 - 101.0 |
| C11 | Doolittle Peterson, 1951 ^267^ | | | 6 | 5 | 263.1 - 423.2 | | | 101.0 - 101.0 |
| C11 | Rastorguev Keramidi, 1971 ^268^ | | | 41 | 41 | 302.8 - 513.1 | | | 98.0 - 49000.0 |
| C11 | Guseinov Naziev, 1973 ^269^ | | | 87 | 87 | 292.8 - 520.1 | | | 245.0 - 49050.0 |
| C11 | Ratkovics et al, 1974 ^270^ | | | 1 | 1 | 293.1 - 293.1 | | | 101.0 - 101.0 |
| C11 | Iwahashi et al, 1990 ^271^ | | | 2 | 2 | 298.1 - 323.1 | | | 101.0 - 101.0 |
| C11 | Assael Papadaki, 1991 ^272^ | | | 30 | 30 | 303.2 - 333.2 | | | 100.0 - 62420.0 |
| C11 | Aucejo et al, 1995 ^14^ | | | 1 | 1 | 298.2 - 298.2 | | | 101.0 - 101.0 |
| C11 | Zhang et al, 2010 ^273^ | | | 4 | 4 | 293.2 - 313.2 | | | 101.0 - 101.0 |
| C11 | Chi et al, 2013 ^274^ | | | 7 | 7 | 293.2 - 323.2 | | | 100.0 - 100.0 |

| Material | | Author | *N*_all_ | | *N*_used_ | | | *T* / K | *p* / kPa | |
| --- | --- | --- | --- | --- | --- | --- | --- | --- | --- | --- |
| C12 | Dymond Young, 1980 ^275^ | | | 8 | | 3 | 353.3 - 393.2 | | | 1.0 - 5.0 |
| C12 | Bingham Fornwalt, 1930 ^265^ | | | 8 | | 8 | 273.2 - 373.1 | | | 101.0 - 101.0 |
| C12 | Shepard, 1931 ^266^ | | | 1 | | 1 | 298.1 - 298.1 | | | 101.0 - 101.0 |
| C12 | Evans, 1938 ^276^ | | | 7 | | 6 | 288.1 - 362.1 | | | 101.0 - 101.0 |
| C12 | Cutler, 1955 ^277^ | | | 1 | | 1 | 372.0 - 372.0 | | | 101.0 - 101.0 |
| C12 | Hogenboon et al, 1967 ^278^ | | | 55 | | 24 | 310.9 - 408.1 | | | 101.0 - 120000.0 |
| C12 | Anonymous, 1968 ^279^ | | | 5 | | 4 | 293.1 - 372.0 | | | 101.0 - 101.0 |
| C12 | Keramidi Rastorguev, 1970 ^280^ | | | 41 | | 41 | 303.6 - 518.1 | | | 98.0 - 49000.0 |
| C12 | Jambon Delmas, 1977 ^281^ | | | 1 | | 1 | 298.1 - 298.1 | | | 101.0 - 101.0 |
| C12 | Dymond et al, 1981 ^151^ | | | 32 | | 17 | 298.3 - 373.2 | | | 100.0 - 152500.0 |
| C12 | kashiwagi Makita, 1982 ^152^ | | | 48 | | 48 | 298.1 - 348.1 | | | 100.0 - 110000.0 |
| C12 | Awwad Allos, 1985 ^282^ | | | 1 | | 1 | 298.1 - 298.1 | | | 101.0 - 101.0 |
| C12 | Aucejo, 1986 ^283^ | | | 1 | | 1 | 298.1 - 298.1 | | | 101.0 - 101.0 |
| C12 | Awwad et al, 1986 ^156^ | | | 1 | | 1 | 298.1 - 298.1 | | | 101.0 - 101.0 |
| C12 | Awwad Salman, 1986 ^284^ | | | 1 | | 1 | 298.1 - 298.1 | | | 101.0 - 101.0 |
| C12 | Ducoulombier, 1986 ^285^ | | | 30 | | 30 | 293.1 - 373.1 | | | 100.0 - 100000.0 |
| C12 | Wakefield Marsh, 1987 ^286^ | | | 3 | | 3 | 318.2 - 338.1 | | | 101.0 - 101.0 |
| C12 | Wakefield, 1988 ^287^ | | | 2 | | 2 | 303.2 - 308.2 | | | 101.0 - 101.0 |
| C12 | Knapstad, 1989 ^164^ | | | 11 | | 11 | 293.8 - 425.4 | | | 101.0 - 101.0 |
| C12 | Iwahashi , 1990 ^271^ | | | 2 | | 2 | 298.1 - 323.1 | | | 101.0 - 101.0 |
| C12 | Knapstad, 1991 ^288^ | | | 8 | | 8 | 288.8 - 342.6 | | | 101.0 - 101.0 |
| C12 | Aminabhavi Gopalkrishna, 1994 ^289^ | | | 3 | | 3 | 298.2 - 318.2 | | | 101.0 - 101.0 |
| C12 | Aminabhavi Gopalkrishna, 1995 ^290^ | | | 4 | | 4 | 298.2 - 313.2 | | | 101.0 - 101.0 |
| C12 | Aucejo et al, 1995 ^14^ | | | 1 | | 1 | 298.2 - 298.2 | | | 101.0 - 101.0 |
| C12 | Aucejo et al, 1995 ^14^ | | | 1 | | 1 | 298.0 - 298.0 | | | 101.0 - 101.0 |
| C12 | Aminabhavi Patil, 1997 ^291^ | | | 3 | | 3 | 298.2 - 308.2 | | | 101.0 - 101.0 |
| C12 | Aralaguppi et al, 1999 ^189^ | | | 3 | | 3 | 298.2 - 308.2 | | | 101.0 - 101.0 |
| C12 | Nayak et al, 2001 ^292^ | | | 3 | | 3 | 298.2 - 308.2 | | | 101.0 - 101.0 |
| C12 | Garcia et al, 2002 ^293^ | | | 5 | | 5 | 278.2 - 318.2 | | | 101.0 - 101.0 |
| C12 | Gonzalez et al, 2003 ^294^ | | | 3 | | 3 | 293.2 - 303.2 | | | 101.0 - 101.0 |
| C12 | Moreiras et al, 2003 ^295^ | | | 1 | | 1 | 298.2 - 298.2 | | | 101.0 - 101.0 |
| C12 | Caudwell, 2004 ^296^ | | | 92 | | 72 | 298.2 - 473.2 | | | 100.0 - 161300.0 |
| C12 | Caudwell et al, 2004 ^297^ | | | 64 | | 56 | 298.2 - 473.2 | | | 100.0 - 161330.0 |
| C12 | Gonzalez et al, 2004 ^298^ | | | 3 | | 3 | 293.2 - 303.2 | | | 101.0 - 101.0 |
| C12 | Gonzalez et al, 2004 ^299^ | | | 3 | | 3 | 293.2 - 303.2 | | | 101.0 - 101.0 |
| C12 | Aljimaz et al, 2005 ^300^ | | | 3 | | 3 | 293.2 - 303.2 | | | 101.0 - 101.0 |
| C12 | Goodwin et al, 2006 ^301^ | | | 3 | | 3 | 298.2 - 308.2 | | | 101.0 - 101.0 |
| C12 | Tian Liu, 2007 ^302^ | | | 7 | | 7 | 298.2 - 328.2 | | | 101.0 - 101.0 |
| C12 | Zhang et al, 2011 ^303^ | | | 4 | | 4 | 293.2 - 313.2 | | | 101.0 - 101.0 |
| C12 | Mahajan Mirgane, 2013 ^304^ | | | 1 | | 1 | 298.2 - 298.2 | | | 101.0 - 101.0 |
| C12 | Blahusiak Schlosser, 2014 ^305^ | | | 10 | | 10 | 288.2 - 333.2 | | | 101.0 - 101.0 |
| C12 | Lago et al, 2014 ^306^ | | | 1 | | 1 | 298.2 - 298.2 | | | 101.0 - 101.0 |
| C12 | Liu Zhu, 2014 ^307^ | | | 5 | | 5 | 293.2 - 313.2 | | | 100.0 - 100.0 |
| C12 | Prak et al, 2014 ^308^ | | | 9 | | 9 | 293.2 - 373.2 | | | 101.0 - 101.0 |
| C12 | Singh et al, 2014 ^309^ | | | 1 | | 1 | 298.2 - 298.2 | | | 100.0 - 100.0 |
| C12 | Slusar et al, 1973 ^310^ | | | 8 | | 8 | 503.1 - 681.2 | | | 101.0 - 101.0 |

| Material | | Author | *N*_all_ | | *N*_used_ | | | *T* / K | *p* / kPa | |
| --- | --- | --- | --- | --- | --- | --- | --- | --- | --- | --- |
| C16 | Golubev, 1959 ^8^ | | | 16 | | 6 | 413.1 - 513.2 | | | 1.0 - 28.0 |
| C16 | Dymond Young, 1980 ^275^ | | | 10 | | 0 | 10000.0 - 0.0 | | | 1000000000.0 - 0.0 |
| C16 | Evans, 1938 ^276^ | | | 6 | | 6 | 293.1 - 363.1 | | | 101.0 - 101.0 |
| C16 | Anonymous, 1968 ^279^ | | | 4 | | 4 | 293.1 - 372.0 | | | 101.0 - 101.0 |
| C16 | Coursey Heric, 1969 ^311^ | | | 1 | | 1 | 298.1 - 298.1 | | | 101.0 - 101.0 |
| C16 | Rastorguev Keramidi, 1972 ^312^ | | | 18 | | 13 | 335.9 - 531.9 | | | 98.0 - 49000.0 |
| C16 | Ratkovics et al, 1974 ^250^ | | | 1 | | 1 | 293.1 - 293.1 | | | 101.0 - 101.0 |
| C16 | Dymond Young, 1980 ^275^ | | | 28 | | 17 | 298.1 - 373.2 | | | 100.0 - 78600.0 |
| C16 | Awwad Allos, 1985 ^282^ | | | 1 | | 1 | 298.1 - 298.1 | | | 101.0 - 101.0 |
| C16 | Awwad, 1986 ^156^ | | | 1 | | 1 | 298.1 - 298.1 | | | 101.0 - 101.0 |
| C16 | Awwad Salman, 1986 ^284^ | | | 1 | | 1 | 298.1 - 298.1 | | | 101.0 - 101.0 |
| C16 | Ducoulombier, 1986 ^285^ | | | 24 | | 19 | 313.1 - 373.1 | | | 100.0 - 80000.0 |
| C16 | Matthews, 1987 ^313^ | | | 10 | | 9 | 323.1 - 564.1 | | | 1416.0 - 3505.0 |
| C16 | Wakefield, 1988 ^287^ | | | 2 | | 2 | 303.2 - 308.1 | | | 101.0 - 101.0 |
| C16 | Aminabhavi Gopalkrishna, 1994 ^289^ | | | 3 | | 3 | 298.2 - 318.2 | | | 101.0 - 101.0 |
| C16 | Aucejo et al, 1995 ^14^ | | | 1 | | 1 | 298.2 - 298.2 | | | 101.0 - 101.0 |
| C16 | Aucejo et al, 1995 ^14^ | | | 1 | | 1 | 298.0 - 298.0 | | | 101.0 - 101.0 |
| C16 | Dandekar et al, 1998 ^314^ | | | 1 | | 1 | 293.2 - 293.2 | | | 101.0 - 101.0 |
| C16 | Fermeglia Torriano, 1999 ^315^ | | | 1 | | 1 | 298.2 - 298.2 | | | 101.0 - 101.0 |
| C16 | Queimada, 2003 ^316^ | | | 6 | | 6 | 293.2 - 343.2 | | | 101.0 - 101.0 |
| C16 | Tripathi, 2005 ^317^ | | | 1 | | 1 | 298.2 - 298.2 | | | 101.0 - 101.0 |
| C16 | Aguila-Hernandez et al, 2008 ^318^ | | | 3 | | 3 | 293.2 - 303.2 | | | 101.0 - 101.0 |
| C16 | Dubey Sharma, 2008 ^319^ | | | 3 | | 3 | 298.2 - 308.2 | | | 101.0 - 101.0 |
| C16 | Dubey Sharma, 2009 ^320^ | | | 3 | | 3 | 298.2 - 308.2 | | | 101.0 - 101.0 |
| C16 | Dubey Sharma, 2009 ^320^ | | | 3 | | 3 | 298.2 - 308.2 | | | 101.0 - 101.0 |
| C16 | Rajagopal, 2009 ^321^ | | | 54 | | 53 | 318.2 - 413.2 | | | 6895.0 - 62050.0 |
| C16 | Prak et al, 2013 ^322^ | | | 9 | | 9 | 293.2 - 373.2 | | | 101.0 - 101.0 |
| C16 | Baled et al, 2014 ^323^ | | | 42 | | 0 | 10000.0 - 0.0 | | | 1000000000.0 - 0.0 |
| C16 | Prak et al, 2014 ^308^ | | | 9 | | 9 | 293.2 - 373.2 | | | 101.0 - 101.0 |
| C16 | Slusar et al, 1973 ^310^ | | | 5 | | 4 | 584.1 - 723.1 | | | 101.0 - 101.0 |

| Material | | Author | *N*_all_ | | *N*_used_ | | | *T* / K | *p* / kPa | |
| --- | --- | --- | --- | --- | --- | --- | --- | --- | --- | --- |
| C1CC6 | Golubev, 1959 ^8^ | | | 5 | | 5 | 273.2 - 313.1 | | | 2.0 - 13.0 |
| C1CC6 | Chavanne Van, 1922 ^13^ | | | 3 | | 3 | 273.2 - 303.1 | | | 101.0 - 101.0 |
| C1CC6 | Vorlander Walter, 1925 ^324^ | | | 1 | | 1 | 293.1 - 293.1 | | | 101.0 - 101.0 |
| C1CC6 | Dessart, 1926 ^130^ | | | 2 | | 2 | 288.1 - 303.1 | | | 101.0 - 101.0 |
| C1CC6 | Timmermans Martin, 1926 ^131^ | | | 2 | | 2 | 288.1 - 303.1 | | | 101.0 - 101.0 |
| C1CC6 | Tausz Staab, 1930 ^133^ | | | 1 | | 1 | 293.1 - 293.1 | | | 101.0 - 101.0 |
| C1CC6 | Godchot Cauquil, 1931 ^325^ | | | 1 | | 1 | 290.6 - 290.6 | | | 101.0 - 101.0 |
| C1CC6 | Hugel, 1931 ^326^ | | | 1 | | 1 | 288.1 - 288.1 | | | 101.0 - 101.0 |
| C1CC6 | Evans, 1938 ^276^ | | | 7 | | 7 | 278.2 - 373.1 | | | 101.0 - 101.0 |
| C1CC6 | Friend Hargreaves, 1944 ^327^ | | | 5 | | 5 | 285.6 - 370.6 | | | 101.0 - 101.0 |
| C1CC6 | Brazier Freeman, 1969 ^11^ | | | 9 | | 5 | 303.1 - 303.1 | | | 100.0 - 200000.0 |
| C1CC6 | Jonas, 1979 ^328^ | | | 22 | | 10 | 223.0 - 298.0 | | | 100.0 - 150000.0 |
| C1CC6 | Chandrasekhar, 1988 ^161^ | | | 1 | | 1 | 303.1 - 303.1 | | | 101.0 - 101.0 |
| C1CC6 | Rammakanth et al, 1991 ^174^ | | | 1 | | 1 | 298.2 - 298.2 | | | 101.0 - 101.0 |
| C1CC6 | Et-Tahir et al, 1995 ^329^ | | | 30 | | 30 | 298.2 - 363.2 | | | 100.0 - 100000.0 |
| C1CC6 | Oswal et al, 1996 ^330^ | | | 1 | | 1 | 303.2 - 303.2 | | | 101.0 - 101.0 |
| C1CC6 | Baylaucq, 1997 ^331^ | | | 18 | | 18 | 303.2 - 343.2 | | | 100.0 - 100000.0 |
| C1CC6 | Baylaucq, 1997 ^331^ | | | 1 | | 1 | 303.2 - 303.2 | | | 101.0 - 101.0 |
| C1CC6 | Oswal et al, 2003 ^205^ | | | 1 | | 1 | 303.2 - 303.2 | | | 101.0 - 101.0 |
| C1CC6 | Zeberg- Mikkelsen et al, 2003 ^332^ | | | 42 | | 42 | 293.2 - 353.2 | | | 100.0 - 100000.0 |
| C1CC6 | Pereiro et al, 2004 ^333^ | | | 5 | | 5 | 293.2 - 313.2 | | | 101.0 - 101.0 |
| C1CC6 | Oswal et al, 2005 ^210^ | | | 1 | | 1 | 303.2 - 303.2 | | | 101.0 - 101.0 |
| C1CC6 | Oswal et al, 2005 ^334^ | | | 1 | | 1 | 303.2 - 303.2 | | | 101.0 - 101.0 |
| C1CC6 | Ye Tu, 2005 ^335^ | | | 3 | | 3 | 288.2 - 308.2 | | | 101.0 - 101.0 |
| C1CC6 | Aralaguppi Baragi, 2006 ^336^ | | | 3 | | 3 | 298.2 - 308.2 | | | 101.0 - 101.0 |
| C1CC6 | Baragi Aralaguppi, 2006 ^337^ | | | 3 | | 3 | 298.2 - 308.2 | | | 101.0 - 101.0 |
| C1CC6 | Baragi Aralaguppi, 2006 ^337^ | | | 3 | | 3 | 298.2 - 308.2 | | | 101.0 - 101.0 |
| C1CC6 | Davies et al, 2006 ^338^ | | | 7 | | 7 | 298.6 - 333.7 | | | 101.0 - 101.0 |
| C1CC6 | Iloukhani, 2006 ^339^ | | | 3 | | 3 | 293.2 - 303.2 | | | 101.0 - 101.0 |
| C1CC6 | Iloukhani, 2006 ^339^ | | | 1 | | 1 | 298.2 - 298.2 | | | 101.0 - 101.0 |
| C1CC6 | Iloukhani Samiey, 2007 ^340^ | | | 1 | | 1 | 298.2 - 298.2 | | | 101.0 - 101.0 |
| C1CC6 | Iloukhani Samiey, 2007 ^340^ | | | 1 | | 1 | 298.2 - 298.2 | | | 101.0 - 101.0 |
| C1CC6 | Yang et al, 2010 ^341^ | | | 3 | | 3 | 298.2 - 313.2 | | | 101.0 - 101.0 |
| C1CC6 | Ranjbar Momenian, 2011 ^342^ | | | 4 | | 4 | 293.2 - 308.2 | | | 101.0 - 101.0 |
| C1CC6 | Xing et al, 2011 ^343^ | | | 4 | | 4 | 298.2 - 313.2 | | | 101.0 - 101.0 |
| C1CC6 | Li et al, 2013 ^344^ | | | 6 | | 6 | 293.2 - 318.2 | | | 101.0 - 101.0 |
| C1CC6 | Jiang et al, 2014 ^345^ | | | 6 | | 6 | 293.2 - 318.2 | | | 100.0 - 100.0 |

| Material | | Author | *N*_all_ | | *N*_used_ | | | *T* / K | *p* / kPa | |
| --- | --- | --- | --- | --- | --- | --- | --- | --- | --- | --- |
| C22 | Keramidi et al, 1976 ^346^ | | | 24 | | 11 | 353.1 - 396.8 | | | 98.0 - 49000.0 |
| C22 | Queimada et al, 2003 ^316^ | | | 3 | | 0 | 10000.0 - 0.0 | | | 1000000000.0 - 0.0 |
| C22 | Queimada et al, 2005 ^347^ | | | 3 | | 0 | 10000.0 - 0.0 | | | 1000000000.0 - 0.0 |
| C22 | Queimada et al, 2003 ^316^ | | | 3 | | 1 | 343.2 - 343.2 | | | 101.0 - 101.0 |
| C22 | Queimada et al, 2005 ^347^ | | | 3 | | 1 | 343.2 - 343.2 | | | 101.0 - 101.0 |

| Material | Author | | *N*_all_ | | *N*_used_ | | | *T* / K | *p* / kPa | |
| --- | --- | --- | --- | --- | --- | --- | --- | --- | --- | --- |
| C2BUTENE | | Liessmann et al, 1995 ^5^ | | 7 | | 7 | 198.2 - 348.2 | | | 1.0 - 835.0 |
| C2BUTENE | | Vargaftik, 1972 ^6^ | | 9 | | 8 | 298.2 - 573.2 | | | 100.0 - 100.0 |
| C2BUTENE | | Scholz Kley, 1981 ^7^ | | 4 | | 4 | 323.2 - 351.2 | | | 449.0 - 894.0 |
| C2BUTENE | | Titani, 1930 ^9^ | | 6 | | 6 | 273.2 - 393.1 | | | 90.0 - 2087.0 |
| C2BUTENE | | Trautz Husseini, 1934 ^348^ | | 6 | | 6 | 291.9 - 523.2 | | | 101.0 - 101.0 |

| Material | Author | | *N*_all_ | | *N*_used_ | | | *T* / K | *p* / kPa | |
| --- | --- | --- | --- | --- | --- | --- | --- | --- | --- | --- |
| C5F12 | | Sargent Seffl, 1970 ^349^ | | 1 | | 1 | 298.2 - 298.2 | | | 87.0 - 87.0 |
| C5F12 | | Morgado, 2011 ^350^ | | 5 | | 5 | 278.1 - 297.0 | | | 38.0 - 83.0 |
| C5F12 | | Liessmann et al, 1995 ^5^ | | 10 | | 8 | 213.2 - 353.2 | | | 1.0 - 479.0 |
| C5F12 | | Cochran et al, 1974 ^351^ | | 5 | | 5 | 233.0 - 253.0 | | | 101.0 - 101.0 |

| Material | Author | | *N*_all_ | | *N*_used_ | | | *T* / K | *p* / kPa | |
| --- | --- | --- | --- | --- | --- | --- | --- | --- | --- | --- |
| C6F14 | | Freire et al, 2008 ^352^ | | 5 | | 1 | 318.2 - 318.2 | | | 64.0 - 64.0 |
| C6F14 | | Sargent Seffl, 1970 ^349^ | | 1 | | 0 | 10000.0 - 0.0 | | | 1000000000.0 - 0.0 |
| C6F14 | | Morgado, 2011 ^350^ | | 11 | | 0 | 10000.0 - 0.0 | | | 1000000000.0 - 0.0 |
| C6F14 | | Morgado et al, 2013 ^353^ | | 3 | | 0 | 10000.0 - 0.0 | | | 1000000000.0 - 0.0 |
| C6F14 | | Liessmann et al, 1995 ^5^ | | 9 | | 2 | 363.2 - 383.2 | | | 268.0 - 449.0 |

| Material | | Author | *N*_all_ | | *N*_used_ | | | *T* / K | *p* / kPa | |
| --- | --- | --- | --- | --- | --- | --- | --- | --- | --- | --- |
| CF3I | Duan et al, 1999 ^354^ | | | 18 | | 18 | 253.2 - 338.2 | | | 110.0 - 1330.0 |

| Material | | Author | *N*_all_ | | *N*_used_ | | | *T* / K | *p* / kPa | |
| --- | --- | --- | --- | --- | --- | --- | --- | --- | --- | --- |
| CHLORINE | Wobser Mueller, 1941 ^79^ | | | 5 | | 5 | 293.2 - 371.2 | | | 101.0 - 101.0 |
| CHLORINE | Trautz Winterkorn, 1931 ^355^ | | | 6 | | 6 | 293.1 - 523.0 | | | 101.0 - 3739.0 |
| CHLORINE | Vogel, 1914 ^80^ | | | 3 | | 1 | 273.1 - 273.1 | | | 83.0 - 83.0 |
| CHLORINE | Braune Linke, 1930 ^356^ | | | 9 | | 9 | 288.8 - 772.0 | | | 12.0 - 12.0 |
| CHLORINE | Steacie Johnson, 1925 ^357^ | | | 13 | | 13 | 196.7 - 239.4 | | | 9.0 - 96.0 |
| CHLORINE | Van Aubel, 1926 ^358^ | | | 7 | | 7 | 196.7 - 239.4 | | | 9.0 - 96.0 |
| CHLORINE | Liessmann et al, 1995 ^5^ | | | 8 | | 5 | 203.2 - 283.2 | | | 14.0 - 474.0 |
| CHLORINE | Trautz Ruf, 1934 ^359^ | | | 6 | | 6 | 293.2 - 523.2 | | | 101.0 - 3782.0 |
| CHLORINE | Scholz Kley, 1981 ^7^ | | | 31 | | 31 | 196.7 - 772.0 | | | 9.0 - 3782.0 |
| CHLORINE | Steacie Johnson, 1925 ^357^ | | | 13 | | 13 | 196.7 - 239.4 | | | 9.0 - 96.0 |

| Material | Author | | *N*_all_ | | *N*_used_ | | | *T* / K | *p* / kPa | |
| --- | --- | --- | --- | --- | --- | --- | --- | --- | --- | --- |
| CHLOROBENZENE | | Timmermans Martin, 1926^131^ | | 2 | | 2 | 288.1 - 303.1 | | | 101.0 - 101.0 |
| CHLOROBENZENE | | Heston et al, 1950 ^360^ | | 4 | | 4 | 274.2 - 328.1 | | | 101.0 - 101.0 |
| CHLOROBENZENE | | Mumford Phillips, 1950 ^361^ | | 2 | | 2 | 293.1 - 298.1 | | | 101.0 - 101.0 |
| CHLOROBENZENE | | Fort Moore, 1966 ^362^ | | 1 | | 1 | 298.1 - 298.1 | | | 101.0 - 101.0 |
| CHLOROBENZENE | | Nigam Singh, 1971 ^363^ | | 3 | | 3 | 298.1 - 308.1 | | | 101.0 - 101.0 |
| CHLOROBENZENE | | Abdullaev Djafarova, 1980 ^364^ | | 89 | | 89 | 296.1 - 548.1 | | | 100.0 - 40000.0 |
| CHLOROBENZENE | | Singh Sinha, 1982 ^365^ | | 1 | | 1 | 298.1 - 298.1 | | | 101.0 - 101.0 |
| CHLOROBENZENE | | Koshechko krylov, 1984 ^366^ | | 6 | | 6 | 258.0 - 343.0 | | | 101.0 - 101.0 |
| CHLOROBENZENE | | Singh Sinha, 1984 ^367^ | | 4 | | 4 | 303.1 - 333.1 | | | 101.0 - 101.0 |
| CHLOROBENZENE | | Singh Sinha, 1985 ^368^ | | 5 | | 5 | 298.1 - 333.1 | | | 101.0 - 101.0 |
| CHLOROBENZENE | | Gokavi et al, 1986 ^369^ | | 4 | | 4 | 298.1 - 328.1 | | | 101.0 - 101.0 |
| CHLOROBENZENE | | Singh et al, 1986 ^370^ | | 1 | | 1 | 298.1 - 298.1 | | | 101.0 - 101.0 |
| CHLOROBENZENE | | Sivaramprasad et al, 1990 ^170^ | | 1 | | 1 | 298.2 - 298.2 | | | 101.0 - 101.0 |
| CHLOROBENZENE | | Aminabhavi, 1991 ^171^ | | 4 | | 4 | 298.2 - 313.2 | | | 101.0 - 101.0 |
| CHLOROBENZENE | | Das Jha, 1991 ^55^ | | 1 | | 0 | 10000.0 - 0.0 | | | 1000000000.0 - 0.0 |
| CHLOROBENZENE | | Liu Luo, 1992 ^57^ | | 4 | | 4 | 293.2 - 313.2 | | | 101.0 - 101.0 |
| CHLOROBENZENE | | Krishnan, 1995 ^182^ | | 1 | | 1 | 303.2 - 303.2 | | | 101.0 - 101.0 |
| CHLOROBENZENE | | Ramadevi et al, 1996 ^371^ | | 2 | | 2 | 303.2 - 313.2 | | | 101.0 - 101.0 |
| CHLOROBENZENE | | Artigas et al, 1997 ^372^ | | 1 | | 1 | 298.2 - 298.2 | | | 101.0 - 101.0 |
| CHLOROBENZENE | | Artigas et al, 1998 ^373^ | | 1 | | 1 | 298.2 - 298.2 | | | 101.0 - 101.0 |
| CHLOROBENZENE | | Sastry et al, 1999 ^191^ | | 3 | | 3 | 298.2 - 318.2 | | | 101.0 - 101.0 |
| CHLOROBENZENE | | Tsierkezos et al, 2000 ^195^ | | 1 | | 1 | 293.2 - 293.2 | | | 101.0 - 101.0 |
| CHLOROBENZENE | | Nayak et al, 2003 ^374^ | | 3 | | 3 | 298.2 - 308.2 | | | 101.0 - 101.0 |
| CHLOROBENZENE | | Nikam kharat, 2003 ^204^ | | 4 | | 4 | 298.2 - 313.2 | | | 101.0 - 101.0 |
| CHLOROBENZENE | | Li et al,2004 ^375^ | | 1 | | 1 | 298.2 - 298.2 | | | 101.0 - 101.0 |
| CHLOROBENZENE | | Yang et al, 2004 ^209^ | | 7 | | 7 | 293.2 - 343.2 | | | 101.0 - 101.0 |
| CHLOROBENZENE | | Oswal et al, 2005 ^376^ | | 1 | | 1 | 303.2 - 303.2 | | | 101.0 - 101.0 |
| CHLOROBENZENE | | Ali Tariq, 2006 ^212^ | | 1 | | 1 | 303.2 - 303.2 | | | 101.0 - 101.0 |
| CHLOROBENZENE | | Bhuiyan et al, 2007 ^377^ | | 4 | | 4 | 298.2 - 313.2 | | | 101.0 - 101.0 |
| CHLOROBENZENE | | Ali et al, 2008 ^378^ | | 2 | | 2 | 298.2 - 303.2 | | | 101.0 - 101.0 |
| CHLOROBENZENE | | Baskaran Kubendran, 2008 ^379^ | | 3 | | 3 | 303.2 - 323.2 | | | 100.0 - 100.0 |
| CHLOROBENZENE | | Sastry et al, 2008 ^380^ | | 2 | | 2 | 298.2 - 308.2 | | | 101.0 - 101.0 |
| CHLOROBENZENE | | Shukla et al, 2008 ^381^ | | 3 | | 3 | 293.0 - 313.0 | | | 101.0 - 101.0 |
| CHLOROBENZENE | | Sastry et al, 2009 ^231^ | | 2 | | 2 | 298.2 - 308.2 | | | 101.0 - 101.0 |
| CHLOROBENZENE | | Ni et al, 2010 ^382^ | | 4 | | 4 | 298.2 - 313.2 | | | 101.0 - 101.0 |
| CHLOROBENZENE | | Pradhan Roy, 2011 ^236^ | | 1 | | 1 | 298.2 - 298.2 | | | 101.0 - 101.0 |
| CHLOROBENZENE | | Shukla et al, 2011 ^237^ | | 4 | | 4 | 298.2 - 313.2 | | | 101.0 - 101.0 |
| CHLOROBENZENE | | Yadava et al, 2011 ^238^ | | 1 | | 1 | 308.2 - 308.2 | | | 101.0 - 101.0 |
| CHLOROBENZENE | | Hamzehlouia Asfour, 2012 ^383^ | | 2 | | 2 | 308.2 - 313.2 | | | 101.0 - 101.0 |
| CHLOROBENZENE | | Kumar et al, 2012 ^384^ | | 4 | | 4 | 303.2 - 318.2 | | | 101.0 - 101.0 |
| CHLOROBENZENE | | Rathnam et al, 2012 ^385^ | | 2 | | 2 | 303.2 - 313.2 | | | 101.0 - 101.0 |
| CHLOROBENZENE | | Sarkar et al, 2012 ^239^ | | 1 | | 1 | 298.2 - 298.2 | | | 101.0 - 101.0 |
| CHLOROBENZENE | | Yadava et al, 2012 ^240^ | | 2 | | 2 | 303.2 - 308.2 | | | 101.0 - 101.0 |
| CHLOROBENZENE | | Bhatia et al, 2013 ^241^ | | 3 | | 3 | 298.2 - 308.2 | | | 101.0 - 101.0 |
| CHLOROBENZENE | | Lavanya et al, 2013 ^386^ | | 3 | | 3 | 303.2 - 313.2 | | | 101.0 - 101.0 |
| CHLOROBENZENE | | Panchenkov et al, 1958 ^387^ | | 7 | | 7 | 293.1 - 353.1 | | | 1.0 - 20.0 |
| CHLOROBENZENE | | Abdullaev Akhundov, 1983 ^153^ | | 67 | | 61 | 406.1 - 648.1 | | | 103.0 - 2974.0 |
| CHLOROBENZENE | | Akhundov et al, 1984 ^388^ | | 134 | | 0 | 10000.0 - 0.0 | | | 1000000000.0 - 0.0 |

| Material | Author | | *N*_all_ | | *N*_used_ | | | *T* / K | *p* / kPa | |
| --- | --- | --- | --- | --- | --- | --- | --- | --- | --- | --- |
| CO | | Vogel, 2012 ^389^ | | 78 | | 78 | 288.7 - 681.1 | | | 0.0 - 158.0 |

| Material | | Author | *N*_all_ | | *N*_used_ | | | *T* / K | *p* / kPa | |
| --- | --- | --- | --- | --- | --- | --- | --- | --- | --- | --- |
| CO2 | Rabe, 1981 ^390^ | | | 5 | | 5 | 220.0 - 300.0 | | | 606.0 - 6715.0 |
| CO2 | Diller Ball, 1985 ^391^ | | | 14 | | 14 | 220.0 - 285.0 | | | 606.0 - 4725.0 |
| CO2 | Gururaja et al, 1967 ^392^ | | | 3 | | 3 | 300.7 - 300.7 | | | 98.0 - 98.0 |
| CO2 | Borisov et al, 1973 ^98^ | | | 1 | | 1 | 300.0 - 300.0 | | | 101.0 - 101.0 |
| CO2 | Kurin Golubev, 1974 ^99^ | | | 79 | | 79 | 293.1 - 423.1 | | | 0.0 - 355981.0 |
| CO2 | Timrot et al, 1975 ^100^ | | | 26 | | 26 | 294.6 - 447.2 | | | 100.0 - 7820.0 |
| CO2 | Kestin et al, 1976 ^393^ | | | 3 | | 3 | 296.1 - 477.1 | | | 101.0 - 101.0 |
| CO2 | Kestin et al, 1977 ^256^ | | | 6 | | 6 | 298.1 - 773.1 | | | 101.0 - 101.0 |
| CO2 | Harris et al, 1979 ^394^ | | | 10 | | 10 | 203.1 - 310.0 | | | 101.0 - 101.0 |
| CO2 | Lusternik Kuznetsov, 1986 ^395^ | | | 62 | | 62 | 508.2 - 1871.5 | | | 101.0 - 101.0 |
| CO2 | Hobley et al, 1989 ^103^ | | | 5 | | 5 | 301.0 - 521.0 | | | 101.0 - 101.0 |
| CO2 | Hunter et al, 1993 ^396^ | | | 21 | | 21 | 213.2 - 353.2 | | | 19.0 - 41.0 |
| CO2 | Timrot et al, 1975 ^100^ | | | 26 | | 26 | 294.6 - 447.2 | | | 100.0 - 7820.0 |
| CO2 | Ulybin Makarushkin, 1976 ^397^ | | | 57 | | 57 | 223.2 - 293.1 | | | 6180.0 - 54680.0 |
| CO2 | Padua et al, 1994 ^398^ | | | 64 | | 64 | 260.0 - 300.0 | | | 5950.0 - 100000.0 |
| CO2 | Diller Ball, 1985 ^391^ | | | 82 | | 80 | 220.0 - 320.0 | | | 720.0 - 31051.0 |
| CO2 | Maltsev et al, 2004 ^399^ | | | 3 | | 3 | 500.0 - 1100.0 | | | 300.0 - 300.0 |

| Material | | Author | *N*_all_ | | *N*_used_ | | | *T* / K | *p* / kPa | |
| --- | --- | --- | --- | --- | --- | --- | --- | --- | --- | --- |
| CYCLOHEX | Kuss, 1955 ^114^ | | | 8 | | 8 | 293.1 - 353.1 | | | 11.0 - 102.0 |
| CYCLOHEX | Golubev, 1959 ^8^ | | | 7 | | 7 | 293.1 - 353.1 | | | 11.0 - 102.0 |
| CYCLOHEX | Isdale et al, 1979 ^400^ | | | 3 | | 3 | 298.1 - 348.1 | | | 14.0 - 88.0 |
| CYCLOHEX | Lavrent Yakovlev, 1985 ^401^ | | | 20 | | 20 | 292.9 - 547.2 | | | 11.0 - 3769.0 |
| CYCLOHEX | Drapier, 1911 ^402^ | | | 5 | | 5 | 290.1 - 308.1 | | | 101.0 - 101.0 |
| CYCLOHEX | Chavanne Van Risseghem, 1922 ^13^ | | | 3 | | 3 | 288.1 - 303.1 | | | 101.0 - 101.0 |
| CYCLOHEX | Vorlander Walter, 1925 ^324^ | | | 1 | | 1 | 293.1 - 293.1 | | | 101.0 - 101.0 |
| CYCLOHEX | Dessart, 1926 ^130^ | | | 2 | | 2 | 288.1 - 303.1 | | | 101.0 - 101.0 |
| CYCLOHEX | Timmermans Martin, 1926 ^37^ | | | 3 | | 3 | 288.1 - 333.1 | | | 101.0 - 101.0 |
| CYCLOHEX | Tausz Staab, 1930 ^133^ | | | 1 | | 1 | 293.1 - 293.1 | | | 101.0 - 101.0 |
| CYCLOHEX | Godchot Cauquil, 1931 ^325^ | | | 1 | | 1 | 286.6 - 286.6 | | | 101.0 - 101.0 |
| CYCLOHEX | Hugel, 1931 ^326^ | | | 2 | | 2 | 288.1 - 303.1 | | | 101.0 - 101.0 |
| CYCLOHEX | Vorlander Fischer, 1932 ^403^ | | | 1 | | 1 | 290.1 - 290.1 | | | 101.0 - 101.0 |
| CYCLOHEX | Evans, 1938 ^276^ | | | 6 | | 6 | 281.1 - 348.1 | | | 101.0 - 101.0 |
| CYCLOHEX | Linke, 1941 ^139^ | | | 4 | | 4 | 290.6 - 312.7 | | | 101.0 - 101.0 |
| CYCLOHEX | Friend Hargreavers, 1944 ^327^ | | | 5 | | 5 | 284.3 - 348.8 | | | 101.0 - 101.0 |
| CYCLOHEX | Hammond Stokes, 1955 ^113^ | | | 1 | | 1 | 298.1 - 298.1 | | | 101.0 - 101.0 |
| CYCLOHEX | Katti et al, 1966 ^143^ | | | 1 | | 1 | 298.1 - 298.1 | | | 101.0 - 101.0 |
| CYCLOHEX | Ridgway Butler, 1967 ^144^ | | | 1 | | 1 | 298.1 - 298.1 | | | 101.0 - 101.0 |
| CYCLOHEX | Konobeev Lyapin, 1970 ^45^ | | | 3 | | 3 | 293.1 - 333.1 | | | 101.0 - 101.0 |
| CYCLOHEX | Collings Mclaughlin, 1971 ^145^ | | | 17 | | 17 | 303.1 - 323.1 | | | 98.0 - 78432.0 |
| CYCLOHEX | Guseinov Naziev, 1973 ^269^ | | | 88 | | 87 | 290.7 - 523.1 | | | 101.0 - 49150.0 |
| CYCLOHEX | Pownall Smith, 1973 ^404^ | | | 1 | | 1 | 296.1 - 296.1 | | | 101.0 - 101.0 |
| CYCLOHEX | Dhillon Chugh, 1976 ^149^ | | | 2 | | 2 | 298.1 - 308.1 | | | 101.0 - 101.0 |
| CYCLOHEX | Isdale et al, 1979 ^400^ | | | 12 | | 11 | 298.1 - 373.1 | | | 100.0 - 200000.0 |
| CYCLOHEX | Jonas et al, 1980 ^405^ | | | 25 | | 23 | 313.0 - 383.0 | | | 100.0 - 160000.0 |
| CYCLOHEX | Mishra, 1981 ^406^ | | | 1 | | 1 | 303.1 - 303.1 | | | 101.0 - 101.0 |
| CYCLOHEX | Yadava Yadava, 1981 ^51^ | | | 1 | | 1 | 307.3 - 307.3 | | | 101.0 - 101.0 |
| CYCLOHEX | Kashiwagi Makita, 1982 ^152^ | | | 48 | | 48 | 298.1 - 348.1 | | | 100.0 - 110000.0 |
| CYCLOHEX | Albright et al, 1983 ^154^ | | | 1 | | 1 | 298.2 - 298.2 | | | 101.0 - 101.0 |
| CYCLOHEX | Oswal Rathnam, 1984 ^155^ | | | 1 | | 1 | 303.1 - 303.1 | | | 101.0 - 101.0 |
| CYCLOHEX | Durov Artykov, 1985 ^407^ | | | 4 | | 4 | 283.0 - 313.0 | | | 101.0 - 101.0 |
| CYCLOHEX | Oswal Rao, 1985 ^157^ | | | 1 | | 1 | 303.1 - 303.1 | | | 101.0 - 101.0 |
| CYCLOHEX | Awwad et al, 1986 ^156^ | | | 1 | | 1 | 298.1 - 298.1 | | | 101.0 - 101.0 |
| CYCLOHEX | Fischer Weiss, 1986 ^158^ | | | 7 | | 7 | 285.0 - 345.0 | | | 101.0 - 101.0 |
| CYCLOHEX | Manjeshwar Aminabhavi, 1987 ^159^ | | | 2 | | 2 | 318.0 - 318.0 | | | 101.0 - 101.0 |
| CYCLOHEX | Chandrasekhar, 1988 ^161^ | | | 1 | | 1 | 303.1 - 303.1 | | | 101.0 - 101.0 |
| CYCLOHEX | Manjeshwar Aminabhavi, 1988 ^408^ | | | 1 | | 1 | 298.1 - 298.1 | | | 101.0 - 101.0 |
| CYCLOHEX | Knapstad et al, 1989 ^164^ | | | 13 | | 13 | 288.7 - 333.8 | | | 101.0 - 101.0 |
| CYCLOHEX | Ruiz et al, 1989 ^166^ | | | 3 | | 3 | 303.1 - 323.1 | | | 101.0 - 101.0 |
| CYCLOHEX | Joshi et al, 1990 ^168^ | | | 4 | | 4 | 298.2 - 313.2 | | | 101.0 - 101.0 |
| CYCLOHEX | Schumpe Luehring, 1990 ^54^ | | | 1 | | 1 | 293.2 - 293.2 | | | 101.0 - 101.0 |
| CYCLOHEX | Sekar et al, 1990 ^169^ | | | 1 | | 1 | 303.2 - 303.2 | | | 101.0 - 101.0 |
| CYCLOHEX | Aminabhavi et al, 1991 ^171^ | | | 4 | | 4 | 298.2 - 313.2 | | | 101.0 - 101.0 |
| CYCLOHEX | Cueto et al, 1991 ^409^ | | | 7 | | 7 | 283.0 - 313.0 | | | 101.0 - 101.0 |
| CYCLOHEX | Papaioannou et al, 1991 ^173^ | | | 1 | | 1 | 298.2 - 298.2 | | | 101.0 - 101.0 |
| CYCLOHEX | Papaioannou Ziogas, 1991 ^410^ | | | 5 | | 5 | 288.2 - 308.2 | | | 101.0 - 101.0 |
| CYCLOHEX | Ramakanth et al, 1991 ^174^ | | | 1 | | 1 | 298.2 - 298.2 | | | 101.0 - 101.0 |
| CYCLOHEX | Zhang Liu, 1991 ^56^ | | | 21 | | 21 | 298.2 - 343.2 | | | 100.0 - 29500.0 |
| CYCLOHEX | Nath chaudhari, 1992 ^411^ | | | 1 | | 1 | 303.2 - 303.2 | | | 101.0 - 101.0 |
| CYCLOHEX | Oswal Patel, 1992 ^177^ | | | 2 | | 2 | 303.2 - 303.2 | | | 101.0 - 101.0 |
| CYCLOHEX | Balsco et al, 1993 ^412^ | | | 1 | | 1 | 298.2 - 298.2 | | | 101.0 - 101.0 |
| CYCLOHEX | Munoz et al, 1994 ^413^ | | | 5 | | 5 | 293.0 - 313.0 | | | 101.0 - 101.0 |
| CYCLOHEX | Pandey et al, 1994 ^414^ | | | 1 | | 1 | 298.2 - 298.2 | | | 101.0 - 101.0 |
| CYCLOHEX | Aminabhavi Gopalakrishna, 1995 ^290^ | | | 3 | | 3 | 298.2 - 308.2 | | | 101.0 - 101.0 |
| CYCLOHEX | Aminabhavi Gopalakrishna, 1995 ^415^ | | | 4 | | 4 | 298.2 - 313.2 | | | 101.0 - 101.0 |
| CYCLOHEX | Artigas et al, 1995 ^416^ | | | 1 | | 1 | 303.2 - 303.2 | | | 101.0 - 101.0 |
| CYCLOHEX | Exarchos et al, 1995 ^181^ | | | 3 | | 3 | 298.2 - 308.2 | | | 101.0 - 101.0 |
| CYCLOHEX | Aminabhavi et al, 1996 ^417^ | | | 3 | | 3 | 298.2 - 308.2 | | | 101.0 - 101.0 |
| CYCLOHEX | Padua et al, 1996 ^418^ | | | 25 | | 25 | 298.2 - 348.1 | | | 100.0 - 38000.0 |
| CYCLOHEX | Aminabhavi Banerjee, 1998 ^419^ | | | 3 | | 3 | 298.2 - 308.2 | | | 101.0 - 101.0 |
| CYCLOHEX | Gascon et al, 1999 ^420^ | | | 2 | | 2 | 298.2 - 313.2 | | | 101.0 - 101.0 |
| CYCLOHEX | Guzman et al, 1999 ^190^ | | | 3 | | 3 | 283.2 - 313.2 | | | 101.0 - 101.0 |
| CYCLOHEX | Gascon et al, 2000 ^421^ | | | 2 | | 2 | 298.2 - 313.2 | | | 101.0 - 101.0 |
| CYCLOHEX | Gascon et al, 2000 ^422^ | | | 2 | | 2 | 298.2 - 313.2 | | | 101.0 - 101.0 |
| CYCLOHEX | Chowdhury et al, 2001 ^197^ | | | 3 | | 3 | 303.0 - 323.0 | | | 101.0 - 101.0 |
| CYCLOHEX | Gomez-Diaz et al, 2001 ^423^ | | | 6 | | 6 | 298.2 - 323.2 | | | 101.0 - 101.0 |
| CYCLOHEX | Gomez-Diaz et al, 2002 ^424^ | | | 1 | | 1 | 298.2 - 298.2 | | | 101.0 - 101.0 |
| CYCLOHEX | George Sastry, 2003 ^202^ | | | 2 | | 2 | 298.2 - 308.2 | | | 101.0 - 101.0 |
| CYCLOHEX | Oswal et al, 2003 ^205^ | | | 2 | | 2 | 303.2 - 303.2 | | | 101.0 - 101.0 |
| CYCLOHEX | Ali et al, 2004 ^425^ | | | 1 | | 1 | 308.0 - 308.0 | | | 101.0 - 101.0 |
| CYCLOHEX | Haro et al, 2004 ^426^ | | | 2 | | 2 | 298.2 - 313.2 | | | 101.0 - 101.0 |
| CYCLOHEX | Ma et al, 2004 ^207^ | | | 16 | | 13 | 323.2 - 413.2 | | | 100.0 - 6100.0 |
| CYCLOHEX | Oswal et al, 2004 ^208^ | | | 2 | | 2 | 303.2 - 313.2 | | | 101.0 - 101.0 |
| CYCLOHEX | Oswal et al, 2004 ^427^ | | | 1 | | 1 | 303.2 - 303.2 | | | 101.0 - 101.0 |
| CYCLOHEX | Yang et al, 2004 ^209^ | | | 5 | | 5 | 298.2 - 333.2 | | | 101.0 - 101.0 |
| CYCLOHEX | gonzalez, 2005 ^66^ | | | 3 | | 3 | 293.2 - 303.2 | | | 101.0 - 101.0 |
| CYCLOHEX | Iloukhani et al, 2005 ^428^ | | | 1 | | 1 | 298.2 - 298.2 | | | 101.0 - 101.0 |
| CYCLOHEX | Oswal et al, 2005 ^210^ | | | 1 | | 1 | 303.2 - 303.2 | | | 101.0 - 101.0 |
| CYCLOHEX | Oswal et al, 2005 ^334^ | | | 1 | | 1 | 303.2 - 303.2 | | | 101.0 - 101.0 |
| CYCLOHEX | Singh et al, 2005 ^429^ | | | 3 | | 3 | 298.2 - 308.2 | | | 101.0 - 101.0 |
| CYCLOHEX | Tripathi, 2005 ^317^ | | | 1 | | 1 | 298.2 - 298.2 | | | 101.0 - 101.0 |
| CYCLOHEX | Gonzalez et al, 2006 ^68^ | | | 3 | | 3 | 293.2 - 303.2 | | | 101.0 - 101.0 |
| CYCLOHEX | Suresh Reddy et al, 2006 ^215^ | | | 1 | | 1 | 303.2 - 303.2 | | | 101.0 - 101.0 |
| CYCLOHEX | Gonzalez et al, 2007 ^430^ | | | 3 | | 3 | 293.2 - 303.2 | | | 101.0 - 101.0 |
| CYCLOHEX | Reddy et al, 2007 ^220^ | | | 1 | | 1 | 303.2 - 303.2 | | | 101.0 - 101.0 |
| CYCLOHEX | Yang et al, 2007 ^222^ | | | 4 | | 4 | 303.2 - 333.2 | | | 101.0 - 101.0 |
| CYCLOHEX | Awwad Abu-Daabes, 2008 ^223^ | | | 4 | | 4 | 298.2 - 323.2 | | | 101.0 - 101.0 |
| CYCLOHEX | Fan et al, 2008 ^431^ | | | 5 | | 5 | 288.2 - 308.2 | | | 101.0 - 101.0 |
| CYCLOHEX | Fan et al, 2008 ^431^ | | | 3 | | 3 | 293.2 - 303.2 | | | 101.0 - 101.0 |
| CYCLOHEX | Gardas Oswal, 2008 ^432^ | | | 1 | | 1 | 303.2 - 303.2 | | | 101.0 - 101.0 |
| CYCLOHEX | Shukla et al, 2008 ^224^ | | | 1 | | 1 | 298.2 - 298.2 | | | 101.0 - 101.0 |
| CYCLOHEX | Ghael et al, 2009 ^433^ | | | 1 | | 1 | 303.2 - 303.2 | | | 101.0 - 101.0 |
| CYCLOHEX | Hernandez Galvan, 2009 ^434^ | | | 40 | | 40 | 313.2 - 393.2 | | | 690.0 - 60000.0 |
| CYCLOHEX | Oswal et al, 2009 ^230^ | | | 1 | | 1 | 303.2 - 303.2 | | | 101.0 - 101.0 |
| CYCLOHEX | Rajagopal, 2009 ^321^ | | | 82 | | 82 | 298.2 - 413.2 | | | 100.0 - 62050.0 |
| CYCLOHEX | Roy et al, 2009 ^435^ | | | 1 | | 1 | 298.2 - 298.2 | | | 101.0 - 101.0 |
| CYCLOHEX | Silva et al, 2009 ^436^ | | | 5 | | 5 | 283.2 - 323.2 | | | 101.0 - 101.0 |
| CYCLOHEX | Rathnam et al, 2010 ^437^ | | | 3 | | 3 | 303.2 - 313.2 | | | 101.0 - 101.0 |
| CYCLOHEX | Yadava Yadava, 2010 ^438^ | | | 1 | | 1 | 308.2 - 308.2 | | | 101.0 - 101.0 |
| CYCLOHEX | Pradhan Roy, 2011 ^236^ | | | 1 | | 1 | 298.2 - 298.2 | | | 101.0 - 101.0 |
| CYCLOHEX | Hamzehlouia Asfour, 2012 ^383^ | | | 2 | | 2 | 308.2 - 313.2 | | | 101.0 - 101.0 |
| CYCLOHEX | Rathnam et al, 2012 ^439^ | | | 3 | | 3 | 303.2 - 313.2 | | | 101.0 - 101.0 |
| CYCLOHEX | Li et al, 2013 ^344^ | | | 6 | | 6 | 293.2 - 318.2 | | | 101.0 - 101.0 |
| CYCLOHEX | Sastry et al, 2013 ^440^ | | | 2 | | 2 | 298.2 - 308.2 | | | 101.0 - 101.0 |
| CYCLOHEX | Hammani et al, 2014 ^441^ | | | 3 | | 3 | 313.2 - 323.2 | | | 101.0 - 101.0 |
| CYCLOHEX | Torin-Ollarves et al, 2014 ^442^ | | | 5 | | 5 | 293.2 - 333.2 | | | 101.0 - 101.0 |
| CYCLOHEX | Titani, 1933 ^75^ | | | 7 | | 7 | 394.8 - 579.6 | | | 101.0 - 101.0 |
| CYCLOHEX | Guseinov Naziev, 1973 ^269^ | | | 22 | | 20 | 398.1 - 523.1 | | | 101.0 - 2000.0 |

| Material | | Author | *N*_all_ | | *N*_used_ | | | *T* / K | *p* / kPa | |
| --- | --- | --- | --- | --- | --- | --- | --- | --- | --- | --- |
| CYCLOPEN | Golubev, 1959 ^8^ | | | 5 | | 5 | 273.2 - 313.1 | | | 15.0 - 75.0 |
| CYCLOPEN | Ma et al, 2003 ^443^ | | | 21 | | 21 | 253.2 - 353.2 | | | 5.0 - 254.0 |
| CYCLOPEN | Timmermans Hennaut, 1937 ^444^ | | | 1 | | 1 | 288.1 - 288.1 | | | 101.0 - 101.0 |
| CYCLOPEN | Evans, 1938 ^276^ | | | 4 | | 4 | 278.2 - 318.1 | | | 101.0 - 101.0 |
| CYCLOPEN | Brazier Freeman, 1969 ^11^ | | | 9 | | 5 | 303.1 - 303.1 | | | 100.0 - 200000.0 |
| CYCLOPEN | Fischer Weiss, 1986 ^158^ | | | 4 | | 4 | 285.0 - 315.0 | | | 101.0 - 101.0 |
| CYCLOPEN | Guzman et al, 1999 ^190^ | | | 3 | | 3 | 283.2 - 313.2 | | | 101.0 - 101.0 |
| CYCLOPEN | Assael et al, 2000 ^445^ | | | 78 | | 78 | 218.7 - 308.7 | | | 101.0 - 22620.0 |
| CYCLOPEN | Oswal et al, 2003 ^205^ | | | 1 | | 1 | 303.2 - 303.2 | | | 101.0 - 101.0 |
| CYCLOPEN | Harris et al, 2004 ^446^ | | | 189 | | 157 | 257.9 - 298.2 | | | 100.0 - 250900.0 |
| CYCLOPEN | Pereiro et al, 2004 ^333^ | | | 5 | | 5 | 293.2 - 313.2 | | | 101.0 - 101.0 |
| CYCLOPEN | Gonzalez et al, 2005 ^66^ | | | 3 | | 3 | 293.2 - 303.2 | | | 101.0 - 101.0 |
| CYCLOPEN | Gonzalez et al, 2006 ^447^ | | | 4 | | 4 | 293.2 - 303.2 | | | 101.0 - 101.0 |
| CYCLOPEN | Gonzalez et al, 2007 ^430^ | | | 3 | | 3 | 293.2 - 303.2 | | | 101.0 - 101.0 |
| CYCLOPEN | Kurihara et al, 2007 ^448^ | | | 21 | | 21 | 273.2 - 353.2 | | | 100.0 - 45000.0 |
| CYCLOPEN | Kumar et al, 2011 ^449^ | | | 2 | | 2 | 298.2 - 308.2 | | | 101.0 - 101.0 |
| CYCLOPEN | Kumar et al, 2012 ^450^ | | | 2 | | 2 | 298.2 - 308.2 | | | 101.0 - 101.0 |

| Material | | Author | *N*_all_ | | *N*_used_ | | | *T* / K | *p* / kPa | |
| --- | --- | --- | --- | --- | --- | --- | --- | --- | --- | --- |
| CYCLOPRO | Liessmann et al, 1995 ^5^ | | | 4 | | 3 | 293.2 - 333.2 | | | 619.0 - 1669.0 |
| CYCLOPRO | Dunlop, 1994 ^451^ | | | 1 | | 1 | 298.2 - 298.2 | | | 710.0 - 710.0 |
| CYCLOPRO | Lambert et al, 1955 ^12^ | | | 8 | | 0 | 10000.0 - 0.0 | | | 1000000000.0 - 0.0 |

| Material | Author | *N*_all_ | *N*_used_ | *T* / K | *p* / kPa |
| --- | --- | --- | --- | --- | --- |
| D2 | Lukin et al, 1983 ^452^ | 23 | 23 | 76.5 - 293.2 | 101.0 - 101.0 |
| D2 | Van Itterbeek Paemel, 1940 ^453^ | 10 | 5 | 63.2 - 293.6 | 101.0 - 101.0 |
| D2 | Coremans et al, 1958 ^454^ | 16 | 16 | 20.4 - 73.9 | 13.0 - 13.0 |
| D2 | Rudenko Konareva, 1963 ^455^ | 5 | 5 | 18.8 - 20.4 | 11.0 - 24.0 |
| D2 | Becker Misenta, 1955 ^456^ | 8 | 8 | 14.3 - 90.0 | 0.0 - 0.0 |
| D2 | Van Itterbeek Claes, 1938 ^457^ | 10 | 8 | 18.5 - 293.5 | 5.0 - 16.0 |
| D2 | Van Itterbeek Paemel, 1940 ^453^ | 50 | 26 | 14.5 - 293.1 | 0.0 - 101.0 |
| D2 | Michels et al, 1953 ^458^ | 144 | 143 | 298.2 - 348.2 | 2338.0 - 217778.0 |
| D2 | Menabde, 1965 ^459^ | 10 | 10 | 77.4 - 299.1 | 6.0 - 6.0 |
| D2 | Van Itterbeek Paemel, 1941 ^460^ | 20 | 20 | 18.7 - 23.7 | 11.0 - 83.0 |
| D2 | Borisov et al, 1973 ^98^ | 2 | 2 | 300.0 - 300.0 | 7.0 - 27.0 |
| D2 | Rietveld Velds, 1959 ^461^ | 13 | 11 | 20.4 - 293.1 | 1.0 - 4.0 |
| D2 | Van Itterbeek Paemel, 1940 ^453^ | 3 | 2 | 90.2 - 293.1 | 100.0 - 100.0 |
| D2 | Barua et al, 1964 ^462^ | 37 | 37 | 223.2 - 423.2 | 1444.0 - 16257.0 |
| D2 | Lukin et al, 1981 ^463^ | 3 | 3 | 77.2 - 293.2 | 10.0 - 10.0 |
| D2 | Lukin et al, 1983 ^464^ | 23 | 23 | 76.5 - 293.2 | 7.0 - 7.0 |
| D2 | Konareva Rudenko, 1967 ^465^ | 7 | 7 | 18.8 - 30.0 | 11.0 - 429.0 |
| D2 | Rudenko Slyusar, 1968 ^466^ | 83 | 72 | 38.3 - 300.0 | 101.0 - 286986.0 |
| D2 | Michels et al, 1953 ^458^ | 148 | 148 | 298.2 - 348.2 | 100.0 - 217778.0 |
| D2 | Kestin Leidenfrost, 1959 ^467^ | 9 | 9 | 293.2 - 293.2 | 13.0 - 2168.0 |
| D2 | Konareva Rudenko, 1965 ^468^ | 2 | 2 | 18.8 - 20.3 | 11.0 - 23.0 |
| D2 | De Troyer et al, 1950 ^469^ | 1 | 1 | 293.1 - 293.1 | 101.0 - 101.0 |
| D2 | Kestin Nagashima, 1964 ^470^ | 7 | 7 | 293.2 - 303.2 | 106.0 - 577.0 |
| D2 | Maitland et al, 1983 ^471^ | 1 | 1 | 300.7 - 300.7 | 101.0 - 101.0 |
| D2 | Menabde, 1965 ^459^ | 10 | 10 | 77.4 - 299.1 | 101.0 - 101.0 |
| D2 | Van Itterbeek Claes, 1938 ^457^ | 10 | 8 | 18.5 - 293.5 | 5.0 - 16.0 |
| D2 | Michels et al, 1953 ^458^ | 145 | 145 | 298.2 - 348.2 | 2338.0 - 217780.0 |
| D2 | Van Itterbeek Paemel, 1941 ^460^ | 20 | 20 | 18.7 - 23.7 | 11.0 - 83.0 |
| D2 | konareva Rudenko, 1967 ^465^ | 10 | 8 | 18.8 - 32.0 | 11.0 - 632.0 |
| D2 | Borisov et al, 1973 ^98^ | 1 | 1 | 300.0 - 300.0 | 101.0 - 101.0 |

| Material | Author | *N*_all_ | *N*_used_ | *T* / K | *p* / kPa |
| --- | --- | --- | --- | --- | --- |
| D2O | Rivkin et al, 1974 ^472^ | 17 | 15 | 473.1 - 638.1 | 1449.0 - 20087.0 |
| D2O | Kellomaki, 1975 ^473^ | 5 | 1 | 303.1 - 303.1 | 3.0 - 3.0 |
| D2O | Selwood Frost, 1933 ^474^ | 1 | 1 | 293.1 - 293.1 | 101.0 - 101.0 |
| D2O | Taylor Selwood, 1934 ^475^ | 1 | 1 | 293.1 - 293.1 | 101.0 - 101.0 |
| D2O | Timrot Shuiskaya, 1959 ^476^ | 24 | 24 | 288.3 - 561.2 | 4511.0 - 31872.0 |
| D2O | Tourky et al, 1962 ^477^ | 3 | 3 | 303.1 - 323.1 | 101.0 - 101.0 |
| D2O | Agaev Usibova, 1968 ^478^ | 257 | 257 | 277.1 - 423.1 | 101.0 - 117790.0 |
| D2O | Rivkin et al, 1974 ^472^ | 112 | 111 | 473.1 - 648.1 | 4120.0 - 50000.0 |
| D2O | Rivkin et al, 1976 ^479^ | 20 | 20 | 323.1 - 448.1 | 10000.0 - 50000.0 |
| D2O | Harris et al, 2004 ^446^ | 128 | 128 | 255.7 - 298.2 | 100.0 - 395200.0 |
| D2O | Timrot et al, 1974 ^480^ | 15 | 15 | 374.9 - 777.9 | 8.0 - 127.0 |
| D2O | Rivkin et al, 1977 ^481^ | 24 | 24 | 673.1 - 773.1 | 25000.0 - 50000.0 |

| Material | Author | *N*_all_ | *N*_used_ | *T* / K | *p* / kPa |
| --- | --- | --- | --- | --- | --- |
| D4 | Fischer Weiss, 1986 ^158^ | 1 | 1 | 298.2 - 298.2 | 101.0 - 101.0 |
| D4 | Dickinson, 1977 ^482^ | 2 | 2 | 323.2 - 323.2 | 30000.0 - 52000.0 |
| D4 | Abbas, 2010 ^483^ | 11 | 7 | 313.2 - 373.2 | 0.0 - 8.0 |
| D4 | Fischer, 1986 ^484^ | 6 | 4 | 318.8 - 364.7 | 1.0 - 6.0 |
| D4 | Markgraf, 1968 ^485^ | 4 | 1 | 318.2 - 318.2 | 1.0 - 1.0 |
| D4 | Fischer Weiss, 1986 ^158^ | 2 | 0 | 10000.0 - 0.0 | 1000000000.0 - 0.0 |
| D4 | Fischer Weiss, 1986 ^486^ | 7 | 7 | 285.0 - 345.0 | 101.0 - 101.0 |
| D4 | Palczewska Oracz, 2005 ^487^ | 13 | 13 | 293.2 - 423.2 | 101.0 - 101.0 |

| Material | Author | *N*_all_ | *N*_used_ | *T* / K | *p* / kPa |
| --- | --- | --- | --- | --- | --- |
| D5 | Fischer Weiss, 1986 ^158^ | 1 | 0 | 10000.0 - 0.0 | 1000000000.0 - 0.0 |
| D5 | Fischer, 1986 ^484^ | 8 | 2 | 336.1 - 350.8 | 0.0 - 1.0 |
| D5 | Palczewska Oracz, 2005 ^487^ | 15 | 6 | 343.2 - 423.2 | 1.0 - 17.0 |
| D5 | Abbas et al, 2010 ^483^ | 17 | 4 | 343.2 - 373.2 | 1.0 - 2.0 |
| D5 | Fischer Weiss, 1986 ^486^ | 7 | 7 | 285.0 - 345.0 | 101.0 - 101.0 |
| D5 | Palczewska Oracz, 2005 ^487^ | 15 | 12 | 293.2 - 423.2 | 101.0 - 101.0 |

| Material | Author | *N*_all_ | *N*_used_ | *T* / K | *p* / kPa |
| --- | --- | --- | --- | --- | --- |
| DEA | Kuss, 1955 ^114^ | 8 | 0 | 10000.0 - 0.0 | 1000000000.0 - 0.0 |
| DEA | Whorton Amis, 1956 ^488^ | 1 | 0 | 10000.0 - 0.0 | 1000000000.0 - 0.0 |
| DEA | DiGuilio et al, 1992 ^489^ | 9 | 8 | 303.8 - 423.2 | 101.0 - 101.0 |
| DEA | Teng et al, 1994 ^490^ | 5 | 5 | 298.2 - 353.2 | 101.0 - 101.0 |
| DEA | Aguila-Hernandez et al, 2008 ^318^ | 20 | 20 | 303.2 - 423.2 | 101.0 - 101.0 |
| DEA | Blanco et al, 2013 ^491^ | 4 | 3 | 303.2 - 323.2 | 100.0 - 100.0 |
| DEA | Li et al, 2003 ^105^ | 5 | 4 | 303.2 - 333.2 | 101.0 - 101.0 |
| DEA | Lopez et al, 2013 ^492^ | 5 | 4 | 298.2 - 323.2 | 101.0 - 101.0 |
| DEA | Spasojevic et al, 2013 ^493^ | 1 | 1 | 298.2 - 298.2 | 101.0 - 101.0 |
| DEA | Haghtalab shojaeian, 2014 ^494^ | 6 | 6 | 303.2 - 353.2 | 101.0 - 101.0 |

| Material | Author | *N*_all_ | *N*_used_ | *T* / K | *p* / kPa |
| --- | --- | --- | --- | --- | --- |
| DECANE | Golubev, 1959 ^8^ | 6 | 0 | 10000.0 - 0.0 | 1000000000.0 - 0.0 |
| DECANE | Bingham Fornwalt, 1930 ^265^ | 16 | 16 | 273.2 - 373.1 | 101.0 - 101.0 |
| DECANE | Shepard et al, 1931 ^266^ | 1 | 1 | 298.1 - 298.1 | 101.0 - 101.0 |
| DECANE | Sage et al, 1940 ^495^ | 1 | 1 | 310.9 - 310.9 | 101.0 - 101.0 |
| DECANE | Mumford Phillips, 1950 ^361^ | 2 | 2 | 293.1 - 298.1 | 101.0 - 101.0 |
| DECANE | Lee Ellington, 1965 ^496^ | 49 | 49 | 310.9 - 444.3 | 1379.0 - 55158.0 |
| DECANE | Carmichael et al, 1969 ^497^ | 43 | 43 | 277.6 - 477.6 | 110.0 - 36038.0 |
| DECANE | Moore Wellek, 1974 ^498^ | 4 | 4 | 293.1 - 313.1 | 101.0 - 101.0 |
| DECANE | Ratkovics et al, 1974 ^250^ | 1 | 1 | 293.1 - 293.1 | 101.0 - 101.0 |
| DECANE | Jambon Delmas, 1977 ^281^ | 1 | 1 | 298.1 - 298.1 | 101.0 - 101.0 |
| DECANE | Atrops et al, 1982 ^499^ | 2 | 2 | 293.1 - 313.1 | 101.0 - 101.0 |
| DECANE | Kashiwagi Makita, 1982 ^152^ | 48 | 48 | 298.1 - 348.1 | 100.0 - 110000.0 |
| DECANE | Awwad Allos, 1985 ^282^ | 1 | 1 | 298.1 - 298.1 | 101.0 - 101.0 |
| DECANE | Aucejo et al, 1986 ^283^ | 1 | 1 | 298.1 - 298.1 | 101.0 - 101.0 |
| DECANE | Awwad et al, 1986 ^156^ | 1 | 1 | 298.1 - 298.1 | 101.0 - 101.0 |
| DECANE | Awwad Salman, 1986 ^284^ | 1 | 1 | 298.1 - 298.1 | 101.0 - 101.0 |
| DECANE | Ducoulombier et al, 1986 ^285^ | 30 | 30 | 293.1 - 373.1 | 100.0 - 100000.0 |
| DECANE | Wakefield Marsh, 1987 ^286^ | 3 | 3 | 318.1 - 338.1 | 101.0 - 101.0 |
| DECANE | Wakefield, 1988 ^287^ | 2 | 2 | 303.2 - 308.1 | 101.0 - 101.0 |
| DECANE | Wakefield et al, 1988 ^500^ | 3 | 3 | 318.2 - 338.1 | 101.0 - 101.0 |
| DECANE | Knapstad et al, 1989 ^164^ | 11 | 11 | 293.1 - 423.1 | 101.0 - 101.0 |
| DECANE | Iwahashi et al, 1990 ^271^ | 2 | 2 | 298.1 - 323.1 | 101.0 - 101.0 |
| DECANE | Assael et al, 1992 ^501^ | 60 | 60 | 293.8 - 333.2 | 100.0 - 64240.0 |
| DECANE | Oliveria Wakeham, 1992 ^502^ | 46 | 32 | 303.2 - 348.2 | 100.0 - 126260.0 |
| DECANE | Papaioannou et al, 1993 ^503^ | 4 | 4 | 293.2 - 308.2 | 101.0 - 101.0 |
| DECANE | Aminabhavi Gopalkrishna, 1994 ^289^ | 3 | 3 | 298.2 - 318.2 | 101.0 - 101.0 |
| DECANE | Aminabhavi Gopalkrishna, 1995 ^290^ | 4 | 4 | 298.2 - 313.2 | 101.0 - 101.0 |
| DECANE | Aucejo et al, 1995 ^14^ | 1 | 1 | 298.2 - 298.2 | 101.0 - 101.0 |
| DECANE | Aminabhavi et al, 1996 ^417^ | 3 | 3 | 298.2 - 308.2 | 101.0 - 101.0 |
| DECANE | Aminabhavi Patil, 1997 ^291^ | 3 | 3 | 298.2 - 308.2 | 101.0 - 101.0 |
| DECANE | Dandekar et al, 1998 ^314^ | 1 | 1 | 293.2 - 293.2 | 101.0 - 101.0 |
| DECANE | Estrada-Baltazar et al, 1998 ^504^ | 36 | 36 | 298.0 - 373.4 | 101.0 - 24626.0 |
| DECANE | Aralaguppi et al, 1999 ^189^ | 3 | 3 | 298.2 - 308.2 | 101.0 - 101.0 |
| DECANE | Franjo et al, 2001 ^505^ | 1 | 1 | 298.2 - 298.2 | 101.0 - 101.0 |
| DECANE | Nayak et al, 2001 ^292^ | 3 | 3 | 298.2 - 308.2 | 101.0 - 101.0 |
| DECANE | Segade et al, 2001 ^506^ | 1 | 1 | 308.2 - 308.2 | 101.0 - 101.0 |
| DECANE | Tohidi et al, 2001 ^507^ | 21 | 18 | 323.0 - 423.0 | 2250.0 - 106290.0 |
| DECANE | Garcia et al, 2002 ^293^ | 5 | 5 | 278.2 - 318.2 | 101.0 - 101.0 |
| DECANE | Gomez-Diaz et al, 2002 ^424^ | 1 | 1 | 298.2 - 298.2 | 101.0 - 101.0 |
| DECANE | Gonzalez et al, 2003 ^294^ | 3 | 3 | 293.2 - 303.2 | 101.0 - 101.0 |
| DECANE | Audonnet Padua, 2004 ^508^ | 40 | 40 | 303.2 - 393.2 | 100.0 - 76190.0 |
| DECANE | Caudwell et al, 2004 ^297^ | 34 | 22 | 298.2 - 373.2 | 100.0 - 102800.0 |
| DECANE | Caudwell et al, 2004 ^297^ | 43 | 43 | 323.2 - 423.2 | 100.0 - 88200.0 |
| DECANE | Gonzalez et al, 2004 ^298^ | 3 | 3 | 293.2 - 303.2 | 101.0 - 101.0 |
| DECANE | Gonzalez et al, 2004 ^298^ | 3 | 3 | 293.2 - 303.2 | 101.0 - 101.0 |
| DECANE | Aljimaz et al, 2005 ^300^ | 3 | 3 | 293.2 - 303.2 | 101.0 - 101.0 |
| DECANE | Kapoor Rattan, 2005 ^509^ | 3 | 3 | 303.2 - 313.2 | 101.0 - 101.0 |
| DECANE | Queimada et al, 2005 ^347^ | 6 | 6 | 293.2 - 343.2 | 101.0 - 101.0 |
| DECANE | Tripathi, 2005 ^317^ | 1 | 1 | 298.2 - 298.2 | 101.0 - 101.0 |
| DECANE | Baragi Aralaguppi, 2006 ^337^ | 3 | 3 | 298.2 - 308.2 | 101.0 - 101.0 |
| DECANE | Iloukhani et al, 2006 ^339^ | 3 | 3 | 293.2 - 303.2 | 101.0 - 101.0 |
| DECANE | Dubey Sharma, 2007 ^510^ | 1 | 1 | 298.2 - 298.2 | 101.0 - 101.0 |
| DECANE | Dubey Sharma, 2008 ^319^ | 1 | 1 | 298.2 - 298.2 | 101.0 - 101.0 |
| DECANE | Dubey Sharma, 2008 ^319^ | 1 | 1 | 298.2 - 298.2 | 101.0 - 101.0 |
| DECANE | Dubey Sharma, 2008 ^319^ | 1 | 1 | 298.2 - 298.2 | 101.0 - 101.0 |
| DECANE | Dubey Sharma, 2008 ^319^ | 1 | 1 | 298.2 - 298.2 | 101.0 - 101.0 |
| DECANE | Fang et al, 2008 ^511^ | 20 | 20 | 313.2 - 473.2 | 100.0 - 3200.0 |
| DECANE | Shukla et al, 2008 ^224^ | 1 | 1 | 298.2 - 298.2 | 101.0 - 101.0 |
| DECANE | Yang et al, 2008 ^512^ | 4 | 4 | 293.2 - 313.2 | 101.0 - 101.0 |
| DECANE | Caudwell et al, 2009 ^513^ | 32 | 20 | 298.2 - 373.2 | 100.0 - 102800.0 |
| DECANE | Feitosa et al, 2009 ^514^ | 2 | 2 | 293.2 - 298.2 | 101.0 - 101.0 |
| DECANE | Sato et al, 2010 ^515^ | 7 | 7 | 323.1 - 333.1 | 100.0 - 10200.0 |
| DECANE | Zivkovic et al, 2010 ^516^ | 1 | 1 | 303.2 - 303.2 | 101.0 - 101.0 |
| DECANE | Gong et al, 2012 ^517^ | 4 | 4 | 293.2 - 313.2 | 101.0 - 101.0 |
| DECANE | Hamzehlouia Asfour, 2012 ^383^ | 2 | 2 | 308.2 - 313.2 | 101.0 - 101.0 |
| DECANE | Chi et al, 2013 ^274^ | 7 | 7 | 293.2 - 323.2 | 100.0 - 100.0 |
| DECANE | Mahajan Mirgane, 2013 ^304^ | 1 | 1 | 298.2 - 298.2 | 101.0 - 101.0 |
| DECANE | Fang et al, 2014 ^518^ | 4 | 4 | 283.2 - 303.2 | 101.0 - 101.0 |
| DECANE | Liu Zhu, 2014 ^307^ | 5 | 5 | 293.2 - 313.2 | 100.0 - 100.0 |
| DECANE | Qin et al, 2014 ^519^ | 9 | 9 | 293.2 - 363.2 | 100.0 - 100.0 |
| DECANE | Carmichael et al, 1969 ^497^ | 6 | 6 | 377.6 - 477.6 | 7.0 - 110.0 |
| DECANE | Slusar et al, 1973 ^310^ | 3 | 2 | 518.7 - 613.5 | 101.0 - 101.0 |

| Material | Author | *N*_all_ | *N*_used_ | *T* / K | *p* / kPa |
| --- | --- | --- | --- | --- | --- |
| DEE | Thorpe Rodger, 1894 ^28^ | 12 | 12 | 280.3 - 305.2 | 35.0 - 95.0 |
| DEE | Bulanov et al, 1974 ^117^ | 20 | 20 | 293.1 - 368.1 | 60.0 - 589.0 |
| DEE | Faust, 1912 ^33^ | 3 | 3 | 273.2 - 305.1 | 101.0 - 101.0 |
| DEE | Kendall Wright, 1920 ^520^ | 1 | 1 | 298.1 - 298.1 | 101.0 - 101.0 |
| DEE | Weissenberger Piatti, 1924 ^34^ | 1 | 1 | 291.1 - 291.1 | 101.0 - 101.0 |
| DEE | Lewis, 1925 ^36^ | 1 | 1 | 298.1 - 298.1 | 101.0 - 101.0 |
| DEE | Timmermans Martin, 1928 ^37^ | 3 | 3 | 273.2 - 303.1 | 101.0 - 101.0 |
| DEE | Bingha Brown, 1932 ^38^ | 3 | 3 | 283.1 - 303.1 | 101.0 - 101.0 |
| DEE | George Sastry, 2003 ^202^ | 1 | 1 | 298.2 - 298.2 | 101.0 - 101.0 |
| DEE | Meng et al, 2008 ^521^ | 70 | 68 | 243.2 - 373.2 | 100.0 - 19610.0 |
| DEE | Sah et al, 2010 ^522^ | 1 | 1 | 298.2 - 298.2 | 101.0 - 101.0 |
| DEE | Sah Roy, 2011 ^523^ | 1 | 1 | 298.2 - 298.2 | 101.0 - 101.0 |
| DEE | Dash et al, 2012 ^524^ | 1 | 1 | 303.2 - 303.2 | 101.0 - 101.0 |
| DEE | Titani, 1933 ^75^ | 7 | 7 | 394.9 - 582.1 | 101.0 - 101.0 |
| DEE | Adzumi, 1937 ^244^ | 1 | 1 | 293.1 - 293.1 | 0.0 - 0.0 |

| Material | Author | *N*_all_ | *N*_used_ | *T* / K | *p* / kPa |
| --- | --- | --- | --- | --- | --- |
| DMC | Bi et al, 2003 ^525^ | 21 | 21 | 282.3 - 383.1 | 3.0 - 185.0 |
| DMC | Wright, 1961 ^526^ | 7 | 7 | 293.1 - 353.1 | 101.0 - 101.0 |
| DMC | Naejus et al, 1997 ^527^ | 2 | 2 | 298.2 - 313.2 | 101.0 - 101.0 |
| DMC | Aminabhavi Banerjee, 1998 ^419^ | 3 | 3 | 298.2 - 308.2 | 101.0 - 101.0 |
| DMC | Pal et al, 1998 ^528^ | 1 | 1 | 298.2 - 298.2 | 101.0 - 101.0 |
| DMC | Pal et al, 1999 ^529^ | 1 | 1 | 298.2 - 298.2 | 101.0 - 101.0 |
| DMC | Pal et al, 1999 ^529^ | 1 | 1 | 298.2 - 298.2 | 101.0 - 101.0 |
| DMC | Comunas et al, 2001 ^530^ | 40 | 40 | 293.2 - 353.2 | 100.0 - 100000.0 |
| DMC | Pal et al, 2001 ^531^ | 3 | 3 | 298.2 - 318.2 | 101.0 - 101.0 |
| DMC | Mialkowski et al, 2002 ^532^ | 1 | 1 | 298.2 - 298.2 | 101.0 - 101.0 |
| DMC | Moreiras et al,2003 ^295^ | 1 | 1 | 298.2 - 298.2 | 101.0 - 101.0 |
| DMC | Li et al, 2004 ^375^ | 1 | 1 | 298.2 - 298.2 | 101.0 - 101.0 |
| DMC | Pereiro et al, 2004 ^333^ | 5 | 5 | 293.2 - 313.2 | 101.0 - 101.0 |
| DMC | Rodiguez et al, 2004 ^533^ | 4 | 4 | 293.2 - 313.2 | 101.0 - 101.0 |
| DMC | Yang et al, 2004 ^209^ | 12 | 12 | 293.2 - 343.2 | 101.0 - 101.0 |
| DMC | Queimada et al, 2005 ^347^ | 1 | 1 | 298.2 - 298.2 | 101.0 - 101.0 |
| DMC | Arbad et al, 2006 ^534^ | 2 | 2 | 288.2 - 298.2 | 101.0 - 101.0 |
| DMC | Comelli et al, 2006 ^535^ | 1 | 1 | 308.2 - 308.2 | 101.0 - 101.0 |
| DMC | Rodiguez et al, 2006 ^536^ | 4 | 4 | 293.2 - 313.2 | 101.0 - 101.0 |
| DMC | Iloukhani et al, 2007 ^537^ | 1 | 1 | 298.2 - 298.2 | 101.0 - 101.0 |
| DMC | Meng et al, 2009 ^538^ | 40 | 40 | 283.2 - 353.2 | 100.0 - 19540.0 |
| DMC | Xing et al, 2009 ^539^ | 1 | 1 | 298.2 - 298.2 | 101.0 - 101.0 |
| DMC | Comelli et al, 2010 ^540^ | 2 | 2 | 293.2 - 313.2 | 101.0 - 101.0 |
| DMC | Litaiem Dhahbi, 2012 ^541^ | 5 | 5 | 298.2 - 318.2 | 101.0 - 101.0 |
| DME | Meng et al, 2012 ^542^ | 27 | 27 | 243.2 - 373.1 | 80.0 - 3303.0 |
| DME | Van Cleav Maass, 1935 ^87^ | 8 | 8 | 216.3 - 296.0 | 0.0 - 0.0 |
| DME | Tomida et al, 2009 ^543^ | 40 | 40 | 298.2 - 423.2 | 101.0 - 4912.0 |
| DME | Wang et al, 2011 ^544^ | 54 | 54 | 299.6 - 395.9 | 101.0 - 1248.0 |
| DME | Cousins Laesecke, 2012 ^545^ | 23 | 23 | 242.5 - 350.1 | 78.0 - 2098.0 |
| DME | Meng et al, 2012 ^542^ | 77 | 77 | 253.2 - 373.1 | 5000.0 - 30000.0 |

| Material | Author | *N*_all_ | *N*_used_ | *T* / K | *p* / kPa |
| --- | --- | --- | --- | --- | --- |
| EBENZENE | Thorpe Rodger, 1894 ^28^ | 12 | 12 | 273.6 - 404.5 | 0.0 - 88.0 |
| EBENZENE | Batschinski, 1913 ^29^ | 14 | 14 | 273.1 - 403.1 | 0.0 - 84.0 |
| EBENZENE | Gartenmeister, 1890 ^118^ | 1 | 1 | 293.1 - 293.1 | 101.0 - 101.0 |
| EBENZENE | Thorpe Rodger, 1894 ^28^ | 12 | 12 | 273.6 - 404.5 | 101.0 - 101.0 |
| EBENZENE | Thorpe Rodger, 1897 ^546^ | 11 | 11 | 273.4 - 405.1 | 100.0 - 101.0 |
| EBENZENE | Thorpe Rodger, 1897 ^546^ | 11 | 11 | 273.4 - 405.1 | 101.0 - 101.0 |
| EBENZENE | Dunstan et al, 1913 ^122^ | 1 | 1 | 298.1 - 298.1 | 101.0 - 101.0 |
| EBENZENE | Timmermans Martin, 1926 ^131^ | 3 | 3 | 288.1 - 304.1 | 101.0 - 101.0 |
| EBENZENE | Schmidt et al, 1939 ^547^ | 7 | 7 | 283.1 - 343.1 | 101.0 - 101.0 |
| EBENZENE | Panchenkov Erchenkov, 1962 ^548^ | 6 | 6 | 283.1 - 353.1 | 101.0 - 101.0 |
| EBENZENE | Barlow et al, 1966 ^549^ | 4 | 3 | 173.0 - 303.2 | 101.0 - 101.0 |
| EBENZENE | Blank, 1968 ^44^ | 1 | 1 | 298.1 - 298.1 | 101.0 - 101.0 |
| EBENZENE | Akhundov, 1973 ^550^ | 131 | 131 | 302.1 - 548.1 | 100.0 - 40000.0 |
| EBENZENE | Shikhaliev, 1977 ^551^ | 1 | 1 | 293.0 - 293.0 | 101.0 - 101.0 |
| EBENZENE | Kashiwagi Makita, 1982 ^152^ | 48 | 48 | 298.1 - 348.1 | 100.0 - 110000.0 |
| EBENZENE | Oswal Rathnam, 1984 ^155^ | 1 | 1 | 303.1 - 303.1 | 101.0 - 101.0 |
| EBENZENE | Abdullaev Akhundov, 1985 ^552^ | 104 | 104 | 288.2 - 548.1 | 100.0 - 60000.0 |
| EBENZENE | Awwad et al, 1985 ^156^ | 1 | 1 | 298.1 - 298.1 | 101.0 - 101.0 |
| EBENZENE | Fermeglia Lapasin, 1988 ^553^ | 1 | 1 | 298.1 - 298.1 | 101.0 - 101.0 |
| EBENZENE | Singh et al, 1989 ^554^ | 5 | 5 | 298.1 - 333.1 | 101.0 - 101.0 |
| EBENZENE | Schumpe Luehring, 1990 ^54^ | 1 | 1 | 293.2 - 293.2 | 101.0 - 101.0 |
| EBENZENE | Krahn Luft, 1994 ^555^ | 16 | 15 | 298.2 - 453.2 | 100.0 - 195000.0 |
| EBENZENE | Ramadevi et al, 1996 ^371^ | 2 | 2 | 303.2 - 313.2 | 101.0 - 101.0 |
| EBENZENE | George Sastry, 2003 ^202^ | 2 | 2 | 298.2 - 308.2 | 101.0 - 101.0 |
| EBENZENE | Katyal et al, 2003 ^556^ | 3 | 3 | 293.2 - 313.2 | 101.0 - 101.0 |
| EBENZENE | Lark et al, 2003 ^203^ | 2 | 2 | 298.2 - 303.2 | 101.0 - 101.0 |
| EBENZENE | Oswal et al, 2003 ^205^ | 1 | 1 | 303.2 - 303.2 | 101.0 - 101.0 |
| EBENZENE | Oswal et al, 2004 ^208^ | 1 | 1 | 303.2 - 303.2 | 101.0 - 101.0 |
| EBENZENE | Rattan et al, 2004 ^557^ | 2 | 2 | 298.2 - 308.2 | 101.0 - 101.0 |
| EBENZENE | Yang et al, 2004 ^209^ | 3 | 3 | 298.2 - 323.2 | 101.0 - 101.0 |
| EBENZENE | Yang et al, 2004 ^209^ | 5 | 5 | 298.2 - 333.2 | 101.0 - 101.0 |
| EBENZENE | Rathnam et al, 2005 ^558^ | 2 | 2 | 303.2 - 313.2 | 101.0 - 101.0 |
| EBENZENE | Yang et al, 2005 ^559^ | 6 | 6 | 303.2 - 353.2 | 101.0 - 101.0 |
| EBENZENE | Aljimaz et al, 2006 ^300^ | 2 | 2 | 293.2 - 303.2 | 101.0 - 101.0 |
| EBENZENE | Baskaran Kubendran, 2008 ^379^ | 3 | 3 | 303.2 - 323.2 | 101.0 - 101.0 |
| EBENZENE | Song et al, 2008 ^225^ | 7 | 7 | 303.2 - 333.2 | 101.0 - 101.0 |
| EBENZENE | Wankhede et al, 2008 ^226^ | 3 | 3 | 288.2 - 308.2 | 101.0 - 101.0 |
| EBENZENE | Dominguez Perez et al, 2009 ^560^ | 1 | 1 | 298.2 - 298.2 | 101.0 - 101.0 |
| EBENZENE | Baylaucq et al, 2009 ^228^ | 2 | 2 | 308.2 - 313.2 | 101.0 - 101.0 |
| EBENZENE | Rathnam et al, 2009 ^561^ | 2 | 2 | 303.2 - 313.2 | 101.0 - 101.0 |
| EBENZENE | Ikeuchi et al, 2010 ^233^ | 6 | 6 | 283.2 - 308.2 | 101.0 - 101.0 |
| EBENZENE | Rathnam et al, 2010 ^562^ | 2 | 2 | 303.2 - 313.2 | 101.0 - 101.0 |
| EBENZENE | Bhatia et al, 2011 ^563^ | 2 | 2 | 298.2 - 308.2 | 101.0 - 101.0 |
| EBENZENE | Rathnam et al, 2011 ^564^ | 2 | 2 | 303.2 - 313.2 | 101.0 - 101.0 |
| EBENZENE | Saravanakumar et al, 2012 ^565^ | 1 | 1 | 303.2 - 303.2 | 101.0 - 101.0 |
| EBENZENE | Barega et al, 2013 ^566^ | 4 | 4 | 298.2 - 313.2 | 101.0 - 101.0 |
| EBENZENE | Prak et al, 2014 ^308^ | 8 | 8 | 293.2 - 373.2 | 101.0 - 101.0 |
| EBENZENE | Abdullaev Akhundov, 1983 ^153^ | 29 | 28 | 473.1 - 673.1 | 103.0 - 4599.0 |

| Material | Author | *N*_all_ | *N*_used_ | *T* / K | *p* / kPa |
| --- | --- | --- | --- | --- | --- |
| EGLYCOL | Zhang et al, 2008 ^567^ | 4 | 0 | 10000.0 - 0.0 | 1000000000.0 - 0.0 |
| EGLYCOL | Taylor Rinkenbach, 1926 ^568^ | 1 | 1 | 299.1 - 299.1 | 101.0 - 101.0 |
| EGLYCOL | Rinkenbach, 1927 ^569^ | 6 | 6 | 288.1 - 300.1 | 101.0 - 101.0 |
| EGLYCOL | Timmermans Hennaut, 1935 ^570^ | 2 | 2 | 288.1 - 303.1 | 101.0 - 101.0 |
| EGLYCOL | Mellan, 1962 ^571^ | 4 | 4 | 283.1 - 333.1 | 101.0 - 101.0 |
| EGLYCOL | Davids Findlay, 1967 ^572^ | 9 | 9 | 292.8 - 332.6 | 101.0 - 101.0 |
| EGLYCOL | Jerome et al, 1968 ^573^ | 1 | 1 | 298.1 - 298.1 | 101.0 - 101.0 |
| EGLYCOL | Oyevaar et al, 1989 ^574^ | 1 | 1 | 298.0 - 298.0 | 101.0 - 101.0 |
| EGLYCOL | Kumagai et al, 1993 ^575^ | 4 | 3 | 293.2 - 333.2 | 101.0 - 101.0 |
| EGLYCOL | Reddy et al, 1994 ^576^ | 1 | 1 | 308.2 - 308.2 | 101.0 - 101.0 |
| EGLYCOL | Aminabhavi Gopalakrishna, 1995 ^290^ | 1 | 0 | 10000.0 - 0.0 | 1000000000.0 - 0.0 |
| EGLYCOL | Pal Singh, 1997 ^577^ | 2 | 2 | 303.2 - 308.2 | 101.0 - 101.0 |
| EGLYCOL | Aminabhavi Banerjee, 1998 ^419^ | 3 | 3 | 298.2 - 308.2 | 101.0 - 101.0 |
| EGLYCOL | Pal sharma, 1998 ^578^ | 1 | 1 | 298.2 - 298.2 | 101.0 - 101.0 |
| EGLYCOL | Tsierkezos Molinou, 1998 ^579^ | 4 | 4 | 283.2 - 313.2 | 101.0 - 101.0 |
| EGLYCOL | Saleh et al, 1999 ^580^ | 5 | 5 | 303.2 - 323.2 | 101.0 - 101.0 |
| EGLYCOL | Tsierkezos Molinou, 1999 ^59^ | 1 | 1 | 293.2 - 293.2 | 101.0 - 101.0 |
| EGLYCOL | Lech et al, 2001 ^581^ | 15 | 15 | 293.4 - 359.3 | 101.0 - 101.0 |
| EGLYCOL | Vijaya Kumar Naidu et al, 2002 ^582^ | 1 | 1 | 308.2 - 308.2 | 101.0 - 101.0 |
| EGLYCOL | Naidu et al, 2003 ^583^ | 1 | 1 | 308.2 - 308.2 | 101.0 - 101.0 |
| EGLYCOL | Nayak et al, 2003 ^374^ | 3 | 2 | 298.2 - 303.2 | 101.0 - 101.0 |
| EGLYCOL | Sastry Patel, 2003 ^584^ | 2 | 2 | 298.2 - 308.2 | 101.0 - 101.0 |
| EGLYCOL | Sun Teja, 2003 ^585^ | 8 | 8 | 295.6 - 428.3 | 100.0 - 100.0 |
| EGLYCOL | Yang et al, 2003 ^586^ | 7 | 7 | 293.2 - 353.2 | 101.0 - 101.0 |
| EGLYCOL | Sinha Roy, 2006 ^587^ | 3 | 3 | 298.2 - 318.2 | 101.0 - 101.0 |
| EGLYCOL | Sinha Roy, 2006 ^587^ | 3 | 3 | 298.2 - 318.2 | 101.0 - 101.0 |
| EGLYCOL | Yang et al, 2006 ^588^ | 7 | 7 | 308.2 - 353.2 | 101.0 - 101.0 |
| EGLYCOL | Yang et al, 2006 ^589^ | 7 | 7 | 308.2 - 353.2 | 101.0 - 101.0 |
| EGLYCOL | Gurung Roy, 2007 ^590^ | 3 | 3 | 298.2 - 318.2 | 101.0 - 101.0 |
| EGLYCOL | Mehta et al, 2007 ^591^ | 1 | 1 | 308.2 - 308.2 | 101.0 - 101.0 |
| EGLYCOL | Song et al, 2007 ^592^ | 1 | 1 | 293.2 - 293.2 | 101.0 - 101.0 |
| EGLYCOL | Wu et al, 2007 ^593^ | 6 | 4 | 303.0 - 373.0 | 101.0 - 101.0 |
| EGLYCOL | Mehta et al, 2008 ^594^ | 1 | 1 | 308.2 - 308.2 | 101.0 - 101.0 |
| EGLYCOL | Nain, 2008 ^595^ | 6 | 6 | 293.2 - 318.2 | 101.0 - 101.0 |
| EGLYCOL | Shi et al, 2008 ^596^ | 1 | 1 | 295.0 - 295.0 | 101.0 - 101.0 |
| EGLYCOL | Zorebski Lubowiecka, 2009 ^597^ | 4 | 4 | 298.2 - 313.2 | 101.0 - 101.0 |
| EGLYCOL | Doghaeei et al, 2010 ^598^ | 3 | 3 | 298.2 - 308.2 | 101.0 - 101.0 |
| EGLYCOL | Ge et al, 2010 ^599^ | 9 | 9 | 298.2 - 338.2 | 101.0 - 101.0 |
| EGLYCOL | Li et al, 2010 ^600^ | 3 | 3 | 298.2 - 308.2 | 101.0 - 101.0 |
| EGLYCOL | Kinart et al, 2011 ^601^ | 1 | 1 | 298.2 - 298.2 | 101.0 - 101.0 |
| EGLYCOL | kinart Maj, 2011 ^602^ | 1 | 1 | 303.2 - 303.2 | 101.0 - 101.0 |
| EGLYCOL | Sagdeev et al, 2011 ^603^ | 9 | 9 | 293.2 - 464.4 | 101.0 - 101.0 |
| EGLYCOL | Sagdeev et al, 2012 ^604^ | 53 | 46 | 293.2 - 464.6 | 98.0 - 245160.0 |
| EGLYCOL | Gao et al, 2013 ^605^ | 5 | 5 | 298.2 - 318.2 | 101.0 - 101.0 |
| EGLYCOL | Moosavi et al, 2013 ^606^ | 3 | 3 | 298.2 - 308.2 | 101.0 - 101.0 |
| EGLYCOL | Moosavi et al, 2013 ^606^ | 3 | 3 | 298.2 - 308.2 | 101.0 - 101.0 |
| EGLYCOL | Quijada-Maldonado et al, 2013 ^607^ | 7 | 7 | 298.2 - 328.2 | 101.0 - 101.0 |
| EGLYCOL | Kondaiah et al, 2014 ^608^ | 1 | 1 | 308.2 - 308.2 | 101.0 - 101.0 |
| EGLYCOL | Li et al, 2014 ^609^ | 4 | 4 | 313.2 - 343.2 | 101.0 - 101.0 |
| EGLYCOL | Pastoriza-Gallego et al, 2014 ^610^ | 8 | 8 | 283.2 - 323.2 | 101.0 - 101.0 |
| EGLYCOL | Zhang et al, 2014 ^611^ | 1 | 1 | 295.2 - 295.2 | 101.0 - 101.0 |
| EGLYCOL | Sun et al, 2015 ^612^ | 5 | 5 | 298.2 - 318.2 | 101.0 - 101.0 |
| EGLYCOL | Zhang et al, 2015 ^613^ | 6 | 6 | 298.2 - 323.2 | 101.0 - 101.0 |

| Material | Author | *N*_all_ | *N*_used_ | *T* / K | *p* / kPa |
| --- | --- | --- | --- | --- | --- |
| ETHANE | Diller Saber, 1981 ^614^ | 72 | 72 | 100.0 - 250.0 | 300.0 - 32108.0 |
| ETHANE | Gerf Galkov, 1940 ^615^ | 13 | 10 | 108.0 - 167.3 | 0.0 - 35.0 |
| ETHANE | Galkov Gerf, 1941 ^616^ | 9 | 9 | 111.2 - 164.0 | 0.0 - 28.0 |
| ETHANE | Gerf Galkov, 1941 ^616^ | 11 | 10 | 172.8 - 273.1 | 49.0 - 2389.0 |
| ETHANE | Rabe, 1981 ^390^ | 6 | 6 | 185.0 - 300.0 | 101.0 - 4365.0 |
| ETHANE | Vogel, 1914 ^80^ | 1 | 1 | 273.2 - 273.2 | 101.0 - 101.0 |
| ETHANE | Ishida, 1923 ^617^ | 1 | 1 | 296.1 - 296.1 | 101.0 - 101.0 |
| ETHANE | Titani, 1930 ^9^ | 6 | 6 | 293.1 - 393.1 | 101.0 - 101.0 |
| ETHANE | Trautz Sorg, 1931 ^618^ | 6 | 6 | 290.3 - 523.2 | 101.0 - 101.0 |
| ETHANE | Adzumi, 1937 ^244^ | 9 | 9 | 293.1 - 373.1 | 101.0 - 101.0 |
| ETHANE | Craven Lambert, 1951 ^76^ | 4 | 4 | 308.1 - 350.9 | 101.0 - 101.0 |
| ETHANE | Lambert et al, 1955 ^12^ | 4 | 4 | 308.1 - 351.1 | 101.0 - 101.0 |
| ETHANE | De Rocco Halford, 1958 ^619^ | 18 | 18 | 250.1 - 473.2 | 101.0 - 101.0 |
| ETHANE | Baron et al, 1959 ^620^ | 40 | 40 | 324.8 - 408.1 | 689.0 - 55158.0 |
| ETHANE | Golubev Agaev, 1964 ^253^ | 9 | 9 | 273.2 - 523.2 | 101.0 - 101.0 |
| ETHANE | Kestin et al, 1971 ^254^ | 2 | 2 | 296.4 - 303.7 | 101.0 - 101.0 |
| ETHANE | Diaz Pena Cheda, 1975 ^255^ | 13 | 13 | 305.3 - 408.1 | 27.0 - 27.0 |
| ETHANE | Kestin et al, 1977 ^256^ | 5 | 5 | 301.4 - 476.8 | 101.0 - 101.0 |
| ETHANE | Abe et al, 1978 ^257^ | 5 | 5 | 298.1 - 468.1 | 101.0 - 101.0 |
| ETHANE | Diller Saber, 1981 ^614^ | 2 | 2 | 290.0 - 320.0 | 101.0 - 101.0 |
| ETHANE | Iwasaki Takahashi, 1981 ^621^ | 8 | 8 | 298.1 - 348.1 | 101.0 - 101.0 |
| ETHANE | Wilhelm et al, 2006 ^622^ | 672 | 672 | 290.0 - 430.0 | 71.0 - 30906.0 |
| ETHANE | Smith Brown, 1943 ^623^ | 155 | 155 | 288.1 - 473.2 | 689.0 - 34474.0 |
| ETHANE | Golubev Agaev, 1963 ^261^ | 2 | 0 | 10000.0 - 0.0 | 1000000000.0 - 0.0 |
| ETHANE | Eakin et al, 1962 ^624^ | 78 | 78 | 294.3 - 444.3 | 1379.0 - 55158.0 |
| ETHANE | Carmichael Sage, 1963 ^260^ | 348 | 348 | 299.8 - 477.6 | 97.0 - 36031.0 |
| ETHANE | Seibt et al, 2011 ^625^ | 322 | 322 | 293.2 - 423.2 | 64.0 - 30183.0 |
| ETHANE | Hendl Vogel, 1992 ^626^ | 73 | 73 | 288.7 - 630.1 | 26.0 - 282.0 |

| Material | Author | | | *N*_all_ | *N*_used_ | | | *T* / K | *p* / kPa | |
| --- | --- | --- | --- | --- | --- | --- | --- | --- | --- | --- |
| ETHANOL | | Thorpe Rodger, 1894 ^28^ | 12 | | | 12 | 280.3 - 346.7 | | | 2.0 - 76.0 |
| ETHANOL | | Mizushima, 1926 ^627^ | 9 | | | 2 | 283.1 - 293.1 | | | 2.0 - 5.0 |
| ETHANOL | | Hatern, 1949 ^628^ | 1 | | | 1 | 293.1 - 293.1 | | | 5.0 - 5.0 |
| ETHANOL | | Khalimonova, 1962 ^629^ | 10 | | | 10 | 313.1 - 493.2 | | | 16.0 - 4233.0 |
| ETHANOL | | Brunson Byers, 1989 ^630^ | 49 | | | 49 | 293.7 - 503.2 | | | 5.0 - 5083.0 |
| ETHANOL | | Ali et al, 1996 ^631^ | 5 | | | 5 | 298.2 - 318.2 | | | 7.0 - 20.0 |
| ETHANOL | | Dunstan, 1904 ^31^ | 4 | | | 4 | 298.1 - 298.1 | | | 101.0 - 101.0 |
| ETHANOL | | Dunstan et al, 1907 ^119^ | 2 | | | 2 | 298.1 - 298.1 | | | 101.0 - 101.0 |
| ETHANOL | | Dunstan Stubbs, 1908 ^120^ | 1 | | | 1 | 298.1 - 298.1 | | | 101.0 - 101.0 |
| ETHANOL | | Thole, 1910 ^632^ | 1 | | | 1 | 298.1 - 298.1 | | | 101.0 - 101.0 |
| ETHANOL | | Bingham et al, 1913 ^633^ | 22 | | | 22 | 298.1 - 348.1 | | | 101.0 - 101.0 |
| ETHANOL | | Muchin, 1913 ^123^ | 3 | | | 3 | 291.5 - 293.2 | | | 101.0 - 101.0 |
| ETHANOL | | Weissenberger Piatti, 1924 ^34^ | 1 | | | 1 | 291.1 - 291.1 | | | 101.0 - 101.0 |
| ETHANOL | | Dolian Briscoe, 1937 ^634^ | 2 | | | 2 | 298.1 - 298.2 | | | 101.0 - 101.0 |
| ETHANOL | | Khalilov, 1939 ^112^ | 21 | | | 7 | 293.1 - 353.1 | | | 101.0 - 101.0 |
| ETHANOL | | Hatem, 1949 ^628^ | 1 | | | 1 | 293.1 - 293.1 | | | 101.0 - 101.0 |
| ETHANOL | | Teitel Baum et al, 1950 ^140^ | 1 | | | 1 | 293.1 - 293.1 | | | 101.0 - 101.0 |
| ETHANOL | | Vierk, 1950 ^635^ | 1 | | | 1 | 293.1 - 293.1 | | | 101.0 - 101.0 |
| ETHANOL | | Hammond Stokes, 1955 ^113^ | 1 | | | 1 | 298.1 - 298.1 | | | 101.0 - 101.0 |
| ETHANOL | | Ling Van Winkle, 1958 ^43^ | 3 | | | 3 | 303.1 - 348.1 | | | 101.0 - 101.0 |
| ETHANOL | | Misra Varshni, 1961 ^636^ | 8 | | | 8 | 273.2 - 343.1 | | | 101.0 - 101.0 |
| ETHANOL | | Bamelis et al. 1965 ^637^ | 4 | | | 4 | 298.1 - 328.1 | | | 101.0 - 101.0 |
| ETHANOL | | Komarenko et al, 1967 ^638^ | 16 | | | 16 | 159.2 - 298.1 | | | 101.0 - 101.0 |
| ETHANOL | | Tommila et al, 1969 ^639^ | 5 | | | 5 | 293.1 - 323.1 | | | 101.0 - 101.0 |
| ETHANOL | | Konobeev Lyapin, 1970 ^45^ | 5 | | | 5 | 288.2 - 333.1 | | | 101.0 - 101.0 |
| ETHANOL | | Phillips Murphy, 1970 ^640^ | 16 | | | 16 | 273.2 - 348.1 | | | 101.0 - 101.0 |
| ETHANOL | | Ratkovics et al, 1974 ^250^ | 1 | | | 1 | 293.1 - 293.1 | | | 101.0 - 101.0 |
| ETHANOL | | Shikhaliev et al, 1974 ^641^ | 14 | | | 14 | 223.0 - 353.0 | | | 101.0 - 101.0 |
| ETHANOL | | Lee et al, 1976 ^642^ | 6 | | | 6 | 283.1 - 333.1 | | | 101.0 - 101.0 |
| ETHANOL | | Shikhaliev, 1977 ^551^ | 1 | | | 1 | 293.0 - 293.0 | | | 101.0 - 101.0 |
| ETHANOL | | Tanaka et al, 1977 ^643^ | 16 | | | 16 | 298.1 - 323.1 | | | 100.0 - 78500.0 |
| ETHANOL | | Baykut Tanrikulu, 1980 ^644^ | 2 | | | 2 | 293.1 - 293.1 | | | 101.0 - 101.0 |
| ETHANOL | | Pikkarainen, 1980 ^645^ | 2 | | | 2 | 303.1 - 333.1 | | | 101.0 - 101.0 |
| ETHANOL | | Kumar et al, 1981 ^646^ | 1 | | | 1 | 298.1 - 298.1 | | | 101.0 - 101.0 |
| ETHANOL | | Pikkarainen, 1983 ^647^ | 1 | | | 1 | 333.1 - 333.1 | | | 101.0 - 101.0 |
| ETHANOL | | Pikkarainen, 1983 ^648^ | 1 | | | 1 | 303.1 - 303.1 | | | 101.0 - 101.0 |
| ETHANOL | | Rauf et al, 1983 ^649^ | 5 | | | 5 | 288.2 - 328.1 | | | 101.0 - 101.0 |
| ETHANOL | | Papanastasiou et al, 1987 ^650^ | 5 | | | 5 | 288.2 - 308.1 | | | 101.0 - 101.0 |
| ETHANOL | | Tanaka et al, 1987 ^651^ | 16 | | | 16 | 298.1 - 323.1 | | | 100.0 - 78600.0 |
| ETHANOL | | Kouris Panayioton, 1989 ^165^ | 1 | | | 1 | 298.1 - 298.1 | | | 101.0 - 101.0 |
| ETHANOL | | Sivaramprasad et al, 1990 ^170^ | 4 | | | 4 | 303.0 - 333.2 | | | 101.0 - 101.0 |
| ETHANOL | | Crabtree O’Brien, 1991 ^652^ | 3 | | | 3 | 303.0 - 319.0 | | | 101.0 - 101.0 |
| ETHANOL | | Das Jha, 1991 ^55^ | 1 | | | 0 | 10000.0 - 0.0 | | | 1000000000.0 - 0.0 |
| ETHANOL | | Garcia et al, 1991 ^653^ | 5 | | | 5 | 298.2 - 333.2 | | | 101.0 - 101.0 |
| ETHANOL | | Papaionnou et al, 1991 ^173^ | 1 | | | 1 | 298.2 - 298.2 | | | 101.0 - 101.0 |
| ETHANOL | | Papaionnou Ziogas, 1991 ^410^ | 5 | | | 5 | 288.2 - 308.2 | | | 101.0 - 101.0 |
| ETHANOL | | Ramakanth et al, 1991 ^174^ | 1 | | | 1 | 293.2 - 293.2 | | | 101.0 - 101.0 |
| ETHANOL | | Ramakanth et al, 1991 ^174^ | 1 | | | 1 | 298.2 - 298.2 | | | 101.0 - 101.0 |
| ETHANOL | | Pikkarainen, 1992 ^654^ | 1 | | | 1 | 303.2 - 303.2 | | | 101.0 - 101.0 |
| ETHANOL | | Aminabhvi et al, 1993 ^655^ | 3 | | | 3 | 298.2 - 308.2 | | | 101.0 - 101.0 |
| ETHANOL | | Papaionnou et al, 1993 ^503^ | 11 | | | 11 | 298.2 - 298.2 | | | 100.0 - 71750.0 |
| ETHANOL | | Shastri et al, 1993 ^179^ | 1 | | | 1 | 293.2 - 293.2 | | | 101.0 - 101.0 |
| ETHANOL | | Assail et al, 1994 ^656^ | 27 | | | 27 | 298.2 - 328.2 | | | 100.0 - 27560.0 |
| ETHANOL | | Papaionnou Panayton, 1994 ^657^ | 18 | | | 18 | 298.2 - 298.2 | | | 101.0 - 51850.0 |
| ETHANOL | | Rodriguez et al, 1994 ^658^ | 8 | | | 8 | 278.0 - 313.0 | | | 101.0 - 101.0 |
| ETHANOL | | Elb-Banna Ramanda, 1995 ^659^ | 3 | | | 3 | 298.2 - 318.2 | | | 101.0 - 101.0 |
| ETHANOL | | Lee Lin, 1995 ^660^ | 3 | | | 3 | 303.2 - 323.2 | | | 101.0 - 101.0 |
| ETHANOL | | Nikam et al, 1995 ^661^ | 2 | | | 2 | 298.2 - 303.2 | | | 101.0 - 101.0 |
| ETHANOL | | Papaionnou Panayton, 1995 ^662^ | 12 | | | 12 | 298.2 - 298.2 | | | 100.0 - 51800.0 |
| ETHANOL | | Rived et al, 1995 ^183^ | 1 | | | 1 | 303.2 - 303.2 | | | 101.0 - 101.0 |
| ETHANOL | | Aucejo et al, 1996 ^663^ | 1 | | | 1 | 298.2 - 298.2 | | | 101.0 - 101.0 |
| ETHANOL | | Aalto Liukkonen, 1996 ^664^ | 1 | | | 1 | 303.2 - 303.2 | | | 101.0 - 101.0 |
| ETHANOL | | Nikam et al, 1996 ^665^ | 2 | | | 2 | 298.2 - 303.2 | | | 101.0 - 101.0 |
| ETHANOL | | Nikam et al, 1996 ^666^ | 1 | | | 1 | 298.2 - 298.2 | | | 101.0 - 101.0 |
| ETHANOL | | elb, 1997 | 1 | | | 1 | 298.2 - 298.2 | | | 101.0 - 101.0 |
| ETHANOL | | Orge et al, 1997 ^667^ | 1 | | | 1 | 298.2 - 298.2 | | | 101.0 - 101.0 |
| ETHANOL | | Aminabhvi Banerjee, 1998 ^419^ | 3 | | | 3 | 298.2 - 308.2 | | | 101.0 - 101.0 |
| ETHANOL | | Aminabhvi Patil, 1998 ^668^ | 3 | | | 3 | 298.2 - 308.2 | | | 101.0 - 101.0 |
| ETHANOL | | Canosa et al, 1998 ^669^ | 1 | | | 1 | 298.2 - 298.2 | | | 101.0 - 101.0 |
| ETHANOL | | Canosa et al, 1998 ^669^ | 1 | | | 1 | 298.2 - 298.2 | | | 101.0 - 101.0 |
| ETHANOL | | Kumagai Yokoyama, 1998 ^670^ | 4 | | | 4 | 273.2 - 333.2 | | | 101.0 - 101.0 |
| ETHANOL | | Nikam et al, 1998 ^671^ | 4 | | | 4 | 298.2 - 313.2 | | | 101.0 - 101.0 |
| ETHANOL | | Oswal Desai, 1998 ^672^ | 2 | | | 2 | 303.2 - 313.2 | | | 101.0 - 101.0 |
| ETHANOL | | Ali Hyd, 1999 ^55^ | 1 | | | 1 | 303.2 - 303.2 | | | 101.0 - 101.0 |
| ETHANOL | | Aminabhvi et al, 1999 ^673^ | 3 | | | 3 | 298.2 - 308.2 | | | 101.0 - 101.0 |
| ETHANOL | | Aralaguppi et al, 1999 ^189^ | 3 | | | 3 | 298.2 - 308.2 | | | 101.0 - 101.0 |
| ETHANOL | | Harashta et al, 1999 ^674^ | 1 | | | 1 | 298.2 - 298.2 | | | 101.0 - 101.0 |
| ETHANOL | | Oswal Desai, 1999 ^675^ | 2 | | | 2 | 303.2 - 313.2 | | | 101.0 - 101.0 |
| ETHANOL | | Prasad et al, 1999 ^676^ | 5 | | | 5 | 293.2 - 323.2 | | | 101.0 - 101.0 |
| ETHANOL | | Henni et al, 2000 ^677^ | 1 | | | 1 | 313.0 - 313.0 | | | 101.0 - 101.0 |
| ETHANOL | | Nikam et al, 2000 ^678^ | 2 | | | 2 | 303.2 - 308.2 | | | 101.0 - 101.0 |
| ETHANOL | | Nikam et al, 2000 ^678^ | 3 | | | 3 | 303.2 - 313.2 | | | 101.0 - 101.0 |
| ETHANOL | | Pan et al, 2000 ^679^ | 1 | | | 1 | 303.2 - 303.2 | | | 101.0 - 101.0 |
| ETHANOL | | Tu et al, 2000 ^680^ | 4 | | | 4 | 293.2 - 313.2 | | | 101.0 - 101.0 |
| ETHANOL | | Ku et al, 2001 ^681^ | 3 | | | 3 | 298.2 - 318.2 | | | 101.0 - 101.0 |
| ETHANOL | | Oswal Desai, 2001 ^682^ | 2 | | | 2 | 303.2 - 313.2 | | | 101.0 - 101.0 |
| ETHANOL | | Tu et al, 2001 ^683^ | 4 | | | 4 | 293.2 - 313.2 | | | 101.0 - 101.0 |
| ETHANOL | | Peng Tu, 2002 ^61^ | 1 | | | 1 | 298.2 - 298.2 | | | 101.0 - 101.0 |
| ETHANOL | | Prasad et al, 2002 ^684^ | 3 | | | 3 | 293.2 - 313.2 | | | 101.0 - 101.0 |
| ETHANOL | | Agarwal Singh, 2004 ^63^ | 1 | | | 1 | 298.2 - 298.2 | | | 101.0 - 101.0 |
| ETHANOL | | Belda et al, 2004 ^685^ | 7 | | | 7 | 283.2 - 343.2 | | | 101.0 - 101.0 |
| ETHANOL | | Chen et al, 2004 ^686^ | 3 | | | 3 | 288.2 - 308.2 | | | 101.0 - 101.0 |
| ETHANOL | | Gonzalez et al, 2004 ^298^ | 3 | | | 3 | 293.2 - 303.2 | | | 101.0 - 101.0 |
| ETHANOL | | Rattan et al, 2004 ^557^ | 2 | | | 2 | 298.2 - 308.2 | | | 101.0 - 101.0 |
| ETHANOL | | Rodriguez et al, 2004 ^533^ | 4 | | | 4 | 293.2 - 313.2 | | | 101.0 - 101.0 |
| ETHANOL | | Rodriguez et al, 2004 ^687^ | 4 | | | 4 | 293.2 - 313.2 | | | 101.0 - 101.0 |
| ETHANOL | | Saleh et al, 2004 ^688^ | 5 | | | 5 | 303.2 - 323.2 | | | 101.0 - 101.0 |
| ETHANOL | | Calvar et al, 2005 ^689^ | 1 | | | 1 | 298.2 - 298.2 | | | 101.0 - 101.0 |
| ETHANOL | | Chen et al, 2005 ^65^ | 3 | | | 3 | 293.2 - 303.2 | | | 101.0 - 101.0 |
| ETHANOL | | Chen et al, 2005 ^65^ | 3 | | | 3 | 288.2 - 308.2 | | | 101.0 - 101.0 |
| ETHANOL | | Djojoputro Ismadji, 2005 ^690^ | 3 | | | 3 | 293.2 - 313.2 | | | 101.0 - 101.0 |
| ETHANOL | | Djojoputro Ismadji, 2005 ^691^ | 3 | | | 3 | 293.2 - 313.2 | | | 101.0 - 101.0 |
| ETHANOL | | Djojoputro Ismadji, 2005 ^690^ | 3 | | | 3 | 298.2 - 318.2 | | | 101.0 - 101.0 |
| ETHANOL | | Gonzalez et al, 2005 ^66^ | 1 | | | 1 | 298.2 - 298.2 | | | 100.0 - 100.0 |
| ETHANOL | | Ku Tu, 2005 ^692^ | 3 | | | 3 | 298.2 - 318.2 | | | 101.0 - 101.0 |
| ETHANOL | | Oswal et al, 2005 ^334^ | 1 | | | 1 | 303.2 - 303.2 | | | 101.0 - 101.0 |
| ETHANOL | | Wang et al, 2005 ^693^ | 3 | | | 3 | 298.2 - 318.2 | | | 101.0 - 101.0 |
| ETHANOL | | Wankheda et al, 2005 ^694^ | 3 | | | 3 | 298.2 - 308.2 | | | 101.0 - 101.0 |
| ETHANOL | | Ye Tu, 2005 ^335^ | 3 | | | 3 | 288.2 - 308.2 | | | 101.0 - 101.0 |
| ETHANOL | | Zeberg-Mikkelsen et al, 2005 ^695^ | 23 | | | 23 | 293.2 - 353.2 | | | 100.0 - 100000.0 |
| ETHANOL | | Alvarez et al, 2006 ^696^ | 6 | | | 6 | 298.2 - 323.2 | | | 101.0 - 101.0 |
| ETHANOL | | Aralaguppi Baragi, 2006 ^336^ | 3 | | | 3 | 298.2 - 308.2 | | | 101.0 - 101.0 |
| ETHANOL | | Arce et al, 2006 ^697^ | 1 | | | 1 | 298.2 - 298.2 | | | 101.0 - 101.0 |
| ETHANOL | | Arce et al, 2006 ^697^ | 1 | | | 1 | 298.2 - 298.2 | | | 101.0 - 101.0 |
| ETHANOL | | Arce et al, 2006 ^697^ | 1 | | | 1 | 298.2 - 298.2 | | | 101.0 - 101.0 |
| ETHANOL | | Calvar et al, 2006 ^698^ | 1 | | | 1 | 298.2 - 298.2 | | | 101.0 - 101.0 |
| ETHANOL | | Chen Tu, 2006 ^699^ | 6 | | | 6 | 288.2 - 308.2 | | | 100.0 - 101.0 |
| ETHANOL | | Gomez et al, 2006 ^700^ | 1 | | | 1 | 298.2 - 298.2 | | | 101.0 - 101.0 |
| ETHANOL | | Gomez et al, 2006 ^700^ | 3 | | | 3 | 298.2 - 328.2 | | | 101.0 - 101.0 |
| ETHANOL | | Gonzalez et al, 2006 ^68^ | 3 | | | 3 | 298.2 - 328.2 | | | 101.0 - 101.0 |
| ETHANOL | | Gonzalez et al, 2006 ^68^ | 3 | | | 3 | 293.2 - 303.2 | | | 101.0 - 101.0 |
| ETHANOL | | Iloukhani et al, 2006 ^339^ | 1 | | | 1 | 298.2 - 298.2 | | | 101.0 - 101.0 |
| ETHANOL | | Kadam et al, 2006 ^701^ | 2 | | | 2 | 298.2 - 308.2 | | | 101.0 - 101.0 |
| ETHANOL | | Mutalik et al, 2006 ^702^ | 3 | | | 3 | 298.2 - 308.2 | | | 203.0 - 203.0 |
| ETHANOL | | Mutalik et al, 2006 ^702^ | 3 | | | 3 | 298.2 - 308.2 | | | 101.0 - 101.0 |
| ETHANOL | | Rodriguez et al, 2006 ^536^ | 1 | | | 1 | 298.2 - 298.2 | | | 101.0 - 101.0 |
| ETHANOL | | Roy Singh, 2006 ^703^ | 1 | | | 1 | 303.2 - 303.2 | | | 101.0 - 101.0 |
| ETHANOL | | Sheu Tu, 2006 ^704^ | 4 | | | 4 | 288.2 - 318.2 | | | 100.0 - 100.0 |
| ETHANOL | | Sheu Tu, 2006 ^704^ | 4 | | | 4 | 288.2 - 318.2 | | | 100.0 - 100.0 |
| ETHANOL | | Zeberg Mikkelsen et al, 2006 ^705^ | 23 | | | 23 | 293.2 - 353.2 | | | 100.0 - 100000.0 |
| ETHANOL | | Arce et al, 2007 ^706^ | 1 | | | 1 | 298.2 - 298.2 | | | 101.0 - 101.0 |
| ETHANOL | | Bhuiyan et al, 2007 ^377^ | 5 | | | 5 | 303.2 - 323.2 | | | 101.0 - 101.0 |
| ETHANOL | | Calvar et al, 2007 ^707^ | 1 | | | 1 | 298.2 - 298.2 | | | 101.0 - 101.0 |
| ETHANOL | | Calvar et al, 2007 ^708^ | 1 | | | 1 | 298.2 - 298.2 | | | 101.0 - 101.0 |
| ETHANOL | | Gonzalez et al, 2007 ^430^ | 3 | | | 3 | 293.2 - 303.2 | | | 101.0 - 101.0 |
| ETHANOL | | Gonzalez et al, 2007 ^430^ | 3 | | | 3 | 293.2 - 303.2 | | | 101.0 - 101.0 |
| ETHANOL | | Gurung Roy, 2007 ^590^ | 3 | | | 3 | 298.2 - 318.2 | | | 101.0 - 101.0 |
| ETHANOL | | Sun Ma, 2007 ^709^ | 2 | | | 2 | 293.2 - 298.2 | | | 101.0 - 101.0 |
| ETHANOL | | Ali et al, 2008 ^378^ | 2 | | | 2 | 298.2 - 303.2 | | | 101.0 - 101.0 |
| ETHANOL | | Arce et al, 2008 ^710^ | 1 | | | 1 | 298.2 - 298.2 | | | 101.0 - 101.0 |
| ETHANOL | | Awwad et al, 2008 ^711^ | 4 | | | 4 | 293.2 - 323.2 | | | 101.0 - 101.0 |
| ETHANOL | | Calvar et al, 2008 ^712^ | 1 | | | 1 | 298.2 - 298.2 | | | 101.0 - 101.0 |
| ETHANOL | | Gomez et al, 2008 ^713^ | 3 | | | 3 | 298.2 - 328.2 | | | 100.0 - 100.0 |
| ETHANOL | | Gonzalez et al, 2008 ^713^ | 3 | | | 3 | 298.2 - 328.2 | | | 101.0 - 101.0 |
| ETHANOL | | Gonzalez et al, 2008 ^714^ | 3 | | | 3 | 298.2 - 328.2 | | | 100.0 - 100.0 |
| ETHANOL | | Nain, 2008 ^595^ | 6 | | | 6 | 293.2 - 318.2 | | | 101.0 - 101.0 |
| ETHANOL | | Rehman Ansari, 2008 ^715^ | 12 | | | 12 | 268.1 - 323.2 | | | 101.0 - 101.0 |
| ETHANOL | | Sih et al, 2008 ^70^ | 4 | | | 4 | 298.2 - 313.2 | | | 100.0 - 100.0 |
| ETHANOL | | Yang et al, 2008 ^716^ | 6 | | | 6 | 293.2 - 333.2 | | | 101.0 - 101.0 |
| ETHANOL | | Andreatta et al, 2009 ^717^ | 1 | | | 1 | 298.2 - 298.2 | | | 101.0 - 101.0 |
| ETHANOL | | Domanska Laskowska, 2009 ^718^ | 6 | | | 6 | 298.2 - 348.2 | | | 101.0 - 101.0 |
| ETHANOL | | Feitosa et al, 2009 ^514^ | 2 | | | 2 | 293.2 - 298.2 | | | 101.0 - 101.0 |
| ETHANOL | | Gonzalez et al, 2009 ^719^ | 3 | | | 3 | 298.2 - 328.2 | | | 101.0 - 101.0 |
| ETHANOL | | Mokhtarani, 2009 ^720^ | 11 | | | 11 | 283.2 - 333.2 | | | 101.0 - 101.0 |
| ETHANOL | | Roy et al, 2009 ^721^ | 3 | | | 3 | 298.2 - 318.2 | | | 101.0 - 101.0 |
| ETHANOL | | Tsierkezos Molinou, 2009 ^722^ | 1 | | | 1 | 293.2 - 293.2 | | | 101.0 - 101.0 |
| ETHANOL | | Wang et al, 2009 ^723^ | 6 | | | 6 | 303.2 - 328.2 | | | 101.0 - 101.0 |
| ETHANOL | | Yan et al, 2009 ^232^ | 4 | | | 4 | 298.2 - 328.2 | | | 101.0 - 101.0 |
| ETHANOL | | Zarei et al, 2009 ^724^ | 1 | | | 1 | 303.2 - 303.2 | | | 82.0 - 82.0 |
| ETHANOL | | Andreatta et al, 2010 ^725^ | 1 | | | 1 | 298.2 - 298.2 | | | 101.0 - 101.0 |
| ETHANOL | | Anouti et al, 2010 ^726^ | 7 | | | 7 | 293.2 - 323.2 | | | 101.0 - 101.0 |
| ETHANOL | | Bhattachayjee Roy, 2010 ^727^ | 1 | | | 1 | 298.2 - 298.2 | | | 101.0 - 101.0 |
| ETHANOL | | Calvar et al, 2010 ^728^ | 1 | | | 1 | 298.2 - 298.2 | | | 101.0 - 101.0 |
| ETHANOL | | Goncalves et al, 2010 ^729^ | 23 | | | 23 | 273.2 - 346.2 | | | 101.0 - 101.0 |
| ETHANOL | | Hou et al, 2010 ^730^ | 8 | | | 8 | 278.2 - 313.2 | | | 101.0 - 101.0 |
| ETHANOL | | Li et al, 2010 ^600^ | 5 | | | 5 | 298.2 - 318.2 | | | 101.0 - 101.0 |
| ETHANOL | | Rilo et al, 2010 ^731^ | 4 | | | 4 | 288.2 - 318.2 | | | 101.0 - 101.0 |
| ETHANOL | | Yang et al, 2010 ^74^ | 8 | | | 8 | 278.2 - 313.2 | | | 101.0 - 101.0 |
| ETHANOL | | Garcia Abuin et al, 2011 ^732^ | 4 | | | 4 | 293.2 - 323.2 | | | 101.0 - 101.0 |
| ETHANOL | | Hasan et al, 2011 ^733^ | 1 | | | 1 | 298.2 - 298.2 | | | 101.0 - 101.0 |
| ETHANOL | | Kurnia Mutalib, 2011 ^734^ | 4 | | | 4 | 293.2 - 323.2 | | | 101.0 - 101.0 |
| ETHANOL | | Kurnia Mutalib, 2011 ^734^ | 5 | | | 5 | 293.2 - 323.2 | | | 101.0 - 101.0 |
| ETHANOL | | Liu et al, 2011 ^735^ | 5 | | | 5 | 293.2 - 333.2 | | | 101.0 - 101.0 |
| ETHANOL | | Trenzado et al, 2011 ^736^ | 5 | | | 5 | 288.2 - 313.2 | | | 101.0 - 101.0 |
| ETHANOL | | Blanco et al, 2012 ^737^ | 1 | | | 1 | 298.2 - 298.2 | | | 101.0 - 101.0 |
| ETHANOL | | Chen et al, 2012 ^738^ | 4 | | | 4 | 293.2 - 323.2 | | | 101.0 - 101.0 |
| ETHANOL | | Garcia Mardones et al, 2012 ^739^ | 4 | | | 4 | 293.2 - 323.2 | | | 101.0 - 101.0 |
| ETHANOL | | Gong et al, 2012 ^740^ | 9 | | | 9 | 293.2 - 333.2 | | | 101.0 - 101.0 |
| ETHANOL | | Han et al, 2012 ^741^ | 5 | | | 5 | 293.2 - 333.2 | | | 101.0 - 101.0 |
| ETHANOL | | Quijada et al, 2012 ^742^ | 1 | | | 1 | 298.2 - 298.2 | | | 101.0 - 101.0 |
| ETHANOL | | Shao et al, 2012 ^743^ | 4 | | | 4 | 298.2 - 313.2 | | | 101.0 - 101.0 |
| ETHANOL | | Sreekanth et al, 2012 ^744^ | 3 | | | 3 | 308.2 - 318.2 | | | 101.0 - 101.0 |
| ETHANOL | | Xu et al, 2012 ^745^ | 5 | | | 5 | 293.2 - 313.2 | | | 101.0 - 101.0 |
| ETHANOL | | Yao et al, 2012 ^746^ | 7 | | | 7 | 293.2 - 323.2 | | | 101.0 - 101.0 |
| ETHANOL | | Caro et al, 2013 ^747^ | 5 | | | 5 | 283.2 - 313.2 | | | 101.0 - 101.0 |
| ETHANOL | | Faria et al, 2013 ^748^ | 6 | | | 6 | 283.2 - 313.2 | | | 100.0 - 100.0 |
| ETHANOL | | Kondaiah et al, 2013 ^749^ | 3 | | | 3 | 303.2 - 323.2 | | | 101.0 - 101.0 |
| ETHANOL | | Quijada Maldonado, 2013 ^607^ | 7 | | | 7 | 298.2 - 328.2 | | | 101.0 - 101.0 |
| ETHANOL | | Xu et al, 2013 ^750^ | 7 | | | 7 | 293.2 - 313.2 | | | 101.0 - 101.0 |
| ETHANOL | | Yang et al, 2013 ^751^ | 4 | | | 4 | 293.2 - 308.2 | | | 101.0 - 101.0 |
| ETHANOL | | Castro et al, 2014 ^752^ | 7 | | | 7 | 278.2 - 338.2 | | | 101.0 - 101.0 |
| ETHANOL | | Florido et al, 2004 ^753^ | 1 | | | 1 | 298.2 - 298.2 | | | 102.0 - 102.0 |
| ETHANOL | | Goncalves et al, 2014 ^754^ | 1 | | | 1 | 298.2 - 298.2 | | | 100.0 - 100.0 |
| ETHANOL | | Moosavi et al, 2014 ^755^ | 3 | | | 3 | 298.2 - 308.2 | | | 101.0 - 101.0 |
| ETHANOL | | Zhu et al, 2014 ^756^ | 7 | | | 7 | 293.2 - 323.2 | | | 101.0 - 101.0 |
| ETHANOL | | Zhu et al, 2014 ^756^ | 5 | | | 5 | 293.2 - 333.2 | | | 101.0 - 101.0 |
| ETHANOL | | Cao et al, 2015 ^757^ | 1 | | | 1 | 298.2 - 298.2 | | | 101.0 - 101.0 |
| ETHANOL | | Salinas et al, 2015 ^758^ | 4 | | | 4 | 278.2 - 338.2 | | | 101.0 - 101.0 |
| ETHANOL | | Titani, 1933 ^75^ | 7 | | | 7 | 403.3 - 581.9 | | | 101.0 - 101.0 |
| ETHANOL | | Khalilov, 1939 ^112^ | 17 | | | 16 | 363.1 - 513.2 | | | 101.0 - 101.0 |
| ETHANOL | | Ling Van Winkle, 1958 ^43^ | 3 | | | 3 | 383.1 - 423.1 | | | 101.0 - 101.0 |
| ETHANOL | | Reid Belenyessy, 1960 ^759^ | 1 | | | 1 | 423.1 - 423.1 | | | 101.0 - 101.0 |

| Material | Author | | *N*_all_ | | | *N*_used_ | | *T* / K | *p* / kPa | |
| --- | --- | --- | --- | --- | --- | --- | --- | --- | --- | --- |
| ETHYLENE | | Gerf Galkov, 1940 ^615^ | | 9 | 9 | | 105.0 - 168.2 | | | 0.0 - 92.0 |
| ETHYLENE | | Thausen, 1911 ^760^ | | 1 | 1 | | 285.5 - 285.5 | | | 101.0 - 101.0 |
| ETHYLENE | | Tratz Stauf, 1929 ^761^ | | 13 | 13 | | 193.9 - 525.5 | | | 101.0 - 101.0 |
| ETHYLENE | | Titani, 1930 ^9^ | | 7 | 7 | | 273.2 - 393.1 | | | 101.0 - 101.0 |
| ETHYLENE | | Trautz et al, 1930 ^762^ | | 3 | 3 | | 292.7 - 374.1 | | | 101.0 - 101.0 |
| ETHYLENE | | Trautz Heberling, 1931 ^86^ | | 10 | 10 | | 291.7 - 523.9 | | | 101.0 - 101.0 |
| ETHYLENE | | Van Cleave Maass, 1935 ^87^ | | 11 | 11 | | 192.3 - 296.5 | | | 0.0 - 0.0 |
| ETHYLENE | | Comings et al, 1944 ^763^ | | 4 | 4 | | 303.1 - 368.1 | | | 101.0 - 101.0 |
| ETHYLENE | | Craven Lambert, 1951 ^76^ | | 5 | 5 | | 193.2 - 523.2 | | | 101.0 - 101.0 |
| ETHYLENE | | Golubev Petrov, 1953 ^764^ | | 1 | 1 | | 313.1 - 313.1 | | | 101.0 - 101.0 |
| ETHYLENE | | Lambert et al, 1955 ^12^ | | 8 | 8 | | 308.1 - 374.3 | | | 101.0 - 101.0 |
| ETHYLENE | | Misic Thodos, 1961 ^765^ | | 5 | 5 | | 293.1 - 523.2 | | | 101.0 - 101.0 |
| ETHYLENE | | Golubev Agaev, 1964 ^253^ | | 4 | 4 | | 297.1 - 423.1 | | | 101.0 - 101.0 |
| ETHYLENE | | Neduzhii Khmara, 1968 ^2^ | | 6 | 6 | | 193.2 - 297.1 | | | 101.0 - 101.0 |
| ETHYLENE | | Kestin et al, 1971 ^254^ | | 2 | 2 | | 296.2 - 303.7 | | | 101.0 - 101.0 |
| ETHYLENE | | Vaschenko et al, 1971 ^766^ | | 17 | 17 | | 196.2 - 575.7 | | | 101.0 - 101.0 |
| ETHYLENE | | Timrot et al, 1975 ^100^ | | 7 | 7 | | 296.9 - 577.4 | | | 101.0 - 101.0 |
| ETHYLENE | | Kestin et al, 1977 ^256^ | | 5 | 5 | | 300.6 - 477.2 | | | 101.0 - 101.0 |

| Material | Author | | *N*_all_ | | *N*_used_ | | | *T* / K | *p* / kPa | |
| --- | --- | --- | --- | --- | --- | --- | --- | --- | --- | --- |
| ETHYLENEOXIDE | | Scholz Kley, 1981 ^7^ | | 14 | | 0 | 10000.0 - 0.0 | | | 1000000000.0 - 0.0 |
| ETHYLENEOXIDE | | Liessmann et al, 1995 ^5^ | | 11 | | 6 | 298.2 - 373.2 | | | 47.0 - 511.0 |
| ETHYLENEOXIDE | | Timmermans Hennaut, 1937 ^444^ | | 3 | | 0 | 10000.0 - 0.0 | | | 1000000000.0 - 0.0 |
| ETHYLENEOXIDE | | Maass Boomer, 1922 ^767^ | | 12 | | 0 | 10000.0 - 0.0 | | | 1000000000.0 - 0.0 |
| ETHYLENEOXIDE | | Senftleben, 1953 ^768^ | | 1 | | 0 | 10000.0 - 0.0 | | | 1000000000.0 - 0.0 |
| ETHYLENEOXIDE | | Maass Boomer, 1922 ^767^ | | 9 | | 0 | 10000.0 - 0.0 | | | 1000000000.0 - 0.0 |
| ETHYLENEOXIDE | | Zhou et al, 2000 ^769^ | | 13 | | 0 | 10000.0 - 0.0 | | | 1000000000.0 - 0.0 |
| ETHYLENEOXIDE | | Timmermans Hennaut, 1937 ^444^ | | 3 | | 3 | 273.2 - 283.1 | | | 101.0 - 101.0 |

| Material | Author | | *N*_all_ | | *N*_used_ | | | *T* / K | *p* / kPa | |
| --- | --- | --- | --- | --- | --- | --- | --- | --- | --- | --- |
| FLUORINE | | Scholz Kley, 1981 ^7^ | | 6 | | 6 | 69.2 - 83.2 | | | 9.0 - 74.0 |
| FLUORINE | | Stephan Heckenberger, 2000 ^770^ | | 470 | | 470 | 90.0 - 300.0 | | | 100.0 - 20000.0 |
| FLUORINE | | Scholz Kley, 1981 ^7^ | | 25 | | 25 | 86.8 - 471.0 | | | 101.0 - 2082.0 |
| FLUORINE | | Maczynska Maczynski, 1991 ^771^ | | 3 | | 3 | 73.2 - 83.2 | | | 18.0 - 73.0 |
| FLUORINE | | Vargraftik, 1972 ^6^ | | 142 | | 142 | 69.2 - 900.0 | | | 9.0 - 10000.0 |
| FLUORINE | | Liessmann, 1995 ^5^ | | 8 | | 8 | 63.2 - 98.2 | | | 3.0 - 341.0 |
| FLUORINE | | Hersh Gaynor, 1966 ^772^ | | 2 | | 2 | 77.4 - 90.2 | | | 34.0 - 161.0 |
| FLUORINE | | Wayaku, 1974 ^773^ | | 293 | | 292 | 70.0 - 300.0 | | | 10.0 - 22095.0 |
| FLUORINE | | Elverum Doescher, 1952 ^774^ | | 6 | | 6 | 69.2 - 83.2 | | | 9.0 - 74.0 |
| FLUORINE | | Kanda, 1937 ^775^ | | 9 | | 9 | 86.8 - 273.2 | | | 101.0 - 102.0 |
| FLUORINE | | Franck Stoeber, 1952 ^776^ | | 7 | | 7 | 90.0 - 471.0 | | | 101.0 - 158.0 |
| FLUORINE | | Elverum Doescher, 1952 ^774^ | | 6 | | 6 | 69.2 - 83.2 | | | 9.0 - 74.0 |

| Material | Author | | *N*_all_ | | *N*_used_ | | | *T* / K | *p* / kPa | |
| --- | --- | --- | --- | --- | --- | --- | --- | --- | --- | --- |
| H2S | | Rankine Smith, 1921 ^777^ | | 2 | | 2 | 290.2 - 373.2 | | | 101.0 - 101.0 |
| H2S | | Bhattacharyya, 1970 ^778^ | | 4 | | 4 | 238.2 - 308.2 | | | 314.0 - 2569.0 |
| H2S | | Pal Bhattacharyya, 1969 ^779^ | | 3 | | 3 | 298.0 - 373.0 | | | 101.0 - 4204.0 |
| H2S | | Bhattacharyya Ghosi, 1970 ^780^ | | 5 | | 5 | 220.9 - 308.0 | | | 151.0 - 2560.0 |
| H2S | | Pal Barua, 1967 ^781^ | | 5 | | 5 | 306.7 - 483.2 | | | 101.0 - 4041.0 |
| H2S | | Monteil et al, 1969 ^782^ | | 33 | | 0 | 10000.0 - 0.0 | | | 1000000000.0 - 0.0 |
| H2S | | Hennel Krynicki, 1959 ^783^ | | 8 | | 8 | 261.7 - 323.2 | | | 725.0 - 3577.0 |
| H2S | | Jung Schmick, 1930 ^784^ | | 1 | | 1 | 290.4 - 290.4 | | | 101.0 - 101.0 |
| H2S | | Steel et al, 1906 ^785^ | | 3 | | 3 | 191.0 - 198.2 | | | 30.0 - 46.0 |
| H2S | | Runovskaya et al, 1970 ^786^ | | 11 | | 11 | 190.1 - 211.8 | | | 28.0 - 97.0 |
| H2S | | Vogel, 2011 ^787^ | | 34 | | 34 | 291.6 - 682.0 | | | 52.0 - 123.0 |
| H2S | | Liessmann et al, 1995 ^5^ | | 7 | | 7 | 193.2 - 328.2 | | | 34.0 - 3968.0 |
| H2S | | Maczynska Maczynski, 1991 ^771^ | | 2 | | 2 | 193.2 - 198.2 | | | 34.0 - 46.0 |
| H2S | | Scholz Kley, 1981 ^7^ | | 9 | | 9 | 191.0 - 483.2 | | | 30.0 - 4041.0 |
| H2S | | Steele et al, 1906 ^785^ | | 6 | | 6 | 191.0 - 209.9 | | | 30.0 - 88.0 |
| H2S | | Vogel, 2011 ^787^ | | 34 | | 34 | 291.6 - 682.0 | | | 52.0 - 123.0 |

| Material | Author | | *N*_all_ | | *N*_used_ | | | *T* / K | *p* / kPa | |
| --- | --- | --- | --- | --- | --- | --- | --- | --- | --- | --- |
| HCL | | Harle, 1922 ^788^ | | 2 | | 2 | 285.7 - 373.5 | | | 101.0 - 3489.0 |
| HCL | | Trautz Narath, 1926 ^789^ | | 7 | | 7 | 293.7 - 524.2 | | | 99.0 - 101.0 |
| HCL | | Krynicki Hennel, 1963 ^790^ | | 13 | | 13 | 240.8 - 318.2 | | | 938.0 - 7252.0 |
| HCL | | Jung Schmick, 1930 ^784^ | | 2 | | 2 | 289.7 - 291.2 | | | 101.0 - 101.0 |
| HCL | | Steele et al, 1906 ^785^ | | 6 | | 6 | 160.8 - 188.2 | | | 13.0 - 87.0 |
| HCL | | Liessmann et al, 1995 ^5^ | | 11 | | 10 | 223.2 - 313.2 | | | 484.0 - 6536.0 |
| HCL | | Krynicki Hennel, 1963 ^790^ | | 13 | | 12 | 240.8 - 313.2 | | | 1058.0 - 6561.0 |
| HCL | | Maczynska Maczynski, 1991 ^771^ | | 6 | | 6 | 163.2 - 188.2 | | | 15.0 - 87.0 |
| HCL | | Scholz Kley, 1981 ^7^ | | 29 | | 29 | 160.8 - 475.2 | | | 13.0 - 7252.0 |
| HCL | | Steele et al, 1906 ^785^ | | 6 | | 6 | 160.8 - 188.2 | | | 13.0 - 87.0 |

| Material | Author | | *N*_all_ | | *N*_used_ | | | *T* / K | *p* / kPa | |
| --- | --- | --- | --- | --- | --- | --- | --- | --- | --- | --- |
| HELIUM | | Zinovieva, 1958 ^791^ | | 14 | | 6 | 3.2 - 4.2 | | | 16.0 - 81.0 |
| HELIUM | | Luker Johnson, 1959 ^792^ | | 30 | | 30 | 328.1 - 682.2 | | | 820.0 - 13114.0 |
| HELIUM | | Kob Kobayashi, 1967 ^793^ | | 53 | | 53 | 183.2 - 323.1 | | | 1013.0 - 50662.0 |
| HELIUM | | Kestin et al, 1972 ^97^ | | 6 | | 6 | 298.1 - 973.1 | | | 101.0 - 101.0 |
| HELIUM | | Borisov et al, 1973 ^98^ | | 1 | | 1 | 300.0 - 300.0 | | | 101.0 - 101.0 |
| HELIUM | | Tsederberg et al, 1974 ^794^ | | 62 | | 62 | 79.6 - 273.2 | | | 100.0 - 40100.0 |
| HELIUM | | Tanaka et al, 1980 ^795^ | | 17 | | 17 | 298.1 - 323.1 | | | 101.0 - 1067.0 |
| HELIUM | | Evers et al, 2002 ^104^ | | 44 | | 44 | 293.2 - 348.2 | | | 123.0 - 9540.0 |
| HELIUM | | Reynes Thodos, 1966 ^796^ | | 36 | | 36 | 373.1 - 473.2 | | | 7136.0 - 82978.0 |
| HELIUM | | Rudenko Karnus, 1968 ^797^ | | 99 | | 0 | 10000.0 - 0.0 | | | 1000000000.0 - 0.0 |
| HELIUM | | Evers et al, 2002 ^104^ | | 42 | | 42 | 293.2 - 348.2 | | | 121.0 - 9721.0 |
| HELIUM | | Seibt et al, 2009 ^798^ | | 96 | | 96 | 293.1 - 293.3 | | | 121.0 - 30175.0 |

| Material | Author | | *N*_all_ | | *N*_used_ | | | *T* / K | *p* / kPa | |
| --- | --- | --- | --- | --- | --- | --- | --- | --- | --- | --- |
| HEPTANE | | Thorpe Rodger, 1894 ^28^ | | 13 | | 13 | 279.6 - 365.3 | | | 2.0 - 84.0 |
| HEPTANE | | Batschinski, 1913 ^29^ | | 10 | | 10 | 273.1 - 363.1 | | | 2.0 - 79.0 |
| HEPTANE | | khalilov, 1939 ^112^ | | 26 | | 25 | 293.1 - 533.2 | | | 5.0 - 2473.0 |
| HEPTANE | | Albright Lohrenz, 1956 ^799^ | | 2 | | 2 | 397.9 - 423.7 | | | 206.0 - 376.0 |
| HEPTANE | | Ledneva, 1956 ^800^ | | 11 | | 11 | 293.1 - 493.2 | | | 5.0 - 1365.0 |
| HEPTANE | | Golubev, 1959 ^8^ | | 18 | | 13 | 243.2 - 373.1 | | | 0.0 - 106.0 |
| HEPTANE | | khalimonova, 1962 ^629^ | | 12 | | 12 | 293.1 - 513.2 | | | 5.0 - 1857.0 |
| HEPTANE | | Bagdasaryan, 1964 ^30^ | | 7 | | 6 | 243.0 - 353.0 | | | 0.0 - 57.0 |
| HEPTANE | | Rabe, 1981 ^390^ | | 22 | | 13 | 250.0 - 300.0 | | | 0.0 - 7.0 |
| HEPTANE | | Brunson Byers, 1989 ^630^ | | 28 | | 28 | 292.1 - 448.2 | | | 5.0 - 620.0 |
| HEPTANE | | Abdulagatov Rasulov, 1992 ^801^ | | 9 | | 0 | 10000.0 - 0.0 | | | 1000000000.0 - 0.0 |
| HEPTANE | | Abdulagatov Rasulov, 1996 ^802^ | | 26 | | 22 | 298.0 - 537.0 | | | 6.0 - 2607.0 |
| HEPTANE | | Abdulagatov Rasulov, 1992 ^801^ | | 9 | | 3 | 536.2 - 537.2 | | | 2576.0 - 2612.0 |
| HEPTANE | | Abdulagatov Rasulov, 1996 ^802^ | | 22 | | 18 | 373.0 - 537.0 | | | 106.0 - 2607.0 |
| HEPTANE | | Thorpe Rodger, 1894 ^28^ | | 26 | | 26 | 279.6 - 365.3 | | | 101.0 - 101.0 |
| HEPTANE | | Lewis, 1925 ^36^ | | 1 | | 1 | 298.1 - 298.1 | | | 101.0 - 101.0 |
| HEPTANE | | Smyth Stoops, 1928 ^803^ | | 1 | | 1 | 293.1 - 293.1 | | | 101.0 - 101.0 |
| HEPTANE | | Tausz Staab, 1930 ^133^ | | 1 | | 1 | 293.1 - 293.1 | | | 101.0 - 101.0 |
| HEPTANE | | Shepard et al, 1931 ^266^ | | 1 | | 1 | 298.1 - 298.1 | | | 101.0 - 101.0 |
| HEPTANE | | Timmermans Hennaut, 1935 ^570^ | | 2 | | 2 | 288.1 - 303.1 | | | 101.0 - 101.0 |
| HEPTANE | | Fawcett, 1946 ^804^ | | 1 | | 1 | 293.1 - 293.1 | | | 101.0 - 101.0 |
| HEPTANE | | Giller Drickamer, 1949 ^805^ | | 9 | | 9 | 180.2 - 293.1 | | | 101.0 - 101.0 |
| HEPTANE | | Doolittle Peterson, 1951 ^267^ | | 4 | | 3 | 263.1 - 323.1 | | | 101.0 - 101.0 |
| HEPTANE | | Koshkin Nozdrev, 1953 ^806^ | | 5 | | 5 | 223.2 - 263.2 | | | 101.0 - 101.0 |
| HEPTANE | | Hammond Stokes, 1955 ^113^ | | 1 | | 1 | 298.1 - 298.1 | | | 101.0 - 101.0 |
| HEPTANE | | Golubev Agaev, 1963 ^261^ | | 1 | | 0 | 10000.0 - 0.0 | | | 1000000000.0 - 0.0 |
| HEPTANE | | Konobeev Lyapin, 1970 ^45^ | | 3 | | 3 | 293.1 - 333.1 | | | 101.0 - 101.0 |
| HEPTANE | | Zhigunov Ampelogova, 1972 ^807^ | | 3 | | 3 | 293.1 - 313.1 | | | 101.0 - 101.0 |
| HEPTANE | | Moore Wellek, 1974 ^498^ | | 4 | | 4 | 293.1 - 313.1 | | | 101.0 - 101.0 |
| HEPTANE | | Ratkovics et al, 1974 ^250^ | | 1 | | 1 | 293.1 - 293.1 | | | 101.0 - 101.0 |
| HEPTANE | | Badalov et al, 1975 ^808^ | | 73 | | 73 | 290.7 - 540.1 | | | 101.0 - 49131.0 |
| HEPTANE | | Kessel et al, 1976 ^809^ | | 7 | | 7 | 211.8 - 301.8 | | | 101.0 - 101.0 |
| HEPTANE | | Gunchuk et al, 1977 ^810^ | | 13 | | 13 | 208.2 - 363.4 | | | 101.0 - 101.0 |
| HEPTANE | | Kerchove De Vijlder, 1977 ^50^ | | 2 | | 2 | 296.0 - 296.0 | | | 101.0 - 101.0 |
| HEPTANE | | Rabe, 1981 ^390^ | | 53 | | 53 | 185.0 - 300.0 | | | 100.0 - 10000.0 |
| HEPTANE | | Kashiwagi Makita, 1982 ^152^ | | 48 | | 48 | 298.1 - 348.1 | | | 100.0 - 110000.0 |
| HEPTANE | | Awwad Allos, 1985 ^282^ | | 1 | | 1 | 298.1 - 298.1 | | | 101.0 - 101.0 |
| HEPTANE | | Awwad et al, 1986 ^156^ | | 1 | | 1 | 298.1 - 298.1 | | | 101.0 - 101.0 |
| HEPTANE | | Chandrasekhar et al, 1988 ^161^ | | 1 | | 1 | 303.1 - 303.1 | | | 101.0 - 101.0 |
| HEPTANE | | Rao Reddy, 1988 ^162^ | | 1 | | 1 | 298.1 - 298.1 | | | 101.0 - 101.0 |
| HEPTANE | | Rappon Kaukinen, 1988 ^811^ | | 6 | | 6 | 223.5 - 294.4 | | | 101.0 - 101.0 |
| HEPTANE | | Wakefield, 1988 ^287^ | | 2 | | 2 | 303.2 - 308.2 | | | 101.0 - 101.0 |
| HEPTANE | | Knapstad et al, 1989 ^164^ | | 9 | | 9 | 292.0 - 346.5 | | | 101.0 - 101.0 |
| HEPTANE | | Kouris Panayioton, 1989 ^165^ | | 1 | | 1 | 298.1 - 298.1 | | | 101.0 - 101.0 |
| HEPTANE | | Schrodt Akel, 1989 ^167^ | | 2 | | 2 | 298.0 - 298.0 | | | 101.0 - 101.0 |
| HEPTANE | | Iwahashi et al, 1990 ^271^ | | 2 | | 2 | 298.1 - 323.1 | | | 101.0 - 101.0 |
| HEPTANE | | Assael Papadaki, 1991 ^272^ | | 32 | | 32 | 298.2 - 323.2 | | | 100.0 - 69410.0 |
| HEPTANE | | Kanti et al, 1991 ^812^ | | 15 | | 15 | 313.2 - 353.2 | | | 100.0 - 40000.0 |
| HEPTANE | | Papaioannou et al, 1991 ^173^ | | 1 | | 1 | 298.2 - 298.2 | | | 101.0 - 101.0 |
| HEPTANE | | Zhang Liu, 1991 ^56^ | | 7 | | 7 | 298.2 - 298.2 | | | 100.0 - 29500.0 |
| HEPTANE | | Assael et al, 1992 ^813^ | | 46 | | 36 | 303.0 - 348.0 | | | 100.0 - 151720.0 |
| HEPTANE | | Blanco et al, 1993 ^814^ | | 4 | | 4 | 288.2 - 318.2 | | | 101.0 - 101.0 |
| HEPTANE | | Aminabhavi et al, 1994 ^815^ | | 5 | | 5 | 298.2 - 318.2 | | | 101.0 - 101.0 |
| HEPTANE | | Pandey et al, 1994 ^414^ | | 1 | | 1 | 298.2 - 298.2 | | | 101.0 - 101.0 |
| HEPTANE | | Papaionnou Panayioton, 1994 ^657^ | | 18 | | 18 | 298.2 - 298.2 | | | 101.0 - 51850.0 |
| HEPTANE | | Aminabhavi Gopalakrishna, 1995 ^290^ | | 4 | | 4 | 298.2 - 313.2 | | | 101.0 - 101.0 |
| HEPTANE | | Aucejo et al, 1995 ^14^ | | 2 | | 2 | 298.2 - 298.2 | | | 101.0 - 101.0 |
| HEPTANE | | Aminabhavi et al, 1996 ^417^ | | 3 | | 3 | 298.2 - 308.2 | | | 101.0 - 101.0 |
| HEPTANE | | Sastry Raj, 1996 ^816^ | | 2 | | 2 | 303.2 - 313.2 | | | 101.0 - 101.0 |
| HEPTANE | | Sastry Valand, 1996 ^817^ | | 2 | | 2 | 298.2 - 308.2 | | | 101.0 - 101.0 |
| HEPTANE | | Sastry Valand, 1996 ^817^ | | 2 | | 2 | 298.2 - 308.2 | | | 101.0 - 101.0 |
| HEPTANE | | Aminabhavi Patil, 1997 ^291^ | | 3 | | 3 | 298.2 - 308.2 | | | 101.0 - 101.0 |
| HEPTANE | | Baylaucq et al, 1997 ^331^ | | 19 | | 19 | 303.2 - 343.2 | | | 100.0 - 100000.0 |
| HEPTANE | | Baylaucq et al, 1997 ^331^ | | 1 | | 1 | 303.2 - 303.2 | | | 101.0 - 101.0 |
| HEPTANE | | Orge et al, 1997 ^667^ | | 1 | | 1 | 298.2 - 298.2 | | | 101.0 - 101.0 |
| HEPTANE | | Will Leipertz, 1997 ^818^ | | 8 | | 8 | 293.2 - 353.2 | | | 101.0 - 101.0 |
| HEPTANE | | Dandekar et al, 1998 ^314^ | | 1 | | 1 | 293.2 - 293.2 | | | 101.0 - 101.0 |
| HEPTANE | | Jimenez et al, 1998 ^819^ | | 4 | | 4 | 293.2 - 308.2 | | | 101.0 - 101.0 |
| HEPTANE | | Aralaguppi et al, 1999 ^189^ | | 3 | | 3 | 298.2 - 308.2 | | | 101.0 - 101.0 |
| HEPTANE | | Sastry et al, 1999 ^191^ | | 3 | | 3 | 298.2 - 318.2 | | | 101.0 - 101.0 |
| HEPTANE | | Matos et al, 2001 ^820^ | | 4 | | 4 | 283.2 - 313.2 | | | 101.0 - 101.0 |
| HEPTANE | | Nayak et al, 2001 ^292^ | | 3 | | 3 | 298.2 - 308.2 | | | 101.0 - 101.0 |
| HEPTANE | | Peng Tu, 2002 ^61^ | | 1 | | 1 | 298.2 - 298.2 | | | 101.0 - 101.0 |
| HEPTANE | | Oswal et al, 2003 ^205^ | | 1 | | 1 | 303.2 - 303.2 | | | 101.0 - 101.0 |
| HEPTANE | | Queimada et al, 2003 ^316^ | | 5 | | 5 | 293.2 - 323.2 | | | 101.0 - 101.0 |
| HEPTANE | | Ma et al, 2004 ^207^ | | 18 | | 18 | 298.2 - 403.2 | | | 100.0 - 5100.0 |
| HEPTANE | | Oswal et al, 2004 ^208^ | | 1 | | 1 | 303.2 - 303.2 | | | 101.0 - 101.0 |
| HEPTANE | | Yang et al, 2004 ^209^ | | 7 | | 7 | 293.2 - 343.2 | | | 101.0 - 101.0 |
| HEPTANE | | Yang et al, 2004 ^209^ | | 6 | | 6 | 298.2 - 343.2 | | | 101.0 - 101.0 |
| HEPTANE | | Aljimaz et al, 2005 ^300^ | | 3 | | 3 | 293.2 - 303.2 | | | 101.0 - 101.0 |
| HEPTANE | | Ma et al, 2005 ^821^ | | 1 | | 1 | 298.2 - 298.2 | | | 101.0 - 101.0 |
| HEPTANE | | Pensado et al, 2005 ^822^ | | 84 | | 84 | 303.2 - 353.2 | | | 100.0 - 60000.0 |
| HEPTANE | | Abdulagatov et al, 2006 ^823^ | | 15 | | 15 | 298.2 - 473.5 | | | 100.0 - 10000.0 |
| HEPTANE | | Baragi Aralaguppi, 2006 ^337^ | | 3 | | 3 | 298.2 - 308.2 | | | 101.0 - 101.0 |
| HEPTANE | | Iloukhani et al, 2006 ^339^ | | 3 | | 3 | 293.2 - 303.2 | | | 101.0 - 101.0 |
| HEPTANE | | Suresh Reddy et al, 2006 ^215^ | | 1 | | 1 | 303.2 - 303.2 | | | 101.0 - 101.0 |
| HEPTANE | | Zeberg Mikkelsen et al, 2006 ^705^ | | 24 | | 24 | 293.2 - 353.2 | | | 100.0 - 100000.0 |
| HEPTANE | | Landaverde-Cortes et al, 2007 ^824^ | | 9 | | 9 | 273.2 - 313.2 | | | 101.0 - 101.0 |
| HEPTANE | | Reddy et al, 2007 ^220^ | | 1 | | 1 | 303.2 - 303.2 | | | 101.0 - 101.0 |
| HEPTANE | | Wang et al, 2007 ^825^ | | 5 | | 5 | 293.2 - 313.2 | | | 101.0 - 101.0 |
| HEPTANE | | Fan et al, 2008 ^431^ | | 5 | | 5 | 288.2 - 308.2 | | | 101.0 - 101.0 |
| HEPTANE | | EI-Sayed Asfour, 2009 ^826^ | | 2 | | 2 | 308.2 - 313.2 | | | 101.0 - 101.0 |
| HEPTANE | | Rathnam et al, 2010 ^437^ | | 3 | | 3 | 303.2 - 313.2 | | | 101.0 - 101.0 |
| HEPTANE | | Yang et al, 2010 ^341^ | | 3 | | 3 | 298.2 - 313.2 | | | 101.0 - 101.0 |
| HEPTANE | | Zivkovic et al, 2010 ^516^ | | 1 | | 1 | 303.2 - 303.2 | | | 101.0 - 101.0 |
| HEPTANE | | Rathnam et al, 2012 ^439^ | | 3 | | 3 | 303.2 - 313.2 | | | 101.0 - 101.0 |
| HEPTANE | | Sagdeev et al, 2013 ^827^ | | 52 | | 36 | 298.2 - 470.2 | | | 98.0 - 147100.0 |
| HEPTANE | | Fang et al, 2014 ^518^ | | 4 | | 4 | 283.2 - 303.2 | | | 101.0 - 101.0 |
| HEPTANE | | Liu Zhu, 2014 ^307^ | | 5 | | 5 | 293.2 - 313.2 | | | 100.0 - 100.0 |
| HEPTANE | | Luning Prak et al, 2014 ^828^ | | 8 | | 8 | 293.2 - 333.2 | | | 100.0 - 100.0 |
| HEPTANE | | Melaven Mack, 1932 ^829^ | | 4 | | 4 | 373.5 - 524.8 | | | 101.0 - 101.0 |
| HEPTANE | | Khalilov et al, 1939 ^112^ | | 22 | | 17 | 373.1 - 533.2 | | | 101.0 - 101.0 |
| HEPTANE | | Lambert et al, 1955 ^12^ | | 4 | | 0 | 10000.0 - 0.0 | | | 1000000000.0 - 0.0 |
| HEPTANE | | Agaev Golubev, 1963 ^830^ | | 5 | | 5 | 373.6 - 474.1 | | | 101.0 - 101.0 |
| HEPTANE | | Carmichael et al, 1966 ^831^ | | 2 | | 2 | 310.9 - 344.2 | | | 3.0 - 3.0 |
| HEPTANE | | Diaz Pena Cheda, 1974 ^832^ | | 10 | | 6 | 373.1 - 523.1 | | | 101.0 - 101.0 |
| HEPTANE | | Vogel Holdt, 1991 ^833^ | | 74 | | 74 | 317.7 - 634.4 | | | 13.0 - 212.0 |
| HEPTANE | | Abdulagatov Rasulov, 1992 ^801^ | | 56 | | 54 | 298.3 - 550.4 | | | 100.0 - 50000.0 |
| HEPTANE | | Abdulagatov Rasulov, 1996 ^802^ | | 96 | | 96 | 540.2 - 548.0 | | | 2000.0 - 3100.0 |
| HEPTANE | | Abdulagatov Rasulov, 1996 ^834^ | | 16 | | 16 | 540.2 - 540.2 | | | 2000.0 - 3100.0 |

| Material | | Author | *N*_all_ | | *N*_used_ | *T* / K | *p* / kPa |
| --- | --- | --- | --- | --- | --- | --- | --- |
| HEXANE | Thorpe Rodger, 1894 ^28^ | | | 7 | 7 | 273.2 - 333.1 | 6.0 - 77.0 |
| HEXANE | Batschinski, 1913 ^29^ | | | 7 | 7 | 273.1 - 333.1 | 6.0 - 77.0 |
| HEXANE | Khalilov, 1939 ^112^ | | | 19 | 19 | 293.1 - 473.2 | 17.0 - 1816.0 |
| HEXANE | Albright Lohrenz, 1956 ^799^ | | | 3 | 3 | 367.9 - 425.8 | 216.0 - 796.0 |
| HEXANE | Ledneva, 1956 ^800^ | | | 11 | 11 | 293.1 - 453.1 | 17.0 - 1308.0 |
| HEXANE | Agaev Golubev, 1963 ^830^ | | | 20 | 18 | 343.1 - 503.2 | 106.0 - 2841.0 |
| HEXANE | Bagdasaryan, 1964 ^30^ | | | 7 | 6 | 213.1 - 313.0 | 0.0 - 38.0 |
| HEXANE | Bulanov et al, 1974 ^117^ | | | 11 | 11 | 293.1 - 372.1 | 17.0 - 241.0 |
| HEXANE | Isdale et al, 1979 ^400^ | | | 2 | 2 | 298.1 - 323.1 | 21.0 - 55.0 |
| HEXANE | Dymond Young, 1980 ^275^ | | | 11 | 11 | 283.2 - 393.2 | 11.0 - 402.0 |
| HEXANE | Agaev Golubev, 1963 ^830^ | | | 20 | 18 | 343.1 - 503.2 | 106.0 - 2840.0 |
| HEXANE | Thorpe Rodger, 1894 ^28^ | | | 24 | 24 | 274.0 - 336.7 | 101.0 - 101.0 |
| HEXANE | Drapier, 1911 ^402^ | | | 5 | 5 | 289.5 - 296.4 | 101.0 - 101.0 |
| HEXANE | Bingham, 1913 ^633^ | | | 4 | 4 | 298.1 - 338.1 | 101.0 - 101.0 |
| HEXANE | Muchin, 1913 ^123^ | | | 2 | 2 | 291.8 - 293.1 | 101.0 - 101.0 |
| HEXANE | Timmermans Martin, 1928 ^37^ | | | 2 | 2 | 288.1 - 303.1 | 101.0 - 101.0 |
| HEXANE | Tausz Staab, 1930 ^133^ | | | 1 | 1 | 293.1 - 293.1 | 101.0 - 101.0 |
| HEXANE | Shepard et al, 1931 ^266^ | | | 1 | 1 | 298.1 - 298.1 | 101.0 - 101.0 |
| HEXANE | Andrade Rotheham, 1936 ^835^ | | | 2 | 2 | 273.2 - 295.0 | 101.0 - 101.0 |
| HEXANE | Giller Drickamer, 1949 ^805^ | | | 7 | 7 | 174.7 - 293.1 | 101.0 - 101.0 |
| HEXANE | Grunberg, 1950 ^836^ | | | 1 | 1 | 293.1 - 293.1 | 101.0 - 101.0 |
| HEXANE | Koshkin Nozdrev, 1953 ^806^ | | | 5 | 5 | 223.2 - 263.2 | 101.0 - 101.0 |
| HEXANE | Hammond Stokes, 1955 ^113^ | | | 1 | 1 | 298.1 - 298.1 | 101.0 - 101.0 |
| HEXANE | Parisot Johnson, 1961 ^837^ | | | 11 | 11 | 299.7 - 454.8 | 103.0 - 2213.0 |
| HEXANE | Golubev Agaev, 1963 ^261^ | | | 2 | 0 | 10000.0 - 0.0 | 1000000000.0 - 0.0 |
| HEXANE | Ridgway Butler, 1967 ^144^ | | | 1 | 1 | 298.1 - 298.1 | 101.0 - 101.0 |
| HEXANE | Brazier Freeman, 1969 ^11^ | | | 27 | 17 | 273.1 - 333.1 | 100.0 - 250000.0 |
| HEXANE | Konobeev Lyapin, 1970 ^45^ | | | 3 | 3 | 293.1 - 333.1 | 101.0 - 101.0 |
| HEXANE | Naziev et al, 1972 ^838^ | | | 30 | 30 | 297.1 - 333.1 | 101.0 - 39200.0 |
| HEXANE | Moore Wellek, 1974 ^498^ | | | 4 | 4 | 293.1 - 313.1 | 101.0 - 101.0 |
| HEXANE | Ratkovics et al, 1974 ^250^ | | | 1 | 1 | 293.1 - 293.1 | 101.0 - 101.0 |
| HEXANE | Strumpf et al, 1974 ^839^ | | | 8 | 8 | 290.2 - 323.1 | 101.0 - 101.0 |
| HEXANE | Bulanov Skripov, 1975 ^840^ | | | 61 | 31 | 373.1 - 473.1 | 507.0 - 2533.0 |
| HEXANE | Isdale et al, 1979 ^400^ | | | 27 | 14 | 298.1 - 373.1 | 100.0 - 200000.0 |
| HEXANE | Dymond Young, 1980 ^275^ | | | 35 | 20 | 298.3 - 373.3 | 100.0 - 204500.0 |
| HEXANE | Asfour Dullien, 1981 ^150^ | | | 1 | 1 | 298.1 - 298.1 | 101.0 - 101.0 |
| HEXANE | Kaschiwagi Makita, 1982 ^152^ | | | 48 | 48 | 298.1 - 333.1 | 100.0 - 110000.0 |
| HEXANE | Singh Sinha, 1982 ^365^ | | | 1 | 1 | 298.1 - 298.1 | 101.0 - 101.0 |
| HEXANE | Albright et al, 1983 ^154^ | | | 1 | 1 | 298.2 - 298.2 | 101.0 - 101.0 |
| HEXANE | Singh Sinha, 1984 ^367^ | | | 4 | 4 | 303.1 - 333.1 | 101.0 - 101.0 |
| HEXANE | Awwad Allos, 1985 ^282^ | | | 1 | 1 | 298.1 - 298.1 | 101.0 - 101.0 |
| HEXANE | Awwad et al, 1986 ^156^ | | | 1 | 1 | 298.1 - 298.1 | 101.0 - 101.0 |
| HEXANE | Awwad Salman, 1986 ^284^ | | | 1 | 1 | 298.1 - 298.1 | 101.0 - 101.0 |
| HEXANE | Craubner, 1986 ^841^ | | | 1 | 1 | 293.1 - 293.1 | 101.0 - 101.0 |
| HEXANE | Singh et al, 1986 ^370^ | | | 1 | 1 | 298.1 - 298.1 | 101.0 - 101.0 |
| HEXANE | Hamelin et al, 1988 ^842^ | | | 1 | 1 | 303.1 - 303.1 | 101.0 - 101.0 |
| HEXANE | Grigor’ev et al, 1988 ^843^ | | | 53 | 53 | 178.2 - 323.1 | 100.0 - 58900.0 |
| HEXANE | Rao Reddy, 1988 ^162^ | | | 1 | 1 | 298.1 - 298.1 | 101.0 - 101.0 |
| HEXANE | Knapstad et al, 1989 ^164^ | | | 7 | 7 | 288.6 - 326.6 | 101.0 - 101.0 |
| HEXANE | Schrodt Akel, 1989 ^167^ | | | 1 | 1 | 298.0 - 298.0 | 101.0 - 101.0 |
| HEXANE | Iwahashi et al, 1990 ^271^ | | | 2 | 2 | 298.1 - 323.1 | 101.0 - 101.0 |
| HEXANE | Sekar et al, 1990 ^169^ | | | 1 | 1 | 303.2 - 303.2 | 101.0 - 101.0 |
| HEXANE | Cueto et al, 1991 ^409^ | | | 6 | 6 | 288.0 - 313.0 | 101.0 - 101.0 |
| HEXANE | Kiran Sen, 1992 ^263^ | | | 76 | 76 | 313.0 - 448.0 | 210.0 - 65850.0 |
| HEXANE | Liu Luo, 1992 ^57^ | | | 4 | 4 | 293.2 - 313.2 | 101.0 - 101.0 |
| HEXANE | Oliveira Wakeham, 1992 ^502^ | | | 38 | 34 | 303.2 - 348.2 | 100.0 - 201090.0 |
| HEXANE | Oswad Davel, 1992 ^844^ | | | 7 | 7 | 303.2 - 303.2 | 101.0 - 101.0 |
| HEXANE | Oswad Patel, 1992 ^177^ | | | 2 | 2 | 303.2 - 313.2 | 101.0 - 101.0 |
| HEXANE | Aminabhavi et al, 1994 ^815^ | | | 5 | 5 | 298.2 - 318.2 | 101.0 - 101.0 |
| HEXANE | Munoz et al, 1994 ^413^ | | | 5 | 5 | 293.0 - 313.0 | 101.0 - 101.0 |
| HEXANE | Pandey et al,1994 ^414^ | | | 1 | 1 | 298.2 - 298.2 | 101.0 - 101.0 |
| HEXANE | Papaioannou Panayioton, 1994 ^657^ | | | 12 | 12 | 298.2 - 298.2 | 101.0 - 51800.0 |
| HEXANE | Aminabhavi Gopalakrishna, 1995 ^290^ | | | 4 | 4 | 298.2 - 313.2 | 101.0 - 101.0 |
| HEXANE | Aucejo et al, 1995 ^14^ | | | 1 | 1 | 298.2 - 298.2 | 101.0 - 101.0 |
| HEXANE | Franjo et al, 1995 ^845^ | | | 1 | 1 | 298.2 - 298.2 | 101.0 - 101.0 |
| HEXANE | Rived et al, 1995 ^183^ | | | 1 | 1 | 303.2 - 303.2 | 101.0 - 101.0 |
| HEXANE | Aminabhavi et al, 1996 ^417^ | | | 3 | 3 | 298.2 - 308.2 | 101.0 - 101.0 |
| HEXANE | Dominguez et al, 1996 ^846^ | | | 2 | 2 | 298.2 - 313.2 | 101.0 - 101.0 |
| HEXANE | Sastry Raj, 1996 ^816^ | | | 2 | 2 | 303.2 - 313.2 | 101.0 - 101.0 |
| HEXANE | Aminabhavi Patil, 1997 ^291^ | | | 3 | 3 | 298.2 - 308.2 | 101.0 - 101.0 |
| HEXANE | Orge et al, 1997 ^667^ | | | 1 | 1 | 298.2 - 298.2 | 101.0 - 101.0 |
| HEXANE | Casad et al, 1998 ^186^ | | | 1 | 1 | 298.2 - 298.2 | 101.0 - 101.0 |
| HEXANE | Dominguez et al, 1998 ^847^ | | | 2 | 2 | 298.2 - 313.2 | 101.0 - 101.0 |
| HEXANE | Aralaguppi et al, 1999 ^189^ | | | 3 | 3 | 298.2 - 308.2 | 101.0 - 101.0 |
| HEXANE | Oswal Dave, 1999 ^844^ | | | 1 | 1 | 303.2 - 303.2 | 101.0 - 101.0 |
| HEXANE | Dominguez et al, 2000 ^848^ | | | 2 | 2 | 298.2 - 313.2 | 101.0 - 101.0 |
| HEXANE | Chowdhury et al, 2001 ^197^ | | | 3 | 3 | 303.0 - 323.0 | 101.0 - 101.0 |
| HEXANE | Gascon et al, 2001 ^849^ | | | 2 | 2 | 298.2 - 313.2 | 101.0 - 101.0 |
| HEXANE | Nayak et al, 2001 ^292^ | | | 3 | 3 | 298.2 - 308.2 | 101.0 - 101.0 |
| HEXANE | Comelli et al, 2002 ^850^ | | | 1 | 1 | 298.2 - 298.2 | 101.0 - 101.0 |
| HEXANE | Garcia et al, 2002 ^293^ | | | 5 | 5 | 278.2 - 318.2 | 101.0 - 101.0 |
| HEXANE | Peng Tu, 2002 ^61^ | | | 1 | 1 | 298.2 - 298.2 | 101.0 - 101.0 |
| HEXANE | Bolotnikov Neruchev, 2003 ^851^ | | | 9 | 9 | 293.2 - 333.2 | 101.0 - 101.0 |
| HEXANE | Dominguez et al, 2003 ^852^ | | | 2 | 2 | 298.2 - 313.2 | 101.0 - 101.0 |
| HEXANE | Nayak et al, 2003 ^374^ | | | 3 | 3 | 298.2 - 308.2 | 101.0 - 101.0 |
| HEXANE | Oswal et al, 2003 ^205^ | | | 2 | 2 | 303.2 - 303.2 | 101.0 - 101.0 |
| HEXANE | Yang et al, 2004 ^209^ | | | 6 | 6 | 293.2 - 333.2 | 101.0 - 101.0 |
| HEXANE | Aljimaz et al, 2005 ^300^ | | | 3 | 3 | 293.2 - 303.2 | 101.0 - 101.0 |
| HEXANE | Gascon et al, 2005 ^853^ | | | 2 | 2 | 298.2 - 313.2 | 101.0 - 101.0 |
| HEXANE | Ma et al, 2005 ^821^ | | | 1 | 1 | 298.2 - 298.2 | 101.0 - 101.0 |
| HEXANE | Oswal et al, 2005 ^210^ | | | 1 | 1 | 303.2 - 303.2 | 101.0 - 101.0 |
| HEXANE | Tripathi et al, 2005 ^317^ | | | 1 | 1 | 298.2 - 298.2 | 101.0 - 101.0 |
| HEXANE | Baragi Aralaguppi, 2006 ^337^ | | | 3 | 3 | 298.2 - 308.2 | 101.0 - 101.0 |
| HEXANE | Iloukhani et al, 2006 ^339^ | | | 3 | 3 | 293.2 - 303.2 | 101.0 - 101.0 |
| HEXANE | Kumagai et al, 2006 ^264^ | | | 16 | 16 | 273.2 - 333.2 | 100.0 - 30000.0 |
| HEXANE | Modarress Mohsen-Nia, 2006 ^854^ | | | 3 | 3 | 298.2 - 313.2 | 101.0 - 101.0 |
| HEXANE | Rodriguez et al, 2006 ^536^ | | | 4 | 4 | 293.2 - 313.2 | 101.0 - 101.0 |
| HEXANE | Suresh Reddy et al, 2006 ^215^ | | | 1 | 1 | 303.2 - 303.2 | 101.0 - 101.0 |
| HEXANE | Dubey Sharma, 2007 ^510^ | | | 1 | 1 | 298.2 - 298.2 | 101.0 - 101.0 |
| HEXANE | Reddy et al, 2007 ^220^ | | | 1 | 1 | 303.2 - 303.2 | 101.0 - 101.0 |
| HEXANE | Tian Liu, 2007 ^302^ | | | 7 | 7 | 298.2 - 328.2 | 101.0 - 101.0 |
| HEXANE | Carrasco et al, 2008 ^855^ | | | 1 | 1 | 298.2 - 298.2 | 101.0 - 101.0 |
| HEXANE | Dubey Sharma, 2008 ^319^ | | | 1 | 1 | 298.2 - 298.2 | 101.0 - 101.0 |
| HEXANE | Dubey Sharma, 2008 ^319^ | | | 1 | 1 | 298.2 - 298.2 | 101.0 - 101.0 |
| HEXANE | Dubey Sharma, 2008 ^319^ | | | 1 | 1 | 298.2 - 298.2 | 101.0 - 101.0 |
| HEXANE | Dubey Sharma, 2008 ^319^ | | | 1 | 1 | 298.2 - 298.2 | 101.0 - 101.0 |
| HEXANE | Fan et al, 2008 ^431^ | | | 3 | 3 | 293.2 - 303.2 | 101.0 - 101.0 |
| HEXANE | Shukla et al, 2008 ^224^ | | | 1 | 1 | 298.2 - 298.2 | 101.0 - 101.0 |
| HEXANE | Dominguez-Perez et al, 2009 ^560^ | | | 1 | 1 | 298.2 - 298.2 | 101.0 - 101.0 |
| HEXANE | Feitosa et al, 2009 ^514^ | | | 2 | 2 | 293.2 - 298.2 | 101.0 - 101.0 |
| HEXANE | Sastry et al, 2009 ^231^ | | | 2 | 2 | 298.2 - 308.2 | 101.0 - 101.0 |
| HEXANE | Rathnam et al, 2010 ^437^ | | | 3 | 3 | 303.2 - 313.2 | 101.0 - 101.0 |
| HEXANE | Rathnam et al, 2012 ^439^ | | | 3 | 3 | 303.2 - 313.2 | 101.0 - 101.0 |
| HEXANE | Morgado et al, 2013 ^353^ | | | 3 | 3 | 298.2 - 308.1 | 101.0 - 101.0 |
| HEXANE | Titani, 1930 ^9^ | | | 7 | 7 | 393.8 - 579.8 | 101.0 - 101.0 |
| HEXANE | Titani, 1933 ^75^ | | | 7 | 7 | 393.8 - 579.8 | 101.0 - 101.0 |
| HEXANE | Khalilov, 1939 ^112^ | | | 19 | 14 | 343.1 - 473.2 | 101.0 - 101.0 |
| HEXANE | Craven Lambert, 1951 ^76^ | | | 4 | 1 | 350.9 - 350.9 | 101.0 - 101.0 |
| HEXANE | McCoubrey et al, 1951 ^856^ | | | 10 | 8 | 349.1 - 462.2 | 101.0 - 101.0 |
| HEXANE | Lambert et al, 1955 ^12^ | | | 4 | 1 | 351.1 - 351.1 | 101.0 - 101.0 |
| HEXANE | Schuler Michel, 1956 ^857^ | | | 4 | 1 | 351.1 - 351.1 | 101.0 - 101.0 |
| HEXANE | Agaev Usibova, 1968 ^478^ | | | 7 | 7 | 348.0 - 493.5 | 101.0 - 101.0 |
| HEXANE | Diaz Pena Cheda, 1975 ^255^ | | | 9 | 9 | 323.1 - 408.1 | 27.0 - 27.0 |
| HEXANE | Grachev et al, 1989 ^858^ | | | 132 | 131 | 505.1 - 623.1 | 1000.0 - 60000.0 |
| HEXANE | Assael Papadaki, 1991 ^272^ | | | 15 | 15 | 298.2 - 298.2 | 100.0 - 70600.0 |

| Material | Author | | *N*_all_ | | | *N*_used_ | | *T* / K | | *p* / kPa |
| --- | --- | --- | --- | --- | --- | --- | --- | --- | --- | --- |
| HYDROGEN | | Van Itterbeek Claes, 1938 ^457^ | | 4 | 1 | | 14.1 - 14.1 | | 3.0 - 3.0 | |
| HYDROGEN | | Van Itterbeek Paemel, 1940 ^859^ | | 2 | 0 | | 10000.0 - 0.0 | | 1000000000.0 - 0.0 | |
| HYDROGEN | | Van Itterbeek Paemel, 1941 ^460^ | | 5 | 5 | | 14.7 - 20.4 | | 4.0 - 71.0 | |
| HYDROGEN | | Rudenko Konareva, 1963 ^455^ | | 8 | 8 | | 14.5 - 20.4 | | 4.0 - 72.0 | |
| HYDROGEN | | Diller, 1965 ^860^ | | 13 | 13 | | 14.0 - 26.0 | | 2.0 - 355.0 | |
| HYDROGEN | | Konareva Rudenko, 1967 ^465^ | | 10 | 8 | | 14.0 - 28.0 | | 2.0 - 542.0 | |
| HYDROGEN | | Breitenbath, 1901 ^861^ | | 5 | 5 | | 252.6 - 575.2 | | 101.0 - 101.0 | |
| HYDROGEN | | Vogel, 1914 ^80^ | | 7 | 7 | | 21.0 - 273.1 | | 15.0 - 99.0 | |
| HYDROGEN | | Verschaffelt Nicaise, 1917 ^862^ | | 1 | 1 | | 20.4 - 20.4 | | 102.0 - 102.0 | |
| HYDROGEN | | Yen, 1919 ^863^ | | 1 | 1 | | 296.1 - 296.1 | | 101.0 - 101.0 | |
| HYDROGEN | | Klein et al, 1997 ^864^ | | 1 | 1 | | 273.0 - 273.0 | | 101.0 - 101.0 | |
| HYDROGEN | | Trautz Baumann, 1929 ^865^ | | 12 | 12 | | 195.1 - 523.2 | | 101.0 - 101.0 | |
| HYDROGEN | | Trautz Ludewigs, 1929 ^866^ | | 20 | 20 | | 288.6 - 523.6 | | 101.0 - 101.0 | |
| HYDROGEN | | Trautz Stauf, 1929 ^761^ | | 15 | 15 | | 192.4 - 524.9 | | 101.0 - 101.0 | |
| HYDROGEN | | Trautz Zink, 1930 ^762^ | | 8 | 8 | | 293.1 - 550.1 | | 101.0 - 101.0 | |
| HYDROGEN | | Trautz Zink, 1930 ^762^ | | 8 | 8 | | 289.8 - 1099.2 | | 101.0 - 101.0 | |
| HYDROGEN | | Trautz Kurtz, 1931 ^867^ | | 12 | 12 | | 300.0 - 551.2 | | 101.0 - 101.0 | |
| HYDROGEN | | Trautz Sorg, 1931 ^618^ | | 12 | 12 | | 292.6 - 523.2 | | 101.0 - 101.0 | |
| HYDROGEN | | Sutherland Maass, 1932 ^868^ | | 11 | 11 | | 74.8 - 293.8 | | 101.0 - 101.0 | |
| HYDROGEN | | Trautz Zimmermann, 1935 ^869^ | | 1 | 1 | | 90.0 - 90.0 | | 101.0 - 101.0 | |
| HYDROGEN | | Van Cleave Maass, 1935 ^87^ | | 1 | 1 | | 295.1 - 295.1 | | 101.0 - 101.0 | |
| HYDROGEN | | Van Itterbeek Claes, 1938 ^457^ | | 11 | 6 | | 14.0 - 292.9 | | 1.0 - 22.0 | |
| HYDROGEN | | Johns, 1939 ^870^ | | 42 | 42 | | 14.3 - 20.7 | | 152.0 - 278.0 | |
| HYDROGEN | | Johnston McCloskey, 1940 ^871^ | | 7 | 7 | | 299.9 - 299.9 | | 21.0 - 101.0 | |
| HYDROGEN | | Wobser Mueller,1941 ^79^ | | 5 | 5 | | 293.1 - 371.1 | | 101.0 - 101.0 | |
| HYDROGEN | | Buddenberg Wilke, 1951 ^872^ | | 7 | 7 | | 293.1 - 301.2 | | 101.0 - 101.0 | |
| HYDROGEN | | Kuss, 1952 ^873^ | | 27 | 27 | | 298.1 - 348.1 | | 98.0 - 49033.0 | |
| HYDROGEN | | Kompaneets, 1953 ^874^ | | 7 | 7 | | 284.1 - 873.2 | | 101.0 - 101.0 | |
| HYDROGEN | | Michels, 1953 ^458^ | | 95 | 95 | | 298.1 - 398.1 | | 2592.0 - 186258.0 | |
| HYDROGEN | | kestin pilarczyk, 1954 ^875^ | | 1 | 1 | | 294.1 - 294.1 | | 101.0 - 101.0 | |
| HYDROGEN | | Rietveld, 1957 ^876^ | | 7 | 5 | | 71.5 - 293.1 | | 4.0 - 5.0 | |
| HYDROGEN | | Coremans et al, 1958 ^454^ | | 14 | 5 | | 47.9 - 77.8 | | 101.0 - 101.0 | |
| HYDROGEN | | Kestin Wang, 1958 ^877^ | | 10 | 10 | | 298.1 - 298.1 | | 101.0 - 7093.0 | |
| HYDROGEN | | Golubev, 1959 ^8^ | | 58 | 58 | | 288.1 - 523.2 | | 101.0 - 81060.0 | |
| HYDROGEN | | Kestin Leidenfrost, 1959 ^467^ | | 9 | 9 | | 293.1 - 293.2 | | 444.0 - 8362.0 | |
| HYDROGEN | | Rietveld Velds, 1959 ^461^ | | 14 | 9 | | 71.5 - 293.1 | | 2.0 - 5.0 | |
| HYDROGEN | | Barua et al, 1964 ^462^ | | 38 | 38 | | 223.1 - 423.1 | | 1019.0 - 17784.0 | |
| HYDROGEN | | Menabde, 1965 ^459^ | | 11 | 11 | | 77.4 - 299.6 | | 6.0 - 6.0 | |
| HYDROGEN | | Golubev Shepeleva, 1966 ^878^ | | 96 | 96 | | 77.4 - 273.2 | | 899.0 - 48183.0 | |
| HYDROGEN | | Gracki et al, 1969 ^879^ | | 42 | 42 | | 173.2 - 298.1 | | 462.0 - 17134.0 | |
| HYDROGEN | | Guevara et al, 1969 ^880^ | | 24 | 24 | | 283.0 - 2127.4 | | 101.0 - 101.0 | |
| HYDROGEN | | Kestin, 1972 ^97^ | | 3 | 3 | | 295.5 - 308.4 | | 103.0 - 106.0 | |
| HYDROGEN | | Carey et al, 1974 ^109^ | | 18 | 18 | | 269.3 - 299.2 | | 146.0 - 11024.0 | |
| HYDROGEN | | Chuang et al, 1976 ^881^ | | 37 | 37 | | 173.2 - 273.2 | | 404.0 - 50588.0 | |
| HYDROGEN | | Clifford et al, 1981 ^882^ | | 2 | 2 | | 298.1 - 308.1 | | 100.0 - 100.0 | |
| HYDROGEN | | Lukin et al, 1983 ^452^ | | 23 | 23 | | 76.5 - 293.2 | | 101.0 - 101.0 | |
| HYDROGEN | | Handel et al, 1992 ^883^ | | 3 | 3 | | 500.0 - 1100.0 | | 300.0 - 300.0 | |
| HYDROGEN | | Nabizadeh Mayinger, 1999^884^ | | 76 | 76 | | 295.6 - 399.2 | | 98.0 - 5775.0 | |
| HYDROGEN | | Maltsev et al, 2004 ^399^ | | 3 | 3 | | 500.0 - 1100.0 | | 300.0 - 300.0 | |
| HYDROGEN | | Boyd, 1930 ^885^ | | 56 | 56 | | 303.1 - 343.1 | | 7214.0 - 19424.0 | |
| HYDROGEN | | Trautz Heberling, 1931 ^86^ | | 12 | 12 | | 295.2 - 523.4 | | 101.0 - 101.0 | |
| HYDROGEN | | Adzumi, 1937 ^244^ | | 9 | 9 | | 293.1 - 373.1 | | 101.0 - 101.0 | |
| HYDROGEN | | Adzumi, 1937 ^244^ | | 1 | 1 | | 293.1 - 293.1 | | 0.0 - 0.0 | |
| HYDROGEN | | Keesom MacWood, 1938 ^886^ | | 14 | 0 | | 10000.0 - 0.0 | | 1000000000.0 - 0.0 | |
| HYDROGEN | | Van Itterbeek Claes, 1938 ^457^ | | 4 | 3 | | 77.3 - 293.0 | | 100.0 - 100.0 | |
| HYDROGEN | | Keesom Keesom, 1940^887^ | | 21 | 0 | | 10000.0 - 0.0 | | 1000000000.0 - 0.0 | |
| HYDROGEN | | Van Itterbeek Paemel, 1940 ^859^ | | 4 | 3 | | 68.9 - 291.8 | | 100.0 - 100.0 | |
| HYDROGEN | | Van Itterbeek Paemel, 1940 ^453^ | | 34 | 12 | | 14.9 - 68.9 | | 0.0 - 1.0 | |
| HYDROGEN | | Tsederberg et al, 1965 ^888^ | | 28 | 28 | | 288.6 - 990.3 | | 4350.0 - 50550.0 | |
| HYDROGEN | | Borisov et al, 1973 ^98^ | | 1 | 1 | | 300.0 - 300.0 | | 101.0 - 101.0 | |
| HYDROGEN | | Yusibani et al, 2011 ^889^ | | 17 | 17 | | 294.1 - 400.1 | | 4680.0 - 99320.0 | |
| HYDROGEN | | Rudenko Slyusar, 1968 ^466^ | | 84 | 60 | | 100.0 - 300.0 | | 5644.0 - 278736.0 | |

| Material | | Author | *N*_all_ | | | *N*_used_ | | *T* / K | *p* / kPa | |
| --- | --- | --- | --- | --- | --- | --- | --- | --- | --- | --- |
| IBUTENE | Trautz Husseini, 1934 ^348^ | | | 8 | 8 | | 290.8 - 523.0 | | | 101.0 - 1876.0 |
| IBUTENE | Garner et al, 1945 ^890^ | | | 1 | 1 | | 273.2 - 273.2 | | | 130.0 - 130.0 |
| IBUTENE | Neduzhii Khmara, 1968 ^2^ | | | 18 | 18 | | 190.0 - 360.0 | | | 1.0 - 1423.0 |
| IBUTENE | Liessmann et al, 1995 ^5^ | | | 10 | 10 | | 243.2 - 333.2 | | | 39.0 - 786.0 |
| IBUTENE | Vargaftik, 1972 ^6^ | | | 9 | 9 | | 273.2 - 573.2 | | | 100.0 - 100.0 |
| IBUTENE | Scholz Kley, 1981 ^7^ | | | 28 | 25 | | 163.2 - 403.2 | | | 0.0 - 3147.0 |
| IBUTENE | Titani, 1930 ^9^ | | | 7 | 7 | | 273.2 - 393.1 | | | 101.0 - 101.0 |

| Material | | Author | *N*_all_ | | | *N*_used_ | | *T* / K | *p* / kPa | |
| --- | --- | --- | --- | --- | --- | --- | --- | --- | --- | --- |
| IHEXANE | Tatevskii et al, 1985 ^891^ | | | 2 | 2 | | 273.2 - 293.2 | | | 10.0 - 24.0 |
| IHEXANE | Thorpe Rodger, 1894 ^28^ | | | 12 | 12 | | 273.8 - 328.6 | | | 10.0 - 89.0 |
| IHEXANE | Neumann, 1995 ^19^ | | | 9 | 9 | | 283.0 - 323.0 | | | 6092.0 - 8842.0 |
| IHEXANE | Chavanne Van Risseghem, 1922 ^13^ | | | 5 | 5 | | 273.2 - 303.2 | | | 10.0 - 36.0 |
| IHEXANE | Aucejo et al, 1995 ^14^ | | | 1 | 1 | | 298.2 - 298.2 | | | 29.0 - 29.0 |
| IHEXANE | Kuss Pollmann, 1969 ^15^ | | | 6 | 6 | | 313.2 - 313.2 | | | 98.0 - 147100.0 |
| IHEXANE | Rappenecker, 1910 ^892^ | | | 2 | 2 | | 373.2 - 485.7 | | | 120.0 - 120.0 |
| IHEXANE | McCall et al, 1959 ^17^ | | | 1 | 1 | | 298.2 - 298.2 | | | 29.0 - 29.0 |
| IHEXANE | Diaz Pena et al, 1967 ^16^ | | | 13 | 13 | | 303.4 - 422.9 | | | 36.0 - 905.0 |
| IHEXANE | Wen et al, 2017 ^26^ | | | 49 | 49 | | 283.1 - 343.2 | | | 1000.0 - 30000.0 |
| IHEXANE | Liessmann et al, 1995 ^5^ | | | 11 | 11 | | 243.2 - 443.2 | | | 2.0 - 1305.0 |
| IHEXANE | Scholz Kley, 1981 ^7^ | | | 49 | 40 | | 213.2 - 483.2 | | | 0.0 - 2461.0 |
| IHEXANE | Ratkovics et al, 1974 ^250^ | | | 1 | 1 | | 293.2 - 293.2 | | | 24.0 - 24.0 |
| IHEXANE | Thorpe Rodger, 1894 ^28^ | | | 12 | 12 | | 273.8 - 328.6 | | | 10.0 - 88.0 |
| IHEXANE | Batschinski, 1913 ^29^ | | | 7 | 7 | | 273.1 - 333.1 | | | 10.0 - 102.0 |
| IHEXANE | Chavanne Van Risseghem, 1922 ^13^ | | | 5 | 5 | | 273.2 - 303.1 | | | 101.0 - 101.0 |
| IHEXANE | Ratkovics et al, 1974 ^250^ | | | 1 | 1 | | 293.1 - 293.1 | | | 101.0 - 101.0 |
| IHEXANE | Aucejo et al, 1995 ^14^ | | | 1 | 1 | | 298.0 - 298.0 | | | 101.0 - 101.0 |
| IHEXANE | Bouzas et al, 2000 ^25^ | | | 1 | 1 | | 298.2 - 298.2 | | | 101.0 - 101.0 |

| Material | | Author | *N*_all_ | | | *N*_used_ | | *T* / K | *p* / kPa | |
| --- | --- | --- | --- | --- | --- | --- | --- | --- | --- | --- |
| IOCTANE | Evans, 1938 ^276^ | | | 6 | 6 | | 278.2 - 363.1 | | | 3.0 - 79.0 |
| IOCTANE | Smyth Stoops, 1928 ^803^ | | | 1 | 1 | | 293.1 - 293.1 | | | 101.0 - 101.0 |
| IOCTANE | Bratton Felsing, 1936 ^893^ | | | 1 | 1 | | 363.1 - 363.1 | | | 101.0 - 101.0 |
| IOCTANE | Maman, 1938 ^894^ | | | 4 | 4 | | 278.2 - 293.1 | | | 101.0 - 101.0 |
| IOCTANE | Johnson Fawcett, 1946 ^895^ | | | 1 | 1 | | 293.1 - 293.1 | | | 101.0 - 101.0 |
| IOCTANE | Hammond Stokes, 1955 ^113^ | | | 1 | 1 | | 298.1 - 298.1 | | | 101.0 - 101.0 |
| IOCTANE | Awwad Allos, 1985 ^282^ | | | 1 | 1 | | 298.1 - 298.1 | | | 101.0 - 101.0 |
| IOCTANE | Dymond et al, 1985 ^896^ | | | 32 | 0 | | 10000.0 - 0.0 | | | 1000000000.0 - 0.0 |
| IOCTANE | Rao Reddy, 1988 ^162^ | | | 1 | 1 | | 298.1 - 298.1 | | | 101.0 - 101.0 |
| IOCTANE | Oswal Patel, 1992 ^177^ | | | 2 | 2 | | 303.2 - 313.2 | | | 101.0 - 101.0 |
| IOCTANE | Amimabhavi et al, 1994 ^815^ | | | 5 | 5 | | 298.2 - 318.2 | | | 101.0 - 101.0 |
| IOCTANE | Krahn Luft, 1994 ^555^ | | | 16 | 10 | | 298.2 - 453.2 | | | 100.0 - 120000.0 |
| IOCTANE | Amimabhavi Gopalakrishna, 1995 ^290^ | | | 4 | 4 | | 298.2 - 313.2 | | | 101.0 - 101.0 |
| IOCTANE | Padua et al, 1996 ^418^ | | | 8 | 8 | | 197.9 - 348.2 | | | 101.0 - 101.0 |
| IOCTANE | Padua et al, 1996 ^418^ | | | 87 | 83 | | 197.9 - 348.2 | | | 100.0 - 100100.0 |
| IOCTANE | Aralaguppi et al, 1999 ^189^ | | | 3 | 3 | | 298.2 - 308.2 | | | 101.0 - 101.0 |
| IOCTANE | Fermeglia Torriano, 1999 ^315^ | | | 1 | 1 | | 298.2 - 298.2 | | | 101.0 - 101.0 |
| IOCTANE | Bouzas et al, 2000 ^25^ | | | 1 | 1 | | 298.2 - 298.2 | | | 101.0 - 101.0 |
| IOCTANE | Gomez-Diaz et al, 2001 ^423^ | | | 6 | 6 | | 298.2 - 323.2 | | | 101.0 - 101.0 |
| IOCTANE | Gomez-Diaz et al, 2002 ^424^ | | | 1 | 1 | | 298.2 - 298.2 | | | 101.0 - 101.0 |
| IOCTANE | Peng Tu, 2002 ^61^ | | | 1 | 1 | | 298.2 - 298.2 | | | 101.0 - 101.0 |
| IOCTANE | Oswal et al, 2003 ^205^ | | | 1 | 1 | | 303.2 - 303.2 | | | 101.0 - 101.0 |
| IOCTANE | Ma et al, 2004 ^207^ | | | 14 | 14 | | 298.2 - 403.2 | | | 100.0 - 5100.0 |
| IOCTANE | Chen et al, 2005 ^65^ | | | 3 | 3 | | 288.2 - 308.2 | | | 101.0 - 101.0 |
| IOCTANE | Ku Tu, 2005 ^692^ | | | 4 | 4 | | 298.2 - 318.2 | | | 101.0 - 101.0 |
| IOCTANE | Ma Li, 2005 ^897^ | | | 1 | 1 | | 298.2 - 298.2 | | | 101.0 - 101.0 |
| IOCTANE | Wang et al, 2005 ^693^ | | | 4 | 4 | | 298.2 - 318.2 | | | 101.0 - 101.0 |
| IOCTANE | Ali et al, 2006 ^211^ | | | 1 | 1 | | 308.2 - 308.2 | | | 101.0 - 101.0 |
| IOCTANE | Baragi et al, 2006 ^213^ | | | 3 | 3 | | 298.2 - 308.2 | | | 101.0 - 101.0 |
| IOCTANE | Chen Tu, 2006 ^699^ | | | 3 | 3 | | 288.2 - 308.2 | | | 101.0 - 101.0 |
| IOCTANE | Salas et al, 2006 ^898^ | | | 1 | 1 | | 298.2 - 298.2 | | | 101.0 - 101.0 |
| IOCTANE | Suresh Reddy et al, 2006 ^215^ | | | 1 | 1 | | 303.2 - 303.2 | | | 101.0 - 101.0 |
| IOCTANE | Reddy et al, 2007 ^220^ | | | 1 | 1 | | 303.2 - 303.2 | | | 101.0 - 101.0 |
| IOCTANE | Ku et al, 2008 ^899^ | | | 4 | 4 | | 288.2 - 308.2 | | | 101.0 - 101.0 |
| IOCTANE | Ku et al, 2009 ^900^ | | | 3 | 3 | | 288.2 - 308.2 | | | 101.0 - 101.0 |
| IOCTANE | Kao Tu, 2011 ^901^ | | | 1 | 1 | | 298.2 - 298.2 | | | 101.0 - 101.0 |
| IOCTANE | Rafiee et al, 2012 ^902^ | | | 6 | 6 | | 288.2 - 313.2 | | | 101.0 - 101.0 |
| IOCTANE | Luning Prak et al, 2014 ^828^ | | | 8 | 8 | | 293.2 - 333.2 | | | 100.0 - 100.0 |
| IOCTANE | Yue et al, 2015 ^903^ | | | 5 | 5 | | 293.2 - 313.2 | | | 100.0 - 100.0 |
| IOCTANE | McCoubrey et al, 1951 ^856^ | | | 4 | 4 | | 374.8 - 467.1 | | | 101.0 - 101.0 |
| IOCTANE | Slusar et al, 1973 ^310^ | | | 4 | 4 | | 421.0 - 687.4 | | | 101.0 - 101.0 |
| IOCTANE | Diaz Pena, 1975 ^904^ | | | 11 | 4 | | 354.1 - 481.4 | | | 60.0 - 1036.0 |

| Material | | Author | *N*_all_ | | | *N*_used_ | | *T* / K | *p* / kPa | |
| --- | --- | --- | --- | --- | --- | --- | --- | --- | --- | --- |
| IPENTANE | Batschinski, 1913 ^29^ | | | 4 | 4 | | 273.1 - 303.1 | | | 35.0 - 109.0 |
| IPENTANE | Ledneva, 1956 ^800^ | | | 8 | 8 | | 293.1 - 433.1 | | | 77.0 - 2215.0 |
| IPENTANE | Ma et al, 2003 ^443^ | | | 21 | 21 | | 253.2 - 353.2 | | | 14.0 - 457.0 |
| IPENTANE | Thorpe Rodger, 1894 ^28^ | | | 25 | 1 | | 283.4 - 283.4 | | | 101.0 - 101.0 |
| IPENTANE | Thorpe Rodger, 1897 ^546^ | | | 18 | 17 | | 273.5 - 298.3 | | | 99.0 - 101.0 |
| IPENTANE | Timmermans Martin, 1926 ^131^ | | | 2 | 2 | | 273.2 - 288.1 | | | 101.0 - 101.0 |
| IPENTANE | Collings Mclaughlin, 1971 ^145^ | | | 12 | 8 | | 303.1 - 323.1 | | | 49020.0 - 196080.0 |
| IPENTANE | Ratkovics et al, 1974 ^250^ | | | 1 | 1 | | 293.1 - 293.1 | | | 101.0 - 101.0 |
| IPENTANE | Lesche et al, 1984 ^905^ | | | 20 | 19 | | 115.0 - 293.2 | | | 101.0 - 101.0 |
| IPENTANE | Rappenecker, 1910 ^892^ | | | 2 | 2 | | 373.1 - 485.7 | | | 101.0 - 101.0 |
| IPENTANE | Bleakney, 1932 ^906^ | | | 2 | 1 | | 373.1 - 373.1 | | | 101.0 - 101.0 |
| IPENTANE | Day, 1932 ^907^ | | | 37 | 37 | | 298.1 - 298.1 | | | 0.0 - 88.0 |
| IPENTANE | McCoubrey et al, 1951 ^856^ | | | 4 | 3 | | 341.1 - 453.1 | | | 101.0 - 101.0 |
| IPENTANE | Lambert et al, 1955 ^12^ | | | 4 | 4 | | 323.1 - 351.1 | | | 101.0 - 101.0 |
| IPENTANE | McCoubrey Singh, 1963 ^908^ | | | 5 | 5 | | 309.0 - 463.5 | | | 101.0 - 101.0 |

| Material | | Author | *N*_all_ | | | *N*_used_ | | *T* / K | *p* / kPa | |
| --- | --- | --- | --- | --- | --- | --- | --- | --- | --- | --- |
| ISOBUTAN | Golubev, 1959 ^8^ | | | 8 | 8 | | 203.2 - 273.2 | | | 5.0 - 158.0 |
| ISOBUTAN | Sage et al, 1939 ^248^ | | | 28 | 23 | | 310.9 - 360.9 | | | 1379.0 - 13790.0 |
| ISOBUTAN | Lipkin et al, 1942 ^259^ | | | 8 | 6 | | 199.9 - 255.4 | | | 101.0 - 101.0 |
| ISOBUTAN | Diller Van Poolen, 1985 ^262^ | | | 119 | 119 | | 120.0 - 300.0 | | | 414.0 - 34485.0 |
| ISOBUTAN | Kumagai et al, 2007 ^909^ | | | 16 | 16 | | 273.2 - 333.2 | | | 2000.0 - 30000.0 |
| ISOBUTAN | Sage et al, 1939 ^248^ | | | 27 | 27 | | 310.9 - 377.6 | | | 101.0 - 1034.0 |
| ISOBUTAN | Lambert et al, 1955 ^12^ | | | 8 | 8 | | 308.1 - 363.3 | | | 101.0 - 101.0 |
| ISOBUTAN | Gonzalez Lee, 1966 ^910^ | | | 5 | 5 | | 310.9 - 444.3 | | | 101.0 - 101.0 |
| ISOBUTAN | Agaev Yusibova, 1969 ^911^ | | | 11 | 11 | | 273.2 - 548.1 | | | 101.0 - 101.0 |
| ISOBUTAN | Abe et al, 1979 ^258^ | | | 7 | 7 | | 298.1 - 468.1 | | | 101.0 - 101.0 |
| ISOBUTAN | Diller Van Poolen, 1985 ^262^ | | | 32 | 30 | | 115.0 - 260.0 | | | 101.0 - 101.0 |
| ISOBUTAN | Kuechenmeinster Vogel, 2000 ^912^ | | | 84 | 84 | | 297.2 - 627.2 | | | 24.0 - 248.0 |

| Material | | Author | *N*_all_ | | | *N*_used_ | | *T* / K | *p* / kPa | |
| --- | --- | --- | --- | --- | --- | --- | --- | --- | --- | --- |
| KRYPTON | Kalelkar Kestin, 1970 ^95^ | | | 9 | 0 | | 10000.0 - 0.0 | | | 1000000000.0 - 0.0 |
| KRYPTON | Kestin et al, 1970 ^96^ | | | 8 | 8 | | 298.1 - 973.1 | | | 101.0 - 101.0 |
| KRYPTON | Kestin et al, 1970 ^96^ | | | 8 | 8 | | 298.1 - 973.1 | | | 101.0 - 101.0 |
| KRYPTON | Borisov et al, 1973 ^98^ | | | 1 | 1 | | 300.0 - 300.0 | | | 101.0 - 101.0 |
| KRYPTON | Kestin et al, 1977 ^256^ | | | 12 | 12 | | 298.1 - 773.1 | | | 101.0 - 101.0 |
| KRYPTON | Ulybin et al, 1978 ^913^ | | | 37 | 23 | | 210.0 - 300.0 | | | 8711.0 - 40910.0 |
| KRYPTON | Evers et al, 2002 ^104^ | | | 22 | 22 | | 298.2 - 348.2 | | | 99.0 - 8543.0 |
| KRYPTON | Wilhelm Vogel, 2000 ^107^ | | | 107 | 96 | | 298.0 - 348.0 | | | 0.0 - 16.0 |
| KRYPTON | Evers et al, 2002 ^104^ | | | 22 | 22 | | 298.2 - 348.2 | | | 98.0 - 8554.0 |

| Material | | Author | *N*_all_ | | | *N*_used_ | | *T* / K | *p* / kPa | |
| --- | --- | --- | --- | --- | --- | --- | --- | --- | --- | --- |
| MDM | Abbas et al, 2010 ^483^ | | | 16 | 11 | | 283.2 - 373.2 | | | 0.0 - 20.0 |
| MDM | Golik Cholpan, 1960 ^914^ | | | 1 | 1 | | 303.2 - 303.2 | | | 1.0 - 1.0 |
| MDM | Sperkach Cholpan, 1979 ^915^ | | | 3 | 1 | | 303.2 - 303.2 | | | 1.0 - 1.0 |
| MDM | Maczek Edwards, 1979 ^245^ | | | 2 | 0 | | 10000.0 - 0.0 | | | 1000000000.0 - 0.0 |

| Material | | Author | *N*_all_ | | | *N*_used_ | | *T* / K | *p* / kPa | |
| --- | --- | --- | --- | --- | --- | --- | --- | --- | --- | --- |
| MEA | Whorton Amis, 1956 ^488^ | | | 1 | 0 | | 10000.0 - 0.0 | | | 1000000000.0 - 0.0 |
| MEA | Matthews et al, 1950 ^916^ | | | 9 | 9 | | 293.1 - 313.1 | | | 101.0 - 101.0 |
| MEA | Anonymous, 1981 ^917^ | | | 10 | 6 | | 303.1 - 353.1 | | | 101.0 - 101.0 |
| MEA | DiGuilio et al, 1992 ^489^ | | | 8 | 4 | | 303.6 - 353.4 | | | 101.0 - 101.0 |
| MEA | Lee Lin, 1995 ^660^ | | | 3 | 3 | | 303.2 - 323.2 | | | 101.0 - 101.0 |
| MEA | Song et al, 1996 ^918^ | | | 5 | 5 | | 303.2 - 343.2 | | | 101.0 - 101.0 |
| MEA | lee et al, 1997 ^919^ | | | 3 | 3 | | 303.2 - 323.2 | | | 101.0 - 101.0 |
| MEA | Tsierkezos Molinou, 1999 ^59^ | | | 1 | 1 | | 293.2 - 293.2 | | | 101.0 - 101.0 |
| MEA | Maham et al, 2002 ^920^ | | | 5 | 5 | | 298.2 - 353.2 | | | 101.0 - 101.0 |
| MEA | Islam et al, 2004 ^921^ | | | 6 | 6 | | 298.2 - 323.2 | | | 101.0 - 101.0 |
| MEA | Aguila-Hernandez et al, 2008 ^318^ | | | 10 | 7 | | 303.2 - 363.2 | | | 101.0 - 101.0 |
| MEA | Amundsen et al, 2009 ^922^ | | | 5 | 5 | | 298.2 - 353.2 | | | 101.0 - 101.0 |
| MEA | Garcia Abuin et al, 2011 ^732^ | | | 4 | 4 | | 293.2 - 323.2 | | | 101.0 - 101.0 |
| MEA | Song et al, 2011 ^923^ | | | 3 | 3 | | 303.2 - 323.2 | | | 101.0 - 101.0 |
| MEA | Blanco et al, 2012 ^737^ | | | 1 | 1 | | 298.2 - 298.2 | | | 101.0 - 101.0 |
| MEA | Blanco et al, 2013 ^491^ | | | 4 | 4 | | 293.2 - 323.2 | | | 100.0 - 100.0 |
| MEA | Li et al, 2013 ^344^ | | | 5 | 5 | | 293.2 - 333.2 | | | 101.0 - 101.0 |
| MEA | Tian et al, 2013 ^924^ | | | 6 | 6 | | 303.2 - 328.2 | | | 101.0 - 101.0 |
| MEA | Shaikh et al, 2014 ^925^ | | | 5 | 5 | | 298.2 - 333.2 | | | 100.0 - 100.0 |
| MEA | Xu et al, 2014 ^926^ | | | 5 | 5 | | 293.2 - 333.2 | | | 101.0 - 101.0 |
| MEA | Shen et al, 2015 ^927^ | | | 5 | 5 | | 303.2 - 343.2 | | | 101.0 - 101.0 |

| Material | | Author | *N*_all_ | | *N*_used_ | | | *T* / K | | *p* / kPa |
| --- | --- | --- | --- | --- | --- | --- | --- | --- | --- | --- |
| METHANE | Rudenko Shubnikov, 1935 ^928^ | | | 5 | | 5 | 90.1 - 111.2 | | 9.0 - 88.0 | |
| METHANE | Rudenko, 1939 ^929^ | | | 6 | | 4 | 111.2 - 154.6 | | 88.0 - 1252.0 | |
| METHANE | Bresler Landerman, 1940 ^930^ | | | 1 | | 1 | 90.1 - 90.1 | | 9.0 - 9.0 | |
| METHANE | Gerf Galkov, 1940 ^615^ | | | 5 | | 5 | 94.4 - 111.2 | | 16.0 - 88.0 | |
| METHANE | Gerf Galkov, 1941 ^931^ | | | 5 | | 2 | 130.6 - 148.6 | | 360.0 - 952.0 | |
| METHANE | Vogel, 1914 ^80^ | | | 3 | | 2 | 194.8 - 273.1 | | 101.0 - 101.0 | |
| METHANE | Rankine Smith, 1921 ^777^ | | | 2 | | 2 | 290.1 - 373.1 | | 101.0 - 101.0 | |
| METHANE | Ishida, 1923 ^617^ | | | 1 | | 1 | 296.1 - 296.1 | | 101.0 - 101.0 | |
| METHANE | Adzumi, 1937 ^244^ | | | 9 | | 9 | 293.1 - 373.1 | | 101.0 - 101.0 | |
| METHANE | Mescheriaakov Golubev, 1954 ^932^ | | | 170 | | 170 | 258.2 - 523.2 | | 101.0 - 81060.0 | |
| METHANE | Ross Brown, 1957 ^933^ | | | 28 | | 28 | 223.0 - 298.1 | | 6996.0 - 69053.0 | |
| METHANE | Pavlovich et al, 1959 ^934^ | | | 29 | | 29 | 191.1 - 323.1 | | 1960.0 - 19610.0 | |
| METHANE | Baron et al, 1959 ^620^ | | | 40 | | 40 | 324.8 - 408.1 | | 689.0 - 55158.0 | |
| METHANE | Iwasaki Takahashi, 1959 ^935^ | | | 48 | | 48 | 298.1 - 348.1 | | 101.0 - 51068.0 | |
| METHANE | Barua et al, 1964 ^462^ | | | 39 | | 39 | 223.1 - 423.1 | | 1013.0 - 17755.0 | |
| METHANE | Carmichael et al, 1965 ^936^ | | | 173 | | 173 | 277.6 - 477.6 | | 101.0 - 35671.0 | |
| METHANE | Huang et al, 1966 ^937^ | | | 59 | | 59 | 113.2 - 273.2 | | 101.0 - 34471.0 | |
| METHANE | Hellemans et al, 1973 ^938^ | | | 5 | | 5 | 298.1 - 468.1 | | 101.0 - 101.0 | |
| METHANE | Diaz Pena et al, 1975 ^904^ | | | 9 | | 9 | 303.1 - 403.1 | | 27.0 - 27.0 | |
| METHANE | Kestin et al, 1976 ^393^ | | | 3 | | 3 | 296.1 - 477.1 | | 101.0 - 101.0 | |
| METHANE | Kestin et al, 1977 ^256^ | | | 8 | | 8 | 298.1 - 473.1 | | 101.0 - 101.0 | |
| METHANE | Kestin et al, 1977 ^256^ | | | 5 | | 5 | 298.1 - 473.1 | | 101.0 - 101.0 | |
| METHANE | Evers et al, 2002 ^104^ | | | 61 | | 61 | 233.2 - 523.2 | | 127.0 - 29674.0 | |
| METHANE | Hurly et al, 2003 ^105^ | | | 26 | | 26 | 293.2 - 293.2 | | 118.0 - 3324.0 | |
| METHANE | Bresler Landerman, 1940 ^930^ | | | 1 | | 1 | 90.1 - 90.1 | | 100.0 - 100.0 | |
| METHANE | Pavlovich, 1958 ^934^ | | | 4 | | 3 | 112.2 - 112.2 | | 1960.0 - 14710.0 | |
| METHANE | Swift et al, 1959 ^939^ | | | 24 | | 18 | 123.2 - 197.2 | | 2410.0 - 4900.0 | |
| METHANE | Swift et al, 1960 ^940^ | | | 10 | | 9 | 133.2 - 191.0 | | 586.0 - 4650.0 | |
| METHANE | Golubev Agaev, 1963 ^261^ | | | 2 | | 0 | 10000.0 - 0.0 | | 1000000000.0 - 0.0 | |
| METHANE | Giddings et al, 1966 ^941^ | | | 100 | | 100 | 283.1 - 410.9 | | 101.0 - 55158.0 | |
| METHANE | Huang et al, 1966 ^937^ | | | 43 | | 43 | 103.2 - 173.2 | | 101.0 - 34471.0 | |
| METHANE | Hellemans et al, 1970 ^91^ | | | 56 | | 55 | 96.8 - 187.3 | | 41.0 - 10122.0 | |
| METHANE | Hongo et al, 1988 ^942^ | | | 32 | | 32 | 298.1 - 373.1 | | 101.0 - 4988.0 | |
| METHANE | Chuang et al, 1976 ^881^ | | | 36 | | 36 | 173.2 - 273.2 | | 406.0 - 50580.0 | |
| METHANE | VanderGulik et al, 1992 ^943^ | | | 125 | | 115 | 273.1 - 273.1 | | 1062.0 - 701500.0 | |
| METHANE | Evers et al, 2002 ^104^ | | | 61 | | 61 | 233.2 - 523.2 | | 126.0 - 30066.0 | |
| METHANE | Vogel, 2011 ^787^ | | | 35 | | 35 | 289.1 - 682.1 | | 52.0 - 176.0 | |

| Material | | Author | *N*_all_ | | *N*_used_ | | | *T* / K | | *p* / kPa |
| --- | --- | --- | --- | --- | --- | --- | --- | --- | --- | --- |
| METHANOL | Thorpe Rodger, 1894 ^28^ | | | 13 | | 13 | 276.9 - 336.4 | | 4.0 - 89.0 | |
| METHANOL | Lewis, 1925 ^36^ | | | 1 | | 1 | 298.1 - 298.1 | | 14.0 - 14.0 | |
| METHANOL | Ledneva, 1956 ^800^ | | | 16 | | 16 | 293.1 - 443.1 | | 11.0 - 2146.0 | |
| METHANOL | Bingham et al, 1913 ^633^ | | | 8 | | 8 | 298.1 - 328.1 | | 101.0 - 101.0 | |
| METHANOL | Weissenberger Schuster, 1924 ^35^ | | | 1 | | 1 | 293.1 - 293.1 | | 101.0 - 101.0 | |
| METHANOL | Jones Fornwalt, 1938 ^944^ | | | 1 | | 1 | 298.1 - 298.1 | | 101.0 - 101.0 | |
| METHANOL | Khalilov, 1939 ^112^ | | | 16 | | 5 | 293.1 - 333.1 | | 101.0 - 101.0 | |
| METHANOL | Sadek Fuoss, 1950 ^945^ | | | 1 | | 1 | 298.1 - 298.1 | | 101.0 - 101.0 | |
| METHANOL | Teitel baum et al, 1950 ^140^ | | | 1 | | 1 | 293.1 - 293.1 | | 101.0 - 101.0 | |
| METHANOL | Subnis, 1952 ^141^ | | | 3 | | 3 | 303.1 - 323.1 | | 101.0 - 101.0 | |
| METHANOL | Hammond Stokes, 1955 ^113^ | | | 1 | | 1 | 298.1 - 298.1 | | 101.0 - 101.0 | |
| METHANOL | Ling Van Winkle, 1958 ^43^ | | | 2 | | 2 | 303.1 - 328.1 | | 101.0 - 101.0 | |
| METHANOL | Mikhail Kimel, 1961 ^946^ | | | 5 | | 5 | 298.1 - 323.1 | | 101.0 - 101.0 | |
| METHANOL | Bamelis et al, 1965 ^637^ | | | 4 | | 4 | 298.1 - 328.1 | | 101.0 - 101.0 | |
| METHANOL | Isakova Oshueva, 1966 ^947^ | | | 47 | | 43 | 293.1 - 433.1 | | 98.0 - 24517.0 | |
| METHANOL | Katti et al, 1966 ^143^ | | | 1 | | 1 | 313.1 - 313.1 | | 101.0 - 101.0 | |
| METHANOL | Komarenko et al, 1967 ^638^ | | | 13 | | 13 | 176.2 - 298.1 | | 101.0 - 101.0 | |
| METHANOL | Tommila et al, 1969 ^639^ | | | 4 | | 4 | 298.1 - 323.1 | | 101.0 - 101.0 | |
| METHANOL | Konobeev Lyapin, 1970 ^45^ | | | 3 | | 3 | 293.1 - 333.1 | | 101.0 - 101.0 | |
| METHANOL | Campbell Kartzmark, 1973 ^46^ | | | 2 | | 2 | 298.1 - 298.1 | | 101.0 - 101.0 | |
| METHANOL | Ratkovics et al, 1974 ^250^ | | | 1 | | 1 | 293.1 - 293.1 | | 101.0 - 101.0 | |
| METHANOL | Lee et al, 1976 ^642^ | | | 6 | | 6 | 288.2 - 323.1 | | 101.0 - 101.0 | |
| METHANOL | Kubota et al, 1979 ^948^ | | | 31 | | 31 | 283.2 - 348.1 | | 100.0 - 68750.0 | |
| METHANOL | Baykut Tanrikulu, 1980 ^644^ | | | 2 | | 2 | 293.1 - 293.1 | | 101.0 - 101.0 | |
| METHANOL | Kumar et al, 1981 ^646^ | | | 1 | | 1 | 298.1 - 298.1 | | 101.0 - 101.0 | |
| METHANOL | Singh Haque, 1981 ^949^ | | | 1 | | 1 | 303.1 - 303.1 | | 101.0 - 101.0 | |
| METHANOL | Noda et al, 1982 ^52^ | | | 1 | | 1 | 298.1 - 298.1 | | 101.0 - 101.0 | |
| METHANOL | Pikkarainen, 1983 ^647^ | | | 1 | | 1 | 303.1 - 303.1 | | 101.0 - 101.0 | |
| METHANOL | Rauf et al, 1983 ^649^ | | | 5 | | 5 | 288.2 - 328.1 | | 101.0 - 101.0 | |
| METHANOL | Isdale et al, 1985 ^950^ | | | 44 | | 35 | 298.1 - 323.1 | | 101.0 - 366400.0 | |
| METHANOL | Craubner, 1986 ^841^ | | | 1 | | 1 | 293.1 - 293.1 | | 101.0 - 101.0 | |
| METHANOL | Manjeshwar Aminabhavi, 1987 ^159^ | | | 1 | | 1 | 318.0 - 318.0 | | 101.0 - 101.0 | |
| METHANOL | Tanaka et al, 1987 ^651^ | | | 31 | | 31 | 283.2 - 348.1 | | 100.0 - 68800.0 | |
| METHANOL | Manjeshwar Aminabhavi, 1988 ^408^ | | | 1 | | 1 | 298.1 - 298.1 | | 101.0 - 101.0 | |
| METHANOL | Joshi et al, 1990 ^168^ | | | 4 | | 4 | 298.2 - 313.2 | | 101.0 - 101.0 | |
| METHANOL | Crabtree O’Brien, 1991 ^652^ | | | 3 | | 3 | 303.0 - 319.0 | | 101.0 - 101.0 | |
| METHANOL | Das Jha, 1991 ^55^ | | | 1 | | 0 | 10000.0 - 0.0 | | 1000000000.0 - 0.0 | |
| METHANOL | Garcia et al, 1991 ^653^ | | | 4 | | 4 | 298.2 - 323.2 | | 101.0 - 101.0 | |
| METHANOL | 1991 ramakanth et al, 1991 ^174^ | | | 1 | | 1 | 293.2 - 293.2 | | 101.0 - 101.0 | |
| METHANOL | Papanastasiou Ziogas, 1992 ^410^ | | | 5 | | 5 | 288.2 - 308.2 | | 101.0 - 101.0 | |
| METHANOL | Pikkaranien, 1992 ^654^ | | | 1 | | 1 | 303.2 - 303.2 | | 101.0 - 101.0 | |
| METHANOL | Aminabhavi et al, 1993 ^655^ | | | 3 | | 3 | 298.2 - 308.2 | | 101.0 - 101.0 | |
| METHANOL | Papaioannou et al, 1993 ^503^ | | | 11 | | 11 | 298.2 - 298.2 | | 100.0 - 71750.0 | |
| METHANOL | Shastri et al, 1993 ^179^ | | | 1 | | 1 | 293.2 - 293.2 | | 101.0 - 101.0 | |
| METHANOL | Assael et al, 1994 ^656^ | | | 26 | | 26 | 295.0 - 323.2 | | 100.0 - 27050.0 | |
| METHANOL | Liu et al, 1994 ^951^ | | | 4 | | 4 | 293.2 - 323.2 | | 101.0 - 101.0 | |
| METHANOL | Nikam et al, 1995 ^661^ | | | 2 | | 2 | 298.2 - 303.2 | | 101.0 - 101.0 | |
| METHANOL | Papaioannou Panayioton, 1995 ^662^ | | | 12 | | 12 | 298.2 - 298.2 | | 100.0 - 51800.0 | |
| METHANOL | Reddy et al, 1995 ^952^ | | | 1 | | 1 | 308.2 - 308.2 | | 101.0 - 101.0 | |
| METHANOL | Rived et al, 1995 ^183^ | | | 1 | | 1 | 303.2 - 303.2 | | 101.0 - 101.0 | |
| METHANOL | Saha et al, 1995 ^953^ | | | 3 | | 3 | 298.2 - 318.2 | | 101.0 - 101.0 | |
| METHANOL | Sastry Raj, 1995 ^954^ | | | 2 | | 2 | 303.2 - 313.2 | | 101.0 - 101.0 | |
| METHANOL | Aucejo et al, 1996 ^663^ | | | 1 | | 1 | 298.2 - 298.2 | | 101.0 - 101.0 | |
| METHANOL | Aalto Liukkonen, 1996 ^664^ | | | 1 | | 1 | 303.2 - 303.2 | | 101.0 - 101.0 | |
| METHANOL | Muhuri et al, 1996 ^955^ | | | 3 | | 3 | 298.2 - 318.2 | | 101.0 - 101.0 | |
| METHANOL | Nikam et al, 1996 ^665^ | | | 2 | | 2 | 298.2 - 303.2 | | 101.0 - 101.0 | |
| METHANOL | Nikam et al, 1996 ^666^ | | | 1 | | 1 | 298.2 - 298.2 | | 101.0 - 101.0 | |
| METHANOL | Bakshi Kaur, 1997 ^956^ | | | 1 | | 1 | 298.0 - 298.0 | | 101.0 - 101.0 | |
| METHANOL | Orge et al, 1997 ^667^ | | | 1 | | 1 | 298.2 - 298.2 | | 101.0 - 101.0 | |
| METHANOL | Aminabhavi Banerjee, 1998 ^419^ | | | 3 | | 3 | 298.2 - 308.2 | | 101.0 - 101.0 | |
| METHANOL | Aminabhavi Banerjee, 1998 ^419^ | | | 3 | | 3 | 298.2 - 308.2 | | 101.0 - 101.0 | |
| METHANOL | Aminabhavi patil, 1998 ^668^ | | | 3 | | 3 | 298.2 - 308.2 | | 101.0 - 101.0 | |
| METHANOL | Canosa et al, 1998 ^669^ | | | 1 | | 1 | 298.2 - 298.2 | | 101.0 - 101.0 | |
| METHANOL | Canosa et al, 1998^669^ | | | 1 | | 1 | 298.2 - 298.2 | | 101.0 - 101.0 | |
| METHANOL | George et al, 1998 ^957^ | | | 2 | | 2 | 293.2 - 293.2 | | 101.0 - 101.0 | |
| METHANOL | Kumagai Yokoyama, 1998 ^670^ | | | 4 | | 4 | 273.2 - 333.2 | | 101.0 - 101.0 | |
| METHANOL | Nikam et al, 1998 ^671^ | | | 4 | | 4 | 298.2 - 313.2 | | 101.0 - 101.0 | |
| METHANOL | Rodriguez et al, 1998 ^958^ | | | 1 | | 1 | 298.2 - 298.2 | | 101.0 - 101.0 | |
| METHANOL | Sastry Valand, 1998 ^959^ | | | 1 | | 1 | 298.2 - 298.2 | | 101.0 - 101.0 | |
| METHANOL | Aminabhavi et al, 1999^673^ | | | 3 | | 3 | 298.2 - 308.2 | | 101.0 - 101.0 | |
| METHANOL | Aralaguppi et al, 1999 ^189^ | | | 3 | | 3 | 298.2 - 308.2 | | 101.0 - 101.0 | |
| METHANOL | Haraschta et al, 1999 ^674^ | | | 1 | | 1 | 298.2 - 298.2 | | 101.0 - 101.0 | |
| METHANOL | Pal et al, 1999 ^529^ | | | 1 | | 1 | 298.2 - 298.2 | | 101.0 - 101.0 | |
| METHANOL | Prasad et al, 1999 ^676^ | | | 4 | | 4 | 293.2 - 323.2 | | 101.0 - 101.0 | |
| METHANOL | Tsierkozos Molinou, 1999 ^59^ | | | 1 | | 1 | 293.2 - 293.2 | | 101.0 - 101.0 | |
| METHANOL | Canosa et al, 2000 ^960^ | | | 1 | | 1 | 298.2 - 298.2 | | 101.0 - 101.0 | |
| METHANOL | Canosa et al, 2000 ^960^ | | | 1 | | 1 | 298.2 - 298.2 | | 101.0 - 101.0 | |
| METHANOL | Nikam et al, 2000 ^678^ | | | 2 | | 2 | 303.2 - 308.2 | | 101.0 - 101.0 | |
| METHANOL | Nikam et al, 2000 ^678^ | | | 2 | | 2 | 303.2 - 308.2 | | 101.0 - 101.0 | |
| METHANOL | Pal Dass, 2000 ^961^ | | | 1 | | 1 | 298.2 - 298.2 | | 101.0 - 101.0 | |
| METHANOL | Prasad et al, 2000 ^962^ | | | 4 | | 4 | 293.2 - 323.2 | | 101.0 - 101.0 | |
| METHANOL | Prasad et al, 2000 ^963^ | | | 4 | | 4 | 293.2 - 323.2 | | 101.0 - 101.0 | |
| METHANOL | Saha Das, 2000 ^964^ | | | 1 | | 1 | 298.2 - 298.2 | | 101.0 - 101.0 | |
| METHANOL | Tu et al, 2000 ^680^ | | | 4 | | 4 | 293.2 - 313.2 | | 101.0 - 101.0 | |
| METHANOL | Visak et al, 2000 ^965^ | | | 1 | | 1 | 303.2 - 303.2 | | 101.0 - 101.0 | |
| METHANOL | Tu et al, 2001 ^683^ | | | 4 | | 4 | 293.2 - 313.2 | | 101.0 - 101.0 | |
| METHANOL | George et al, 2002 ^966^ | | | 2 | | 2 | 308.2 - 318.2 | | 101.0 - 101.0 | |
| METHANOL | Heintz et al, 2002 ^967^ | | | 3 | | 3 | 298.2 - 323.2 | | 101.0 - 101.0 | |
| METHANOL | Prasad et al, 2002 ^684^ | | | 3 | | 3 | 293.2 - 313.2 | | 101.0 - 101.0 | |
| METHANOL | Giro et al, 2003 ^968^ | | | 1 | | 1 | 303.2 - 303.2 | | 101.0 - 101.0 | |
| METHANOL | Govindarajan et al, 2003 ^62^ | | | 5 | | 5 | 293.2 - 313.2 | | 101.0 - 101.0 | |
| METHANOL | Kijevcanin et al, 2003 ^969^ | | | 1 | | 1 | 303.2 - 303.2 | | 101.0 - 101.0 | |
| METHANOL | Agarwal Singh, 2004 ^63^ | | | 1 | | 1 | 298.2 - 298.2 | | 101.0 - 101.0 | |
| METHANOL | Chen et al, 2004^686^ | | | 3 | | 3 | 288.2 - 308.2 | | 101.0 - 101.0 | |
| METHANOL | Gonzalez et al, 2004 ^298^ | | | 3 | | 3 | 293.2 - 303.2 | | 101.0 - 101.0 | |
| METHANOL | Li et al, 2004 ^375^ | | | 1 | | 1 | 298.2 - 298.2 | | 101.0 - 101.0 | |
| METHANOL | Rodriguez et al, 2004 ^533^ | | | 4 | | 4 | 293.2 - 313.2 | | 101.0 - 101.0 | |
| METHANOL | Rodriguez et al, 2004 ^533^ | | | 4 | | 4 | 293.2 - 313.2 | | 101.0 - 101.0 | |
| METHANOL | Saleh et al, 2004 ^688^ | | | 5 | | 5 | 303.2 - 323.2 | | 101.0 - 101.0 | |
| METHANOL | Chen et al, 2005 ^65^ | | | 3 | | 3 | 293.2 - 303.2 | | 101.0 - 101.0 | |
| METHANOL | Djojoputro ismadji, 2005 ^690^ | | | 3 | | 3 | 293.2 - 313.2 | | 101.0 - 101.0 | |
| METHANOL | Gonzalez et al, 2005 ^66^ | | | 1 | | 1 | 298.2 - 298.2 | | 100.0 - 100.0 | |
| METHANOL | Zeberg-Mikkelsen et al, 2005 ^695^ | | | 23 | | 23 | 293.2 - 353.2 | | 100.0 - 100000.0 | |
| METHANOL | Arce et al, 2006 ^697^ | | | 1 | | 1 | 298.2 - 298.2 | | 101.0 - 101.0 | |
| METHANOL | Arce et al, 2006 ^697^ | | | 1 | | 1 | 298.2 - 298.2 | | 101.0 - 101.0 | |
| METHANOL | Chatterjee Das, 2006 ^970^ | | | 3 | | 3 | 298.2 - 318.2 | | 101.0 - 101.0 | |
| METHANOL | Gonzalez et al, 2006 ^68^ | | | 3 | | 3 | 298.2 - 328.2 | | 101.0 - 101.0 | |
| METHANOL | Han et al, 2006 ^214^ | | | 1 | | 1 | 298.2 - 298.2 | | 101.0 - 101.0 | |
| METHANOL | Kadam et al, 2006 ^701^ | | | 2 | | 2 | 298.2 - 308.2 | | 101.0 - 101.0 | |
| METHANOL | Rodriguez et al, 2006 ^536^ | | | 4 | | 4 | 293.2 - 313.2 | | 101.0 - 101.0 | |
| METHANOL | Roy Sinha, 2006 ^703^ | | | 1 | | 1 | 303.2 - 303.2 | | 101.0 - 101.0 | |
| METHANOL | Yang et al, 2006 ^588^ | | | 6 | | 6 | 293.2 - 333.2 | | 101.0 - 101.0 | |
| METHANOL | Yang et al, 2006 ^588^ | | | 5 | | 5 | 308.2 - 328.2 | | 101.0 - 101.0 | |
| METHANOL | Yang et al, 2006 ^589^ | | | 5 | | 5 | 308.2 - 328.2 | | 101.0 - 101.0 | |
| METHANOL | Bhuiyan et al, 2007 ^377^ | | | 5 | | 5 | 303.2 - 323.2 | | 101.0 - 101.0 | |
| METHANOL | Gonzalez et al, 2007 ^430^ | | | 3 | | 3 | 293.2 - 303.2 | | 101.0 - 101.0 | |
| METHANOL | Gonzalez et al, 2007 ^430^ | | | 3 | | 3 | 298.2 - 328.2 | | 101.0 - 101.0 | |
| METHANOL | Gurung Roy, 2007 ^590^ | | | 3 | | 3 | 298.2 - 318.2 | | 101.0 - 101.0 | |
| METHANOL | Song et al, 2007 ^592^ | | | 1 | | 1 | 293.2 - 293.2 | | 101.0 - 101.0 | |
| METHANOL | Zafarani Majdan, 2007 ^971^ | | | 1 | | 1 | 298.2 - 298.2 | | 101.0 - 101.0 | |
| METHANOL | Fan et al, 2008 ^431^ | | | 11 | | 11 | 283.2 - 333.2 | | 101.0 - 101.0 | |
| METHANOL | Pal Gaba, 2008 ^972^ | | | 1 | | 1 | 298.2 - 298.2 | | 101.0 - 101.0 | |
| METHANOL | Shukla et al, 2008 ^381^ | | | 3 | | 3 | 293.0 - 313.0 | | 101.0 - 101.0 | |
| METHANOL | Yang et al, 2008 ^716^ | | | 5 | | 5 | 293.2 - 323.2 | | 101.0 - 101.0 | |
| METHANOL | Davila et al, 2009 ^973^ | | | 1 | | 1 | 298.2 - 298.2 | | 101.0 - 101.0 | |
| METHANOL | Domanska Laskowska, 2009 ^718^ | | | 4 | | 4 | 298.2 - 328.2 | | 101.0 - 101.0 | |
| METHANOL | Jain et al, 2009 ^974^ | | | 3 | | 3 | 303.2 - 323.2 | | 101.0 - 101.0 | |
| METHANOL | Roy et al, 2009 ^721^ | | | 3 | | 3 | 298.2 - 318.2 | | 101.0 - 101.0 | |
| METHANOL | Tsierkezos Molinou, 2009 ^722^ | | | 1 | | 1 | 293.2 - 293.2 | | 101.0 - 101.0 | |
| METHANOL | Yan et al, 2009 ^232^ | | | 4 | | 4 | 298.2 - 328.2 | | 101.0 - 101.0 | |
| METHANOL | Zarei et al, 2009 ^724^ | | | 1 | | 1 | 303.2 - 303.2 | | 82.0 - 82.0 | |
| METHANOL | Bhattacharjee Roy, 2010 ^727^ | | | 1 | | 1 | 298.2 - 298.2 | | 101.0 - 101.0 | |
| METHANOL | Hou et al, 2010 ^730^ | | | 8 | | 8 | 278.2 - 313.2 | | 101.0 - 101.0 | |
| METHANOL | Roy et al, 2010 ^975^ | | | 1 | | 1 | 303.2 - 303.2 | | 101.0 - 101.0 | |
| METHANOL | Shekaari Mousavi, 2010 ^976^ | | | 4 | | 1 | 328.2 - 328.2 | | 101.0 - 101.0 | |
| METHANOL | Kurnia Mutalib, 2011 ^734^ | | | 4 | | 4 | 293.2 - 323.2 | | 101.0 - 101.0 | |
| METHANOL | Kurnia Mutalib, 2011 ^734^ | | | 5 | | 5 | 293.2 - 323.2 | | 101.0 - 101.0 | |
| METHANOL | Pal et al, 2011 ^977^ | | | 1 | | 1 | 298.2 - 298.2 | | 101.0 - 101.0 | |
| METHANOL | Pradhan Roy, 2011 ^236^ | | | 1 | | 1 | 298.2 - 298.2 | | 101.0 - 101.0 | |
| METHANOL | Wang tian, 2011 ^978^ | | | 7 | | 7 | 293.2 - 333.2 | | 101.0 - 101.0 | |
| METHANOL | Warrier Teja, 2011 ^979^ | | | 1 | | 1 | 297.8 - 297.8 | | 101.0 - 101.0 | |
| METHANOL | Yu et al, 2011 ^980^ | | | 4 | | 4 | 298.2 - 313.2 | | 101.0 - 101.0 | |
| METHANOL | Dash et al, 2012 ^524^ | | | 1 | | 1 | 303.2 - 303.2 | | 101.0 - 101.0 | |
| METHANOL | Garcia-Mardones et al, 2012 ^739^ | | | 4 | | 4 | 293.2 - 323.2 | | 101.0 - 101.0 | |
| METHANOL | Gong et al, 2012 ^740^ | | | 9 | | 9 | 293.2 - 333.2 | | 101.0 - 101.0 | |
| METHANOL | Marczak et al, 2012 ^981^ | | | 7 | | 7 | 293.2 - 318.3 | | 101.0 - 101.0 | |
| METHANOL | Qian et al, 2012 ^982^ | | | 5 | | 5 | 293.2 - 313.2 | | 101.0 - 101.0 | |
| METHANOL | Shao et al, 2012 ^743^ | | | 4 | | 4 | 298.2 - 313.2 | | 101.0 - 101.0 | |
| METHANOL | Singh et al, 2012 ^983^ | | | 3 | | 3 | 293.2 - 313.2 | | 101.0 - 101.0 | |
| METHANOL | Xu et al, 2012 ^745^ | | | 5 | | 5 | 293.2 - 313.2 | | 101.0 - 101.0 | |
| METHANOL | Yu et al, 2012 ^984^ | | | 4 | | 4 | 298.2 - 313.2 | | 101.0 - 101.0 | |
| METHANOL | Roy et al, 2013 ^721^ | | | 1 | | 1 | 298.2 - 298.2 | | 101.0 - 101.0 | |
| METHANOL | Brahman Sinha, 2013 ^985^ | | | 3 | | 3 | 298.2 - 318.2 | | 101.0 - 101.0 | |
| METHANOL | Gupta et al, 2013 ^986^ | | | 3 | | 3 | 308.2 - 318.2 | | 101.0 - 101.0 | |
| METHANOL | Jain et al, 2013 ^987^ | | | 1 | | 1 | 298.2 - 298.2 | | 101.0 - 101.0 | |
| METHANOL | Kondaiah et al, 2013 ^749^ | | | 3 | | 3 | 303.2 - 323.2 | | 101.0 - 101.0 | |
| METHANOL | Xu et al, 2013 ^750^ | | | 5 | | 5 | 293.2 - 313.2 | | 101.0 - 101.0 | |
| METHANOL | Yang et al, 2013 ^751^ | | | 4 | | 4 | 293.2 - 308.2 | | 101.0 - 101.0 | |
| METHANOL | Brahman Sinha, 2014 ^988^ | | | 3 | | 3 | 298.2 - 318.2 | | 101.0 - 101.0 | |
| METHANOL | Brahman Sinha, 2014 ^988^ | | | 3 | | 3 | 298.2 - 318.2 | | 101.0 - 101.0 | |
| METHANOL | Garcia-Mardones, 2014 ^989^ | | | 4 | | 4 | 293.2 - 323.2 | | 101.0 - 101.0 | |
| METHANOL | Hammami et al, 2014 ^441^ | | | 3 | | 3 | 313.2 - 323.2 | | 101.0 - 101.0 | |
| METHANOL | Zhang et al, 2014 ^611^ | | | 7 | | 7 | 293.2 - 323.2 | | 101.0 - 101.0 | |
| METHANOL | Cao et al, 2015 ^757^ | | | 1 | | 1 | 298.2 - 298.2 | | 101.0 - 101.0 | |
| METHANOL | Wu et al, 2015 ^990^ | | | 4 | | 4 | 303.2 - 333.2 | | 101.0 - 101.0 | |
| METHANOL | Titani, 1933 ^75^ | | | 7 | | 7 | 384.4 - 584.7 | | 101.0 - 101.0 | |
| METHANOL | Khalilov, 1939 ^112^ | | | 22 | | 18 | 343.1 - 507.2 | | 101.0 - 101.0 | |
| METHANOL | Ling Van Winkle, 1958 ^43^ | | | 3 | | 3 | 383.1 - 423.1 | | 101.0 - 101.0 | |
| METHANOL | Reid Belenyessy, 1960 ^759^ | | | 1 | | 1 | 423.1 - 423.1 | | 101.0 - 101.0 | |
| METHANOL | Vogel et al, 1986 ^246^ | | | 51 | | 0 | 10000.0 - 0.0 | | 1000000000.0 - 0.0 | |
| METHANOL | Teske Vogel, 2006 ^991^ | | | 165 | | 0 | 10000.0 - 0.0 | | 1000000000.0 - 0.0 | |

| Material | | Author | *N*_all_ | | *N*_used_ | | | *T* / K | | *p* / kPa |
| --- | --- | --- | --- | --- | --- | --- | --- | --- | --- | --- |
| MLINOLEA | Ceriani et al, 2007 ^992^ | | | 6 | | 6 | 293.2 - 343.2 | | 101.0 - 101.0 | |
| MLINOLEA | Pratas et al, 2010 ^993^ | | | 16 | | 16 | 278.2 - 353.2 | | 101.0 - 101.0 | |
| MLINOLEN | Pratas et al, 2011 ^994^ | | | 20 | | 0 | 10000.0 - 0.0 | | 1000000000.0 - 0.0 | |
| MLINOLEN | Treibs, 1942 ^995^ | | | 1 | | 0 | 10000.0 - 0.0 | | 1000000000.0 - 0.0 | |
| MLINOLEN | Allen et al, 1999 ^996^ | | | 1 | | 0 | 10000.0 - 0.0 | | 1000000000.0 - 0.0 | |
| MLINOLEN | Ceriani et al, 2007 ^992^ | | | 12 | | 6 | 293.2 - 343.2 | | 101.0 - 101.0 | |
| MLINOLEN | Pratas et al, 2011 ^994^ | | | 20 | | 18 | 278.2 - 363.2 | | 101.0 - 101.0 | |

| Material | | Author | *N*_all_ | | *N*_used_ | | | *T* / K | | *p* / kPa |
| --- | --- | --- | --- | --- | --- | --- | --- | --- | --- | --- |
| MM | Gubareva, 1983 ^997^ | | | 8 | | 8 | 293.2 - 353.2 | | 5.0 - 54.0 | |
| MM | Golik Cholpan, 1960 ^914^ | | | 1 | | 1 | 303.2 - 303.2 | | 8.0 - 8.0 | |
| MM | Abbas et al, 2010 ^483^ | | | 7 | | 7 | 273.2 - 323.2 | | 2.0 - 18.0 | |
| MM | Dickinson, 1977 ^482^ | | | 5 | | 3 | 303.2 - 323.2 | | 50500.0 - 104500.0 | |
| MM | Maczek Edwards, 1979 ^245^ | | | 2 | | 0 | 10000.0 - 0.0 | | 1000000000.0 - 0.0 | |

| Material | | Author | *N*_all_ | | *N*_used_ | | | *T* / K | | *p* / kPa |
| --- | --- | --- | --- | --- | --- | --- | --- | --- | --- | --- |
| MOLEATE | Keffler McLean, 1935 ^998^ | | | 3 | | 0 | 10000.0 - 0.0 | | 1000000000.0 - 0.0 | |
| MOLEATE | Albert, 1938 ^999^ | | | 4 | | 0 | 10000.0 - 0.0 | | 1000000000.0 - 0.0 | |
| MOLEATE | Allen et al, 1999 ^996^ | | | 1 | | 0 | 10000.0 - 0.0 | | 1000000000.0 - 0.0 | |
| MOLEATE | Menzel et al, 1949 ^1000^ | | | 2 | | 0 | 10000.0 - 0.0 | | 1000000000.0 - 0.0 | |
| MOLEATE | Yamawaki, 2020 ^1001^ | | | 119 | | 0 | 10000.0 - 0.0 | | 1000000000.0 - 0.0 | |
| MOLEATE | Keffler McLean, 1935 ^1002^ | | | 3 | | 3 | 303.1 - 363.1 | | 101.0 - 101.0 | |
| MOLEATE | Albert, 1938 ^999^ | | | 4 | | 3 | 293.1 - 373.1 | | 101.0 - 101.0 | |
| MOLEATE | Bhuyan et al, 2006 ^1003^ | | | 1 | | 1 | 343.2 - 343.2 | | 101.0 - 101.0 | |
| MOLEATE | Ceriani et al, 2007 ^992^ | | | 6 | | 6 | 293.2 - 343.2 | | 101.0 - 101.0 | |
| MOLEATE | Yao et al, 2008 ^1004^ | | | 15 | | 15 | 273.2 - 342.9 | | 101.0 - 101.0 | |
| MOLEATE | Pratas et al, 2010 ^993^ | | | 15 | | 15 | 283.2 - 353.2 | | 101.0 - 101.0 | |

| Material | | Author | *N*_all_ | | *N*_used_ | | | *T* / K | | *p* / kPa |
| --- | --- | --- | --- | --- | --- | --- | --- | --- | --- | --- |
| MPALMITA | Gaikwad Subrahmanyam, 1988 ^1005^ | | | 1 | | 1 | 353.1 - 353.1 | | 101.0 - 101.0 | |
| MPALMITA | Ceriani et al, 2007 ^992^ | | | 4 | | 4 | 313.2 - 343.2 | | 101.0 - 101.0 | |
| MPALMITA | Yao et al, 2008 ^1004^ | | | 9 | | 9 | 303.2 - 342.9 | | 101.0 - 101.0 | |
| MPALMITA | Pratas et al, 2010 ^993^ | | | 12 | | 12 | 308.2 - 363.2 | | 101.0 - 101.0 | |

| Material | | Author | *N*_all_ | | *N*_used_ | | | *T* / K | | *p* / kPa |
| --- | --- | --- | --- | --- | --- | --- | --- | --- | --- | --- |
| MSTEARAT | Allen et al, 1999 ^996^ | | | 1 | | 0 | 10000.0 - 0.0 | | 1000000000.0 - 0.0 | |
| MSTEARAT | Gros Feuge, 1952 ^1006^ | | | 1 | | 1 | 348.1 - 348.1 | | 101.0 - 101.0 | |
| MSTEARAT | Gaikwad Subrahmanyam, 1988 ^1005^ | | | 1 | | 1 | 353.1 - 353.1 | | 101.0 - 101.0 | |
| MSTEARAT | Cueto et al, 1991 ^409^ | | | 1 | | 1 | 313.0 - 313.0 | | 101.0 - 101.0 | |
| MSTEARAT | Bhuyan et al, 2006 ^1003^ | | | 1 | | 1 | 343.2 - 343.2 | | 101.0 - 101.0 | |
| MSTEARAT | Pratas et al, 2010 ^993^ | | | 11 | | 11 | 313.2 - 363.2 | | 101.0 - 101.0 | |

| Material | | Author | *N*_all_ | | *N*_used_ | | | *T* / K | | *p* / kPa |
| --- | --- | --- | --- | --- | --- | --- | --- | --- | --- | --- |
| MXYLENE | Thorpe Rodger, 1894 ^28^ | | | 14 | | 14 | 273.2 - 403.1 | | 0.0 - 78.0 | |
| MXYLENE | Batschinski, 1913 ^29^ | | | 14 | | 14 | 273.1 - 403.1 | | 0.0 - 78.0 | |
| MXYLENE | Thorpe Rodger, 1894 ^28^ | | | 13 | | 12 | 273.4 - 396.7 | | 101.0 - 101.0 | |
| MXYLENE | Miller, 1924 ^127^ | | | 2 | | 2 | 283.0 - 293.1 | | 101.0 - 101.0 | |
| MXYLENE | Timmermans Hennaut, 1930 ^1007^ | | | 2 | | 2 | 288.1 - 303.1 | | 101.0 - 101.0 | |
| MXYLENE | Schepard et al, 1931 ^266^ | | | 2 | | 2 | 293.1 - 303.1 | | 101.0 - 101.0 | |
| MXYLENE | Teitel’baum et al, 1950 ^140^ | | | 1 | | 1 | 293.1 - 293.1 | | 101.0 - 101.0 | |
| MXYLENE | Blank, 1968 ^44^ | | | 186 | | 186 | 295.4 - 548.1 | | 100.0 - 39325.0 | |
| MXYLENE | Dhillon Chugh, 1976 ^149^ | | | 2 | | 2 | 298.1 - 308.1 | | 101.0 - 101.0 | |
| MXYLENE | Kashiwagi Makita, 1982 ^152^ | | | 48 | | 48 | 298.1 - 348.1 | | 100.0 - 110000.0 | |
| MXYLENE | Al-Madfai et al, 1985 ^1008^ | | | 1 | | 1 | 298.1 - 298.1 | | 101.0 - 101.0 | |
| MXYLENE | Oshmyansky et al, 1986 ^1009^ | | | 4 | | 4 | 298.1 - 358.1 | | 101.0 - 101.0 | |
| MXYLENE | Ramanjaneyulu et al, 1987 ^160^ | | | 1 | | 1 | 303.1 - 303.1 | | 101.0 - 101.0 | |
| MXYLENE | Schumpe Luehring, 1990 ^54^ | | | 1 | | 1 | 293.2 - 293.2 | | 101.0 - 101.0 | |
| MXYLENE | Assael Papadaki, 1991 ^272^ | | | 23 | | 23 | 303.2 - 323.2 | | 100.0 - 56340.0 | |
| MXYLENE | Aralaguppi et al, 1992 ^175^ | | | 3 | | 3 | 298.2 - 308.2 | | 101.0 - 101.0 | |
| MXYLENE | Et-Tahir et al, 1995 ^329^ | | | 24 | | 24 | 298.2 - 363.2 | | 100.0 - 100000.0 | |
| MXYLENE | Reddy et al, 1995 ^952^ | | | 1 | | 1 | 303.2 - 303.2 | | 101.0 - 101.0 | |
| MXYLENE | Goud et al, 1999 ^1010^ | | | 1 | | 1 | 308.2 - 308.2 | | 101.0 - 101.0 | |
| MXYLENE | Moumouzias Ritzoulis, 1999 ^1011^ | | | 4 | | 4 | 293.2 - 308.2 | | 101.0 - 101.0 | |
| MXYLENE | Prasad et al, 1999 ^676^ | | | 4 | | 4 | 293.2 - 323.2 | | 101.0 - 101.0 | |
| MXYLENE | George Sastry, 2003 ^202^ | | | 2 | | 2 | 298.2 - 308.2 | | 101.0 - 101.0 | |
| MXYLENE | Lark et al, 2003 ^203^ | | | 2 | | 2 | 298.2 - 303.2 | | 101.0 - 101.0 | |
| MXYLENE | Caudwell, 2004 ^296^ | | | 114 | | 114 | 298.2 - 473.2 | | 100.0 - 198500.0 | |
| MXYLENE | Singh et al, 2004 ^1012^ | | | 1 | | 1 | 298.2 - 298.2 | | 101.0 - 101.0 | |
| MXYLENE | Yang et al, 2004 ^209^ | | | 3 | | 3 | 298.2 - 323.2 | | 101.0 - 101.0 | |
| MXYLENE | Rathnam et al, 2005 ^558^ | | | 2 | | 2 | 303.2 - 313.2 | | 101.0 - 101.0 | |
| MXYLENE | Singh et al, 2005 ^429^ | | | 1 | | 1 | 298.2 - 298.2 | | 101.0 - 101.0 | |
| MXYLENE | Ali et al, 2006 ^211^ | | | 1 | | 1 | 308.2 - 308.2 | | 101.0 - 101.0 | |
| MXYLENE | Al-Kandary et al, 2006 ^1013^ | | | 4 | | 4 | 288.2 - 303.2 | | 101.0 - 101.0 | |
| MXYLENE | Nain, 2007 ^219^ | | | 4 | | 4 | 288.2 - 318.2 | | 101.0 - 101.0 | |
| MXYLENE | Nain, 2007 ^219^ | | | 4 | | 4 | 288.2 - 318.2 | | 101.0 - 101.0 | |
| MXYLENE | Yang et al, 2007 ^1014^ | | | 7 | | 7 | 298.2 - 353.2 | | 101.0 - 101.0 | |
| MXYLENE | Song et al, 2008 ^225^ | | | 7 | | 7 | 303.2 - 333.2 | | 101.0 - 101.0 | |
| MXYLENE | Caudwell et al, 2009 ^513^ | | | 81 | | 81 | 298.2 - 473.2 | | 100.0 - 198500.0 | |
| MXYLENE | Das et al, 2009 ^1015^ | | | 3 | | 3 | 303.2 - 323.2 | | 101.0 - 101.0 | |
| MXYLENE | Dominguez-Perez, 2009 ^560^ | | | 1 | | 1 | 298.2 - 298.2 | | 101.0 - 101.0 | |
| MXYLENE | Nain et al, 2009 ^229^ | | | 1 | | 1 | 298.2 - 298.2 | | 101.0 - 101.0 | |
| MXYLENE | Rathnam et al, 2009 ^561^ | | | 2 | | 2 | 303.2 - 313.2 | | 101.0 - 101.0 | |
| MXYLENE | Sastry et al, 2009 ^231^ | | | 2 | | 2 | 298.2 - 308.2 | | 101.0 - 101.0 | |
| MXYLENE | Yan et al, 2009 ^232^ | | | 3 | | 3 | 298.2 - 318.2 | | 101.0 - 101.0 | |
| MXYLENE | Habibullah et al, 2010 ^1016^ | | | 2 | | 2 | 308.2 - 318.2 | | 101.0 - 101.0 | |
| MXYLENE | Rathnam et al, 2010 ^562^ | | | 2 | | 2 | 303.2 - 313.2 | | 101.0 - 101.0 | |
| MXYLENE | Bhatia et al, 2011 ^563^ | | | 2 | | 2 | 298.2 - 308.2 | | 101.0 - 101.0 | |
| MXYLENE | Rathnam et al, 2011 ^564^ | | | 2 | | 2 | 303.2 - 313.2 | | 101.0 - 101.0 | |
| MXYLENE | Hamzehlouia Asfour, 2012 ^383^ | | | 2 | | 2 | 308.2 - 313.2 | | 101.0 - 101.0 | |
| MXYLENE | Zarei Salami, 2012 ^1017^ | | | 1 | | 1 | 298.2 - 298.2 | | 101.0 - 101.0 | |
| MXYLENE | Bhalodia Sharma, 2013 ^242^ | | | 3 | | 3 | 303.2 - 313.2 | | 101.0 - 101.0 | |
| MXYLENE | Dikio et al, 2013 ^1018^ | | | 4 | | 4 | 293.2 - 323.2 | | 101.0 - 101.0 | |
| MXYLENE | Abdullaev Akhundov, 1983 ^153^ | | | 28 | | 28 | 473.1 - 673.1 | | 103.0 - 4258.0 | |

| Material | | Author | *N*_all_ | | *N*_used_ | | | *T* / K | | *p* / kPa |
| --- | --- | --- | --- | --- | --- | --- | --- | --- | --- | --- |
| N2O | Harris et al, 1979 ^394^ | | | 5 | | 5 | 196.8 - 276.7 | | 101.0 - 101.0 | |
| N2O | Kestin Wakeham, 1979 ^102^ | | | 5 | | 5 | 300.6 - 473.1 | | 101.0 - 101.0 | |
| N2O | Yokoyama et al, 1994 ^1019^ | | | 373 | | 373 | 309.7 - 318.2 | | 101.0 - 9546.0 | |
| N2O | Hurly, 2004 ^1020^ | | | 152 | | 152 | 225.0 - 375.0 | | 107.0 - 3303.0 | |
| N2O | Takahashi et al, 1996 ^1021^ | | | 275 | | 275 | 298.2 - 398.2 | | 100.0 - 24020.0 | |

| Material | | Author | *N*_all_ | | *N*_used_ | | | *T* / K | | *p* / kPa |
| --- | --- | --- | --- | --- | --- | --- | --- | --- | --- | --- |
| NEON | slusar’ et al, 1973 ^310^ | | | 30 | | 24 | 24.5 - 44.2 | | 38.0 - 2609.0 | |
| NEON | Reynes Thodos, 1966 ^796^ | | | 36 | | 36 | 373.1 - 473.2 | | 7067.0 - 83054.0 | |
| NEON | Kestin et al, 1970 ^96^ | | | 8 | | 8 | 298.1 - 973.1 | | 101.0 - 101.0 | |
| NEON | Kestin et al, 1972 ^97^ | | | 8 | | 8 | 298.1 - 973.1 | | 101.0 - 101.0 | |
| NEON | Kestin et al, 1972 ^97^ | | | 6 | | 6 | 298.1 - 973.1 | | 101.0 - 101.0 | |
| NEON | Borisov et al, 1973 ^98^ | | | 1 | | 0 | 10000.0 - 0.0 | | 1000000000.0 - 0.0 | |
| NEON | Evers et al, 2002 ^104^ | | | 18 | | 18 | 298.2 - 348.2 | | 101.0 - 15827.0 | |
| NEON | Evers et al, 2002 ^104^ | | | 18 | | 18 | 298.2 - 348.2 | | 101.0 - 15720.0 | |

| Material | | Author | *N*_all_ | | *N*_used_ | | | *T* / K | | *p* / kPa |
| --- | --- | --- | --- | --- | --- | --- | --- | --- | --- | --- |
| NEOPENTN | van Itterbeek Paemel, 1940 ^859^ | | | 19 | | 19 | 304.0 - 422.6 | | 4413.0 - 122583.0 | |
| NEOPENTN | Phibbs, 1951 ^1022^ | | | 10 | | 5 | 258.2 - 278.2 | | 101.0 - 101.0 | |
| NEOPENTN | Gonzalez Lee, 1968 ^1023^ | | | 101 | | 101 | 310.9 - 444.2 | | 101.0 - 55158.0 | |
| NEOPENTN | Lambert, 1955 ^12^ | | | 4 | | 4 | 323.1 - 351.1 | | 101.0 - 101.0 | |
| NEOPENTN | McCoubrey Singh, 1963 ^908^ | | | 6 | | 6 | 305.0 - 457.5 | | 101.0 - 101.0 | |
| NEOPENTN | Diaz Pena Esteban, 1966 ^1024^ | | | 16 | | 13 | 283.0 - 393.1 | | 101.0 - 101.0 | |

| Material | | Author | *N*_all_ | | *N*_used_ | | | *T* / K | | *p* / kPa |
| --- | --- | --- | --- | --- | --- | --- | --- | --- | --- | --- |
| NF3 | Hurly, 2004 ^1020^ | | | 156 | | 156 | 225.0 - 325.0 | | 104.0 - 3445.0 | |

| Material | Author | | *N*_all_ | | | *N*_used_ | | *T* / K | *p* / kPa | |
| --- | --- | --- | --- | --- | --- | --- | --- | --- | --- | --- |
| NITROGEN | | Gerf Galkov, 1940 ^615^ | | 7 | 7 | | 66.2 - 77.3 | | | 20.0 - 97.0 |
| NITROGEN | | Boyd, 1930 ^885^ | | 68 | 68 | | 303.1 - 343.1 | | | 7133.0 - 19353.0 |
| NITROGEN | | Trautz Heberling, 1931 ^86^ | | 9 | 9 | | 293.3 - 523.8 | | | 101.0 - 101.0 |
| NITROGEN | | Michels Botzen, 1953 ^1025^ | | 33 | 33 | | 298.1 - 348.1 | | | 1556.0 - 97860.0 |
| NITROGEN | | Baron et al, 1959 ^620^ | | 40 | 40 | | 324.8 - 408.1 | | | 689.0 - 55158.0 |
| NITROGEN | | Ellis Raw, 1959 ^1026^ | | 7 | 7 | | 973.2 - 1274.4 | | | 101.0 - 101.0 |
| NITROGEN | | Flynn et al, 1963 ^1027^ | | 34 | 34 | | 195.1 - 373.1 | | | 686.0 - 17892.0 |
| NITROGEN | | Goldman, 1963 ^1028^ | | 16 | 16 | | 194.8 - 298.2 | | | 5168.0 - 12716.0 |
| NITROGEN | | Iwasaki Kestin, 1963 ^1029^ | | 32 | 32 | | 292.8 - 298.0 | | | 101.0 - 9982.0 |
| NITROGEN | | Kestin Whitelaw, 1963 ^1030^ | | 37 | 37 | | 344.3 - 538.5 | | | 122.0 - 14842.0 |
| NITROGEN | | DiPippo et al, 1966 ^1031^ | | 24 | 24 | | 296.3 - 773.2 | | | 40.0 - 172.0 |
| NITROGEN | | Gururaja et al, 1967 ^392^ | | 2 | 2 | | 298.2 - 298.2 | | | 98.0 - 98.0 |
| NITROGEN | | Clarke Smith, 1968 ^1032^ | | 12 | 12 | | 114.4 - 374.6 | | | 101.0 - 101.0 |
| NITROGEN | | Clarke Smith, 1969 ^1033^ | | 13 | 13 | | 120.0 - 360.0 | | | 101.0 - 101.0 |
| NITROGEN | | Gracki et al, 1969 ^879^ | | 46 | 46 | | 183.1 - 298.1 | | | 535.0 - 25688.0 |
| NITROGEN | | Timrot et al, 1969 ^94^ | | 8 | 8 | | 300.0 - 650.0 | | | 101.0 - 101.0 |
| NITROGEN | | Vermesse, 1969 ^1034^ | | 89 | 89 | | 273.2 - 369.5 | | | 10700.0 - 651000.0 |
| NITROGEN | | Dawe Smith, 1970 ^1035^ | | 15 | 15 | | 293.2 - 1599.6 | | | 101.0 - 101.0 |
| NITROGEN | | Kestin et al, 1971 ^1036^ | | 33 | 33 | | 297.8 - 298.5 | | | 101.0 - 10663.0 |
| NITROGEN | | Kestin et al, 1972 ^97^ | | 6 | 6 | | 298.1 - 973.1 | | | 101.0 - 101.0 |
| NITROGEN | | Borisov et al, 1973 ^98^ | | 1 | 1 | | 300.0 - 300.0 | | | 101.0 - 101.0 |
| NITROGEN | | Golubev Kurin, 1974 ^1037^ | | 70 | 70 | | 273.0 - 423.0 | | | 9807.0 - 401092.0 |
| NITROGEN | | Maitland Smith, 1974 ^1038^ | | 24 | 24 | | 394.8 - 1552.5 | | | 101.0 - 101.0 |
| NITROGEN | | Zozulya Blagoi, 1974 ^1039^ | | 79 | 79 | | 127.0 - 135.0 | | | 3412.0 - 6395.0 |
| NITROGEN | | Timrot et al, 1975 ^100^ | | 31 | 31 | | 295.0 - 573.4 | | | 98.0 - 11750.0 |
| NITROGEN | | Hoogland et al, 1985 ^1040^ | | 15 | 15 | | 298.1 - 333.1 | | | 208.0 - 11692.0 |
| NITROGEN | | Audonnet Padua, 2001 ^1041^ | | 8 | 8 | | 303.2 - 323.2 | | | 4980.0 - 21250.0 |
| NITROGEN | | Evers et al, 2002 ^104^ | | 76 | 76 | | 233.2 - 523.2 | | | 98.0 - 29736.0 |
| NITROGEN | | Hurly et al, 2003 ^105^ | | 23 | 23 | | 298.2 - 298.2 | | | 418.0 - 3278.0 |
| NITROGEN | | Tomida et al, 2009 ^543^ | | 30 | 30 | | 298.2 - 423.2 | | | 101.0 - 4966.0 |
| NITROGEN | | Yusibani et al, 2011 ^889^ | | 32 | 32 | | 297.2 - 499.4 | | | 570.0 - 95150.0 |
| NITROGEN | | Lv et al, 2014 ^1042^ | | 24 | 24 | | 303.4 - 303.5 | | | 98.0 - 4713.0 |
| NITROGEN | | Reynes Thodos, 1966 ^796^ | | 30 | 30 | | 373.1 - 473.2 | | | 7136.0 - 69396.0 |
| NITROGEN | | Van Itterbeek et al, 1966 ^1043^ | | 33 | 33 | | 70.1 - 90.2 | | | 98.0 - 2401.0 |
| NITROGEN | | Grevendonk et al, 1970 ^1044^ | | 134 | 133 | | 66.5 - 123.4 | | | 590.0 - 19470.0 |
| NITROGEN | | Hellemans et al, 1970 ^91^ | | 44 | 36 | | 96.7 - 124.5 | | | 648.0 - 9849.0 |
| NITROGEN | | Ruterford, 1984 ^1045^ | | 5 | 5 | | 298.1 - 298.1 | | | 444.0 - 6994.0 |
| NITROGEN | | Evers et al, 2002 ^104^ | | 76 | 66 | | 233.2 - 523.2 | | | 7.0 - 4181.0 |
| NITROGEN | | Carey et al, 1974 ^109^ | | 44 | 44 | | 289.2 - 992.3 | | | 101.0 - 52800.0 |
| NITROGEN | | Seibt et al, 2006 ^1046^ | | 321 | 321 | | 298.2 - 423.2 | | | 100.0 - 35066.0 |
| NITROGEN | | Sih et al, 2008 ^70^ | | 20 | 20 | | 298.2 - 313.2 | | | 470.0 - 7710.0 |
| NITROGEN | | Seibt et al, 2009 ^798^ | | 66 | 66 | | 293.1 - 423.2 | | | 70.0 - 30306.0 |
| NITROGEN | | Vogel, 2012 ^389^ | | 34 | 34 | | 291.6 - 682.0 | | | 0.0 - 146.0 |

| Material | Author | | *N*_all_ | | | *N*_used_ | | *T* / K | *p* / kPa | |
| --- | --- | --- | --- | --- | --- | --- | --- | --- | --- | --- |
| NONANE | | Bingham Fornwalt, 1930 ^265^ | | 8 | 8 | | 273.2 - 373.1 | | | 101.0 - 101.0 |
| NONANE | | Shepard et al, 1931 ^266^ | | 1 | 1 | | 298.1 - 298.1 | | | 101.0 - 101.0 |
| NONANE | | Doolittle Peterson, 1951 ^267^ | | 5 | 5 | | 263.1 - 423.2 | | | 101.0 - 101.0 |
| NONANE | | Keramidi Rastorguev, 1972 ^1047^ | | 98 | 98 | | 303.1 - 463.1 | | | 98.0 - 49000.0 |
| NONANE | | Moore Wellek, 1974 ^498^ | | 4 | 4 | | 293.1 - 313.1 | | | 101.0 - 101.0 |
| NONANE | | Ratkovics et al, 1974 ^250^ | | 1 | 1 | | 293.1 - 293.1 | | | 101.0 - 101.0 |
| NONANE | | Awwad Allos, 1985 ^282^ | | 1 | 1 | | 298.1 - 298.1 | | | 101.0 - 101.0 |
| NONANE | | Awwad et al, 1986 ^156^ | | 1 | 1 | | 298.1 - 298.1 | | | 101.0 - 101.0 |
| NONANE | | Wakefield Marsh, 1987 ^286^ | | 3 | 3 | | 318.2 - 338.1 | | | 101.0 - 101.0 |
| NONANE | | Wakefield, 1988 ^287^ | | 2 | 2 | | 303.2 - 308.2 | | | 101.0 - 101.0 |
| NONANE | | Iwahashi et al, 1990 ^271^ | | 2 | 2 | | 298.1 - 323.1 | | | 101.0 - 101.0 |
| NONANE | | Assael Papadaki, 1991 ^272^ | | 30 | 30 | | 303.2 - 323.2 | | | 100.0 - 69000.0 |
| NONANE | | Aminabhavi Gopalkrishna, 1994 ^289^ | | 3 | 3 | | 298.2 - 318.2 | | | 101.0 - 101.0 |
| NONANE | | Aminabhavi Gopalkrishna, 1995 ^290^ | | 4 | 4 | | 298.2 - 313.2 | | | 101.0 - 101.0 |
| NONANE | | Aucejo et al, 1995 ^14^ | | 1 | 1 | | 298.2 - 298.2 | | | 101.0 - 101.0 |
| NONANE | | Aminabhavi et al, 1996 ^417^ | | 3 | 3 | | 298.2 - 308.2 | | | 101.0 - 101.0 |
| NONANE | | Aminabhavi Patil, 1997 ^291^ | | 3 | 3 | | 298.2 - 308.2 | | | 101.0 - 101.0 |
| NONANE | | Aralaguppi et al, 1999 ^189^ | | 3 | 3 | | 298.2 - 308.2 | | | 101.0 - 101.0 |
| NONANE | | Nayak et al, 2001 ^292^ | | 3 | 3 | | 298.2 - 308.2 | | | 101.0 - 101.0 |
| NONANE | | Aljmaz et al, 2005 ^300^ | | 3 | 3 | | 293.2 - 303.2 | | | 101.0 - 101.0 |
| NONANE | | Baragi Aralaguppi, 2006 ^337^ | | 3 | 3 | | 298.2 - 308.2 | | | 101.0 - 101.0 |
| NONANE | | Iloukhani et al, 2006 ^339^ | | 3 | 3 | | 293.2 - 303.2 | | | 101.0 - 101.0 |
| NONANE | | Wang et al, 2007 ^825^ | | 5 | 5 | | 293.2 - 313.2 | | | 101.0 - 101.0 |
| NONANE | | Landaverde-Cortes et al, 2008 ^1048^ | | 18 | 18 | | 273.2 - 358.2 | | | 101.0 - 101.0 |
| NONANE | | Zivkovic et al, 2010 ^516^ | | 1 | 1 | | 303.2 - 303.2 | | | 101.0 - 101.0 |
| NONANE | | Zhang et al, 2011 ^303^ | | 4 | 4 | | 293.2 - 313.2 | | | 101.0 - 101.0 |
| NONANE | | Gong et al, 2012 ^517^ | | 4 | 4 | | 293.2 - 313.2 | | | 101.0 - 101.0 |
| NONANE | | Chi et al, 2013 ^274^ | | 7 | 7 | | 293.2 - 323.2 | | | 100.0 - 100.0 |
| NONANE | | Fang et al, 2014 ^518^ | | 4 | 4 | | 283.2 - 303.2 | | | 101.0 - 101.0 |
| NONANE | | Liu Zhu, 2014 ^307^ | | 5 | 5 | | 293.2 - 313.2 | | | 100.0 - 100.0 |
| NONANE | | Melaven Mack, 1932 ^829^ | | 5 | 3 | | 475.3 - 527.0 | | | 101.0 - 101.0 |

| Material | Author | | *N*_all_ | | | *N*_used_ | | *T* / K | *p* / kPa | |
| --- | --- | --- | --- | --- | --- | --- | --- | --- | --- | --- |
| OCTANE | | Thorpe Rodger, 1894 ^28^ | | 11 | 11 | | 273.4 - 395.2 | | | 0.0 - 91.0 |
| OCTANE | | Batschinski, 1913 ^29^ | | 13 | 13 | | 273.1 - 393.1 | | | 0.0 - 86.0 |
| OCTANE | | Albright Lohrenz, 1956 ^799^ | | 2 | 2 | | 416.3 - 448.3 | | | 159.0 - 333.0 |
| OCTANE | | Ledneva, 1956 ^800^ | | 12 | 12 | | 293.1 - 513.2 | | | 1.0 - 1099.0 |
| OCTANE | | Bagdasaryan, 1964 ^30^ | | 6 | 6 | | 273.0 - 373.0 | | | 0.0 - 46.0 |
| OCTANE | | Dymond Young, 1980 ^275^ | | 8 | 8 | | 283.2 - 393.2 | | | 1.0 - 86.0 |
| OCTANE | | Thorpe Rodger, 1894 ^28^ | | 24 | 24 | | 273.4 - 395.2 | | | 101.0 - 101.0 |
| OCTANE | | Timmermans Martin, 1928 ^37^ | | 2 | 2 | | 288.1 - 303.1 | | | 101.0 - 101.0 |
| OCTANE | | Madge, 1930 ^132^ | | 13 | 13 | | 273.2 - 393.1 | | | 101.0 - 101.0 |
| OCTANE | | Shepard et al, 1931 ^266^ | | 1 | 1 | | 298.1 - 298.1 | | | 101.0 - 101.0 |
| OCTANE | | Giller Drickamer, 1949 ^805^ | | 14 | 3 | | 253.2 - 293.1 | | | 101.0 - 101.0 |
| OCTANE | | Doolittle Peterson, 1951 ^267^ | | 4 | 4 | | 263.2 - 372.6 | | | 101.0 - 101.0 |
| OCTANE | | Koshkin Nozdrev, 1953 ^806^ | | 5 | 2 | | 253.2 - 263.2 | | | 101.0 - 101.0 |
| OCTANE | | Ling Van Winkle, 1958 ^43^ | | 4 | 4 | | 303.1 - 368.1 | | | 101.0 - 101.0 |
| OCTANE | | Golubev Agaev, 1963 ^261^ | | 1 | 0 | | 10000.0 - 0.0 | | | 1000000000.0 - 0.0 |
| OCTANE | | Brazier Freeman, 1969 ^11^ | | 24 | 18 | | 273.1 - 333.1 | | | 100.0 - 250000.0 |
| OCTANE | | Moore Wellek, 1974 ^498^ | | 4 | 4 | | 293.1 - 313.1 | | | 101.0 - 101.0 |
| OCTANE | | Ratkovics et al, 1974 ^250^ | | 1 | 1 | | 293.1 - 293.1 | | | 101.0 - 101.0 |
| OCTANE | | Islam Waris, 1976 ^1049^ | | 6 | 6 | | 313.0 - 363.5 | | | 101.0 - 101.0 |
| OCTANE | | Shikhaliev, 1977 ^551^ | | 1 | 1 | | 293.0 - 293.0 | | | 101.0 - 101.0 |
| OCTANE | | Dymond et al, 1981^151^ | | 41 | 19 | | 298.2 - 373.1 | | | 100.0 - 203400.0 |
| OCTANE | | Kashiwagi Makita, 1982 ^152^ | | 48 | 48 | | 298.1 - 348.1 | | | 100.0 - 110000.0 |
| OCTANE | | Awwad et al, 1986 ^156^ | | 1 | 1 | | 298.1 - 298.1 | | | 101.0 - 101.0 |
| OCTANE | | Awwad Salman, 1986 ^284^ | | 1 | 1 | | 298.1 - 298.1 | | | 101.0 - 101.0 |
| OCTANE | | Badalyan Rodchenko, 1986 ^1050^ | | 120 | 108 | | 228.2 - 623.1 | | | 114.0 - 58920.0 |
| OCTANE | | Chandrasekhar et al, 1988 ^161^ | | 1 | 1 | | 303.1 - 303.1 | | | 101.0 - 101.0 |
| OCTANE | | Wakefield, 1988 ^287^ | | 2 | 2 | | 303.2 - 308.2 | | | 101.0 - 101.0 |
| OCTANE | | Wakefield Marsh, 1988 ^286^ | | 2 | 2 | | 318.2 - 328.2 | | | 101.0 - 101.0 |
| OCTANE | | Knapstad et al, 1989 ^164^ | | 5 | 5 | | 293.7 - 369.9 | | | 101.0 - 101.0 |
| OCTANE | | Iwahashi et al, 1990 ^271^ | | 2 | 2 | | 298.1 - 323.1 | | | 101.0 - 101.0 |
| OCTANE | | Kiran Sen, 1992 ^263^ | | 47 | 47 | | 323.0 - 448.0 | | | 8000.0 - 66480.0 |
| OCTANE | | Oliveria Wakeham, 1992 ^502^ | | 48 | 43 | | 303.2 - 348.2 | | | 100.0 - 226480.0 |
| OCTANE | | Oswal Patel, 1992 ^177^ | | 2 | 2 | | 303.2 - 313.2 | | | 101.0 - 101.0 |
| OCTANE | | Aminabhavi et al, 1994 ^815^ | | 5 | 5 | | 298.2 - 318.2 | | | 101.0 - 101.0 |
| OCTANE | | Aminabhavi Gopalkrishna, 1995 ^290^ | | 4 | 4 | | 298.2 - 313.2 | | | 101.0 - 101.0 |
| OCTANE | | Aucejo et al, 1995 ^14^ | | 1 | 1 | | 298.2 - 298.2 | | | 101.0 - 101.0 |
| OCTANE | | Exarchos et al, 1995 ^181^ | | 5 | 5 | | 293.2 - 313.2 | | | 101.0 - 101.0 |
| OCTANE | | Franjo et al, 1995 ^845^ | | 1 | 1 | | 298.2 - 298.2 | | | 101.0 - 101.0 |
| OCTANE | | Aminabhavi et al, 1996 ^417^ | | 3 | 3 | | 298.2 - 308.2 | | | 101.0 - 101.0 |
| OCTANE | | Aminabhavi Patil, 1997 ^291^ | | 3 | 3 | | 298.2 - 308.2 | | | 101.0 - 101.0 |
| OCTANE | | Harris et al, 1997 ^1051^ | | 57 | 32 | | 283.2 - 353.2 | | | 100.0 - 199450.0 |
| OCTANE | | Orge et al, 1997 ^667^ | | 1 | 1 | | 298.2 - 298.2 | | | 101.0 - 101.0 |
| OCTANE | | Dandekar et al, 1998 ^314^ | | 1 | 1 | | 293.2 - 293.2 | | | 101.0 - 101.0 |
| OCTANE | | Jimenez et al, 1998 ^819^ | | 4 | 4 | | 293.2 - 308.2 | | | 101.0 - 101.0 |
| OCTANE | | Aralaguppi et al, 1999 ^189^ | | 3 | 3 | | 298.2 - 308.2 | | | 101.0 - 101.0 |
| OCTANE | | Barrufet et al, 1999 ^1052^ | | 36 | 36 | | 298.0 - 373.4 | | | 101.0 - 24626.0 |
| OCTANE | | Matos et al, 2001 ^820^ | | 4 | 4 | | 283.2 - 313.2 | | | 101.0 - 101.0 |
| OCTANE | | Nayak et al, 2001 ^292^ | | 3 | 3 | | 298.2 - 308.2 | | | 101.0 - 101.0 |
| OCTANE | | Garcia et al, 2002 ^293^ | | 5 | 5 | | 278.2 - 318.2 | | | 101.0 - 101.0 |
| OCTANE | | Gonzalez et al, 2003 ^294^ | | 3 | 3 | | 293.2 - 303.2 | | | 101.0 - 101.0 |
| OCTANE | | Moreiras et al, 2003 ^295^ | | 1 | 1 | | 298.2 - 298.2 | | | 101.0 - 101.0 |
| OCTANE | | Oswal et al, 2003 ^205^ | | 1 | 1 | | 303.2 - 303.2 | | | 101.0 - 101.0 |
| OCTANE | | Trenzado et al, 2003 ^1053^ | | 5 | 5 | | 283.2 - 313.2 | | | 101.0 - 101.0 |
| OCTANE | | Caudwell, 2004 ^296^ | | 175 | 154 | | 298.2 - 473.2 | | | 100.0 - 201800.0 |
| OCTANE | | Gonzalez et al, 2004 ^298^ | | 3 | 3 | | 293.2 - 303.2 | | | 101.0 - 101.0 |
| OCTANE | | Gonzalez et al, 2004 ^298^ | | 3 | 3 | | 293.2 - 303.2 | | | 101.0 - 101.0 |
| OCTANE | | Ma et al, 2004 ^207^ | | 10 | 10 | | 313.2 - 393.2 | | | 150.0 - 6100.0 |
| OCTANE | | Yang et al, 2004 ^209^ | | 7 | 7 | | 298.2 - 353.2 | | | 101.0 - 101.0 |
| OCTANE | | Aljimaz et al, 2005 ^300^ | | 3 | 3 | | 293.2 - 303.2 | | | 101.0 - 101.0 |
| OCTANE | | Ma et al, 2005 ^821^ | | 1 | 1 | | 298.2 - 298.2 | | | 101.0 - 101.0 |
| OCTANE | | Yang et al, 2005 ^559^ | | 9 | 9 | | 293.2 - 363.2 | | | 101.0 - 101.0 |
| OCTANE | | Abdulagatov Azizov, 2006 ^1054^ | | 13 | 13 | | 298.2 - 467.5 | | | 100.0 - 10000.0 |
| OCTANE | | Baragi Aralaguppi, 2006 ^337^ | | 3 | 3 | | 298.2 - 308.2 | | | 101.0 - 101.0 |
| OCTANE | | Goodwin et al, 2006 ^301^ | | 44 | 44 | | 323.2 - 423.1 | | | 100.0 - 68420.0 |
| OCTANE | | Iloukhani et al, 2006 ^339^ | | 3 | 3 | | 293.2 - 303.2 | | | 101.0 - 101.0 |
| OCTANE | | Kumagai et al, 2006 ^264^ | | 16 | 16 | | 273.2 - 333.2 | | | 100.0 - 30000.0 |
| OCTANE | | Suresh Reddy et al, 2006 ^215^ | | 1 | 1 | | 303.2 - 303.2 | | | 101.0 - 101.0 |
| OCTANE | | Dubey Sharma, 2007 ^510^ | | 1 | 1 | | 298.2 - 298.2 | | | 101.0 - 101.0 |
| OCTANE | | Landaverde-Cortes et al, 2007 ^824^ | | 13 | 13 | | 273.2 - 333.2 | | | 101.0 - 101.0 |
| OCTANE | | Reddy et al, 2007 ^220^ | | 1 | 1 | | 303.2 - 303.2 | | | 101.0 - 101.0 |
| OCTANE | | Dubey Sharma, 2008 ^319^ | | 1 | 1 | | 298.2 - 298.2 | | | 101.0 - 101.0 |
| OCTANE | | Dubey Sharma, 2008 ^319^ | | 1 | 1 | | 298.2 - 298.2 | | | 101.0 - 101.0 |
| OCTANE | | Dubey Sharma, 2008 ^319^ | | 1 | 1 | | 298.2 - 298.2 | | | 101.0 - 101.0 |
| OCTANE | | Dubey Sharma, 2008 ^319^ | | 1 | 1 | | 298.2 - 298.2 | | | 101.0 - 101.0 |
| OCTANE | | Yang et al, 2008 ^512^ | | 4 | 4 | | 293.2 - 313.2 | | | 101.0 - 101.0 |
| OCTANE | | Caudwell et al, 2009 ^513^ | | 88 | 79 | | 298.2 - 473.2 | | | 100.0 - 201800.0 |
| OCTANE | | Feitosa et al, 2009 ^514^ | | 2 | 2 | | 293.2 - 298.2 | | | 101.0 - 101.0 |
| OCTANE | | Rathnam et al, 2010 ^437^ | | 3 | 3 | | 303.2 - 313.2 | | | 101.0 - 101.0 |
| OCTANE | | Sato et al, 2010 ^515^ | | 4 | 4 | | 323.2 - 323.2 | | | 180.0 - 10380.0 |
| OCTANE | | Zivkovic et al, 2010 ^516^ | | 1 | 1 | | 303.2 - 303.2 | | | 101.0 - 101.0 |
| OCTANE | | Gong et al, 2012 ^517^ | | 4 | 4 | | 293.2 - 313.2 | | | 101.0 - 101.0 |
| OCTANE | | Rathnam et al, 2012 ^439^ | | 3 | 3 | | 303.2 - 313.2 | | | 101.0 - 101.0 |
| OCTANE | | Estrada-Baltazar et al, 2013 ^1055^ | | 7 | 7 | | 293.2 - 323.2 | | | 101.0 - 101.0 |
| OCTANE | | Fang et al, 2014 ^518^ | | 4 | 4 | | 283.2 - 303.2 | | | 101.0 - 101.0 |
| OCTANE | | Liu Zhu, 2014 ^307^ | | 5 | 5 | | 293.2 - 313.2 | | | 100.0 - 100.0 |
| OCTANE | | Dumitrescu et al, 2015 ^1056^ | | 4 | 4 | | 293.2 - 308.2 | | | 101.0 - 101.0 |
| OCTANE | | Yue et al, 2015 ^903^ | | 5 | 5 | | 293.2 - 313.2 | | | 100.0 - 100.0 |
| OCTANE | | Melaven Mack, 1932 ^829^ | | 4 | 3 | | 423.7 - 524.1 | | | 101.0 - 101.0 |
| OCTANE | | McCoubrey et al, 1951 ^856^ | | 4 | 3 | | 419.1 - 474.2 | | | 101.0 - 101.0 |
| OCTANE | | Lambert et al, 1955 ^12^ | | 7 | 0 | | 10000.0 - 0.0 | | | 1000000000.0 - 0.0 |
| OCTANE | | Ling Van Winkle, 1958 ^43^ | | 2 | 2 | | 403.1 - 423.1 | | | 101.0 - 101.0 |
| OCTANE | | Golubev Agaev, 1964 ^253^ | | 3 | 3 | | 423.1 - 473.5 | | | 101.0 - 101.0 |
| OCTANE | | Carmichael et al sag, 1966 ^831^ | | 2 | 2 | | 310.9 - 344.2 | | | 3.0 - 3.0 |
| OCTANE | | Slusar’ et al, 1973 ^310^ | | 7 | 6 | | 429.3 - 687.3 | | | 101.0 - 101.0 |
| OCTANE | | Diaz Pena et al, 1975 ^904^ | | 11 | 9 | | 413.6 - 719.6 | | | 101.0 - 101.0 |
| OCTANE | | Diaz Pena Cheda, 1975 ^255^ | | 9 | 8 | | 363.0 - 423.1 | | | 27.0 - 27.0 |

| Material | Author | | *N*_all_ | | | *N*_used_ | | *T* / K | *p* / kPa | |
| --- | --- | --- | --- | --- | --- | --- | --- | --- | --- | --- |
| OXYGEN | | Trautz Heberling, 1931 ^86^ | | 9 | 9 | | 293.8 - 523.2 | | | 101.0 - 101.0 |
| OXYGEN | | Luker Johnson, 1959 ^792^ | | 33 | 33 | | 329.1 - 724.2 | | | 5047.0 - 13004.0 |
| OXYGEN | | Gururaja et al, 1967 ^392^ | | 3 | 3 | | 302.6 - 302.6 | | | 98.0 - 98.0 |
| OXYGEN | | Lavushev Lyusternik, 1976 ^1057^ | | 73 | 73 | | 400.0 - 1992.4 | | | 101.0 - 101.0 |

| Material | Author | | *N*_all_ | | | *N*_used_ | | *T* / K | *p* / kPa | |
| --- | --- | --- | --- | --- | --- | --- | --- | --- | --- | --- |
| OXYLENE | | Thorpe Rodger, 1894 ^28^ | | 12 | 12 | | 273.2 - 413.0 | | | 0.0 - 91.0 |
| OXYLENE | | Batschinski et al, 1913 ^29^ | | 15 | 15 | | 273.1 - 413.1 | | | 0.0 - 91.0 |
| OXYLENE | | Timmermans Hennaut, 1955 ^1058^ | | 2 | 2 | | 288.1 - 303.1 | | | 1.0 - 1.0 |
| OXYLENE | | Panchenkov et al, 1958 ^387^ | | 7 | 7 | | 293.1 - 353.1 | | | 1.0 - 13.0 |
| OXYLENE | | Thorpe Rodger, 1894 ^28^ | | 24 | 24 | | 273.5 - 414.3 | | | 101.0 - 101.0 |
| OXYLENE | | 1967 mamedov et al, 1967 ^1059^ | | 142 | 142 | | 296.9 - 548.2 | | | 100.0 - 40000.0 |
| OXYLENE | | Dhillon Chugh, 1976 ^149^ | | 1 | 1 | | 298.1 - 298.1 | | | 101.0 - 101.0 |
| OXYLENE | | Kashiwagi Makita, 1982 ^152^ | | 48 | 48 | | 298.1 - 348.1 | | | 100.0 - 110000.0 |
| OXYLENE | | Al-Madfai et al, 1985 ^1008^ | | 1 | 1 | | 298.1 - 298.1 | | | 101.0 - 101.0 |
| OXYLENE | | Ramanyaneyulu et al, 1987 ^160^ | | 1 | 1 | | 303.1 - 303.1 | | | 101.0 - 101.0 |
| OXYLENE | | Rathnam, 1988 ^163^ | | 1 | 1 | | 303.1 - 303.1 | | | 101.0 - 101.0 |
| OXYLENE | | Et-Tahir et al, 1995 ^329^ | | 30 | 30 | | 298.2 - 363.2 | | | 100.0 - 100000.0 |
| OXYLENE | | Ramachandran et al, 1995 ^1060^ | | 1 | 1 | | 303.2 - 303.2 | | | 101.0 - 101.0 |
| OXYLENE | | Swain et al, 1997 ^184^ | | 4 | 4 | | 303.2 - 318.2 | | | 101.0 - 101.0 |
| OXYLENE | | Swain et al, 1998 ^187^ | | 4 | 4 | | 303.2 - 318.2 | | | 101.0 - 101.0 |
| OXYLENE | | Goud et al, 1999 ^1010^ | | 1 | 1 | | 308.2 - 308.2 | | | 101.0 - 101.0 |
| OXYLENE | | Moumouzias et al, 1999 ^1061^ | | 4 | 4 | | 293.2 - 308.2 | | | 101.0 - 101.0 |
| OXYLENE | | Swain et al, 2000 ^194^ | | 13 | 13 | | 293.2 - 318.2 | | | 101.0 - 101.0 |
| OXYLENE | | Swain et al, 2001 ^200^ | | 5 | 5 | | 293.2 - 318.2 | | | 101.0 - 101.0 |
| OXYLENE | | George Sastry, 2003 ^202^ | | 2 | 2 | | 298.2 - 308.2 | | | 101.0 - 101.0 |
| OXYLENE | | Katyal et al, 2003 ^556^ | | 3 | 3 | | 293.2 - 313.2 | | | 101.0 - 101.0 |
| OXYLENE | | Lark et al, 2003 ^203^ | | 2 | 2 | | 298.2 - 303.2 | | | 101.0 - 101.0 |
| OXYLENE | | Jain Singh, 2004 ^64^ | | 1 | 1 | | 303.2 - 303.2 | | | 101.0 - 101.0 |
| OXYLENE | | Singh et al, 2004 ^1012^ | | 1 | 1 | | 298.2 - 298.2 | | | 101.0 - 101.0 |
| OXYLENE | | Yang et al, 2004 ^209^ | | 3 | 3 | | 298.2 - 323.2 | | | 101.0 - 101.0 |
| OXYLENE | | Rathnam et al, 2005 ^558^ | | 2 | 2 | | 303.2 - 313.2 | | | 101.0 - 101.0 |
| OXYLENE | | Singh et al, 2005 ^429^ | | 1 | 1 | | 298.2 - 298.2 | | | 101.0 - 101.0 |
| OXYLENE | | Ali et al, 2006 ^211^ | | 1 | 1 | | 308.2 - 308.2 | | | 101.0 - 101.0 |
| OXYLENE | | Iloukhani et al, 2006 ^339^ | | 4 | 4 | | 288.2 - 303.2 | | | 101.0 - 101.0 |
| OXYLENE | | Das et al, 2006 ^1062^ | | 2 | 2 | | 303.2 - 313.2 | | | 101.0 - 101.0 |
| OXYLENE | | Nain, 2007 ^219^ | | 4 | 4 | | 288.2 - 318.2 | | | 101.0 - 101.0 |
| OXYLENE | | Nain, 2007 ^219^ | | 4 | 4 | | 288.2 - 318.2 | | | 101.0 - 101.0 |
| OXYLENE | | Yang et al, 2007 ^1014^ | | 7 | 7 | | 298.2 - 353.2 | | | 101.0 - 101.0 |
| OXYLENE | | Song et al, 2008 ^225^ | | 7 | 7 | | 303.2 - 333.2 | | | 101.0 - 101.0 |
| OXYLENE | | Dominguez-Perez, 2009 ^560^ | | 1 | 1 | | 298.2 - 298.2 | | | 101.0 - 101.0 |
| OXYLENE | | Nain et al, 2009 ^229^ | | 1 | 1 | | 298.2 - 298.2 | | | 101.0 - 101.0 |
| OXYLENE | | Rathnam et al, 2009 ^561^ | | 2 | 2 | | 303.2 - 313.2 | | | 101.0 - 101.0 |
| OXYLENE | | Sastry et al, 2009 ^231^ | | 2 | 2 | | 298.2 - 308.2 | | | 101.0 - 101.0 |
| OXYLENE | | Yan et al, 2009 ^232^ | | 3 | 3 | | 298.2 - 318.2 | | | 101.0 - 101.0 |
| OXYLENE | | Habibullah et al, 2010 ^1016^ | | 2 | 2 | | 308.2 - 318.2 | | | 101.0 - 101.0 |
| OXYLENE | | Rathnam et al, 2010 ^562^ | | 2 | 2 | | 303.2 - 313.2 | | | 101.0 - 101.0 |
| OXYLENE | | Bhatia et al, 2011 ^563^ | | 2 | 2 | | 298.2 - 308.2 | | | 101.0 - 101.0 |
| OXYLENE | | Rathnam et al, 2011 ^564^ | | 2 | 2 | | 303.2 - 313.2 | | | 101.0 - 101.0 |
| OXYLENE | | Zarei Salami, 2012 ^1017^ | | 1 | 1 | | 298.2 - 298.2 | | | 101.0 - 101.0 |
| OXYLENE | | Dikio et al, 2013 ^1018^ | | 4 | 4 | | 293.2 - 323.2 | | | 101.0 - 101.0 |
| OXYLENE | | Abdullaev Akhundov, 1983 ^153^ | | 28 | 28 | | 473.1 - 673.1 | | | 103.0 - 4132.0 |

| Material | Author | | *N*_all_ | | | *N*_used_ | | *T* / K | *p* / kPa | |
| --- | --- | --- | --- | --- | --- | --- | --- | --- | --- | --- |
| PENTANE | | Thorpe Rodger, 1894 ^28^ | | 7 | 7 | | 273.9 - 305.8 | | | 26.0 - 91.0 |
| PENTANE | | Batchinkski, 1913 ^29^ | | 4 | 4 | | 273.1 - 303.1 | | | 25.0 - 83.0 |
| PENTANE | | Sage Lacey, 1938 ^1063^ | | 3 | 3 | | 310.9 - 366.5 | | | 108.0 - 509.0 |
| PENTANE | | Khalilov, 1939 ^112^ | | 14 | 14 | | 293.1 - 423.1 | | | 57.0 - 1598.0 |
| PENTANE | | Hubbard Brown, 1943 ^1064^ | | 12 | 9 | | 298.1 - 453.1 | | | 69.0 - 2618.0 |
| PENTANE | | Albright Lohrenz, 1956 ^799^ | | 2 | 2 | | 396.3 - 414.2 | | | 968.0 - 1363.0 |
| PENTANE | | Golubev, 1959 ^8^ | | 14 | 12 | | 193.2 - 303.1 | | | 0.0 - 83.0 |
| PENTANE | | Bulanov et al, 1974 ^117^ | | 11 | 11 | | 293.1 - 363.1 | | | 57.0 - 470.0 |
| PENTANE | | Abdulagatov Rasulov, 1992 ^801^ | | 9 | 0 | | 10000.0 - 0.0 | | | 1000000000.0 - 0.0 |
| PENTANE | | Abdulagatov Rasulov, 1996 ^802^ | | 18 | 14 | | 298.0 - 466.0 | | | 69.0 - 3179.0 |
| PENTANE | | Abdulagatov Rasulov, 1992 ^801^ | | 9 | 3 | | 466.2 - 467.2 | | | 3185.0 - 3232.0 |
| PENTANE | | Abdulagatov Rasulov, 1996 ^802^ | | 17 | 13 | | 313.0 - 466.0 | | | 116.0 - 3178.0 |
| PENTANE | | Thorpe Rodger, 1894 ^28^ | | 6 | 6 | | 273.9 - 303.5 | | | 101.0 - 101.0 |
| PENTANE | | Tausz Staab, 1930 ^133^ | | 1 | 1 | | 293.1 - 293.1 | | | 101.0 - 101.0 |
| PENTANE | | Shepard et al, 1931 ^266^ | | 1 | 1 | | 298.1 - 298.1 | | | 101.0 - 101.0 |
| PENTANE | | Timmermans Hennaut, 1935 ^570^ | | 2 | 2 | | 288.1 - 303.1 | | | 101.0 - 101.0 |
| PENTANE | | Sage Lacey, 1938 ^1063^ | | 9 | 9 | | 310.9 - 366.5 | | | 3447.0 - 10342.0 |
| PENTANE | | Giller Drickamer, 1949 ^805^ | | 18 | 18 | | 137.2 - 293.1 | | | 101.0 - 101.0 |
| PENTANE | | Doolittle Peterson, 1951 ^267^ | | 2 | 2 | | 263.1 - 293.1 | | | 101.0 - 101.0 |
| PENTANE | | Reamer et al, 1959 ^1065^ | | 51 | 51 | | 310.9 - 410.9 | | | 685.0 - 34958.0 |
| PENTANE | | Lee Ellington, 1965 ^496^ | | 33 | 33 | | 310.9 - 444.3 | | | 1379.0 - 20684.0 |
| PENTANE | | Brazier Freeman, 1969 ^11^ | | 9 | 5 | | 303.1 - 303.1 | | | 100.0 - 200000.0 |
| PENTANE | | Collings McLaughlin, 1971 ^145^ | | 18 | 9 | | 303.1 - 323.1 | | | 98.0 - 196080.0 |
| PENTANE | | Belinskii Ikramov, 1973 ^1066^ | | 48 | 7 | | 293.1 - 313.1 | | | 98.0 - 98100.0 |
| PENTANE | | Ratkovics et al, 1974 ^250^ | | 1 | 1 | | 293.1 - 293.1 | | | 101.0 - 101.0 |
| PENTANE | | Aucejo et al, 1986 ^283^ | | 1 | 1 | | 298.1 - 298.1 | | | 101.0 - 101.0 |
| PENTANE | | Awwad Salami, 1986 ^284^ | | 1 | 1 | | 298.1 - 298.1 | | | 101.0 - 101.0 |
| PENTANE | | Postigo et al, 1986 ^1067^ | | 1 | 1 | | 298.1 - 298.1 | | | 101.0 - 101.0 |
| PENTANE | | De Soria et al, 1988 ^1068^ | | 1 | 1 | | 298.1 - 298.1 | | | 101.0 - 101.0 |
| PENTANE | | Iwahashi et al, 1990 ^271^ | | 1 | 1 | | 298.1 - 298.1 | | | 101.0 - 101.0 |
| PENTANE | | Kiran Sen, 1992 ^263^ | | 139 | 139 | | 318.0 - 443.0 | | | 7500.0 - 70260.0 |
| PENTANE | | Oliveria Wakeham, 1992 ^502^ | | 29 | 28 | | 303.2 - 323.2 | | | 100.0 - 251600.0 |
| PENTANE | | Acevedo et al, 1993 ^1069^ | | 1 | 1 | | 298.2 - 298.2 | | | 101.0 - 101.0 |
| PENTANE | | Aucejo et al, 1995 ^14^ | | 1 | 1 | | 298.2 - 298.2 | | | 101.0 - 101.0 |
| PENTANE | | Orge et al, 1997 ^667^ | | 1 | 1 | | 298.2 - 298.2 | | | 101.0 - 101.0 |
| PENTANE | | Estrada Baltazar, 1998 ^504^ | | 31 | 31 | | 298.0 - 373.4 | | | 101.0 - 24626.0 |
| PENTANE | | Sastry Valand, 1998 ^959^ | | 1 | 1 | | 298.2 - 298.2 | | | 101.0 - 101.0 |
| PENTANE | | Audonnet Padua, 2001 ^1041^ | | 40 | 40 | | 303.2 - 383.2 | | | 110.0 - 100060.0 |
| PENTANE | | Tohidi et al, 2001 ^507^ | | 48 | 48 | | 323.0 - 423.0 | | | 3920.0 - 138790.0 |
| PENTANE | | Ma et al, 2004 ^207^ | | 14 | 14 | | 293.2 - 373.2 | | | 100.0 - 5100.0 |
| PENTANE | | Iloukhani et al, 2005 ^428^ | | 1 | 1 | | 298.2 - 298.2 | | | 101.0 - 101.0 |
| PENTANE | | Iloukhani et al, 2006 ^339^ | | 3 | 3 | | 293.2 - 303.2 | | | 101.0 - 101.0 |
| PENTANE | | Titani, 1930 ^9^ | | 7 | 7 | | 394.7 - 579.1 | | | 101.0 - 101.0 |
| PENTANE | | Bleakney et al, 1932 ^906^ | | 2 | 1 | | 373.1 - 373.1 | | | 101.0 - 101.0 |
| PENTANE | | Day, 1932 ^907^ | | 28 | 28 | | 298.1 - 298.1 | | | 0.0 - 65.0 |
| PENTANE | | Titani, 1933 ^75^ | | 7 | 7 | | 394.7 - 579.1 | | | 101.0 - 101.0 |
| PENTANE | | Lambert et al, 1955 ^12^ | | 8 | 7 | | 323.1 - 363.3 | | | 101.0 - 101.0 |
| PENTANE | | McCoubrey Singh, 1963 ^908^ | | 7 | 6 | | 330.5 - 474.0 | | | 101.0 - 101.0 |
| PENTANE | | Agaev Usibova, 1968 ^478^ | | 9 | 9 | | 324.1 - 483.1 | | | 101.0 - 101.0 |
| PENTANE | | Diaz Pena et al, 1975 ^904^ | | 14 | 14 | | 295.4 - 423.1 | | | 27.0 - 27.0 |
| PENTANE | | Abdulagatov Rasulov, 1992 ^801^ | | 13 | 13 | | 352.7 - 524.1 | | | 100.0 - 3000.0 |
| PENTANE | | Hubbard Brown, 1943 ^1064^ | | 145 | 139 | | 298.1 - 523.2 | | | 689.0 - 6895.0 |
| PENTANE | | Agaev Golubev, 1963 ^830^ | | 315 | 312 | | 298.1 - 548.2 | | | 101.0 - 50662.0 |
| PENTANE | | Abdulagatov et al, 1991 ^1070^ | | 48 | 48 | | 457.2 - 493.0 | | | 2500.0 - 8000.0 |
| PENTANE | | Abdulagatov Rasulov, 1992 ^801^ | | 63 | 63 | | 298.5 - 524.1 | | | 100.0 - 50000.0 |
| PENTANE | | Vogel Holdt, 1991 ^833^ | | 67 | 67 | | 299.5 - 631.8 | | | 40.0 - 295.0 |

| Material | Author | | | *N*_all_ | | *N*_used_ | | *T* / K | *p* / kPa | |
| --- | --- | --- | --- | --- | --- | --- | --- | --- | --- | --- |
| PROPANE | | Huang et al, 1966 ^937^ | 15 | | 15 | | 173.2 - 213.2 | | | 6895.0 - 34474.0 |
| PROPANE | | Sage Lacey, 1938 ^1063^ | 4 | | 4 | | 310.9 - 360.9 | | | 1300.0 - 3624.0 |
| PROPANE | | Gerf Galkov, 1940 ^615^ | 14 | | 4 | | 141.8 - 169.6 | | | 0.0 - 2.0 |
| PROPANE | | Galkov Gerf, 1941 ^616^ | 5 | | 2 | | 144.4 - 175.8 | | | 0.0 - 4.0 |
| PROPANE | | Rabe, 1981 ^390^ | 6 | | 6 | | 185.0 - 300.0 | | | 8.0 - 997.0 |
| PROPANE | | Diller, 1982 ^1071^ | 24 | | 14 | | 140.0 - 300.0 | | | 0.0 - 997.0 |
| PROPANE | | Sage Lacey, 1938 ^1063^ | 3 | | 0 | | 10000.0 - 0.0 | | | 1000000000.0 - 0.0 |
| PROPANE | | Klemenc Remi, 1924 ^1072^ | 1 | | 1 | | 293.0 - 293.0 | | | 101.0 - 101.0 |
| PROPANE | | Titani, 1930 ^9^ | 6 | | 6 | | 293.1 - 393.1 | | | 101.0 - 101.0 |
| PROPANE | | Trautz Kurtz, 1931 ^867^ | 6 | | 6 | | 301.3 - 548.8 | | | 101.0 - 101.0 |
| PROPANE | | Trautz Sorg, 1931 ^618^ | 6 | | 6 | | 291.0 - 523.8 | | | 101.0 - 101.0 |
| PROPANE | | Adzumi, 1937 ^244^ | 8 | | 8 | | 303.1 - 373.1 | | | 101.0 - 101.0 |
| PROPANE | | Sage Lacey, 1938 ^1063^ | 5 | | 5 | | 310.9 - 373.5 | | | 101.0 - 101.0 |
| PROPANE | | Sage Lacey, 1938 ^1063^ | 28 | | 23 | | 310.9 - 360.9 | | | 101.0 - 2758.0 |
| PROPANE | | Wobser Muller, 1941 ^251^ | 5 | | 5 | | 293.1 - 373.1 | | | 101.0 - 101.0 |
| PROPANE | | Comings et al, 1944 ^763^ | 6 | | 6 | | 303.1 - 377.6 | | | 101.0 - 101.0 |
| PROPANE | | Golubev Petrov, 1953 ^764^ | 8 | | 8 | | 297.6 - 523.2 | | | 101.0 - 101.0 |
| PROPANE | | Lambert et al, 1955 ^12^ | 7 | | 7 | | 308.1 - 363.7 | | | 101.0 - 101.0 |
| PROPANE | | Baron et al, 1959 ^620^ | 39 | | 38 | | 324.8 - 408.1 | | | 689.0 - 55158.0 |
| PROPANE | | Starling et al, 1960 ^1073^ | 11 | | 11 | | 294.2 - 511.0 | | | 101.0 - 101.0 |
| PROPANE | | Carmichael et al, 1964 ^1074^ | 23 | | 23 | | 277.6 - 477.6 | | | 110.0 - 3595.0 |
| PROPANE | | Golubev Gnezdilov, 1971 ^1075^ | 130 | | 130 | | 297.7 - 523.1 | | | 100.0 - 81040.0 |
| PROPANE | | Kestin et al, 1971 ^254^ | 2 | | 2 | | 296.4 - 303.5 | | | 101.0 - 101.0 |
| PROPANE | | Diaz Pena Cheda, 1975 ^255^ | 11 | | 11 | | 304.0 - 407.8 | | | 27.0 - 27.0 |
| PROPANE | | Kestin et al, 1977 ^256^ | 5 | | 5 | | 299.5 - 478.1 | | | 101.0 - 101.0 |
| PROPANE | | Abe et al, 1978 ^257^ | 5 | | 5 | | 298.1 - 468.1 | | | 101.0 - 101.0 |
| PROPANE | | Nagaoka et al, 1986 ^1076^ | 3 | | 3 | | 298.1 - 348.1 | | | 101.0 - 101.0 |
| PROPANE | | Hurly et al, 2003 ^105^ | 11 | | 11 | | 298.2 - 298.2 | | | 99.0 - 831.0 |
| PROPANE | | Sage Lacey, 1938 ^1063^ | 36 | | 35 | | 310.9 - 360.9 | | | 1379.0 - 13790.0 |
| PROPANE | | Lipkin et al, 1942 ^259^ | 9 | | 3 | | 199.9 - 222.1 | | | 101.0 - 101.0 |
| PROPANE | | Smith Brown, 1943 ^623^ | 119 | | 119 | | 295.0 - 463.2 | | | 689.0 - 34474.0 |
| PROPANE | | Golubev Agaev, 1963 ^261^ | 2 | | 0 | | 10000.0 - 0.0 | | | 1000000000.0 - 0.0 |
| PROPANE | | Carmichael et al, 1964 ^1074^ | 25 | | 25 | | 277.6 - 477.6 | | | 7005.0 - 34672.0 |
| PROPANE | | Giddings et al, 1966 ^941^ | 23 | | 12 | | 298.1 - 298.1 | | | 689.0 - 54951.0 |
| PROPANE | | Diller, 1982 ^1071^ | 60 | | 60 | | 90.0 - 300.0 | | | 1705.0 - 31489.0 |
| PROPANE | | Sage Lacey, 1938 ^1063^ | 22 | | 20 | | 377.6 - 377.6 | | | 101.0 - 13790.0 |
| PROPANE | | Seibt et al, 2011 ^625^ | 212 | | 212 | | 273.2 - 423.2 | | | 41.0 - 29830.0 |
| PROPANE | | Vogel, 1995 ^1077^ | 70 | | 70 | | 297.2 - 625.8 | | | 25.0 - 263.0 |
| PROPANE | | Wilhelm Vogel, 2001 ^1078^ | 614 | | 614 | | 298.2 - 423.2 | | | 39.0 - 19795.0 |

| Material | Author | | *N*_all_ | | | *N*_used_ | | *T* / K | *p* / kPa | |
| --- | --- | --- | --- | --- | --- | --- | --- | --- | --- | --- |
| PROPYLEN | | Gerf Galkov, 1940 ^615^ | | 13 | 5 | | 141.6 - 169.6 | | | 0.0 - 3.0 |
| PROPYLEN | | Galkov Gerf, 1941 ^616^ | | 5 | 2 | | 173.0 - 174.8 | | | 4.0 - 5.0 |
| PROPYLEN | | Neduzhii Khmara, 1968 ^2^ | | 34 | 33 | | 193.9 - 355.2 | | | 18.0 - 3871.0 |
| PROPYLEN | | Titani, 1930 ^9^ | | 7 | 7 | | 273.2 - 393.1 | | | 101.0 - 101.0 |
| PROPYLEN | | Trautz Husseini, 1934 ^348^ | | 10 | 10 | | 289.9 - 521.7 | | | 101.0 - 101.0 |
| PROPYLEN | | Van Cleave Maass, 1935 ^87^ | | 13 | 13 | | 193.5 - 297.3 | | | 0.0 - 0.0 |
| PROPYLEN | | Adzumi, 1937 ^244^ | | 8 | 8 | | 303.1 - 373.1 | | | 101.0 - 101.0 |
| PROPYLEN | | Adzumi, 1937 ^244^ | | 1 | 1 | | 293.1 - 293.1 | | | 0.0 - 0.0 |
| PROPYLEN | | Craven Lambert, 1951 ^76^ | | 4 | 4 | | 293.1 - 523.2 | | | 101.0 - 101.0 |
| PROPYLEN | | Lambert et al, 1955 ^12^ | | 9 | 9 | | 308.1 - 363.9 | | | 101.0 - 101.0 |
| PROPYLEN | | Golubev Agaev, 1964 ^253^ | | 7 | 7 | | 291.1 - 523.2 | | | 101.0 - 101.0 |
| PROPYLEN | | Neduzhii Khmara, 1968 ^2^ | | 22 | 20 | | 230.0 - 310.0 | | | 98.0 - 784.0 |
| PROPYLEN | | Haepp, 1976 ^1079^ | | 10 | 10 | | 303.5 - 484.8 | | | 101.0 - 101.0 |
| PROPYLEN | | Golubev, 1959 ^8^ | | 96 | 96 | | 291.1 - 523.2 | | | 101.0 - 81056.0 |

| Material | Author | | *N*_all_ | | | *N*_used_ | | *T* / K | *p* / kPa | |
| --- | --- | --- | --- | --- | --- | --- | --- | --- | --- | --- |
| PROPYLENEOXIDE | | Scholz Kley, 1981 ^7^ | | 4 | 4 | | 273.2 - 293.2 | | | 24.0 - 58.0 |
| PROPYLENEOXIDE | | Liessmann et al, 1995 ^5^ | | 11 | 11 | | 223.2 - 373.2 | | | 1.0 - 677.0 |
| PROPYLENEOXIDE | | Zimakov Sokolava, 1953 ^1080^ | | 8 | 8 | | 273.1 - 293.2 | | | 24.0 - 101.0 |

| Material | Author | | *N*_all_ | | | *N*_used_ | | *T* / K | *p* / kPa | |
| --- | --- | --- | --- | --- | --- | --- | --- | --- | --- | --- |
| PROPYNE | | Liessmann et al, 1995 ^5^ | | 5 | 5 | | 273.2 - 353.2 | | | 266.0 - 2276.0 |
| PROPYNE | | Titani, 1930 ^9^ | | 6 | 6 | | 293.1 - 373.1 | | | 101.0 - 101.0 |

| Material | Author | *N*_all_ | | | *N*_used_ | | *T* / K | *p* / kPa | |
| --- | --- | --- | --- | --- | --- | --- | --- | --- | --- |
| PXYLENE | Thorpe Rodger, 1894^28^ | | 13 | 13 | | 281.5 - 408.3 | | | 0.0 - 91.0 |
| PXYLENE | Batschinski, 1913 ^29^ | | 13 | 13 | | 283.1 - 403.1 | | | 0.0 - 79.0 |
| PXYLENE | Panchenkov et al, 1958 ^387^ | | 7 | 7 | | 293.1 - 353.1 | | | 1.0 - 15.0 |
| PXYLENE | Thorpe Rodger, 1894 ^28^ | | 12 | 12 | | 281.4 - 408.3 | | | 101.0 - 101.0 |
| PXYLENE | Timmermans Martin, 1926 ^131^ | | 2 | 2 | | 288.1 - 303.1 | | | 101.0 - 101.0 |
| PXYLENE | Alms et al, 1973 ^147^ | | 1 | 1 | | 296.8 - 296.8 | | | 101.0 - 101.0 |
| PXYLENE | Dhillon Chugh, 1976 ^149^ | | 2 | 2 | | 298.1 - 308.1 | | | 101.0 - 101.0 |
| PXYLENE | Yadava Yadava, 1981 ^51^ | | 1 | 1 | | 307.3 - 307.3 | | | 101.0 - 101.0 |
| PXYLENE | Kashiwagi Makita, 1982 ^152^ | | 48 | 48 | | 298.1 - 348.1 | | | 100.0 - 110000.0 |
| PXYLENE | Al-Madfai et al, 1985 ^1008^ | | 1 | 1 | | 298.1 - 298.1 | | | 101.0 - 101.0 |
| PXYLENE | Manjeshwar Aminabhavi, 1987 ^159^ | | 1 | 1 | | 318.0 - 318.0 | | | 101.0 - 101.0 |
| PXYLENE | Ramanjaneyulu et al, 1987 ^160^ | | 1 | 1 | | 303.1 - 303.1 | | | 101.0 - 101.0 |
| PXYLENE | Chandrasekhar et al, 1988 ^161^ | | 1 | 1 | | 303.1 - 303.1 | | | 101.0 - 101.0 |
| PXYLENE | Fermeglia Lapasin, 1988 ^553^ | | 1 | 1 | | 298.1 - 298.1 | | | 101.0 - 101.0 |
| PXYLENE | Rathnam, 1988 ^163^ | | 1 | 1 | | 303.1 - 303.1 | | | 101.0 - 101.0 |
| PXYLENE | Ruiz et al, 1989 ^166^ | | 3 | 3 | | 303.1 - 323.1 | | | 101.0 - 101.0 |
| PXYLENE | Sivaramprasad et al, 1990 ^170^ | | 1 | 1 | | 298.2 - 298.2 | | | 101.0 - 101.0 |
| PXYLENE | Tasioula Demetropolous, 1992 ^178^ | | 5 | 5 | | 298.2 - 318.2 | | | 101.0 - 101.0 |
| PXYLENE | Et-Tahir et al, 1995 ^329^ | | 15 | 15 | | 313.2 - 353.2 | | | 100.0 - 100000.0 |
| PXYLENE | Exarchos et al, 1995 ^181^ | | 5 | 5 | | 293.2 - 313.2 | | | 101.0 - 101.0 |
| PXYLENE | Ramachandran et al, 1995 ^1060^ | | 1 | 1 | | 303.2 - 303.2 | | | 101.0 - 101.0 |
| PXYLENE | Oswal et al, 1996 ^330^ | | 1 | 1 | | 303.2 - 303.2 | | | 101.0 - 101.0 |
| PXYLENE | Aminabhavi Banerjee, 1999 ^188^ | | 3 | 3 | | 298.2 - 308.2 | | | 101.0 - 101.0 |
| PXYLENE | Aralaguppi et al, 1999 ^189^ | | 3 | 3 | | 298.2 - 308.2 | | | 101.0 - 101.0 |
| PXYLENE | Goud et al, 1999 ^1010^ | | 1 | 1 | | 308.2 - 308.2 | | | 101.0 - 101.0 |
| PXYLENE | Prasad et al, 1999 ^676^ | | 4 | 4 | | 293.2 - 323.2 | | | 101.0 - 101.0 |
| PXYLENE | Prasad et al, 2000 ^963^ | | 4 | 4 | | 293.2 - 323.2 | | | 101.0 - 101.0 |
| PXYLENE | Tsierkezos et al, 2000 ^195^ | | 1 | 1 | | 293.2 - 293.2 | | | 101.0 - 101.0 |
| PXYLENE | Nayak et al, 2002 ^201^ | | 3 | 3 | | 298.2 - 308.2 | | | 101.0 - 101.0 |
| PXYLENE | Peng Tu, 2002 ^61^ | | 1 | 1 | | 298.2 - 298.2 | | | 101.0 - 101.0 |
| PXYLENE | Yang et al, 2002 ^1081^ | | 1 | 1 | | 298.2 - 298.2 | | | 101.0 - 101.0 |
| PXYLENE | George Sastry, 2003 ^202^ | | 2 | 2 | | 298.2 - 308.2 | | | 101.0 - 101.0 |
| PXYLENE | George Sastry, 2003 ^202^ | | 2 | 2 | | 298.2 - 308.2 | | | 101.0 - 101.0 |
| PXYLENE | Lark et al, 2003 ^203^ | | 2 | 2 | | 298.2 - 303.2 | | | 101.0 - 101.0 |
| PXYLENE | Moreiras et al, 2003 ^295^ | | 1 | 1 | | 298.2 - 298.2 | | | 101.0 - 101.0 |
| PXYLENE | Oswal et al, 2004 ^208^ | | 1 | 1 | | 303.2 - 303.2 | | | 101.0 - 101.0 |
| PXYLENE | Singh et al, 2004 ^1012^ | | 1 | 1 | | 298.2 - 298.2 | | | 101.0 - 101.0 |
| PXYLENE | Yang et al, 2004 ^209^ | | 3 | 3 | | 298.2 - 323.2 | | | 101.0 - 101.0 |
| PXYLENE | Yang et al, 2004 ^209^ | | 4 | 4 | | 298.2 - 323.2 | | | 101.0 - 101.0 |
| PXYLENE | Yang et al, 2004 ^209^ | | 7 | 7 | | 298.2 - 353.2 | | | 101.0 - 101.0 |
| PXYLENE | Feng et al, 2005 ^1082^ | | 1 | 1 | | 298.2 - 298.2 | | | 101.0 - 101.0 |
| PXYLENE | Kapoor Rattan, 2005 ^509^ | | 3 | 3 | | 303.2 - 313.2 | | | 101.0 - 101.0 |
| PXYLENE | Kapoor Rattan, 2005 ^509^ | | 3 | 3 | | 303.2 - 313.2 | | | 101.0 - 101.0 |
| PXYLENE | Oswal et al, 2005 ^210^ | | 1 | 1 | | 303.2 - 303.2 | | | 101.0 - 101.0 |
| PXYLENE | Rathnam et al, 2005 ^558^ | | 2 | 2 | | 303.2 - 313.2 | | | 101.0 - 101.0 |
| PXYLENE | Singh et al, 2005 ^429^ | | 1 | 1 | | 298.2 - 298.2 | | | 101.0 - 101.0 |
| PXYLENE | Ali et al, 2006 ^211^ | | 1 | 1 | | 308.2 - 308.2 | | | 101.0 - 101.0 |
| PXYLENE | Al-Kandary et al, 2006 ^1013^ | | 4 | 4 | | 288.2 - 303.2 | | | 101.0 - 101.0 |
| PXYLENE | Baragi Aralaguppi, 2006 ^337^ | | 3 | 3 | | 298.2 - 308.2 | | | 101.0 - 101.0 |
| PXYLENE | Di et al, 2006 ^1083^ | | 10 | 10 | | 298.2 - 353.2 | | | 101.0 - 101.0 |
| PXYLENE | Nain, 2007 ^219^ | | 4 | 4 | | 288.2 - 318.2 | | | 101.0 - 101.0 |
| PXYLENE | Nain, 2007 ^219^ | | 4 | 4 | | 288.2 - 318.2 | | | 101.0 - 101.0 |
| PXYLENE | Yang et al, 2007 ^1014^ | | 7 | 7 | | 298.2 - 353.2 | | | 101.0 - 101.0 |
| PXYLENE | Fang et al, 2008 ^511^ | | 39 | 39 | | 313.2 - 473.2 | | | 100.0 - 3200.0 |
| PXYLENE | Song et al, 2008 ^225^ | | 7 | 7 | | 303.2 - 333.2 | | | 101.0 - 101.0 |
| PXYLENE | Wankhede et al, 2008 ^226^ | | 3 | 3 | | 288.2 - 308.2 | | | 101.0 - 101.0 |
| PXYLENE | Dominguez-Perez et al, 2009 ^560^ | | 1 | 1 | | 298.2 - 298.2 | | | 101.0 - 101.0 |
| PXYLENE | Nain et al, 2009 ^229^ | | 1 | 1 | | 298.2 - 298.2 | | | 101.0 - 101.0 |
| PXYLENE | Rathnam et al, 2009 ^561^ | | 2 | 2 | | 303.2 - 313.2 | | | 101.0 - 101.0 |
| PXYLENE | Sastry et al, 2009 ^231^ | | 2 | 2 | | 298.2 - 308.2 | | | 101.0 - 101.0 |
| PXYLENE | Yan et al, 2009 ^232^ | | 3 | 3 | | 298.2 - 318.2 | | | 101.0 - 101.0 |
| PXYLENE | Habibullah et al, 2010 ^1016^ | | 3 | 3 | | 303.2 - 323.2 | | | 101.0 - 101.0 |
| PXYLENE | Habibullah et al, 2010 ^1016^ | | 2 | 2 | | 308.2 - 318.2 | | | 101.0 - 101.0 |
| PXYLENE | Ikeuchi et al, 2010 ^233^ | | 6 | 6 | | 288.1 - 313.1 | | | 101.0 - 101.0 |
| PXYLENE | Rathnam et al, 2010 ^562^ | | 2 | 2 | | 303.2 - 313.2 | | | 101.0 - 101.0 |
| PXYLENE | Yadava Yadava, 2010 ^438^ | | 1 | 1 | | 308.2 - 308.2 | | | 101.0 - 101.0 |
| PXYLENE | Bhatia et al, 2011 ^563^ | | 2 | 2 | | 298.2 - 308.2 | | | 101.0 - 101.0 |
| PXYLENE | Rathnam et al, 2011 ^564^ | | 2 | 2 | | 303.2 - 313.2 | | | 101.0 - 101.0 |
| PXYLENE | Yadava et al, 2011 ^238^ | | 1 | 1 | | 308.2 - 308.2 | | | 101.0 - 101.0 |
| PXYLENE | Yadava et al, 2012 ^240^ | | 2 | 2 | | 303.2 - 308.2 | | | 101.0 - 101.0 |
| PXYLENE | Zarei Salami, 2012 ^1017^ | | 1 | 1 | | 298.2 - 298.2 | | | 101.0 - 101.0 |
| PXYLENE | Dikio et al, 2013 ^1018^ | | 4 | 4 | | 293.2 - 323.2 | | | 101.0 - 101.0 |
| PXYLENE | Abdullaev Akhundov, 1983 ^153^ | | 28 | 0 | | 10000.0 - 0.0 | | | 1000000000.0 - 0.0 |
| PXYLENE | Vogel Hendl, 1992 ^1084^ | | 66 | 66 | | 338.1 - 634.6 | | | 8.0 - 196.0 |

| Material | | Author | *N*_all_ | | | *N*_used_ | | *T* / K | *p* / kPa | |
| --- | --- | --- | --- | --- | --- | --- | --- | --- | --- | --- |
| R11 | Phillips Murphy, 1970 ^640^ | | | 9 | 9 | | 209.1 - 351.7 | | | 1.0 - 511.0 |
| R11 | Lavochinik Solovey, 1980 ^1085^ | | | 12 | 12 | | 208.2 - 263.2 | | | 1.0 - 27.0 |
| R11 | Kumagai Takahashi, 1991 ^1086^ | | | 9 | 9 | | 273.2 - 353.2 | | | 42.0 - 530.0 |
| R11 | Benning Markwood, 1939 ^1087^ | | | 5 | 5 | | 274.6 - 352.6 | | | 30.0 - 101.0 |
| R11 | Kestin Wakeham, 1979 ^102^ | | | 5 | 5 | | 300.6 - 473.1 | | | 101.0 - 101.0 |
| R11 | Rivkin et al, 1971 ^1088^ | | | 32 | 32 | | 443.1 - 503.1 | | | 1068.0 - 11780.0 |
| R11 | Ivanchenko, 1974 ^1089^ | | | 93 | 93 | | 242.0 - 453.2 | | | 100.0 - 58940.0 |
| R11 | Geller et al, 1975 ^1090^ | | | 93 | 93 | | 242.0 - 453.2 | | | 100.0 - 58940.0 |
| R11 | Assael et al, 1994 ^656^ | | | 32 | 32 | | 273.1 - 333.3 | | | 1400.0 - 18900.0 |

| Material | | Author | *N*_all_ | | | *N*_used_ | | *T* / K | *p* / kPa | |
| --- | --- | --- | --- | --- | --- | --- | --- | --- | --- | --- |
| R113 | Geller, 1976 ^1091^ | | | 7 | 7 | | 263.1 - 323.0 | | | 10.0 - 115.0 |
| R113 | karbanov, 1978 ^1092^ | | | 14 | 14 | | 253.9 - 375.4 | | | 6.0 - 472.0 |
| R113 | Kumagai Takahashi, 1991 ^1086^ | | | 9 | 9 | | 273.2 - 353.2 | | | 17.0 - 273.0 |
| R113 | Hovorka Geiger, 1933 ^1093^ | | | 5 | 5 | | 273.2 - 313.1 | | | 101.0 - 101.0 |
| R113 | Ivanchenko, 1972 ^1094^ | | | 106 | 106 | | 243.1 - 473.1 | | | 100.0 - 60000.0 |
| R113 | Ivanchenko, 1974 ^1089^ | | | 81 | 81 | | 242.9 - 472.7 | | | 100.0 - 58940.0 |
| R113 | Kessel’man et al, 1976 ^809^ | | | 10 | 10 | | 272.6 - 293.0 | | | 100.0 - 9900.0 |
| R113 | Rasszakov et al, 1980 ^1095^ | | | 11 | 10 | | 238.2 - 317.1 | | | 101.0 - 101.0 |
| R113 | Benning Markwood, 1939 ^1087^ | | | 6 | 5 | | 304.0 - 352.5 | | | 10.0 - 30.0 |

| Material | Author | | *N*_all_ | | | *N*_used_ | | *T* / K | *p* / kPa | |
| --- | --- | --- | --- | --- | --- | --- | --- | --- | --- | --- |
| R114 | | Phillips Murphy, 1970 ^640^ | | 10 | 10 | | 198.3 - 331.1 | | | 1.0 - 558.0 |
| R114 | | Kumagai Takahashi, 1991 ^1086^ | | 9 | 9 | | 273.2 - 353.2 | | | 91.0 - 941.0 |
| R114 | | Geller, 1980 ^1096^ | | 90 | 90 | | 183.0 - 313.0 | | | 1080.0 - 58960.0 |
| R114 | | Arnemann Kruse, 1991 ^1097^ | | 7 | 2 | | 255.1 - 273.7 | | | 101.0 - 101.0 |
| R114 | | Bondar’, 1984 ^1098^ | | 89 | 89 | | 183.0 - 313.0 | | | 1080.0 - 58950.0 |

| Material | Author | | *N*_all_ | | | *N*_used_ | | *T* / K | *p* / kPa | |
| --- | --- | --- | --- | --- | --- | --- | --- | --- | --- | --- |
| R115 | | Phillips Murphy, 1970 ^640^ | | 8 | 8 | | 198.6 - 303.4 | | | 15.0 - 1039.0 |
| R115 | | Karbanov, 1978 ^1092^ | | 11 | 11 | | 183.4 - 283.1 | | | 5.0 - 597.0 |
| R115 | | Geller, 1980 ^1096^ | | 80 | 80 | | 176.9 - 333.2 | | | 3040.0 - 58960.0 |
| R115 | | Kronberg, 1979 ^1099^ | | 68 | 68 | | 281.6 - 402.9 | | | 101.0 - 3176.0 |
| R115 | | Nagaoka et al, 1986 ^1076^ | | 3 | 3 | | 298.1 - 348.1 | | | 101.0 - 101.0 |
| R115 | | Bondar’, 1984 ^1098^ | | 79 | 79 | | 176.9 - 333.2 | | | 3040.0 - 58960.0 |

| Material | Author | | *N*_all_ | | | *N*_used_ | | *T* / K | *p* / kPa | |
| --- | --- | --- | --- | --- | --- | --- | --- | --- | --- | --- |
| R116 | | Hurly et al, 2003 ^105^ | | 181 | 181 | | 225.0 - 375.0 | | | 81.0 - 3181.0 |

| Material | Author | | *N*_all_ | | | *N*_used_ | | *T* / K | *p* / kPa | |
| --- | --- | --- | --- | --- | --- | --- | --- | --- | --- | --- |
| R12 | | Phillips Murphy, 1970 ^640^ | | 9 | 9 | | 201.7 - 312.5 | | | 12.0 - 947.0 |
| R12 | | Lavochnik Solovey, 1980 ^1085^ | | 7 | 7 | | 208.2 - 238.2 | | | 17.0 - 82.0 |
| R12 | | Kumagai Takahashi, 1991 ^1086^ | | 8 | 8 | | 273.2 - 343.2 | | | 310.0 - 1894.0 |
| R12 | | Awbery Griffiths, 1936 ^1100^ | | 10 | 10 | | 258.1 - 303.1 | | | 183.0 - 746.0 |
| R12 | | Ivanchenko, 1974 ^1089^ | | 107 | 107 | | 243.5 - 432.5 | | | 1080.0 - 58940.0 |
| R12 | | Geller et al, 1975 ^1090^ | | 107 | 107 | | 243.5 - 432.5 | | | 1080.0 - 58940.0 |
| R12 | | Assael, 1994 ^656^ | | 32 | 32 | | 273.1 - 333.3 | | | 1150.0 - 15100.0 |
| R12 | | Benning Markwood, 1939 ^1087^ | | 4 | 4 | | 274.6 - 352.6 | | | 101.0 - 101.0 |
| R12 | | Zaloudik, 1962 ^1101^ | | 7 | 7 | | 283.1 - 343.1 | | | 101.0 - 101.0 |
| R12 | | Srichand et al, 1970 ^1102^ | | 1 | 1 | | 299.9 - 299.9 | | | 101.0 - 101.0 |
| R12 | | Nagaoka et al, 1986 ^1076^ | | 2 | 2 | | 298.1 - 323.1 | | | 101.0 - 101.0 |
| R12 | | Chaikovskii et al, 1973 ^1103^ | | 130 | 130 | | 243.1 - 433.1 | | | 1000.0 - 60000.0 |

| Material | Author | | *N*_all_ | | | *N*_used_ | | *T* / K | *p* / kPa | |
| --- | --- | --- | --- | --- | --- | --- | --- | --- | --- | --- |
| R1224YDZ | | Alam et al, 2019 ^1104^ | | 109 | 105 | | 303.1 - 474.8 | | | 587.0 - 4058.0 |

| Material | | Author | *N*_all_ | | | *N*_used_ | | *T* / K | *p* / kPa | |
| --- | --- | --- | --- | --- | --- | --- | --- | --- | --- | --- |
| R123 | Matsuo et al, 1988 ^1105^ | | | 2 | 2 | | 293.0 - 313.0 | | | 77.0 - 155.0 |
| R123 | Kumagai Takahashi, 1991 ^1086^ | | | 9 | 9 | | 273.2 - 353.2 | | | 34.0 - 490.0 |
| R123 | Diller et al, 1993 ^1106^ | | | 23 | 21 | | 190.0 - 320.0 | | | 0.0 - 194.0 |
| R123 | Nabizadeh Mayinger, 1990 ^1107^ | | | 31 | 30 | | 303.1 - 423.4 | | | 101.0 - 2028.0 |
| R123 | Nabizadeh Mayinger, 1992 ^1108^ | | | 31 | 30 | | 303.1 - 423.4 | | | 101.0 - 2028.0 |
| R123 | Dowdell Matthews, 1993 ^1109^ | | | 4 | 4 | | 308.2 - 363.2 | | | 101.0 - 101.0 |
| R123 | Yokoyama et al, 2000 ^1110^ | | | 42 | 42 | | 323.2 - 423.2 | | | 100.0 - 1965.0 |
| R123 | Diller et al, 1993 ^1106^ | | | 29 | 29 | | 200.0 - 300.0 | | | 3235.0 - 33610.0 |
| R123 | Nakazawa et al, 2002 ^1111^ | | | 1 | 1 | | 287.4 - 287.4 | | | 101.0 - 101.0 |

| Material | Author | | *N*_all_ | | | *N*_used_ | | *T* / K | *p* / kPa | |
| --- | --- | --- | --- | --- | --- | --- | --- | --- | --- | --- |
| R1233ZDE | | Meng et al, 2018 ^1112^ | | 92 | 78 | | 243.2 - 373.2 | | | 200.0 - 40000.0 |

| Material | Author | | *N*_all_ | | | *N*_used_ | | *T* / K | *p* / kPa | |
| --- | --- | --- | --- | --- | --- | --- | --- | --- | --- | --- |
| R1234YF | | Hulse et al, 2009 ^1113^ | | 39 | 39 | | 257.5 - 307.5 | | | 326.0 - 2109.0 |
| R1234YF | | Cousins Laesecke, 2012 ^545^ | | 20 | 20 | | 246.5 - 340.0 | | | 115.0 - 1916.0 |
| R1234YF | | Meng et al, 2013 ^1114^ | | 110 | 105 | | 243.2 - 363.2 | | | 151.0 - 30000.0 |

| Material | Author | | *N*_all_ | | | *N*_used_ | | *T* / K | *p* / kPa | |
| --- | --- | --- | --- | --- | --- | --- | --- | --- | --- | --- |
| R1234ZEE | | Grebenkov et al, 2009 ^1115^ | | 53 | 53 | | 257.1 - 368.9 | | | 100.0 - 6080.0 |
| R1234ZEE | | Cousins Laesecke, 2012 ^545^ | | 20 | 20 | | 246.7 - 340.0 | | | 74.0 - 1504.0 |
| R1234ZEE | | Meng et al, 2013 ^1114^ | | 119 | 105 | | 243.2 - 373.2 | | | 1000.0 - 30000.0 |

| Material | | Author | *N*_all_ | | | *N*_used_ | | *T* / K | *p* / kPa | |
| --- | --- | --- | --- | --- | --- | --- | --- | --- | --- | --- |
| R124 | Diller Peterson, 1993 ^1116^ | | | 32 | 27 | | 170.0 - 385.0 | | | 0.0 - 2991.0 |
| R124 | Ripple Matar, 1993 ^1117^ | | | 14 | 14 | | 251.7 - 313.1 | | | 68.0 - 593.0 |
| R124 | Diller Peterson, 1993 ^1116^ | | | 103 | 103 | | 150.0 - 420.0 | | | 3439.0 - 56170.0 |
| R124 | Assael et al, 1994 ^656^ | | | 32 | 32 | | 273.1 - 333.2 | | | 1500.0 - 16700.0 |
| R124 | Dowdell Matthews, 1993 ^1109^ | | | 7 | 7 | | 283.2 - 403.2 | | | 101.0 - 101.0 |
| R124 | Assael Polimatidou, 1997 ^1118^ | | | 14 | 14 | | 273.5 - 333.2 | | | 121.0 - 922.0 |

| Material | | Author | *N*_all_ | | | *N*_used_ | | *T* / K | *p* / kPa | |
| --- | --- | --- | --- | --- | --- | --- | --- | --- | --- | --- |
| R125 | Diller Peterson, 1993 ^1116^ | | | 32 | 32 | | 176.0 - 330.0 | | | 4.0 - 2964.0 |
| R125 | Oliveria Wakeham, 1993 ^1119^ | | | 13 | 12 | | 252.0 - 323.1 | | | 324.0 - 2542.0 |
| R125 | Ripple Matar, 1993 ^1117^ | | | 15 | 15 | | 250.1 - 302.0 | | | 302.0 - 1526.0 |
| R125 | Heide Schenk, 1996 ^1120^ | | | 12 | 12 | | 223.6 - 333.2 | | | 96.0 - 3174.0 |
| R125 | Oliveria Wakeham, 1993 ^1119^ | | | 13 | 13 | | 224.0 - 332.0 | | | 98.0 - 3095.0 |
| R125 | Wilson et al, 1992 ^1121^ | | | 6 | 6 | | 216.5 - 333.2 | | | 2800.0 - 3400.0 |
| R125 | Diller Peterson, 1993 ^1116^ | | | 105 | 105 | | 200.0 - 420.0 | | | 3307.0 - 53070.0 |
| R125 | Assael et al, 1994 ^656^ | | | 22 | 22 | | 273.1 - 313.3 | | | 2450.0 - 14450.0 |
| R125 | Ripple Defibaugh, 1997 ^1122^ | | | 8 | 8 | | 256.0 - 303.1 | | | 376.0 - 1570.0 |
| R125 | Avelino et al, 2006 ^1123^ | | | 116 | 116 | | 254.0 - 293.1 | | | 1430.0 - 10080.0 |
| R125 | Wilson et al, 1992 ^1121^ | | | 5 | 5 | | 310.9 - 422.0 | | | 90.0 - 90.0 |
| R125 | Assael Polimatidou, 1997 ^1118^ | | | 24 | 24 | | 274.5 - 313.2 | | | 128.0 - 1285.0 |
| R125 | Oliveria Wakeham, 1999 ^1124^ | | | 13 | 13 | | 224.0 - 332.0 | | | 98.0 - 3095.0 |
| R125 | Takahashi et al, 1999 ^1125^ | | | 131 | 131 | | 298.2 - 423.2 | | | 102.0 - 8398.0 |

| Material | | Author | *N*_all_ | | | *N*_used_ | | *T* / K | *p* / kPa | |
| --- | --- | --- | --- | --- | --- | --- | --- | --- | --- | --- |
| R13 | Phillips Murphy, 1970 ^640^ | | | 8 | 6 | | 191.9 - 250.4 | | | 104.0 - 1056.0 |
| R13 | Reed Rabinovitch, 1957 ^1126^ | | | 1 | 1 | | 298.1 - 298.1 | | | 101.0 - 101.0 |
| R13 | Ivanchenko et al, 1974 ^1127^ | | | 3 | 3 | | 253.0 - 293.0 | | | 101.0 - 101.0 |
| R13 | Kronberg, 1979 ^1099^ | | | 66 | 66 | | 245.9 - 344.0 | | | 101.0 - 3525.0 |
| R13 | Takahashi et al, 1985 ^1128^ | | | 170 | 170 | | 273.2 - 373.1 | | | 101.0 - 10410.0 |
| R13 | Nagaoka et al, 1986 ^1076^ | | | 2 | 2 | | 298.1 - 323.1 | | | 101.0 - 101.0 |
| R13 | Ivanchenko et al, 1974 ^1127^ | | | 108 | 107 | | 243.8 - 433.3 | | | 110.0 - 58880.0 |
| R13 | Geller et al, 1975 ^1090^ | | | 108 | 104 | | 243.8 - 433.3 | | | 110.0 - 58880.0 |
| R13 | Chajkovskij et al, 1977 ^1129^ | | | 101 | 92 | | 91.1 - 299.2 | | | 0.0 - 58960.0 |
| R13 | Geller, 1980 ^1096^ | | | 118 | 118 | | 89.4 - 299.2 | | | 0.0 - 58960.0 |
| R13 | Diller Van Poolen, 1989 ^1130^ | | | 25 | 23 | | 120.0 - 300.0 | | | 0.0 - 3708.0 |
| R13 | Bondar’, 1984 ^1098^ | | | 118 | 118 | | 89.4 - 299.2 | | | 3530.0 - 58960.0 |
| R13 | Diller Van Poolen, 1989 ^1130^ | | | 56 | 15 | | 150.0 - 250.0 | | | 6.0 - 1044.0 |

| Material | Author | | *N*_all_ | | | *N*_used_ | | *T* / K | *p* / kPa | |
| --- | --- | --- | --- | --- | --- | --- | --- | --- | --- | --- |
| R1336MZZZ | | Alam et al, 2018 ^1131^ | | 97 | 84 | | 313.8 - 474.7 | | | 492.0 - 4062.0 |

| Material | | Author | *N*_all_ | | | *N*_used_ | | *T* / K | *p* / kPa | |
| --- | --- | --- | --- | --- | --- | --- | --- | --- | --- | --- |
| R134A | Kumagai Takahashi, 1991 ^1086^ | | | 8 | 8 | | 273.2 - 343.2 | | | 290.0 - 2114.0 |
| R134A | Diller et al, 1993 ^1106^ | | | 30 | 30 | | 175.0 - 320.0 | | | 1.0 - 1210.0 |
| R134A | Ripple Matar, 1993 ^1117^ | | | 14 | 14 | | 250.1 - 306.1 | | | 115.0 - 830.0 |
| R134A | Han et al, 1995 ^1132^ | | | 21 | 21 | | 233.2 - 333.2 | | | 51.0 - 1677.0 |
| R134A | Heide Schenk, 1996 ^1120^ | | | 14 | 14 | | 223.1 - 353.2 | | | 30.0 - 2634.0 |
| R134A | Padua et al, 1996 ^418^ | | | 5 | 5 | | 198.1 - 298.1 | | | 6.0 - 659.0 |
| R134A | Huber et al, 1999 ^1133^ | | | 91 | 91 | | 240.9 - 350.1 | | | 75.0 - 2466.0 |
| R134A | Assael et al, 1994 ^656^ | | | 7 | 7 | | 273.2 - 333.2 | | | 290.0 - 1677.0 |
| R134A | Nabizadeh Mayinger, 1990 ^1107^ | | | 41 | 41 | | 302.9 - 424.5 | | | 100.0 - 6425.0 |
| R134A | Nabizadeh Mayinger, 1992 ^1108^ | | | 41 | 41 | | 302.9 - 424.5 | | | 100.0 - 6425.0 |
| R134A | Dowdell Matthews, 1993 ^1109^ | | | 6 | 6 | | 308.2 - 403.2 | | | 101.0 - 101.0 |
| R134A | Pasekov Ustjushanin, 1994 ^1134^ | | | 37 | 37 | | 275.3 - 370.5 | | | 277.0 - 595.0 |
| R134A | Assael Polimatidou, 1997 ^1118^ | | | 22 | 22 | | 273.9 - 333.2 | | | 138.0 - 1473.0 |
| R134A | Shibasaki-Kitakawa et al, 1998 ^1135^ | | | 126 | 126 | | 298.2 - 423.2 | | | 100.0 - 5634.0 |
| R134A | Oliveria Wakeham, 1999 ^1124^ | | | 14 | 14 | | 243.0 - 342.8 | | | 83.0 - 2096.0 |
| R134A | Ruvinskii et al, 1990 ^1136^ | | | 11 | 11 | | 381.9 - 404.6 | | | 665.0 - 6235.0 |
| R134A | Lavrenchenko et al, 1992 ^1137^ | | | 34 | 34 | | 257.6 - 404.6 | | | 318.0 - 6437.0 |
| R134A | Laesecke Bair, 2011 ^1138^ | | | 59 | 47 | | 293.2 - 438.2 | | | 10000.0 - 300000.0 |
| R134A | Ruvinskii et al, 1990 ^1136^ | | | 23 | 23 | | 257.6 - 360.8 | | | 318.0 - 6437.0 |
| R134A | Diller et al, 1993 ^1106^ | | | 63 | 63 | | 200.0 - 300.0 | | | 623.0 - 33695.0 |
| R134A | Oliveria Wakeham, 1993 ^1119^ | | | 54 | 54 | | 237.7 - 343.2 | | | 92.0 - 51010.0 |
| R134A | Assael et al, 1994 ^656^ | | | 32 | 32 | | 273.2 - 333.3 | | | 950.0 - 14600.0 |
| R134A | Padua et al, 1996 ^418^ | | | 37 | 37 | | 248.2 - 298.1 | | | 730.0 - 100100.0 |
| R134A | Comunas et al, 2003 ^1139^ | | | 70 | 70 | | 293.2 - 373.2 | | | 10000.0 - 140000.0 |
| R134A | Meng et al, 2011 ^1140^ | | | 43 | 43 | | 258.2 - 338.1 | | | 1000.0 - 40000.0 |
| R134A | Meng et al, 2013 ^1114^ | | | 54 | 54 | | 253.1 - 353.2 | | | 1000.0 - 30000.0 |
| R134A | Wilhelm Vogel, 1996 ^1141^ | | | 71 | 71 | | 297.3 - 438.5 | | | 29.0 - 321.0 |

| Material | | Author | *N*_all_ | | | *N*_used_ | | *T* / K | *p* / kPa | |
| --- | --- | --- | --- | --- | --- | --- | --- | --- | --- | --- |
| R14 | Raw Tang, 1963 ^1142^ | | | 4 | 4 | | 303.1 - 342.0 | | | 101.0 - 101.0 |
| R14 | Genot, 1970 ^1143^ | | | 9 | 9 | | 245.7 - 343.1 | | | 101.0 - 101.0 |
| R14 | Hellmans et al, 1973 ^938^ | | | 9 | 9 | | 298.1 - 873.0 | | | 101.0 - 101.0 |
| R14 | Ivanchenko et al, 1974 ^1127^ | | | 8 | 8 | | 293.0 - 433.0 | | | 101.0 - 101.0 |
| R14 | Filatov, 1975 ^1144^ | | | 98 | 98 | | 203.2 - 523.1 | | | 0.0 - 30000.0 |
| R14 | Geller et al, 1975 ^1090^ | | | 75 | 74 | | 293.2 - 432.7 | | | 108.0 - 39280.0 |
| R14 | Kestin et al, 1976 ^393^ | | | 3 | 3 | | 296.1 - 477.1 | | | 101.0 - 101.0 |
| R14 | Kestin et al, 1977 ^256^ | | | 5 | 5 | | 298.1 - 668.1 | | | 101.0 - 101.0 |
| R14 | Kochubey Moin, 1978 ^1145^ | | | 1 | 1 | | 273.0 - 273.0 | | | 101.0 - 101.0 |
| R14 | Rasskazov et al, 1980 ^1095^ | | | 10 | 10 | | 203.2 - 473.1 | | | 101.0 - 101.0 |
| R14 | Nagaoka et al, 1986 ^1076^ | | | 2 | 2 | | 298.1 - 323.1 | | | 101.0 - 101.0 |
| R14 | Ivanchenko et al, 1974 ^1127^ | | | 75 | 75 | | 293.2 - 432.7 | | | 110.0 - 39280.0 |
| R14 | Rasskazov et al, 1975 ^1146^ | | | 78 | 78 | | 203.2 - 523.1 | | | 100.0 - 30000.0 |

| Material | | Author | *N*_all_ | | | *N*_used_ | | *T* / K | *p* / kPa | |
| --- | --- | --- | --- | --- | --- | --- | --- | --- | --- | --- |
| R141B | Kumagai Takahashi, 1990 ^1147^ | | | 9 | 9 | | 273.2 - 353.2 | | | 29.0 - 420.0 |
| R141B | Diller et al, 1993 ^1106^ | | | 22 | 19 | | 190.0 - 320.0 | | | 0.0 - 166.0 |
| R141B | Kumagai Takahashi, 1993 ^1148^ | | | 9 | 9 | | 273.2 - 353.2 | | | 29.0 - 420.0 |
| R141B | Diller et al, 1993 ^1106^ | | | 25 | 25 | | 200.0 - 300.0 | | | 3444.0 - 30176.0 |
| R141B | Assael et al, 1994 ^656^ | | | 32 | 32 | | 273.2 - 333.2 | | | 850.0 - 18900.0 |
| R142B | Mears et al, 1955 ^1149^ | | | 6 | 4 | | 242.3 - 293.1 | | | 39.0 - 287.0 |
| R142B | Kumagai Takahashi, 1993 ^1148^ | | | 9 | 9 | | 273.2 - 353.2 | | | 144.0 - 1380.0 |
| R142B | Konrad, 1985 ^1150^ | | | 5 | 5 | | 313.1 - 433.1 | | | 101.0 - 101.0 |
| R142B | Takahashi et al, 1987 ^1151^ | | | 96 | 96 | | 298.1 - 423.1 | | | 102.0 - 5092.0 |
| R142B | Nabizadeh Mayinger, 1990 ^1107^ | | | 29 | 29 | | 303.5 - 372.7 | | | 100.0 - 1988.0 |
| R142B | Nabizadeh Mayinger, 1992 ^1108^ | | | 29 | 29 | | 303.5 - 372.7 | | | 100.0 - 1988.0 |
| R142B | Arnemann Kruse, 1991 ^1097^ | | | 16 | 16 | | 239.4 - 352.5 | | | 34.0 - 1359.0 |

| Material | | Author | *N*_all_ | | | *N*_used_ | | *T* / K | *p* / kPa | |
| --- | --- | --- | --- | --- | --- | --- | --- | --- | --- | --- |
| R143A | Kumagai Takahashi, 1991 ^1086^ | | | 6 | 6 | | 273.2 - 323.2 | | | 606.0 - 2299.0 |
| R143A | Heide Schenk, 1996 ^1120^ | | | 12 | 12 | | 223.7 - 333.2 | | | 87.0 - 2869.0 |
| R143A | Takahashi et al, 1999 ^1125^ | | | 97 | 97 | | 298.2 - 423.2 | | | 101.0 - 8852.0 |
| R143A | Wang et al, 2009 ^1152^ | | | 33 | 33 | | 299.5 - 338.8 | | | 104.0 - 2690.0 |
| R143A | Ripple Defibaugh, 1997 ^1122^ | | | 10 | 10 | | 255.6 - 308.4 | | | 335.0 - 1621.0 |
| R143A | Avelino et al, 2006 ^1123^ | | | 172 | 172 | | 254.4 - 293.2 | | | 1240.0 - 10070.0 |

| Material | | Author | *N*_all_ | | | *N*_used_ | | *T* / K | *p* / kPa | |
| --- | --- | --- | --- | --- | --- | --- | --- | --- | --- | --- |
| R150 | Thorpe Rodger, 1894 ^28^ | | | 12 | 12 | | 273.5 - 354.2 | | | 3.0 - 91.0 |
| R150 | Batshinski, 1913 ^29^ | | | 9 | 9 | | 273.1 - 353.1 | | | 3.0 - 88.0 |
| R150 | Faust, 1912 ^33^ | | | 3 | 3 | | 273.2 - 323.1 | | | 101.0 - 101.0 |
| R150 | Timmermans Martin, 1928 ^37^ | | | 2 | 2 | | 288.1 - 303.1 | | | 101.0 - 101.0 |
| R150 | Kireev Skvortsova, 1936 ^134^ | | | 1 | 1 | | 293.1 - 293.1 | | | 101.0 - 101.0 |
| R150 | Stern Martell, 1955 ^1153^ | | | 3 | 3 | | 278.8 - 308.1 | | | 101.0 - 101.0 |
| R150 | Alluraiah et al, 1989 ^1154^ | | | 1 | 1 | | 303.1 - 303.1 | | | 101.0 - 101.0 |
| R150 | Malhotra et al, 1990 ^1155^ | | | 3 | 3 | | 298.2 - 323.2 | | | 100.0 - 100.0 |
| R150 | Schumpe Luehring, 1990 ^54^ | | | 1 | 1 | | 293.2 - 293.2 | | | 101.0 - 101.0 |
| R150 | Sivaramprasad et al, 1990 ^170^ | | | 4 | 4 | | 303.2 - 333.2 | | | 101.0 - 101.0 |
| R150 | Klofutar et al, 1992 ^176^ | | | 6 | 6 | | 293.0 - 333.0 | | | 101.0 - 101.0 |
| R150 | Nath Chaudhari, 1992 ^411^ | | | 1 | 1 | | 303.2 - 303.2 | | | 101.0 - 101.0 |
| R150 | Krishnaiah Surendranath, 1996 ^1156^ | | | 1 | 1 | | 303.2 - 303.2 | | | 101.0 - 101.0 |
| R150 | Zhang Han, 1997 ^1157^ | | | 1 | 1 | | 303.2 - 303.2 | | | 101.0 - 101.0 |
| R150 | Mussari et al, 2000 ^1158^ | | | 1 | 1 | | 298.2 - 298.2 | | | 101.0 - 101.0 |
| R150 | Oswal et al, 2000 ^1159^ | | | 1 | 1 | | 303.2 - 303.2 | | | 101.0 - 101.0 |
| R150 | Aznarez et al, 2002 ^1160^ | | | 1 | 1 | | 298.2 - 298.2 | | | 101.0 - 101.0 |
| R150 | Iloukhani et al, 2005 ^428^ | | | 1 | 1 | | 298.2 - 298.2 | | | 101.0 - 101.0 |
| R150 | Sathyanarayana et al, 2007 ^1161^ | | | 1 | 1 | | 308.2 - 308.2 | | | 101.0 - 101.0 |
| R150 | Bhatia et al, 2009 ^1162^ | | | 1 | 1 | | 293.2 - 293.2 | | | 101.0 - 101.0 |
| R150 | Ranjbar et al, 2009 ^1163^ | | | 6 | 6 | | 288.2 - 313.2 | | | 101.0 - 101.0 |
| R150 | Ni et al, 2010 ^382^ | | | 4 | 4 | | 298.2 - 313.2 | | | 101.0 - 101.0 |
| R150 | Syeda et al, 2010 ^1164^ | | | 1 | 1 | | 298.2 - 298.2 | | | 101.0 - 101.0 |

| Material | | Author | *N*_all_ | | | *N*_used_ | | *T* / K | *p* / kPa | |
| --- | --- | --- | --- | --- | --- | --- | --- | --- | --- | --- |
| R152A | Phillips Murphy, 1970 ^640^ | | | 9 | 9 | | 200.2 - 318.2 | | | 6.0 - 1016.0 |
| R152A | Karbanov, 1978 ^1092^ | | | 5 | 5 | | 206.8 - 242.4 | | | 9.0 - 71.0 |
| R152A | Kumagai Takahashi, 1991 ^1086^ | | | 8 | 8 | | 273.2 - 343.2 | | | 254.0 - 1866.0 |
| R152A | VanderGulik, 1995 ^1165^ | | | 35 | 35 | | 243.2 - 373.0 | | | 74.0 - 3483.0 |
| R152A | Heide Schenk, 1996 ^1120^ | | | 12 | 12 | | 223.2 - 333.2 | | | 26.0 - 1479.0 |
| R152A | VanderGulik, 1995 ^1165^ | | | 33 | 33 | | 243.0 - 373.1 | | | 73.0 - 3491.0 |
| R152A | Arnemann Kruse, 1991 ^1097^ | | | 12 | 12 | | 242.5 - 352.3 | | | 71.0 - 2281.0 |
| R152A | Assael, 1994 ^656^ | | | 32 | 32 | | 273.1 - 333.3 | | | 1450.0 - 17700.0 |
| R152A | Ripple Defibaugh, 1997 ^1122^ | | | 11 | 11 | | 254.7 - 323.0 | | | 123.0 - 1151.0 |
| R152A | Lapardin, 1982 ^1166^ | | | 29 | 29 | | 386.8 - 433.4 | | | 102.0 - 50110.0 |
| R152A | Lapardin, 1983 ^1167^ | | | 91 | 89 | | 156.3 - 433.4 | | | 100.0 - 50110.0 |
| R152A | Nagaoka et al, 1986 ^1076^ | | | 2 | 2 | | 298.1 - 323.1 | | | 101.0 - 101.0 |
| R152A | Takahashi et al, 1987 ^1151^ | | | 113 | 113 | | 298.1 - 423.1 | | | 101.0 - 5282.0 |
| R152A | Takahashi et al, 1987 ^1151^ | | | 49 | 47 | | 273.2 - 303.1 | | | 101.0 - 580.0 |
| R152A | Assael Polimatidou, 1997 ^1118^ | | | 21 | 21 | | 275.2 - 333.2 | | | 193.0 - 1242.0 |
| R152A | Wang et al, 1997 ^1168^ | | | 16 | 16 | | 333.9 - 374.1 | | | 98.0 - 2348.0 |

| Material | | Author | *N*_all_ | | | *N*_used_ | | *T* / K | *p* / kPa | |
| --- | --- | --- | --- | --- | --- | --- | --- | --- | --- | --- |
| R161 | Lv et al, 2014 ^1042^ | | | 97 | 97 | | 293.3 - 369.5 | | | 100.0 - 3806.0 |

| Material | | Author | *N*_all_ | | | *N*_used_ | | *T* / K | *p* / kPa | |
| --- | --- | --- | --- | --- | --- | --- | --- | --- | --- | --- |
| R21 | Benning Markwood, 1939 ^1087^ | | | 7 | 7 | | 274.6 - 352.6 | | | 30.0 - 101.0 |
| R21 | Geller et al, 1975 ^1090^ | | | 65 | 64 | | 293.0 - 433.8 | | | 1070.0 - 58920.0 |
| R21 | Rasskazov et al, 1980 ^1095^ | | | 7 | 7 | | 303.1 - 473.1 | | | 101.0 - 101.0 |
| R21 | Phillips Murphy, 1970 ^640^ | | | 11 | 11 | | 207.8 - 346.7 | | | 2.0 - 728.0 |
| R21 | Ivanchenko et al, 1974 ^1127^ | | | 65 | 65 | | 273.0 - 433.8 | | | 1070.0 - 58920.0 |
| R21 | Rasskazov et al, 1980 ^1095^ | | | 13 | 13 | | 173.2 - 281.2 | | | 101.0 - 101.0 |
| R21 | Filatov, 1975 ^1144^ | | | 58 | 58 | | 273.2 - 473.1 | | | 100.0 - 20000.0 |
| R21 | Rasskazov et al, 1975 ^1146^ | | | 47 | 47 | | 273.2 - 473.1 | | | 100.0 - 20000.0 |

| Material | | Author | *N*_all_ | | | *N*_used_ | | *T* / K | *p* / kPa | |
| --- | --- | --- | --- | --- | --- | --- | --- | --- | --- | --- |
| R218 | Geller, 1980 ^1096^ | | | 193 | 191 | | 126.0 - 343.1 | | | 1080.0 - 58960.0 |
| R218 | Lapardin, 1983 ^1167^ | | | 71 | 68 | | 303.7 - 421.4 | | | 100.0 - 50190.0 |
| R218 | Bondar’, 1984 ^1098^ | | | 100 | 99 | | 126.0 - 343.1 | | | 1080.0 - 58960.0 |

| Material | | Author | *N*_all_ | | | *N*_used_ | | *T* / K | *p* / kPa | |
| --- | --- | --- | --- | --- | --- | --- | --- | --- | --- | --- |
| R22 | Phillips Murphy, 1970 ^640^ | | | 8 | 8 | | 200.7 - 299.1 | | | 18.0 - 1064.0 |
| R22 | Geller et al, 1973 ^1169^ | | | 12 | 12 | | 253.1 - 363.1 | | | 243.0 - 4438.0 |
| R22 | Geller et al, 196 ^1091^ | | | 6 | 6 | | 191.6 - 228.0 | | | 10.0 - 82.0 |
| R22 | Lavochinik Solovey, 1980 ^1085^ | | | 5 | 5 | | 208.2 - 228.2 | | | 28.0 - 83.0 |
| R22 | Kumagai Takahashi, 1991 ^1086^ | | | 6 | 6 | | 273.2 - 323.2 | | | 494.0 - 1938.0 |
| R22 | Diller et al, 1993 ^1106^ | | | 23 | 20 | | 150.0 - 320.0 | | | 0.0 - 1800.0 |
| R22 | Butryskaya et al, 1970 ^1170^ | | | 8 | 8 | | 253.2 - 443.1 | | | 101.0 - 101.0 |
| R22 | Srichand et al, 1970 ^1102^ | | | 1 | 1 | | 299.8 - 299.8 | | | 101.0 - 101.0 |
| R22 | Kestin Wakeham, 1979 ^102^ | | | 5 | 5 | | 300.6 - 473.1 | | | 101.0 - 101.0 |
| R22 | Kronberg, 1979^1099^ | | | 152 | 151 | | 283.5 - 423.2 | | | 101.0 - 5065.0 |
| R22 | Takahashi et al, 1983 ^1171^ | | | 150 | 149 | | 273.2 - 398.1 | | | 0.0 - 6090.0 |
| R22 | Nagaoka et al, 1986 ^1076^ | | | 5 | 5 | | 298.1 - 348.1 | | | 101.0 - 101.0 |
| R22 | Takahashi et al, 1987 ^1151^ | | | 55 | 55 | | 273.2 - 303.1 | | | 101.0 - 685.0 |
| R22 | Zhang et al, 2002 ^1172^ | | | 19 | 19 | | 296.0 - 373.3 | | | 96.0 - 2202.0 |
| R22 | Phillips Murphy, 1970 ^640^ | | | 8 | 8 | | 200.7 - 299.1 | | | 18.0 - 1064.0 |
| R22 | Geller et al, 1973 ^1169^ | | | 124 | 124 | | 253.1 - 368.1 | | | 1000.0 - 60000.0 |
| R22 | Ivanchenko et al, 1974 ^1127^ | | | 57 | 57 | | 254.9 - 368.6 | | | 1050.0 - 58910.0 |
| R22 | Chaikovskiy et al, 1976 ^1173^ | | | 95 | 95 | | 115.6 - 313.2 | | | 1570.0 - 58960.0 |
| R22 | Geller et al, 1980 ^1096^ | | | 95 | 95 | | 115.6 - 313.2 | | | 1229.0 - 58960.0 |
| R22 | Hongo Takahashi, 1982 ^1174^ | | | 48 | 48 | | 298.1 - 373.1 | | | 101.0 - 4821.0 |
| R22 | Arnemann Kruse, 1991 ^1097^ | | | 6 | 6 | | 241.8 - 333.4 | | | 154.0 - 2436.0 |
| R22 | Diller et al, 1993 ^1106^ | | | 77 | 77 | | 150.0 - 320.0 | | | 947.0 - 31463.0 |
| R22 | Assael et al, 1994 ^656^ | | | 32 | 32 | | 273.1 - 333.3 | | | 1300.0 - 15000.0 |
| R22 | Bondar’, 1984 ^1098^ | | | 95 | 95 | | 115.6 - 313.2 | | | 0.0 - 58960.0 |

| Material | | Author | | *N*_all_ | | | *N*_used_ | | *T* / K | *p* / kPa | |
| --- | --- | --- | --- | --- | --- | --- | --- | --- | --- | --- | --- |
| R227EA | Laesecke Hafer, 1998 ^1175^ | | | | 100 | 100 | | 248.2 - 348.1 | | | 70.0 - 1671.0 |
| R227EA | Liu et al, 1999 ^1176^ | | 18 | | | 18 | | 263.2 - 343.2 | | | 134.0 - 1493.0 |
| R227EA | Tang et al, 2012 ^1177^ | | 52 | | | 51 | | 243.2 - 363.2 | | | 540.0 - 11490.0 |

| Material | | Author | *N*_all_ | | | *N*_used_ | | *T* / K | *p* / kPa | |
| --- | --- | --- | --- | --- | --- | --- | --- | --- | --- | --- |
| R23 | Benning Markwood, 1939 ^1087^ | | | 7 | 7 | | 274.6 - 352.6 | | | 101.0 - 912.0 |
| R23 | McCoubrey Singh, 1957 ^1178^ | | | 10 | 10 | | 314.6 - 455.8 | | | 101.0 - 101.0 |
| R23 | Tomassini et al, 1970 ^1179^ | | | 1 | 1 | | 300.0 - 300.0 | | | 101.0 - 101.0 |
| R23 | Rasskazov et al, 1974 ^1180^ | | | 15 | 15 | | 256.2 - 453.1 | | | 101.0 - 101.0 |
| R23 | Rasskazov et al, 1975 ^1146^ | | | 24 | 24 | | 263.2 - 453.1 | | | 303.0 - 8000.0 |
| R23 | Sagaidakova, 1977 ^1181^ | | | 6 | 6 | | 343.1 - 343.1 | | | 100.0 - 3890.0 |
| R23 | Kochubey Moin, 1978 ^1145^ | | | 1 | 1 | | 273.0 - 273.0 | | | 101.0 - 101.0 |
| R23 | Rasskazov et al, 1980 ^1095^ | | | 15 | 15 | | 203.2 - 453.1 | | | 101.0 - 101.0 |
| R23 | Phillips Murphy, 1970 ^640^ | | | 7 | 1 | | 256.9 - 256.9 | | | 101.0 - 101.0 |
| R23 | Ivanchenko et al, 1974 ^1127^ | | | 93 | 91 | | 273.2 - 432.4 | | | 1060.0 - 58900.0 |
| R23 | Rasskazov et al, 1975 ^1146^ | | | 34 | 33 | | 203.2 - 363.1 | | | 169.0 - 40000.0 |
| R23 | Geller, 1980 ^1096^ | | | 92 | 92 | | 117.9 - 293.1 | | | 5000.0 - 58960.0 |
| R23 | Rasskazov et al, 1980 ^1095^ | | | 5 | 5 | | 153.2 - 188.2 | | | 101.0 - 101.0 |
| R23 | Filatov, 1975 ^1144^ | | | 162 | 161 | | 203.2 - 453.1 | | | 30.0 - 40000.0 |
| R23 | Bondar’, 1984 ^1098^ | | | 91 | 91 | | 117.9 - 293.1 | | | 5000.0 - 58960.0 |
| R23 | Yokoyama Takahashi, 1997 ^1182^ | | | 295 | 295 | | 299.2 - 303.2 | | | 101.0 - 5652.0 |

| Material | | Author | *N*_all_ | | | *N*_used_ | | *T* / K | *p* / kPa | |
| --- | --- | --- | --- | --- | --- | --- | --- | --- | --- | --- |
| R236EA | Laesecke Defibaugh, 1996 ^1183^ | | | 37 | 37 | | 262.1 - 343.2 | | | 49.0 - 787.0 |
| R236FA | Laesecke Defibaugh, 1996 ^1183^ | | | 38 | 38 | | 262.1 - 353.2 | | | 68.0 - 1256.0 |
| R236FA | Meng et al, 2011 ^1140^ | | | 117 | 117 | | 253.2 - 373.1 | | | 1000.0 - 40000.0 |

| Material | Author | *N*_all_ | | | *N*_used_ | | *T* / K | *p* / kPa | |
| --- | --- | --- | --- | --- | --- | --- | --- | --- | --- |
| R245CA | Laesecke Hafer, 1998 ^1175^ | | 84 | 84 | | 248.5 - 333.2 | | | 10.0 - 332.0 |

| Material | | Author | *N*_all_ | | | *N*_used_ | | *T* / K | *p* / kPa | |
| --- | --- | --- | --- | --- | --- | --- | --- | --- | --- | --- |
| R245FA | Laesecke Hafer, 1998 ^1175^ | | | 65 | 65 | | 250.1 - 315.1 | | | 17.0 - 267.0 |
| R245FA | Wang et al, 2010 ^106^ | | | 47 | 47 | | 300.8 - 396.5 | | | 100.0 - 1614.0 |
| R245FA | Meng et al, 2011 ^1140^ | | | 107 | 107 | | 263.2 - 373.1 | | | 1000.0 - 40000.0 |

| Material | | Author | *N*_all_ | | | *N*_used_ | | *T* / K | *p* / kPa | |
| --- | --- | --- | --- | --- | --- | --- | --- | --- | --- | --- |
| R32 | Oliveria Wakeham, 1993 ^1119^ | | | 14 | 14 | | 223.2 - 343.2 | | | 101.0 - 4860.0 |
| R32 | Assael et al, 1994 ^656^ | | | 5 | 5 | | 273.2 - 313.2 | | | 778.0 - 2435.0 |
| R32 | Oliveria Wakeham, 1993 ^1119^ | | | 19 | 19 | | 231.8 - 343.1 | | | 154.0 - 4852.0 |
| R32 | Ripple Matar, 1993 ^1117^ | | | 10 | 10 | | 250.9 - 293.2 | | | 350.0 - 1433.0 |
| R32 | Heide Schenk, 1996 ^1120^ | | | 12 | 12 | | 223.2 - 333.2 | | | 101.0 - 3903.0 |
| R32 | Huber et al, 1999 ^1133^ | | | 60 | 60 | | 250.2 - 315.1 | | | 341.0 - 2553.0 |
| R32 | Phillips Murphy, 1970 ^640^ | | | 10 | 8 | | 222.9 - 287.3 | | | 100.0 - 1208.0 |
| R32 | Assael et al, 1994 ^656^ | | | 26 | 26 | | 273.1 - 313.2 | | | 1600.0 - 15550.0 |
| R32 | Kochubey Moin, 1978 ^1145^ | | | 1 | 1 | | 273.0 - 273.0 | | | 101.0 - 101.0 |
| R32 | Takahashi et al, 1995 ^1184^ | | | 114 | 113 | | 298.2 - 423.2 | | | 101.0 - 10020.0 |
| R32 | Oliveria Wakeham, 1999 ^1124^ | | | 13 | 13 | | 223.2 - 343.2 | | | 101.0 - 4860.0 |
| R32 | Geller et al, 1996 ^1185^ | | | 51 | 51 | | 253.1 - 363.2 | | | 100.0 - 5427.0 |

| Material | | Author | *N*_all_ | | | *N*_used_ | | *T* / K | *p* / kPa | |
| --- | --- | --- | --- | --- | --- | --- | --- | --- | --- | --- |
| R40 | Badyleks, 1960 ^1186^ | | | 6 | 5 | | 253.2 - 293.1 | | | 116.0 - 482.0 |
| R40 | Awbery Griffiths, 1936 ^1100^ | | | 10 | 10 | | 258.1 - 303.1 | | | 142.0 - 646.0 |
| R40 | McGovern, 1943 ^1187^ | | | 1 | 0 | | 10000.0 - 0.0 | | | 1000000000.0 - 0.0 |
| R40 | Rutherford, 1984 ^1045^ | | | 16 | 16 | | 293.1 - 423.1 | | | 3546.0 - 10440.0 |
| R40 | Benning Markwood, 1939 ^1087^ | | | 3 | 3 | | 275.0 - 352.5 | | | 101.0 - 101.0 |
| R40 | Reed Rabinovitch, 1957 ^1126^ | | | 1 | 1 | | 298.1 - 298.1 | | | 101.0 - 101.0 |
| R40 | Tomassini et al, 1970 ^1179^ | | | 1 | 1 | | 293.1 - 293.1 | | | 101.0 - 101.0 |

| Material | | Author | *N*_all_ | | | *N*_used_ | | *T* / K | *p* / kPa | |
| --- | --- | --- | --- | --- | --- | --- | --- | --- | --- | --- |
| R41 | Tomassini et al, 1970 ^1179^ | | | 1 | 1 | | 300.0 - 300.0 | | | 101.0 - 101.0 |
| R41 | Kochubey Moin, 1978 ^1145^ | | | 1 | 1 | | 273.0 - 273.0 | | | 101.0 - 101.0 |

| Material | | Author | *N*_all_ | | | *N*_used_ | | *T* / K | *p* / kPa | |
| --- | --- | --- | --- | --- | --- | --- | --- | --- | --- | --- |
| RC318 | Geller et al, 1976 ^1091^ | | | 4 | 4 | | 235.5 - 263.8 | | | 24.0 - 92.0 |
| RC318 | Karbanov, 1978 ^1092^ | | | 10 | 10 | | 235.5 - 323.2 | | | 24.0 - 656.0 |
| RC318 | Kessel’man et al, 1976 ^809^ | | | 30 | 30 | | 262.0 - 322.1 | | | 1100.0 - 50000.0 |
| RC318 | Finney et al, 1977 ^121^ | | | 27 | 25 | | 323.0 - 423.0 | | | 8000.0 - 160000.0 |
| RC318 | Kessel’man et al, 1977 ^1188^ | | | 5 | 5 | | 262.0 - 322.1 | | | 1100.0 - 1100.0 |

| Material | Author | *N*_all_ | | | *N*_used_ | | *T* / K | *p* / kPa | |
| --- | --- | --- | --- | --- | --- | --- | --- | --- | --- |
| RE245FA2 | Ripple Matar, 1993 ^1117^ | | 20 | 20 | | 250.0 - 331.7 | | | 9.0 - 277.0 |

| Material | Author | | *N*_all_ | | | *N*_used_ | | *T* / K | *p* / kPa | |
| --- | --- | --- | --- | --- | --- | --- | --- | --- | --- | --- |
| RE347MCC | | Sekiya Misaki, 2000 ^1189^ | | 1 | 1 | | 296.2 - 296.2 | | | 2909.0 - 2909.0 |
| RE347MCC | | Hu et al, 2015 ^1190^ | | 84 | 84 | | 253.2 - 363.2 | | | 990.0 - 30010.0 |
| RE347MCC | | Sekiya Misaki, 2000 ^1189^ | | 1 | 1 | | 296.2 - 296.2 | | | 101.0 - 101.0 |
| RE347MCC | | Nakazawa et al, 2002 ^1111^ | | 6 | 6 | | 278.1 - 303.2 | | | 101.0 - 101.0 |

| Material | | Author | *N*_all_ | | | *N*_used_ | | *T* / K | *p* / kPa | |
| --- | --- | --- | --- | --- | --- | --- | --- | --- | --- | --- |
| SF6 | Ulybin Makarushkin, 1977 ^1191^ | | | 26 | 12 | | 233.2 - 317.1 | | | 348.0 - 3627.0 |
| SF6 | Ellis Raw, 1959 ^1026^ | | | 17 | 17 | | 470.4 - 1125.9 | | | 101.0 - 101.0 |
| SF6 | Raw Tang, 1963 ^1142^ | | | 4 | 4 | | 303.1 - 342.0 | | | 101.0 - 101.0 |
| SF6 | Hellemans, 1973 ^938^ | | | 7 | 7 | | 298.1 - 573.1 | | | 101.0 - 101.0 |
| SF6 | Ueda Kigoshi, 1974 ^1192^ | | | 8 | 8 | | 273.5 - 346.1 | | | 101.0 - 101.0 |
| SF6 | Timrot et al, 1975 ^100^ | | | 62 | 62 | | 297.4 - 525.9 | | | 40.0 - 3460.0 |
| SF6 | Kestin et al, 1976 ^393^ | | | 3 | 3 | | 296.1 - 477.1 | | | 101.0 - 101.0 |
| SF6 | Kestin et al, 1977 ^256^ | | | 5 | 5 | | 298.1 - 473.1 | | | 101.0 - 101.0 |
| SF6 | Ulybin Makarushkin, 1977 ^1191^ | | | 63 | 0 | | 10000.0 - 0.0 | | | 1000000000.0 - 0.0 |
| SF6 | Grigor’ev et al, 1978 ^1193^ | | | 133 | 133 | | 245.1 - 473.1 | | | 2000.0 - 40000.0 |
| SF6 | Harris et al, 1979 ^394^ | | | 7 | 7 | | 218.3 - 302.2 | | | 101.0 - 101.0 |
| SF6 | Tanaka et al, 1980 ^795^ | | | 12 | 12 | | 298.1 - 348.1 | | | 101.0 - 205.0 |
| SF6 | Hoogland et al, 1985 ^1040^ | | | 31 | 31 | | 298.1 - 333.2 | | | 100.0 - 9573.0 |
| SF6 | Hafer, 1999 ^1194^ | | | 1112 | 1112 | | 225.6 - 315.0 | | | 27.0 - 3440.0 |
| SF6 | Hurly et al, 2003 ^105^ | | | 15 | 15 | | 298.2 - 298.2 | | | 296.0 - 1558.0 |
| SF6 | Wilhelm et al, 2005 ^1195^ | | | 677 | 677 | | 300.0 - 425.0 | | | 26.0 - 20442.0 |
| SF6 | Grigor’ev et al, 1977 ^1196^ | | | 133 | 132 | | 245.1 - 473.1 | | | 2000.0 - 40000.0 |
| SF6 | Hafer, 1999 ^1194^ | | | 963 | 963 | | 225.2 - 326.7 | | | 102.0 - 34470.0 |

| Material | | Author | *N*_all_ | | | *N*_used_ | | *T* / K | *p* / kPa | |
| --- | --- | --- | --- | --- | --- | --- | --- | --- | --- | --- |
| SO2 | Trautz Weizel, 1925 ^1197^ | | | 23 | 23 | | 287.2 - 472.2 | | | 101.0 - 101.0 |
| SO2 | Smith, 1922 ^1198^ | | | 3 | 3 | | 273.2 - 373.2 | | | 160.0 - 2796.0 |
| SO2 | Trautz Zink, 1930 ^762^ | | | 14 | 14 | | 290.6 - 1096.2 | | | 101.0 - 101.0 |
| SO2 | Awbery Griffiths, 1936 ^1100^ | | | 10 | 10 | | 258.2 - 303.2 | | | 84.0 - 469.0 |
| SO2 | Titani, 1930 ^9^ | | | 6 | 6 | | 293.2 - 393.2 | | | 337.0 - 4136.0 |
| SO2 | Wobser Mueller, 1941 ^79^ | | | 5 | 5 | | 293.2 - 371.2 | | | 101.0 - 101.0 |
| SO2 | Trautz Winterkorn, 1931 ^355^ | | | 4 | 4 | | 292.6 - 472.6 | | | 101.0 - 6943.0 |
| SO2 | Lewis, 1925 ^36^ | | | 1 | 1 | | 298.2 - 298.2 | | | 399.0 - 399.0 |
| SO2 | Vogel, 1914 ^80^ | | | 1 | 1 | | 273.1 - 273.1 | | | 84.0 - 84.0 |
| SO2 | Trautz Winterkorn, 1931 ^355^ | | | 2 | 2 | | 412.8 - 540.8 | | | 101.0 - 5868.0 |
| SO2 | Shimotake Thodos, 1963 ^1199^ | | | 9 | 9 | | 473.2 - 473.2 | | | 1827.0 - 24235.0 |
| SO2 | Chang et al, 1970 ^1200^ | | | 9 | 9 | | 273.0 - 673.0 | | | 101.0 - 6955.0 |
| SO2 | Bhattacharyya, 1970 ^778^ | | | 4 | 4 | | 238.2 - 308.2 | | | 31.0 - 548.0 |
| SO2 | Bhattacharyya Ghosi, 1970 ^780^ | | | 8 | 8 | | 238.2 - 308.7 | | | 7.0 - 558.0 |
| SO2 | Pal Barua, 1967 ^781^ | | | 5 | 5 | | 306.2 - 477.2 | | | 101.0 - 5158.0 |
| SO2 | Chakraborti Gray, 1966 ^1201^ | | | 2 | 2 | | 308.2 - 353.2 | | | 548.0 - 1810.0 |
| SO2 | Chakraborti Gray, 1965 ^1202^ | | | 3 | 3 | | 298.2 - 353.2 | | | 101.0 - 101.0 |
| SO2 | Pal Barua, 1967 ^781^ | | | 5 | 5 | | 306.2 - 473.2 | | | 101.0 - 6972.0 |
| SO2 | Kestin et al, 1971 ^254^ | | | 2 | 2 | | 297.3 - 303.9 | | | 99.0 - 102.0 |
| SO2 | Stakelbeck, 1933 ^1203^ | | | 113 | 106 | | 253.2 - 313.2 | | | 49.0 - 785.0 |
| SO2 | Lucatu, 1938^1204^ | | | 7 | 7 | | 233.2 - 273.2 | | | 23.0 - 160.0 |
| SO2 | Jung Schmick, 1930 ^784^ | | | 1 | 1 | | 289.0 - 289.0 | | | 101.0 - 101.0 |
| SO2 | Adams Rogers, 1939 ^1205^ | | | 1 | 1 | | 298.2 - 298.2 | | | 399.0 - 399.0 |
| SO2 | Takahashi et al, 1987 ^1151^ | | | 43 | 34 | | 298.2 - 430.5 | | | 398.0 - 7851.0 |
| SO2 | Cupp Rogers, 1939 ^1206^ | | | 1 | 1 | | 298.2 - 298.2 | | | 399.0 - 399.0 |
| SO2 | Scheuer, 1911 ^1207^ | | | 5 | 5 | | 218.2 - 263.2 | | | 9.0 - 105.0 |
| SO2 | Clifford et al, 1981 ^1208^ | | | 9 | 9 | | 321.0 - 1300.0 | | | 101.0 - 6299.0 |
| SO2 | Salomon, 1989 ^1209^ | | | 1 | 1 | | 273.2 - 273.2 | | | 160.0 - 160.0 |
| SO2 | Liessmann et al, 1995 ^5^ | | | 11 | 11 | | 233.2 - 333.2 | | | 23.0 - 1111.0 |
| SO2 | Vargaftik, 1972 ^6^ | | | 11 | 0 | | 10000.0 - 0.0 | | | 1000000000.0 - 0.0 |
| SO2 | Iwasaki Takahashi, 1975 ^1210^ | | | 152 | 152 | | 298.2 - 433.2 | | | 101.0 - 8085.0 |
| SO2 | Stakelbeck, 1933 ^1203^ | | | 76 | 70 | | 253.2 - 303.2 | | | 64.0 - 785.0 |
| SO2 | Trautz Weizel, 1925 ^1197^ | | | 8 | 8 | | 287.2 - 472.2 | | | 101.0 - 4436.0 |
| SO2 | Traut Zink, 1930 ^762^ | | | 7 | 7 | | 290.6 - 1096.2 | | | 101.0 - 308.0 |
| SO2 | Shimotake Thodos, 1963 ^1199^ | | | 9 | 9 | | 473.2 - 473.2 | | | 1827.0 - 24235.0 |
| SO2 | Scholz Kley, 1981 ^7^ | | | 60 | 60 | | 198.2 - 1096.2 | | | 2.0 - 6938.0 |
| SO2 | Awbery Griffiths, 1936 ^1100^ | | | 20 | 20 | | 258.1 - 303.2 | | | 84.0 - 469.0 |
| SO2 | Cupp Rogers, 1939 ^1206^ | | | 1 | 1 | | 298.1 - 298.1 | | | 399.0 - 399.0 |
| SO2 | Shimotake Thodos, 1963 ^1199^ | | | 9 | 9 | | 473.2 - 473.2 | | | 1827.0 - 24235.0 |

| Material | Author | *N*_all_ | | | *N*_used_ | | *T* / K | *p* / kPa | |
| --- | --- | --- | --- | --- | --- | --- | --- | --- | --- |
| TOLUENE | Heydweiller, 1896 ^111^ | | 7 | 7 | | 293.7 - 456.2 | | | 3.0 - 546.0 |
| TOLUENE | Batschinski, 1913 ^29^ | | 11 | 11 | | 273.1 - 373.1 | | | 1.0 - 74.0 |
| TOLUENE | litkenous et al, 1940 ^1211^ | | 1 | 1 | | 298.1 - 298.1 | | | 4.0 - 4.0 |
| TOLUENE | Ledneva, 1956 ^800^ | | 10 | 10 | | 293.1 - 473.2 | | | 3.0 - 749.0 |
| TOLUENE | Rabe, 1981 ^390^ | | 8 | 4 | | 250.0 - 300.0 | | | 0.0 - 4.0 |
| TOLUENE | Xu et al, 2007 ^1212^ | | 7 | 7 | | 303.2 - 363.2 | | | 5.0 - 54.0 |
| TOLUENE | Thorpe Rodger, 1894 ^28^ | | 12 | 12 | | 273.4 - 380.2 | | | 101.0 - 101.0 |
| TOLUENE | Dunstan et al, 1913 ^122^ | | 1 | 1 | | 298.1 - 298.1 | | | 101.0 - 101.0 |
| TOLUENE | Schaaf, 1923 ^126^ | | 1 | 1 | | 290.1 - 290.1 | | | 101.0 - 101.0 |
| TOLUENE | Miller, 1924 ^127^ | | 2 | 2 | | 281.8 - 293.1 | | | 101.0 - 101.0 |
| TOLUENE | Kollosowsky, 1925 ^128^ | | 9 | 9 | | 287.0 - 305.8 | | | 101.0 - 101.0 |
| TOLUENE | Lewis, 1925 ^36^ | | 1 | 1 | | 298.1 - 298.1 | | | 101.0 - 101.0 |
| TOLUENE | Dessart, 1926 ^130^ | | 2 | 2 | | 288.1 - 303.1 | | | 101.0 - 101.0 |
| TOLUENE | Timmermans Martin, 1926 ^131^ | | 3 | 3 | | 288.1 - 333.1 | | | 101.0 - 101.0 |
| TOLUENE | Tausz Staab, 1930 ^133^ | | 1 | 1 | | 293.1 - 293.1 | | | 101.0 - 101.0 |
| TOLUENE | Il’in Ivanov, 1935 ^1213^ | | 1 | 1 | | 293.1 - 293.1 | | | 101.0 - 101.0 |
| TOLUENE | Bratton Felsing, 1936 ^893^ | | 1 | 1 | | 363.1 - 363.1 | | | 101.0 - 101.0 |
| TOLUENE | Linke, 1941 ^139^ | | 5 | 5 | | 286.2 - 322.6 | | | 101.0 - 101.0 |
| TOLUENE | Zeidler Schuster, 1942 ^1214^ | | 3 | 3 | | 293.1 - 313.1 | | | 101.0 - 101.0 |
| TOLUENE | Teitel’baum et al, 1950 ^140^ | | 1 | 1 | | 293.1 - 293.1 | | | 101.0 - 101.0 |
| TOLUENE | Hammond Stokes, 1955 ^113^ | | 1 | 1 | | 298.1 - 298.1 | | | 101.0 - 101.0 |
| TOLUENE | Ling Van Winkle, 1958 ^43^ | | 4 | 4 | | 303.1 - 368.1 | | | 101.0 - 101.0 |
| TOLUENE | Barlow et al, 1966 ^549^ | | 6 | 4 | | 174.9 - 303.2 | | | 101.0 - 101.0 |
| TOLUENE | Blank, 1968 ^44^ | | 1 | 1 | | 298.1 - 298.1 | | | 101.0 - 101.0 |
| TOLUENE | Akhundov et al, 1970 ^1215^ | | 153 | 153 | | 296.9 - 548.1 | | | 100.0 - 40000.0 |
| TOLUENE | Konobeev Lyapin, 1970 ^45^ | | 3 | 3 | | 293.1 - 333.1 | | | 101.0 - 101.0 |
| TOLUENE | Katz et al, 1971 ^1216^ | | 4 | 4 | | 298.1 - 313.1 | | | 101.0 - 101.0 |
| TOLUENE | Alms et al, 1973 ^147^ | | 1 | 1 | | 296.8 - 296.8 | | | 101.0 - 101.0 |
| TOLUENE | Shikhaliev et al 1974 ^641^ | | 14 | 14 | | 223.0 - 353.0 | | | 101.0 - 101.0 |
| TOLUENE | Pugachevich et al, 1975 ^1217^ | | 5 | 5 | | 303.1 - 363.1 | | | 101.0 - 101.0 |
| TOLUENE | Dhillon Chugh, 1976 ^149^ | | 2 | 2 | | 298.1 - 308.1 | | | 101.0 - 101.0 |
| TOLUENE | Hafez Hartland, 1976 ^49^ | | 4 | 4 | | 293.1 - 318.1 | | | 101.0 - 101.0 |
| TOLUENE | Asfour Dullien, 1981 ^150^ | | 1 | 1 | | 298.1 - 298.1 | | | 101.0 - 101.0 |
| TOLUENE | Rabe, 1981 ^390^ | | 53 | 53 | | 183.4 - 300.6 | | | 100.0 - 10000.0 |
| TOLUENE | Kashiwagi Makita, 1982 ^152^ | | 48 | 48 | | 298.1 - 348.1 | | | 100.0 - 110000.0 |
| TOLUENE | Singh Sinha, 1982 ^365^ | | 1 | 1 | | 298.1 - 298.1 | | | 101.0 - 101.0 |
| TOLUENE | Akhundov et al, 1983 ^1218^ | | 16 | 14 | | 548.1 - 573.1 | | | 3310.0 - 39832.0 |
| TOLUENE | Albright et al, 1983 ^154^ | | 1 | 1 | | 298.2 - 298.2 | | | 101.0 - 101.0 |
| TOLUENE | Oswal Rathnam, 1984 ^155^ | | 1 | 1 | | 303.1 - 303.1 | | | 101.0 - 101.0 |
| TOLUENE | Singh Sinha, 1984 ^367^ | | 3 | 3 | | 303.1 - 323.1 | | | 101.0 - 101.0 |
| TOLUENE | Al-Madfai et al, 1985 ^1008^ | | 1 | 1 | | 298.1 - 298.1 | | | 101.0 - 101.0 |
| TOLUENE | Singh Sinha, 1985 ^368^ | | 5 | 5 | | 298.1 - 333.1 | | | 101.0 - 101.0 |
| TOLUENE | Craubner, 1986 ^841^ | | 1 | 1 | | 293.1 - 293.1 | | | 101.0 - 101.0 |
| TOLUENE | Ritzoulis et al, 1986 ^1219^ | | 3 | 3 | | 288.2 - 308.1 | | | 101.0 - 101.0 |
| TOLUENE | Singh et al, 1986 ^370^ | | 1 | 1 | | 298.1 - 298.1 | | | 101.0 - 101.0 |
| TOLUENE | Byers Williams, 1987 ^1220^ | | 8 | 8 | | 298.2 - 368.2 | | | 101.0 - 101.0 |
| TOLUENE | Ramanjaneyulu et al, 1987 ^160^ | | 1 | 1 | | 303.1 - 303.1 | | | 101.0 - 101.0 |
| TOLUENE | Chandrasekhar et al, 1988 ^161^ | | 1 | 1 | | 303.1 - 303.1 | | | 101.0 - 101.0 |
| TOLUENE | Fermeglia Lapasin, 1988 ^553^ | | 1 | 1 | | 298.1 - 298.1 | | | 101.0 - 101.0 |
| TOLUENE | Rao Reddy, 1988 ^162^ | | 1 | 1 | | 298.1 - 298.1 | | | 101.0 - 101.0 |
| TOLUENE | Singh et al, 1989 ^554^ | | 5 | 5 | | 298.1 - 333.1 | | | 101.0 - 101.0 |
| TOLUENE | Schumpe Luehring, 1990 ^54^ | | 1 | 1 | | 293.2 - 293.2 | | | 101.0 - 101.0 |
| TOLUENE | Singh et al, 1990 ^1221^ | | 5 | 5 | | 298.2 - 333.2 | | | 101.0 - 101.0 |
| TOLUENE | Sivaramprasad et al, 1990 ^170^ | | 1 | 1 | | 298.2 - 298.2 | | | 101.0 - 101.0 |
| TOLUENE | Aminabhavi et al, 1991 ^171^ | | 8 | 8 | | 298.2 - 313.2 | | | 101.0 - 101.0 |
| TOLUENE | Assael et al, 1991 ^172^ | | 36 | 36 | | 303.2 - 334.7 | | | 100.0 - 71330.0 |
| TOLUENE | Assael Papadaki, 1991 ^272^ | | 4 | 4 | | 304.7 - 334.7 | | | 101.0 - 101.0 |
| TOLUENE | Das Jha, 1991 ^55^ | | 1 | 0 | | 10000.0 - 0.0 | | | 1000000000.0 - 0.0 |
| TOLUENE | Aralaguppi et al, 1992 ^175^ | | 3 | 3 | | 298.2 - 308.2 | | | 101.0 - 101.0 |
| TOLUENE | Klofutar et al, 1992 ^176^ | | 6 | 6 | | 293.0 - 333.0 | | | 101.0 - 101.0 |
| TOLUENE | Krall et al, 1992 ^1222^ | | 60 | 60 | | 297.5 - 426.0 | | | 100.0 - 30000.0 |
| TOLUENE | Liu Luo, 1992 ^57^ | | 4 | 4 | | 293.2 - 313.2 | | | 101.0 - 101.0 |
| TOLUENE | Moumouzias Ritzoulis, 1992 ^1011^ | | 5 | 5 | | 288.2 - 308.2 | | | 101.0 - 101.0 |
| TOLUENE | Oliveira Wakeham, 1992 ^502^ | | 46 | 43 | | 303.2 - 348.2 | | | 100.0 - 251910.0 |
| TOLUENE | Shastri et al, 1993 ^179^ | | 1 | 1 | | 293.2 - 293.2 | | | 101.0 - 101.0 |
| TOLUENE | Pandey et al, 1994 ^414^ | | 1 | 1 | | 298.2 - 298.2 | | | 101.0 - 101.0 |
| TOLUENE | Dymond et al, 1995 ^1223^ | | 23 | 16 | | 298.6 - 323.3 | | | 100.0 - 200400.0 |
| TOLUENE | Et-Tahir et al, 1995 ^329^ | | 30 | 30 | | 298.2 - 363.2 | | | 100.0 - 100000.0 |
| TOLUENE | Exarchos et al, 1995 ^181^ | | 5 | 5 | | 293.2 - 313.2 | | | 101.0 - 101.0 |
| TOLUENE | Krishnan et al, 1995 ^182^ | | 1 | 1 | | 303.2 - 303.2 | | | 101.0 - 101.0 |
| TOLUENE | Abdulagatov Rasulov, 1996 ^802^ | | 6 | 6 | | 436.0 - 436.0 | | | 5000.0 - 30000.0 |
| TOLUENE | Ramadevi et al, 1996 ^371^ | | 2 | 2 | | 303.2 - 313.2 | | | 101.0 - 101.0 |
| TOLUENE | Harris et al, 1997 ^1051^ | | 20 | 12 | | 298.2 - 323.2 | | | 12560.0 - 198930.0 |
| TOLUENE | Viera Nieto, 1997 ^185^ | | 31 | 31 | | 298.2 - 373.2 | | | 100.0 - 207200.0 |
| TOLUENE | George et al, 1998 ^957^ | | 1 | 1 | | 293.2 - 293.2 | | | 101.0 - 101.0 |
| TOLUENE | Swain et al, 1998 ^187^ | | 4 | 4 | | 303.2 - 318.2 | | | 101.0 - 101.0 |
| TOLUENE | Aminabhavi Banerjee, 1999 ^188^ | | 3 | 3 | | 298.2 - 308.2 | | | 101.0 - 101.0 |
| TOLUENE | Aralaguppi et al, 1999 ^189^ | | 3 | 3 | | 298.2 - 308.2 | | | 101.0 - 101.0 |
| TOLUENE | Assael et al, 1999 ^1224^ | | 106 | 106 | | 213.7 - 363.1 | | | 101.0 - 25530.0 |
| TOLUENE | Swain et al, 1999 ^192^ | | 5 | 5 | | 298.2 - 318.2 | | | 101.0 - 101.0 |
| TOLUENE | Assael et al, 2000 ^445^ | | 16 | 16 | | 213.7 - 363.1 | | | 101.0 - 101.0 |
| TOLUENE | Harris, 2000 ^1225^ | | 200 | 146 | | 255.1 - 323.2 | | | 100.0 - 225300.0 |
| TOLUENE | Nikam et al, 2000 ^678^ | | 3 | 3 | | 303.2 - 313.2 | | | 101.0 - 101.0 |
| TOLUENE | Tsierkezos et al, 2000 ^195^ | | 1 | 1 | | 293.2 - 293.2 | | | 101.0 - 101.0 |
| TOLUENE | Kapadi et al, 2001 ^198^ | | 1 | 1 | | 293.2 - 293.2 | | | 101.0 - 101.0 |
| TOLUENE | Roos Bart, 2001 ^1226^ | | 1 | 1 | | 298.2 - 298.2 | | | 101.0 - 101.0 |
| TOLUENE | Swain et al, 2001 ^200^ | | 5 | 5 | | 298.2 - 318.2 | | | 101.0 - 101.0 |
| TOLUENE | Akhtar et al, 2002 ^1227^ | | 5 | 5 | | 303.2 - 323.2 | | | 101.0 - 101.0 |
| TOLUENE | Cruz et al, 2002 ^1228^ | | 2 | 2 | | 293.2 - 298.2 | | | 100.0 - 101.0 |
| TOLUENE | Nayak et al, 2002 ^201^ | | 3 | 3 | | 298.2 - 308.2 | | | 101.0 - 101.0 |
| TOLUENE | Peng Tu, 2002 ^61^ | | 1 | 1 | | 298.2 - 298.2 | | | 101.0 - 101.0 |
| TOLUENE | Rattan et al, 2002 ^1229^ | | 4 | 4 | | 293.2 - 323.2 | | | 101.0 - 101.0 |
| TOLUENE | George Sastry, 2003 ^202^ | | 2 | 2 | | 298.2 - 308.2 | | | 101.0 - 101.0 |
| TOLUENE | George Sastry, 2003 ^202^ | | 2 | 2 | | 298.2 - 308.2 | | | 101.0 - 101.0 |
| TOLUENE | Lark et al, 2003 ^203^ | | 2 | 2 | | 298.2 - 303.2 | | | 101.0 - 101.0 |
| TOLUENE | Oswal et al, 2003 ^205^ | | 1 | 1 | | 303.2 - 303.2 | | | 101.0 - 101.0 |
| TOLUENE | Agarwal Singh, 2004 ^63^ | | 1 | 1 | | 298.2 - 298.2 | | | 101.0 - 101.0 |
| TOLUENE | Caetano et al, 2004 ^1230^ | | 79 | 79 | | 213.5 - 298.4 | | | 110.0 - 20050.0 |
| TOLUENE | Caudwell et al, 2004 ^297^ | | 38 | 38 | | 323.2 - 373.2 | | | 100.0 - 90100.0 |
| TOLUENE | Jain Singh, 2004 ^64^ | | 1 | 1 | | 303.2 - 303.2 | | | 101.0 - 101.0 |
| TOLUENE | Oswal et al, 2004 ^208^ | | 1 | 1 | | 303.2 - 303.2 | | | 101.0 - 101.0 |
| TOLUENE | Yang et al, 2004 ^209^ | | 3 | 3 | | 298.2 - 323.2 | | | 101.0 - 101.0 |
| TOLUENE | Iloukhani et al, 2005 ^428^ | | 1 | 1 | | 298.2 - 298.2 | | | 101.0 - 101.0 |
| TOLUENE | Kandil et al, 2005 ^1231^ | | 33 | 33 | | 298.2 - 348.2 | | | 100.0 - 40000.0 |
| TOLUENE | Nikam Kharat, 2005 ^1232^ | | 4 | 4 | | 298.2 - 313.2 | | | 101.0 - 101.0 |
| TOLUENE | Oswal et al, 2005 ^210^ | | 1 | 1 | | 303.2 - 303.2 | | | 101.0 - 101.0 |
| TOLUENE | Pensado et al, 2005 ^822^ | | 84 | 84 | | 303.2 - 353.2 | | | 100.0 - 60000.0 |
| TOLUENE | Ali et al, 2006 ^211^ | | 1 | 1 | | 308.2 - 308.2 | | | 101.0 - 101.0 |
| TOLUENE | Al-Kandary et al, 2006 ^1013^ | | 2 | 2 | | 293.2 - 303.2 | | | 101.0 - 101.0 |
| TOLUENE | Al-Kandary et al, 2006 ^1013^ | | 4 | 4 | | 288.2 - 303.2 | | | 101.0 - 101.0 |
| TOLUENE | Baragi et al, 2006 ^213^ | | 3 | 3 | | 298.2 - 308.2 | | | 101.0 - 101.0 |
| TOLUENE | Goodwin et al, 2006 ^301^ | | 11 | 11 | | 323.2 - 373.1 | | | 100.0 - 68196.0 |
| TOLUENE | Han et al, 2006 ^214^ | | 1 | 1 | | 298.2 - 298.2 | | | 101.0 - 101.0 |
| TOLUENE | Hasan et al, 2006 ^1233^ | | 2 | 2 | | 303.2 - 313.2 | | | 101.0 - 101.0 |
| TOLUENE | Tsierkezos Filippou, 2006 ^1234^ | | 3 | 3 | | 278.2 - 293.2 | | | 101.0 - 101.0 |
| TOLUENE | Varshney Singh, 2006 ^216^ | | 1 | 1 | | 303.2 - 303.2 | | | 101.0 - 101.0 |
| TOLUENE | Yang et al, 2006 ^588^ | | 8 | 8 | | 298.2 - 363.2 | | | 101.0 - 101.0 |
| TOLUENE | Hasan et al, 2007 ^1235^ | | 2 | 2 | | 298.2 - 308.2 | | | 101.0 - 101.0 |
| TOLUENE | Nain, 2007 ^219^ | | 4 | 4 | | 288.2 - 318.2 | | | 101.0 - 101.0 |
| TOLUENE | Nain, 2007 ^219^ | | 4 | 4 | | 288.2 - 318.2 | | | 101.0 - 101.0 |
| TOLUENE | Ren et al, 2007 ^69^ | | 1 | 1 | | 298.2 - 298.2 | | | 101.0 - 101.0 |
| TOLUENE | Song et al, 2007 ^221^ | | 7 | 7 | | 298.2 - 353.2 | | | 101.0 - 101.0 |
| TOLUENE | Yang et al, 2007 ^222^ | | 6 | 6 | | 303.2 - 353.2 | | | 101.0 - 101.0 |
| TOLUENE | Alonso et al, 2008 ^1236^ | | 1 | 1 | | 298.2 - 298.2 | | | 101.0 - 101.0 |
| TOLUENE | Awwad Abu-Daabes, 2008 ^223^ | | 4 | 4 | | 298.2 - 323.2 | | | 101.0 - 101.0 |
| TOLUENE | Baskaran Kubendran, 2008 ^379^ | | 1 | 1 | | 298.2 - 298.2 | | | 101.0 - 101.0 |
| TOLUENE | Fang et al, 2008 ^511^ | | 20 | 20 | | 313.2 - 473.2 | | | 100.0 - 3200.0 |
| TOLUENE | Meng et al, 2008 ^521^ | | 13 | 13 | | 298.2 - 323.2 | | | 100.0 - 14770.0 |
| TOLUENE | Song et al, 2008 ^225^ | | 7 | 7 | | 303.2 - 333.2 | | | 101.0 - 101.0 |
| TOLUENE | Baylaucq et al, 2009 ^228^ | | 24 | 24 | | 293.2 - 353.2 | | | 100.0 - 100000.0 |
| TOLUENE | El-Sayed Asfour, 2009 ^826^ | | 3 | 3 | | 308.2 - 313.2 | | | 101.0 - 101.0 |
| TOLUENE | Nain et al, 2009 ^229^ | | 1 | 1 | | 298.2 - 298.2 | | | 101.0 - 101.0 |
| TOLUENE | Sastry et al, 2009 ^231^ | | 2 | 2 | | 298.2 - 308.2 | | | 101.0 - 101.0 |
| TOLUENE | Silva et al, 2009 ^436^ | | 5 | 5 | | 283.2 - 323.2 | | | 101.0 - 101.0 |
| TOLUENE | Yan et al, 2009 ^232^ | | 2 | 2 | | 308.2 - 318.2 | | | 101.0 - 101.0 |
| TOLUENE | Ikeuchi et al, 2010 ^233^ | | 6 | 6 | | 283.2 - 308.1 | | | 101.0 - 101.0 |
| TOLUENE | Rajagopal Chenthilnath, 2010 ^72^ | | 3 | 3 | | 298.2 - 308.2 | | | 101.0 - 101.0 |
| TOLUENE | Rajagopal Chenthilnath, 2010 ^72^ | | 3 | 3 | | 298.2 - 308.2 | | | 101.0 - 101.0 |
| TOLUENE | Rajagopal Chenthilnath, 2010 ^72^ | | 3 | 3 | | 298.2 - 308.2 | | | 101.0 - 101.0 |
| TOLUENE | Meng et al, 2011 ^1140^ | | 24 | 24 | | 248.2 - 348.1 | | | 1000.0 - 40000.0 |
| TOLUENE | Pradhan Roy, 2011 ^236^ | | 1 | 1 | | 298.2 - 298.2 | | | 101.0 - 101.0 |
| TOLUENE | Yadava et al, 2011 ^238^ | | 1 | 1 | | 308.2 - 308.2 | | | 101.0 - 101.0 |
| TOLUENE | Narendra et al, 2012 ^1237^ | | 4 | 4 | | 303.2 - 318.2 | | | 101.0 - 101.0 |
| TOLUENE | Sarkar et al, 2012 ^239^ | | 1 | 1 | | 298.2 - 298.2 | | | 101.0 - 101.0 |
| TOLUENE | Yadava et al, 2012 ^240^ | | 2 | 2 | | 303.2 - 308.2 | | | 101.0 - 101.0 |
| TOLUENE | Assael Mylona, 2013 ^1238^ | | 24 | 24 | | 293.3 - 332.7 | | | 101.0 - 17830.0 |
| TOLUENE | Bhalodia Sharma, 2013 ^242^ | | 3 | 3 | | 303.2 - 313.2 | | | 101.0 - 101.0 |
| TOLUENE | Meng et al, 2013 ^1114^ | | 25 | 25 | | 273.1 - 363.2 | | | 1000.0 - 30000.0 |
| TOLUENE | Prak et al, 2014 ^308^ | | 7 | 7 | | 293.2 - 363.2 | | | 101.0 - 101.0 |
| TOLUENE | Vijayalakshmi et al, 2014 ^1239^ | | 2 | 2 | | 303.2 - 313.2 | | | 101.0 - 101.0 |
| TOLUENE | Bajic et al, 2015 ^1240^ | | 1 | 1 | | 298.2 - 298.2 | | | 101.0 - 101.0 |
| TOLUENE | Lowry Nasini, 1929 ^243^ | | 1 | 0 | | 10000.0 - 0.0 | | | 1000000000.0 - 0.0 |
| TOLUENE | Ling Van Winkle, 1958 ^43^ | | 2 | 2 | | 403.1 - 423.1 | | | 101.0 - 101.0 |
| TOLUENE | Akhundov et al, 1983 ^1218^ | | 114 | 109 | | 398.1 - 673.1 | | | 102.0 - 39412.0 |
| TOLUENE | Vogel Hendl, 1992 ^1084^ | | 88 | 84 | | 307.8 - 630.8 | | | 6.0 - 234.0 |

| Material | | Author | *N*_all_ | | | *N*_used_ | | *T* / K | *p* / kPa | |
| --- | --- | --- | --- | --- | --- | --- | --- | --- | --- | --- |
| WATER | Thorpe Rodger, 1894 ^28^ | | | 12 | 9 | | 303.9 - 371.2 | | | 3.0 - 81.0 |
| WATER | Kerimov et al, 1969 ^1241^ | | | 36 | 36 | | 373.1 - 548.1 | | | 87.0 - 5758.0 |
| WATER | Rivkin et al, 1970 ^1242^ | | | 17 | 14 | | 523.1 - 638.1 | | | 3810.0 - 19703.0 |
| WATER | Kingham et al, 1974 ^1243^ | | | 19 | 0 | | 10000.0 - 0.0 | | | 1000000000.0 - 0.0 |
| WATER | Assael et al, 1993 ^1244^ | | | 5 | 4 | | 298.0 - 312.9 | | | 2.0 - 6.0 |
| WATER | Dunstan, 1904 ^31^ | | | 1 | 1 | | 298.1 - 298.1 | | | 101.0 - 101.0 |
| WATER | Bingham et al, 1913 ^633^ | | | 32 | 32 | | 298.1 - 368.1 | | | 101.0 - 101.0 |
| WATER | Jones Davis, 1913 ^1245^ | | | 6 | 6 | | 298.1 - 318.1 | | | 101.0 - 101.0 |
| WATER | Bingham Jackson, 1918 ^1246^ | | | 21 | 21 | | 273.1 - 373.1 | | | 101.0 - 101.0 |
| WATER | Selwood Frost, 1933 ^474^ | | | 1 | 1 | | 293.1 - 293.1 | | | 101.0 - 101.0 |
| WATER | Bhagwat Mandloi, 1946 ^1247^ | | | 10 | 10 | | 293.1 - 370.1 | | | 101.0 - 101.0 |
| WATER | Babak, 1949 ^1248^ | | | 3 | 3 | | 298.1 - 323.1 | | | 101.0 - 101.0 |
| WATER | Vierk, 1950 ^635^ | | | 1 | 1 | | 293.1 - 293.1 | | | 101.0 - 101.0 |
| WATER | Ling Van Winkle, 1958 ^43^ | | | 8 | 8 | | 303.1 - 368.1 | | | 101.0 - 101.0 |
| WATER | Mikhail Kimel, 1961 ^946^ | | | 5 | 5 | | 298.1 - 323.1 | | | 101.0 - 101.0 |
| WATER | LeBel Goring, 1962 ^1249^ | | | 1 | 1 | | 298.0 - 298.0 | | | 101.0 - 101.0 |
| WATER | Mikhail Kimel, 1963 ^946^ | | | 5 | 5 | | 298.1 - 323.1 | | | 101.0 - 101.0 |
| WATER | Wallace Mathews, 1963 ^1250^ | | | 1 | 1 | | 298.0 - 298.0 | | | 101.0 - 101.0 |
| WATER | Wallace Mathews, ^1251^ | | | 1 | 1 | | 298.1 - 298.1 | | | 101.0 - 101.0 |
| WATER | Blumenshine Sears, 1966 ^1252^ | | | 5 | 5 | | 304.1 - 323.1 | | | 101.0 - 101.0 |
| WATER | Rivkin Levin, 1966^1253^ | | | 28 | 28 | | 323.1 - 473.2 | | | 5000.0 - 5000.0 |
| WATER | Agaev Usibova, 1968 ^478^ | | | 277 | 277 | | 273.1 - 423.1 | | | 101.0 - 117790.0 |
| WATER | Jerome et al, 1968 ^573^ | | | 1 | 1 | | 298.1 - 298.1 | | | 101.0 - 101.0 |
| WATER | Wallace et al, 1968 ^1254^ | | | 1 | 1 | | 298.0 - 298.0 | | | 101.0 - 101.0 |
| WATER | Kerimov et al, 1969 ^1241^ | | | 189 | 189 | | 373.1 - 548.1 | | | 199.0 - 117778.0 |
| WATER | Konobeev Lyapin, 1970 ^45^ | | | 3 | 3 | | 293.1 - 333.1 | | | 101.0 - 101.0 |
| WATER | Rivkin et al, 1970 ^1242^ | | | 94 | 94 | | 496.6 - 648.1 | | | 2500.0 - 50000.0 |
| WATER | Zhigunov Ampelogova, 1972 ^807^ | | | 3 | 3 | | 293.1 - 313.1 | | | 101.0 - 101.0 |
| WATER | Hayduk et al, 1973 ^1255^ | | | 1 | 1 | | 298.1 - 298.1 | | | 101.0 - 101.0 |
| WATER | Kestin et al, 1977 ^256^ | | | 70 | 70 | | 283.7 - 311.9 | | | 100.0 - 31130.0 |
| WATER | Kubota et al, 1979 ^948^ | | | 28 | 28 | | 283.2 - 348.1 | | | 9910.0 - 68750.0 |
| WATER | Deckwer, 1980 ^1256^ | | | 7 | 7 | | 293.1 - 353.1 | | | 101.0 - 101.0 |
| WATER | Sacco et al, 1981 ^1257^ | | | 3 | 3 | | 303.1 - 323.1 | | | 101.0 - 101.0 |
| WATER | Collings Bajenov, 1983 ^1258^ | | | 59 | 59 | | 274.2 - 343.1 | | | 101.0 - 101.0 |
| WATER | James et al, 1984 ^1259^ | | | 8 | 8 | | 273.2 - 333.1 | | | 101.0 - 101.0 |
| WATER | Guarino et al, 1985 ^1260^ | | | 1 | 1 | | 278.0 - 278.0 | | | 101.0 - 101.0 |
| WATER | Al-Ghawas, 1989 ^1261^ | | | 10 | 10 | | 288.2 - 333.1 | | | 101.0 - 101.0 |
| WATER | Knapstad et al, 1989 ^164^ | | | 6 | 6 | | 292.9 - 347.6 | | | 101.0 - 101.0 |
| WATER | Oyevaar et al, 1989 ^574^ | | | 1 | 1 | | 298.0 - 298.0 | | | 101.0 - 101.0 |
| WATER | Palepu Clarke, 1989 ^1262^ | | | 4 | 4 | | 298.0 - 313.0 | | | 101.0 - 101.0 |
| WATER | Pandey et al, 1989 ^1263^ | | | 1 | 1 | | 303.1 - 303.1 | | | 101.0 - 101.0 |
| WATER | Laachach et al, 1992 ^1264^ | | | 6 | 4 | | 268.2 - 293.2 | | | 101.0 - 101.0 |
| WATER | Liu et al, 1992 ^1265^ | | | 3 | 3 | | 293.2 - 298.2 | | | 101.0 - 101.0 |
| WATER | Assael et al, 1993 ^1244^ | | | 13 | 13 | | 298.0 - 312.9 | | | 100.0 - 32010.0 |
| WATER | Das Hazra, 1993 ^1266^ | | | 3 | 3 | | 298.2 - 318.2 | | | 101.0 - 101.0 |
| WATER | Lee et al, 1993 ^1267^ | | | 5 | 5 | | 303.2 - 323.2 | | | 100.0 - 100.0 |
| WATER | Assael et al, 1994 ^656^ | | | 21 | 21 | | 293.1 - 328.1 | | | 100.0 - 32020.0 |
| WATER | Teng et al, 1994 ^490^ | | | 1 | 1 | | 298.2 - 298.2 | | | 101.0 - 101.0 |
| WATER | Liu et al, 1994 ^951^ | | | 4 | 4 | | 293.2 - 323.2 | | | 101.0 - 101.0 |
| WATER | Reddy et al, 1994 ^576^ | | | 1 | 1 | | 308.2 - 308.2 | | | 101.0 - 101.0 |
| WATER | Aminabhavi Gopalakrishna, 1995 ^290^ | | | 1 | 1 | | 298.2 - 298.2 | | | 101.0 - 101.0 |
| WATER | Lee Lin, 1995 ^660^ | | | 3 | 3 | | 303.2 - 323.2 | | | 101.0 - 101.0 |
| WATER | Rived et al, 1995 ^183^ | | | 1 | 1 | | 303.2 - 303.2 | | | 101.0 - 101.0 |
| WATER | Solimo Gramajo, 1995 ^1268^ | | | 1 | 1 | | 298.2 - 298.2 | | | 101.0 - 101.0 |
| WATER | Solimo Gramajo, 1995 ^1268^ | | | 1 | 1 | | 298.2 - 298.2 | | | 101.0 - 101.0 |
| WATER | Pal Singh, 1996 ^1269^ | | | 1 | 1 | | 308.2 - 308.2 | | | 101.0 - 101.0 |
| WATER | Kumagai Yokoyama, 1998 ^670^ | | | 2 | 2 | | 273.2 - 273.2 | | | 100.0 - 20000.0 |
| WATER | Moha-Ouchane et al, 1998 ^1270^ | | | 18 | 18 | | 303.2 - 343.2 | | | 100.0 - 100000.0 |
| WATER | Tsierkezos Molinou, 1998 ^579^ | | | 4 | 4 | | 283.2 - 313.2 | | | 101.0 - 101.0 |
| WATER | Henni et al, 1999 ^1271^ | | | 6 | 6 | | 298.2 - 353.2 | | | 101.0 - 101.0 |
| WATER | Pai Chen, 1999 ^1272^ | | | 1 | 1 | | 293.2 - 293.2 | | | 101.0 - 101.0 |
| WATER | Romero Rojas, 2000 ^1273^ | | | 1 | 1 | | 298.2 - 298.2 | | | 101.0 - 101.0 |
| WATER | Akhtar et al, 2001 ^1274^ | | | 5 | 5 | | 303.2 - 323.2 | | | 101.0 - 101.0 |
| WATER | Henni et al, 2001 ^1275^ | | | 5 | 5 | | 298.2 - 343.2 | | | 101.0 - 101.0 |
| WATER | Kapadi et al, 2001 ^198^ | | | 4 | 4 | | 303.2 - 318.2 | | | 101.0 - 101.0 |
| WATER | Roos Bart, 2001 ^1226^ | | | 1 | 1 | | 298.2 - 298.2 | | | 101.0 - 101.0 |
| WATER | Saleh et al, 2001 ^1276^ | | | 5 | 5 | | 303.2 - 323.2 | | | 101.0 - 101.0 |
| WATER | Saleh et al, 2001 ^1276^ | | | 1 | 1 | | 298.2 - 298.2 | | | 101.0 - 101.0 |
| WATER | Maham et al, 2002 ^1277^ | | | 5 | 5 | | 298.2 - 353.2 | | | 101.0 - 101.0 |
| WATER | Palaiologou et al, 2002 ^1278^ | | | 2 | 2 | | 278.2 - 293.2 | | | 101.0 - 101.0 |
| WATER | Saleh et al, 2002 ^1279^ | | | 5 | 5 | | 303.2 - 323.2 | | | 101.0 - 101.0 |
| WATER | Abdulagatov Azizov, 2003 ^1280^ | | | 27 | 27 | | 297.9 - 574.3 | | | 10000.0 - 30000.0 |
| WATER | George Sastry, 2003 ^202^ | | | 5 | 5 | | 298.2 - 338.2 | | | 101.0 - 101.0 |
| WATER | Henni et al, 2003 ^1281^ | | | 6 | 6 | | 298.2 - 343.2 | | | 92.0 - 92.0 |
| WATER | Kapadi et al, 2003 ^1282^ | | | 4 | 4 | | 303.2 - 318.2 | | | 101.0 - 101.0 |
| WATER | Uchida et al, 2003 ^1283^ | | | 40 | 40 | | 273.9 - 282.2 | | | 101.0 - 4920.0 |
| WATER | Belda et al, 2004 ^685^ | | | 7 | 7 | | 283.2 - 343.2 | | | 101.0 - 101.0 |
| WATER | Gonzalez et al, 2004 ^298^ | | | 3 | 3 | | 293.2 - 303.2 | | | 101.0 - 101.0 |
| WATER | Harris et al, 2004 ^446^ | | | 109 | 109 | | 255.7 - 298.2 | | | 100.0 - 389000.0 |
| WATER | Henni et al, 2004 ^1284^ | | | 6 | 6 | | 298.2 - 343.2 | | | 101.0 - 101.0 |
| WATER | Li et al, 2004 ^1285^ | | | 1 | 1 | | 293.2 - 293.2 | | | 101.0 - 101.0 |
| WATER | Abdulagatov et al, 2005 ^1286^ | | | 28 | 28 | | 299.8 - 574.5 | | | 100.0 - 30000.0 |
| WATER | Abdulagatov et al, 2005 ^1286^ | | | 16 | 16 | | 284.6 - 435.6 | | | 100.0 - 40000.0 |
| WATER | Abdulagatov et al, 2005 ^1286^ | | | 28 | 28 | | 296.7 - 573.7 | | | 5000.0 - 40000.0 |
| WATER | Bernal-Garcia et al, 2005 ^1287^ | | | 5 | 5 | | 313.2 - 353.2 | | | 101.0 - 101.0 |
| WATER | Abdulagatov Azizov, 2006 ^1054^ | | | 18 | 18 | | 308.5 - 578.5 | | | 10000.0 - 60000.0 |
| WATER | Abdulagatov et al, 2006 ^823^ | | | 16 | 16 | | 296.6 - 580.7 | | | 10000.0 - 30000.0 |
| WATER | Di et al, 2006 ^1083^ | | | 10 | 10 | | 298.2 - 353.2 | | | 101.0 - 101.0 |
| WATER | Liu et al, 2006 ^1288^ | | | 1 | 1 | | 298.2 - 298.2 | | | 101.0 - 101.0 |
| WATER | Roy Sinha, 2006 ^703^ | | | 1 | 1 | | 303.2 - 303.2 | | | 101.0 - 101.0 |
| WATER | Sinha Roy, 2006 ^587^ | | | 3 | 3 | | 298.2 - 318.2 | | | 101.0 - 101.0 |
| WATER | Sinha Roy, 2006 ^587^ | | | 3 | 3 | | 298.2 - 318.2 | | | 101.0 - 101.0 |
| WATER | Gurung Roy, 2007 ^590^ | | | 3 | 3 | | 298.2 - 318.2 | | | 101.0 - 101.0 |
| WATER | Pang et al, 2007 ^1289^ | | | 9 | 9 | | 293.2 - 333.2 | | | 101.0 - 101.0 |
| WATER | Abdulagatov Azizov, 2008 ^1290^ | | | 6 | 6 | | 298.2 - 368.6 | | | 100.0 - 40000.0 |
| WATER | Abu-Daabes Awwad, 2008 ^1291^ | | | 5 | 5 | | 293.2 - 333.2 | | | 101.0 - 101.0 |
| WATER | Gomez et al, 2008 ^713^ | | | 3 | 3 | | 298.2 - 328.2 | | | 100.0 - 100.0 |
| WATER | Gomez et al, 2008 ^713^ | | | 1 | 1 | | 298.2 - 298.2 | | | 101.0 - 101.0 |
| WATER | Gomez et al, 2008 ^713^ | | | 3 | 3 | | 298.2 - 328.2 | | | 100.0 - 100.0 |
| WATER | Nikam et al, 2008 ^1292^ | | | 2 | 2 | | 298.2 - 308.2 | | | 101.0 - 101.0 |
| WATER | Rehman ansari, 2008 ^715^ | | | 10 | 10 | | 278.1 - 323.2 | | | 101.0 - 101.0 |
| WATER | Besbes et al, 2009 ^1293^ | | | 1 | 1 | | 298.2 - 298.2 | | | 101.0 - 101.0 |
| WATER | Hartono Svendsen, 2009 ^1294^ | | | 8 | 8 | | 293.2 - 363.2 | | | 101.0 - 101.0 |
| WATER | Palani Geetha, 2009 ^1295^ | | | 3 | 3 | | 303.0 - 313.0 | | | 101.0 - 101.0 |
| WATER | Sadeghi et al, 2009 ^1296^ | | | 4 | 4 | | 293.2 - 308.2 | | | 101.0 - 101.0 |
| WATER | Wang et al, 2009 ^1297^ | | | 9 | 9 | | 298.2 - 338.2 | | | 101.0 - 101.0 |
| WATER | Anouti et al, 2010 ^726^ | | | 7 | 7 | | 293.2 - 323.2 | | | 101.0 - 101.0 |
| WATER | Blanco et al, 2010 ^1298^ | | | 5 | 5 | | 293.0 - 323.0 | | | 101.0 - 101.0 |
| WATER | Qiao et al, 2010 ^1299^ | | | 38 | 38 | | 313.2 - 473.2 | | | 100.0 - 3200.0 |
| WATER | Shekaari Mousavi, 2010 ^976^ | | | 4 | 4 | | 298.2 - 328.2 | | | 101.0 - 101.0 |
| WATER | Dhondge et al, 2011 ^1300^ | | | 2 | 2 | | 308.2 - 318.2 | | | 101.0 - 101.0 |
| WATER | Garcia-Abuin et al, 2011 ^732^ | | | 4 | 4 | | 293.2 - 323.2 | | | 101.0 - 101.0 |
| WATER | Goodwin Marsh, 2011 ^1301^ | | | 3 | 3 | | 293.2 - 293.2 | | | 101.0 - 101.0 |
| WATER | Liu et al, 2011 ^735^ | | | 12 | 12 | | 303.5 - 383.3 | | | 12000.0 - 28000.0 |
| WATER | Nain et al, 2011 ^1302^ | | | 6 | 6 | | 293.2 - 318.2 | | | 101.0 - 101.0 |
| WATER | Rajagopal Gladson, 2011 ^1303^ | | | 4 | 4 | | 303.2 - 318.2 | | | 101.0 - 101.0 |
| WATER | Lu et al, 2012 ^1304^ | | | 1 | 1 | | 288.2 - 288.2 | | | 101.0 - 101.0 |
| WATER | Narendra et al, 2012 ^1237^ | | | 6 | 6 | | 298.2 - 343.2 | | | 101.0 - 101.0 |
| WATER | Ouerfelli et al, 2012 ^1305^ | | | 3 | 3 | | 311.2 - 320.2 | | | 101.0 - 101.0 |
| WATER | Qian et al, 2012 ^982^ | | | 5 | 5 | | 293.2 - 313.2 | | | 101.0 - 101.0 |
| WATER | Quijada et al, 2012 ^742^ | | | 1 | 1 | | 298.2 - 298.2 | | | 101.0 - 101.0 |
| WATER | Ren et al, 2012 ^1306^ | | | 6 | 6 | | 293.2 - 342.2 | | | 101.0 - 101.0 |
| WATER | Bald et al, 2013 ^1307^ | | | 1 | 1 | | 298.2 - 298.2 | | | 101.0 - 101.0 |
| WATER | Banipal et al, 2013 ^1308^ | | | 1 | 1 | | 288.2 - 288.2 | | | 101.0 - 101.0 |
| WATER | Barega et al, 2013 ^566^ | | | 1 | 1 | | 298.2 - 298.2 | | | 101.0 - 101.0 |
| WATER | Chandra et al, 2013 ^1309^ | | | 4 | 4 | | 293.2 - 308.2 | | | 101.0 - 101.0 |
| WATER | Dhondge et al, 2013 ^1310^ | | | 3 | 3 | | 278.2 - 298.2 | | | 101.0 - 101.0 |
| WATER | Jozwiak et al, 2013 ^1311^ | | | 4 | 4 | | 293.2 - 308.2 | | | 101.0 - 101.0 |
| WATER | Li et al, 2013 ^344^ | | | 8 | 8 | | 278.2 - 313.2 | | | 101.0 - 101.0 |
| WATER | Moosavi et al, 2013 ^606^ | | | 3 | 3 | | 298.2 - 308.2 | | | 101.0 - 101.0 |
| WATER | Siongco et al, 2013 ^1312^ | | | 7 | 7 | | 293.2 - 343.2 | | | 101.0 - 101.0 |
| WATER | Spasojevic et al, 2013 ^493^ | | | 2 | 2 | | 293.2 - 298.2 | | | 101.0 - 101.0 |
| WATER | Xu et al, 2013 ^750^ | | | 5 | 5 | | 293.2 - 313.2 | | | 101.0 - 101.0 |
| WATER | Yang et al, 2013 ^751^ | | | 4 | 4 | | 293.2 - 308.2 | | | 101.0 - 101.0 |
| WATER | Andanson et al, 2014 ^1313^ | | | 1 | 1 | | 298.2 - 298.2 | | | 101.0 - 101.0 |
| WATER | Dhondge et al, 2014 ^1314^ | | | 3 | 3 | | 275.2 - 279.2 | | | 101.0 - 101.0 |
| WATER | Goncalves et al, 2014 ^754^ | | | 1 | 1 | | 298.2 - 298.2 | | | 100.0 - 100.0 |
| WATER | He et al, 2014 ^1315^ | | | 5 | 5 | | 298.2 - 338.2 | | | 101.0 - 101.0 |
| WATER | Jiang et al, 2014 ^1316^ | | | 4 | 4 | | 293.2 - 323.2 | | | 101.0 - 101.0 |
| WATER | Krolikowska et al, 2014 ^1317^ | | | 12 | 12 | | 298.2 - 343.2 | | | 101.0 - 101.0 |
| WATER | Kumar et al, 2014 ^1318^ | | | 3 | 3 | | 305.2 - 315.2 | | | 101.0 - 101.0 |
| WATER | Kumar et al, 2014 ^1318^ | | | 4 | 4 | | 288.2 - 318.2 | | | 101.0 - 101.0 |
| WATER | Lago et al, 2014 ^306^ | | | 1 | 1 | | 298.2 - 298.2 | | | 101.0 - 101.0 |
| WATER | Mehrdad et al, 2014 ^1319^ | | | 6 | 6 | | 288.2 - 313.2 | | | 100.0 - 100.0 |
| WATER | Navarro et al, 2014 ^1320^ | | | 9 | 9 | | 303.2 - 343.2 | | | 101.0 - 101.0 |
| WATER | Zawadzki et al, 2014 ^1321^ | | | 10 | 10 | | 298.2 - 343.2 | | | 101.0 - 101.0 |
| WATER | Zhu et al, 2014 ^756^ | | | 5 | 5 | | 293.2 - 333.2 | | | 101.0 - 101.0 |
| WATER | Cao et al, 2015 ^757^ | | | 1 | 1 | | 298.2 - 298.2 | | | 101.0 - 101.0 |
| WATER | Gercia et al, 2015 ^1322^ | | | 9 | 9 | | 303.2 - 343.2 | | | 101.0 - 101.0 |
| WATER | Kumar Chauchan, 2015 ^1323^ | | | 6 | 6 | | 293.2 - 318.2 | | | 100.0 - 100.0 |
| WATER | Raman et al, 2015 ^1324^ | | | 5 | 5 | | 303.2 - 323.2 | | | 101.0 - 101.0 |
| WATER | Xu et al, 2015 ^1325^ | | | 5 | 5 | | 293.2 - 313.2 | | | 101.0 - 101.0 |
| WATER | Ling Van Winkle, 1958 ^43^ | | | 3 | 3 | | 383.1 - 423.1 | | | 101.0 - 101.0 |
| WATER | Ling Van Winkle, 1958 ^43^ | | | 3 | 3 | | 383.1 - 423.1 | | | 101.0 - 101.0 |
| WATER | Luker Johnson, 1959 ^792^ | | | 2 | 0 | | 10000.0 - 0.0 | | | 1000000000.0 - 0.0 |
| WATER | Shifrin, 1959 ^1326^ | | | 114 | 114 | | 422.1 - 1139.7 | | | 101.0 - 101.0 |
| WATER | Rivkin et al, 1968 ^1327^ | | | 127 | 127 | | 548.1 - 723.1 | | | 614.0 - 35436.0 |
| WATER | Zhdanov et al, 1968 ^1328^ | | | 16 | 15 | | 423.1 - 923.1 | | | 0.0 - 137.0 |
| WATER | Timrot et al, 1973 ^1329^ | | | 46 | 46 | | 325.7 - 772.1 | | | 6.0 - 140.0 |
| WATER | Nagashima et al, 1974 ^1330^ | | | 53 | 53 | | 523.3 - 874.4 | | | 490.0 - 20780.0 |
| WATER | Timrot, 1950 ^1331^ | | | 69 | 65 | | 292.0 - 874.8 | | | 9091.0 - 34912.0 |
| WATER | Mineev Zaidel, 1968 ^1332^ | | | 4 | 2 | | 700.0 - 980.0 | | | 8.0 - 12.0 |
| WATER | Rivkin et al, 1973 ^1333^ | | | 59 | 59 | | 648.6 - 773.1 | | | 22500.0 - 50000.0 |
| WATER | Liu et al, 2012 ^1334^ | | | 128 | 128 | | 571.9 - 715.6 | | | 19000.0 - 28000.0 |
| WATER | Teske et al, 2005 ^1335^ | | | 109 | 85 | | 310.8 - 440.4 | | | 3.0 - 47.0 |

| Material | | Author | *N*_all_ | | | *N*_used_ | | *T* / K | *p* / kPa | |
| --- | --- | --- | --- | --- | --- | --- | --- | --- | --- | --- |
| XENON | Kestin et al, 1972 ^97^ | | | 8 | 8 | | 298.2 - 973.2 | | | 101.0 - 101.0 |
| XENON | Vogel, 1984 ^1336^ | | | 10 | 10 | | 295.1 - 647.2 | | | 101.0 - 101.0 |
| XENON | Strumpf et al, 1974 ^839^ | | | 189 | 189 | | 287.4 - 303.2 | | | 5827.0 - 9587.0 |
| XENON | Trautz Zimmermann, 1935 ^869^ | | | 5 | 5 | | 293.0 - 550.0 | | | 101.0 - 101.0 |
| XENON | Trautz Heberling, 1934 ^1337^ | | | 6 | 6 | | 289.7 - 550.0 | | | 101.0 - 101.0 |
| XENON | Trappeniers et al, 1965 ^1338^ | | | 23 | 13 | | 273.2 - 348.2 | | | 1207.0 - 42840.0 |
| XENON | Reyneys Thodos, 1964 ^1339^ | | | 48 | 48 | | 323.2 - 473.2 | | | 7136.0 - 83046.0 |
| XENON | Rankine, 1910 ^1340^ | | | 2 | 0 | | 10000.0 - 0.0 | | | 1000000000.0 - 0.0 |
| XENON | Nasini Rossi, 1928 ^1341^ | | | 4 | 4 | | 289.7 - 372.6 | | | 101.0 - 101.0 |
| XENON | Boon et al, 1967 ^1342^ | | | 9 | 9 | | 163.3 - 168.9 | | | 83.0 - 115.0 |
| XENON | Ulybin Makarushkin, 1977 ^1191^ | | | 68 | 68 | | 170.0 - 292.3 | | | 6651.0 - 54150.0 |
| XENON | Slyusar et al, 1972 ^1343^ | | | 27 | 24 | | 164.8 - 289.4 | | | 91.0 - 5806.0 |
| XENON | Borisov et al, 1973 ^98^ | | | 2 | 2 | | 300.0 - 300.0 | | | 7.0 - 27.0 |
| XENON | Dawe Smith, 1970 ^1035^ | | | 15 | 15 | | 293.2 - 1600.0 | | | 101.0 - 101.0 |
| XENON | Clarke Smith, 1968 ^1032^ | | | 9 | 8 | | 176.0 - 374.6 | | | 101.0 - 2552.0 |
| XENON | Roy et al, 1973 ^1344^ | | | 1 | 0 | | 10000.0 - 0.0 | | | 1000000000.0 - 0.0 |
| XENON | Thomas, 1960 ^1345^ | | | 1 | 1 | | 291.2 - 291.2 | | | 93.0 - 93.0 |
| XENON | Lukin et al, 1981 ^463^ | | | 2 | 2 | | 273.2 - 293.2 | | | 10.0 - 10.0 |
| XENON | Lukin et al, 1983 ^452^ | | | 15 | 15 | | 153.2 - 293.2 | | | 7.0 - 7.0 |
| XENON | Rigby Smith, 1966 ^1346^ | | | 14 | 14 | | 293.0 - 972.0 | | | 101.0 - 101.0 |
| XENON | Maitland Smith, 1972 ^1347^ | | | 19 | 19 | | 180.0 - 1500.0 | | | 101.0 - 101.0 |
| XENON | Kestin Leidenfrost, 1959 ^467^ | | | 8 | 8 | | 298.2 - 298.2 | | | 102.0 - 2841.0 |
| XENON | Goldblatt Wageman, 1971 ^1348^ | | | 11 | 11 | | 283.0 - 2000.0 | | | 101.0 - 101.0 |
| XENON | May et al, 2007 ^1349^ | | | 7 | 6 | | 202.9 - 298.2 | | | 101.0 - 2174.0 |
| XENON | Vogel et al, 1984 ^1350^ | | | 10 | 10 | | 295.1 - 647.2 | | | 101.0 - 101.0 |
| XENON | Rakshit Roy, 1974 ^1351^ | | | 4 | 3 | | 237.0 - 308.0 | | | 101.0 - 2597.0 |
| XENON | Kestin et al, 1978 ^1352^ | | | 9 | 9 | | 298.2 - 778.2 | | | 101.0 - 101.0 |
| XENON | Vogel, 2016 ^1353^ | | | 20 | 20 | | 295.0 - 649.5 | | | 62.0 - 339.0 |
| XENON | Vogel, 1984 ^1336^ | | | 10 | 10 | | 295.2 - 649.6 | | | 101.0 - 101.0 |
| XENON | Lin et al, 2016 ^1354^ | | | 9 | 9 | | 303.2 - 393.2 | | | 101.0 - 101.0 |
| XENON | Vogel, 1984 ^1336^ | | | 20 | 20 | | 295.1 - 649.6 | | | 101.0 - 101.0 |
| XENON | Golovicher, 1989 ^1355^ | | | 27 | 26 | | 170.0 - 2000.0 | | | 101.0 - 101.0 |
| XENON | Vargaftik, 1972 ^6^ | | | 47 | 46 | | 293.2 - 1600.0 | | | 100.0 - 80000.0 |
| XENON | Barr et al, 1981 ^1356^ | | | 18 | 15 | | 173.3 - 1597.4 | | | 101.0 - 1877.0 |
| XENON | Lukin et al, 1983 ^452^ | | | 14 | 11 | | 163.2 - 293.2 | | | 82.0 - 2610.0 |
| XENON | Sevast’yanov Zykov, 1978 ^1357^ | | | 24 | 23 | | 200.0 - 3000.0 | | | 100.0 - 100.0 |
| XENON | Scholz Kley, 1981 ^7^ | | | 9 | 9 | | 163.3 - 168.9 | | | 83.0 - 115.0 |
| XENON | Stephan Heckenberger ^770^ | | | 1787 | 1774 | | 170.0 - 1300.0 | | | 100.0 - 100000.0 |
| XENON | Scholz Kley, 1981 ^7^ | | | 23 | 21 | | 288.5 - 972.0 | | | 101.0 - 101.0 |
| XENON | Slyusar et al, 1972 ^1343^ | | | 29 | 24 | | 161.4 - 289.7 | | | 1249.0 - 20232.0 |
| XENON | Reynes Thodos, 1964 ^1339^ | | | 48 | 48 | | 323.2 - 473.2 | | | 7136.0 - 83046.0 |
| XENON | Boon et al, 1967 ^1342^ | | | 9 | 9 | | 163.3 - 168.9 | | | 18474.0 - 20768.0 |
| XENON | Strumpf et al, 1974 ^839^ | | | 190 | 190 | | 287.4 - 303.2 | | | 5827.0 - 9581.0 |
| XENON | Borisov et al, 1973 ^98^ | | | 1 | 1 | | 300.0 - 300.0 | | | 101.0 - 101.0 |
| XENON | Ulybin Makarushkin, 1977 ^1191^ | | | 45 | 45 | | 170.0 - 250.0 | | | 6651.0 - 54030.0 |

Reference

(1)	Edwards, D. A.; Bonilla, C. F. Viscosity of Liquid Styrene and Butadiene. *Ind. Eng. Chem.* **1944**, *36*, 1038–1040. https://doi.org/10.1021/ie50419a016.

(2)	Neduzhii, I. A.; Khmara, Yu. I. Experimental Investigations of the Liquid - Viscosity of Propylene, Isobutylene, Butadiene-1,3, Toluol and Cyclohexane. *Thermophys. Prop. Gases Liquids* **1968**, *1*, 158–160.

(3)	Senftleben, H.; Gladisch. A Method of Determining the Specific Heats, the Viscosities and the Thermal Conductivities of Gases (in German). *Z. Phys.* **1949**, *125*, 653.

(4)	Dunlop, P. J. Viscosities of a Series of Gaseous Fluorocarbons at 25 .Degree.C. *J. Chem. Phys.* **1994**, *100*, 3149–3151. https://doi.org/10.1063/1.466405.

(5)	Liessmann, G.; Schmidt, W.; Reiffarth, S. Recommended Thermophysical Data. *Data compilation of the Saechsische Olefinwerke Boehlen Germany* **1995**, 1.

(6)	Vargaftik, N. B. Dictionary of Thermophysical Properties of Gases and Liquids. **1972**, 178–321.

(7)	Scholz, A.; Kley, G. Collection of Transport Property Data. **1981**.

(8)	Golubev, I. F. *Viscosity of Gases and Gas Mixtures*; 1959.

(9)	Titani, T. The Viscosity of Vapors of Organic Compounds. Part II. *Bull. Chem. Soc. Jpn.* **1930**, *5*, 98.

(10)	Smutny, E. J.; Bondi, A. A. Di-Tert-Butyl Ether: Strain Energy and Physical Properties. *J. Phys. Chem.* **1961**, *65*, 546–550.

(11)	Brazier, D. W.; Freeman, G. R. The Effect of Pressure on the Density, Dielectric Constant, and Viscosity of Several Hydrocarbons and Other Organic Liquids. *Can. J. Chem.* **1969**, *47*, 893–899. https://doi.org/10.1139/v69-147.

(12)	Lambert, J. D.; Cotton, K. J.; Pailthorpe, M. W.; Robinson, A. M.; Scrivins, J.; Vale, W. R. F.; Young, R. M. Transport Properties of Gaseous Hydrocarbons. *Proc. R. Soc. London, Ser. A* **1955**, *231*, 280–290. https://doi.org/10.1098/rspa.1955.0173.

(13)	Chavanne, G.; Van Risseghem, H. *Bull. Soc. Chim. Belg.* **1922**, *31*, 87.

(14)	Aucejo, A.; CruzBurguet, M.; Munoz, R.; Marques, J. L. Densities, Viscosities, and Refractive Indices of the Binary Liquid Systems n-Alkanes + Isomers of Hexane at 298.15 K. *J. Chem. Eng. Data* **1995**, *40*, 871–874. https://doi.org/10.1021/je00020a029.

(15)	Kuss, E.; Pollmann, P. The Pressure Dependence of the Viscosity and the Degree of Branching of Liquid Alkanes. *Z. Phys. Chem. (Munich)* **1969**, *68*, 205–227.

(16)	Diaz Pena, M.; Esteban, F.; Sanchez, M. A. Viscosity of Hydrocarbons. I. Hexane and Isomers. *An. R. Soc. Esp. Fis. Quim. Ser. A* **1967**, *63*, 103–108.

(17)	McCall, D. W.; Douglass, D. C.; Anderson, E. W. Self-Diffusion in Liquids: Paraffin Hydrocarbons. *Phys. Fluids (1958-1988)* **1959**, *2*, 87–91. https://doi.org/10.1063/1.1724398.

(18)	Touazi, A. A.; Khellili, E.; Didaoui, S.; Khimeche, K.; Benziane, M. Measurement and Correlation of Isobaric Densities and Viscosities for Endo-Dicyclopentadiene with Hydrocarbons (C6-C8) at Different Temperatures. *J. Therm. Anal. Calorim* **2018**, *134*, 1223–1242. https://doi.org/10.1007/s10973-018-7583-2.

(19)	Neumann, S. Unpublished Data from Thesis. *Unpublished Data* **1995**.

(20)	Gorbachev, M. Y.; Bersuker, I. B.; Dimoglo, A. S. The Dependence of the Viscosity of Organic Nonassociated Liquids on the Structural and Conformational Characteristics of Two Molecules. *Teor. Eksp. Khim.* **1989**, *25*, 563.

(21)	Eicher, L. D.; Zwolinski, B. J. Molecular Structure and Shear Viscosity. Isomeric Hexanes. *J. Phys. Chem.* **1972**, *76*, 3295.

(22)	Ling, A. C.; Willard, J. E. Viscosities of Some Organic Glasses Used as Trapping Matrices. *J. Phys. Chem.* **1968**, *72*, 1918.

(23)	Gramajo de Doz, M. B.; Cases, A. M.; Bonatti, C. M.; Solimo, H. N. Influence of Temperature on the (Liquid + Liquid) Equilibria of (3-Methyl Pentane + Cyclopentane + Methanol) Ternary System at T = (293.15, 297.15, and 299.15) K. *J. Chem. Thermodyn.* **2009**, *41*, 1279–1283. https://doi.org/10.1016/j.jct.2009.05.016.

(24)	Tu, C.-H.; Ku, H.-C.; Wang, W.-F.; Chou, Y.-T. Volumetric and Viscometric Properties of Methanol, Ethanol, Propan-2-Ol, and 2-Methylpropan-2-Ol with a Synthetic C6+ Mixture from 298.15 K to 318.15 K. *J. Chem. Eng. Data* **2001**, *46*, 317–321. https://doi.org/10.1021/je0002888.

(25)	Bouzas, A.; Burguet, M. C.; Monton, J. B.; Munoz, R. Densities, Viscosities, and Refractive Indices of the Binary Systems Methyl Tert-Butyl Ether + 2-Methylpentane, + 3-Methylpentane, + 2,3-Dimethylpentane, and + 2,2,4-Trimethylpentane at 298.15 K. *J. Chem. Eng. Data* **2000**, *45*, 331–333. https://doi.org/10.1021/je9902793.

(26)	Wen, C.; Meng, X.; Wei, K.; Wu, J. Compressed Liquid Viscosity of 2-Methylpentane, 3-Methylpentane, and 2,3-Dimethylbutane at Temperatures from (273 to 343) K and Pressures up to 40 MPa. *J. Chem. Eng. Data* **2017**, *62*, 1146–1152. https://doi.org/10.1021/acs.jced.6b01024.

(27)	Mohammed, G. A.; Oudah, M. Kh. Density, Viscosity and Refractive Index of the Ternary System (Ethanol + N-Hexane + 3-Methyl Pentane) at 20, 30, 40 C and 101.325 Kpa. *Iraqi J. Chem. Petrol. Eng.* **2015**, *16*, 51–58.

(28)	Thorpe, T. E.; Rodger, J. W. *Philos. Trans. R. Soc. London, Ser. A* **1894**, *185*, 522.

(29)	Batschinski, A. J. Investigations of the Internal Friction of Fluids. *Z. Phys. Chem.* **1913**, *84*, 643–706.

(30)	Bagdasaryan, S. S. Theory of Nuclei of a New Phase and the Limits of the Liquid State. *Zh. Fiz. Khim.* **1964**, *38*, 1816–1820.

(31)	Dunstan, A. E. LXXXIII. The Viscosity of Liquid Mixtures. Part. *J. Chem. Soc.* **1904**, *85*, 817–827.

(32)	Dunstan, A. E.; Hilditch, T. P. Determination of Viscosity and Other Physical Properties. Part II. Influence of Association of Unsaturted Groups. *Ber. Bunsen-Ges. Phys. Chem.* **1912**, *18*, 185.

(33)	Faust, O. The Viscosity of Liquid Mixtures, Their Dependence on Temperature and the Relation between the Viscosity of Liquids with Their Vapor Pressures. *Z. Phys. Chem., Stoechiom. Verwandschaftsl.* **1912**, *79*, 97.

(34)	Weissenberger, G.; Piatti, L. Uber Die Molekulberbindungen Der Phenole II. Das Verhalten Der Kresole Gegen Aromatische Kohlenwasserstoffe. *Monatsh. Chem.* **1924**, *45*, 281–285.

(35)	Weissenberger, G.; Schuster, F. Molecualr Compounds of Phenols III Behavior of Binary Systems with Phenols and Phenol Ethers. *Monatsh. Chem.* **1924**, *45*, 413.

(36)	Lewis, J. R. The Viscosity of Liquids Containing Dissolved Gases. *J. Am. Chem. Soc.* **1925**, *47*, 626–640. https://doi.org/10.1021/ja01680a007.

(37)	Timmermans, J.; Martin, F. Study of the Physical Constants of Twenty Organic Compounds of the Physical Constants of Twenty Organic Compounds. *J. Chim. Phys. Phys.-Chim. Biol.* **1928**, *25*, 411–451.

(38)	Bingham, E. C.; Brown, D. F. The Mixture Law. *J. Rheol.* **1932**, *3*, 95–112.

(39)	Hughes, O. L.; Hartley, H. Effect of Small Water Additions upon the Conductivity of Electrolytes in Non-Aqueous Solvents. *Philos. Mag.* **1933**, *15*, 610.

(40)	Reynolds, M. B.; Kraus, C. A. *J. Am. Chem. Soc.* **1948**, *70*, 1709–1713. https://doi.org/10.1021/ja01185a012.

(41)	Karr, A. E.; Bowes, W. M.; Scheibel, E. G. Analysis of Binary and Ternary Mixtures System Acetone + Chloroform + Methyl Isobutyl Ketone. *Anal. Chem.* **1951**, *23*, 459–463.

(42)	Jullander, I. Solubility Properties of Nonionic Water-Soluble Cellulose Ethers in Mixtures of Water and Alcohol. *Acta Chem. Scand.* **1955**, *9*, 1620.

(43)	Ling, T. D.; Van Winkle, M. Interfacial Tension at the Boiling Point and Vapor Viscosity Near the Dew Point of Binary Mixtures. *Ind. Eng. Chem. Chem. Eng. Data Ser.* **1958**, *3*, 82–88.

(44)	Blank, M. G. Miscibility in Ternary Liquid Systems Containing Formamide and Acetone. *Zh. Fiz. Khim.* **1968**, *42*, 3056–3062.

(45)	Konobeev, B. I.; Lyapin, V. V. Density, Viscosity, and Surface Tension Data on Certain Binary Systems. *Zh. Prikl. Khim.* **1970**, *43*, 803–810.

(46)	Campbell, R. H.; Kartzmark, E. M. Thermodynamic and Other Properties of Methanol + Acetone, Carbon Disulphide + Acetone, Carbon Disulphide + Methanol, and Carbon Disulphide + Methanol + Acetone. *J. Chem. Thermodyn.* **1973**, *5*, 163–172. https://doi.org/10.1016/s0021-9614(73)80076-x.

(47)	Terent’eva, A. A.; Krumgal’z, B. S.; Gerzhberg, Yu. I. Density and Viscosity of Acetone and Methyl Ethyl Ketone at Temp. below 0. *Zh. Prikl. Khim.* **1973**, *46*, 1143.

(48)	Atoyan, V. A.; Mamedov, I. A. Complex Study of Acoustic and Viscous Properties of Dimethyl Ketone in a Wide Range of Temperatures and Pressures. *Zh. Fiz. Khim.* **1976**, *50*, 2976–2978.

(49)	Hafez, M.; Hartland, S. Densities and Viscosities of Binary Systems Toluene-Acetone and 4-Methyl-2-Pentanone-Acetic Acid at 20, 25, 35, and 45 Deg C. *J. Chem. Eng. Data* **1976**, *21*, 179–182. https://doi.org/10.1021/je60069a011.

(50)	Kerchove, F. V.; De Vijlder, M. Some Physicochemical Properties of the Binary Mixtures Heptane-Propanone and Heptane-Ethyl Acetate. *J. Chem. Eng. Data* **1977**, *22*, 333–337. https://doi.org/10.1021/je60074a005.

(51)	Yadava, R. R.; Yadava, S. S. Viscosmetric Study of Molecular Interactions in Binary Liquid Mixtures of Polar and Nonpolar Solvents. *Indian J. Chem., Sect. A: Inorg., Phys., Theor. Anal.* **1981**, *20*, 221–224.

(52)	Noda, K.; Ohashi, M.; Ishida, K. Viscosities and Densities at 298.15 K for Mixtures of Methanol, Acetone and Water. *J. Chem. Eng. Data* **1982**, *27*, 326–328. https://doi.org/10.1021/je00029a028.

(53)	Prakash, S.; Srivastava, S.; Singh, R. Molecular Interaction of Benzyl Alcohol with Dioxan and Acetone at 30deg C, 35deg C and 40deg C. *Acustica* **1990**, *71*, 1990.

(54)	Schumpe, A.; Luehring, P. Oxygen Diffusivities in Organic Liquids at 293.15 K. *J. Chem. Eng. Data* **1990**, *35*, 24–25. https://doi.org/10.1021/je00059a007.

(55)	Das, A. K.; Jha, B. L. Molecular Interactions in Binary Mixture of DMSO-Aromatic/Aliphatic Hydrocarbons. *J. Mol. Liq.* **1991**, *50*, 155–186.

(56)	Zhang, J.; Liu, H. Determination of Viscosity under Pressure for Organic Substances and Its Correlation with Density under High Pressure. *Huagong Xuebao* **1991**, *42*, 269–277.

(57)	Liu, H.; Luo, Z. Experimental Determination of the Density and Viscosity for Liquid Mixtures. *Huaxue Gongcheng* **1992**, *20*, 65–68.

(58)	Lorenzi, L. D.; Fermeglia, M.; Torriano, G. Densities and Viscosities of 1,1,1-Trichloroethane with 13 Different Solvents at 298.15 K. *J. Chem. Eng. Data* **1995**, *40*, 1172–1177. https://doi.org/10.1021/je00022a003.

(59)	Tsierkezos, N. G.; Molinou, I. E. Densities and Viscosities of Ethylene Glycol Binary Mixtures at 293.15 K. *J. Chem. Eng. Data* **1999**, *44*, 955–958. https://doi.org/10.1021/je990059p.

(60)	Kinart, C. M.; Kinart, W. J.; Cwiklinska, A. Density and Viscosity at Various Temperatures for 2-Methoxyethanol + Acetone Mixtures. *J. Chem. Eng. Data* **2002**, *47*, 76–78. https://doi.org/10.1021/je010213j.

(61)	Peng, I.-H.; Tu, C.-H. Densities and Viscosities of Acetone, Diisopropyl Ether, Ethanol, and Methyl Ethyl Ketone with a Five-Component Hydrocarbon Mixture from 288.15 K to 308.15 K. *J. Chem. Eng. Data* **2002**, *47*, 1457–1461. https://doi.org/10.1021/je020077y.

(62)	Govindarajan, S.; Kannappan, V.; Naresh, M. D.; Venkataboopathy, K.; Lokanadam, B. Ultrasonic Studies on the Molecular Interaction of Gallic Acid in Aqueous Methanol and Acetone Solutions and the Role of Gallic Acid as Viscosity Reducer. *J. Mol. Liq.* **2003**, *107*, 289–316.

(63)	Agarwal, D.; Singh, M. Densities and Viscosities of Binary Liquid Mixtures of Trichloroethylene and Tetrachloroethylene with Some Polar and Nonpolar Solvents. *J. Chem. Eng. Data* **2004**, *49*, 1218–1224. https://doi.org/10.1021/je034203p.

(64)	Jain, P.; Singh, M. Density, Viscosity, and Excess Properties of Binary Liquid Mixtures of Propylene Carbonate with Polar and Nonpolar Solvents. *J. Chem. Eng. Data* **2004**, *49*, 1214–1217. https://doi.org/10.1021/je034204h.

(65)	Chen, S.; Lei, Q.; Fang, W. Viscosities and Densities for Binary Mixtures of N-Methylpiperazine with Methanol, Ethanol, n-Propanol, Iso-Propanol, n-Butanol and Iso-Butanol at 293.15, 298.15 and 303.15K. *Fluid Phase Equilib.* **2005**, *234*, 22–33. https://doi.org/10.1016/j.fluid.2005.05.012.

(66)	Gonzalez, B.; Dominguez, A.; Tojo, J.; Esteves, M. J. C.; Cardoso, M. J. E. d. M.; Barcia, O. E. Dynamic Viscosities of KI or NH4I in Methanol and NH4I in Ethanol at Several Temperatures and 0.1 MPa. *J. Chem. Eng. Data* **2005**, *50*, 109–112. https://doi.org/10.1021/je049808f.

(67)	Wang, J.; Zhu, A.; Zhuo, K. Excess Molar Volumes and Excess Logarithm Viscosities for Binary Mixtures of the Ionic Liquid 1-Butyl-3-Methylimidazolium Hexaflurophosphate with Some Organic Compounds. *J. Solution Chem.* **2005**, *34*, 585–596.

(68)	Gonzalez, E. J.; Alonso, L.; Dominguez, A. Physical Properties of Binary Mixtures of the Ionic Liquid 1-Methyl-3-Octylimidazolium Chloride with Methanol, Ethanol, and 1-Propanol at T = (298.15, 313.15, and 328.15) K and at P ) 0.1 MPa. *J. Chem. Eng. Data* **2006**, *51*, 1446–1452. https://doi.org/10.1021/je060123k.

(69)	Ren, Y.; Xia, S.; Ma, P. Measurement of Densities and Viscosities of Crotonic Acid - Solvent Binary Systems. *Shiyou Huagong* **2007**, *36*, 267.

(70)	Sih, R.; Armenti, M.; Mammucari, R.; Dehghani, F.; Foster, N. R. Viscosity Measurements on Saturated Gas-Expanded Liquid Systems Ethanol and Carbon Dioxide. *J. Supercrit. Fluids* **2008**, *43*, 460–468.

(71)	Tian, Y.; Wang, X.; Wang, J. Densities and Viscosities of 1-Butyl-3-Methylimidazolium Tetrafluoroborate + Molecular Solvent Binary Mixtures. *J. Chem. Eng. Data* **2008**, *53*, 2056–2059. https://doi.org/10.1021/je8001128.

(72)	Rajagopal, K.; Chenthilnath, S. Molecular Interaction Studies and Theoretical Estimation of Ultrasonic Speeds Using Scaled Particle Theory in Binary Mixtures of Toluene with Homologous Nitriles at Different Temperatures. *Thermochim. Acta* **2010**, *498*, 45–53. https://doi.org/10.1016/j.tca.2009.10.001.

(73)	Rajagopal, K.; Chenthilnath, S. Density and Viscosity of Ketones with Toluene at Different Temperatures and at Atmospheric Pressure. *J. Chem. Eng. Data* **2010**, *55*, 1060–1063. https://doi.org/10.1021/je900523k.

(74)	Yang, L.; Luo, T.-L.; Lian, H.-L.; Liu, G.-J. Density and Viscosity of (2,2-Dichloro-N,N-Di-2-Propenylacetamide + Acetone) and (2,2-Dichloro-N,N-Di-2-Propenylacetamide + Ethanol) at T = (278.15 to 313.15) K. *J. Chem. Eng. Data* **2010**, *55*, 1364–1367. https://doi.org/10.1021/je900535d.

(75)	Titani, T. The Viscosity of Vapors of Organic Compounds. Part III. *Bull. Chem. Soc. Jpn.* **1933**, *8*, 255–276.

(76)	Craven, P. M.; Lambert, J. D. The Viscosities of Organic Vapors. *Proc. R. Soc. (London)* **1951**, *205*, 439–449.

(77)	Atoyan, V. A.; Mamedov, I. A. Study of Acoustic and Viscous Properties of Certain Ketones in the Liquid Phase at V = Const and Dependence on Molecular Weight. *Zh. Fiz. Khim.* **1980**, *54*, 856–860.

(78)	Kiyama, R.; Makita, T. The Viscosity of Carbon Dioxide, Ammonia, Acetylene, Argon and Oxygen under High Pressures. *Rev. Phys. Chem. Jpn.* **1952**, *22*, 49.

(79)	Wobser, R.; Mueller, F. The Viscosity of Gases and Vapors and Their Mixtures in the Hoeppler Viscosimeter (in German). *Kolloid-Beih.* **1941**, *52*, 165–276.

(80)	Vogel, H. Uber Die Viskosital Einiger Gase Und Ihre Temperaturabhangigkeit Bei Tiefen Temperaturen. *Ann. Phys. (Leipzig)* **1914**, *43*, 1235.

(81)	Bailey, B. J. The Viscosity of Carbon Dioxide and Acetylene at Atmospheric Pressure. *J. Phys. D* **1970**, *3*, 550–562. https://doi.org/10.1088/0022-3727/3/4/312.

(82)	Pal, A. K.; Barua, A. K. Viscosity of Some Quadrupolar Gases and Vapors. *J. Chem. Phys.* **1968**, *48*, 872–874. https://doi.org/10.1063/1.1668726.

(83)	Makita, T. *Mem. Fac. Ind. Arts, Kyoto Techn. Univ., Sci. Technol.* **1955**, *4*, 19.

(84)	Makhija, R. C.; Stairs, R. A. Viscosity of Associated Liquids. *Can. J. Chem.* **1970**, *48*, 1214.

(85)	Laesecke, A.; Perking, R. A.; Nieto de Castro, C. A. Thermal Conductivity of R134a. *Fluid Phase Equilib.* **1992**, *80*, 263–274. https://doi.org/10.1016/0378-3812(92)87073-v.

(86)	Trautz, M.; Heberling, R. Viscosity of Ammonia & Its Mixture with Hydrogen, Nitrogen, Ethylene. *Ann. Phys. (Berlin)* **1931**, *10*, 155.

(87)	Van Cleave, A. B.; Maass, O. The Molecular Diameter of Deuterium as Determined by Viscosity Measurements. *Can. J. Res.* **1935**, *12*, 57–62.

(88)	Burch, L. G.; Raw, C. J. G. Transport Properties of Polar-Gas Mixtures. I. Viscosities of Ammonia- Methylamine Mixtures. *J. Chem. Phys.* **1967**, *47*, 2798–2801.

(89)	Carmichael, L. T.; Reamer, H. H.; Sage, B. H. Viscosity of Ammonia at High Pressures. *J. Chem. Eng. Data* **1963**, *8*, 400–404. https://doi.org/10.1021/je60018a032.

(90)	Sun, S. B. K.; Storvick, T. S. Viscosity of Ammonia at High Temperature and Pressure. *J. Chem. Eng. Data* **1979**, *24*, 88–91. https://doi.org/10.1021/je60081a009.

(91)	Hellemans, J.; Zink, H.; Van Paemel, O. The Viscosity of Liquid Nitrogen and Liguid Oxigen along Isoterms as a Function of Pressure. *Physica (Amsterdam)* **1970**, *47*, 45.

(92)	vanderGulik, P. S.; Trappeniers, N. J. The Viscosity of Argon at High Densities. *Physica A (Amsterdam)* **1986**, *135*, 1–20.

(93)	Andreev, I. I.; Tsderberg, N. V.; Popov, V. N. Experimental Study of the Viscosity of Argon. *Teploenergetika* **1966**, 78–81.

(94)	Timrot, D. L.; Serednitskaya, M. A.; Traktueva, S. A. Viscosity of Gases as Studied by the Oscillating Disk Method. *Teploenergetika* **1969**, *16*, 83–85.

(95)	Kalelkar, A. S.; Kestin, J. Viscosity of He-Ar and He-Kr Binary Gaseous Mixtures in the Temperature Range 25-720 C. *J. Chem. Phys.* **1970**, *52*, 4248.

(96)	Kestin, J.; Wakeham, W.; Watanabe, K. Viscosity, Thermal Conductivity, and Diffusion Coefficient of Ar-Ne and Ar- Kr Gaseous Mixtures in the Temperature Range 25-700 C. *J. Chem. Phys.* **1970**, *53*, 3773–3780.

(97)	Kestin, J.; Ro, S. T.; Wakeham, W. An Extended Law of Corresponding States for the Equilibrium and Transport Properties of the Noble Gases. *Phys. Norv.* **1972**, *58*, 165–211.

(98)	Borisov, S. F.; Kalinin, B. A.; Porodnov, B. T.; Suetin, P. E. Measurements of the Viscosity Coefficients of Gases by the Method of Non Steady-State Flow. *Inzh.-Fiz. Zh.* **1973**, *24*, 67–69.

(99)	Kurin, V. I.; Golubev, I. F. Viscosity of Argon, Air, and Carbon Dioxide at Pressures to 400 at and Different Temperatures. *Teploenergetika* **1974**, *21*, 125.

(100)	Timrot, D. L.; Serednitskaya, M. A.; Traktueva, S. A. Investigation of Viscosity of Air at Temperatures 300-700 K and Pressures 0 .1 - 12 MPa by Oscillating Disk Method. *Teploenergetika* **1975**, *22*, 84–87.

(101)	Lyusternik, V. E.; Lavushev, A. V. Study of the Viscosity of Argon up to 2000 K with the Method of Flow through the Porous Media. *Teplofiz. Vys. Temp.* **1976**, *14*, 970–977.

(102)	Kestin, J.; Wakeham, W. A. The Viscosity of Three Polar Gases. *Ber. Bunsen-Ges. Phys. Chem.* **1979**, *83*, 573.

(103)	Hobley, A.; Matthews, G. P.; Townsend, A. The Use of a Novel Capillary Flow Viscometer for the Study of the Argon/ Carbon Dioxide System. *Int. J. Thermophys.* **1989**, *10*, 1165–1179. https://doi.org/10.1007/bf00500569.

(104)	Evers, C.; Loesch, H. W.; Wagner, W. An Absolute Viscometer-Densimeter and Measurements of the Viscosity of Nitrogen, Methane, Helium, Neon, Argon, and Krypton over a Wide Range of Density and Temperature. *Int. J. Thermophys.* **2002**, *23*, 1411–1439. https://doi.org/10.1023/a:1020784330515.

(105)	Hurly, J. J.; Gillis, K. A.; Mehl, J. B.; Moldover, M. R. The Viscosity of Seven Gases Measured with a Greenspan Viscometer. *Int. J. Thermophys.* **2003**, *24*, 1441–1474. https://doi.org/10.1023/b:ijot.0000004088.04964.4c.

(106)	Wang, X.; Wu, J.; Liu, Z. Viscosity of Gaseous HFC245fa. *J. Chem. Eng. Data* **2010**, *55*, 496–499. https://doi.org/10.1021/je900279z.

(107)	Wilhelm, J.; Vogel, E. Viscosity Measurements of Gaseous Argon, Krypton, and Propane. *Int. J. Thermophys.* **2000**, *21*, 301–318. https://doi.org/10.1023/a:1006667125801.

(108)	Vogel, E. Reference Viscosity of Argon at Low Density in the Temperature Range from 290 K to 680 K. *Int. J. Thermophys.* **2010**, *31*, 447–461. https://doi.org/10.1007/s10765-010-0760-6.

(109)	Carey, C.; Bradshaw, J.; Lin, E.; Carnevale, E. H. *Experimental Determination of Gas Properties at High Temperatures and/or Pressures*; AEDC-TR-74-33; Panametrics Inc. Experimetnal Determination of Gas Properties at High Temperatures and/or Pressures: Waltham, MA, 1974.

(110)	Goodwin, A. R. H.; Fitt, A. D.; Ronaldson, K. A.; Wakeham, W. A. A Vibrating Plate Fabricated by the Methods of Microelectromechanical Systems (MEMS) for the Simultaneous Measurement of Density and Viscosity: Results for Argon at Temperatures Between 323 and 423K at Pressures up to 68MPa. *Int. J. Thermophys.* **2006**, *27*, 1650–1676. https://doi.org/10.1007/s10765-006-0114-6.

(111)	Heydweiller, A. The Viscosity of Liquids above Their Boiling Points. *Wied. Ann.* **1896**, *59*, 193.

(112)	Khalilov, Kh. M. Viscosity of Liquids and Saturated Vapors at High Temperatures & Pressures. *Zh. Eksp. Teor. Fiz.* **1939**, *9*, 335–345.

(113)	Hammond, B. R.; Stokes, R. H. Diffusion in Binary Liquid Mixtures. Part 2 The Diffusion of Carbon Tetrachloride in Some Organic Solvents at 25 Deg. *Trans. Faraday Soc.* **1955**, *51*, 1641.

(114)	Kuss, E. High Pressure Measurements III. The Viscosity and Compressibility of Fluids. *Z. Angew. Phys.* **1955**, *7*, 372–378.

(115)	Heiks, J. R.; Orban, E. Liquid Viscosities at Elevated Tempaeratures and Pressures: Viscosity of Benzene from 90 Deg to Its Critical Temperature. *J. Phys. Chem.* **1956**, *60*, 1025.

(116)	Teller, A. J.; Walsh, T. J. Viscosities of Benzene-Methyl Ethyl Ketone Systems. *J. Chem. Eng. Data* **1959**, *4*, 279–283. https://doi.org/10.1021/je60003a023.

(117)	Bulanov, N. V.; Nikitin, E. D.; Skripov, V. P. Thermal Conductivbity of Liquids in the Metastable State. *Inzh.-Fiz. Zh.* **1974**, *26*, 204–207.

(118)	Gartenmeister, R. The Viscosity of Liquid Carbon Compounds and Its Relationship to Chemical Consitution. *Z. Phys. Chem., Stoechiom. Verwandschaftsl.* **1890**, *6*, 524.

(119)	Dunstan, A. E.; Thole, B. T.; Hunt, J. S. The Relation between Viscosity and Vhemical Constitution. Part I. The Viscosity of Pyridine Solutions. *J. Chem. Soc.* **1907**, *91*, 1728–1736.

(120)	Dunstan, A. E.; Stubbs, J. A. The Relation between Viscosity and Chemical Constitution. Part III. the Enol-Ketonic Tautomerism. *J. Chem. Soc. Trans.* **1908**, *93*, 1919.

(121)	Finney, R. J.; Fury, M.; Jonas, J. Density and Temperature Dependence of Self-Diffusion and Shear Viscosity of Perfluorocyclobutane in the Dense Fluid Region. *J. Chem. Phys.* **1977**, *66*, 760–765.

(122)	Dunstan, A. E.; Hilditch, T. P.; Thole, F. B. The Relation between Viscosity and Chemical Constitution: Vii the Effect of the Relative Position of Two Unsaturated Groups on Viscosity. *J. Chem. Soc. Trans.* **1913**, *103*, 133.

(123)	Muchin, G. Measurement of Fluidity on Solutions. *Z. Elektrochem. Angew. Phys. Chem.* **1913**, *19*, 819.

(124)	Bingham, E. C.; Sarver, L. A. Fluidities and Specific Volumes of Mixtures of Benzyl Benzoate and Benzene XXIX. *J. Am. Chem. Soc.* **1920**, *42*, 2011–2022. https://doi.org/10.1021/ja01455a007.

(125)	Meyer, J.; Mylius, B. Knowledge of Viscosity of Binary Liquid Mixtures. *Z. Phys. Chem., Stoechiom. Verwandschaftsl.* **1920**, *95*, 349.

(126)	Schaaf, F. Higher Alcohols from Petroleum Olefins. *Z. Phys. Chem. (Leipzig)* **1923**, *126*, 237.

(127)	Miller, C. C. The Stokes-Einstein Law for Diffusion in Solution. *Proc. R. Soc. London, Ser. A* **1924**, *106*, 724.

(128)	Kolossowsky, N. D. *Bull. Soc. Chim. Belg.* **1925**, *34*, 221.

(129)	Martin, F. *Bull. Soc. Chim. Belg.* **1925**, *34*, 82.

(130)	Dessart, A. Theory of Concentrated Solutions: III. Physical Constants of Mixtures of m-Nitrotoluene and m-Toluidine with Some Hydrocarbons. *Bull. Soc. Chim. Belg.* **1926**, *35*, 9.

(131)	Timmermans, J.; Martin, F. ? *J. Chim. Phys. Phys.-Chim. Biol.* **1926**, *23*, 747.

(132)	Madge, E. W. *J. Phys. Chem.* **1930**, *34*, 1599.

(133)	Tausz, J.; Staab, A. Lubricating Oils. *Petroleum Z.* **1930**, *26*, 1129–1140.

(134)	Kireev, V. A.; Skvortsova, A. A. Equilibrium in Liquid Mixtures and Solutions: Vii Elasticity, Vapor- Tension, Compositon and Some Other Properties of Binary Mixtures Benzene + Dichloroethane. *Zh. Fiz. Khim.* **1936**, *7*, 63–70.

(135)	Shalberov, N. A.; Ostroumov, V. V. Use of Gas Viscometers Opf the Rankin Type for Measuring Viscosity of Liquids. *Zh. Fiz. Khim.* **1936**, *7*, 552.

(136)	Lemonde, H. Diffusion and Viscosity in Binary Liquid Mixtures. *Ann. Phys. (Berlin)* **1938**, *9*, 539–644.

(137)	Salceanu, C. New Method for the Determination of the Viscosity of Liquids. *C. R. Hebd. Seances Acad. Sci.* **1939**, *208*, 1797–1799.

(138)	Udovenko, V. V.; Toropov, A. P. *Zh. Obshch. Khim.* **1940**, *10*, 11–16.

(139)	Linke, R. The Viscosity of Binary Liquid Mixtures. *Z. Phys. Chem. Abt. A* **1941**, *188*, 17.

(140)	Teitel’baum, B. Ya.; Gortalova, T. A.; Ganelina, S. G. Viscosity of Systems Formed by Benzenes and Its Homologs with the Lower Alcohols. *Zh. Obshch. Khim.* **1950**, *20*, 1422.

(141)	Subnis, M. V. Rheochor Part II. (Application of Hammick and Andrews Equation to Binary Mixtures). *Univ. Rajputana Studies, Phys. Sci.* **1952**, 121–127.

(142)	Dixon, J. A.; Schiessler, R. W. *J. Phys. Chem.* **1954**, *58*, 430.

(143)	Katti, P. K.; Chaudhri, M. M.; Prakash, O. Viscosities of Binary Mixtures Involving Benzene, Carbon Tetrachloride and Cyclohexane. *J. Chem. Eng. Data* **1966**, *11*, 593–594. https://doi.org/10.1021/je60031a044.

(144)	Ridgway, K.; Butler, P. A. Some Physical Properties of the Ternary System Benzene-Cyclohexane-n-Hexane. *J. Chem. Eng. Data* **1967**, *12*, 509–515. https://doi.org/10.1021/je60035a012.

(145)	Collings, A. F.; McLaughlin, E. Torsional Crystal Technique for the Measurement of Viscosities of Liquids at High Pressure. *Trans. Faraday Soc.* **1971**, *67*, 340.

(146)	Mamedov, A. M.; Akhundov, T. S.; Ismail-Zade, Sh. M.; Tairov, A. D. Viscosity of Benzene. *Izv. Vyssh. Uchebn. Zaved., Neft Gaz* **1971**, *14*, 74–76.

(147)	Alms, G. R.; Bauer, D. R.; Brauman, J. I.; Pecora, R. Depolarized Rayleigh Scattering and Orientational Relaxation of Molecules in Solution. I. Benzene, Toluene, and p-Xylene. *J. Chem. Phys.* **1973**, *58*, 5570–5578.

(148)	Parkhurst, H. J.; Jonas, J. Dense Liquids. II. The Effect of Density and Temperature on Viscosity of Tetramethylsilane and Benzene. *J. Chem. Phys.* **1975**, *63*, 2705–2709.

(149)	Dhillon, M. S.; Chugh, H. S. Viscosities and Activation Energies of Flow of Mixtures Containing 1,2- Dibromoethane. *Thermochim. Acta* **1976**, *16*, 345–353. https://doi.org/10.1016/0040-6031(76)80027-5.

(150)	Asfour, A. A.; Dullien, F. A. L. Viscosities and Densities of Four Binary Liquid Systems at 25.00 Deg C. *J. Chem. Eng. Data* **1981**, *26*, 312–316. https://doi.org/10.1021/je00025a028.

(151)	Dymond, J. H.; Robertson, J.; Isdale, J. D. Transport Properties of Nonelectrolyte Liquid Mixtures. III. Viscosity Coefficients for n-Octane, n-Dodecane, and Equimolar Mixtures of n-Octane + n-Dodecane from 25 to 100.Degree. C at Pressures Up. *Int. J. Thermophys.* **1981**, *2*, 133–154. https://doi.org/10.1007/bf00503937.

(152)	Kashiwagi, H.; Makita, T. Viscosity of Twelve Hydrocarbon Liquids in the Temperature Range 298-348 K at Pressures Up To 110 MPa. *Int. J. Thermophys.* **1982**, *3*, 289–305. https://doi.org/10.1007/bf00502346.

(153)	Abdullaev, F. G.; Akhundov, R. T. Experimental Study of the Viscosity of Ethylbenzene and O-, m-, and p-Xylene in Gaseous Phase. *Izv. Vyssh. Uchebn. Zaved., Neft Gaz* **1983**, 28.

(154)	Albright, J. G.; Edge, A. V. J.; Mills, R. Relations Between Intradiffusion and Viscosity Coefficients Albright Equation. *J. Chem. Soc., Faraday Trans. 1* **1983**, *79*, 1327.

(155)	Oswal, S.; Rathnam, M. V. Viscosity Data of Binary Mixtures: Ethyl Acetate + Cyclohexane, + Benzene, + Toluene, + Ethylbenzene + Carbon Tetrachloride, and + Chloroform at 303.1 5 K. *Can. J. Chem.* **1984**, *62*, 2851–2853. https://doi.org/10.1139/v84-482.

(156)	Awwad, A. M.; Al-Azzawi, S. F.; Salman, M. A. Volumes and Viscosities of Benzene + N-Alkane Mixtures. *Fluid Phase Equilib.* **1986**, *31*, 171–182. https://doi.org/10.1016/0378-3812(86)90011-7.

(157)	Oswal, S. L.; Rao, A. V. Studies on Viscosity & Volume of Mixing of Binary Liquid Mixtures of Tertiary Amines with Cyclohexane & Benzene. *Indian J. Chem., Sect. A: Inorg., Phys., Theor. Anal.* **1985**, *24*, 1026.

(158)	Fischer, J.; Weiss, A. Transport Properties of Liquids. V. Self-Diffusion, Viscosity, and Mass Density of Ellipsoidal Shaped Molecules in the Pure Liquid Phase. *Ber. Bunsen-Ges. Phys. Chem.* **1986**, *90*, 896.

(159)	Manjeshwar, L. S.; Aminabhavi, T. M. Densities and Viscosities of Binary Liquid Mixtures at 45 Deg. C. *J. Chem. Eng. Data* **1987**, *32*, 409–412. https://doi.org/10.1021/je00050a006.

(160)	Ramanjaneyulu, K.; Chandrasekhar, A. C. H.; Venkateswarlu, P.; Krishnaiah, A. A Study of Interactions in Binary Mixtures from Viscosity Measurements. *Phys. Chem. Liq.* **1987**, *16*, 217–223.

(161)	Chandrasekhar, A. C. H.; Surendra Nath, K. N.; Krishnaiah, A. Volumetric and Transport Properties of Volumetric and Transport Properties of 1,1,1-Trichloroethane with Some Aliphatic, Alicyclic and Aromatic Hydrocarbons at 303.15 K. *Chem. Scr.* **1988**, *28*, 421–425.

(162)	Rao, K. P. C.; Reddy, K. S. Viscosities of Binary Liquid Mixtures of Cyclohexanone with Alkanes, Benzene, Toluene, and Tetrachloromethane at 298.15 K. *Can. J. Chem. Eng.* **1988**, *66*, 474.

(163)	Rathnam, M. V. Viscosities and Densities of SOme Binary Liquid Mixtures of Esters at 303.15 and 313.15 K. *J. Chem. Eng. Data* **1988**, *33*, 509–511. https://doi.org/10.1021/je00054a034.

(164)	Knapstad, B.; Skjolsvik, P. A.; Oye, H. A. Viscosity of Pure Hydrocarbons. *J. Chem. Eng. Data* **1989**, *34*, 37–43. https://doi.org/10.1021/je00055a013.

(165)	Kouris, S.; Panayioton, C. Dynamic Viscosity of Mixtures of Benzene, Ethanol, and n-Heptane. *J. Chem. Eng. Data* **1989**, *34*, 200–203. https://doi.org/10.1021/je00056a016.

(166)	Ruiz, E.; Urieta, J. S.; Santafe, J.; Gutierrez Losa, C. Viscosities of Benzene or Cyclohexane + 2,4-Lutidine, + 2,6-Lutidine, + Collidine, + Mesitylene, + m-Xylene, and + p-Xylene at 303.15, 313.15 and 323.15 K. *Bull. Soc. Chim. Fr.* **1989**, 304.

(167)	Schrodt, J. T.; Akel, R. M. Binary Liquid Viscosities and Their Estimation from Classical Solution Thermodynamics. *J. Chem. Eng. Data* **1989**, *34*, 8–13. https://doi.org/10.1021/je00055a003.

(168)	Joshi, S. S.; Aminabhavi, T. M.; Balundgi, R. H.; Shukla, S. S. Densities and Viscosities of Binary Liquid Mixtures of Nitrobenzene with Cyclohexane and N,N-Dimethylformamide. *J. Chem. Eng. Data* **1990**, *35*, 185–187. https://doi.org/10.1021/je00060a027.

(169)	Sekar, P. R.; Venkateswarlu, R.; Reddy, K. S. Excess Volumes, Isentropic Compressibilities, and Viscosities of Binary Mixtures Containing Cyclohexene. *Can. J. Chem.* **1990**, *68*, 363–368. https://doi.org/10.1139/v90-054.

(170)	Sivaramprasad, G.; Rao, M. V.; Prasad, D. H. L. Density and Viscosity of Ethanol + 1,2-Dichloroethane, Ethanol + 1,1,1- Trichloroethane, and Ethanol + 1,1,2,2-Tetrachloroethane Binary Mixtures. *J. Chem. Eng. Data* **1990**, *35*, 122–124. https://doi.org/10.1021/je00060a006.

(171)	Aminabhavi, T. M.; Aminabhavi, V. A.; Joshi, S. S.; Balundgi, R. H. Excess Properties of Some Binary Liquid Mixtures in the Temperature Range 298.15-313.15 K. *Indian J. Technol.* **1991**, *29*, 545–557.

(172)	Assael, M. J.; Papadaki, M.; Wakeham, W. A. Measurements of the Viscosity of Benzene, Toluene, and m-Xylene at Pressure up to 80 MPa. *Int. J. Thermophys.* **1991**, *12*, 449–457. https://doi.org/10.1007/bf00502361.

(173)	Papaioannou, D.; Evangelou, T.; Panayioton, C. Dynamic Viscosity of Multicomponent Liquid Mixtures. *J. Chem. Eng. Data* **1991**, *36*, 43–46. https://doi.org/10.1021/je00001a013.

(174)	Ramakanth, C.; Mukherjee, A. K.; Das, T. R. Diffusion Coeffcients for Selected Binary Liquid Systems: Cyclohexanes and n-Alkyl Alcohols. *J. Chem. Eng. Data* **1991**, *36*, 384–387. https://doi.org/10.1021/je00004a012.

(175)	Aralaguppi, M. I.; Aminabhavi, T. M.; Harogoppad, S. B.; Balundgi, R. H. Thermodynamic Interactions in Binary Mixtures of Dimethyl Sulfoxide with Benzene, Toluene, 1,3-Dimethylbenzene, 1,3,5-Trimethylbenzene, and Methoxybenzene from 298.15 to 308.15 K. *J. Chem. Eng. Data* **1992**, *37*, 298–303. https://doi.org/10.1021/je00007a006.

(176)	Klofutar, C.; Paljk, S.; Golc-Teger, S. Thermodynamic Functions of Activation for Viscous Flow of Cholesterol in Some Nonaqueous Solutions. *Thermochim. Acta* **1992**, *206*, 19–32. https://doi.org/10.1016/0040-6031(92)85280-9.

(177)	Oswal, S. L.; Patel, A. T. Viscosity of Binary Mixtures. II. n-Butyl, n-Hexyl, n-Octyl, n-Decyl, and n-Dodecylamine with Benzene and n-Hexyl, n-Decyl, and n-Dodecylamine with Cyclohexane. *Int. J. Thermophys.* **1992**, *13*, 629–641. https://doi.org/10.1007/bf00501945.

(178)	Tasioula-Margari, M.; Demetropoulos, I. N. Viscosities of Dilute Solutions of the Triglycerides Triolein, Trilinolein, Tristearin, Tripalmitin, and Trimyristin in Benzene and p-Xylene. *J. Chem. Eng. Data* **1992**, *37*, 77–79. https://doi.org/10.1021/je00005a023.

(179)	Shastri, S. S.; Mukherjee, A. K.; Das, T. R. Surface Tension of Some Binary Organic Solutions of Alkylbenzenes and 1-Alkanols. *J. Chem. Eng. Data* **1993**, *38*, 399–400. https://doi.org/10.1021/je00011a017.

(180)	Lafuente, C.; Lopez, M. C.; Santafe, J.; Royo, F. M.; Urieta, J. S. Excess Volumes and Excess Viscosities of Benzene with Picolines. *Thermochim. Acta* **1994**, *237*, 35–41. https://doi.org/10.1016/0040-6031(94)85181-6.

(181)	Exarchos, N. C.; Tasioula-Margari, M.; Demetropoulos, I. N. Viscosities and Densities of Dilute Solutions of Glycerol Trioleate + Octane, + p-Xylene, + Toluene, and + Chloroform. *J. Chem. Eng. Data* **1995**, *40*, 567–571. https://doi.org/10.1021/je00019a005.

(182)	Krishnan, K. M.; Rambabu, K.; Venkateswarlu, P.; Raman, G. K. A Study on Mixing Properties of Binary Mixtures of 2-Methoxyethanol with Aromatic Hydrocarbons. *J. Chem. Eng. Data* **1995**, *40*, 132–135. https://doi.org/10.1021/je00017a030.

(183)	Rived, F.; Roses, M.; Bosch, E. Densities, Refractive Indices, Absolute Viscosities, and Static Dielectric Constants of 2-Methylpropan-2-Ol + Hexane, + Benzene, + Propan-2-Ol, + Methanol, + Ethanol, and + Water at 303.2 K. *J. Chem. Eng. Data* **1995**, *40*, 1111–1114. https://doi.org/10.1021/je00021a017.

(184)	Swain, N.; Panda, D.; Singh, S. K.; Chakravortty, V. Viscosity and Density of Tri-n-Butyl Phosphate + Benzene + o-Xylene from 30 0C to 450C. *J. Chem. Eng. Data* **1997**, *42*, 1235–1237. https://doi.org/10.1021/je970129x.

(185)	Viera dos Santos, F. J.; Nieto de Castro, C. A. Viscosity of Toluene and Benzene under High Pressure. *Int. J. Thermophys.* **1997**, *18*, 367–378. https://doi.org/10.1007/bf02575167.

(186)	Casad, H.; Segade, L.; Franjo, C.; Jimenez Cuesta, E.; Paz Andrade, M. I. Excess Properties for Propyl Propanoate + Hexane + Benzene at 298.15 K. *J. Chem. Eng. Data* **1998**, *43*, 756–762. https://doi.org/10.1021/je9800609.

(187)	Swain, N.; Panda, D.; Singh, S. K.; Chakrovortty, V. VISCOSITIES AND DENSITIES OF BINARY LIQUID MIXTURES OF TBP WITH BENZENE, TOLUENE AND ORTHO-XYLENE. *J. Mol. Liq.* **1998**, *75*, 211–218.

(188)	Aminabhavi, T. M.; Banerjee, K. Density, Viscosity, Refractive Index, and Speed of Sound in Binary Mixtures of 1-Chloronaphthalene with Benzene, Methylbenzene, 1,4-Dimethylbenzene, 1,3,5-Trimethylbenzene, and Methoxybenzene at (298.15, 303.15, and 308.15) K. *J. Chem. Eng. Data* **1999**, *44*, 547–552. https://doi.org/10.1021/je980310l.

(189)	Aralaguppi, M. I.; Jadar, C. V.; Aminabhavi, T. M. Density, Refractive Index, Viscosity, and Speed of Sound in Binary Mixtures of Cyclohexanone with Hexane, Heptane, Octane, Nonane, Decane, Dodecane, and 2,2,4-Trimethylpentane. *J. Chem. Eng. Data* **1999**, *44*, 435–440. https://doi.org/10.1021/je9802266.

(190)	Guzman, C.; Lafuente, C.; Santafe, J.; Royo, F. M.; Urieta, J. S. Thermodynamic and Transport Properties of Binary Mixtures Containing 1,3-Dioxolane. *Int. J. Thermophys.* **1999**, *20*, 1435–1448. https://doi.org/10.1023/a:1021489005178.

(191)	Sastry, N. V.; Jain, N. J.; George, A.; Bahadur, P. Viscosities, Speeds of Sound and Excess Isentropic Compressibilities of Binary Mixtures of Alkyl Alkanoate-Hydrocarbons at 308.15 K and 318.15 K. *Fluid Phase Equilib.* **1999**, *163*, 275–289. https://doi.org/10.1016/s0378-3812(99)00232-0.

(192)	Swain, N.; Panda, D.; Singh, S. K.; Chakravortty, V. Viscosity and Density of Tri-n-Butyl Phosphate + Benzene + Toluene from 30 Deg C to 45 Deg C. *J. Chem. Eng. Data* **1999**, *44*, 32–34. https://doi.org/10.1021/je980143p.

(193)	Mariano, A.; Postigo, M.; Artigas, H.; Pardo, J.; Royo, F. M. Densities and Viscosities of the Ternary Mixture (Benzene 1-Propanol Ethyl Acetate) at 298.15 K. *Phys. Chem. Liq.* **2000**, *38*, 567–581.

(194)	Swain, N.; Singh, S. K.; Panda, D.; Chakravortty, V. Viscosities and Densities of Ternary Liquid Mixtures of DEHPA, Benzene and Ortho-Xylene. *J. Mol. Liq.* **2000**, *85*, 321–330.

(195)	Tsierkezos, N. G.; Palaiologou, M. M.; Molinou, I. E. Densities and Viscosities of 1-Pentanol Binary Mixtures at 293.15 K. *J. Chem. Eng. Data* **2000**, *45*, 272–275. https://doi.org/10.1021/je9902138.

(196)	Casad, H.; Segade, L.; Cabeza, O.; Franjo, C.; Jimenez Cuesta, E. Densities, Viscosities, and Refractive Indexes for Propyl Propanoate + 1-Hexanol + Benzene at 298.15 K. *J. Chem. Eng. Data* **2001**, *46*, 651–656. https://doi.org/10.1021/je0003593.

(197)	Chowdhury, M. A.; Majid, M. A.; Saleh, M. A. Volumetric and Viscometric Behaviour of Binary Systems: (1-Hexanol + Hydrocarbons). *J. Chem. Thermodyn.* **2001**, *33*, 347–360. https://doi.org/10.1006/jcht.2000.0751.

(198)	Kapadi, U. R.; Hundiwale, D. G.; Patil, N. B.; Lande, M. K.; Patil, P. R. Studies of Viscosity and Excess Molar Volume of Binary Mixtures of Propane-1,2 Diol with Water at Various Temperatures. *Fluid Phase Equilib.* **2001**, *192*, 63–70. https://doi.org/10.1016/s0378-3812(01)00621-5.

(199)	Pandey, J. D.; Dubey, N.; Dwivedi, D. C.; Dey, R. PREDICTION OF HEAT OF MIXING FROM INTERNAL PRESSURE DATA. *Phys. Chem. Liq.* **2001**, *39*, 781–790.

(200)	Swain, N.; Singh, S. K.; Panda, D.; Chakravortty, V. Viscosities, Densities and Excess Properties of Binary Mixtures of Di-(2-Ethyl Hexyl) Phosphoric Acid (DEHPA) with Benzene, Toluene and Ortho-Xylene. *J. Mol. Liq.* **2001**, *94*, 233–248.

(201)	Nayak, J. N.; Aralaguppi, M. I.; Aminabhavi, T. M. Density, Viscosity, Refractive Index, and Speed of Sound in the Binary Mixtures of Ethyl Chloroacetate with Aromatic Liquids at 298.15, 303.15, and 308.15 K. *J. Chem. Eng. Data* **2002**, *47*, 964–969. https://doi.org/10.1021/je0200158.

(202)	George, J.; Sastry, N. V. Thermophysical Properties of Binary Mixtures of Methyl Methacrylate+Di-Ethers (Ethyl, Isopropyl, and Butyl) at 298.15 and 308.15 K. *Int. J. Thermophys.* **2003**, *24*, 1697–1719. https://doi.org/10.1023/b:ijot.0000004100.28834.0a.

(203)	Lark, B. S.; Mehra, M.; Oswal, S. L.; Ghael, N. Y. Viscosity of Some Binary Mixtures of Arenes. *Int. J. Thermophys.* **2003**, *24*, 1475–1494. https://doi.org/10.1023/b:ijot.0000004089.27285.6c.

(204)	Nikam, P. S.; Kharat, S. J. Densities, Viscosities, and Thermodynamic Properties of (N,N-Dimethylformamide + Benzene + Chlorobenzene) Ternary Mixtures at (298.15, 303.15, 308.15, and 313.15) K. *J. Chem. Eng. Data* **2003**, *48*, 1202–1207. https://doi.org/10.1021/je030130y.

(205)	Oswal, S. L.; Maisuria, M. M.; Gardas, R. L. Viscosity of Binary Mixtures of Cycloalkane with Cycloalkane, Alkane and Aromatic Hydrocarbon at 303.15 K. *J. Mol. Liq.* **2003**, *108*, 199–215.

(206)	Ali, A.; Nain, A. K.; Lal, B.; Chand, D. Densities, Viscosities, and Refractive Indices of Binary Mixtures of Benzene with Isomeric Butanols at 30C. *Int. J. Thermophys.* **2004**, *25*, 1835–1847. https://doi.org/10.1007/s10765-004-7738-1.

(207)	Ma, P. S.; Zhou, Q.; Yang, C.; Xia, S. MEASUREMENT OF VISCOSITIES AND DENSITIES OF L IQUIDS BY VIBRATIN G-WIRE METHOD. *Huagong Xuebao* **2004**, *55*, 1608.

(208)	Oswal, S. L.; Desai, J. S.; Ijardar, S. P. Studies of Viscosities of Dilute Solutions of Alkylamine in Non-Electrolyte Solvents I. Aliphatic and Aromatic Hydrocarbons. *Thermochim. Acta* **2004**, *423*, 29–41. https://doi.org/10.1016/j.tca.2004.04.015.

(209)	Yang, C.; Xu, W.; Ma, P. Thermodynamic Properties of Binary Mixtures of P-Xylene with Cyclohexane, Heptane, Octane, and N-Methyl-2-Pyrrolidone at Several Temperatures. *J. Chem. Eng. Data* **2004**, *49*, 1794–1801. https://doi.org/10.1021/je049776w.

(210)	Oswal, S. L.; Gardas, R. L.; Phalak, R. P. Densities, Speeds of Sound, Isentropic Compressibilities, Refractive Indices and Viscosities of Binary Mixtures of Tetrahydrofuran with Hydrocarbons at 303.15 K. *J. Mol. Liq.* **2005**, *116*, 109–118.

(211)	Ali, A.; Nain, A. K.; Chand, D.; Ahmad, R. Volumetric, Ultrasonic, Viscometric and Refractive Index Behavior of Binary Mixtures of 2,2,4-Trimethylpentane with Aromatic Hydrocarbons: An Experimental and Theoretical Study. *J. Mol. Liq.* **2006**, *128*, 32–41.

(212)	Ali, A.; Tariq, M. Thermodynamic and Transport Behaviour of Binary Liquid Mixtures of Benzyl Alcohol with Monocyclic Aromatics at 303.15 K. *J. Mol. Liq.* **2006**, *128*, 50–55.

(213)	Baragi, J. G.; Aralaguppi, M. I.; Kariduraganavar, M. Y.; Kulkarni, S. S.; Kittur, A.; Aminabhavi, T. M. Excess Properties of the Binary Mixtures of Methylcyclohexane + Alkanes (C6 to C12) at T = 298.15 K to T = 308.15 K. *J. Chem. Thermodyn.* **2006**, *38*, 75–83. https://doi.org/10.1016/j.jct.2005.03.024.

(214)	Han, K. J.; Oh, J.-H.; Park, S.-J. Densities and Viscosities for the Ternary Systems of Methyl Tert-Butyl Ether + Methanol + Benzene and Methyl Tert-Butyl Ether + Methanol + Toluene and Their Sub-Binary Systems at 298.15 K. *J. Chem. Eng. Data* **2006**, *51*, 1339–1344. https://doi.org/10.1021/je0600799.

(215)	Suresh Reddy, K. V. N.; Sankara Reddy, G.; Krishnaiah, A. Excess Volumes, Densities, Speeds of Sound and Viscosities for the Binary Systems of Diisopropyl Ether with Hydrocarbons at 303.15K. *Thermochim. Acta* **2006**, *440*, 43–50. https://doi.org/10.1016/j.tca.2005.10.008.

(216)	Varshney, S.; Singh, M. Densities, Viscosities, and Excess Molar Volumes of Ternary Liquid Mixtures of Bromobenzene + 1,4-Dioxane + (Benzene or + Toluene or + Carbon Tetrachloride) and Some Associated Binary Liquid Mixtures. *J. Chem. Eng. Data* **2006**, *51*, 1136–1140. https://doi.org/10.1021/je0600303.

(217)	Hernandez-Galvan, M. A.; Garcia-Sanchez, F.; Macias-Salinas, R. Liquid Viscosities of Benzene, n-Tetradecane, and Benzene + n-Tetradecane from 313 to 393K and Pressures up to 60MPa: Experiment and Modeling. *Fluid Phase Equilib.* **2007**, *262*, 51–60. https://doi.org/10.1016/j.fluid.2007.08.010.

(218)	Kharat, S. J.; Nikam, P. S. Density and Viscosity Studies of Binary Mixtures of Aniline+benzene and Ternary Mixtures of (Aniline+benzene+N,N-Dimethylformamide) at 298.15, 303.15, 308.15, and 313.15 K. *J. Mol. Liq.* **2007**, *131–132*, 81–86.

(219)	Nain, A. K. Ultrasonic and Viscometric Studies of Molecular Interactions in Binary Mixtures of Tetrahydrofuran with Some Aromatic Hydrocarbons at Temperatures from 288.15 to 318.15 K. *Phys. Chem. Liq.* **2007**, *45*, 371–388.

(220)	Reddy, K. V. N. S.; Rao, P. S.; Krishnaiah, A. Experimental and Theoretical Values of Sound Speeds and Viscosities for the Binary Systems of MTBE with Hydrocarbons. *J. Mol. Liq.* **2007**, *135*, 14–20.

(221)	Song, S.; Xia, S.; Ma, P.; Di, Z. Densities and Viscosities of N-Formylmorpholine + Benzene, + Toluene at Different Temperatures and Atmospheric Pressures. *J. Chem. Eng. Data* **2007**, *52*, 591–595. https://doi.org/10.1021/je060475r.

(222)	Yang, C.; Liu, Z.; Lai, H.; Ma, P. Thermodynamic Properties of Binary Mixtures of N-Methyl-2-Pyrrolidinone with Cyclohexane, Benzene, Toluene at (303.15 to 353.15) K and Atmospheric Pressure. *J. Chem. Thermodyn.* **2007**, *39*, 28–38. https://doi.org/10.1016/j.jct.2006.06.005.

(223)	Awwad, A. M.; Abu-Daabes, M. A. Densities, Viscosities, and Excess Properties of (N-Methylmorpholine + Cyclohexane, + Benzene, and + Toluene) at T = (298.15, 303.15, 313.15, 323.15) K. *J. Chem. Thermodyn.* **2008**, *40*, 645–652. https://doi.org/10.1016/j.jct.2007.11.002.

(224)	Shukla, R. K.; Shukla, S. K.; Pandey, V. K.; Awasthi, P. Excess Internal Pressure, Excess Energy of Vaporization and Excess Pseudo-Gruneisen Parameter of Binary, Ternary and Quaternary Liquid Mixtures. *J. Mol. Liq.* **2008**, *137*, 104–109.

(225)	Song, C.-Y.; Shen, H.-Z.; Zhao, J.-H.; Wang, L.-C.; Wang, F.-A. Densities and Viscosities of Binary Mixtures of Vitamin K3 with Benzene, Toluene, Ethylbenzene, o-Xylene, m-Xylene, and p-Xylene from (303.15 to 333.15) K. *J. Chem. Eng. Data* **2008**, *53*, 1110–1115. https://doi.org/10.1021/je7006549.

(226)	Wankhede, D. S.; Wankhede, N.; Lande, M.; Arbad, B. Densities and Viscosities of Propylene Carbonate with Aromatic Hydrocarbons (Benzene, 1,4-Dimethylbenzene and Ethylbenzene) at 288.15, 298.15 and 308.15?K. *Phys. Chem. Liq.* **2008**, *46*, 319–327.

(227)	Ali, A.; Nabi, F.; Tariq, M. Volumetric, Viscometric, Ultrasonic, and Refractive Index Properties of Liquid Mixtures of Benzene with Industrially Important Monomers at Different Temperatures. *Int. J. Thermophys.* **2009**, *30*, 464–474. https://doi.org/10.1007/s10765-009-0562-x.

(228)	Baylaucq, A.; Watson, G.; Zeberg-Mikkelsen, C.; Bazile, J.-P.; Boned, C. Dynamic Viscosity of the Binary System 1-Propanol + Toluene as a Function of Temperature and Pressure. *J. Chem. Eng. Data* **2009**, *54*, 2715–2721. https://doi.org/10.1021/je9002477.

(229)	Nain, A. K.; Chand, D.; Chandra, P.; Pandey, J. D. Excess Internal Pressures, Excess Free Volumes and Excess Thermodynamic Parameters of Some Non-Aqueous Binary Mixtures from Ultrasonic Speed, Density and Viscosity Data. *Phys. Chem. Liq.* **2009**, *47*, 195–209. https://doi.org/10.1080/00319100802072664.

(230)	Oswal, S. L.; Ghael, N. Y.; Gardas, R. L. Volumetric and Transport Properties of Ternary Mixtures Containing 1-Propanol + Ethyl Ethanoate + Cyclohexane or Benzene at 303.15 K: Experimental Data, Correlation and Prediction by ERAS Model. *Thermochim. Acta* **2009**, *484*, 11–21. https://doi.org/10.1016/j.tca.2008.11.007.

(231)	Sastry, N. V.; Thakor, R. R.; Patel, M. C. Excess Molar Volumes, Viscosity Deviations, Excess Isentropic Compressibilities and Deviations in Relative Permittivities of (Alkyl Acetates (Methyl, Ethyl, Butyl and Isoamyl) + n-Hexane, + Benzene, + Toluene, + (o-, m-, p-) Xylenes, + (Chloro-, Bromo-, Nitro-) Benzene at Temperatures from 298.15 to 313.15 K. *J. Mol. Liq.* **2009**, *144*, 13–22.

(232)	Yan, J.-H.; Dai, L.-Y.; Wang, X.-Z.; Chen, Y.-Q. Densities and Viscosities of Binary Mixtures of Cyclopropanecarboxylic Acid with Methanol, Ethanol, Propan-1-Ol, and Butan-1-Ol at Different Temperatures. *J. Chem. Eng. Data* **2009**, *54*, 1147–1152. https://doi.org/10.1021/je800930v.

(233)	Ikeuchi, H.; Kanakubo, M.; Okuno, S.; Sato, R.; Fujita, K.; Hamada, M.; Shoda, N.; Fukui, K.; Okada, K.; Kanazawa, H.; Iimori, A.; Miyake, D.; Takeda, T.; Sato, G. P. Densities and Viscosities of Tris(Acetylacetonato)Cobalt(III) Complex Solutions in Various Solvents. *J. Solution Chem.* **2010**, *39*, 1428–1453. https://doi.org/10.1007/s10953-010-9598-6.

(234)	Rathnam, M. V.; Jain, K.; Kumar, M. S. Physical Properties of Binary Mixtures of Ethyl Formate with Benzene, Isopropyl Benzene, Isobutyl Benzene, and Butylbenzene at (303.15, 308.15, and 313.15) K. *J. Chem. Eng. Data* **2010**, *55*, 1722–1726. https://doi.org/10.1021/je900689m.

(235)	Mariano, A.; Canzonieri, S.; Camacho, A.; Mainar, A.; Postigo, M. Viscometric and Volumetric Properties of Benzene + Methyl Acetate, or + Methyl Propanoate, or + Methyl Butanoate Binary Systems at 283.15, 298.15 and 313.15 K. *Phys. Chem. Liq.* **2011**, *49*, 720–728.

(236)	Pradhan, P.; Roy, M. N. Viscous Synergy and Antagonism, Excess Molar Volume, Isoentropic Compressibility and Excess Molar Refraction of Ternary Mixtures Containing Tetrahydrofuran, Methanol and Six Membered Cyclic Compounds at 298.15K. *Phys. Chem. Liq.* **2011**, *49*, 286–301. https://doi.org/10.1080/00319100903147886.

(237)	Shukla, R. K.; Awasthi, N.; Kumar, A.; Shukla, A.; Pandey, V. K. Prediction of Associational Behaviour of Binary Liquid Mixtures from Viscosity Data at 298.15, 303.15, 308.15 and 313.15 K. *J. Mol. Liq.* **2011**, *2011*, 131–138. https://doi.org/10.1016/j.molliq.2010.11.006.

(238)	Yadava, S. S.; Singh, S.; Bhan, R.; Yadav, N. Viscosities of Binary Mixtures of Ethanenitrile with Benzene and Several Substituted Benzenes. *Korean J. Chem. Eng.* **2011**, *28*, 256–260. https://doi.org/10.1007/s11814-010-0319-z.

(239)	Sarkar, B. K.; Choudhury, A.; Sinha, B. Excess Molar Volumes, Excess Viscosities and Ultrasonic Speeds of Sound of Binary Mixtures of 1,2-Dimethoxyethane with Some Aromatic Liquids at 298.15 K. *J. Solution Chem.* **2012**, *41*, 53–74. https://doi.org/10.1007/s10953-011-9780-5.

(240)	Yadava, S. S.; Bhan, R.; Yadav, N. Viscosities of Binary Liquid Mixtures of 2-Methylpropane Nitrile and Some Aromatic Hydrocarbons at 303.15 K and 308.15 K. *J. Solution Chem.* **2012**, *41*, 926–936. https://doi.org/10.1007/s10953-012-9850-3.

(241)	Bhatia, S. C.; Sangwan, J.; Rani, R.; Bhatia, R. Densities, Viscosities, Speeds of Sound, and Refractive Indices of Binary Mixtures of 2-Ethyl-1-Hexanol with Benzene and Halobenzenes. *Int. J. Thermophys.* **2013**, *34*, 2076–2088. https://doi.org/10.1007/s10765-013-1526-8.

(242)	Bhalodia, J.; Sharma, S. Viscometric, Acoustical and Spectroscopic Investigation of b-Pinene with Benzene, Toluene, m-Xylene and Mesitylene at 303.15, 308.15 and 313.15 K and Atmospheric Pressure. *J. Solution Chem.* **2013**, *42*, 1794–1815. https://doi.org/10.1007/s10953-013-0073-z.

(243)	Lowry, T. M.; Nasini, A. G. *Proc. R. Soc. London, Ser. A* **1929**, *123*, 686.

(244)	Adzumi, H. Studies on the Flow of Gaseous Mixtures through Capillaries. III. The Flow of Gaseous Mixtures at Medium Pressures. *Bull. Chem. Soc. Jpn.* **1937**, *12*, 292–303.

(245)	Maczek, A. O. S.; Edwards, C. J. C. The Viscosity and Binary Diffusion Coefficients of Some Gaseous Hydrocarbons, Fluorocarbons and Siloxanes. In *Symp. Transp. Prop. Fluids Fluid Mixtures: Their Meas. Estim. Correl. Use*; 1979; p 3.5.

(246)	Vogel, E.; Bich, E.; Nimz, R. The Initial Density Dependence of the Viscosity of Organic Vapors: Benzene and Methanol. *Physica A (Amsterdam)* **1986**, *139*, 188–207.

(247)	Kuenen, J. P.; Visser, S. W. The Virial Coefficient B for Normal Butane. *Versl. Gewone Vergad. Afd. Natuurk-d., K. Ned. Akad. Wet.* **1913**, *22*, 330.

(248)	Sage, B. H.; Yale, W. D.; Lacey, W. N. Effect of Pressure on Viscosity of Butane and Isobutane. *Ind. Eng. Chem.* **1939**, *31*, 223.

(249)	Goldhahn, H. Crotyl Bromide. *Chem. Tech. (Leipzig)* **1955**, *7*, 16.

(250)	Ratkovics, F.; Salamon, T.; Domonkos, L. Properties of Alcohol-Amine Mixtures, VI A Method for the Determination of the Average Degree of Association. *Acta Chim. Acad. Sci. Hung.* **1974**, *83*, 71.

(251)	Wobser, R.; Muller, F. Die Innere Reibung von Gasen Und Dampfen Und Ihre Messung. *Kolloidchem. Beih.* **1941**, *52*, 165.

(252)	Dolan, J. P.; Starling, K. E.; Lee, A. L.; Eakin, B. E.; Ellington, R. T. Liquid, Gas and Dense Fluid Viscosity of n-Butane. *J. Chem. Eng. Data* **1963**, *8*, 396. https://doi.org/10.1021/je60018a031.

(253)	Golubev, I. F.; Agaev, N. A. *Viscosity of Saturated Hydrocarbons*; 1964.

(254)	Kestin, J.; Ro, S. T.; Wakeham, W. A. Reference Values of the Viscosity of Twelve Gases at 25 Degrees C. *Trans. Faraday Soc.* **1971**, *67*, 2308–2313.

(255)	Diaz Pena, M.; Cheda, J. A. R. Viscosidad De Mezclas De N-Alcanos. *An. Quim.* **1975**, *71*, 257–261.

(256)	Kestin, J.; Khalifa, H. E.; Ro, S. T.; Wakeham, W. A. Preliminary Data on the Pressure Effect on the Viscosity of Sodium Chloride-Water Solutions in the Range 10-40 OC. *J. Chem. Eng. Data* **1977**, *22*, 207–214. https://doi.org/10.1021/je60073a008.

(257)	Abe, J.; Kestin, J.; Khalifa, H. E.; Wakeham, W. A. The Viscosity and Diffusion Coefficients of the Mixtures of Four Light Hydrocarbon Gases. *Physica A (Amsterdam)* **1978**, *93*, 155–170.

(258)	Abe, J.; Kestin, J.; Khalifa, H. E.; Wakeham, W. A. The Viscosity of Normal Butane, Isobutane, and Their Mixtures. *Physica A (Amsterdam)* **1979**, *97*, 296.

(259)	Lipkin, M. R.; Davison, J. A.; Kurtz, S. S. Viscosity of Propane, Butane, and Isobutane. *Ind. Eng. Chem.* **1942**, *34*, 976.

(260)	Carmichael, L. T.; Sage, B. H. Viscosity of Ethane at High Pressures. *J. Chem. Eng. Data* **1963**, *8*, 94–98. https://doi.org/10.1021/je60016a028.

(261)	Golubev, I. F.; Agaev, N. A. Generalization of the Viscosity Data for Saturated Hydrocarbons at Various Temperatures and Pressures. *Dokl. Akad. Nauk SSSR* **1963**, *151*, 875–878.

(262)	Diller, D. E.; Van Poolen, L. J. Measurements of the Viscosities of Saturated and Compressed Liquid Normal Butane and Isobutane. *Int. J. Thermophys.* **1985**, *6*, 43–62. https://doi.org/10.1007/bf00505791.

(263)	Kiran, E.; Sen, Y. L. High-Pressure Viscosity and Density of n-Alkanes. *Int. J. Thermophys.* **1992**, *13*, 411–442. https://doi.org/10.1007/bf00503880.

(264)	Kumagai, A.; Tomida, D.; Yokoyama, C. Measurements of the Liquid Viscosities of Mixtures of N-Butane, n-Hexane, and n-Octane with Squalane to 30MPa. *Int. J. Thermophys.* **2006**, *27*, 376–393. https://doi.org/10.1007/s10765-006-0053-2.

(265)	Bingham, E. C.; Fornwalt, H. J. Chemical Consitution and Association. *J. Rheol.* **1930**, *1*, 372–417.

(266)	Shepard, A. F.; Henne, A. L.; Midgley, T. Physical Properties of the Normal Paraffin Hydrocarbons Pentane to Dodecane. *J. Am. Chem. Soc.* **1931**, *53*, 1948–1958. https://doi.org/10.1021/ja01356a052.

(267)	Doolittle, A. K.; Peterson, R. H. Preparation of Physical Properties of a Series of N-Alkanes. *J. Am. Chem. Soc.* **1951**, *73*, 2145–2151. https://doi.org/10.1021/ja01149a069.

(268)	Rastorguev, Yu. L.; Keramidi, A. S. Experimental Study of the Viscosity of N-Undecane at High Pressures. *Izv. Vyssh. Uchebn. Zaved., Neft Gaz* **1971**, 59–63.

(269)	Guseinov, S. O.; Naziev, Y. M. Viscosity of N-Undecane and n-Heptadecane at High Pressures. *Izv. Vyssh. Uchebn. Zaved., Neft Gaz* **1973**, *16*, 61–63.

(270)	Ratkovics, F.; Salamon, T.; Domonkos, L. Properties of Mixtures Containing Amines and Alcohols. IV. Viscosities and Average Degrees of Association in n-Butylamine - 1-Butanol Mixtures. *Magy. Kem. Foly.* **1974**, *80*, 155–159.

(271)	Iwahashi, M.; Yamaguchi, Y.; Ogura, Y.; Suzuki, M. Dynamical Structures of Normal Alkanes, Alcohols. and Fatty Acids in the Liquid State as Determined by Viscosity, Self-Diffusion Coefficient, Infrared Spectra, and C NMR Spin-Lattice Relaxation Time Measurements. *Bull. Chem. Soc. Jpn.* **1990**, *63*, 2154–2158.

(272)	Assael, M. J.; Papadaki, M. Measurements of the Viscosity of N-Heptane, n-Nonane, and n-Undecane at Pressures up to 70 MPa. *Int. J. Thermophys.* **1991**, *12*, 801–810. https://doi.org/10.1007/bf00502407.

(273)	Zhang, L.; Guo, Y.; Wei, H.; Yang, F.; Fang, W.; Lin, R. Densities and Viscosities of Binary Mixtures of Exo-Tetrahydrodicyclopentadiene with N-Undecane or N-Tetradecane at T = (293.15 to 313.15) K. *J. Chem. Eng. Data* **2010**, *55*, 4108–4113. https://doi.org/10.1021/je100313a.

(274)	Chi, H.; Li, G.; Guo, Y.; Xu, L.; Fang, W. Excess Molar Volume along with Viscosity, Flash Point, and Refractive Index for Binary Mixtures of Cis-Decalin or Trans-Decalin with C9 to C11 n-Alkanes. *J. Chem. Eng. Data* **2013**, *58*, 2224–2232. https://doi.org/10.1021/je400250u.

(275)	Dymond, J. H.; Young, K. J. Transport Properties of Nonelectrolyte Liquid Mixtures I. Viscosity Coefficients for n-Alkane Mixtures at Saturation Pressure from 283 to 378 K. *Int. J. Thermophys.* **1980**, *1*, 331–344. https://doi.org/10.1007/bf00516562.

(276)	Evans, E. B. The Viscosity of Hydrocarbons. Parts IV-VI. *J. Inst. Pet.* **1938**, *24*, 321–327.

(277)	Cutler, W. G. A Study of the Compressions of Several High Molecular Weight Hydrocarbons. PhD Thesis, Pennsylvania State University, 1955.

(278)	Hogenboon, D. L.; Webb, W.; Dixon, J. A. Viscosity of Several Liquid Hydrocarbons as a Function of Temperature, Pressure, and Free Volume. *J. Chem. Phys.* **1967**, *46*, 2586–2597.

(279)	Anonymous, R. *Properties of Hydrocarbon of High Molecular Weight*; Univ.; Am. Pet. Inst. Res. Proj. 42, 1968.

(280)	Keramidi, A. S.; Rastorguev, Yu. L. Study of the Viscosity of N-Dodecane at High Pressures. *Izv. Vyssh. Uchebn. Zaved., Neft Gaz* **1970**, 108–114.

(281)	Jambon, C.; Delmas, G. Viscosities of Mixtures of SnR4 Compounds and of Some Other Large Globular Molecules. Effect of Size Difference. *Can. J. Chem.* **1977**, *55*, 1360–1366. https://doi.org/10.1139/v77-188.

(282)	Awwad, A. M.; Allos, E. I. Thermodynamic Properties of Binary Mixtures of Isooctane and N-Alkane at 298.15 K. *Fluid Phase Equilib.* **1985**, *22*, 353–366. https://doi.org/10.1016/0378-3812(85)87031-x.

(283)	Aucejo, A.; Part, E.; Medina, P.; Sancho-Tello, M. Viscosity of Some N-Alkane/1-Chloroalkane Binary Liquid Mixtures. *J. Chem. Eng. Data* **1986**, *31*, 143–145. https://doi.org/10.1021/je00044a003.

(284)	Awwad, A. M.; Salman, M. A. Excess Molar Volumes and Viscosities of Binary Mixtures of Cyclohexane and N-Alkane at 298.15 K. *Fluid Phase Equilib.* **1986**, *25*, 195–208. https://doi.org/10.1016/0378-3812(86)80015-2.

(285)	Ducoulombier, D.; Zhou, H.; Boned, C.; Peyrelasse, J.; Saint-Guirons, H.; Xans, P. Pressure (1-1000 Bars) and Temperature (20-100 C) Dependence of the Viscosity of Liquid Hydrocarbons. *J. Phys. Chem.* **1986**, *90*, 1692.

(286)	Wakefield, D. L.; Marsh, K. N. Viscosities of Nonelectrolyte Liquid Mixt. I. n-Hexadecane + n-Octane. *Int. J. Thermophys.* **1987**, *8*, 649–662. https://doi.org/10.1007/bf00500785.

(287)	Wakefield, D. L. Viscosities of Nonelectrolyte Liquid Mixtures. III. Selected Binar and Quaternary Mixtures. *Int. J. Thermophys.* **1988**, *9*, 365–381. https://doi.org/10.1007/bf00513077.

(288)	Knapstad, B.; Skjolsvik, P. A.; Oye, H. A. Viscosity of Three Binary Hydrocarbon Mixtures. *J. Chem. Eng. Data* **1991**, *36*, 84–88. https://doi.org/10.1021/je00001a025.

(289)	Aminabhavi, T. M.; Gopalkrishna, B. Densities, Viscosities, Refractive Indices, and Speeds of Sound of the Binary Mixtures of Bis(2-Methoxyethyl) Ether with Nonane,Decane, Dodecane, Tetradecane, and Hexadecane at 298.15, 308.15, and 31. *J. Chem. Eng. Data* **1994**, *39*, 529–534. https://doi.org/10.1021/je00015a029.

(290)	Aminabhavi, T. M.; Gopalakrishna, B. Density, Viscosity, Refractive Index, and Speed of Sound in Aqueous Mixtures of N,N-Dimethylformamide, Dimethyl Sulfoxide, N,N- Dimethylacetamide, Acetonitrile, Ethylene Glycol, Diethylene Glycol, 1,. *J. Chem. Eng. Data* **1995**, *40*, 856–861. https://doi.org/10.1021/je00020a026.

(291)	Aminabhavi, T. M.; Patil, V. B. Density, Refractive Index, Viscosity, and Speed of Sound in Binary Mixtures of Ethenylbenzene with Hexane, Heptane, Octane, Nonane, Decane, and Dodecane. *J. Chem. Eng. Data* **1997**, *42*, 641–646. https://doi.org/10.1021/je960382h.

(292)	Nayak, J. N.; Aralaguppi, M. I.; Aminabhavi, T. M. Density, Viscosity, Refractive Index, and Speed of Sound for the Binary Mixtures of Ethyl Chloroacetate with n-Alkanes (C6 to C12) at (298.15, 303.15, and 308.15) K. *J. Chem. Eng. Data* **2001**, *46*, 891–896. https://doi.org/10.1021/je010020w.

(293)	Garcia, B.; Alcalde, R.; Aparicio, S.; Leal, J. M. Thermophysical Behavior of Methylbenzoate + N-Alkanes Mixed Solvents. Application of Cubic Equations of State and Viscosity Models. *Ind. Eng. Chem. Res.* **2002**, *41*, 4399–4408. https://doi.org/10.1021/ie020008c.

(294)	Gonzalez, B.; Dominguez, A.; Tojo, J. Viscosities, Densities and Speeds of Sound of the Binary Systems: 2-Propanol with Octane, or Decane, or Dodecane at T=(293.15, 298.15, and 303.15) K. *J. Chem. Thermodyn.* **2003**, *35*, 939–953. https://doi.org/10.1016/s0021-9614(03)00047-8.

(295)	Moreiras, A. F.; Garcia, J.; Lugo, L.; Comunas, M. J. P.; Lopez, E. R.; Fernandez, J. Experimental Densities and Dynamic Viscosities of Organic Carbonate + N-Alkane or p-Xylene Systems at 298.15 K. *Fluid Phase Equilib.* **2003**, *204*, 233–243. https://doi.org/10.1016/s0378-3812(02)00261-3.

(296)	Caudwell, D. R. Viscosity of Dense Fluid Mixtures. PhD Thesis, Imperial College, 2004.

(297)	Caudwell, D.; Goodwin, A. R. H.; Trusler, J. P. M. A Robust Vibrating Wire Viscometer for Reservoir Fluids: Results for Toluene and n-Decane. *J. Pet. Sci. Eng.* **2004**, *44*, 333–340. https://doi.org/10.1016/j.petrol.2004.02.019.

(298)	Gonzalez, B.; Dominguez, A.; Tojo, J. Dynamic Viscosities, Densities, and Speed of Sound and Derived Properties of the Binary Systems Acetic Acid with Water, Methanol, Ethanol, Ethyl Acetate and Methyl Acetate at T = (293.15, 298.15, and 303.15) K at Atmospheric Pressure. *J. Chem. Eng. Data* **2004**, *49*, 1590–1596. https://doi.org/10.1021/je0342825.

(299)	Gonzalez, B.; Dominguez, A.; Tojo, J. Dynamic Viscosities of 2-Butanol with Alkanes (C8, C10, and C12) at Several Temperatures. *J. Chem. Thermodyn.* **2004**, *36*, 267–275. https://doi.org/10.1016/j.jct.2003.12.005.

(300)	Aljimaz, A. S.; Al-Kandary, J. A.; Abdul-Latif, A.-H. M.; Al-Zanki, A. M. Physical Properties of (Anisole + n-Alkanes) at Temperatures between (293.15 and 303.15) K. *J. Chem. Thermodyn.* **2005**, *37*, 631–642. https://doi.org/10.1016/j.jct.2004.09.021.

(301)	Goodwin, A. R. H.; Donzier, E. P.; Fitt, A. D.; Ronaldson, K. A.; Wakeham, W. A.; Manrique de Lara, M.; Marty, F.; Mercier, B. A Vibrating Edge Supported Plate, Fabricated by the Methods of Micro Electro Mechanical System for the Simultaneous Measurement of Density and Viscosity: Results for Methylbenzene and Octane at Temperatures between (323 and 423) K and Pressures in the Range (0.1 to 68) MPa. *J. Chem. Eng. Data* **2006**, *51*, 190–208. https://doi.org/10.1021/je0503296.

(302)	Tian, Q.; Liu, H. Densities and Viscosities of Binary Mixtures of Tributyl Phosphate with Hexane and Dodecane from (298.15 to 328.15) K. *J. Chem. Eng. Data* **2007**, *52*, 892–897. https://doi.org/10.1021/je060491o.

(303)	Zhang, L.; Yongsheng, G.; Xiao, J.; Gong, X.; Fang, W. Density, Refractive Index, Viscosity, and Surface Tension of Binary Mixtures of Exo-Tetrahydrodicyclopentadiene with Some n-Alkanes from (293.15 to 313.15) K. *J. Chem. Eng. Data* **2011**, *56*, 4268–4273. https://doi.org/10.1021/je200757a.

(304)	Mahajan, A. R. R. ].; Mirgane, S. R. R. ]. Study of Thermodynamic and Transport Properties of Binary Liquid Mixtures of N-Decane with Hexan-2-Ol, Heptan-2-Ol and Octan-2-Ol at T = 298.15 K. Experimental Results and Application of the Prigogine-Flory-Patterson Theory. *J. Chem. Thermodyn.* **2013**, *64*, 159–166. https://doi.org/10.1016/j.jct.2013.05.014.

(305)	Blahusiak, M.; Schlosser, S. Physical Properties of Phosphonium Ionic Liquid and Its Mixtures with Dodecane and Water. *J. Chem. Thermodyn.* **2014**, *72*, 54–64. https://doi.org/10.1016/j.jct.2013.12.022.

(306)	Lago, S.; Rodriguez-Cabo, B.; Arce, A.; Soto, A. Water/Oil/[P6,6,6,14][NTf2] Phase Equilibria. *J. Chem. Thermodyn.* **2014**, *75*, 63–68. https://doi.org/10.1016/j.jct.2014.02.012.

(307)	Liu, H.; Zhu, L. Excess Molar Volumes and Viscosities of Binary Systems of Butylcyclohexane with N-Alkanes (C7 to C14) at T = 293.15 K to 313.15 K. *J. Chem. Eng. Data* **2014**, *59*, 369–375. https://doi.org/10.1021/je400835u.

(308)	Prak, D. J. L.; Alexandre, S. M.; Cowart, J. S.; Trulove, P. C. Density, Viscosity, Speed of Sound, Bulk Modulus, Surface Tension, and Flash Point of Binary Mixtures of n-Dodecane with 2,2,4,6,6-Pentamethylheptane or 2,2,4,4,6,8,8-Heptamethylnonane. *J. Chem. Eng. Data* **2014**, *59*, 1334–1346. https://doi.org/10.1021/je5000132.

(309)	Singh, M. L.; Tripathi, S.; Lokhande, M.; Gandhi, P.; Gaikar, V. Density, Viscosity, and Interfacial Tension of Binary Mixture of Tri-Iso-Amyl Phosphate (TiAP) and n-Dodecane: Effect of Compositions and Gamma Absorbed Doses. *J. Chem. Eng. Data* **2014**, *59*, 1130–1139. https://doi.org/10.1021/je400493x.

(310)	Slusar’, V. P.; Rudenko, N. S.; Tret’yakov, V. M. Experimental Study of the Viscosity of Simple Substances along the Saturation Curve and under Pressure. *Ukr. Fiz. Zh.* **1973**, *18*, 190–194.

(311)	Coursey, B. M.; Heric, E. L. Viscosity of Some Binary Systems of Hexadecane and Normal Choroalkanes. *J. Chem. Eng. Data* **1969**, *14*, 426–430. https://doi.org/10.1021/je60043a015.

(312)	Rastorguev, Yu. L.; Keramidi, A. S. Viscosity of Hydrocarbons. *Izv. Vyssh. Uchebn. Zaved., Neft Gaz* **1972**, *15*, 61–66.

(313)	Matthews, M. A.; Rodden, J. B.; Akgerman, A. High-Temperature Diffusion, Viscosity, and Density Measurements in n-Hexadecane. *J. Chem. Eng. Data* **1987**, *32*, 317–319. https://doi.org/10.1021/je00049a011.

(314)	Dandekar, A. Y.; Andersen, S. I.; Stenby, E. H. Measurement of Viscosity of Hydrocarbon Liquids Using a Microviscometer. *J. Chem. Eng. Data* **1998**, *43*, 551–554. https://doi.org/10.1021/je9702807.

(315)	Fermeglia, M.; Torriano, G. Density, Viscosity, and Refractive Index for Binary Systems of n-C16 and Four Nonlinear Alkanes at 298.15 K. *J. Chem. Eng. Data* **1999**, *44*, 965–969. https://doi.org/10.1021/je9900171.

(316)	Queimada, A. J.; Quinones-Cisneros, S. E.; Marrucho, I. M.; Coutinho, J. A. P.; Stendy, E. H. Viscosity and Liquid Density of Asymmetric Hydrocarbon Mixtures. *Int. J. Thermophys.* **2003**, *24*, 1221–1239. https://doi.org/10.1023/a:1026142932619.

(317)	Tripathi, N. Densities, Viscosities, and Refractive Indices of Mixtures of Hexane with Cyclohexane, Decane, Hexadecane, and Squalane at 298.15K. *Int. J. Thermophys.* **2005**, *26*, 693–703. https://doi.org/10.1007/s10765-005-5572-8.

(318)	Aguila-Hernandez, J.; Trejo, A.; Garcia-Flores, B. E.; Molnar, R. Viscometric and Volumetric Behaviour of Binary Mixtures of Sulfolane and N-Methylpyrrolidone with Monoethanolamine and Diethanolamine in the Range 303 373 K. *Fluid Phase Equilib.* **2008**, *267*, 172–180. https://doi.org/10.1016/j.fluid.2008.02.023.

(319)	Dubey, G. P.; Sharma, M. Temperature and Composition Dependence of the Densities, Viscosities, and Speeds of Sound of Binary Liquid Mixtures of 1-Butanol with Hexadecane and Squalane. *J. Chem. Eng. Data* **2008**, *53*, 1032–1038. https://doi.org/10.1021/je7007654.

(320)	Dubey, G. P.; Sharma, M. Studies of Mixing Properties of Binary Systems of 2-Propanol with Hexadecane and Squalane at T = (298.15, 303.15, and 308.15) K. *J. Chem. Thermodyn.* **2009**, *41*, 115–122. https://doi.org/10.1016/j.jct.2008.07.010.

(321)	Rajagopal, K.; Andrade, L. L. P. R.; Paredes, M. L. L. High-Pressure Viscosity Measurements for the Binary System Cyclohexane + n-Hexadecane in the Temperature Range of (318.15 to 413.15) K. *J. Chem. Eng. Data* **2009**, *54*, 2967–2970. https://doi.org/10.1021/je9000262.

(322)	Prak, D. J. L.; Morris, R. E.; Cowart, J. S.; Hamilton, L. J.; Trulove, P. C. Density, Viscosity, Speed of Sound, Bulk Modulus, Surface Tension, and Flash Point of Direct Sugar to Hydrocarbon Diesel (DSH-76) and Binary Mixtures of N-Hexadecane and 2,2,4,6,6-Pentamethylheptane. *J. Chem. Eng. Data* **2013**, *58*, 3536–3544. https://doi.org/10.1021/je400839x.

(323)	Baled, H. O.; Xing, D.; Katz, H.; Tapriyal, D.; Gamwo, I. K.; Soong, Y.; Bamgbade, B. A.; Wu, Y.; McHugh, M. A.; Enick, R. M. Viscosity of N-Hexadecane, n-Octadecane and n-Eicosane at Pressures up to 243 MPa and Temperatures up to 534 K. *J. Chem. Thermodyn.* **2014**, *72*, 108–116. https://doi.org/10.1016/j.jct.2014.01.008.

(324)	Vorlander, D.; Walter, R. *Z. Phys. Chem. (Leipzig)* **1925**, *118*, 1.

(325)	Godchot, M.; Cauquil, G. On the Viscosities, Surface Tensions and Parachors of Some Cyclic Hydrocarbons (in French). *C. R. Hebd. Seances Acad. Sci.* **1931**, *192*, 1560.

(326)	Hugel, G. Relation Between the Viscosity and Constitution of Hydrocarbons of High Molecular Weight. *Chim. Ind. (Paris)* **1931**, *26*, 1282–1288.

(327)	Friend, J. N.; Hargreaves, W. D. VII. Viscosities at the Boiling Point of Some Primary Amines, Cyclohexane and Some of Its Derivatives. *Philos. Mag. Ser. 7* **1944**, *35*, 57–64.

(328)	Jonas, J.; Hasha, D.; Huang, S. G. Self-Diffusion and Viscosity of Methylcyclohexane in the Dense Liquid Region. *J. Chem. Phys.* **1979**, *71*, 3996.

(329)	Et-Tahir, A.; Boned, C.; Lagourette, B.; Xans, P. Determination of the Viscosity of Various Hydrocarbons and Mixtures of Hydrocarbons versus Temperature and Pressure. *Int. J. Thermophys.* **1995**, *16*, 1309–1334. https://doi.org/10.1007/bf02083543.

(330)	Oswal, S. L.; Oswal, P.; Phalak, R. P. Viscosity of Nonelectrolyte Liquid Mixtures. IV. Binary Mixtures Containing p-Dioxane. *Int. J. Thermophys.* **1996**, *17*, 1255–1267. https://doi.org/10.1007/bf01438668.

(331)	Baylaucq, A.; Dauge, P.; Boned, C. Viscosity and Density of the Ternary Mixture Heptane + Methylcyclohexane + 1 -Methylnaphthalene. *Int. J. Thermophys.* **1997**, *18*, 1089–1107. https://doi.org/10.1007/bf02575251.

(332)	Zeberg-Mikkelsen, C. K.; Barrouhou, M.; Boned, C. Viscosity and Density Measurements of Binary Mixtures Composed of Methylcyclohexane + Cis-Decalin Versus Temperature and Pressure. *Int. J. Thermophys.* **2003**, *24*, 361–374. https://doi.org/10.1023/a:1022911703225.

(333)	Pereiro, A. B.; Rodriguez, A.; Canosa, J.; Tojo, J. Density, Viscosity, and Speed of Sound of Dialkyl Carbonates with Cyclopentane and Methyl Cyclohexane at Several Temperatures. *J. Chem. Eng. Data* **2004**, *49*, 1392–1399. https://doi.org/10.1021/je049895b.

(334)	Oswal, S. L.; Prajapati, K. D.; Oswal, P.; Ghael, N. Y.; Ijardar, S. P. Viscosity of Binary Mixtures of 1-Alkanol+cyclohexane, 2-Alkanol+cyclohexane and 1-Alkanol+methylcyclohexane at 303.15 K. *J. Mol. Liq.* **2005**, *116*, 73–82.

(335)	Ye, J.-D.; Tu, C.-H. Densities and Viscosities for Binary and Ternary Mixtures of Diisopropyl Ether, Ethanol, and Methylcyclohexane. *J. Chem. Eng. Data* **2005**, *50*, 1060–1067. https://doi.org/10.1021/je050031f.

(336)	Aralaguppi, M. I.; Baragi, J. G. Physico-Chemical and Excess Properties of the Binary Mixtures of Methylcyclohexane + Ethanol, + Propan-1-Ol, + Propan-2-Ol, + Butan-1-Ol, + 2-Methyl-1-Propanol, or 3-Methyl-1-Butanol at T = (298.15, 303.15, and 308.15) K. *J. Chem. Thermodyn.* **2006**, *38*, 434–442. https://doi.org/10.1016/j.jct.2005.06.011.

(337)	Baragi, J. G.; Aralaguppi, M. I. Excess and Deviation Properties for the Binary Mixtures of Methylcyclohexane with Benzene, Toluene, p-Xylene, Mesitylene, and Anisole at T = (298.15, 303.15, and 308.15) K. *J. Chem. Thermodyn.* **2006**, *38*, 1717–1724. https://doi.org/10.1016/j.jct.2005.12.005.

(338)	Davies, M. K.; Archer, A. L.; Maczek, A. O. S.; Manzanares-Papayanopoulos, E.; McLure, I. A. Shear Viscosities of Methycyclohexane, Perfluoromethylcyclohexane, and Their Mixtures in the Vicinity of the Upper Critical Mixing Temperature. 1. Critical Isopleth and Coexistence Curve. *J. Chem. Eng. Data* **2006**, *51*, 1502–1508. https://doi.org/10.1021/je0502593.

(339)	Iloukhani, H.; Rezaei-Sameti, M.; Basiri-Parsa, J.; Azizian, S. Studies of Dynamic Viscosity and Gibbs Energy of Activation of Binary Mixtures of Methylcyclohexane with N-Alkanes (C5 C10) at Various Temperatures. *J. Mol. Liq.* **2006**, *126*, 117–123.

(340)	Iloukhani, H.; Samiey, B. Excess Molar Volumes, Viscosities, and Speeds of Sound of the Ternary Mixture (1-Heptanol (1) + Trichloroethylene (2) + Methylcyclohexane (3)) at T = 298.15 K. *J. Chem. Thermodyn.* **2007**, *39*, 206–217. https://doi.org/10.1016/j.jct.2006.07.015.

(341)	Yang, X.; Xing, Y.; Li, D.; Guo, Y.; Fang, W. Volumetric and Viscous Properties at Several Temperatures for Binary Mixtures of N-Methylpiperazine with Methylcyclohexane or n-Heptane. *J. Chem. Eng. Data* **2010**, *55*, 2914–2916. https://doi.org/10.1021/je900969u.

(342)	Ranjbar, S.; Momenian, S. H. Densities and Viscosities of Binary and Ternary Mixtures of (Nitrobenzene + 1-Bromobutane), (1-Bromobutane + Methylcyclohexane), (Nitrobenzene + Methylcyclohexane), and (Methylcyclohexane + Nitrobenzene + 1-Bromobutane) from (293.15 to 308.15) K. *J. Chem. Eng. Data* **2011**, *56*, 3949–3954. https://doi.org/10.1021/je200350a.

(343)	Xing, Y.; Yang, X.; Fang, W.; Guo, Y.; Lin, R. Measurement on Vapor Pressure, Density and Viscosity for Binary Mixtures of JP-10 and Methylcyclohexane. *Fluid Phase Equilib.* **2011**, *305*, 192–196. https://doi.org/10.1016/j.fluid.2011.03.030.

(344)	Li, G.; Chi, H.; Fang, W.; Hu, S. Excess Molar Volume along with Viscosity and Refractive Index for Binary Systems of Tricyclo[5.2.1.0(2.6)]Decane with Five Cycloalkanes. *J. Chem. Eng. Data* **2013**, *58*, 3078–3086. https://doi.org/10.1021/je400529k.

(345)	Jiang, X.; He, G.; Wu, X.; Guo, Y.; Fang, W.; Xu, L. Density, Viscosity, Refractive Index, and Freezing Point for Binary Mixtures of 1,1’-Bicyclohexyl with Alkylcyclohexane. *J. Chem. Eng. Data* **2014**, *59*, 2499–2504. https://doi.org/10.1021/je500275j.

(346)	Keramidi, A. S.; Rastorguev, Yu. L.; Grigor’ev, B. A. Experimental Investigation of the Dynamic Viscosity Coefficient of N-Hydrocarbons. *Teplofiz. Svoist. Zhidk.* **1976**, 90–96.

(347)	Queimada, A. J.; Marrucho, I. M.; Coutinho, J. A. P.; Stenby, E. H. Viscosity and Liquid Density of Asymmetric N-Alkane Mixtures: Measurement and Modeling. *Int. J. Thermophys.* **2005**, *26*, 47–61. https://doi.org/10.1007/s10765-005-2352-4.

(348)	Trautz, M.; Husseini, I. Die Reibung, Warmeleitung Und Diffusion Im Gasmischungen. *Ann. Phys. (Berlin)* **1934**, *20*, 121.

(349)	Sargent, J. W.; Seffl, R. J. Properties of Perfluorinated Liquids. *Fed. Proc.* **1970**, *29*, 1699–1703.

(350)	Morgado, P. J. R. Semifluorinated Alkanes - Structure - Properties Relations. *Thesis* **2011**, 1–196.

(351)	Cochran, M. A.; North, A. M.; Pethrick, R. A. Ultrasonic Studies of Perfluoro-n-Alkanes. *J. Chem. Soc., Faraday Trans.* **1974**, *70*, 1274.

(352)	Freire, M. G.; Ferreira, A. G. M.; Fonseca, I. M. A.; Marrucho, I. M.; Coutinho, J. A. P. Viscosities of Liquid Fluorocompounds. *J. Chem. Eng. Data* **2008**, *53*, 538–542. https://doi.org/10.1021/je700632z.

(353)	Morgado, P.; Black, J.; Lewis, J. B.; Iacovella, C. R.; McCabe, C.; Martins, L. F. G.; Filipe, E. J. M. Viscosity of Liquid Systems Involving Hydrogenated and Fluorinated Substances: Liquid Mixtures of (Hexane + Perfluorohexane). *Fluid Phase Equilib.* **2013**, *358*, 161–165. https://doi.org/10.1016/j.fluid.2013.07.060.

(354)	Duan, Y.-Y.; Shi, L.; Zhu, M.-S.; Han, L.-Z. Critical Parameters and Saturated Density of Trifluoroiodomethane (CF3I). *J. Chem. Eng. Data* **1999**, *44*, 501–504. https://doi.org/10.1021/je980251b.

(355)	Trautz, M.; Winterkorn, H. The Viscosity, Thermal Conductivity and Diffusion in Gases - XVIII. Measurements of Viscosity on Aggressive Gases (Cl2, HI). *Ann. Phys. (Leipzig)* **1931**, *10*, 511–528. https://doi.org/10.1002/andp.19314020406.

(356)	Braune, H.; Linke, R. The Internal Friction Some Gases and Vapors. III. Influence of the Dipole Moment on the Size of the Sutherland Constant. *Z. Phys. Chem. Abt. A* **1930**, *148*, 195–215.

(357)	Steacie, E. W. R.; Johnson, F. M. G. The Viscosities of the Liquid Halogens. *J. Am. Chem. Soc.* **1925**, *47*, 754–762. https://doi.org/10.1021/ja01680a022.

(358)	van Aubel, E. Sur La Viscosite Du Chlore Liquide. *Bull. Cl. Sci. Acad. R. Belg.* **1926**, *12*, 369–373.

(359)	Trautz, M.; Ruf, F. Viscosity, Thermal Conductivity and Diffusion in Gas Mixtures. XXVII. Internal Viscosity of Chlorine and of Hydrogen Iodide. A Test of Methods of Viscosity Measurements on Corrosive Gases. *Ann. Phys. (Berlin)* **1934**, *20*, 127.

(360)	Heston, W. M.; Hennelly, E. J.; Smyth, C. P. Dielectric Constants, Viscosities, Densities, Refractive Indices and Dipole Moment Calculations for Some Organic Halides. *J. Am. Chem. Soc.* **1950**, *72*, 2071–2075. https://doi.org/10.1021/ja01161a054.

(361)	Mumford, S. A.; Phillips, J. W. C. 19. The Physical Properties of Some Aliphatic Compounds. *J. Chem. Soc.* **1950**, 75–84.

(362)	Fort, R. J.; Moore, W. R. Viscosities of Binary Mixtures of Benzyl Acetate with Dioxane. *Trans. Faraday Soc.* **1966**, *62*, 1112.

(363)	Nigam, R. K.; Singh, P. P. Viscosity and Molecular Interactions in Binary Mixtures of Benzene and Toluene with Chloro- and Bromobenzenes. *Indian J. Chem.* **1971**, *9*, 691–695.

(364)	Abdullaev, F. G.; Djafarova, N. I. Experimental Study of the Dynamic Viscosity Coefficient of Chlorbenzene. *Izv. Vyssh. Uchebn. Zaved., Neft Gaz* **1980**, *23*, 89–90.

(365)	Singh, R. P.; Sinha, C. P. Dielectric Behavior of the Binary Mixtures of N-Hexane with Toluene, Chlorobenzene, and 1-Hexanol. *J. Chem. Eng. Data* **1982**, *27*, 283–287. https://doi.org/10.1021/je00029a016.

(366)	Koshechko, V. G.; Krylov, V. A. Thermodynamic Equilibrium of the Ionic Pairs of the Kation -Radical Salts in Acetonitrile and Chlorobenzene. *Zh. Fiz. Khim.* **1984**, *58*, 1334–1339.

(367)	Singh, R. P.; Sinha, C. P. Viscosities and Activation Energies of Viscous Flow of the Binary Mixtures of N-Hexane with Toluene, Chlorobenzene, and 1-Hexanol. *J. Chem. Eng. Data* **1984**, *29*, 132–135. https://doi.org/10.1021/je00036a010.

(368)	Singh, R. P.; Sinha, C. P. Viscosities and Acyivation Energies of Viscous Flow of Ternary Mixtures of Toluene, Chlorobenzene, 1-Hexanol and Benzyl Alcohol. *J. Chem. Eng. Data* **1985**, *30*, 470–474. https://doi.org/10.1021/je00042a031.

(369)	Gokavi, G. S.; Raju, J. R.; Aminabhavi, T. M.; Balundgi, R. H.; Muddapur, M. V. Viscosities and Densities of Binary Liquid Mixtures of Dimethyl Sulfoxide with Chlorobenzene, Pyridine, and Methyl Ethyl Ketone at 25 35, 45, and 55 Deg C. *J. Chem. Eng. Data* **1986**, *31*, 15–18. https://doi.org/10.1021/je00043a004.

(370)	Singh, R. P.; Sinha, C. P.; Singh, B. N. Viscosities, Densities, and Activation Energies of Viscous Flow of the Ternary Systems n-Hexane-Benzyle Alcohol-TOluene, n-Hexane-Benzyl Alcohol-Chlorobenzene, and n-Hexane-Benzyl Alcohol-1-Hexanol a. *J. Chem. Eng. Data* **1986**, *31*, 107–111. https://doi.org/10.1021/je00043a031.

(371)	Ramadevi, R. S.; Venkatesu, P.; Venkata, M.; Rao, P. Viscosities of Binary Liquid Mixtures of N,N-Dimethylformamide with Substituted Benzenes at 303.15 and 313.15 K. *J. Chem. Eng. Data* **1996**, *41*, 479–481. https://doi.org/10.1021/je950187n.

(372)	Artigas, H.; Cea, P.; Mainar, A. M.; Pardo, J.; Royo, F. M. EXCESS VOLUMES AND EXCESS VISCOSITIES OF MIXTURES HALOHYDROCARBON + 2-BUTANOL AT 298.15 AND 313.15 K. *Phys. Chem. Liq.* **1997**, *35*, 105–119.

(373)	Artigas, H.; Dominguez, M.; Mainar, A. M.; Lopez, M. C.; Royo, F. M. Densities and Viscosities of Binary Mixtures of Some Halohydrocarbons with 2-Methyl-1-Propanol at 298.15 and 313.15 K. *J. Chem. Eng. Data* **1998**, *43*, 580–584. https://doi.org/10.1021/je970268e.

(374)	Nayak, J. N.; Aralaguppi, M. I.; Aminabhavi, T. M. Density, Viscosity, Refractive Index, and Speed of Sound in the Binary Mixtures of Ethyl Chloroacetate + Cyclohexanone, + Chlorobenzene, + Bromobenzene, or + Benzyl Alcohol at (298.15, 303.15, and 308.15) K. *J. Chem. Eng. Data* **2003**, *48*, 628–631. https://doi.org/10.1021/je0201828.

(375)	Li, N.; Ma, P.; Jin, H.; Yang, C. Densities and Viscosities for Correlative System of Dimethyl Carbonate at Different Temperatures. *Gaoxiao Huaxue Gongcheng Xuebao* **2004**, *18*, 7;766.

(376)	Oswal, S. L.; Desai, J. S.; Ijardar, S. P.; Malek, N. I. Studies of Viscosities of Dilute Solutions of Alkylamine in Non-Electrolyte Solvents. II. Haloalkanes and Other Polar Solvents. *Thermochim. Acta* **2005**, *427*, 51–60. https://doi.org/10.1016/j.tca.2004.08.013.

(377)	Bhuiyan, M. M. H.; Ferdaush, J.; Uddin, M. H. Densities and Viscosities of Binary Mixtures of (Dimethylsulfoxide + Aliphatic Lower Alkanols (C1 C3)) at Temperatures from T = 303.15 K to T = 323.15 K. *J. Chem. Thermodyn.* **2007**, *39*, 675–683. https://doi.org/10.1016/j.jct.2006.10.016.

(378)	Ali, A.; Nabi, F.; Itoo, F. A.; Tasneem, S. Volumetric, Ultrasonic, Viscometric and Refractive Index Behaviour of Binary Mixtures of Styrene with Ethylalcohol and Chlorobenzene at Different Temperatures (T=298.15 K to 313.15 K). *J. Mol. Liq.* **2008**, *143*, 141–146.

(379)	Baskaran, R.; Kubendran, T. R. Thermo Physical Properties of 4-Hydroxy 4-Methyl Pentanone with Nitrobenzene or Ethyl Benzene at Temperatures of (303.15, 313.15, and 323.15) K and a Pressure of 0.1 MPa. *J. Chem. Eng. Data* **2008**, *53*, 1956–1961. https://doi.org/10.1021/je8002699.

(380)	Sastry, N. V.; Thakor, R. R.; Patel, M. C. Thermophysical Properties For Diethylene Glycol + Nitrobenzene and Triethylene Glycol + (Chloro-, Bromo-, Nitro-) Benzene Systems at Different Temperatures. *Int. J. Thermophys.* **2008**, *29*, 610–618. https://doi.org/10.1007/s10765-008-0399-8.

(381)	Shukla, D.; Singh, S.; Parveen, S.; Gupta, M.; Shukla, J. P. Thermophysical Properties of Binary Mixtures of Methanol with Chlorobenzene and Bromobenzene from 293K to 313K. *Int. J. Thermophys.* **2008**, *29*, 1376–1384. https://doi.org/10.1007/s10765-008-0444-7.

(382)	Ni, B.; Su, L.; Wang, H.; Qiu, H. Thermophysical Properties of the Binary Mixtures of 1,2-Dichloroethane with Chlorobenzene and Bromobenzene from 298.15 to 313.15 K. *J. Chem. Eng. Data* **2010**, *55*, 4541–4545. https://doi.org/10.1021/je100552a.

(383)	Hamzehlouia, S.; Asfour, A. A. Densities and Viscosities of Ten Binary and Ten Ternary Regular Solution Systems at 308.15 and 313.15 K. *J. Mol. Liq.* **2012**, *174*, 143–152. https://doi.org/10.1016/j.molliq.2012.06.020.

(384)	Kumar, D. B. K.; Reddy, K. R.; Rao, G. S.; Rao, G. V. R.; Rambabu, C. Thermodynamic and Spectroscopic Study of Molecular Interactions in the Binary Liquid Mixtures of N-Methyl-2-Pyrrolidone and Some Substituted Benzenes at Different Temperatures. *J. Mol. Liq.* **2012**, *174*, 100–111. https://doi.org/10.1016/j.molliq.2012.07.019.

(385)	Rathnam, M. V.; Bhanushali, K. R.; Sayed, R. T.; Kumar, M. S. S. Acoustic, Thermodynamic and Transport Properties of Binary Liquid Mixtures of Isopentyl Acetate with Aryl Halides at 303.15 and 313.15 K. *J. Mol. Liq.* **2012**, *173*, 35–41. https://doi.org/10.1016/j.molliq.2012.05.022.

(386)	Lavanya, T. G.; Saravanakumar, K.; Baskaran, R.; Kubendran, T. R. Density, Viscosity, Sound Speed, and Thermoacoustical Parameters of Benzaldehyde with Chlorobenzene or Nitrobenzene at 303.15 K, 308.15 K, and 313.15 K. *Int. J. Thermophys.* **2013**, *34*, 1280–1287. https://doi.org/10.1007/s10765-013-1492-1.

(387)	Panchenkov, G. M.; Maksareva, T. S.; Erchenkov, V. V. ? *Zh. Fiz. Khim.* **1958**, *32*, 2787–2791.

(388)	Akhundov, T. S.; Abdullaev, F. G.; Ishkhanov, Yu. B. Experimental Study of the Viscosity of Chlorobenzene in the Gaseous Phase and Critical Region. *Izv. Vyssh. Uchebn. Zaved., Neft Gaz* **1984**, *86*, 93.

(389)	Vogel, E. Towards Reference Viscosities of Carbon Monoxide and Nitrogen at Low Density Using Measurements between 290K and 680K as Well as Theoretically Calculated Viscosities. *Int. J. Thermophys.* **2012**, *33*, 741–757. https://doi.org/10.1007/s10765-012-1185-1.

(390)	Rabe, D. Experimental Determination of Dynamic Viscosity and Density of Binary Liquid Mixtures of N-Heptane with CO2, CH4, C2H6, C3H8 and of Toluene with CH4 and C3H8. PhD Thesis, Technical University Berlin, 1981.

(391)	Diller, D. E.; Ball, M. J. Shear Viscosity Coefficients of Compressed Gaseous and Liquid Carbon Dioxide at Temperatures between 220 and 320 K and at Pressures to 30 MPa. *Int. J. Thermophys.* **1985**, *6*, 619. https://doi.org/10.1007/bf00500334.

(392)	Gururaja, G. J.; Tirunarayanan, M. A.; Ramachandran, A. Dynamic Viscosity of Gas Mixtures. *J. Chem. Eng. Data* **1967**, *12*, 562–567. https://doi.org/10.1021/je60035a024.

(393)	Kestin, J.; Khalifa, H. E.; Wakeham, W. A. Viscosity of Multicomponent Mixtures of Four Complex Gases. *J. Chem. Phys.* **1976**, *65*, 5186–5188.

(394)	Harris, E. J. R.; Hope, C. G.; Gough, D. W.; Smith, E. B. Viscosity of Gaseous Carbon Dioxide, Sulfur Hexafluoride, and Nitrous Oxide at Low Temperatures. *J. Chem. Soc., Faraday Trans. 1* **1979**, *75*, 892–897.

(395)	Lusternik, V. E.; Kuznetsov, V. I. Measurements of the Viscosity of CO2 up to 1870 Kat 1 Atm. *Teplofiz. Vys. Temp.* **1986**, *24*, 169–170.

(396)	Hunter, I. N.; Marsh, G.; Matthews, G. P.; Smith, E. B. Argon + Carbon Dioxide Gaseous Mixture Viscosities and Anisotropic Pair Potential Energy Functions. *Int. J. Thermophys.* **1993**, *14*, 819–833. https://doi.org/10.1007/bf00502110.

(397)	Ulybin, S. A.; Makarushkin, V. I. Viscosity of CO2 at 220-1300 k up to 300 MPa. *Teploenergetika* **1976**, *23*, 65.

(398)	Padua, A.; Wakeham, W. A.; Wilhelm, J. The Viscosity of Liquid Carbon Dioxide. *Int. J. Thermophys.* **1994**, *15*, 767–777. https://doi.org/10.1007/bf01447093.

(399)	Maltsev, V. A.; Nerushev, O. A.; Novopashin, S. A.; Radchenko, V. V.; Licht, W.; Miller, E. J.; Parekh, V. S. Viscosity of H2-CO2 Mixtures at (500, 800, and 1100) K. *J. Chem. Eng. Data* **2004**, *49*, 684–687. https://doi.org/10.1021/je0342419.

(400)	Isdale, J. D.; Dymond, J. H.; Brawn, T. A. Viscosity and Density of N-Hexane-Cyclohexane Mixtures between 25 and 100 C up to 500 MPa. *High Temp. - High Pressures* **1979**, *11*, 571–580.

(401)	Lavrent’ev, I. P.; Yakovlev, V. F. The Velocity of Ultrasound in Cyclohexane and Its Shear Viscosity along the Saturation Line. *Zh. Fiz. Khim.* **1985**, *59*, 2893.

(402)	Drapier, P. Research on the Viscosity of Binary Liquid Mixtures in the Vicinity of the Critical Temperature. *Bull. Cl. Sci. Acad. R. Belg.* **1911**, *1*, 621–640.

(403)	Vorlander, D.; Fischer, J. *Chem. Ber.* **1932**, *65*, 1756.

(404)	Pownall, H. J.; Smith, L. C. Viscosity of the Hydrocarbon Region of Micelles. Measurement of Excimer Fluorescence. *J. Am. Chem. Soc.* **1973**, *95*, 3136–3140. https://doi.org/10.1021/ja00791a009.

(405)	Jonas, J.; Hasha, D.; Huang, S. G. Density Effects of Transport Properties in Liquid Cyclohexane. *J. Phys. Chem.* **1980**, *84*, 109.

(406)	Mishra, R. Interaction Studies in Binary Liquid Mixtures from Viscosity Measurements. *Acta Chim. Acad. Sci. Hung.* **1981**, *108*, 103–110.

(407)	Durov, V. A.; Artykov, A. Acoustic Spectroscope of Liquid Cyclohexane. *Zh. Fiz. Khim.* **1985**, *59*, 2550–2554.

(408)	Manjeshwar, L. S.; Aminabhavi, T. M. Densities and Viscosities of Binary Liquid Mixtures Containing Bromoform at 45 Deg C. *J. Chem. Eng. Data* **1988**, *33*, 184–185. https://doi.org/10.1021/je00052a036.

(409)	Cueto, M. J. M.; Vallejo, M. G.; Luque, V. F. Physiochemical Studies Concerning Miscibility of Vegetable Oils. XIV. Molar Volumes, Molar Refractions, and Viscosities of Solutions of Methyl Stearate in Cyclohexane, Hexane, Tetrachloroethylene, and Trichloroethylene. *Grasas Aceites* **1991**, *42*, 14–21.

(410)	Papanastasiou, G. E.; Ziogas, I. I. Physical Behavior of Some Reaction Media. Density, Viscosity, Dielectric Constant, and Refractive Index Changes of Ethanol-Cyclohexane Mixtures at Several Temperatures. *J. Chem. Eng. Data* **1991**, *36*, 46–51. https://doi.org/10.1021/je00001a014.

(411)	Nath, J.; Chaudhari, S. K. Excess Volumes, Dielectric Constants, Refractive Indexes, and Viscosities for Anisole + Methylene Chloride, 1,2-Dichloroethane, Trichloroethene, Tetrachloroethene, and Cyclohexane. *J. Chem. Eng. Data* **1992**, *37*, 387–390. https://doi.org/10.1021/je00008a003.

(412)	Blasco, D.; Lafuente, C.; Santafe, J.; Royo, F. M.; Urieta, J. S. Excess Volumes and Excess Viscosities of Binary Mixtures of Cyclohexane + Picoline. *Thermochim. Acta* **1993**, *230*, 55–63. https://doi.org/10.1016/0040-6031(93)80346-c.

(413)	Munoz, M. J.; Rodriguez, M.; Martin, R.; Galan, M. A Study of the Viscosity of Methyl Myristate Solutions in Organic Solvents. *An. Quim.* **1994**, *90*, 177–180.

(414)	Pandey, J. D.; Jain, P.; Vyas, V. Speed of Sound, Viscosity, and Refractive Index of Multicomponent Systems: Analysis of Experimental Data from the Bertrand-Acree- Burchfield Equation. *Can. J. Chem.* **1994**, *72*, 2486–2492. https://doi.org/10.1139/v94-315.

(415)	Aminabhavi, T. M.; Gopalakrishna, B. Density, Viscosity, Refractive Index, and Speed of Sound in Binary Mixtures of 2-Ethoxyethanol with n-Alkanes (C6 to C12), 2,2,4- Trimethylpentane, and Cyclohexane in the Temperature Interval 298.15-. *J. Chem. Eng. Data* **1995**, *40*, 632–641. https://doi.org/10.1021/je00019a022.

(416)	Artigas, H.; Sanz, M. L.; Mainar, A. M.; Royo, F. M.; Urieta, J. S. EXCESS VOLUMES AND EXCESS VISCOSITIES OF BINARY MIXTURES OF CYCLOHEXANE + AN ISOMER OF BUTANOL AT SEVERAL TEMPERATURES. *Phys. Chem. Liq.* **1995**, *30*, 17–28.

(417)	Aminabhavi, T. M.; Patil, V. B.; Aralaguppi, M. I.; Phayde, H. T. S. Density, Viscosity, and Refractive Index of the Binary Mixtures of Cyclohexane with Hexane, Heptane, Octane, Nonane, and Decane at (298.15, 30 3.15, and 308.15) K. *J. Chem. Eng. Data* **1996**, *41*, 521–525. https://doi.org/10.1021/je950279c.

(418)	Padua, A. A. H.; Fareleira, J. M. N. A.; Calado, J. C. G.; Wakeham, W. A. Validation of an Accurate Vibrating-Wire Densimeter: Density and Viscosity of Liquids over Wide Ranges of Temperature and Pressure. *Int. J. Thermophys.* **1996**, *17*, 781–802. https://doi.org/10.1007/bf01439190.

(419)	Aminabhavi, T. M.; Banerjee, K. Density, Viscosity, Refractive Index, and Speed of Sound in Binary Mixtures of Methyl Acetate + Ethylene Glycol or + Poly(Ethylene Glycol) in the Temperature Interval (298.15-308.15) K. *J. Chem. Eng. Data* **1998**, *43*, 852–855. https://doi.org/10.1021/je9800757.

(420)	Gascon, I.; Lafuente, C.; Cea, P.; Royo, F. M.; Urieta, J. S. Viscosities of the Ternary Mixture (Cyclohexane + Tetrahydrofuran + Chiorocyclohexane) at 298.15 and 313.15 K. *Fluid Phase Equilib.* **1999**, *164*, 143–155. https://doi.org/10.1016/s0378-3812(99)00257-5.

(421)	Gascon, I.; Lopez, M. C.; Dominguez, M. M.; Royo, F. M.; Urieta, J. S. Viscosities and Viscosity Predictions of the Ternary Mixture Cyclohexane + 1-3-Dioxolane + 1-Butanol at 298.15 and 313.15 K. *J. Chem. Eng. Jpn.* **2000**, *33*, 740–746.

(422)	Gascon, I.; Mainar, A. M.; Royo, F. M.; Urieta, J. S.; Alvarez-Cerdeirina, L. Experimental Viscosities and Viscosity Predictions of the Ternary Mixture (Cyclohexane + 1,3-Dioxolane + 2-Butanol) at 298.15 and 313.15 K. *J. Chem. Eng. Data* **2000**, *45*, 751–755. https://doi.org/10.1021/je000081e.

(423)	Gomez-Diaz, D.; Mejuto, J. C.; Navaza, J. M. Physicochemical Properties of Liquid Mixtures. 1. Viscosity, Density, Surface Tension and Refractive Index of Cyclohexane + 2,2,4-Trimethylpentane Binary Liquid Systems from 25 C to 50 C. *J. Chem. Eng. Data* **2001**, *46*, 720–724. https://doi.org/10.1021/je000310x.

(424)	Gomez-Diaz, D.; Mejuto, J. C.; Navaza, J. M.; Rodriguez-Alvarez, A. Viscosities, Densities, Surface Tensions, and Refractive Indexes of 2,2,4-Trimethylpentane + Cyclohexane + Decane Ternary Liquid Systems at 298.15 K. *J. Chem. Eng. Data* **2002**, *47*, 872–875. https://doi.org/10.1021/je010288n.

(425)	Ali, A.; Abida; Hyder, S. STUDY OF INTERMOLECULAR INTERACTIONS IN BINARY MIXTURES OF CYCLOHEXANE WITH 1-ALKANOLS AT 308K. *Phys. Chem. Liq.* **2004**, *42*, 411–422.

(426)	Haro, M.; Rodriguez, V.; Cea, P.; Lopez, M. C.; Lafuente, C. Viscosimetric Study of Multicomponent Liquid Mixtures Containing Oxygenated Compounds. *Int. J. Thermophys.* **2004**, *25*, 669–678. https://doi.org/10.1023/b:ijot.0000034231.98922.7e.

(427)	Oswal, S. L.; Ghael, N. Y.; Prajapati, K. D. Speeds of Sound, Isentropic Compressibilities, Viscosities and Excessmolar Volumes of Cyclohexanol + Cyclohexane at 303.15 K. *Thermochim. Acta* **2004**, *419*, 59–65. https://doi.org/10.1016/j.tca.2004.01.034.

(428)	Iloukhani, H.; Rezaei-Sameti, M.; Zarei, H. A. Volumetric and Viscometric Studies of Molecular Interaction of the Ternary System Toluene (1) + Cyclohexane (2) + n-Hexane (3) at 298.15K. *Thermochim. Acta* **2005**, *438*, 9–15. https://doi.org/10.1016/j.tca.2005.08.007.

(429)	Singh, S.; Sethi, B. P. S.; Katyal, R. C.; Rattan, V. K. Viscosities, Densities, and Speeds of Sound of Binary Mixtures of o-Xylene, m-Xylene, p-Xylene, and Isopropylbenzene with 2-Butanone at 298.15 K. *J. Chem. Eng. Data* **2005**, *50*, 125–127. https://doi.org/10.1021/je049793l.

(430)	Gonzalez, E. J.; Gonzales, B.; Calvar, N.; Dominguez, A. Physical Properties of Binary Mixtures of the Ionic Liquid 1-Ethyl-3-Methylimidazolium Ethyl Sulfate with Several Alcohols at T = (298.15, 313.15, and 328.15) K and Atmospheric Pressure. *J. Chem. Eng. Data* **2007**, *52*, 1641–1648. https://doi.org/10.1021/je700029q.

(431)	Fan, W.; Zhou, Q.; Zhang, S.; Yan, R. Excess Molar Volume and Viscosity Deviation for the Methanol + Methyl Methacrylate Binary System at T) (283.15 to 333.15) K. *J. Chem. Eng. Data* **2008**, *53*, 1836–1840. https://doi.org/10.1021/je800164w.

(432)	Gardas, R. L.; Oswal, S. L. Volumetric and Transport Properties of Ternary Mixtures Containing 1-Butanol or 1-Pentanol, Triethylamine and Cyclohexane at 303.15 K: Experimental Data, Correlation and Prediction by the ERAS Model. *J. Solution Chem.* **2008**, *37*, 1449–1470.

(433)	Ghael, N. Y.; Gardas, R. L.; Oswal, S. L. Volumetric and Transport Properties of Ternary Mixtures Containing 1-Alkanol + Ethyl Ethanoate + Cyclohexane at 303.15 K: Experimental Data, Correlation and Prediction by ERAS Model. *Thermochim. Acta* **2009**, *491*, 44–57. https://doi.org/10.1016/j.tca.2009.02.022.

(434)	Hernandez-Galvan, M. A.; Garcia-Sanchez, F.; Garcia-Flores, B. E.; Castro-Arellano, J. Liquid Viscosities of Cyclohexane, Cyclohexane + Tetradecane, and Cyclohexane + Benzene from (313 to 393) K and Pressures Up to 60 MPa. *J. Chem. Eng. Data* **2009**, *54*, 2831–2838. https://doi.org/10.1021/je800882f.

(435)	Roy, M. N.; Sarkar, B. K.; Sinha, B. Densities and Viscosities of Ternary Mixtures of Cyclohexane + Cyclohexanone + Some Alkyl Acetates at 298.15 K. *J. Chem. Eng. Data* **2009**, *54*, 1076–1083. https://doi.org/10.1021/je8007217.

(436)	Silva, A.; Reis, R. A.; Paredes, M. L. L. Density and Viscosity of Decalin, Cyclohexane, and Toluene Binary Mixtures at (283.15, 293.15, 303.15, 313.15, and 323.15) K. *J. Chem. Eng. Data* **2009**, *54*, 2067–2072. https://doi.org/10.1021/je800940j.

(437)	Rathnam, M. V.; Mankumare, S.; Kumar, M. S. S. Density, Viscosity, and Speed of Sound of (Methyl Benzoate + Cyclohexane), (Methyl Benzoate + n-Hexane), (Methyl Benzoate + Heptane), and (Methyl Benzoate + Octane) at Temperatures of (303.15, 308.15, and 313.15) K. *J. Chem. Eng. Data* **2010**, *55*, 1354–1358. https://doi.org/10.1021/je9006597.

(438)	Yadava, S. S.; Yadav, N. Densities, Viscosities and Ultrasound Velocities for Binary Mixtures of Isopropylethanoate and Non-Polar Hydrocarbon Solvents. *J. Mol. Liq.* **2010**, *157*, 6–12. https://doi.org/10.1016/j.molliq.2010.07.010.

(439)	Rathnam, M. V.; Mankumare, S.; Jain, K.; Kumar, M. S. S. Densities, Viscosities and Speeds of Sound of Binary Mixtures of Ethyl Benzoate + Hydrocarbons at (303.15, 308.15 and 313.15) K. *J. Solution Chem.* **2012**, *41*, 475–490. https://doi.org/10.1007/s10953-012-9802-y.

(440)	Sastry, N. V.; Patel, S. R.; Soni, S. S. Excess Molar Volumes, Excess Isentropic Compressibilities, Excess Viscosities, Relative Permittivity and Molar Polarization Deviations for Methyl Acetate+, Ethyl Acetate+, Butyl Acetate+, Isoamyl Acetate+, Methyl Propionate+, Ethyl Propionate+, Ethyl Butyrate+, Methyl Methacrylate+, Ethyl Methacrylate+, and Butyl Methacrylate + Cyclohexane at T = 298.15 and 303.15 K. *J. Mol. Liq.* **2013**, *183*, 102–112. https://doi.org/10.1016/j.molliq.2013.04.015.

(441)	Hammami, N. E.; Bouanz, M.; Toumi, A. Thermodynamic Properties of Cyclohexane Methanol Liquid Mixture from Shear Viscosity Measurements. *Fluid Phase Equilib.* **2014**, *384*, 25–35. https://doi.org/10.1016/j.fluid.2014.10.013.

(442)	Torin-Ollarves, G. A.; Martin, M. C.; Chamorro, C. R.; Segovia, J. J. Densities, Viscosities, and Isobaric Heat Capacities of the System (1-Butanol + Cyclohexane) at High Pressures. *J. Chem. Thermodyn.* **2014**, *74*, 153–160. https://doi.org/10.1016/j.jct.2014.01.020.

(443)	Ma, R.-F.; Shi, L.; Duan, Y.-Y.; Han, L.-Z.; Liu, N.-X. Saturated Liquid Viscosity of Cyclopentane and Isopentane. *J. Chem. Eng. Data* **2003**, *48*, 1418–1421. https://doi.org/10.1021/je0202174.

(444)	Timmermans, J.; Hennaut-Roland, M. Works from International Bureau at Physical-Chemical Standards. VIII. Physical Constants of 20 Organic Compounds. *J. Chim. Phys. Phys.-Chim. Biol.* **1937**, *34*, 693.

(445)	Assael, M. J.; Dalouti, N. K.; Dymond, J. H. The Viscosity of Toluene in the Temperature Range 210 to 370 K. *Int. J. Thermophys.* **2000**, *21*, 291. https://doi.org/10.1023/a:1006628008963.

(446)	Harris, K. R.; Newitt, P. J.; Woolf, L. A. Temperature and Density Dependence of the Viscosity of Cyclopentane. *J. Chem. Eng. Data* **2004**, *49*, 138–142. https://doi.org/10.1021/je034142v.

(447)	Gonzalez, B.; Dominguez, D.; Tojo, J. Physical Properties of the Binary Systems Methylcyclopentane with Ketones (Acetone, Butanone and 2-Pentanone) at T = (293.15, 298.15, and 303.15) K. New UNIFAC-VISCO Interaction Parameters. *J. Chem. Thermodyn.* **2006**, *38*, 707–716. https://doi.org/10.1016/j.jct.2005.08.002.

(448)	Kurihara, K.; Kandil, M. E.; Marsh, K. N.; Goodwin, A. R. H. Measurement of the Viscosity of Liquid Cyclopentane Obtained with a Vibrating Wire Viscometer at Temperatures between (273 and 353) K and Pressures below 45 MPa. *J. Chem. Eng. Data* **2007**, *52*, 803–807. https://doi.org/10.1021/je060416d.

(449)	Kumar, H.; Singla, M.; khosla, A.; Gaba, R. Viscometric Studies of Binary Liquid Mixtures of Cyclopentane (1) +2-Propanol (2), +1-Butanol (2), and +2-Butanol (2) at T=(298.15 and 308.15) K. *J. Mol. Liq.* **2011**, *158*, 182–186. https://doi.org/10.1016/j.molliq.2010.12.002.

(450)	Kumar, H.; Gulati, N.; Kundi, V. Viscometric Studies of Binary Liquid Mixtures of Cyclopentane (1)+branched Alkanols (2) at T=(298.15 and 308.15) K. *J. Mol. Liq.* **2012**, *174*, 63–69. https://doi.org/10.1016/j.molliq.2012.07.020.

(451)	Dunlop, P. J. Viscosities of a Series of Gaseous Fluorocarbons at 25 .Degree.C. *J. Chem. Phys.* **1994**, *100*, 3149–3151. https://doi.org/10.1063/1.466405.

(452)	Lukin, V. I.; Ivakin, B. A.; Suetin, P. E. Temperature Dependence of the Viscosity Coefficients of Some Gases. *Sov. Phys. - Tech. Phys.* **1983**, *28*, 597.

(453)	Van Itterbeek, A.; Paemel, van. Measurements on the Viscosity of Neon, Hydrogen, Deuterium and Helium as a Function of the Temperature, between Room Temperature and Liquid Hydrogen Temperatures. *Physica (Amsterdam)* **1940**, *7*, 265.

(454)	Coremans, J. M. J.; van Itterbeek, A.; Beenakker, J. J. M.; Knaap, H. F. P.; Zandbergen, P. The Viscosity of Gaseous He, Ne, H2 and D2 below 80 K. *Physica Amsterdam* **1958**, *24*, 557–576. https://doi.org/10.1016/S0031-8914(58)80070-1.

(455)	Rudenko, N. S.; Konareva, V. G. Viscosity of Liquid Hydrogen and Deuterium. *Russ. J. Phys. Chem.* **1963**, *37*, 1493–1494.

(456)	Becker, E. N.; Misenta, R. Viscosity of HD and Helium-3 between 14 and 20 K. *Z. Phys.* **1955**, *140*, 535–539. https://doi.org/10.1007/BF01337559.

(457)	Van Itterbeek, A.; Claes, A. Viscosity of Light Hydrogen Gas and Deuterium between 293 Degrees K. and 14 Degrees K. *Nature (London)* **1938**, *142(3600)*, 793–794.

(458)	Michels, A.; Schipper, A. C. J.; Rintoul, W. H. The Viscosity of Hydrogen and Deuterium at Pressures up to 2000 Atmospheres. *Physica (Amsterdam)* **1953**, *19*, 1011–1028.

(459)	Menabde, N. E. Viscosity Coefficient of Hydrogen (H(2), D(2)), Neon (Ne(20), Ne(22)) and Helium (He(3)) Isotopes in the Temperature Range -195 to +25 Degrees C. *Sov. At. Energy (Engl. Transl.)* **1965**, *19*, 1421–1422.

(460)	Van Itterbeek, A.; Van Paemel, O. Determination of Internal Friction of Liquid Hydrogen and Deuterium. *Physica (Amsterdam)* **1941**, *8*, 133–143.

(461)	Rietveld, A., A. O. ,. van Itterbeek; Velds, C. A. Viscosity of Binary Mixtures of Hydrogen Isotopes and Mixtures of He and Ne. *Comm. Phys. Lab. Univ. Leiden* **1959**, 1–12.

(462)	Barua, A. K.; Afzal, M.; Flynn, G. P.; Ross, J. Viscosity of Hydrogen, Deuterium, Methane, and Carbon Monoxide from 50 to 150 C below 200 Atmospheres. *J. Chem. Phys.* **1964**, *41*, 374.

(463)	Lukin, V. I.; Ivakin, B. A.; Suetin, P. E. Viscosity of Some Gases Measured by the Non-Steady Flow Method. *Zh. Tekhn. Fiz.* **1981**, *51*, 860–862.

(464)	Lukin, V. I.; Ivakin, B. A.; Suetin, P. E. Temperature Dependence of Then Viscosity Coefficients of Some Gases. *Zh. Tekhn. Fiz.* **1983**, *53*, 931–932.

(465)	Konareva, V. G.; Rudenko, N. S. Measurements of Viscosity of the Hydrogen Isotops along the Liquid-Gas Coexistence Curve. *Zh. Fiz. Khim.* **1967**, *41*, 2387–2388.

(466)	Rudenko, N. P.; Slyusar, V. P. Density of Liquid Deiterium. *Zh. Fiz. Khim.* **1968**, *42*, 242–243.

(467)	Kestin, J.; Leidenfrost, W. Effect of Pressure on the Viscosity of N2-CO2 Mixtures. *Physica (Amsterdam)* **1959**, *25*, 525–536.

(468)	Rudenko, N. S.; Konareva, V. G. Viscosity of Liquid PH2 and OH2. *Zh. Eksp. Teor. Fiz.* **1965**, *1965*, 769–770.

(469)	De Troyer, A.; Van Itterbeek, A.; Van den Berg, G. J. Measurements on the Thermal Diffusion of Ne - H2, Ne - D2 and Ne - He Mixtures at Liquid Hydrogen Temperatures. *Physica Amsterdam* **1950**, *16*, 669–677. https://doi.org/10.1016/0031-8914(50)90035-8.

(470)	Kestin, J.; Nagashima, A. Viscosity of Neon-Helium and Neon-Argon Mixtures at 20 and 30 C. *J. Chem. Phys.* **1964**, *40*, 3648–3654.

(471)	Maitland, G. C.; Mustapha, M.; Wakeham, W. A. Thermal Conductivity of Polyatomic Gases at Low Density. *J. Chem. Soc. Faraday Trans. I* **1983**, *79*, 163–172. https://doi.org/10.1039/f19837900163.

(472)	Rivkin, S. L.; Levin, A. Ya.; Izrailevskii, L. B.; Kharitonov, K. G. Experimental Study of Viscosity of the Heavy Water in Liquid Phase at Pressures up to 500 Bar. *Teploenergetika* **1974**, 69–71.

(473)	Kellomaki, A. *Finn. Chem. Lett.* **1975**, *2*, 51.

(474)	Selwood, P. W.; Frost, A. A. Some Properties of Heavy Water. *J. Am. Chem. Soc.* **1933**, *55*, 4335–4336. https://doi.org/10.1021/ja01337a508.

(475)	Taylor, H. S.; Selwood, P. W. Some Properties of Heavy Water. *J. Am. Chem. Soc.* **1934**, *56*, 998–999. https://doi.org/10.1021/ja01319a509.

(476)	Timrot, D. L.; Shuiskaya, K. F. Experimental Study of the Viscosity of Heavy Water. *At. Energ.* **1959**, *7*, 459–462.

(477)	Tourky, A. R.; Rizk, H. A.; Girgis, Y. M. The Dielectric Properties of Heavy Water in Dioxane. *Z. Phys. Chem. (Munich)* **1962**, *32*, 102–109.

(478)	Agaev, N. A.; Usibova, A. D. Viscosity of Ordinary and Heavy Water at High Pressures in the Temperature Range from 0 to 150 C. *Dokl. Akad. Nauk Az. SSR* **1968**, *180*, 334–337.

(479)	Rivkin, S. L.; Levin, A. Ya.; Izrailevskii, L. B.; Kharitonov, K. G.; Gromova, I. A. Dependence between the Dynamic Viscosity Coefficient of Heavy and Ordinary Water in the Liquid Phase. *Teploenergetika* **1976**, 79–82.

(480)	Timrot, D. L.; Serednitskaya, M. A.; Traktueva, C. A. Viscosity of Heavy Water Vapor at 100-500.Deg. and 0.08-1.3 Bars. *Teploenergetika* **1974**, 83–84.

(481)	Rivkin, S. L.; Levin, A. Ya.; Izrailevskii, L. B.; Kharitonov, K. G.; Ptitsyna, V. N. Experimental Study of the Dynamic Viscosity Coefficient of Heavy Water at Supercrtical Parameters of State. *Teploenergetika* **1977**, 65–67.

(482)	Dickinson, E. Pressure Dependence of Shear Viscosity in N-Alkane + Dimethylsiloxane Mixtures. *J. Phys. Chem.* **1977**, *81*, 2108–2113.

(483)	Abbas, R.; Ihmels, E. C.; Enders, S.; Gmehling, J. Measurement of Transport Properties for Selected Siloxanes and Their Mixtures Used as Working Fluids for Organic Rankine Cycles. *Ind. Eng. Chem. Res.* **2011**, *50*, 8756–8763. https://doi.org/10.1021/ie2002632.

(484)	Fischer, J. D. Transport Properties of Pure Fluids and Binary Mixtures with Different Interaction Parameters. *Thesis* **1986**, 1–290.

(485)	Markgraf, J. H. 1-Chloro-2,3-Dimethylbutane. *J. Chem. Eng. Data* **1968**, *13*, 447–448. https://doi.org/10.1021/je60038a047.

(486)	Fischer, J.; Weiss, A. Transport Properties of Liquids. VII. Viscosity, Excess Volumes, and Self-Diffusion of Binary Mixtures of Donor-Acceptor and Carbon Tetrachloride-Carboxylic Acid Systems. *Ber. Bunsen-Ges. Phys. Chem.* **1986**, *90*, 1141. https://doi.org/10.1002/bbpc.19860901205.

(487)	Palczewska-Tulinska, M.; Oracz, P. Selected Physicochemical Properties of Hexamethylcyclotrisiloxane, Octamethylcyclotetrasiloxane, and Decamethylcyclopentasiloxane. *J. Chem. Eng. Data* **2005**, *50*, 1711–1719. https://doi.org/10.1021/je050173+.

(488)	Whorton, R.; Amis, E. S. The Equivalent Conductance of Electrolytes in Mixed Solvents: IV Tetraethylammonium Picrate in the Methanol + Ethanol System. *Z. Phys. Chem. (Munich)* **1956**, *8*, 9–19.

(489)	DiGuilio, R. M.; Lee, R. J.; Schaeffer, S. T.; Brasher, L. L.; Teja, A. S. Densities and Viscosities of the Ethanolamines. *J. Chem. Eng. Data* **1992**, *37*, 239–242. https://doi.org/10.1021/je00006a028.

(490)	Teng, T. T.; Maham, Y.; Hepler, L. G.; Mather, A. E. Viscosity of Aqueous Solutions of N-Methyldiethanolamine and of Diethanolamine. *J. Chem. Eng. Data* **1994**, *39*, 290–293. https://doi.org/10.1021/je00014a021.

(491)	Blanco, A.; Garcia-Abuin, A.; Gomez-Diaz, D.; Navaza, J.; Villaverde, O. Density, Speed of Sound, Viscosity, Surface Tension, and Excess Volume of N-Ethyl-2-Pyrrolidone + Ethanolamine (or Diethanolamine or Triethanolamine) from T = (293.15 to 323.15) K. *J. Chem. Eng. Data* **2013**, *58*, 653–659. https://doi.org/10.1021/je301123j.

(492)	Lopez, A. B.; Garcia-Abuin, A.; Gomez-Diaz, D.; La Rubia, M. D.; Navaza, J. M. Density, Speed of Sound, Viscosity, Refractive Index and Surface Tension of N-Methyl-2-Pyrrolidone + Diethanolamine (or Triethanolamine) from T = (293.15 to 323.15) K. *J. Chem. Thermodyn.* **2013**, *61*, 1–6. https://doi.org/10.1016/j.jct.2013.01.020.

(493)	Spasojevic, V. D.; Serbanovic, S. P.; Djordjevic, B. D.; Kijeveanin, M. L. Densities, Viscosities, and Refractive Indices of Aqueous Alkanolamine Solutions as Potential Carbon Dioxide Removal Reagents. *J. Chem. Eng. Data* **2013**, *58*, 84–92. https://doi.org/10.1021/je300938w.

(494)	Haghtalab, A.; Shojaeian, A. Volumetric and Viscometric Behaviour of the Binary Systems of N-Methyldiethanolamine and Diethanolamine with 1-Butyl-3-Methylimidazolium Acetate at Various Temperatures. *J. Chem. Thermodyn.* **2014**, *68*, 128–137. https://doi.org/10.1016/j.jct.2013.09.001.

(495)	Sage, B. H.; Lavender, H. M.; Lacey, W. N. Phase Equilibria in Hydrocarbon Systems: Methane - Decane System. *Ind. Eng. Chem.* **1940**, *32*, 743–747.

(496)	Lee, A. L.; Ellington, R. T. Viscosity of N-Decane in the Liquid Phase. *J. Chem. Eng. Data* **1965**, *10*, 346–348. https://doi.org/10.1021/je60027a013.

(497)	Carmichael, L. T.; Berry, V. M.; Sage, B. H. Viscosity of Hydrocarbons. n-Decane. *J. Chem. Eng. Data* **1969**, *14*, 27–31. https://doi.org/10.1021/je60040a009.

(498)	Moore, J. W.; Wellek, R. M. Diffusion Coefficients of N-Heptane and n-Decane in n-Alkanes and n-Alcohols at Several Temperatures. *J. Chem. Eng. Data* **1974**, *19*, 136–140. https://doi.org/10.1021/je60061a023.

(499)	Atrops, H.; Kalali, H. E.; Kohler, F. Melting Curves, Densities, Viscosities and Thermodymamic Excess Properties of 1,2,4-Trichlorobenzene + n-Decane and + n-Tetradecane. *Ber. Bunsen-Ges. Phys. Chem.* **1982**, *86*, 26–31. https://doi.org/10.1002/bbpc.19820860106.

(500)	Wakefield, D. L.; Marsh, K. N.; Zwolinski, B. J. Viscosities of Nonelectrolyte Liquid Mixtures. II. Binary and Quaternary Systems of Some n-Alkanes. *Int. J. Thermophys.* **1988**, *9*, 47. https://doi.org/10.1007/bf00503999.

(501)	Assael, M. J.; Dymond, J. H.; Papadaki, M.; Patterson, P. M. Correlation and Prediction of Dense Fluid Transport Coefficients. I. n-Alkanes. *Int. J. Thermophys.* **1992**, *13* (2), 269–281.

(502)	Oliveira, C. M. B. P.; Wakeham, W. A. The Viscosity of Five Liquid Hydrocarbons at Pressures up to 250 MPa. *Int. J. Thermophys.* **1992**, *13*, 773–790. https://doi.org/10.1007/bf00503906.

(503)	Papaioannou, D.; Bridakis, M.; Panayiotou, C. G. Excess Dynamic Viscosity and Excess Volume of N-Butylamine + 1-Alkanol Mixtures at Moderately High Pressures. *J. Chem. Eng. Data* **1993**, *38*, 370–378. https://doi.org/10.1021/je00011a010.

(504)	Estrada-Baltazar, A.; Alvarado, J. F. J.; Iglesias-Silva, G. A.; Barrufet, M. A. Experimental Liquid Viscosities of Decane and Octane + Decane from 298.15 K to 373.15 K and Up to 25 MPa. *J. Chem. Eng. Data* **1998**, *43*, 441–446. https://doi.org/10.1021/je970233e.

(505)	Franjo, C.; Segade, L.; Menaut, C. P.; Pico, J. M.; Jimenez, E. Viscosities and Densities of Solutions of N-Decane, or n-Tetradecane with Several Esters at 25 C. *J. Solution Chem.* **2001**, *30*, 995–1005.

(506)	Segade, L.; Casad, H.; Cabeza, O.; Prego, M.; Garcia-Garabal, S.; Franjo, C.; Jimenez Cuesta, E. Viscosities of Di-n-Butyl Ether + 1-Propanol + n-Decane Mixture at 308.15 K. *Fluid Phase Equilib.* **2001**, *182*, 353–364. https://doi.org/10.1016/s0378-3812(01)00393-4.

(507)	Tohidi, B.; Burgass, R. W.; Danesh, A.; Todd, A. C. Viscosity and Density of Methane + Methylcyclohexane from (323 to 423) K and Pressures to 140 MPa. *J. Chem. Eng. Data* **2001**, *46*, 385–390. https://doi.org/10.1021/je000191l.

(508)	Audonnet, F.; Padua, A. A. H. Viscosity and Density of Mixtures of Methane and N-Decane from 298 to 393 K and up to 75 MPa. *Fluid Phase Equilib.* **2004**, *216*, 235–244. https://doi.org/10.1016/j.fluid.2003.10.017.

(509)	Kapoor, S.; Rattan, V. K. Isobaric Vapor-Liquid Equilibria at 97.3 KPa and Excess Properties at (303.15, 308.15, and 313.15) K of Binary Mixture of p-Xylene + Decane. *J. Chem. Eng. Data* **2005**, *50*, 1891–1896. https://doi.org/10.1021/je0501585.

(510)	Dubey, G. P.; Sharma, M. Thermophysical Properties of Binary Mixtures of 2-Methyl-1-Propanol with Hexane, Octane, and Decane at 298.15 K. *J. Chem. Eng. Data* **2007**, *52*, 449–453. https://doi.org/10.1021/je060389r.

(511)	Fang, Z.; Qiao, Y.; Di, Z.; Huo, Y.; Ma, P.; Xia, S. Viscosities of P-Xylene, Acetic Acid, and p-Xylene + Acetic Acid at (313.15 to 473.15) K and (0.10 to 3.20) MPa: Determined by the Rolling-Ball Method. *J. Chem. Eng. Data* **2008**, *53*, 2787–2792. https://doi.org/10.1021/je800635g.

(512)	Yang, F.; Guo, Y.; Xing, Y.; Li, D.; Fang, W.; Lin, R. Densities and Viscosities of Binary Mixtures of JP-10 with n-Octane or n-Decane at Several Temperatures. *J. Chem. Eng. Data* **2008**, *53*, 2237–2240. https://doi.org/10.1021/je800348s.

(513)	Caudwell, D. R.; Trusler, J. P. M.; Vesovic, V.; Wakeham, W. A. Viscosity and Density of Five Hydrocarbon Liquids at Pressures up to 200 MPa and Temperatures up to 473 K. *J. Chem. Eng. Data* **2009**, *54*, 359–366. https://doi.org/10.1021/je800417q.

(514)	Feitosa, F. X.; Caetano, A. C. R.; Cidade, T. B.; de Sant Ana, H. B. Viscosity and Density of Binary Mixtures of Ethyl Alcohol with N-Alkanes (C6, C8, and C10). *J. Chem. Eng. Data* **2009**, *54*, 2957–2963. https://doi.org/10.1021/je800925v.

(515)	Sato, Y.; Yoshioka, H.; Aikawa, S.; Smith Jr., R. L. A Digital Variable-Angle Rolling-Ball Viscometer for Measurement of Viscosity, Density, and Bubble-Point Pressure of CO2 and Organic Liquid Mixtures. *Int. J. Thermophys.* **2010**, *31*, 1896–1903. https://doi.org/10.1007/s10765-008-0542-6.

(516)	Zivkovic, E. M.; Kijevcanin, M. L.; Radovic, I. R.; Serbanovic, S. P.; Djordjevic, B. D. Viscosity of the Binary Systems 2-Methyl-2-Propanol with n-Alkanes at T = (303.15, 308.15, 313.15, 318.15 and 323.15) K: Prediction and Correlation - New UNIFAC-VISCO Interaction Parameters. *Fluid Phase Equilib.* **2010**, *299*, 191–197. https://doi.org/10.1016/j.fluid.2010.10.009.

(517)	Gong, X.; Guo, Y.; Xiao, J.; Yang, Y.; Fang, W. Densities, Viscosities, and Refractive Indices of Binary Mixtures of 1,2,3,4-Tetrahydronaphthalene with Some n-Alkanes at T = (293.15 to 313.15) K. *J. Chem. Eng. Data* **2012**, *57*, 3278–3282. https://doi.org/10.1021/je300899n.

(518)	Fang, S.; Zuo, X.-B.; Xu, X.-J.; Ren, D.-H. Density, Viscosity and Excess Molar Volume of Binary Mixtures of Tri-n-Octylamine + Diluents (n-Heptane, n-Octane, n-Nonane, and n-Decane) at Various Temperatures. *J. Chem. Thermodyn.* **2014**, *68*, 281–287. https://doi.org/10.1016/j.jct.2013.09.017.

(519)	Qin, X.; Cao, X.; Guo, Y.; Xu, L.; Hu, S.; Fang, W. Density, Viscosity, Surface Tension, and Refractive Index for Binary Mixtures of 1,3-Dimethyladamantane with Four C10 Alkanes. *J. Chem. Eng. Data* **2014**, *59*, 775–783. https://doi.org/10.1021/je4008926.

(520)	Kendall, J.; Wright, A. H. The Viscosity of Liquids. IV. Ideal Mixtures of the Types Ether-Ether and Ester-Ester. *J. Am. Chem. Soc.* **1920**, *42*, 1776–1779.

(521)	Meng, X.; Zheng, P.; Wu, J.; Liu, Z. Density and Viscosity Measurements of Diethyl Adipate from (303 to 373) K and up to 20 MPa. *J. Chem. Eng. Data* **2008**, *53*, 1474–1478. https://doi.org/10.1021/je700740e.

(522)	Sah, R. S.; Sinha, B.; Roy, M. N. Study of the Solution Properties of Ternary Mixtures of 1,3-Dioxolane (1), Diethyl Ether (2), and n-Amyl Alcohol (3) and the Corresponding Binary Mixtures by Density, Viscosity, Refractivity, and Ultrasonic Speed Measurements at 298.15 K. *J. Chem. Eng. Data* **2010**, *55*, 4536–4540. https://doi.org/10.1021/je100594u.

(523)	Sah, R. S.; Roy, M. N. Volumetric, Viscometric, Interferrometric and Refractometric Properties of 2-MethoxyethanolYdiethyletherYdichloromethane Ternary System and Its Corresponding Binaries at 298.15K. *Phys. Chem. Liq.* **2011**, *49*, 133–144. https://doi.org/10.1080/00319100903012395.

(524)	Dash, S. K.; Pradhan, S. K.; Dalai, B.; Moharana, L.; Swain, B. B. Studies on Molecular Interaction in Binary Mixtures of Diethyl Ether with Some Alkanols an Acoustic Approach. *Phys. Chem. Liq.* **2012**, *50*, 735–749. https://doi.org/10.1080/00319104.2012.713554.

(525)	Bi, S.; Wu, J.; Liu, Z.; Meng, X. Experimental Investigation of the Viscosity of Saturated Liquid Dimethyl Carbonate. *Xi’an Jiaotong Daxue Xuebao* **2003**, *37*, 903.

(526)	Wright, F. J. Influence of Temperature on Viscosity of Nonassociated Liquids. *J. Chem. Eng. Data* **1961**, *6*, 454–456. https://doi.org/10.1021/je00103a035.

(527)	Naejus, R.; Lemordant, D.; Coudert, R.; Willmann, P. Excess Thermodynamic Properties of Binary Mixtures Containing Linear or Cyclic Carbonates as Solvents at the Temperatures 298.15 K and 315.15 K. *J. Chem. Thermodyn.* **1997**, *29*, 1503–1515. https://doi.org/10.1006/jcht.1997.0260.

(528)	Pal, A.; Dass, G.; Kumar, A. Excess Molar Volumes, Viscosities, and Refractive Indices of Triethylene Glycol Dimethyl Ether with Dimethyl Carbonate, Diethyl Carbonate, and Propylene Carbonate at 298.15 K. *J. Chem. Eng. Data* **1998**, *43*, 738–741. https://doi.org/10.1021/je980016t.

(529)	Pal, A.; Kumar, H.; Kumar, A.; Dass, G. Excess Molar Volumes and Viscosities of Mixtures of Some N-Alkoxyethanols with Dialkyl Carbonates at 298.15 K. *Fluid Phase Equilib.* **1999**, *166*, 245–258. https://doi.org/10.1016/s0378-3812(99)00282-4.

(530)	Comunas, M. J. P.; Baylaucq, A.; Boned, C.; Fernandez, J. High-Pressure Measurements of the Viscosity and Density of Two Polyethers and Two Dialkyl Carbonates. *Int. J. Thermophys.* **2001**, *22*, 749–768. https://doi.org/10.1023/a:1010770831215.

(531)	Pal, A.; Bhardwaj, R. K.; Dass, G. Excess Molar Volumes and Viscosities of Mixtures of Diethylene Glycol Dibutyl Ether with Dialkyl Carbonates at 298.15, 308.15, and 318.15 K. *Int. J. Thermophys.* **2001**, *22*, 769–787. https://doi.org/10.1023/a:1010722915285.

(532)	Mialkowski, C.; Chagnes, A.; Carre, B.; Lemordant, D.; Willmann, P. Excess Thermodynamic Properties of Binary Liquid Mixtures Containing Dimethylcarbonate and -Butyrolactone. *J. Chem. Thermodyn.* **2002**, *34*, 1847–1856. https://doi.org/10.1016/s0021-9614(02)00232-x.

(533)	Rodriguez, A.; Canosa, J.; Dominguez, A.; Tojo, J. Dynamic Viscosities of Diethyl Carbonate with Linear and Secondary Alcohols at Several Temperatures. *J. Chem. Eng. Data* **2004**, *49*, 157–162. https://doi.org/10.1021/je0341413.

(534)	Arbad, B. R. R.; Lande, M. K. K.; Wankhede, N.; Wankhede, D. S. Viscosities, Ultrasonic Velocities at (288.15 and 298.15) K, and Refractive Indices at (298.15) K of Binary Mixtures of 2,4,6-Trimethyl-1,3,5-Trioxane with Dimethyl Carbonate, Diethyl Carbonate, and Propylene Carbonate. *J. Chem. Eng. Data* **2006**, *51*, 68–72. https://doi.org/10.1021/je050183a.

(535)	Comelli, F.; Francesconi, R.; Bigi, A.; Rubini, K. Excess Molar Enthalpies, Molar Heat Capacities, Densities, Viscosities, and Refractive Indices of Dimethyl Sulfoxide + Esters of Carbonic Acid at 308.15 K and Atmospheric Pressure. *J. Chem. Eng. Data* **2006**, *51*, 665–670. https://doi.org/10.1021/je050444g.

(536)	Rodriguez, A.; Pereiro, A. B.; Canosa, J.; Tojo, J. Dynamic Viscosities of the Ternary Liquid Mixtures (Dimethyl Carbonate + Methanol + Ethanol) and (Dimethyl Carbonate + Methanol + Hexane) at Several Temperatures. *J. Chem. Thermodyn.* **2006**, *38*, 505–519. https://doi.org/10.1016/j.jct.2005.07.008.

(537)	Iloukhani, H.; Zarei, H. A.; Behroozi, M. Thermodynamic Studies of Ternary Mixtures of Diethylcarbonate (1)+dimethylcarbonate (2)+N,N-Dimethylacetamide (3) at 298.15 K. *J. Mol. Liq.* **2007**, *135*, 141–145.

(538)	Meng, X.; Zheng, P.; Wu, J.; Liu, Z.-G. Viscosity and Density Measurements of Dimethyl Carbonate. *J. Eng. Thermophys.* **2009**, *30*, 26–30.

(539)	Xing, Y.; Shao, D.; Fang, W.; Guo, Y.; Lin, R. Vapor Pressures and Flash Points for Binary Mixtures of Tricyclo [5.2.1.0^(2.6)] Decane and Dimethyl Carbonate. *Fluid Phase Equilib.* **2009**, *284*, 14–18. https://doi.org/10.1016/j.fluid.2009.06.002.

(540)	Comelli, F.; Bigi, A.; Vitalini, D.; Rubini, K. Densities, Viscosities, Refractive Indices, and Heat Capacities of Poly(Ethylene Glycol-Ran-Propylene Glycol) + Esters of Carbonic Acid at (293.15 and 313.15) K and at Atmospheric Pressure. *J. Chem. Eng. Data* **2010**, *55*, 205–210. https://doi.org/10.1021/je900307z.

(541)	Litaiem, Y.; Dhahbi, M. Measurements and Correlations of Viscosity, Conductivity and Density of an Hydrophobic Ionic Liquid (Aliquat 336) Mixtures with a Non-Associated Dipolar Aprotic Solvent (DMC). *J. Mol. Liq.* **2012**, *169*, 54–62. https://doi.org/10.1016/j.molliq.2012.02.019.

(542)	Meng, X.; Zhang, J.; Wu, J. T.; Liu, Z.-G. Experimental Measurement and Modeling of the Viscosity of Dimethyl Ether. *J. Chem. Eng. Data* **2012**, *57*, 988–993. https://doi.org/10.1021/je201297j.

(543)	Tomida, D.; Nagasaka, T.; Hongo, M.; Yokoyama, C. Viscosity of Gaseous Mixtures of Methoxymethane + Nitrogen. *J. Chem. Eng. Data* **2009**, *54*, 1343–1347. https://doi.org/10.1021/je8009545.

(544)	Wang, X. -p.; Song, B.; Wu, J. -t.; Liu, Z.-G. Gaseous Viscosity of Dimethyl Ether in the Low Density Region. *J. Eng. Thermophys. (China)* **2011**, *32*, 910–912.

(545)	Cousins, D. S.; Laesecke, A. Sealed Gravitational Capillary Viscometry of Dimethyl Ether and Two Next-Generation Alternative Refrigerants. *J. Res. Natl. Inst. Stand. Technol.* **2012**, *117*, 231–255. https://doi.org/10.6028/jres.117.014.

(546)	Thorpe, T. E.; Rodger, J. W. On the Relations between the Viscosity (Internal Friction) of Liquids and Their Chemical Nature. Part II. *Philos. Trans. R. Soc. London, Ser. A* **1897**, *189*, 71.

(547)	Schmidt, A. W.; Hopp, G.; Schoeller, V. Preparation and Physical Data for Monoalkyl Benzenes. *Ber. Dtsch. Chem. Ges., B* **1939**, *72*, 1893–1897.

(548)	Panchenkov, G. M.; Erchenkov, V. V. Temperature Variation of the Coordination Number and Diffusion Coefficient in a Liquid. *Russ. J. Phys. Chem.* **1962**, *36*, 455–457.

(549)	Barlow, A. J.; Lamb, J. D.; Matheson, A. J. *Proc. R. Soc. London, Ser. A* **1966**, *292*, 322.

(550)	Akhundov, T. S. Viscosity of Ethylbenzene. *Izv. Vyssh. Uchebn. Zaved., Neft Gaz* **1973**, *16*, 46;74.

(551)	Shikhaliev, Y. A. Relationship between Viscosity and Activation Energy for Binary Liquid Solutions. *Zh. Fiz. Khim.* **1977**, *51*, 961–963.

(552)	Abdullaev, F. G.; Akhundov, T. S. Experimental Study of the Viscosity of Ethylbenzene. *Izv. Vyssh. Uchebn. Zaved., Neft Gaz* **1985**, *28*, 52.

(553)	Fermeglia, M.; Lapasin, R. Excess Volumes and Viscosities of Binary Mixtures of Organics. *J. Chem. Eng. Data* **1988**, *33*, 415–417. https://doi.org/10.1021/je00054a008.

(554)	Singh, R. P.; Sinha, C. P.; Das, J. C.; Ghosh, P. Viscosity and Density of Ternary Mixtures for Toluene, Ethylbenzene, Bromobenzene, and 1-Hexanol. *J. Chem. Eng. Data* **1989**, *34*, 335–338. https://doi.org/10.1021/je00057a022.

(555)	Krahn, U. G.; Luft, G. Viscosity of Several Liquid Hydrocarbons in the Temperature Range 298-453 K at Pressures up to 200 MPa. *J. Chem. Eng. Data* **1994**, *39*, 670–672. https://doi.org/10.1021/je00016a006.

(556)	Katyal, R. C.; Singh, S.; Rattan, V. K.; Kanda, P.; Acharya, S. Viscosities, Densities, and Ultrasonic Velocities of 3-Pentanone + Ethylbenzene and 3-Pentanone + o-Xylene at (293.15, 303.15, and 313.15) K. *J. Chem. Eng. Data* **2003**, *48*, 1262–1265. https://doi.org/10.1021/je030151s.

(557)	Rattan, V. K.; Singh, S.; Sethi, B. P. S. Viscosities, Densities, and Ultrasonic Velocities of Binary Mixtures of Ethylbenzene with Ethanol, 1-Propanol, and 1-Butanol at (298.15 and 308.15)K. *J. Chem. Eng. Data* **2004**, *49*, 1074–1077. https://doi.org/10.1021/je049912x.

(558)	Rathnam, M. V.; Mohite, S.; Kumar, M. S. S. Viscosity, Density, and Refractive Index of Some (Ester + Hydrocarbon) Binary Mixtures at 303.15 K and 313.15 K. *J. Chem. Eng. Data* **2005**, *50*, 325–329. https://doi.org/10.1021/je0400052.

(559)	Yang, C.; Yu, W.; Ma, P. Densities and Viscosities of Binary Mixtures of Ethylbenzene + N-Methyl-2-Pyrrolidone, Ethylbenzene + Sulfolane, and Styrene + Octane from (303.15 to 353.15) K and Atmospheric Pressure. *J. Chem. Eng. Data* **2005**, *50*, 1197–1203. https://doi.org/10.1021/je049572f.

(560)	Dominguez-Perez, M.; Franjo, C.; Pico, J.; Segade, L.; Cabeza, O.; Jimenez, E. Experimental Study of the Dynamic Viscosity Deviations in the Binary Systems: Hexane + Ethylbenzene, + o-Xylene, + m-Xylene, + p-Xylene at 298.15 K. *Int. J. Thermophys.* **2009**, *30*, 1197–1201. https://doi.org/10.1007/s10765-009-0622-2.

(561)	Rathnam, M. V.; Mohite, S.; Kumar, M. S. Thermophysical Properties of Isoamyl Acetate or Methyl Benzoate + Hydrocarbon Binary Mixtures, at (303.15 and 313.15) K. *J. Chem. Eng. Data* **2009**, *54*, 305–309. https://doi.org/10.1021/je800325d.

(562)	Rathnam, M. V.; Mohite, S.; Kumar, M. S. S. Density, Excess Volume, and Viscosity of Vinyl Acetate or Benzyl Acetate with (o-, m-, p-)Xylenes and Ethylbenzene at T = (303.15 and 313.15) K. *J. Solution Chem.* **2010**, *39*, 1735–1748. https://doi.org/10.1007/s10953-010-9610-1.

(563)	Bhatia, S. C.; Rani, R.; Bhatia, R. Viscosities, Densities, Speeds of Sound and Refractive Indices of Binary Mixtures of o-Xylene, m-Xylene, p-Xylene, Ethylbenzene and Mesitylene with 1-Decanol at 298.15 and 308.15 K. *J. Mol. Liq.* **2011**, *159*, 132–141. https://doi.org/10.1016/j.molliq.2010.12.011.

(564)	Rathnam, M. V.; Mohite, S.; Kumar, M. S. S. Density and Viscosity of Alkyl Acetates + Aromatic Hydrocarbons at (303.15 and 313.15) K. *J. Solution Chem.* **2011**, *40*, 390–402.

(565)	Saravanakumar, K.; Lavanya, T. G.; Baskaran, R.; Kubendran, T. R. Thermophysical and Thermoacoustical Properties of Benzaldehyde Mixtures with Ethylbenzene at 303.15, 308.15, and 313.15 K and a Pressure of 0.1 MPa. *Zh. Fiz. Khim.* **2012**, *86*, 647–655.

(566)	Barega, E. W.; Zondervan, E.; Haan, A. B. d. Experimental Density, Viscosity, Interfacial Tension and Water Solubility of Ethyl Benzene - a-Methyl Benzyl Alcohol - Water System. *J. Chem. Thermodyn.* **2013**, *63*, 31–37. https://doi.org/10.1016/j.jct.2013.03.023.

(567)	Zhang, J.; Zhang, P.; Han, F.; Chen, G.; Deng, R.; Wei, X.-H. Gas-Liquid Equilibrium Data for a Mixture Gas of Sulfur Dioxide + Nitrogen with Ethylene Glycol Aqueous Solutions at 298.15 K and 123.15 KPa. *J. Chem. Eng. Data* **2008**, *53*, 2372–2374. https://doi.org/10.1021/je800271e.

(568)	Taylor, C. A.; Rinkenbach, W. H. Ethylene Glycol an Evaluation of Available Information on the Physical Properties of This Compound. *Ind. Eng. Chem.* **1926**, *18*, 676–678.

(569)	Rinkenbach, W. H. Properties of Diethylene Glycol. *Ind. Eng. Chem.* **1927**, *19*, 475–476.

(570)	Timmermans, J.; Hennaut-Roland, M. Physical Constants of 20 Organic Compounds. VII. *J. Chim. Phys. Phys.-Chim. Biol.* **1935**, *32*, 501.

(571)	Mellan, I. *Polyhydric Alcohols*; 1962.

(572)	Davids, E. L.; Findlay, T. J. V. Phase Diagram for Ethylene Glycol and Triethylamine. *Aust. J. Chem.* **1967**, *20*, 1343.

(573)	Jerome, F. S.; Tseng, J. T.; Fan, L. T. Viscosities of Aqueous Glycerol Solutions. *J. Chem. Eng. Data* **1968**, *13*, 496–496. https://doi.org/10.1021/je60039a010.

(574)	Oyevaar, M. H.; Morssinkhof, R. W. J.; Westerterp, K. R. Density, Viscosity, Solubility, and Diffusivity of Carbon Dioxide an Nitrous Oxide in Solutions of Diethanolamine in Aqueous Ethylene Glycol at 298 K. *J. Chem. Eng. Data* **1989**, *34*, 77–82. https://doi.org/10.1021/je00055a022.

(575)	Kumagai, A.; Mochida, H.; Takahashi, S. Liquid Viscosities and Densities of HFC-134a (1,1,1,2- Tetrafluoroethane) + Glycol Mixtures. *Int. J. Thermophys.* **1993**, *14*, 45–53. https://doi.org/10.1007/bf00522660.

(576)	Reddy, V. K.; Reddy, K. S.; Krishnaiah, A. Excess Volumes, Speeds of Sound, and Viscosities for Mixtures of 1,2- Ethanediol and Alkoxy Alcohols with Water at 308.15 K. *J. Chem. Eng. Data* **1994**, *39*, 615–617. https://doi.org/10.1021/je00015a051.

(577)	Pal, A.; Singh, W. Speeds of Sound and Viscosities in Aqueous Poly(Ethylene Glycol) Solutions at 303.15 and 308.15 K. *J. Chem. Eng. Data* **1997**, *42*, 234–237. https://doi.org/10.1021/je960173x.

(578)	Pal, A.; Sharma, S. Excess Molar Volumes and Viscosities of 1-Propanol + Ethylene Glycol, + Ethylene Glycol Monomethyl, + Ethylene Glycol Dimethyl, + Diethylene Glycol Dimethyl, + Triethylene Glycol Dimethyl, + Diethylene Glycol Diethyl, and + Diethylene Glycol Dibutyl Ethers at 298.15 K. *J. Chem. Eng. Data* **1998**, *43*, 532–536. https://doi.org/10.1021/je9702955.

(579)	Tsierkezos, N. G.; Molinou, I. E. Thermodynamic Properties of Water + Ethylene Glycol at 283.15, 293.15, 303.15, 313.15. *J. Chem. Eng. Data* **1998**, *43*, 989–993. https://doi.org/10.1021/je9800914.

(580)	Saleh, M.; Begum, S.; Begum, S. K.; Begum, B. A. VISCOSITY OF DILUTE AQUEOUS SOLUTIONS OF SOME DIOLS. *Phys. Chem. Liq.* **1999**, *37*, 785–801.

(581)	Lech, T.; Czechowski, G.; Jadzyn, J. Viscosity of the Series of 1,n-Alkanediols. *J. Chem. Eng. Data* **2001**, *46*, 725–727. https://doi.org/10.1021/je0003750.

(582)	Vijaya Kumar Naidu, B.; Chowdoji Rao, K.; Subha, M. C. S. Densities and Viscosities of Mixtures of Some Glycols and Polyglycols in Dimethyl Sulfoxide at 308.15 K. *J. Chem. Eng. Data* **2002**, *47*, 379–382. https://doi.org/10.1021/je0101395.

(583)	Naidu, B. V. K.; Rao, K. C.; Subha, M. C. S. Densities, Viscosities, and Excess Properties for Binary Mixtures of Some Glycols and Polyglycols in N-Methylacetamide at 308.15 K. *J. Chem. Eng. Data* **2003**, *48*, 625–627. https://doi.org/10.1021/je020178w.

(584)	Sastry, N. V.; Patel, M. C. Densities, Excess Molar Volumes, Viscosities, Speeds of Sound, Excess Isentropic Compressibilities, and Relative Permittivities for Alkyl (Methyl, Ethyl, Butyl, and Isoamyl) Acetates + Glycols at Different Temperatures. *J. Chem. Eng. Data* **2003**, *48*, 1019–1027. https://doi.org/10.1021/je0340248.

(585)	Sun, T.; Teja, A. S. Density, Viscosity and Thermal Conductivity of Aqueous Ethylene, Diethylene and Triethylene Glycol Mixtures between 290 K and 450 K. *J. Chem. Eng. Data* **2003**, *48*, 198–202. https://doi.org/10.1021/je025610o.

(586)	Yang, C.; Ma, P.; Jing, F.; Tang, D. Excess Molar Volumes, Viscosities, and Heat Capacities for the Mixtures of Ethylene Glycol + Water from 273.15 K to 353.15 K. *J. Chem. Eng. Data* **2003**, *48*, 836–840. https://doi.org/10.1021/je020140j.

(587)	Sinha, A.; Roy, M. N. Densities, Viscosities, and Sound Speeds of Some Acetate Salts in Binary Mixtures of Tetrahydrofuran and Methanol at (303.15, 313.15, and 323.15) K. *J. Chem. Eng. Data* **2006**, *51*, 1415–1423. https://doi.org/10.1021/je060113j.

(588)	Yang, C.; Liu, Z.; Lai, H.; Ma, P. Excess Molar Volumes and Viscosities of Binary Mixtures of P-Cresol with Ethylene Glycol and Methanol at Different Temperature and Atmospheric Pressure. *J. Chem. Eng. Data* **2006**, *51*, 457–461. https://doi.org/10.1021/je050376y.

(589)	Yang, C.; Yu, W.; Tang, D. Densities and Viscosities of Binary Mixtures of M-Cresol with Ethylene Glycol or Methanol over Several Temperatures. *J. Chem. Eng. Data* **2006**, *51*, 935–939. https://doi.org/10.1021/je0504156.

(590)	Gurung, B. B.; Roy, M. N. Study of Densities, Viscosity Deviations, and Isentropic Compressibilities of Ternary Liquid Mixtures of Water and Ethane-1,2-Diol with Some Monoalcohols at Various Temperatures. *Phys. Chem. Liq.* **2007**, *45*, 331–343.

(591)	Mehta, S. K.; Ram, G.; Kumar, V.; Bhasin, K. K. Structural and Interactional Studies of Homologous Series of a,x-Alkanediols in N,N-Dimethylformamide. *J. Chem. Thermodyn.* **2007**, *39*, 781–790. https://doi.org/10.1016/j.jct.2006.10.004.

(592)	Song, X.; Luo, Y.; Wang, J.; Yu, J. Determination and Correlation of the Densities and Viscosities of Methanol-Glycol Binary Mixtures. *Huadong Huagong Xueyuan Xuebao* **2007**, *33*, 4;750.

(593)	Wu, C.; Liu, D.; Ma, Z.; Liu, X. Determination and Correlation of Viscosities for Ethylene Glycol and Diethylene Glycol with a Catalyst. *Nanjing Huagong Daxue Xuebao* **2007**, *29*, 4;51.

(594)	Mehta, S. K.; Ram, G.; Bhasin, K. K. Molecular Interactions of a,x-Alkanediols in Pyrrolidin-2-One: Thermophysical and Spectroscopic Measurements. *J. Chem. Thermodyn.* **2008**, *40*, 498–508. https://doi.org/10.1016/j.jct.2007.08.005.

(595)	Nain, A. K. Ultrasonic and Viscometric Studies of Molecular Interactions in Binary Mixtures of Formamide with Ethanol, 1-Propanol, 1,2-Ethanediol and 1,2-Propanediol at Different Temperatures. *J. Mol. Liq.* **2008**, *140*, 108–116.

(596)	Shi, D.; Gao, G.; Li, D.; Dong, J.; Wang, L. New Device for Fast Measuring Surface Tension, Density and Viscosity of Liquids. *Fluid Phase Equilib.* **2008**, *273*, 87–91. https://doi.org/10.1016/j.fluid.2008.08.015.

(597)	Zorebski, E.; Lubowiecka-Kostka, B. Thermodynamic and Transport Properties of (1,2-Ethanediol + 1-Nonanol) at Temperatures from (298.15 to 313.15) K. *J. Chem. Thermodyn.* **2009**, *41*, 197–204. https://doi.org/10.1016/j.jct.2008.09.018.

(598)	Doghaeei, A.; Rostami, A.; Omrani, A. Densities, Viscosities, and Volumetric Properties of Binary Mixtures of 1,2-Propanediol + 1-Heptanol or 1-Hexanol and 1,2-Ethanediol + 2-Butanol or 2-Propanol at T = (298.15, 303.15, and 308.15) K. *J. Chem. Eng. Data* **2010**, *55*, 2894–2899. https://doi.org/10.1021/je900954y.

(599)	Ge, M.-L.; Ma, J.-L.; Chu, B. Densities and Viscosities of Propane-1,2,3-Triol + Ethane-1,2-Diol at T = (298.15 to 338.15) K. *J. Chem. Eng. Data* **2010**, *55*, 2649–2651. https://doi.org/10.1021/je900938b.

(600)	Li, H.; Chen, X.; Zhang, Z. Measurement and Correlation of the Densities and Viscosities of 2-Bromopropane + Ethanol Binary Mixtures at Temperatures from (298.15 to 318.15) K. *J. Chem. Eng. Data* **2010**, *55*, 3441–3444. https://doi.org/10.1021/je100159m.

(601)	Kinart, C. M.; Klimczak, M.; Kinart, W. Viscosimetric and Structural Properties of Binary Mixtures of 2-Butoxyethanol with Ethylene Glycol, Diethylene Glycol, Triethylene Glycol and Tetraethylene Glycol Systems at T^293.15, 298.15 and 303.15K. *Phys. Chem. Liq.* **2011**, *49*, 181–191. https://doi.org/10.1080/00319100903191405.

(602)	Kinart, C. M.; Maj, M. Viscosities of Ternary Mixtures 2-Alkoxyethanol + Sulfolane + Ethylene Glycols at T^303.15K. *Phys. Chem. Liq.* **2011**, *49*, 508–518.

(603)	Sagdeev, D. I.; Fomina, M. G.; Mukhamedzyanov, G. K.; Abdulagatov, I. M. Experimental Study of the Density and Viscosity of Polyethylene Glycols and Their Mixtures at Temperatures from 293 K to 473 K and at Atmospheric Pressure. *J. Chem. Thermodyn.* **2011**, *43*, 1824–1843. https://doi.org/10.1016/j.jct.2011.06.013.

(604)	Sagdeev, D. I.; Fomina, M. G.; Mukhamedzyanov, G. K.; Abdulagatov, I. M. Experimental Study of the Density and Viscosity of Polyethylene Glycols and Their Mixtures at Temperatures from 293 K to 465 K and at High Pressures up to 245 MPa. *Fluid Phase Equilib.* **2012**, *315*, 64–76. https://doi.org/10.1016/j.fluid.2011.11.022.

(605)	Gao, F.; Niu, Y.; Zhang, J.; Sun, S.; Wei, X. Solubility for Dilute Sulfur Dioxide in Binary Mixtures of N,N-Dimethylformamide + Ethylene Glycol at T = 308.15 Kand p = 122.66 KPa. *J. Chem. Thermodyn.* **2013**, *62*, 8–16. https://doi.org/10.1016/j.jct.2013.02.010.

(606)	Moosavi, M.; Motahari, A.; Omrani, A.; Rostami, A. A. Investigation on Some Thermophysical Properties of Poly(Ethylene Glycol) Binary Mixtures at Different Temperatures. *J. Chem. Thermodyn.* **2013**, *58*, 340–350. https://doi.org/10.1016/j.jct.2012.11.016.

(607)	Quijada-Maldonado, E.; Meindersma, G. W.; De Haan, A. B. Viscosity and Density Data for the Ternary System Water(1) Ethanol(2) Ethylene Glycol(3) between 298.15 K and 328.15 K. *J. Chem. Thermodyn.* **2013**, *57*, 500–505. https://doi.org/10.1016/j.jct.2012.08.024.

(608)	Kondaiah, M.; Sreekanth, K.; Kumar, D. S.; Nayeem, S. M.; Rao, D. K. Densities, Viscosities, and Excess Properties for Binary Mixtures of Ethylene Glycol with Amides at 308.15 K. *J. Therm. Anal. Calorim.* **2014**, *118*, 475–483. https://doi.org/10.1007/s10973-014-4019-5.

(609)	Li, Y.; Zheng, D.; Dong, L.; Nie, N.; Xiong, B. Solubilities of CO2 in, and Densities and Viscosities of, the Piperazine + 1-Ethyl-3-Methyl-Imidazolium Acetate + H2O System. *J. Chem. Eng. Data* **2014**, *59*, 618–625. https://doi.org/10.1021/je400491p.

(610)	Pastoriza-Gallego, M. J.; Lugo, L.; Cabaleiro, D.; Legido, J. L.; Pineiro, M. M. Thermophysical Profile of Ethylene Glycol-Based ZnO Nanofluids. *J. Chem. Thermodyn.* **2014**, *73*, 23–30. https://doi.org/10.1016/j.jct.2013.07.002.

(611)	Zhang, Z.; Zhou, Q.; Lu, X.; Qiao, C.; Zhang, S. Densities and Viscosities of Binary Mixtures Containing 1,3-Dimethylimidazolium Dimethylphosphate and Alcohols. *J. Chem. Eng. Data* **2014**, *59*, 2377–2388. https://doi.org/10.1021/je401124t.

(612)	Sun, S.; Niu, Y.; Gao, F.; Shen, J.; Wei, X. Solubility Properties and Spectral Characterization of Dilute SO2 in Binary Mixtures of Urea + Ethylene Glycol. *J. Chem. Eng. Data* **2015**, *60*, 161–170. https://doi.org/10.1021/je5009119.

(613)	Zhang, H.; Gong, M.; Li, H.; Guo, H.; Dong, X.; Wu, J. Gaseous PVTx Measurements for (HFC134a+HC290) by a Compact Single-Sinker Densimeter. *J. Chem. Thermodyn.* **2015**, *81*, 60–65. https://doi.org/10.1016/j.jct.2014.10.001.

(614)	Diller, D. E.; Saber, J. M. Measurements of the Viscosity of Compressed Gaseous and Liquid Ethane. *Physica A (Amsterdam)* **1981**, *108*, 143–152.

(615)	Gerf, S. F.; Galkov, G. I. Viscosity of Liquified Pure Gases and Their Mixtures. *Zh. Tekh. Fiz.* **1940**, *10*, 725–732.

(616)	Galkov, G. I.; Gerf, S. F. Viscosity of Liquefied Pure Gases and Their Mixtures. *Zh. Tekh. Fiz.* **1941**, *11*, 613–615.

(617)	Ishida, Y. Determination of Viscosities and of the Stokes-Millikan Law Constant by the Oil-Drop Method. *Phys. Rev.* **1923**, *21*, 550.

(618)	Trautz, M.; Sorg, K. G. Viscosity, Heat Conductivity and Diffusion in Gas Mixtures. XVI. The Viscosity of u Hydrogen, Methane, Ethane, Propane and Their Binary Mixtures. *Ann. Phys. (Berlin)* **1931**, *10*, 81–96.

(619)	De Rocco, A.; Halford, I. Intermolecular Potentials of Argon, Methane and Ethane. *J. Chem. Phys.* **1958**, *28*, 1152.

(620)	Baron, J. D.; Roof, J. G.; Wells, F. W. Viscosity of Nitrogen, Methane, Ethane, and Propane at Elevated Temperature and Pressure. *J. Chem. Eng. Data* **1959**, *4*, 283–288. https://doi.org/10.1021/je60003a024.

(621)	Iwasaki, H.; Takahashi, M. Viscosity of Carbon Dioxide and Ethane. *J. Chem. Phys.* **1981**, *74*, 1930.

(622)	Wilhelm, J.; Seibt, D.; Vogel, E.; Buttig, D.; Hassel, E. Viscosity Measurements on Gaseous Ethane. *J. Chem. Eng. Data* **2006**, *51*, 136–144. https://doi.org/10.1021/je050287l.

(623)	Smith, A. S.; Brown, G. G. Correlating Fluid Viscosity. *Ind. Eng. Chem.* **1943**, *35*, 705.

(624)	Eakin, B. E.; Starling, K. E.; Dolan, J. P.; Ellington, R. T. Liquid, Gas, and Dense Fluid Viscosity of Ethane. *J. Chem. Eng. Data* **1962**, *7*, 33–36. https://doi.org/10.1021/je60012a010.

(625)	Seibt, D.; Voss, K.; Herrmann, S.; Vogel, E.; Hassel, E. Simultaneous Viscosity-Density Measurements on Ethane and Propane over a Wide Range of Temperature and Pressure Including the Near-Critical Region. *J. Chem. Eng. Data* **2011**, *56*, 1476–1493. https://doi.org/10.1021/je101178u.

(626)	Hendl, S.; Vogel, E. The Viscosity of Gaseous Ethane and Its Initial Density Dependence. *Fluid Phase Equilib.* **1992**, *76*, 259–272. https://doi.org/10.1016/0378-3812(92)85093-n.

(627)	Mizushima, S. *Bull. Chem. Soc. Jpn.* **1926**, *1*, 143.

(628)	Hatem, S. Study of the Mixtures Ethanol-Pyridine, Ethanol-Quinoline. *Bull. Soc. Chim. Fr.* **1949**, 599.

(629)	Khalimonova. *Ukr. Fiz. Zh.* **1962**, *7*, 483.

(630)	Brunson, R. R.; Byers, C. H. Viscosities of Alcohol-Hydrocarbon Systems in the Critical Region. A Dynamic Laser Light Scattering Approach. *J. Chem. Eng. Data* **1989**, *34*, 46–52. https://doi.org/10.1021/je00055a015.

(631)	Ali, A.; Nain, A. K.; Kamil, M. Physical-Chemical Studies of Nonaqueous Binary Liquid Mixtures at Various Temperatures. *Thermochim. Acta* **1996**, *274*, 209–221. https://doi.org/10.1016/0040-6031(95)02719-x.

(632)	Thole, F. B. CCLXVI. Viscosity and Association Part I. Association of the Phenols. *J. Chem. Soc.* **1910**, *97*, 2596–2606.

(633)	Bingham, E. C.; White, G. F.; Thomas, A.; Cadwell, J. L. Fluidity and Hydration Theory. II. *Z. Phys. Chem. (Leipzig)* **1913**, *83*, 641–673.

(634)	Dolian, F. E.; Briscoe, H. T. The Viscosities of Solutions of Chlorides in Certain Solvents. *J. Phys. Chem.* **1937**, *41*, 1129.

(635)	Vierk, A.-L. Experimental Investigation of the Two-Component Systems: Water-Aceto- Nitrole, Water-Dioxane, Ethanol-Acetonitrile and Cyclohexane-Dioxane. *Z. Anorg. Chem.* **1950**, *261*, 283–296. https://doi.org/10.1002/zaac.19502610504.

(636)	Misra, B. N.; Varshni, Y. P. Viscosity Temperature Relation for Solutions. *J. Chem. Eng. Data* **1961**, *6*, 194–196. https://doi.org/10.1021/je60010a007.

(637)	Bamelis, P.; Huyskens, P.; Meeussen, E. No.18 Influence of the Association of Alcohols on The Viscosity of Solutions. *J. Chim. Phys. Phys.-Chim. Biol.* **1965**, *62*, 158–170.

(638)	Komarenko, V. G.; Manzhelii, V. G.; Radtsig, A. V. Density and Viscosity of Normal Alcohols at Low Temperatures. *Ukr. Fiz. Zh.* **1967**, *12*, 676.

(639)	Tommila, E.; Lindell, E.; Virtalaine, M.-L.; Laakso, R. Densities, Viscosities, Surface Tensions, Dielectric Constants, Vapor Pressures, Activities, and Heats of Mixing of Sulpholane + Water, Sulpholane + Methanol, and Sulpholane + Ethanol Mixtures. *Suom. Kemistil. B* **1969**, *42*, 95–104.

(640)	Phillips, T. W.; Murphy, K. P. Liquid Viscosity in Halogenated Refrigerants. *ASHRAE Trans.* **1970**, *76*, 146–156.

(641)	Shikhaliev, Y. A.; Mamedov, A. A.; Aleskerov, M. A.; Khalilov, S. K. Temperature and Concentration Variation of the Viscosities of Toluene- Ethanol and Heptane-Ethanol Mixtures. *Zh. Fiz. Khim.* **1974**, *48*, 1696.

(642)	Lee, F.-M.; Lahti, L. E.; Stoops, C. E. Solution Properties of Urea + Alcohol + Water Mixtures. *J. Chem. Eng. Data* **1976**, *21*, 36–40. https://doi.org/10.1021/je60068a003.

(643)	Tanaka, Y.; Yamamoto, T.; Satomi, Y.; Kubota, H.; Makita, T. Specific Volume and Viscosity of Ethanol-Water Mixtures under High Pressure. *Rev. Phys. Chem. Jpn.* **1977**, *47*, 12–24.

(644)	Baykut, S.; Tanrikulu, M. Investigations of Certain Properties of N-Acids and n-Alcohols and Some Mathematical Relationships between These Properties and Their Carbon Numbers. *Chim. Acta Turc.* **1980**, *8*, 69–76.

(645)	Pikkarainen, L. Density & Viscosity of Binary Solvent Mixtures of N-Methylmethane Sulfonamide with Ethanol, Pyridine & N,N-Dimethylacetamide. *Finn. Chem. Lett.* **1980**, 185.

(646)	Kumar, A.; Prakash, O.; Prakash, S. Ultrasonic Velocities, Densities, and Viscosities of Trimethylamine in Methanol, Ethanol, and 1-Propanol. *J. Chem. Eng. Data* **1981**, *26*, 64–67. https://doi.org/10.1021/je00023a021.

(647)	Pikkarainen, L. Densities and Viscosities of Binary Mixtures of N,N-Dimethylacetamide with Aliphatic Alcohols. *J. Chem. Eng. Data* **1983**, *28*, 344–347. https://doi.org/10.1021/je00033a019.

(648)	Pikkarainen, L. Densities and Viscosities of Binary Solvent Mixtures of N,N-Dimethyl- Mathanesulfonamide with Aliphatic Alcohols. *Finn. Chem. Lett.* **1983**, 63.

(649)	Rauf, M. A.; Stewart, G. H.; Farhat-Aziz. Viscosities and Densities of Binary Mixtures of 1-Alkanols from 15 to 55 C. *J. Chem. Eng. Data* **1983**, *28*, 324–328. https://doi.org/10.1021/je00033a012.

(650)	Papanastasiou, G. E.; Papoutsis, A. D.; Kokkinidis, I. Physical Behavior of Some Reaction Media. Density, Viscosity, Dielectric Constant, and Refractive Index Changes of Ethano-Dioxane Mixtures at Several Temperatures. *J. Chem. Eng. Data* **1987**, *32*, 377–381. https://doi.org/10.1021/je00049a028.

(651)	Tanaka, Y.; Matsuda, Y.; Fujiwara, H.; Kubota, H.; Makita, T. Viscosity of (Water + Alcohol) Mixtures under High Pressure. *Int. J. Thermophys.* **1987**, *8*, 147–163. https://doi.org/10.1007/bf00515199.

(652)	Crabtree, A. M.; O’Brien, J. F. Excess Viscosities of Binary Mixtures of Chloroform and Alcohols. *J. Chem. Eng. Data* **1991**, *36*, 140–142. https://doi.org/10.1021/je00002a003.

(653)	Garcia, B.; Herrera, C.; Leal, J. M. Shear Viscosities of Binary Liquid Mixtures: 2-Pyrrolidinone with 1- Alkanols. *J. Chem. Eng. Data* **1991**, *36*, 269–274. https://doi.org/10.1021/je00003a004.

(654)	Pikkarainen, L. Thermodynamic and Physicochemical Properties of Binary Solvent Mixtures of Hexamethylphosphoric Triamide with Aliphatic Alcohols. A Viscometric Study. *Thermochim. Acta* **1992**, *197*, 463–468. https://doi.org/10.1016/0040-6031(92)85047-y.

(655)	Aminabhavi, T. M.; Aralaguppi, M. I.; Harogoppad, S. B.; Balundgi, R. H. Densities, Viscosities, Refractive Indices, and Speeds of Sound for Methyl Acetoacetate + Aliphatic Alcohols (C1-C8). *J. Chem. Eng. Data* **1993**, *38*, 31–39. https://doi.org/10.1021/je00009a008.

(656)	Assael, M. J.; Dymond, J. H.; Polimatidou, S. K. Correlation and Prediction of Dense Fluid Transport Coefficients. VI. n-Alcohols. *Int. J. Thermophys.* **1994**, *15* (2), 189–201.

(657)	Papaioannou, D.; Panayioton, C. Viscosity of Alkanol + Alkane Mixtures at Moderately High Pressures. *J. Chem. Eng. Data* **1994**, *39*, 463–466. https://doi.org/10.1021/je00015a013.

(658)	Rodriguez, M.; Galan, M.; Munoz, M. J.; Martin, R. Viscosity of Triglycerides + Alcohols from 278 to 313 K. *J. Chem. Eng. Data* **1994**, *39*, 102–105. https://doi.org/10.1021/je00013a027.

(659)	El-Banna, M. M.; Ramadan, M. Sh. Density and Viscosity of Octyl, Decyl, and Dodecyl Sodium Sulfates in Ethanol. *J. Chem. Eng. Data* **1995**, *40*, 367–370. https://doi.org/10.1021/je00018a004.

(660)	Lee, M. J.; Lin, T.-K. Density and Viscosity for Monoethanolamine + Water, + Ethanol, and + 2- Propanol. *J. Chem. Eng. Data* **1995**, *40*, 336–339. https://doi.org/10.1021/je00017a074.

(661)	Nikam, P. S.; Jadhav, M. C.; Hasan, M. Density and Viscosity of Mixtures of Nitrobenzene with Methanol, Ethanol, Propan-1-Ol, Propan-2-Ol, Butan-1-Ol, 2-Methylpropan-1-Ol, and 2- Methylpropan-2-Ol at 298.15 and 303.15 K. *J. Chem. Eng. Data* **1995**, *40*, 931–934. https://doi.org/10.1021/je00020a044.

(662)	Papaioannou, D.; Panayioton, C. Viscosity of Binary Mixtures of Propylamine with Alkanols at Moderately High Pressures. *J. Chem. Eng. Data* **1995**, *40*, 202–209. https://doi.org/10.1021/je00017a042.

(663)	Aucejo, A.; Burguet, M. C.; Munoz, R. Densities, Viscosities, and Refractive Indices of Some Binary Liquid Systems of Ethanol + Isomers of Hexanol at 298.15 K. *J. Chem. Eng. Data* **1996**, *41*, 1131–1134. https://doi.org/10.1021/je9601265.

(664)	Aalto, M.; Liukkonen, S. Bubble Point Pressures and Densities for the Binary Systems of Propane with Triacontane, Hexatriacontane, Tetracontane, Pentacontane, and Squalane at 353−373 K and 4.00−7.00 MPa. *J. Chem. Eng. Data* **1996**, *41* (1), 79–83. https://doi.org/10.1021/je9501393.

(665)	Nikam, P. S.; Jadhay, M. C.; Hasan, M. Density and Viscosity of Mixtures of Dimethyl Sulfoxide + Methanol, + Ethanol, +Propan-1-Ol, +Propan-2-Ol, +Butan-1-Ol, +2-Methylpropan-1-Ol, and +2-Methylpropan-2-Ol at 298.15 K and 303.15 K. *J. Chem. Eng. Data* **1996**, *41*, 1028–1031. https://doi.org/10.1021/je9600358.

(666)	Nikam, P. S.; Mahale, T. R.; Hasan, M. Density and Viscosity of Binary Mixtures of Ethyl Acetate with Methanol, Ethanol, Propan-1-Ol, Propan-2-Ol, Butan-1-Ol, 2-Methylpropan-1-Ol, and 2- Methylpropan-2-Ol at (298.15, 303.15, and 308.15) K. *J. Chem. Eng. Data* **1996**, *41*, 1055–1058. https://doi.org/10.1021/je960090g.

(667)	Orge, B.; Iglesias, M.; Rodriguez, A.; Canosa, J. M.; Tojo, J. Mixing Properties of (Methanol, Ethanol, or 1-Propanol) with (n-Pentane, n-Hexane, n-Heptane and n-Octane) at 298.15 K. *Fluid Phase Equilib.* **1997**, *133*, 213–227. https://doi.org/10.1016/s0378-3812(97)00031-9.

(668)	Aminabhavi, T. M.; Patil, V. B. Density, Viscosity, and Speed of Sound in Binary Mixtures of 1-Chloronaphthalene with Methanol, Ethanol, Propan-1-Ol, Butan-1-Ol, Pentan-1-Ol, and Hexan-1-Ol in the Temperature Range (298.15-308.15) K. *J. Chem. Eng. Data* **1998**, *43*, 504–508. https://doi.org/10.1021/je980032q.

(669)	Canosa, J.; Rodriguez, A.; Tojo, J. Dynamic Viscosity and Speed of Sound of the Ternary Mixture Methyl Acetate + Methanol + Ethanol at 298.15. *J. Chem. Eng. Data* **1998**, *43*, 961–966. https://doi.org/10.1021/je980076z.

(670)	Kumagai, A.; Yokoyama, C. Liquid Viscosity of Binary Mixtures of Methanol with Ethanol and 1-Propanol from 273.15 to 333.15 K. *Int. J. Thermophys.* **1998**, *19*, 3–13. https://doi.org/10.1023/a:1021438800094.

(671)	Nikam, P. S.; Shirsat, L. N.; Hasan, M. Density and Viscosity Studies of Binary Mixtures of Acetonitrile with Methanol, Ethanol, Propan-1-Ol, Propan-2-Ol, Butan-1-Ol, 2-Methylpropan-1-Ol, and 2-Methylpropan-2-Ol at (298.15, 303.15, 308.15,. *J. Chem. Eng. Data* **1998**, *43*, 732–737. https://doi.org/10.1021/je980028e.

(672)	Oswal, S. L.; Desai, H. S. Studies of Viscosity and Excess Molar Volume of Binary Mixtures. 1. Propylamine + 1 -Alkanol Mixtures at 303.15 and 313.15 K. *Fluid Phase Equilib.* **1998**, *149*, 359–376. https://doi.org/10.1016/s0378-3812(98)00318-5.

(673)	Aminabhavi, T. M.; Patil, V. B.; Banerjee, K. Thermodynamic Interactions in Binary Mixtures of Ethenylbenzene with Methanol, Ethanol, Butan-1-Ol, Pentan-1-Ol, and Hexan-1-Ol in the Temperature Range 298.15-308.15 K. *J. Chem. Eng. Data* **1999**, *44*, 1291–1297. https://doi.org/10.1021/je990120q.

(674)	Haraschta, P.; Heintz, A.; Lehmann, J. K.; Peters, A. Excess Molar Volumes and Viscosities of Binary Mixtures of 2-Methylpuridine with Methanol, Ethanol, Propan-1-Ol, Propan-2-Ol, Butan-2-Ol, and 2-Methylpropan-2-Ol at 298.15 K and Atmospheric Pressure. *J. Chem. Eng. Data* **1999**, *44*, 932–935. https://doi.org/10.1021/je990086f.

(675)	Oswal, S. L.; Desai, H. S. Studies of Viscosity and Excess Molar Volume of Binary Mixtures 2. Butylamine + I -Alkanol Mixtures at 303.15 and 313.15 K. *Fluid Phase Equilib.* **1999**, *161*, 191–204. https://doi.org/10.1016/s0378-3812(99)00165-x.

(676)	Prasad, T. E. V.; Chandrika, K.; Haritha, M.; Geetha, N. B.; Prasad, D. H. L. DENSITY AND VISCOSITY OF ETHANOL + 0-XYLENE, ETHANOL + m-XYLENE, ETHANOL +p-XYLENE AND METHANOL + O-XYLENE MIXTURES. *Phys. Chem. Liq.* **1999**, *1999*, 429–434.

(677)	Henni, A.; Maham, Y.; Tontiwachwuthlkul, P.; Chakma, A.; Mather, A. E. Densities and Viscosities for Binary Mixtures of N-Methyldiethanolamine + Triethylene Glycol Monomethyl Ether from 25 C to 70 C and N-Methyldiethanolamine + Ethanol Mixtures at 40 C. *J. Chem. Eng. Data* **2000**, *45*, 247–253. https://doi.org/10.1021/je9902140.

(678)	Nikam, P. S.; Jagdale, B. S.; Sawant, A. B.; Hasan, M. Densities and Viscosities of Binary Mixtures of Toluene with Methanol, Ethanol, Propan-1-Ol, Butan-1-Ol, Pentan-1-Ol, and 2-Methylpropan-2-Ol at (303.15, 308.15, 313.15) K. *J. Chem. Eng. Data* **2000**, *45*, 559–563. https://doi.org/10.1021/je990317i.

(679)	Pan, I.-C.; Tang, M.; Chen, Y. P. Densities and Viscosities of Binary Liquid Mixtures of Vinyl Acetate, Diethyl Oxalate, and Dibutyl Phthalate with Normal Alkanols at 303.15 K. *J. Chem. Eng. Data* **2000**, *45*, 1012–1015. https://doi.org/10.1021/je0001328.

(680)	Tu, C.-H.; Liu, C.-Y.; Wang, W.-F.; Chou, Y.-T. Volumetric and Viscometric Properties of Binary Mixtures of Aliphatic Alcohols (C1-C4) with Nitroethane from 293.15 K to 313.15 K. *J. Chem. Eng. Data* **2000**, *45*, 450–456. https://doi.org/10.1021/je9902589.

(681)	Ku, S.-C.; Peng, I.-H.; Tu, C.-H. Density and Viscosity of Mixtures of Alkoxypropanols with Ethanol at T = (298.15, 308.15, and 318.15) K. *J. Chem. Eng. Data* **2001**, *46*, 1392–1398. https://doi.org/10.1021/je010067h.

(682)	Oswal, S. L.; Desai, H. S. Studies of Viscosity and Excess Molar Volume of Binary Mixtures 3. 1-Alkanol + Di-n-Propylamine, and + Di-n-Butylamine Mixtures at 303.15 and 313.15 K. *Fluid Phase Equilib.* **2001**, *186*, 81–102. https://doi.org/10.1016/s0378-3812(01)00504-0.

(683)	Tu, C.-H.; Lee, S.-L.; Peng, I.-H. Excess Volumes and Viscosities of Binary Mixtures of Aliphatic Alcohols (C1-C4) with Nitromethane. *J. Chem. Eng. Data* **2001**, *46*, 151–155. https://doi.org/10.1021/je0002080.

(684)	Prasad, T. E. V.; Mythili, R.; Nirmala, G. S.; Prasad, D. H. L. Densities and Viscosities of the Binary Mixtures of Chloroethylenes with Some Aliphatic Alcohols. *J. Chem. Eng. Data* **2002**, *47*, 68–71. https://doi.org/10.1021/je010141w.

(685)	Belda, R.; Herraez, J. V.; Diez, O. RHEOLOGICAL STUDY AND THERMODYNAMIC ANALYSIS OF THE BINARY SYSTEM (WATER/ETHANOL): INFLUENCE OF CONCENTRATION. *Phys. Chem. Liq.* **2004**, *42*, 467–479.

(686)	Chen, H.-W.; Wen, C.-C.; Tu, C.-H. Excess Molar Volumes, Viscosities, and Refractive Indexes for Binary Mixtures of 1-Chlorobutane with Four Alcohols at T = (288.15, 298.15 and 308.15) K. *J. Chem. Eng. Data* **2004**, *49*, 347–351. https://doi.org/10.1021/je030226s.

(687)	Rodriquez, A.; Canosa, J.; Dominguez, A.; Tojo, J. Viscosities of Dimethyl Carbonate with Alcohols at Several Temperatures UNIFAC-VISCO Interaction Parameters (?OCOO?/Alcohol). *Fluid Phase Equilib.* **2004**, *216*, 167–174. https://doi.org/10.1016/j.fluid.2003.10.014.

(688)	Saleh, M. A.; Akhtar, S.; Begum, S.; Ahmed, M. S.; Begum, S. K. DENSITY AND VISCOSITY OF 1-ALKANOLS. *Phys. Chem. Liq.* **2004**, *42*, 615–623.

(689)	Calvar, N.; Dominguez, A.; Tojo, J. Vapor Liquid Equilibria for the Quaternary Reactive System Ethyl Acetate + Ethanol +water + Acetic Acid and Some of the Constituent Binary Systems at 101.3 KPa. *Fluid Phase Equilib.* **2005**, *235*, 215–222. https://doi.org/10.1016/j.fluid.2005.07.010.

(690)	Djojoputro, H.; Ismadji, S. Density and Viscosity for a Binary Mixture of Cis-3-Hexenyl Formate, Butyl Acetate, Trans-2-Hexenyl Acetate, and Cis-3-Hexenyl Acetate with Ethanol at Several Temperatures. *J. Chem. Eng. Data* **2005**, *50*, 1009–1013. https://doi.org/10.1021/je0500025.

(691)	Djojoputro, H.; Ismadji, S. Density and Viscosity of Binary Mixtures of Ethyl-2-Methylbutyrate and Ethyl Hexanoate with Methanol, Ethanol, and 1-Propanol at (293.15, 303.15, and 313.15) K. *J. Chem. Eng. Data* **2005**, *50*, 1343–1347. https://doi.org/10.1021/je0500633.

(692)	Ku, H.-C.; Tu, C.-H. Densities and Viscosities for Binary and Ternary Mixtures of Ethanol, 2-Butanone, and 2,2,4-Trimethylpentane at T = (298.15, 308.15, and 318.15) K. *J. Chem. Eng. Data* **2005**, *50*, 608–615. https://doi.org/10.1021/je049655w.

(693)	Wang, C.-C.; Chen, H.-W.; Tu, C.-H. Densities, Viscosities, and Refractive Indices for Binary and Ternary Mixtures of Ethanol, 2-Methylpropan-2-Ol, and 2,2,4-Trimethylpentane. *J. Chem. Eng. Data* **2005**, *50*, 1687–1693. https://doi.org/10.1021/je0501639.

(694)	Wankhede, N.; Lande, M. K. K.; Arbad, B. R. R. Excess Molar Volumes and Viscosity Deviations of Binary Mixtures of 2,4,6-Trimethyl-1,3,5-Trioxane + Ethanol, 1-Propanol, and 1-Butanol at (298.15, 303.15, and 308.15) K. *J. Chem. Eng. Data* **2005**, *50*, 969–972. https://doi.org/10.1021/je049556i.

(695)	Zeberg-Mikkelsen, C. K.; Baylaucq, A.; Watson, G.; Boned, C. High-Pressure Viscosity Measurements for the Ethanol + Toluene Binary System. *Int. J. Thermophys.* **2005**, *26*, 1289–1302. https://doi.org/10.1007/s10765-005-8089-2.

(696)	Alvarez, E.; Gomez-Diaz, D.; La Rubia, M. D.; Navaza, J. M. Densities and Viscosities of Aqueous Ternary Mixtures of 2-(Methylamino)Ethanol and 2-(Ethylamino)Ethanol with Diethanolamine, Triethanolamine, N-Methyldiethanolamine, or 2-Amino-1-Methyl-1-Propanol from 298.15 to 323.15 K. *J. Chem. Eng. Data* **2006**, *51*, 955–962. https://doi.org/10.1021/je050463q.

(697)	Arce, A.; Rodriguez, H.; Soto, A. Effect of Anion Fluorination in 1-Ethyl-3-Methylimidazolium as Solvent for the Liquid Extraction of Ethanol from Ethyl Tert-Butyl Ether. *Fluid Phase Equilib.* **2006**, *242*, 164–168. https://doi.org/10.1016/j.fluid.2006.01.008.

(698)	Calvar, N.; Gonzalez, B.; Dominguez, A.; Tojo, J. Physical Properties of the Ternary Mixture Ethanol+Water+1-Butyl-3-Methylimidazolium Chloride at 298.15K. *J. Solution Chem.* **2006**, *35*, 1217–1225. https://doi.org/10.1007/s10953-006-9073-6.

(699)	Chen, H.-W.; Tu, C.-H. Densities, Viscosities, and Refractive Indices for Binary and Ternary Mixtures of Diisopropyl Ether, Ethanol, and 2,2,4-Trimethylpentane. *J. Chem. Eng. Data* **2006**, *51*, 261–267. https://doi.org/10.1021/je050367p.

(700)	Gomez, E.; Gonzalez, B.; Calvar, N.; Tojo, E.; Dominguez, A. Physical Properties of Pure 1-Ethyl-3-Methylimidazolium Ethylsulfate and Its Binary Mixtures with Ethanol and Water at Several Temperatures. *J. Chem. Eng. Data* **2006**, *51*, 2096–2102. https://doi.org/10.1021/je060228n.

(701)	Kadam, U. B.; Hiray, A. P.; Sawant, A. B.; Hasan, M. Densities, Viscosities, and Ultrasonic Velocity Studies of Binary Mixtures of Trichloromethane with Methanol, Ethanol, Propan-1-Ol, and Butan-1-Ol at T=(298.15 and 308.15) K. *J. Chem. Thermodyn.* **2006**, *38*, 1675–1683. https://doi.org/10.1016/j.jct.2006.03.010.

(702)	Mutalik, V.; Manjeshwar, L. S.; Sairam, M.; Aminabhavi, T. M. Thermodynamic Properties of (Tetradecane + Benzene, + Toluene, + Chlorobenzene, + Bromobenzene, + Anisole) Binary Mixtures at T = (298.15, 303.15, and 308.15) K. *J. Chem. Thermodyn.* **2006**, *38*, 1062–1071. https://doi.org/10.1016/j.jct.2005.10.022.

(703)	Roy, M. N.; Sinha, A. Viscous Synergy and Antagonism and Isentropic Compressibility of Ternary Mixtures Containing 1,3-Dioxolane, Water and Monoalkanols at 303.15K. *Fluid Phase Equilib.* **2006**, *243*, 133–141. https://doi.org/10.1016/j.fluid.2006.02.023.

(704)	Sheu, Y.-W.; Tu, C.-H. Densities and Viscosities of Binary Mixtures of Isoamyl Acetate, Ethyl Caproate, Ethyl Benzoate, Isoamyl Butyrate, Ethyl Phenylacetate, and Ethyl Caprylate with Ethanol at T = (288.15, 298.15, 308.15, and 318.15) K. *J. Chem. Eng. Data* **2006**, *51*, 496–503. https://doi.org/10.1021/je050389b.

(705)	Zeberg-Mikkelsen, C. K.; Watson, G.; Baylaucq, A.; Galliero, G.; Boned, C. Comparative Experimental and Modeling Studies of the Viscosity Behavior of Ethanol +C7 Hydrocarbon Mixtures versus Pressure and Temperature. *Fluid Phase Equilib.* **2006**, *245*, 6–19. https://doi.org/10.1016/j.fluid.2006.01.030.

(706)	Arce, A.; Rodriquez, H.; Soto, A. Use of a Green and Cheap Ionic Liquid to Purify Gasoline Octane Boosters. *Green Chem.* **2007**, *9*, 247–253. https://doi.org/10.1039/b611139d.

(707)	Calvar, N.; Gonzalez, B.; Gomez, E.; Dominguez, A. Study of the Behaviour of the Azeotropic Mixture Ethanol Water with Imidazolium-Based Ionic Liquids. *Fluid Phase Equilib.* **2007**, *259*, 51–56. https://doi.org/10.1016/j.fluid.2007.03.021.

(708)	Calvar, N.; Gomez, E.; Gonzalez, B.; Dominguez, A. Experimental Determination, Correlation, and Prediction of Physical Properties of the Ternary Mixtures Ethanol + Water with 1-Octyl-3-Methylimidazolium Chloride and 1-Ethyl-3-Methylimidazolium Ethylsulfate. *J. Chem. Eng. Data* **2007**, *52*, 2529–2535. https://doi.org/10.1021/je700318e.

(709)	Sun, Y.; Ma, P. Densities and Viscosities for Binary Mixture of the Ethyl Crotonate with Ethanol at Different Temperatures. *Gaoxiao Huaxue Gongcheng Xuebao* **2007**, *21*, 511.

(710)	Arce, A.; Soto, A.; Ortega, J.; Sabater, G. Viscosities and Volumetric Properties of Binary and Ternary Mixtures of Tris(2-Hydroxyethyl) Methylammonium Methylsulfate + Water + Ethanol at 298.15 K. *J. Chem. Eng. Data* **2008**, *53*, 770–775. https://doi.org/10.1021/je700618y.

(711)	Awwad, A. M.; Alsyouri, H. A.; Jbara, K. A. Viscosities and Densities of Binary Mixtures of (N-Acetylmorpholine + Alkanols) from (293.15 to 323.15) K. *J. Chem. Eng. Data* **2008**, *53*, 1655–1659. https://doi.org/10.1021/je8003194.

(712)	Calvar, N.; Gonzalez, B.; Gomez, E.; Dominguez, A. Vapor Liquid Equilibria for the Ternary System Ethanol + Water + 1-Ethyl-3-Methylimidazolium Ethylsulfate and the Corresponding Binary Systems Containing the Ionic Liquid at 101.3 KPa. *J. Chem. Eng. Data* **2008**, *53*, 820–825. https://doi.org/10.1021/je700710d.

(713)	Gomez, E.; Gonzalez, B.; Calvar, N.; Dominguez, A. Excess Molar Properties of Ternary System (Ethanol + Water + 1,3-Dimethylimidazolium Methyl Sulfate) and Its Binary Mixtures at Several Temperatures. *J. Chem. Thermodyn.* **2008**, *40*, 1208–1216. https://doi.org/10.1016/j.jct.2008.04.006.

(714)	Gonzalez, B.; Calvar, N.; Gomez, E.; Macedo, E. A.; Dominguez, A. Synthesis and Physical Properties of 1-Ethyl 3-Methylpyridinium Ethylsulfate and Its Binary Mixtures with Ethanol and Water at Several Temperatures. *J. Chem. Eng. Data* **2008**, *53*, 1824–1828. https://doi.org/10.1021/je800159w.

(715)	Rehman, H.; Ansari, M. S. Density, Viscosity, and Electrical Conductivity Measurements on the Ternary System H2O + C2H5OH + LiCl over the Entire Ranges of Solvent Composition and LiCl Solubility from (-5 to +50) Deg C. *J. Chem. Eng. Data* **2008**, *53*, 2072–2088. https://doi.org/10.1021/je800150h.

(716)	Yang, C.; Sun, Y.; He, Y.; Ma, P. Volumetric Properties and Viscosities of Binary Mixtures of N,N-Dimethylformamide with Methanol and Ethanol in the Temperature Range (293.15 to 333.15) K. *J. Chem. Eng. Data* **2008**, *53*, 293–297. https://doi.org/10.1021/je700430g.

(717)	Andreatta, A. E.; Arce, A.; Rodil, E.; Soto, A. Physical Properties of Binary and Ternary Mixtures of Ethyl Acetate, Ethanol, and 1-Octyl-3-Methyl-Imidazolium Bis(Trifluoromethylsulfonyl)Imide at 298.15 K. *J. Chem. Eng. Data* **2009**, *54*, 1022. https://doi.org/10.1021/je800899w.

(718)	Domanska, U.; Laskowska, M. Temperature and Composition Dependence of the Density and Viscosity of Binary Mixtures of (1-Butyl-3-Methylimidazolium Thiocyanate + 1-Alcohols). *J. Chem. Eng. Data* **2009**, *54*, 2113–2119. https://doi.org/10.1021/je8008254.

(719)	Gonzalez, B.; Calvar, N.; Gomez, E.; Dominguez, I.; Dominguez, A. Synthesis and Physical Properties of 1-Ethylpyridinium Ethylsulfate and Its Binary Mixtures with Ethanol and 1-Propanol at Several Temperatures. *J. Chem. Eng. Data* **2009**, *54*, 1353–1358. https://doi.org/10.1021/je800981d.

(720)	Mokhtarani, B.; Sharifi, A.; Mortaheb, H. R.; Mirzaei, M.; Mafi, M.; Sadeghian, F. Density and Viscosity of 1-Butyl-3-Methylimidazolium Nitrate with Ethanol, 1-Propanol, or 1-Butanol at Several Temperatures. *J. Chem. Thermodyn.* **2009**, *41*, 1432–1438. https://doi.org/10.1016/j.jct.2009.06.023.

(721)	Roy, M. N.; Banerjee, A.; Choudhury, A. Rheological, Thermodynamic and Ultrasonic Study of Binary Mixtures Containing 2-Methoxyethanol and Some Alkan-1-Ol. *Phys. Chem. Liq.* **2009**, *47*, 412–436. https://doi.org/10.1080/00319100802087183.

(722)	Tsierkezos, N. G.; Molinou, I. E. An Investigation on Ion Association and Ion Solvation of Potassium Acetate in Alcohol Solutions. *Phys. Chem. Liq.* **2009**, *47*, 505-(-514). https://doi.org/10.1080/00319100802307672.

(723)	Wang, Y.; Lian, H.-L.; Luo, T.-L.; Liu, G.-J. Densities and Viscosities of (1,6-Hexanediamine + Ethanol) and (1,6-Hexanediamine + Ethanol + Water) Mixtures at T ) (303.15 to 328.15) K. *J. Chem. Eng. Data* **2009**, *54*, 1158–1162. https://doi.org/10.1021/je800942u.

(724)	Zarei, H. A.; Mirhidari, N.; Zangeneh, Z. Densities, Excess Molar Volumes, Viscosity, and Refractive Indices of Binary and Ternary Liquid Mixtures of Methanol (1) + Ethanol (2) + 1,2-Propanediol (3) at P = 81.5 KPa. *J. Chem. Eng. Data* **2009**, *54*, 847–854. https://doi.org/10.1021/je800633a.

(725)	Andreatta, A. E.; Arce, A.; Rodil, E.; Soto, A. Physico-Chemical Properties of Binary and Ternary Mixtures of Ethyl Acetate + Ethanol + 1-Butyl-3-Methyl-Imidazolium Bis(Trifluoromethylsulfonyl)Imide at 298.15 K and Atmospheric Pressure. *J. Solution Chem.* **2010**, *39*, 371–383.

(726)	Anouti, M.; Jacquemin, J.; Lemordant, D. Volumetric Properties, Viscosities, and Isobaric Heat Capacities of Imidazolium Octanoate Protic Ionic Liquid in Molecular Solvents. *J. Chem. Eng. Data* **2010**, *55*, 5719–5728. https://doi.org/10.1021/je100671v.

(727)	Bhattacharjee, A.; Roy, M. N. Study on Molecular Interactions by Antagonism, Synergy and Excess Functions of Ternary Mixtures of Tetrahydrofuran with Dimethylsulphoxide and Monoalkanols. *Phys. Chem. Liq.* **2010**, *48*, 618–637. https://doi.org/10.1080/00319100903350167.

(728)	Calvar, N.; Gomez, E.; Gonzalez, B.; Dominguez, A. Experimental Vapor-Liquid Equilibria for the Ternary System Ethanol + Water + 1-Ethyl-3-Methylpyridinium Ethylsulfate and the Corresponding Binary Systems at 101.3 KPa: Study of the Effect of the Cation. *J. Chem. Eng. Data* **2010**, *55*, 2786–2791. https://doi.org/10.1021/je900998f.

(729)	Goncalves, F. A. M. M.; Trindade, A. R.; Costa, C. S. M. F.; Bernardo, J. C. S.; Johnson, I.; Fonseca, I. M. A.; Ferreira, A. G. M. PVT, Viscosity, and Surface Tension of Ethanol: New Measurements and Literature Data Evaluation. *J. Chem. Thermodyn.* **2010**, *42*, 1039–1049. https://doi.org/10.1016/j.jct.2010.03.022.

(730)	Hou, C.; Jiang, Z.; Ren, B. Density and Viscosity of Clopidogrel Hydrogen Sulfate + Methanol and Clopidogrel Hydrogen Sulfate + Ethanol from (278.15 to 313.15) K. *J. Chem. Eng. Data* **2010**, *55*, 4943–4945. https://doi.org/10.1021/je1000796.

(731)	Rilo, E.; Vila, J.; Garcia, M.; Varela, L. M.; Cabeza, O. Viscosity and Electrical Conductivity of Binary Mixtures of CnMIM-BF4 with Ethanol at 288 K, 298 K, 308 K, and 318 K. *J. Chem. Eng. Data* **2010**, *55*, 5156–5163. https://doi.org/10.1021/je100687x.

(732)	Garcia-Abuin, A.; Gomez-Diaz, D.; La Rubia, M. D.; Navaza, J. M. Density, Speed of Sound, Viscosity, Refractive Index, and Excess Volume of N-Methyl-2-Pyrrolidone + Ethanol (or Water or Ethanolamine) from T = (293.15 to 323.15) K. *J. Chem. Eng. Data* **2011**, *56*, 646–651. https://doi.org/10.1021/je100967k.

(733)	Hasan, M.; Hiray, A. P.; Kadam, U. B.; Shirude, D. F.; Kurhe, K. J.; Sawant, A. B. Densities, Viscosities, Speeds of Sound, FT-IR and 1H-NMR Studies of Binary Mixtures of n-Butyl Acetate with Ethanol, Propan-1-Ol, Butan-1-Ol and Pentan-1-Ol at 298.15, 303.15, 308.15 and 313.15 K. *J. Solution Chem.* **2011**, *40*, 415–429.

(734)	Kurnia, K. A.; Mutalib, M. I. A. Densities and Viscosities of Binary Mixture of the Ionic Liquid Bis(2-Hydroxyethyl)Ammonium Propionate with Methanol, Ethanol, and 1-Propanol at T = (293.15, 303.15, 313.15, and 323.15) K and at P = 0.1 MPa. *J. Chem. Eng. Data* **2011**, *56*, 79–83. https://doi.org/10.1021/je100848r.

(735)	Liu, J.; Zhu, C.; Ma, Y. Densities and Viscosities of Binary Solutions of Benzene-1,3-Diol + Water, Ethanol, Propan-1-Ol, and Butan-1-Ol at T = (293.15 to 333.15) K. *J. Chem. Eng. Data* **2011**, *56*, 2095–2099. https://doi.org/10.1021/je101173w.

(736)	Trenzado, J. L.; Romano, E.; Segade, L.; Caro, M. N.; Gonzalez, E.; Galvan, S. Densities and Viscosities of Four Binary Diethyl Carbonate + 1-Alcohol Systems from (288.15 to 313.15) K. *J. Chem. Eng. Data* **2011**, *56*, 2841–2848. https://doi.org/10.1021/je1013476.

(737)	Blanco, A.; Garcia-Abuin, A.; Gomez-Diaz, D.; Navaza, J. M. Density, Speed of Sound, Viscosity, Refractive Index, and Excess Volume of N-Methyl-2-Pyrrolidone (NMP) + Water + Ethanol from T = (293.15 to 323.15) K. *J. Chem. Eng. Data* **2012**, *57*, 1009–1014. https://doi.org/10.1021/je201152j.

(738)	Chen, K.-D.; Lin, Y.-F.; Tu, C.-H. Densities, Viscosities, Refractive Indexes, and Surface Tensions for Mixtures of Ethanol, Benzyl Acetate, and Benzyl Alcohol. *J. Chem. Eng. Data* **2012**, *57*, 1118–1127. https://doi.org/10.1021/je201009c.

(739)	Garcia-Mardones, M.; Gascon, I.; Lopez, M. C.; Royo, F. M.; Lafuente, C. Viscosimetric Study of Binary Mixtures Containing Pyridinium-Based Ionic Liquids and Alkanols. *J. Chem. Eng. Data* **2012**, *57*, 3549–3556. https://doi.org/10.1021/je300557g.

(740)	Gong, Y. -h.; Shen, C.; Lu, Y.-Z.; Meng, H.; Li, C.-X. Viscosity and Density Measurement for Six Binary Mixtures of Water (Methanol or Ethanol) with an Ionic Liquid [BMIM][DMP] or [EMIM][DMP] at Atmospheric Pressure in the Temperature Range of (293.15 to 333.15) K. *J. Chem. Eng. Data* **2012**, *57*, 33–39. https://doi.org/10.1021/je200600p.

(741)	Han, S.; Zhu, C.; Ma, Y. Densities and Viscosities of Naphthalen-1-Ol, Naphthalen-2-Ol, and 1-Aminonaphthalene in the Solvents of Ethanol, Propan-1-Ol, and Butan-1-Ol. *J. Chem. Eng. Data* **2012**, *57*, 1396–1403. https://doi.org/10.1021/je201222n.

(742)	Quijada, E.; Boogaart, S. v. d.; Lijbers, J. H.; Meindersma, G. W.; Haan, A. B. d. Experimental Densities, Dynamic Viscosities and Surface Tensions of the Ionic Liquids Series 1-Ethyl-3-Methylimizazolium Acetate and Dicyanamide and Their Binary and Ternary Mixtures with Water and Ethanol at T = (298.15 K to 343.15 K). *J. Chem. Thermodyn.* **2012**, *51*, 51–58. https://doi.org/10.1016/j.jct.2012.02.027.

(743)	Shao, D.; Lu, X.; Fang, W.; Yongsheng, G.; Xu, L. Densities and Viscosities for Binary Mixtures of the Ionic Liquid N-Ethyl Piperazinium Propionate with n-Alcohols at Several Temperatures. *J. Chem. Eng. Data* **2012**, *57*, 937–942. https://doi.org/10.1021/je201116t.

(744)	Sreekanth, K.; Kondaiah, M.; Kumar, D. S.; Rao, D. K. Influence of Temperature on Thermodynamic Properties of Acid Base Liquid Mixtures. *J. Therm. Anal. Calorim.* **2012**, *110*, 1341–1352. https://doi.org/10.1007/s10973-011-2131-3.

(745)	Xu, Y. J.; Yao, J.; Wang, C.; Li, H. Density, Viscosity, and Refractive Index Properties for the Binary Mixtures of n-Butylammonium Acetate Ionic Liquid + Alkanols at Several Temperatures. *J. Chem. Eng. Data* **2012**, *57*, 298–308. https://doi.org/10.1021/je200707b.

(746)	Yao, H.; Zhang, S.; wang, J.; Zhou, Q.; Dong, H.; Zhang, X. Densities and Viscosities of the Binary Mixtures of 1-Ethyl-3-Methylimidazolium Bis(Trifluoromethylsulfonyl)Imide with N-Methyl-2-Pyrrolidone or Ethanol at T = (293.15 to 323.15) K. *J. Chem. Eng. Data* **2012**, *57*, 875–881. https://doi.org/10.1021/je200922s.

(747)	Caro, M. N.; Trenzado, J. L.; Galvan, S.; Romano, E.; Gonzalez, E.; Alcalde, R.; Aparicio, S. Densities and Viscosities of Three Binary Monoglyme + 1-Alcohol Systems from (283.15 to 313.15) K. *J. Chem. Eng. Data* **2013**, *58*, 909–914. https://doi.org/10.1021/je301095j.

(748)	Faria, M. A. F.; Martins, R. J.; Cardoso, M. J. E. M.; Barcia, O. E. Density and Viscosity of the Binary Systems Ethanol + Butan-1-Ol, + Pentan-1-Ol, + Heptan-1-Ol, + Octan-1-Ol, Nonan-1-Ol, + Decan-1-Ol at 0.1 MPa and Temperatures from 283.15 K to 313.15 K. *J. Chem. Eng. Data* **2013**, *58*, 3405–3419. https://doi.org/10.1021/je400630f.

(749)	Kondaiah, M.; Sreekanth, K.; Kumar, D. S.; Rao, D. K. Volumetric and Viscometric Properties of Propanoic Acid in Equimolar Mixtures of N,N-Dimethyl Formamide + Alkanols at T/K 5 303.15, 313.15, and 323.15. *J. Solution Chem.* **2013**, *42*, 494–515. https://doi.org/10.1007/s10953-012-9898-0.

(750)	Xu, Y.; Chen, B.; Qian, W.; Li, H. Properties of Pure N-Butylammonium Nitrate Ionic Liquid and Its Binary Mixtures of with Alcohols at T = (293.15 to 313.15) K. *J. Chem. Thermodyn.* **2013**, *58*, 449–459. https://doi.org/10.1016/j.jct.2012.10.008.

(751)	Yang, Q.; Yu, K.; Xing, H.; Su, B.; Bao, Z.; Yang, Y.; Ren, Q. The Effect of Molecular Solvents on the Viscosity, Conductivity and Ionicity of Mixtures Containing Chloride Anion-Based Ionic Liquid. *J. Ind. Eng. Chem. (Amsterdam, Neth.)* **2013**, *19*, 1708–1714. https://doi.org/10.1016/j.jiec.2013.02.010.

(752)	Castro, M. C.; Rodriguez, H.; Arce, A.; Soto, A. Mixtures of Ethanol and the Ionic Liquid 1-Ethyl-3- Methylimidazolium Acetate for the Fractionated Solubility of Biopolymers of Lignocellulosic Biomass. *Ind. Eng. Chem. Res.* **2014**, *53*, 11850–11861. https://doi.org/10.1021/ie501956x.

(753)	Florido, P. M.; Andrade, I. M. G.; Capellini, M. C.; Carvalho, F. H.; Aracava, K. K.; Koshima, C. C.; Rodrigues, C. E. C.; Goncalves, C. B. Viscosities and Densities of Systems Involved in the Deterpenation of Essential Oils by Liquid-Liquid Extraction: New UNIFAC-VISCO Parameters. *J. Chem. Thermodyn.* **2014**, *72*, 152–160. https://doi.org/10.1016/j.jct.2013.11.026.

(754)	Goncalves, D.; Koshima, C. C.; Nakamoto, K. T.; Umeda, T. K.; Aracava, K. K.; Goncalves, C. B.; Rodrigues, C. E. d. C. Deterpenation of Eucalyptus Essential Oil by Liquid + Liquid Extraction: Phase Equilibrium and Physical Properties for Model Systems at T = 298.2 K. *J. Chem. Thermodyn.* **2014**, *69*, 66–72. https://doi.org/10.1016/j.jct.2013.09.044.

(755)	Moosavi, M.; Omrani, A.; Rostami, A. A.; Motahari, A. Isobaric, Isothermal Theoretical Investigation and Examination of Different Prediction Equations on Some Physicochemical Properties in PEG Liquid Polymer System. *J. Chem. Thermodyn.* **2014**, *68*, 205–215. https://doi.org/10.1016/j.jct.2013.09.006.

(756)	Zhu, C.; Han, S.; Liu, J.; Ma, Y. Densities and Viscosities of Diaminotoluene with Water, Ethanol, Propan-1-Ol, and Butan-1-Ol from (293.15 to 333.15) K. *J. Chem. Eng. Data* **2014**, *59*, 880–889. https://doi.org/10.1021/je400968v.

(757)	Cao, Q.; Lu, X.; Wu, X.; Guo, Y.; Xu, L.; Fang, W. Density, Viscosity, and Conductivity of Binary Mixtures of the Ionic Liquid N-(2-Hydroxyethyl)Piperazinium Propionate with Water, Methanol, or Ethanol. *J. Chem. Eng. Data* **2015**, *60*, 455–463. https://doi.org/10.1021/je500380x.

(758)	Salinas, R.; Pla-Franco, J.; Lladosa, E.; Monton, J. Density, Speed of Sound, Viscosity, and Excess Properties of Binary Mixtures Formed by Ethanol and Bis(Trifluorosulfonyl)Imide-Based Ionic Liquids. *J. Chem. Eng. Data* **2015**, *60*, 525–540. https://doi.org/10.1021/je500594z.

(759)	Reid, R. C.; Belenyessy, L. I. Viscosity of Polar Vapor Mixtures. *J. Chem. Eng. Data* **1960**, *5*, 150–151. https://doi.org/10.1021/je60006a007.

(760)	Thausen, E. Uber Die Innere Reibung von Gasgemischen. *Ann. Phys. (Leipzig)* **1911**, *36*, 815.

(761)	Trautz, M.; Stauf, F. W. Viscosity, Heat Conductivity and Diffusion in Gaseous Mixtures. III. Viscosity of H2-C3H4 Mixtures. *Ann. Phys. (Leipzig)* **1929**, *2*, 737.

(762)	Trautz, M.; Zink, R. Viscosity, Thermal Conductivity, and Diffusion in Mas Mixutres. XII. Gas Viscosity at High Temperatures. *Ann. Phys. (Berlin)* **1930**, *7*, 427.

(763)	Comings, E. W.; Mayland, B. J.; Egly, R. S. *Viscosity of Gases at High Pressures*; 42; Bull.-Ill., Univ., Eng. Exp. Stn.: 1, 1944.

(764)	Golubev, I. F.; Petrov, V. A. *Viscosity of Gases and Gaseous Mixtures*; 1953.

(765)	Misic, D.; Thodos, G. The Thermal Conductivity of Hydrocarbon Gases at Normal Pressures. *AIChE J.* **1961**, *7*, 264.

(766)	Vaschenko, D. M.; Voinov, Y.; Voityuk, B. V.; Dregulyas, E. K.; Kolomiets, A. Y.; Labinov, S. D.; Morozov, A. A.; Neduzhil, I. A.; Provotar, V. P.; Soldatenko, Yu. A.; Storozhenko, E. I.; Khmara, Yu. I. *Thermodynamic and Transport Properties of Ethylene and Propylene*; 1971.

(767)	Maass, O.; Boomer, E. H. Vapor Densities at Low Pressures and over an Extended Temperature Range: I the Properties of Ethylene Oxide Compared to Oxygen Compounds of Similar Molecular Weight. *J. Am. Chem. Soc.* **1922**, *44*, 1709–1728. https://doi.org/10.1021/ja01429a013.

(768)	Senftleben, H. *Z. Angew. Phys.* **1953**, *5*, 33.

(769)	Zhou, F.; Pan, Y.; Zhu, B. Ethylene Oxide Synthesis in Three-Phase Slurry Reactor under Pressure V. Determination and Correlation of Physical Property Data of Solvent No. 2. *Huadong Huagong Xueyuan Xuebao* **2000**, *26*, 581–583.

(770)	Stephan, K.; Heckenberger, T. Viscosity Data on Pure Compounds.

(771)	Maczynska, Z.; Maczynski, A. Floppy Book on Viscosity of Liquids, Version 1991.04.13. **1991**.

(772)	Hersh, C. K.; Gaynor, A. J. Symposium on Advanced Propellant Chemistry. 149th National Meeting ACS, Vol. 9 No. 1. *AIChE-Meeting* **1966**, 128–135.

(773)	Wayaku, M. Vapor Pressure–Temperature Equation of Hydrocarbon Compounds. *Aromatikkusu* **1974**, *26*, 541–553.

(774)	Elverum, G.; Doescher, R. N. Physical Properties of Liquid Fluorine. *J. Chem. Phys.* **1952**, *20*, 1834.

(775)	Kanda, E. Studies on Fluorine at Low Temperatures. VIII. Determination of Molecular Heat, Heat of Fusion of Condensed Fluorine and the Entropy of Fluorine. *Bull. Chem. Soc. Jpn.* **1937**, *12*, 511.

(776)	Franck, E. U.; Stoeber, W. Viscosity and Molecular Effect Diameter of Fluorine. *Z. Naturforsch. Sec. A* **1952**, *7*, 822–823. https://doi.org/10.1515/zna-1952-1210.

(777)	Rankine, A. O.; Smith, J. O. The Viscosities and Molecular Dimensions of Methane, Sulphuretted Hydrogen and Cyanogen. *Philos. Mag.* **1921**, *42*, 615.

(778)	Bhattacharyya, P. K. Viscosity of Binary Polar-Gas Mixtures: CH3Cl - H2S and CH3Cl - SO2. *J. Chem. Phys.* **1970**, *53*, 893–895. https://doi.org/10.1063/1.1674154.

(779)	Pal, A. K.; Bhattacharyya, P. K. Viscosity of Binary Polar-Gas Mixtures. *J. Chem. Phys.* **1969**, *51*, 828–831. https://doi.org/10.1063/1.1672075.

(780)	Bhattacharyya, P. K.; Ghosi, A. K. Viscosity of Polar - Quadrupolar Gas Mixtures. *J. Chem. Phys.* **1970**, *52*, 2719–2723.

(781)	Pal, A. K.; Barua, A. K. Viscosity of Hydrogen Nitrogen and Hydrogen Ammonia Gas Mixtures. *J. Chem. Phys.* **1967**, *47*, 216–218. https://doi.org/10.1063/1.1711848.

(782)	Monteil, J. M.; Lazarre, F.; Salvinien, J.; Viallet, P. Viscometer for the Study of Relative Viscosity of H2S. *J. Chim. Phys. Phys. Chim. Biol.* **1969**, *66*, 1673–1675. https://doi.org/10.1051/jcp/196966s21673.

(783)	Hennel, J. W.; Krynicki, K. Viscosity of Liquid Hydrogen Sulfide. *Acta Phys. Pol.* **1959**, *19*, 523–526.

(784)	Jung, G.; Schmick, H. Der Einfluss Der Molekularen Anziehungskraefte Auf Die Innere Reibung von Gasgemischen. *Z. Phys. Chem. Abt. B* **1930**, *7*, 130–147.

(785)	Steele, B. D.; McIntosh, D.; Archibald, E. H. The Vapor Pressures, Densities, Surface Energies and Viscosities of Pure Solvents. *Z. Phys. Chem., Stoechiom. Verwandschaftsl.* **1906**, *55*, 129–150.

(786)	Runovskaya, I. V.; Zorin, A. D.; Devyatykh, G. G. Viscosity of Condensed Volatile Inorganic Hydrides of Group III- VI Elements. *Zh. Neorg. Khim.* **1970**, *15*, 2581.

(787)	Vogel, E. Reference Viscosities of Gaseous Methane and Hydrogen Sulfide at Low Density in the Temperature Range from (292 to 682) K. *J. Chem. Eng. Data* **2011**, *56*, 3265–3272. https://doi.org/10.1021/je200371n.

(788)	Harle, H. On the Viscosities of the Hydrogen Halides. *Proc. Roy. Soc. London Ser. A* **1922**, *100*, 429–440. https://doi.org/10.1098/rspa.1922.0008.

(789)	Trautz, M.; Narath, A. Die Innere Reibung von Gasgemischen. *Ann. Phys. (Leipzig)* **1926**, *79*, 637.

(790)	Krynicki, K.; Hennel, J. W. VISCOSITY OF LIQUID AMMONIA AND HYDROGEN CHLORIDE. *Acta Phys. Pol.* **1963**, *24*, 269–271.

(791)	Zinovieva, K. N. Viscosity of Liquid He-3 in the Interval 0.35 - 3.2 K and He-4 above the Lambda-Point. *Zh. Eksp. Teor. Fiz.* **1958**, *34*, 609–615.

(792)	Luker, J. A.; Johnson, C. A. Viscosity of Helium, Oxygen, Helium-Oxygen, Helium-Steam, and Oxygen-Steam Mixtures at High Temperatures and Pressures. *J. Chem. Eng. Data* **1959**, *4*, 176–182. https://doi.org/10.1021/je60002a019.

(793)	Kao, J. T.; Kobayashi, R. Viscosity of Helium and Nitrogen and Their Mixtures at Low Temperatures and Elevated Pressures. *J. Chem. Phys.* **1967**, *47*, 2836–2849.

(794)	Tsederberg, N. V.; Popov, V. N.; Panchenko, S. S. Experimental Study of the Viscosity of Helium at Temperatures from 80 to 273 K and at Pressures to 40 MPa. *Teploenergetika* **1974**, 78–79.

(795)	Tanaka, Y.; Nakajima, M.; Kubota, H.; Makita, T. Viscosity of SF6 + N2 and SF6 + He Gaseous Mixtures. *J. Chem. Eng. Jpn.* **1980**, *13*, 155–159.

(796)	Reynes, E. G.; Thodos, G. Viscosity of Helium, Neon, and Nitrogen in the Dense Gaseous Region. *J. Chem. Eng. Data* **1966**, *11*, 137–140. https://doi.org/10.1021/je60029a001.

(797)	Rudenko, N. S.; Karnus, A. I. Viscosity of He4 in the Temperature Range from 14 to 31 K and at Pressures to 100 Atm. *Ukr. Fiz. Zh.* **1968**, *13*, 1334–1337.

(798)	Seibt, D.; Herrmann, S.; Vogel, E.; Bich, E.; Hassel, E. Simultaneous Measurements on Helium and Nitrogen with a Newly Designed Viscometer-Densimeter over a Wide Range of Temperature and Pressure. *J. Chem. Eng. Data* **2009**, *54*, 2626–2637. https://doi.org/10.1021/je900131q.

(799)	Albright, L. F.; Lohrenz, J. Viscosity of Liquids. *AIChE J.* **1956**, *2*, 290–295.

(800)	Ledneva, T. M. Bulk Viscosity and Its Temperature Dependence for Some Organc Liquids. *Vestn. Mosk. Univ.* **1956**, *2*, 49–60.

(801)	Abdulagatov, I. M.; Rasulov, S. M. Viscosity of Binary N-Pentane (0.5)-n-Heptane (0.5) System and Its Pure Components near the Liquid-Gas Critical Points. *Teplofiz. Vys. Temp.* **1992**, *30*, 501–508.

(802)	Abdulagatov, I. M.; Rasulov, S. M. Anomaly of Viscosity of the N-Heptane near the Liquid-Gas Critical Point. *Zh. Fiz. Khim.* **1996**, *70*, 2187–2190.

(803)	Smyth, C. P.; Stoops, W. N. The Dielectric Polarization of Liquids III. The Polarization of the Isomers of Heptane. *J. Am. Chem. Soc.* **1928**, *50*, 1883–1890. https://doi.org/10.1021/ja01394a008.

(804)	Fawcett, F. S. Boiling Points of Three Isomeric Heptanes. *Ind. Eng. Chem.* **1946**, *38*, 338–340. https://doi.org/10.1021/ie50435a026.

(805)	Giller, E. B.; Drickamer, H. G. Viscosity of Normal Paraffins near the Freezing Point. *Ind. Eng. Chem.* **1949**, *41*, 2067.

(806)	Koshkin, N. I.; Nozdrev, V. F. Study of Ultrasound Attenuation for a Series of Saturated Hydrocarbons by the Pulse Method. *Dokl. Akad. Nauk* **1953**, *92*, 793–796.

(807)	Zhigunov, I. S.; Ampelogova, N. I. Some Properties of Nitrogen-Containing Solvents and Their Binary Mixtures II. Properties of n-Butylamine + Water System. *Fiz. Khim. Rastvorov* **1972**, 181.

(808)	Badalov, Y. A.; Naziev, Y. M.; Guseinov, S. O. Viscosity of Liquids of Binary Mixtures of N-Heptane and Benzene at High Pressures. *Izv. Vyssh. Uchebn. Zaved., Neft Gaz* **1975**, *18*, 67–70.

(809)	Kessel’man, P. M.; Porichanskii, E. G.; Karbanov, E. M. Study of Dynamic Viscosity Coefficients for Freons in the Wide Temperature and Pressure Interval. *Kholod. Tekh. Tekhnol.* **1976**, *22*, 48–50.

(810)	Gunchuk, B. V.; Karbanov, E. M.; Lapardin, N. I.; Zakharzhevskiy, V. Y. Investigation of the Viscosity of Some Freons Along the Saturated Line. In *Teplofiz. Svoistva Veshchestv. Mater.*; No. 11, Rabinovich, V. A., Ed., Standards Publ.: Moscow, 1977; pp 39–46.

(811)	Rappon, M.; Kaukinen, J. A. MOLECULAR ASSOCIATION OF PENTANOLS IN N-HEPTANE II : VISCOSITIES AS A FUNCTION OF TEMPERATURE COVERING LOW CONCENTRATION RANGE. *J. Mol. Liq.* **1988**, *38*, 107–133. https://doi.org/10.1016/0167-7322(88)80011-4.

(812)	Kanti, M.; Lagourette, B.; Alliez, J.; Boned, C. Viscosity of Binary Heptane-Nonylbenzene as a Function of Pressure and Temperature: Application of Flory’s Theory. *Fluid Phase Equilib.* **1991**, *65*, 291–304. https://doi.org/10.1016/0378-3812(91)87031-4.

(813)	Assael, M. J.; Oliveira, C.; Papadaki, M.; Wakeham, W. A. Vibrating-Wire Viscometers for Liquids at High Pressures. *Int. J. Thermophys.* **1992**, *13*, 593–615. https://doi.org/10.1007/bf00501943.

(814)	Blanco, A. M.; Ortega, J.; Garcia, B.; Leal, J. M. Studies on Densities and Viscosities of Binary Mixtures of Alkyl Benzoates in N-Heptane. *Thermochim. Acta* **1993**, *222*, 127–136. https://doi.org/10.1016/0040-6031(93)80546-m.

(815)	Aminabhavi, T. M.; Aralaguppi, M. I.; Gopalakrishna, B.; Khinnavar, R. S. Densities, Shear Viscosities, Refractive Indices, and Speeds of Sound of Bis(2-Methoxyethyl) Ether with Hexane, Heptane, Octane, and 2,2,4- Trimethylpentane in the Temperature Interval 298.15-318.15. *J. Chem. Eng. Data* **1994**, *39*, 522–528. https://doi.org/10.1021/je00015a028.

(816)	Sastry, N. V.; Raj, M. M. Densities, Speeds of Sound, Viscosities, Dielectric Constants, and Refractive Indices for 1-Heptanol + Hexane and +Heptane at 303.15 and 313.1 5 K. *J. Chem. Eng. Data* **1996**, *41*, 612–618. https://doi.org/10.1021/je950172p.

(817)	Sastry, N. V.; Valand, M. K. Densities, Speeds of Sound, Viscosities, and Relative Permittivities for 1- Propanol + and 1-Butanol + Heptane at 298.15 K and 308.15 K. *J. Chem. Eng. Data* **1996**, *41*, 1421–1425. https://doi.org/10.1021/je960135d.

(818)	Will, S.; Leipertz, A. Viscosity of Liquid N-Heptane by Dynamic Light Scattering. *Int. J. Thermophys.* **1997**, *18*, 1339–1354. https://doi.org/10.1007/bf02575338.

(819)	Jimenez, E.; Franjo, C.; Segade, L.; Legido, J. L.; Andrade, M. I. P. Viscosities and Densities for the 1-Propanol + n -Heptane System at Several Temperatures. *J. Solution Chem.* **1998**, *27*, 569–579.

(820)	Matos, J. S.; Trenzado, J. L.; Gonzalez, E.; Alcalde, R. Volumetric Properties and Viscosities of the Methyl Butanoate + N-Heptane + n-Octane Ternary System and Its Binary Constituents in the Temperature Range from 283.15 to 313.15 K. *Fluid Phase Equilib.* **2001**, *186*, 207–234. https://doi.org/10.1016/s0378-3812(01)00511-8.

(821)	Ma, P.; Jin, H.; Li, N.; Yang, C. Densities and Viscosities for Binary Mixture of the Styrene with N-Octane at Different Temperatures. *Tianjin Daxue Xuebao* **2005**, *38*, 5;356.

(822)	Pensado, A. S.; Comunas, M. J. P.; Lugo, L.; Fernandez, J. Experimental Dynamic Viscosities of 2,3-Dimethylpentane up to 60 MPa and from (303.15 to 353.15) K Using a Rolling-Ball Viscometer. *J. Chem. Eng. Data* **2005**, *50*, 849–855. https://doi.org/10.1021/je049662k.

(823)	Abdulagatov, I. M.; Zeinalova, A. B.; Azizov, N. D. Viscosity of Aqueous Electrolyte Solutions at High Temperatures and High Pressures. Viscosity B-Coefficient. Sodium Iodide. *J. Chem. Eng. Data* **2006**, *51*, 1645–1659. https://doi.org/10.1021/je060124c.

(824)	Landaverde-Cortes, D. C.; Estrada-Baltazar, A.; Iglesias-Silva, G. A.; Hall, K. R. Densities and Viscosities of MTBE + Heptane or Octane at p ) 0.1 MPa from (273.15 to 363.15) K. *J. Chem. Eng. Data* **2007**, *52*, 1226–1232. https://doi.org/10.1021/je600554h.

(825)	Wang, Z. F.; Wang, L.-S.; Fan, T.-B. Densities and Viscosities of Ternary Mixtures of Heptane, Octane, Nonane, and Hexyl Benzene from 293.15 K to 313.15 K. *J. Chem. Eng. Data* **2007**, *52*, 1866–1871. https://doi.org/10.1021/je700202h.

(826)	El-Sayed, H. E. M.; Asfour, A.-F. A. Viscometric and Volumetric Properties of 10 Regular Binary Systems at 308.15 K and 313.15 K. *Int. J. Thermophys.* **2009**, *30*, 1773–1790. https://doi.org/10.1007/s10765-009-0667-2.

(827)	Sagdeev, D.; Fomina, M.; Mukhamedzyanov, G.; Abdulagatov, I. M. Experimental Study of the Density and Viscosity of N-Heptane at Temperatures from 298 K to 470 K and Pressure up to 245 MPa. *Int. J. Thermophys.* **2013**, *34*, 1–33. https://doi.org/10.1007/s10765-012-1373-z.

(828)	Luning Prak, D.; Cowart, J.; Trulove, P. Density, Viscosity, Speed of Sound, Bulk Modulus, and Surface Tension of Binary Mixtures of n-Heptane + 2,2,4-Trimethylpentane at (293.15 to 338.15) K and 0.1 MPa. *J. Chem. Eng. Data* **2014**, *59*, 3842–3851. https://doi.org/10.1021/je5007532.

(829)	Melaven, R. M.; Mack, E. Jr. The Collision Areas and Shapes of Carbon Chain Molecules in the Gaseous State: Normal-Heptane, Normal-Octane, Normal-Nonane. *J. Am. Chem. Soc.* **1932**, *54*, 888–904. https://doi.org/10.1021/ja01342a007.

(830)	Agaev, N. A.; Golubev, I. F. Viscosity of N-Pentane in the Liquid and Gaseous State at High Pressures and Different Temperatures. *Gaz. Prom.* **1963**, 45–50.

(831)	Carmichael, L. T.; Reamer, H. H.; Sage, B. H. Thermal Conductivity of Fluids. Methane. *J. Chem. Eng. Data* **1966**, *11*, 52–57. https://doi.org/10.1021/je60028a014.

(832)	Diaz Pena, M.; Cheda, J. A. R. Viscosidad de Hidrocarburos. II. n-Heptano y Sus Isomeros. *An. Quim.* **1974**, *70*, 107.

(833)	Vogel, E.; Holdt, B. Temperature and Initial-Density Dependence of the Vapour Phase Viscosity of n-Alkanes (n = 5- 7). *High Temp. - High Pressures* **1991**, *23*, 473–483.

(834)	Abdulagatov, I. M.; Rasulov, S. M. Viscosity of N-Pentane, N-Heptane and Their Mixtures within the Temperature Range from 298K up to Critical Points at the Saturation Vapor Pressure. *Ber. Bunsen-Ges. Phys. Chem.* **1996**, *100*, 148–154.

(835)	Andrade, E. N. d. C.; Rotherham, L. On the Determination of Viscosity by the Oscillation of a Vessel Enclosing a Fluid: Part 2. *Proc. Phys. Soc.* **1936**, *48*, 261–266. https://doi.org/10.1088/0959-5309/48/2/303.

(836)	Grunberg, L. Viscosity of Regular Solutions Systems Involving Carbon Tetrachloride, Benzene, and Cyclohexane. *Trans. Faraday Soc.* **1954**, *50*, 1293.

(837)	Parisot, P. E.; Johnson, E. F. Liquid Viscosity above the Normal Boiling Point. *J. Chem. Eng. Data* **1961**, *6*, 263–267. https://doi.org/10.1021/je60010a024.

(838)	Naziev, Y. M.; Guseinov, S. O.; Akhmedov, A. K. Experimental Study of the Viscosity of Some Hydrocarbons at Various Pressures and Temperatures. *Izv. Vyssh. Uchebn. Zaved., Neft Gaz* **1972**, *15*, 65–68.

(839)	Strumpf, H. J.; Collings, A. F.; Pings, C. J. Viscosity of Xenon and Ethane in the Critical Region. *J. Chem. Phys.* **1974**, *60*, 3109–3123.

(840)	Bulanov, N. V.; Skripov, V. P. Measurements of the Viscosity of Overheated Liquids at High Pressures. *Inzh.-Fiz. Zh.* **1975**, *29*, 1074–1078.

(841)	Craubner, H. Densitometer for Absolute Meas. of the Temperature Dependence of Den- Sity, Partial Volumes, and Thermal Expansivity of Solids and Liquids. *Rev. Sci. Instrum.* **1986**, *57*, 2817.

(842)	Hamelin, J.; Mehl, J. B.; Moldover, M. R. Resonators for Accurate Dielectric Measurements in Conducting Liquids. *Rev. Sci. Instrum.* **1998**, *69* (1), 255–260. https://doi.org/10.1063/1.1148505.

(843)	Grigor’ev, B. A.; Keramidi, A. S.; Rodchenko, S. I.; Grachev, V. K. Viscosity of Normal Hexane at Low Temperature. *Izv. Vyssh. Uchebn. Zaved., Neft Gaz* **1988**, *31*, 53.

(844)	Oswal, S. L.; Dave, J. P. Viscosities of Nonelectrolyte Liquid Mixtures. II. Binary Mixtures of n- Hexane with Alkanoates and Bromoalkanoates. *Int. J. Thermophys.* **1992**, *13*, 943–955. https://doi.org/10.1007/bf01141208.

(845)	Franjo, C.; Menaut, C. P.; Jimenez Cuesta, E.; Legido, J. L.; Paz Andrade, M. I. Viscosities and Densities of Octane + Butan-1-Ol, Hexan-1-Ol, and Octan-1- Ol at 298.15 K. *J. Chem. Eng. Data* **1995**, *40*, 992–994. https://doi.org/10.1021/je00020a058.

(846)	Dominguez, M.; Pardo, J.; Lopez, M. C.; Royo, F. M.; Urieta, J. S. Viscosities of the Ternary Mixture (1-Butanol + n-Hexane + 1-Butylamine) at the Temperatures 298.15 and 313.15 K. *Fluid Phase Equilib.* **1996**, *124*, 147–159. https://doi.org/10.1016/s0378-3812(96)03070-1.

(847)	Dominguez, M.; Santafe, J.; Lopez, M. C.; Royo, F. M.; Urieta, J. S. Viscosities of the Ternary Mixture (1-Butanol + n-Hexane + 1-Chiorobutane) at 298.15 K and 313.15 K. *Fluid Phase Equilib.* **1998**, *152*, 133–148. https://doi.org/10.1016/s0378-3812(98)00377-x.

(848)	Dominguez, M.; Pardo, J. I.; Gascon, I.; Royo, F. M.; Urieta, J. S. Viscosities of the Ternary Mixture (2-Butanol + n-Hexane + 1-Butylamine) at 298.15 and 313.15 K. *Fluid Phase Equilib.* **2000**, *169*, 277–292. https://doi.org/10.1016/s0378-3812(00)00332-0.

(849)	Gascon, I.; Pardo, J.; Santafe, J.; Dominguez, M.; Urieta, J. S. Experimental and Predicted Viscosities of the Binary System (n-Hexane + 1,3-Dioxolane) and for the Ternary System (n-Hexane + 1,2-Dioxolane + 1-Butanol) at 298.15 and 313.15 K. *Fluid Phase Equilib.* **2001**, *180*, 211–220. https://doi.org/10.1016/s0378-3812(01)00348-x.

(850)	Comelli, F.; Ottani, S.; Francesconi, R.; Castellari, C. Densities, Viscosities, and Refractive Indices of Binary Mixtures Containing n-Hexane + Components of Pine Resins and Essential Oils at 298.15 K. *J. Chem. Eng. Data* **2002**, *47*, 93–97. https://doi.org/10.1021/je010216w.

(851)	Bolotnikov, M. F.; Neruchev, Yu. A. Viscosities and Densities of Binary Mixtures of Hexane with 1-Chlorohexane between 293.15 K and 333.15 K. *J. Chem. Eng. Data* **2003**, *48*, 739–741. https://doi.org/10.1021/je034002l.

(852)	Dominguez, M.; Langa, E.; Mainar, A. M.; Santafe, J.; Urieta, J. S. Measurements, Correlations, and Predictions of Viscosities for the Ternary Mixture (2-Butanol + Hexane + 1-Chlorobutane) at 298.15 K and 313.15 K. *J. Chem. Eng. Data* **2003**, *48*, 302–307. https://doi.org/10.1021/je020114l.

(853)	Gascon, I.; Villares, A.; Haro, M.; Martin, S.; Artigas, H. Experimental and Predicted Viscosities of the Ternary Mixture (Hexane + 1,3-Dioxolane + 2-Butanol) at 298.15 and 313.15 K. *J. Chem. Eng. Data* **2005**, *50*, 722–726. https://doi.org/10.1021/je049576k.

(854)	Modarress, H.; Mohsen-Nia, M. Experimental and Theoretical Studies of Viscosities of Ternary Mixture [2-PropanolQethyl AcetateQn-Hexane] and Its Binary Constituents at 298.15, 308.15 and 313.15K. *Phys. Chem. Liq.* **2006**, *44*, 67–76.

(855)	Carrasco, A.; Perez-Navarro, M.; Gascon, I.; Lopez, M. C.; Lafuente, C. Densities and Viscosities of the Ternary Mixtures 2-Methyl-1-Propanol (or 2-Methyl-2-Propanol) + N-Hexane + 1-Chlorobutane at 298.15 K. *J. Chem. Eng. Data* **2008**, *53*, 1223–1227. https://doi.org/10.1021/je800048f.

(856)	McCoubrey, J. C.; McCrea, J. N.; Ubbelohde, A. R. The Configuration of Flexible Polymethylene Molecules in the Gas Phase. *J. Chem. Soc.* **1951**, 1961.

(857)	Schuler; Michel. *Z. Naturforsch., A: Astrophys. Phys. Phys. Chem.* **1956**, *11*, 403.

(858)	Grachev, V. K.; Grigor’ev, B. A.; Keramidi, A. S. Study of the Viscosity of Normal Hexane at High Teperatures. *Izv. Vyssh. Uchebn. Zaved., Neft Gaz* **1989**, 41–44.

(859)	van Itterbeek, A.; van Paemel, O. Measurements on the Viscosity of Gases for Low Pressures at Room Temperature and at Low Temperatures. *Physica Amsterdam* **1940**, *7*, 273–283. https://doi.org/10.1016/S0031-8914(40)90116-1.

(860)	Diller, D. E. Measurements of the Viscosity of Parahydrogen. *J. Chem. Phys.* **1965**, *42*, 2089.

(861)	Breitenbach, P. On the Viscosity of Gases and Their Change with Temperature (in German). *Ann. Phys. (Leipzig)* **1901**, *5*, 166–169.

(862)	Verschaffelt, J. E.; Nicaise, C. The Viscosity of Liquefied Gases. IX. Preliminary Determination of the Viscosity of Liquid Hydrogen. *Comm. Phys. Lab. Univ. Leiden* **1917**, 66–71.

(863)	Yen, K. An Absolute Determination of the Coefficients of Viscosity of Hydrogen, Nitrogen and Oxygen. *Philos. Mag. Ser. 6* **1919**, *38*, 582.

(864)	Klein, S. A.; McLinden, M. O.; Laesecke, A. An Improved Extended Corresponding States Method for Estimation of Viscosity of Pure Refrigerants and Mixtures. *Int. J. Refrig.* **1997**, *20* (3), 208–217. https://doi.org/10.1016/S0140-7007(96)00073-4.

(865)	Trautz, M.; Baumann. Viscosity, Thermal COnductivey and Diffusion in Gas Mixtures II. The Viscosity of H2-N2 and H2-CO Mixtures. *Ann. Phys. (Leipzig)* **1929**, *2*, 733.

(866)	Trautz, M.; Ludewigs, W. The Viscosity, Thermal Conductivity and Diffusion in Gas Mixtures. VI. Viscosity Determinations on Pure Gases by Direct Measurement and by the Same Means on Their Mixtures (in German). *Ann. Phys. (Leipzig)* **1929**, *3(5)*, 409–428.

(867)	Trautz, M.; Kurtz, F. Viscosity, Thermal Conductivity, and Diffusion in Gas Mixtures. *Ann. Phys. (Berlin)* **1931**, *9*, 981.

(868)	Sutherland, B. P.; Maass, O. Measurement of the Viscosity of Gases over a Large Temperature Range. *Can. J. Res.* **1932**, *6*, 428.

(869)	Trautz, M.; Zimmermann, H. Viscosity, Thermal Conductivity and Diffusion in Gas Mixtures. XXX. Viscosity at Low Temperatures of Hydrogen, Helium and Neon and Their Binary Mixtures Down to 90 Deg Abs. *Ann. Phys. (Berlin)* **1935**, *22*, 189–194.

(870)	Johns, H. E. The Viscosity of Liquid Hydrogen. *Can. J. Res. A* **1939**, *17*, 221–226.

(871)	Johnston, H. L.; McCloskey, K. E. Viscosities of Several Common Gases Between 90 K and Room Temperature. *J. Phys. Chem.* **1940**, *44*, 1038–1058.

(872)	Buddenberg, J. W.; Wilke, C. R. Viscosities of Some Mixed Gases. *J. Phys. Colloid Chem.* **1951**, *55*, 1491–1498.

(873)	Kuss, E. Viscosity of Compressed Gases. *Z. Angew. Phys.* **1952**, *4*, 203.

(874)	Kompaneets, V. Y. Experimental Investigation of the Viscosity of Gases and Gaseous Mixtures at High Temperatures. *Sb. Nauchn. Rab., Leningr. Inst. Mekhan. Selsk. Khoz.* **1953**, 113.

(875)	Kestin, J.; Pilarczyk, K. Measurement of the Viscosity of Five Gases at Elevated Pressures by the Oscillating-Disk Method. *Trans. Am. Soc. Mech. Eng.* **1954**, *76*, 987–999.

(876)	Rietveld, A., A. O. ,. van Itterbeek. Viscosity of Mixtures of H(2) and HD between 300 and 14 Degrees K. *Comm. Phys. Lab. Univ. Leiden* **1957**, 1–5.

(877)	Kestin, J.; Wang, H. E. The Viscosity of Five Gases: A Re-Evaluation. *Trans. Am. Soc. Mech. Eng.* **1958**, *80*, 11.

(878)	Golubev, I. F.; Shepeleva, R. I. Viscosity of Hydrogen at Low Temperatures and High Pressures (in Russian). *Gaz. Prom.* **1966**, *11*, 54–58.

(879)	Gracki, J. A.; Flynn, G. P.; Ross, J.-J. Viscosity of Nitrogen, Helium, Hydrogen, and Argon from -100 to 25 C up 150 - 200 Atm. *J. Chem. Phys.* **1969**, *51*, 3856.

(880)	Guevara, F. A.; McInter, B. B.; Ottesen, D. High-Temperature Viscosity Ratios for Hydrogen, Helium, Argon, and Nitrogen. *Phys. Fluids* **1969**, *12*, 2493.

(881)	Chuang, S.-. y; Chappelear, P. S.; Kobayashi, R. Viscosity of Methane, Hydrogen, and Four Mixtures of Methane and Hydrogen from -100 C to 0 C at High Pressures. *J. Chem. Eng. Data* **1976**, *21*, 403–411. https://doi.org/10.1021/je60071a010.

(882)	Clifford, A. A.; Kestin, J.; Wakeham, W. A. The Viscosity of Mixtures of Hydrogen with Three Noble Gases. *Ber. Bunsen-Ges. Phys. Chem.* **1981**, *85*, 385–388.

(883)	Händel, G.; Kleinrahm, R.; Wagner, W. Measurements of the (Pressure, Density, Temperature) Relation of Methane in the Homogeneous Gas and Liquid Regions in the Temperature Range from 100 K to 260 K and at Pressures up to 8 MPa. *J. Chem. Thermodyn.* **1992**, *24* (7), 685–695. https://doi.org/10.1016/S0021-9614(05)80076-X.

(884)	Nabizadeh, H.; Mayinger, F. Viscosity of Gaseous R404A, R407C, R410A, and R507. *Int. J. Thermophys.* **1999**, *20* (3), 777–790.

(885)	Boyd, J. H. The Viscosity of Compressed Gases. *Phys. Rev.* **1930**, *35*, 1284.

(886)	Keesom, W. H.; MacWood, G. E. The Viscosity of Hydrogen Vapour. *Comm. Phys. Lab. Univ. Leiden* **1938**, 1–4.

(887)	Keesom, W. H.; Keesom, P. H. The Viscosity of Hydrogen Vapour. *Comm. Phys. Lab. Univ. Leiden* **1940**, 1–4.

(888)	Tsederberg, N. V.; Popov, V. N.; Andreev, I. I. Experimental Study of the Viscosity of Hydrogen. *Teploenergetika* **1965**, 84–86.

(889)	Yusibani, E.; Nagahama, Y.; Kohno, M.; Takata, Y.; Woodfield, P.; Shinzato, K.; Fujii, M. A Capillary Tube Viscometer Designed for Measurements of Hydrogen Gas Viscosity at High Pressure and High Temperature. *Int. J. Thermophys.* **2011**, *32*, 1111–1124. https://doi.org/10.1007/s10765-011-0999-6.

(890)	Garner, J. B.; Adams, L.; Stuchell, R. M. Physical and Chemical Properties of Isobutylene. *Pet. Refiner* **1945**, *24*, 99–102.

(891)	Tatevskii, V. M.; Grikina, O. E.; Abramenkov, A. V.; Tkachik, Z. A. Relationship between the Viscosity of Liquids and the Chemical Structure of Their Molecules. *Russ. J. Phys. Chem.* **1985**, *59*, 1643–1644.

(892)	Rappenecker, K. On the Viscosity Coefficient of Gases and Their Temperature Dependence. *Z. Phys. Chem., Stoechiom. Verwandschaftsl.* **1910**, *72*, 695.

(893)	Bratton, . J., A. C.; Felsing, W. A. A High-Efficiency Method Applicable to Certain Classes of Compounds-. *Ind. Eng. Chem.* **1936**, *28*, 424–430.

(894)	Maman, A. Octanes. *C. R. Hebd. Seances Acad. Sci.* **1938**, *207*, 1401–1402.

(895)	Johnson, G. C.; Fawcett, F. S. Inter-Polymerization of Isobutane and 2-Methyl-2-Butene Using an Alumina-Silica Catalyst. Compostion of the Hydrogenated Nonanes. *J. Am. Chem. Soc.* **1946**, *68*, 1416–1419. https://doi.org/10.1021/ja01212a005.

(896)	Dymond, J. H.; Glen, N. F.; Isdale, J. D. Transport Properties of Nonelectrolyte Liquid Mixtures–VII. Visocity Coeff. for Isooctane and for Equimolar Mixtures of Isooctane + n-Octane and Isooctane + n-Dodecane from 25 to 100 Deg. C at Pr. *Int. J. Thermophys.* **1985**, *6*, 233–250. https://doi.org/10.1007/bf00522146.

(897)	Ma, P.; Li, N. Measurement and Correlation of Densities and Viscosities for Correlative Systems of Dimethyl Carbonate. *Tianjin Daxue Xuebao* **2005**, *38*, 577.

(898)	Salas, J. A.; Pedrosa, G. C.; Acevedo, I. L.; Arancibia, E. L. Excess Molar Volumes and Excess Viscosities of the 2,2,4-Trimethylpentane (1) + 3-Methyl-1-Butanol (2) and Propan-2-Ol (3) Ternary System at 298.15 K. *J. Mol. Liq.* **2006**, *124*, 37–44.

(899)	Ku, H.-C.; Wang, C.-C.; Tu, C.-H. Densities, Viscosities, Refractive Indexes, and Surface Tensions for Binary and Ternary Mixtures of Tetrahydofuran, 2-Propanol, and 2,2,4-Trimethylpentane. *J. Chem. Eng. Data* **2008**, *53*, 566–573. https://doi.org/10.1021/je700626v.

(900)	Ku, H.-C.; Wang, C.-C.; Tu, C.-H. Densities, Viscosities, Refractive Indices, and Surface Tensions for the Mixtures of 1,3-Dioxolane + 2-Propanol or + 2,2,4-Trimethylpentane at (288.15, 298.15, and 308.15) K and 1,3-Dioxolane + 2-Propanol + 2,2,4-Trimethylpentane at 298.15 K. *J. Chem. Eng. Data* **2009**, *54*, 131–136. https://doi.org/10.1021/je800664z.

(901)	Kao, Y.-C.; Tu, C.-H. Densities, Viscosities, Refractive Indices, and Surface Tensions for Binary and Ternary Mixtures of 2-Propanol, Tetrahydropyran, and 2,2,4-Trimethylpentane. *J. Chem. Thermodyn.* **2011**, *43*, 216–226. https://doi.org/10.1016/j.jct.2010.08.019.

(902)	Rafiee, H. R.; Ranjbar, S.; Poursalman, F. Densities and Viscosities of Binary and Ternary Mixtures of Cyclohexanone, 1,4-Dioxane and Isooctane from T = (288.15 to 313.15) K. *J. Chem. Thermodyn.* **2012**, *54*, 266–271. https://doi.org/10.1016/j.jct.2012.05.005.

(903)	Yue, L.; Qin, X.; Wu, X.; Xu, L.; Guo, Y.; Fang, W. Excess Molar Volume along with Viscosity, Refractive Index and Relative Permittivity for Binary Mixtures of Exo-Tetrahydrodicyclopentadiene with Four Octane Isomers. *J. Chem. Thermodyn.* **2015**, *81*, 26–33. https://doi.org/10.1016/j.jct.2014.09.015.

(904)	Diaz Pena, M.; Cabello, A.; Cheda, J. A. R. Viscosidad Del N-Octano e Isomeros En Fase de Vapor. *An. Quim.* **1975**, *71*, 637.

(905)	Lesche, H.; Klemp, D.; Nickel, B. Dichte Und Viskositat Des Isopentans von 115 K Bis 293 K. *Z. Phys. Chem. (Leipzig)* **1984**, *141*, 239.

(906)	Bleakney, J. W. Measurement on the Vapor Viscosities of the Two Common Pentanes, Two Pentanes and Carbon Tetrachloride. *Physica (The Hague)* **1932**, *3*, 123.

(907)	Day, R. Variation of the Vapor Viscosities of Pentane and Isopentane with Pressure by Rotating Cylynder Method. *Phys. Rev.* **1932**, *40*, 281–290.

(908)	McCoubrey, J. C.; Singh, N. M. The Vapor Phase Viscosities of the Pentanes. *J. Phys. Chem.* **1963**, *67*, 517.

(909)	Kumagai, A.; Tomida, D.; Yokoyama, C. Measurements of the Liquid Viscosities of Mixtures of Isobutane with Squalane to 30 MPa. *Int. J. Thermophys.* **2007**, *28*, 1111–1119. https://doi.org/10.1007/s10765-007-0259-y.

(910)	Gonzalez, M. H.; Lee, A. L. Viscosity of Isobutane. *J. Chem. Eng. Data* **1966**, *11*, 357–359. https://doi.org/10.1021/je60030a019.

(911)	Agaev, N. A.; Yusibova, A. D. Viscosity of Isobutane at High Pressures. *Gaz. Prom.* **1969**, *14*, 41.

(912)	Kuechenmeister, C.; Vogel, E. The Viscosity of Gaseous Isobutane and Its Initial Density Dependence. *Int. J. Thermophys.* **2000**, *21*, 329. https://doi.org/10.1023/a:1006671226710.

(913)	Ulybin, S. A.; Makarushkin, V. I.; Skorodumov, S. V. Viscosity of Krypton at Temperatures from 120-1300 K and at Pressures up to 100 Mpa. *Teplofiz. Vys. Temp.* **1978**, *16*, 282–287.

(914)	Golik, O. Z.; Cholpan, P. P. Molecular Structure, Compressibility, Surface Tension, and Viscosity of Some Polysiloxanes. *Ukr. Fiz. Zh.* **1960**, *5*, 242–251.

(915)	Sperkach, V. S.; Cholpan, P. F. Study of Acoustic Scattering in Some Siloxanes. *Fiz. Zhidk. Sostoyaniya* **1979**, 104–109.

(916)	Matthews, J. B.; Sumner, J. F.; Moelwyn-Hughes, E. A. The Vapour Pressures of Certain Liquids. *Trans. Faraday Soc.* **1950**, *46*, 797–803.

(917)	Anonymous, X. The Physical and Chemical Properties for Devulcanized Solution. *Chem. Eng. Oil Gas* **1981**, 1–24.

(918)	Song, J.-H.; Park, S.-B.; Yoon, J.-H.; Lee, H.; Lee, K.-H. Densities and Viscosities of Monoethanolamine + Ethylene Glycol + Water. *J. Chem. Eng. Data* **1996**, *41*, 1152–1154. https://doi.org/10.1021/je9601366.

(919)	Lee, M. J.; Lin, T.-K.; Pai, Y.-H.; Lin, K.-S. Density and Viscosity for Monoethanolamine + 1-Propanol, + 1-Hexanol, and + 1-Octanol. *J. Chem. Eng. Data* **1997**, *42*, 854–857. https://doi.org/10.1021/je9700636.

(920)	Maham, Y.; Liew, C. N.; Mather, A. E. Viscosities and Excess Properties of Aqueous Solutions of Ethanolamines from 25 to 80deg C. *J. Solution Chem.* **2002**, *31*, 743–756.

(921)	Islam, M. N.; Islam, M. M.; Yeasmin, M. N. Viscosity of Aqueous Solutions of 2-Methoxyethanol, 2-Ethoxyethanol, and Ethanolamine. *J. Chem. Thermodyn.* **2004**, *36*, 889–893. https://doi.org/10.1016/j.jct.2004.06.004.

(922)	Amundsen, T. G.; Oi, L. E.; Eimer, D. A. Density and Viscosity of Monoethanolamine + Water + Carbon Dioxide from (25 to 80) Deg C. *J. Chem. Eng. Data* **2009**, *54*, 3096–3100. https://doi.org/10.1021/je900188m.

(923)	Song, H.-J.; Lee, M.-G.; Kim, H.; Gaur, A.; Park, J.-W. Density, Viscosity, Heat Capacity, Surface Tension, and Solubility of CO2 in Aqueous Solutions of Potassium Serinate. *J. Chem. Eng. Data* **2011**, *56*, 1371–1377. https://doi.org/10.1021/je101144k.

(924)	Tian, S.; Ren, S.; Hou, Y.; Wu, W.; Peng, W. Densities, Viscosities and Excess Properties of Binary Mixtures of 1,1,3,3-Tetramethylguanidinium Lactate + Water at T = (303.15 to 328.15) K. *J. Chem. Eng. Data* **2013**, *58*, 1885–1892. https://doi.org/10.1021/je3009073.

(925)	Shaikh, M. S.; Shariff, A. M.; Bustam, M. A.; Murshid, G. Physicochemical Properties of Aqueous Solutions of Sodium L-Prolinate as an Absorbent for CO2 Removal. *J. Chem. Eng. Data* **2014**, *59*, 362–368. https://doi.org/10.1021/je400830w.

(926)	Xu, F.; Gao, H.; Dong, H.; Wang, Z.; Zhang, X.; Ren, B.; Zhang, S. Solubility of CO2 in Aqueous Mixtures of Monoethanolamine and Dicyanamide-Based Ionic Liquids. *Fluid Phase Equilib.* **2014**, *365*, 80–87. https://doi.org/10.1016/j.fluid.2013.12.020.

(927)	Shen, S.; Yang, Y. -n.; Wang, Y.; Ren, S.; Han, J.; Chen, A. CO2 Absorption into Aqueous Potassium Salts of Lysine and Proline: Density, Viscosity and Solubility of CO2. *Fluid Phase Equilib.* **2015**, *399*, 40–49. https://doi.org/10.1016/j.fluid.2015.04.021.

(928)	Rudenko, N. S.; Shubnikov, L. V. Variation in the Viscosity of Liquid Methane and Ethylene with Temperature. *Phys. Z. Sowjetunion* **1935**, *8*, 179.

(929)	Rudenko, N. S. Viscosity of the Liquids O(2), N(2), CH(4), C(2)H(4) and Air (in Russian). *Zh. Eksp. Teor. Fiz.* **1939**, *9*, 1078.

(930)	Bresler, S. E.; Landerman, A. Viscosity of Liquid Methane and Deuteriomethane. *Zh. Eksp. Teor. Fiz.* **1940**, *10*, 250.

(931)	Gerf, S. F.; Galkov, G. I. Viscosity of Liquid Pure Gases and Their Mixtures. *Zh. Tekh. Fiz.* **1941**, *11*, 801–808.

(932)	Mescheriakov, N. V.; Golubev, I. F. *Viazkost’ Uglevodorodnykh Gazov Pri Vysokikh Davleniakh,*; 1954.

(933)	Ross, I. F.; Brown, G. M. Viscosities of Gases at High Pressures. *Ind. Eng. Chem.* **1957**, *49*, 2026.

(934)	Pavlovich, N. V. Study of the Thermal Conductivity of Natural Gases and Methane. *Gaz. Prom.* **1959**, 45–49.

(935)	Iwasaki, M.; Takahashi, H. The Viscosity of Methane at High Pressures. *Kogyo Kagaku Zasshi* **1959**, *62*, 918.

(936)	Carmichael, L. T.; Berry, V. M.; Sage, B. H. Viscosity of Hydrocarbons. Methane. *J. Chem. Eng. Data* **1965**, *10*, 57–61. https://doi.org/10.1021/je60024a020.

(937)	Huang, E. T. S.; Swift, G. W.; Kurata, F. Viscosities of Methane and Propane at Low Temperatures and High Pressures. *AIChE J.* **1966**, *12*, 932–936.

(938)	Hellemans, J. M.; Kestin, J.; Ro, S. T. Viscosity of Methane, Carbon Tetrafluoride and Sulfur Hexafluoride over a Range of Temperatures. *Physica (Amsterdam)* **1973**, *65*, 376.

(939)	Swift, G. W.; Christy, J. A.; Kurata, F. Liquid Viscosities of Methane and Propane. *AIChE J.* **1959**, *5*, 98–102.

(940)	Swift, G. W.; Lohrenz, J.; Kurata, F. Liquid Viscosities above the Normal Boiling Point for Methane, Ethane, Propane, and n-Butane. *AIChE J.* **1960**, *6*, 415–419.

(941)	Giddings, J. G.; Kao, J. T.; Kobayashi, R. Development of a High Pressure Capillary-Tube Viscometer and Its Application to Methane, Propane, and Their Mixtures in the Gaseous and Liquid Regions. *J. Chem. Phys.* **1966**, *45*, 578.

(942)	Hongo, M.; Yokoyama, C.; Takahashi, S. Viscosity of Methane-Chlorodiflouromethane (R22) Gaseous Mixtures in the Temperature Range from 298.15 to 373.15 K and at Pressures up to 5 MPa. *J. Chem. Eng. Jpn.* **1988**, *21*, 632–638.

(943)	vanderGulik, P. S.; Mostert, R.; vandenBerg, H. R. The Viscosity of Methane at 273 K up to 1 GPa. *Fluid Phase Equilib.* **1992**, *79*, 301–311. https://doi.org/10.1016/0378-3812(92)85139-y.

(944)	Jones, G.; Fornwalt, H. J. The Influence of Surface Tension on the Measurement of Viscosity The Viscosity of Methanol. *J. Am. Chem. Soc.* **1938**, *60*, 1683–1693. https://doi.org/10.1021/ja01274a043.

(945)	Sadek, H.; Fuoss, R. M. Electrolyte-Solvent Interaction I. Tetrabutylammonium Bromide in Methanol- Nitrobenzene Mixtures. *J. Am. Chem. Soc.* **1950**, *72*, 301–306. https://doi.org/10.1021/ja01157a080.

(946)	Mikhail, S. Z.; Kimel, W. R. Densities and Viscosities of Methanol-Water Mixtures. *J. Chem. Eng. Data* **1961**, *6*, 533–537. https://doi.org/10.1021/je60011a015.

(947)	Isakova, N. P.; Oshueva, L. A. Viscosity of Liquid Metyhanol at High Pressures. *Zh. Fiz. Khim.* **1966**, *40*, 1130–1131.

(948)	Kubota, H.; Tsuda, S.; Murata, M.; Yamamoto, T.; Tanaka, Y.; Makita, T. Specific Volume and Viscosity of Methanol-Water Mixtures under High Pressure. *Rev. Phys. Chem. Jpn.* **1979**, *49*, 59–69.

(949)	Singh, R. P.; Haque, M. M. Viscosity Variations in Associated Liquid Systems Showing Partial Miscibility: Part I-Kinematic Viscosities of Methanol-n-Butanol-Water Ternary System of Different Compositions. *Indian J. Chem., Sect. A: Inorg., Phys., Theor. Anal.* **1981**, *20*, 874–878.

(950)	Isdale, J. D.; Easteal, A. J.; Woolf, L. A. Shear Viscosity of Methanol and Methanol + Water Mixtures Under Pressure. *Int. J. Thermophys.* **1985**, *6*, 439–450. https://doi.org/10.1007/bf00508889.

(951)	Liu, H.; Yu, D.; Feng, G.; Yang, X.; Nie, Q. Measurement and Correlation of the Viscosities of Water-Methanol-Lithium Chloride System. *Tianranqi Huagong* **1994**, *19*, 52–56.

(952)	Reddy, V. K.; Rambabu, K.; Devarajulu, T.; Krishnaiah, A. Volume of Mixing, Speed of Sound, and Viscosity of Methyl Cellosolve with Aliphatic Alcohols at 308.15 K. *J. Chem. Eng. Data* **1995**, *40*, 124–127. https://doi.org/10.1021/je00017a028.

(953)	Saha, N.; Das, B.; Hazra, D. K. Viscosities and Excess Molar Volumes for Acetonitrile + Methanol at 298.15, 308.15, and 318.15 K. *J. Chem. Eng. Data* **1995**, *40*, 1264–1266. https://doi.org/10.1021/je00022a026.

(954)	Sastry, N. V.; Raj, M. M. Excess Molar Volumes and Isentropic Compressibilities of Binary Mixtures Containing Methyl Methacrylate-n-Alcohols at 303.15 and 313.15 K. *Thermochim. Acta* **1995**, *257*, 39–50. https://doi.org/10.1016/0040-6031(94)02207-5.

(955)	Muhuri, P. K.; Das, B.; Hazra, D. K. Apparent Molar Volumes and Apparent Molar Adiabatic Compressibilities of Some Symmetrical Tetraalkylammonium Bromides in 1,2-Dimethoxyethane. *Indian J. Chem., Sect. A: Inorg., Phys., Theor. Anal.* **1996**, *35*, 288–293.

(956)	Bakshi, M. S.; Kaur, G. Thermodynamic Behavior of Mixtures. 4. Mixtures of Methanol with Pyridine and N,N-Dimethylformamide at 25 C. *J. Chem. Eng. Data* **1997**, *42*, 298–300. https://doi.org/10.1021/je960300p.

(957)	George, M. A.; Young, K. M.; Robertson III, E. A.; Beck, S. E.; Voloshin, G. Vapor Pressure and Viscosity of 1,1,1,5,5,5-Hexafluoro-2,4-Pentanedione. *J. Chem. Eng. Data* **1998**, *43*, 60–64. https://doi.org/10.1021/je9700941.

(958)	Rodriguez, A.; Canosa, J.; Tojo, J. Dynamic Viscosities of the Ternary Liquid Mixture Involving CH3COOCH3, CH3OH, and CH3CH2CH2OH at T = 298.15 K. *J. Chem. Thermodyn.* **1998**, *30*, 1307–1318. https://doi.org/10.1006/jcht.1998.0396.

(959)	Sastry, N. V.; Valand, M. K. Densities, Viscosities, and Relative Permittivities for Pentane + 1-Alcohols (C1 to C12) at 298.15 K. *J. Chem. Eng. Data* **1998**, *43*, 152–157. https://doi.org/10.1021/je9701801.

(960)	Canosa, J.; Rodriguez, A.; Tojo, J. Dynamic Viscosities of the Binary Mixtures (Methyl Acetate or Methanol + 2-Methyl-2-Butanol) and the Ternary Mixtures (Methyl Acetate + Methanol + 2-Propanol, or 2-Butanol, or 2-Methyl-2-Butanol) at T=298.15 K. *J. Chem. Thermodyn.* **2000**, *32*, 551–565. https://doi.org/10.1006/jcht.2000.0628.

(961)	Pal, A.; Dass, G. Excess Molar Volumes and Viscosities for Binary Liquid Mixtures of 2-Propoxyethanol and of 2-Isopropoxyethanol with Methanol, 1-Propanol, 2-Propanol, and 1-Pentanol at 298.15 K. *J. Chem. Eng. Data* **2000**, *45*, 693–698. https://doi.org/10.1021/je0000120.

(962)	Prasad, T. E. V.; Agrawal, S. B.; Bajaj, A. B.; Prasad, D. H. L. DENSITY AND VISCOSITY OF METHANOL + TRICHLOROETHYLENE, n-PROP ANOL + TRICHLOROETHYLENE AND n-BUTANOL + TRICHLOROETHYLENE MIXTURES. *Phys. Chem. Liq.* **2000**, *38*, 433–438.

(963)	Prasad, T. E. V.; Vallabhaneni, G. K.; Sameera, M. K.; Bose, V. P.; Prasad, D. H. L. Density and Viscosity of the Binary Mixtures Formed by P-Xylene with Methanol, n-Propanol and n-Butanol. *Phys. Chem. Liq.* **2000**, *38*, 475–480.

(964)	Saha, N.; Das, B. Apparent Molar Volumes of Some Symmetrical Tetraalkylammonium Bromides in Methanol + Acetonitrile Mixtures at 298.15 K. *J. Mol. Liq.* **2000**, *89*, 169–174.

(965)	Visak, Z. P.; Ferreira, A. G. M.; Fonseca, I. M. A. Densities and Viscosities of the Ternary Mixtures Water + Butyl Acetate + Methanol and Water + Ethyl Propionate + Methanol at 303.15 K. *J. Chem. Eng. Data* **2000**, *45*, 296–931. https://doi.org/10.1021/je000085j.

(966)	George, J.; Sastry, N. V.; Patel, S. R.; Valand, M. K. Densities, Viscosities, Speeds of Sound, and Relative Permittivities for Methyl Acrylate + 1-Alcohols (C1-C6) at T = (308.15 and 318.15) K. *J. Chem. Eng. Data* **2002**, *47*, 262–269. https://doi.org/10.1021/je010268l.

(967)	Heintz, A.; Klasen, D.; Lehmann, J. K. Excess Molar Volumes and Viscosities of Binary Mixtures of Methanol and the Ionic Liquid 4-Methyl-N-Butylpyridinium Tetrafluoroborate at 25, 40, and 50 Deg C. *J. Solution Chem.* **2002**, *31*, 467–476.

(968)	Giro, F.; Goncalves, M.; Ferreira, A. G. M.; Fonseca, I. M. A. Viscosity and Density Data of the System Water + N-Pentyl Acetate + Methanol Calculations with a Modified Redlich-Kwong-Soave Equation of State. *Fluid Phase Equilib.* **2003**, *204*, 217–232. https://doi.org/10.1016/s0378-3812(02)00262-5.

(969)	Kijevcanin, M. L.; Ribeiro, I. S. A.; Ferreira, A. G. M.; Fonseca, I. M. A. Densities, Viscosities, and Surface and Interfacial Tensions of the Ternary Mixture Water + Ethyl Butyrate + Methanol at 303.15 K. *J. Chem. Eng. Data* **2003**, *48*, 1266–1270. https://doi.org/10.1021/je0301500.

(970)	Chatterjee, A.; Das, B. Electrical Conductances of Tetrabutylammonium Bromide, Sodium Tetraphenylborate, and Sodium Bromide in Methanol (1) + Water (2) Mixtures at (298.15, 308.15, and 318.15) K. *J. Chem. Eng. Data* **2006**, *51*, 1352–1355. https://doi.org/10.1021/je0600810.

(971)	Zafarani-Moattar, M. T.; Majdan-Cegincara, R. Viscosity, Density, Speed of Sound, and Refractive Index of Binary Mixtures of Organic Solvent + Ionic Liquid, 1-Butyl-3-Methylimidazolium Hexafluorophosphate at 298.15 K. *J. Chem. Eng. Data* **2007**, *52*, 2359–2364. https://doi.org/10.1021/je700338t.

(972)	Pal, A.; Gaba, R. Volumetric, Acoustic, and Viscometric Studies of Molecular Interactions in Binary Mixtures of Dipropylene Glycol Dimethyl Ether with 1-Alkanols at 298.15 K. *J. Chem. Thermodyn.* **2008**, *40*, 818–828. https://doi.org/10.1016/j.jct.2008.01.008.

(973)	Davila, M. J.; Aparicio, S.; Alcalde, R. Thermophysical Properties of Binary and Ternary Mixtures Containing Lactams and Methanol. *Ind. Eng. Chem. Res.* **2009**, *48*, 10065–10076. https://doi.org/10.1021/ie900822e.

(974)	Jain, D. M.; Shah, V.; Rabadiya, S.; Oswal, S. Viscosity and Excess Molar Volume of Binary Mixtures of Methanol with N-Butylamine and Di-n-Butylamine at 303.15, 313.15 and 323.15 K. Characterization in Terms of ERAS Model. *J. Mol. Liq.* **2009**, *144*, 65–70.

(975)	Roy, M. N.; Dewan, R. K.; Sarkar, L. Ion-Solvent Interactions of Some Halides of Common Cations with Organic Solvent Mixtures by Conductometric, Volumetric, Viscometric, and Refractometric Techniques. *J. Chem. Eng. Data* **2010**, *55*, 1347–1353. https://doi.org/10.1021/je900656c.

(976)	Shekaari, H.; Mousavi, S. S. Volumetric Properties of Ionic Liquid 1,3-Dimethylimidazolium Methyl Sulfate + Molecular Solvents at T = (298.15 - 328.15) K. *Fluid Phase Equilib.* **2010**, *291*, 201–207. https://doi.org/10.1016/j.fluid.2010.01.002.

(977)	Pal, A.; Gaba, R.; Kumar, H. Acoustic, Viscometric, and Spectroscopic Studies of Dipropylene Glycol Monopropyl Ether with n-Alkanols at Temperatures of 288.15, 298.15, and 308.15 K. *J. Solution Chem.* **2011**, *40*, 786–802. https://doi.org/10.1007/s10953-011-9688-0.

(978)	Wang, L.-S.; Tian, N.-N. Densities and Viscosities of Binary Mixtures of Methanol with Dimethyl Methylphosphonate and Dimethyl Phosphite from (293.15 to 333.15) K. *Int. J. Thermophys.* **2011**, *32*, 1175–1187. https://doi.org/10.1007/s10765-011-0989-8.

(979)	Warrier, P.; Teja, A. S. Density, Viscosity, and Thermal Conductivity of Mixtures of 1-Ethoxy-1,1,2,2,3,3,4,4,4-Nonafluorobutane (HFE 7200) with Methanol and 1-Ethoxybutane. *J. Chem. Eng. Data* **2011**, *56*, 4291–4294. https://doi.org/10.1021/je1011828.

(980)	Yu, Z.; Gao, H.; Wang, H.; Chen, L. Densities, Viscosities, and Refractive Properties of the Binary Mixtures of the Amino Acid Ionic Liquid [Bmim][Ala] with Methanol or Benzylalcohol at T = (298.15 to 313.15) K. *J. Chem. Eng. Data* **2011**, *56*, 2877–2883. https://doi.org/10.1021/je2000606.

(981)	Marczak, W.; Adamczyk, N.; Lezniak, M. Viscosity of Associated Mixtures Approximated by the Grunberg-Nissan Model. *Int. J. Thermophys.* **2012**, *33*, 680–691. https://doi.org/10.1007/s10765-011-1100-1.

(982)	Qian, W.; Xu, Y. J.; Zhu, H.; Yu, C. Properties of Pure 1-Methylimidazolium Acetate Ionic Liquid and Its Binary Mixtures with Alcohols. *J. Chem. Thermodyn.* **2012**, *49*, 87–94. https://doi.org/10.1016/j.jct.2012.01.013.

(983)	Singh, S.; Yasmin, M.; Shukla, D.; Parveen, S.; Gupta, M. Thermoacoustical Study of Molecular Interactions in Binary Mixtures of Pentane with Methanol and Tert-Butanol. *Phys. Chem. Liq.* **2012**, *50*, 210–221. https://doi.org/10.1080/00319104.2011.597030.

(984)	Yu, Z.; Gao, H.; Wang, H.; Chen, L. Thermodynamic Properties of Binary Mixtures of the Amino Acid Ionic Liquids [Bmim][Glu] or [Bmim][Gly] with Methanol at T = 298.15 to 313.15 K. *J. Solution Chem.* **2012**, *41*, 173–186. https://doi.org/10.1007/s10953-011-9788-x.

(985)	Brahman, D.; Sinha, B. Effect of Tetrabutylammonium Bromide on Solution Behavior of Salicylaldehyde Anil Zinc (II) in Methanol at T = (298.15, 308.15 and 318.15) K. *J. Chem. Thermodyn.* **2013**, *67*, 13–20. https://doi.org/10.1016/j.jct.2013.07.010.

(986)	Gupta, S.; Chattejee, A.; Das, S.; Basu, B. Electrical Conductances of 1-Butyl-3-Propylimidazolium Bromide and 1-Butyl-3-Propylbenzimidazolium Bromide in Water, Methanol, and Acetonitrile at (308, 313, and 318) K at 0.1 MPa. *J. Chem. Eng. Data* **2013**, *58*, 1–6. https://doi.org/10.1021/je300339q.

(987)	Jain, P.; Sharma, S.; Shukla, R. K. Density and Viscosity of Tetrabutyl Ammonium Hydrogen Sulphate and Tetrabutyl Ammonium Chloride Salts in Aqueous and Methanolic Solution at 303 K. *Phys. Chem. Liq.* **2013**, *51*, 547–566. https://doi.org/10.1080/00319104.2012.760084.

(988)	Brahman, D.; Sinha, B. Partial Molar Volumes and Viscosity B-Coefficients for N,N’-Ethylenebis(Salicylideneiminato)-Diaquochromium(III) Chloride in Methanolic Solutions of 1-Butyl-2,3-Dimethylimidazolium Tetrafluoroborate at T = (298.15, 308.15, and 318.15) K. *J. Chem. Thermodyn.* **2014**, *68*, 260–269. https://doi.org/10.1016/j.jct.2013.09.018.

(989)	Garcia-Mardones, M.; Martin, S.; Gascon, I.; Lafuente, C. Thermophysical Properties of the Binary Mixture 1-Propylpyridinium Tetrafluoroborate with Methanol. *J. Chem. Eng. Data* **2014**, *59*, 1564–1573. https://doi.org/10.1021/je401120a.

(990)	Wu, J.-Y.; Chen, Y.-P.; Su, C.-S. Density and Viscosity of Ionic Liquid Binary Mixtures of 1-n-Butyl-3-Methylimidazolium Tetrafluoroborate with Acetonitrile, N,N-Dimethylacetamide, Methanol, and N-Methyl-2-Pyrrolidone. *J. Solution Chem.* **2015**, *44*, 395–412. https://doi.org/10.1007/s10953-014-0273-1.

(991)	Teske, V.; Vogel, E. Viscosity Measurements on Methanol Vapor and Their Evaluation. *J. Chem. Eng. Data* **2006**, *51*, 628–635. https://doi.org/10.1021/je050429b.

(992)	Ceriani, R.; Goncalves, C. B.; Rabelo, J.; Caruso, M.; Cunha, A. C. C.; Cavaleri, F. W.; Batista, E. A. C.; Meirelles, A. J. A. Group Contribution Model for Predicting Viscosity of Fatty Compounds. *J. Chem. Eng. Data* **2007**, *52*, 965–972. https://doi.org/10.1021/je600552b.

(993)	Pratas, M. J.; Freitas, S.; Oliveira, M.; Monteiro, S.; Lima, A. S.; Coutinho, J. A. P. Densities and Viscosities of Fatty Acid Methyl and Ethyl Esters. *J. Chem. Eng. Data* **2010**, *55*, 3983–3990. https://doi.org/10.1021/je100042c.

(994)	Pratas, M. J.; Freitas, S.; Oliveira, M. B.; Monteiro, S. C.; Lima, A. S.; Coutinho, J. A. P. Densities and Viscosities of Minority Fatty Acid Methyl and Ethyl Esters Present in Biodiesel. *J. Chem. Eng. Data* **2011**, *56*, 2175–2180. https://doi.org/10.1021/je1012235.

(995)	Treibs, W. *Ber. Dtsch. Chem. Ges. B* **1942**, *75*, 331–335. https://doi.org/10.1002/cber.19420750405.

(996)	Allen, C. A. W.; Watts, K. C.; Ackmann, R. G.; Pegg, M. J. Predicting the Viscosity of Biodiesel Fuels from Their Fatty Acid Ester Composition. *Fuel* **1999**, *78*, 1319–1326. https://doi.org/10.1016/S0016-2361(99)00059-9.

(997)	Gubareva, A. I. *Temperature Depencence of Physico-Chemical Properties of Hexamethyldisiloxane and Diphenylmethylsilane*; 874khp-D83; ONIITeKhim (Cherkassy UA), 1983.

(998)	Keffler, L. J. P.; MacLean, J. H. Homology in Long-Chain Compounds - Oleic Acid and the n-Alkyl Oleates. *J. Soc. Chem. Ind. London Trans. Commun.* **1935**, *54*, 178–185.

(999)	Albert, O. Viscosity Measurements on Homologous Ester Series with Special Regard to the Relations of Thorpe and Roger. *Z. Phys. Chem. Abt. A* **1938**, *182*, 421–429.

(1000)	Menzel; Berger; Nikuradse. *Chem. Ber.* **1949**, *82*, 418.

(1001)	YAMAWAKI, H. Pressure Dependence of Viscosity for Methyl Oleate and Methyl Linoleate up to 400 MPa. *Int. J. Thermophys.* **2020**, *41*, 112. https://doi.org/10.1007/s10765-020-02693-w.

(1002)	Keffler, L.; McLean, J. H. Homology in Long-Chain Compounds. I. Oleic Acid and the n-Alkyl Oleates. *J. Soc. Chem. Ind. (London)* **1935**, *54*, 178–185.

(1003)	Bhuyan, S.; Sundararajan, S.; Yao, L.; Hammond, E. G.; Wang, T. Boundary Lubrication Properties of Lipid-Based Compounds Evaluated Using Microtribological Methods. *Tribol. Lett.* **2006**, *22*, 167–172.

(1004)	Yao, L.; Hammond, E.; Wang, T. Melting Points and Viscosities of Fatty Acid Esters That Are Potential Targets for Engineered Oilseed. *J. Am. Oil Chem. Soc.* **2008**, *85*, 77–82. https://doi.org/10.1007/s11746-007-1159-9.

(1005)	Gaikwad, B. R.; Subrahmanyam, V. V. R. Physical Properties of N-Saturated Higher Fatty Alcohols in Liquid State. *J. Indian Chem. Soc.* **1988**, *65*, 266–268.

(1006)	Gros, A. T.; Feuge, R. O. Surface and Interfacial Tensions, Viscosities, and Other Physical Properties of Some n-Aliphatic Acids and Their Methyl and Ethyl Esters. *J. Am. Oil Chem. Soc.* **1952**, *29*, 313–317.

(1007)	Timmermans, J.; Hennaut-Roland, M. The Work of the International Bureau of Physical-Chemical Standards IV Study of the Physical Constants of Twenty Organic Compounds. *J. Chim. Phys. Phys.-Chim. Biol.* **1930**, *27*, 401–438.

(1008)	Al-Madfai, S. F.; Awwad, A. M.; Jbara, K. A. Some Properties of Binary Mixtures of N-Formylmorpholine and an Aromatic Hydrocarbon. *Thermochim. Acta* **1985**, *84*, 33–40. https://doi.org/10.1016/0040-6031(85)85372-7.

(1009)	Oshmyansky, Y.; Hanley, H. J. M.; Ely, J. F.; Kidnay, A. J. The Viscosities and Densities of Selected Organic Compounds and Mixtures of Interest in Coal Liquefaction Studies. *Int. J. Thermophys.* **1986**, *7*, 599. https://doi.org/10.1007/bf00502393.

(1010)	Goud, B. B.; Venkateswarlu, P.; Rao, M. V. P. Excess Volumes and Viscosities of Isomeric Xylenes with O-Nitrotoluene or m-Nitrotoluene at 308.15 K. *J. Chem. Eng. Data* **1999**, *44*, 731–734. https://doi.org/10.1021/je9900023.

(1011)	Moumouzias, G.; Ritzoulis, G. Viscosities and Densities for Propylene Carbonate + Toluene at 15, 20, 25, 30, and 35.Degree.C. *J. Chem. Eng. Data* **1992**, *37*, 482–483. https://doi.org/10.1021/je00008a024.

(1012)	Singh, S.; Sethi, B. P. S.; Katyal, R. C.; Rattan, V. K. Viscosities, Densities, and Speeds of Sound of Binary Mixtures of o-Xylene, m-Xylene, p-Xylene, and Isopropylbenzene with 4-Methylpentan-2-One at 298.15 K. *J. Chem. Eng. Data* **2004**, *49*, 1373–1375. https://doi.org/10.1021/je049899g.

(1013)	Al-Kandary, J. A.; Aljimaz, A. S.; Abdul-Latif, A.-H. M. Densities, Viscosities, and Refractive Indices of Binary Mixtures of Anisole with Benzene, Methylbenzene, Ethylbenzene, Propylbenzene, and Butylbenzene at (293.15 and 303.15) K. *J. Chem. Eng. Data* **2006**, *51*, 99–103. https://doi.org/10.1021/je0502546.

(1014)	Yang, T.; Xia, S.; Song, S.; Fu, X.; Ma, P. Densities and Viscosities of N-Formylmorpholine (NFM) + p-Xylene, + o-Xylene, + m-Xylene at Different Temperatures and Atmospheric Pressure. *J. Chem. Eng. Data* **2007**, *52*, 2062–2066. https://doi.org/10.1021/je7002513.

(1015)	Das, K.; Habibullah, M.; Rahman, I. M. M.; Hasegawa, H.; Uddin, K. S.; Saifuddin, K. Thermodynamic Properties of the Binary Mixture of Hexan-1-Ol with m-Xylene at T = (303.15, 313.15, and 323.15) K. *J. Chem. Eng. Data* **2009**, *54*, 3300–3302. https://doi.org/10.1021/je900219w.

(1016)	Habibullah, M.; Das, K. N.; Rahman, I. M. M.; Hasegawa, H.; Uddin, M. A.; Saifuddin, K. Volumetric and Viscometric Behavior of the Binary System: (Hexan-1-Ol + p-Xylene). *J. Chem. Eng. Data* **2010**, *55*, 5311–5313. https://doi.org/10.1021/je100390b.

(1017)	Zarei, H.; Salami, Z. Densities, Excess Molar Volumes, Viscosity, and Refractive Indices of Binary Mixtures of Ethanoic Acid and Trichloroethylene with Dimethylbenzenes at Different Temperatures. *J. Chem. Eng. Data* **2012**, *57*, 620–625. https://doi.org/10.1021/je201211f.

(1018)	Dikio, E. Z. D.; Vilakazi, G.; Ngoy, P. Density, Dynamic Viscosity and Derived Properties of Binary Mixtures of m-Xylene, o-Xylene, and p-Xylene, with Pyridine at T=293.15, 303.15, 313.15 and 323.15 K. *J. Mol. Liq.* **2013**, *177*, 190–197. https://doi.org/10.1016/j.molliq.2012.10.021.

(1019)	Yokoyama, C.; Takahashi, M.; Takahashi, S. Viscosity of Nitrous Oxide in the Critical Region. *Int. J. Thermophys.* **1994**, *15*, 603–626. https://doi.org/10.1007/bf01563790.

(1020)	Hurly, J. J. Viscosity and Speed of Sound of Gaseous Nitrous Oxide and Nitrogen Trifluoride Measured with a Greenspan Viscometer. *Int. J. Thermophys.* **2004**, *25*, 625–641. https://doi.org/10.1023/b:ijot.0000034229.03525.aa.

(1021)	Takahashi, M.; Shibasaki-Kitakawa, N.; Yokoyama, C.; Takahashi, S. Viscosity of Gaseous Nitrous Oxide from 298.15 K to 398.15 K at Pressures up to 25 MPa. *J. Chem. Eng. Data* **1996**, *41*, 1495–1498. https://doi.org/10.1021/je960060d.

(1022)	Phibbs, M. K. Viscosity of Neopentane. *J. Chem. Phys.* **1951**, *19*, 1420.

(1023)	Gonzalez, M. H.; Lee, A. L. Viscosity of 2,2-Dimethylpropane. *J. Chem. Eng. Data* **1968**, *13*, 66–69. https://doi.org/10.1021/je60036a021.

(1024)	Diaz Pena, M.; Esteban, F. Viscosity of Quasi-Spherical Molecules in the Vapor Phase. *An. R. Soc. Esp. Fis. Quim., A* **1966**, *62*, 347–357.

(1025)	Michels, A.; Botzen, A. The Thermal Conductivity of Nitrogen at Pressures up to 2500 Atm. *Physica (Amsterdam)* **1953**, *19*, 585.

(1026)	Ellis, C. P.; Raw, C. J. G. High-Temperature Gas Viscosities. II. Nitrogen, Nitric Oxide, Boron Trifluoride, Silicon Tetrafluoride, and Sulfur Hexafluoride. *J. Chem. Phys.* **1959**, *30*, 574–576.

(1027)	Flynn, G. P.; Hanks, R. V.; Lemaire, N. A.; Ross, J. Viscosity of Nitrogen, Helium, Neon, and Argon from -78.5 Degrees to 100 Degrees C below 200 Atmospheres. *J. Chem. Phys.* **1963**, *38*, 154.

(1028)	Goldman, K. Viscosity of Nitrogen at Low Temperatures and High Pressures. *Physica (Amsterdam)* **1963**, *29*, 499.

(1029)	Iwasaki, H.; Kestin, J. The Viscosity of Argon-Helium Mixtures. *Physica (Amsterdam)* **1963**, *29*, 1345–1372. https://doi.org/10.1016/s0031-8914(63)80243-8.

(1030)	Kestin, J.; Whitelaw, J. H. A Relative Determination of the Viscosity of Several Gases by the Oscillating Disk Method. *Physica (Amsterdam)* **1963**, *29*, 335.

(1031)	DiPippo, R.; Kestin, J.; Whitelaw, J. H. *Physica (Amsterdam)* **1966**, *32*, 2064.

(1032)	Clarke, A. G.; Smith, E. B. Low-Temperature Viscosities of Argon, Krypton and Xenon. *J. Chem. Phys.* **1968**, *48*, 3988.

(1033)	Clarke, A. G.; Smith, E. B. Low Temperature Viscosities and Intermolecular Forces of Simple Gases. *J. Chem. Phys.* **1969**, *51*, 4156–4161.

(1034)	Vermesse, J. Messure Du Coefficient de Viscosite de l’azote a Haute Pression. *Ann. Phys. (Paris)* **1969**, *4*, 245.

(1035)	Dawe, R. A.; Smith, E. B. Viscosities of the Inert Gases at High Temperatures. *J. Chem. Phys.* **1970**, *52*, 693.

(1036)	Kestin, J.; Paykoc, E.; Sengers, J. V. On the Density Expansion for Viscosity in Gases. *Physica (Amsterdam)* **1971**, *54*, 1.

(1037)	Golubev, I. F.; Kurin, V. I. Measurement of the Viscosity of Gases at Pressures to 4,000 Kgf/Cm2 and Different Temperatures. *Teploenergetika* **1974**, 83–85.

(1038)	Maitland, G. C.; Smith, E. B. Viscosity of Binary Gas Mixtures at High Temperatures. *J. Chem. Soc., Faraday Trans. 1* **1974**, *70*, 1191–1211.

(1039)	Zozulya, V. N.; Blagoi, Yu. P. Viscosity of Nitrogen Nearby Critical Point Liquid-Gas. *Zh. Eksp. Teor. Fiz.* **1974**, *66*, 212.

(1040)	Hoogland, J. H. B.; Van Den Berg, H. R.; Trappeniers, N. J. Measurements of the Viscosity of Sulfur Hexafluoride up to 100 Bar by a Capillary-Flow Viscometer. *Phys. A (Amsterdam, Neth.)* **1985**, *134*, 169–192.

(1041)	Audonnet, F.; Padua, A. A. H. Simultaneous Measurement of Density and Viscosity of N-Pentane from 298 to 383 K and up to 100 MPa Using a Vibrating-Wire Instrument. *Fluid Phase Equilib.* **2001**, *181*, 147–161. https://doi.org/10.1016/s0378-3812(01)00487-3.

(1042)	Lv, S.; Zhao, X.; Yao, C.; Wang, W.; Guo, Z. Viscosity of Gaseous Ethyl Fluoride (HFC-161). *Fluid Phase Equilib.* **2014**, *384*, 100–105. https://doi.org/10.1016/j.fluid.2014.10.023.

(1043)	Van Itterbeek, A.; Zink, H.; Hellemans, J. Viscosity of Liquified Gases at Pressures above One Atmosphere. *Physica (Amsterdam)* **1966**, *32*, 489.

(1044)	Grevendonk, W.; Herreman, W.; DeBock, A. Measurement of the Viscosity of Liquid Nitrogen. *Physica (Amsterdam)* **1970**, *46*, 600.

(1045)	Rutherford, W. M. Viscosity of Liquid-Phase Methyl Chloride. *J. Chem. Eng. Data* **1984**, *29*, 163–165. https://doi.org/10.1021/je00036a019.

(1046)	Seibt, D.; Vogel, E.; Bich, E.; Buttig, D.; Hassel, E. Viscosity Measurements on Nitrogen. *J. Chem. Eng. Data* **2006**, *51*, 526–533. https://doi.org/10.1021/je050399c.

(1047)	Keramidi, A. S.; Rastorguev, Yu. L. Viscosity of Hydrocarbons. n-Nonane. *Izv. Vyssh. Uchebn. Zaved., Neft Gaz* **1972**, *15*, 65–68.

(1048)	Landaverde-Cortes, D. C.; Iglesias-Silva, G. A.; Ramos-Estrada, M.; Hall, K. R. Densities and Viscosities of MTBE + Nonane or Decane at p = 0.1 MPa from (273.15 to 363.15) K. *J. Chem. Eng. Data* **2008**, *53*, 288–292. https://doi.org/10.1021/je700428f.

(1049)	Islam, N.; Waris, B. Viscosities o Organic Liquids and the Free Volume Model. *Indian J. Chem., Sect. A: Inorg., Phys., Theor. Anal.* **1976**, *14*, 30.

(1050)	Badalyan, A. G.; Rodchenko, S. I. Viscosity of N-Octane. *Izv. Vyssh. Uchebn. Zaved., Neft Gaz* **1986**, *29*, 61.

(1051)	Harris, K. R.; Malhotra, R.; Woolf, L. A. Temperature and Density Dependence of the Viscosity of Octane and Toluene. *J. Chem. Eng. Data* **1997**, *42*, 1254–1260. https://doi.org/10.1021/je970105q.

(1052)	Barrufet, M. A.; Hall, K. R.; Estrada-Baltazar, A.; Iglesias-Silva, G. A. Liquid Viscosity of Octane and Pentane + Octane Mixtures from 298.15 K to 373.15 K up to 25 MPa. *J. Chem. Eng. Data* **1999**, *44*, 1310–1314. https://doi.org/10.1021/je990043z.

(1053)	Trenzado, J. L.; Matos, J. S.; Gonzalez, E.; Romano, E.; Caro, M. N. A Study on Properties Derived from Densities and Viscosities for the Ternary Systems (Methyl Pentanoate or Methyl Heptanoate) + n-Octane + 1-Hexanol and Their Binary Subsystems at Various Temperatures. *J. Chem. Eng. Data* **2003**, *48*, 1004–1014. https://doi.org/10.1021/je034017j.

(1054)	Abdulagatov, I. M.; Azizov, N. D. (P,Rho,T,x) and Viscosity Measurements of (X1 n-Heptane + (1 - X1) n-Octane) Mixtures at High Temperatures and High Pressures. *J. Chem. Thermodyn.* **2006**, *38*, 1402–1415. https://doi.org/10.1016/j.jct.2006.01.012.

(1055)	Estrada-Baltazar, A.; Iglesias-Silva, G.; Caballero-Ceron, C. Volumetric and Transport Properties of Binary Mixtures of N-Octane + Ethanol, + 1-Propanol, + 1-Butanol, and + 1-Pentanol from (293.15 to 323.15) K at Atmospheric Pressure. *J. Chem. Eng. Data* **2013**, *58*, 3351–3363. https://doi.org/10.1021/je4004806.

(1056)	Dumitrescu, V.; Budeanu, M. M.; Radu, S.; Camenita, A. D. Densities and Viscosities of Binary Mixtures of 2-Methoxy-2-Methylpropane with n-Octane. *Phys. Chem. Liq.* **2015**, *53*, 242–251. https://doi.org/10.1080/00319104.2014.972554.

(1057)	Lavushev, A. V.; Lyusternik, V. E. Measurements of the Viscosity of Oxygen at Atmospheric Pressure up to 2000 K. *Zh. Fiz. Khim.* **1976**, *50*, 3009–3311.

(1058)	Timmermans, J.; Hennaut-Roland, M. Work of the International Bureau of Physical-Chemical Standards. IX. The Physical Constants of Twenty Organic Compounds. *J. Chim. Phys. Phys.-Chim. Biol.* **1955**, *52*, 223.

(1059)	Mamedov, A. M.; Akhundov, T. S.; Tairov, A. D. Experimental Study of O-Xylene Viscosity at Temperatures up to 275 c and Pressures up to 400 Bars. *Izv. Vyssh. Uchebn. Zaved., Neft Gaz* **1967**, *10*, 72–74.

(1060)	Ramachandran, D.; Rambabu, K.; Krishnan, K. M.; Venkateswarlu, P.; Raman, G. K. Volume of Mixing, Speed of Sound, and Viscosity of Butyl Acetate with Xylenes at 303.15 K. *J. Chem. Eng. Data* **1995**, *40*, 815–817. https://doi.org/10.1021/je00020a017.

(1061)	Moumouzias, G.; Ritzoulis, C.; Ritzoulis, G. A Study in Mixtures of -Butyrolactone with o-Xylene and m-Xylene: Densities and Viscosities. *J. Chem. Eng. Data* **1999**, *44*, 1187–1191. https://doi.org/10.1021/je990094w.

(1062)	Das, K. N.; Habibullah, M.; Uddin, K. S.; Barua, P. The Thermodynamic Excess Properties of Binary Mixtures: I. Hexanol-1 + o-Xylene. *J. Solution Chem.* **2006**, *35*, 1017–1028.

(1063)	Sage, B. H.; Lacey, W. N. Viscosity of Hydrocarbon Solutions. Viscosity of Liquid and Gaseous Propane. *Ind. Eng. Chem.* **1938**, *30*, 829–834.

(1064)	Hubbard, R. M.; Brown, G. G. Viscosity of N-Pentane. *Ind. Eng. Chem.* **1943**, *35*, 1276–1280.

(1065)	Reamer, H. H.; Cokelet, G.; Sage, B. H. Viscosity of Fluids at High Pressures. Rotating Cylinder Viscometer and the Viscosity of Pentane. *Anal. Chem.* **1959**, *31*, 1422.

(1066)	Belinskii, B. A.; Ikramov, S. K. COMPREHENSIVE INVESTIGATION OF THE ACOUSTICAL PARAMETERS, VISCOSITY, AND DENSITY OF n-PENTANE OVER A WIDE PRESSURE INTER VAL. *Sov. Phys. - Acoust.* **1973**, *18*, 300–303.

(1067)	Postigo, M. A.; Zurita, J. L.; De Soria, M. L. G.; Katz, M. Excess Thermodynamic Properties of the N-Pentanedichloromethane System at 298.15 K. *Can. J. Chem.* **1986**, *64*, 1966–1968. https://doi.org/10.1139/v86-325.

(1068)	De Soria, M. L. G.; Zurita, J. L.; Postigo, M. A.; Katz, M. Excess Thermodynamic Properties of the N-Pentane + Methylacetate System at 298.15 K. *Thermochim. Acta* **1988**, *130*, 249–258. https://doi.org/10.1016/0040-6031(88)87071-0.

(1069)	Acevedo, I. L.; Pedrosa, G. C.; Katz, M. Dynamic Viscosities of Non-Electrolyte Mixtures. *Phys. Chem. Liq.* **1993**, *26*, 99–106.

(1070)	Abdulagatov, I. M.; Rasulov, S. M.; Abdurakhmanov, I. M. Pentane Dynamics near the Liquid-Gas Critical Point. *Zh. Fiz. Khim.* **1991**, *65*, 1306–1311.

(1071)	Diller, D. E. Measurements of the Viscosity of Saturated and Compressed Liquid Propane. *J. Chem. Eng. Data* **1982**, *27*, 240–243. https://doi.org/10.1021/je00029a003.

(1072)	Klemenc-Wien, A.; Remi, W. Experimental Investigation of the Viscosity of Nitric Oxide, Propane, and Their Mixtures with Hydrogen. *Monatsh. Chem.* **1924**, *44*, 307.

(1073)	Starling, K. E.; Eakin, B. E.; Ellington, R. T. Liquid, Gas, and Dense-Fluid Viscosity of Propane. *AIChE J.* **1960**, *6*, 438–442.

(1074)	Carmichael, L. T.; Berry, V. M.; Sage, B. H. Viscosity of Hydrocarbons. Propane. *J. Chem. Eng. Data* **1964**, *9*, 411–415. https://doi.org/10.1021/je60022a038.

(1075)	Golubev, I. F.; Gnezdilov, N. E. *Viazkost’ Gazovykh Smesei*; 1971.

(1076)	Nagaoka, K.; Yamashita, Y.; Tanaka, Y.; Kubota, H.; Makita, T. Viscosity of Binary Gaseous Mixtures of Fluorocarbons. *J. Chem. Eng. Jpn.* **1986**, *19*, 263.

(1077)	Vogel, E. The Viscosity of Gaseous Propane and Its Initial Density Dependence. *Int. J. Thermophys.* **1995**, *16*, 1335–1351. https://doi.org/10.1007/bf02083544.

(1078)	Wilhelm, J.; Vogel, E. Viscosity Measurements on Gaseous Propane. *J. Chem. Eng. Data* **2001**, *46*, 1467–1471. https://doi.org/10.1021/je010062k.

(1079)	Haepp, H. J. Messung Der Viscositat von Kohlendioxid Und Propylen. *Warme- Stoffubertrag.* **1976**, *9*, 281.

(1080)	Zimakov, P. V.; Sokolava, A. V. Physical Properties of Propylene Oxide. *Zh. Fiz. Khim.* **1953**, *27*, 1079.

(1081)	Yang, C.; Ma, P.; Tang, D.; Yin, Q.-X.; Zhao, C. Excess Molar Volume,Viscosity and Heat Capacity for the Binary Mixture of p-Xylene and Acetic Acid at Different Temperatute. *Chin. J. Chem. Eng.* **2002**, *10*, 604.

(1082)	Feng, L.; Ma, P.; Xiang, Z.; Yang, C. Determination of Densities and Viscosities for Binary Mixture of Diethyl Carbonate and P-Xylene at Atmospheric Pressure and Different Temperatures. *Shiyou Huagong* **2005**, *34*, 965.

(1083)	Di, Z.; Ma, P.; Xia, S.; Yang, T. Determination of Densities and Viscosities of P-Xylene-Acetic Acid-Water Ternary System. *Shiyou Huagong* **2006**, *35*, 851.

(1084)	Vogel, E.; Hendl, S. Vapor Phase Viscosity of Toluene and P-Xylene. *Fluid Phase Equilib.* **1992**, *79*, 313–326. https://doi.org/10.1016/0378-3812(92)85140-4.

(1085)	Lavochnik, A. I.; Solovey, R. L. Viscosity of Freons and Their Mixtures in Liquid State at Atmospheric Pressure. *Kholod. Tekhn.* **1980**, 32–34.

(1086)	Kumagai, A.; Takahashi, S. Viscosity of Saturated Liquid Fluorocarbon Refrigerants from 273 to 353 K. *Int. J. Thermophys.* **1991**, *12*, 105–117. https://doi.org/10.1007/bf00506125.

(1087)	Benning, A. F.; Markwood, W. H. The Viscosities of “Freon” Refrigerants. *ASHRAE J.* **1939**, *37*, 243–247.

(1088)	Rivkin, S. L.; Levin, A. Ya.; Izrailevskij, L. B. *Teplofiz. Svoistva Vesch. Mater.*; 1971.

(1089)	Ivanchenko, S. I. Dynamic Viscosity Investigation of Freons of Methane and Ethane Rows. PhD Thesis, Odessa Technological Institute of Food Industry, 1974.

(1090)	Geller, V. Z.; Ivanchenko, S. I.; Kronberg, A. V. Study of Dynamic Viscosity Coefficient of Methane Type Freons. In *Teplofiz. Svoistva Veshchestv. Mater.*; Collect. Vol. No., V. A. Rabinovich, Ed., Standards Publ.: Moscow, 1975; pp 148–161.

(1091)	Geller, V. Z.; Karabanov, E. M.; Gunchuk, B. V.; Zakharzhevskiy, V. Y.; Lapardin, N. I. The Investigation of the Viscosity Coefficient of Some of the Liquid Gases About the Saturated Line. *Gaz. Prom.* **1976**, 32–2.

(1092)	Karbanov, E. M. Investigation of the Dynamic Viscosity of Some Freons of Ethane Type and of the Bromide Freons. PhD Thesis, Grozny Petroleum Institute, 1978.

(1093)	Hovorka, F.; Geiger, F. E. Thermodynamic Prop. of Trifluorotrichloroethane and Difluorotetrachlo- Ethane. *J. Am. Chem. Soc.* **1933**, *55*, 4759–4761. https://doi.org/10.1021/ja01339a004.

(1094)	Ivanchenko, S. I. Viscosity of Triflourotricloroethane. *Izv. Vyssh. Uchebn. Zaved., Neft Gaz* **1972**, *15*, 82.

(1095)	Rasskazov, D. C.; Babikov, Yu. M.; Filatov, N. Ya. *Teplofiz. Svoistva Veshch. Mater.*; 1980.

(1096)	Geller, V. Z. Investigation of the Viscosity of Freons of the Methane, Ethane, and Propane Types. Summary of Experimental Data. In *Teplofiz. Svoistva Veshchestv. Mater.*; No. 15, Sychev, V. V., Ed., Standards Publ.: Moscow, 1980; pp 89–114.

(1097)	Arnemann, M.; Kruse, H. Liquid Viscosities of the Non-Azeotropic Binary Refrigerant Mixtures R22/R1 14, R22/R152A, R22/R142B. Measurements and Calculations. In *Actes Congr. Int. Froid, 18th*; Vol. 2, Int. Congr. Refrig., Saint-Hyacinthe: Que, 1991; pp 379–383.

(1098)	Bondar’, G. E. The Viscosity of Freons at Low Temperature. PhD Thesis, Odessa Technological Institute of Chemical Industry, 1984.

(1099)	Kronberg, A. V. Experimental and Theoretical Investigation of the Viscosity of Methane and Ethane Row’s Refrigerants. PhD Thesis, Azerbaijan Institute of Petroleum Industry, 1979.

(1100)	Awbery, J. H.; Griffiths, E. The Viscosities of Some Liquid Refrigerants. *Proc. Phys. Soc.* **1936**, *48*, 372–380.

(1101)	Zaloudik, P. Contribution to Viscosity Measurements with Aid of Hoppler’s Viscometer. *Chem. Prum.* **1962**, *12*, 81–83.

(1102)	Srichand, M.; Tirunarayanan, M. A.; Ramachandran, A. Studies of the Viscosity of Mixtures of R12 and R22 Vapors. *ASHRAE J.* **1970**, *12*, 61–66.

(1103)	Chaikovskii, V. F.; Geller, V.; Ivanchenko, S. I. Viscosity of Diflorodichloromethane. *Izv. Vyssh. Uchebn. Zaved., Neft Gaz* **1973**, *16*, 111–112.

(1104)	Alam, M. J.; Yamaguchi, K.; Hori, Y.; Kariya, K.; Miyara, A. Measurement of Thermal Conductivity and Viscosity of Cis-1-Chloro-2, 3, 3, 3-Tetrafluoropropene (R-1224yd (Z)). *Int. J. Refrig.* **2019**, *104*, 221–228.

(1105)	Matsuo, S.; Tanaka, Y.; Kubota, H.; Makita, T.; Kashiwagi, H. Thermophysical Properties of R123 (PVT Properties, Viscosity, and Thermal Conductivity). In *Proc. Symp. Thermophys. Prop. 9th*; Japan, 1988; p 231.

(1106)	Diller, D. E.; Aragon, A. S.; Laesecke, A. Measurements of the Viscosities of Saturated and Compressed Liquid Chlorodifluoromethane (R22). *Int. J. Refrig.* **1993**, *16*, 19–22.

(1107)	Nabizadeh, H.; Mayinger, F. Viscosity of Gaseous R123, R134a and R142b. In *Proc. European Conference on Thermophysical Properties, 12th*; Vienna, Austria, 1990.

(1108)	Nabizadeh, H.; Mayinger, F. Viscosity of Gaseous R123, R134a, and R142b. *High Temp. - High Pressures* **1992**, *24*, 221–230.

(1109)	Dowdell, D. C.; Matthews, G. P. Gas Viscosities and Intermolecular Interactions of Replacement Refrigerants HCFC 123 (2,2-Dichloro-1,1,1-Trifluoroethane), HCFC 124 (2- Chloro-1,1,1,2-Tetrafluoroethane) and HFC 134a (1,1,1,2-Tetrafl. *J. Chem. Soc., Faraday Trans.* **1993**, *89*, 3545–3552.

(1110)	Yokoyama, C.; Nishino, T.; Takahashi, M. Viscosity of Gaseous Mixtures of HFC-125 (Pentafluoroethane)+ HFC-134a (1, 1, 1, 2-Tetrafluoroethane) under Pressure. *Fluid Phase Equilib.* **2000**, *174* (1–2), 231–240.

(1111)	Nakazawa, N.; Kawamura, M.; Sekiya, A.; Ootake, K.; Tamai, R.; Kurokawa, Y.; Murata, J. Liquid Viscosities of Fluorinated Dialkylethers. *Nippon Reito Kyokai Ronbunshu* **2002**, *19*, 33–41.

(1112)	Meng, X.; Wen, C.; Wu, J. Measurement and Correlation of the Liquid Viscosity of Trans-1-Chloro-3, 3, 3-Trifluoropropene (R1233zd (E)). *J. Chem. Thermodyn.* **2018**, *123*, 140–145.

(1113)	Hulse, R.; Singh, R.; Pham, H. Physical Properties of HFO-1234yf (Paper No. 178). In *3rd Int. Inst. Refrigeration Conference on Thermophysical Properties and Transfer Processes of Refrigerants*; Boulder (CO), June, 2009; pp 1–7.

(1114)	Meng, X.; Qiu, G.; Wu, J.; Abdulagatov, I. M. Viscosity Measurements for 2,3,3,3-Tetrafluoroprop-1-Ene (R1234yf) and Trans-1,3,3,3-Tetrafluoropropene (R1234ze(E)). *J. Chem. Thermodyn.* **2013**, *63*, 24–30. https://doi.org/10.1016/j.jct.2013.03.013.

(1115)	Grebenkov, A. J.; Hulse, R.; Pham, H.; Singh, R. Physical Properties and Equation of State for Trans-1,3,3,3-Tetrafluoropropene. In *3rd Int. Inst. Refrigerantion Conference on Thermophysical Properties and Transfer Processes of Refrigerants*; Boulder, CO, pp Paper No. 191 Pages 1-19, 2009.

(1116)	Diller, D. E.; Peterson, S. M. Measurements of the Viscosities of Saturated and Compressed Fluid 1-Chloro- 1,2,2,2-Tetrafluoroethane (R124) and Pentafluoroethane (R125) at Temperatures between 120 and 420 K. *Int. J. Thermophys.* **1993**, *14*, 55–66. https://doi.org/10.1007/bf00522661.

(1117)	Ripple, D.; Matar, O. Viscosity of the Saturated Liquid Phase of Six Halogenated Compounds and Three Mixtures. *J. Chem. Eng. Data* **1993**, *38* (4), 560–564.

(1118)	Assael, M. J.; Polimatidou, S. K. Measurements of the Viscosity of Refrigerants in the Vapor Phase. *Int. J. Thermophys.* **1997**, *18*, 353–366. https://doi.org/10.1007/bf02575166.

(1119)	Oliveira, C. M. B. P.; Wakeham, W. A. The Viscosity of Liquid R134a. *Int. J. Thermophys.* **1993**, *14*, 33–44. https://doi.org/10.1007/bf00522659.

(1120)	Heide, R.; Schenk, J. *Betimmung Der Transportgroben von HFKW*; 708; Forschungsrat Kaltetechnik E.V. Betimmung der Transportgroben von HFKW: Dresden, 1996.

(1121)	Wilson, L. C.; Wilding, W. V.; Wilson, G. M.; Rowley, R. L.; Felix, V. M.; Chisolm-Carter, T. Thermophysical Properties of HFC-125. *Fluid Phase Equilib.* **1992**, *80*, 167–177. https://doi.org/10.1016/0378-3812(92)87065-u.

(1122)	Ripple, D.; Defibaugh, D. Viscosity of the Saturated Liquid Phase of Three Fluorinated Ethanes: R152a, R143a, and R125. *J. Chem. Eng. Data* **1997**, *42*, 360–364. https://doi.org/10.1021/je960284w.

(1123)	Avelino, H. M. T.; Fareleira, J. M. N. A.; Oliveira, C. M. B. P. Viscosity of Compressed Liquid 1,1,1-Trifluoroethane (HFC-143a) and Pentafluoroethane (HFC-125). *J. Chem. Eng. Data* **2006**, *51*, 1672–1677. https://doi.org/10.1021/je0601356.

(1124)	Oliveira, C. M. B. P.; Wakeham, W. A. Viscosity of R134a, R32, and R125 at Saturation. *Int. J. Thermophys.* **1999**, *20*, 365–373. https://doi.org/10.1023/a:1022640617694.

(1125)	Takahashi, M.; Shibasaki-Kitakawa, N.; Yokoyama, C. Viscosity of Gaseous HFC-125 (Pentafluoroethane) under High Pressures. *Int. J. Thermophys.* **1999**, *20*, 445–453. https://doi.org/10.1023/a:1022648819511.

(1126)	Reed, J. F.; Rabinovitch, B. S. Viscosities of Fluorinated Methyl Bromides and Chlorides. *Ind. Eng. Chem. Chem. Eng. Data Ser.* **1957**, *2*, 75. https://doi.org/10.1021/i460002a021.

(1127)	Ivanchenko, S. I.; Bondar’, G. E.; Geller, V. Z. Experimental Investigation of Viscosity of Freon-13 and 14. In *Kholod. Tekh. Tekhnol., Collect. Vol.*; No. 19, Tekhnika: Kiev, 1974; pp 98–102.

(1128)	Takahashi, M.; Takahashi, S.; Iwasaki, H. Viscosity of Gaseous Chlorotrifluoromethane (R 13) under Pressure. *J. Chem. Eng. Data* **1985**, *30*, 10–14. https://doi.org/10.1021/je00039a004.

(1129)	Chajkovskij, V. F.; Geller, V. Z.; Bondar, E. G. The Viscosity of R13 at Low Temperatures. *Kholod. Tekh. Tekhnol.* **1977**, 66–72.

(1130)	Diller, D. E.; Van Poolen, L. J. Measurement of Viscosities of Saturated and Compressed Fluid Chlorotrifluoromethane (R13). *Cryogenics* **1989**, *29*, 1063–1066.

(1131)	Alam, M. J.; Islam, M. A.; Kariya, K.; Miyara, A. Measurement of Thermal Conductivity and Correlations at Saturated State of Refrigerant Trans-1‑chloro‑3, 3, 3-Trifluoropropene (R-1233zd (E)). *Int. J. Refrig.* **2018**, *90*, 174–180.

(1132)	Han, L.-Z.; Zhu, M.-S.; Li, X.-Y.; Luo, D. Viscosity of Saturated Liquid for 1,1,1,2-Tetrofluoroethane. *J. Chem. Eng. Data* **1995**, *40*, 650–652. https://doi.org/10.1021/je00019a026.

(1133)	Huber, M. L.; Laesecke, A.; Perkins, R. A. Model for the Viscosity and Thermal Conductivity of Refrigerants, Including a New Correlation for the Viscosity of R134a. *Ind. Eng. Chem. Res.* **2003**, *42* (13), 3163–3178. https://doi.org/10.1021/ie0300880.

(1134)	Pasekov, M. F.; Ustjushanin, E. E. Viscosity of R134a in Gas Phase. *Teplofiz. Vys. Temp.* **1994**, *32*, 630–633.

(1135)	Shibasaki-Kitakawa, N.; Takahashi, M.; Yokoyama, C. Viscosity of Gaseous HFC-134a (l,l,l,2-Tetrafluoroethane) Under High Pressures. *Int. J. Thermophys.* **1998**, *19*, 1285–1295. https://doi.org/10.1023/a:1021919215793.

(1136)	Ruvinskii, G. Y.; Lavrenchenko, G. K.; Il’yushenko, S. V. Thermophysical Properties of R-134a. *Kholod. Tekhn.* **1990**, *67*, 20–26.

(1137)	Lavrenchenko, G. K.; Ruvinskii, G. Y.; Iljushenko, S. V.; Kanaev, V. V. Thermophysical Properties of Refrigerant R134a. *Int. J. Refrig.* **1992**, *15*, 386–392.

(1138)	Laesecke, A.; Bair, S. High-Pressure Viscosity Measurements of 1,1,1,2-Tetrafluoroethane. *Int. J. Thermophys.* **2011**, *32*, 925–941. https://doi.org/10.1007/s10765-011-0978-y.

(1139)	Comunas, M. J. P.; Baylaucq, A.; Quinones-Cisneros, S. E.; Zeberg-Mikkelsen, C. K.; Boned, C.; Fernandez, J. Viscosity Measurements and Correlations for 1,1,1,2-Tetrafluoroethane (HFC-134a) up to 140 MPa. *Fluid Phase Equilib.* **2003**, *210*, 21–32. https://doi.org/10.1016/s0378-3812(03)00158-4.

(1140)	Meng, X.; Zhang, J.; Wu, J. Compressed Liquid Viscosity of 1,1,1,3,3-Pentafluoropropane (R245fa) and 1,1,1,3,3,3-Hexafluoropropane (R236fa). *J. Chem. Eng. Data* **2011**, *56*, 4956–4964. https://doi.org/10.1021/je200854k.

(1141)	Wilhelm, J.; Vogel, E. Gas-Phase Viscosity of the Alternative Refrigerant R134a at Low Densities. *Fluid Phase Equilib.* **1996**, *125*, 257–266. https://doi.org/10.1016/s0378-3812(96)03082-8.

(1142)	Raw, C. J. G.; Tang, H. Viscosity and Diffusion Coefficients of Gaseous Sulfer Hexafluoride-Carbon Tetrafluoride Mixtures. *J. Chem. Phys.* **1963**, *39*, 2616–2618.

(1143)	Genot, B. Thermal Effusion and Viscosity of Gaseous Tetrafluoromethane. *J. Chim. Phys. Phys.-Chim. Biol.* **1970**, *67*, 1322–1331.

(1144)	Filatov, N. Ya. Experimental Investigation of Viscosity of Refrigerants-14, 21, 23. PhD Thesis, Moscow Energy Institute, 1975.

(1145)	Kochubey, V. F.; Moin, F. B. Determination of Gas-Kinetic Diameters of Fluoromethane Molecules. *Zh. Fiz. Khim.* **1978**, *52*, 15–17.

(1146)	Rasskazov, D. S.; Babikov, Yu. M.; Filatov, N. Ya. Experimental Investigation of the Viscosity of Freon 23. In *Teplofiz. Svoistva Veshchestv Mater.*; Collect. Vol. No. 8, V. A. Rabinovich, Ed., Standards Publ.: Moscos, 1975; pp 142–147.

(1147)	Kumagai, A.; Takahashi, S. Saturated Liquid Viscosities of Environmentally Acceptable Fluorocarbons. In *Proc. Symp. Thermophys. Prop., 11th*; Tokyl, Japan, 1990; pp 119–122.

(1148)	Kumagai, A.; Takahashi, S. Saturated Liquid Viscosities and Densities of Environmentally Acceptable Hydrochlorofluorocarbons (HCGCs). *Int. J. Thermophys.* **1993**, *14*, 339–342. https://doi.org/10.1007/bf00507818.

(1149)	Mears, W. H.; Stahl, R. F.; Orfeo, S. R.; Shair, R. C.; Kells, L. F.; Thompson, W.; McCann, H. Thermodynamic Properties of Halogenated Ethanes and Ethylenes. *Ind. Eng. Chem.* **1955**, *47*, 1449.

(1150)	Konrad, S. I. Viscosity of Binary and Multicomponent Refrigerant Mixtures. PhD Thesis, Odessa Technological Institute of Food Industry, 1985.

(1151)	Takahashi, M.; Yokoyama, C.; Takahashi, S. Viscosities of Gaseous R13B1, R142b, and R152a. *J. Chem. Eng. Data* **1987**, *32*, 98–103. https://doi.org/10.1021/je00047a027.

(1152)	Wang, X.; Wu, J.; Liu, Z.-G. Viscosity of R143a in Gaseous Phase. *J. Eng. Thermophys.* **2009**, *30*, 4;1455-1458.

(1153)	Stern, K. H.; Martell, A. E. Thermodynamics of Ion Pair Formation in Some Non-Aqueous Solvents. *J. Am. Chem. Soc.* **1955**, *77*, 1983–1986. https://doi.org/10.1021/ja01612a083.

(1154)	Alluraiah, G.; Ramanjaneyulu, K.; Krishnaiah, A. A Study of Interactions in Binary Mixtures of 1-Heptanol with Some Chlorinated Ethanes and Ethenes from Viscosity Measurements at 303.15 K. *Phys. Chem. Liq.* **1989**, *19*, 181.

(1155)	Malhotra, R.; Price, W. E.; Woolf, L. A.; Easteal, A. J. Thermodynamic and Transport Properties of 1,2-Dichloroethane. *Int. J. Thermophys.* **1990**, *11*, 835–861. https://doi.org/10.1007/bf00503578.

(1156)	Krishnaiah, A.; Surendranath, K. N. Densities, Speeds of Sound, and Viscosities of Mixtures of Oxolane with Chloroethanes and Chloroethenes. *J. Chem. Eng. Data* **1996**, *41*, 1012–1014. https://doi.org/10.1021/je950304e.

(1157)	Zhang, H.-L.; Han, S.-J. Viscosmetric and Volumetric Studies on Binary Mixtures of 1,2-Dichloroethane and Chlorinated Methanes or Their Binary Equimolar Mixtures at 303.15 K. *Fluid Phase Equilib.* **1997**, *140*, 233–244. https://doi.org/10.1016/s0378-3812(97)00193-3.

(1158)	Mussari, L.; Postigo, M.; Lafuente, C.; Royo, F. M.; Urieta, J. S. Viscosity Measurements for the Binary Mixtures of 1,2-Dichloroethane or 1,2-Dibromoethane with Isomeric Butanols. *J. Chem. Eng. Data* **2000**, *45*, 86–91. https://doi.org/10.1021/je990211n.

(1159)	Oswal, S. L.; Patel, I. N.; Modi, P. S.; Barad, S. A. Viscosities and Excess Molar Volumes of Binary Mixtures of Alkyl Acetates with Di-, Tri-, and Tetrachloroethane. *Int. J. Thermophys.* **2000**, *21*, 681. https://doi.org/10.1023/a:1006689418706.

(1160)	Aznarez, S.; Katz, M.; Arancibia, E. L. Thermophysical Properties of the Ternary System 2-Methoxyethanol + Acetonitrile + 1,2- Dichloroethane, and the Binary Systems at 25deg C. *J. Solution Chem.* **2002**, *31*, 639–651.

(1161)	Sathyanarayana, B.; Ranjithkumar, B.; Jyostna, T. S.; Satyanarayana, N. Densities and Viscosities of Binary Liquid Mixtures of N-Methylacetamide with Some Chloroethanes and Chloroethenes at T = 308.15 K. *J. Chem. Thermodyn.* **2007**, *39*, 16–21. https://doi.org/10.1016/j.jct.2006.06.009.

(1162)	Bhatia, S. C.; Bhatia, R.; Dubey, G. P. Studies on Transport and Thermodynamic Properties of Binary Mixtures of Hexan-1-Ol with Halogenated Compounds at 293.15 K. *J. Chem. Eng. Data* **2009**, *54*, 3303–3306. https://doi.org/10.1021/je900278v.

(1163)	Ranjbar, S.; Fakhri, K.; Ghasemi, J. B. Densities and Viscosities of (1-Propanol + 1,2-Dichloroethane), (1-Propanol + Benzaldehyde), (Benzaldehyde + 1,2-Dichloroethane), and (1-Propanol + 1,2-Dichloroethane + Benzaldehyde) Mixtures from T = 288.15 K to 313.15 K. *J. Chem. Eng. Data* **2009**, *54*, 3284–3290. https://doi.org/10.1021/je900212d.

(1164)	Syeda, A. B.; Koppula, A. J.; Boodida, S.; Nallani, S. Excess Molar Volumes and Sound Speed in (Phenylacetonitrile + 1,2-Dichloroethane), (Phenylacetonitrile + 1,1,2-Trichloroethane), (Phenylacetonitrile + 1,1,2,2-Tetrachloroethane), (Phenylacetonitrile + Trichloroethene), and (Phenylacetonitrile + Tetrachloroethene) at Temperatures of (303.15, 308.15, and 313.15) K. *J. Chem. Eng. Data* **2010**, *55*, 1405–1410. https://doi.org/10.1021/je900570e.

(1165)	vanderGulik, P. S. Viscosity of Saturated R152a Measured with a Vibrating Wire Viscometer. *Int. J. Thermophys.* **1995**, *16*, 867–876. https://doi.org/10.1007/bf02093469.

(1166)	Lapardin, N. I. Viscosity of Difluooroethane. *Izv. Vyssh. Uchebn. Zaved., Neft Gaz* **1982**, *25*, 24–63.

(1167)	Lapardin, N. I. Investigation of the Viscosity of Freons R 152A and R 218. PhD Thesis, Odessa Technological Institute of Chemical Industry, 1983.

(1168)	Wang, W.; Li, G.; He, Z.; Liu, Z. Experimental Study for Gaseous Viscosities with Oscillating-Disk Viscometer. *Xi’an Jiaotong Daxue Xuebao* **1997**, *31*, 10–14.

(1169)	Geller, V. Z.; Ivanchenko, S. I.; Peredriy, V. G. Experimental Investigation of Dynamic Viscosity and Thermal Conductivity Coeficients of Difluorudichloromethane. *Izv. Vyssh. Uchebn. Zaved., Neft Gaz* **1973**, 61–65.

(1170)	Butyrskaya, S. T. Investigation of the Dynamic Viscosity Coefficient of Freon-22. In *Teplofiz. Svoistva Zhidkostey*; Collect. Vol., Nauka: Moscow, 1970; pp 73–76.

(1171)	Takahashi, M.; Takahashi, S.; Iwasaki, H. Viscosity of Gaseous Chlorodifluoromethane(R-22). *Kagaku Kogaku Ronbunshu* **1983**, *9*, 482–484.

(1172)	Zhang, Z.; Liu, Z.-G.; Wang, W. Gaseous Viscosity Measurement for Several Refrigerant Mixtures. *J. Chem. Eng. Data* **2002**, *47*, 383–387. https://doi.org/10.1021/je015502m.

(1173)	Chaikovskiy, V. F.; Geller, V. Z.; Gorykin, S. F.; Artamonov, S. D.; Bondar’, G. E.; Ivanchenko, S. I.; Lenskiy, L. R.; Peredriy, V. G. Comprehensive Investigation of the Thermophysical Properties of Most Important and Promising Regrigerants in Liquid and Gaseous Phase. In *Teplofiz. Svoistva Zhidkostey, Collect. Vol.*; Nauka: Moscow, 1976; pp 108–117.

(1174)	Hongo, M.; Takahashi, S. Viscosity of Chlrodifluoromethane at High Pressure. *J. Chem. Eng. Jpn.* **1982**, *15*, 155–158.

(1175)	Laesecke, A.; Hafer, R. F. Viscosity of Fluorinated Propane Isomers. 2. Measurements of Three Compounds and Model Comparisons. *J. Chem. Eng. Data* **1998**, *43*, 84–92. https://doi.org/10.1021/je970186q.

(1176)	Liu, X.-J.; Shi, L.; Han, L.-Z.; Zhu, M.-S. Liquid Viscosity of 1,1,1,2,3,3,3-Heptafluoropropane (HFC-227ea) along the Saturation Line. *J. Chem. Eng. Data* **1999**, *44*, 688–692. https://doi.org/10.1021/je980098l.

(1177)	Tang, X.; Zhang, J.; Meng, X.; Wu, J. Viscosity and Density Measurements for Liquid 1,1,1,2,3,3,3,-Heptafluoropropane(HFC-227ea). *J. Eng. Thermophys.* **2012**, *33*, 191–194.

(1178)	McCoubrey, J. C.; Singh, N. M. Intermolecular Forces in Quasi-Spherical Molecules. *Trans. Faraday Soc.* **1957**, *53*, 877–883.

(1179)	Tomassini, F.; Levi, A. C.; Scoles, G. Viscosity and Thermal Conductivity of Polar Gases in an Electric Field. *Physica (Amsterdam)* **1970**, *49*, 299–341.

(1180)	Rasskazov, D. C.; Babikov, Yu. M.; Filatov, N. Ya. Experimental Investigation of Viscosity of Refrigerant R-23. *Tr. Mosk. Energ. Inst.* **1974**, 62–69.

(1181)	Sagaidakova, N. G. Experimental Investigation of Viscosity of Freons-12B1, 13B1, and 502 in Wide Region of State Parameters. PhD Thesis, Leningrad Technological Institute of Refrigeration Industry, 1977.

(1182)	Yokoyama, C.; Takahashi, M. Viscosity of CHF3 in the Critical Region. *Int. J. Thermophys.* **1997**, *18*, 1369–1385. https://doi.org/10.1007/bf02575340.

(1183)	Laesecke, A.; Defibaugh, D. R. Viscosity of 1,1,1,2,3,3-Hexafluoropropane and 1,1,1,3,3,3- Hexafluoropropane at Saturated-Liquid Conditions from 262 K to 353 K. *J. Chem. Eng. Data* **1996**, *41*, 59–62. https://doi.org/10.1021/je950206t.

(1184)	Takahashi, M.; Shibasaki-Kitakawa, N.; Yokoyama, C.; Takahashi, S. Gas Viscosity of Difluoromethane from 298.15 to 423.15 K and up to 10 Mpa. *J. Chem. Eng. Data* **1995**, *40*, 900–902. https://doi.org/10.1021/je00020a036.

(1185)	Geller, V. Z.; Paulaitis, M. E.; Bivens, D. B.; Yokozeki, A. Viscosities of HFC-32 and HFC-32/Lubricant Mixtures. *Int. J. Thermophys.* **1996**, *17*, 75–83. https://doi.org/10.1007/bf01448211.

(1186)	Badylkes, I. S. 1960.

(1187)	McGovern, E. W. Chlorohydrocarbon Solvents. *Ind. Eng. Chem.* **1943**, *35*, 1230–1239.

(1188)	Kessel’man, P. M.; Porichanskii, E. G.; Romanov, V. K.; Ponomareva, O. P.; Svetlichnyi, P. I.; Zhelezny, V. P.; Slyusarev, V. V.; Karbanov, E. M. Complex Study of the Thermophysical Properties of Refrigerant S318. *Teplofiz. Svoistva Veshchestv Mater.* **1977**, 4–10.

(1189)	Sekiya, A.; Misaki, S. The Potential of Hydrofluoroethers to Replace CFCs, HCFCs, and PFCs. *J. Fluorine Chem.* **2000**, *101*, 215–221.

(1190)	Hu, X.; Meng, X.; Wei, K.; Li, W.; Wu, J. Compressed Liquid Viscosity Measurements of HFE-7000, HFE-7100, HFE-7200, and HFE-7500 at Temperatures from (253 to 373) K and Pressures up to 30 MPa. *J. Chem. Eng. Data* **2015**, *60*, 3562–3570. https://doi.org/10.1021/acs.jced.5b00499.

(1191)	Ulybin, S. A.; Makarushkin, V. I. Viscosity of Xenon at Temperatures from 170 to 1300 K and at Pressures up to 1000 Bar. *Teplofiz. Vys. Temp.* **1977**, *15*, 509–514.

(1192)	Ueda, K.; Kigoshi, K. The Viscosity of the Gaseous Heaxfluorides of Sulfur, Selenium, and Tellurium. *J. Inorg. Nucl. Chem.* **1974**, *36*, 989–992.

(1193)	Grigor’ev, V. A.; Keramidi, A. S.; Grachev, V. K.; Rastorguev, Yu. L. Experimental Investigation of Viscosity of Sulfur-Hexafluroide. *Therm. Eng.* **1978**, *24*, 66–68.

(1194)	Hafer, R. F. Torsional Crystal Viscometry: Wide Ranging Resonance Measurements and Feasibility Study of Transient-Decay Measurements. Master’s Thesis, University of Colorado, 1999.

(1195)	Wilhelm, J.; Seibt, D.; Bich, E.; Vogel, E.; Hassel, E. Viscosity Measurements on Gaseous Sulphur Hexafluoride. *J. Chem. Eng. Data* **2005**, *50*, 896–906. https://doi.org/10.1021/je049612f.

(1196)	Grigor’ev, B. A.; Keramidi, A. S.; Grachev, V. K.; Rastorguev, Yu. L. Experimental Study of the Viscosity of Sulfur Hexafluoride. *Teploenergetika* **1977**, 85–87.

(1197)	Trautz, M.; Weizel, W. Determination of Viscosity of Sulphur Dioxide and Its Mixtures with Hydrogen. *Ann. Phys. (Leipzig)* **1925**, *78*, 305–369.

(1198)	Smith, C. J. The Viscosity and Molecular Dimensions of Sulfur Dioxide. *Philos. Mag. Ser. 6* **1922**, *44*, 508.

(1199)	Shimotake, H.; Thodos, G. Viscosity of Sulfur Dioxide at 200OC. for Pressures Up to 3500 P.S.I. *J. Chem. Eng. Data* **1963**, *8*, 88–90. https://doi.org/10.1021/je60016a026.

(1200)	Chang, K. C.; Hesse, R. J.; Raw, C. J. G. Transport Properties of Polar Gas Mixtures SO2+ SO2F2 Mixtures. *Trans. Faraday Soc.* **1970**, *66*, 590–596. https://doi.org/10.1039/tf9706600590.

(1201)	Chakraborti, P. K.; Gray, P. Viscosities of Gaseous Mixtures Containing Polar Gases: More than One Polar Constituent. *Trans. Faraday Soc.* **1966**, *62*, 1769–1775. https://doi.org/10.1039/tf9666201769.

(1202)	Chakraborti, P. K.; Gray, P. Viscosities of Gaseous Mixtures Containing Polar Gases Polar Constituent. *Trans. Faraday Soc.* **1965**, *61*, 2422.

(1203)	Stakelbeck, H. *Z. Gesamte Kalte-Ind.* **1933**, *40*, 33.

(1204)	Lucatu, E. Relation between Vapor Pressure, Viscosity and Molecular Association. *C. R. Hebd. Seances Acad. Sci.* **1938**, *207*, 1403–1405.

(1205)	Adams, H. E.; Rogers, H. E. The Viscosity of Solutions of Sulfur Dioxide in Organic Liquids. *J. Am. Chem. Soc.* **1939**, *61*, 112–115. https://doi.org/10.1021/ja01870a038.

(1206)	Cupp, S. B.; Rogers, H. E. The Viscosities of Solutions of Surfur Dioxide in Organic Liquids. *J. Am. Chem. Soc.* **1939**, *61*, 3353–3354. https://doi.org/10.1021/ja01267a035.

(1207)	Scheuer, O. *Anz. Akad. Wiss. Math.-Natur. Kl.* **1911**, *48*, 304–308.

(1208)	Clifford, A. A.; Gray, P.; Johns, A. I.; Scott, A. C.; Watson, J. T. R. Thermal Conductivity of Argon, Nitrogen, and Hydrogen between 300 and 400 K and up to 25 MPa. *J. Chem. Soc., Faraday Trans. 1* **1981**, *77*, 2679–2691. https://doi.org/10.1039/f19817702679.

(1209)	Salomon, M. Electrolyte Solvation in Aprotic Solvents. *J. Power Src.* **1989**, *26*, 9–21. https://doi.org/10.1016/0378-7753(89)80010-2.

(1210)	Iwasaki, H.; Takahashi, M. The Viscosity of Gaseous Sulfur Dioxide. *Bull. Chem. Soc. Jpn.* **1975**, *48*, 988–992. https://doi.org/10.1246/bcsj.48.988.

(1211)	Litkenhous, E. E.; Van Arsdale, J. D.; Hutchison, I. W. The Physical Properties of the Ternary System Butyl Alcohol-Ethyl Acetate- Toluene. *J. Phys. Chem.* **1940**, *44*, 377–388.

(1212)	Xu, Z.; Meng, X.; Wu, J.; Liu, Z. Development of a Vibrating-Wire Viscometer and Measurement of Saturated Liquid Viscosity of Methyl Tert-Butyl Ether. *J. Eng. Thermophys.* **2007**, *28*, 4;189.

(1213)	Il’in; Ivanov, V. N. Measurement of Absolute Internal-Friction Coefrficients by the Pendulum Viscometer Method. *Kolloid Z.* **1935**, *71*, 267.

(1214)	Zeidler, O.; Schuster. *Farben-Ztg.* **1942**, *47*, 318.

(1215)	Akhundov, T. S.; Ismail-Zade, Sh. M.; Tairov, A. D. Experimental Investigation of the Viscosity of Toluene at High Pressure and Temperature. *Izv. Vyssh. Uchebn. Zaved., Neft Gaz* **1970**, 79.

(1216)	Katz, M.; Lobo, P. W.; Minano, A. S.; Solimo, H. Viscosities, Densities, and Refractive Indices of Binary Liquid Mixt. *Can. J. Chem.* **1971**, *49*, 2605–2609. https://doi.org/10.1139/v71-429.

(1217)	Pugachevich, N. N.; Martirosyan, A. L.; Kamalyan, R. M.; Lavygin, I. A. Viscosity and Density of Solution of Polydimethylsiloksane in Toluene. *Zh. Fiz. Khim.* **1975**, *49*, 130–133.

(1218)	Akhundov, T. S.; Abdullaev, F. G.; Ramazanzade, M. G. Experimental Study of the Viscosity of Toluene in Gaseous Phase and in the Critical Region. *Izv. Vyssh. Uchebn. Zaved., Neft Gaz* **1983**, 53–54.

(1219)	Ritzoulis, G.; Papadopoulos, N.; Jannakoudakis, D. Densities, Viscosities, and Dielectric Constatns of Acetonitrile + Toluene at 15, 25, and 35 Deg. C. *J. Chem. Eng. Data* **1986**, *31*, 146–148. https://doi.org/10.1021/je00044a004.

(1220)	Byers, C. H.; Williams, D. F. Visosities of Pure Polyatomic Hydrocarbons. *J. Chem. Eng. Data* **1987**, *32*, 344–348. https://doi.org/10.1021/je00049a018.

(1221)	Singh, R. P.; Sinha, C. P.; Das, J. C.; Ghosh, P. Viscosity and Density of Ternary Mixtures of Toluene, Bromobenzene, 1-Hexanol and 1-Octanol. *J. Chem. Eng. Data* **1990**, *35*, 93–97. https://doi.org/10.1021/je00059a025.

(1222)	Krall, A. H.; Sengers, J. V.; Kestin, J. Viscosity of Liquid Toluene at Temperatures from 25 to 150.Degree.C and at Pressures up to 30 MPa. *J. Chem. Eng. Data* **1992**, *37*, 349–355. https://doi.org/10.1021/je00007a021.

(1223)	Dymond, J. H.; Glen, N. F.; Isdale, J. D.; Pyda, M. The Viscosity of Liquid Toluene at Elevated Pressures. *Int. J. Thermophys.* **1995**, *16*, 877–882. https://doi.org/10.1007/bf02093470.

(1224)	Assael, M. J.; Dalouti, N. K.; Polimatidou, S. The Viscosity of Toluene in the Temperature Range from 210 to 370 K at Pressures up to 30 Mpa. *Int. J. Thermophys.* **1999**, *20*, 1367–1377. https://doi.org/10.1023/a:1021428819290.

(1225)	Harris, K. R. Temperature and Density Dependence of the Viscosity of Toluene. *J. Chem. Eng. Data* **2000**, *45*, 893–897. https://doi.org/10.1021/je000024l.

(1226)	Roos, M.; Bart, H.-J. Extraction of Acetic Acid with Tri-n-Octylamine: Physical Properties and Phase Equilibria. *J. Chem. Eng. Data* **2001**, *46*, 1198–1202. https://doi.org/10.1021/je000313+.

(1227)	Akhtar, S.; Hossain, K. M. S.; Saleh, M. A. VISCOSITY AND THERMODYNAMICS OF VISCOUS FLOW FOR THE SYSTEMS OF ISOMERIC PENTANOLS WITH TOLUENE. *Phys. Chem. Liq.* **2002**, *40*, 435–448.

(1228)	Cruz, R. d. C.; Martins, R. J.; Cardoso, M. E.; Barcia, O. E. Measurement of the Viscosity of Binary and Ternary Systems Containing Benzaldehyde, Toluene, and Benzyl Alcohol at 293.15 K and 0.1 MPa. *J. Chem. Eng. Data* **2002**, *47*, 927–931. https://doi.org/10.1021/je010327v.

(1229)	Rattan, V. K.; Kapoor, S.; Tochigi, K. Viscosities and Densities of Binary Mixtures of Toluene with Acetic Acid and Propionic Acid at (293.15, 303.15, 313.15, and 323.15) K. *J. Chem. Eng. Data* **2002**, *47*, 1182–1184. https://doi.org/10.1021/je020018k.

(1230)	Caetano, F. J. P.; Correia da Mata, J. L.; Fareleira, J. M. N. A.; Oliveira, C. M. B. P.; Wakeham, W. A. Viscosity Measurements of Liquid Toluene at Low Temperatures Using a Dual Vibrating-Wire Technique. *Int. J. Thermophys.* **2004**, *25*, 1–11. https://doi.org/10.1023/b:ijot.0000022325.10161.39.

(1231)	Kandil, M. E.; Marsh, K. N.; Goodwin, A. R. H. Vibrating Wire Viscometer with Wire Diameters of (0.05 and 0.15) Mm: Results for Methylbenzene and Two Fluids with Nominal Viscosities at T = 298 K and p = 0.01 MPa of (14 and 232) MPa*s at Temperatures between (298 and 373) K and Pressures below 40 MPa. *J. Chem. Eng. Data* **2005**, *50*, 647–655. https://doi.org/10.1021/je049636m.

(1232)	Nikam, P. S.; Kharat, S. J. Density and Viscosity Studies of Binary Mixtures of N,N-Dimethylformamide with Toluene and Methyl Benzoate at (298.15, 303.15, 308.15, and 313.15) K. *J. Chem. Eng. Data* **2005**, *50*, 455–459. https://doi.org/10.1021/je040012q.

(1233)	Hasan, M.; Shirude, D. F.; Hiray, A. P.; Kadam, U. B.; Sawant, A. B. Densities, Viscosities, and Speed of Sound Studies of Binary Mixtures of Methylbenzene with Heptan-1-Ol, Octan-1-Ol, and Decan-1-Ol at (303.15 and 313.15) K. *J. Chem. Eng. Data* **2006**, *51*, 1922–1926. https://doi.org/10.1021/je0602519.

(1234)	Tsierkezos, N. G.; Filippou, A. C. Thermodynamic Investigation of N,N-Dimethylformamide/Toluene Binary Mixtures in the Temperature Range from 278.15 to 293.15 K. *J. Chem. Thermodyn.* **2006**, *38*, 952–961. https://doi.org/10.1016/j.jct.2005.10.008.

(1235)	Hasan, M.; Shirude, D. F.; Hiray, A. P.; Kadam, U. B.; Sawant, A. B. Densities, Viscosities and Ultrasonic Velocity Studies of Binary Mixtures of Toluene with Heptan-1-Ol, Octan-1-Ol and Decan-1-Ol at 298.15 and 308.15 K. *J. Mol. Liq.* **2007**, *135*, 32–37. https://doi.org/10.1016/j.molliq.2006.10.012.

(1236)	Alonso, L.; Arce, A.; Francisco, M.; Soto, A. Liquid-Liquid Equilibria for [C8mim][NTf2] + Thiophene + 2,2,4-Trimethylpentane or + Toluene. *J. Chem. Eng. Data* **2008**, *53*, 1750–1755. https://doi.org/10.1021/je800071q.

(1237)	Narendra, K.; Srinivasu, C.; Kalpana, C.; Narayanamurthy, P. Excess Thermo Dynamical Parameters of Binary Mixtures of Toluene and Mesitylene with Anisaldehyde Using Ultrasonic Technique at Different Temperatures. *J. Therm. Anal. Calorim.* **2012**, *107*, 25–30. https://doi.org/10.1007/s10973-011-1521-x.

(1238)	Assael, M. J.; Mylona, S. A Novel Vibrating-Wire Viscometer for High-Viscosity Liquids at Moderate Pressures. *J. Chem. Eng. Data* **2013**, *58*, 993–1000. https://doi.org/10.1021/je301306e.

(1239)	Vijayalakshmi, D.; Rao, C. N.; Gowrisankar, M.; Sivakumar, K.; Venkateswarlu, P. Densities, Viscosities and Speeds of Sound of Binary Mixtures of Ethyl Benzoate with Toluene, and Isomeric Chlorotoluenes at Different Temperatures. *J. Mol. Liq.* **2014**, *197*, 272–286. https://doi.org/10.1016/j.molliq.2014.05.015.

(1240)	Bajic, D. M.; Zivkovic, E. M.; Jovanovic, J.; Serbanovic, S. P.; Kijevcanin, M. L. Experimental Measurements and Modelling of Volumetric Properties, Refractive Index and Viscosity of Binary Systems of Ethyl Lactate with Methyl Ethyl Ketone, Toluene and n-Methyl-2-Pirrolidone at 288.15 323.15 K and Atmospheric Pressure. New UNIFAC VISCO and ASOG VISCO Interaction Parameters. *Fluid Phase Equilib.* **2015**, *399*, 50–65. https://doi.org/10.1016/j.fluid.2015.04.017.

(1241)	Kerimov, A. M.; Agaev, N. A.; Abaszade, A. Experimental Study of the Viscosity of Water at Temperatures from 100 to 275 C and at High Pressures. *Teploenergetika* **1969**, 87–90.

(1242)	Rivkin, S. L.; Levin, A. Ya.; Izrailevskii, L. B. Experimental Study of the Viscosity of Water at Temperatures from 250 to 375 and at Pressures up to 50 MPa. *Teploenergetika* **1970**, 79–81.

(1243)	Kingham, D. J.; Adams, W. A.; McGuire, M. J. Viscosity Measurements of Water in Regioon of Its Maximum Density. *J. Chem. Eng. Data* **1974**, *19*, 1–3. https://doi.org/10.1021/je60060a028.

(1244)	Assael, M. J.; Polimatidou, S.; Wakeham, W. A. The Viscosity of Liquid Water at Pressures up to 32 MPa. *Int. J. Thermophys.* **1993**, *14*, 795–803. https://doi.org/10.1007/bf00502108.

(1245)	Jones, H. C.; Davis, P. B. *The Freezing-Point Lowering, Conductivity, and Viscosity of Solutions of Certain Electrolytes in Water, Methyl Alcohol, Ethyl Alcohol, Acetone, and Glycerol, and in Mixtures of These Solvents with On*; 180; Carnegie Inst. Monograph: Washington, 179-1, 1913.

(1246)	Bingham; Jackson. *Bur. Stand. (U. S.), Bull.* **1918**, *14*, 59.

(1247)	Bhagwat, W. V.; Mandloi, K. Application of Mixture Law to Rheochor. *J. Indian Chem. Soc.* **1946**, *23*, 349–352.

(1248)	Babak, S. F. Viscosity and Density of Binary Mixtures of Nicotine I. *Zh. Obshch. Khim.* **1949**, *19*, 1604.

(1249)	LeBel, R. G.; Goring, D. A. I. Density, Viscosity, Refractive Index, and Hygroscopicity of Mixtures of Water and Dimethyl Sulfoxide. *J. Chem. Eng. Data* **1962**, *7*, 100–101. https://doi.org/10.1021/je60012a032.

(1250)	Wallace, W. J.; Mathews, A. L. Densities, Refractive Indeices, Molar Refractions, and Viscosities of Ethylene Glycol Dimethyl Ether-Water Solutions at 25 C. *J. Chem. Eng. Data* **1963**, *8*, 496–498. https://doi.org/10.1021/je60019a006.

(1251)	Wallace, W. J.; Mathews, A. L. Density, Refractive Indices, Molar Refractions, and Viscosities of Diethylene Glycol Dimethyl Ether-Water Solutions at 25 C. *J. Chem. Eng. Data* **1964**, *9*, 267–268. https://doi.org/10.1021/je60021a044.

(1252)	Blumenshine, R. L.; Sears, P. G. Several Properties of the 2-Pyrrolidone-Water System as Functions of Composition and Temperature. *J. Chem. Eng. Data* **1966**, *11*, 141–143. https://doi.org/10.1021/je60029a002.

(1253)	Rivkin, S. L.; Levin, A. Ya. Experimental Study of the Viscosity of Water and Vapor. *Teploenergetika* **1966**, 79–82.

(1254)	Wallace, W. J.; Shephard, C. S.; Underwood, C. Densities, Refractive Indicies, Molar Refractions, Viscosities and Dielectric Constants of Triethylene Glycol Dimethyl Ether-Water Solutions at 25C. *J. Chem. Eng. Data* **1968**, *13*, 11–13. https://doi.org/10.1021/je60036a004.

(1255)	Hayduk, W.; Laudie, H.; Smith, O. H. Viscosity, Freezing Point, Vapor-Liquid Equilibria, and Other Properties of Aqueous-Tetrahydrofuran Solutions. *J. Chem. Eng. Data* **1973**, *18*, 373–376. https://doi.org/10.1021/je60059a027.

(1256)	Deckwer, W. Density, Viscosity, Vapor Pressure, and Hydrogen Solubility of Aqueous MnSO4 Solutionos. *J. Chem. Eng. Data* **1980**, *25*, 75–76. https://doi.org/10.1021/je60084a022.

(1257)	Sacco, A.; Petrella, G.; Castagnolo, M.; Dell’Atti, A. Excess Volumes and Viscosity of Water-Sulfolane Mixtures at 30, 40 and 50. Degree.C. *Thermochim. Acta* **1981**, *44*, 59–66. https://doi.org/10.1016/0040-6031(81)80271-7.

(1258)	Collings, A. F.; Bajenov, N. A High Precision Capillary Viscometer and Further Relative Results for the Viscosity of Water. *Metrologia* **1983**, *19*, 61.

(1259)	James, C. J.; Mulcahy, D. E.; Steel, B. J. Viscometer Calibration Standards: Viscosities of Water Between 0 and 60 Deg C and of Selected Aqueous Sucrose Sulutions at 15 Deg C from Measurements with A Flared Capillary Viscometer. *J. Phys. D: Appl. Phys.* **1984**, *17*, 225–230. https://doi.org/10.1088/0022-3727/17/2/006.

(1260)	Guarino, G.; Ortona, O.; sartorio, R.; Vitagliano, V. Diffusion, Viscosity, and Refractivity Data of the Systems Dimethyl- Formamide-Water and N-Methylpyrrolidone-Water at 5 C. *J. Chem. Eng. Data* **1985**, *30*, 366–368. https://doi.org/10.1021/je00041a039.

(1261)	Al-Ghawas, H. A.; Hagewiesche, D. P.; Ruiz-Ibanez, G.; Sandall, O. C. Physicochemical Properties Important for Carbon Dioxide Absorption in Aqueous Methyldiethanolamine. *J. Chem. Eng. Data* **1989**, *34*, 385–391. https://doi.org/10.1021/je00058a004.

(1262)	Palepu, R.; Clarke, J. Viscosities and Densities of 2,2,2-Trifluoroethanol + Water at Various Temperatures. *Thermochim. Acta* **1989**, *156*, 359–363. https://doi.org/10.1016/0040-6031(89)87202-8.

(1263)	Pandey, J. D.; Shukla, A.; Rai, R. D.; Misra, K. Ultrasonic, Volumetric, and Viscometric Studies of Tetracycline and Its Allied Compound. *J. Chem. Eng. Data* **1989**, *34*, 29–31. https://doi.org/10.1021/je00055a010.

(1264)	Laachach, A.; Ferriol, M.; Cohen-Adad, M. T.; Huck, J.; Jorat, L.; Noyel, G.; Getzen, F. W.; Bureau, J. C. Densities, Molar Volumes and Viscosities in the Systems Water-Hydrazine, Water-Methylhydrazine and Water-1,1- Dimethylhydrazine. *Fluid Phase Equilib.* **1992**, *71*, 301–312. https://doi.org/10.1016/0378-3812(92)80005-t.

(1265)	Liu, W.; Wang, J.; Wang, C.; Lu, J. Viscosity of Amino Acids in Methanol - Water Mixtures. *Wuli Huaxue Xuebao* **1992**, *8*, 742–748.

(1266)	Das, B.; Hazra, D. K. Viscosities and Excess Molar Volumes for 2-Methoxy-1-Ethanol + Water at Different Temperatures. *J. Chem. Eng. Data* **1993**, *38*, 361–363. https://doi.org/10.1021/je00011a007.

(1267)	Lee, M. J.; Hwang, S. M.; Kuo, Y. C. Densities and Viscosities of Binary Solutions Containing Butylamine, Benzylamine, and Water. *J. Chem. Eng. Data* **1993**, *38*, 577–579. https://doi.org/10.1021/je00012a026.

(1268)	Solimo, H. N.; Gramajo de Doz, M. B. Liquid-Liquid Equilibrium, Densities, Viscosities, Refractive Indices, and Excess Properties of the Ternary System Water + 4-Hydroxy-4-Methyl-2- Pentanone + Benzyl Alcohol at 298.15 K. *J. Chem. Eng. Data* **1995**, *40*, 563–566. https://doi.org/10.1021/je00019a004.

(1269)	Pal, A.; Singh, Y. P. Excess Molar Volumes and Viscosities for Glycol Ether-Water Solutions at the Temperature 308.15 K: Ethylene Glycol Monomethyl, Diethylene Glycol Monomethyl, and Triethylene Glycol Monomethyl Ethers. *J. Chem. Eng. Data* **1996**, *41*, 425–427. https://doi.org/10.1021/je950198g.

(1270)	Moha-Ouchane, M.; Boned, C.; Allal, A.; Benseddik, M. Viscosity and Excess Volume at High Pressures in Associative Binaries. *Int. J. Thermophys.* **1998**, *19*, 161–189. https://doi.org/10.1023/a:1021455203728.

(1271)	Henni, A.; Tontiwachwuthlkul, P.; Chakma, A.; Mather, A. E. Densities and Viscosities of Triethylene Glycol Monomethyl Ether +Water Solutions in the Temperature Interval 25 Deg C - 80 Deg C. *J. Chem. Eng. Data* **1999**, *44*, 101–107. https://doi.org/10.1021/je980127s.

(1272)	Pai, Y.-H.; Chen, L.-J. Liquid-Liquid Equilibria of Two Binary Systems: Water + 1-Pentanol and Water + 2-Methyl-2-Butanol and Two Ternary Systems: Water + 1 -Pentanol + 2-Butyloxyethanol and Water + 2-Methyl-2-Butanol + 2-Butyloxyethanol. *Fluid Phase Equilib.* **1999**, *155*, 95–105. https://doi.org/10.1016/s0378-3812(98)00456-7.

(1273)	Romero, C. M.; Rojas, J. L. Apparent Molar Volumes and Viscosities of DL-Alpha-Alanine in Water-Ethanol Mixtures. *Phys. Chem. Chem. Phys.* **2000**, *38*, 237–243.

(1274)	Akhtar, S.; Faruk, A. N. M. O.; Saleh, M. A. Viscosity of Aqueous Solutions of Formamide, N-Methylformamide, and N,N-Dimethylformamide. *Phys. Chem. Liq.* **2001**, *39*, 383–399.

(1275)	Henni, A.; Tontiwachwuthlkul, P.; Chakma, A.; Mather, A. E. Volumetric Properties and Viscosities for Aqueous Diglycolamine Solutions from 25 Deg C to 70 Deg C. *J. Chem. Eng. Data* **2001**, *46*, 56–62. https://doi.org/10.1021/je000136c.

(1276)	Saleh, M. A.; Akhtar, S.; Ahmed, M. S.; Uddin, M. H. Viscosities of Aqueous Solutions of Dimethylsulfoxide, 1,4-Dioxane, and Tetrahydrofuran. *Phys. Chem. Liq.* **2001**, *39*, 551–563.

(1277)	Maham, Y.; Lebrette, L.; Mather, A. E. Viscosities and Excess Properties of Aqueous Solutions of Mono-and Diethylethanolamines at Temperatures between 298.15 and 353.15 K. *J. Chem. Eng. Data* **2002**, *47*, 550–553. https://doi.org/10.1021/je015528d.

(1278)	Palaiologou, M. M.; Molinou, I. E.; Tsierkezos, N. G. Viscosity Studies on Lithium Bromide in Water + Dimethyl Sulfoxide Mixtures at 278.15 K and 293.15 K. *J. Chem. Eng. Data* **2002**, *47*, 1285–1289. https://doi.org/10.1021/je020063s.

(1279)	Saleh, M. A.; Ahmed, M. S.; Islam, M. S. VISCOSITY AND EXCESS VISCOSITY OF DILUTE AQUEOUS SOLUTIONS OF ETHYLENEDIAMINE, TRIMETHYLENEDIAMINE AND N,NDIMETHYLTRIMETHYLENEDIAMINE. *Phys. Chem. Liq.* **2002**, *40*, 477–490.

(1280)	Abdulagatov, I. M.; Azizov, N. D. Viscosity for Aqueous Li2SO4 Solutions at Temperatures from 298 to 575 K and at Pressures up to 30 MPa. *J. Chem. Eng. Data* **2003**, *48*, 1549–1556. https://doi.org/10.1021/je030177j.

(1281)	Henni, A.; Hromek, J.; Tontiwachwuthlkul, P.; Chakma, A. Volumetric Properties and Viscosities for Aqueous AMP Solutions from 25 0C to 70 0C. *J. Chem. Eng. Data* **2003**, *48*, 551–556. https://doi.org/10.1021/je0201119.

(1282)	Kapadi, U. R.; Hundiwale, D. G.; Patil, N. B.; Lande, M. K. Effect of Temperature on Excess Molar Volumes and Viscosities of Binary Mixtures of Ethylenediamine and Water. *Fluid Phase Equilib.* **2003**, *205*, 267–274. https://doi.org/10.1016/s0378-3812(02)00293-5.

(1283)	Uchida, T.; Ohmura, R.; Nagao, J.; Takeya, S.; Ebinuma, T.; Narita, H. Viscosity of Aqueous CO2 Solutions Measured by Dynamic Light Scattering. *J. Chem. Eng. Data* **2003**, *48*, 1225–1229. https://doi.org/10.1021/je034041x.

(1284)	Henni, A.; Tontiwachwuthikul, P.; Chakma, A. Volumetric Properties and Viscosities for Aqueous NFM Solutions from 25 Deg C to 70 Deg C. *J. Chem. Eng. Data* **2004**, *49*, 1724–1726. https://doi.org/10.1021/je0498208.

(1285)	Li, X.-X.; Liu, Y.-X.; Wei, X.-H. Physical-Chemical Properties and Liquid-Liquid Equilibria for Binary Mixture of Diethylene Glycol Diethyl Ether with Water. *J. Chem. Eng. Data* **2004**, *49*, 1043–1045. https://doi.org/10.1021/je049932z.

(1286)	Abdulagatov, I. M.; Zeinalova, A.; Azizov, N. D. Viscosity of Aqueous Na2SO4 Solutions at Temperatures from 298 to 573K and at Pressures up to 40MPa. *Fluid Phase Equilib.* **2005**, *227*, 57–70. https://doi.org/10.1016/j.fluid.2004.10.028.

(1287)	Bernal-Garcia, J. M.; Hall, K. R.; Estrada-Baltazar, A.; Iglesias-Silva, G. A. Density and Viscosity of Aqueous Solutions of N,N-Dimethylethanolamine at p = 0.1 MPa from T = (293.15 to 363.15) K. *J. Chem. Thermodyn.* **2005**, *37*, 762–767. https://doi.org/10.1016/j.jct.2004.11.016.

(1288)	Liu, W.; Zhao, T.; Zhang, Y.; Wang, H.; Yu, M. The Physical Properties of Aqueous Solutions of the Ionic Liquid [BMIM][BF4]. *J. Solution Chem.* **2006**, *35*, 1337–1346.

(1289)	Pang, F.-M.; Seng, C.-E.; Teng, T.-T.; Ibrahim, M. H. Densities and Viscosities of Aqueous Solutions of 1-Propanol and 2-Propanol at Temperatures from 293.15 K to 333.15 K. *J. Mol. Liq.* **2007**, *136*, 71–78. https://doi.org/10.1016/j.molliq.2007.01.003.

(1290)	Abdulagatov, I.; Azizov, N. Experimental Study of the Effect of Temperature, Pressure, and Concentration on the Viscosity of Aqueous KBr Solutions. *J. Solution Chem.* **2008**, *37*, 3–26.

(1291)	Abu-Daabes, M. A.; Awwad, A. M. Volumetric and Viscometric Properties of Aqueous Solutions of N-(2-Hydroxyethyl)Morpholine at T = (293.15, 303.15, 313.15, 323.15, 333.15) K. *J. Chem. Thermodyn.* **2008**, *40*, 874–878. https://doi.org/10.1016/j.jct.2008.01.007.

(1292)	Nikam, P. S.; Aher, J. S.; Kharat, S. J. Viscosities of Ammonium Sulfate, Potassium Sulfate, and Aluminum Sulfate in Water and Water + N,N-Dimethylformamide Mixtures at Different Temperatures. *J. Chem. Eng. Data* **2008**, *53*, 2469–2472. https://doi.org/10.1021/je800330d.

(1293)	Besbes, R.; Ouerfelli, N.; Latrous, H. Density, Dynamic Viscosity, and Derived Properties of Binary Mixtures of 1,4 Dioxane with Water at T=298.15 K. *J. Mol. Liq.* **2009**, *145*, 1–4. https://doi.org/10.1016/j.molliq.2008.09.009.

(1294)	Hartono, A.; Svendsen, H. F. Density, Viscosity, and Excess Properties of Aqueous Solution of Diethylenetriamine (DETA). *J. Chem. Thermodyn.* **2009**, *41*, 973–979. https://doi.org/10.1016/j.jct.2008.11.012.

(1295)	Palani, R.; Geetha, A. Acoustical and Excess Thermodynamic Studies of Molecular Interaction in Aqueous Mixed Solvent Systems at 303, 308 and 313K. *Phys. Chem. Liq.* **2009**, *47*, 542–552. https://doi.org/10.1080/00319100802562862.

(1296)	Sadeghi, R.; Goodarzi, B.; Karami, K. Effect of Potassium Citrate Salts on the Transport Behavior of L-Alanine in Aqueous Solutions at T = (293.15 to 308.15) K. *J. Chem. Eng. Data* **2009**, *54*, 791–794. https://doi.org/10.1021/je800559d.

(1297)	Wang, L.-C.; Ding, H.; Zhao, J.-H.; Song, C.-Y.; Wang, J.-S. Density and Viscosity of (4-Picoline + Water) Binary Mixtures from T ) (298.15 to 338.15) K. *J. Chem. Eng. Data* **2009**, *54*, 1000–1003. https://doi.org/10.1021/je8008182.

(1298)	Blanco, A.; Garcia-Abuin, A.; Gomez-Diaz, D.; Navaza, J. M.; Vidal-Tato, I. Influence of Temperature and Composition upon Density, Viscosity, Speed of Sound, and Refractive Index of Aqueous Solutions of 1-Ethyl-2-Pyrrolidinone. *J. Chem. Eng. Data* **2010**, *55*, 962–965. https://doi.org/10.1021/je9003959.

(1299)	Qiao, Y.; Di, Z.; Ma, P. S.; Ma, Y.; Xia, S. Viscosities of Pure Water, Acetic Acid + Water, and p-Xylene + Acetic Acid + Water at Different Temperature and Pressure*. *Chin. J. Chem. Eng.* **2010**, *18*, 446–454.

(1300)	Dhondge, S. S.; Pandhurnekar, C.; Garade, S.; Dadure, K. Volumetric and Transport Behavior of Different Carbohydrates in Aqueous and Aqueous Urea Mixtures at Different Temperatures. *J. Chem. Eng. Data* **2011**, *56*, 3484–3491. https://doi.org/10.1021/je2000099.

(1301)	Goodwin, A. R. H.; Marsh, K. M. An Absolute Viscometer for Liquids: Measurement of the Viscosity of Water at T = 298.15 K and p = 0.1 MPa. *J. Chem. Eng. Data* **2011**, *56*, 167–170. https://doi.org/10.1021/je100990n.

(1302)	Nain, A. K.; Lather, M.; Sharma, R. K. Volumetric, Ultrasonic and Viscometric Behavior of l-Methionine in Aqueous-Glucose Solutions at Different Temperatures. *J. Mol. Liq.* **2011**, *159*, 180–188.

(1303)	Rajagopal, K.; Gladson, S. E. THERMODYNAMIC INTERACTIONS OF POTASSIUM FLUORIDE IN AQUEOUS DIMETHYL SULFOXIDE SOLUTIONS AT DIFFERENT TEMPERATURES. *Thermochim. Acta* **2011**, *525*, 197–205. https://doi.org/10.1016/j.tca.2011.08.013.

(1304)	Lu, J.-G.; Hua, A.-C.; Xu, Z.-W.; Fan, F.; Cheng, L.; Lin, F.-Y. Measurement and Prediction of Densities, Viscosities, and Surface Tensions for Aqueous Solutions of Potassium Citrate. *Fluid Phase Equilib.* **2012**, *327*, 9–13. https://doi.org/10.1016/j.fluid.2012.05.007.

(1305)	Ouerfelli, N.; Barhoumi, Z.; Iulian, O. Viscosity Arrhenius Activation Energy and Derived Partial Molar Properties in 1,4-Dioxane +Water Binary Mixtures from 293.15 to 323.15 K. *J. Solution Chem.* **2012**, *41*, 458–474. https://doi.org/10.1007/s10953-012-9812-9.

(1306)	Ren, N.; Wu, Y.-T.; Ma, C.-F. Experimental Study of Viscosity of New Kind of Molten Salt With Low Melting Point. *J. Eng. Thermophys.* **2012**, *33*, 497–500.

(1307)	Bald, A.; Kinart, Z.; Wypych-Stasiewicz, A.; Tomas, R. Conductance Studies of NaCl, KCl, NaBr, KBr, Bu4NBr, and NaBPh4 in Water + 2-Methoxyethanol Mixtures at 298.15 K. *J. Mol. Liq.* **2013**, *182*, 14–24. https://doi.org/10.1016/j.molliq.2013.02.017.

(1308)	Banipal, T. S.; Singh, H.; Banipal, P. K.; Singh, V. Volumetric and Viscometric Studies on L-Ascorbic Acid, Nicotinic Acid, Thiamine Hydrochloride and Pyridoxine Hydrochloride in Water at Temperatures (288.15 to 318.15) K and at Atmospheric Pressure. *Thermochim. Acta* **2013**, *553*, 31–39. https://doi.org/10.1016/j.tca.2012.10.017.

(1309)	Chandra, A.; Patidar, V.; Singh, M.; Kale, R. K. Physicochemical and Friccohesity Study of Glycine, L-Alanine and L-Phenylalanine with Aqueous Methyltrioctylammonium and Cetylpyridinium Chloride from T = (293.15 to 308.15) K. *J. Chem. Thermodyn.* **2013**, *65*, 18–28. https://doi.org/10.1016/j.jct.2013.05.037.

(1310)	Dhondge, S. S.; Deshmukh, D. W.; Paliwal, L. J.; Dahasahasra, P. N. The Study of Molecular Interactions of Ascorbic Acid and Sodium Ascorbate with Water at Temperatures (278.15, 288.15 and 298.15) K. *J. Chem. Thermodyn.* **2013**, *67*, 217–226. https://doi.org/10.1016/j.jct.2013.08.016.

(1311)	Jozwiak, M.; Tyczynska, M.; Bald, A. Viscosity of Urea in the Mixture of N,N-Dimethylformamide and Water. *J. Chem. Eng. Data* **2013**, *58*, 217–224. https://doi.org/10.1021/je300608v.

(1312)	Siongco, K. R.; Leron, R. B.; Li, M.-H. Densities, Refractive Indices, and Viscosities of N,N-Diethylethanol Ammonium Chloride - Glycerol or - Ethylene Glycol Deep Eutectic Solvents and Their Aqueous Solutions. *J. Chem. Thermodyn.* **2013**, *65*, 65–72. https://doi.org/10.1016/j.jct.2013.05.041.

(1313)	Andanson, J.-M.; Traikia, M.; Husson, P. Ionic Association and Interactions in Aqueous Methylsulfate Alkyl-Imidazolium-Based Ionic Liquids. *J. Chem. Thermodyn.* **2014**, *77*, 214–221. https://doi.org/10.1016/j.jct.2014.01.031.

(1314)	Dhondge, S. S.; Dahasahasra, P. N.; Paliwal, L. J.; Deshmukh, D. W. Density and Viscosity Study of Nicotinic Acid and Nicotinamide in Dilute Aqueous Solutions at and around the Temperature of the Maximum Density of Water. *J. Chem. Thermodyn.* **2014**, *76*, 16–23. https://doi.org/10.1016/j.jct.2014.02.024.

(1315)	He, X.; Shao, Q.; Kong, W.; Yu, L.; Zhang, X.; Deng, Y. A Simple Method for Estimating Mutual Diffusion Coefficients of Ionic Liquids-Water Based on an Optofluidic Chip. *Fluid Phase Equilib.* **2014**, *366*, 9–15. https://doi.org/10.1016/j.fluid.2014.01.003.

(1316)	Jiang, X.; Zhu, C.; Ma, Y. Volumetric and Viscometric Studies of Amino Acids in L-Ascorbic Acid Aqueous Solutions at T = (293.15 to 323.15) K. *J. Chem. Thermodyn.* **2014**, *71*, 50–63. https://doi.org/10.1016/j.jct.2013.11.002.

(1317)	Krolikowska, M.; Lipinski, P.; Maik, D. Density, Viscosity and Phase Equilibria Study of (Ethylsulfate - Based Ionic Liquid + Water) Binary Systems as a Function of Temperature and Composition. *Thermochim. Acta* **2014**, *582*, 1–9. https://doi.org/10.1016/j.tca.2014.02.019.

(1318)	Kumar, H.; Singla, M.; Katal, A. Effect of N-Acetylglycine on Volumetric, Acoustic and Viscometric Behaviour of Aqueous Amoxicillin Solutions. *Thermochim. Acta* **2014**, *583*, 49–58. https://doi.org/10.1016/j.tca.2014.03.013.

(1319)	Mehrdad, A.; Shekaari, H.; Niknam, Z. Viscometric Studies of Interactions between Ionic Liquid 1-Octyl-3-Methyl-Imidazolium Bromide and Polyvinyl Pyrrolidone in Aqueous Solutions. *J. Chem. Thermodyn.* **2014**, *79*, 1–7. https://doi.org/10.1016/j.jct.2014.07.005.

(1320)	Navarro, S. S.; Leron, R. B.; Soriano, A. N.; Li, M.-H. Thermophysical Property Characterization of Aqueous Amino Acid Salt Solution Containing Serine. *J. Chem. Thermodyn.* **2014**, *78*, 23–31. https://doi.org/10.1016/j.jct.2014.05.019.

(1321)	Zawadzki, M.; Krolikowska, M.; Lipinski, P. Physicochemical and Thermodynamic Characterization of N-Alkyl-N-Methylpyrrolidinium Bromides and Its Aqueous Solutions. *Thermochim. Acta* **2014**, *589*, 148–157. https://doi.org/10.1016/j.tca.2014.05.028.

(1322)	Garcia, A. A.; Leron, R. B.; Soriano, A. N.; Li, M.-H. Thermophysical Property Characterization of Aqueous Amino Acid Salt Solutions Containing .Alpha.-Aminobutyric Acid. *J. Chem. Thermodyn.* **2015**, *81*, 136–142. https://doi.org/10.1016/j.jct.2014.10.005.

(1323)	Kumar, K.; Chauhan, S. Volumetric, Compressibility and Viscometric Studies on Sodium Cholate/Sodium Deoxycholate Amino Acid Interactions in Aqueous Medium. *Thermochim. Acta* **2015**, *606*, 12–24. https://doi.org/10.1016/j.tca.2015.02.014.

(1324)	Raman, M. S.; Kesavan, M.; Senthilkumar, K.; Ponnuswamy, V. Ultrasonic, DFT and FT-IR Studies on Hydrogen Bonding Interactions in Aqueous Solutions of Diethylene Glycol. *J. Mol. Liq.* **2015**, *202*, 115–124. https://doi.org/10.1016/j.molliq.2014.12.014.

(1325)	Xu, Q.; Sun, S.; Lan, G.; Xiao, J.; Zhang, J.; Wei, X. Excess Properties and Spectral Investigation for the Binary System Diethylene Glycol Dimethyl Ether + Water at T = (293.15, 298.15, 303.15, 308.15 and 313.15) K. *J. Chem. Eng. Data* **2015**, *60*, 2–10. https://doi.org/10.1021/je500389t.

(1326)	Shifrin, A. S. Viscosity of Water Steam at Atmospheric Pressure. *Teploenergetika* **1959**, 22–27.

(1327)	Rivkin, S. L.; Levin, A. Ya.; Izrailevskii, L. B. Experimental Study of Viscosity of Water at Temperatures to 450 C and at Pressures up to 350 Bar. *Teploenergetika* **1968**, 74–78.

(1328)	Zhdanov, A. G.; Lusternik, V. E.; Timrot, D. L. Capillary Viscometer for the Viscosity Determination of Vapor by Closed Scheme. *Inzh.-Fiz. Zh.* **1968**, *15*, 1120–1123.

(1329)	Timrot, D. L.; Serednitskaya, M. A.; Bespalov, M. S. Experimental Study of the Viscosity of Water Vapor at Temperatures from 50 to 500 C and at Pressures from 0.06 to 1.5 Bar. *Teploenergetika* **1973**, 78–80.

(1330)	Nagashima, A.; Tanishita, I.; Mural, Y. Measurement of Pressure Effect on Viscosity of Steam. *J. Chem. Eng. Data* **1974**, *19*, 212–213. https://doi.org/10.1021/je60062a021.

(1331)	Timrot, D. L. *Par Vysokih Parametrov v Energetike (Steam of High Parameters in Energetics)*; 1950.

(1332)	Mineev, V. N.; Zaidel, R. M. Viscosity of Water and Mercury under Shok Load. *Zh. Eksp. Teor. Fiz.* **1968**, *54*, 1633–1639.

(1333)	Rivkin, S. L.; Levin, A. Ya.; Izrailevskii, L. B.; Kharitonov, K. G. Experimental Study of the Viscosity of Water in the Supercrtical Region of Parameters State at Pressures up to 500 Bar and at Temperatures to 500 C. *Teploenergetika* **1973**, 11–12.

(1334)	Liu, X. Y.; He, M. G.; Zhang, Y. Viscosity of Water in the Region around the Critical Point. *J. Supercrit. Fluids* **2012**, *63*, 150–154. https://doi.org/10.1016/j.supflu.2011.11.021.

(1335)	Teske, V.; Vogel, E.; Bich, E. Viscosity Measurements on Water Vapor and Their Evaluation. *J. Chem. Eng. Data* **2005**, *50*, 2082–2087. https://doi.org/10.1021/je050288d.

(1336)	Vogel, E. Precise Measurements of the Viscosity Coefficients of Nitrogen and Rare Gases between Room Temperature and 650 K. *Ber. Bunsen-Ges. Phys. Chem.* **1984**, *88*, 997–1002.

(1337)	Trautz, M.; Heberling, R. The Friction, Thermal Conduction and Diffusion in Gas Mixtures. XXV. The Internal Friction of Xenon and Its Mixtures with Hydrogen and Helium. *Ann. Phys. (Leipzig)* **1934**, *412*, 118–120. https://doi.org/10.1002/andp.19344120203.

(1338)	Trappeniers, N. J.; Botzen, A.; Van Oosten, J.; Van den Berg, H. R. THE VISCOSITY OF KRYPTON BETWEEN 25deg AND 75deg C AND AT PRESSURES UP TO 2000 ATM. *Phys. Norv.* **1965**, *31*, 945–952. https://doi.org/10.1016/0031-8914(65)90024-8.

(1339)	Reynes, E. G.; Thodos, G. The Viscosities of Argon, Krypton, and Xenon in the Dense Gaseous Region. *Physica (Amsterdam)* **1964**, *30*, 1529–1542. https://doi.org/10.1016/0031-8914(64)90177-6.

(1340)	Rankine, A. O. On the Viscosities of the Gases of the Argon Group. *Proc. Roy. Soc. London Ser. A* **1910**, *83*, 516–525. https://doi.org/10.1098/rspa.1910.0040.

(1341)	Nasini, A. G.; Rossi, C. Viscosita Di Gas Rari. *Gazz. Chim. Ital.* **1928**, *58*, 433–442.

(1342)	Boon, J. P.; Legros, J. C.; Thomaes, G. ON THE PRINCIPLE OF CORRESPONDING STATES FOR THE VISCOSITY OF SIMPLE LIQUIDS. *Physica (Amsterdam)* **1967**, *33*, 547–557.

(1343)	Slyusar, V. P.; Rudenko, N. S.; Tretyakov, V. M. Eksperimentalnoe Issledovanie Vyazkosti Prostykh Veshchestv Na Linii Nasyshcheniya i Pod Davleniem. *Ukr. Fiz. Zh. Russ. Ed.* **1972**, *17*, 1257–1263.

(1344)	Roy, C. S.; Rakshit, A. B.; Barua, A. K. Viscosity of Gas Mixtures and Polar-Nonpolar Molecular Interactions. *Physica (Amsterdam)* **1973**, *66*, 442–454. https://doi.org/10.1016/0031-8914(73)90293-0.

(1345)	Thomas, L. H. Viscosity and Molecular Association. III. Association of Phenols and Amides. *J. Chem. Soc.* **1960**, 4906–4914. https://doi.org/10.1039/jr9600004906.

(1346)	Rigby, M.; Smith, E. B. Viscosities of the Inert Gases. *Trans. Faraday Soc.* **1966**, *62*, 54–58. https://doi.org/10.1039/tf9666200054.

(1347)	Maitland, G. C.; Smith, E. B. Critical Reassessment of Viscosities of 11 Common Gases. *J. Chem. Eng. Data* **1972**, *17*, 150–156. https://doi.org/10.1021/je60053a015.

(1348)	Goldblatt, M.; Wageman, W. E. High Temperature Viscosity Ratios for Xenon. *Phys. Fluids* **1971**, *14*, 1024–1025. https://doi.org/10.1063/1.1693533.

(1349)	May, E. F.; Berg, R. F.; Moldover, M. R. Reference Viscosities of H2, CH4, Ar, and Xe at Low Densities. *Int. J. Thermophys.* **2007**, *28*, 1085–1110. https://doi.org/10.1007/s10765-007-0198-7.

(1350)	Vogel, E.; Bastubbe, E.; Rohde, S. Calibration of an Oscillating Quartz Disc Viscometer for Precision Measurements of Gases and Organic Vapors. *Wiss. Z. Univ. Rostock math. naturw. Reihe* **1984**, *33*, 34–42.

(1351)	Rakshit, A. B.; Roy, C. S. Viscosity and Polar-Nonpolar Interactions in Mixtures of Inert Gases with Ammonia. *Phys. Norv.* **1974**, *78*, 153–164. https://doi.org/10.1016/0031-8914(74)90316-4.

(1352)	Kestin, J.; Khalifa, H. E.; Wakeham, W. A. The Viscosity and Diffusion Coefficients of the Binary Mixtures of Xenon with the Other Noble Gases. *Physica A Amsterdam* **1978**, *90*, 215–228. https://doi.org/10.1016/0378-4371(78)90110-3.

(1353)	Vogel, E. The Viscosities of Dilute Kr, Xe, and CO2 Revisited: New Experimental Reference Data at Temperatures from 295 K to 690 K. *Int. J. Thermophys.* **2016**, *37*, 63. https://doi.org/10.1007/s10765-016-2068-7.

(1354)	Lin, H.; Che, J.; Zhang, J. T.; Feng, X. J. Measurements of the Viscosities of Kr and Xe by the Two-Capillary Viscometry. *Fluid Phase Equilib.* **2016**, *418*, 198–203. https://doi.org/10.1016/j.fluid.2016.01.038.

(1355)	Golovicher, L. E.; Kolenchits, O. A.; Nesterov, N. A. DYNAMIC VISCOSITY OF GASES IN A WIDE RANGE OF TEMPERATURE. I. *Inzh. Fiz. Zh.* **1989**, *56*, 982–987. https://doi.org/10.1007/BF00870442.

(1356)	Barr, I. A.; Matthews, G. P.; Smith, E. B.; Tindell, A. R. Intermolecular Forces and the Gaseous Viscosities of Argon-Xenon Mixtures. *J. Phys. Chem.* **1981**, *85*, 3342–3347. https://doi.org/10.1021/j150622a027.

(1357)	Sevast’yanov, R. M.; Zykov, N. A. Transport Coefficients of the Monatomic Gases in the Temperature Range from 100 to 3000 K. *Inzh. Fiz. Zh.* **1978**, *34*, 118–125.
