# Supplementary material for: Entropy Scaling of Viscosity IV—Application to 124 Industrially Important Fluids
Source: J Chem Eng Data. 2025 Jan 10;70(2):727–42. doi: 10.1021/acs.jced.4c00451 (PMC11831593; doi:10.1021/acs.jced.4c00451)
Supplement: Supplementary file 1 — je4c00451_si_001.zip [file je4c00451_si_001.zip › supporting_information/SI title page.pdf]

# Supporting Information
# Entropy Scaling of Viscosity IV - Application to 124 Industrially Important Fluids

Viktor Martinek,† Ian Bell,‡ Roland Herzog,† Markus Richter,¶ and Xiaoxian Yang*,¶

†*Interdisciplinary Center for Scientific Computing, Heidelberg University, 69120 Heidelberg, Germany*

‡*Applied Chemicals and Materials Division, National Institute of Standards and Technology, Boulder, Colorado 80305, United States*

¶*Chemnitz University of Technology, Faculty of Mechanical Engineering, Applied Thermodynamics, 09107 Chemnitz, Germany*

E-mail: xiaoxian.yang@mb.tu-chemnitz.de

- code_SI (folder)
  - main.py (PY): implementation of the core of the RES model
  - Dilute_gas_viscosity.txt (TXT): parameters for the calculation of the dilute gas viscosity
  - RES_Parameter.txt (TXT): parameters for the models
  - Samples_* (TXT): all files beginning with "Samples_" are sample calculations that may be used to verify the model
  - Data_evaluation_REF_15.txt (TXT): various statistics concerned with the data used for the pure fluid calculations
  - Data_evaluation_mix.txt (TXT): various statistics concerned with the data used for the mixtures calculations
  - Table_multi.txt (TXT): information about the experimental data of mixtures
  - Table_pure_REF_15.txt (TXT): various statistics of the model qualities for the pure fluids
  - Fluid_Constants.txt (TXT): fluid constants used
- Pure fluid data and literature.docx (DOCX): detailed information of pure fluid data and the literature
- figure_pure_devs (folder/*PNG) : relative deviation for all used data points for the RES model and REFPROP models for all substances
- figure_pure_groups (folder/*PNG) : $\eta^+_{\text{res}} + 1$ as a function of $\frac{s^+}{\xi}$ for one group each
- mix_dev_exp_res_ecs (folder/*PNG) : relative deviations for all mixtures for the RES model and the REFPROP models individually, as well as their data sources
- mix_s_eta_all_data (folder/*PNG) : $\eta^+_{\text{res}} + 1$ as a function of $\frac{s^+}{\xi}$ for each mixture individually for all data, as well as the data sources

- mix_s_eta_select_data (folder/*PNG) : $\eta^+_{\text{res}} + 1$ as a function of $\frac{s^+}{\xi}$ for each mixtures individually for the filtered data, as well as the data sources

- pure_dev_exp_res_ecs (folder/*PNG) : relative deviations for all pure fluids for the RES model and the REFPROP models individually, as well as the data sources

- pure_s_eta_all_data (folder/*PNG) : $\eta^+_{\text{res}} + 1$ as a function of $\frac{s^+}{\xi}$ for each pure fluid individually for all data, as well as the data sources

- pure_s_eta_fitted_data (folder/*PNG) : $\eta^+_{\text{res}} + 1$ as a function of $\frac{s^+}{\xi}$ for each pure fluid individually for the filtered data, as well as the data sources
